# Supplementary material for: Unsupervised logic-based mechanism inference for network-driven biological processes
Source: PLoS Comput Biol. 2021 Jun 2;17(6):e1009035. doi: 10.1371/journal.pcbi.1009035 (PMC8202945; doi:10.1371/journal.pcbi.1009035)
Supplement: S1 File — (PDF) [file pcbi.1009035.s001.pdf]

Demonstration of Algorithm 1 for the rule of NICD

We start from the list of transitions from the EMT-BW model, which has a total of 246 states. Due to our expert knowledge information, NICD depends on Notch, TP53, and TP63_TP73. Therefore, we need to eliminate the states that are unsymmetric in miRNA (i.e., bit 4), EMTreg (bit 5), ECM (bit 6), and DNAdam (bit 7):

The transition list contains, e.g., the tuple $(0, 1, 0, 0, 0, 1, 1, 1)$, as well as the tuples

$(0, 1, 0, 0, 1, 1, 1, 1)$ (the symmetric counterpart in miRNA)
$(0, 1, 0, 0, 0, 0, 1, 1)$ (the symmetric counterpart in EMTreg)
$(0, 1, 0, 0, 0, 1, 0, 1)$ (the symmetric counterpart in ECM)
$(0, 1, 0, 0, 0, 1, 1, 0)$ (the symmetric counterpart in DNAdam)

Therefore, all these tuples stay in the list.

On the other hand, we have, e.g., the tuple $(0, 1, 1, 0, 0, 1, 1, 1)$ in the list, but the tuple

$(0, 1, 1, 0, 0, 0, 1, 1)$ (the symmetric complement for EMTreg) is not part of the list!

This means, that this state would include a dependency for EMTreg. We therefore eliminate this transition state from the list. This (unique) elimination of states leads to a total number of 192 states in the transition list.

Eliminating NICD from the transition list, however, is not unique:

in the new list, e.g., both tuples $(0, 1, 0, 1, 0, 1, 1, 1)$ and $(1, 1, 0, 1, 0, 1, 1, 1)$ are present (i.e., the symmetric complements for NICD). We therefore have the choice to eliminate one of the tuples from the list.

Eliminate $(0, 1, 0, 1, 0, 1, 1, 1)$:
We need to keep the pattern for Notch (bit 1), TP53 (bit 2), and TP63_TP73 (bit 3): all tuples of the form $(0, 1, 0, 1, X, X, X, X)$ are eliminated (total of 16 tuples), while all tuples of the form $(1, 1, 0, 1, X, X, X, X)$ stay in the list.

Next, we have to decide, whether to eliminate:
$(1, 1, 1, 1, 0, 1, 1, 1)$ or $(0, 1, 1, 1, 0, 1, 1, 1)$
$(1, 0, 0, 1, 0, 1, 1, 1)$ or $(0, 0, 0, 1, 0, 1, 1, 1)$
$(1, 0, 1, 1, 0, 1, 1, 1)$ or $(0, 0, 1, 1, 0, 1, 1, 1)$

This leads to a total of $2^4 = 16$ possible choices of how to eliminate NICD from the dependency list.

Ruleset for the EMT-FW:

$$
\begin{aligned}
\mathrm{NICD}^{*} &= (\mathrm{DNAdam} \wedge \overline{\mathrm{miRNA}} \wedge \overline{\mathrm{NICD}} \wedge \overline{\mathrm{TP53}}) \vee (\overline{\mathrm{DNAdam}} \wedge \mathrm{EMTreg} \wedge \overline{\mathrm{miRNA}} \wedge \mathrm{Notch} \wedge \overline{\mathrm{TP53}} \wedge \overline{\mathrm{TP63TP73}}) \\
&\vee (\overline{\mathrm{DNAdam}} \wedge \mathrm{EMTreg} \wedge \overline{\mathrm{NICD}} \wedge \mathrm{TP53}) \vee (\overline{\mathrm{DNAdam}} \wedge \mathrm{miRNA} \wedge \overline{\mathrm{NICD}} \wedge \overline{\mathrm{TP53}}) \vee (\overline{\mathrm{DNAdam}} \wedge \overline{\mathrm{miRNA}} \wedge \overline{\mathrm{NICD}} \wedge \mathrm{TP53}) \\
&\vee (\overline{\mathrm{DNAdam}} \wedge \overline{\mathrm{NICD}} \wedge \mathrm{TP63TP73}) \vee (\mathrm{ECM} \wedge \mathrm{EMTreg} \wedge \overline{\mathrm{miRNA}} \wedge \mathrm{Notch} \wedge \overline{\mathrm{TP53}} \wedge \overline{\mathrm{TP63TP73}}) \vee (\mathrm{ECM} \wedge \mathrm{EMTreg} \wedge \overline{\mathrm{NICD}}) \\
&\vee (\mathrm{ECM} \wedge \overline{\mathrm{NICD}} \wedge \overline{\mathrm{TP53}}) \vee (\mathrm{ECM} \wedge \overline{\mathrm{NICD}} \wedge \mathrm{TP63TP73}) \vee (\overline{\mathrm{ECM}} \wedge \overline{\mathrm{miRNA}} \wedge \overline{\mathrm{NICD}} \wedge \mathrm{Notch}) \\
&\vee (\mathrm{EMTreg} \wedge \mathrm{miRNA} \wedge \overline{\mathrm{NICD}} \wedge \overline{\mathrm{TP53}}) \vee (\mathrm{EMTreg} \wedge \overline{\mathrm{miRNA}} \wedge \overline{\mathrm{NICD}} \wedge \mathrm{TP53}) \vee (\mathrm{EMTreg} \wedge \overline{\mathrm{NICD}} \wedge \mathrm{Notch}) \\
&\vee (\mathrm{EMTreg} \wedge \overline{\mathrm{NICD}} \wedge \mathrm{TP63TP73}) \vee (\overline{\mathrm{miRNA}} \wedge \overline{\mathrm{NICD}} \wedge \mathrm{TP63TP73}) \vee (\overline{\mathrm{NICD}} \wedge \mathrm{Notch} \wedge \overline{\mathrm{TP53}}) \vee (\overline{\mathrm{NICD}} \wedge \mathrm{Notch} \wedge \mathrm{TP63TP73}) \\
&\vee (\overline{\mathrm{NICD}} \wedge \overline{\mathrm{TP53}} \wedge \mathrm{TP63TP73}) \\
\mathrm{Notch}^{*} &= (\mathrm{DNAdam} \wedge \overline{\mathrm{miRNA}} \wedge \overline{\mathrm{Notch}} \wedge \overline{\mathrm{TP53}}) \vee (\overline{\mathrm{DNAdam}} \wedge \mathrm{EMTreg} \wedge \overline{\mathrm{Notch}} \wedge \mathrm{TP53}) \vee (\overline{\mathrm{DNAdam}} \wedge \mathrm{miRNA} \wedge \overline{\mathrm{Notch}} \wedge \overline{\mathrm{TP53}}) \\
&\vee (\overline{\mathrm{DNAdam}} \wedge \overline{\mathrm{miRNA}} \wedge \overline{\mathrm{Notch}} \wedge \mathrm{TP53}) \vee (\overline{\mathrm{DNAdam}} \wedge \overline{\mathrm{Notch}} \wedge \mathrm{TP63TP73}) \\
&\vee (\mathrm{ECM} \wedge \mathrm{EMTreg} \wedge \overline{\mathrm{miRNA}} \wedge \mathrm{NICD} \wedge \overline{\mathrm{TP53}} \wedge \overline{\mathrm{TP63TP73}}) \vee (\mathrm{ECM} \wedge \mathrm{EMTreg} \wedge \overline{\mathrm{Notch}}) \vee (\mathrm{ECM} \wedge \overline{\mathrm{Notch}} \wedge \overline{\mathrm{TP53}}) \\
&\vee (\mathrm{ECM} \wedge \overline{\mathrm{Notch}} \wedge \mathrm{TP63TP73}) \vee (\mathrm{EMTreg} \wedge \mathrm{miRNA} \wedge \overline{\mathrm{Notch}} \wedge \overline{\mathrm{TP53}}) \vee (\mathrm{EMTreg} \wedge \overline{\mathrm{miRNA}} \wedge \overline{\mathrm{Notch}} \wedge \mathrm{TP53}) \\
&\vee (\mathrm{EMTreg} \wedge \mathrm{NICD} \wedge \overline{\mathrm{Notch}}) \vee (\mathrm{EMTreg} \wedge \overline{\mathrm{Notch}} \wedge \mathrm{TP63TP73}) \vee (\overline{\mathrm{miRNA}} \wedge \mathrm{NICD} \wedge \overline{\mathrm{Notch}}) \vee (\overline{\mathrm{miRNA}} \wedge \overline{\mathrm{Notch}} \wedge \mathrm{TP63TP73}) \\
&\vee (\mathrm{NICD} \wedge \overline{\mathrm{Notch}} \wedge \overline{\mathrm{TP53}}) \vee (\mathrm{NICD} \wedge \overline{\mathrm{Notch}} \wedge \mathrm{TP63TP73}) \vee (\overline{\mathrm{Notch}} \wedge \overline{\mathrm{TP53}} \wedge \mathrm{TP63TP73}) \\
\mathrm{TP53}^{*} &= (\mathrm{DNAdam} \wedge \mathrm{ECM} \wedge \overline{\mathrm{EMTreg}} \wedge \overline{\mathrm{NICD}} \wedge \overline{\mathrm{TP63TP73}}) \vee (\mathrm{DNAdam} \wedge \overline{\mathrm{ECM}} \wedge \overline{\mathrm{EMTreg}} \wedge \mathrm{miRNA} \wedge \overline{\mathrm{NICD}} \wedge \overline{\mathrm{Notch}}) \\
&\vee (\mathrm{DNAdam} \wedge \overline{\mathrm{ECM}} \wedge \mathrm{miRNA} \wedge \overline{\mathrm{NICD}} \wedge \overline{\mathrm{Notch}} \wedge \overline{\mathrm{TP63TP73}}) \vee (\mathrm{DNAdam} \wedge \overline{\mathrm{ECM}} \wedge \overline{\mathrm{TP53}}) \\
&\vee (\mathrm{DNAdam} \wedge \overline{\mathrm{EMTreg}} \wedge \overline{\mathrm{NICD}} \wedge \overline{\mathrm{Notch}} \wedge \overline{\mathrm{TP63TP73}}) \vee (\overline{\mathrm{DNAdam}} \wedge \overline{\mathrm{EMTreg}} \wedge \mathrm{miRNA} \wedge \overline{\mathrm{TP63TP73}}) \\
&\vee (\mathrm{ECM} \wedge \overline{\mathrm{EMTreg}} \wedge \mathrm{miRNA} \wedge \overline{\mathrm{TP63TP73}}) \vee (\mathrm{ECM} \wedge \overline{\mathrm{Notch}} \wedge \overline{\mathrm{TP53}}) \vee (\overline{\mathrm{EMTreg}} \wedge \mathrm{miRNA} \wedge \overline{\mathrm{NICD}} \wedge \overline{\mathrm{TP63TP73}}) \\
&\vee (\overline{\mathrm{EMTreg}} \wedge \mathrm{miRNA} \wedge \overline{\mathrm{Notch}} \wedge \overline{\mathrm{TP63TP73}}) \vee (\overline{\mathrm{EMTreg}} \wedge \mathrm{NICD} \wedge \overline{\mathrm{TP53}}) \vee (\mathrm{miRNA} \wedge \overline{\mathrm{TP53}}) \vee (\overline{\mathrm{NICD}} \wedge \mathrm{Notch} \wedge \overline{\mathrm{TP53}}) \\
&\vee (\overline{\mathrm{TP53}} \wedge \mathrm{TP63TP73}) \\
\mathrm{TP63TP73}^{*} &= (\mathrm{DNAdam} \wedge \overline{\mathrm{ECM}} \wedge \overline{\mathrm{miRNA}} \wedge \overline{\mathrm{TP53}} \wedge \overline{\mathrm{TP63TP73}}) \vee (\mathrm{DNAdam} \wedge \overline{\mathrm{ECM}} \wedge \mathrm{NICD} \wedge \mathrm{Notch} \wedge \overline{\mathrm{TP63TP73}}) \\
&\vee (\overline{\mathrm{DNAdam}} \wedge \mathrm{EMTreg} \wedge \mathrm{miRNA} \wedge \overline{\mathrm{TP63TP73}}) \vee (\overline{\mathrm{DNAdam}} \wedge \mathrm{miRNA} \wedge \overline{\mathrm{TP53}} \wedge \overline{\mathrm{TP63TP73}}) \\
&\vee (\overline{\mathrm{DNAdam}} \wedge \overline{\mathrm{miRNA}} \wedge \mathrm{TP53} \wedge \overline{\mathrm{TP63TP73}}) \vee (\mathrm{ECM} \wedge \mathrm{EMTreg} \wedge \overline{\mathrm{Notch}} \wedge \overline{\mathrm{TP63TP73}}) \vee (\mathrm{ECM} \wedge \overline{\mathrm{Notch}} \wedge \overline{\mathrm{TP53}} \wedge \overline{\mathrm{TP63TP73}}) \\
&\vee (\overline{\mathrm{ECM}} \wedge \overline{\mathrm{miRNA}} \wedge \overline{\mathrm{NICD}} \wedge \mathrm{Notch} \wedge \overline{\mathrm{TP63TP73}}) \vee (\mathrm{EMTreg} \wedge \mathrm{miRNA} \wedge \mathrm{NICD} \wedge \overline{\mathrm{TP63TP73}}) \\
&\vee (\mathrm{EMTreg} \wedge \mathrm{miRNA} \wedge \overline{\mathrm{TP53}} \wedge \overline{\mathrm{TP63TP73}}) \vee (\mathrm{EMTreg} \wedge \overline{\mathrm{miRNA}} \wedge \mathrm{TP53} \wedge \overline{\mathrm{TP63TP73}}) \\
&\vee (\mathrm{EMTreg} \wedge \overline{\mathrm{NICD}} \wedge \mathrm{Notch} \wedge \overline{\mathrm{TP63TP73}}) \vee (\overline{\mathrm{EMTreg}} \wedge \overline{\mathrm{miRNA}} \wedge \mathrm{NICD} \wedge \overline{\mathrm{TP63TP73}}) \\
&\vee (\overline{\mathrm{EMTreg}} \wedge \mathrm{NICD} \wedge \overline{\mathrm{TP53}} \wedge \overline{\mathrm{TP63TP73}}) \vee (\overline{\mathrm{NICD}} \wedge \mathrm{Notch} \wedge \overline{\mathrm{TP53}} \wedge \overline{\mathrm{TP63TP73}}) \\
\mathrm{miRNA}^{*} &= (\mathrm{DNAdam} \wedge \overline{\mathrm{ECM}} \wedge \overline{\mathrm{EMTreg}} \wedge \overline{\mathrm{NICD}} \wedge \overline{\mathrm{Notch}} \wedge \mathrm{TP53}) \vee (\mathrm{DNAdam} \wedge \overline{\mathrm{ECM}} \wedge \overline{\mathrm{EMTreg}} \wedge \overline{\mathrm{NICD}} \wedge \overline{\mathrm{Notch}} \wedge \mathrm{TP63TP73}) \\
&\vee (\mathrm{DNAdam} \wedge \overline{\mathrm{ECM}} \wedge \overline{\mathrm{miRNA}}) \vee (\mathrm{DNAdam} \wedge \overline{\mathrm{ECM}} \wedge \overline{\mathrm{NICD}} \wedge \overline{\mathrm{Notch}} \wedge \mathrm{TP53} \wedge \overline{\mathrm{TP63TP73}}) \\
&\vee (\overline{\mathrm{DNAdam}} \wedge \overline{\mathrm{EMTreg}} \wedge \mathrm{TP53} \wedge \overline{\mathrm{TP63TP73}}) \vee (\mathrm{ECM} \wedge \overline{\mathrm{EMTreg}} \wedge \mathrm{TP53} \wedge \overline{\mathrm{TP63TP73}}) \vee (\mathrm{ECM} \wedge \overline{\mathrm{miRNA}} \wedge \overline{\mathrm{Notch}}) \\
&\vee (\overline{\mathrm{EMTreg}} \wedge \overline{\mathrm{miRNA}} \wedge \mathrm{NICD}) \vee (\overline{\mathrm{EMTreg}} \wedge \overline{\mathrm{NICD}} \wedge \mathrm{TP53} \wedge \overline{\mathrm{TP63TP73}}) \vee (\overline{\mathrm{EMTreg}} \wedge \overline{\mathrm{Notch}} \wedge \mathrm{TP53} \wedge \overline{\mathrm{TP63TP73}}) \\
&\vee (\overline{\mathrm{miRNA}} \wedge \overline{\mathrm{NICD}} \wedge \mathrm{Notch}) \vee (\overline{\mathrm{miRNA}} \wedge \mathrm{TP53}) \vee (\overline{\mathrm{miRNA}} \wedge \mathrm{TP63TP73}) \\
\mathrm{EMTreg}^{*} &= (\mathrm{DNAdam} \wedge \overline{\mathrm{ECM}} \wedge \overline{\mathrm{EMTreg}} \wedge \mathrm{NICD} \wedge \mathrm{Notch}) \vee (\mathrm{DNAdam} \wedge \overline{\mathrm{EMTreg}} \wedge \overline{\mathrm{miRNA}} \wedge \overline{\mathrm{TP53}}) \\
&\vee (\overline{\mathrm{DNAdam}} \wedge \overline{\mathrm{ECM}} \wedge \overline{\mathrm{miRNA}} \wedge \mathrm{NICD} \wedge \overline{\mathrm{TP53}} \wedge \overline{\mathrm{TP63TP73}}) \vee (\overline{\mathrm{DNAdam}} \wedge \overline{\mathrm{EMTreg}} \wedge \mathrm{miRNA} \wedge \overline{\mathrm{TP53}}) \\
&\vee (\overline{\mathrm{DNAdam}} \wedge \overline{\mathrm{EMTreg}} \wedge \overline{\mathrm{miRNA}} \wedge \mathrm{TP53}) \vee (\overline{\mathrm{DNAdam}} \wedge \overline{\mathrm{EMTreg}} \wedge \mathrm{TP63TP73}) \vee (\mathrm{ECM} \wedge \overline{\mathrm{EMTreg}} \wedge \overline{\mathrm{TP53}}) \\
&\vee (\mathrm{ECM} \wedge \overline{\mathrm{EMTreg}} \wedge \mathrm{TP63TP73}) \vee (\mathrm{ECM} \wedge \overline{\mathrm{miRNA}} \wedge \mathrm{NICD} \wedge \mathrm{Notch} \wedge \overline{\mathrm{TP53}} \wedge \overline{\mathrm{TP63TP73}}) \\
&\vee (\overline{\mathrm{ECM}} \wedge \overline{\mathrm{EMTreg}} \wedge \overline{\mathrm{miRNA}} \wedge \mathrm{Notch}) \vee (\overline{\mathrm{EMTreg}} \wedge \overline{\mathrm{miRNA}} \wedge \mathrm{NICD}) \vee (\overline{\mathrm{EMTreg}} \wedge \overline{\mathrm{miRNA}} \wedge \mathrm{TP63TP73}) \\
&\vee (\overline{\mathrm{EMTreg}} \wedge \mathrm{NICD} \wedge \overline{\mathrm{TP53}}) \vee (\overline{\mathrm{EMTreg}} \wedge \mathrm{NICD} \wedge \mathrm{TP63TP73}) \vee (\overline{\mathrm{EMTreg}} \wedge \mathrm{Notch} \wedge \overline{\mathrm{TP53}}) \vee (\overline{\mathrm{EMTreg}} \wedge \mathrm{Notch} \wedge \mathrm{TP63TP73}) \\
&\vee (\overline{\mathrm{EMTreg}} \wedge \overline{\mathrm{TP53}} \wedge \mathrm{TP63TP73}) \\
\mathrm{ECM}^{*} &= \mathrm{ECM} \\
\mathrm{DNAdam}^{*} &= \mathrm{DNAdam}
\end{aligned}
$$

Ruleset for the EMT-BW:

$$
\begin{array}{rcl}
\text{NICD}^{*} & = & (\text{DNAdam} \wedge \overline{\text{NICD}} \wedge \overline{\text{TP53}}) \vee (\overline{\text{DNAdam}} \wedge \text{ECM} \wedge \overline{\text{miRNA}} \wedge \overline{\text{NICD}}) \\
 & \vee & (\overline{\text{DNAdam}} \wedge \text{EMTreg} \wedge \overline{\text{miRNA}} \wedge \text{Notch} \wedge \overline{\text{TP53}} \wedge \overline{\text{TP63TP73}}) \\
 & \vee & (\text{ECM} \wedge \text{EMTreg} \wedge \overline{\text{miRNA}} \wedge \text{Notch} \wedge \overline{\text{TP53}} \wedge \overline{\text{TP63TP73}}) \vee (\overline{\text{ECM}} \wedge \overline{\text{miRNA}} \wedge \overline{\text{NICD}} \wedge \text{TP53}) \\
 & \vee & (\overline{\text{ECM}} \wedge \overline{\text{NICD}} \wedge \text{Notch}) \vee (\text{EMTreg} \wedge \overline{\text{NICD}}) \vee (\text{miRNA} \wedge \overline{\text{NICD}} \wedge \text{Notch}) \vee (\text{miRNA} \wedge \overline{\text{NICD}} \wedge \overline{\text{TP53}}) \\
 & \vee & (\overline{\text{NICD}} \wedge \text{TP63TP73}) \\
\text{Notch}^{*} & = & (\text{DNAdam} \wedge \overline{\text{Notch}} \wedge \overline{\text{TP53}}) \vee (\text{ECM} \wedge \text{EMTreg} \wedge \overline{\text{miRNA}} \wedge \text{NICD} \wedge \overline{\text{TP53}} \wedge \overline{\text{TP63TP73}}) \vee (\text{ECM} \wedge \overline{\text{Notch}} \wedge \overline{\text{TP53}}) \\
 & \vee & (\text{EMTreg} \wedge \overline{\text{Notch}} \wedge \text{TP53}) \vee (\text{miRNA} \wedge \overline{\text{Notch}} \wedge \overline{\text{TP53}}) \vee (\overline{\text{miRNA}} \wedge \overline{\text{Notch}} \wedge \text{TP53}) \vee (\text{NICD} \wedge \overline{\text{Notch}}) \\
 & \vee & (\overline{\text{Notch}} \wedge \text{TP63TP73}) \\
\text{TP53}^{*} & = & (\text{DNAdam} \wedge \text{ECM} \wedge \overline{\text{EMTreg}} \wedge \overline{\text{NICD}} \wedge \overline{\text{TP63TP73}}) \vee (\text{DNAdam} \wedge \overline{\text{ECM}} \wedge \overline{\text{TP53}}) \\
 & \vee & (\overline{\text{DNAdam}} \wedge \overline{\text{EMTreg}} \wedge \text{miRNA} \wedge \overline{\text{TP63TP73}}) \vee (\text{ECM} \wedge \overline{\text{EMTreg}} \wedge \text{miRNA} \wedge \overline{\text{TP63TP73}}) \vee (\text{ECM} \wedge \overline{\text{Notch}} \wedge \overline{\text{TP53}}) \\
 & \vee & (\overline{\text{EMTreg}} \wedge \text{miRNA} \wedge \overline{\text{NICD}} \wedge \overline{\text{Notch}} \wedge \overline{\text{TP63TP73}}) \vee (\overline{\text{EMTreg}} \wedge \text{NICD} \wedge \overline{\text{TP53}}) \vee (\text{miRNA} \wedge \overline{\text{TP53}}) \\
 & \vee & (\overline{\text{NICD}} \wedge \text{Notch} \wedge \overline{\text{TP53}}) \vee (\overline{\text{TP53}} \wedge \text{TP63TP73}) \\
\text{TP63TP73}^{*} & = & (\text{DNAdam} \wedge \overline{\text{ECM}} \wedge \overline{\text{miRNA}} \wedge \overline{\text{TP63TP73}}) \vee (\text{DNAdam} \wedge \overline{\text{ECM}} \wedge \text{NICD} \wedge \overline{\text{TP63TP73}}) \\
 & \vee & (\text{DNAdam} \wedge \overline{\text{ECM}} \wedge \text{Notch} \wedge \overline{\text{TP63TP73}}) \vee (\overline{\text{DNAdam}} \wedge \overline{\text{miRNA}} \wedge \text{TP53} \wedge \overline{\text{TP63TP73}}) \\
 & \vee & (\text{ECM} \wedge \overline{\text{Notch}} \wedge \overline{\text{TP53}} \wedge \overline{\text{TP63TP73}}) \vee (\text{EMTreg} \wedge \text{TP53} \wedge \overline{\text{TP63TP73}}) \\
 & \vee & (\overline{\text{EMTreg}} \wedge \overline{\text{miRNA}} \wedge \text{NICD} \wedge \overline{\text{TP63TP73}}) \vee (\text{miRNA} \wedge \overline{\text{TP53}} \wedge \overline{\text{TP63TP73}}) \vee (\overline{\text{NICD}} \wedge \text{Notch} \wedge \overline{\text{TP53}} \wedge \overline{\text{TP63TP73}}) \\
\text{miRNA}^{*} & = & (\text{DNAdam} \wedge \overline{\text{ECM}} \wedge \overline{\text{miRNA}}) \vee (\overline{\text{DNAdam}} \wedge \overline{\text{EMTreg}} \wedge \text{TP53} \wedge \overline{\text{TP63TP73}}) \\
 & \vee & (\text{ECM} \wedge \overline{\text{EMTreg}} \wedge \text{NICD} \wedge \text{TP53} \wedge \overline{\text{TP63TP73}}) \vee (\text{ECM} \wedge \overline{\text{miRNA}} \wedge \overline{\text{Notch}}) \vee (\overline{\text{EMTreg}} \wedge \overline{\text{miRNA}} \wedge \text{NICD}) \\
 & \vee & (\overline{\text{EMTreg}} \wedge \overline{\text{NICD}} \wedge \overline{\text{Notch}} \wedge \text{TP53} \wedge \overline{\text{TP63TP73}}) \vee (\overline{\text{miRNA}} \wedge \overline{\text{NICD}} \wedge \text{Notch}) \vee (\overline{\text{miRNA}} \wedge \text{TP53}) \\
 & \vee & (\overline{\text{miRNA}} \wedge \text{TP63TP73}) \\
\text{EMTreg}^{*} & = & (\text{DNAdam} \wedge \overline{\text{ECM}} \wedge \overline{\text{EMTreg}} \wedge \text{NICD}) \vee (\text{DNAdam} \wedge \overline{\text{ECM}} \wedge \overline{\text{EMTreg}} \wedge \text{Notch}) \vee (\text{DNAdam} \wedge \overline{\text{EMTreg}} \wedge \overline{\text{TP53}}) \\
 & \vee & (\overline{\text{DNAdam}} \wedge \text{ECM} \wedge \overline{\text{EMTreg}} \wedge \overline{\text{miRNA}}) \vee (\overline{\text{DNAdam}} \wedge \overline{\text{ECM}} \wedge \overline{\text{miRNA}} \wedge \text{NICD} \wedge \overline{\text{TP53}} \wedge \overline{\text{TP63TP73}}) \\
 & \vee & (\overline{\text{DNAdam}} \wedge \overline{\text{EMTreg}} \wedge \overline{\text{miRNA}} \wedge \text{Notch}) \vee (\text{ECM} \wedge \overline{\text{miRNA}} \wedge \text{NICD} \wedge \text{Notch} \wedge \overline{\text{TP53}} \wedge \overline{\text{TP63TP73}}) \\
 & \vee & (\overline{\text{ECM}} \wedge \overline{\text{EMTreg}} \wedge \overline{\text{miRNA}} \wedge \text{TP53}) \vee (\overline{\text{EMTreg}} \wedge \text{miRNA} \wedge \overline{\text{TP53}}) \vee (\overline{\text{EMTreg}} \wedge \overline{\text{miRNA}} \wedge \text{NICD}) \\
 & \vee & (\overline{\text{EMTreg}} \wedge \text{TP63TP73}) \\
\text{ECM}^{*} & = & \text{ECM} \\
\text{DNAdam}^{*} & = & \text{DNAdam}
\end{array}
$$

Ruleset for the EMT-A (RMS = 13.14):

$\mathrm{NICD^{*} = (DNAdam \wedge ECM \wedge \overline{EMTreg} \wedge miRNA \wedge Notch \wedge TP53 \wedge TP63TP73) \vee (DNAdam \wedge ECM \wedge \overline{EMTreg} \wedge \overline{miRNA} \wedge NICD \wedge \overline{Notch} \wedge TP53 \wedge \overline{TP63TP73})}$
$\mathrm{\vee (DNAdam \wedge ECM \wedge \overline{EMTreg} \wedge \overline{Notch} \wedge \overline{TP53} \wedge TP63TP73) \vee (DNAdam \wedge \overline{ECM} \wedge EMTreg \wedge \overline{NICD}) \vee (DNAdam \wedge \overline{ECM} \wedge \overline{miRNA} \wedge \overline{NICD}) \vee (DNAdam \wedge EMTreg \wedge \overline{NICD} \wedge TP53)}$
$\mathrm{\vee (DNAdam \wedge \overline{EMTreg} \wedge \overline{NICD} \wedge \overline{TP53}) \vee (DNAdam \wedge miRNA \wedge \overline{NICD} \wedge \overline{TP53}) \vee (DNAdam \wedge \overline{NICD} \wedge TP63TP73) \vee (\overline{DNAdam} \wedge ECM \wedge EMTreg \wedge miRNA \wedge Notch \wedge TP53 \wedge \overline{TP63TP73})}$
$\mathrm{\vee (\overline{DNAdam} \wedge ECM \wedge EMTreg \wedge miRNA \wedge \overline{Notch} \wedge \overline{TP53} \wedge \overline{TP63TP73}) \vee (\overline{DNAdam} \wedge ECM \wedge EMTreg \wedge \overline{miRNA} \wedge Notch \wedge \overline{TP53} \wedge \overline{TP63TP73})}$
$\mathrm{\vee (\overline{DNAdam} \wedge ECM \wedge \overline{EMTreg} \wedge \overline{miRNA} \wedge NICD \wedge Notch \wedge TP53 \wedge TP63TP73) \vee (\overline{DNAdam} \wedge ECM \wedge \overline{EMTreg} \wedge \overline{miRNA} \wedge \overline{NICD} \wedge \overline{Notch})}$
$\mathrm{\vee (\overline{DNAdam} \wedge ECM \wedge \overline{NICD} \wedge Notch \wedge TP53 \wedge \overline{TP63TP73}) \vee (\overline{DNAdam} \wedge \overline{ECM} \wedge EMTreg \wedge miRNA \wedge Notch \wedge \overline{TP53} \wedge TP63TP73)}$
$\mathrm{\vee (\overline{DNAdam} \wedge \overline{ECM} \wedge EMTreg \wedge \overline{miRNA} \wedge NICD \wedge Notch \wedge \overline{TP53}) \vee (\overline{DNAdam} \wedge \overline{ECM} \wedge EMTreg \wedge \overline{miRNA} \wedge \overline{Notch} \wedge TP53 \wedge TP63TP73) \vee (ECM \wedge EMTreg \wedge miRNA \wedge \overline{NICD})}$
$\mathrm{\vee (ECM \wedge EMTreg \wedge \overline{miRNA} \wedge NICD \wedge Notch \wedge \overline{TP53} \wedge \overline{TP63TP73}) \vee (ECM \wedge miRNA \wedge \overline{NICD} \wedge Notch \wedge TP53) \vee (ECM \wedge \overline{NICD} \wedge \overline{TP53} \wedge TP63TP73) \vee (\overline{ECM} \wedge EMTreg \wedge \overline{NICD} \wedge \overline{Notch})}$
$\mathrm{\vee (\overline{ECM} \wedge \overline{EMTreg} \wedge miRNA \wedge \overline{NICD} \wedge Notch) \vee (\overline{ECM} \wedge \overline{EMTreg} \wedge \overline{NICD} \wedge Notch \wedge \overline{TP53}) \vee (\overline{ECM} \wedge miRNA \wedge \overline{NICD} \wedge \overline{TP53}) \vee (\overline{ECM} \wedge \overline{miRNA} \wedge \overline{NICD} \wedge \overline{Notch} \wedge TP53)}$
$\mathrm{\vee (\overline{ECM} \wedge \overline{miRNA} \wedge \overline{NICD} \wedge TP53 \wedge TP63TP73) \vee (EMTreg \wedge miRNA \wedge \overline{NICD} \wedge \overline{Notch}) \vee (EMTreg \wedge \overline{NICD} \wedge TP53 \wedge \overline{TP63TP73}) \vee (miRNA \wedge \overline{NICD} \wedge \overline{Notch} \wedge TP63TP73)}$
$\mathrm{\vee (\overline{NICD} \wedge \overline{Notch} \wedge \overline{TP53} \wedge TP63TP73)}$

$\mathrm{Notch^{*} = (DNAdam \wedge ECM \wedge EMTreg \wedge miRNA \wedge \overline{NICD} \wedge Notch \wedge \overline{TP53} \wedge \overline{TP63TP73}) \vee (DNAdam \wedge ECM \wedge \overline{EMTreg} \wedge \overline{miRNA} \wedge \overline{NICD} \wedge \overline{TP53} \wedge \overline{TP63TP73}) \vee (DNAdam \wedge \overline{ECM} \wedge \overline{Notch} \wedge \overline{TP53})}$
$\mathrm{\vee (DNAdam \wedge \overline{ECM} \wedge \overline{Notch} \wedge TP63TP73) \vee (DNAdam \wedge EMTreg \wedge NICD \wedge \overline{Notch} \wedge \overline{TP63TP73}) \vee (DNAdam \wedge EMTreg \wedge \overline{Notch} \wedge TP53) \vee (DNAdam \wedge \overline{miRNA} \wedge \overline{Notch})}$
$\mathrm{\vee (DNAdam \wedge NICD \wedge \overline{Notch} \wedge TP53) \vee (DNAdam \wedge \overline{NICD} \wedge \overline{Notch} \wedge TP63TP73) \vee (\overline{DNAdam} \wedge ECM \wedge EMTreg \wedge miRNA \wedge NICD \wedge TP53 \wedge \overline{TP63TP73})}$
$\mathrm{\vee (\overline{DNAdam} \wedge ECM \wedge EMTreg \wedge \overline{NICD} \wedge \overline{Notch} \wedge \overline{TP63TP73}) \vee (\overline{DNAdam} \wedge ECM \wedge \overline{EMTreg} \wedge NICD \wedge \overline{TP53} \wedge TP63TP73) \vee (\overline{DNAdam} \wedge ECM \wedge miRNA \wedge NICD \wedge \overline{TP53} \wedge TP63TP73)}$
$\mathrm{\vee (\overline{DNAdam} \wedge \overline{EMTreg} \wedge NICD \wedge \overline{Notch} \wedge \overline{TP53}) \vee (ECM \wedge EMTreg \wedge \overline{miRNA} \wedge NICD \wedge \overline{TP53} \wedge \overline{TP63TP73}) \vee (ECM \wedge EMTreg \wedge \overline{Notch} \wedge TP53 \wedge \overline{TP63TP73})}$
$\mathrm{\vee (ECM \wedge \overline{EMTreg} \wedge NICD \wedge \overline{Notch} \wedge TP53) \vee (ECM \wedge \overline{miRNA} \wedge \overline{Notch} \wedge \overline{TP63TP73}) \vee (\overline{ECM} \wedge EMTreg \wedge NICD \wedge \overline{Notch}) \vee (\overline{ECM} \wedge EMTreg \wedge \overline{miRNA} \wedge \overline{NICD} \wedge \overline{Notch} \wedge TP53)}$
$\mathrm{\vee (\overline{ECM} \wedge miRNA \wedge \overline{Notch} \wedge \overline{TP53}) \vee (\overline{ECM} \wedge NICD \wedge \overline{Notch} \wedge \overline{TP53}) \vee (\overline{ECM} \wedge NICD \wedge \overline{Notch} \wedge \overline{TP63TP73}) \vee (\overline{ECM} \wedge \overline{NICD} \wedge \overline{Notch} \wedge TP53 \wedge TP63TP73)}$
$\mathrm{\vee (EMTreg \wedge \overline{NICD} \wedge \overline{Notch} \wedge \overline{TP53} \wedge TP63TP73) \vee (\overline{EMTreg} \wedge miRNA \wedge \overline{Notch} \wedge TP63TP73) \vee (\overline{EMTreg} \wedge NICD \wedge \overline{Notch} \wedge \overline{TP53} \wedge TP63TP73) \vee (miRNA \wedge \overline{Notch} \wedge TP53 \wedge TP63TP73)}$

$\mathrm{TP53^{*} = (DNAdam \wedge ECM \wedge \overline{EMTreg} \wedge miRNA \wedge \overline{TP63TP73}) \vee (DNAdam \wedge ECM \wedge \overline{EMTreg} \wedge \overline{Notch} \wedge \overline{TP63TP73}) \vee (DNAdam \wedge ECM \wedge miRNA \wedge \overline{NICD} \wedge \overline{Notch} \wedge \overline{TP63TP73})}$
$\mathrm{\vee (DNAdam \wedge ECM \wedge \overline{miRNA} \wedge \overline{NICD} \wedge Notch \wedge \overline{TP63TP73}) \vee (DNAdam \wedge \overline{ECM} \wedge \overline{TP53}) \vee (DNAdam \wedge \overline{EMTreg} \wedge \overline{TP53}) \vee (DNAdam \wedge \overline{TP53} \wedge TP63TP73)}$
$\mathrm{\vee (\overline{DNAdam} \wedge ECM \wedge EMTreg \wedge miRNA \wedge \overline{TP53} \wedge \overline{TP63TP73}) \vee (\overline{DNAdam} \wedge ECM \wedge \overline{EMTreg} \wedge miRNA \wedge \overline{NICD} \wedge \overline{Notch} \wedge TP53)}$
$\mathrm{\vee (\overline{DNAdam} \wedge ECM \wedge \overline{EMTreg} \wedge \overline{miRNA} \wedge \overline{NICD} \wedge Notch \wedge TP63TP73) \vee (\overline{DNAdam} \wedge ECM \wedge \overline{EMTreg} \wedge NICD \wedge Notch \wedge TP53 \wedge \overline{TP63TP73})}$
$\mathrm{\vee (\overline{DNAdam} \wedge \overline{ECM} \wedge EMTreg \wedge \overline{NICD} \wedge Notch \wedge TP63TP73) \vee (\overline{DNAdam} \wedge \overline{ECM} \wedge \overline{EMTreg} \wedge miRNA \wedge \overline{TP63TP73}) \vee (\overline{DNAdam} \wedge \overline{ECM} \wedge \overline{EMTreg} \wedge \overline{NICD} \wedge \overline{Notch} \wedge TP53 \wedge \overline{TP63TP73})}$
$\mathrm{\vee (\overline{DNAdam} \wedge EMTreg \wedge miRNA \wedge NICD \wedge Notch \wedge TP53 \wedge TP63TP73) \vee (ECM \wedge EMTreg \wedge \overline{miRNA} \wedge \overline{NICD} \wedge \overline{Notch} \wedge TP63TP73) \vee (ECM \wedge EMTreg \wedge \overline{miRNA} \wedge \overline{Notch} \wedge \overline{TP53})}$
$\mathrm{\vee (ECM \wedge \overline{EMTreg} \wedge \overline{miRNA} \wedge \overline{TP53} \wedge TP63TP73) \vee (ECM \wedge \overline{EMTreg} \wedge miRNA \wedge NICD \wedge \overline{TP63TP73}) \vee (ECM \wedge \overline{EMTreg} \wedge miRNA \wedge TP53 \wedge \overline{TP63TP73})}$
$\mathrm{\vee (ECM \wedge \overline{EMTreg} \wedge \overline{miRNA} \wedge \overline{NICD} \wedge Notch \wedge \overline{TP53}) \vee (\overline{ECM} \wedge EMTreg \wedge \overline{miRNA} \wedge \overline{NICD} \wedge Notch \wedge \overline{TP53}) \vee (\overline{ECM} \wedge \overline{EMTreg} \wedge NICD \wedge \overline{TP53})}$
$\mathrm{\vee (\overline{ECM} \wedge \overline{EMTreg} \wedge Notch \wedge \overline{TP53} \wedge TP63TP73) \vee (\overline{ECM} \wedge miRNA \wedge \overline{Notch} \wedge \overline{TP53}) \vee (EMTreg \wedge \overline{miRNA} \wedge \overline{Notch} \wedge \overline{TP53} \wedge TP63TP73)}$
$\mathrm{\vee (\overline{EMTreg} \wedge miRNA \wedge \overline{NICD} \wedge \overline{Notch} \wedge TP53 \wedge \overline{TP63TP73}) \vee (\overline{EMTreg} \wedge miRNA \wedge \overline{TP53} \wedge TP63TP73) \vee (\overline{EMTreg} \wedge NICD \wedge \overline{Notch} \wedge \overline{TP53} \wedge \overline{TP63TP73}) \vee (miRNA \wedge NICD \wedge \overline{Notch} \wedge \overline{TP53})}$
$\mathrm{\vee (miRNA \wedge NICD \wedge \overline{TP53} \wedge \overline{TP63TP73}) \vee (\overline{NICD} \wedge Notch \wedge \overline{TP53} \wedge TP63TP73)}$

$\mathrm{TP63TP73^{*} = (DNAdam \wedge ECM \wedge EMTreg \wedge NICD \wedge \overline{Notch} \wedge \overline{TP53} \wedge TP63TP73) \vee (DNAdam \wedge ECM \wedge \overline{EMTreg} \wedge \overline{miRNA} \wedge Notch \wedge \overline{TP53}) \vee (DNAdam \wedge ECM \wedge \overline{EMTreg} \wedge NICD \wedge Notch \wedge \overline{TP53})}$
$\mathrm{\vee (DNAdam \wedge \overline{ECM} \wedge EMTreg \wedge \overline{TP63TP73}) \vee (DNAdam \wedge \overline{ECM} \wedge \overline{miRNA} \wedge \overline{TP63TP73}) \vee (DNAdam \wedge \overline{ECM} \wedge NICD \wedge \overline{TP63TP73}) \vee (DNAdam \wedge \overline{ECM} \wedge Notch \wedge \overline{TP63TP73})}$
$\mathrm{\vee (DNAdam \wedge \overline{ECM} \wedge \overline{TP53} \wedge \overline{TP63TP73}) \vee (DNAdam \wedge EMTreg \wedge NICD \wedge \overline{Notch} \wedge TP53 \wedge \overline{TP63TP73}) \vee (\overline{DNAdam} \wedge ECM \wedge EMTreg \wedge \overline{NICD} \wedge \overline{TP63TP73})}$
$\mathrm{\vee (\overline{DNAdam} \wedge ECM \wedge \overline{EMTreg} \wedge miRNA \wedge \overline{NICD} \wedge Notch \wedge TP63TP73) \vee (\overline{DNAdam} \wedge ECM \wedge \overline{miRNA} \wedge \overline{NICD} \wedge \overline{TP63TP73}) \vee (\overline{DNAdam} \wedge ECM \wedge \overline{miRNA} \wedge \overline{Notch} \wedge \overline{TP63TP73})}$
$\mathrm{\vee (\overline{DNAdam} \wedge \overline{ECM} \wedge EMTreg \wedge miRNA \wedge NICD \wedge \overline{Notch} \wedge \overline{TP53}) \vee (\overline{DNAdam} \wedge \overline{ECM} \wedge EMTreg \wedge \overline{miRNA} \wedge \overline{NICD} \wedge Notch \wedge TP53 \wedge TP63TP73)}$
$\mathrm{\vee (\overline{DNAdam} \wedge \overline{ECM} \wedge EMTreg \wedge miRNA \wedge NICD \wedge \overline{Notch} \wedge TP53 \wedge TP63TP73) \vee (\overline{DNAdam} \wedge \overline{ECM} \wedge \overline{EMTreg} \wedge \overline{miRNA} \wedge \overline{NICD} \wedge \overline{Notch} \wedge TP63TP73)}$
$\mathrm{\vee (\overline{DNAdam} \wedge EMTreg \wedge NICD \wedge Notch \wedge TP53 \wedge \overline{TP63TP73}) \vee (ECM \wedge EMTreg \wedge \overline{NICD} \wedge Notch \wedge TP53 \wedge \overline{TP63TP73}) \vee (ECM \wedge \overline{EMTreg} \wedge \overline{miRNA} \wedge NICD \wedge Notch \wedge TP53)}$
$\mathrm{\vee (ECM \wedge \overline{EMTreg} \wedge \overline{miRNA} \wedge NICD \wedge \overline{TP63TP73}) \vee (ECM \wedge \overline{EMTreg} \wedge \overline{TP53} \wedge \overline{TP63TP73}) \vee (ECM \wedge \overline{NICD} \wedge \overline{Notch} \wedge \overline{TP53} \wedge \overline{TP63TP73}) \vee (\overline{ECM} \wedge EMTreg \wedge miRNA \wedge \overline{TP63TP73})}$
$\mathrm{\vee (\overline{ECM} \wedge EMTreg \wedge NICD \wedge TP53 \wedge \overline{TP63TP73}) \vee (\overline{ECM} \wedge EMTreg \wedge \overline{Notch} \wedge TP53 \wedge \overline{TP63TP73}) \vee (\overline{ECM} \wedge \overline{EMTreg} \wedge \overline{miRNA} \wedge \overline{NICD} \wedge Notch \wedge \overline{TP63TP73})}$
$\mathrm{\vee (EMTreg \wedge miRNA \wedge NICD \wedge Notch \wedge \overline{TP63TP73}) \vee (EMTreg \wedge miRNA \wedge \overline{NICD} \wedge Notch \wedge \overline{TP63TP73}) \vee (\overline{EMTreg} \wedge NICD \wedge \overline{TP53} \wedge \overline{TP63TP73}) \vee (\overline{EMTreg} \wedge Notch \wedge \overline{TP53} \wedge \overline{TP63TP73})}$
$\mathrm{\vee (miRNA \wedge NICD \wedge \overline{TP53} \wedge \overline{TP63TP73}) \vee (miRNA \wedge \overline{Notch} \wedge \overline{TP53} \wedge \overline{TP63TP73}) \vee (\overline{miRNA} \wedge NICD \wedge \overline{Notch} \wedge TP53 \wedge \overline{TP63TP73})}$

$\mathrm{miRNA^{*} = (DNAdam \wedge ECM \wedge \overline{EMTreg} \wedge \overline{NICD} \wedge \overline{Notch} \wedge TP53 \wedge TP63TP73) \vee (DNAdam \wedge ECM \wedge \overline{EMTreg} \wedge NICD \wedge Notch \wedge \overline{TP53} \wedge TP63TP73)}$
$\mathrm{\vee (DNAdam \wedge ECM \wedge \overline{EMTreg} \wedge NICD \wedge \overline{Notch} \wedge \overline{TP63TP73}) \vee (DNAdam \wedge \overline{ECM} \wedge \overline{miRNA}) \vee (DNAdam \wedge EMTreg \wedge \overline{miRNA} \wedge \overline{NICD}) \vee (DNAdam \wedge EMTreg \wedge \overline{miRNA} \wedge TP53)}$
$\mathrm{\vee (DNAdam \wedge \overline{EMTreg} \wedge \overline{miRNA} \wedge NICD \wedge \overline{TP53}) \vee (DNAdam \wedge \overline{EMTreg} \wedge \overline{NICD} \wedge \overline{Notch} \wedge TP53 \wedge \overline{TP63TP73}) \vee (DNAdam \wedge \overline{miRNA} \wedge NICD \wedge TP63TP73)}$
$\mathrm{\vee (DNAdam \wedge \overline{miRNA} \wedge \overline{NICD} \wedge \overline{TP63TP73}) \vee (DNAdam \wedge \overline{miRNA} \wedge \overline{Notch}) \vee (\overline{DNAdam} \wedge ECM \wedge EMTreg \wedge NICD \wedge Notch \wedge TP63TP73)}$
$\mathrm{\vee (\overline{DNAdam} \wedge ECM \wedge EMTreg \wedge NICD \wedge \overline{Notch} \wedge TP53 \wedge \overline{TP63TP73}) \vee (\overline{DNAdam} \wedge ECM \wedge \overline{EMTreg} \wedge miRNA \wedge \overline{NICD} \wedge \overline{Notch} \wedge TP53)}$
$\mathrm{\vee (\overline{DNAdam} \wedge ECM \wedge \overline{EMTreg} \wedge miRNA \wedge \overline{NICD} \wedge \overline{TP63TP73}) \vee (\overline{DNAdam} \wedge ECM \wedge \overline{EMTreg} \wedge \overline{NICD} \wedge Notch \wedge \overline{TP63TP73})}$
$\mathrm{\vee (\overline{DNAdam} \wedge \overline{ECM} \wedge \overline{EMTreg} \wedge NICD \wedge \overline{Notch} \wedge TP53 \wedge \overline{TP63TP73}) \vee (\overline{DNAdam} \wedge \overline{ECM} \wedge \overline{EMTreg} \wedge \overline{NICD} \wedge Notch \wedge \overline{TP53} \wedge TP63TP73)}$
$\mathrm{\vee (\overline{DNAdam} \wedge \overline{EMTreg} \wedge miRNA \wedge TP53 \wedge \overline{TP63TP73}) \vee (\overline{DNAdam} \wedge \overline{EMTreg} \wedge \overline{miRNA} \wedge \overline{NICD} \wedge Notch \wedge TP63TP73) \vee (ECM \wedge EMTreg \wedge \overline{miRNA} \wedge NICD \wedge \overline{Notch})}$
$\mathrm{\vee (ECM \wedge \overline{EMTreg} \wedge \overline{miRNA} \wedge TP63TP73) \vee (ECM \wedge \overline{EMTreg} \wedge miRNA \wedge NICD \wedge TP53 \wedge \overline{TP63TP73}) \vee (ECM \wedge \overline{miRNA} \wedge TP53 \wedge TP63TP73) \vee (\overline{ECM} \wedge EMTreg \wedge \overline{miRNA} \wedge TP53)}$
$\mathrm{\vee (\overline{ECM} \wedge \overline{EMTreg} \wedge miRNA \wedge NICD \wedge \overline{TP53}) \vee (\overline{ECM} \wedge \overline{miRNA} \wedge \overline{Notch} \wedge TP63TP73) \vee (EMTreg \wedge \overline{miRNA} \wedge \overline{NICD} \wedge TP53) \vee (EMTreg \wedge \overline{miRNA} \wedge \overline{Notch} \wedge TP63TP73)}$
$\mathrm{\vee (EMTreg \wedge \overline{miRNA} \wedge \overline{TP53} \wedge TP63TP73) \vee (\overline{EMTreg} \wedge \overline{miRNA} \wedge NICD \wedge \overline{Notch} \wedge \overline{TP53}) \vee (\overline{EMTreg} \wedge \overline{miRNA} \wedge \overline{NICD} \wedge Notch \wedge \overline{TP53} \wedge \overline{TP63TP73})}$
$\mathrm{\vee (\overline{miRNA} \wedge NICD \wedge \overline{Notch} \wedge TP63TP73) \vee (\overline{miRNA} \wedge \overline{NICD} \wedge TP53 \wedge TP63TP73)}$

$\mathrm{EMTreg^{*} = (DNAdam \wedge ECM \wedge miRNA \wedge \overline{NICD} \wedge Notch \wedge \overline{TP53} \wedge \overline{TP63TP73}) \vee (DNAdam \wedge ECM \wedge \overline{miRNA} \wedge NICD \wedge Notch \wedge \overline{TP63TP73}) \vee (DNAdam \wedge \overline{ECM} \wedge \overline{EMTreg} \wedge \overline{miRNA})}$
$\mathrm{\vee (DNAdam \wedge \overline{ECM} \wedge \overline{EMTreg} \wedge NICD) \vee (DNAdam \wedge \overline{ECM} \wedge \overline{EMTreg} \wedge Notch) \vee (DNAdam \wedge \overline{EMTreg} \wedge \overline{Notch} \wedge TP63TP73) \vee (DNAdam \wedge \overline{EMTreg} \wedge TP53 \wedge TP63TP73)}$
$\mathrm{\vee (\overline{DNAdam} \wedge ECM \wedge EMTreg \wedge \overline{miRNA} \wedge TP53 \wedge TP63TP73) \vee (\overline{DNAdam} \wedge ECM \wedge miRNA \wedge Notch \wedge \overline{TP53} \wedge TP63TP73) \vee (\overline{DNAdam} \wedge ECM \wedge \overline{miRNA} \wedge \overline{NICD} \wedge \overline{TP63TP73})}$
$\mathrm{\vee (\overline{DNAdam} \wedge ECM \wedge miRNA \wedge \overline{Notch} \wedge TP53 \wedge TP63TP73) \vee (\overline{DNAdam} \wedge \overline{ECM} \wedge EMTreg \wedge miRNA \wedge NICD \wedge \overline{Notch} \wedge TP53)}$
$\mathrm{\vee (\overline{DNAdam} \wedge \overline{ECM} \wedge EMTreg \wedge miRNA \wedge \overline{Notch} \wedge TP53 \wedge \overline{TP63TP73}) \vee (\overline{DNAdam} \wedge \overline{ECM} \wedge EMTreg \wedge \overline{miRNA} \wedge NICD \wedge \overline{TP53} \wedge \overline{TP63TP73})}$
$\mathrm{\vee (\overline{DNAdam} \wedge \overline{ECM} \wedge EMTreg \wedge \overline{miRNA} \wedge \overline{NICD} \wedge \overline{Notch} \wedge \overline{TP53} \wedge TP63TP73) \vee (\overline{DNAdam} \wedge \overline{ECM} \wedge miRNA \wedge \overline{NICD} \wedge Notch \wedge TP53 \wedge TP63TP73)}$
$\mathrm{\vee (\overline{DNAdam} \wedge \overline{EMTreg} \wedge \overline{miRNA} \wedge \overline{NICD} \wedge Notch \wedge \overline{TP53}) \vee (\overline{DNAdam} \wedge miRNA \wedge NICD \wedge Notch \wedge \overline{TP53} \wedge TP63TP73) \vee (\overline{DNAdam} \wedge miRNA \wedge NICD \wedge \overline{Notch} \wedge \overline{TP53} \wedge \overline{TP63TP73})}$
$\mathrm{\vee (\overline{DNAdam} \wedge \overline{miRNA} \wedge \overline{NICD} \wedge Notch \wedge \overline{TP53} \wedge \overline{TP63TP73}) \vee (ECM \wedge EMTreg \wedge miRNA \wedge NICD \wedge \overline{TP53} \wedge \overline{TP63TP73}) \vee (ECM \wedge EMTreg \wedge \overline{miRNA} \wedge NICD \wedge Notch \wedge \overline{TP53} \wedge \overline{TP63TP73})}$
$\mathrm{\vee (ECM \wedge EMTreg \wedge \overline{miRNA} \wedge \overline{NICD} \wedge TP53 \wedge \overline{TP63TP73}) \vee (ECM \wedge \overline{EMTreg} \wedge miRNA \wedge \overline{NICD} \wedge \overline{TP53}) \vee (ECM \wedge \overline{EMTreg} \wedge miRNA \wedge Notch \wedge \overline{TP53})}$
$\mathrm{\vee (ECM \wedge \overline{EMTreg} \wedge \overline{NICD} \wedge \overline{TP53} \wedge \overline{TP63TP73}) \vee (ECM \wedge \overline{miRNA} \wedge \overline{NICD} \wedge \overline{Notch} \wedge \overline{TP53} \wedge \overline{TP63TP73}) \vee (\overline{ECM} \wedge \overline{EMTreg} \wedge miRNA \wedge NICD \wedge \overline{TP53})}$
$\mathrm{\vee (\overline{ECM} \wedge \overline{EMTreg} \wedge miRNA \wedge TP63TP73) \vee (\overline{ECM} \wedge \overline{EMTreg} \wedge \overline{miRNA} \wedge TP53) \vee (\overline{ECM} \wedge \overline{EMTreg} \wedge NICD \wedge TP63TP73) \vee (\overline{ECM} \wedge \overline{EMTreg} \wedge Notch \wedge TP63TP73)}$
$\mathrm{\vee (\overline{EMTreg} \wedge miRNA \wedge NICD \wedge TP63TP73) \vee (\overline{EMTreg} \wedge miRNA \wedge \overline{NICD} \wedge \overline{Notch} \wedge \overline{TP53}) \vee (\overline{EMTreg} \wedge miRNA \wedge Notch \wedge TP63TP73) \vee (\overline{EMTreg} \wedge \overline{miRNA} \wedge NICD \wedge \overline{Notch} \wedge \overline{TP63TP73})}$
$\mathrm{\vee (\overline{EMTreg} \wedge NICD \wedge Notch \wedge TP63TP73)}$

$\mathrm{ECM^{*} = ECM}$
$\mathrm{DNAdam^{*} = DNAdam}$

Ruleset for the EMT-B (RMS = 12.76) :

$$
\begin{aligned}
NICD^{*} &= (DNAdam \land ECM \land EMTreg \land miRNA \land \overline{Notch} \land \overline{TP53} \land TP63TP73) \lor (DNAdam \land ECM \land EMTreg \land \overline{miRNA} \land Notch \land TP53 \land \overline{TP63TP73}) \\
&\lor (DNAdam \land ECM \land \overline{EMTreg} \land \overline{miRNA} \land \overline{Notch} \land TP63TP73) \lor (DNAdam \land \overline{ECM} \land EMTreg \land \overline{NICD}) \lor (DNAdam \land \overline{ECM} \land \overline{miRNA} \land \overline{NICD}) \\
&\lor (DNAdam \land EMTreg \land \overline{miRNA} \land \overline{NICD} \land TP53) \lor (DNAdam \land \overline{EMTreg} \land miRNA \land \overline{NICD} \land Notch) \lor (DNAdam \land \overline{EMTreg} \land \overline{NICD} \land \overline{TP53} \land \overline{TP63TP73}) \\
&\lor (DNAdam \land miRNA \land \overline{NICD} \land \overline{Notch} \land \overline{TP53}) \lor (DNAdam \land \overline{NICD} \land TP53 \land TP63TP73) \lor (\overline{DNAdam} \land ECM \land EMTreg \land miRNA \land Notch \land TP53) \\
&\lor (\overline{DNAdam} \land ECM \land EMTreg \land miRNA \land \overline{Notch} \land \overline{TP53} \land \overline{TP63TP73}) \lor (\overline{DNAdam} \land ECM \land EMTreg \land \overline{NICD} \land \overline{TP53}) \lor (\overline{DNAdam} \land ECM \land \overline{EMTreg} \land miRNA \land \overline{Notch} \land \overline{TP53} \land TP63TP73) \\
&\lor (\overline{DNAdam} \land ECM \land \overline{EMTreg} \land \overline{miRNA} \land NICD \land Notch \land TP53) \lor (\overline{DNAdam} \land ECM \land \overline{EMTreg} \land \overline{miRNA} \land Notch \land TP53 \land \overline{TP63TP73}) \\
&\lor (\overline{DNAdam} \land \overline{ECM} \land \overline{EMTreg} \land \overline{miRNA} \land \overline{Notch} \land TP53 \land TP63TP73) \lor (\overline{DNAdam} \land EMTreg \land miRNA \land \overline{NICD}) \lor (\overline{DNAdam} \land EMTreg \land miRNA \land Notch \land \overline{TP53} \land TP63TP73) \\
&\lor (\overline{DNAdam} \land EMTreg \land \overline{miRNA} \land Notch \land \overline{TP53} \land \overline{TP63TP73}) \lor (\overline{DNAdam} \land \overline{miRNA} \land \overline{NICD} \land Notch \land \overline{TP53}) \lor (ECM \land EMTreg \land \overline{miRNA} \land NICD \land Notch \land \overline{TP53} \land \overline{TP63TP73}) \\
&\lor (ECM \land EMTreg \land \overline{NICD} \land \overline{TP53} \land TP63TP73) \lor (ECM \land \overline{EMTreg} \land miRNA \land \overline{NICD} \land Notch) \lor (ECM \land \overline{EMTreg} \land \overline{miRNA} \land NICD \land \overline{Notch} \land \overline{TP53} \land TP63TP73) \\
&\lor (\overline{ECM} \land EMTreg \land miRNA \land \overline{NICD}) \lor (\overline{ECM} \land EMTreg \land \overline{NICD} \land TP53) \lor (\overline{ECM} \land EMTreg \land \overline{NICD} \land \overline{TP63TP73}) \lor (\overline{ECM} \land miRNA \land \overline{NICD} \land \overline{Notch} \land \overline{TP53} \land \overline{TP63TP73}) \\
&\lor (\overline{ECM} \land \overline{miRNA} \land \overline{NICD} \land Notch) \lor (EMTreg \land \overline{NICD} \land Notch \land TP53 \land \overline{TP63TP73}) \lor (EMTreg \land \overline{NICD} \land Notch \land \overline{TP53} \land TP63TP73) \lor (\overline{EMTreg} \land \overline{NICD} \land \overline{Notch} \land TP53 \land TP63TP73) \\
&\lor (miRNA \land \overline{NICD} \land Notch \land TP53) \lor (miRNA \land \overline{NICD} \land Notch \land TP63TP73) \lor (miRNA \land \overline{NICD} \land TP53 \land TP63TP73) \\
Notch^{*} &= (DNAdam \land ECM \land \overline{EMTreg} \land miRNA \land NICD \land TP53 \land TP63TP73) \lor (DNAdam \land ECM \land \overline{EMTreg} \land \overline{miRNA} \land NICD \land Notch \land TP53 \land \overline{TP63TP73}) \lor (DNAdam \land EMTreg \land \overline{Notch}) \\
&\lor (DNAdam \land \overline{miRNA} \land \overline{NICD} \land \overline{Notch}) \lor (DNAdam \land \overline{Notch} \land \overline{TP53}) \lor (\overline{DNAdam} \land ECM \land EMTreg \land \overline{miRNA} \land \overline{NICD} \land TP53 \land \overline{TP63TP73}) \\
&\lor (\overline{DNAdam} \land ECM \land \overline{EMTreg} \land \overline{miRNA} \land NICD \land \overline{TP53} \land TP63TP73) \lor (\overline{DNAdam} \land ECM \land miRNA \land \overline{NICD} \land Notch \land \overline{TP53} \land \overline{TP63TP73}) \\
&\lor (\overline{DNAdam} \land \overline{ECM} \land EMTreg \land \overline{miRNA} \land \overline{NICD} \land TP53 \land TP63TP73) \lor (\overline{DNAdam} \land \overline{ECM} \land EMTreg \land NICD \land \overline{TP53} \land TP63TP73) \\
&\lor (\overline{DNAdam} \land \overline{ECM} \land EMTreg \land \overline{miRNA} \land \overline{NICD} \land Notch \land \overline{TP63TP73}) \lor (\overline{DNAdam} \land EMTreg \land \overline{miRNA} \land \overline{NICD} \land Notch \land TP53 \land TP63TP73) \lor (\overline{DNAdam} \land \overline{EMTreg} \land NICD \land \overline{Notch}) \\
&\lor (ECM \land EMTreg \land miRNA \land \overline{Notch}) \lor (ECM \land EMTreg \land \overline{miRNA} \land NICD \land \overline{TP53} \land \overline{TP63TP73}) \lor (ECM \land EMTreg \land \overline{Notch} \land \overline{TP53}) \lor (ECM \land \overline{EMTreg} \land \overline{miRNA} \land \overline{NICD} \land \overline{Notch} \land TP53) \\
&\lor (ECM \land \overline{miRNA} \land \overline{Notch} \land \overline{TP53} \land \overline{TP63TP73}) \lor (\overline{ECM} \land miRNA \land \overline{Notch} \land \overline{TP53}) \lor (\overline{ECM} \land NICD \land \overline{Notch}) \lor (EMTreg \land miRNA \land \overline{Notch} \land \overline{TP53}) \\
&\lor (EMTreg \land \overline{miRNA} \land \overline{Notch} \land TP53 \land \overline{TP63TP73}) \lor (EMTreg \land \overline{Notch} \land \overline{TP53} \land TP63TP73) \lor (\overline{EMTreg} \land \overline{Notch} \land TP53 \land TP63TP73) \lor (miRNA \land NICD \land \overline{Notch}) \\
&\lor (miRNA \land \overline{Notch} \land TP63TP73) \\
TP53^{*} &= (DNAdam \land ECM \land \overline{EMTreg} \land \overline{miRNA} \land NICD \land \overline{Notch} \land TP63TP73) \lor (DNAdam \land ECM \land \overline{EMTreg} \land \overline{NICD} \land TP53 \land \overline{TP63TP73}) \lor (DNAdam \land \overline{ECM} \land \overline{TP53}) \\
&\lor (DNAdam \land EMTreg \land miRNA \land \overline{Notch} \land \overline{TP53}) \lor (DNAdam \land EMTreg \land \overline{miRNA} \land \overline{NICD} \land \overline{TP53}) \lor (DNAdam \land \overline{EMTreg} \land miRNA \land \overline{NICD} \land \overline{TP53}) \\
&\lor (DNAdam \land \overline{EMTreg} \land NICD \land Notch \land \overline{TP53}) \lor (DNAdam \land \overline{NICD} \land \overline{Notch} \land \overline{TP53}) \lor (\overline{DNAdam} \land ECM \land EMTreg \land miRNA \land Notch \land \overline{TP53}) \\
&\lor (\overline{DNAdam} \land ECM \land EMTreg \land \overline{miRNA} \land \overline{Notch} \land \overline{TP53}) \lor (\overline{DNAdam} \land ECM \land \overline{EMTreg} \land miRNA \land \overline{Notch} \land TP63TP73) \lor (\overline{DNAdam} \land ECM \land \overline{EMTreg} \land \overline{miRNA} \land NICD \land Notch) \\
&\lor (\overline{DNAdam} \land ECM \land \overline{EMTreg} \land \overline{miRNA} \land Notch \land TP63TP73) \lor (\overline{DNAdam} \land ECM \land miRNA \land NICD \land \overline{Notch} \land TP63TP73) \\
&\lor (\overline{DNAdam} \land \overline{ECM} \land EMTreg \land \overline{miRNA} \land \overline{NICD} \land Notch \land TP53 \land \overline{TP63TP73}) \lor (\overline{DNAdam} \land \overline{ECM} \land \overline{EMTreg} \land \overline{miRNA} \land NICD \land \overline{Notch} \land TP63TP73) \\
&\lor (\overline{DNAdam} \land \overline{ECM} \land \overline{EMTreg} \land NICD \land \overline{Notch} \land TP53 \land \overline{TP63TP73}) \lor (\overline{DNAdam} \land EMTreg \land miRNA \land \overline{NICD} \land \overline{TP53}) \lor (\overline{DNAdam} \land \overline{EMTreg} \land miRNA \land TP53 \land \overline{TP63TP73}) \\
&\lor (\overline{DNAdam} \land \overline{EMTreg} \land \overline{miRNA} \land Notch \land \overline{TP53}) \lor (ECM \land EMTreg \land miRNA \land NICD \land \overline{Notch} \land TP63TP73) \lor (ECM \land \overline{EMTreg} \land miRNA \land TP53 \land \overline{TP63TP73}) \\
&\lor (ECM \land \overline{miRNA} \land NICD \land \overline{Notch} \land \overline{TP53} \land \overline{TP63TP73}) \lor (ECM \land \overline{miRNA} \land Notch \land \overline{TP53} \land TP63TP73) \lor (\overline{ECM} \land \overline{EMTreg} \land miRNA \land \overline{TP53}) \lor (\overline{ECM} \land EMTreg \land Notch \land \overline{TP53}) \\
&\lor (\overline{ECM} \land miRNA \land \overline{Notch} \land \overline{TP53}) \lor (\overline{ECM} \land \overline{miRNA} \land NICD \land \overline{TP53} \land TP63TP73) \lor (EMTreg \land miRNA \land NICD \land Notch \land \overline{TP53} \land \overline{TP63TP73}) \\
&\lor (EMTreg \land miRNA \land \overline{NICD} \land \overline{TP53} \land TP63TP73) \lor (\overline{EMTreg} \land miRNA \land \overline{NICD} \land \overline{Notch} \land TP53 \land \overline{TP63TP73}) \lor (\overline{EMTreg} \land \overline{miRNA} \land NICD \land \overline{TP53}) \\
&\lor (\overline{EMTreg} \land NICD \land \overline{TP53} \land TP63TP73) \lor (\overline{EMTreg} \land \overline{Notch} \land \overline{TP53} \land TP63TP73) \lor (miRNA \land \overline{Notch} \land \overline{TP53} \land TP63TP73) \\
TP63TP73^{*} &= (DNAdam \land ECM \land \overline{EMTreg} \land miRNA \land \overline{Notch} \land TP53 \land TP63TP73) \lor (DNAdam \land \overline{ECM} \land \overline{miRNA} \land \overline{TP63TP73}) \\
&\lor (DNAdam \land \overline{ECM} \land NICD \land \overline{TP63TP73}) \lor (DNAdam \land \overline{ECM} \land Notch \land \overline{TP63TP73}) \lor (DNAdam \land \overline{ECM} \land \overline{TP53} \land \overline{TP63TP73}) \lor (DNAdam \land EMTreg \land miRNA \land TP53 \land \overline{TP63TP73}) \\
&\lor (DNAdam \land \overline{EMTreg} \land \overline{miRNA} \land \overline{NICD} \land Notch \land \overline{TP53} \land \overline{TP63TP73}) \lor (DNAdam \land \overline{EMTreg} \land miRNA \land Notch \land \overline{TP53} \land \overline{TP63TP73}) \\
&\lor (DNAdam \land \overline{EMTreg} \land NICD \land Notch \land \overline{TP53} \land \overline{TP63TP73}) \lor (DNAdam \land \overline{miRNA} \land NICD \land TP53 \land \overline{TP63TP73}) \lor (\overline{DNAdam} \land ECM \land EMTreg \land miRNA \land NICD \land TP53 \land TP63TP73) \\
&\lor (\overline{DNAdam} \land ECM \land EMTreg \land miRNA \land \overline{TP53} \land \overline{TP63TP73}) \lor (\overline{DNAdam} \land ECM \land EMTreg \land \overline{miRNA} \land \overline{Notch} \land \overline{TP53}) \lor (\overline{DNAdam} \land ECM \land EMTreg \land \overline{NICD} \land TP53 \land \overline{TP63TP73}) \\
&\lor (\overline{DNAdam} \land ECM \land \overline{EMTreg} \land miRNA \land \overline{NICD} \land Notch \land TP53 \land TP63TP73) \lor (\overline{DNAdam} \land ECM \land \overline{EMTreg} \land \overline{miRNA} \land NICD \land Notch \land \overline{TP53} \land TP63TP73) \\
&\lor (\overline{DNAdam} \land ECM \land \overline{EMTreg} \land \overline{miRNA} \land TP53 \land \overline{TP63TP73}) \lor (\overline{DNAdam} \land \overline{ECM} \land EMTreg \land miRNA \land \overline{NICD} \land Notch \land TP53) \\
&\lor (\overline{DNAdam} \land \overline{ECM} \land \overline{EMTreg} \land miRNA \land \overline{NICD} \land Notch \land \overline{TP53}) \lor (\overline{DNAdam} \land \overline{ECM} \land \overline{EMTreg} \land miRNA \land NICD \land \overline{Notch} \land TP53) \\
&\lor (\overline{DNAdam} \land \overline{ECM} \land \overline{EMTreg} \land miRNA \land \overline{NICD} \land \overline{Notch} \land \overline{TP53} \land TP63TP73) \lor (\overline{DNAdam} \land EMTreg \land miRNA \land NICD \land \overline{Notch} \land TP53 \land TP63TP73) \\
&\lor (\overline{DNAdam} \land \overline{EMTreg} \land \overline{miRNA} \land \overline{NICD} \land Notch \land \overline{TP53} \land \overline{TP63TP73}) \lor (\overline{DNAdam} \land miRNA \land \overline{Notch} \land \overline{TP53} \land \overline{TP63TP73}) \lor (\overline{DNAdam} \land \overline{miRNA} \land \overline{Notch} \land TP53 \land \overline{TP63TP73}) \\
&\lor (ECM \land EMTreg \land miRNA \land NICD \land \overline{Notch} \land TP53) \lor (ECM \land EMTreg \land NICD \land \overline{Notch} \land \overline{TP63TP73}) \lor (ECM \land EMTreg \land \overline{Notch} \land TP53 \land \overline{TP63TP73}) \\
&\lor (ECM \land \overline{EMTreg} \land \overline{miRNA} \land \overline{Notch} \land \overline{TP53} \land \overline{TP63TP73}) \lor (ECM \land \overline{EMTreg} \land \overline{NICD} \land \overline{Notch} \land \overline{TP53} \land \overline{TP63TP73}) \lor (\overline{ECM} \land EMTreg \land miRNA \land Notch \land TP53 \land \overline{TP63TP73}) \\
&\lor (\overline{ECM} \land EMTreg \land NICD \land Notch \land TP53 \land \overline{TP63TP73}) \lor (\overline{ECM} \land \overline{EMTreg} \land NICD \land \overline{TP53} \land \overline{TP63TP73}) \lor (\overline{ECM} \land \overline{EMTreg} \land Notch \land \overline{TP53} \land \overline{TP63TP73}) \\
&\lor (EMTreg \land miRNA \land NICD \land \overline{TP53} \land \overline{TP63TP73}) \lor (EMTreg \land miRNA \land \overline{NICD} \land Notch \land TP53 \land \overline{TP63TP73}) \lor (EMTreg \land miRNA \land \overline{Notch} \land \overline{TP53} \land \overline{TP63TP73}) \\
&\lor (miRNA \land NICD \land Notch \land \overline{TP53} \land \overline{TP63TP73}) \\
miRNA^{*} &= (DNAdam \land ECM \land EMTreg \land \overline{NICD} \land Notch \land \overline{TP53} \land \overline{TP63TP73}) \lor (DNAdam \land ECM \land EMTreg \land \overline{NICD} \land \overline{Notch} \land TP53 \land TP63TP73) \\
&\lor (DNAdam \land ECM \land \overline{EMTreg} \land NICD \land Notch \land TP53) \lor (DNAdam \land ECM \land \overline{EMTreg} \land NICD \land Notch \land TP63TP73) \\
&\lor (DNAdam \land ECM \land \overline{EMTreg} \land NICD \land \overline{Notch} \land \overline{TP53} \land \overline{TP63TP73}) \lor (DNAdam \land \overline{ECM} \land \overline{miRNA}) \lor (DNAdam \land EMTreg \land \overline{miRNA} \land \overline{Notch} \land TP53) \\
&\lor (DNAdam \land \overline{EMTreg} \land \overline{miRNA} \land Notch \land TP53) \lor (DNAdam \land \overline{miRNA} \land \overline{NICD} \land \overline{Notch}) \lor (\overline{DNAdam} \land ECM \land EMTreg \land \overline{miRNA} \land NICD \land \overline{Notch} \land \overline{TP53}) \\
&\lor (\overline{DNAdam} \land ECM \land \overline{EMTreg} \land miRNA \land \overline{NICD} \land \overline{Notch} \land \overline{TP53} \land TP63TP73) \lor (\overline{DNAdam} \land \overline{ECM} \land \overline{EMTreg} \land \overline{NICD} \land Notch \land \overline{TP63TP73}) \\
&\lor (\overline{DNAdam} \land \overline{ECM} \land EMTreg \land miRNA \land NICD \land \overline{Notch} \land \overline{TP53} \land \overline{TP63TP73}) \lor (\overline{DNAdam} \land \overline{ECM} \land EMTreg \land NICD \land TP53 \land TP63TP73) \\
&\lor (\overline{DNAdam} \land \overline{ECM} \land EMTreg \land \overline{NICD} \land \overline{Notch} \land TP53 \land \overline{TP63TP73}) \lor (\overline{DNAdam} \land \overline{ECM} \land \overline{EMTreg} \land NICD \land TP53 \land \overline{TP63TP73}) \\
&\lor (\overline{DNAdam} \land EMTreg \land miRNA \land NICD \land Notch \land \overline{TP53} \land TP63TP73) \lor (\overline{DNAdam} \land EMTreg \land \overline{miRNA} \land TP53 \land \overline{TP63TP73}) \lor (\overline{DNAdam} \land \overline{EMTreg} \land miRNA \land TP53 \land \overline{TP63TP73}) \\
&\lor (\overline{DNAdam} \land \overline{EMTreg} \land \overline{miRNA} \land \overline{NICD} \land Notch \land \overline{TP53}) \lor (\overline{DNAdam} \land \overline{EMTreg} \land NICD \land \overline{Notch} \land TP53 \land \overline{TP63TP73}) \lor (ECM \land EMTreg \land \overline{miRNA} \land Notch \land TP63TP73) \\
&\lor (ECM \land EMTreg \land NICD \land Notch \land TP53 \land \overline{TP63TP73}) \lor (ECM \land \overline{EMTreg} \land miRNA \land NICD \land Notch \land \overline{TP53} \land TP63TP73) \lor (ECM \land \overline{EMTreg} \land miRNA \land \overline{Notch} \land TP53 \land \overline{TP63TP73}) \\
&\lor (\overline{ECM} \land EMTreg \land \overline{miRNA} \land \overline{Notch} \land TP63TP73) \lor (\overline{ECM} \land \overline{EMTreg} \land \overline{miRNA} \land NICD) \lor (\overline{ECM} \land \overline{EMTreg} \land \overline{miRNA} \land Notch \land \overline{TP53}) \lor (\overline{ECM} \land \overline{EMTreg} \land \overline{miRNA} \land TP53 \land TP63TP73) \\
&\lor (EMTreg \land \overline{miRNA} \land \overline{Notch} \land \overline{TP53} \land TP63TP73) \lor (\overline{EMTreg} \land miRNA \land \overline{NICD} \land \overline{Notch} \land TP53 \land \overline{TP63TP73}) \lor (\overline{EMTreg} \land \overline{miRNA} \land NICD \land \overline{TP53} \land \overline{TP63TP73}) \\
&\lor (\overline{EMTreg} \land \overline{miRNA} \land Notch \land \overline{TP53} \land \overline{TP63TP73}) \lor (\overline{EMTreg} \land \overline{miRNA} \land \overline{Notch} \land TP53 \land TP63TP73) \\
EMTreg^{*} &= (DNAdam \land ECM \land EMTreg \land \overline{miRNA} \land \overline{NICD} \land TP53 \land \overline{TP63TP73}) \lor (DNAdam \land ECM \land miRNA \land \overline{NICD} \land \overline{Notch} \land TP53 \land TP63TP73) \\
&\lor (DNAdam \land ECM \land \overline{miRNA} \land NICD \land Notch \land \overline{TP53}) \lor (DNAdam \land ECM \land \overline{miRNA} \land NICD \land \overline{TP53} \land \overline{TP63TP73}) \lor (DNAdam \land ECM \land \overline{NICD} \land Notch \land \overline{TP53} \land \overline{TP63TP73}) \\
&\lor (DNAdam \land \overline{ECM} \land \overline{EMTreg} \land \overline{miRNA}) \lor (DNAdam \land \overline{ECM} \land \overline{EMTreg} \land NICD) \lor (DNAdam \land \overline{ECM} \land \overline{EMTreg} \land Notch) \lor (DNAdam \land \overline{EMTreg} \land miRNA \land \overline{NICD} \land \overline{TP53}) \\
&\lor (DNAdam \land \overline{EMTreg} \land \overline{miRNA} \land NICD) \lor (DNAdam \land \overline{EMTreg} \land \overline{Notch} \land TP63TP73) \lor (\overline{DNAdam} \land ECM \land EMTreg \land miRNA \land \overline{NICD} \land \overline{Notch} \land TP53 \land \overline{TP63TP73}) \\
&\lor (\overline{DNAdam} \land ECM \land miRNA \land NICD \land \overline{Notch} \land \overline{TP53} \land \overline{TP63TP73}) \lor (\overline{DNAdam} \land ECM \land miRNA \land \overline{NICD} \land \overline{Notch} \land \overline{TP53} \land TP63TP73) \\
&\lor (\overline{DNAdam} \land \overline{ECM} \land EMTreg \land miRNA \land NICD \land \overline{Notch} \land TP53 \land \overline{TP63TP73}) \lor (\overline{DNAdam} \land \overline{ECM} \land EMTreg \land miRNA \land \overline{NICD} \land Notch \land TP53) \\
&\lor (\overline{DNAdam} \land \overline{ECM} \land miRNA \land NICD \land \overline{TP53} \land TP63TP73) \lor (\overline{DNAdam} \land \overline{ECM} \land \overline{miRNA} \land NICD \land \overline{TP53} \land \overline{TP63TP73}) \lor (\overline{DNAdam} \land \overline{ECM} \land NICD \land Notch \land \overline{TP53} \land TP63TP73) \\
&\lor (\overline{DNAdam} \land \overline{EMTreg} \land miRNA \land NICD \land \overline{TP53}) \lor (\overline{DNAdam} \land \overline{EMTreg} \land \overline{miRNA} \land TP53) \lor (\overline{DNAdam} \land \overline{EMTreg} \land Notch \land TP63TP73) \\
&\lor (ECM \land EMTreg \land miRNA \land NICD \land Notch \land TP53 \land \overline{TP63TP73}) \lor (ECM \land EMTreg \land \overline{miRNA} \land NICD \land Notch \land \overline{TP53} \land \overline{TP63TP73}) \\
&\lor (ECM \land \overline{EMTreg} \land \overline{miRNA} \land \overline{NICD} \land \overline{TP53} \land \overline{TP63TP73}) \lor (\overline{ECM} \land \overline{EMTreg} \land miRNA \land \overline{TP53}) \lor (\overline{ECM} \land \overline{EMTreg} \land \overline{miRNA} \land NICD) \lor (\overline{ECM} \land \overline{EMTreg} \land TP63TP73) \\
&\lor (\overline{EMTreg} \land miRNA \land Notch \land \overline{TP53}) \lor (\overline{EMTreg} \land miRNA \land \overline{TP53} \land TP63TP73) \lor (\overline{EMTreg} \land \overline{miRNA} \land NICD \land \overline{Notch}) \lor (\overline{EMTreg} \land \overline{miRNA} \land NICD \land TP53) \\
&\lor (\overline{EMTreg} \land NICD \land \overline{TP53} \land TP63TP73) \lor (\overline{EMTreg} \land \overline{NICD} \land Notch \land \overline{TP53} \land \overline{TP63TP73}) \lor (\overline{EMTreg} \land \overline{NICD} \land TP53 \land TP63TP73) \\
ECM^{*} &= ECM \\
DNAdam^{*} &= DNAdam
\end{aligned}
$$

Ruleset for the EMT-C (RMS = 12.68) :

$\mathrm{NICD}^{*} = (\mathrm{DNAdam} \wedge \mathrm{ECM} \wedge \overline{\mathrm{EMTreg}} \wedge \mathrm{miRNA} \wedge \mathrm{NICD} \wedge \overline{\mathrm{Notch}} \wedge \overline{\mathrm{TP53}} \wedge \overline{\mathrm{TP63TP73}}) \vee (\mathrm{DNAdam} \wedge \mathrm{ECM} \wedge \overline{\mathrm{EMTreg}} \wedge \overline{\mathrm{miRNA}} \wedge \mathrm{NICD} \wedge \overline{\mathrm{Notch}} \wedge \mathrm{TP53} \wedge \overline{\mathrm{TP63TP73}})$
$\vee\ (\mathrm{DNAdam} \wedge \overline{\mathrm{ECM}} \wedge \mathrm{EMTreg} \wedge \overline{\mathrm{NICD}}) \vee (\mathrm{DNAdam} \wedge \overline{\mathrm{ECM}} \wedge \overline{\mathrm{miRNA}} \wedge \overline{\mathrm{NICD}}) \vee (\mathrm{DNAdam} \wedge \overline{\mathrm{EMTreg}} \wedge \overline{\mathrm{NICD}} \wedge \mathrm{Notch} \wedge \overline{\mathrm{TP53}} \wedge \overline{\mathrm{TP63TP73}}) \vee (\mathrm{DNAdam} \wedge \mathrm{miRNA} \wedge \overline{\mathrm{NICD}} \wedge \mathrm{TP63TP73})$
$\vee\ (\mathrm{DNAdam} \wedge \overline{\mathrm{miRNA}} \wedge \overline{\mathrm{NICD}} \wedge \overline{\mathrm{Notch}} \wedge \overline{\mathrm{TP53}}) \vee (\mathrm{DNAdam} \wedge \overline{\mathrm{NICD}} \wedge \overline{\mathrm{Notch}} \wedge \mathrm{TP63TP73}) \vee (\overline{\mathrm{DNAdam}} \wedge \mathrm{ECM} \wedge \mathrm{EMTreg} \wedge \mathrm{miRNA} \wedge \mathrm{Notch} \wedge \mathrm{TP53} \wedge \overline{\mathrm{TP63TP73}})$
$\vee\ (\overline{\mathrm{DNAdam}} \wedge \mathrm{ECM} \wedge \mathrm{EMTreg} \wedge \overline{\mathrm{miRNA}} \wedge \mathrm{NICD} \wedge \overline{\mathrm{Notch}} \wedge \mathrm{TP63TP73}) \vee (\overline{\mathrm{DNAdam}} \wedge \mathrm{ECM} \wedge \overline{\mathrm{EMTreg}} \wedge \overline{\mathrm{miRNA}} \wedge \mathrm{NICD} \wedge \overline{\mathrm{TP53}} \wedge \mathrm{TP63TP73})$
$\vee\ (\overline{\mathrm{DNAdam}} \wedge \mathrm{ECM} \wedge \overline{\mathrm{EMTreg}} \wedge \overline{\mathrm{miRNA}} \wedge \mathrm{Notch} \wedge \mathrm{TP53}) \vee (\overline{\mathrm{DNAdam}} \wedge \mathrm{ECM} \wedge \overline{\mathrm{EMTreg}} \wedge \mathrm{NICD} \wedge \overline{\mathrm{Notch}} \wedge \overline{\mathrm{TP53}} \wedge \mathrm{TP63TP73})$
$\vee\ (\overline{\mathrm{DNAdam}} \wedge \overline{\mathrm{ECM}} \wedge \mathrm{EMTreg} \wedge \mathrm{miRNA} \wedge \overline{\mathrm{Notch}} \wedge \overline{\mathrm{TP53}} \wedge \overline{\mathrm{TP63TP73}}) \vee (\overline{\mathrm{DNAdam}} \wedge \overline{\mathrm{ECM}} \wedge \mathrm{EMTreg} \wedge \overline{\mathrm{miRNA}} \wedge \mathrm{Notch} \wedge \overline{\mathrm{TP53}}) \vee (\overline{\mathrm{DNAdam}} \wedge \overline{\mathrm{ECM}} \wedge \mathrm{EMTreg} \wedge \mathrm{Notch} \wedge \overline{\mathrm{TP53}} \wedge \mathrm{TP63TP73})$
$\vee\ (\overline{\mathrm{DNAdam}} \wedge \overline{\mathrm{ECM}} \wedge \overline{\mathrm{EMTreg}} \wedge \overline{\mathrm{miRNA}} \wedge \overline{\mathrm{Notch}} \wedge \mathrm{TP53} \wedge \mathrm{TP63TP73}) \vee (\overline{\mathrm{DNAdam}} \wedge \mathrm{EMTreg} \wedge \overline{\mathrm{miRNA}} \wedge \overline{\mathrm{NICD}} \wedge \overline{\mathrm{TP53}}) \vee (\overline{\mathrm{DNAdam}} \wedge \mathrm{EMTreg} \wedge \overline{\mathrm{NICD}} \wedge \mathrm{Notch} \wedge \overline{\mathrm{TP53}})$
$\vee\ (\mathrm{DNAdam} \wedge \mathrm{EMTreg} \wedge \overline{\mathrm{NICD}} \wedge \overline{\mathrm{Notch}} \wedge \overline{\mathrm{TP63TP73}}) \vee (\overline{\mathrm{DNAdam}} \wedge \overline{\mathrm{EMTreg}} \wedge \mathrm{miRNA} \wedge \overline{\mathrm{NICD}} \wedge \mathrm{TP53}) \vee (\overline{\mathrm{DNAdam}} \wedge \overline{\mathrm{miRNA}} \wedge \overline{\mathrm{NICD}} \wedge \mathrm{Notch} \wedge \overline{\mathrm{TP53}})$
$\vee\ (\mathrm{ECM} \wedge \mathrm{EMTreg} \wedge \overline{\mathrm{miRNA}} \wedge \mathrm{NICD} \wedge \mathrm{Notch} \wedge \overline{\mathrm{TP53}} \wedge \overline{\mathrm{TP63TP73}}) \vee (\mathrm{ECM} \wedge \mathrm{EMTreg} \wedge \overline{\mathrm{NICD}} \wedge \mathrm{Notch} \wedge \mathrm{TP53}) \vee (\mathrm{ECM} \wedge \overline{\mathrm{miRNA}} \wedge \overline{\mathrm{NICD}} \wedge \overline{\mathrm{Notch}} \wedge \overline{\mathrm{TP53}} \wedge \overline{\mathrm{TP63TP73}})$
$\vee\ (\overline{\mathrm{ECM}} \wedge \mathrm{miRNA} \wedge \overline{\mathrm{NICD}} \wedge \overline{\mathrm{TP53}}) \vee (\overline{\mathrm{ECM}} \wedge \overline{\mathrm{miRNA}} \wedge \overline{\mathrm{NICD}} \wedge \overline{\mathrm{Notch}} \wedge \mathrm{TP53}) \vee (\overline{\mathrm{ECM}} \wedge \overline{\mathrm{NICD}} \wedge \overline{\mathrm{Notch}} \wedge \mathrm{TP63TP73}) \vee (\mathrm{EMTreg} \wedge \mathrm{miRNA} \wedge \overline{\mathrm{NICD}} \wedge \mathrm{TP53})$
$\vee\ (\mathrm{EMTreg} \wedge \overline{\mathrm{miRNA}} \wedge \overline{\mathrm{NICD}} \wedge \overline{\mathrm{Notch}} \wedge \overline{\mathrm{TP53}}) \vee (\mathrm{EMTreg} \wedge \overline{\mathrm{NICD}} \wedge \mathrm{Notch} \wedge \overline{\mathrm{TP53}} \wedge \mathrm{TP63TP73}) \vee (\overline{\mathrm{EMTreg}} \wedge \mathrm{miRNA} \wedge \overline{\mathrm{NICD}} \wedge \mathrm{TP63TP73}) \vee (\overline{\mathrm{EMTreg}} \wedge \overline{\mathrm{NICD}} \wedge \mathrm{TP53} \wedge \mathrm{TP63TP73})$
$\vee\ (\mathrm{miRNA} \wedge \overline{\mathrm{NICD}} \wedge \mathrm{Notch} \wedge \mathrm{TP53})$

$\mathrm{Notch}^{*} = (\mathrm{DNAdam} \wedge \mathrm{ECM} \wedge \mathrm{EMTreg} \wedge \overline{\mathrm{miRNA}} \wedge \overline{\mathrm{NICD}} \wedge \overline{\mathrm{TP63TP73}}) \vee (\mathrm{DNAdam} \wedge \mathrm{ECM} \wedge \overline{\mathrm{EMTreg}} \wedge \mathrm{miRNA} \wedge \mathrm{NICD} \wedge \mathrm{TP53} \wedge \mathrm{TP63TP73})$
$\vee\ (\mathrm{DNAdam} \wedge \mathrm{ECM} \wedge \overline{\mathrm{EMTreg}} \wedge \overline{\mathrm{miRNA}} \wedge \overline{\mathrm{NICD}} \wedge \overline{\mathrm{TP53}} \wedge \mathrm{TP63TP73}) \vee (\mathrm{DNAdam} \wedge \overline{\mathrm{ECM}} \wedge \overline{\mathrm{miRNA}} \wedge \overline{\mathrm{Notch}}) \vee (\mathrm{DNAdam} \wedge \mathrm{EMTreg} \wedge \overline{\mathrm{Notch}}) \vee (\mathrm{DNAdam} \wedge \overline{\mathrm{NICD}} \wedge \overline{\mathrm{Notch}} \wedge \overline{\mathrm{TP53}})$
$\vee\ (\overline{\mathrm{DNAdam}} \wedge \mathrm{ECM} \wedge \overline{\mathrm{EMTreg}} \wedge \mathrm{miRNA} \wedge \mathrm{NICD} \wedge \mathrm{TP63TP73}) \vee (\overline{\mathrm{DNAdam}} \wedge \mathrm{ECM} \wedge \mathrm{EMTreg} \wedge \mathrm{miRNA} \wedge \overline{\mathrm{NICD}} \wedge \overline{\mathrm{TP53}} \wedge \overline{\mathrm{TP63TP73}})$
$\vee\ (\overline{\mathrm{DNAdam}} \wedge \mathrm{ECM} \wedge \overline{\mathrm{EMTreg}} \wedge \overline{\mathrm{miRNA}} \wedge \mathrm{NICD} \wedge \mathrm{TP53}) \vee (\overline{\mathrm{DNAdam}} \wedge \overline{\mathrm{ECM}} \wedge \mathrm{EMTreg} \wedge \overline{\mathrm{miRNA}} \wedge \mathrm{NICD} \wedge \overline{\mathrm{TP53}} \wedge \mathrm{TP63TP73})$
$\vee\ (\overline{\mathrm{DNAdam}} \wedge \overline{\mathrm{ECM}} \wedge \mathrm{EMTreg} \wedge \overline{\mathrm{miRNA}} \wedge \overline{\mathrm{NICD}} \wedge \mathrm{Notch} \wedge \overline{\mathrm{TP53}} \wedge \overline{\mathrm{TP63TP73}}) \vee (\overline{\mathrm{DNAdam}} \wedge \overline{\mathrm{ECM}} \wedge \overline{\mathrm{EMTreg}} \wedge \mathrm{miRNA} \wedge \overline{\mathrm{NICD}} \wedge \overline{\mathrm{TP53}})$
$\vee\ (\overline{\mathrm{DNAdam}} \wedge \overline{\mathrm{EMTreg}} \wedge \mathrm{miRNA} \wedge \mathrm{NICD} \wedge \mathrm{TP53} \wedge \overline{\mathrm{TP63TP73}}) \vee (\overline{\mathrm{DNAdam}} \wedge \overline{\mathrm{EMTreg}} \wedge \mathrm{NICD} \wedge \overline{\mathrm{Notch}}) \vee (\mathrm{ECM} \wedge \mathrm{EMTreg} \wedge \overline{\mathrm{miRNA}} \wedge \mathrm{NICD} \wedge \overline{\mathrm{TP53}} \wedge \overline{\mathrm{TP63TP73}})$
$\vee\ (\mathrm{ECM} \wedge \mathrm{EMTreg} \wedge \overline{\mathrm{NICD}} \wedge \overline{\mathrm{Notch}} \wedge \overline{\mathrm{TP63TP73}}) \vee (\mathrm{ECM} \wedge \overline{\mathrm{miRNA}} \wedge \overline{\mathrm{Notch}} \wedge \mathrm{TP63TP73}) \vee (\overline{\mathrm{ECM}} \wedge \mathrm{EMTreg} \wedge \overline{\mathrm{Notch}} \wedge \mathrm{TP63TP73}) \vee (\overline{\mathrm{ECM}} \wedge \mathrm{NICD} \wedge \overline{\mathrm{Notch}})$
$\vee\ (\mathrm{EMTreg} \wedge \mathrm{miRNA} \wedge \overline{\mathrm{NICD}} \wedge \overline{\mathrm{Notch}} \wedge \overline{\mathrm{TP53}}) \vee (\mathrm{EMTreg} \wedge \overline{\mathrm{miRNA}} \wedge \overline{\mathrm{Notch}} \wedge \mathrm{TP53} \wedge \overline{\mathrm{TP63TP73}}) \vee (\mathrm{EMTreg} \wedge \mathrm{NICD} \wedge \overline{\mathrm{Notch}} \wedge \mathrm{TP63TP73})$
$\vee\ (\mathrm{EMTreg} \wedge \overline{\mathrm{Notch}} \wedge \overline{\mathrm{TP53}} \wedge \mathrm{TP63TP73}) \vee (\overline{\mathrm{EMTreg}} \wedge \mathrm{NICD} \wedge \overline{\mathrm{Notch}} \wedge \mathrm{TP53}) \vee (\overline{\mathrm{EMTreg}} \wedge \overline{\mathrm{Notch}} \wedge \mathrm{TP53} \wedge \mathrm{TP63TP73}) \vee (\mathrm{miRNA} \wedge \overline{\mathrm{NICD}} \wedge \overline{\mathrm{Notch}} \wedge \mathrm{TP63TP73})$
$\vee\ (\overline{\mathrm{miRNA}} \wedge \mathrm{NICD} \wedge \overline{\mathrm{Notch}} \wedge \overline{\mathrm{TP63TP73}})$

$\mathrm{TP53}^{*} = (\mathrm{DNAdam} \wedge \mathrm{ECM} \wedge \overline{\mathrm{EMTreg}} \wedge \mathrm{miRNA} \wedge \mathrm{NICD} \wedge \mathrm{Notch}) \vee (\mathrm{DNAdam} \wedge \mathrm{ECM} \wedge \overline{\mathrm{EMTreg}} \wedge \mathrm{miRNA} \wedge \overline{\mathrm{NICD}} \wedge \overline{\mathrm{TP63TP73}}) \vee (\mathrm{DNAdam} \wedge \mathrm{ECM} \wedge \overline{\mathrm{EMTreg}} \wedge \overline{\mathrm{NICD}} \wedge \overline{\mathrm{Notch}} \wedge \overline{\mathrm{TP63TP73}})$
$\vee\ (\mathrm{DNAdam} \wedge \mathrm{ECM} \wedge \overline{\mathrm{EMTreg}} \wedge \overline{\mathrm{NICD}} \wedge \mathrm{TP53} \wedge \overline{\mathrm{TP63TP73}}) \vee (\mathrm{DNAdam} \wedge \overline{\mathrm{ECM}} \wedge \overline{\mathrm{TP53}}) \vee (\mathrm{DNAdam} \wedge \mathrm{EMTreg} \wedge \overline{\mathrm{miRNA}} \wedge \overline{\mathrm{NICD}} \wedge \overline{\mathrm{TP53}}) \vee (\mathrm{DNAdam} \wedge \mathrm{EMTreg} \wedge \overline{\mathrm{Notch}} \wedge \overline{\mathrm{TP53}})$
$\vee\ (\mathrm{DNAdam} \wedge \mathrm{miRNA} \wedge \mathrm{NICD} \wedge \mathrm{Notch} \wedge \overline{\mathrm{TP53}}) \vee (\mathrm{DNAdam} \wedge \overline{\mathrm{miRNA}} \wedge \overline{\mathrm{Notch}} \wedge \overline{\mathrm{TP53}} \wedge \overline{\mathrm{TP63TP73}}) \vee (\mathrm{DNAdam} \wedge \overline{\mathrm{NICD}} \wedge \overline{\mathrm{Notch}} \wedge \overline{\mathrm{TP53}})$
$\vee\ (\overline{\mathrm{DNAdam}} \wedge \mathrm{ECM} \wedge \mathrm{EMTreg} \wedge \mathrm{miRNA} \wedge \mathrm{NICD} \wedge \mathrm{Notch} \wedge \mathrm{TP53} \wedge \mathrm{TP63TP73}) \vee (\overline{\mathrm{DNAdam}} \wedge \mathrm{ECM} \wedge \overline{\mathrm{EMTreg}} \wedge \mathrm{miRNA} \wedge \overline{\mathrm{NICD}} \wedge \overline{\mathrm{Notch}})$
$\vee\ (\overline{\mathrm{DNAdam}} \wedge \mathrm{ECM} \wedge \overline{\mathrm{EMTreg}} \wedge \overline{\mathrm{miRNA}} \wedge \mathrm{NICD} \wedge \overline{\mathrm{Notch}} \wedge \mathrm{TP53} \wedge \mathrm{TP63TP73}) \vee (\overline{\mathrm{DNAdam}} \wedge \mathrm{ECM} \wedge \mathrm{miRNA} \wedge \mathrm{NICD} \wedge \overline{\mathrm{Notch}} \wedge \mathrm{TP53} \wedge \overline{\mathrm{TP63TP73}})$
$\vee\ (\overline{\mathrm{DNAdam}} \wedge \overline{\mathrm{ECM}} \wedge \overline{\mathrm{EMTreg}} \wedge \mathrm{miRNA} \wedge \overline{\mathrm{Notch}} \wedge \overline{\mathrm{TP63TP73}}) \vee (\overline{\mathrm{DNAdam}} \wedge \overline{\mathrm{ECM}} \wedge \overline{\mathrm{EMTreg}} \wedge \overline{\mathrm{NICD}} \wedge \overline{\mathrm{Notch}} \wedge \mathrm{TP53} \wedge \overline{\mathrm{TP63TP73}})$
$\vee\ (\overline{\mathrm{DNAdam}} \wedge \overline{\mathrm{ECM}} \wedge \mathrm{miRNA} \wedge \overline{\mathrm{NICD}} \wedge \overline{\mathrm{Notch}} \wedge \overline{\mathrm{TP63TP73}}) \vee (\overline{\mathrm{DNAdam}} \wedge \mathrm{EMTreg} \wedge \mathrm{miRNA} \wedge \overline{\mathrm{NICD}} \wedge \overline{\mathrm{TP53}}) \vee (\overline{\mathrm{DNAdam}} \wedge \overline{\mathrm{EMTreg}} \wedge \mathrm{miRNA} \wedge \mathrm{TP53} \wedge \overline{\mathrm{TP63TP73}})$
$\vee\ (\overline{\mathrm{DNAdam}} \wedge \overline{\mathrm{EMTreg}} \wedge \overline{\mathrm{miRNA}} \wedge \overline{\mathrm{NICD}} \wedge \mathrm{Notch} \wedge \mathrm{TP63TP73}) \vee (\overline{\mathrm{DNAdam}} \wedge \overline{\mathrm{EMTreg}} \wedge \overline{\mathrm{miRNA}} \wedge \mathrm{Notch} \wedge \overline{\mathrm{TP53}} \wedge \overline{\mathrm{TP63TP73}}) \vee (\mathrm{ECM} \wedge \mathrm{EMTreg} \wedge \mathrm{NICD} \wedge \overline{\mathrm{Notch}} \wedge \overline{\mathrm{TP53}})$
$\vee\ (\mathrm{ECM} \wedge \overline{\mathrm{EMTreg}} \wedge \mathrm{miRNA} \wedge \mathrm{NICD} \wedge \mathrm{Notch} \wedge \overline{\mathrm{TP63TP73}}) \vee (\mathrm{ECM} \wedge \overline{\mathrm{EMTreg}} \wedge \mathrm{miRNA} \wedge \mathrm{TP53} \wedge \overline{\mathrm{TP63TP73}}) \vee (\mathrm{ECM} \wedge \overline{\mathrm{EMTreg}} \wedge \overline{\mathrm{NICD}} \wedge \overline{\mathrm{Notch}} \wedge \overline{\mathrm{TP53}} \wedge \overline{\mathrm{TP63TP73}})$
$\vee\ (\overline{\mathrm{ECM}} \wedge \overline{\mathrm{EMTreg}} \wedge \mathrm{NICD} \wedge \overline{\mathrm{Notch}} \wedge \overline{\mathrm{TP53}}) \vee (\overline{\mathrm{ECM}} \wedge \overline{\mathrm{miRNA}} \wedge \mathrm{NICD} \wedge \overline{\mathrm{TP53}} \wedge \mathrm{TP63TP73}) \vee (\overline{\mathrm{ECM}} \wedge \overline{\mathrm{Notch}} \wedge \overline{\mathrm{TP53}} \wedge \mathrm{TP63TP73}) \vee (\mathrm{EMTreg} \wedge \mathrm{miRNA} \wedge \mathrm{NICD} \wedge \overline{\mathrm{TP53}} \wedge \overline{\mathrm{TP63TP73}})$
$\vee\ (\mathrm{EMTreg} \wedge \mathrm{miRNA} \wedge \overline{\mathrm{Notch}} \wedge \overline{\mathrm{TP53}}) \vee (\mathrm{EMTreg} \wedge \overline{\mathrm{miRNA}} \wedge \mathrm{NICD} \wedge \overline{\mathrm{TP53}} \wedge \mathrm{TP63TP73}) \vee (\mathrm{EMTreg} \wedge \overline{\mathrm{miRNA}} \wedge \overline{\mathrm{NICD}} \wedge \mathrm{Notch} \wedge \overline{\mathrm{TP53}} \wedge \overline{\mathrm{TP63TP73}})$
$\vee\ (\mathrm{EMTreg} \wedge \overline{\mathrm{Notch}} \wedge \overline{\mathrm{TP53}} \wedge \mathrm{TP63TP73}) \vee (\overline{\mathrm{EMTreg}} \wedge \mathrm{miRNA} \wedge \overline{\mathrm{NICD}} \wedge \overline{\mathrm{Notch}} \wedge \overline{\mathrm{TP63TP73}}) \vee (\overline{\mathrm{EMTreg}} \wedge \mathrm{miRNA} \wedge \overline{\mathrm{TP53}} \wedge \mathrm{TP63TP73})$
$\vee\ (\overline{\mathrm{EMTreg}} \wedge \overline{\mathrm{miRNA}} \wedge \mathrm{NICD} \wedge \mathrm{Notch} \wedge \overline{\mathrm{TP53}} \wedge \overline{\mathrm{TP63TP73}}) \vee (\mathrm{miRNA} \wedge \overline{\mathrm{NICD}} \wedge \overline{\mathrm{TP53}} \wedge \mathrm{TP63TP73})$

$\mathrm{TP63TP73}^{*} = (\mathrm{DNAdam} \wedge \mathrm{ECM} \wedge \overline{\mathrm{EMTreg}} \wedge \overline{\mathrm{miRNA}} \wedge \mathrm{NICD} \wedge \overline{\mathrm{Notch}} \wedge \overline{\mathrm{TP53}}) \vee (\mathrm{DNAdam} \wedge \mathrm{ECM} \wedge \mathrm{EMTreg} \wedge \mathrm{miRNA} \wedge \overline{\mathrm{NICD}} \wedge \overline{\mathrm{Notch}} \wedge \mathrm{TP53})$
$\vee\ (\mathrm{DNAdam} \wedge \mathrm{ECM} \wedge \overline{\mathrm{EMTreg}} \wedge \overline{\mathrm{miRNA}} \wedge \mathrm{Notch} \wedge \mathrm{TP53} \wedge \mathrm{TP63TP73}) \vee (\mathrm{DNAdam} \wedge \overline{\mathrm{ECM}} \wedge \mathrm{EMTreg} \wedge \overline{\mathrm{TP63TP73}}) \vee (\mathrm{DNAdam} \wedge \overline{\mathrm{ECM}} \wedge \overline{\mathrm{miRNA}} \wedge \overline{\mathrm{TP63TP73}})$
$\vee\ (\mathrm{DNAdam} \wedge \overline{\mathrm{ECM}} \wedge \mathrm{NICD} \wedge \overline{\mathrm{TP63TP73}}) \vee (\mathrm{DNAdam} \wedge \overline{\mathrm{ECM}} \wedge \mathrm{Notch} \wedge \overline{\mathrm{TP63TP73}}) \vee (\mathrm{DNAdam} \wedge \mathrm{EMTreg} \wedge \mathrm{NICD} \wedge \mathrm{TP53} \wedge \overline{\mathrm{TP63TP73}})$
$\vee\ (\mathrm{DNAdam} \wedge \overline{\mathrm{EMTreg}} \wedge \overline{\mathrm{NICD}} \wedge \overline{\mathrm{TP53}} \wedge \overline{\mathrm{TP63TP73}}) \vee (\mathrm{DNAdam} \wedge \overline{\mathrm{EMTreg}} \wedge \mathrm{Notch} \wedge \overline{\mathrm{TP53}} \wedge \overline{\mathrm{TP63TP73}}) \vee (\mathrm{DNAdam} \wedge \overline{\mathrm{miRNA}} \wedge \overline{\mathrm{NICD}} \wedge \overline{\mathrm{TP53}} \wedge \overline{\mathrm{TP63TP73}})$
$\vee\ (\overline{\mathrm{DNAdam}} \wedge \mathrm{ECM} \wedge \mathrm{EMTreg} \wedge \overline{\mathrm{miRNA}} \wedge \overline{\mathrm{NICD}} \wedge \overline{\mathrm{Notch}} \wedge \mathrm{TP53}) \vee (\overline{\mathrm{DNAdam}} \wedge \mathrm{ECM} \wedge \overline{\mathrm{EMTreg}} \wedge \mathrm{miRNA} \wedge \overline{\mathrm{NICD}} \wedge \overline{\mathrm{TP53}} \wedge \mathrm{TP63TP73})$
$\vee\ (\overline{\mathrm{DNAdam}} \wedge \overline{\mathrm{ECM}} \wedge \mathrm{EMTreg} \wedge \mathrm{miRNA} \wedge \mathrm{NICD} \wedge \overline{\mathrm{Notch}}) \vee (\overline{\mathrm{DNAdam}} \wedge \overline{\mathrm{ECM}} \wedge \mathrm{EMTreg} \wedge \overline{\mathrm{miRNA}} \wedge \overline{\mathrm{NICD}} \wedge \mathrm{Notch} \wedge \overline{\mathrm{TP53}} \wedge \mathrm{TP63TP73})$
$\vee\ (\overline{\mathrm{DNAdam}} \wedge \overline{\mathrm{ECM}} \wedge \overline{\mathrm{EMTreg}} \wedge \overline{\mathrm{miRNA}} \wedge \mathrm{NICD} \wedge \overline{\mathrm{Notch}} \wedge \overline{\mathrm{TP53}}) \vee (\overline{\mathrm{DNAdam}} \wedge \mathrm{EMTreg} \wedge \mathrm{miRNA} \wedge \mathrm{NICD} \wedge \mathrm{Notch} \wedge \overline{\mathrm{TP53}}) \vee (\overline{\mathrm{DNAdam}} \wedge \mathrm{EMTreg} \wedge \mathrm{miRNA} \wedge \overline{\mathrm{NICD}} \wedge \overline{\mathrm{TP63TP73}})$
$\vee\ (\overline{\mathrm{DNAdam}} \wedge \overline{\mathrm{EMTreg}} \wedge \mathrm{miRNA} \wedge \mathrm{NICD} \wedge \overline{\mathrm{TP53}} \wedge \overline{\mathrm{TP63TP73}}) \vee (\overline{\mathrm{DNAdam}} \wedge \overline{\mathrm{EMTreg}} \wedge \overline{\mathrm{miRNA}} \wedge \overline{\mathrm{NICD}} \wedge \mathrm{Notch} \wedge \overline{\mathrm{TP53}} \wedge \mathrm{TP63TP73}) \vee (\overline{\mathrm{DNAdam}} \wedge \overline{\mathrm{miRNA}} \wedge \overline{\mathrm{NICD}} \wedge \mathrm{TP53} \wedge \overline{\mathrm{TP63TP73}})$
$\vee\ (\mathrm{ECM} \wedge \overline{\mathrm{EMTreg}} \wedge \overline{\mathrm{miRNA}} \wedge \mathrm{NICD} \wedge \mathrm{Notch} \wedge \mathrm{TP53}) \vee (\mathrm{ECM} \wedge \overline{\mathrm{EMTreg}} \wedge \overline{\mathrm{miRNA}} \wedge \mathrm{NICD} \wedge \overline{\mathrm{Notch}} \wedge \overline{\mathrm{TP53}}) \vee (\mathrm{ECM} \wedge \overline{\mathrm{EMTreg}} \wedge \overline{\mathrm{miRNA}} \wedge \overline{\mathrm{NICD}} \wedge \mathrm{Notch} \wedge \overline{\mathrm{TP53}} \wedge \overline{\mathrm{TP63TP73}})$
$\vee\ (\mathrm{ECM} \wedge \overline{\mathrm{miRNA}} \wedge \mathrm{NICD} \wedge \overline{\mathrm{Notch}} \wedge \overline{\mathrm{TP53}} \wedge \overline{\mathrm{TP63TP73}}) \vee (\overline{\mathrm{ECM}} \wedge \overline{\mathrm{EMTreg}} \wedge \mathrm{miRNA} \wedge \overline{\mathrm{TP53}} \wedge \overline{\mathrm{TP63TP73}}) \vee (\overline{\mathrm{ECM}} \wedge \overline{\mathrm{EMTreg}} \wedge \mathrm{NICD} \wedge \overline{\mathrm{TP53}} \wedge \overline{\mathrm{TP63TP73}})$
$\vee\ (\overline{\mathrm{ECM}} \wedge \overline{\mathrm{miRNA}} \wedge \mathrm{TP53} \wedge \overline{\mathrm{TP63TP73}}) \vee (\mathrm{EMTreg} \wedge \mathrm{miRNA} \wedge \overline{\mathrm{NICD}} \wedge \overline{\mathrm{Notch}} \wedge \overline{\mathrm{TP63TP73}}) \vee (\mathrm{EMTreg} \wedge \mathrm{miRNA} \wedge \overline{\mathrm{Notch}} \wedge \mathrm{TP53} \wedge \overline{\mathrm{TP63TP73}})$
$\vee\ (\mathrm{EMTreg} \wedge \overline{\mathrm{NICD}} \wedge \mathrm{Notch} \wedge \mathrm{TP53} \wedge \overline{\mathrm{TP63TP73}}) \vee (\overline{\mathrm{EMTreg}} \wedge \overline{\mathrm{miRNA}} \wedge \mathrm{NICD} \wedge \overline{\mathrm{Notch}} \wedge \overline{\mathrm{TP63TP73}}) \vee (\mathrm{miRNA} \wedge \mathrm{NICD} \wedge \mathrm{Notch} \wedge \overline{\mathrm{TP53}} \wedge \overline{\mathrm{TP63TP73}})$
$\vee\ (\overline{\mathrm{miRNA}} \wedge \mathrm{NICD} \wedge \mathrm{Notch} \wedge \mathrm{TP53} \wedge \overline{\mathrm{TP63TP73}})$

$\mathrm{miRNA}^{*} = (\mathrm{DNAdam} \wedge \mathrm{ECM} \wedge \mathrm{EMTreg} \wedge \mathrm{NICD} \wedge \mathrm{Notch} \wedge \mathrm{TP53} \wedge \overline{\mathrm{TP63TP73}}) \vee (\mathrm{DNAdam} \wedge \mathrm{ECM} \wedge \mathrm{EMTreg} \wedge \mathrm{NICD} \wedge \mathrm{Notch} \wedge \overline{\mathrm{TP53}} \wedge \mathrm{TP63TP73})$
$\vee\ (\mathrm{DNAdam} \wedge \mathrm{ECM} \wedge \mathrm{EMTreg} \wedge \overline{\mathrm{NICD}} \wedge \mathrm{Notch} \wedge \overline{\mathrm{TP53}} \wedge \overline{\mathrm{TP63TP73}}) \vee (\mathrm{DNAdam} \wedge \mathrm{ECM} \wedge \overline{\mathrm{NICD}} \wedge \overline{\mathrm{Notch}} \wedge \mathrm{TP53} \wedge \overline{\mathrm{TP63TP73}}) \vee (\mathrm{DNAdam} \wedge \overline{\mathrm{ECM}} \wedge \overline{\mathrm{miRNA}})$
$\vee\ (\mathrm{DNAdam} \wedge \overline{\mathrm{EMTreg}} \wedge \overline{\mathrm{miRNA}} \wedge \overline{\mathrm{TP53}}) \vee (\mathrm{DNAdam} \wedge \overline{\mathrm{miRNA}} \wedge \mathrm{TP63TP73}) \vee (\overline{\mathrm{DNAdam}} \wedge \mathrm{ECM} \wedge \mathrm{EMTreg} \wedge \mathrm{NICD} \wedge \overline{\mathrm{Notch}} \wedge \overline{\mathrm{TP53}} \wedge \overline{\mathrm{TP63TP73}})$
$\vee\ (\overline{\mathrm{DNAdam}} \wedge \mathrm{ECM} \wedge \overline{\mathrm{EMTreg}} \wedge \mathrm{miRNA} \wedge \overline{\mathrm{NICD}} \wedge \overline{\mathrm{Notch}} \wedge \overline{\mathrm{TP53}}) \vee (\overline{\mathrm{DNAdam}} \wedge \mathrm{ECM} \wedge \overline{\mathrm{EMTreg}} \wedge \overline{\mathrm{NICD}} \wedge \mathrm{TP53} \wedge \overline{\mathrm{TP63TP73}}) \vee (\overline{\mathrm{DNAdam}} \wedge \mathrm{ECM} \wedge \overline{\mathrm{miRNA}} \wedge \overline{\mathrm{NICD}} \wedge \mathrm{TP53} \wedge \overline{\mathrm{TP63TP73}})$
$\vee\ (\overline{\mathrm{DNAdam}} \wedge \overline{\mathrm{ECM}} \wedge \mathrm{EMTreg} \wedge \mathrm{miRNA} \wedge \mathrm{NICD} \wedge \mathrm{Notch} \wedge \overline{\mathrm{TP53}}) \vee (\overline{\mathrm{DNAdam}} \wedge \overline{\mathrm{ECM}} \wedge \mathrm{EMTreg} \wedge \mathrm{NICD} \wedge \overline{\mathrm{Notch}} \wedge \overline{\mathrm{TP53}} \wedge \mathrm{TP63TP73})$
$\vee\ (\overline{\mathrm{DNAdam}} \wedge \overline{\mathrm{ECM}} \wedge \mathrm{NICD} \wedge \overline{\mathrm{Notch}} \wedge \mathrm{TP53} \wedge \overline{\mathrm{TP63TP73}}) \vee (\overline{\mathrm{DNAdam}} \wedge \overline{\mathrm{EMTreg}} \wedge \mathrm{miRNA} \wedge \mathrm{TP53} \wedge \overline{\mathrm{TP63TP73}}) \vee (\mathrm{ECM} \wedge \overline{\mathrm{EMTreg}} \wedge \mathrm{miRNA} \wedge \mathrm{NICD} \wedge \mathrm{TP53} \wedge \overline{\mathrm{TP63TP73}})$
$\vee\ (\mathrm{ECM} \wedge \overline{\mathrm{EMTreg}} \wedge \overline{\mathrm{miRNA}} \wedge \overline{\mathrm{NICD}} \wedge \mathrm{TP53} \wedge \overline{\mathrm{TP63TP73}}) \vee (\mathrm{ECM} \wedge \mathrm{miRNA} \wedge \mathrm{NICD} \wedge \mathrm{Notch} \wedge \mathrm{TP53} \wedge \overline{\mathrm{TP63TP73}}) \vee (\mathrm{ECM} \wedge \overline{\mathrm{miRNA}} \wedge \mathrm{NICD} \wedge \mathrm{TP63TP73})$
$\vee\ (\overline{\mathrm{ECM}} \wedge \mathrm{EMTreg} \wedge \overline{\mathrm{miRNA}} \wedge \mathrm{NICD} \wedge \mathrm{TP53}) \vee (\overline{\mathrm{ECM}} \wedge \overline{\mathrm{EMTreg}} \wedge \overline{\mathrm{miRNA}} \wedge \mathrm{NICD} \wedge \overline{\mathrm{TP53}}) \vee (\overline{\mathrm{ECM}} \wedge \overline{\mathrm{EMTreg}} \wedge \overline{\mathrm{miRNA}} \wedge \mathrm{Notch} \wedge \overline{\mathrm{TP53}}) \vee (\overline{\mathrm{ECM}} \wedge \mathrm{miRNA} \wedge \mathrm{TP53} \wedge \mathrm{TP63TP73})$
$\vee\ (\mathrm{EMTreg} \wedge \overline{\mathrm{miRNA}} \wedge \overline{\mathrm{NICD}} \wedge \overline{\mathrm{TP53}} \wedge \mathrm{TP63TP73}) \vee (\mathrm{EMTreg} \wedge \overline{\mathrm{miRNA}} \wedge \overline{\mathrm{Notch}} \wedge \mathrm{TP53} \wedge \overline{\mathrm{TP63TP73}}) \vee (\overline{\mathrm{EMTreg}} \wedge \overline{\mathrm{miRNA}} \wedge \mathrm{NICD} \wedge \overline{\mathrm{Notch}} \wedge \overline{\mathrm{TP53}})$
$\vee\ (\overline{\mathrm{EMTreg}} \wedge \overline{\mathrm{miRNA}} \wedge \overline{\mathrm{Notch}} \wedge \mathrm{TP53} \wedge \mathrm{TP63TP73}) \vee (\overline{\mathrm{EMTreg}} \wedge \overline{\mathrm{NICD}} \wedge \overline{\mathrm{Notch}} \wedge \mathrm{TP53} \wedge \overline{\mathrm{TP63TP73}}) \vee (\overline{\mathrm{miRNA}} \wedge \mathrm{NICD} \wedge \mathrm{TP53} \wedge \mathrm{TP63TP73})$
$\vee\ (\overline{\mathrm{miRNA}} \wedge \overline{\mathrm{NICD}} \wedge \mathrm{Notch} \wedge \overline{\mathrm{TP53}} \wedge \mathrm{TP63TP73})$

$\mathrm{EMTreg}^{*} = (\mathrm{DNAdam} \wedge \mathrm{ECM} \wedge \mathrm{miRNA} \wedge \overline{\mathrm{NICD}} \wedge \mathrm{Notch} \wedge \overline{\mathrm{TP53}} \wedge \overline{\mathrm{TP63TP73}}) \vee (\mathrm{DNAdam} \wedge \mathrm{ECM} \wedge \overline{\mathrm{miRNA}} \wedge \mathrm{NICD} \wedge \overline{\mathrm{TP53}}) \vee (\mathrm{DNAdam} \wedge \overline{\mathrm{ECM}} \wedge \overline{\mathrm{EMTreg}} \wedge \overline{\mathrm{miRNA}})$
$\vee\ (\mathrm{DNAdam} \wedge \overline{\mathrm{ECM}} \wedge \overline{\mathrm{EMTreg}} \wedge \mathrm{NICD}) \vee (\mathrm{DNAdam} \wedge \overline{\mathrm{ECM}} \wedge \overline{\mathrm{EMTreg}} \wedge \mathrm{Notch}) \vee (\mathrm{DNAdam} \wedge \overline{\mathrm{EMTreg}} \wedge \overline{\mathrm{Notch}} \wedge \overline{\mathrm{TP53}}) \vee (\mathrm{DNAdam} \wedge \overline{\mathrm{EMTreg}} \wedge \mathrm{TP53} \wedge \mathrm{TP63TP73})$
$\vee\ (\overline{\mathrm{DNAdam}} \wedge \mathrm{ECM} \wedge \overline{\mathrm{EMTreg}} \wedge \overline{\mathrm{miRNA}} \wedge \mathrm{Notch} \wedge \overline{\mathrm{TP63TP73}}) \vee (\overline{\mathrm{DNAdam}} \wedge \mathrm{ECM} \wedge \overline{\mathrm{EMTreg}} \wedge \overline{\mathrm{NICD}} \wedge \overline{\mathrm{TP53}} \wedge \mathrm{TP63TP73})$
$\vee\ (\overline{\mathrm{DNAdam}} \wedge \mathrm{ECM} \wedge \mathrm{miRNA} \wedge \mathrm{NICD} \wedge \mathrm{Notch} \wedge \mathrm{TP53} \wedge \mathrm{TP63TP73}) \vee (\overline{\mathrm{DNAdam}} \wedge \overline{\mathrm{ECM}} \wedge \mathrm{EMTreg} \wedge \mathrm{miRNA} \wedge \mathrm{NICD} \wedge \mathrm{TP53} \wedge \overline{\mathrm{TP63TP73}})$
$\vee\ (\overline{\mathrm{DNAdam}} \wedge \overline{\mathrm{ECM}} \wedge \mathrm{miRNA} \wedge \overline{\mathrm{NICD}} \wedge \mathrm{Notch} \wedge \mathrm{TP53} \wedge \mathrm{TP63TP73}) \vee (\overline{\mathrm{DNAdam}} \wedge \overline{\mathrm{ECM}} \wedge \overline{\mathrm{miRNA}} \wedge \mathrm{NICD} \wedge \overline{\mathrm{Notch}} \wedge \overline{\mathrm{TP53}} \wedge \overline{\mathrm{TP63TP73}})$
$\vee\ (\overline{\mathrm{DNAdam}} \wedge \overline{\mathrm{ECM}} \wedge \overline{\mathrm{miRNA}} \wedge \overline{\mathrm{NICD}} \wedge \mathrm{Notch} \wedge \overline{\mathrm{TP53}}) \vee (\overline{\mathrm{DNAdam}} \wedge \mathrm{EMTreg} \wedge \mathrm{miRNA} \wedge \overline{\mathrm{Notch}} \wedge \mathrm{TP53} \wedge \overline{\mathrm{TP63TP73}}) \vee (\overline{\mathrm{DNAdam}} \wedge \mathrm{EMTreg} \wedge \overline{\mathrm{miRNA}} \wedge \mathrm{Notch} \wedge \overline{\mathrm{TP53}} \wedge \overline{\mathrm{TP63TP73}})$
$\vee\ (\overline{\mathrm{DNAdam}} \wedge \overline{\mathrm{miRNA}} \wedge \overline{\mathrm{NICD}} \wedge \mathrm{Notch} \wedge \overline{\mathrm{TP53}} \wedge \overline{\mathrm{TP63TP73}}) \vee (\mathrm{ECM} \wedge \overline{\mathrm{miRNA}} \wedge \mathrm{NICD} \wedge \mathrm{Notch} \wedge \overline{\mathrm{TP53}} \wedge \overline{\mathrm{TP63TP73}}) \vee (\overline{\mathrm{ECM}} \wedge \overline{\mathrm{EMTreg}} \wedge \mathrm{miRNA} \wedge \overline{\mathrm{TP53}})$
$\vee\ (\overline{\mathrm{ECM}} \wedge \overline{\mathrm{EMTreg}} \wedge \mathrm{NICD} \wedge \mathrm{TP63TP73}) \vee (\overline{\mathrm{ECM}} \wedge \overline{\mathrm{EMTreg}} \wedge \mathrm{TP53} \wedge \mathrm{TP63TP73}) \vee (\overline{\mathrm{EMTreg}} \wedge \mathrm{miRNA} \wedge \mathrm{NICD} \wedge \overline{\mathrm{TP53}}) \vee (\overline{\mathrm{EMTreg}} \wedge \mathrm{miRNA} \wedge \overline{\mathrm{Notch}} \wedge \overline{\mathrm{TP53}})$
$\vee\ (\overline{\mathrm{EMTreg}} \wedge \mathrm{miRNA} \wedge \mathrm{TP63TP73}) \vee (\overline{\mathrm{EMTreg}} \wedge \overline{\mathrm{miRNA}} \wedge \mathrm{NICD} \wedge \mathrm{TP53}) \vee (\overline{\mathrm{EMTreg}} \wedge \mathrm{NICD} \wedge \mathrm{Notch} \wedge \mathrm{TP63TP73}) \vee (\overline{\mathrm{EMTreg}} \wedge \mathrm{NICD} \wedge \overline{\mathrm{Notch}} \wedge \overline{\mathrm{TP53}} \wedge \overline{\mathrm{TP63TP73}})$
$\vee\ (\overline{\mathrm{EMTreg}} \wedge \overline{\mathrm{Notch}} \wedge \mathrm{TP53} \wedge \mathrm{TP63TP73})$

$\mathrm{ECM}^{*} = \mathrm{ECM}$

$\mathrm{DNAdam}^{*} = \mathrm{DNAdam}$
