# Supplementary material for: Unsupervised logic-based mechanism inference for network-driven biological processes
Source: PLoS Comput Biol. 2021 Jun 2;17(6):e1009035. doi: 10.1371/journal.pcbi.1009035 (PMC8202945; doi:10.1371/journal.pcbi.1009035)

Transitions to remove

Transitions to remove for NICD:

Transitions for parameter set $(\mathbf{ECM}, \mathbf{DNAdam}) = (0, 0)$

$(0,0,0,0,0,1,0,0)$, $(0,0,0,0,1,0,0,0)$, $(0,0,0,0,1,1,0,0)$, $(0,0,0,1,0,0,0,0)$, $(0,0,0,1,0,1,0,0)$, $(0,0,0,1,1,0,0,0)$, $(0,0,0,1,1,1,0,0)$, $(0,0,1,0,0,0,0,0)$, $(0,0,1,0,0,1,0,0)$, $(0,0,1,0,1,1,0,0)$, $(0,0,1,1,0,0,0,0)$, $(0,0,1,1,0,1,0,0)$, $(0,0,1,1,1,0,0,0)$, $(0,0,1,1,1,1,0,0)$, $(0,1,0,1,0,0,0,0)$, $(0,1,0,1,0,1,0,0)$, $(0,1,0,1,1,0,0,0)$, $(0,1,0,1,1,1,0,0)$, $(0,1,1,0,0,0,0,0)$, $(0,1,1,0,0,1,0,0)$, $(0,1,1,0,1,0,0,0)$, $(0,1,1,0,1,1,0,0)$, $(0,1,1,1,0,0,0,0)$, $(0,1,1,1,0,1,0,0)$, $(0,1,1,1,1,0,0,0)$, $(0,1,1,1,1,1,0,0)$, $(1,1,0,0,0,0,0,0)$, $(1,1,0,0,1,0,0,0)$, $(1,1,0,0,1,1,0,0)$

Transitions for parameter set $(\mathbf{ECM}, \mathbf{DNAdam}) = (0, 1)$

$(0,0,0,0,0,0,0,1)$, $(0,0,0,0,0,1,0,1)$, $(0,0,0,0,1,0,0,1)$, $(0,0,0,0,1,1,0,1)$, $(0,0,0,1,0,0,0,1)$, $(0,0,0,1,0,1,0,1)$, $(0,0,0,1,1,0,0,1)$, $(0,0,0,1,1,1,0,1)$, $(0,0,1,0,0,0,0,1)$, $(0,0,1,0,0,1,0,1)$, $(0,0,1,0,1,1,0,1)$, $(0,0,1,1,0,0,0,1)$, $(0,0,1,1,0,1,0,1)$, $(0,0,1,1,1,0,0,1)$, $(0,0,1,1,1,1,0,1)$, $(0,1,0,1,0,0,0,1)$, $(0,1,0,1,0,1,0,1)$, $(0,1,0,1,1,0,0,1)$, $(0,1,0,1,1,1,0,1)$, $(0,1,1,0,0,0,0,1)$, $(0,1,1,0,0,1,0,1)$, $(0,1,1,0,1,0,0,1)$, $(0,1,1,0,1,1,0,1)$, $(0,1,1,1,0,0,0,1)$, $(0,1,1,1,0,1,0,1)$, $(0,1,1,1,1,0,0,1)$, $(0,1,1,1,1,1,0,1)$, $(1,1,0,0,0,0,0,1)$, $(1,1,0,0,0,1,0,1)$, $(1,1,0,0,1,0,0,1)$, $(1,1,0,0,1,1,0,1)$

Transitions for parameter set $(\mathbf{ECM}, \mathbf{DNAdam}) = (1, 0)$

$(0,0,0,0,0,0,1,0)$, $(0,0,0,0,0,1,1,0)$, $(0,0,0,0,1,0,1,0)$, $(0,0,0,0,1,1,1,0)$, $(0,0,0,1,0,0,1,0)$, $(0,0,0,1,0,1,1,0)$, $(0,0,0,1,1,0,1,0)$, $(0,0,0,1,1,1,1,0)$, $(0,0,1,0,0,0,1,0)$, $(0,0,1,0,0,1,1,0)$, $(0,0,1,0,1,1,1,0)$, $(0,0,1,1,0,0,1,0)$, $(0,0,1,1,0,1,1,0)$, $(0,0,1,1,1,0,1,0)$, $(0,0,1,1,1,1,1,0)$, $(0,1,0,1,0,0,1,0)$, $(0,1,0,1,0,1,1,0)$, $(0,1,0,1,1,0,1,0)$, $(0,1,0,1,1,1,1,0)$, $(0,1,1,0,0,0,1,0)$, $(0,1,1,0,0,1,1,0)$, $(0,1,1,0,1,0,1,0)$, $(0,1,1,0,1,1,1,0)$, $(0,1,1,1,0,0,1,0)$, $(0,1,1,1,0,1,1,0)$, $(0,1,1,1,1,0,1,0)$, $(0,1,1,1,1,1,1,0)$, $(1,1,0,0,0,0,1,0)$, $(1,1,0,0,1,0,1,0)$, $(1,1,0,0,1,1,1,0)$

Transitions for parameter set $(\mathbf{ECM}, \mathbf{DNAdam}) = (1, 1)$

$(0,0,0,0,0,0,1,1)$, $(0,0,0,0,0,1,1,1)$, $(0,0,0,0,1,0,1,1)$, $(0,0,0,0,1,1,1,1)$, $(0,0,0,1,0,0,1,1)$, $(0,0,0,1,0,1,1,1)$, $(0,0,0,1,1,1,1,1)$, $(0,0,0,1,1,0,1,1)$, $(0,0,1,0,0,1,1,1)$, $(0,0,1,0,1,1,1,1)$, $(0,0,1,1,0,0,1,1)$, $(0,0,1,1,0,1,1,1)$, $(0,0,1,1,1,0,1,1)$, $(0,0,1,1,1,1,1,1)$, $(0,1,0,1,0,0,1,1)$, $(0,1,0,1,0,1,1,1)$, $(0,1,0,1,1,0,1,1)$, $(0,1,0,1,1,1,1,1)$, $(0,1,1,0,0,1,1,1)$, $(0,1,1,0,1,0,1,1)$ $(0,1,1,0,1,1,1,1)$, $(0,1,1,1,0,0,1,1)$, $(0,1,1,1,0,1,1,1)$, $(0,1,1,1,1,0,1,1)$, $(0,1,1,1,1,1,1,1)$, $(1,1,0,0,0,0,1,1)$, $(1,1,0,0,1,0,1,1)$, $(1,1,0,0,1,1,1,1)$

Transitions to remove for Notch:

Transitions for parameter set $(\mathbf{ECM}, \mathbf{DNAdam}) = (0, 0)$

$(0,0,0,0,1,0,0,0)$, $(0,0,0,0,1,1,0,0)$, $(0,0,0,1,0,0,0,0)$, $(0,0,0,1,0,1,0,0)$, $(0,0,0,1,1,0,0,0)$, $(0,0,0,1,1,1,0,0)$, $(0,0,1,0,0,0,0,0)$, $(0,0,1,0,0,1,0,0)$, $(0,0,1,0,1,1,0,0)$, $(0,0,1,1,0,0,0,0)$, $(0,0,1,1,0,1,0,0)$, $(0,0,1,1,1,0,0,0)$, $(0,0,1,1,1,1,0,0)$, $(1,0,0,0,0,0,0,0)$, $(1,0,0,0,0,1,0,0)$, $(1,0,0,0,1,0,0,0)$, $(1,0,0,0,1,1,0,0)$, $(1,0,0,1,0,0,0,0)$, $(1,0,0,1,0,1,0,0)$, $(1,0,0,1,1,0,0,0)$, $(1,0,0,1,1,1,0,0)$, $(1,0,1,0,0,0,0,0)$, $(1,0,1,0,0,1,0,0)$, $(1,0,1,0,1,0,0,0)$, $(1,0,1,0,1,1,0,0)$, $(1,0,1,1,0,0,0,0)$, $(1,0,1,1,0,1,0,0)$, $(1,0,1,1,1,0,0,0)$, $(1,0,1,1,1,1,0,0)$

Transitions for parameter set $(\mathbf{ECM}, \mathbf{DNAdam}) = (0, 1)$

$(0,0,0,0,0,0,0,1)$, $(0,0,0,0,0,1,0,1)$, $(0,0,0,0,1,0,0,1)$, $(0,0,0,0,1,1,0,1)$, $(0,0,0,1,0,0,0,1)$, $(0,0,0,1,0,1,0,1)$, $(0,0,0,1,1,0,0,1)$, $(0,0,0,1,1,1,0,1)$, $(0,0,1,0,0,0,0,1)$, $(0,0,1,0,0,1,0,1)$, $(0,0,1,0,1,1,0,1)$, $(0,0,1,1,0,0,0,1)$, $(0,0,1,1,0,1,0,1)$, $(0,0,1,1,1,0,0,1)$, $(0,0,1,1,1,1,0,1)$, $(1,0,0,0,0,0,0,1)$, $(1,0,0,0,0,1,0,1)$, $(1,0,0,0,1,0,0,1)$, $(1,0,0,0,1,1,0,1)$, $(1,0,0,1,0,0,0,1)$, $(1,0,0,1,0,1,0,1)$, $(1,0,0,1,1,1,0,1)$, $(1,0,0,1,1,0,0,1)$, $(1,0,1,0,0,0,0,1)$, $(1,0,1,0,0,1,0,1)$, $(1,0,1,0,1,0,0,1)$, $(1,0,1,0,1,1,0,1)$, $(1,0,1,1,0,0,0,1)$, $(1,0,1,1,0,1,0,1)$, $(1,0,1,1,1,0,0,1)$, $(1,0,1,1,1,1,0,1)$

Transitions for parameter set $(\mathbf{ECM}, \mathbf{DNAdam}) = (1, 0)$

$(0,0,0,0,1,0,1,0)$, $(0,0,0,0,1,1,1,0)$, $(0,0,0,1,1,0,1,0)$, $(0,0,0,1,1,1,1,0)$, $(0,0,1,0,1,1,1,0)$, $(0,0,1,1,1,0,1,0)$, $(0,0,1,1,1,1,1,0)$, $(0,1,0,0,0,0,1,0)$, $(0,1,0,0,0,1,1,0)$, $(0,1,0,1,0,0,1,0)$, $(0,1,0,1,0,1,1,0)$, $(0,1,1,0,0,0,1,0)$, $(0,1,1,0,0,1,1,0)$, $(0,1,1,1,0,0,1,0)$, $(0,1,1,1,0,1,1,0)$, $(1,0,0,0,1,0,1,0)$, $(1,0,0,0,1,1,1,0)$, $(1,0,0,1,1,0,1,0)$, $(1,0,0,1,1,1,1,0)$, $(1,0,1,0,1,0,1,0)$, $(1,0,1,0,1,1,1,0)$, $(1,0,1,1,1,0,1,0)$, $(1,0,1,1,1,1,1,0)$, $(1,1,0,0,0,0,1,0)$, $(1,1,0,1,0,0,1,0)$, $(1,1,0,1,0,1,1,0)$, $(1,1,1,0,0,0,1,0)$, $(1,1,1,0,0,1,1,0)$, $(1,1,1,1,0,0,1,0)$, $(1,1,1,1,0,1,1,0)$

Transitions for parameter set $(\mathbf{ECM}, \mathbf{DNAdam}) = (1, 1)$

$(0,0,0,0,1,0,1,1)$, $(0,0,0,0,1,1,1,1)$, $(0,0,0,1,1,0,1,1)$, $(0,0,0,1,1,1,1,1)$, $(0,0,1,0,1,1,1,1)$, $(0,0,1,1,1,0,1,1)$, $(0,0,1,1,1,1,1,1)$, $(0,1,0,0,0,0,1,1)$, $(0,1,0,0,0,1,1,1)$, $(0,1,0,1,0,0,1,1)$, $(0,1,0,1,0,1,1,1)$, $(0,1,1,0,0,0,1,1)$, $(0,1,1,0,0,1,1,1)$, $(0,1,1,1,0,0,1,1)$, $(0,1,1,1,0,1,1,1)$, $(1,0,0,0,1,0,1,1)$, $(1,0,0,0,1,1,1,1)$, $(1,0,0,1,1,0,1,1)$, $(1,0,0,1,1,1,1,1)$, $(1,0,1,0,1,0,1,1)$, $(1,0,1,0,1,1,1,1)$, $(1,0,1,1,1,0,1,1)$, $(1,0,1,1,1,1,1,1)$, $(1,1,0,0,0,0,1,1)$, $(1,1,0,1,0,0,1,1)$, $(1,1,0,1,0,1,1,1)$, $(1,1,1,0,0,0,1,1)$, $(1,1,1,0,0,1,1,1)$, $(1,1,1,1,0,0,1,1)$, $(1,1,1,1,0,1,1,1)$

Transitions to remove for TP53:

Transitions for parameter set $(\mathbf{ECM}, \mathbf{DNAdam}) = (0, 0)$

$(0,0,0,0,1,1,0,0)$, $(0,0,0,1,0,0,0,0)$, $(0,0,0,1,0,1,0,0)$, $(0,0,0,1,1,0,0,0)$, $(0,0,0,1,1,1,0,0)$, $(0,1,0,0,0,0,0,0)$, $(0,1,0,0,0,1,0,0)$, $(0,1,0,0,1,1,0,0)$, $(0,1,0,1,0,0,0,0)$, $(0,1,0,1,0,1,0,0)$, $(0,1,0,1,1,0,0,0)$, $(0,1,0,1,1,1,0,0)$, $(1,0,0,0,1,1,0,0)$, $(1,0,0,1,0,0,0,0)$, $(1,0,0,1,0,1,0,0)$, $(1,0,0,1,1,0,0,0)$, $(1,0,0,1,1,1,0,0)$, $(1,0,1,0,0,0,0,0)$, $(1,1,0,0,1,1,0,0)$, $(1,1,0,1,0,0,0,0)$, $(1,1,0,1,1,0,0,0)$, $(1,1,0,1,0,1,0,0)$, $(1,1,0,1,1,1,0,0)$, $(1,1,1,0,0,0,0,0)$

Transitions for parameter set $(\mathbf{ECM}, \mathbf{DNAdam}) = (0, 1)$

$(0,0,0,0,0,1,0,1)$, $(0,0,0,0,1,1,0,1)$, $(0,0,0,1,0,0,0,1)$, $(0,0,0,1,0,1,0,1)$, $(0,0,0,1,1,0,0,1)$, $(0,0,0,1,1,1,0,1)$, $(0,0,1,0,0,0,0,1)$, $(0,1,0,0,0,1,0,1)$, $(0,1,0,0,1,1,0,1)$, $(0,1,0,1,0,0,0,1)$, $(0,1,0,1,0,1,0,1)$, $(0,1,0,1,1,0,0,1)$, $(0,1,0,1,1,1,0,1)$, $(0,1,1,0,0,0,0,1)$, $(0,1,1,0,1,0,0,1)$, $(1,0,0,0,0,1,0,1)$, $(1,0,0,0,1,1,0,1)$, $(1,0,0,1,0,0,0,1)$, $(1,0,0,1,0,1,0,1)$, $(1,0,0,1,1,0,0,1)$, $(1,0,0,1,1,1,0,1)$, $(1,0,1,0,0,0,0,1)$, $(1,0,1,0,1,0,0,1)$, $(1,1,0,0,0,1,0,1)$, $(1,1,0,0,1,1,0,1)$, $(1,1,0,1,0,0,0,1)$, $(1,1,0,1,0,1,0,1)$, $(1,1,0,1,1,0,0,1)$, $(1,1,0,1,1,1,0,1)$, $(1,1,1,0,0,0,0,1)$, $(1,1,1,0,1,0,0,1)$,

Transitions for parameter set $(\mathbf{ECM}, \mathbf{DNAdam}) = (1, 0)$

$(0,0,0,0,0,0,1,0)$, $(0,0,0,0,0,1,1,0)$, $(0,0,0,0,1,1,1,0)$, $(0,0,0,1,0,0,1,0)$, $(0,0,0,1,0,1,1,0)$, $(0,0,0,1,1,0,1,0)$, $(0,0,0,1,1,1,1,0)$, $(0,1,0,0,0,0,1,0)$, $(0,1,0,0,0,1,1,0)$, $(0,1,0,0,1,1,1,0)$, $(0,1,0,1,0,0,1,0)$, $(0,1,0,1,0,1,1,0)$, $(0,1,0,1,1,0,1,0)$, $(0,1,0,1,1,1,1,0)$, $(1,0,0,0,0,1,1,0)$, $(1,0,0,0,1,1,1,0)$, $(1,0,0,1,0,0,1,0)$, $(1,0,0,1,0,1,1,0)$, $(1,0,0,1,1,0,1,0)$, $(1,0,0,1,1,1,1,0)$, $(1,0,1,0,0,0,1,0)$, $(1,1,0,0,1,1,1,0)$, $(1,1,0,1,0,0,1,0)$, $(1,1,0,1,0,1,1,0)$, $(1,1,0,1,1,0,1,0)$, $(1,1,0,1,1,1,1,0)$, $(1,1,1,0,0,0,1,0)$

Transitions for parameter set $(\mathbf{ECM}, \mathbf{DNAdam}) = (1, 1)$

$(0,0,0,0,0,1,1,1)$, $(0,0,0,0,1,1,1,1)$, $(0,0,0,1,0,0,1,1)$, $(0,0,0,1,0,1,1,1)$, $(0,0,0,1,1,0,1,1)$, $(0,0,0,1,1,1,1,1)$, $(0,1,0,0,0,1,1,1)$, $(0,1,0,0,1,1,1,1)$, $(0,1,0,1,0,0,1,1)$, $(0,1,0,1,0,1,1,1)$, $(0,1,0,1,1,0,1,1)$, $(0,1,0,1,1,1,1,1)$, $(1,0,0,0,0,1,1,1)$, $(1,0,0,0,1,1,1,1)$, $(1,0,0,1,0,1,1,1)$, $(1,0,0,1,0,0,1,1)$, $(1,0,0,1,1,0,1,1)$, $(1,0,0,1,1,1,1,1)$, $(1,0,1,0,0,0,1,1)$, $(1,1,0,0,1,1,1,1)$, $(1,1,0,1,0,0,1,1)$, $(1,1,0,1,0,1,1,1)$, $(1,1,0,1,1,0,1,1)$, $(1,1,0,1,1,1,1,1)$, $(1,1,1,0,0,0,1,1)$

Transitions to remove for TP63_TP73:

Transitions for parameter set $(\mathbf{ECM}, \mathbf{DNAdam}) = (0, 0)$

$(0,0,0,0,1,0,0,0)$, $(0,0,0,0,1,1,0,0)$, $(0,0,1,0,0,0,0,0)$, $(0,0,1,0,0,1,0,0)$, $(0,0,1,0,1,1,0,0)$, $(0,1,0,0,0,0,0,0)$, $(0,1,0,0,0,1,0,0)$, $(0,1,0,0,1,0,0,0)$, $(0,1,0,0,1,1,0,0)$, $(0,1,1,0,0,0,0,0)$, $(0,1,1,0,0,1,0,0)$, $(0,1,1,0,1,1,0,0)$, $(1,0,0,0,0,0,0,0)$, $(1,0,0,0,1,0,0,0)$, $(1,0,0,0,1,1,0,0)$, $(1,0,1,0,0,0,0,0)$, $(1,0,1,0,0,1,0,0)$, $(1,0,1,0,1,1,0,0)$, $(1,1,0,0,0,0,0,0)$, $(1,1,0,0,1,0,0,0)$, $(1,1,0,0,1,1,0,0)$, $(1,1,1,0,0,0,0,0)$, $(1,1,1,0,0,1,0,0)$, $(1,1,1,0,1,1,0,0)$

Transitions for parameter set $(\mathbf{ECM}, \mathbf{DNAdam}) = (0, 1)$

$(0,0,0,0,0,0,0,1)$, $(0,0,0,0,1,0,0,1)$, $(0,0,0,0,1,1,0,1)$, $(0,0,0,1,0,0,0,1)$, $(0,0,0,1,0,1,0,1)$, $(0,0,1,0,0,0,0,1)$, $(0,0,1,0,1,1,0,1)$, $(0,0,1,1,0,0,0,1)$, $(0,0,1,1,0,1,0,1)$, $(0,1,0,0,0,0,0,1)$, $(0,1,0,0,1,0,0,1)$, $(0,1,0,0,1,1,0,1)$, $(0,1,0,1,0,0,0,1)$, $(0,1,0,1,0,1,0,1)$, $(0,1,1,0,0,0,0,1)$, $(0,1,1,0,1,0,0,1)$, $(0,1,1,0,1,1,0,1)$, $(0,1,1,1,0,0,0,1)$, $(0,1,1,1,0,1,0,1)$, $(1,0,0,0,0,0,0,1)$, $(1,0,0,0,0,1,0,1)$, $(1,0,0,0,1,0,0,1)$, $(1,0,0,0,1,1,0,1)$, $(1,0,1,0,0,0,0,1)$, $(1,0,1,0,0,1,0,1)$, $(1,0,1,0,1,0,0,1)$, $(1,0,1,0,1,1,0,1)$, $(1,1,0,0,0,0,0,1)$, $(1,1,0,0,0,1,0,1)$, $(1,1,0,0,1,0,0,1)$, $(1,1,0,0,1,1,0,1)$, $(1,1,1,0,0,0,0,1)$, $(1,1,1,0,0,1,0,1)$, $(1,1,1,0,1,0,0,1)$, $(1,1,1,0,1,1,0,1)$

Transitions for parameter set $(\mathbf{ECM}, \mathbf{DNAdam}) = (1, 0)$

$(0,0,0,0,0,0,1,0)$, $(0,0,0,0,0,1,1,0)$, $(0,0,0,0,1,0,1,0)$, $(0,0,0,0,1,1,1,0)$, $(0,0,1,0,0,0,1,0)$, $(0,0,1,0,0,1,1,0)$, $(0,0,1,0,1,1,1,0)$, $(0,1,0,0,0,0,1,0)$, $(0,1,0,0,0,1,1,0)$, $(0,1,0,0,1,0,1,0)$, $(0,1,0,0,1,1,1,0)$, $(0,1,1,0,0,0,1,0)$, $(0,1,1,0,0,1,1,0)$, $(0,1,1,0,1,1,1,0)$, $(1,0,0,0,0,0,1,0)$, $(1,0,0,0,0,1,1,0)$, $(1,0,0,0,1,0,1,0)$, $(1,0,0,0,1,1,1,0)$, $(1,0,1,0,0,0,1,0)$, $(1,0,1,0,0,1,1,0)$, $(1,0,1,0,1,1,1,0)$, $(1,1,0,0,0,0,1,0)$, $(1,1,0,0,1,0,1,0)$, $(1,1,0,0,1,1,1,0)$, $(1,1,1,0,0,0,1,0)$, $(1,1,1,0,0,1,1,0)$, $(1,1,1,0,1,1,1,0)$

Transitions for parameter set (ECM, DNAdam) = (1, 1)

(0, 0, 0, 0, 0, 0, 1, 1), (0, 0, 0, 0, 1, 0, 1, 1), (0, 0, 0, 0, 1, 1, 1, 1), (0, 0, 0, 1, 0, 0, 1, 1), (0, 0, 0, 1, 0, 1, 1, 1), (0, 0, 1, 0, 1, 1, 1, 1), (0, 0, 1, 1, 0, 0, 1, 1), (0, 0, 1, 1, 0, 1, 1, 1), (0, 1, 0, 0, 0, 0, 1, 1), (0, 1, 0, 0, 1, 0, 1, 1), (0, 1, 0, 0, 1, 1, 1, 1), (0, 1, 0, 1, 0, 0, 1, 1), (0, 1, 0, 1, 0, 1, 1, 1), (0, 1, 1, 0, 1, 1, 1, 1), (0, 1, 1, 1, 0, 0, 1, 1), (0, 1, 1, 1, 0, 1, 1, 1), (1, 0, 0, 0, 0, 0, 1, 1), (1, 0, 0, 0, 0, 1, 1, 1), (1, 0, 0, 0, 1, 0, 1, 1), (1, 0, 0, 0, 1, 1, 1, 1), (1, 0, 1, 0, 0, 0, 1, 1), (1, 0, 1, 0, 0, 1, 1, 1), (1, 0, 1, 0, 1, 1, 1, 1), (1, 1, 0, 0, 0, 0, 1, 1), (1, 1, 0, 0, 1, 1, 1, 1), (1, 1, 0, 0, 1, 0, 1, 1), (1, 1, 1, 0, 0, 0, 1, 1), (1, 1, 1, 0, 0, 1, 1, 1), (1, 1, 1, 0, 1, 1, 1, 1)

Transitions to remove for miRNA:

Transitions for parameter set (ECM, DNAdam) = (0, 0)

(0, 0, 0, 0, 1, 0, 0, 0), (0, 0, 0, 1, 0, 0, 0, 0), (0, 0, 0, 1, 0, 1, 0, 0), (0, 0, 1, 0, 0, 0, 0, 0), (0, 0, 1, 0, 0, 1, 0, 0), (0, 0, 1, 1, 0, 0, 0, 0), (0, 0, 1, 1, 0, 1, 0, 0), (0, 1, 0, 0, 0, 0, 0, 0), (0, 1, 0, 0, 0, 1, 0, 0), (0, 1, 0, 0, 1, 0, 0, 0), (0, 1, 0, 1, 0, 0, 0, 0), (0, 1, 0, 1, 0, 1, 0, 0), (0, 1, 1, 0, 0, 0, 0, 0), (0, 1, 1, 0, 0, 1, 0, 0), (0, 1, 1, 1, 0, 0, 0, 0), (0, 1, 1, 1, 0, 1, 0, 0), (1, 0, 0, 0, 1, 0, 0, 0), (1, 0, 0, 1, 0, 0, 0, 0), (1, 0, 0, 1, 0, 1, 0, 0), (1, 0, 1, 0, 0, 1, 0, 0), (1, 0, 1, 1, 0, 0, 0, 0), (1, 0, 1, 1, 0, 1, 0, 0), (1, 1, 0, 0, 1, 0, 0, 0), (1, 1, 0, 1, 0, 0, 0, 0), (1, 1, 0, 1, 0, 1, 0, 0), (1, 1, 1, 0, 0, 1, 0, 0), (1, 1, 1, 1, 0, 0, 0, 0), (1, 1, 1, 1, 0, 1, 0, 0)

Transitions for parameter set (ECM, DNAdam) = (0, 1)

(0, 0, 0, 0, 0, 1, 0, 1), (0, 0, 0, 0, 1, 0, 0, 1), (0, 0, 0, 1, 0, 1, 0, 1), (0, 0, 1, 0, 0, 1, 0, 1), (0, 0, 1, 1, 0, 1, 0, 1), (0, 1, 0, 0, 0, 1, 0, 1), (0, 1, 0, 0, 1, 0, 0, 1), (0, 1, 0, 1, 0, 1, 0, 1), (0, 1, 1, 0, 0, 1, 0, 1), (0, 1, 1, 0, 1, 0, 0, 1), (0, 1, 1, 1, 0, 1, 0, 1), (1, 0, 0, 0, 0, 1, 0, 1), (1, 0, 0, 0, 1, 0, 0, 1), (1, 0, 0, 1, 0, 0, 0, 1), (1, 0, 0, 1, 0, 1, 0, 1), (1, 0, 1, 0, 0, 1, 0, 1), (1, 0, 1, 0, 1, 0, 0, 1), (1, 0, 1, 1, 0, 0, 0, 1), (1, 0, 1, 1, 0, 1, 0, 1), (1, 1, 0, 0, 0, 1, 0, 1), (1, 1, 0, 0, 1, 0, 0, 1), (1, 1, 0, 1, 0, 0, 0, 1), (1, 1, 0, 1, 0, 1, 0, 1), (1, 1, 1, 0, 0, 1, 0, 1), (1, 1, 1, 0, 1, 0, 0, 1), (1, 1, 1, 1, 0, 0, 0, 1), (1, 1, 1, 1, 0, 1, 0, 1)

Transitions for parameter set (ECM, DNAdam) = (1, 0)

(0, 0, 0, 0, 0, 0, 1, 0), (0, 0, 0, 0, 0, 1, 1, 0), (0, 0, 0, 0, 1, 0, 1, 0), (0, 0, 0, 1, 0, 0, 1, 0), (0, 0, 0, 1, 0, 1, 1, 0), (0, 0, 1, 1, 0, 1, 1, 0), (0, 0, 1, 0, 0, 0, 1, 0), (0, 0, 1, 0, 0, 1, 1, 0), (0, 0, 1, 1, 0, 0, 1, 0), (0, 1, 0, 0, 0, 1, 1, 0), (0, 1, 0, 0, 0, 0, 1, 0), (0, 1, 0, 0, 1, 0, 1, 0), (0, 1, 0, 1, 0, 0, 1, 0), (0, 1, 0, 1, 0, 1, 1, 0), (0, 1, 1, 0, 0, 0, 1, 0), (0, 1, 1, 0, 0, 1, 1, 0), (0, 1, 1, 1, 0, 0, 1, 0), (0, 1, 1, 1, 0, 1, 1, 0), (1, 0, 0, 0, 0, 1, 1, 0), (1, 0, 0, 0, 1, 0, 1, 0), (1, 0, 0, 1, 0, 0, 1, 0), (1, 0, 0, 1, 0, 1, 1, 0), (1, 0, 1, 0, 0, 1, 1, 0), (1, 0, 1, 1, 0, 1, 1, 0), (1, 0, 1, 1, 0, 0, 1, 0), (1, 1, 0, 0, 1, 0, 1, 0), (1, 1, 0, 1, 0, 0, 1, 0), (1, 1, 0, 1, 0, 1, 1, 0), (1, 1, 1, 0, 0, 1, 1, 0), (1, 1, 1, 1, 0, 0, 1, 0), (1, 1, 1, 1, 0, 1, 1, 0)

Transitions for parameter set (ECM, DNAdam) = (1, 1)

(0, 0, 0, 0, 0, 1, 1, 1), (0, 0, 0, 0, 1, 0, 1, 1), (0, 0, 0, 1, 0, 1, 1, 1), (0, 0, 1, 0, 0, 1, 1, 1), (0, 0, 1, 1, 0, 1, 1, 1), (0, 1, 0, 0, 0, 1, 1, 1), (0, 1, 0, 0, 1, 0, 1, 1), (0, 1, 0, 1, 0, 1, 1, 1), (0, 1, 1, 0, 0, 1, 1, 1), (0, 1, 1, 0, 1, 0, 1, 1), (0, 1, 1, 1, 0, 1, 1, 1), (1, 0, 0, 0, 0, 1, 1, 1), (1, 0, 0, 0, 1, 0, 1, 1), (1, 0, 0, 1, 0, 0, 1, 1), (1, 0, 0, 1, 0, 1, 1, 1), (1, 0, 1, 0, 0, 1, 1, 1), (1, 0, 1, 1, 0, 0, 1, 1), (1, 0, 1, 1, 0, 1, 1, 1), (1, 1, 0, 0, 1, 0, 1, 1), (1, 1, 0, 1, 0, 0, 1, 1), (1, 1, 0, 1, 0, 1, 1, 1), (1, 1, 1, 0, 0, 1, 1, 1), (1, 1, 1, 1, 0, 0, 1, 1), (1, 1, 1, 1, 0, 1, 1, 1)

Transitions to remove for EMTreg:

Transitions for parameter set (ECM, DNAdam) = (0, 0)

(0, 0, 0, 0, 1, 0, 0, 0), (0, 0, 0, 1, 0, 0, 0, 0), (0, 0, 0, 1, 1, 0, 0, 0), (0, 0, 1, 0, 0, 0, 0, 0), (0, 0, 1, 1, 0, 0, 0, 0), (0, 0, 1, 1, 1, 0, 0, 0), (0, 1, 0, 0, 0, 0, 0, 0), (0, 1, 0, 0, 1, 0, 0, 0), (0, 1, 0, 1, 0, 0, 0, 0), (0, 1, 0, 1, 1, 0, 0, 0), (0, 1, 1, 0, 0, 0, 0, 0), (0, 1, 1, 1, 0, 0, 0, 0), (0, 1, 1, 1, 1, 0, 0, 0), (1, 0, 0, 0, 0, 0, 0, 0), (1, 0, 0, 0, 1, 0, 0, 0), (1, 0, 0, 0, 1, 1, 0, 0), (1, 0, 0, 1, 0, 1, 0, 0), (1, 0, 0, 1, 1, 1, 0, 0), (1, 0, 1, 0, 0, 0, 0, 0), (1, 0, 1, 0, 0, 1, 0, 0), (1, 0, 1, 0, 1, 1, 0, 0), (1, 0, 1, 1, 0, 1, 0, 0), (1, 0, 1, 1, 1, 1, 0, 0), (1, 1, 0, 0, 0, 0, 0, 0), (1, 1, 0, 0, 1, 0, 0, 0), (1, 1, 0, 0, 1, 1, 0, 0), (1, 1, 0, 1, 0, 1, 0, 0), (1, 1, 0, 1, 1, 1, 0, 0), (1, 1, 1, 0, 0, 0, 0, 0), (1, 1, 1, 0, 0, 1, 0, 0), (1, 1, 1, 0, 1, 1, 0, 0), (1, 1, 1, 1, 0, 1, 0, 0), (1, 1, 1, 1, 1, 1, 0, 0)

Transitions for parameter set (ECM, DNAdam) = (0, 1)

(0, 0, 0, 0, 0, 0, 0, 1), (0, 0, 0, 0, 1, 0, 0, 1), (0, 0, 0, 1, 0, 0, 0, 1), (0, 0, 0, 1, 1, 0, 0, 1), (0, 0, 1, 0, 0, 0, 0, 1), (0, 0, 1, 1, 0, 0, 0, 1), (0, 0, 1, 1, 1, 0, 0, 1), (0, 1, 0, 0, 0, 0, 0, 1), (0, 1, 0, 0, 1, 0, 0, 1), (0, 1, 0, 1, 0, 0, 0, 1), (0, 1, 0, 1, 1, 0, 0, 1), (0, 1, 1, 0, 0, 0, 0, 1), (0, 1, 1, 0, 1, 0, 0, 1), (0, 1, 1, 1, 0, 0, 0, 1), (0, 1, 1, 1, 1, 0, 0, 1), (1, 0, 0, 0, 0, 0, 0, 1), (1, 0, 0, 0, 0, 1, 0, 1), (1, 0, 0, 0, 1, 0, 0, 1), (1, 0, 0, 0, 1, 1, 0, 1), (1, 0, 0, 1, 0, 1, 0, 1), (1, 0, 0, 1, 1, 1, 0, 1), (1, 0, 1, 0, 0, 0, 0, 1), (1, 0, 1, 0, 0, 1, 0, 1), (1, 0, 1, 0, 1, 0, 0, 1), (1, 0, 1, 0, 1, 1, 0, 1), (1, 0, 1, 1, 0, 1, 0, 1), (1, 0, 1, 1, 1, 1, 0, 1), (1, 1, 0, 0, 0, 0, 0, 1), (1, 1, 0, 0, 0, 1, 0, 1), (1, 1, 0, 0, 1, 0, 0, 1), (1, 1, 0, 0, 1, 1, 0, 1), (1, 1, 0, 1, 0, 1, 0, 1), (1, 1, 0, 1, 1, 1, 0, 1), (1, 1, 1, 0, 0, 0, 0, 1), (1, 1, 1, 0, 0, 1, 0, 1), (1, 1, 1, 0, 1, 0, 0, 1), (1, 1, 1, 0, 1, 1, 0, 1), (1, 1, 1, 1, 0, 1, 0, 1), (1, 1, 1, 1, 1, 1, 0, 1)

Transitions for parameter set $(\mathbf{ECM}, \mathbf{DNAdam}) = (1, 0)$

$(0,0,0,0,0,0,1,0)$, $(0,0,0,0,1,0,1,0)$, $(0,0,0,1,0,0,1,0)$, $(0,0,0,1,1,0,1,0)$, $(0,0,1,0,0,0,1,0)$, $(0,0,1,1,0,0,1,0)$, $(0,0,1,1,1,0,1,0)$, $(0,1,0,0,0,0,1,0)$, $(0,1,0,0,1,0,1,0)$, $(0,1,0,1,0,0,1,0)$, $(0,1,0,1,1,0,1,0)$, $(0,1,1,0,0,0,1,0)$, $(0,1,1,1,0,0,1,0)$, $(0,1,1,1,1,0,1,0)$, $(1,0,0,0,0,0,1,0)$, $(1,0,0,0,0,1,1,0)$, $(1,0,0,0,1,0,1,0)$, $(1,0,0,0,1,1,1,0)$, $(1,0,0,1,0,1,1,0)$, $(1,0,0,1,1,1,1,0)$, $(1,0,1,0,0,0,1,0)$, $(1,0,1,0,0,1,1,0)$, $(1,0,1,0,1,1,1,0)$, $(1,0,1,1,0,1,1,0)$, $(1,0,1,1,1,1,1,0)$, $(1,1,0,0,0,0,1,0)$, $(1,1,0,0,1,0,1,0)$, $(1,1,0,0,1,1,1,0)$, $(1,1,0,1,0,1,1,0)$, $(1,1,0,1,1,1,1,0)$, $(1,1,1,0,0,0,1,0)$, $(1,1,1,0,0,1,1,0)$, $(1,1,1,0,1,1,1,0)$, $(1,1,1,1,0,1,1,0)$, $(1,1,1,1,1,1,1,0)$

Transitions for parameter set $(\mathbf{ECM}, \mathbf{DNAdam}) = (1, 1)$

$(0,0,0,0,0,0,1,1)$, $(0,0,0,0,1,0,1,1)$, $(0,0,0,1,0,0,1,1)$, $(0,0,0,1,1,0,1,1)$, $(0,0,1,1,0,0,1,1)$, $(0,0,1,1,1,0,1,1)$, $(0,1,0,0,0,0,1,1)$, $(0,1,0,0,1,0,1,1)$, $(0,1,0,1,0,0,1,1)$, $(0,1,0,1,1,0,1,1)$, $(0,1,1,1,0,0,1,1)$, $(0,1,1,1,1,0,1,1)$, $(1,0,0,0,0,0,1,1)$, $(1,0,0,0,0,1,1,1)$, $(1,0,0,0,1,0,1,1)$, $(1,0,0,0,1,1,1,1)$, $(1,0,0,1,0,1,1,1)$, $(1,0,0,1,1,1,1,1)$, $(1,0,1,0,0,0,1,1)$, $(1,0,1,0,0,1,1,1)$, $(1,0,1,0,1,1,1,1)$, $(1,0,1,1,0,1,1,1)$, $(1,0,1,1,1,1,1,1)$, $(1,1,0,0,0,0,1,1)$, $(1,1,0,0,1,0,1,1)$, $(1,1,0,0,1,1,1,1)$, $(1,1,0,1,0,1,1,1)$, $(1,1,0,1,1,1,1,1)$, $(1,1,1,0,0,0,1,1)$, $(1,1,1,0,0,1,1,1)$, $(1,1,1,0,1,1,1,1)$, $(1,1,1,1,0,1,1,1)$, $(1,1,1,1,1,1,1,1)$

Resulting networks

Network graph for parameter set $(\mathbf{ECM}, \mathbf{DNAdam}) = (0, 0)$

full backward model:

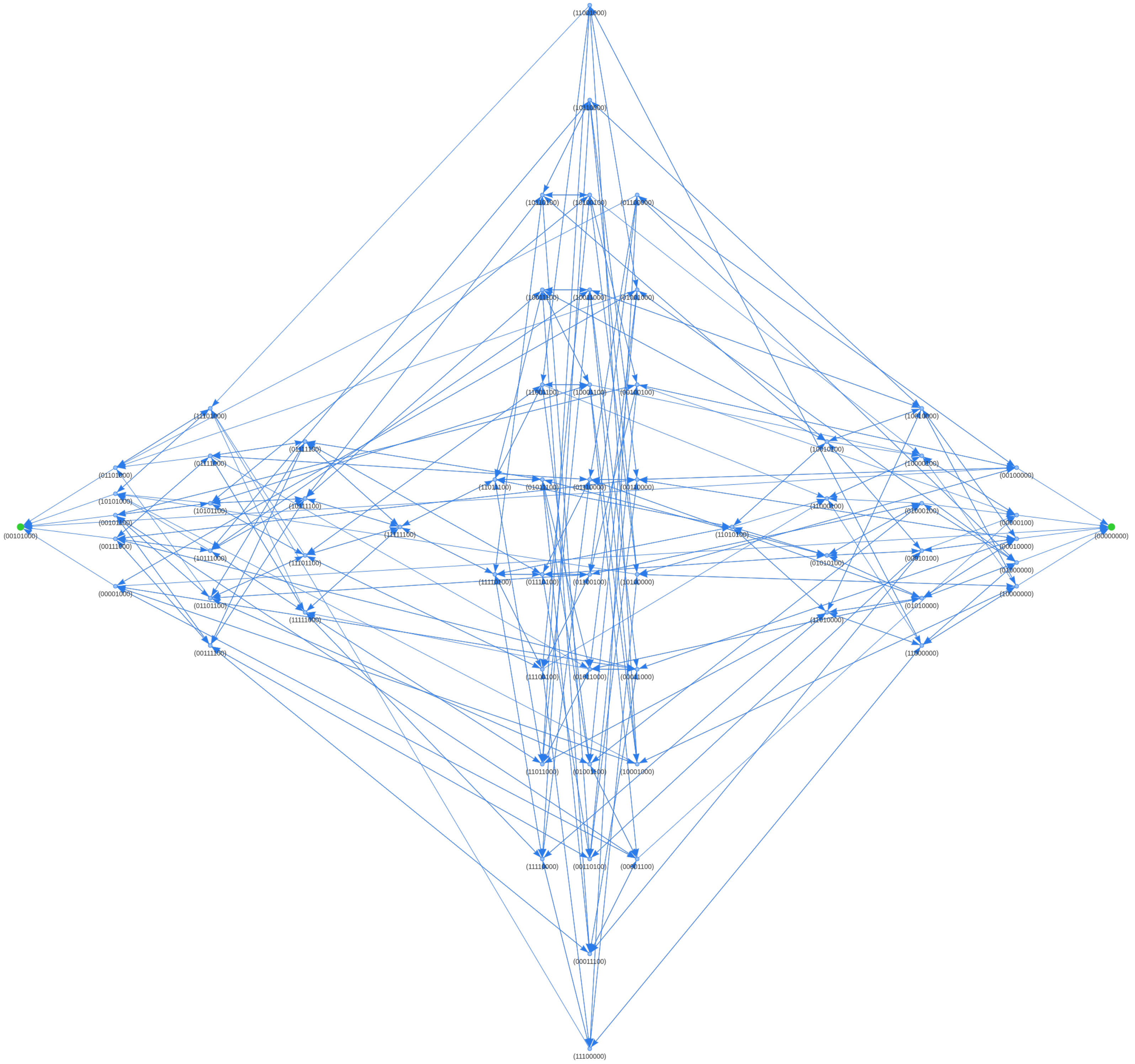

transitions to remove from backward model in red:

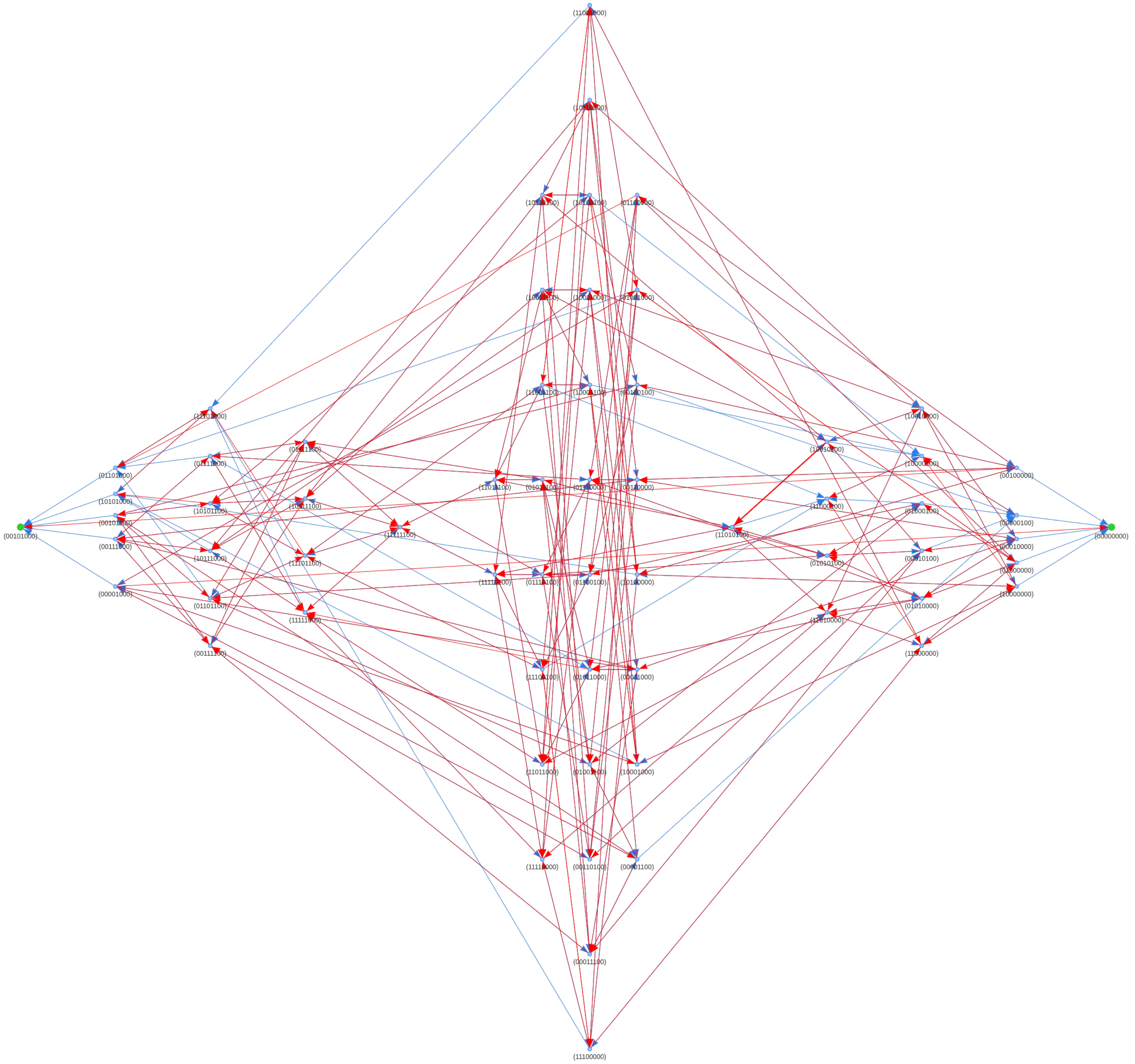

resulting network for the paper model:

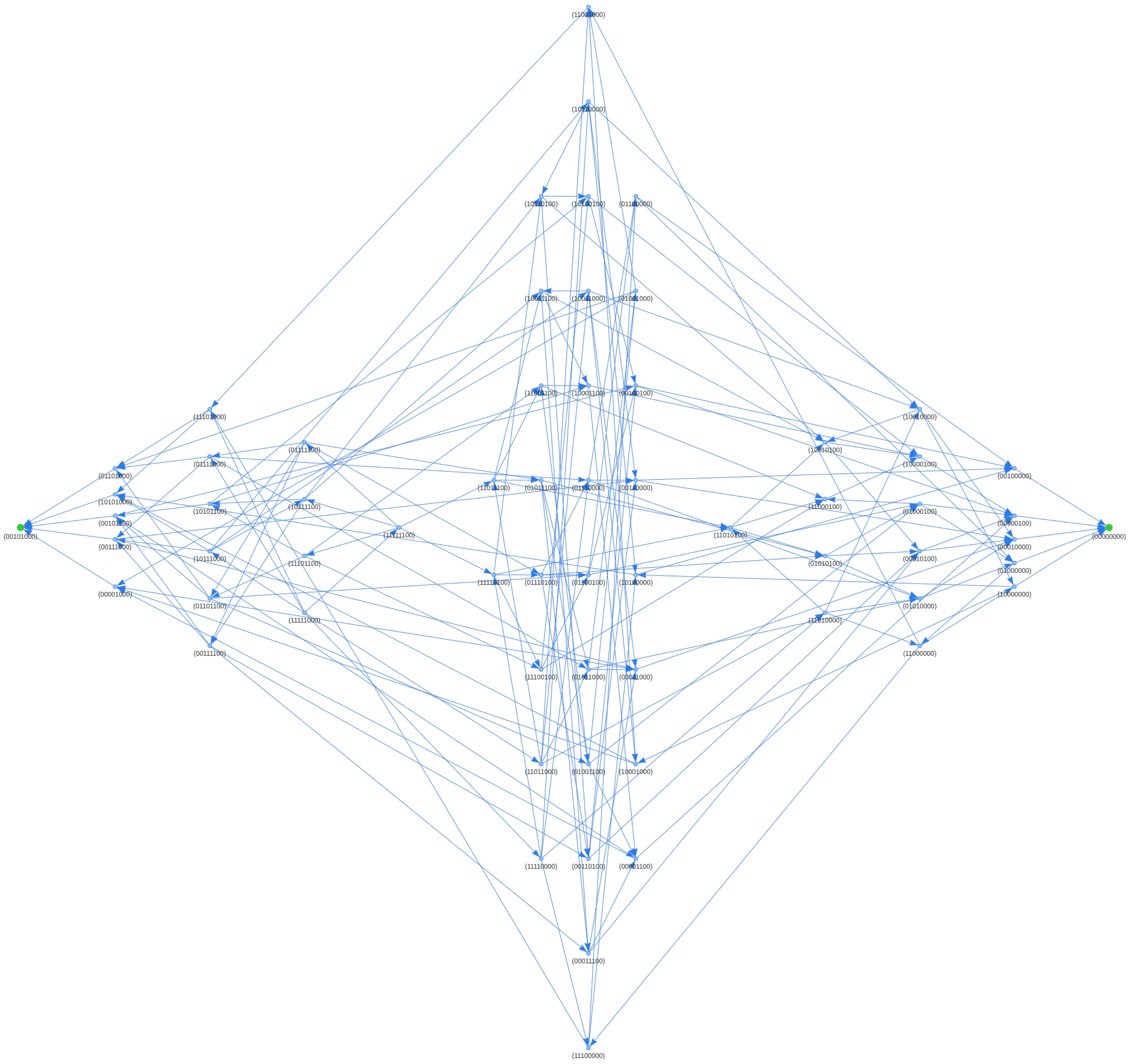

Network graph for parameter set $(\mathbf{ECM}, \mathbf{DNAdam}) = (0, 1)$

full backward model:

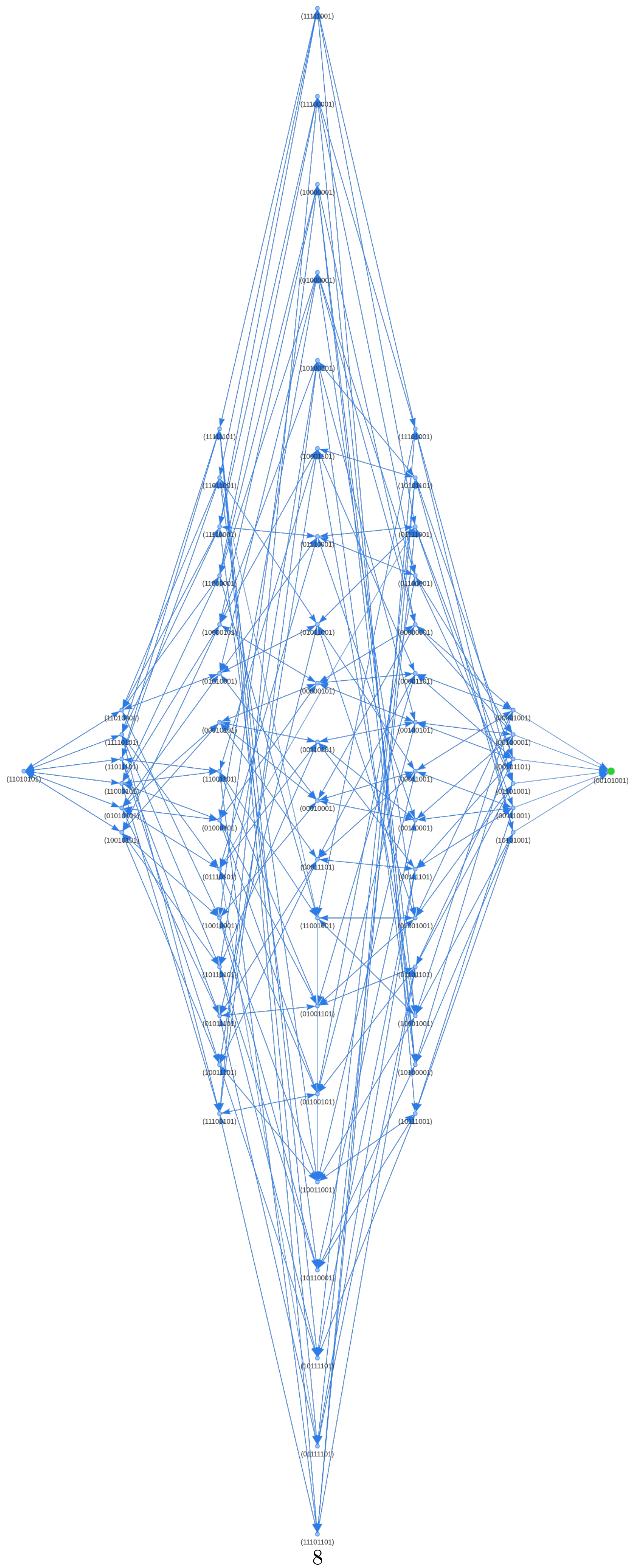

transitions to remove from backward model in red:

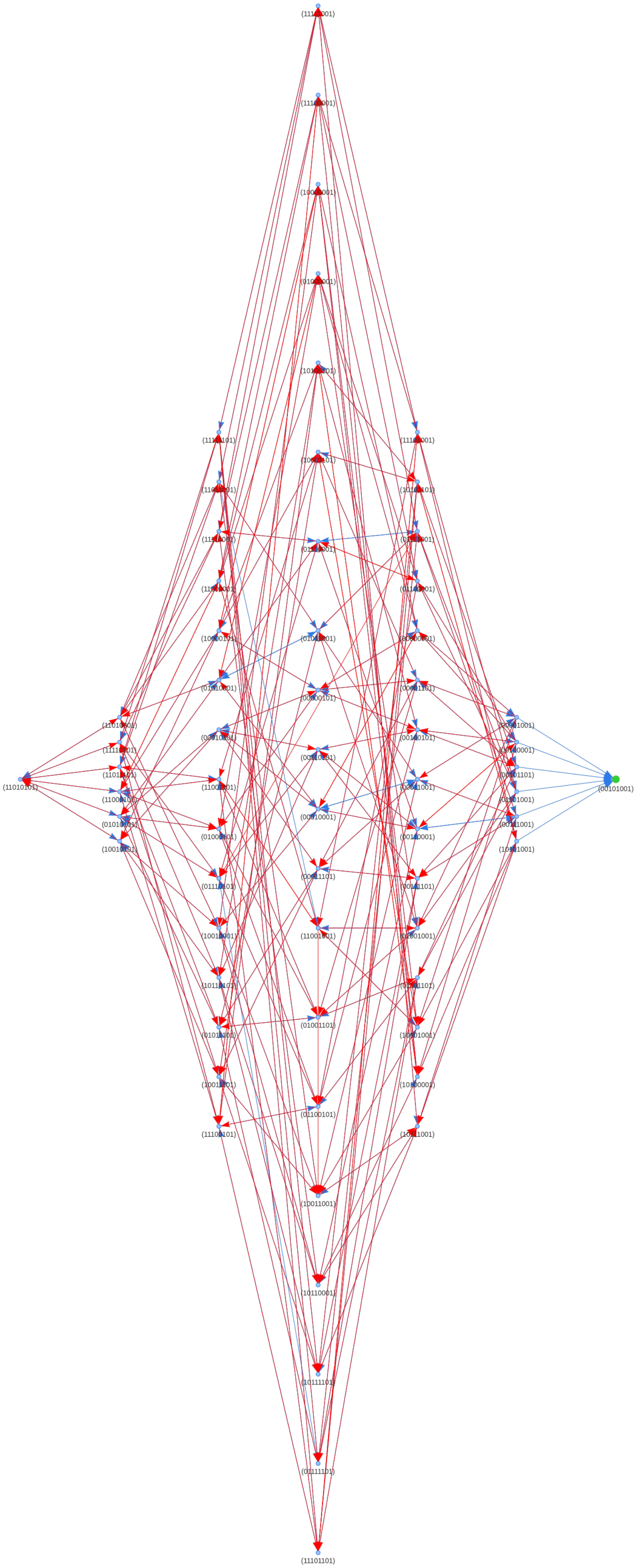

resulting network for the paper model:

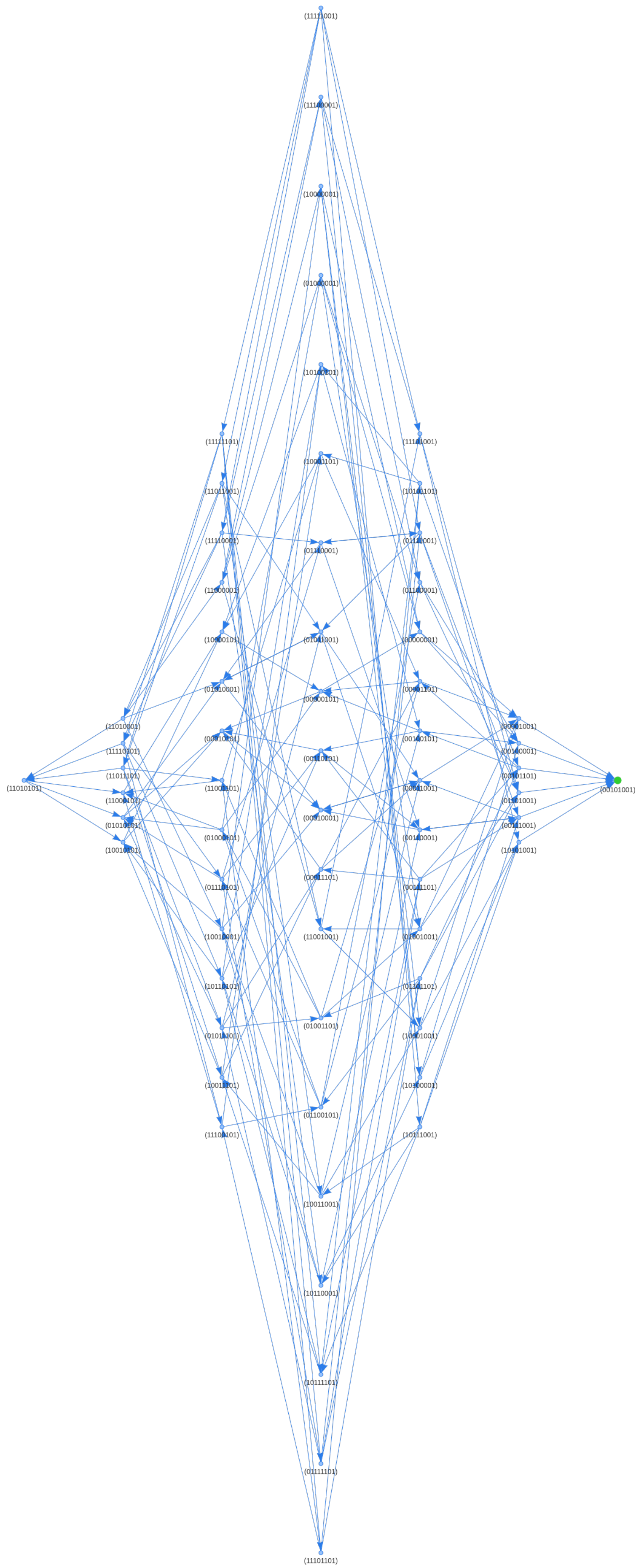

Network graph for parameter set $(\mathbf{ECM}, \mathbf{DNAdam}) = (1, 0)$

full backward model:

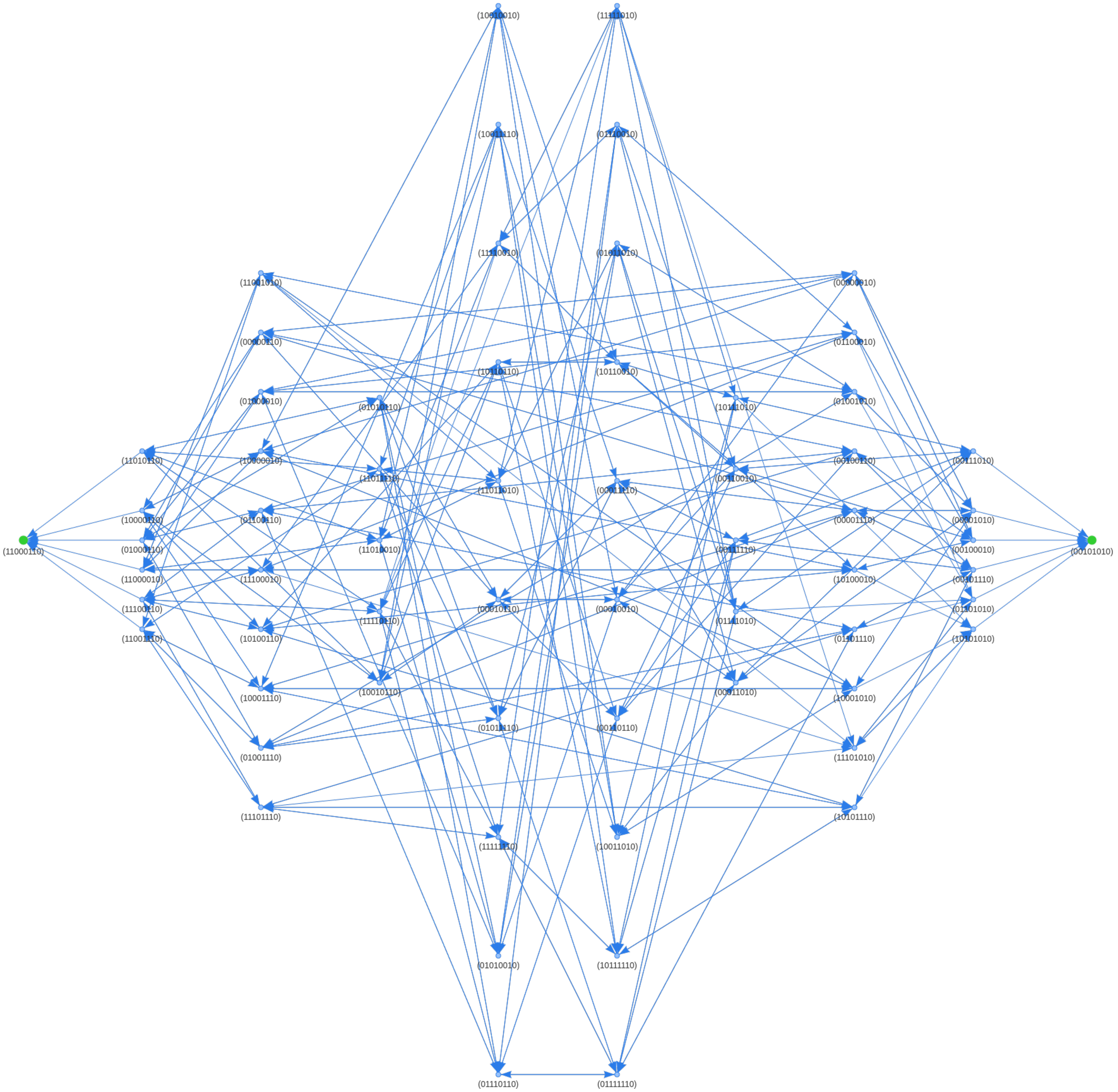

transitions to remove from backward model in red:

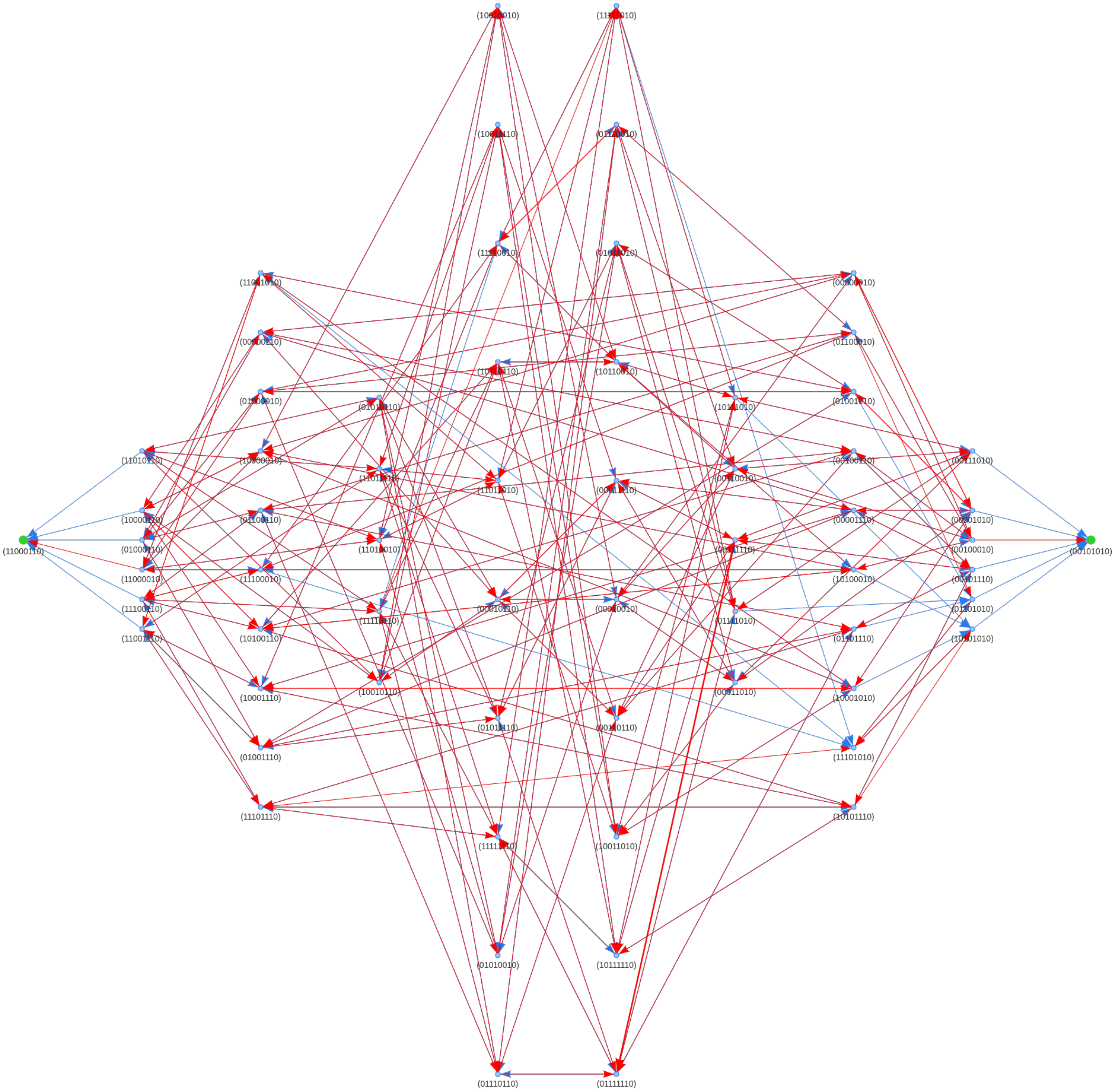

resulting network for the paper model:

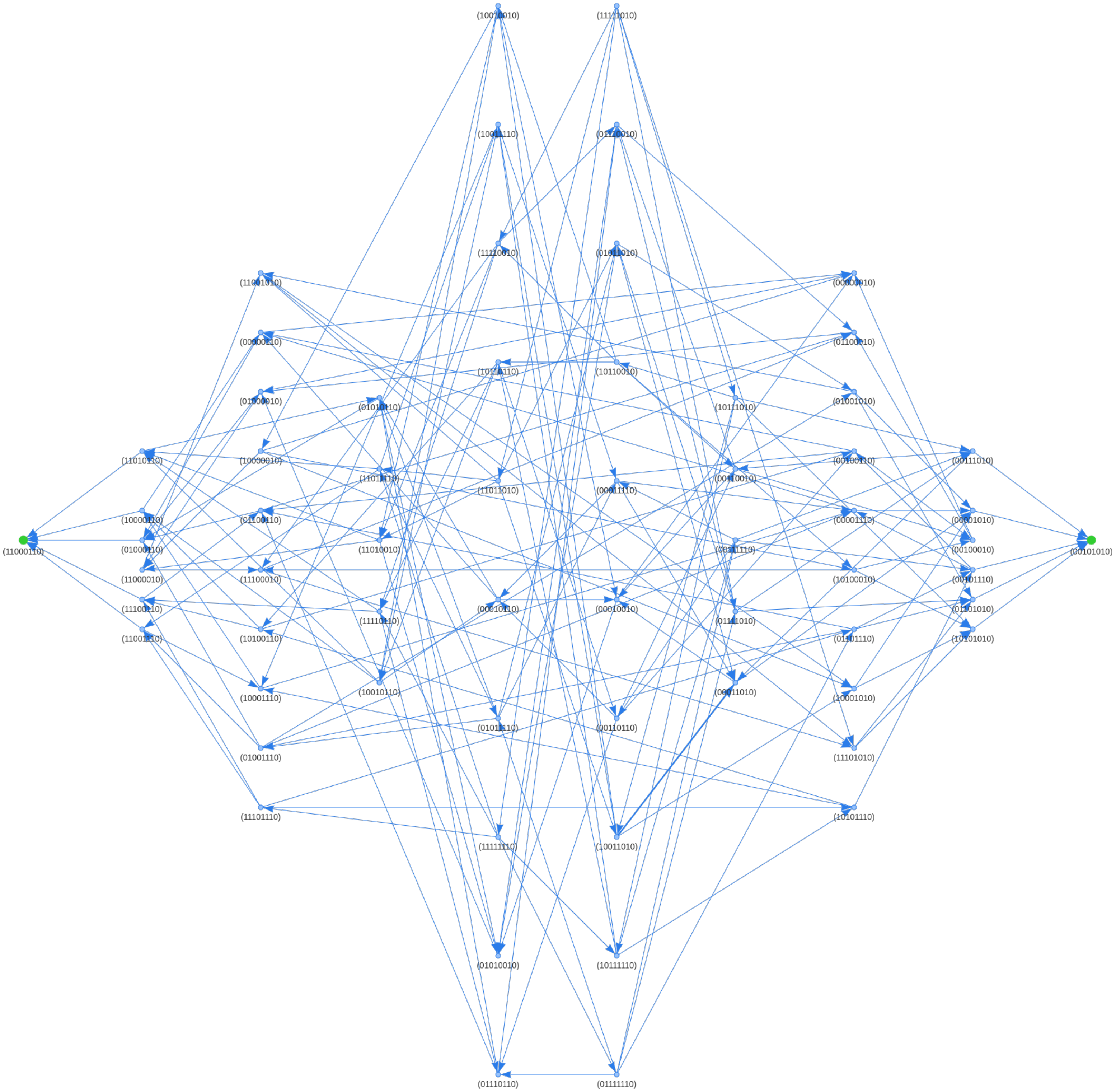

Network graph for parameter set $(\mathbf{ECM}, \mathbf{DNAdam}) = (1, 1)$

full backward model:

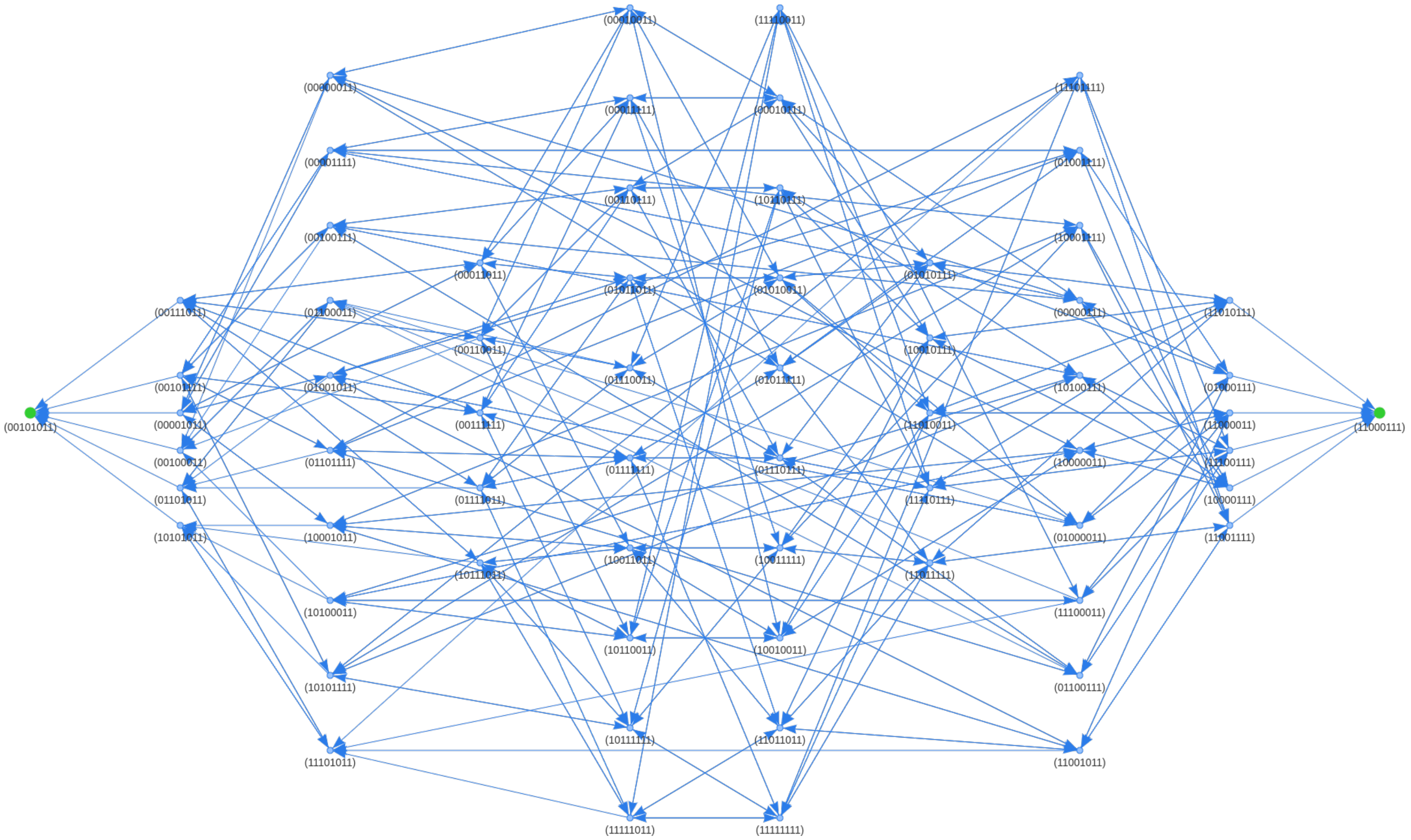

transitions to remove from backward model in red:

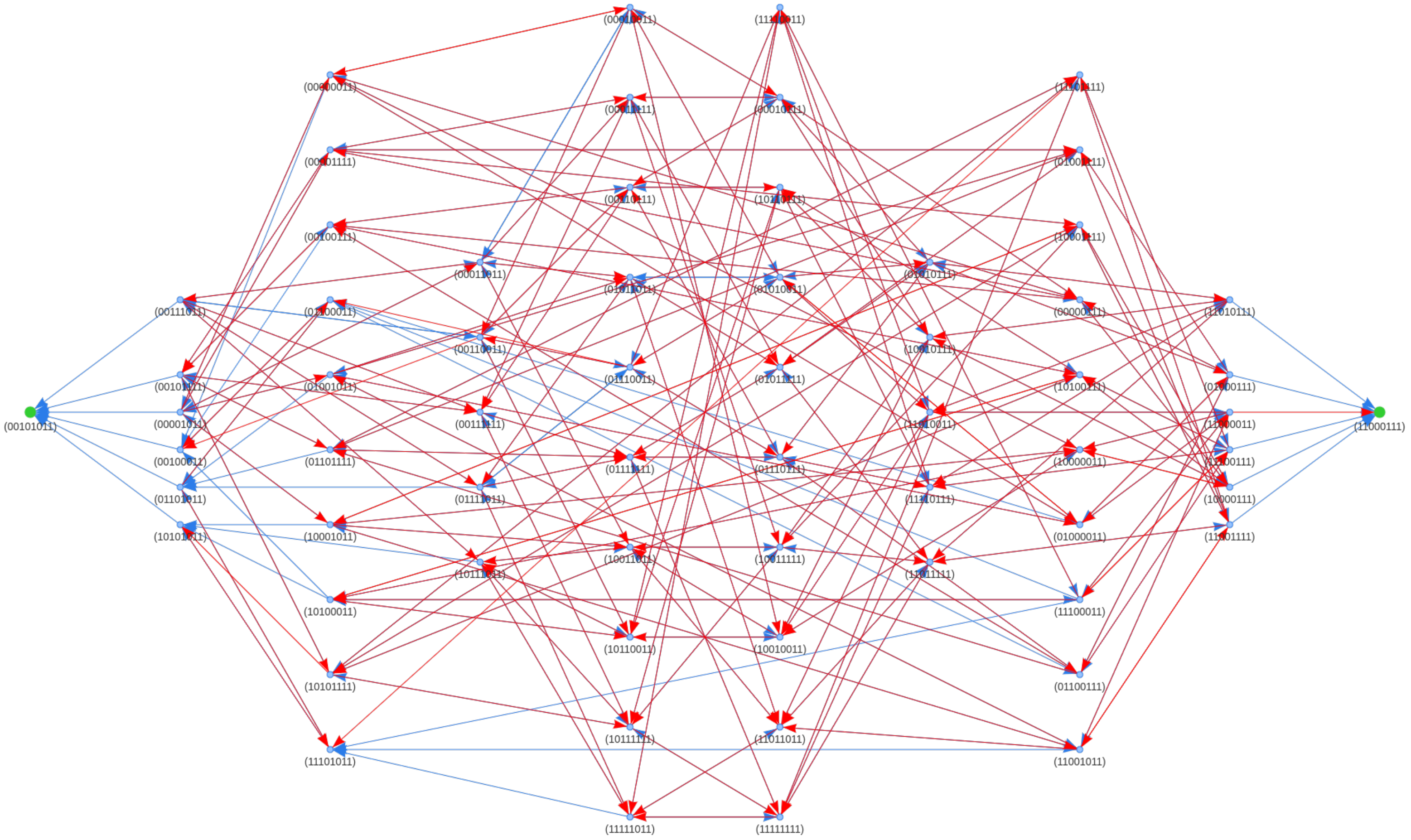

resulting network for the paper model:

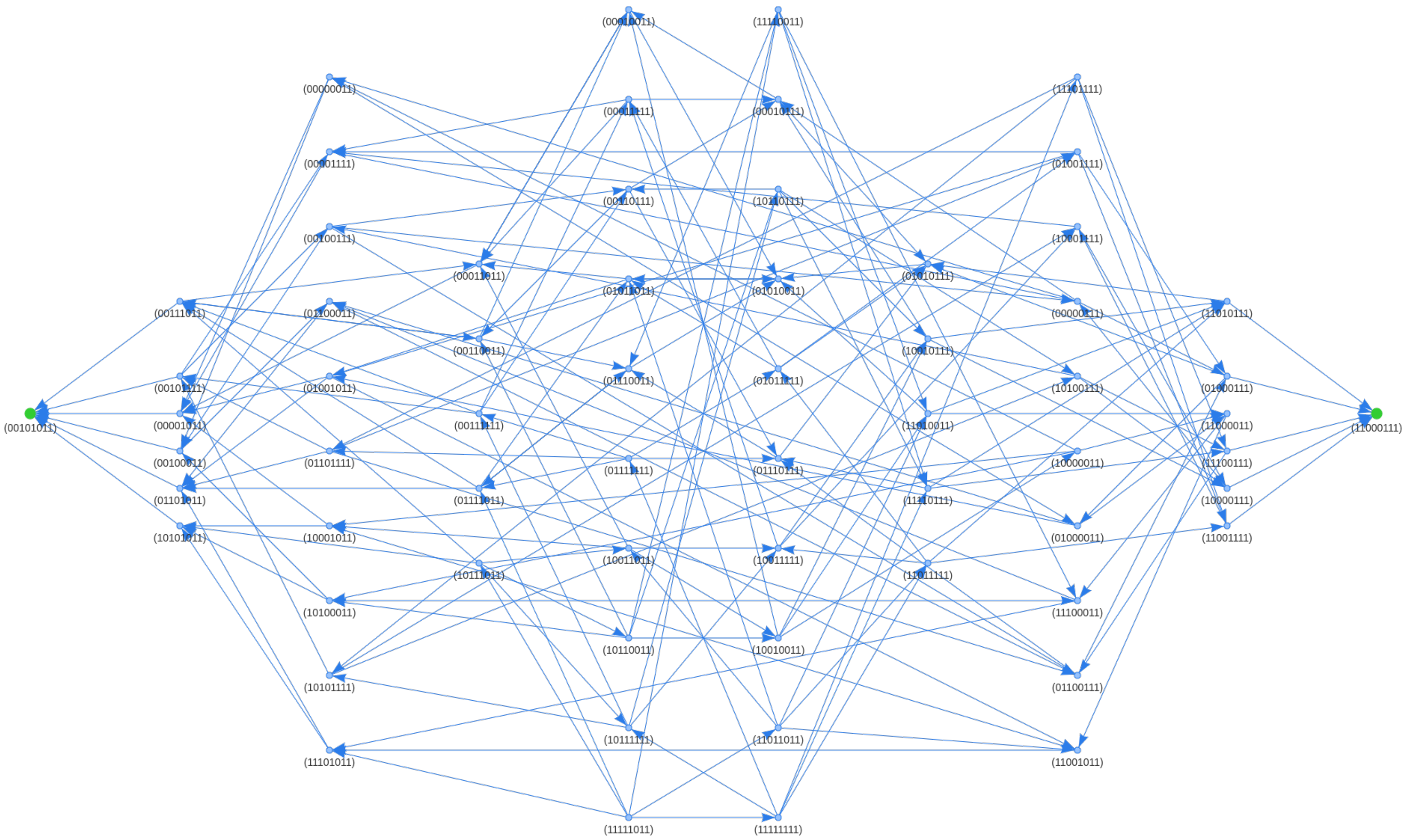

Supplement: S2 File — The states are in the form of lists, as well as the graphic representation of the network. (PDF) [file pcbi.1009035.s002.pdf]
