# Supplementary figures and images for: Unsupervised logic-based mechanism inference for network-driven biological processes
Source: PLoS Comput Biol. 2021 Jun 2;17(6):e1009035. doi: 10.1371/journal.pcbi.1009035 (PMC8202945; doi:10.1371/journal.pcbi.1009035)

# Parameter set (ECM, DNAdam) = (0, 0):

Steady states: (0, 0, 0, 0, 0, 0, 0), (0, 0, 1, 0, 1, 0, 0, 0)

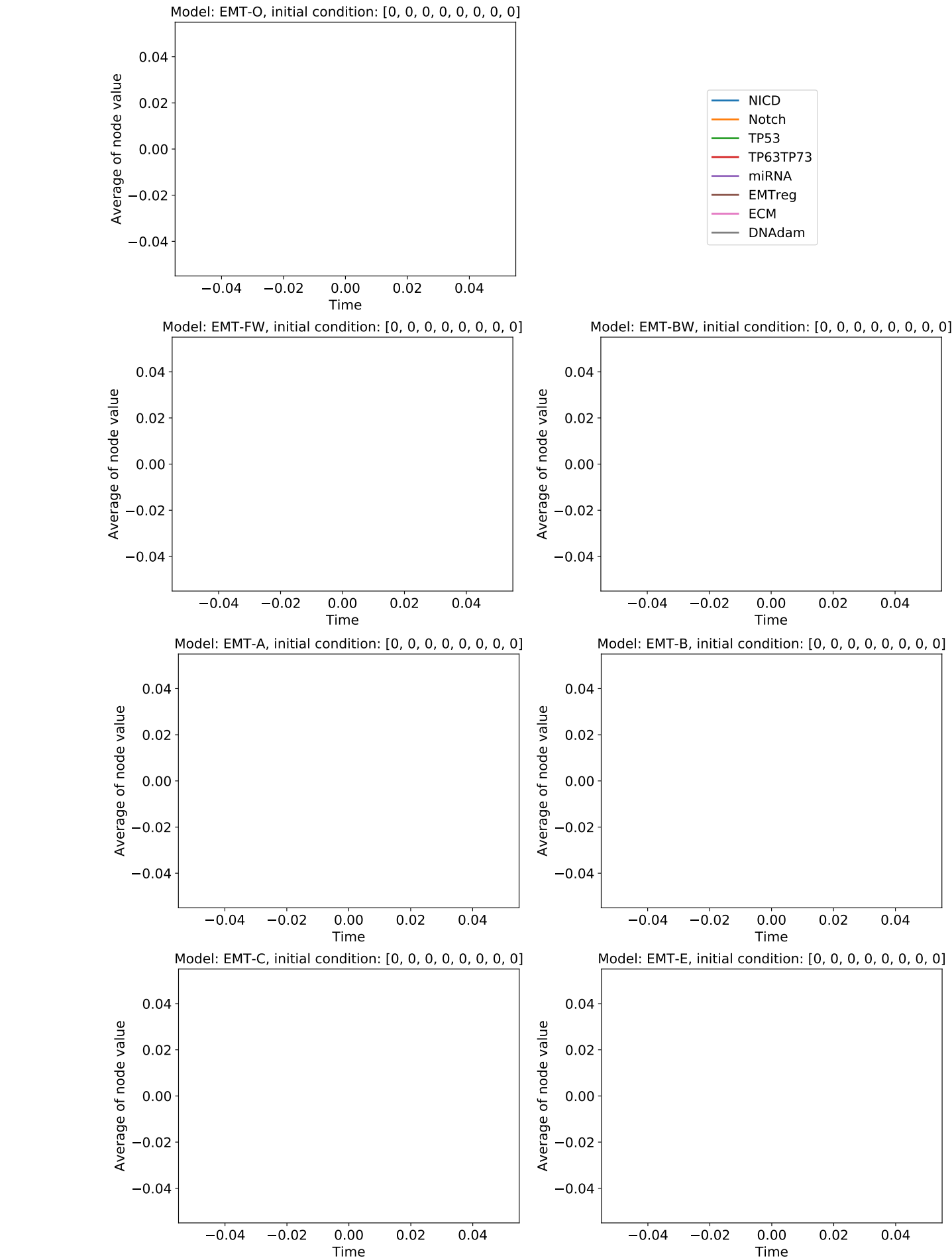

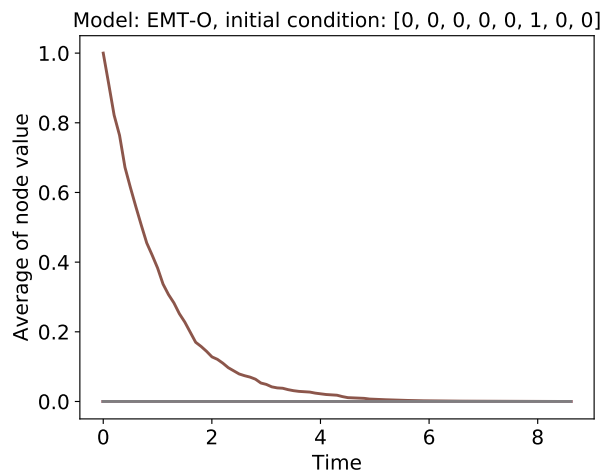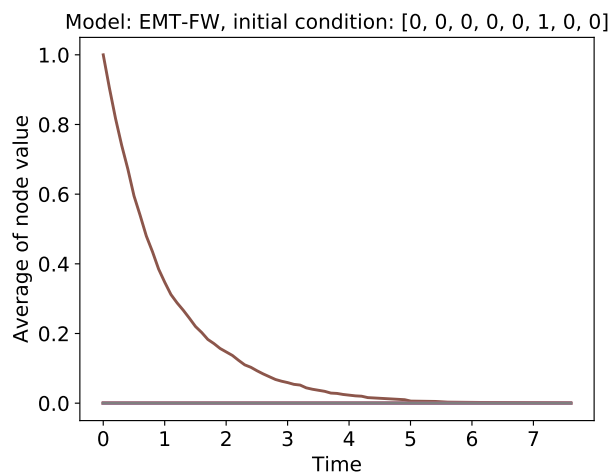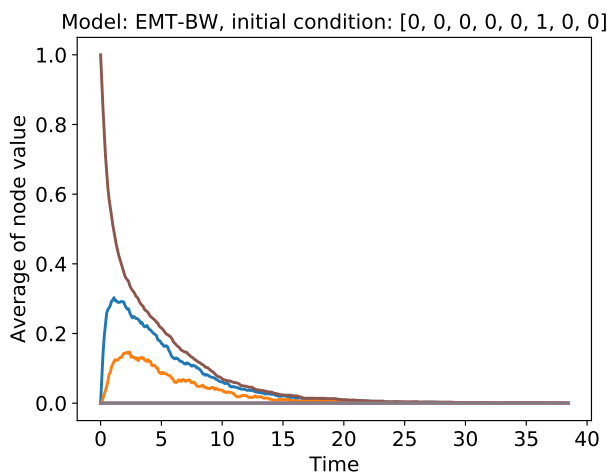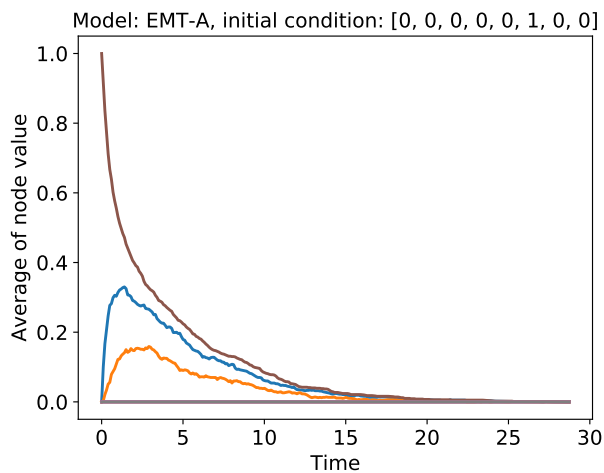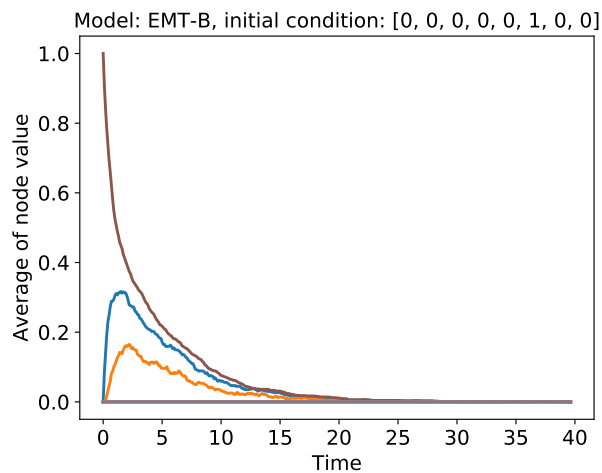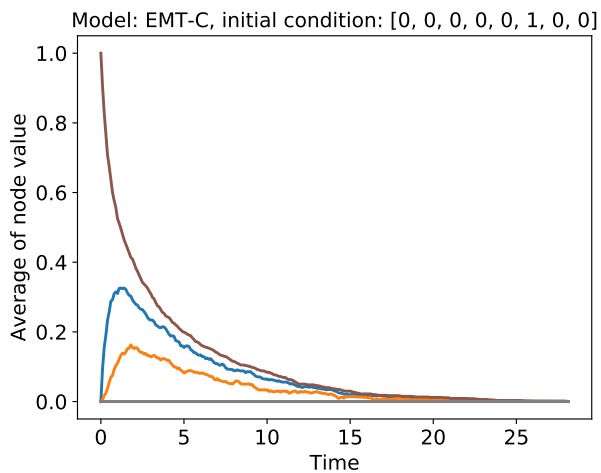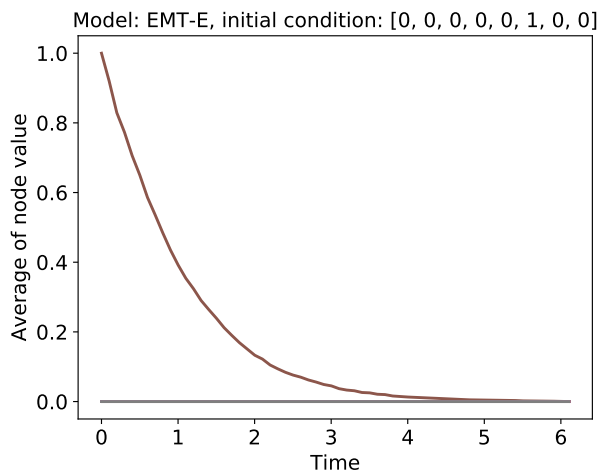

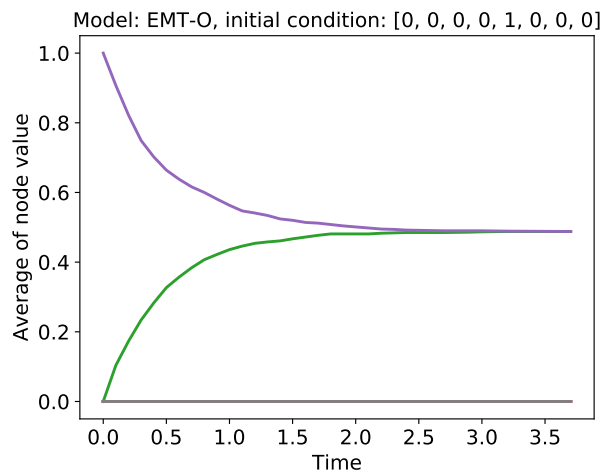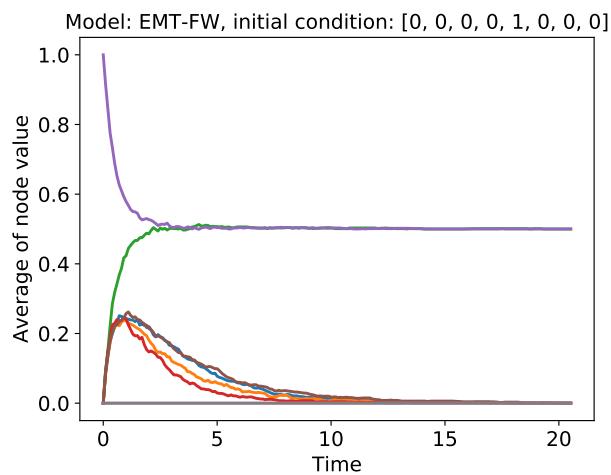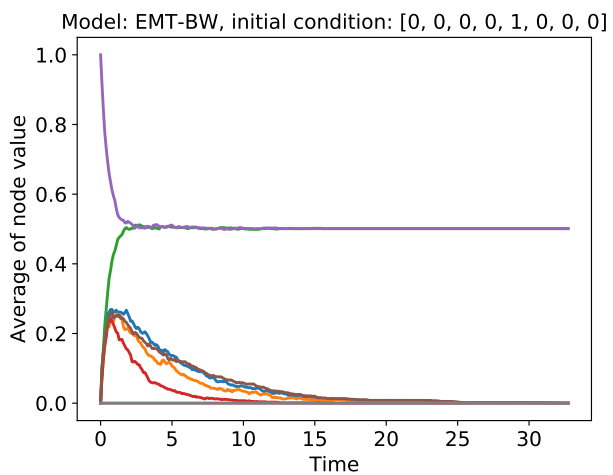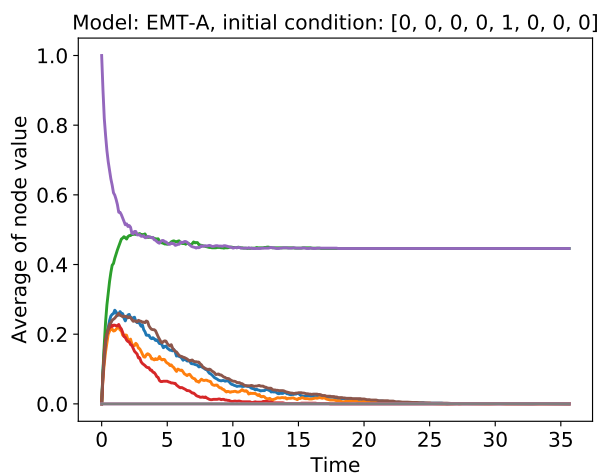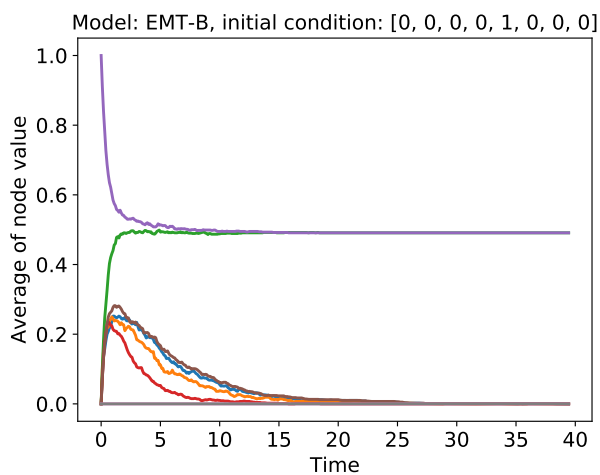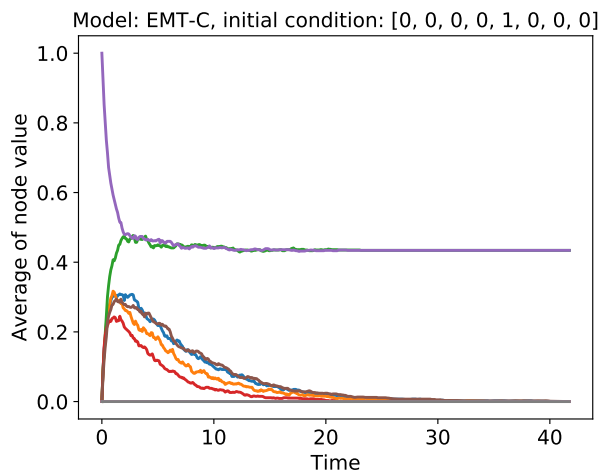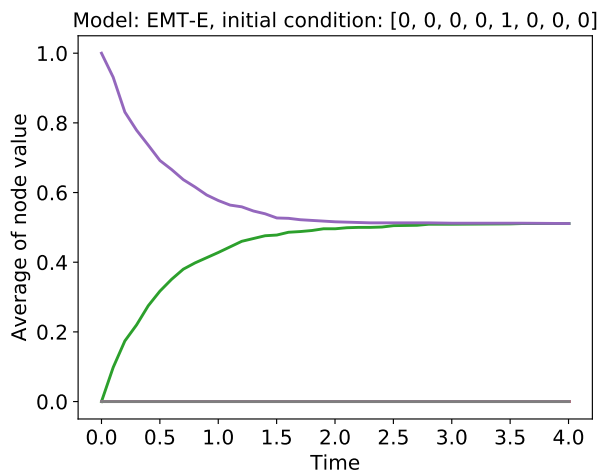

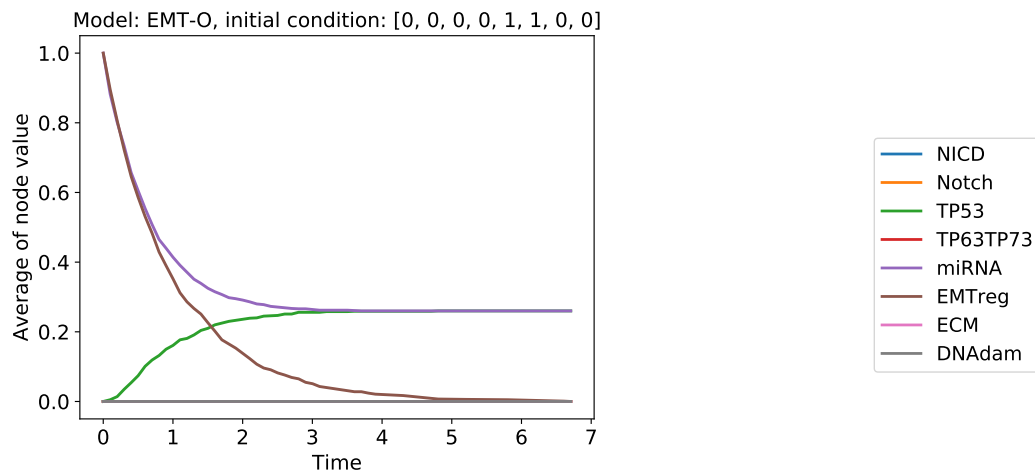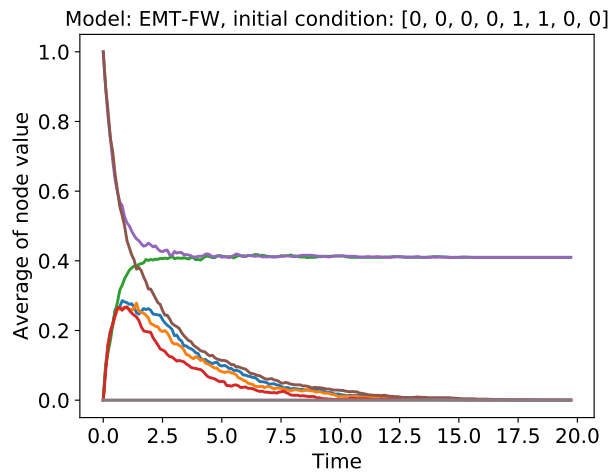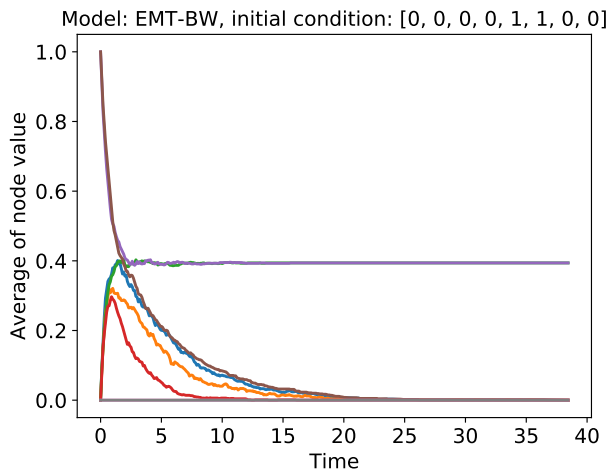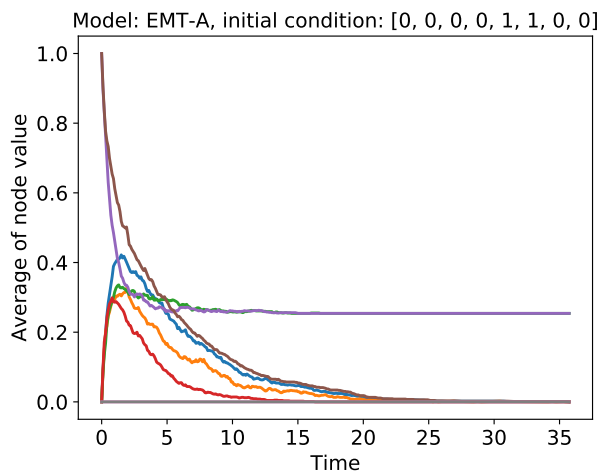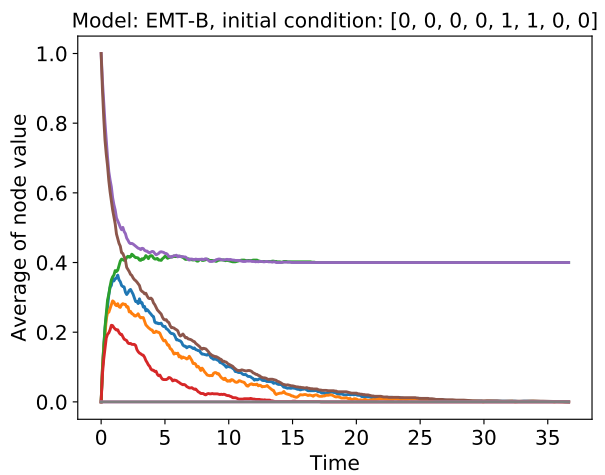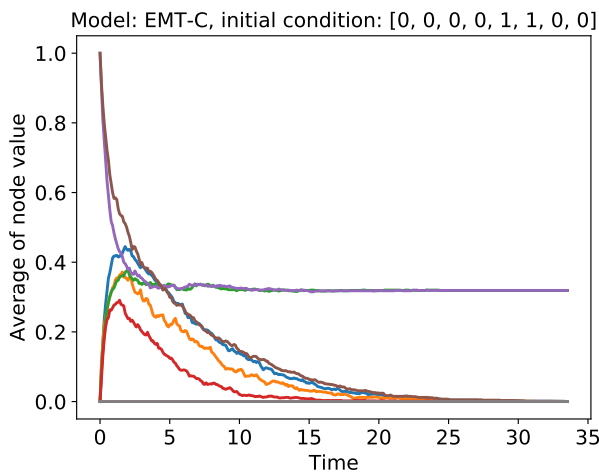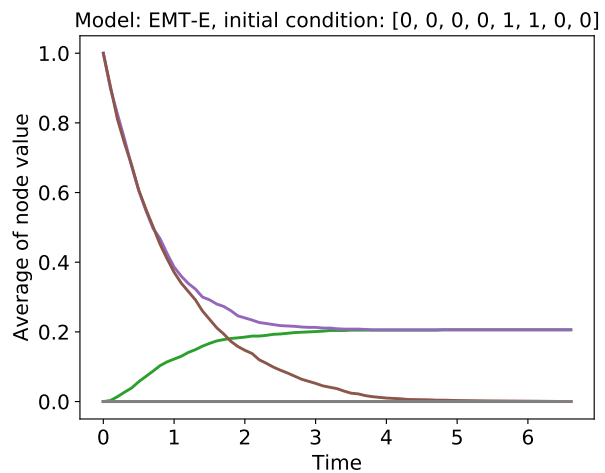

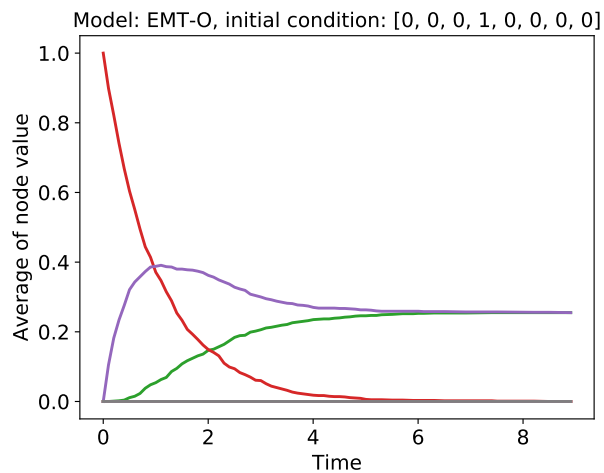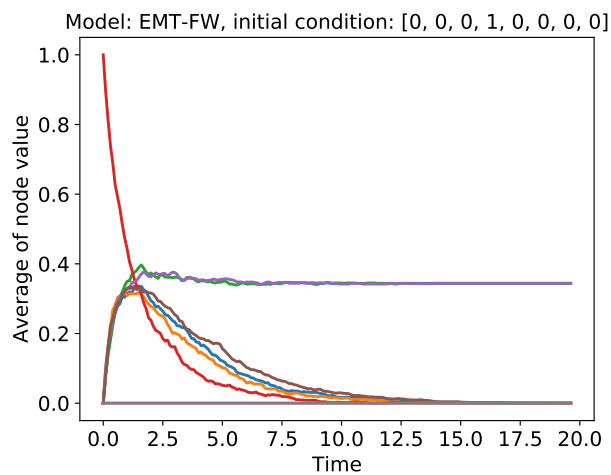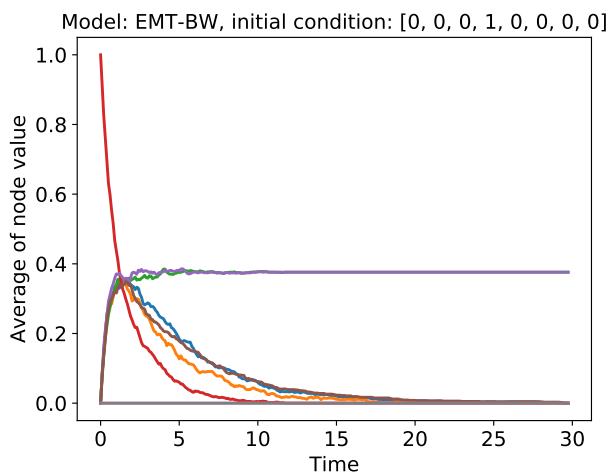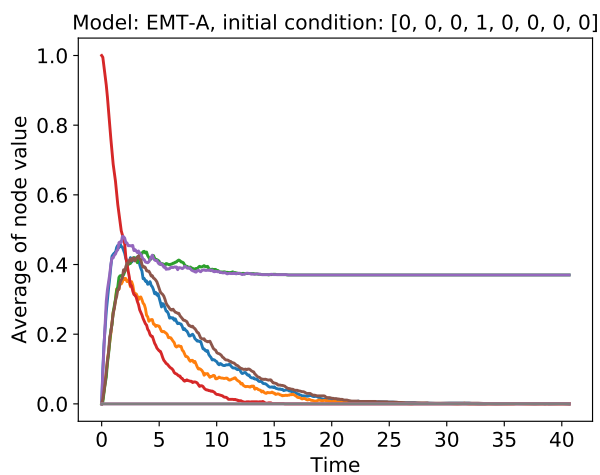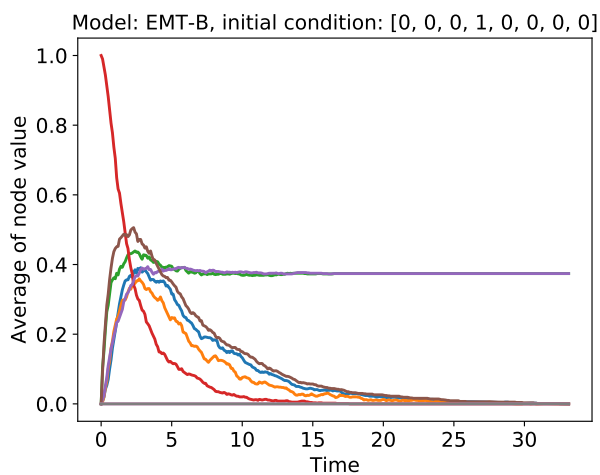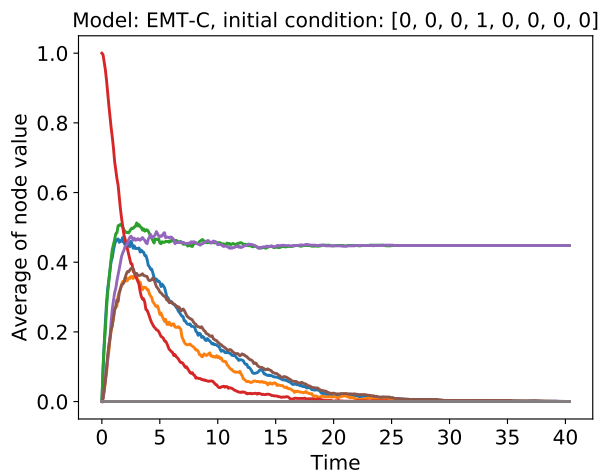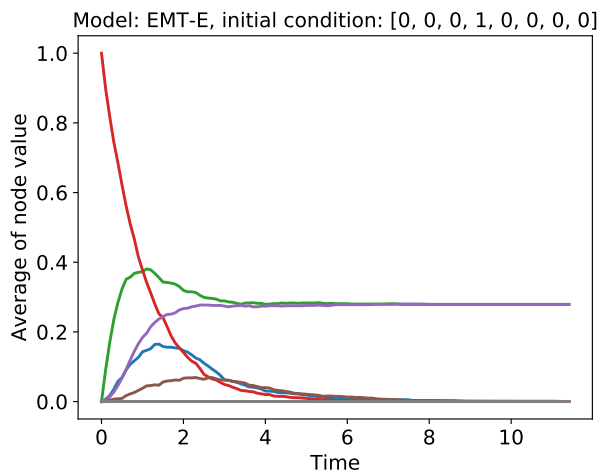

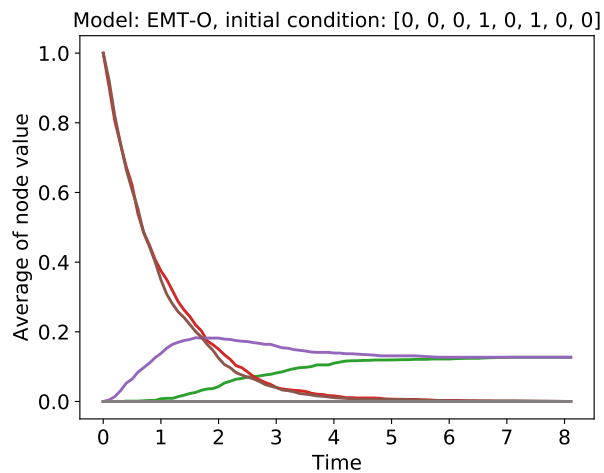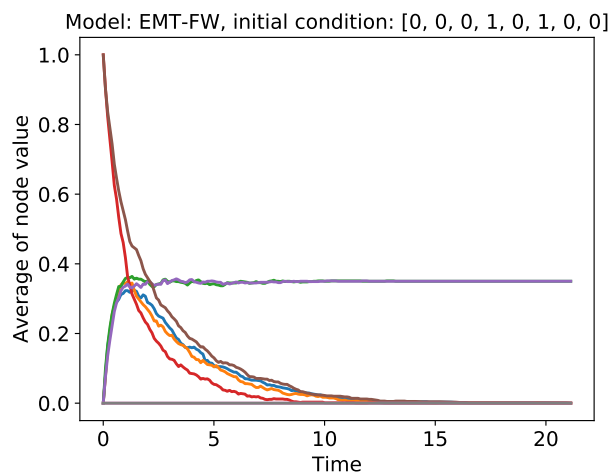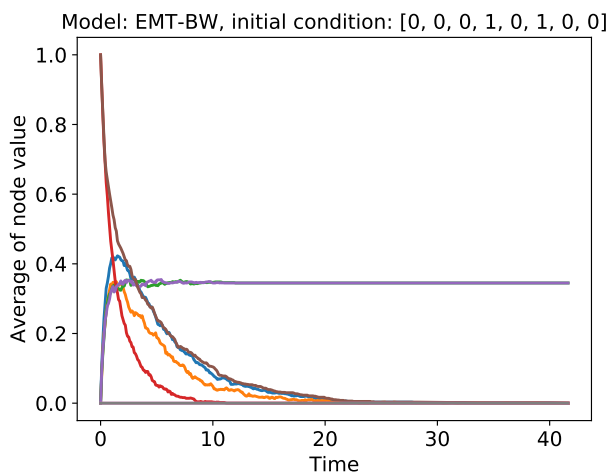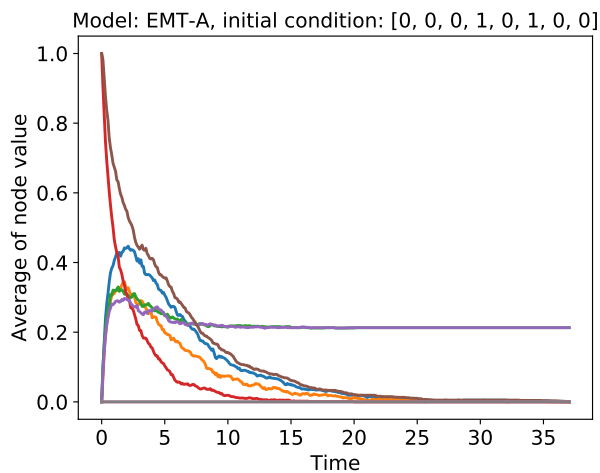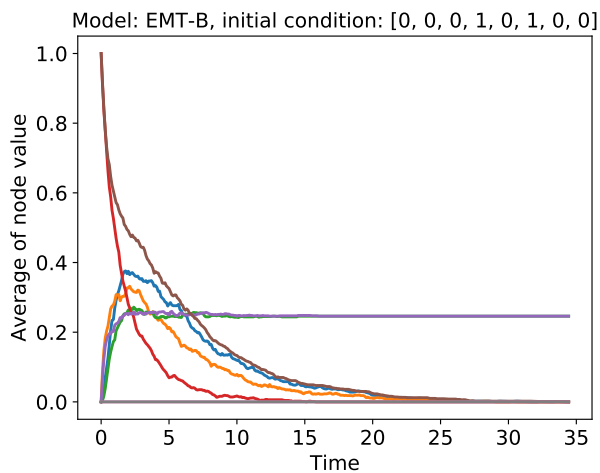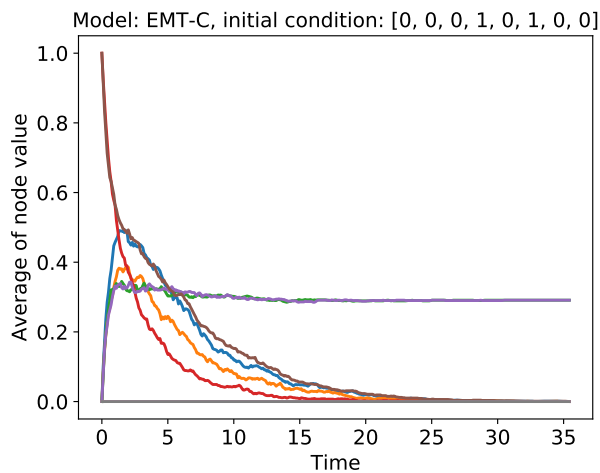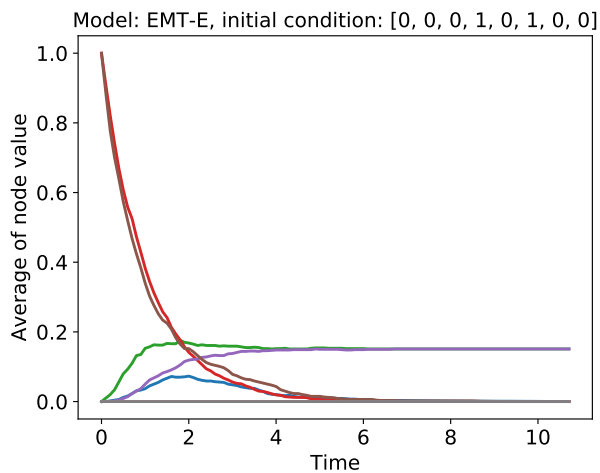

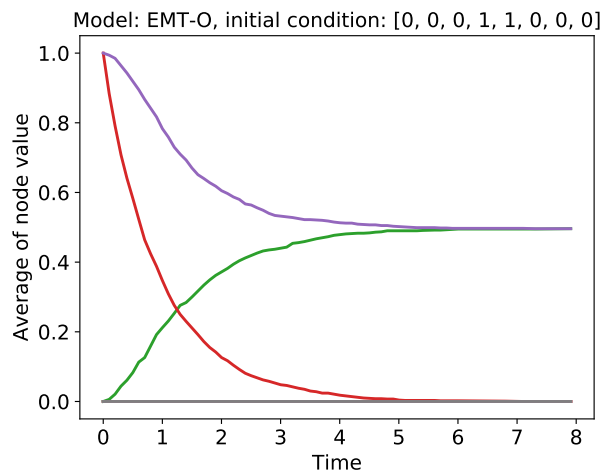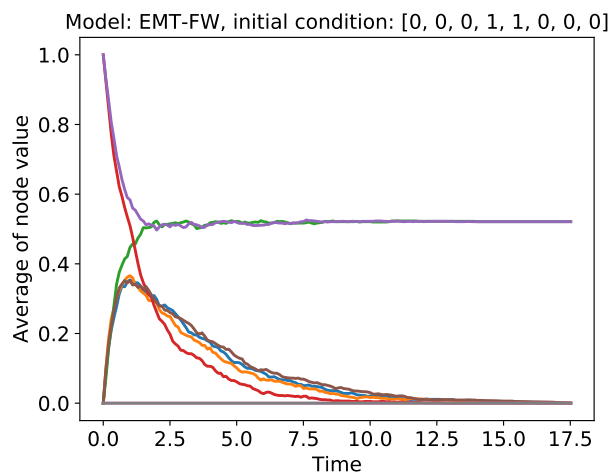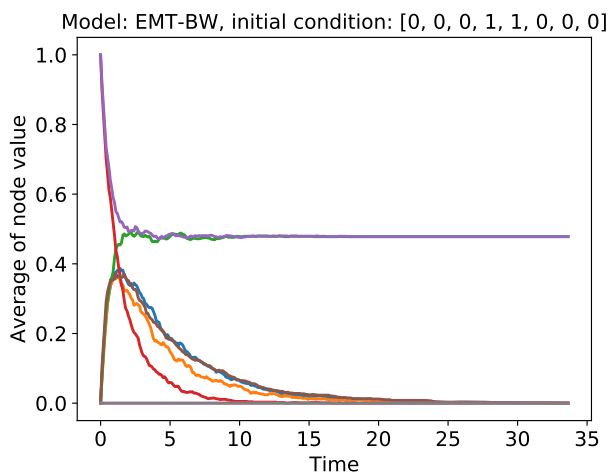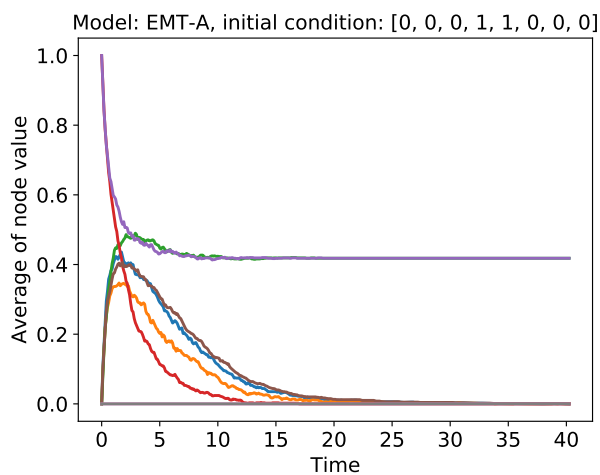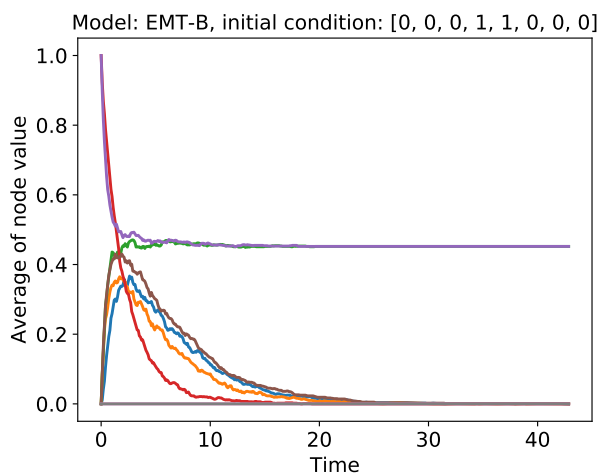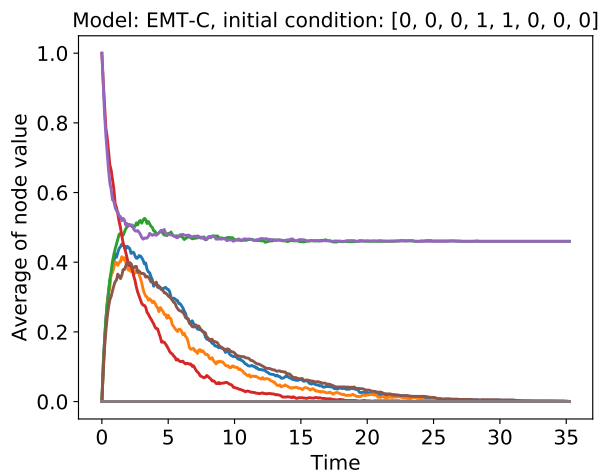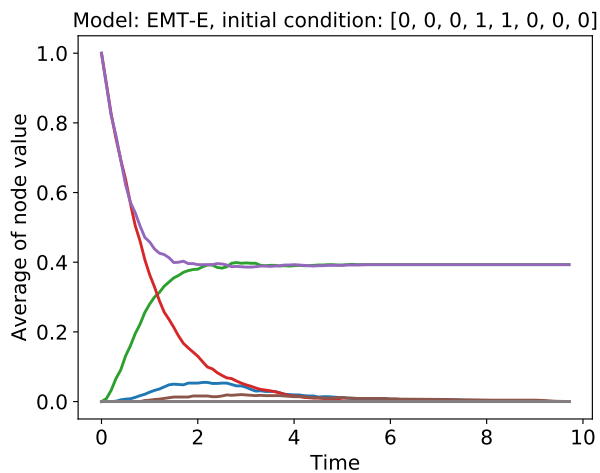

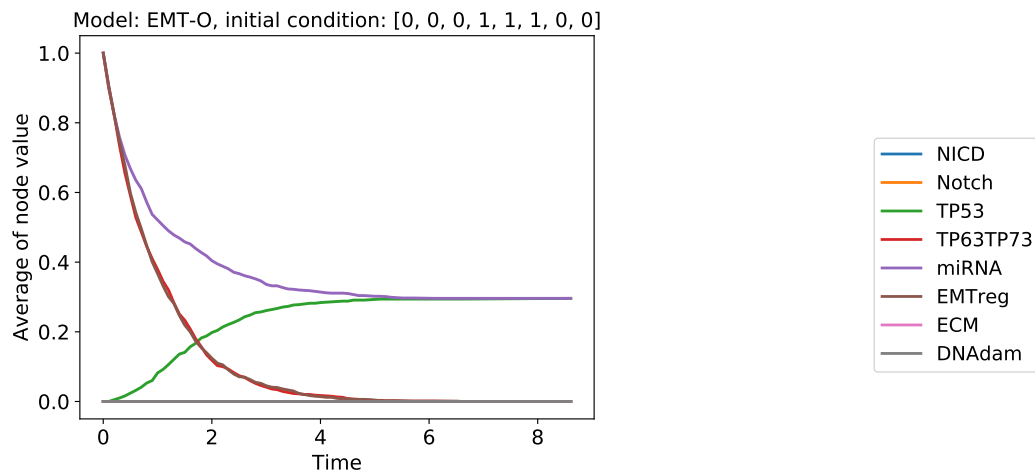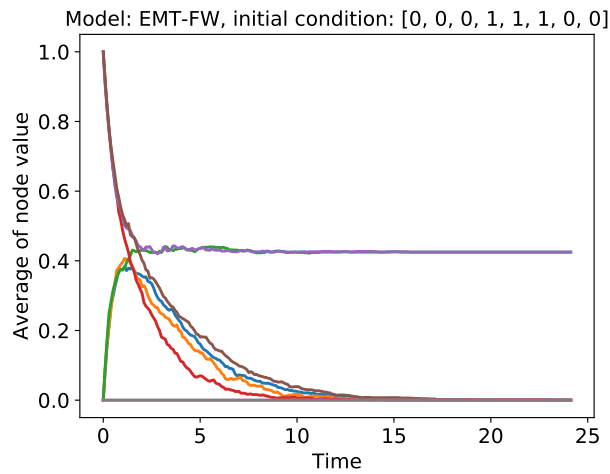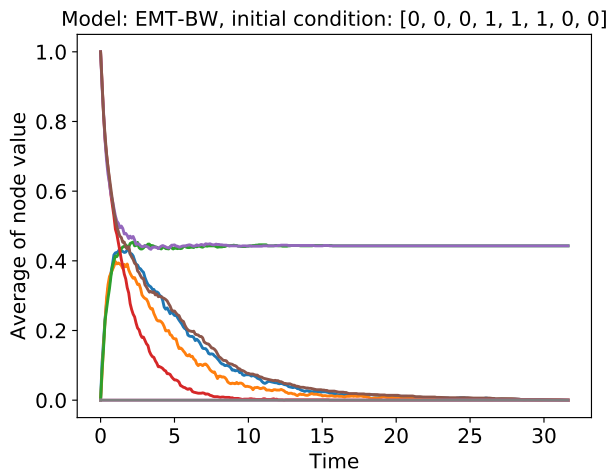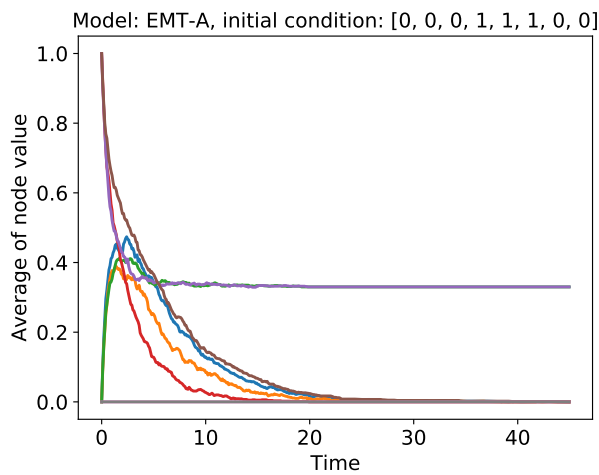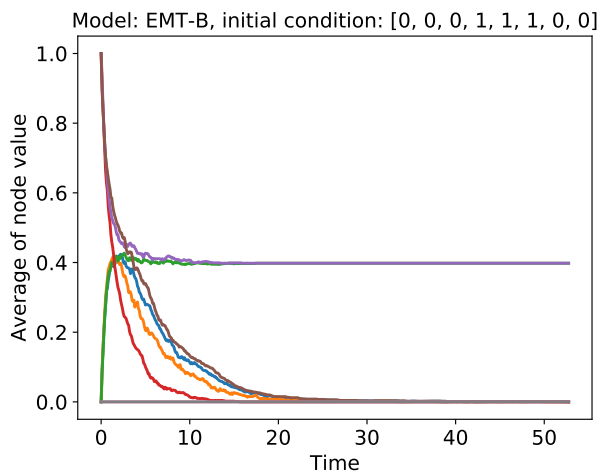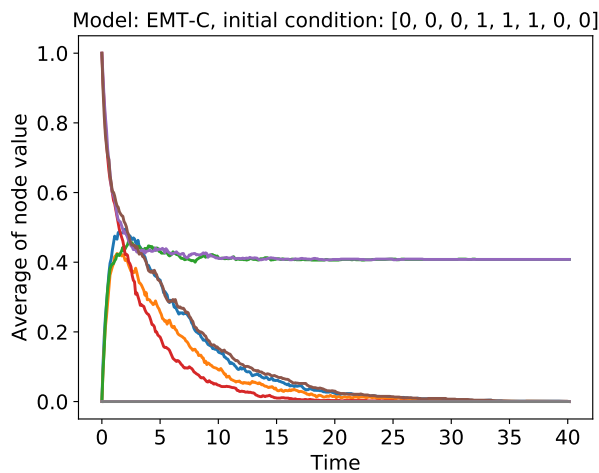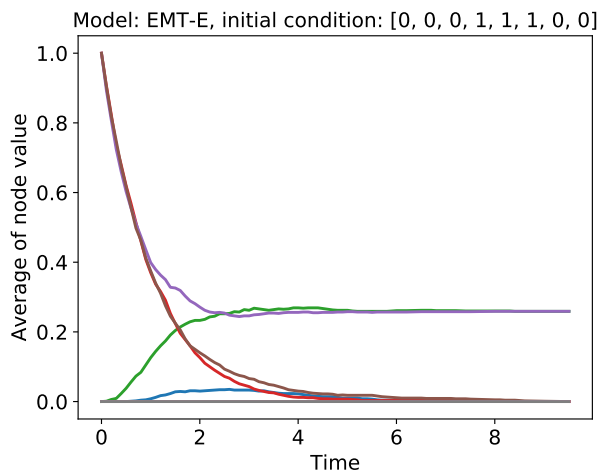

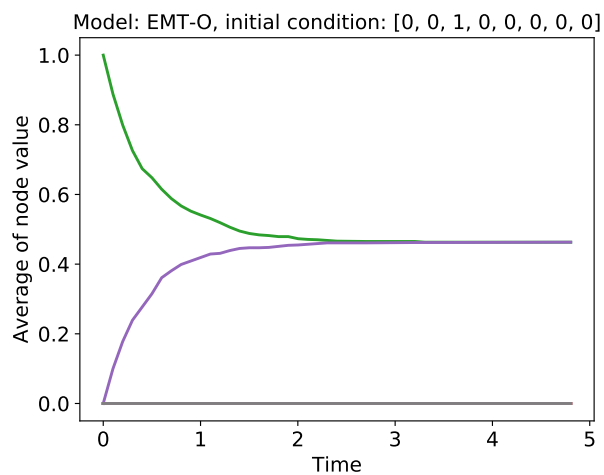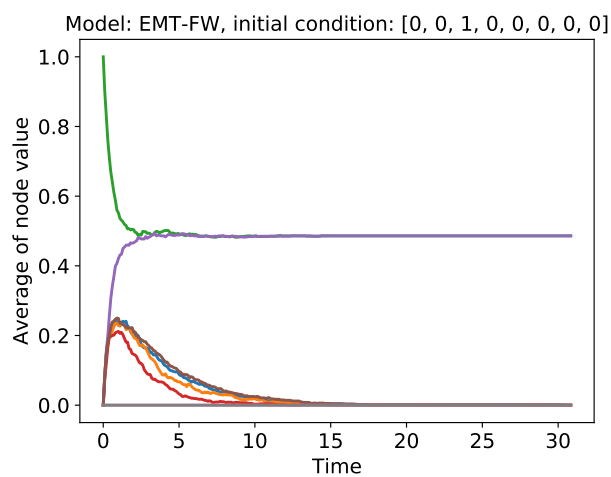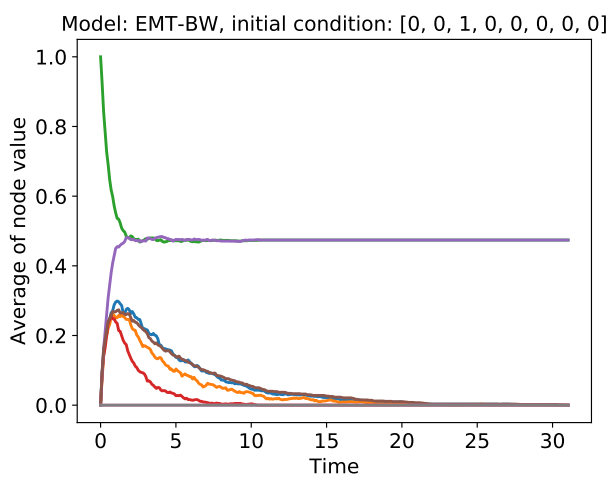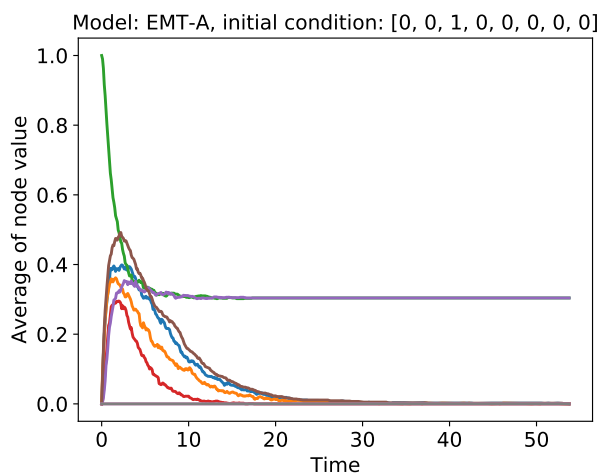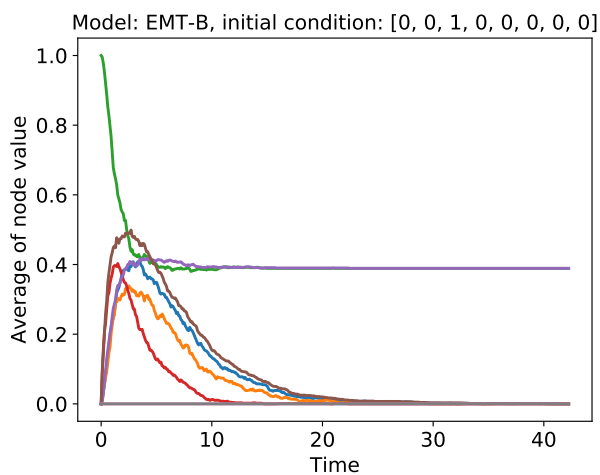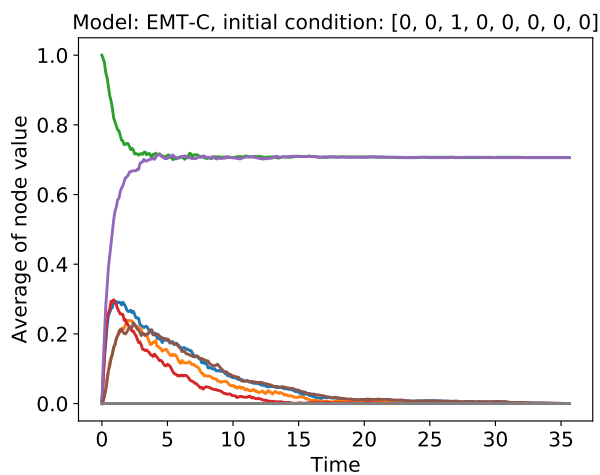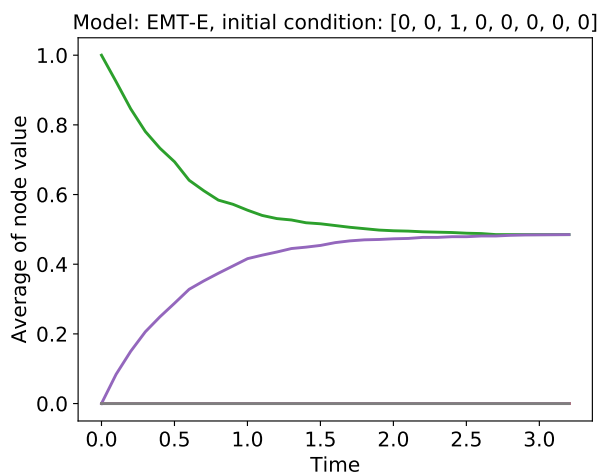

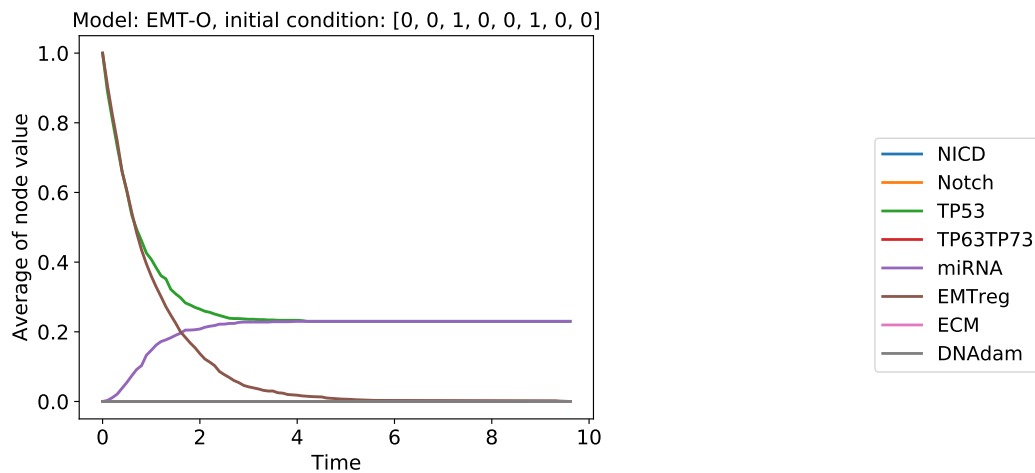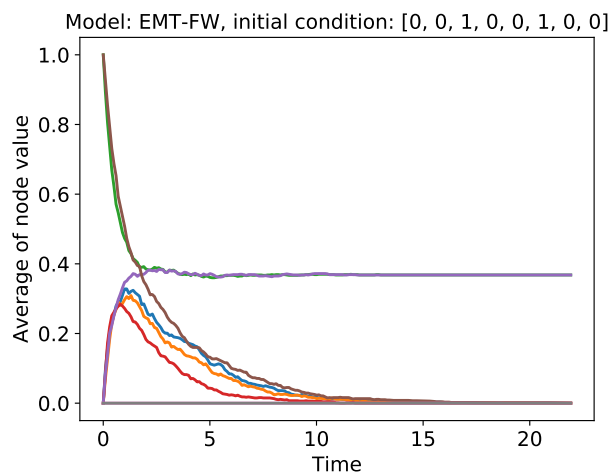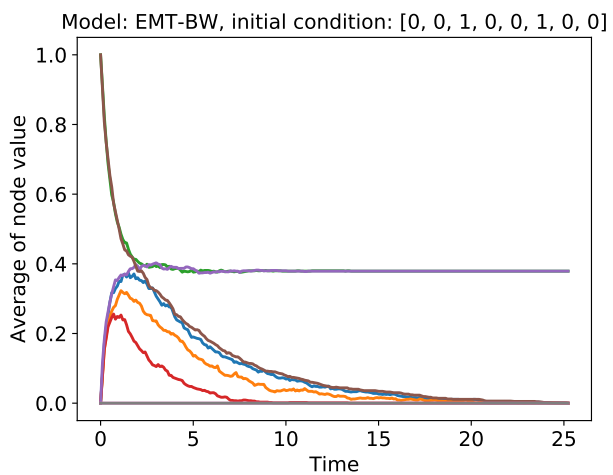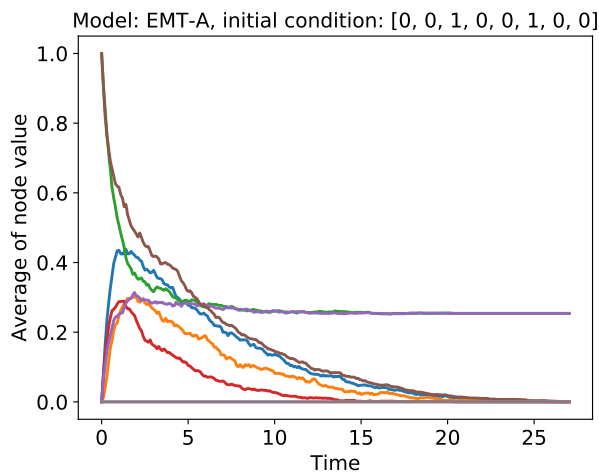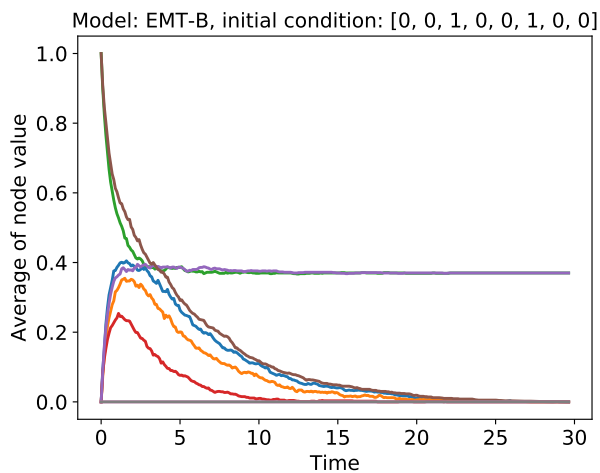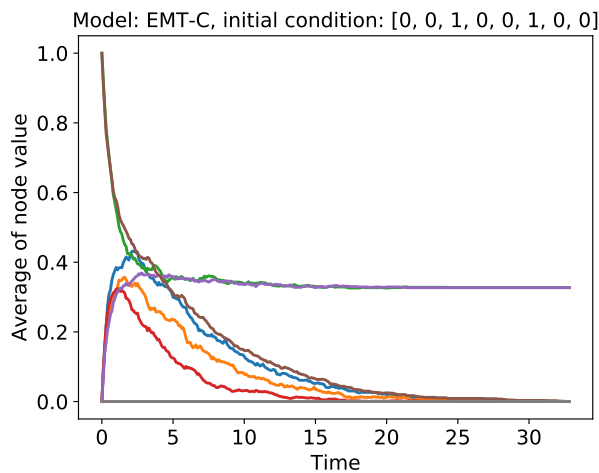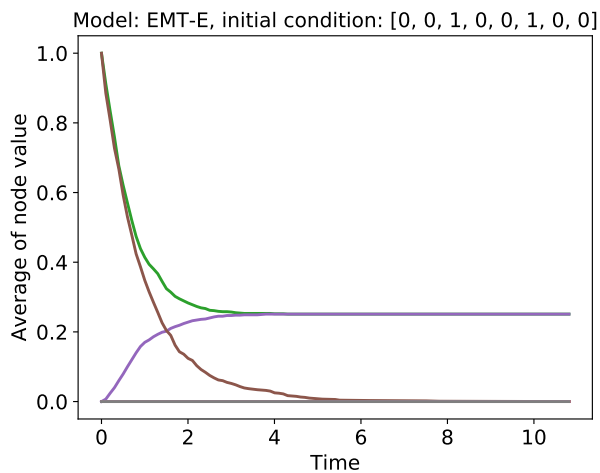

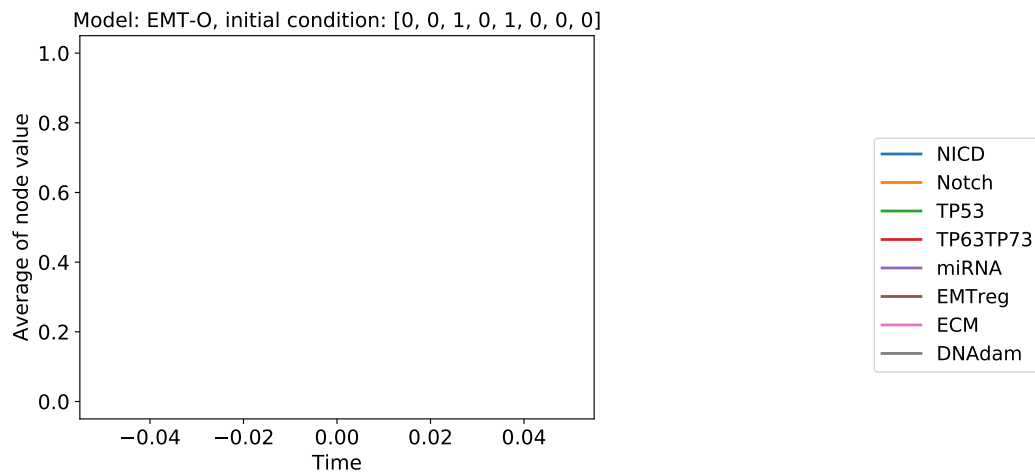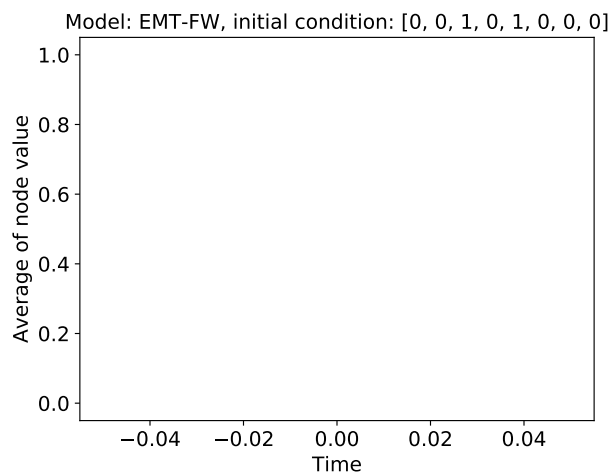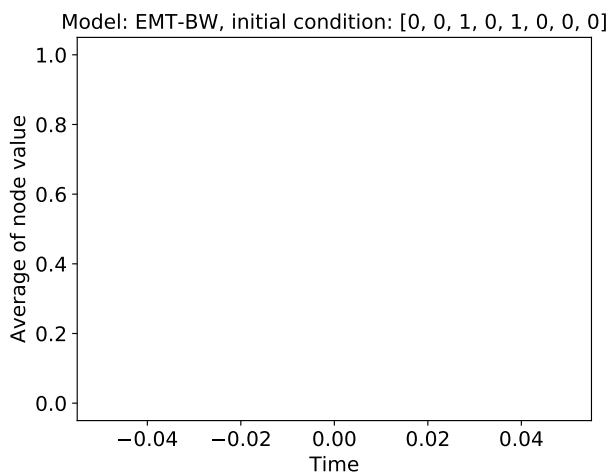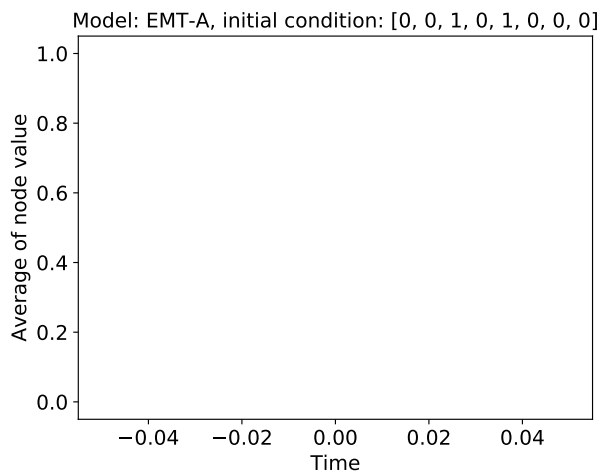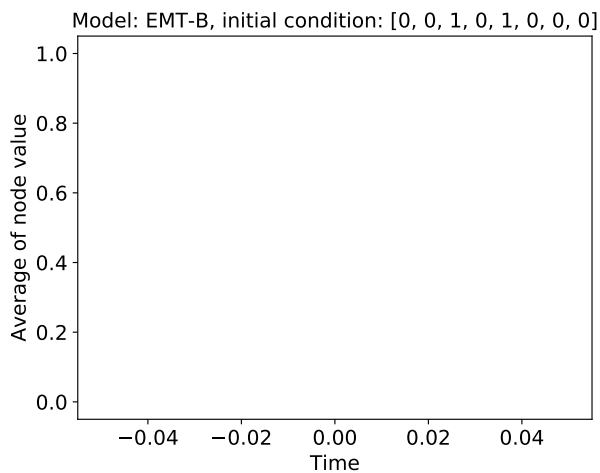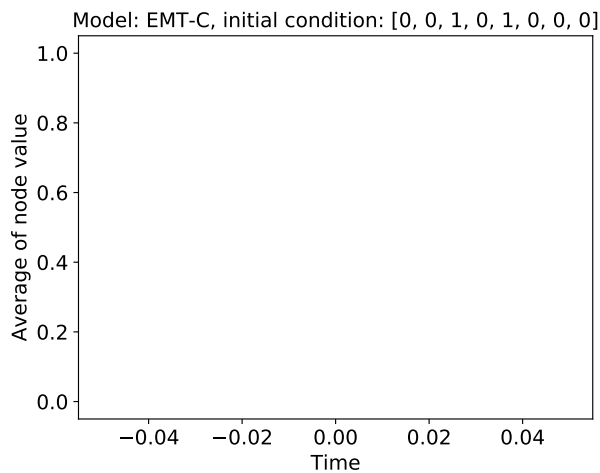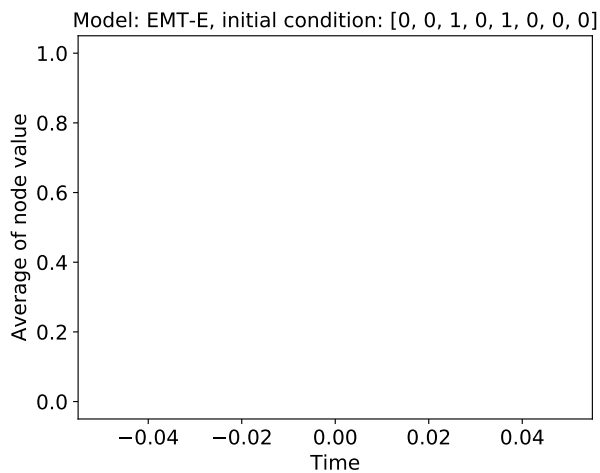

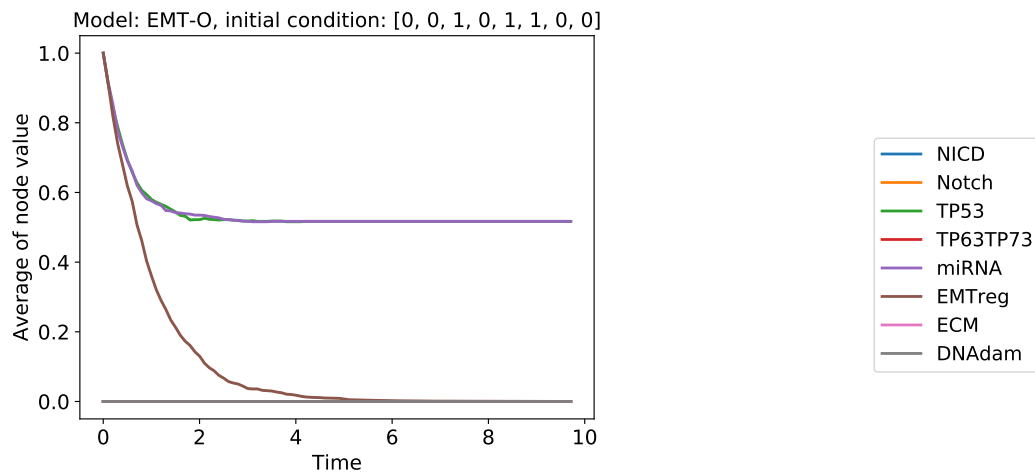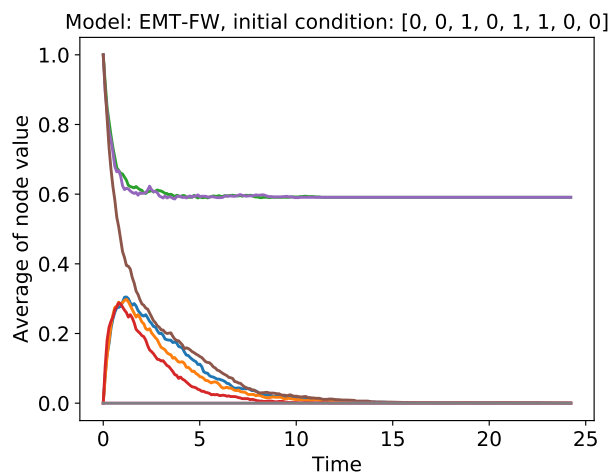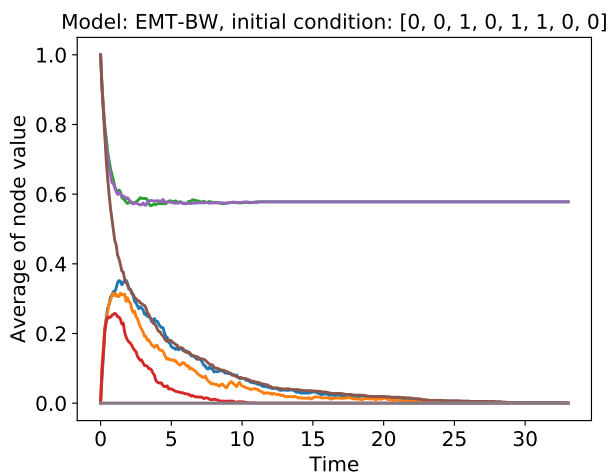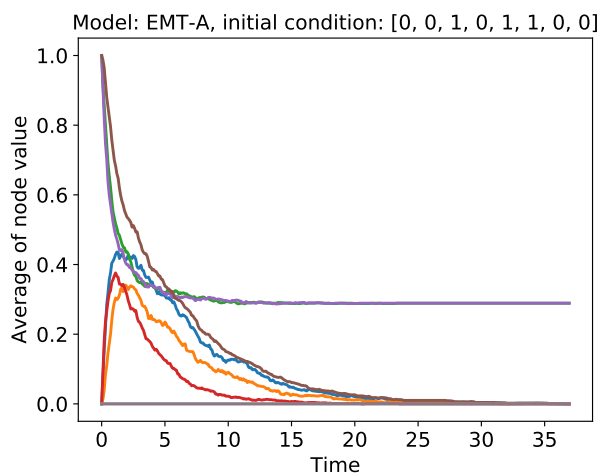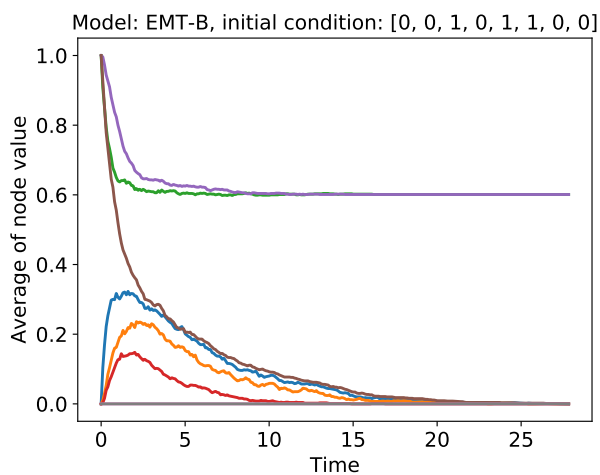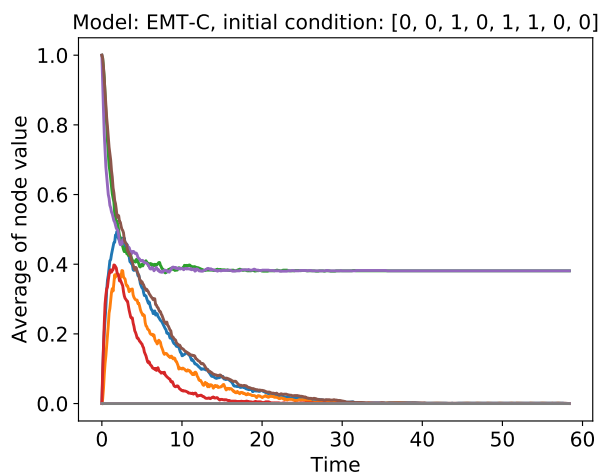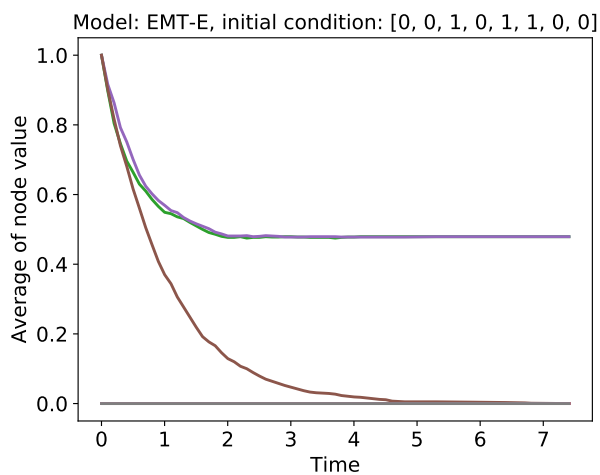

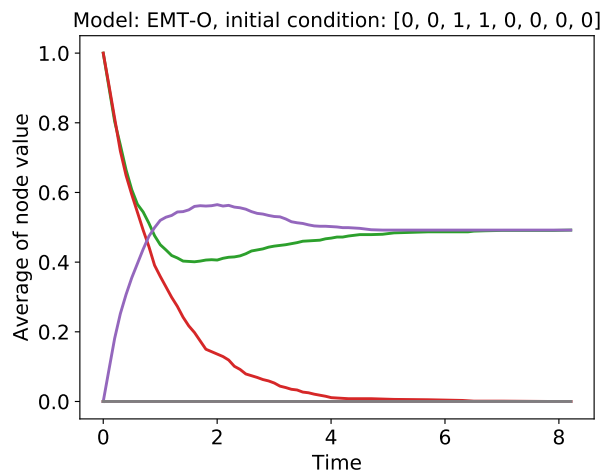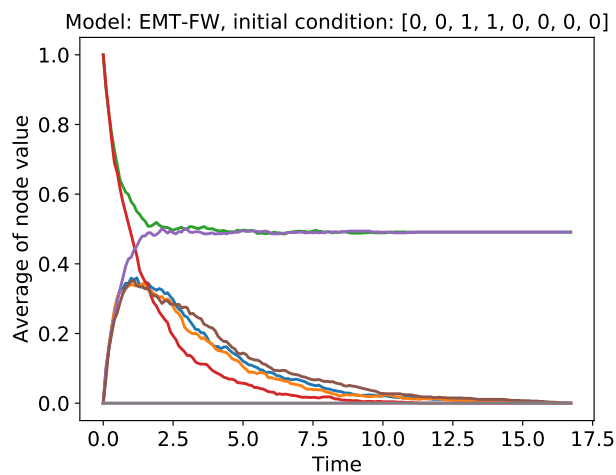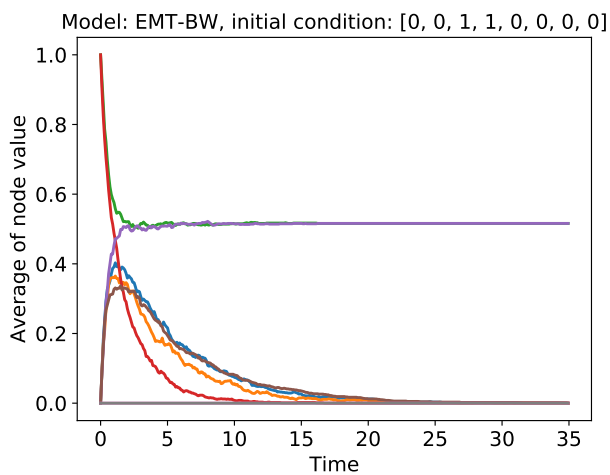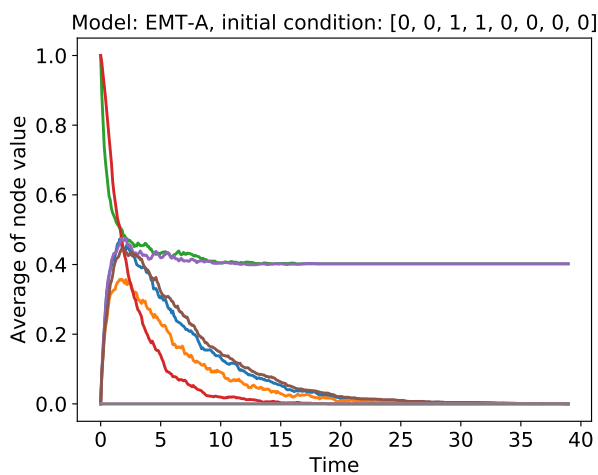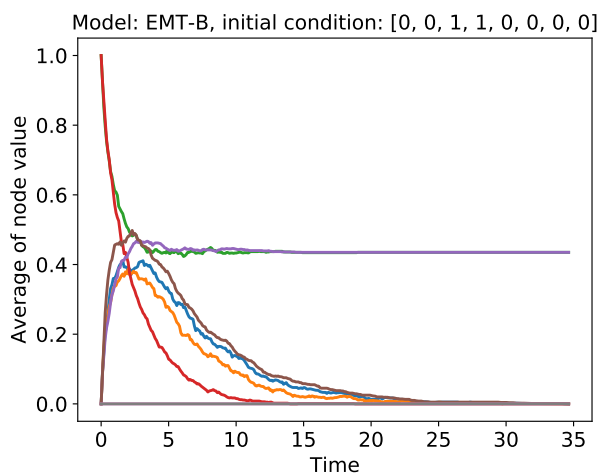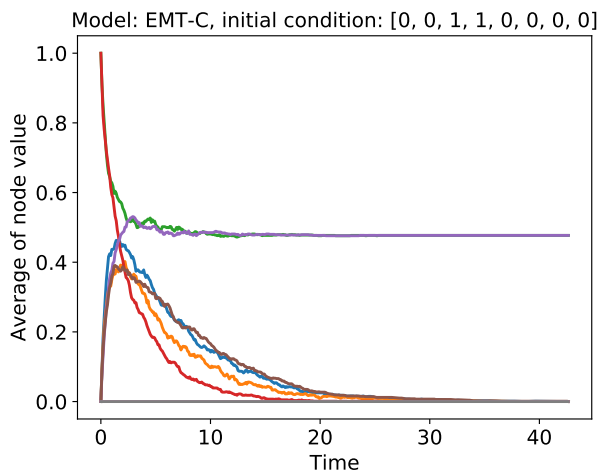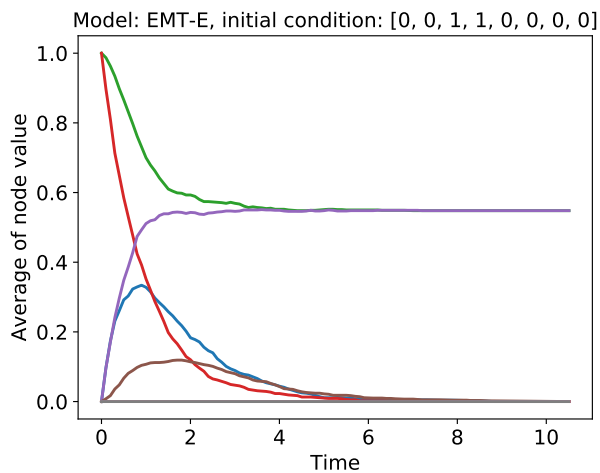

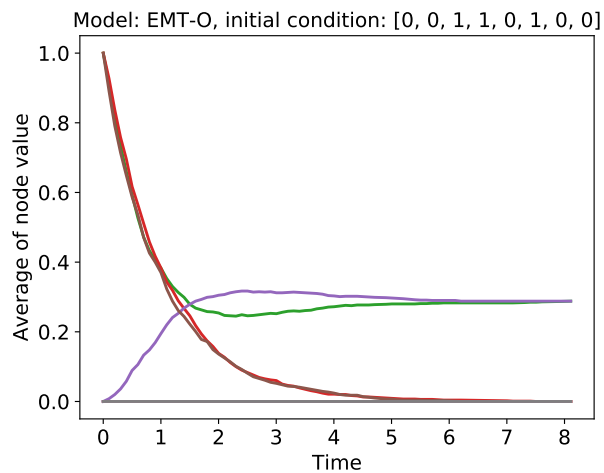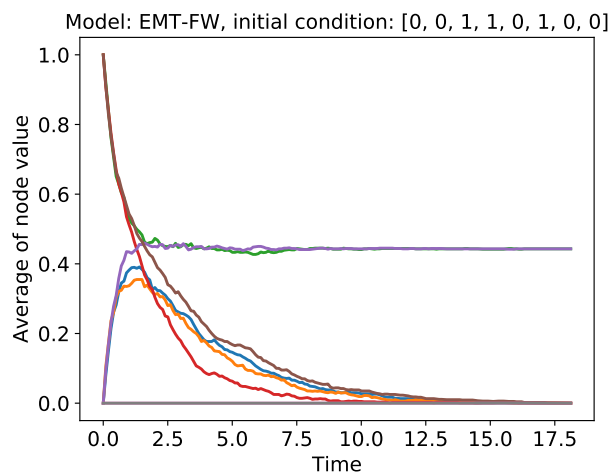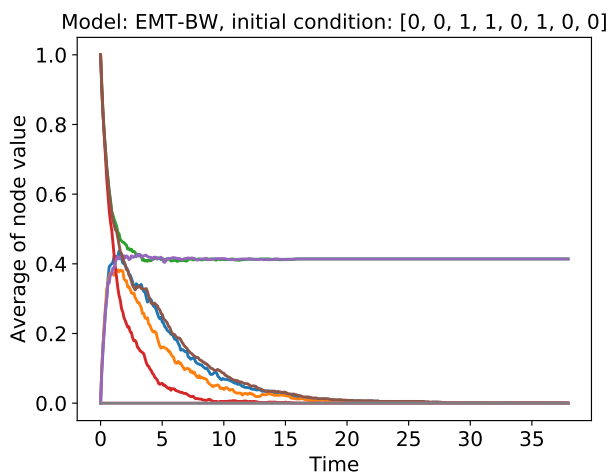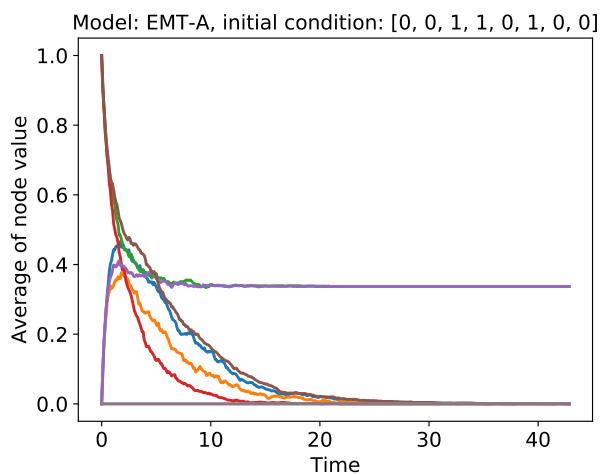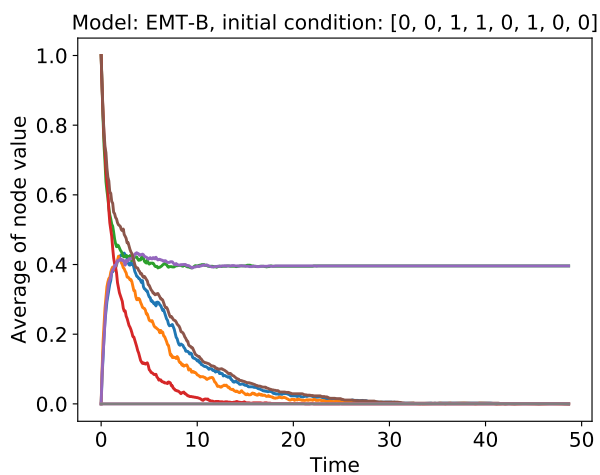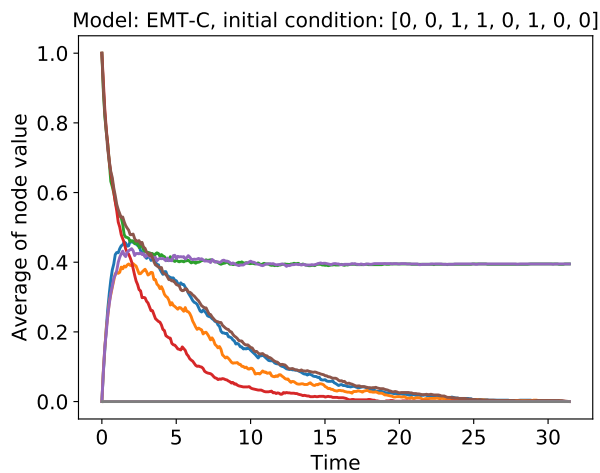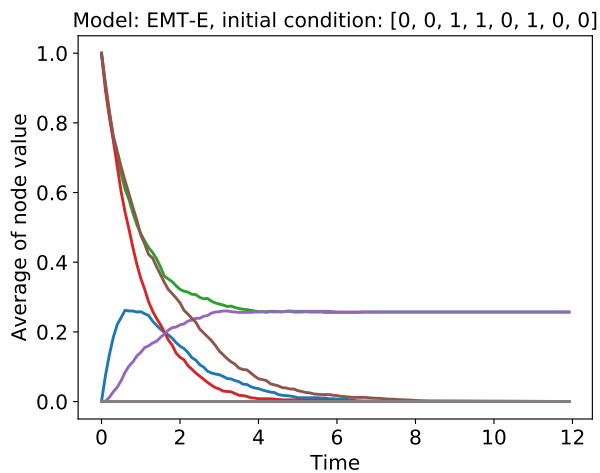

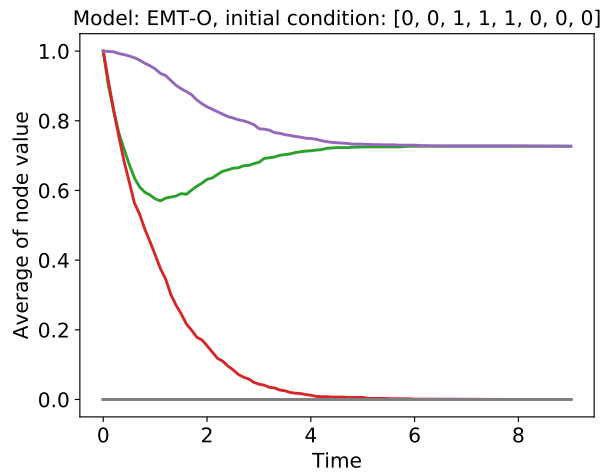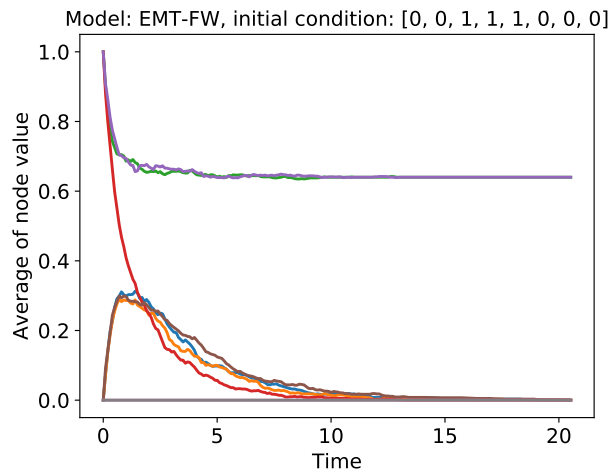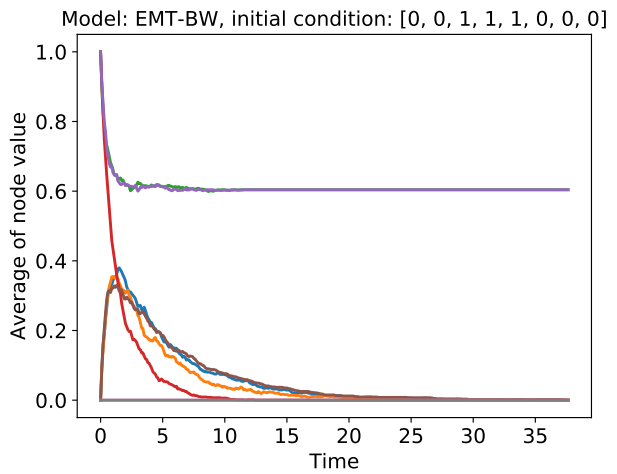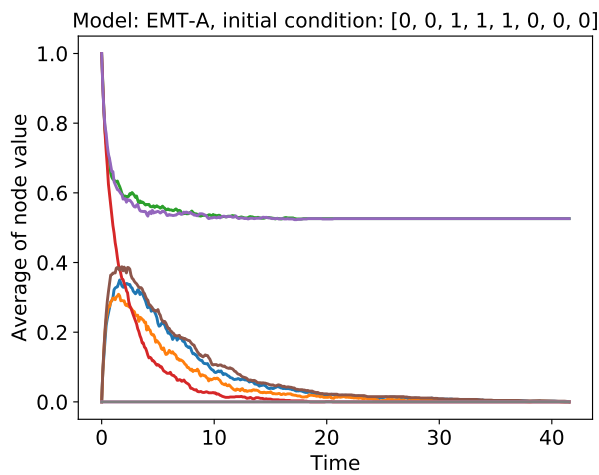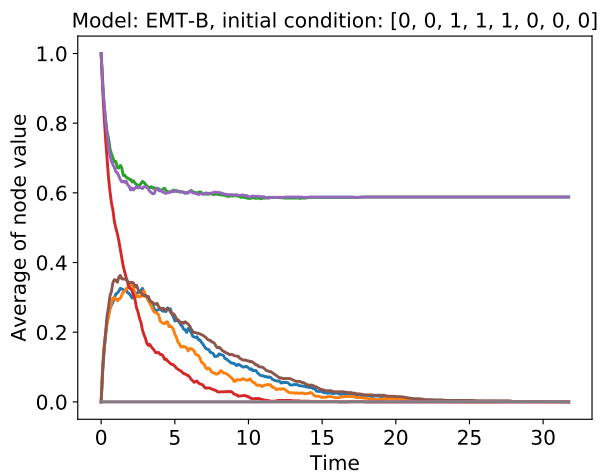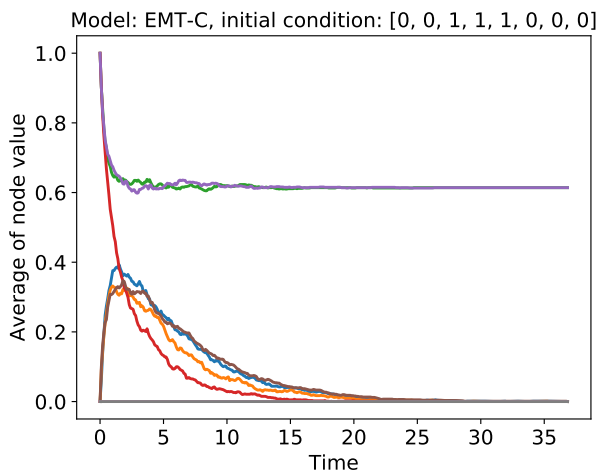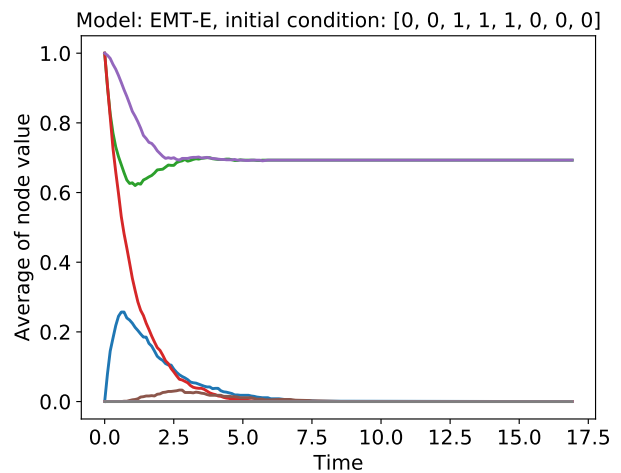

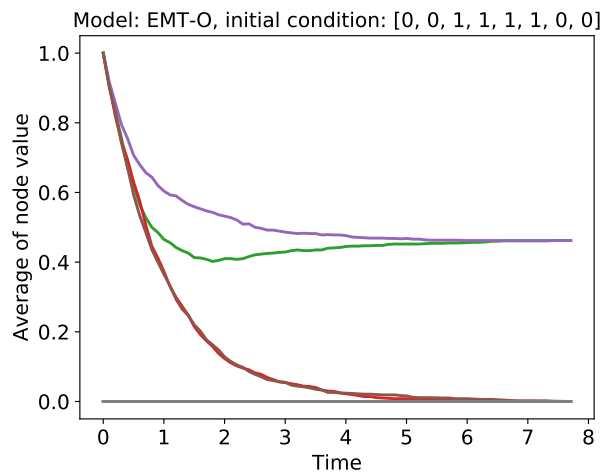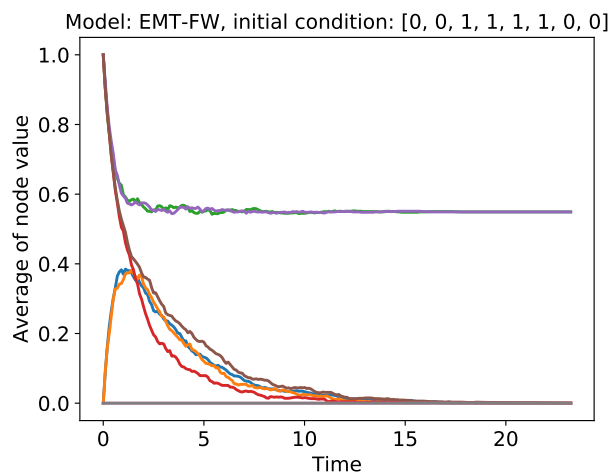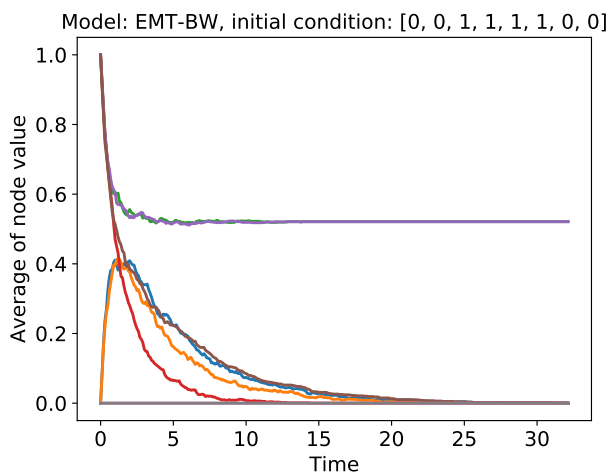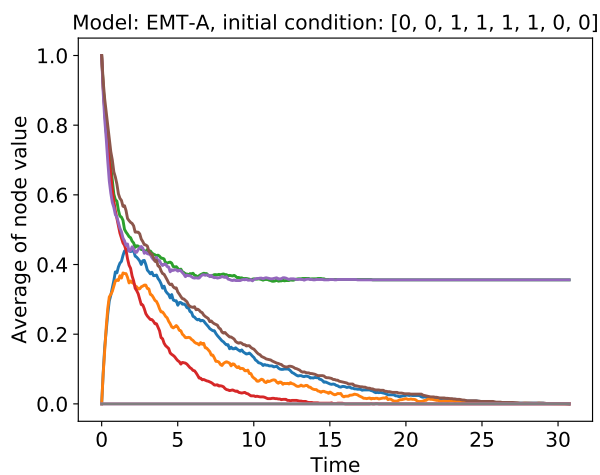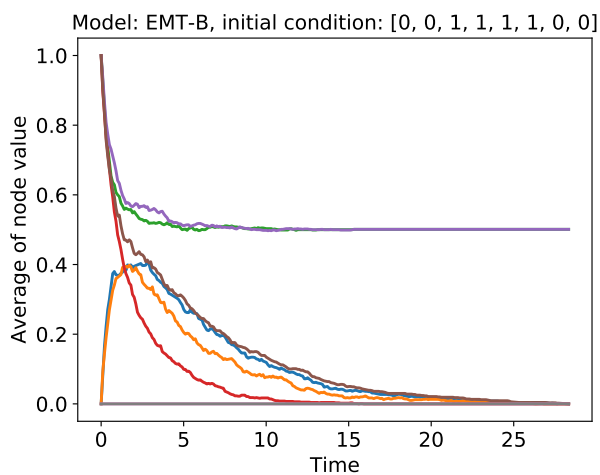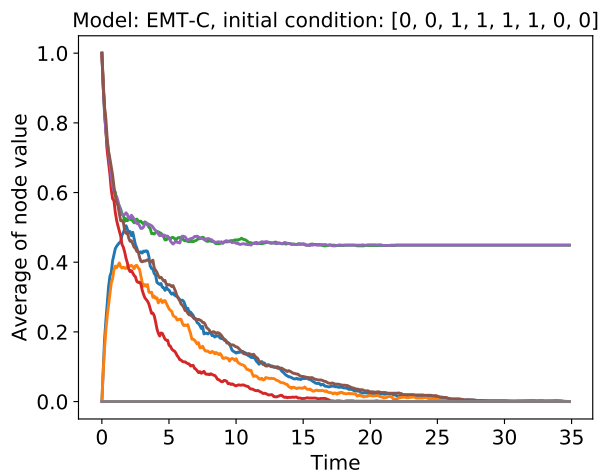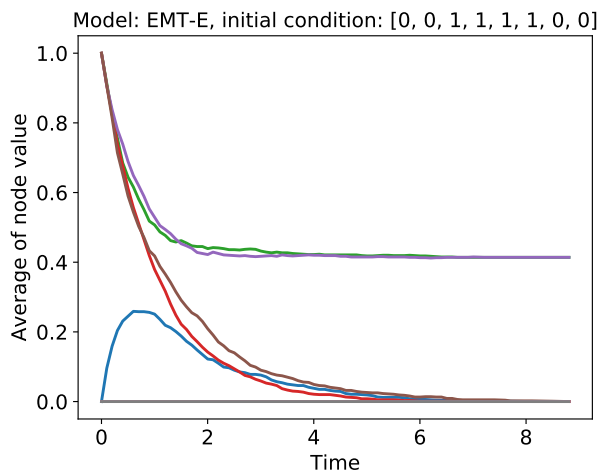

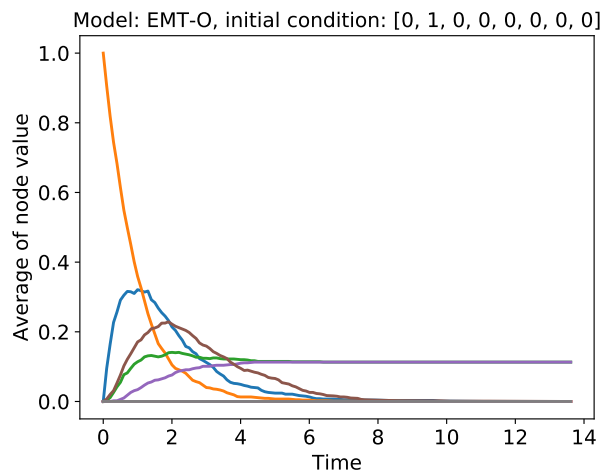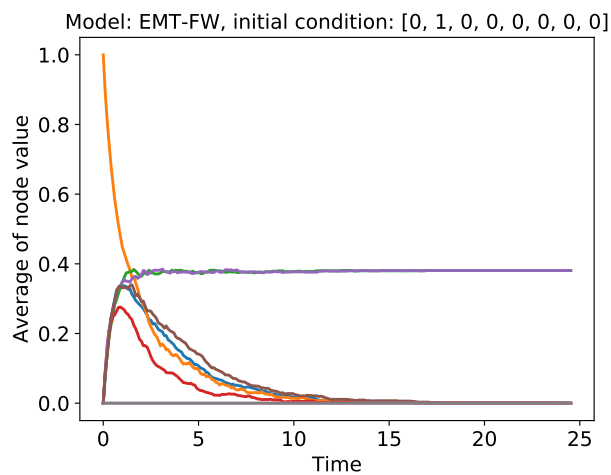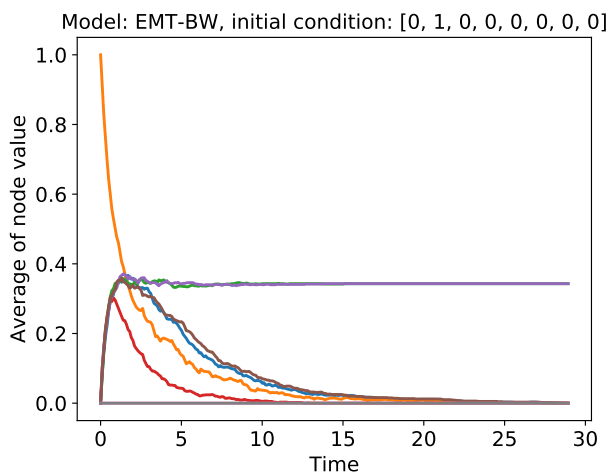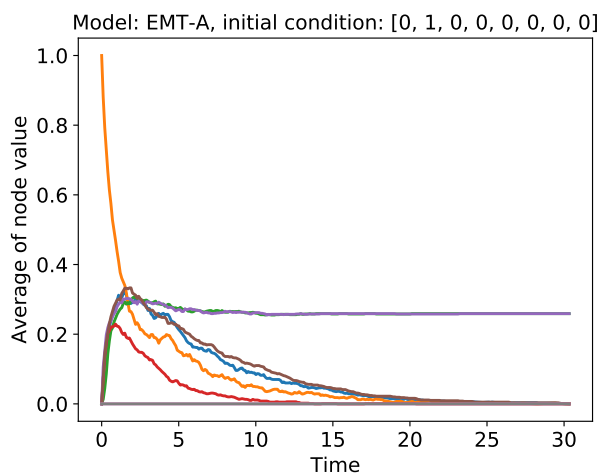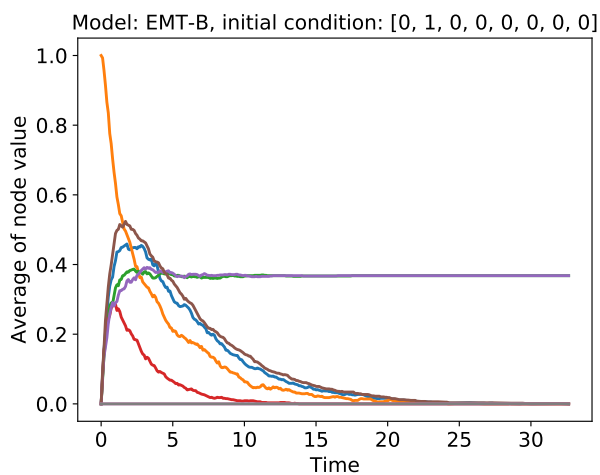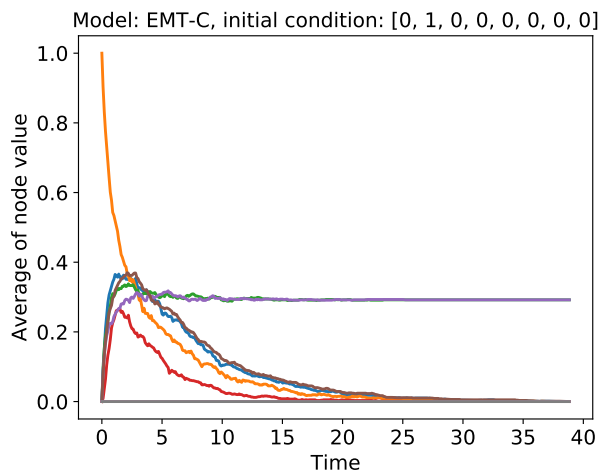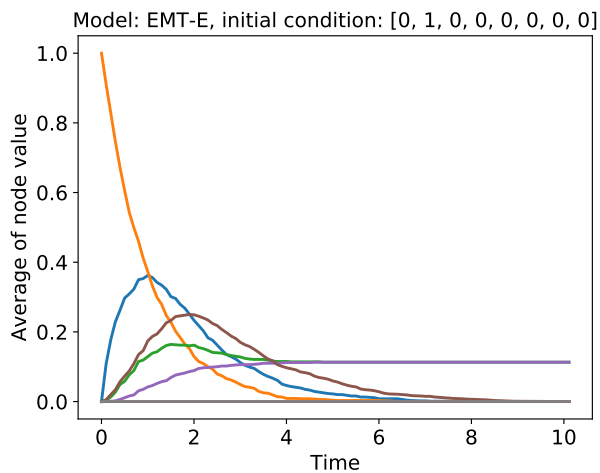

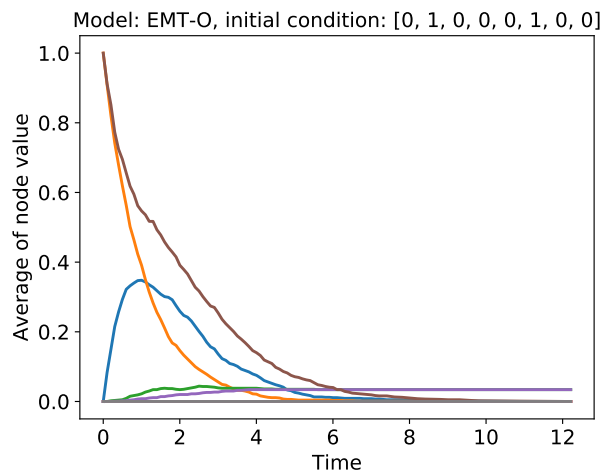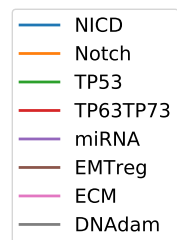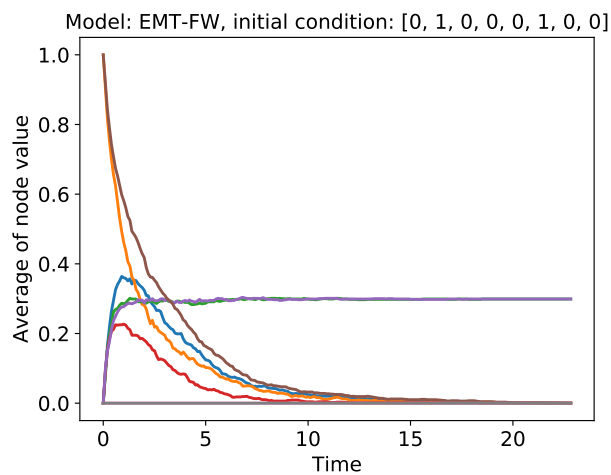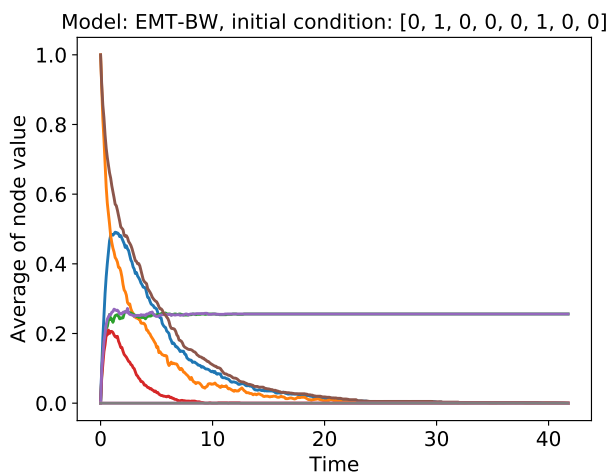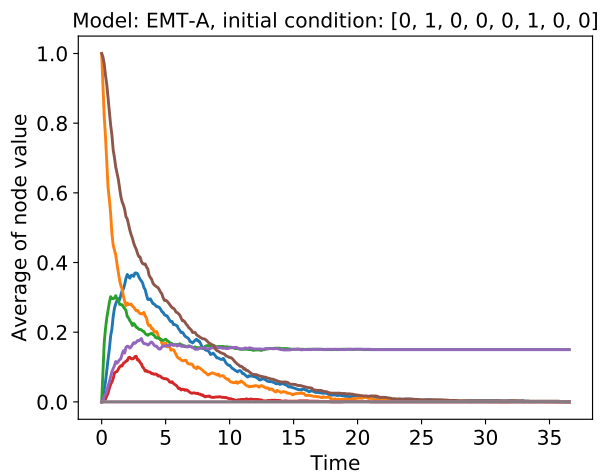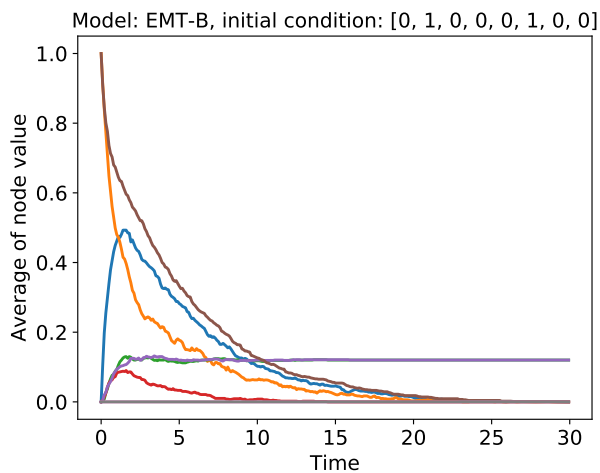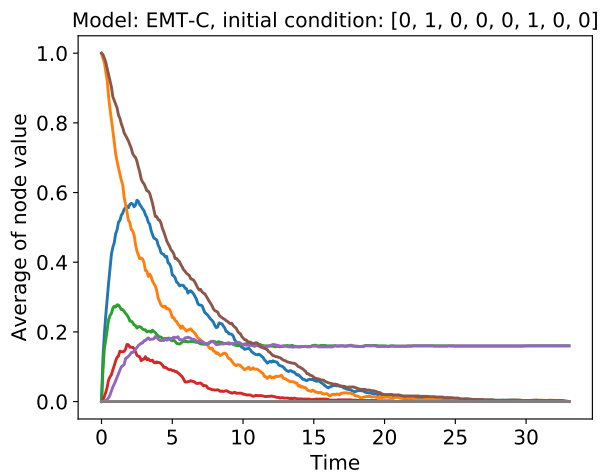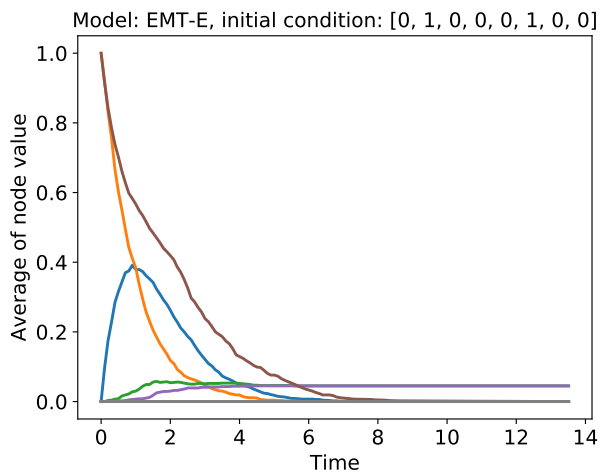

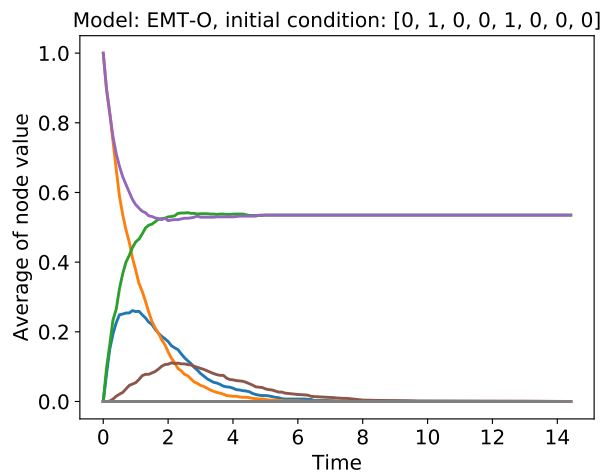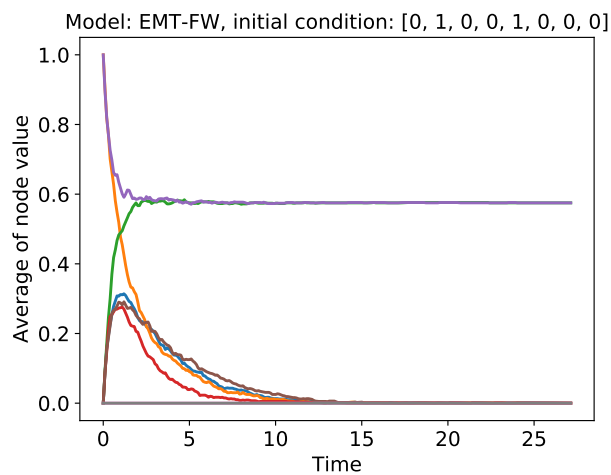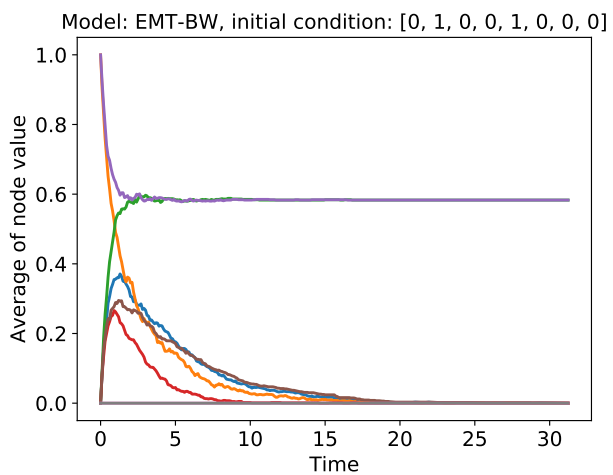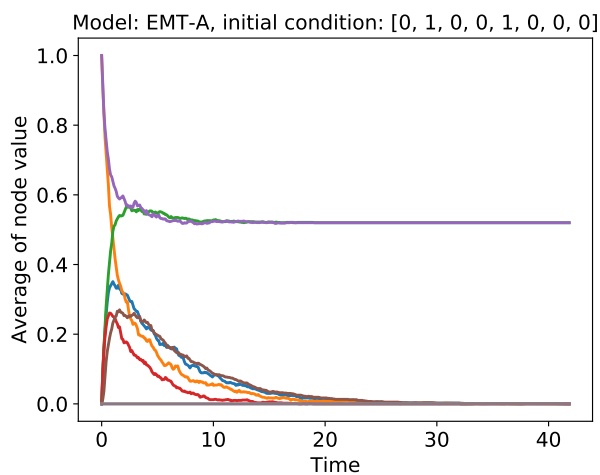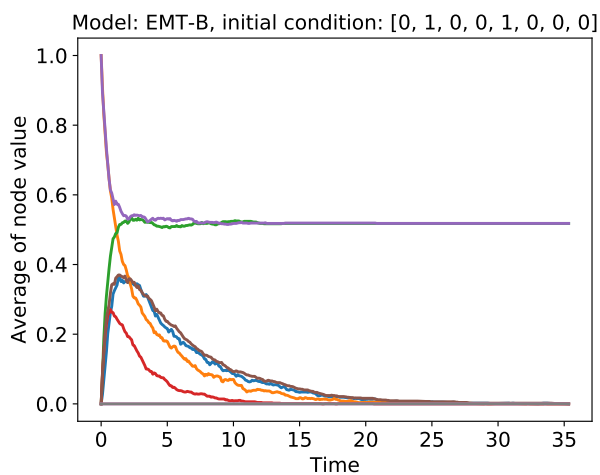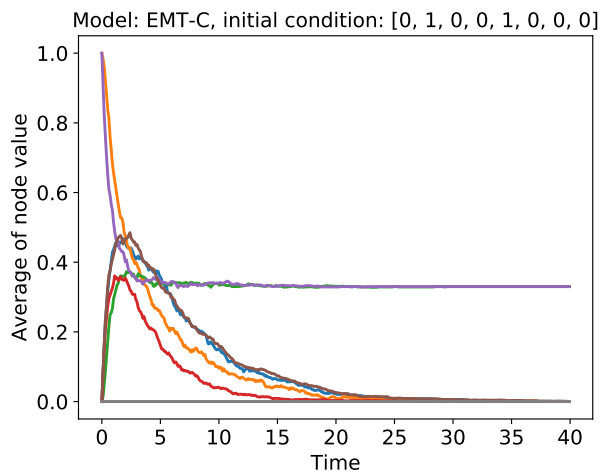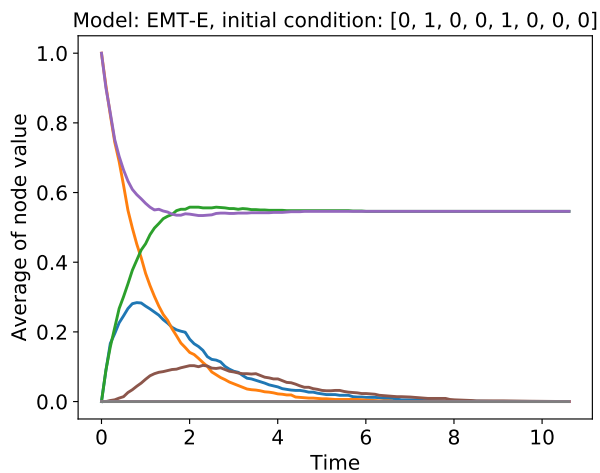

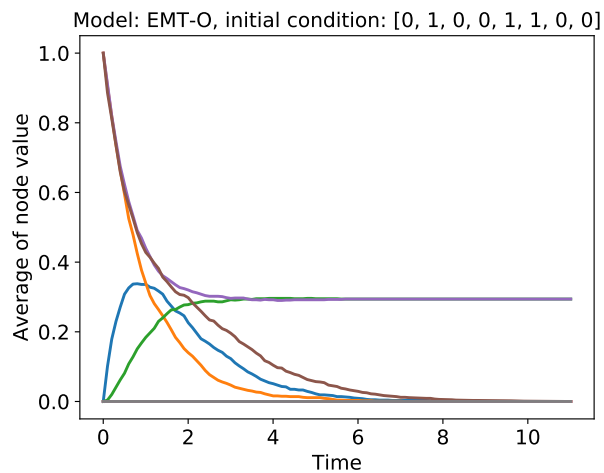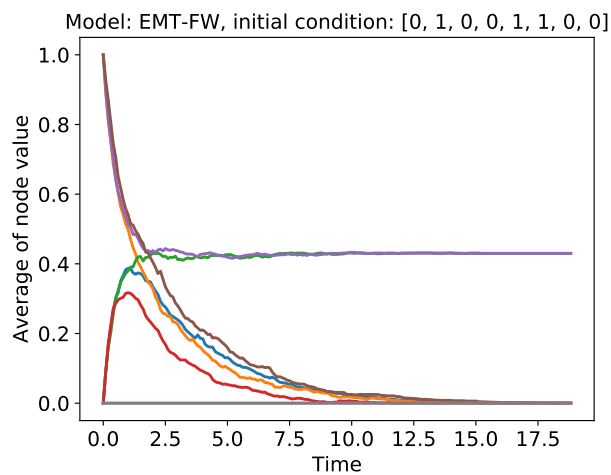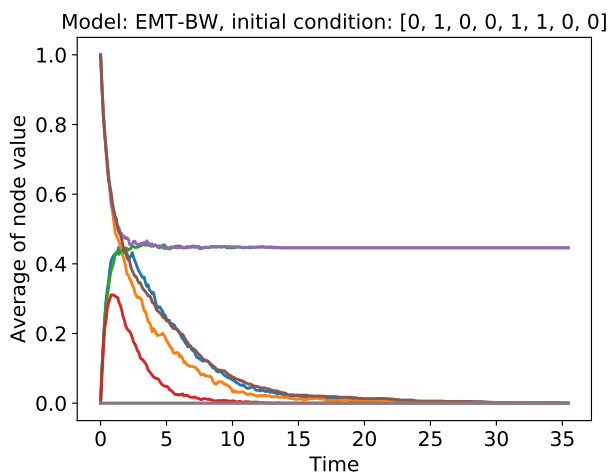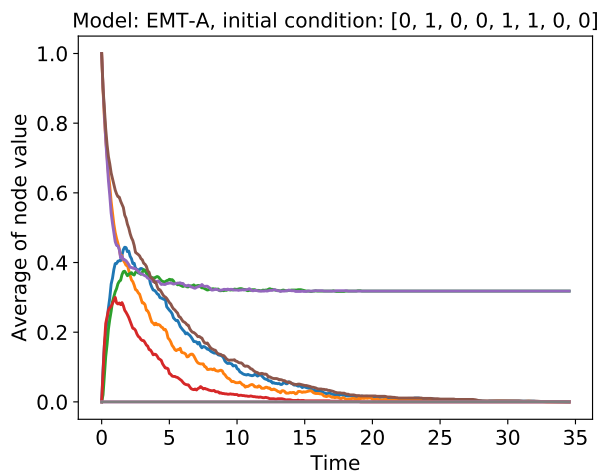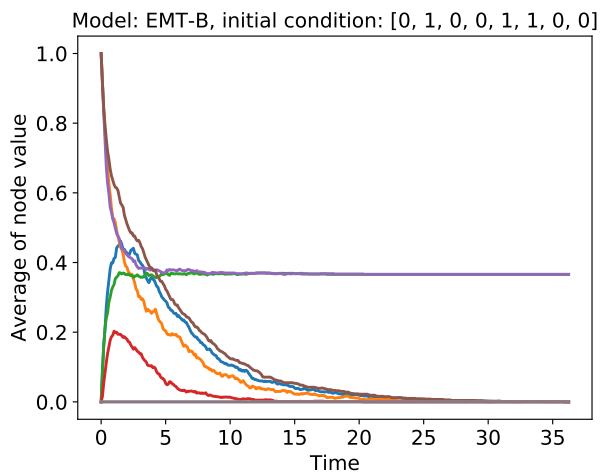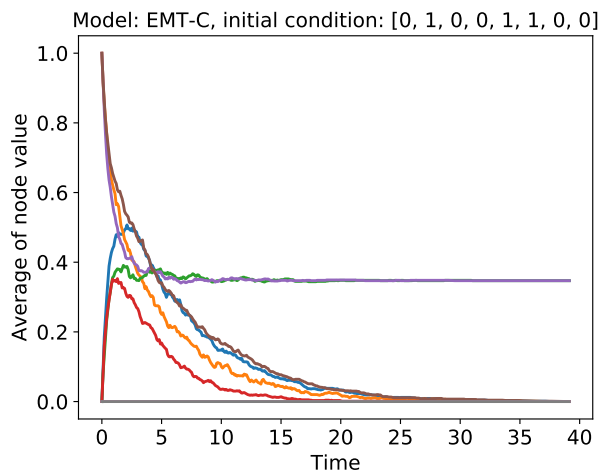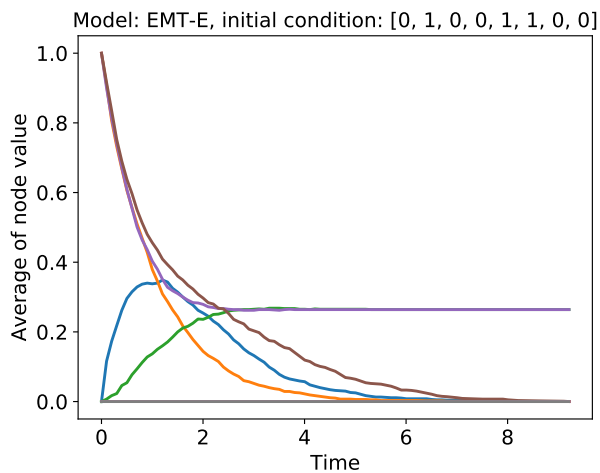

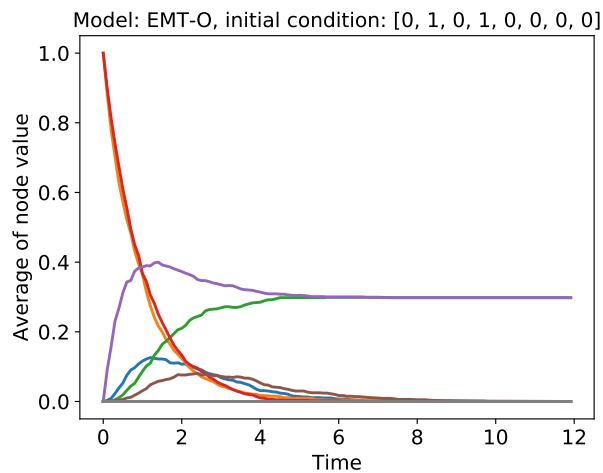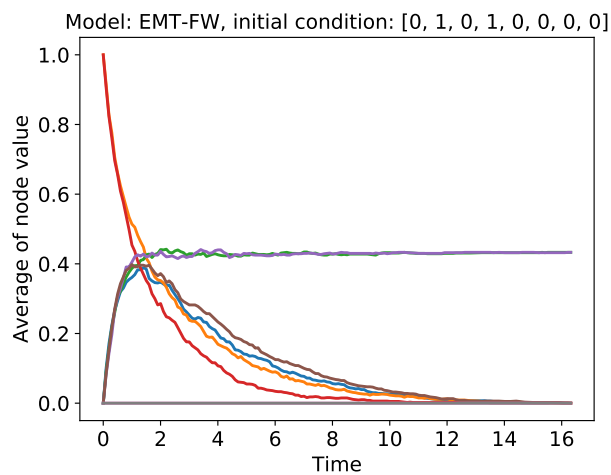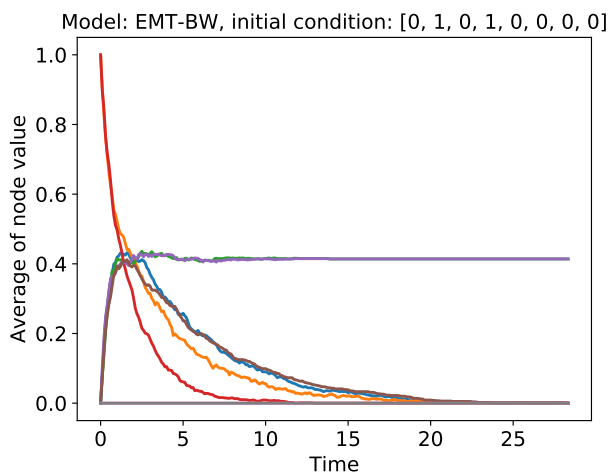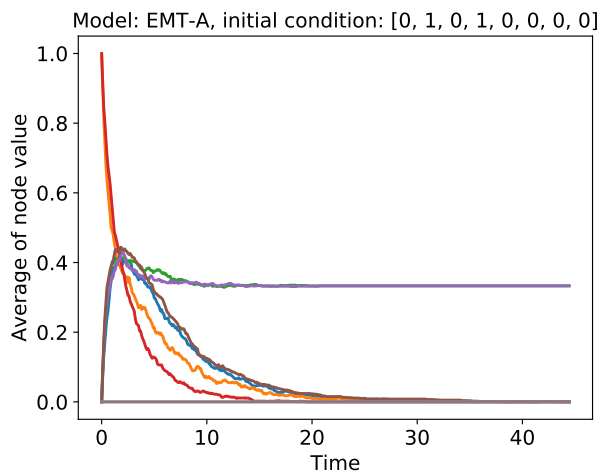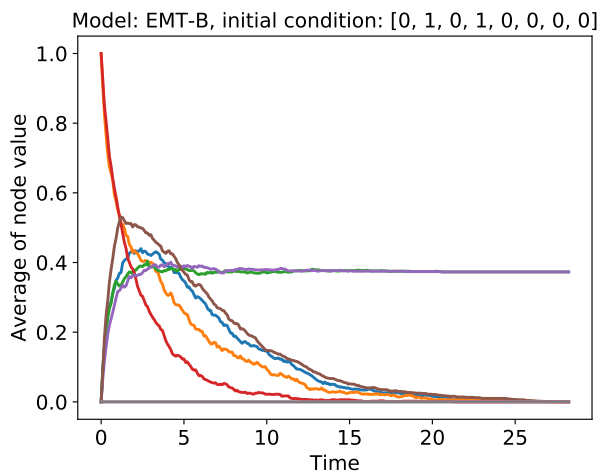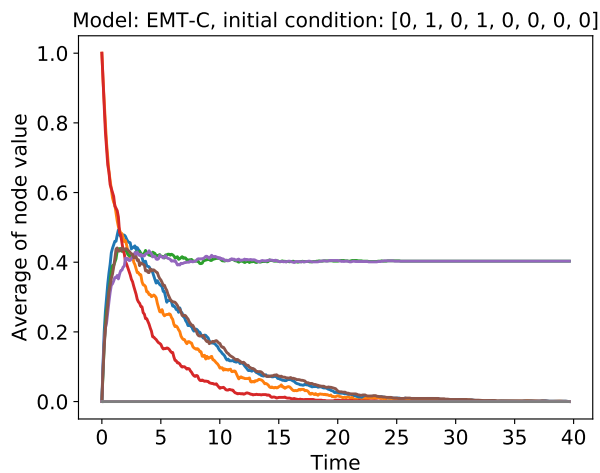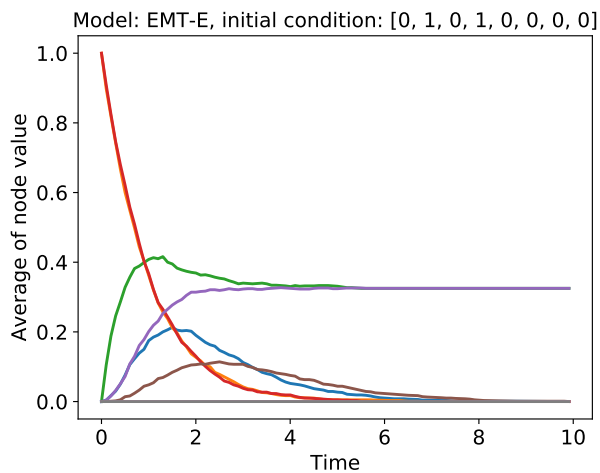

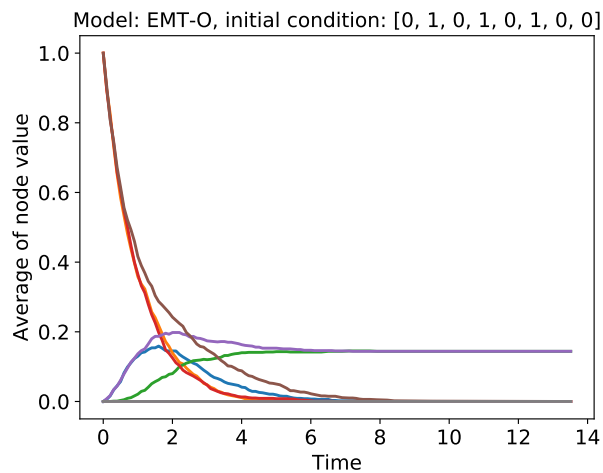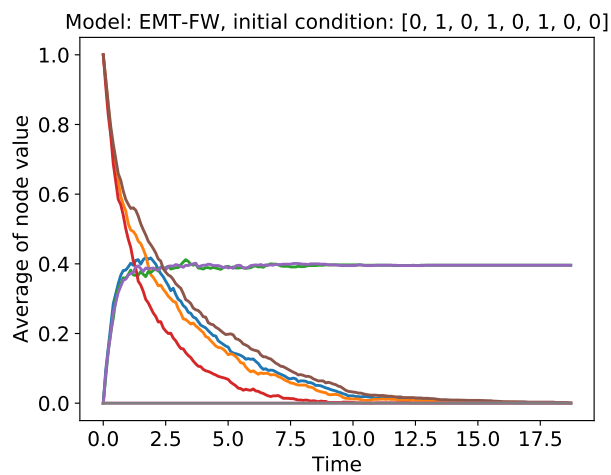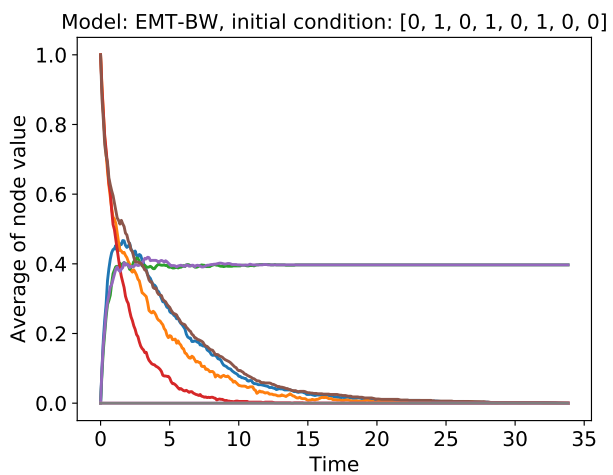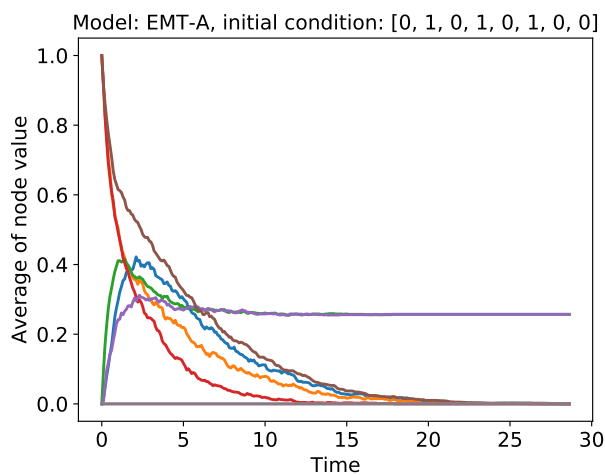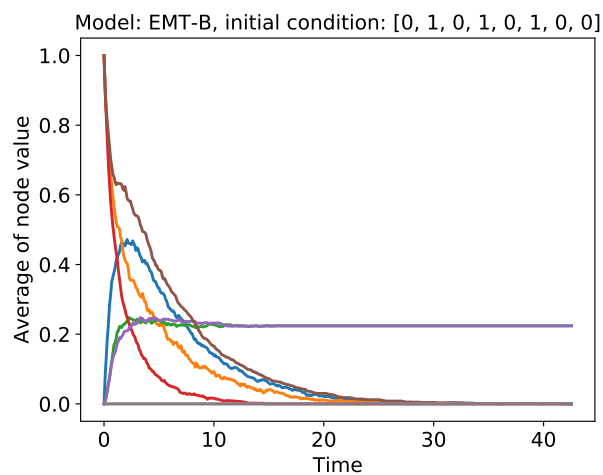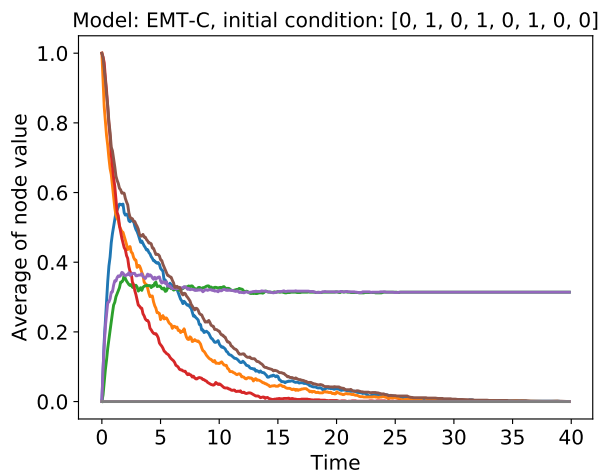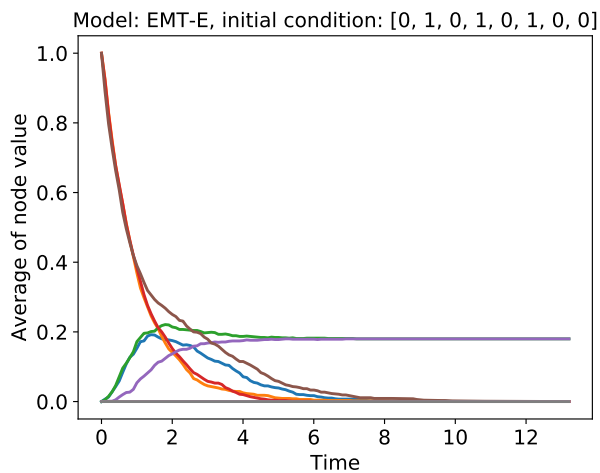

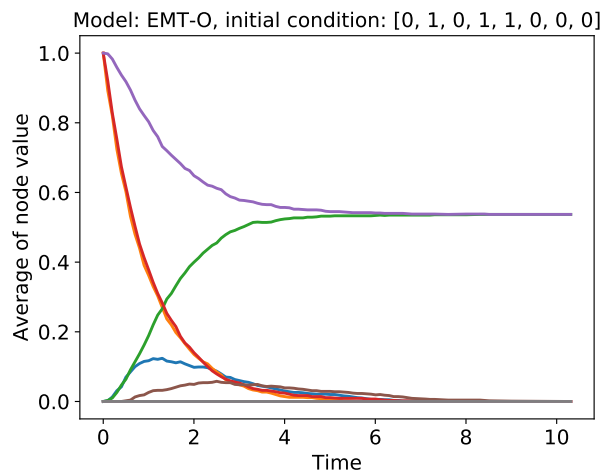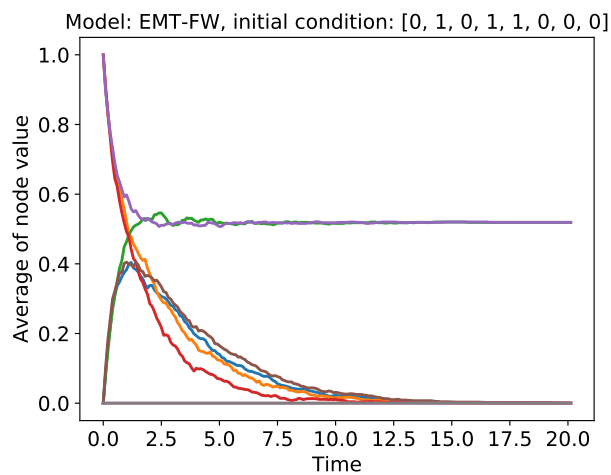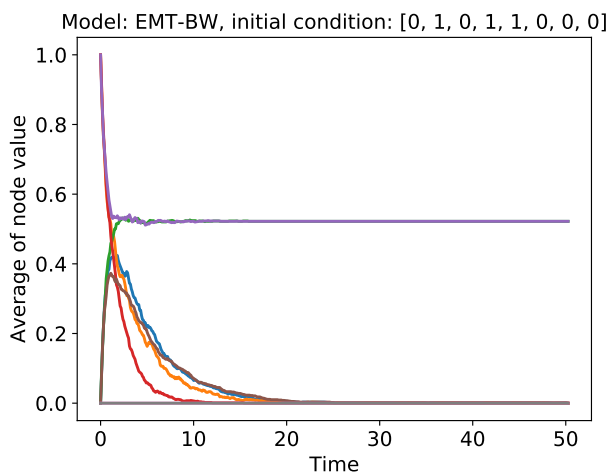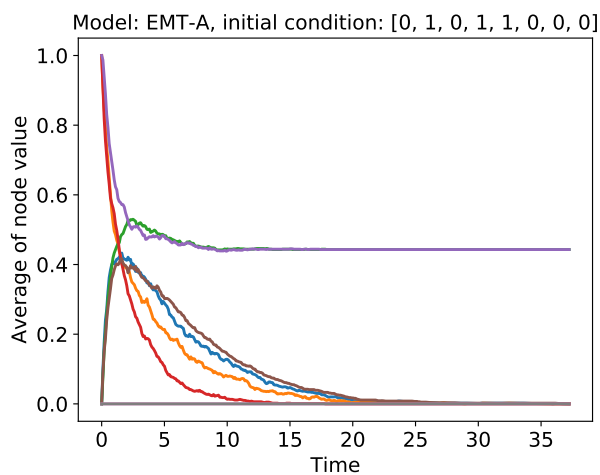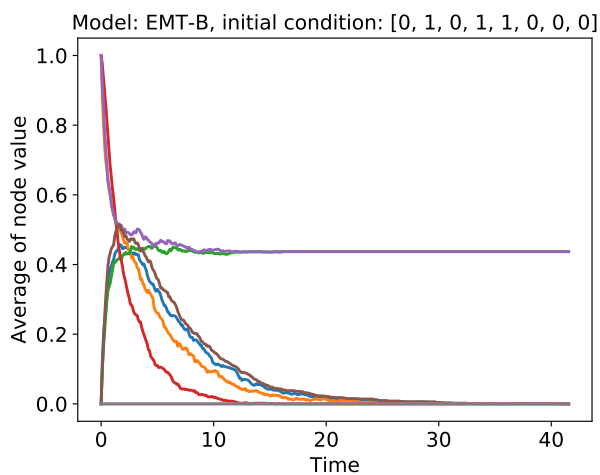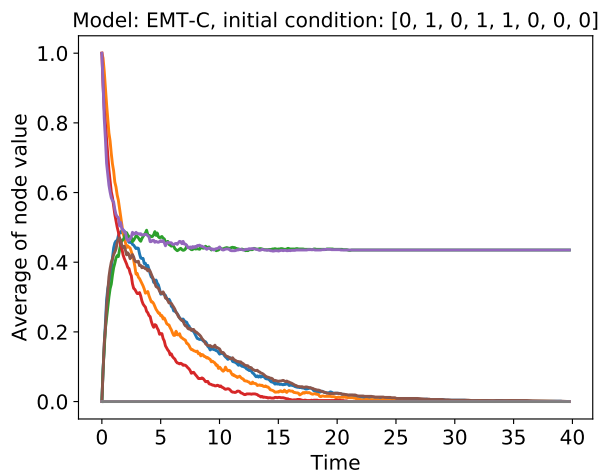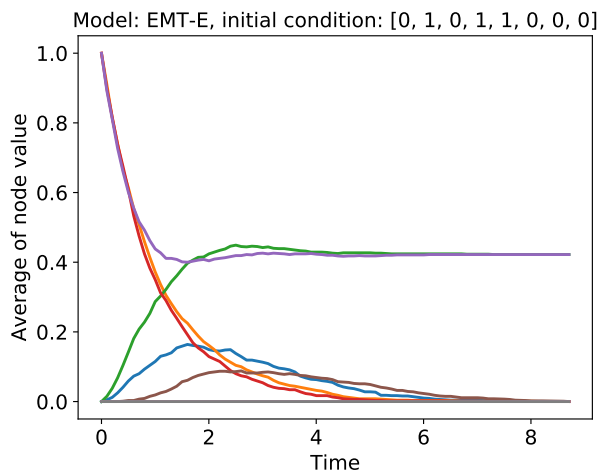

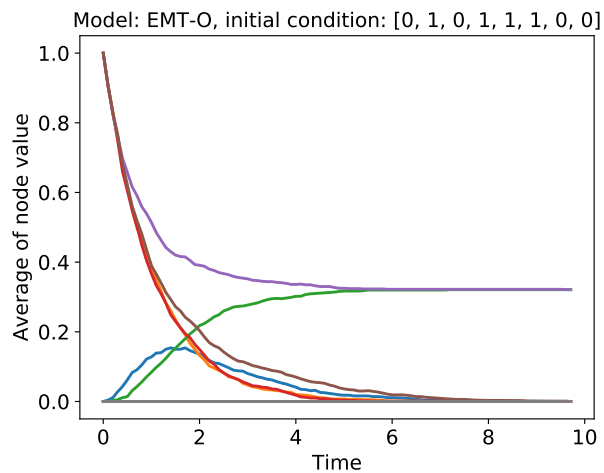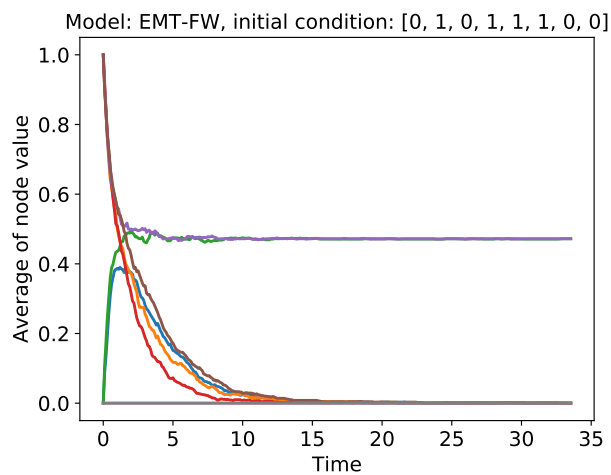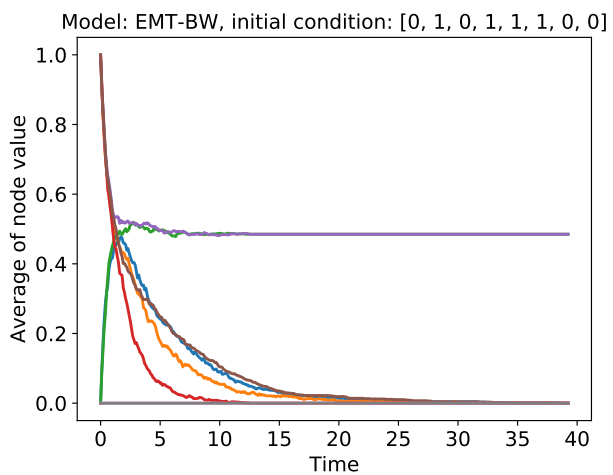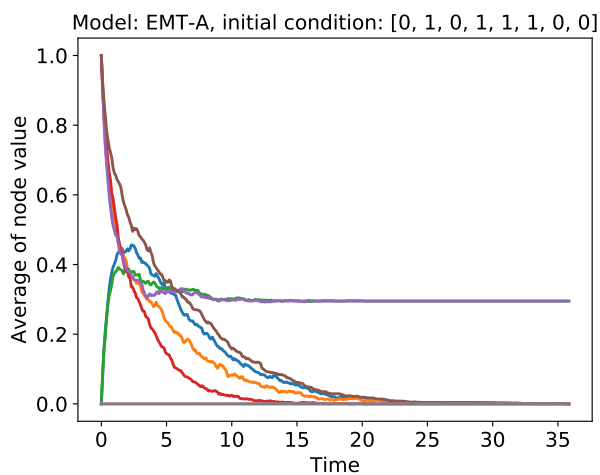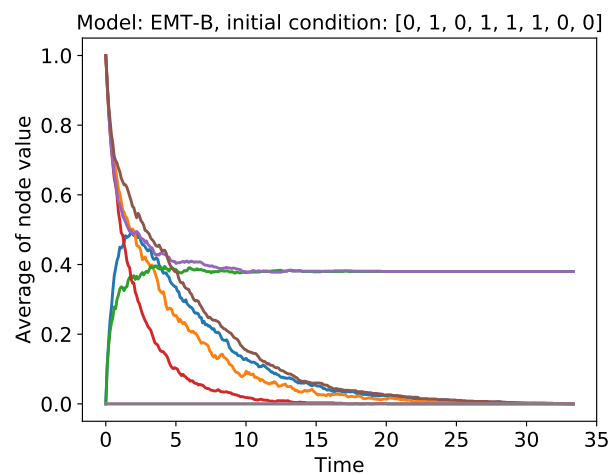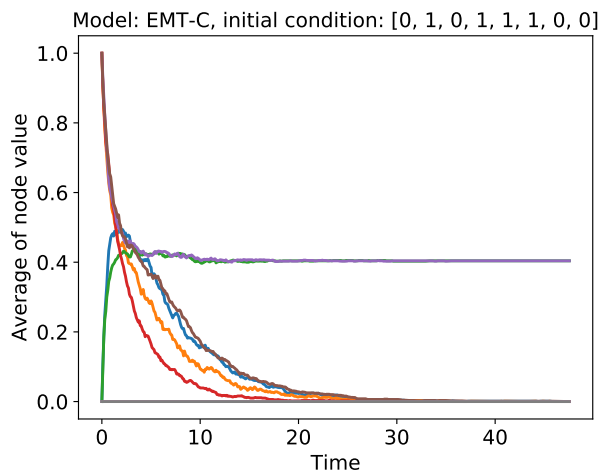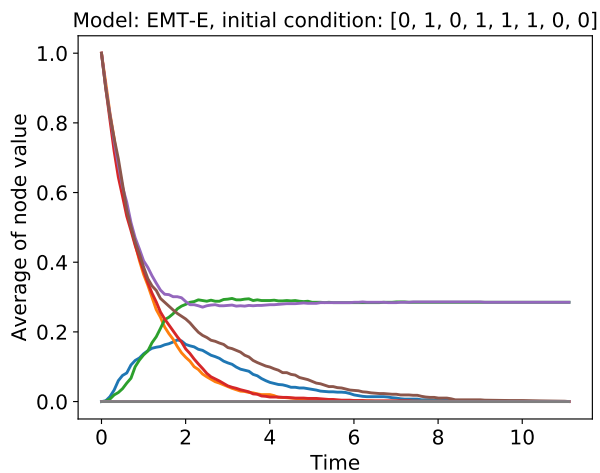

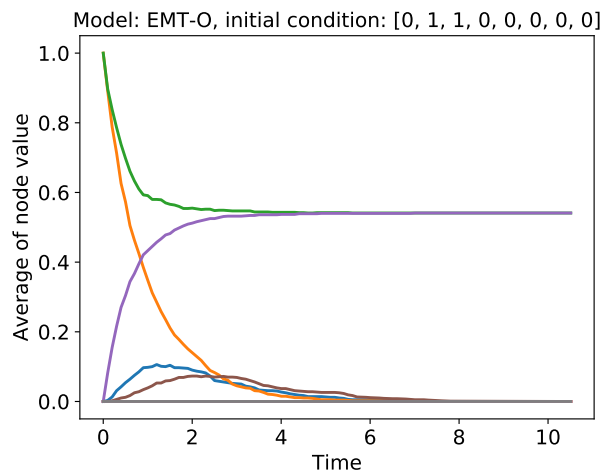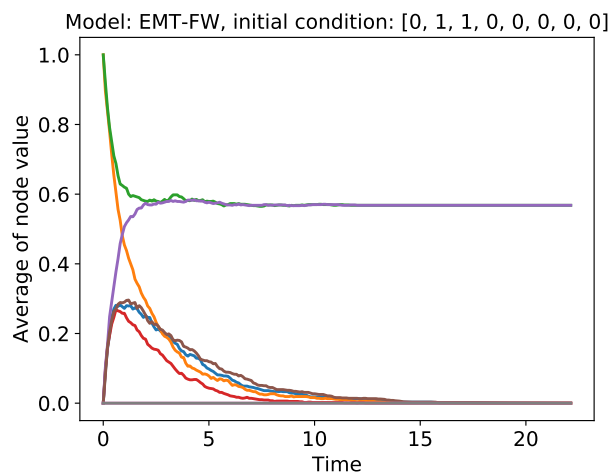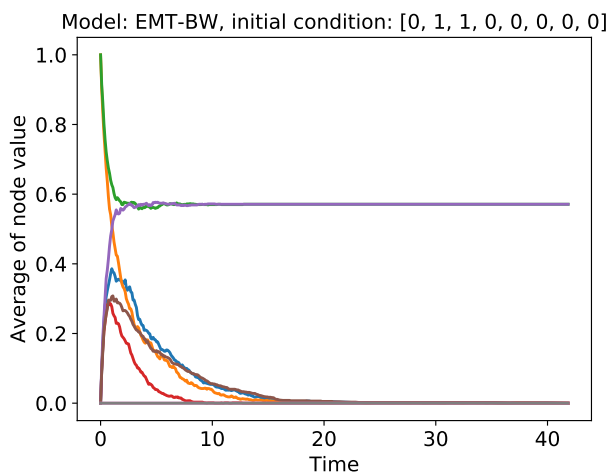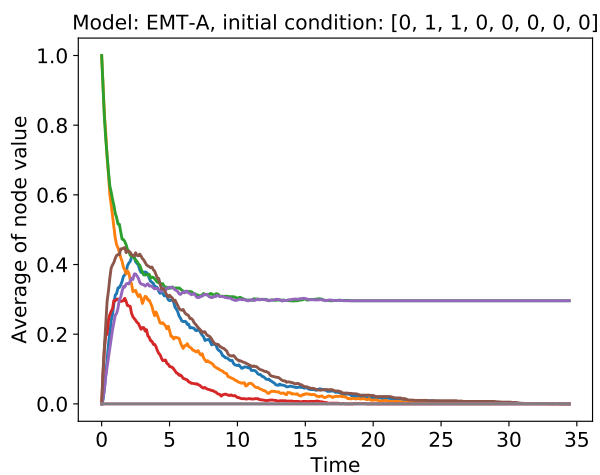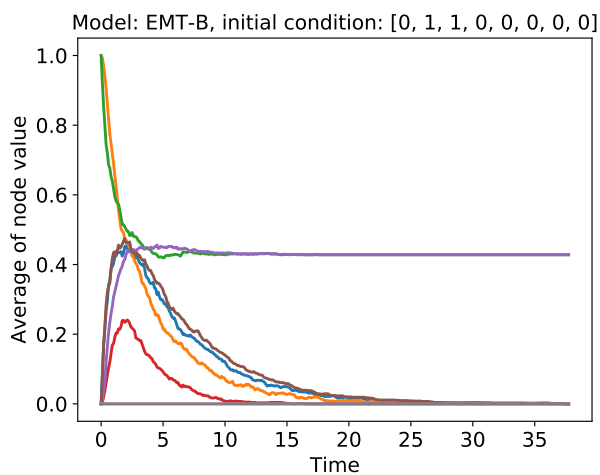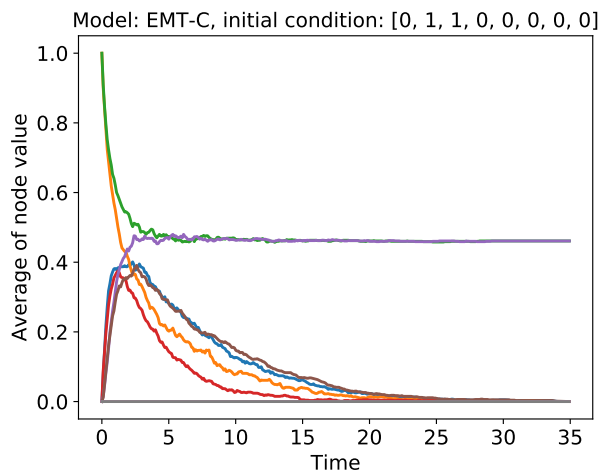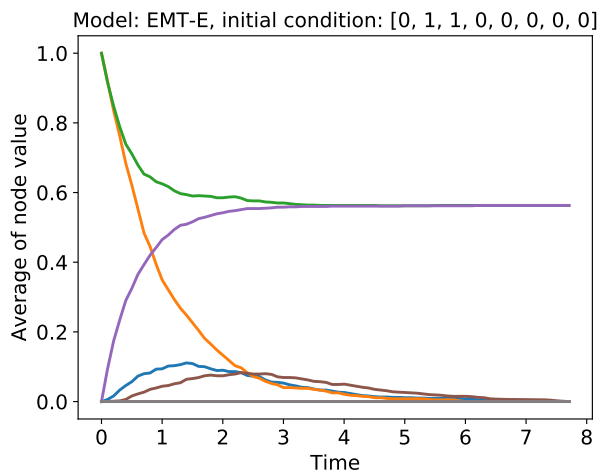

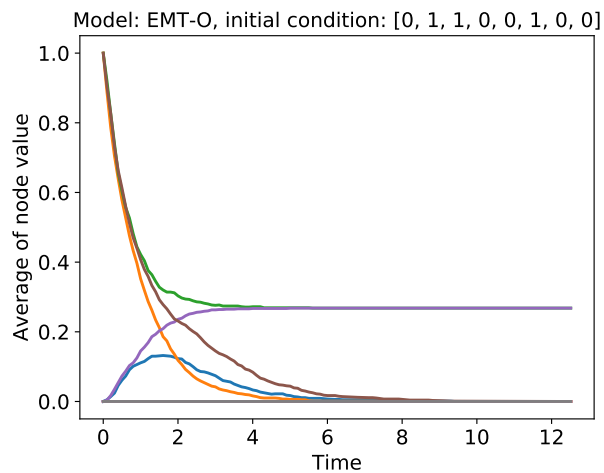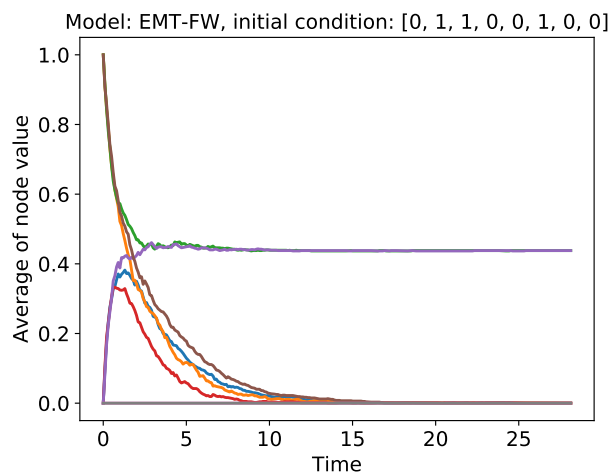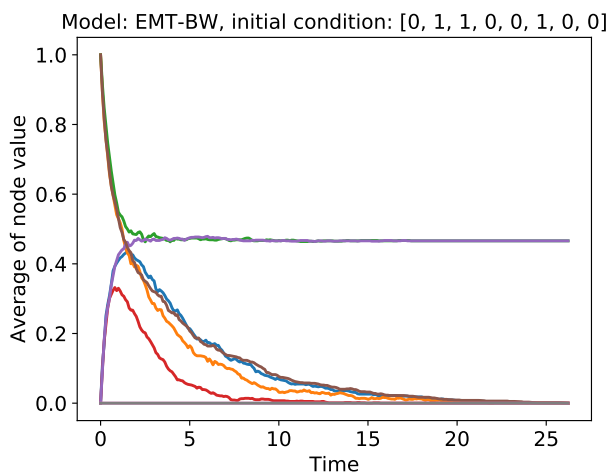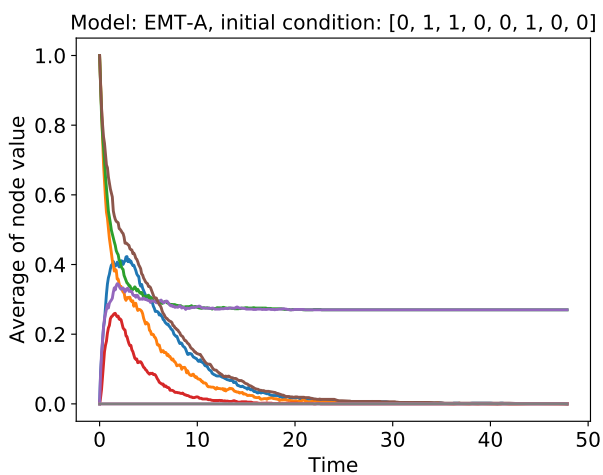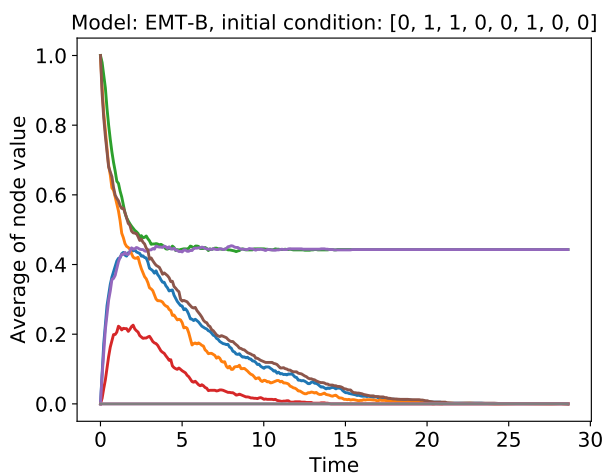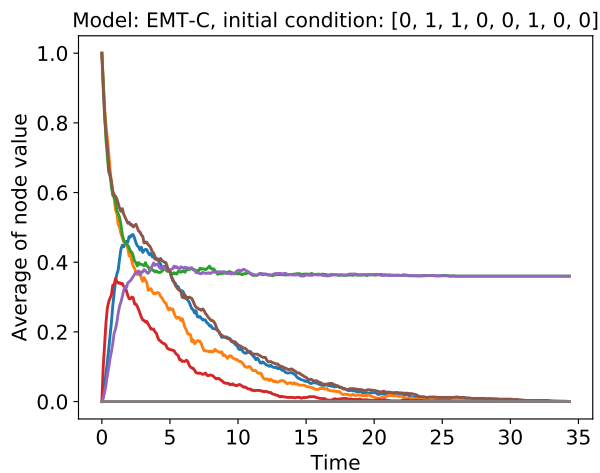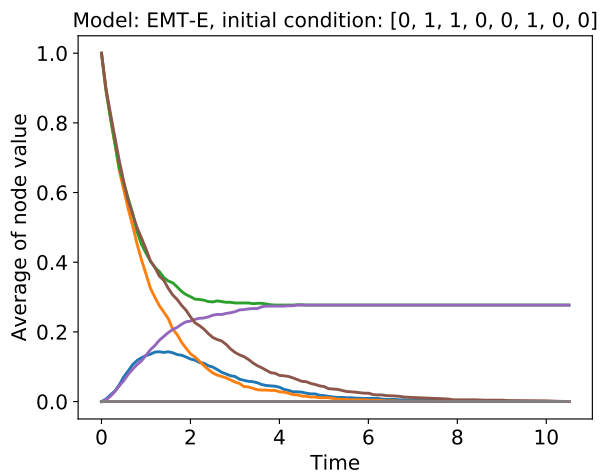



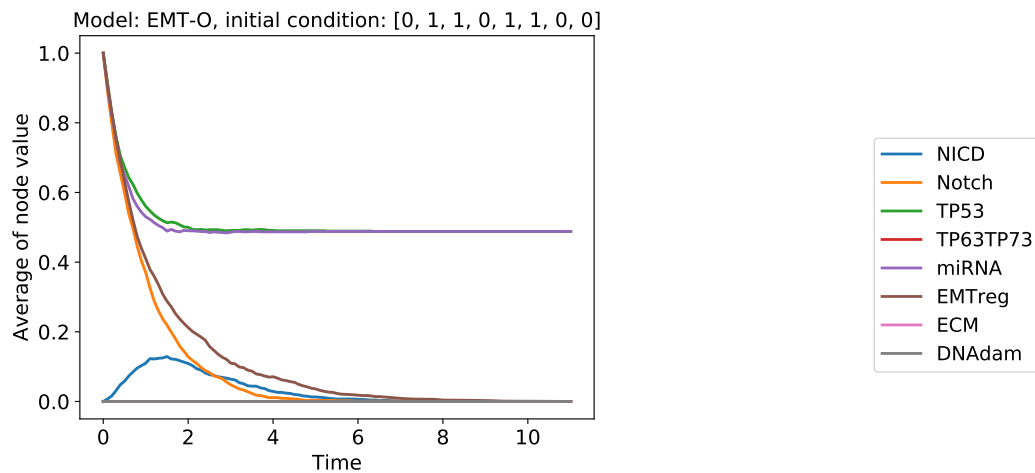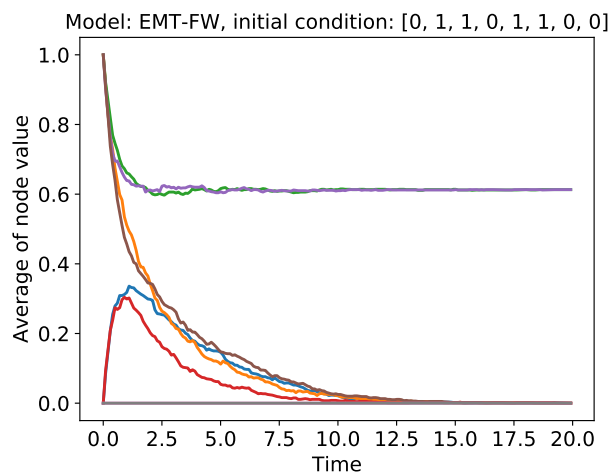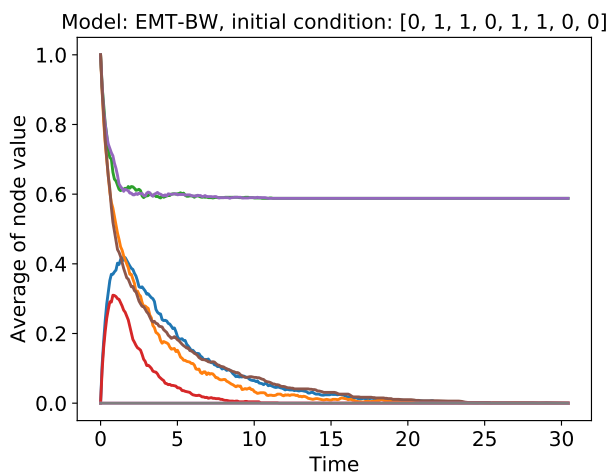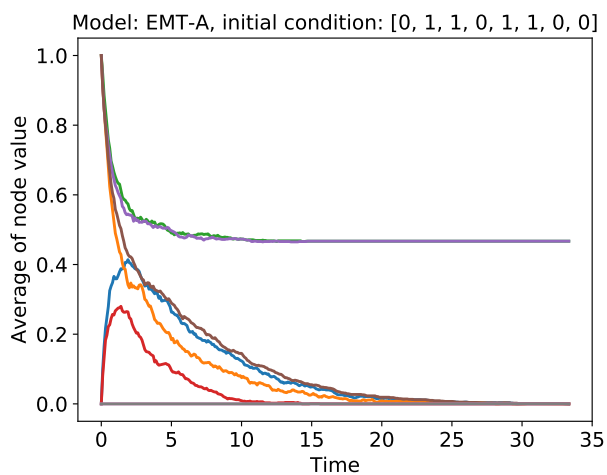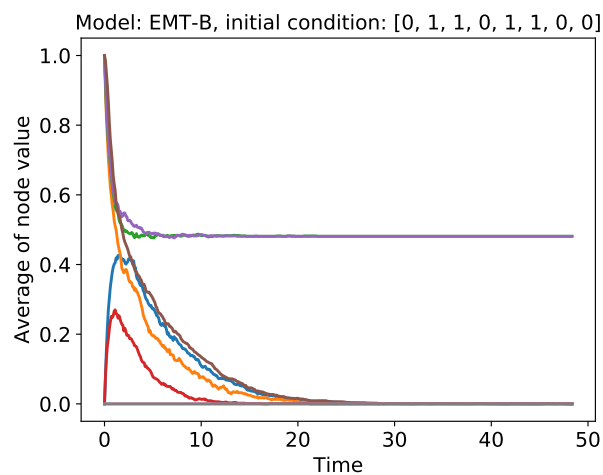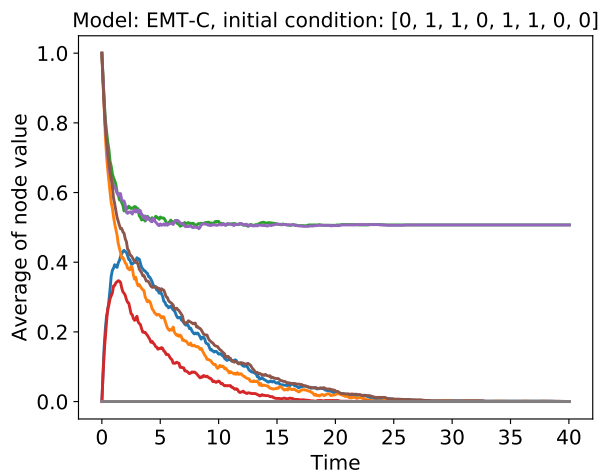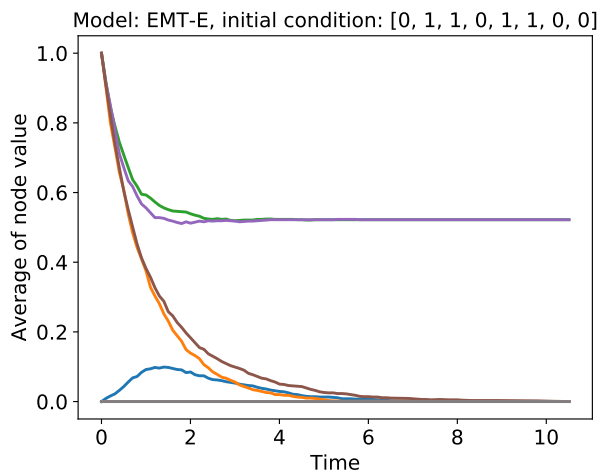

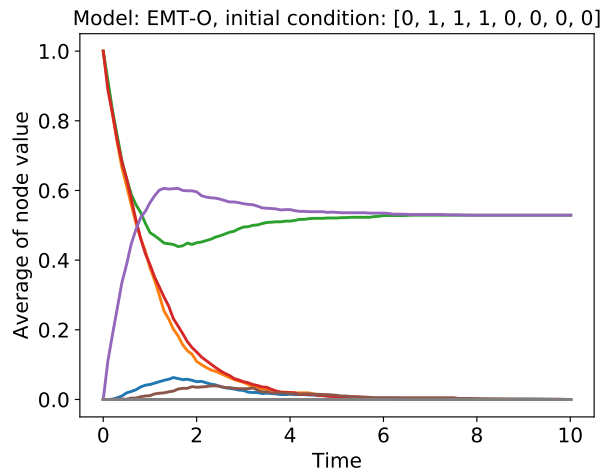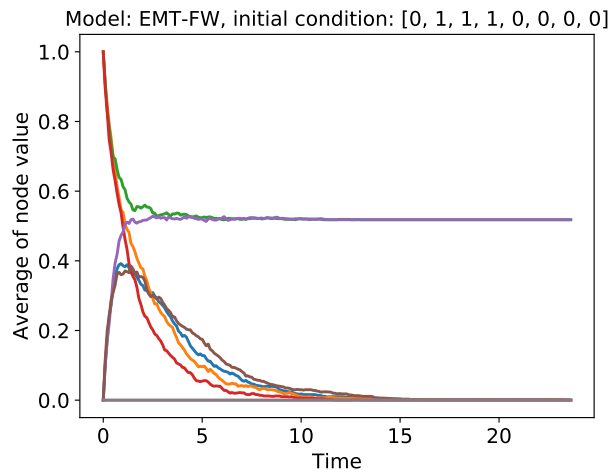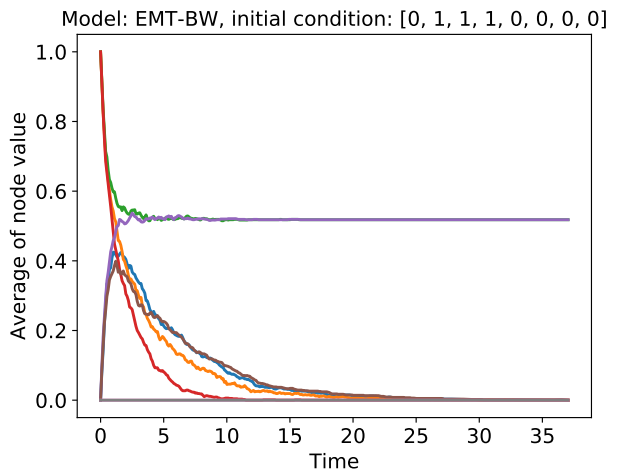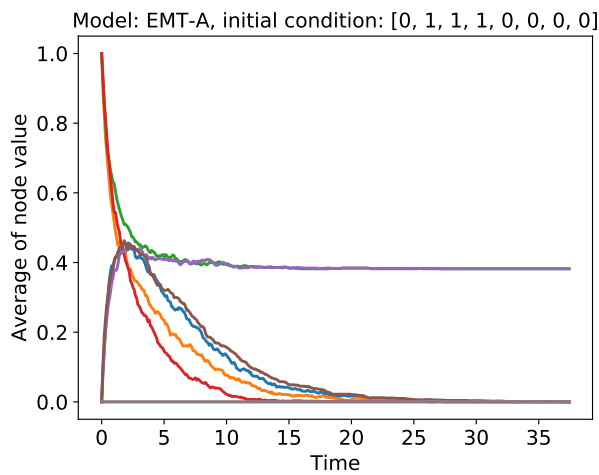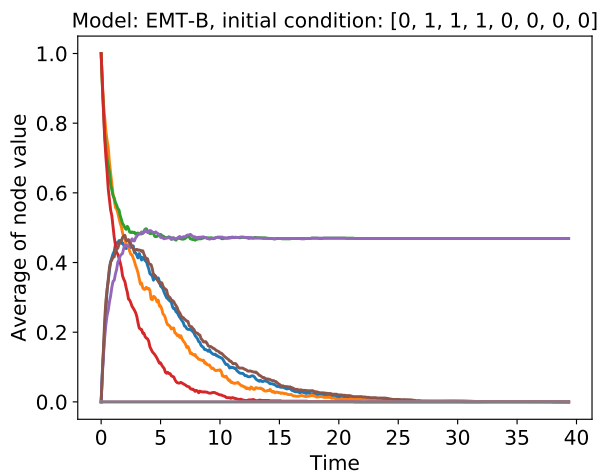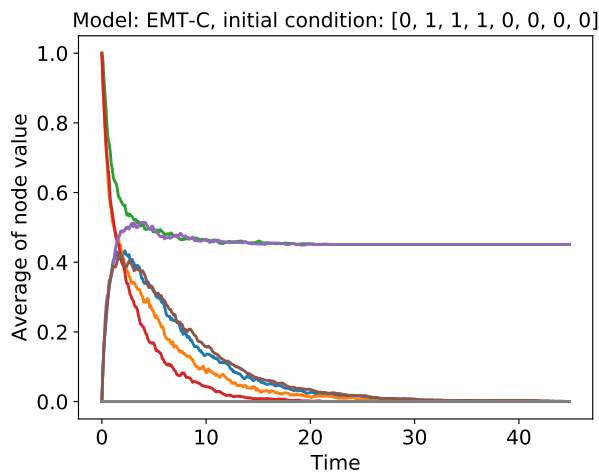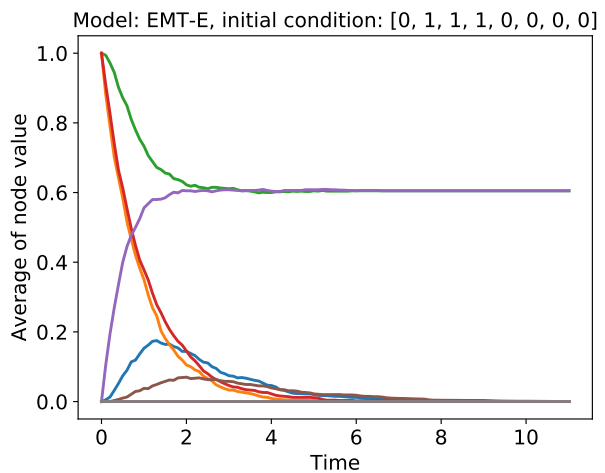

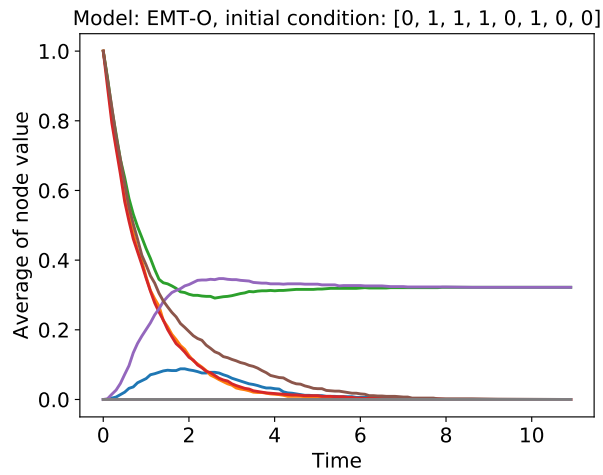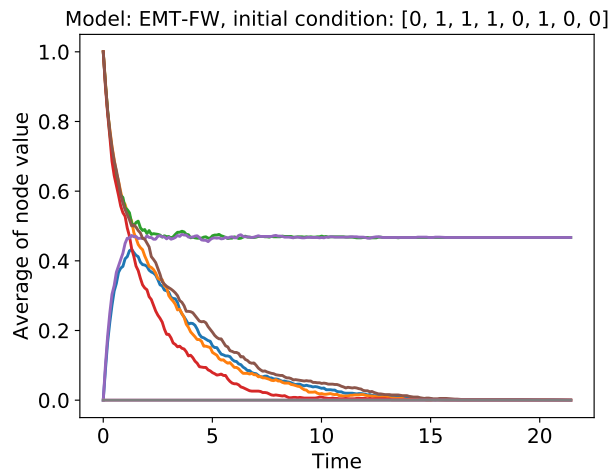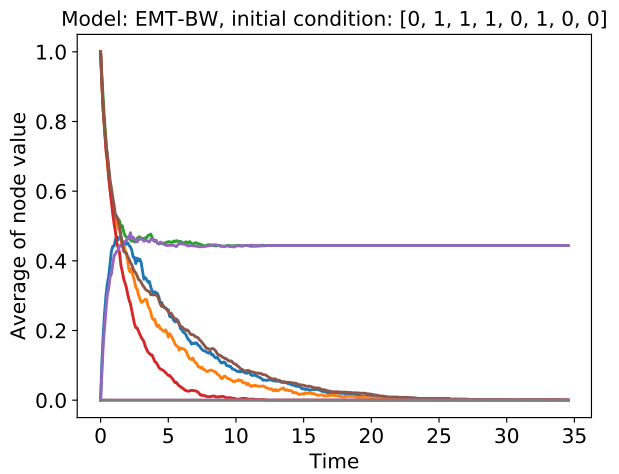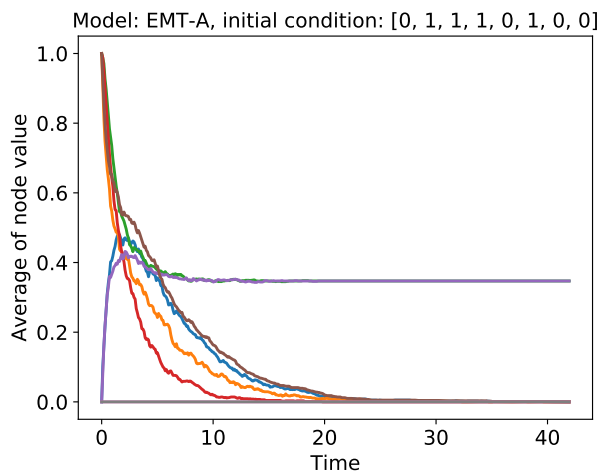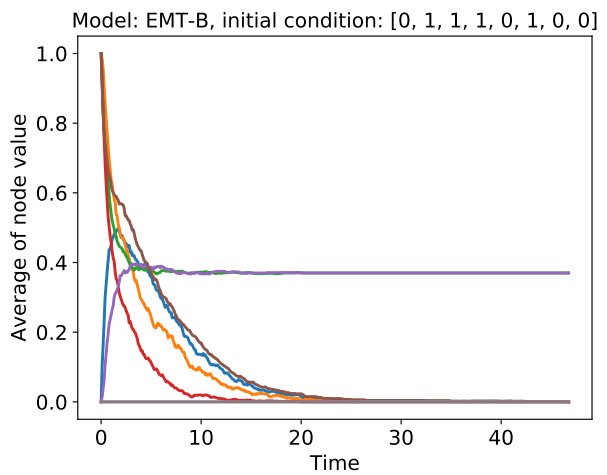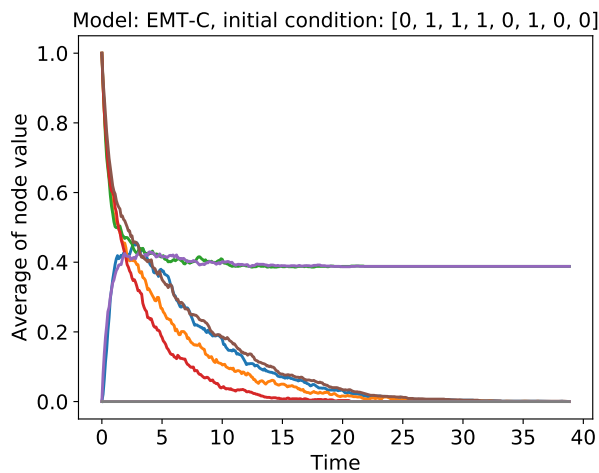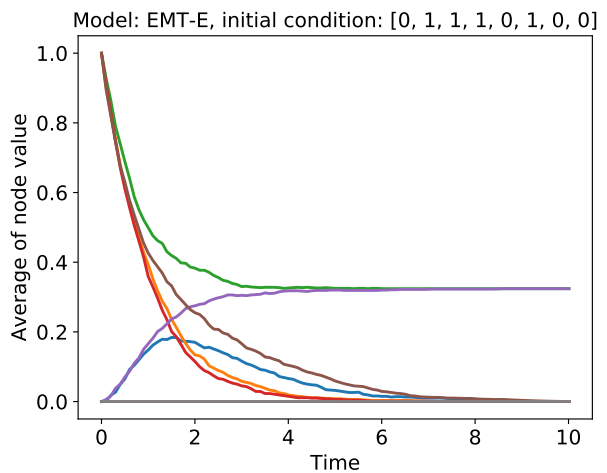

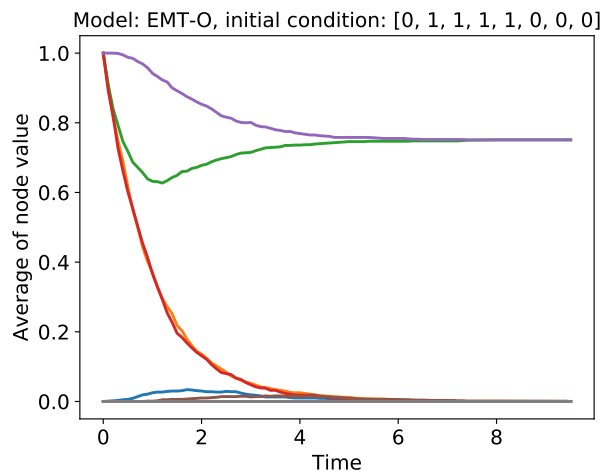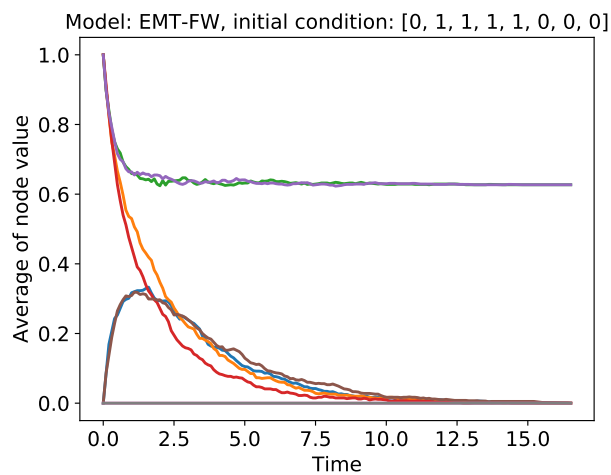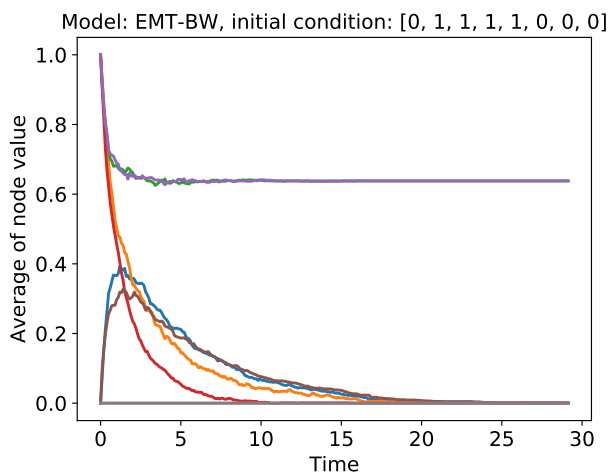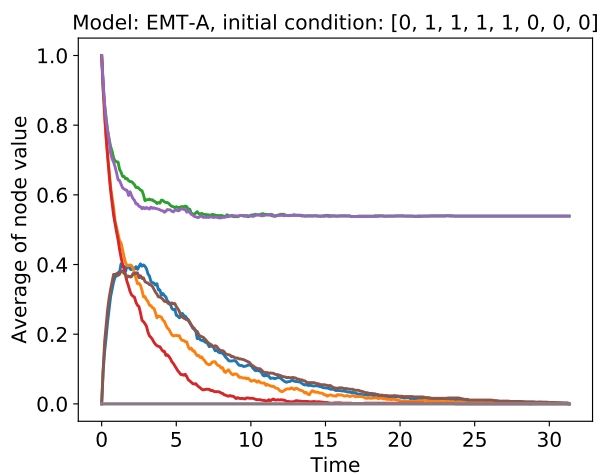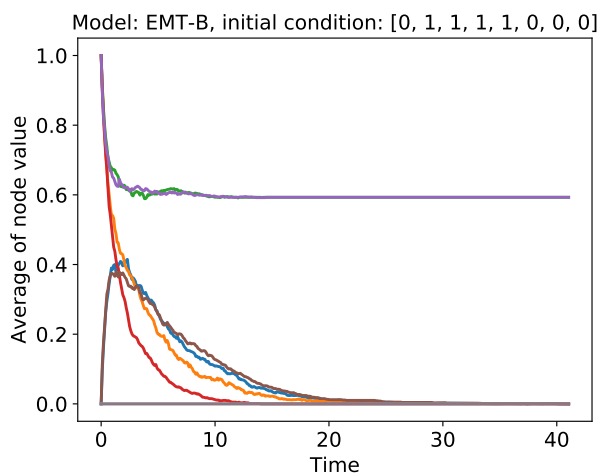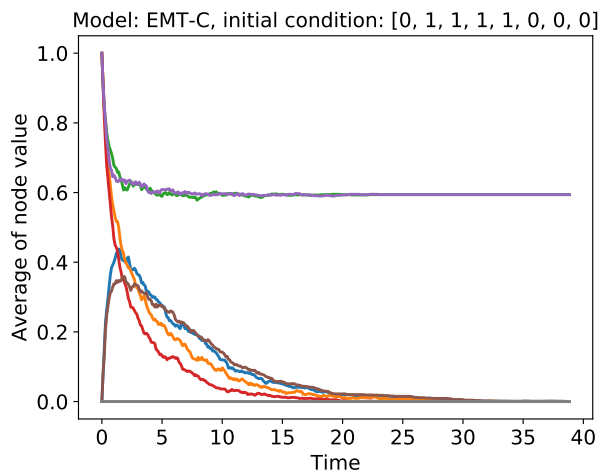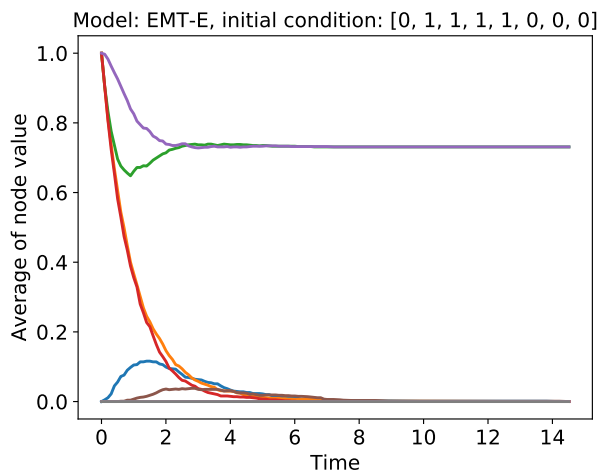

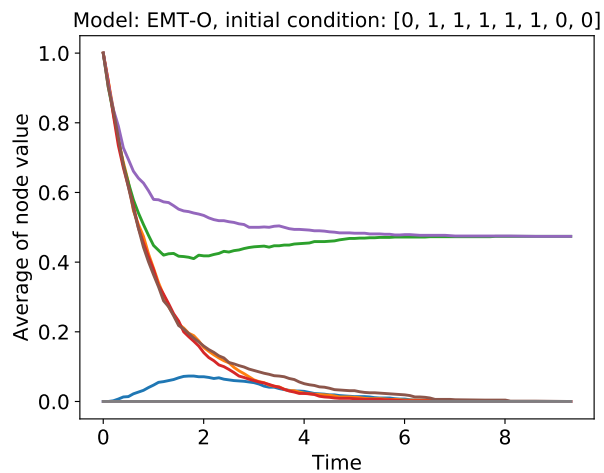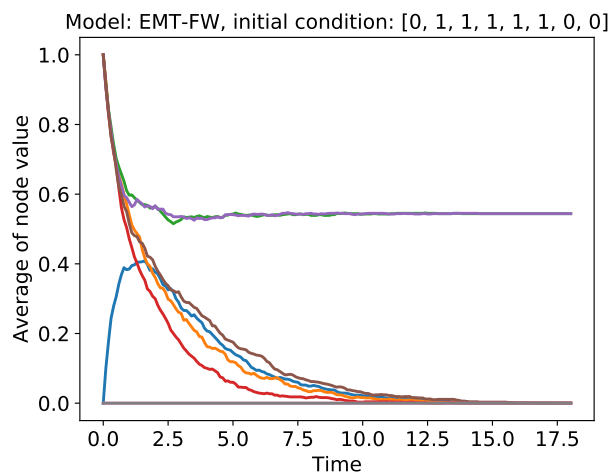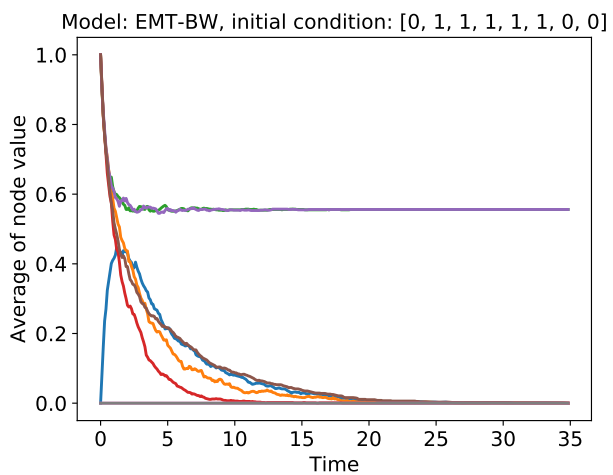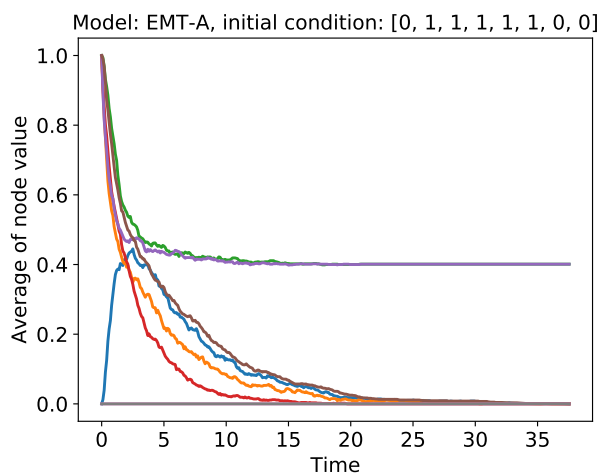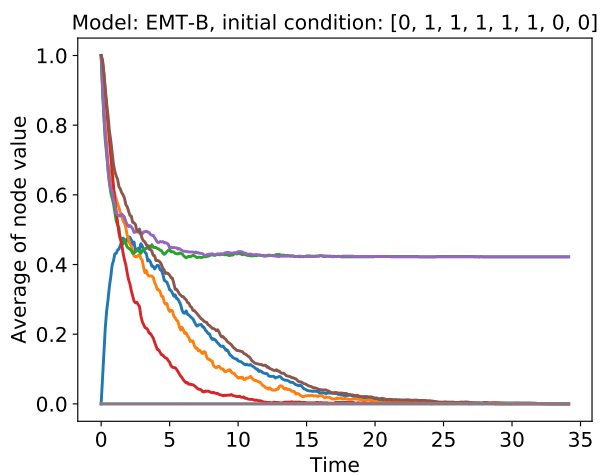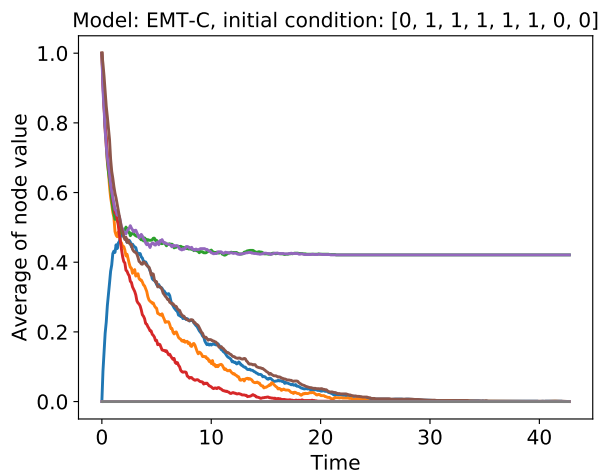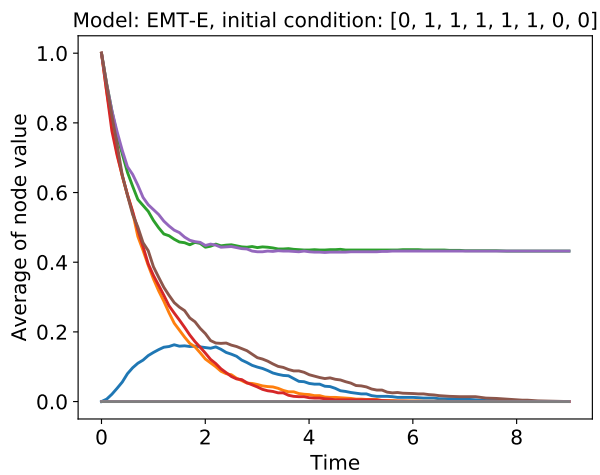

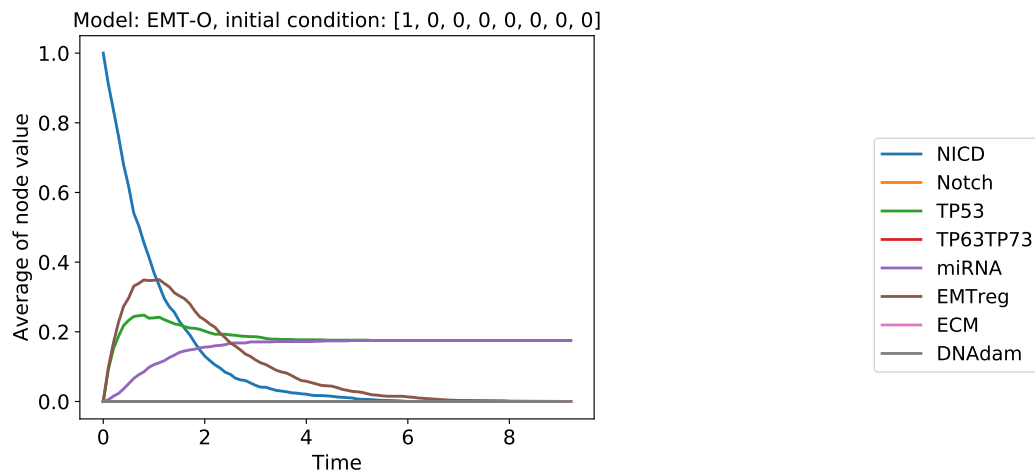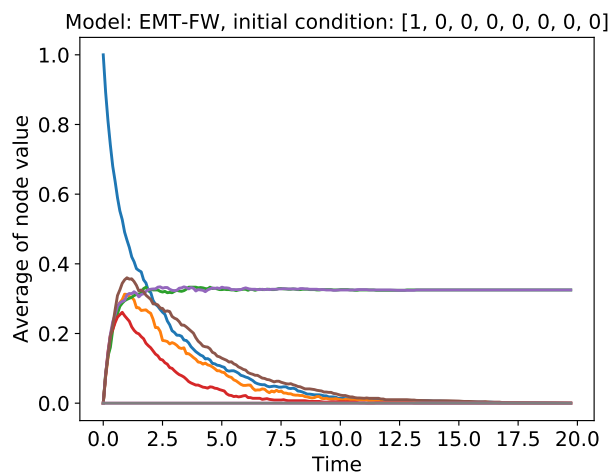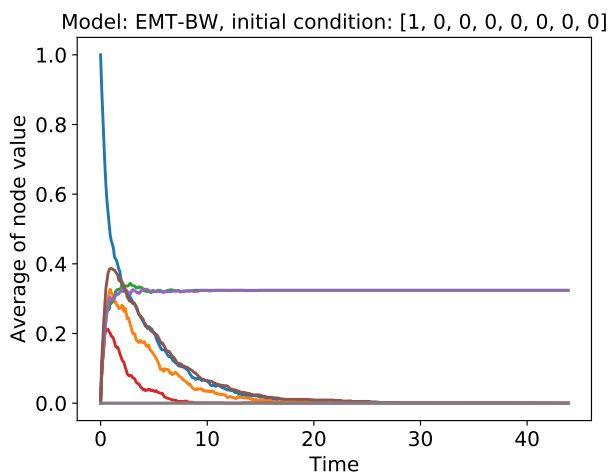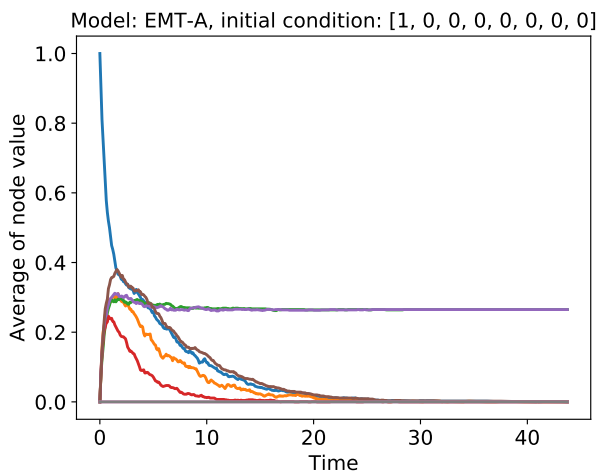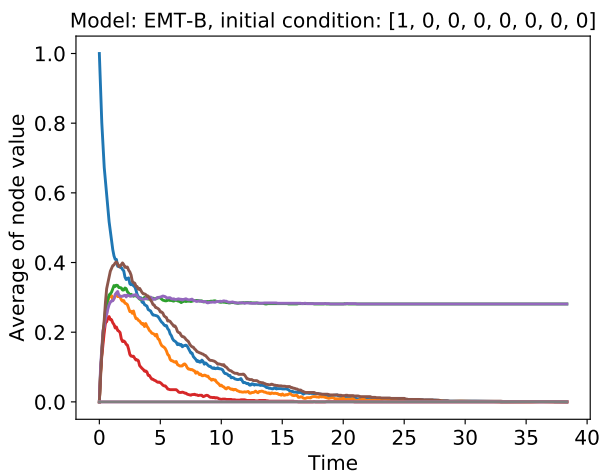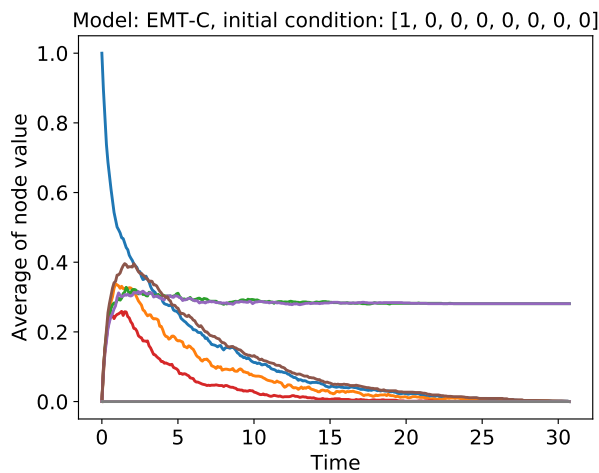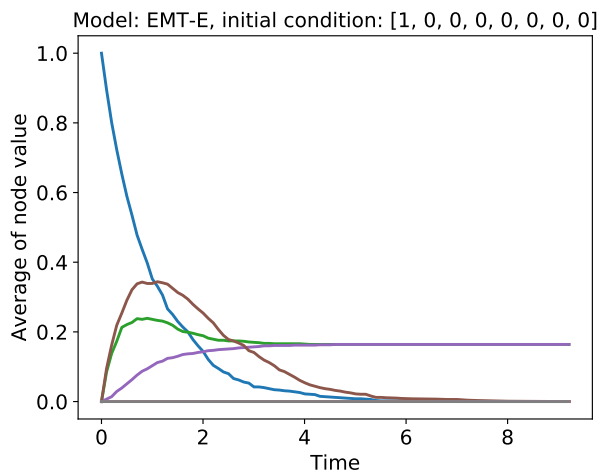

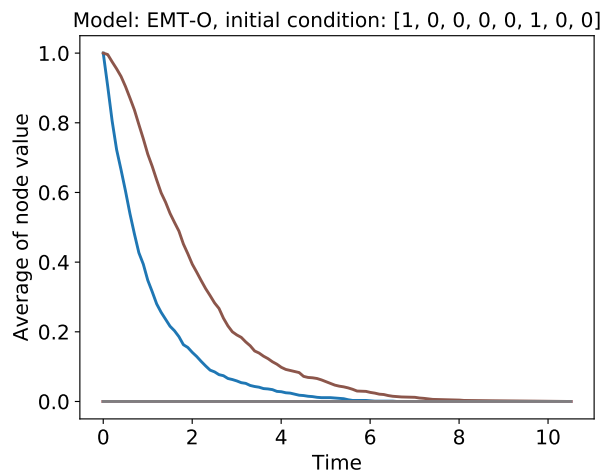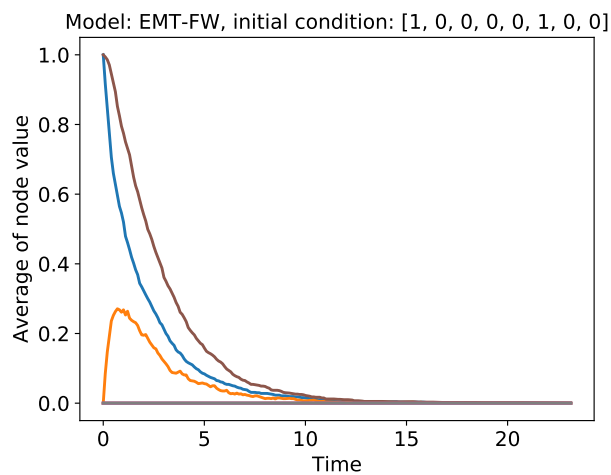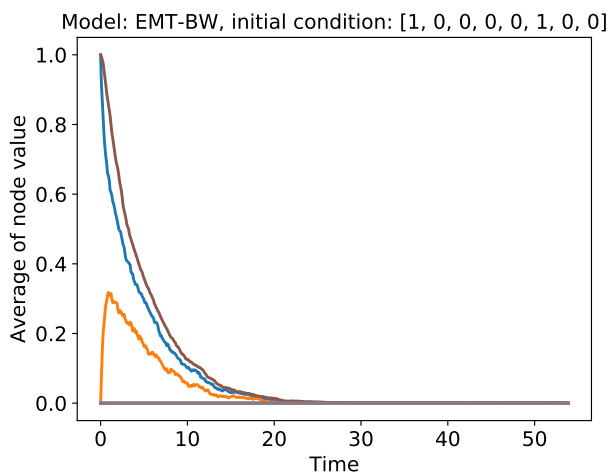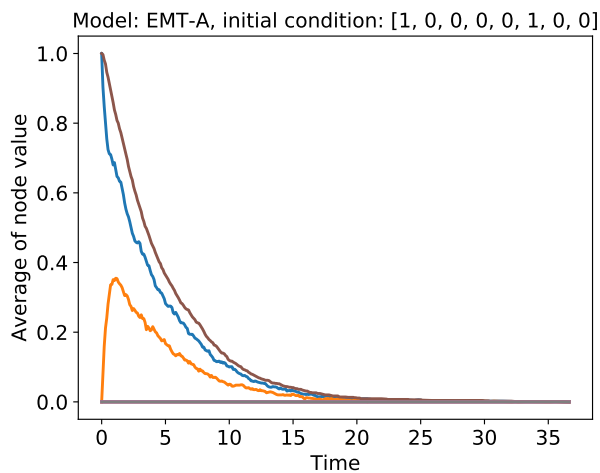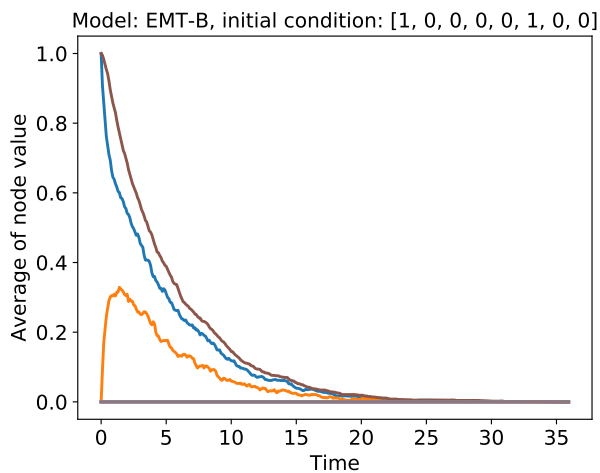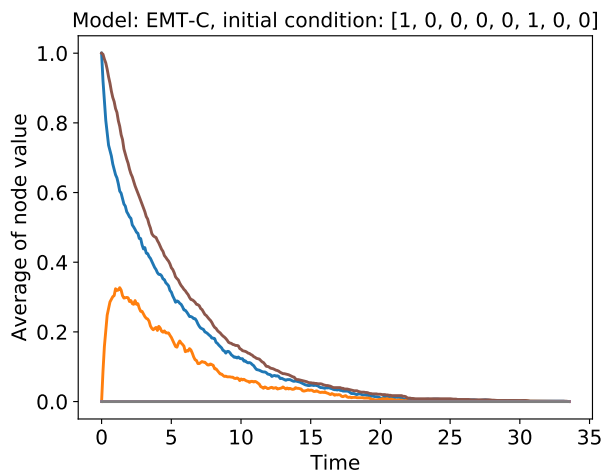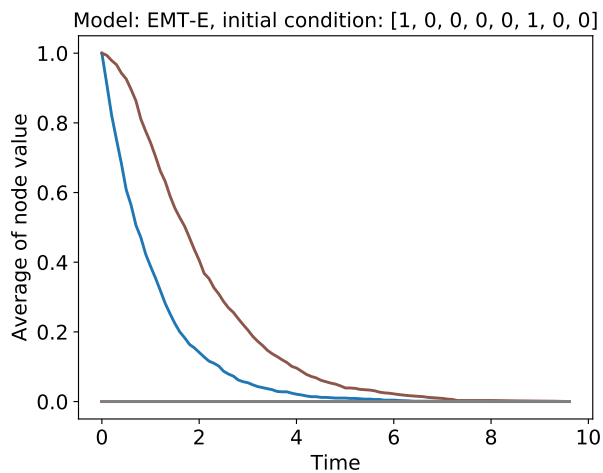

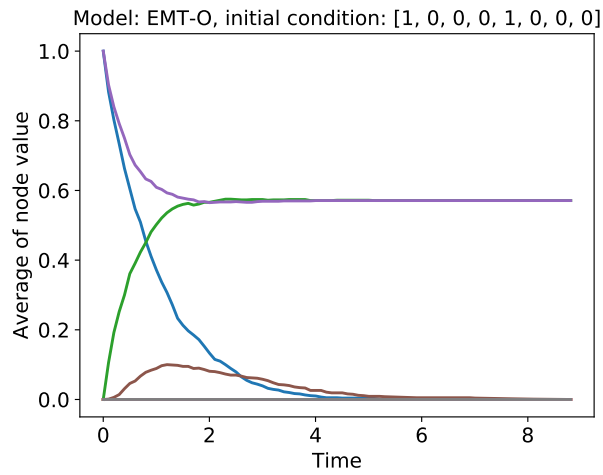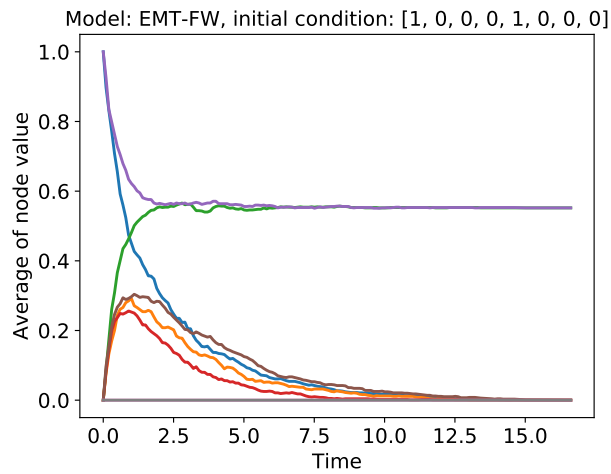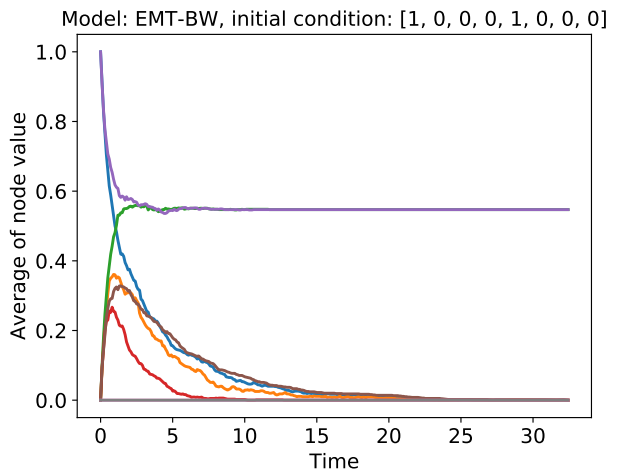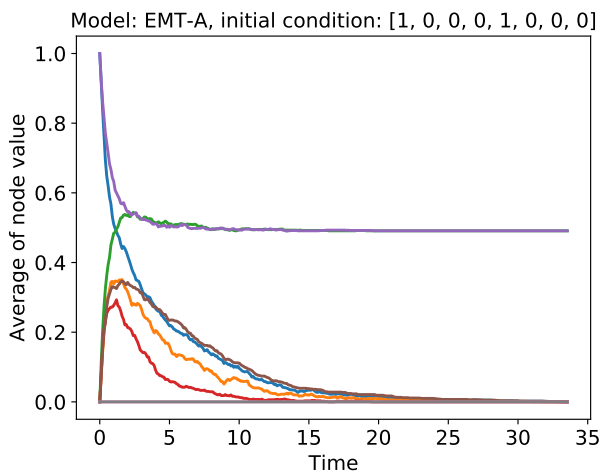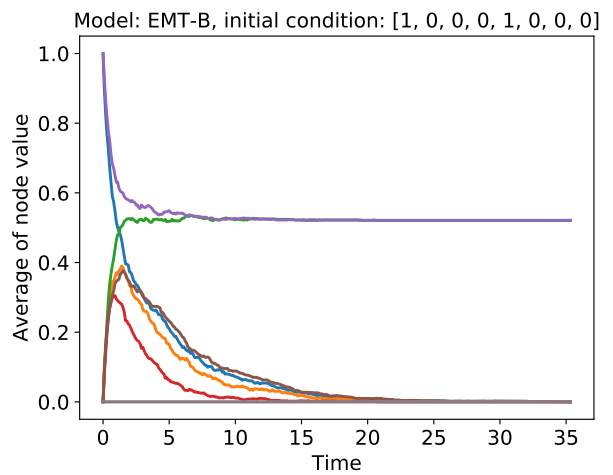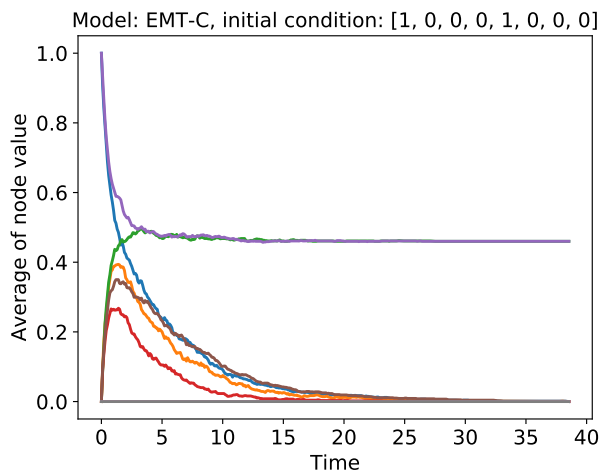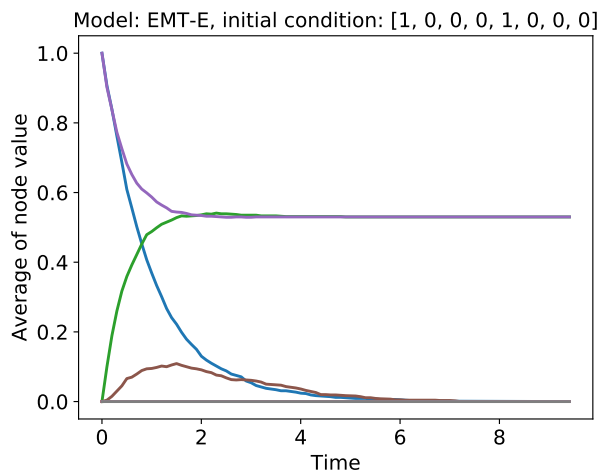

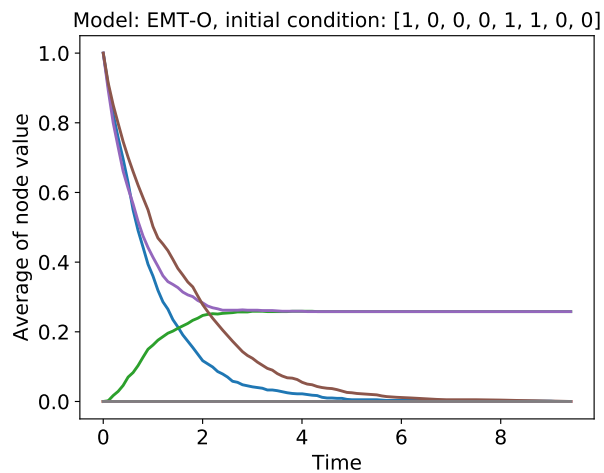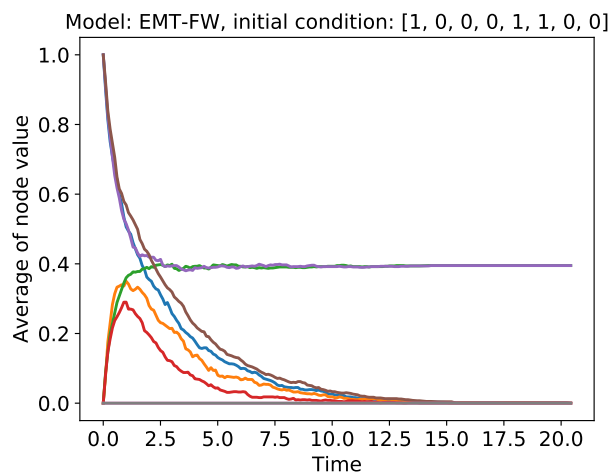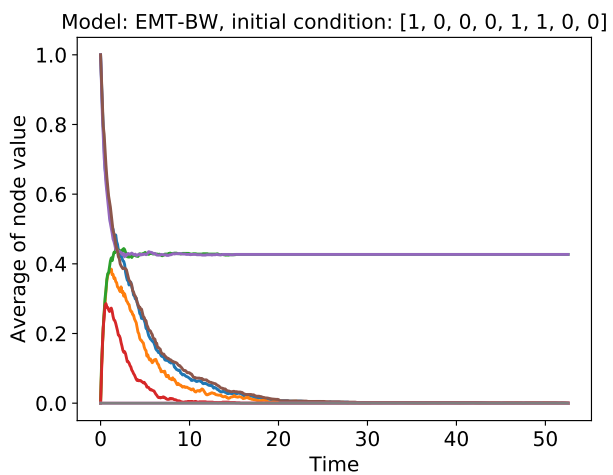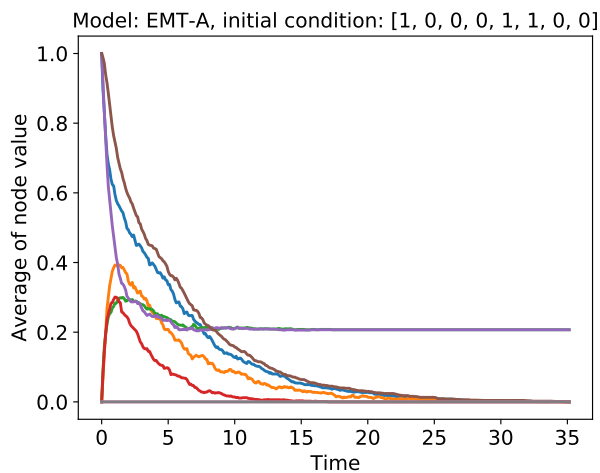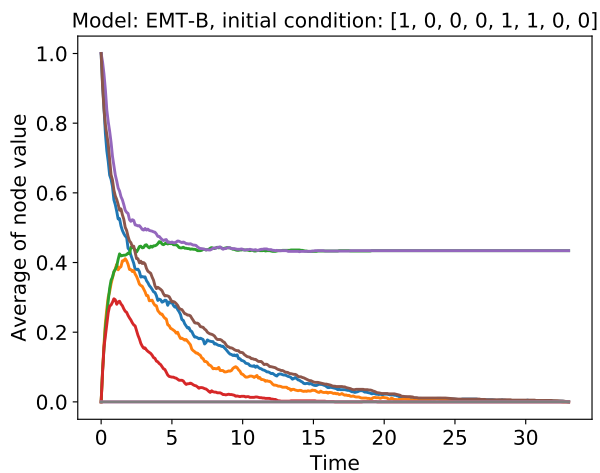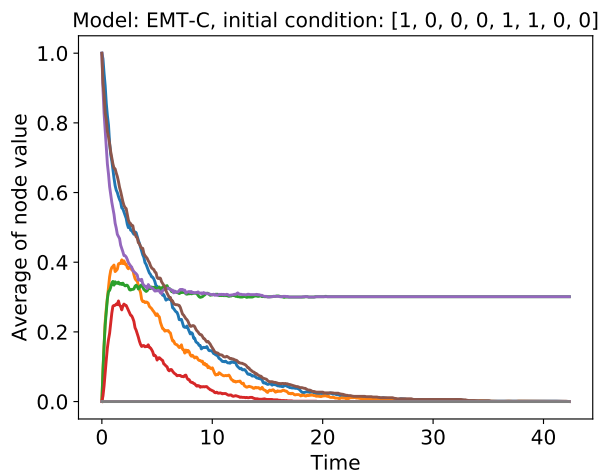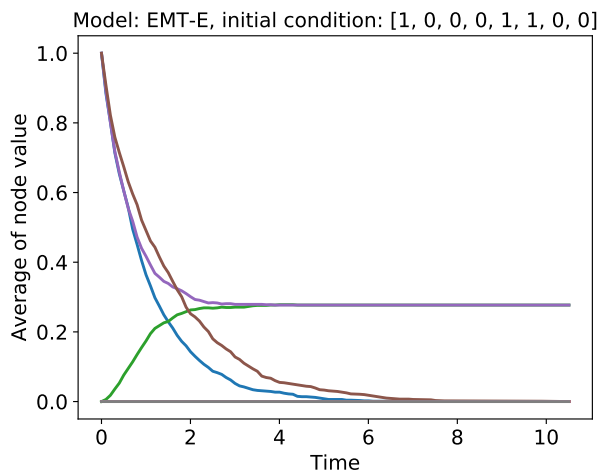

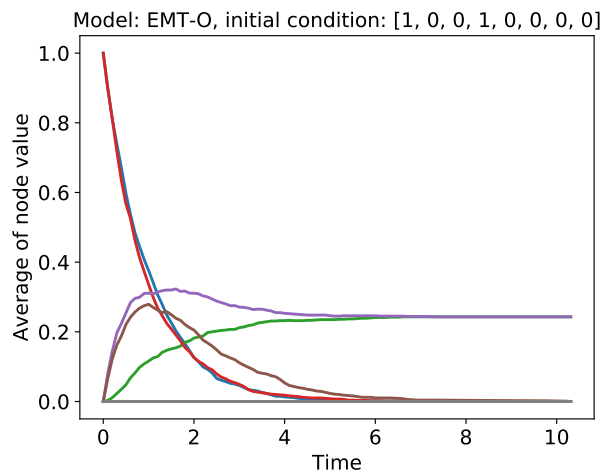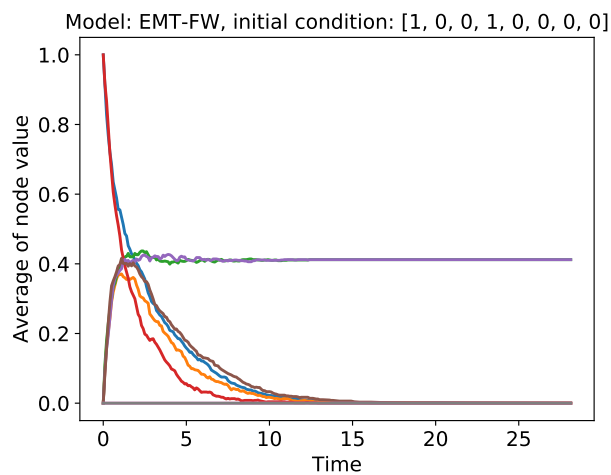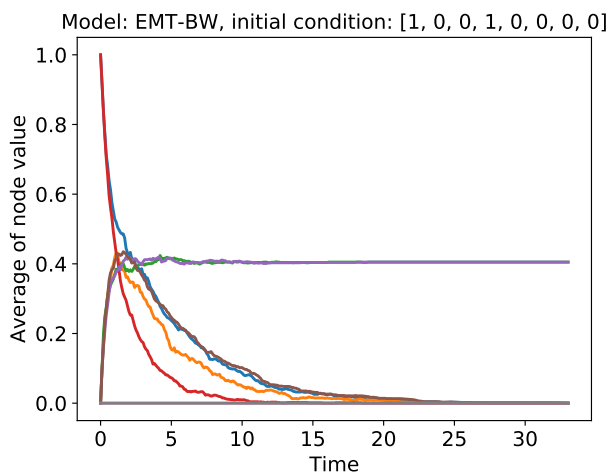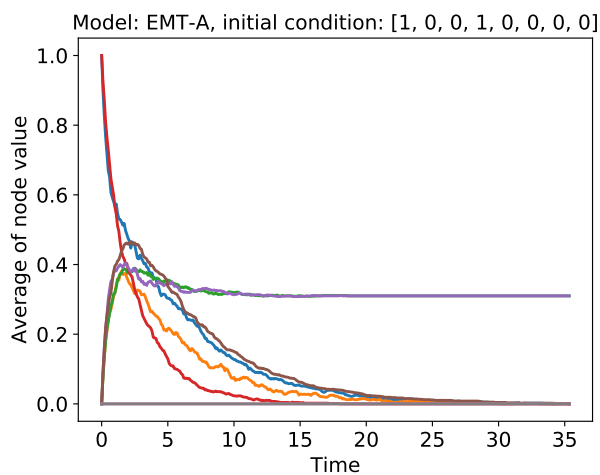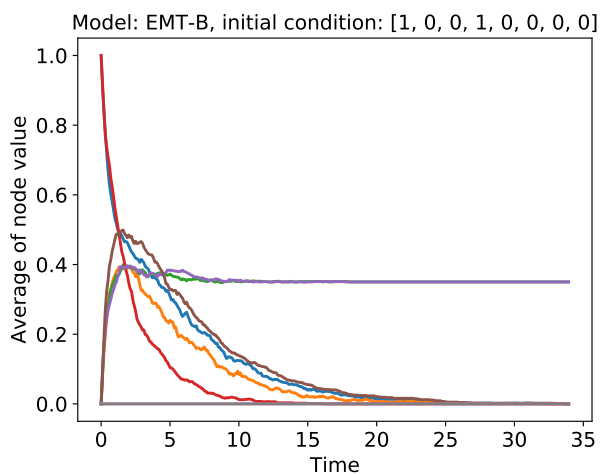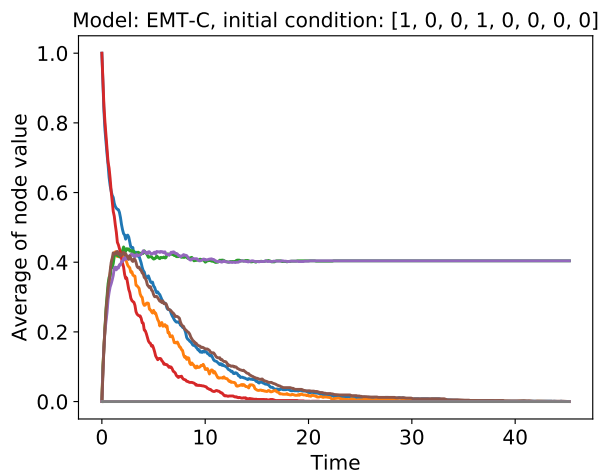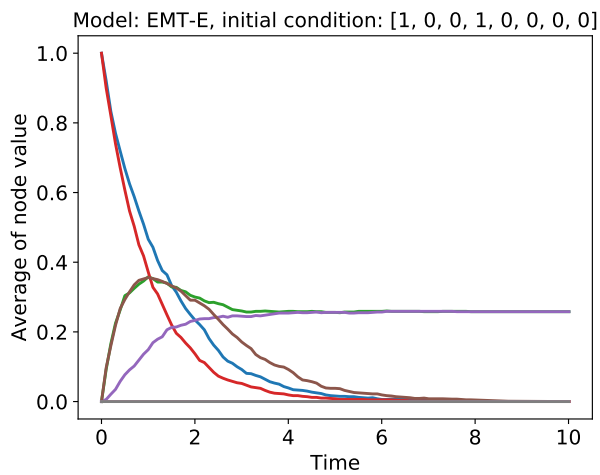

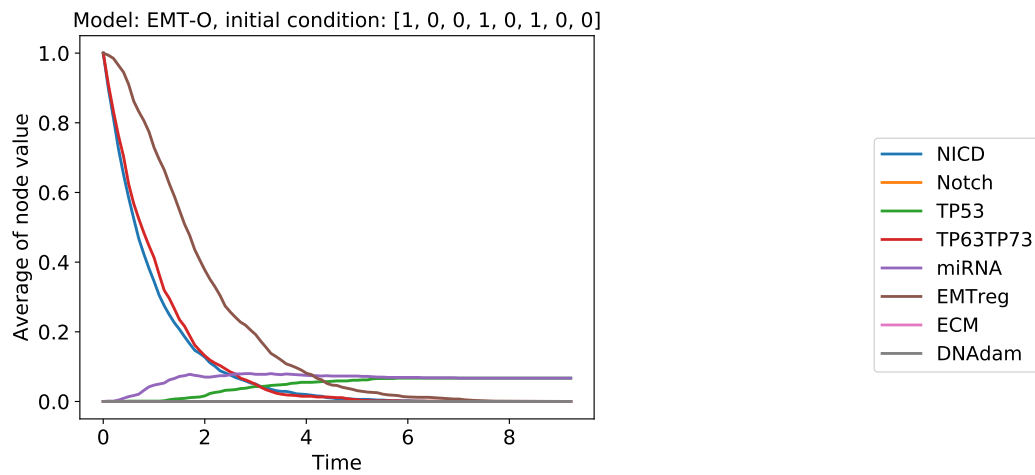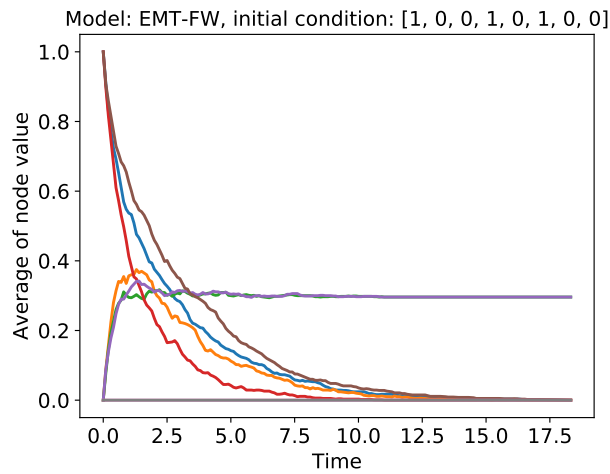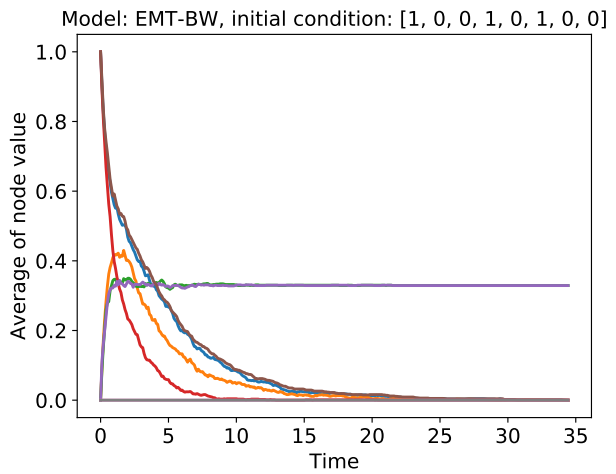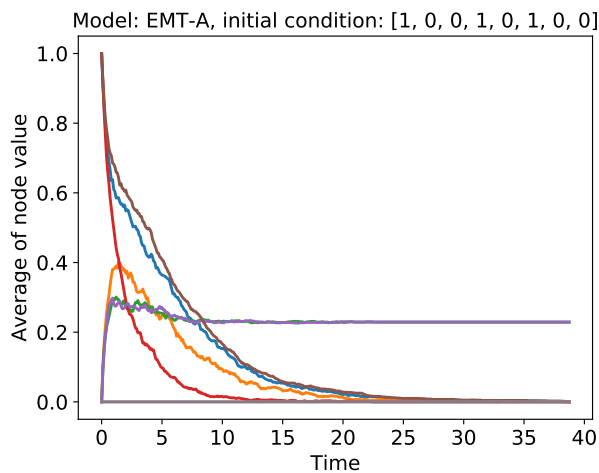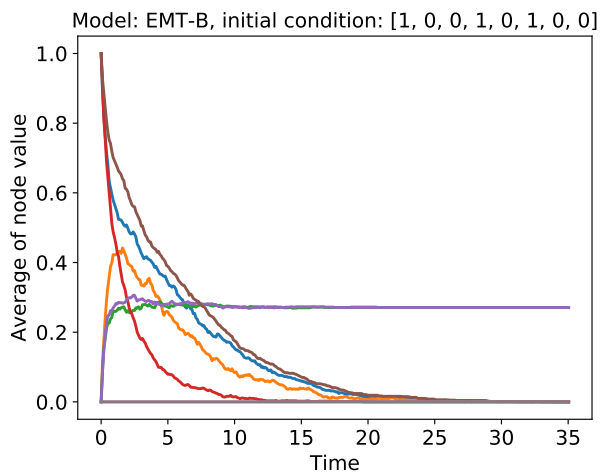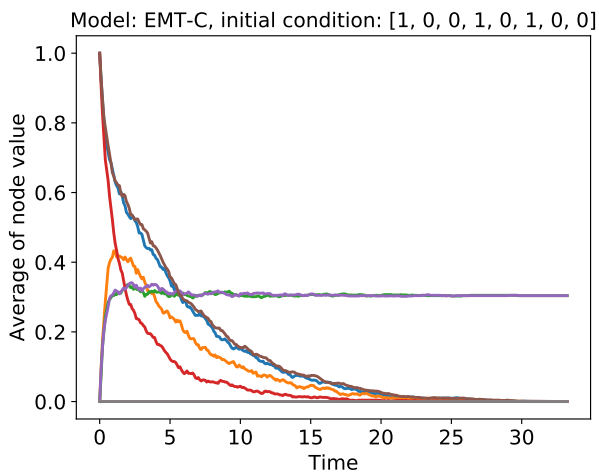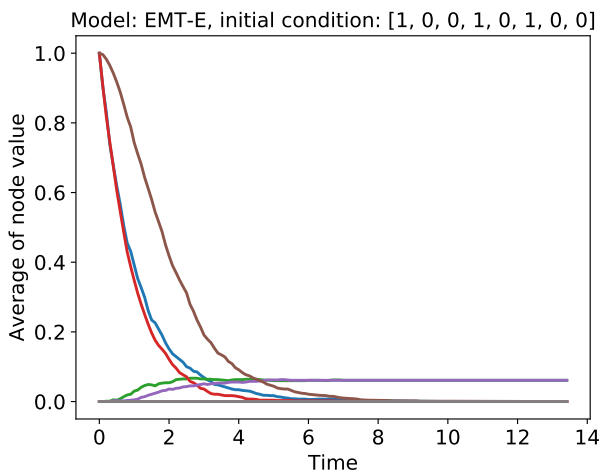

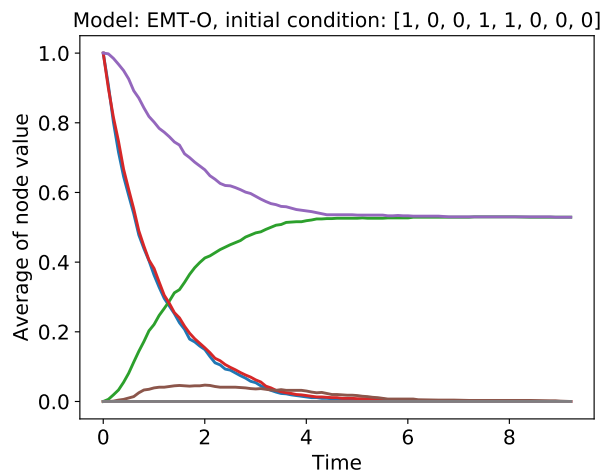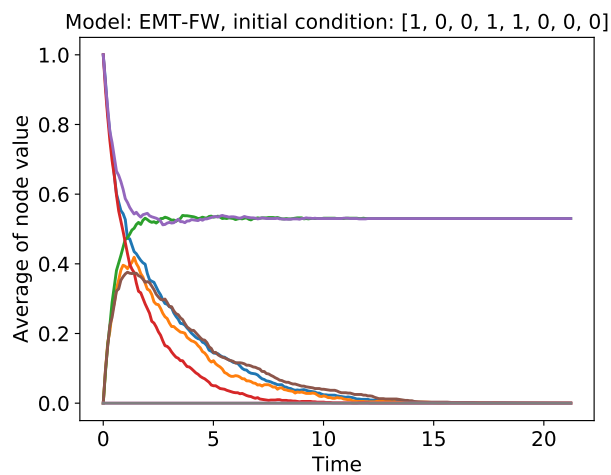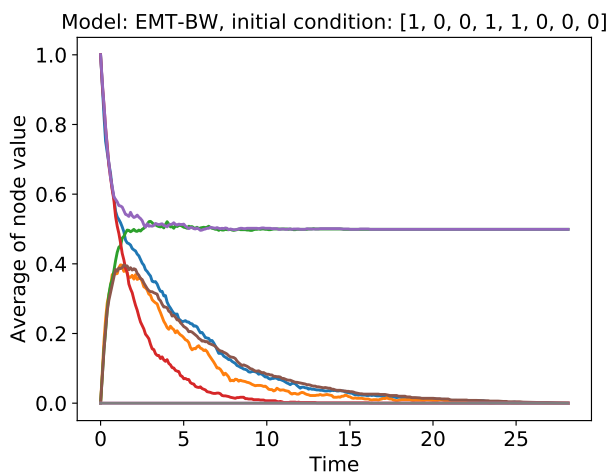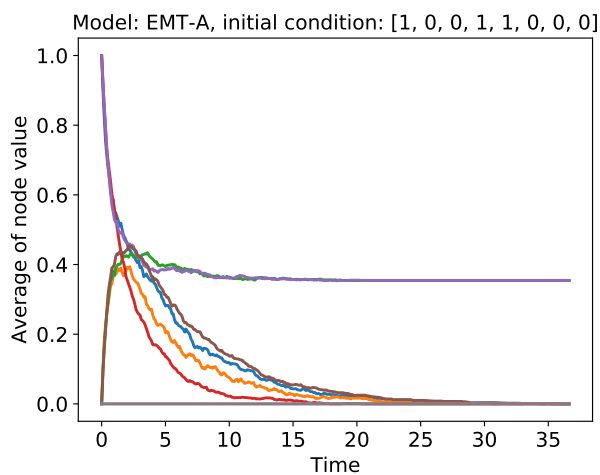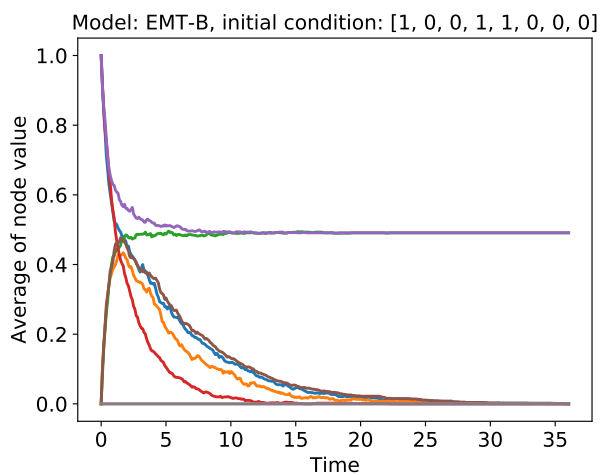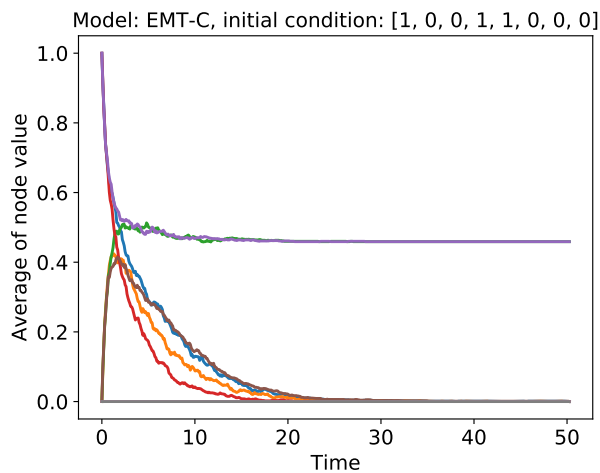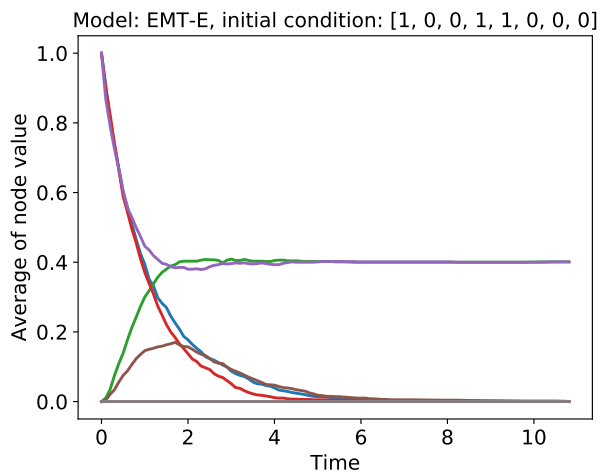

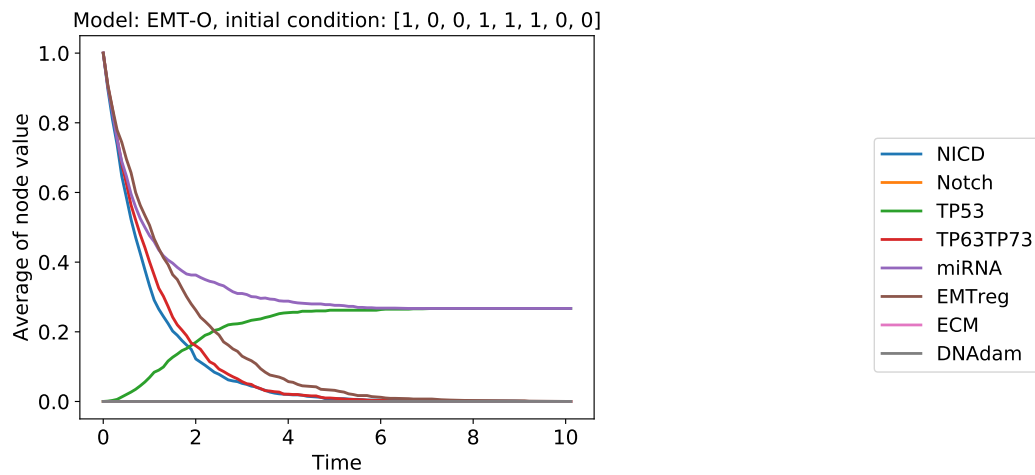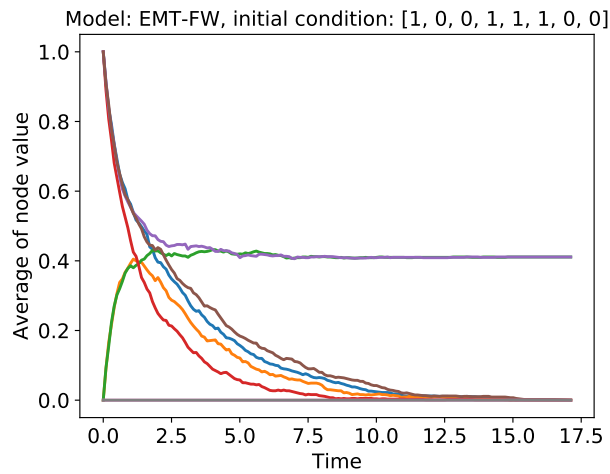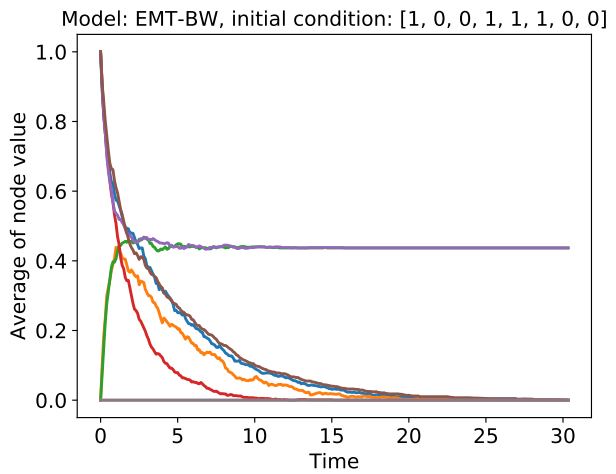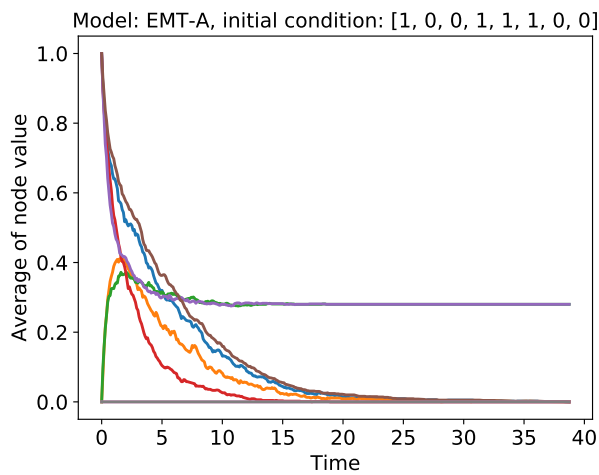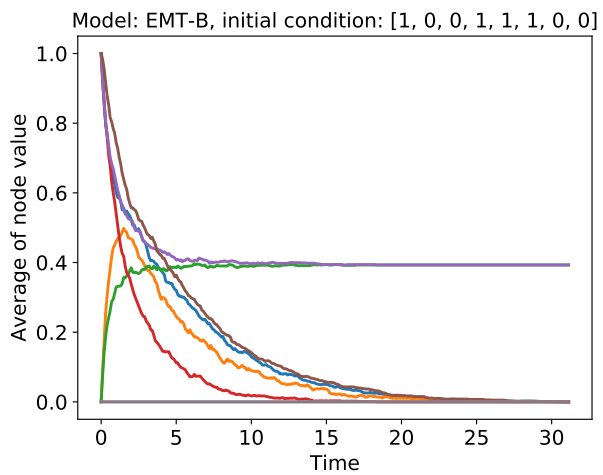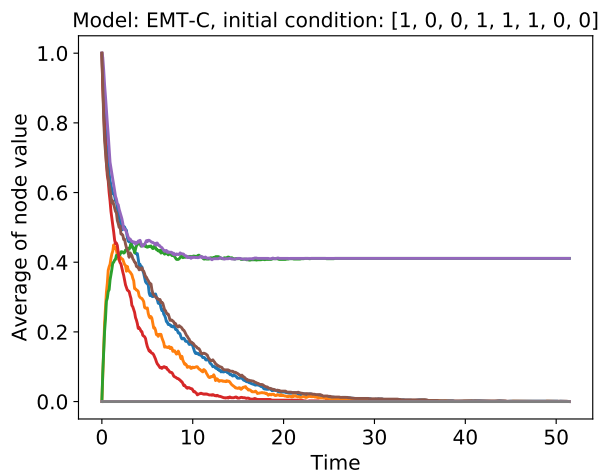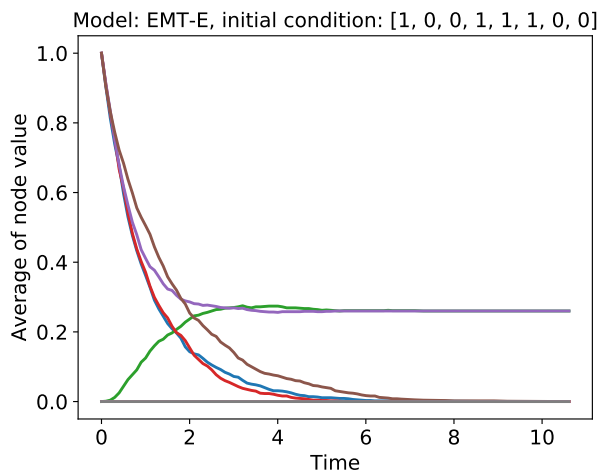

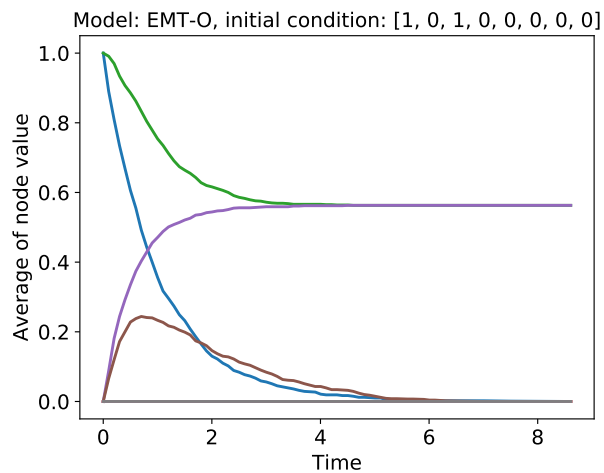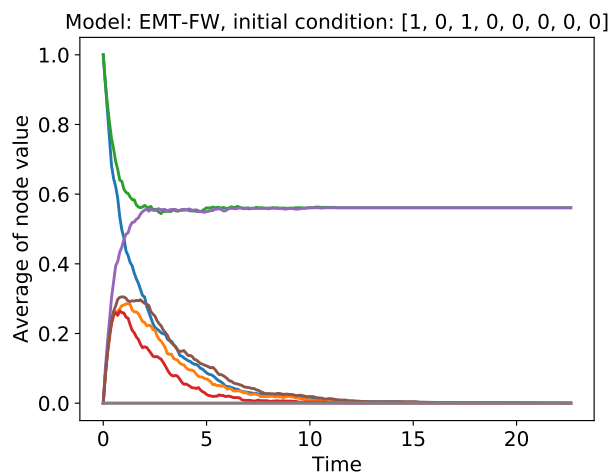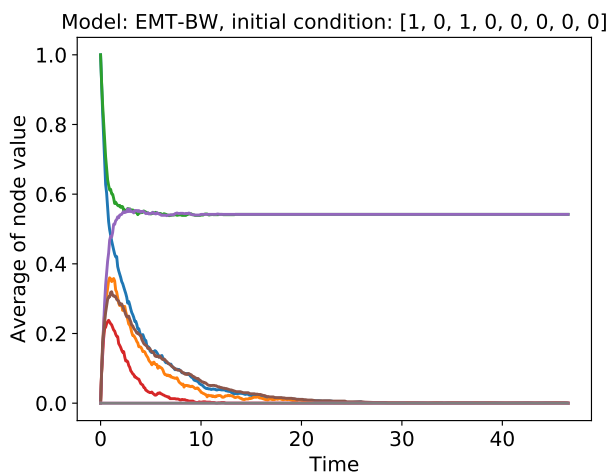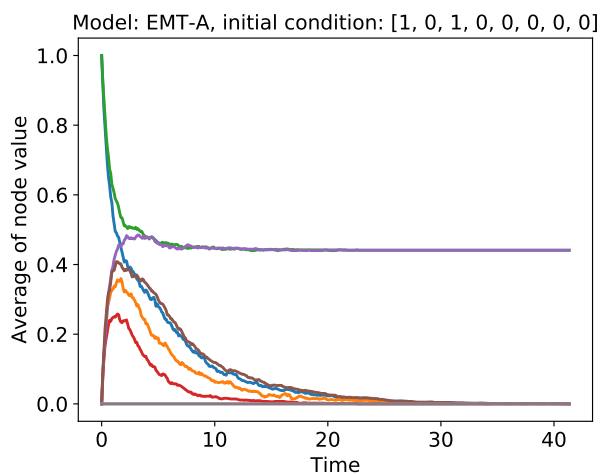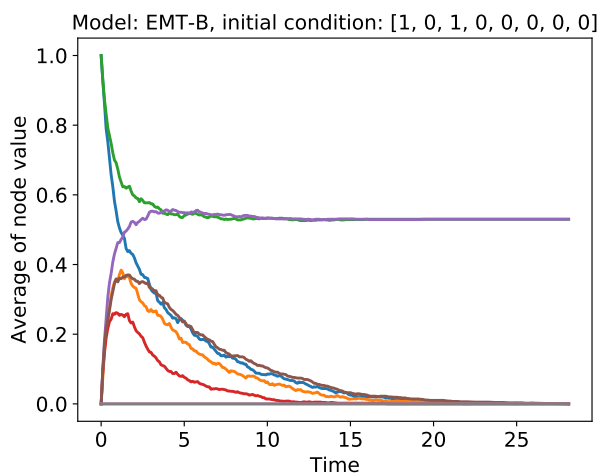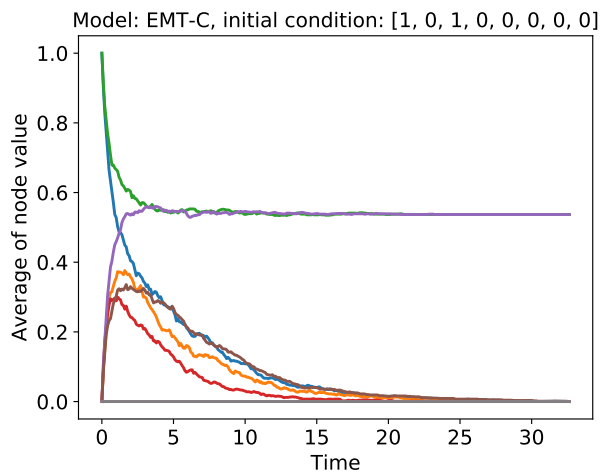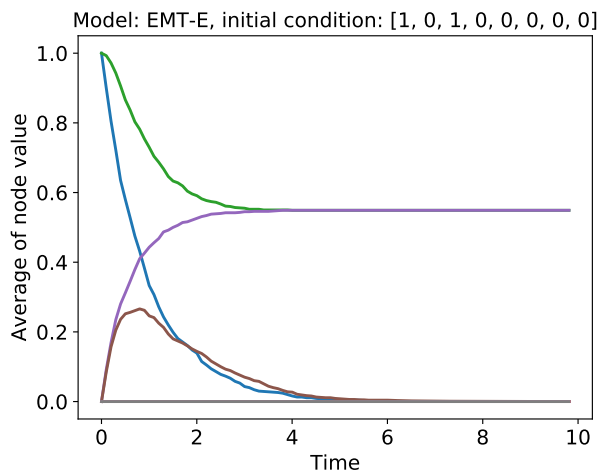

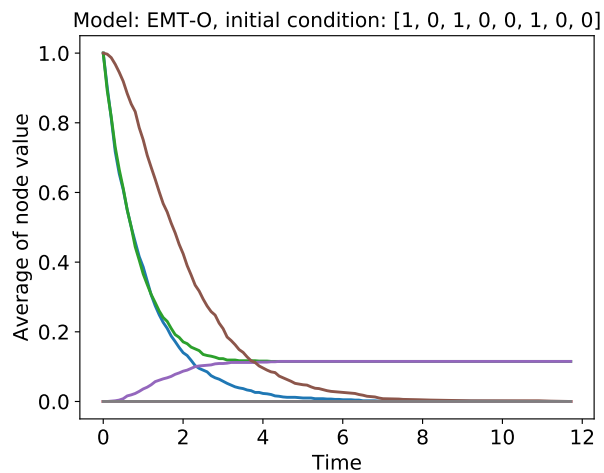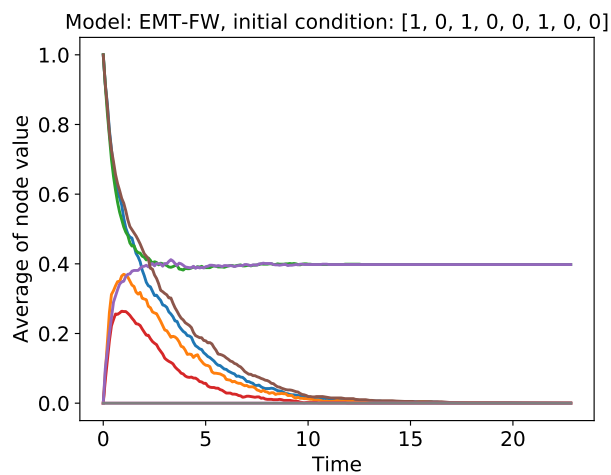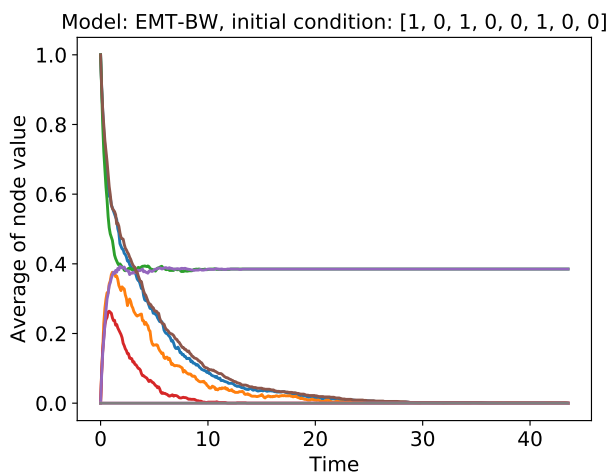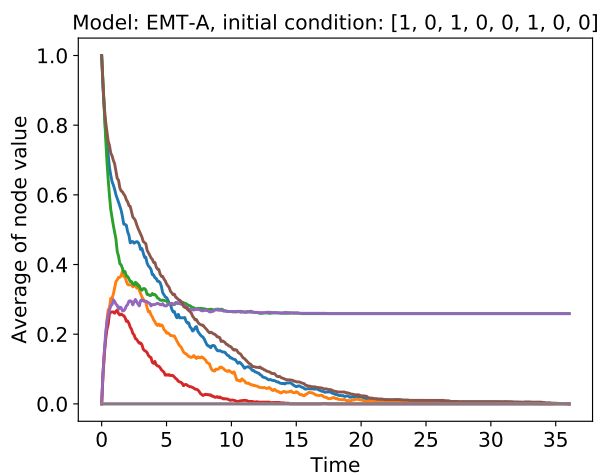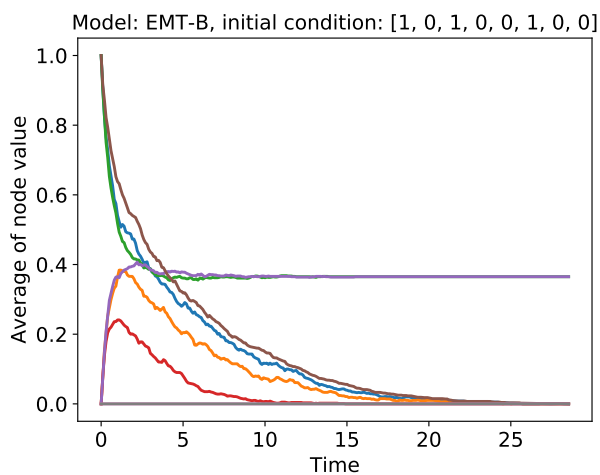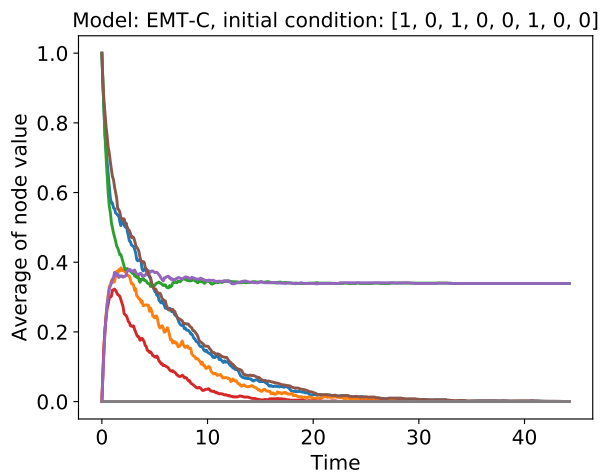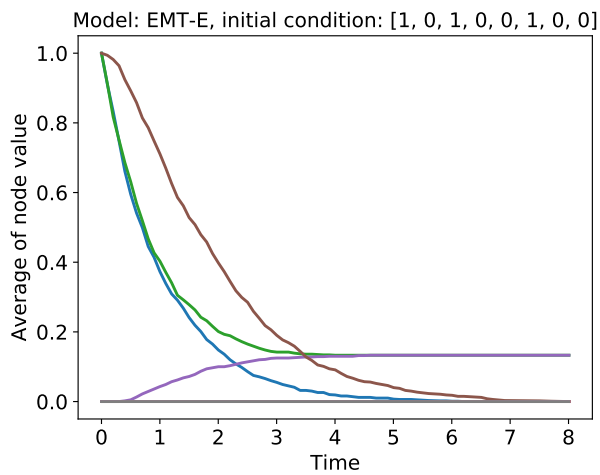

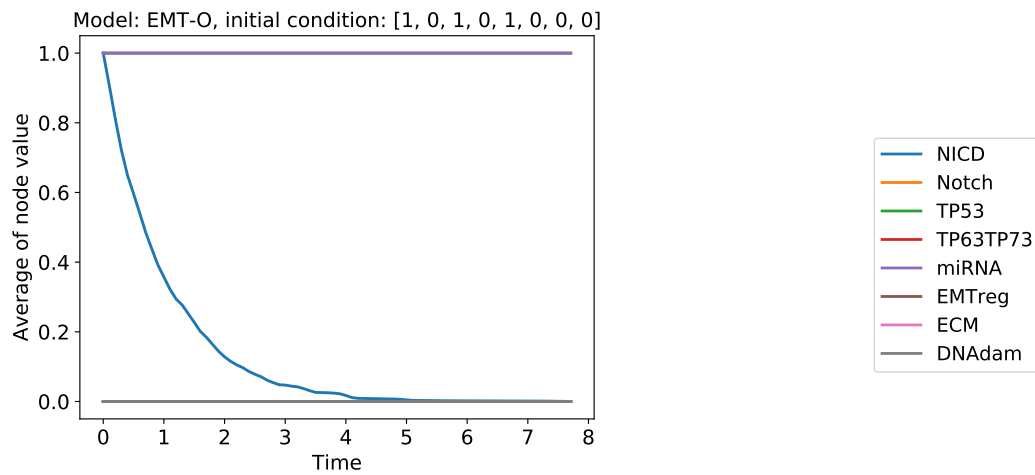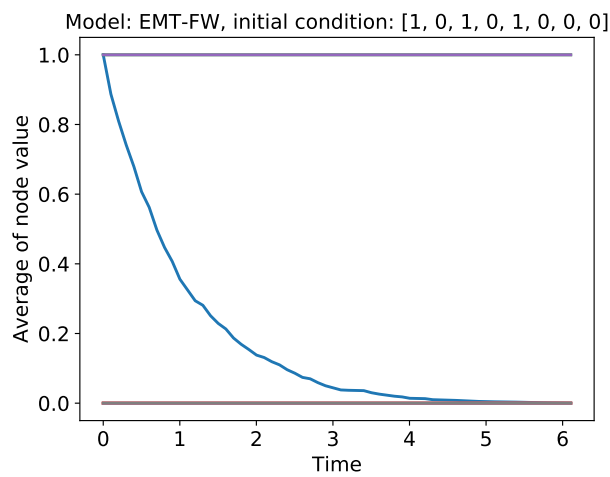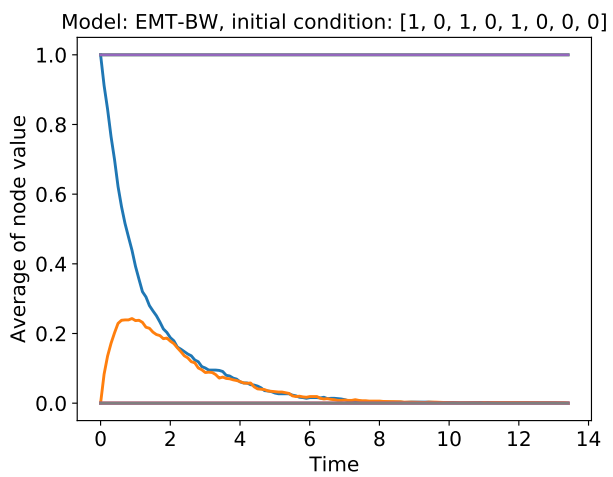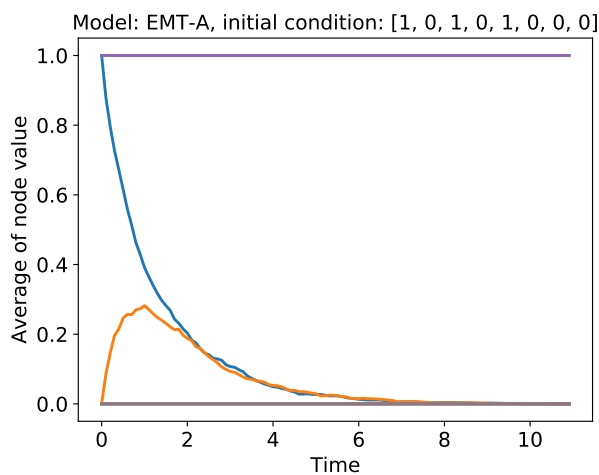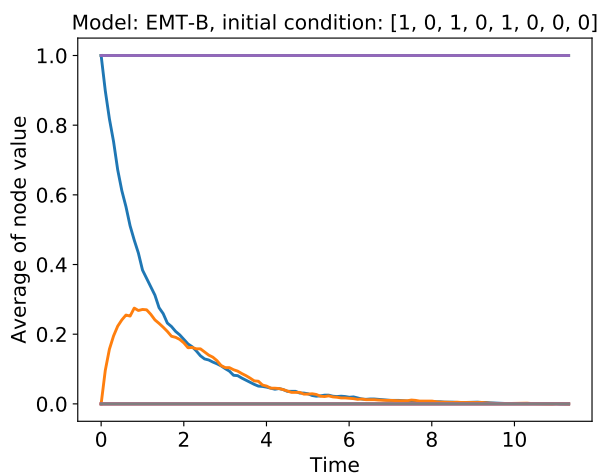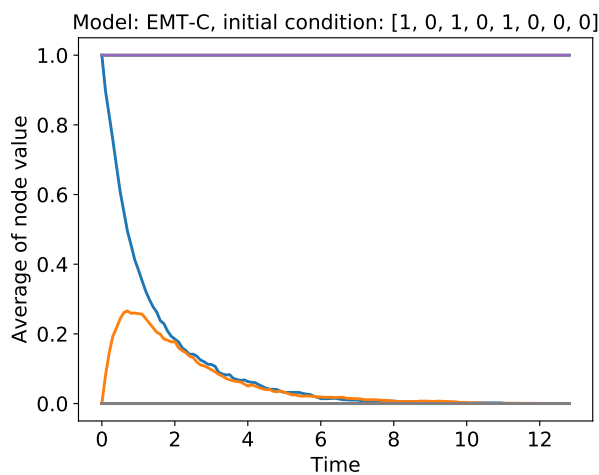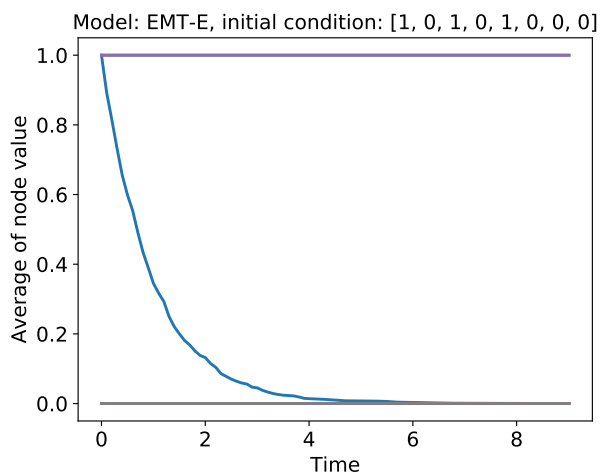

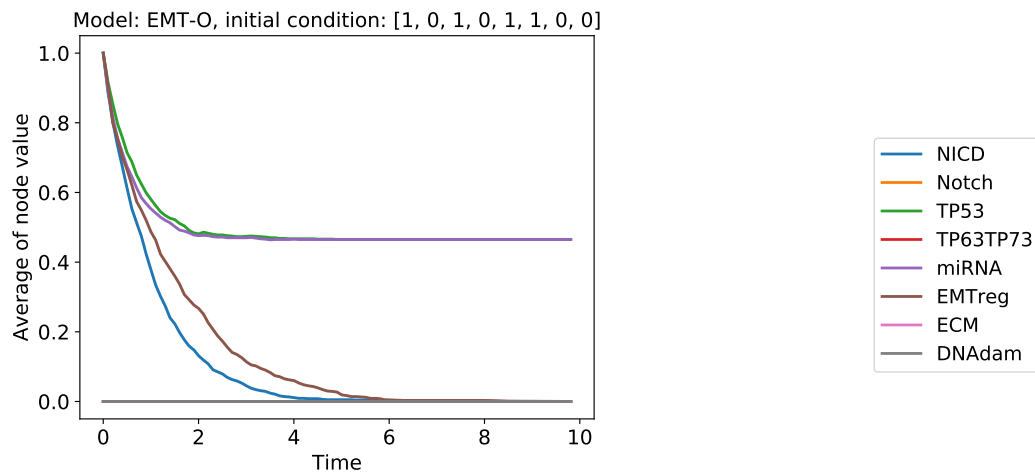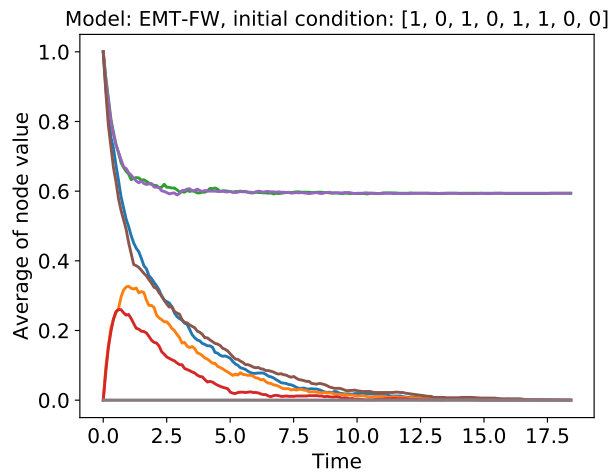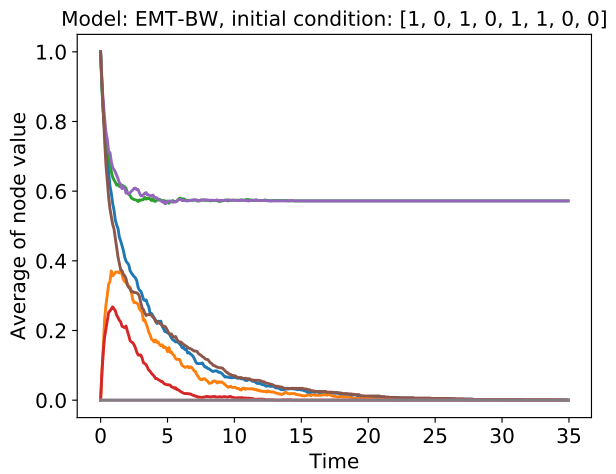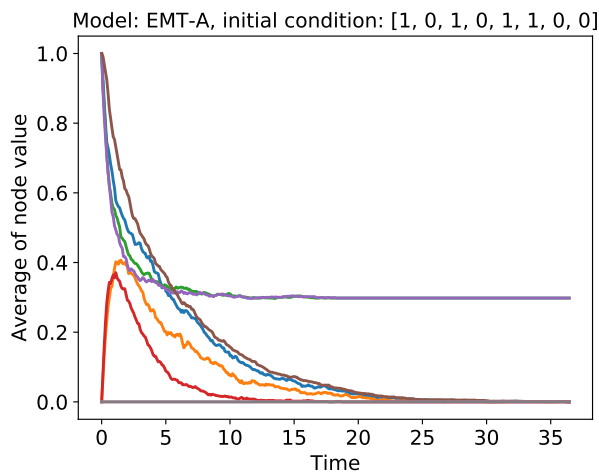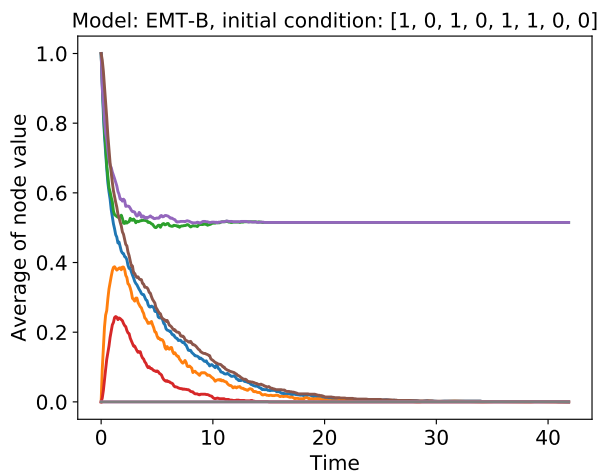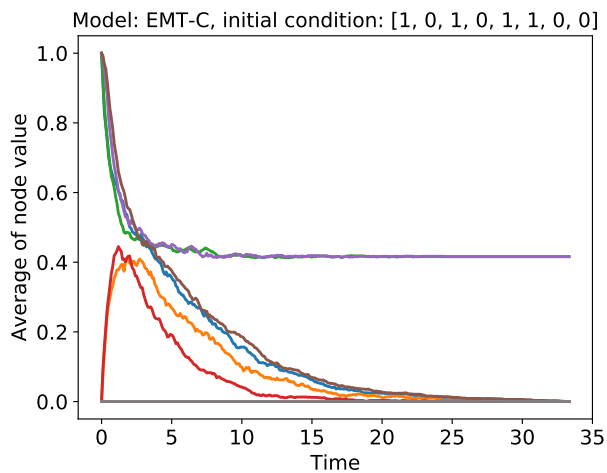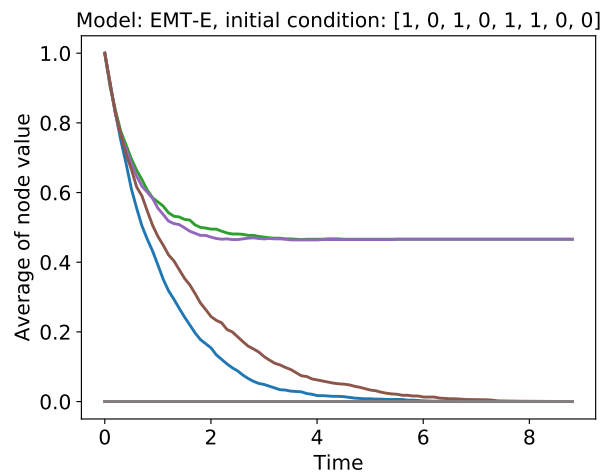

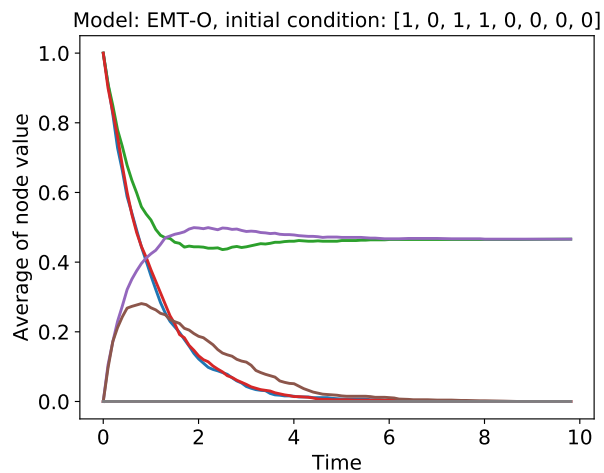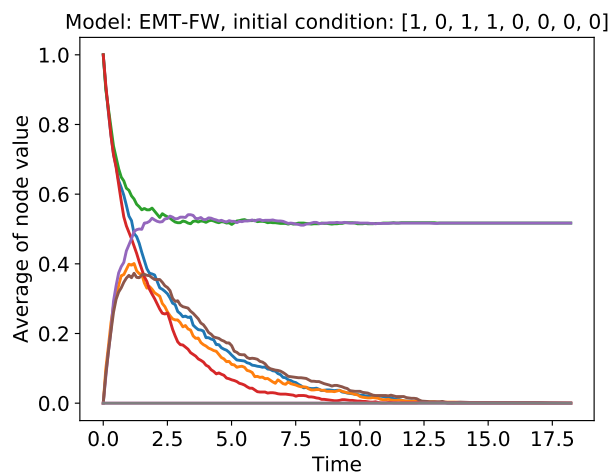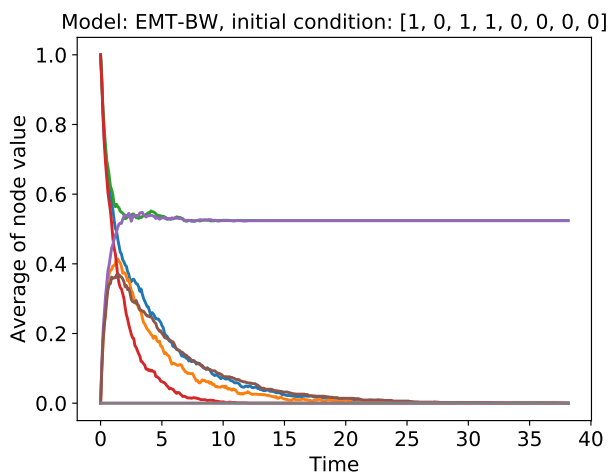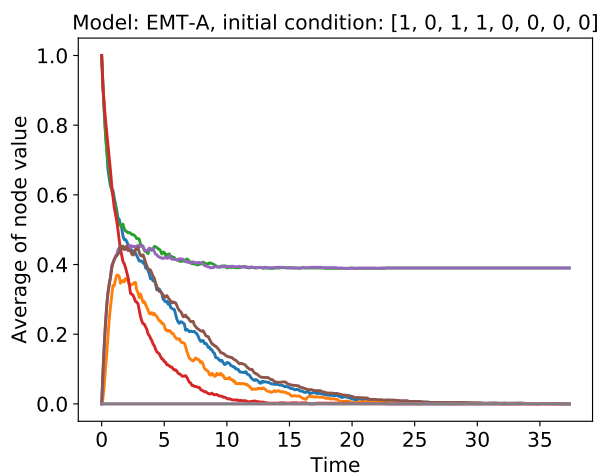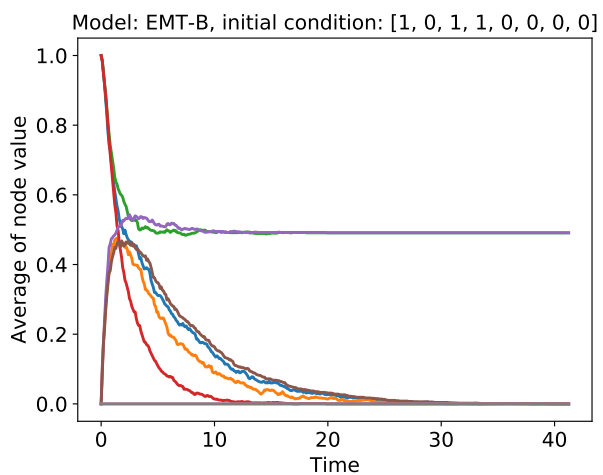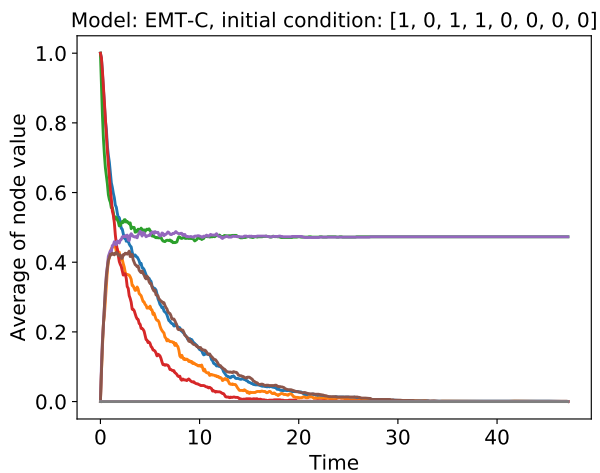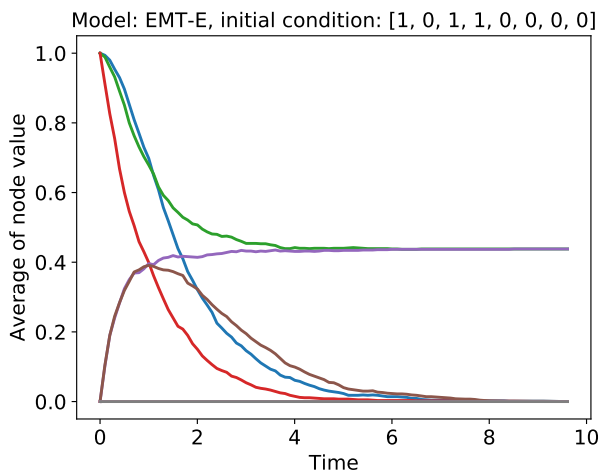

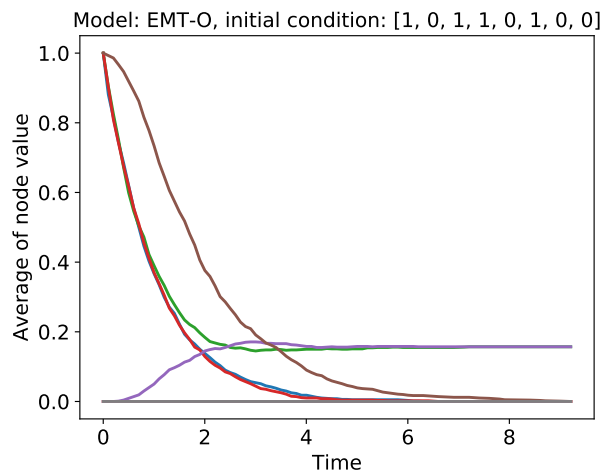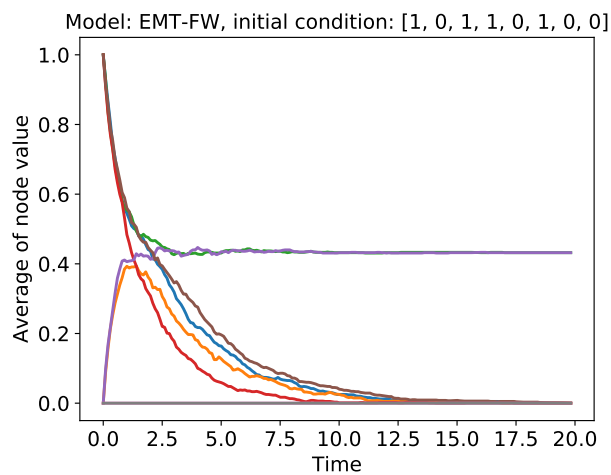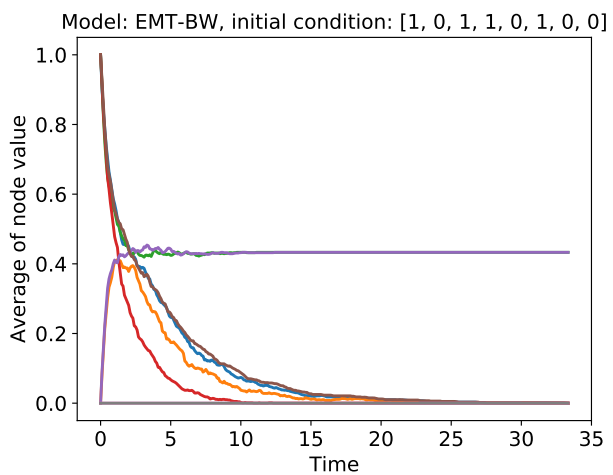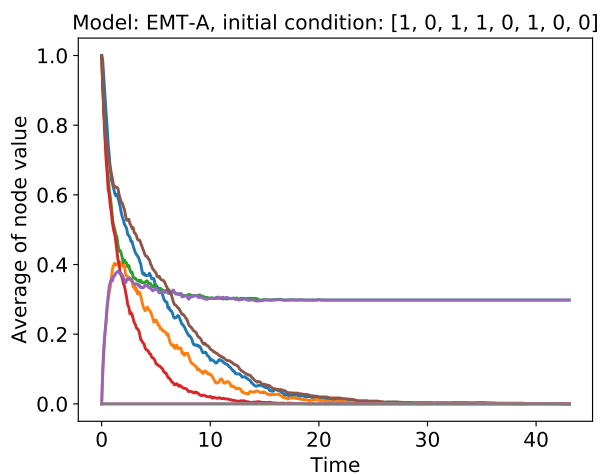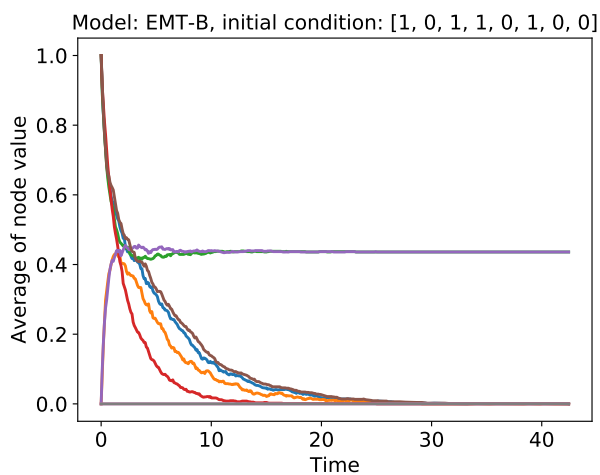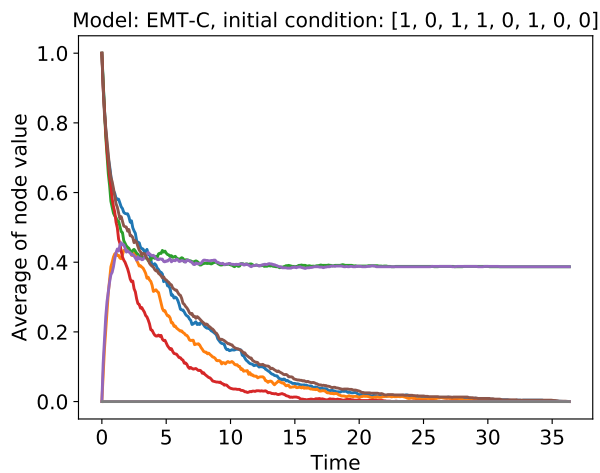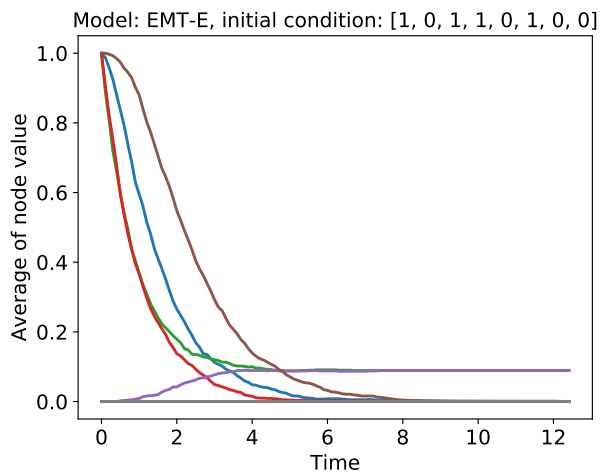

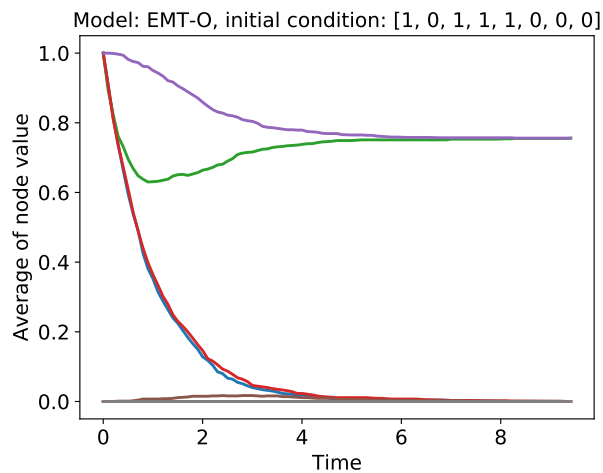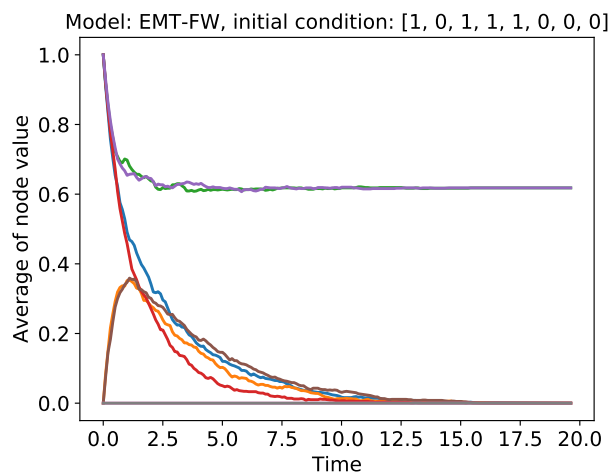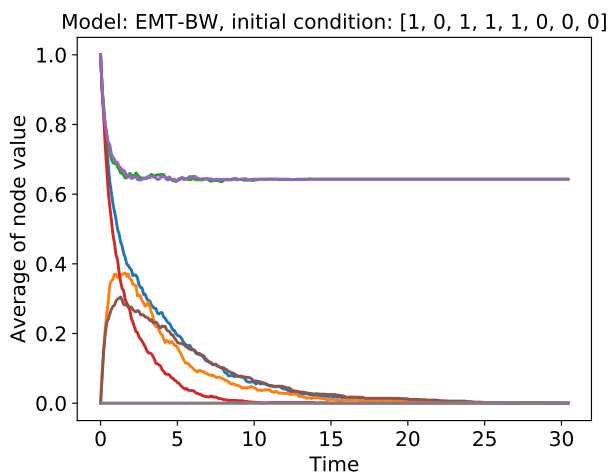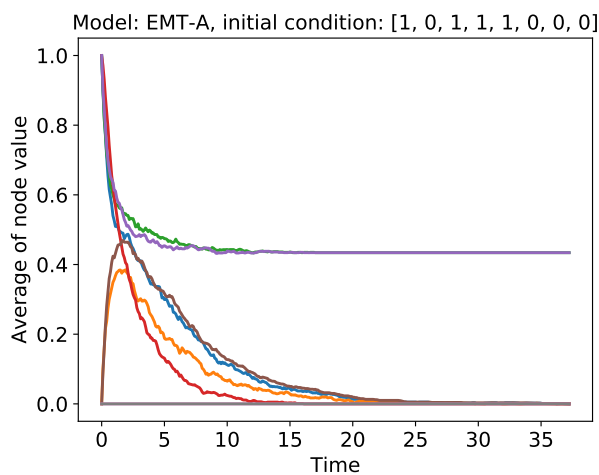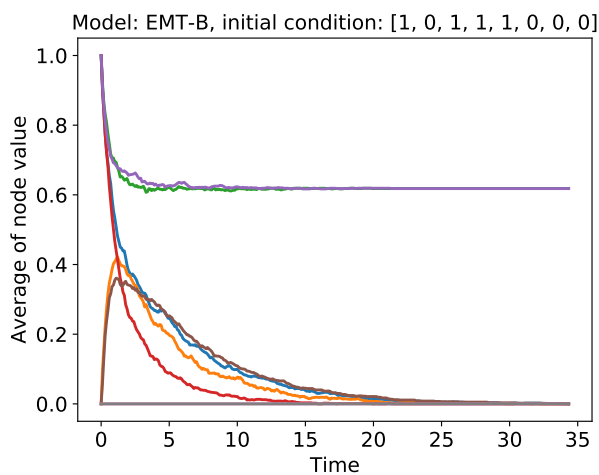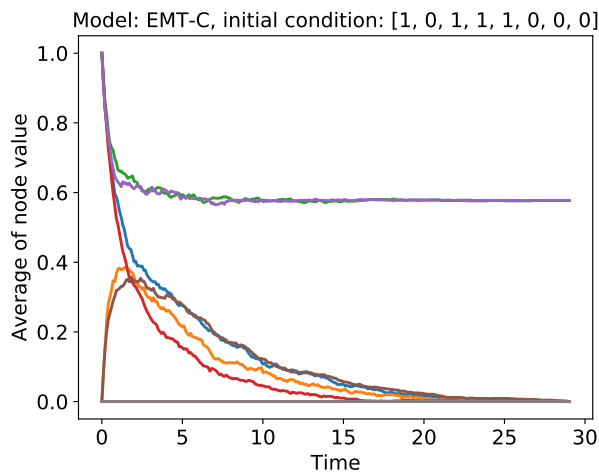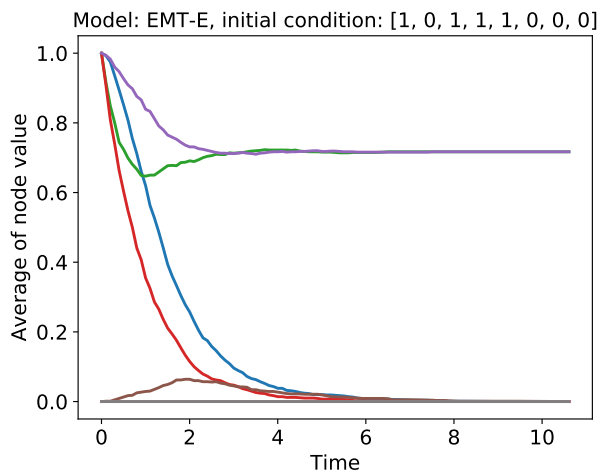

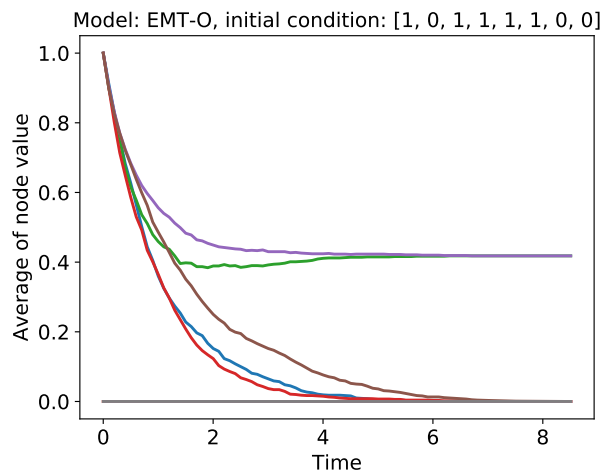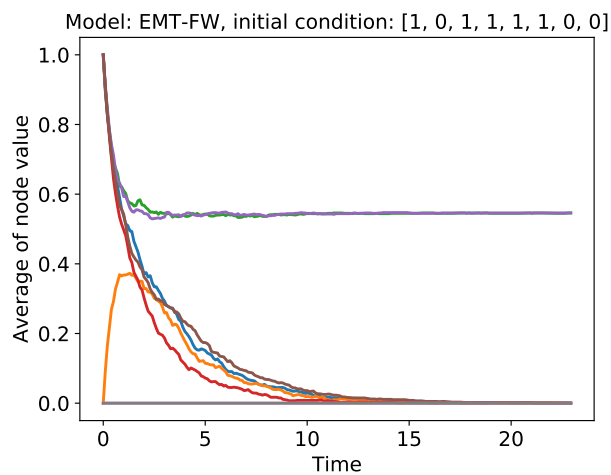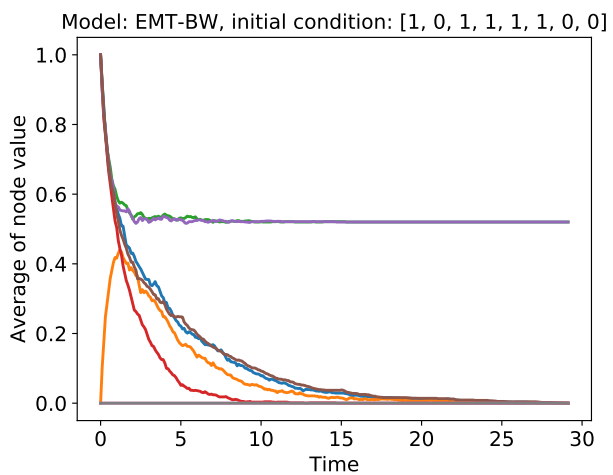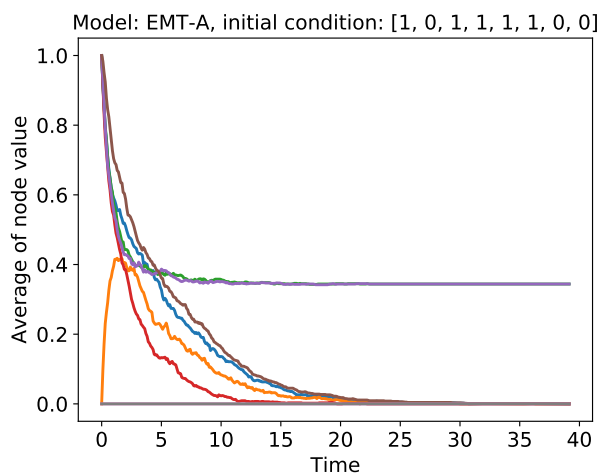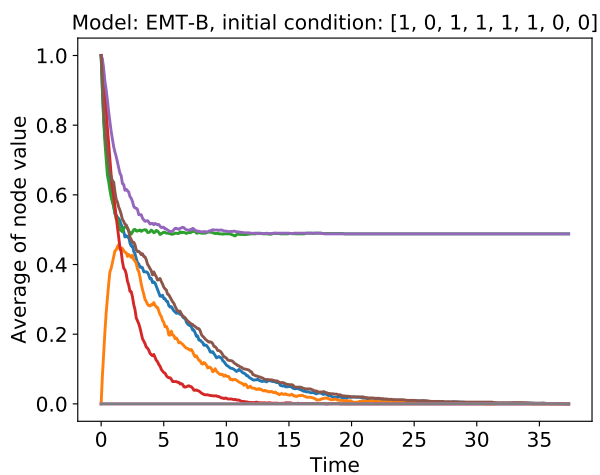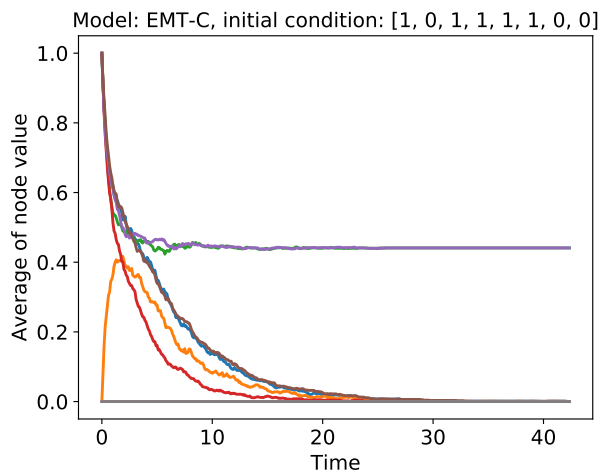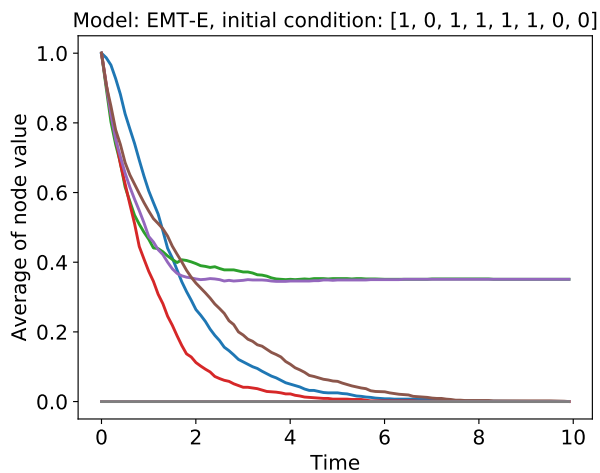

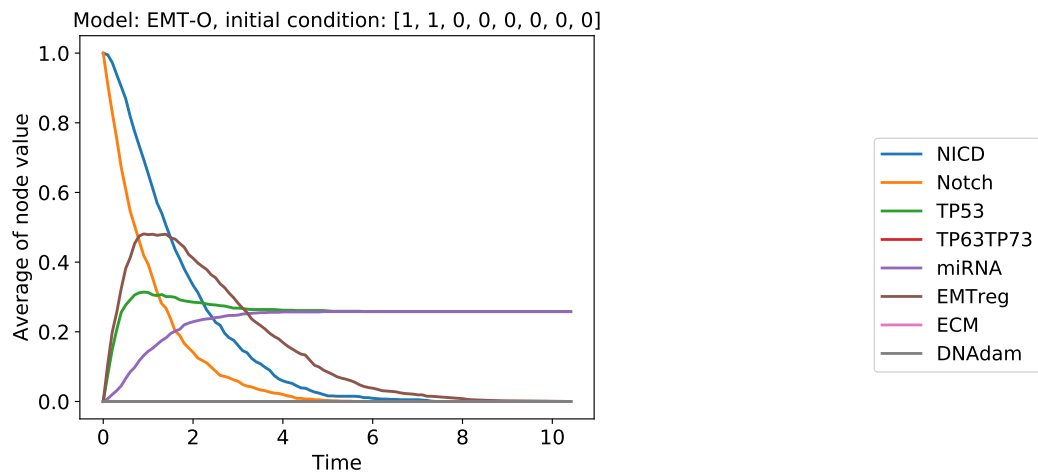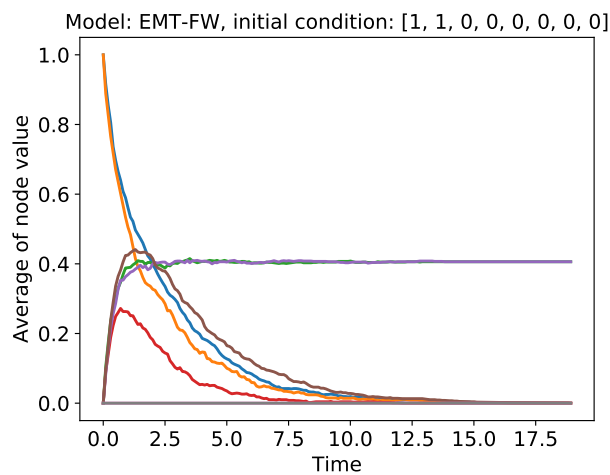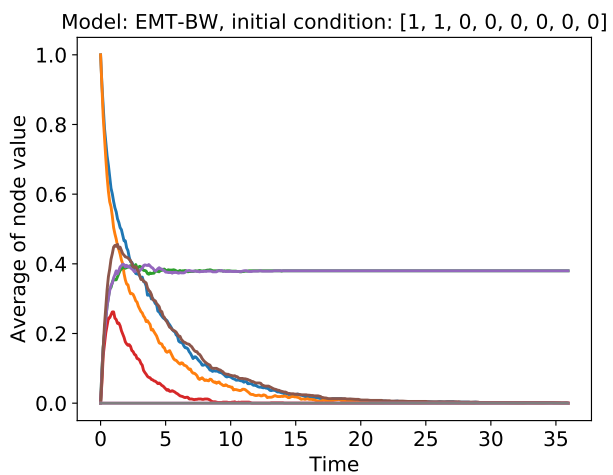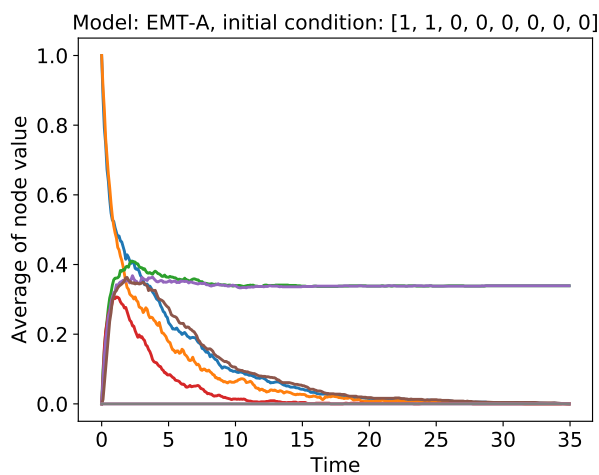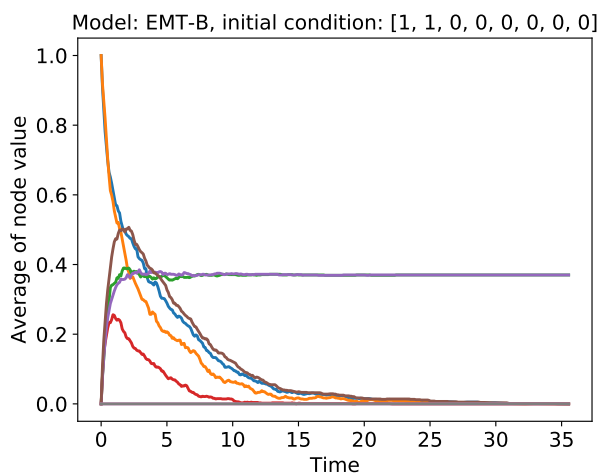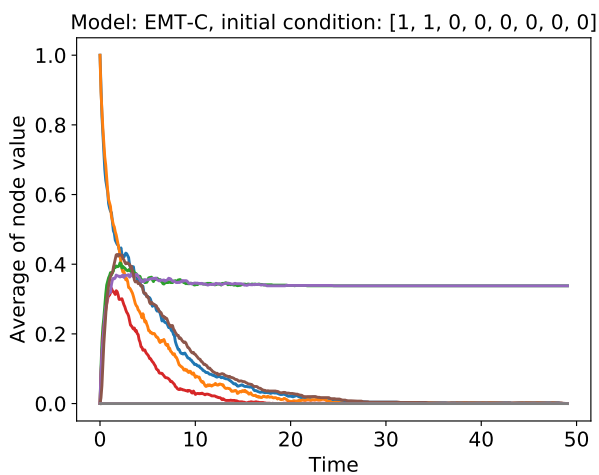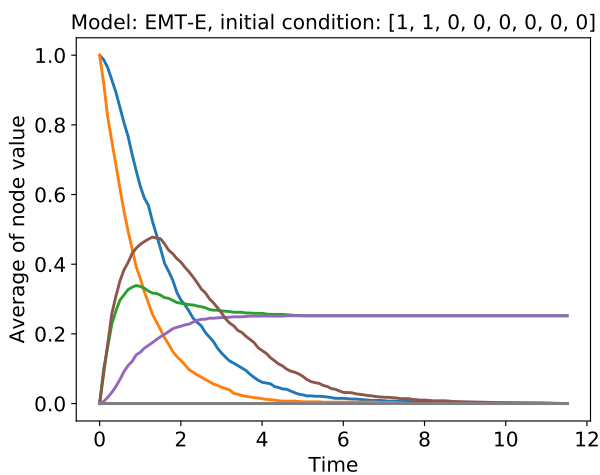

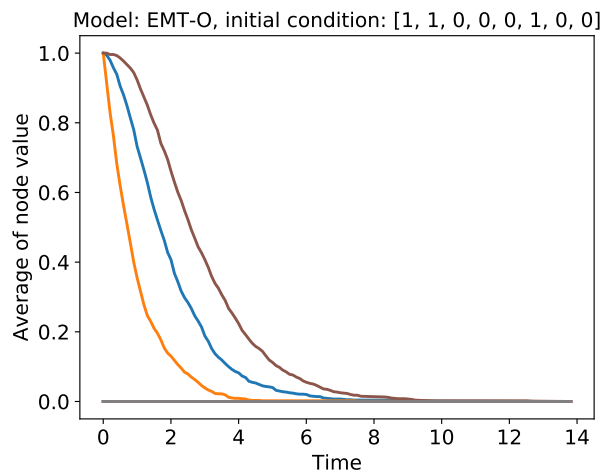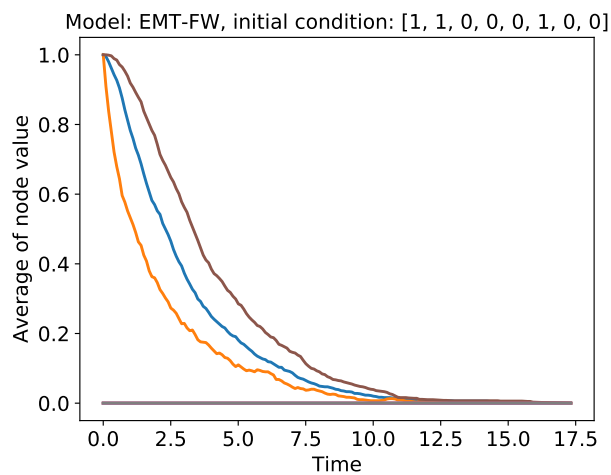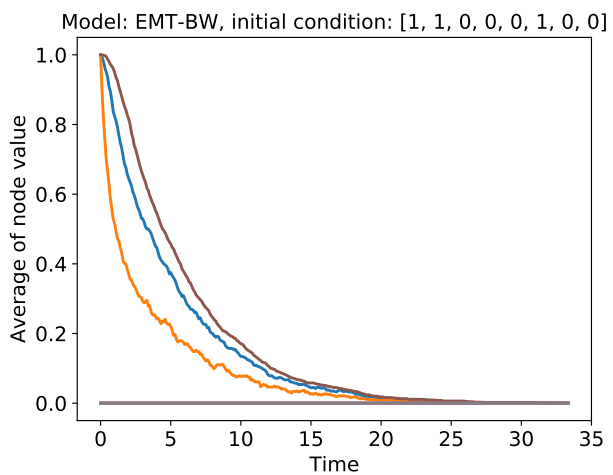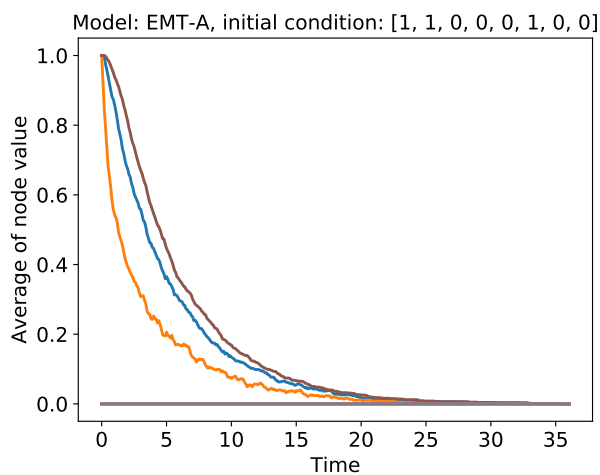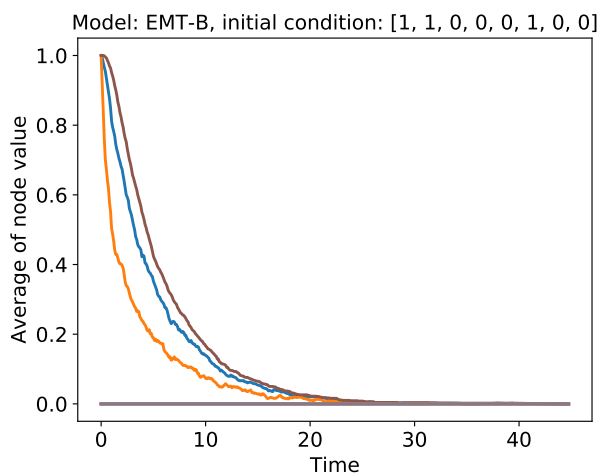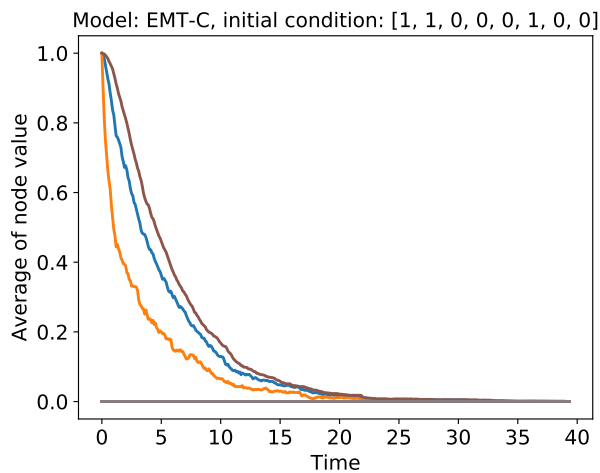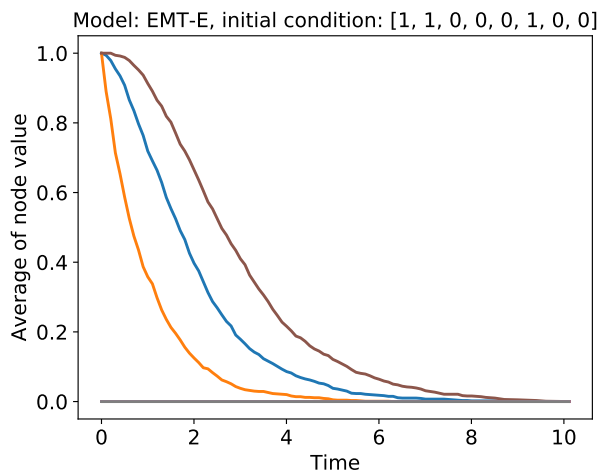

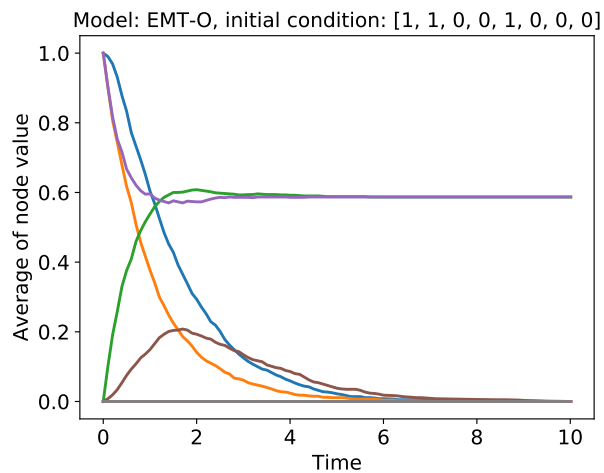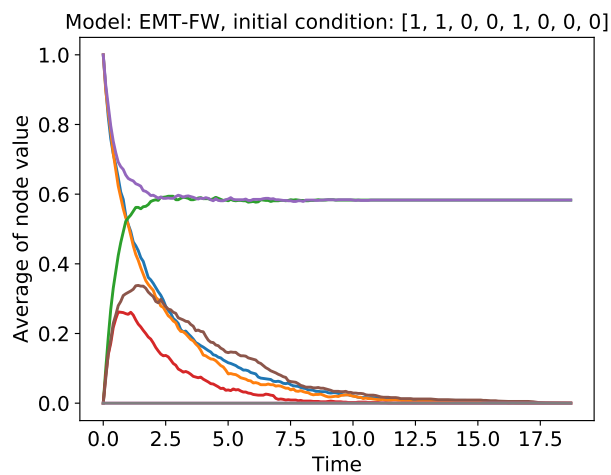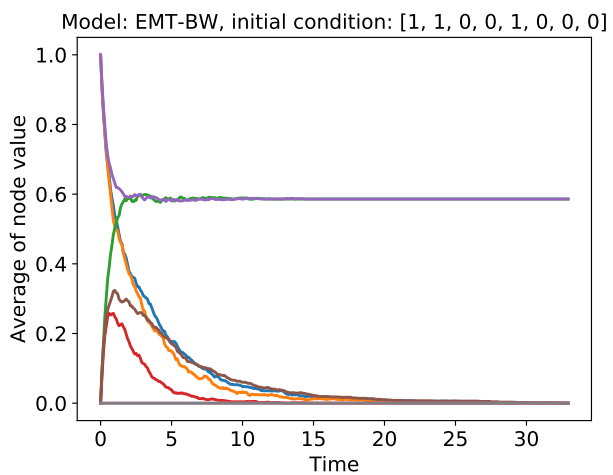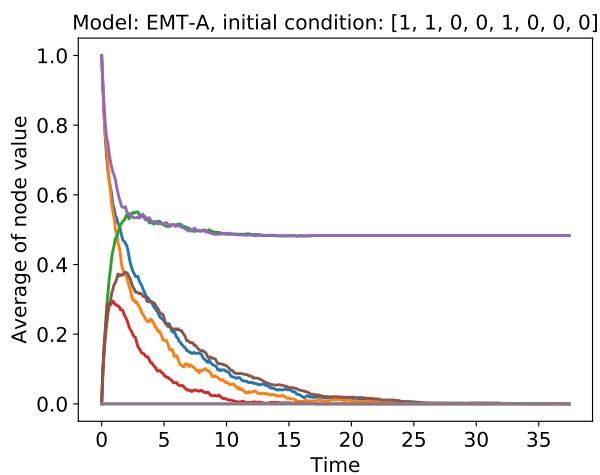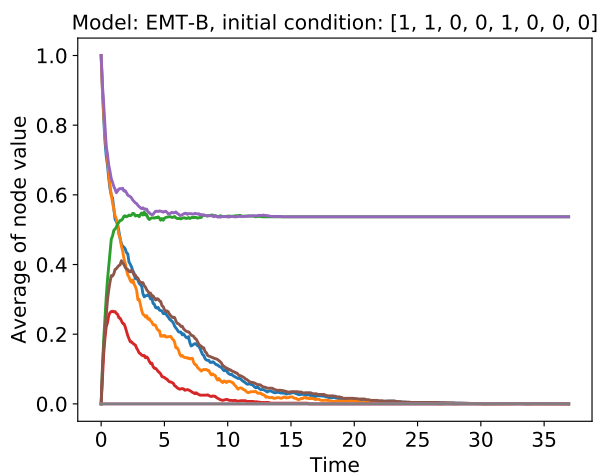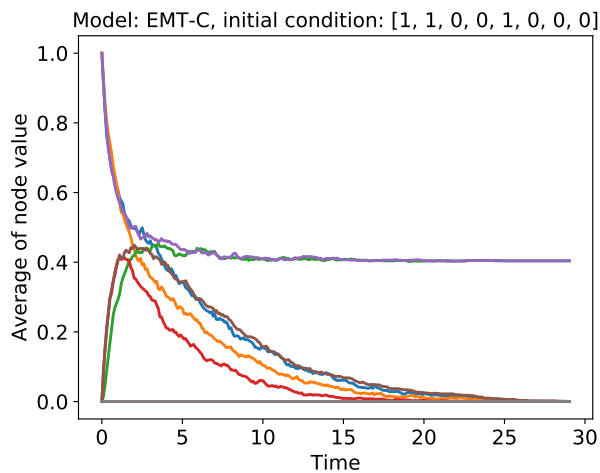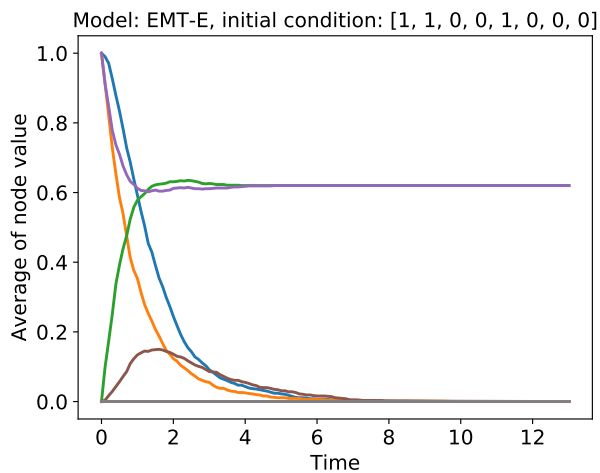

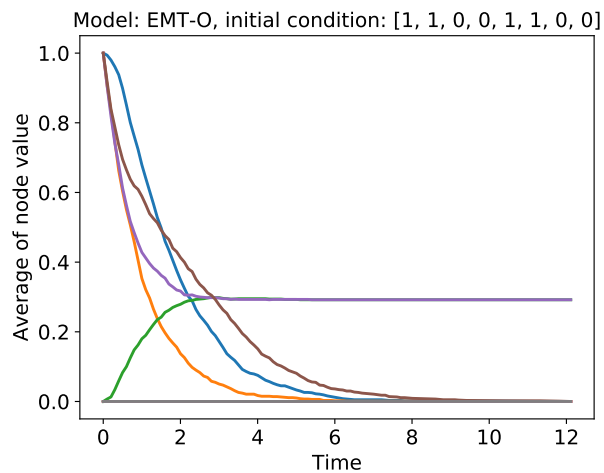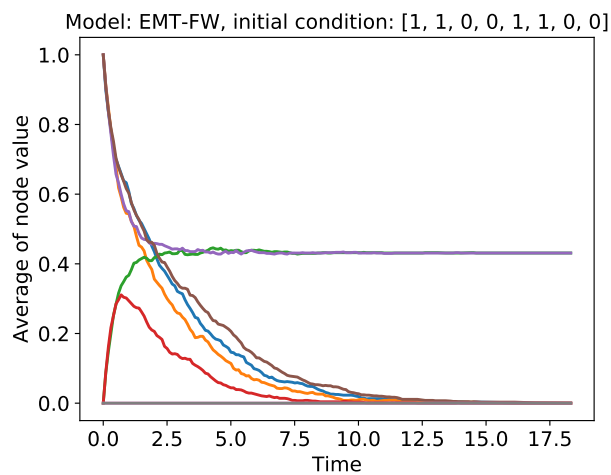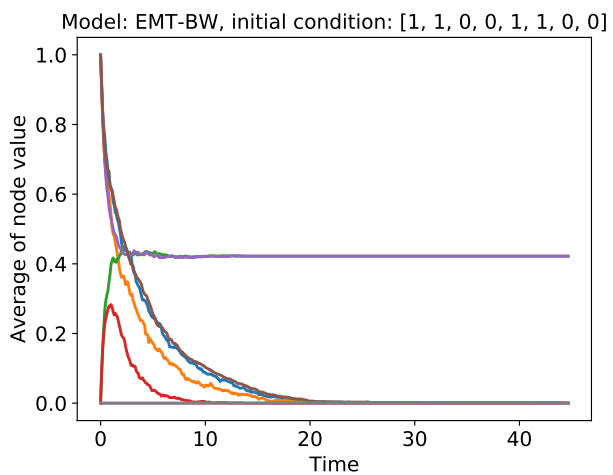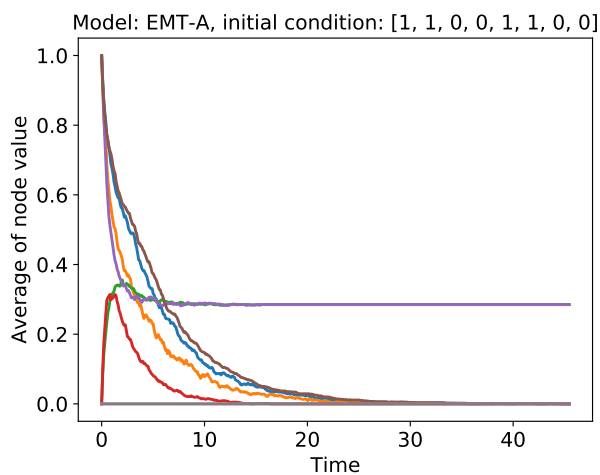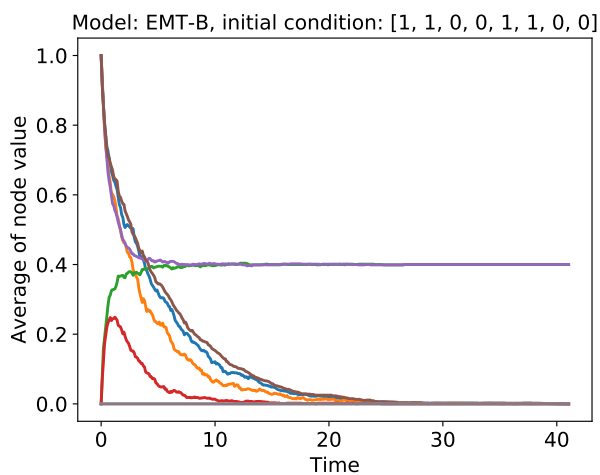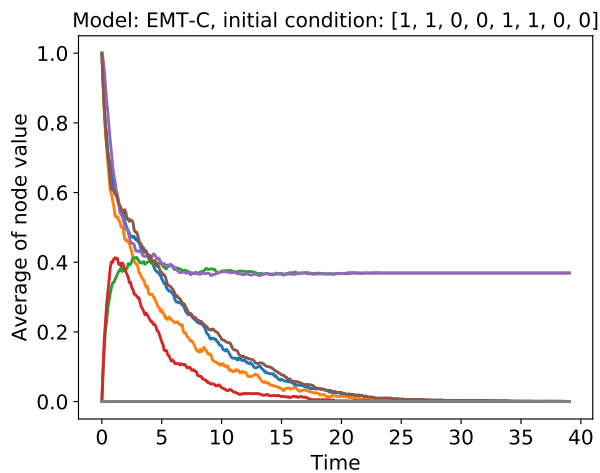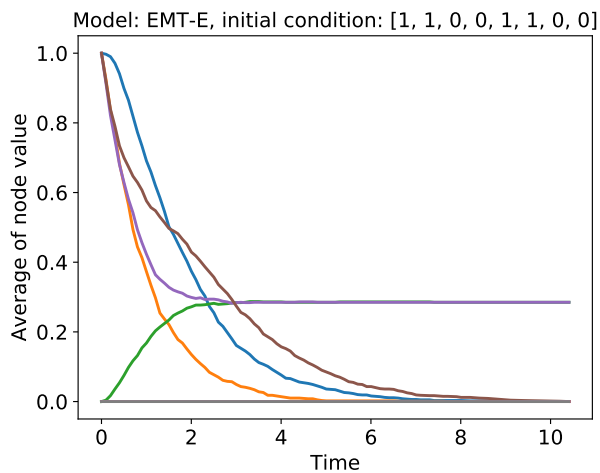

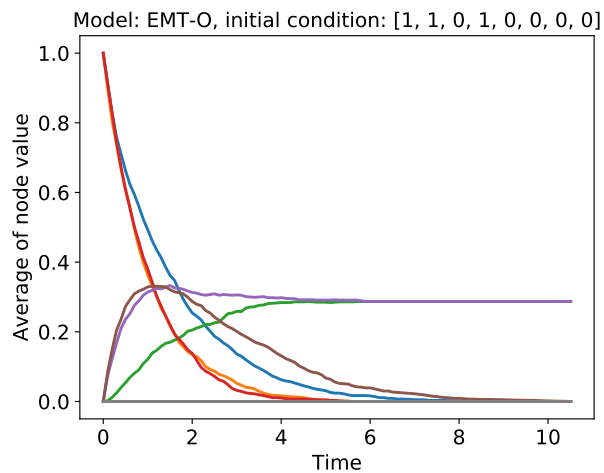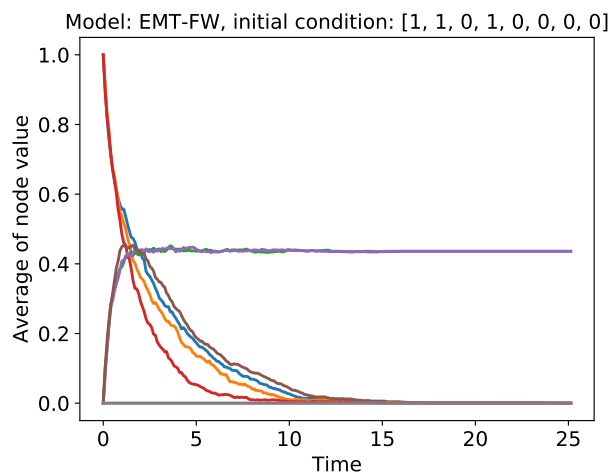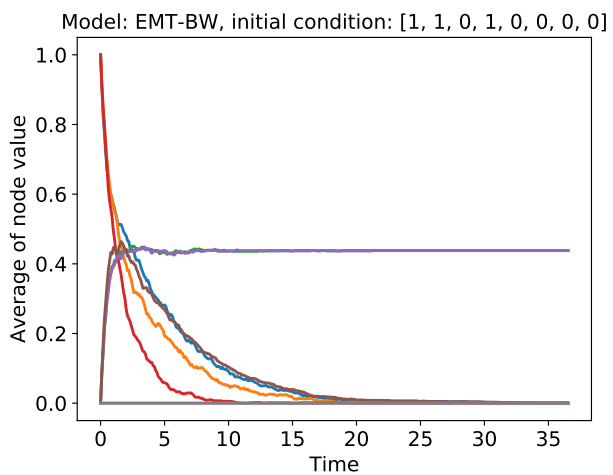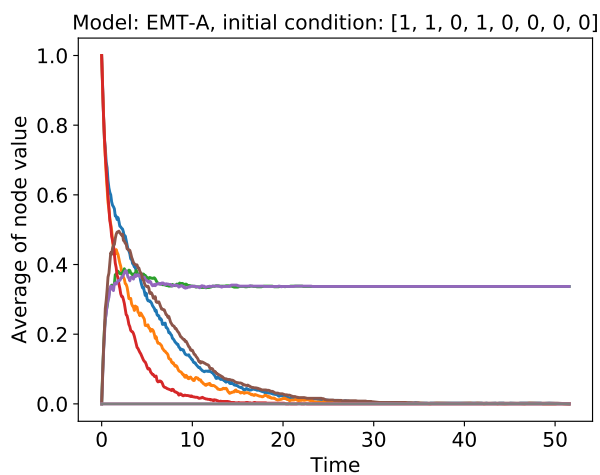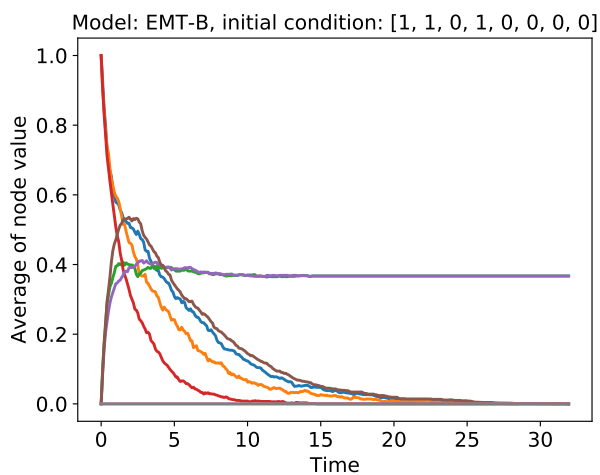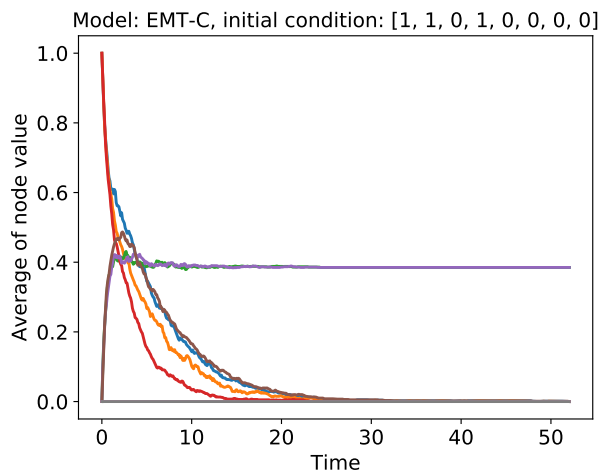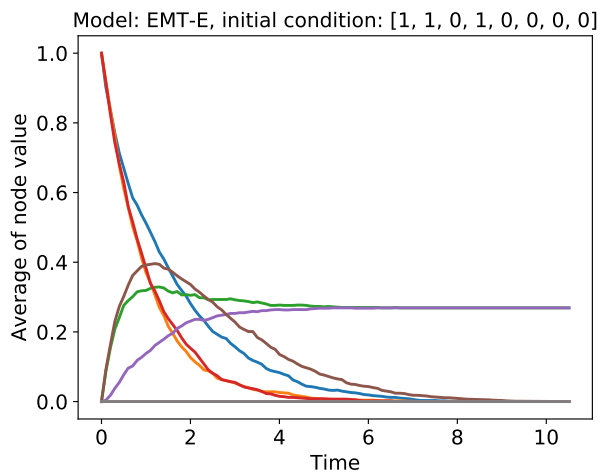

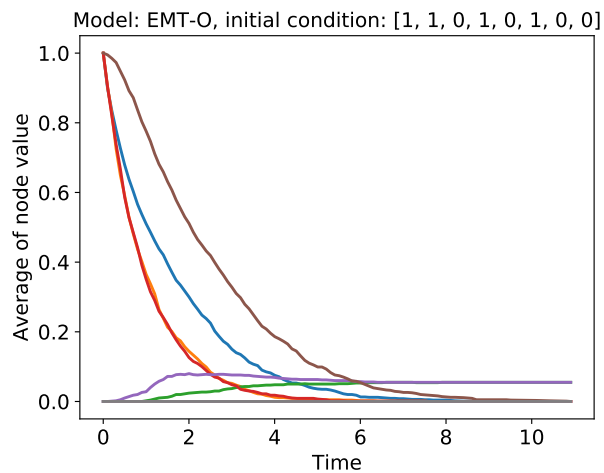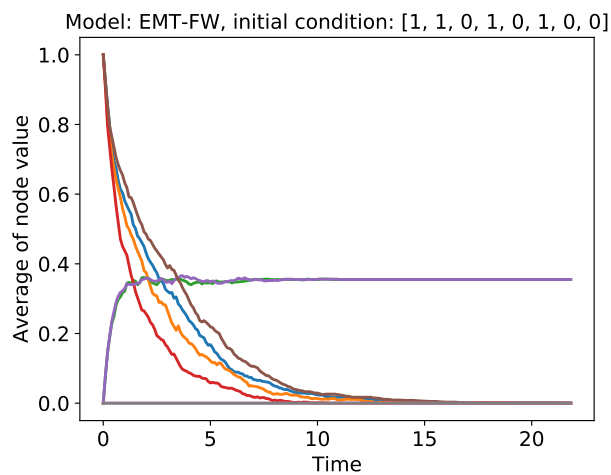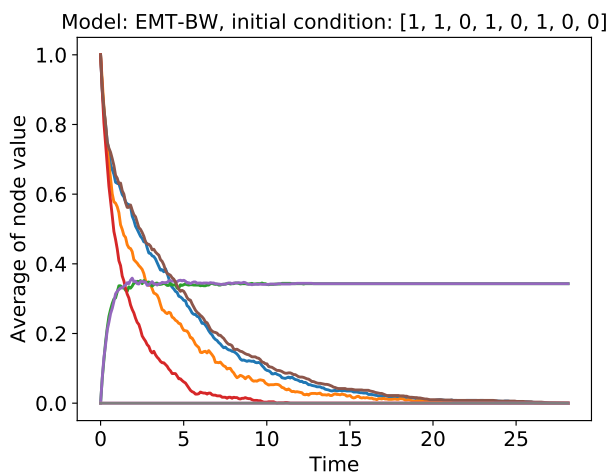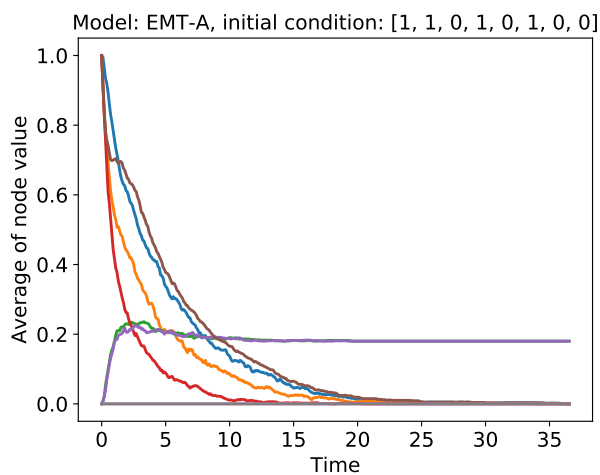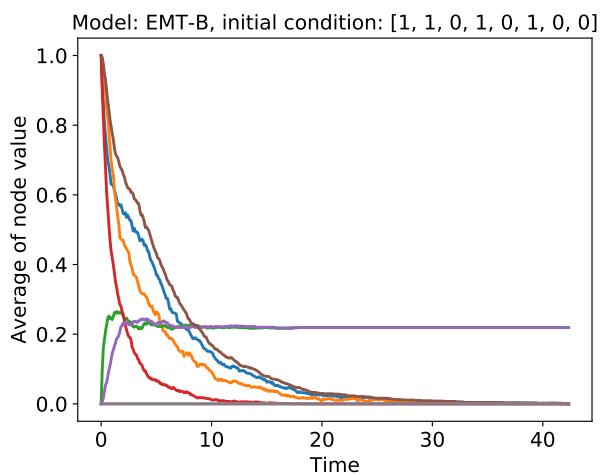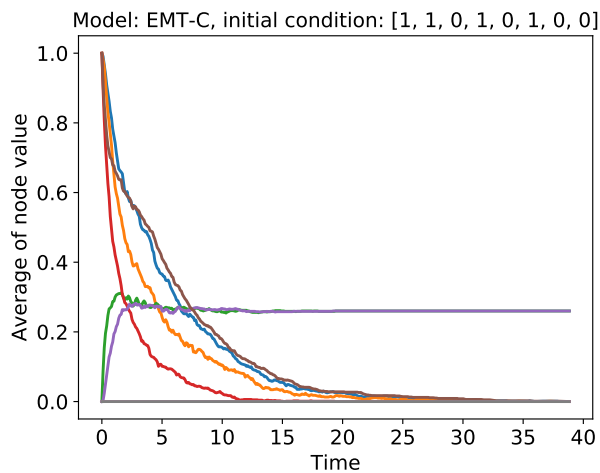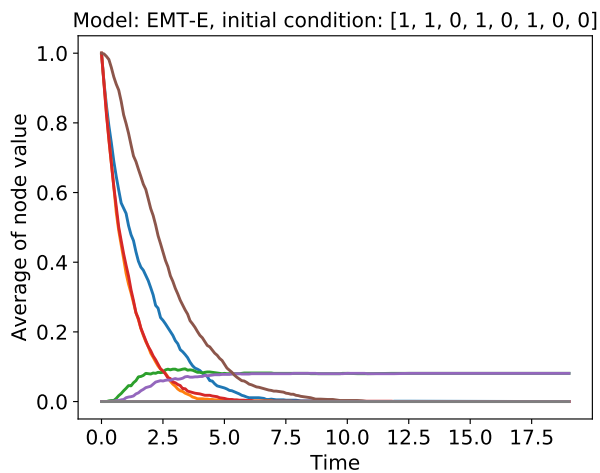

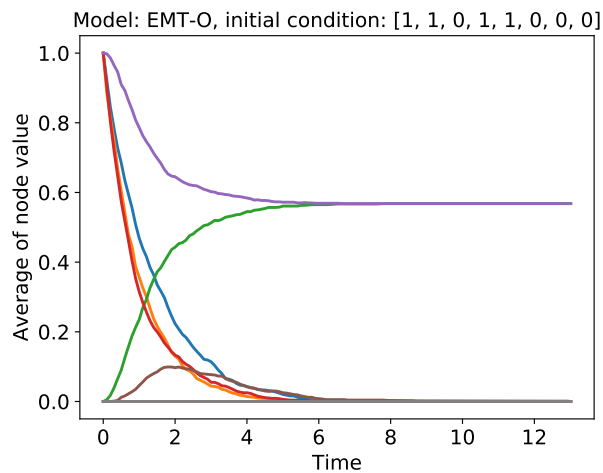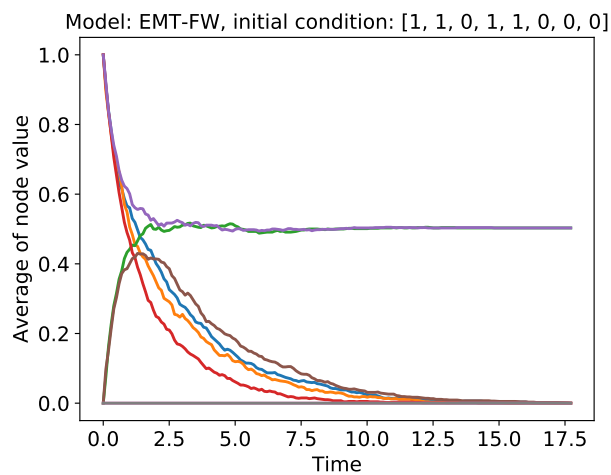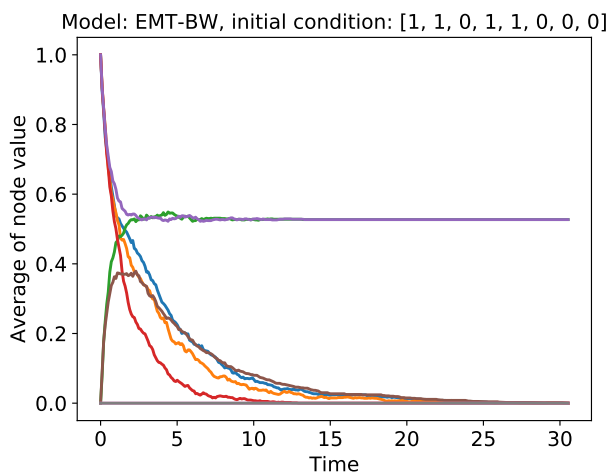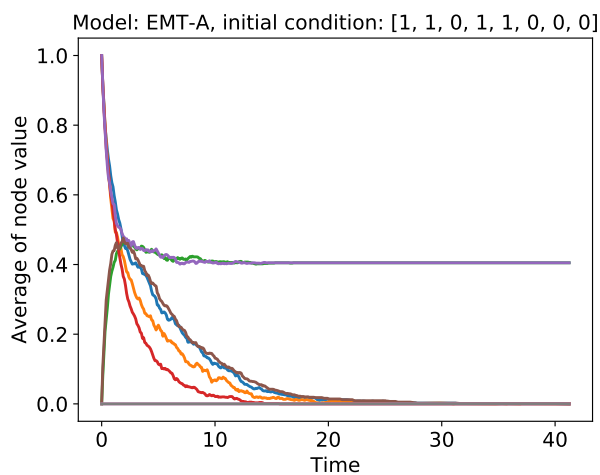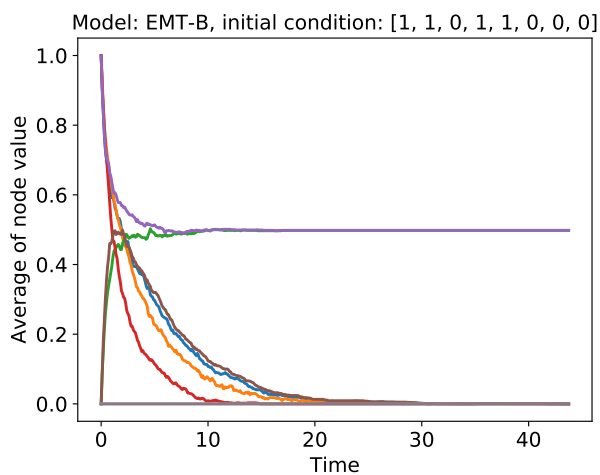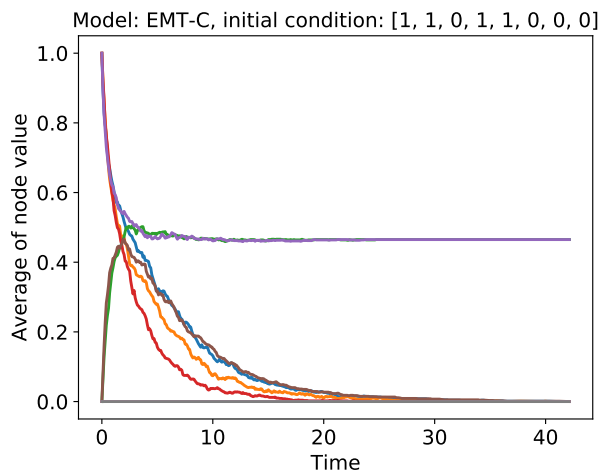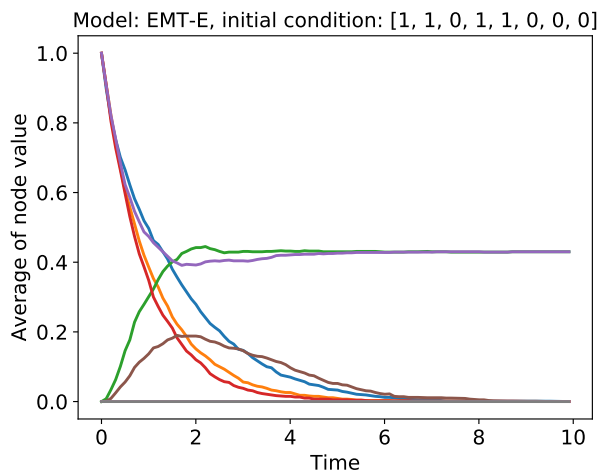

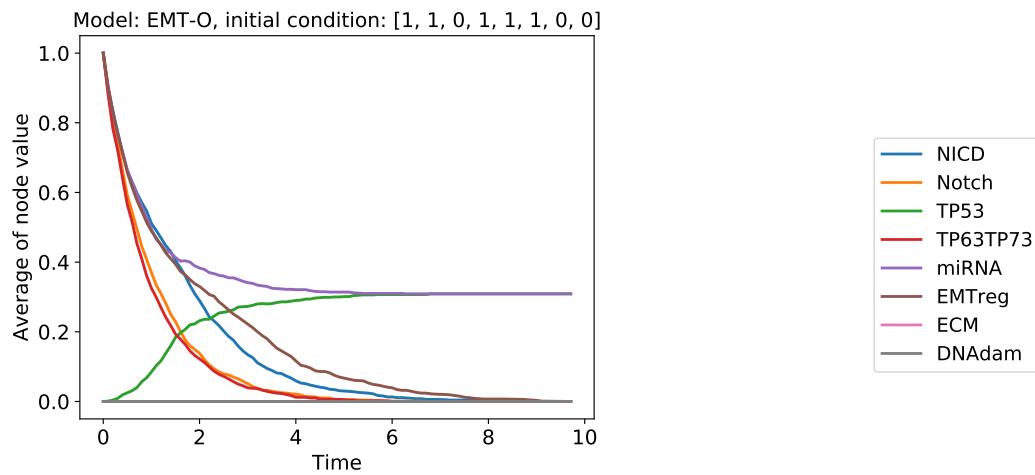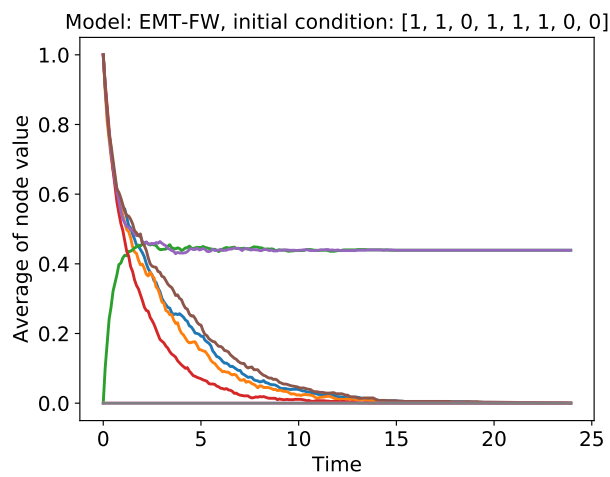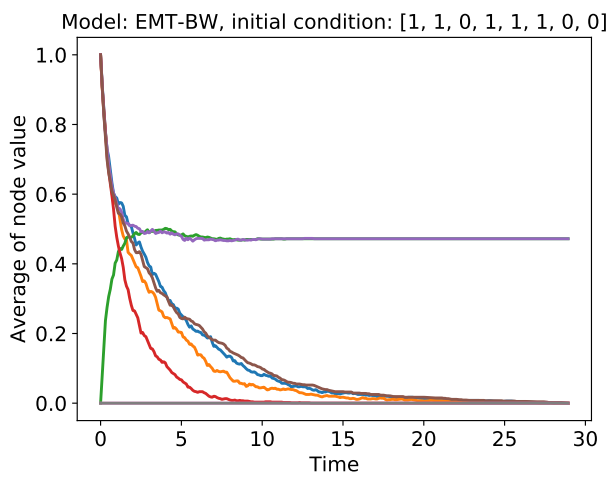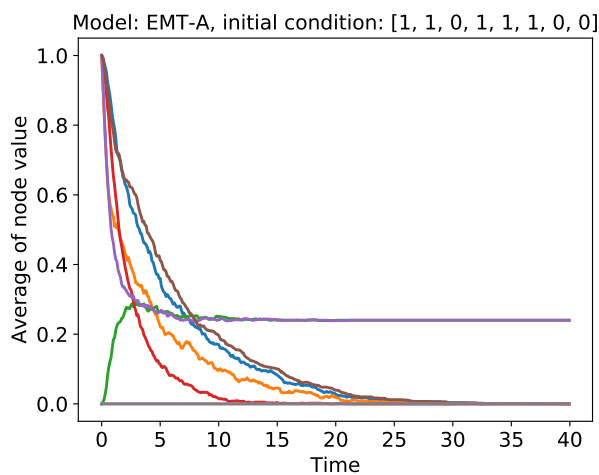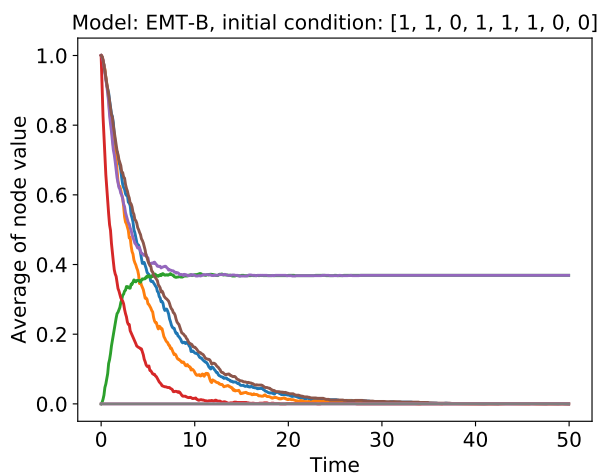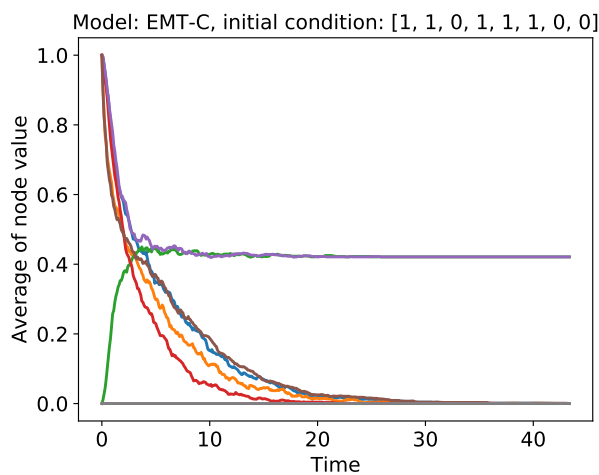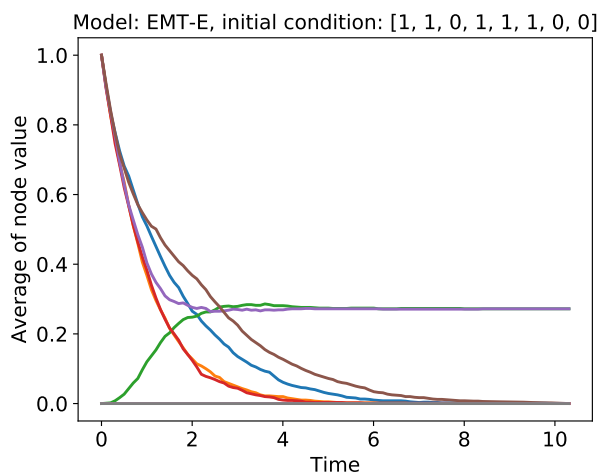

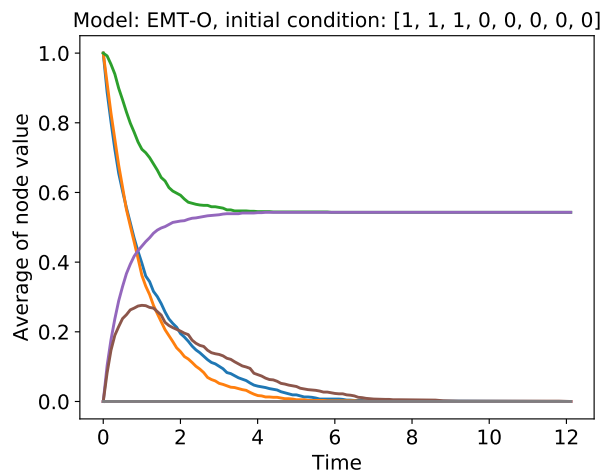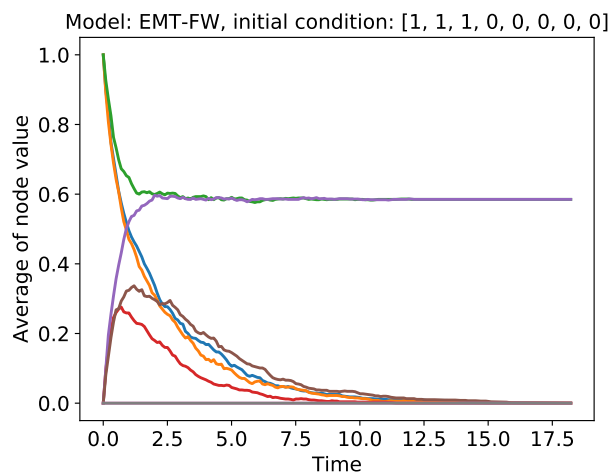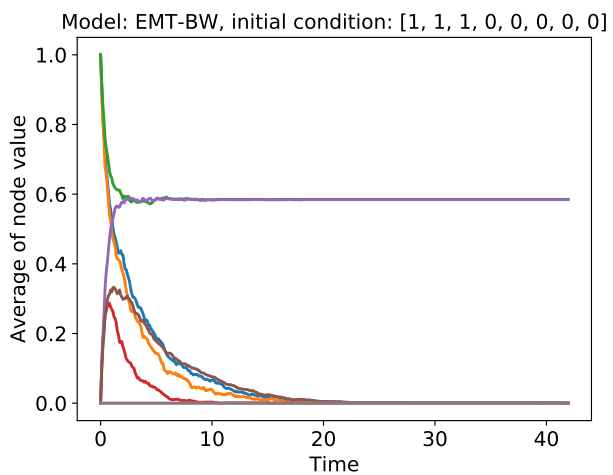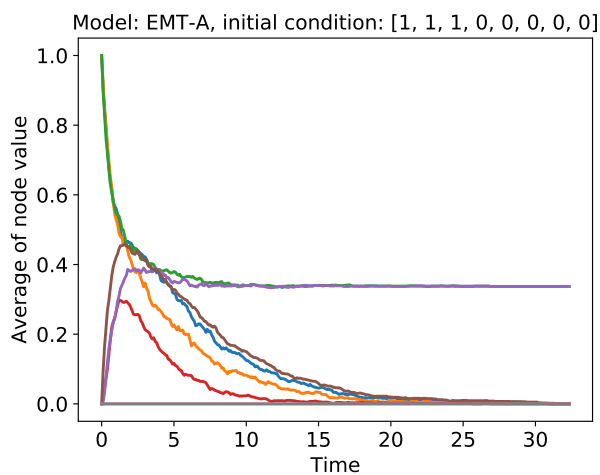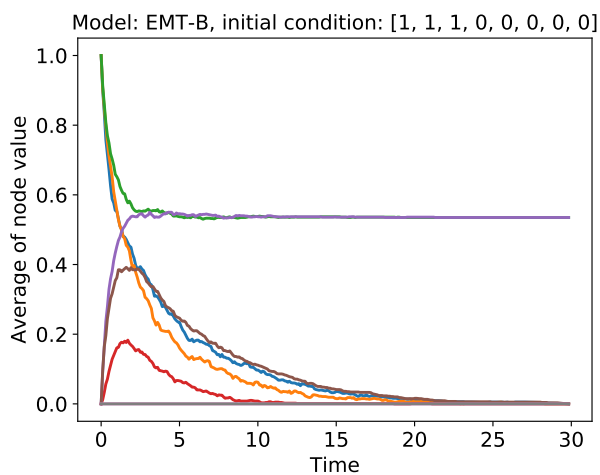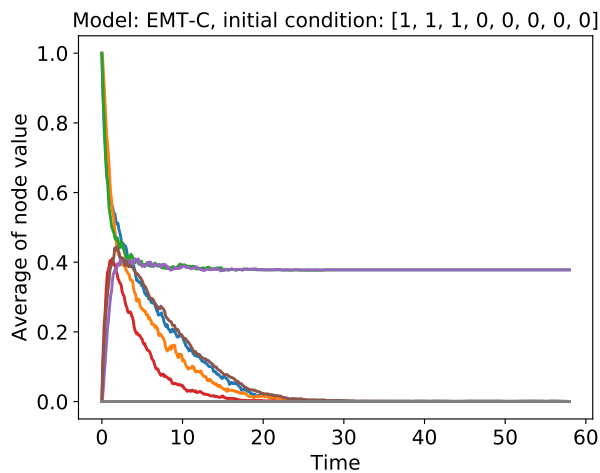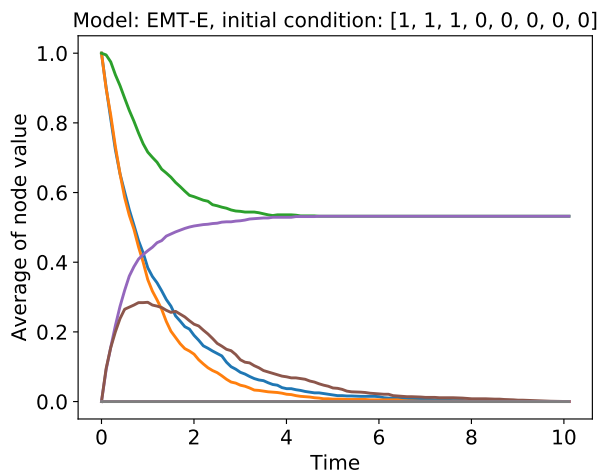

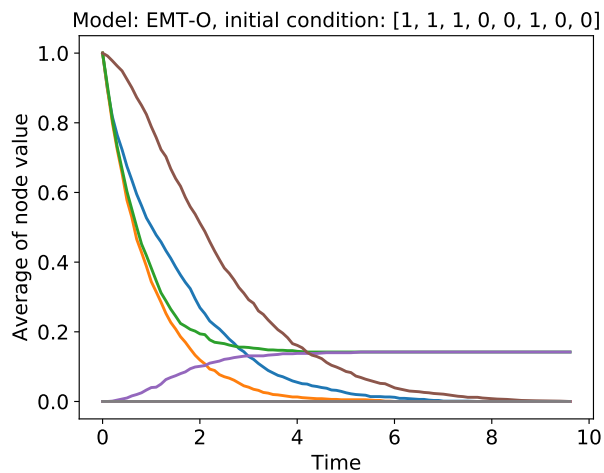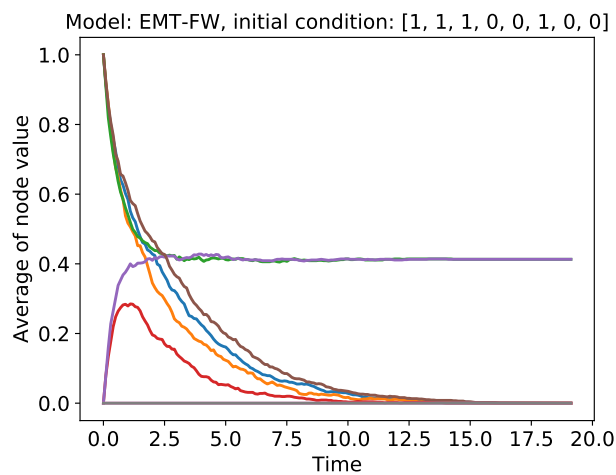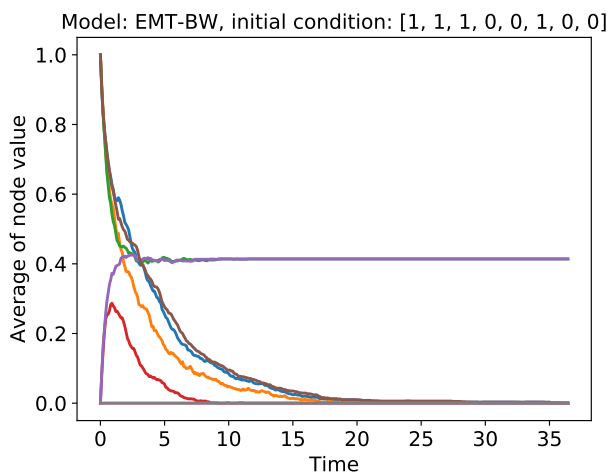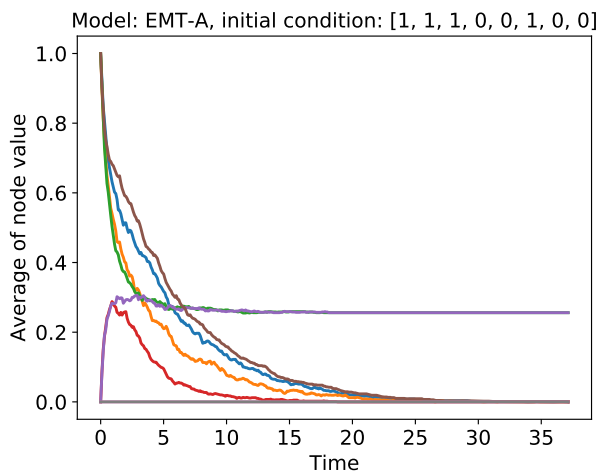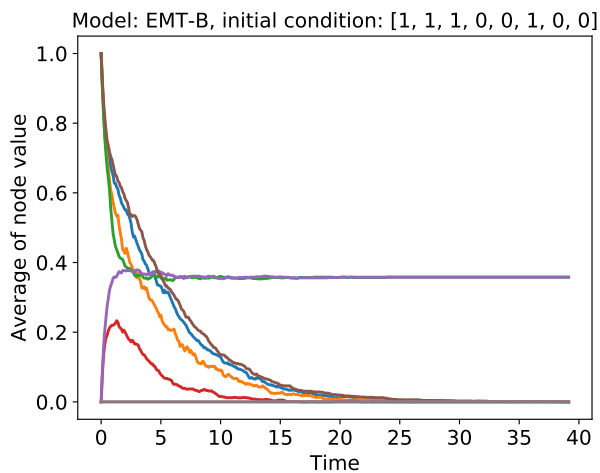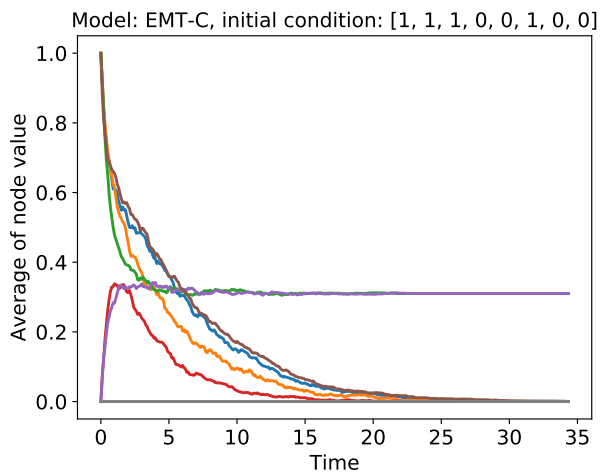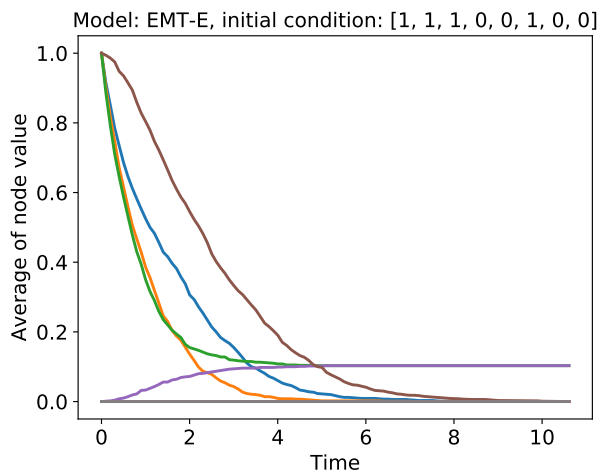

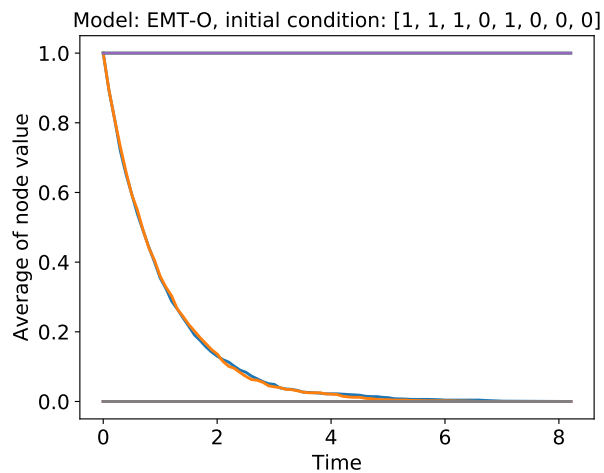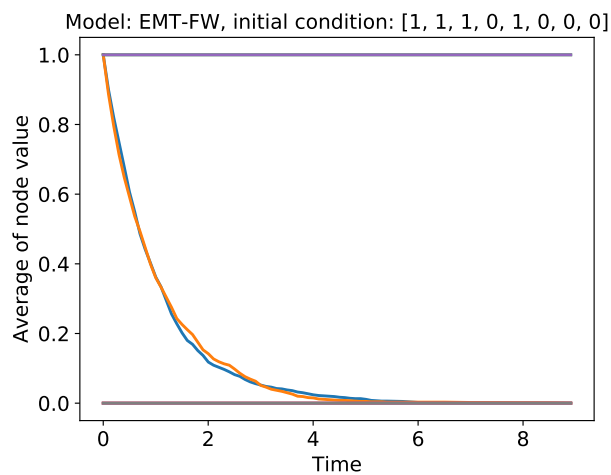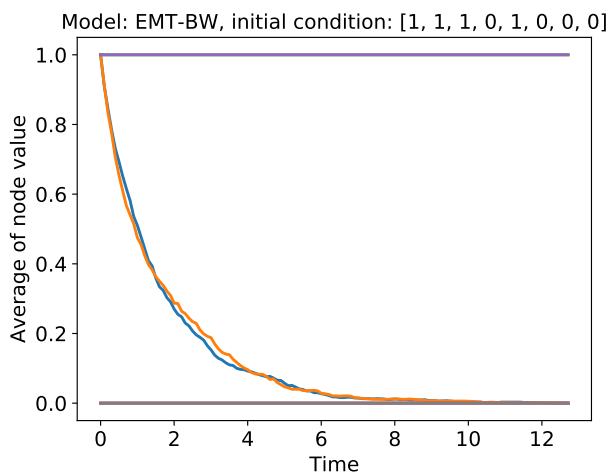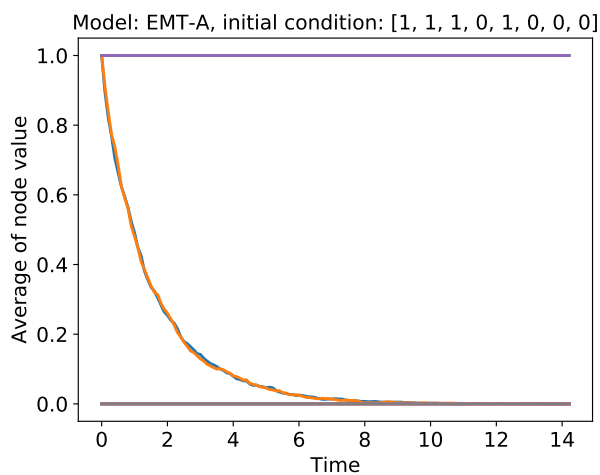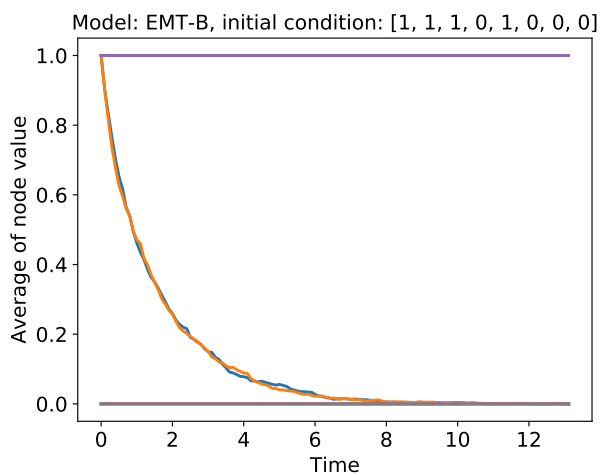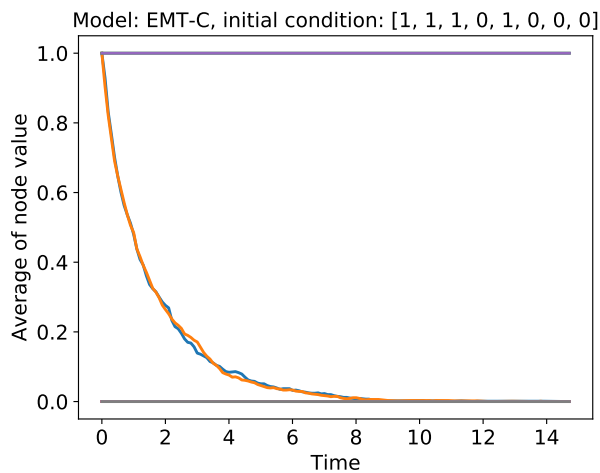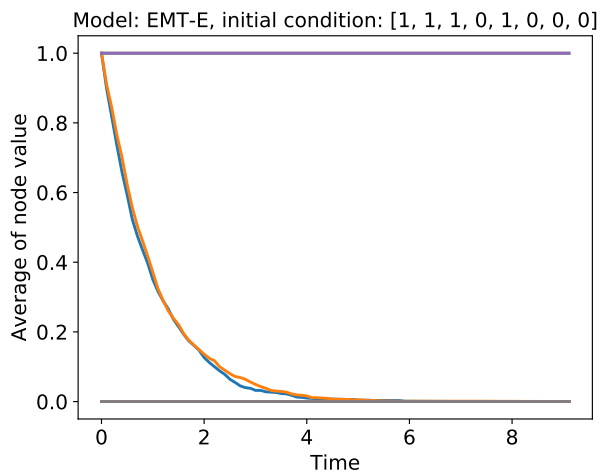

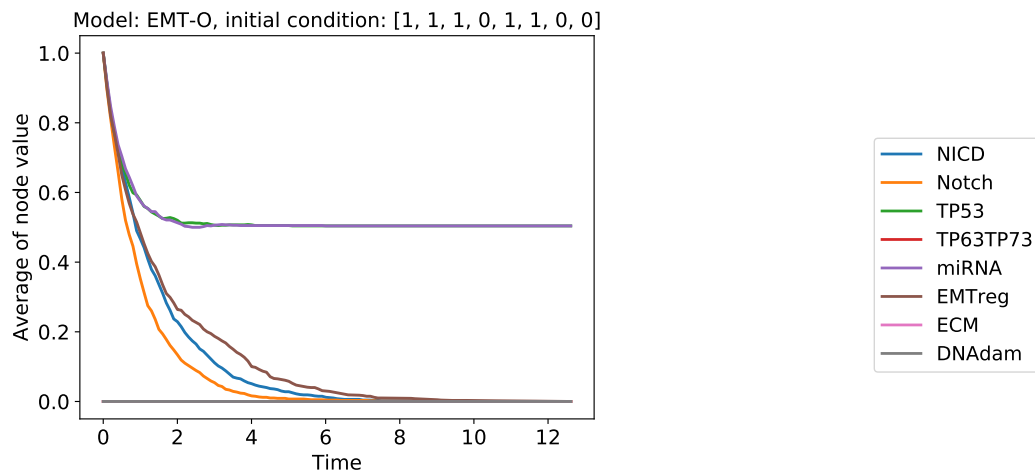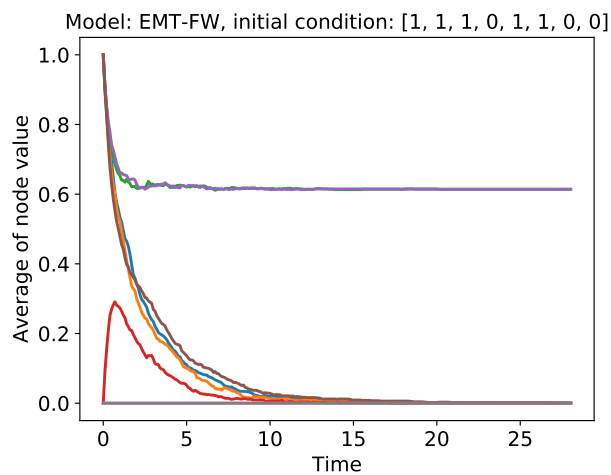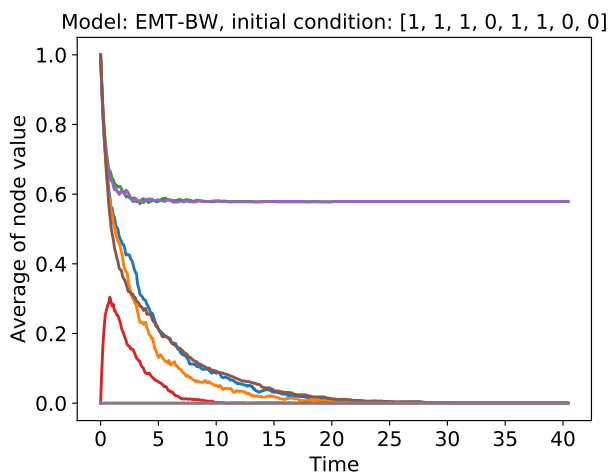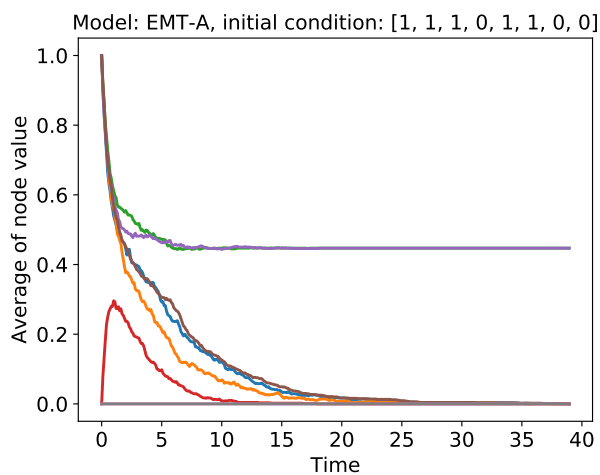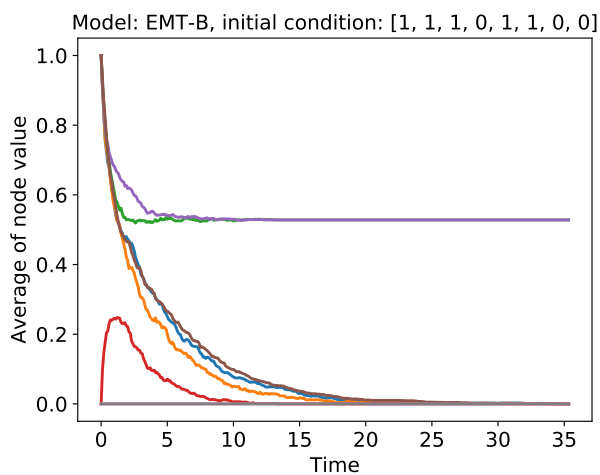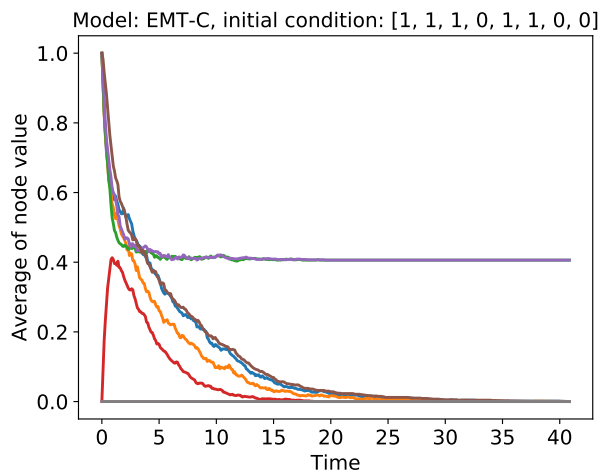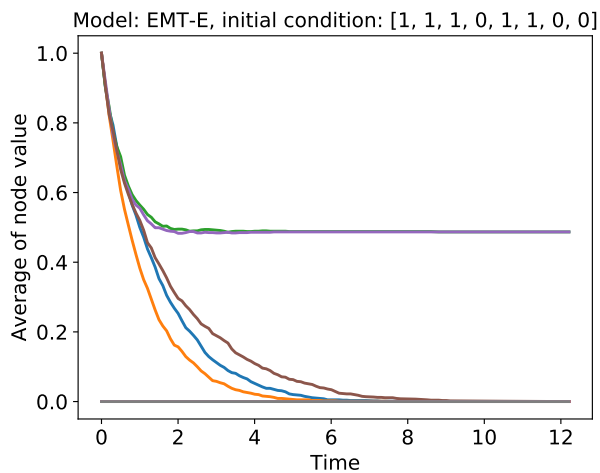

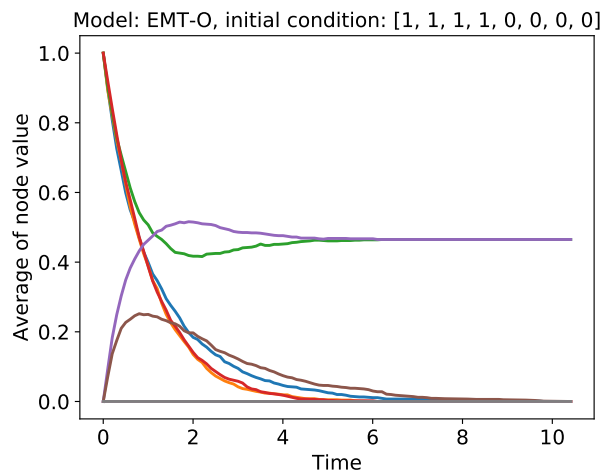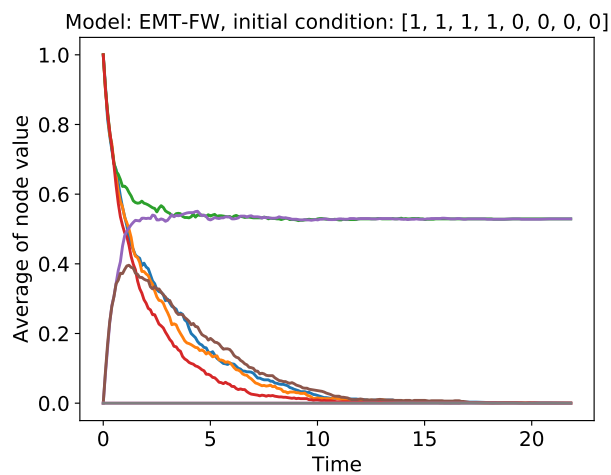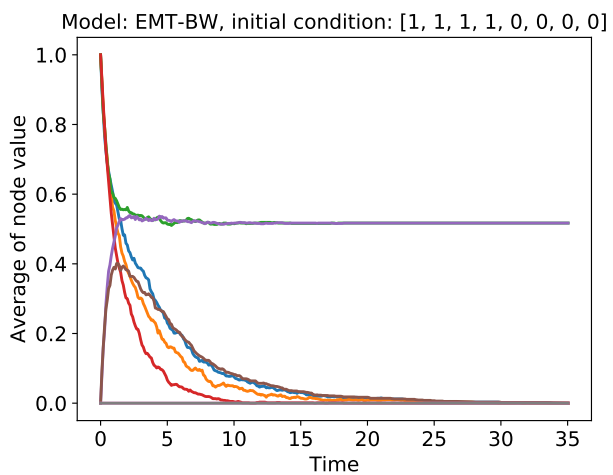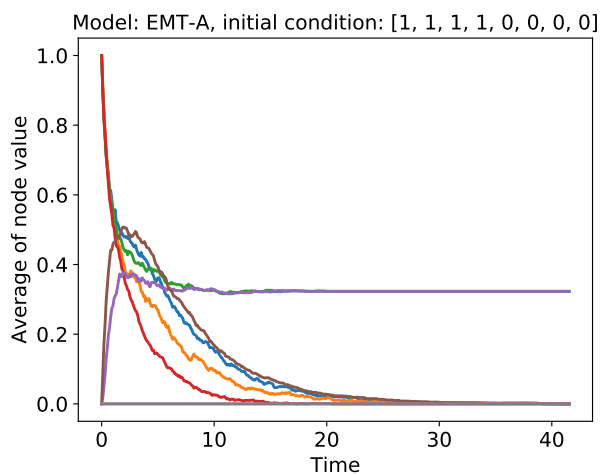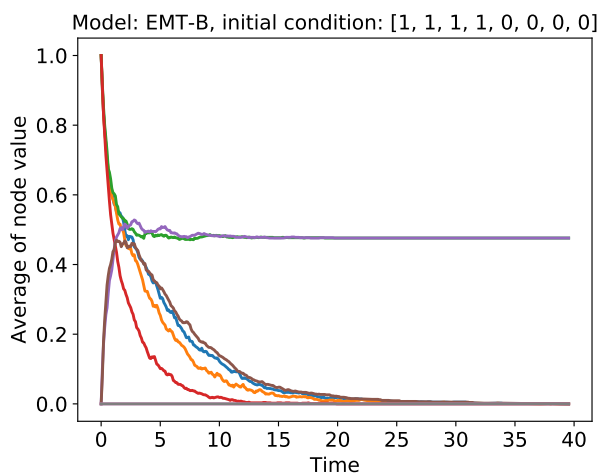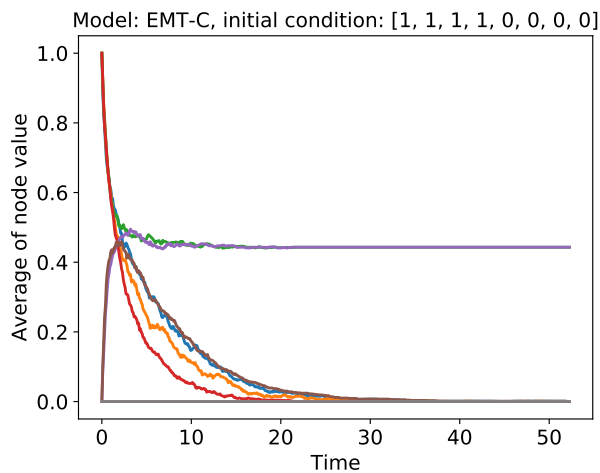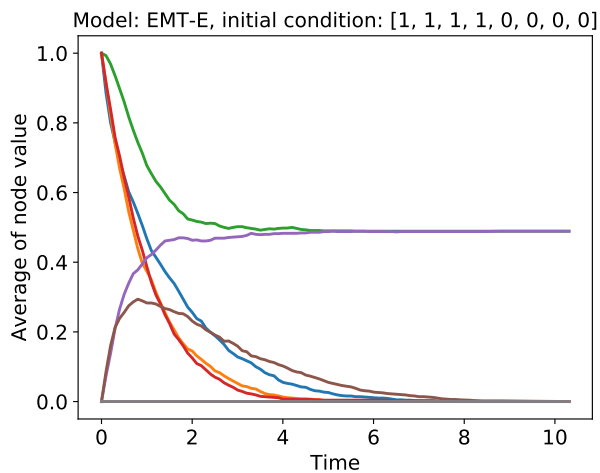

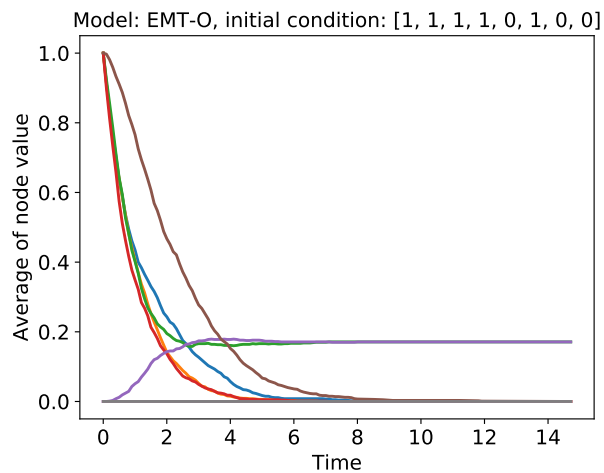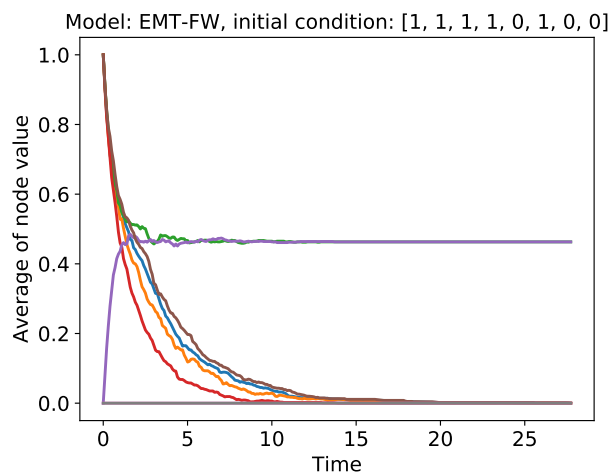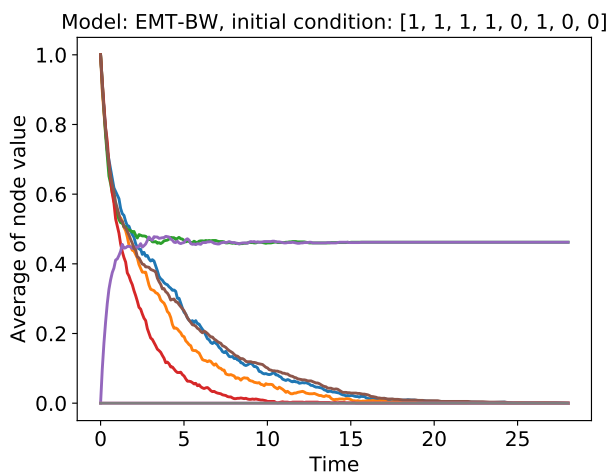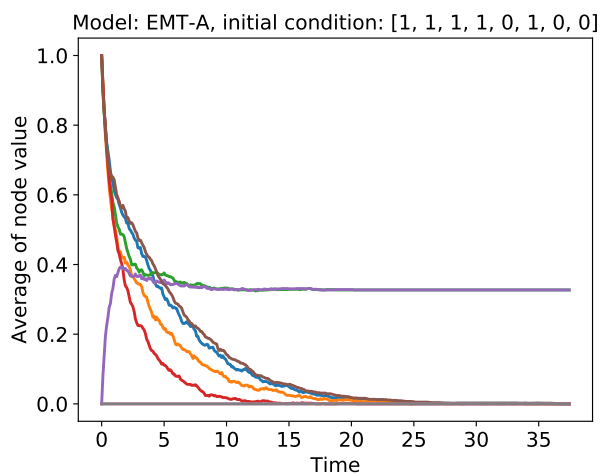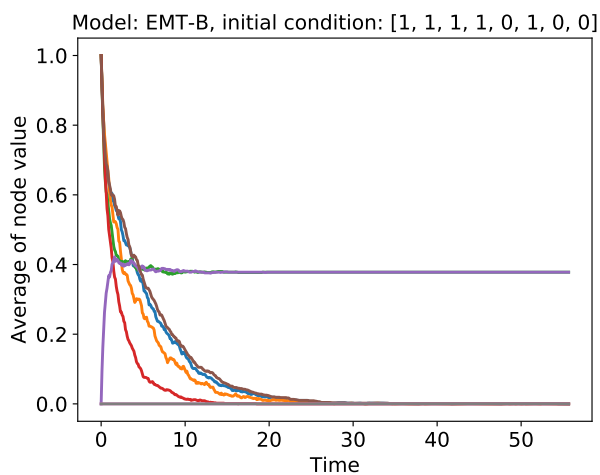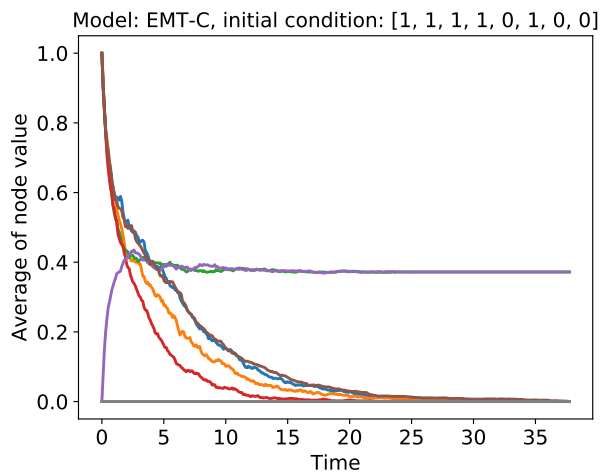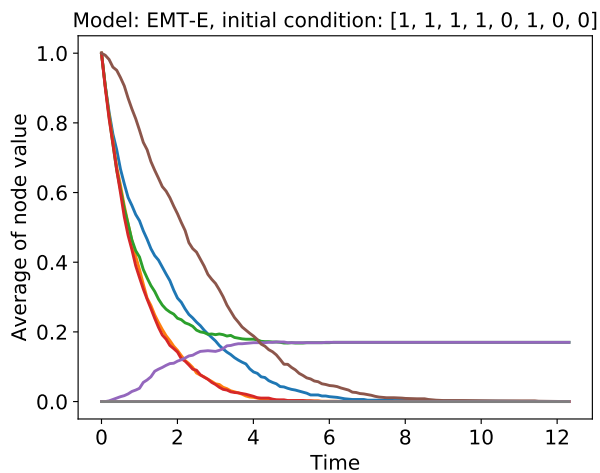

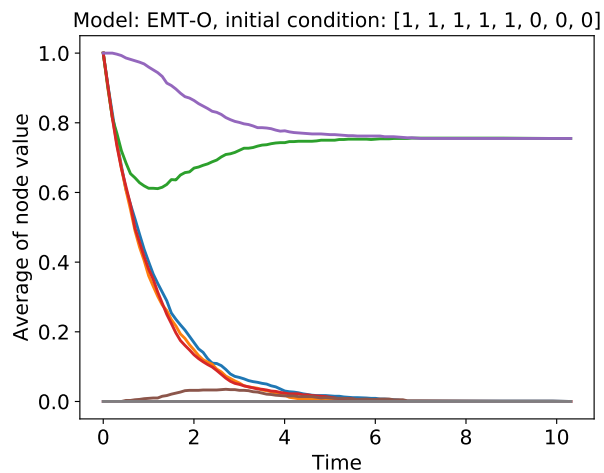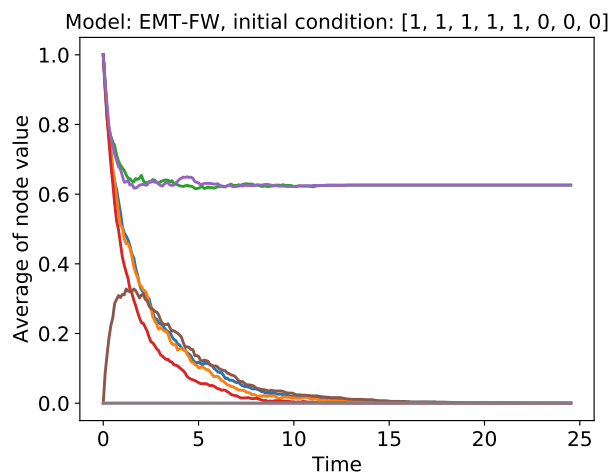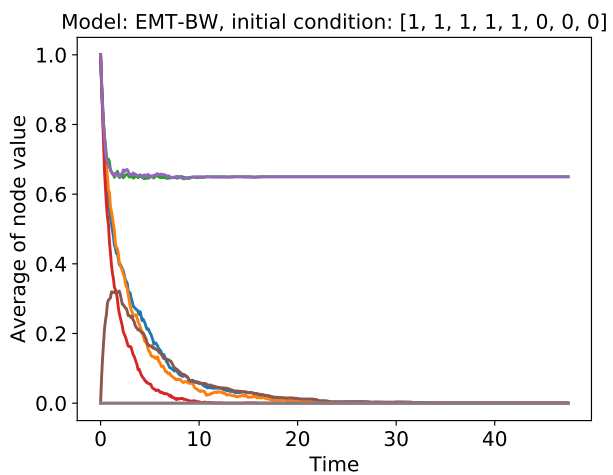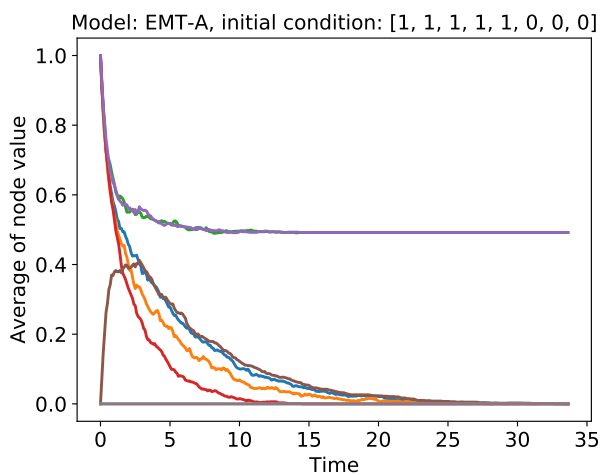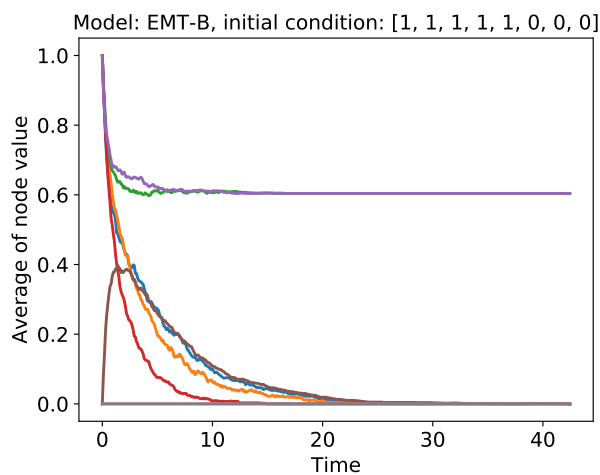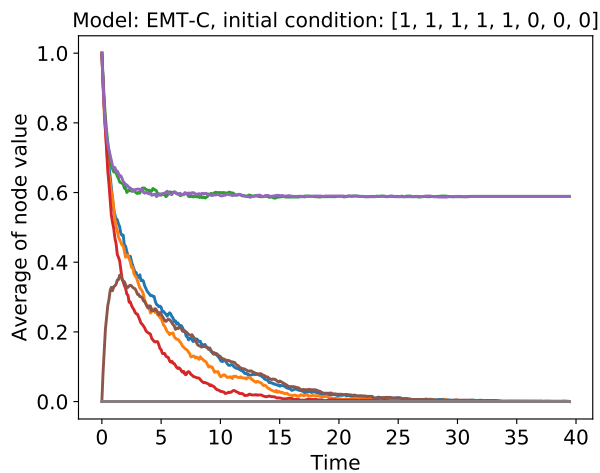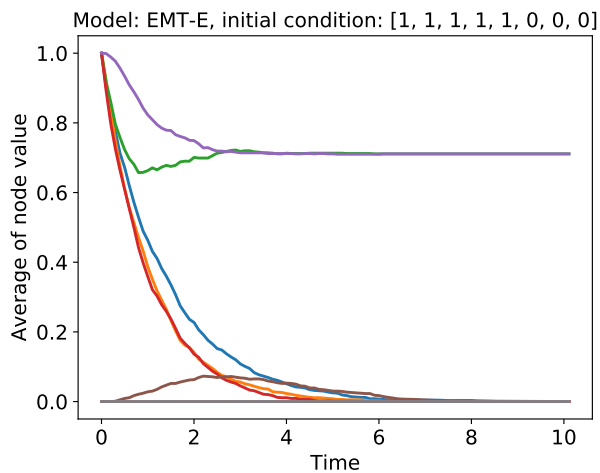

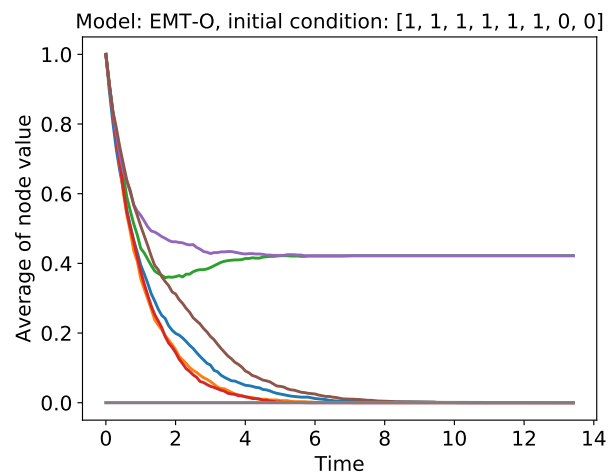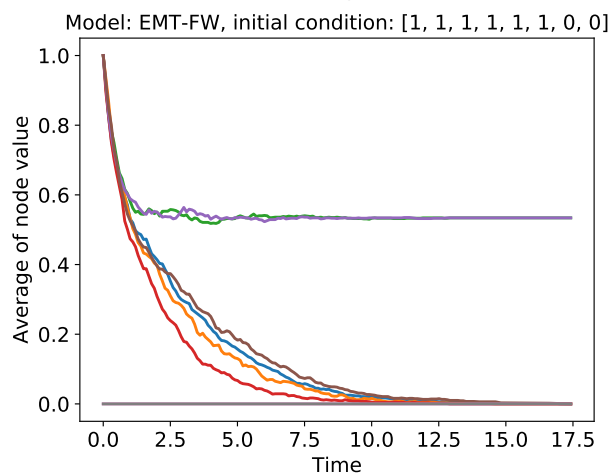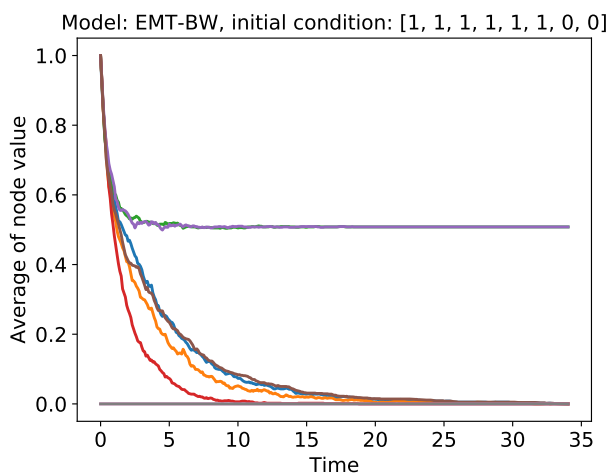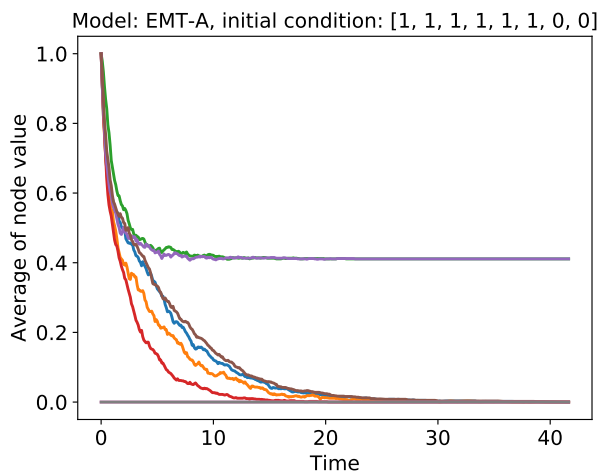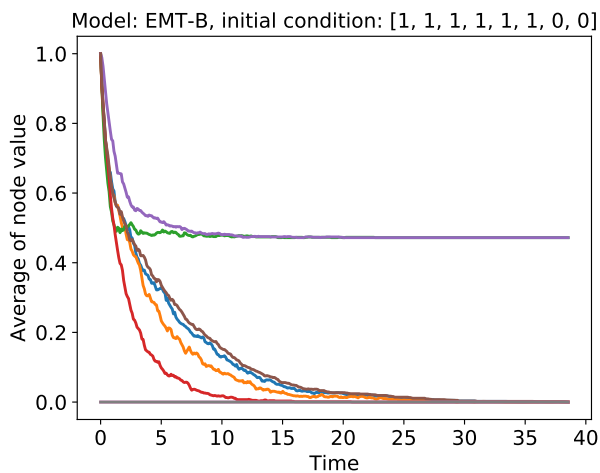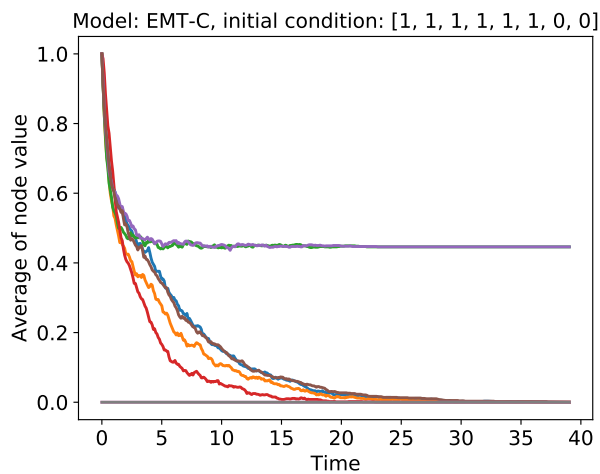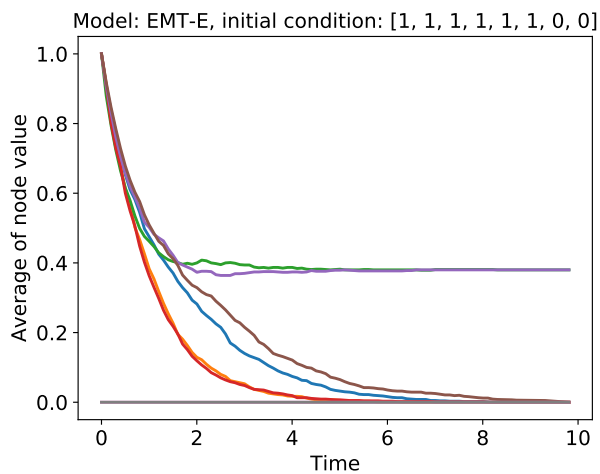

Supplement: S3 File — (PDF) [file pcbi.1009035.s003.pdf]

# Parameter set (ECM, DNAdam) = (0, 1):

Steady state: (0, 0, 1, 0, 1, 0, 0, 1)

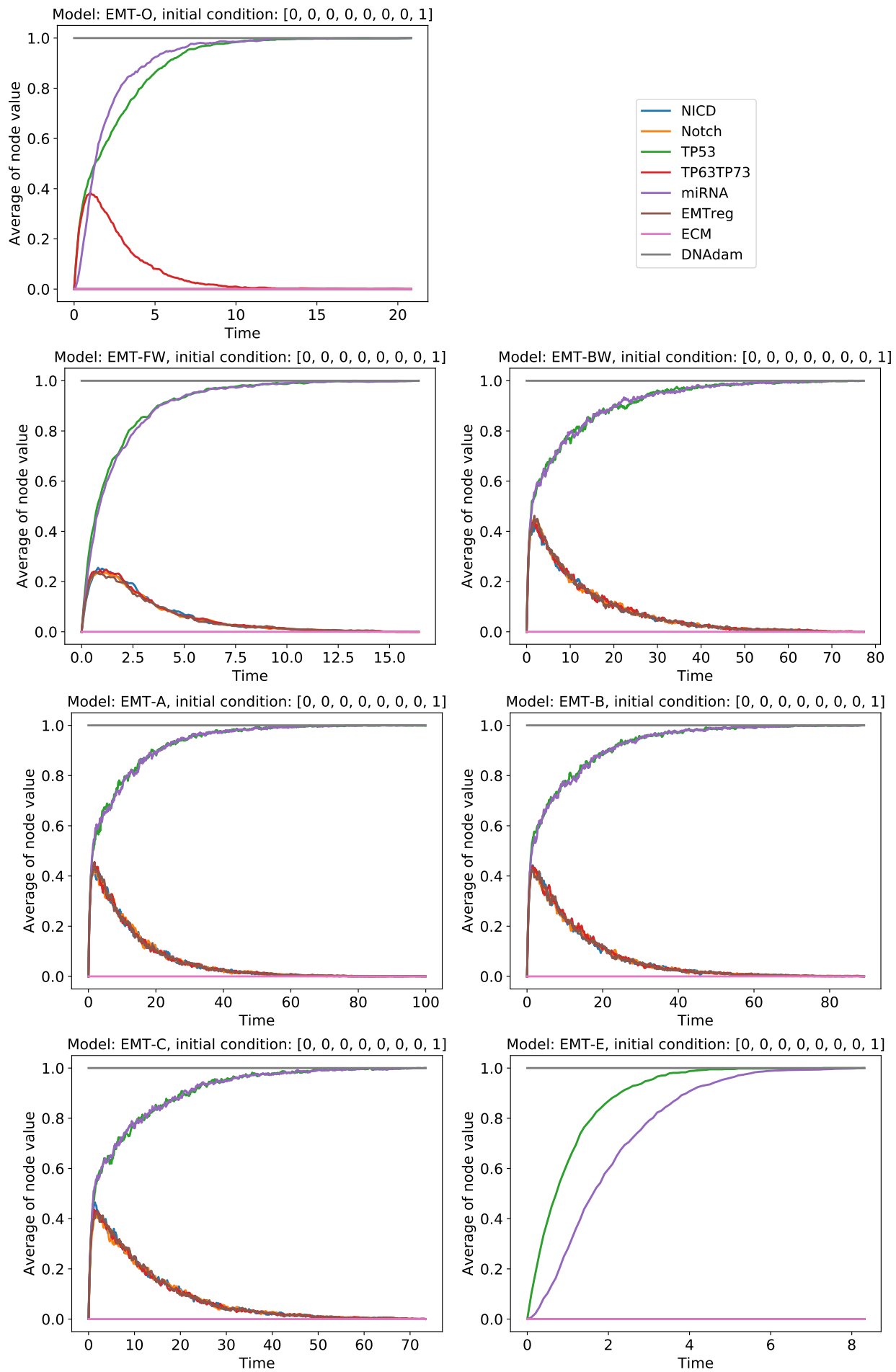

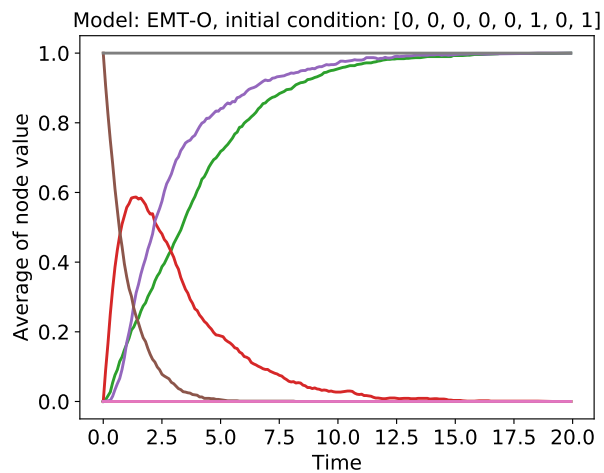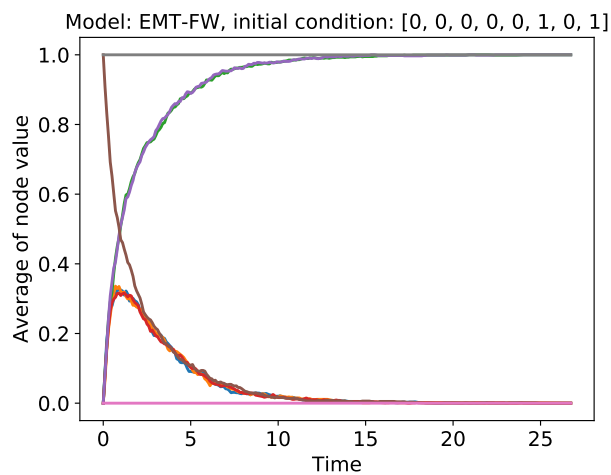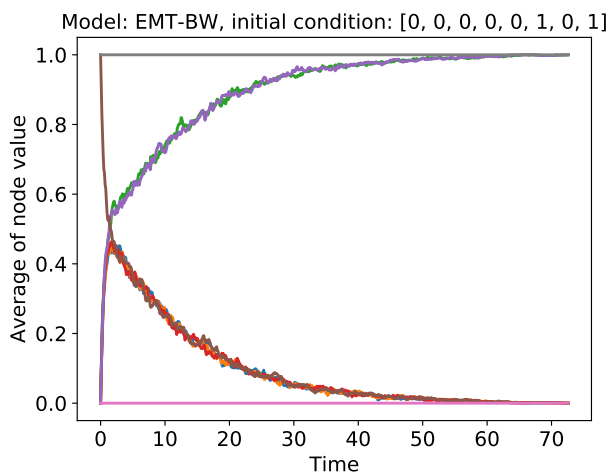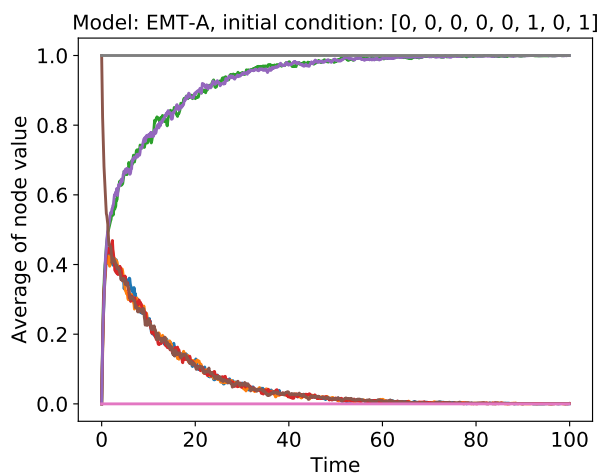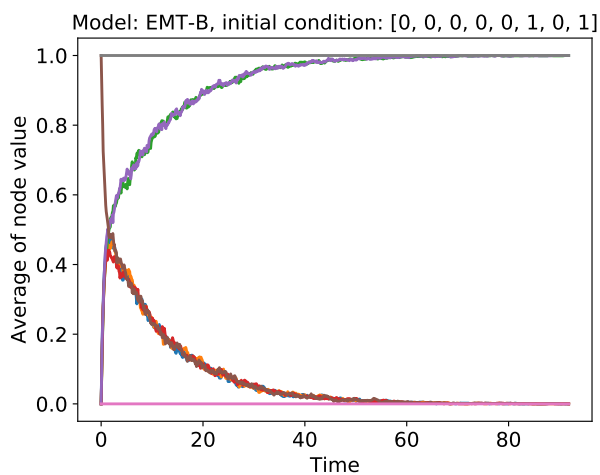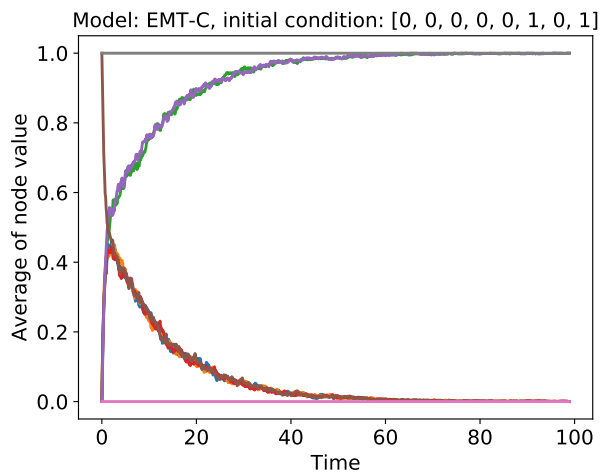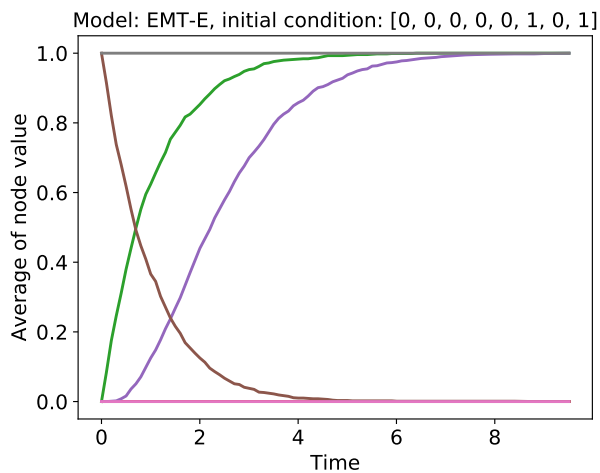

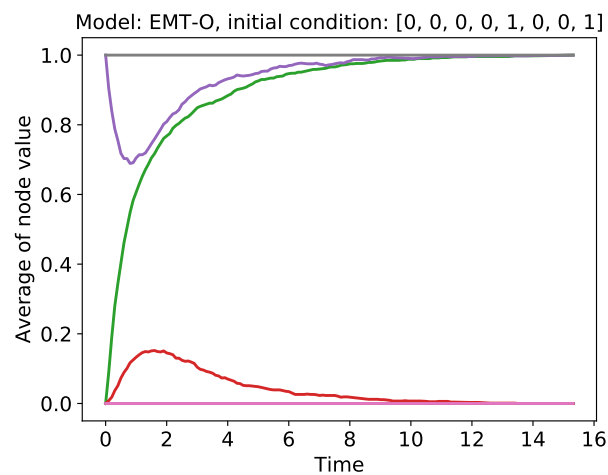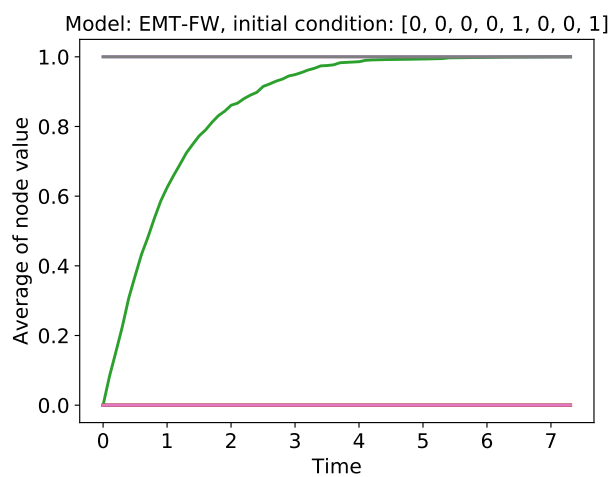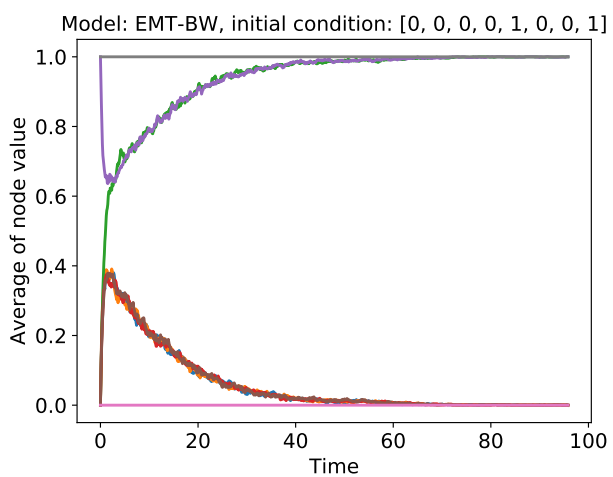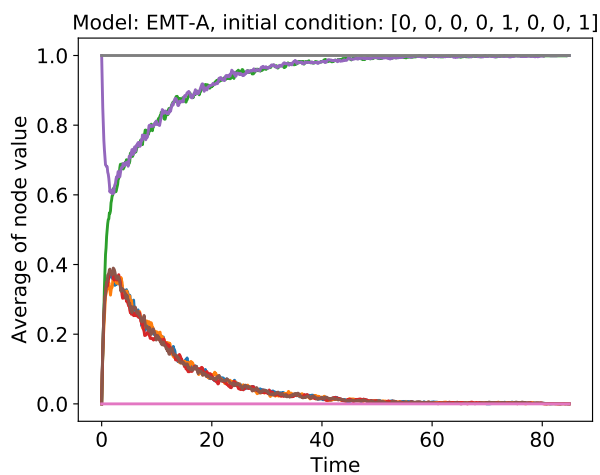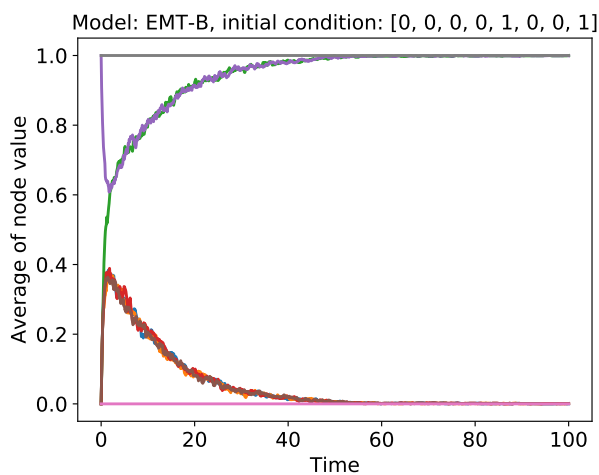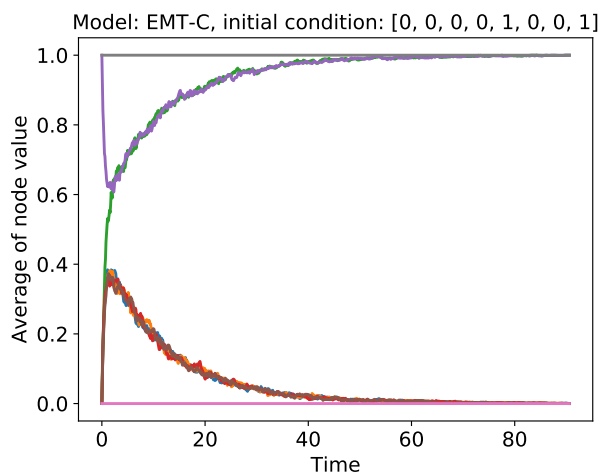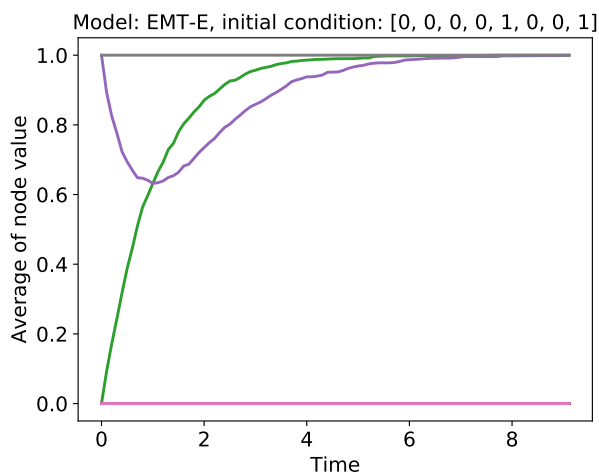

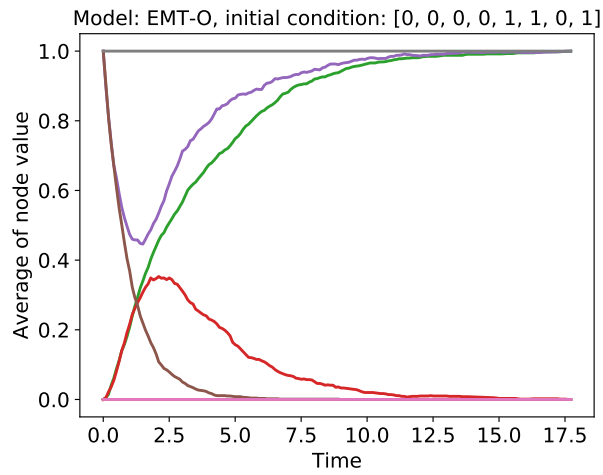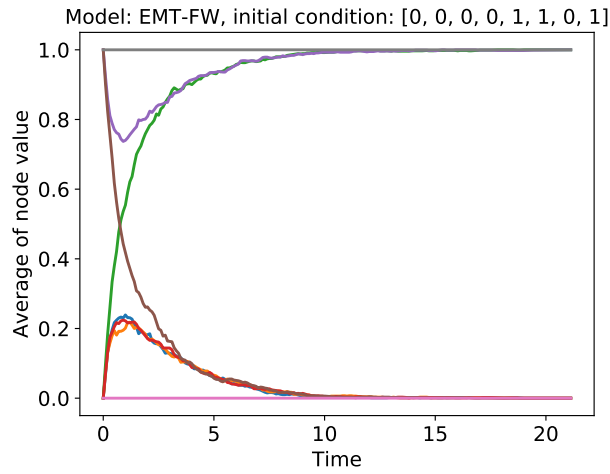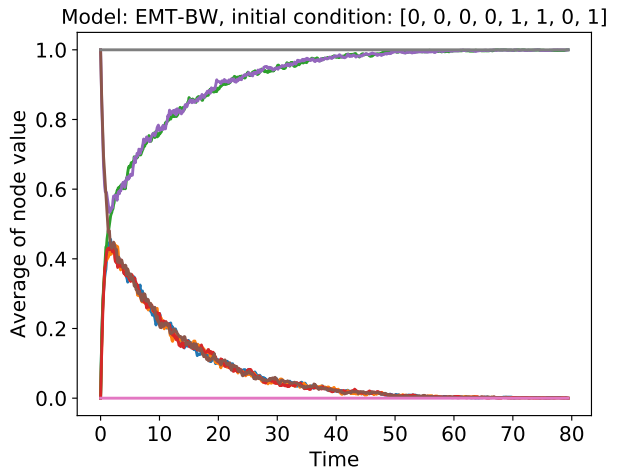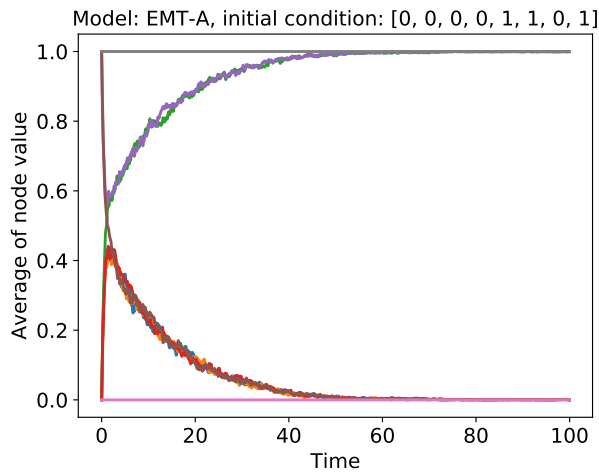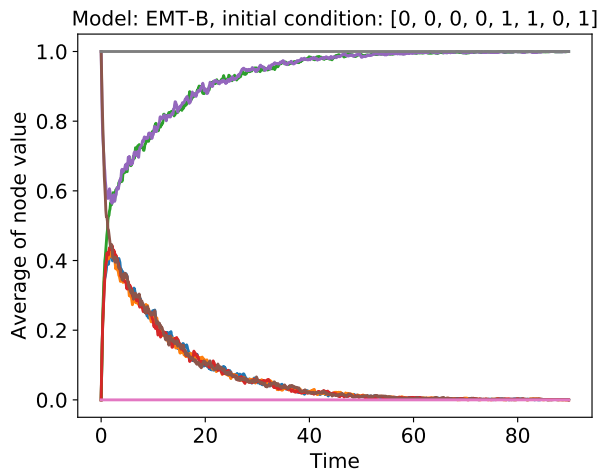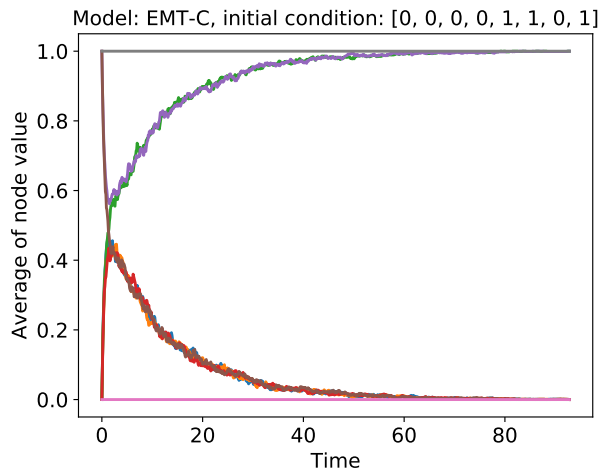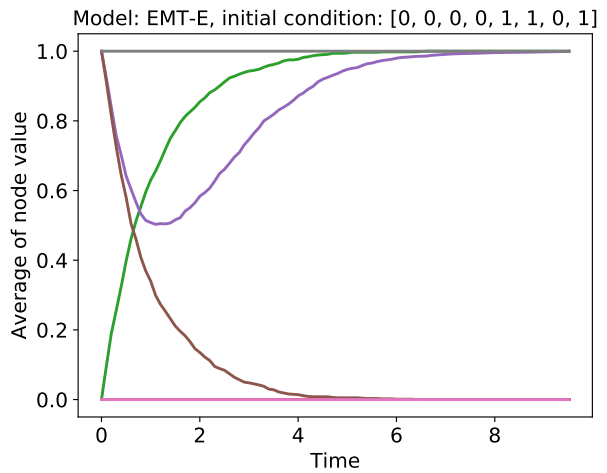

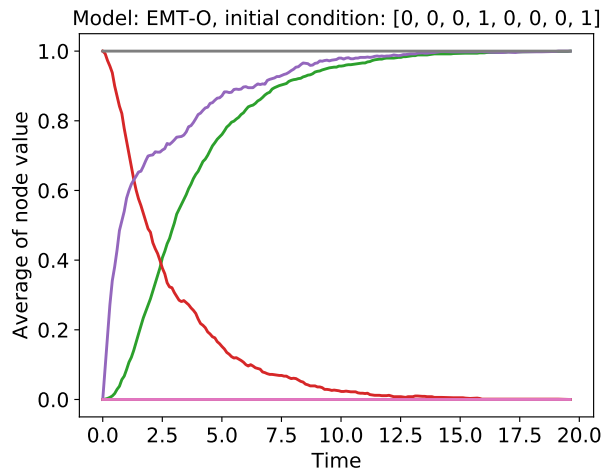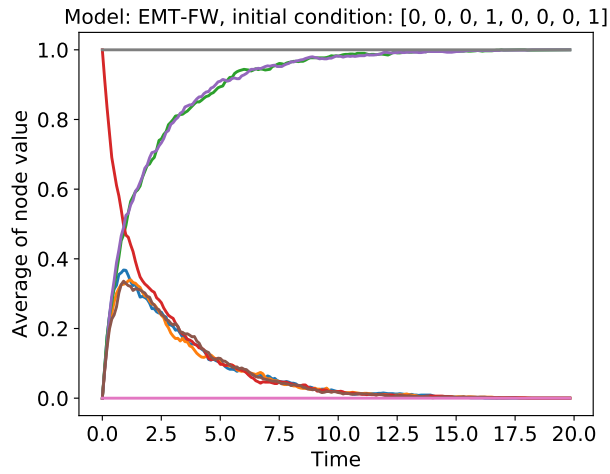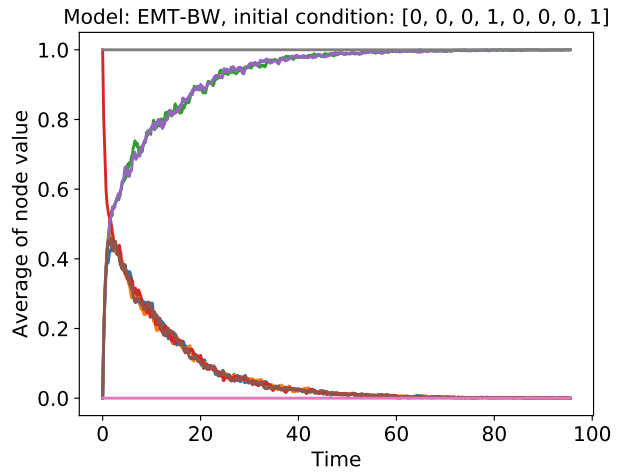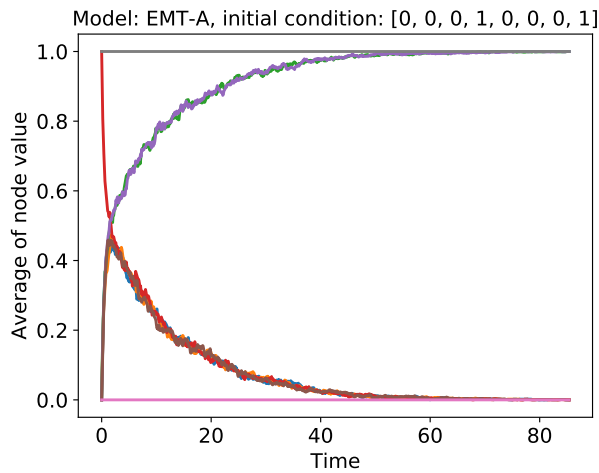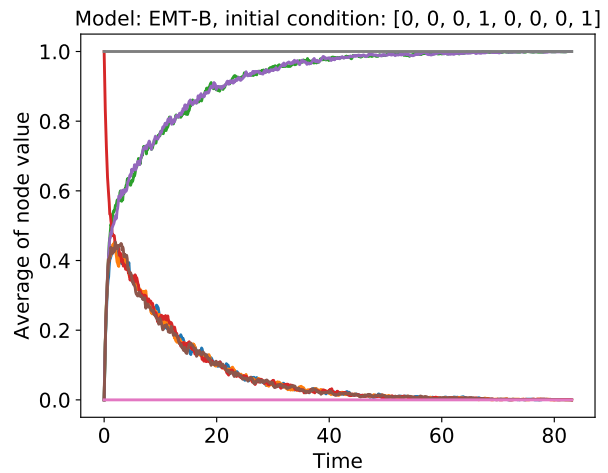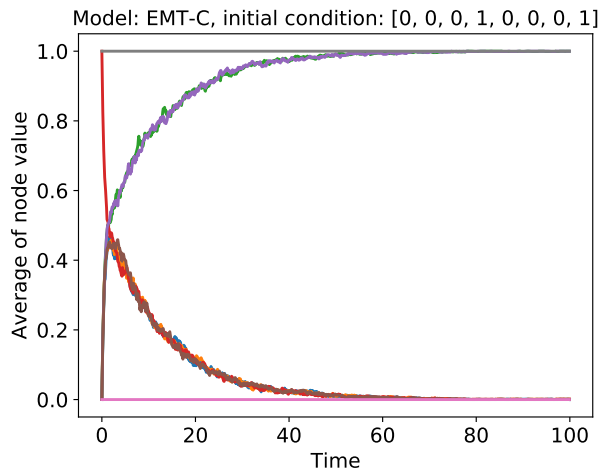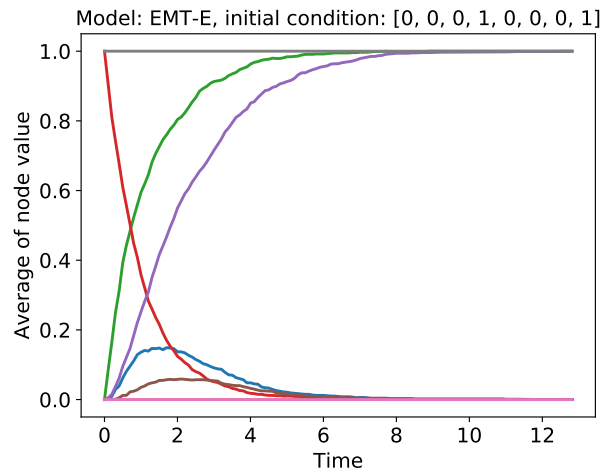

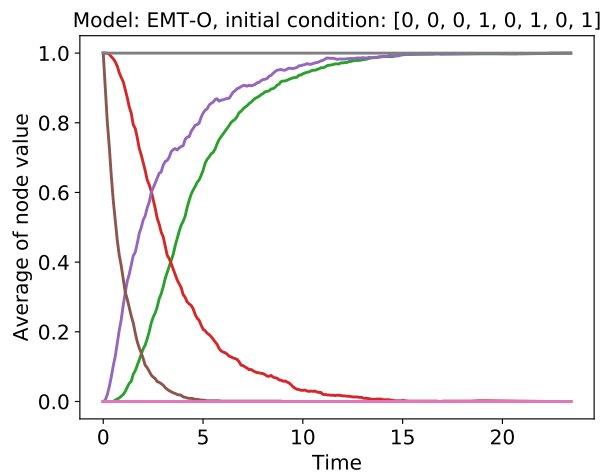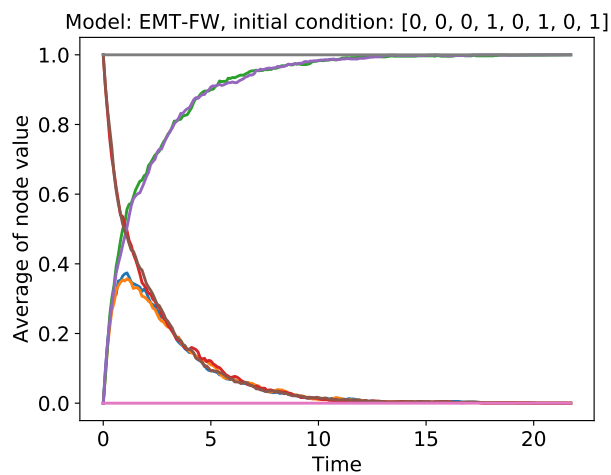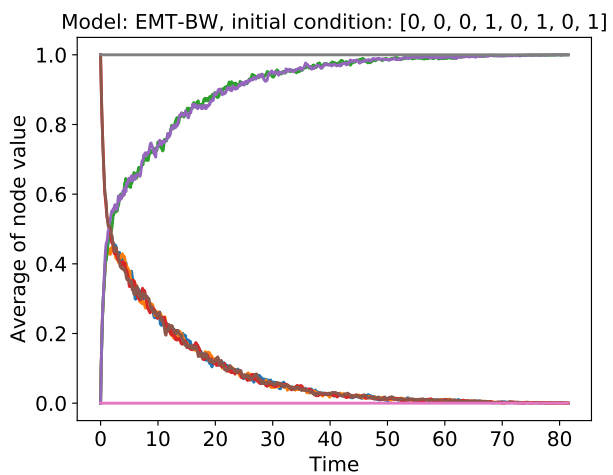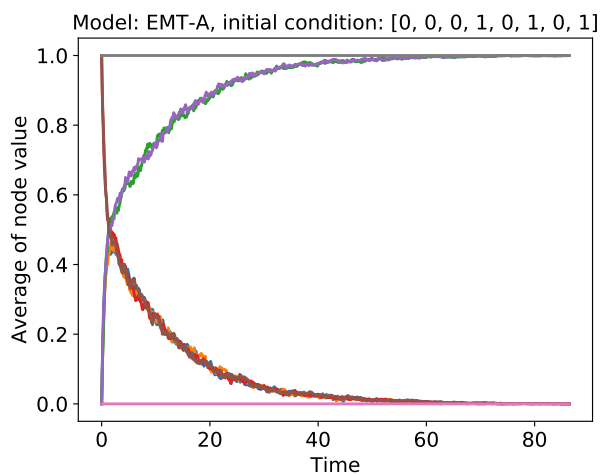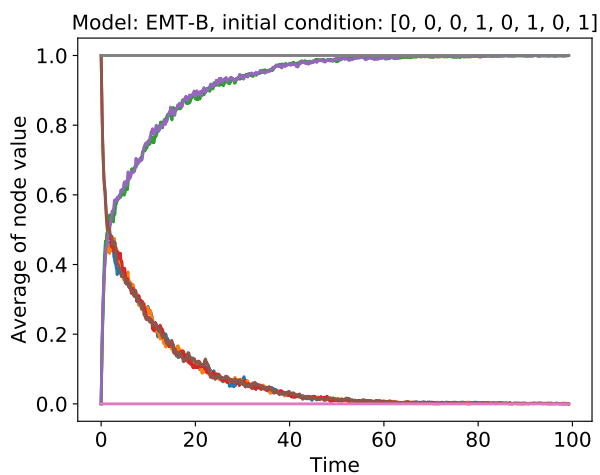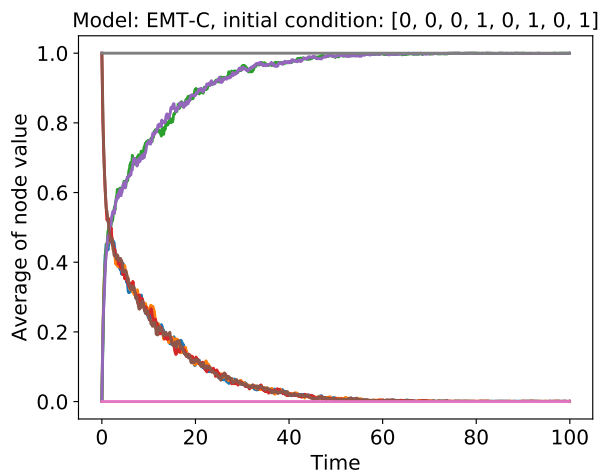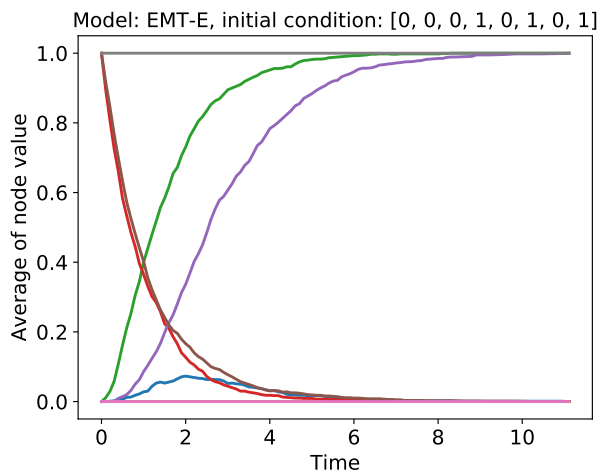

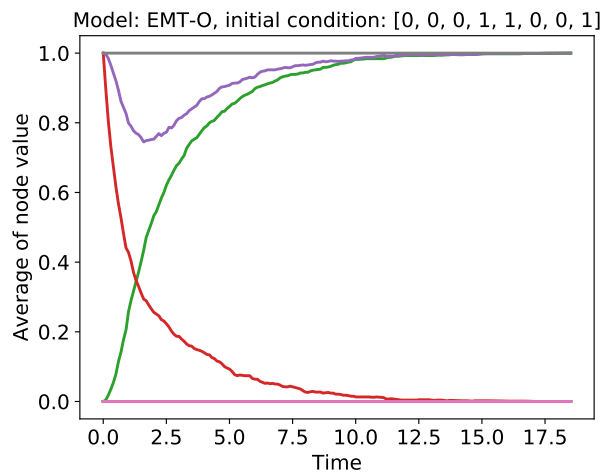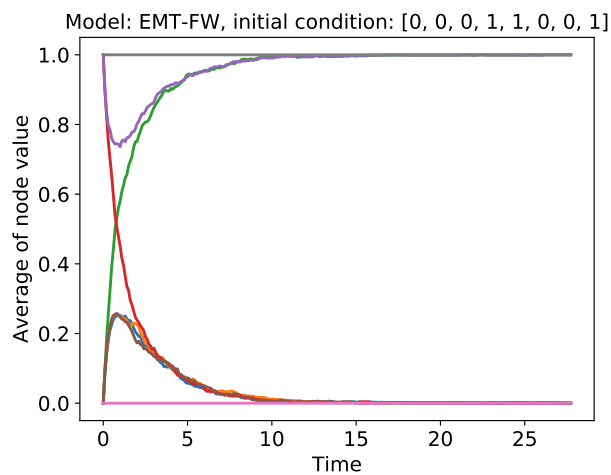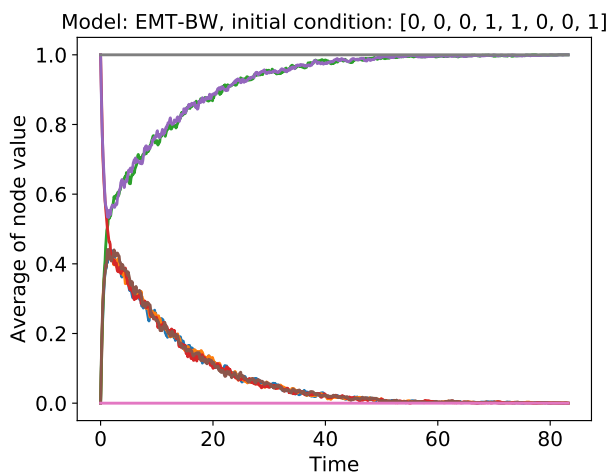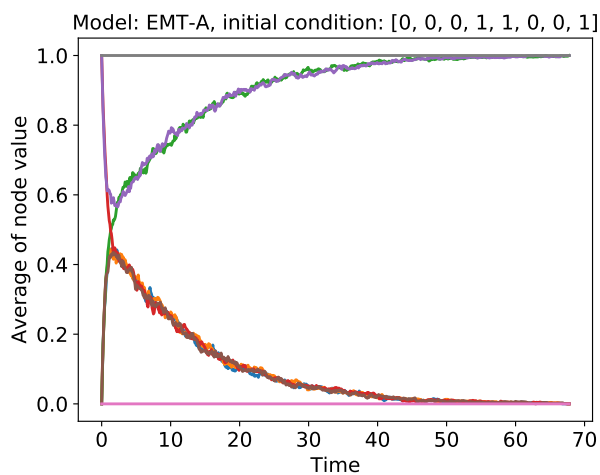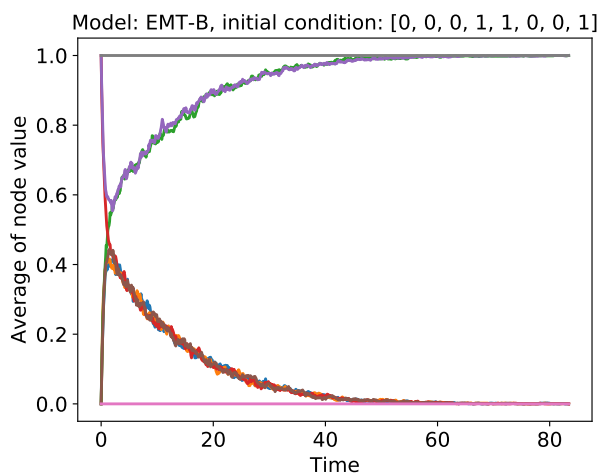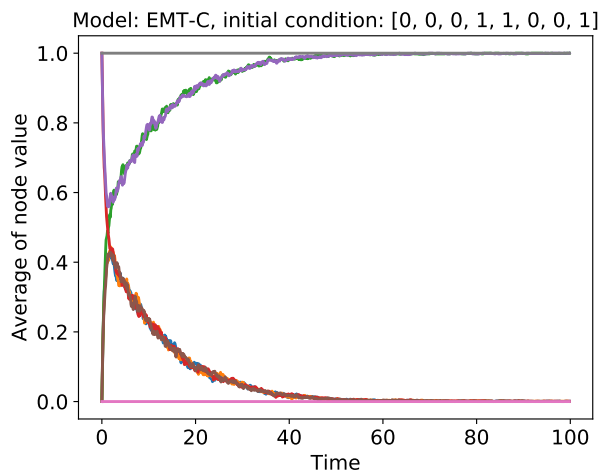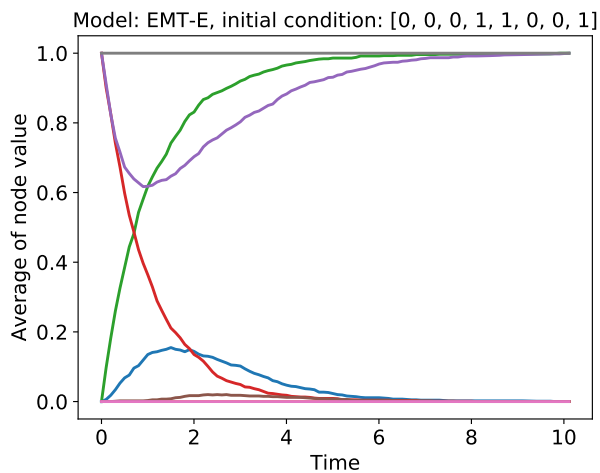

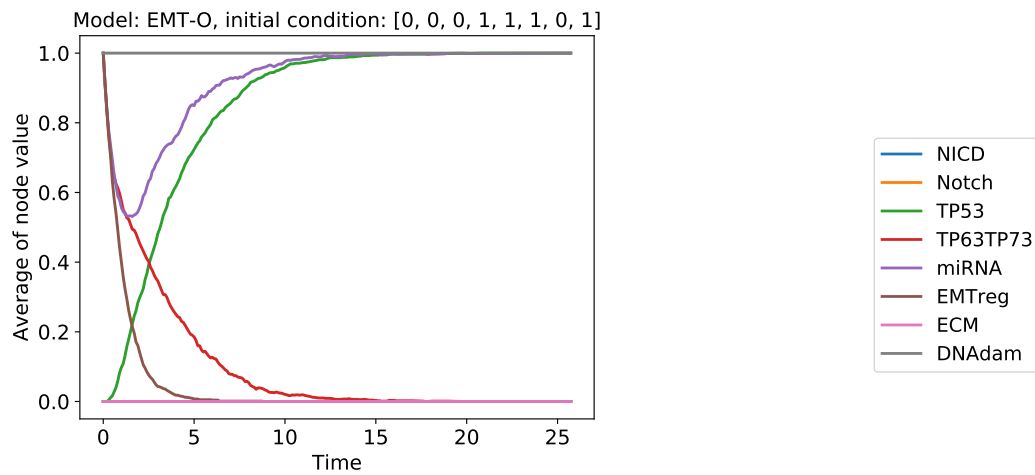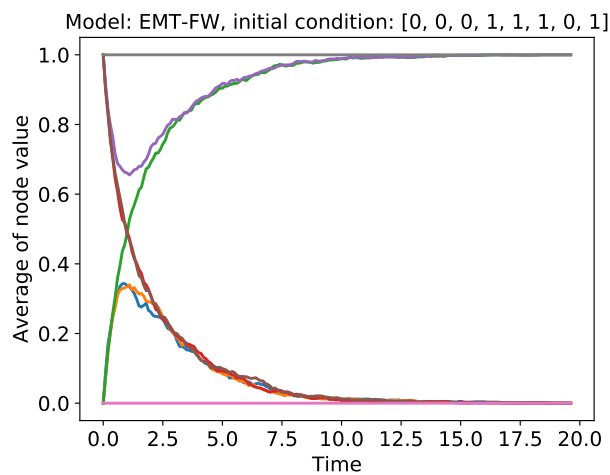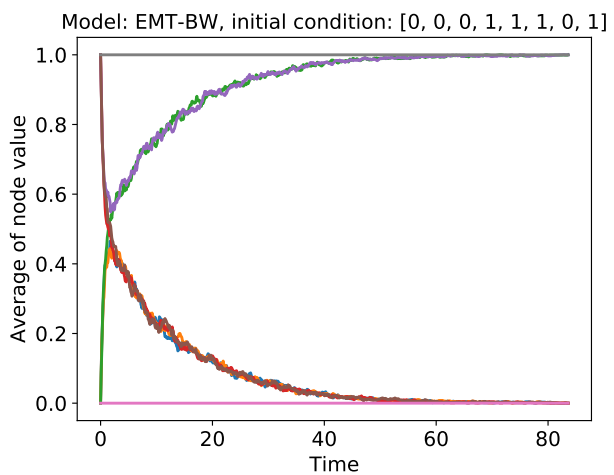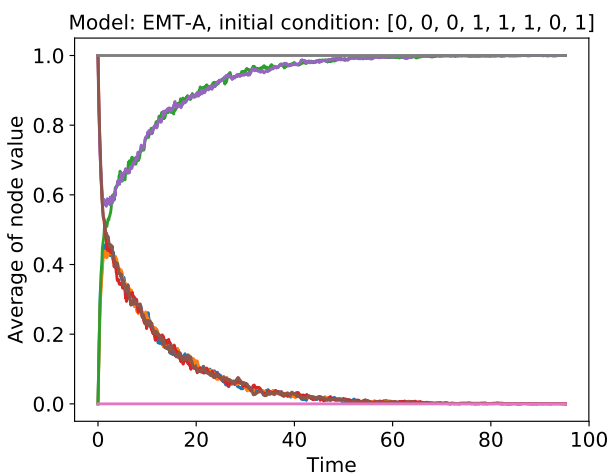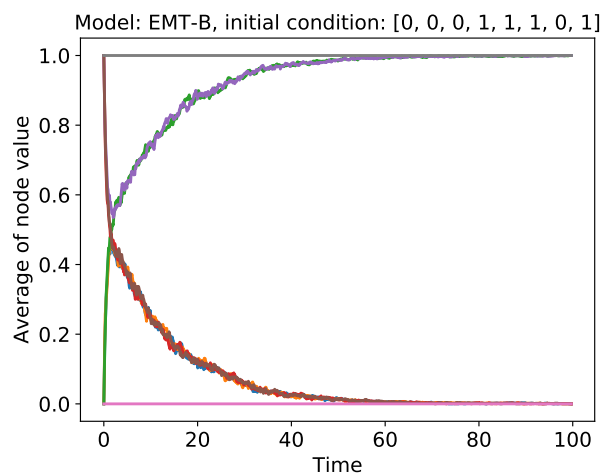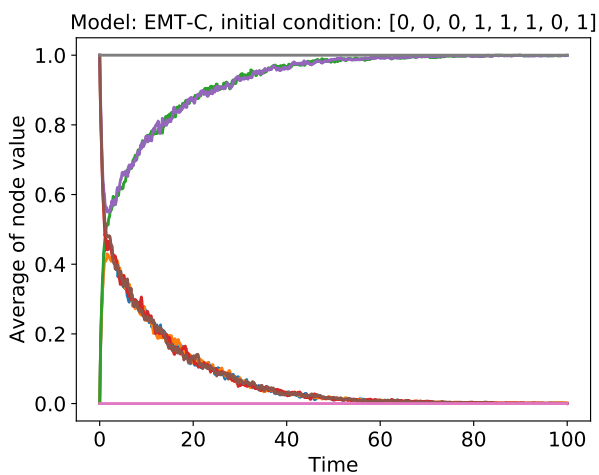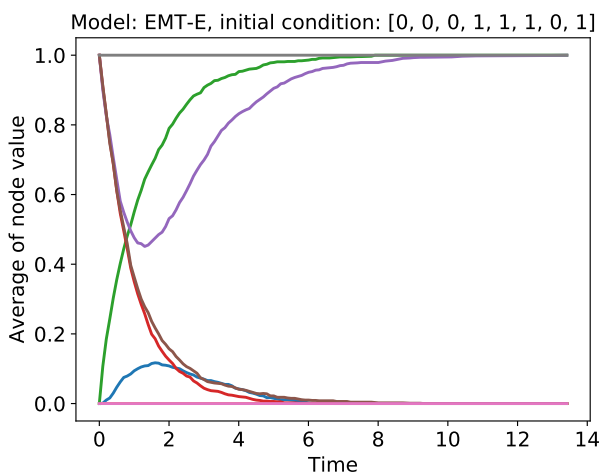

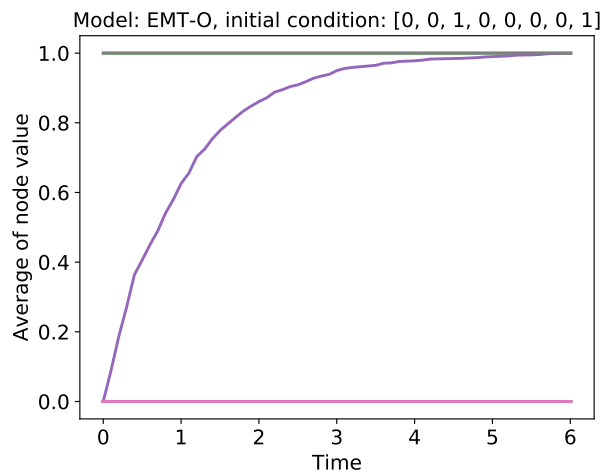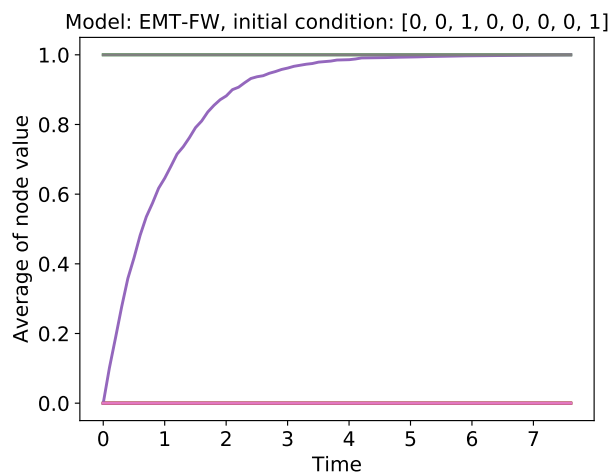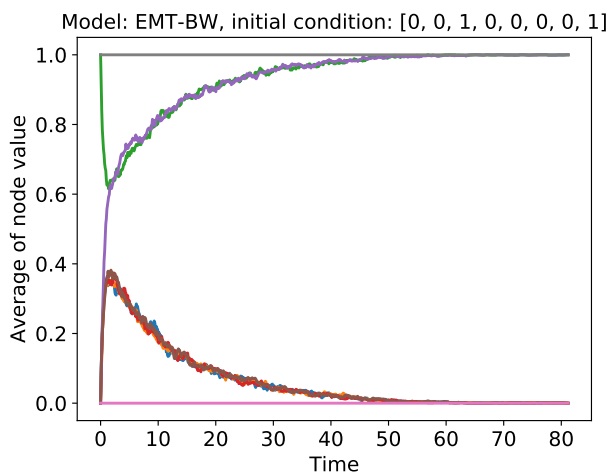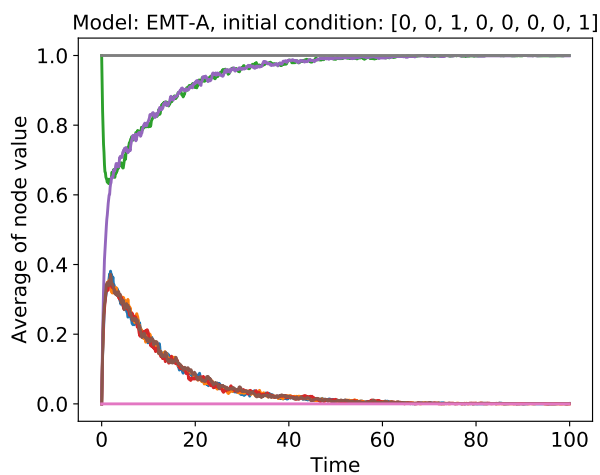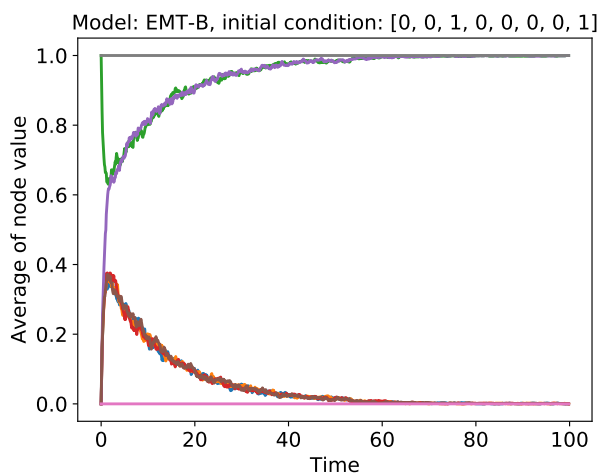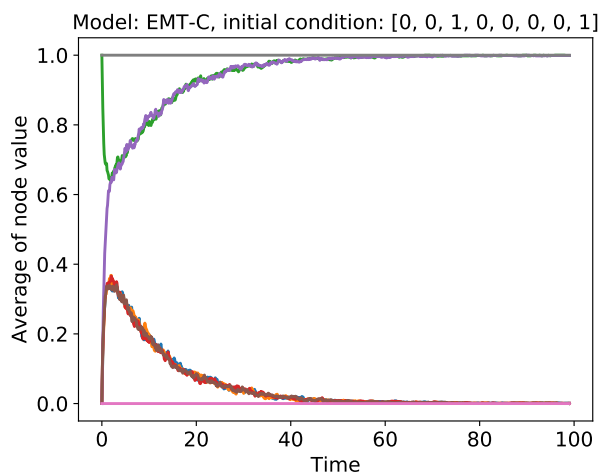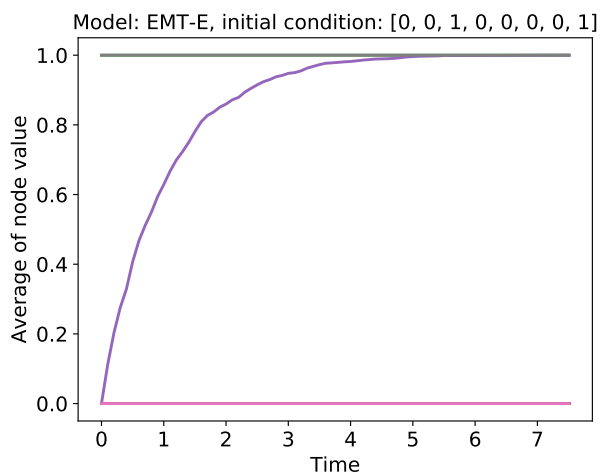

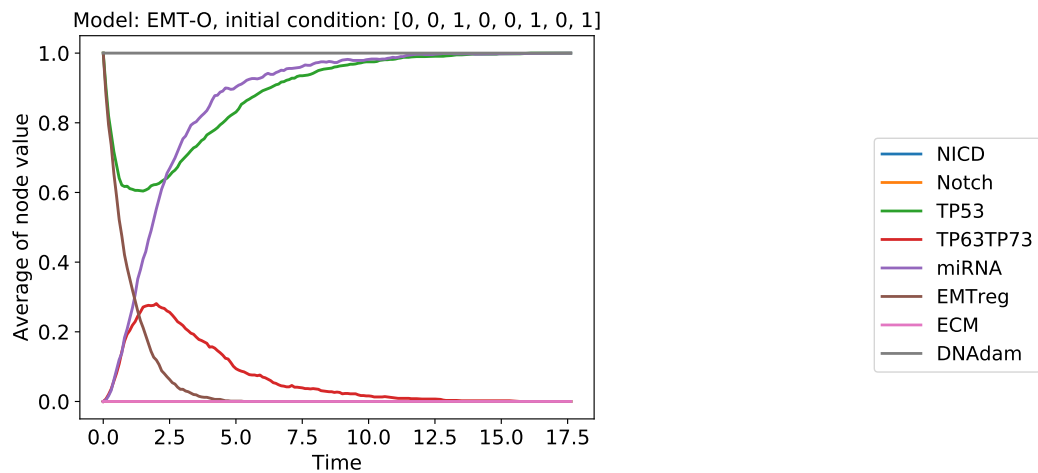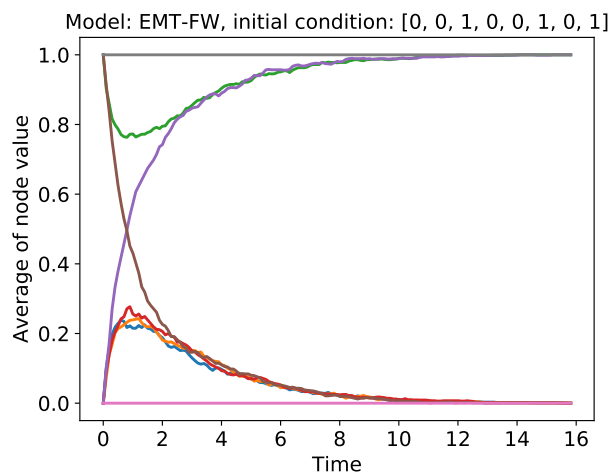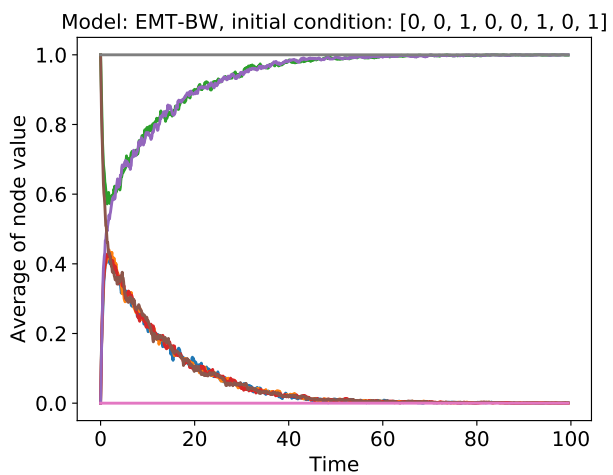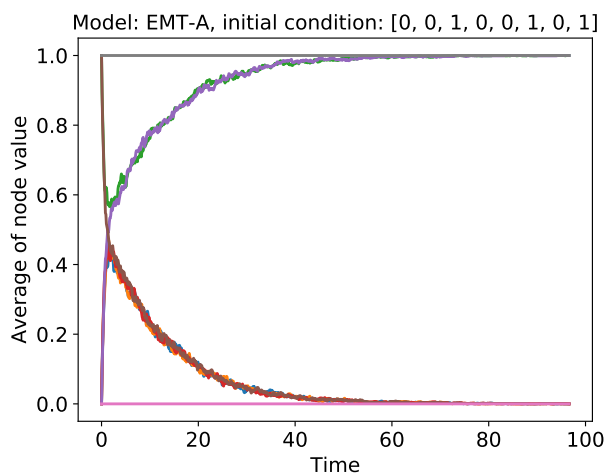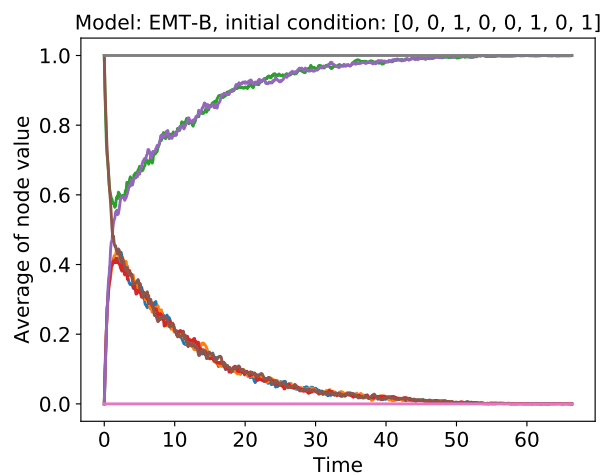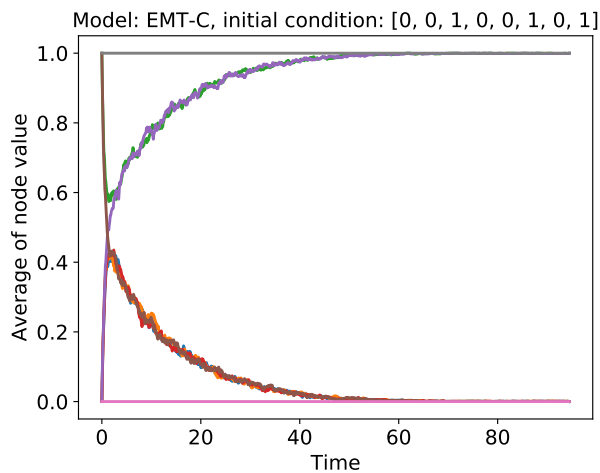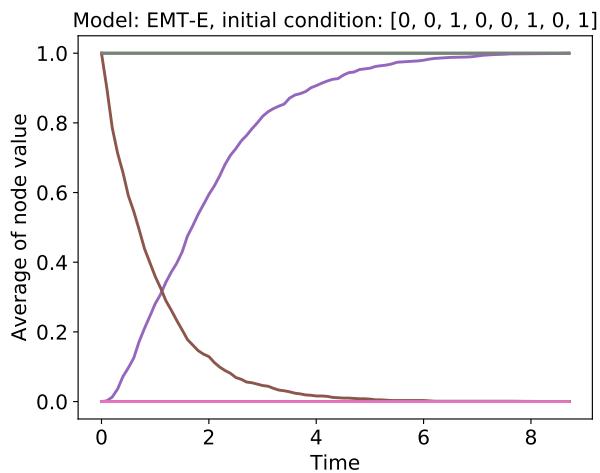

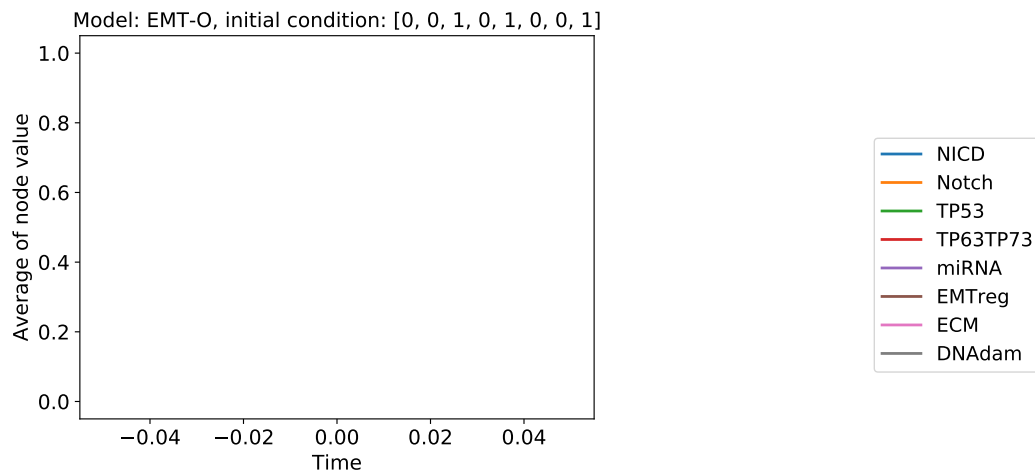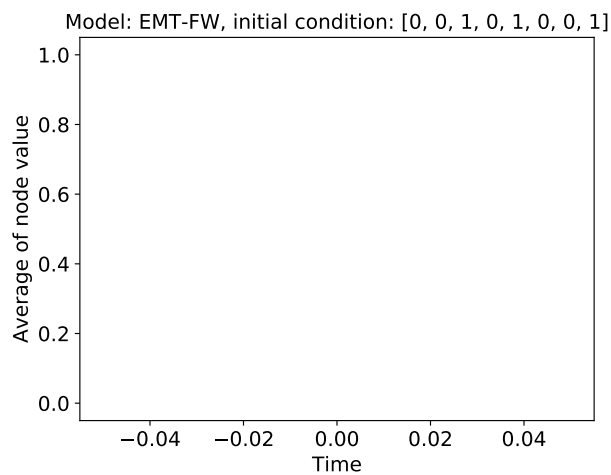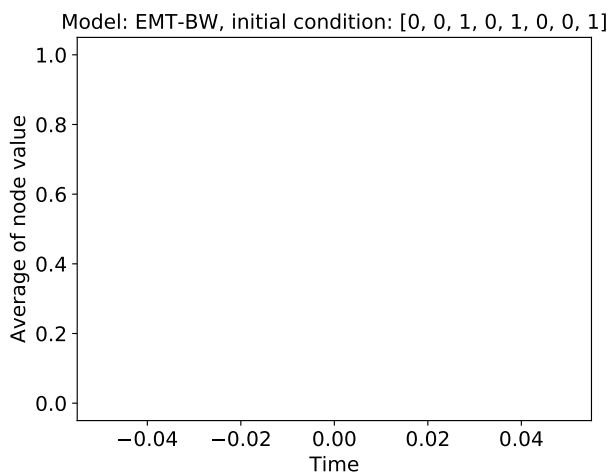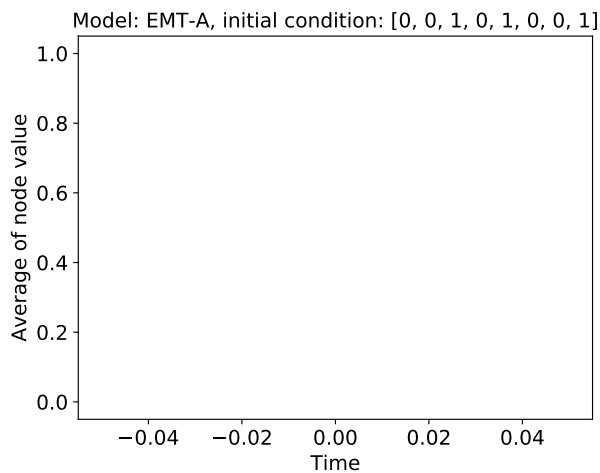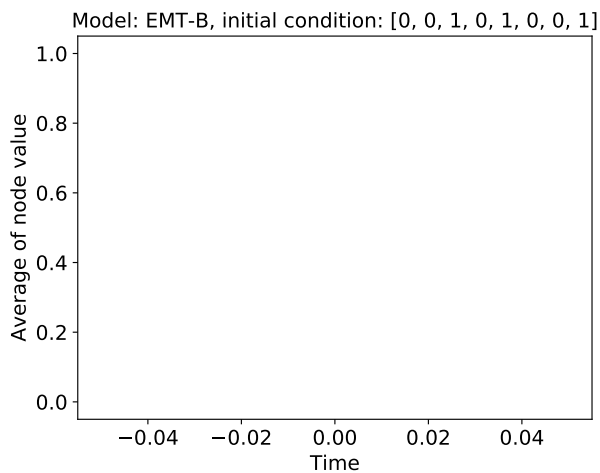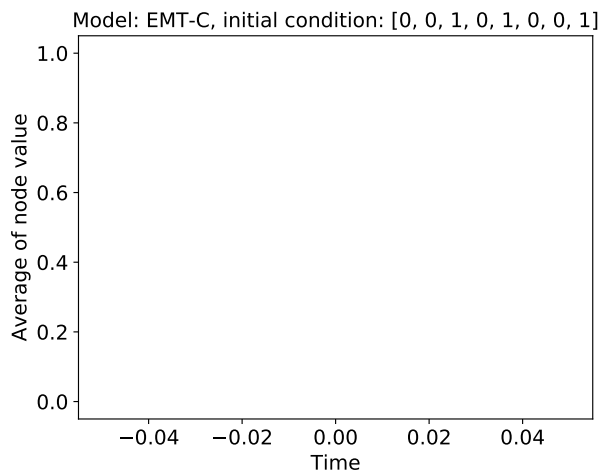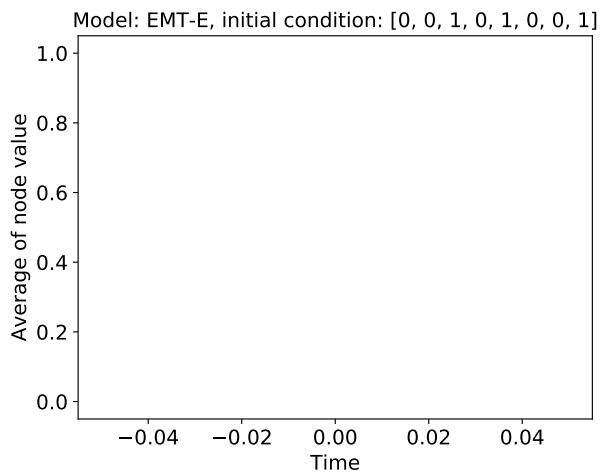

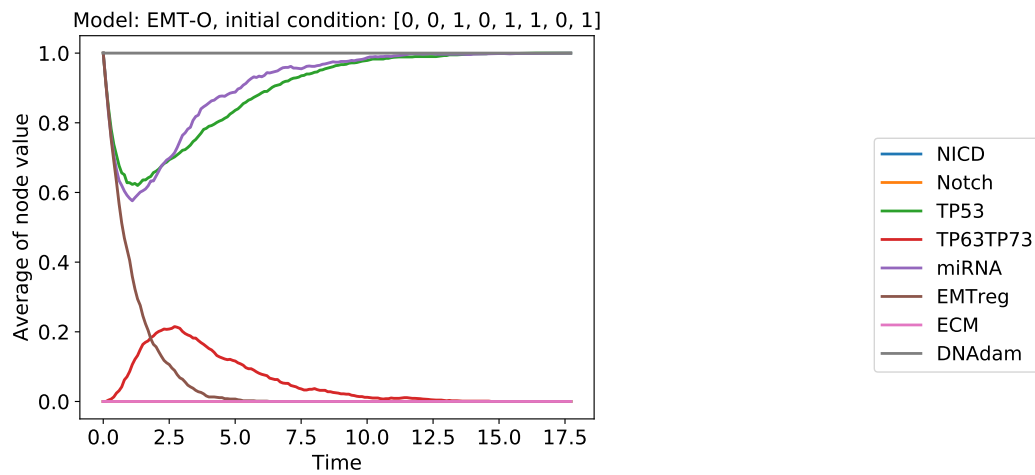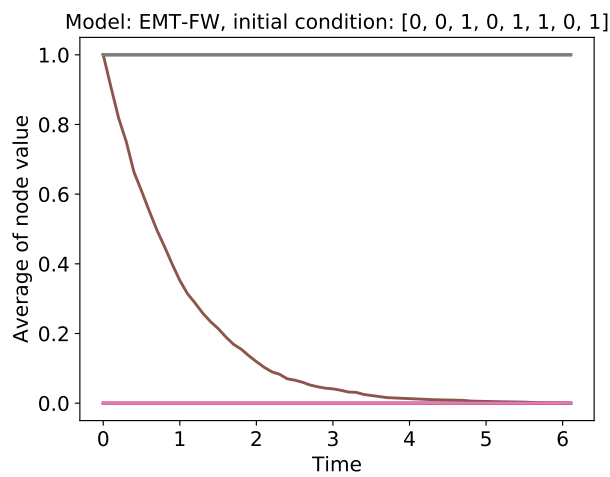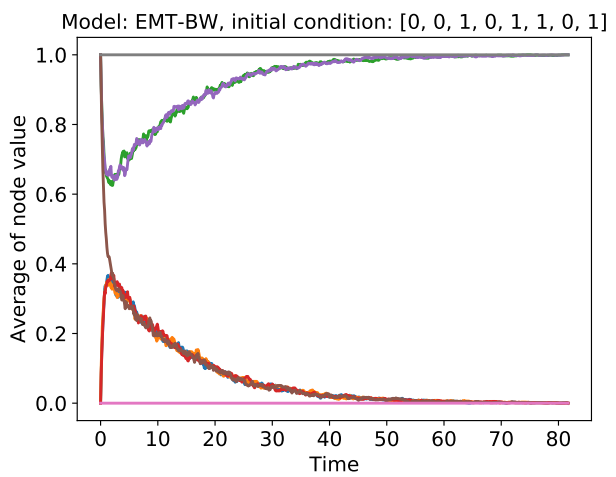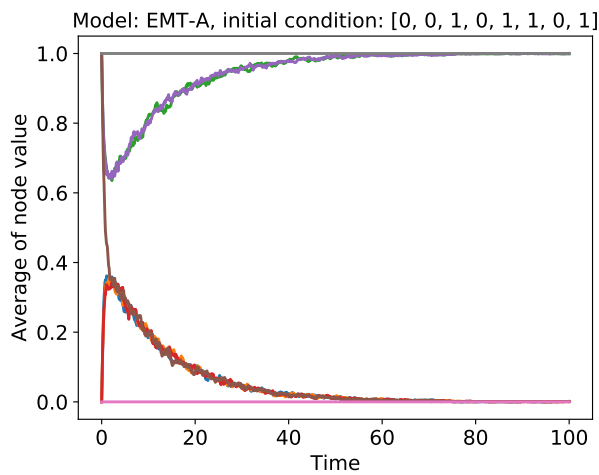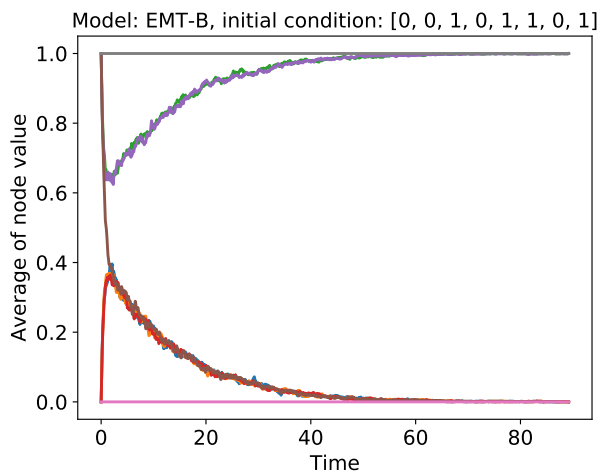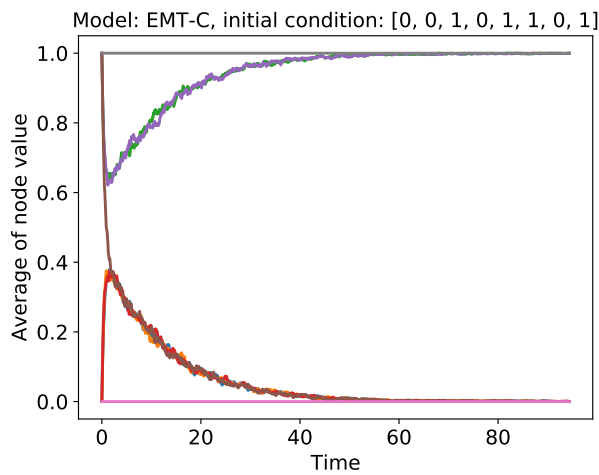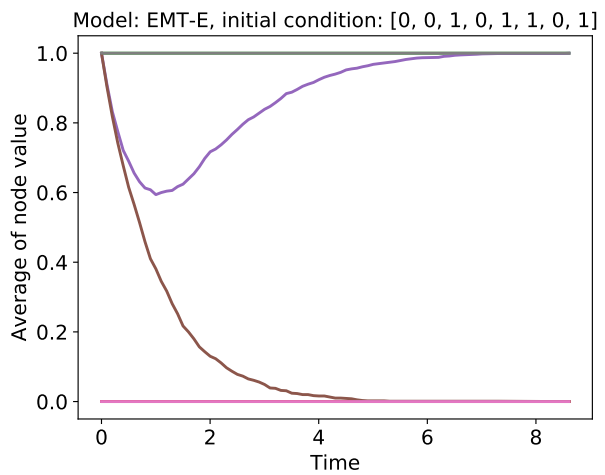

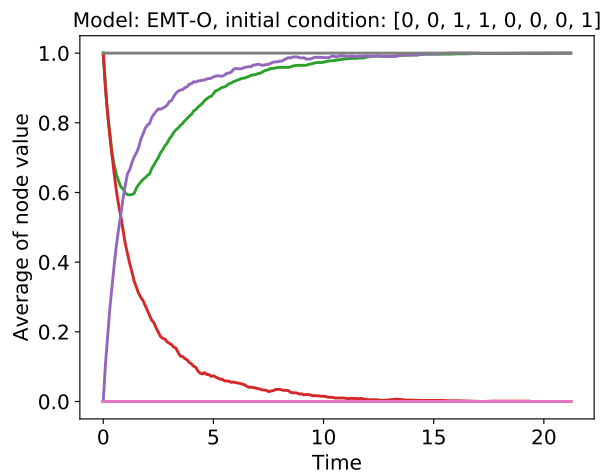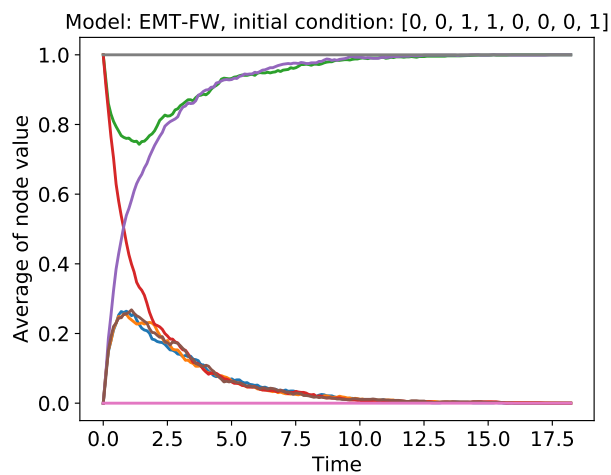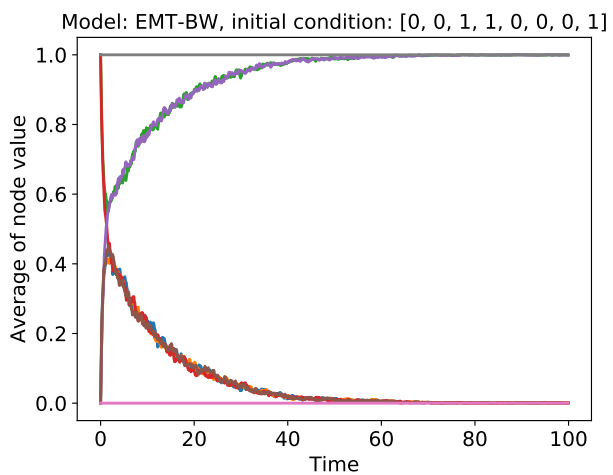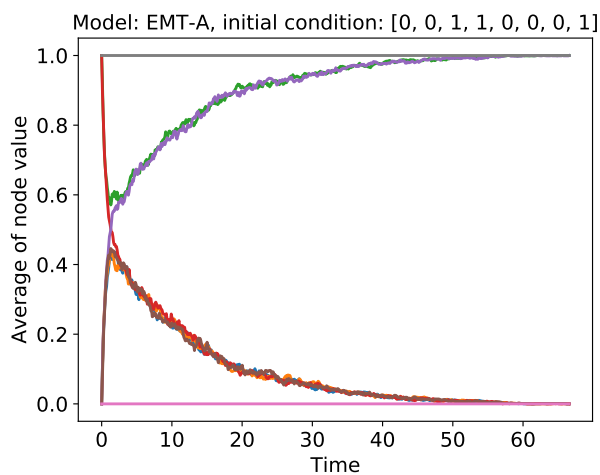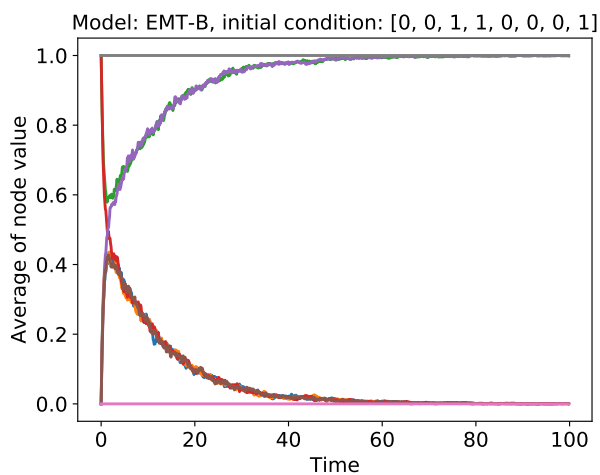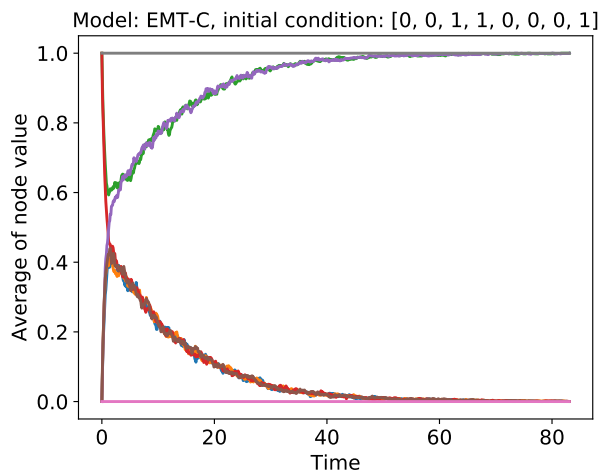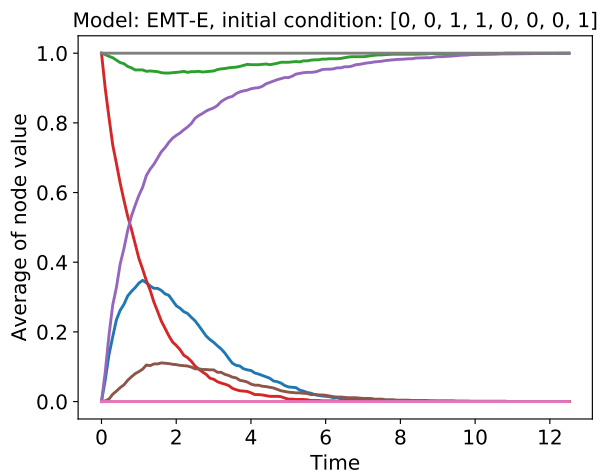

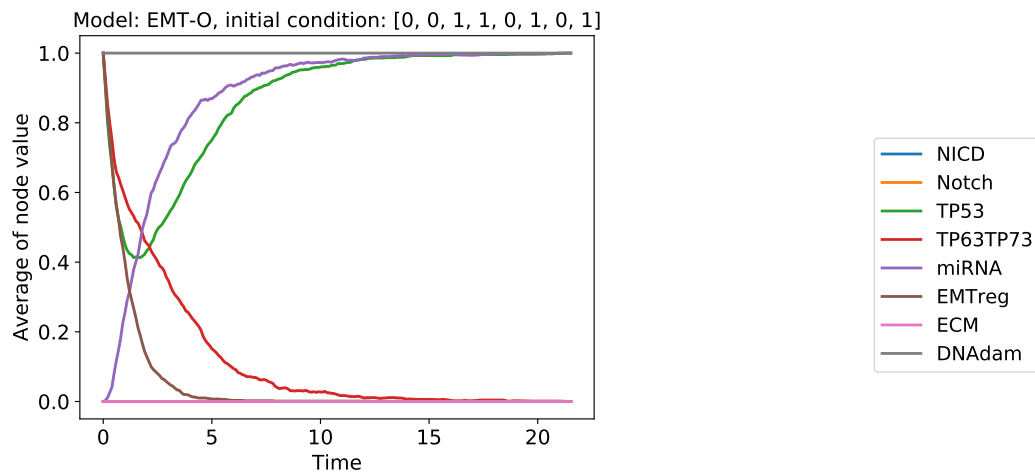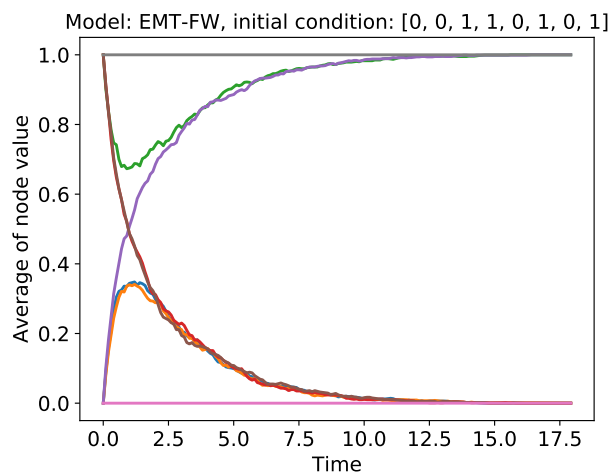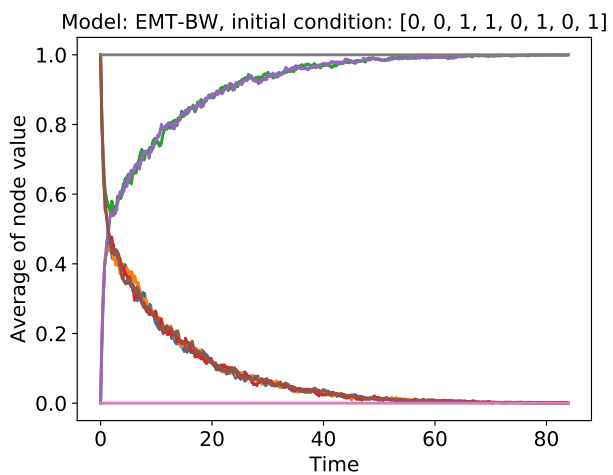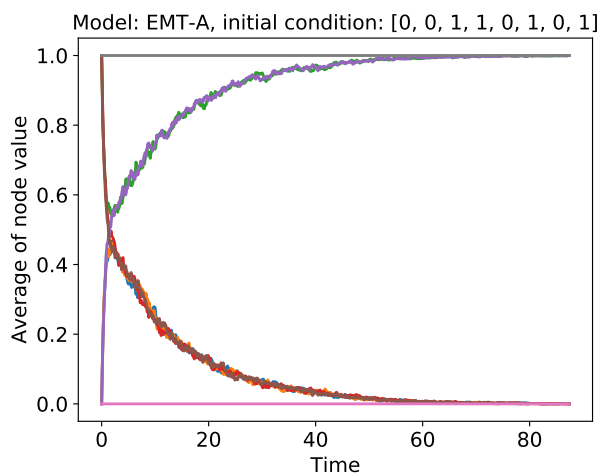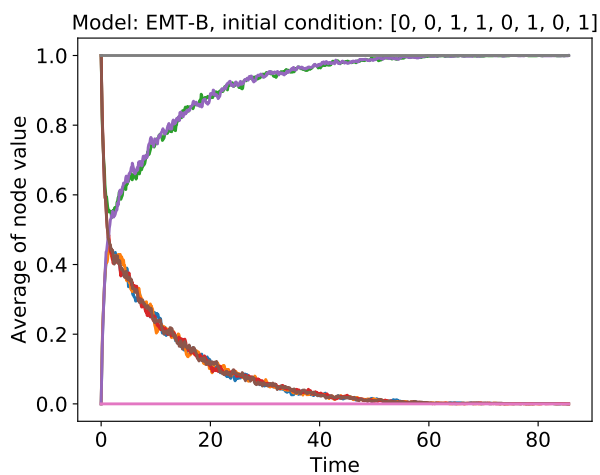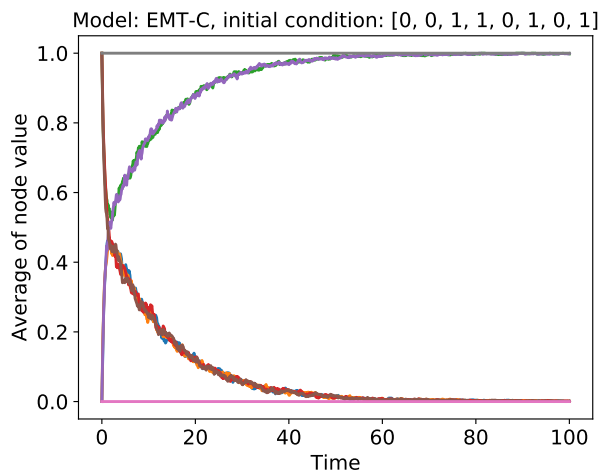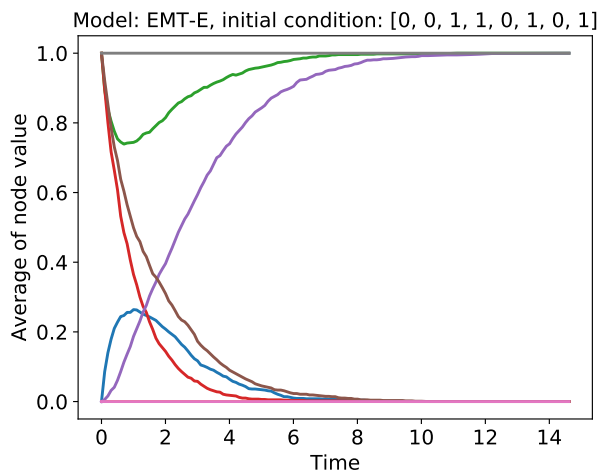

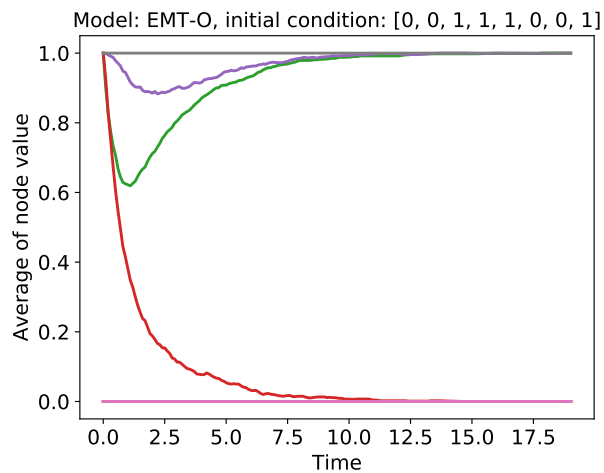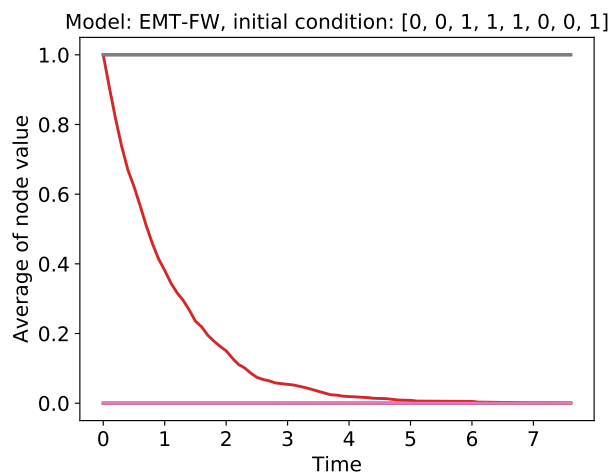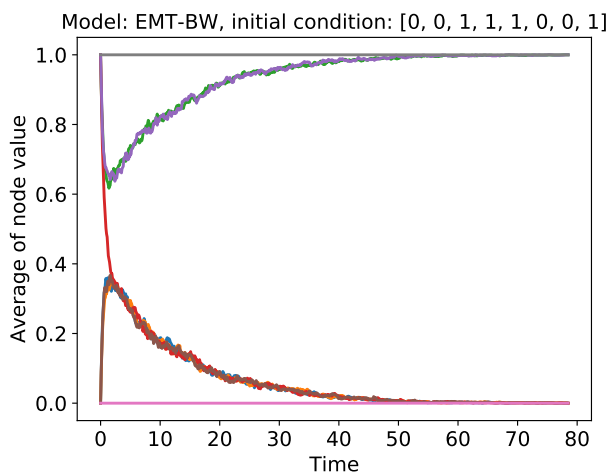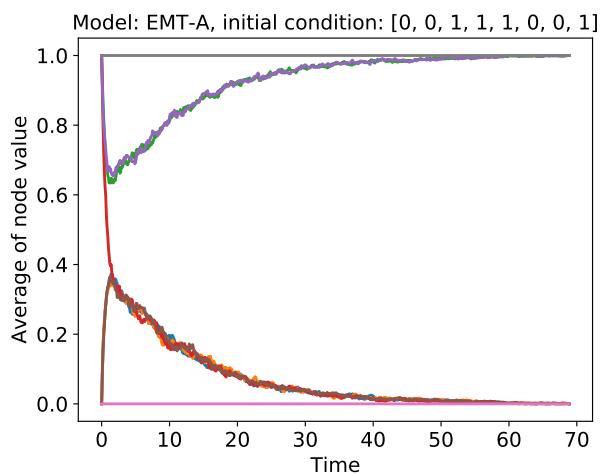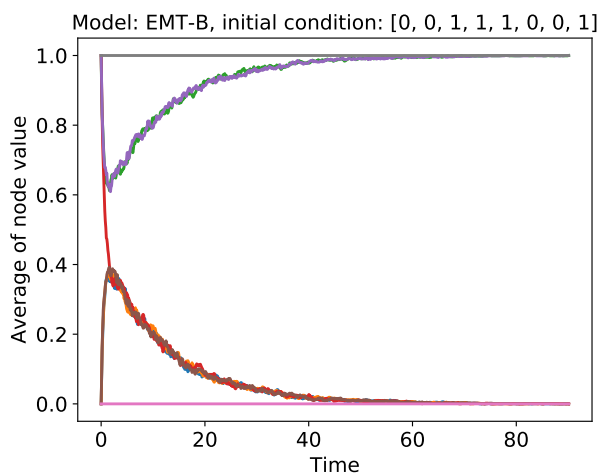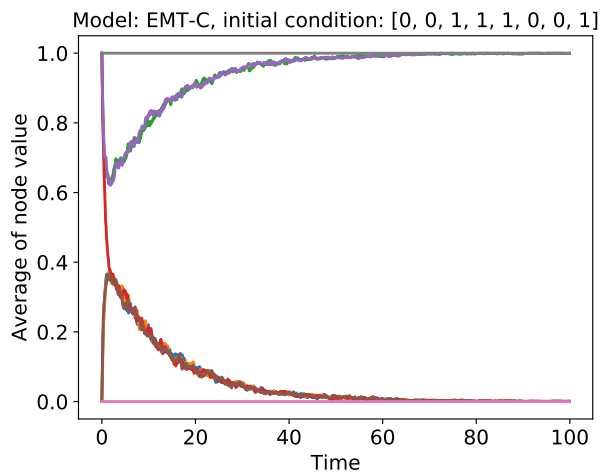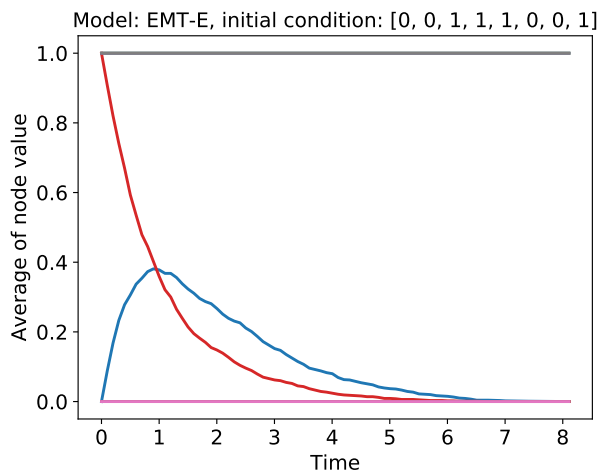

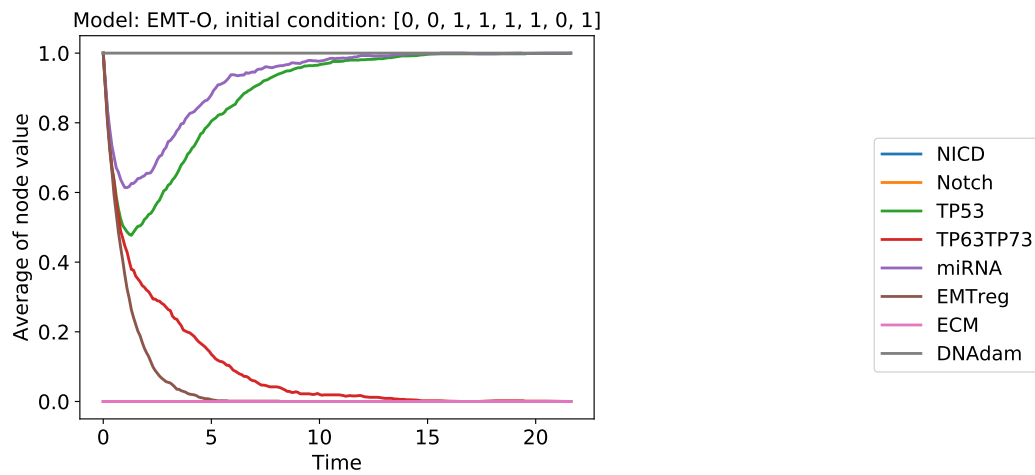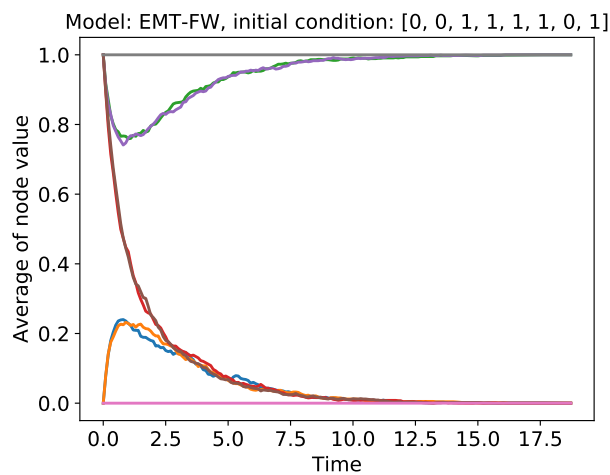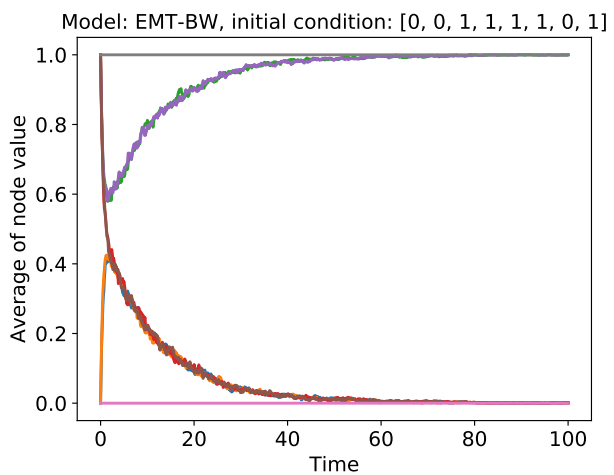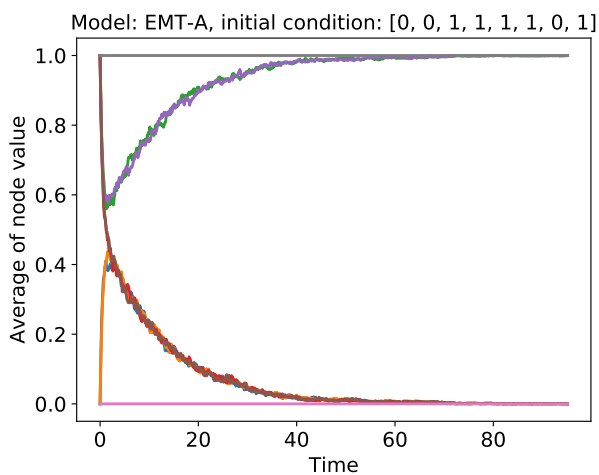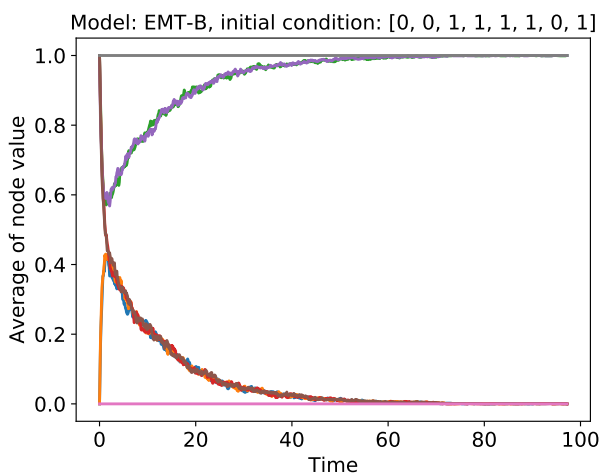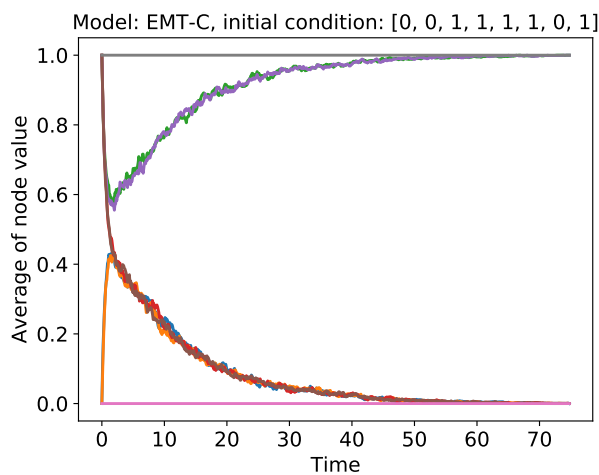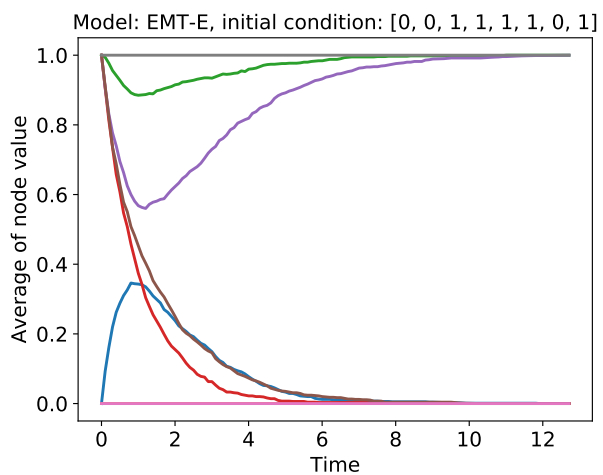

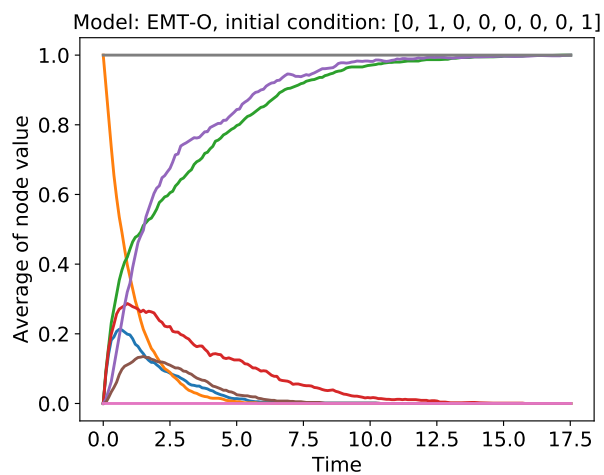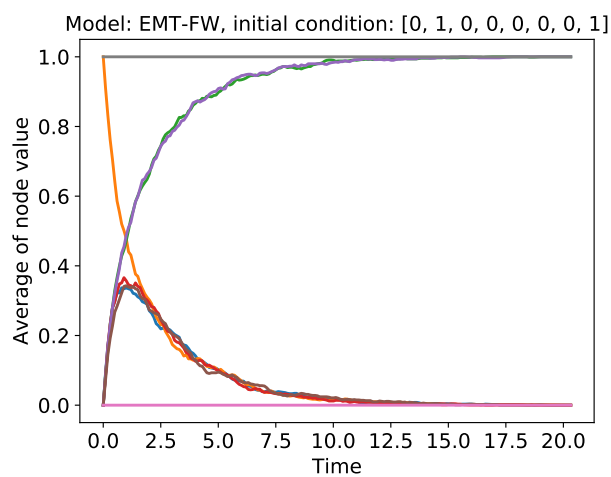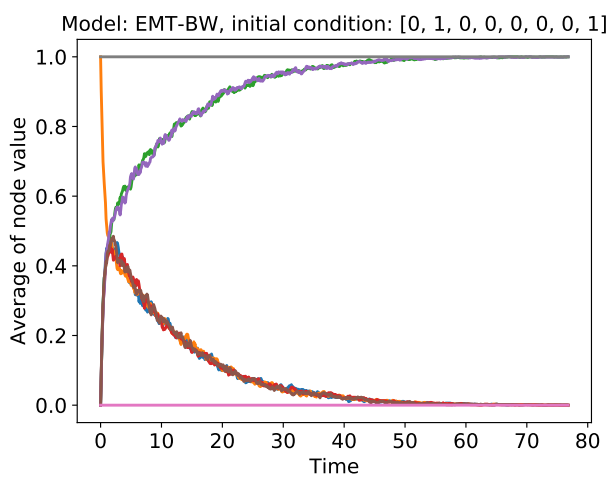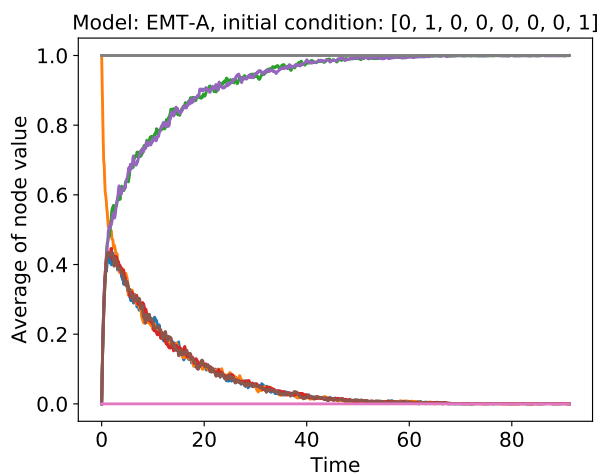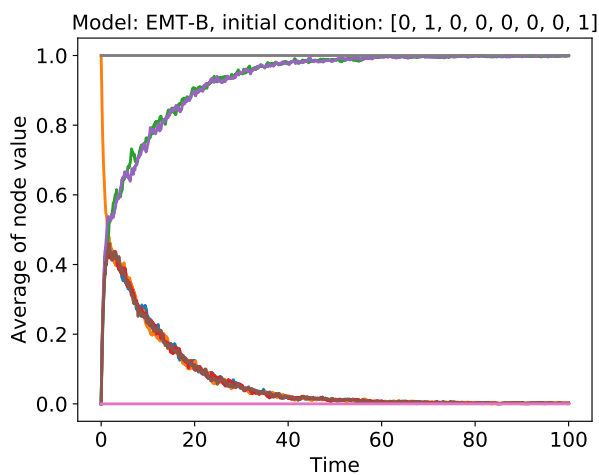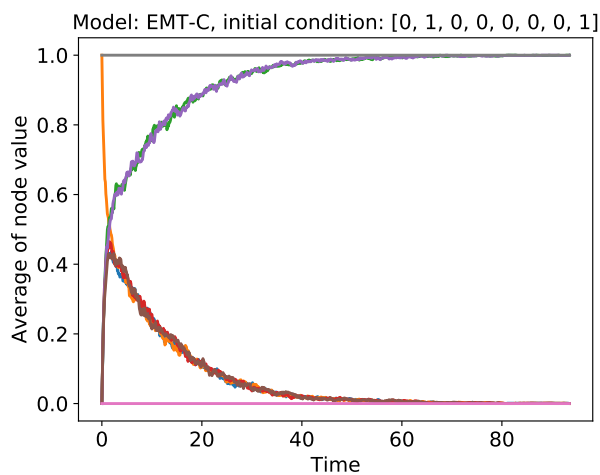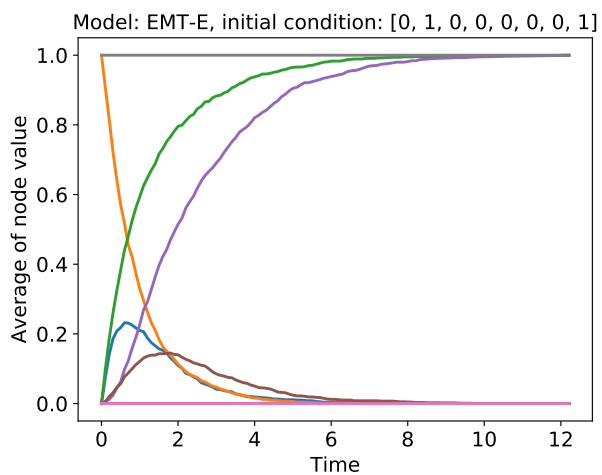

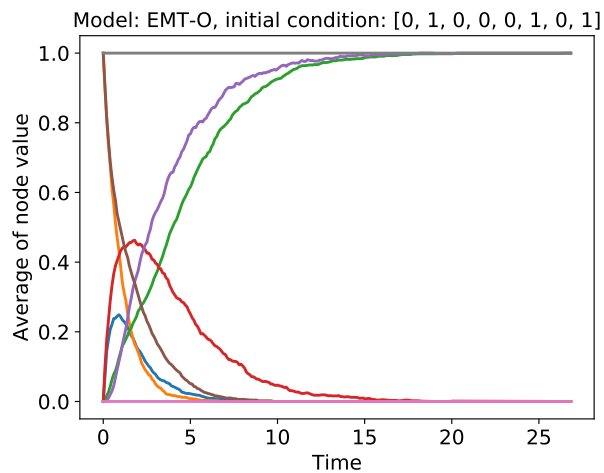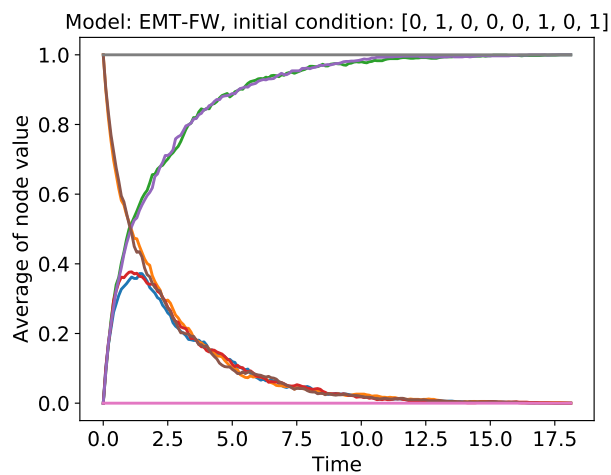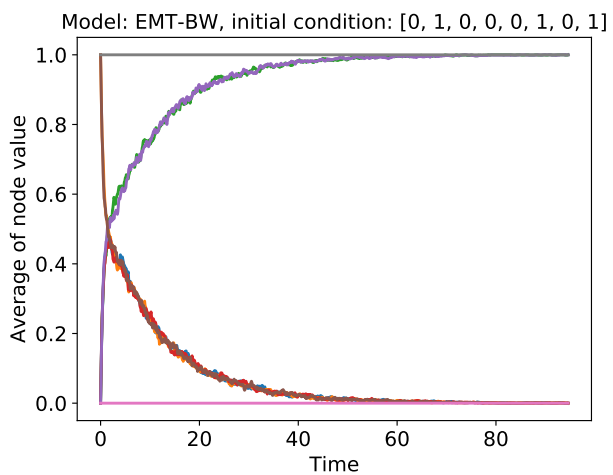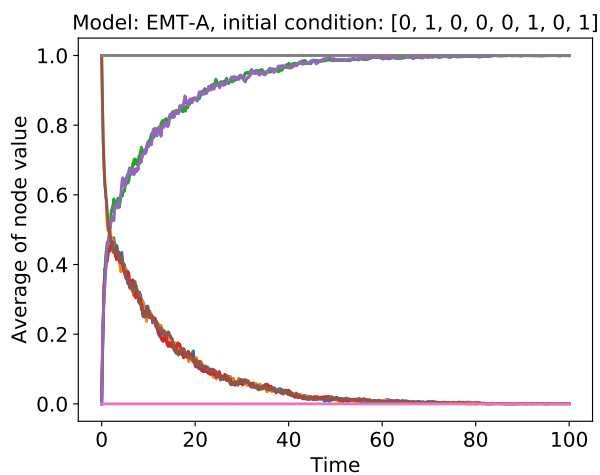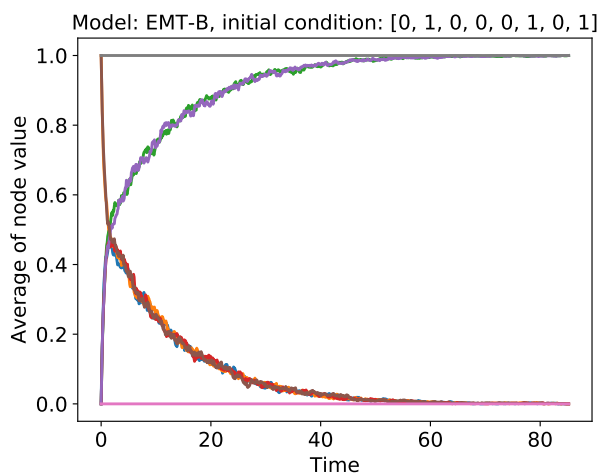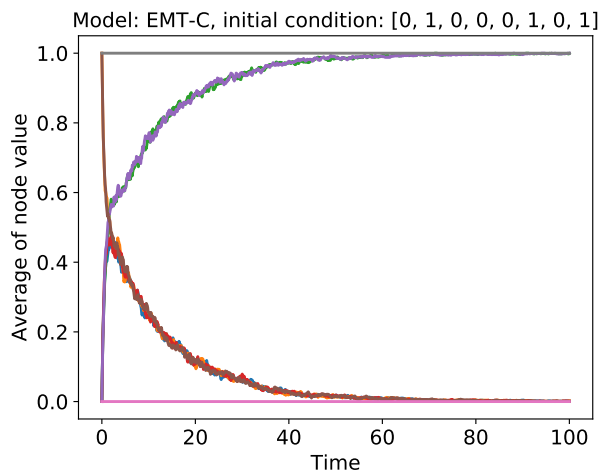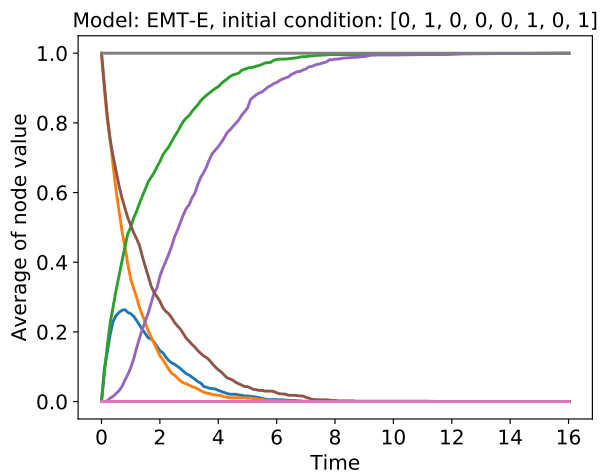

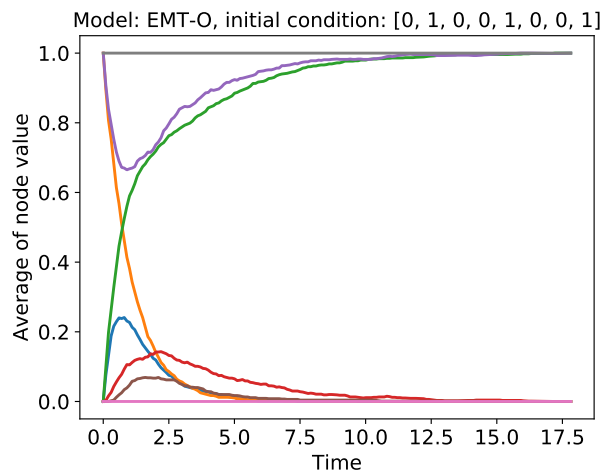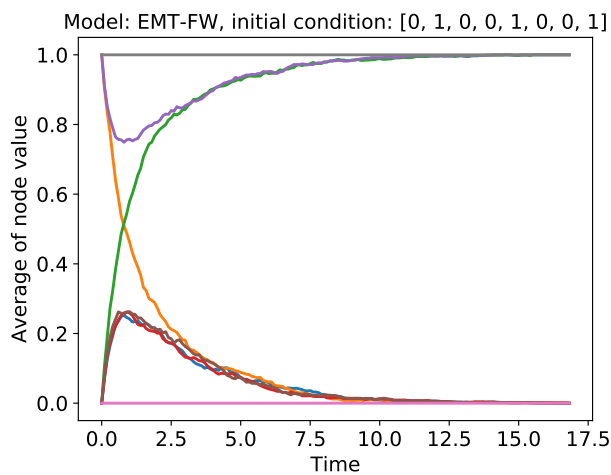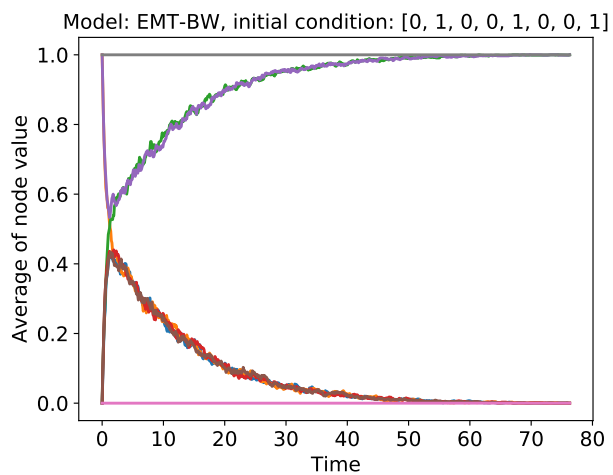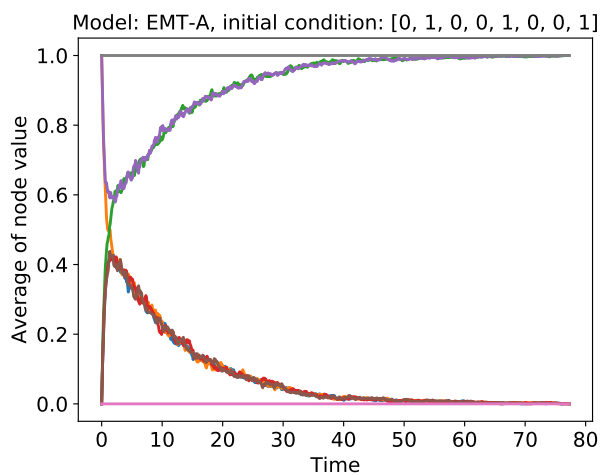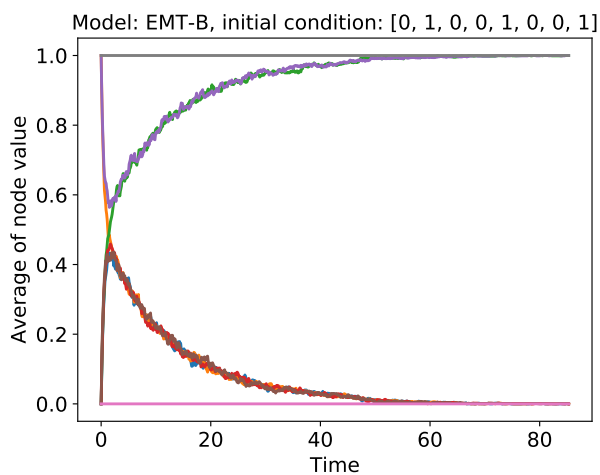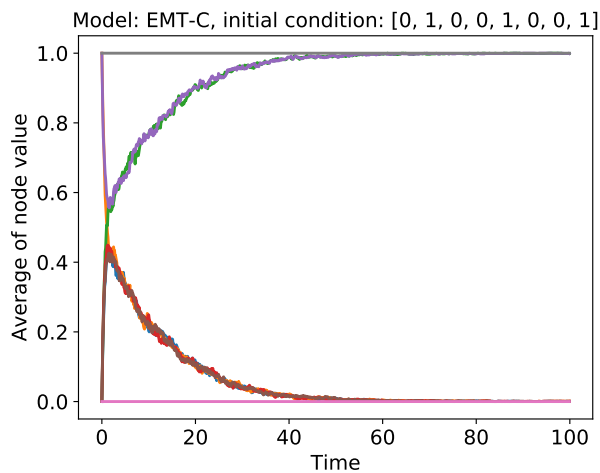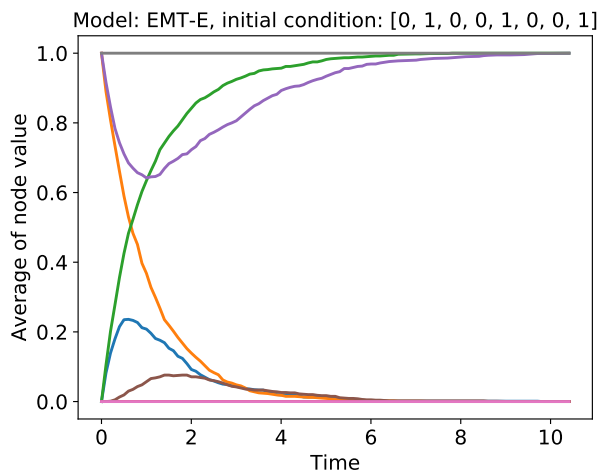

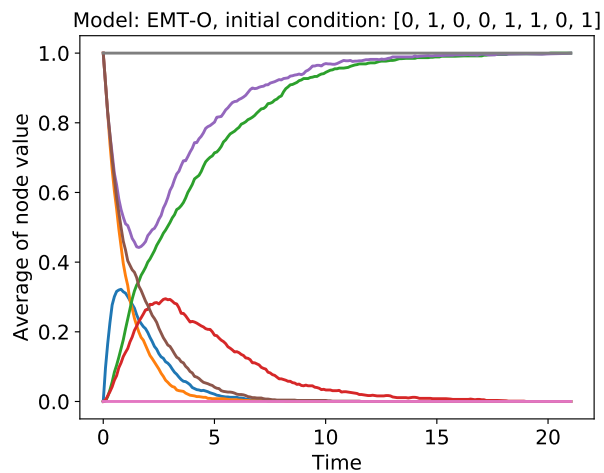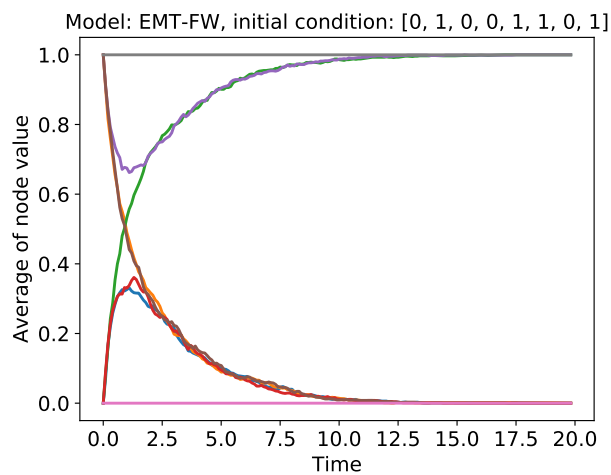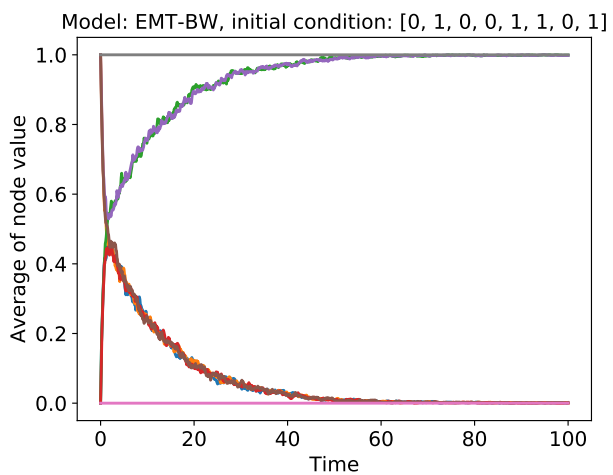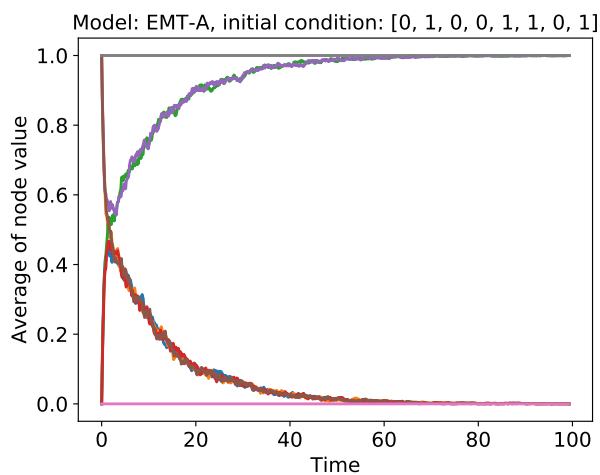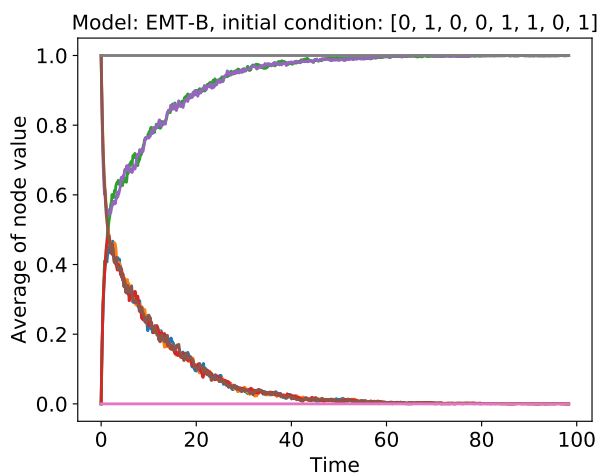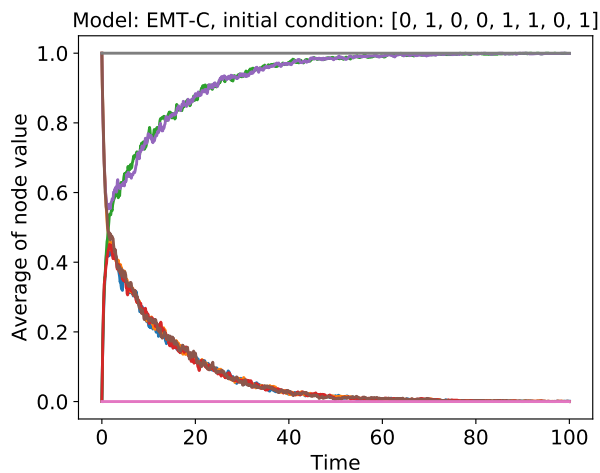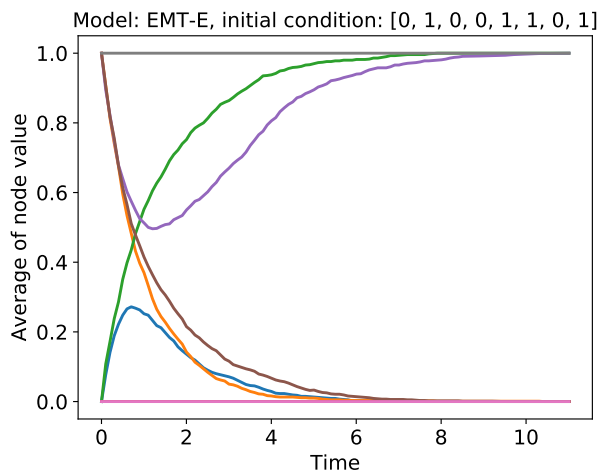

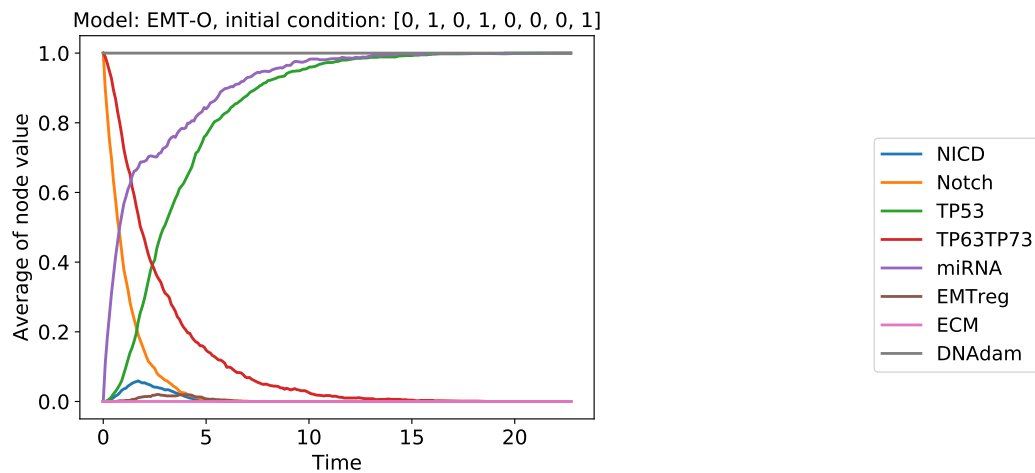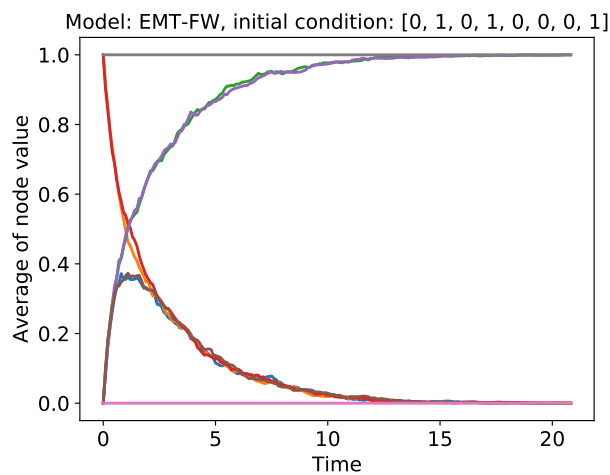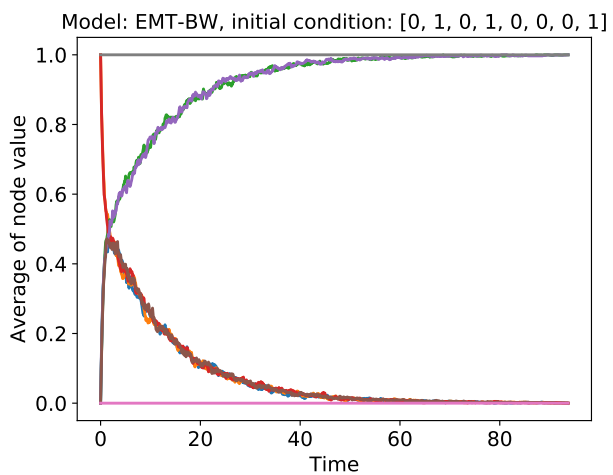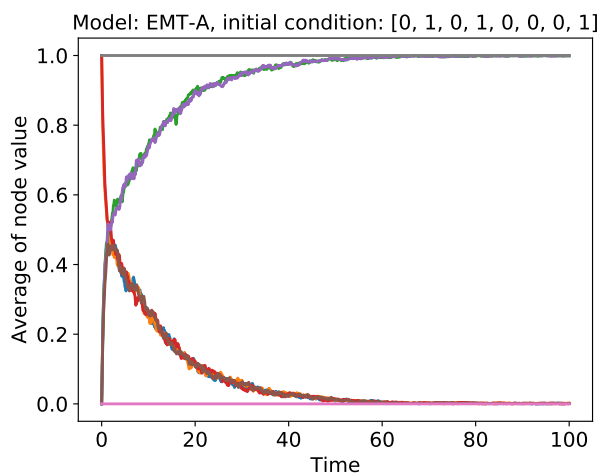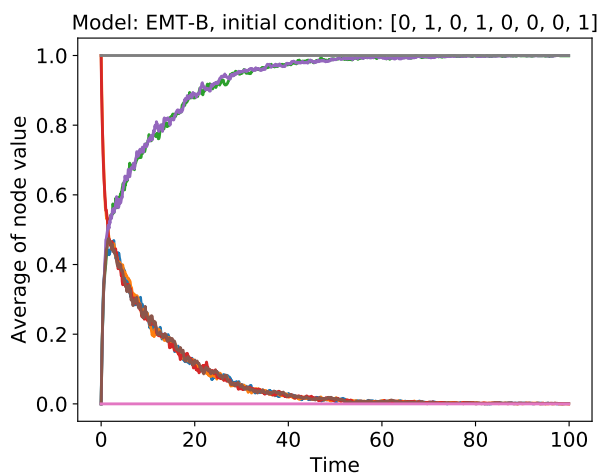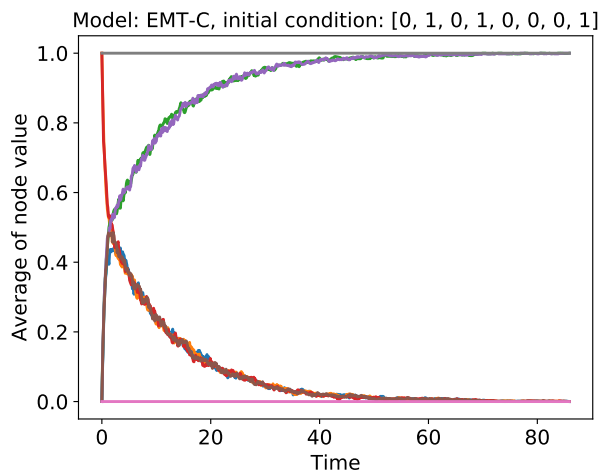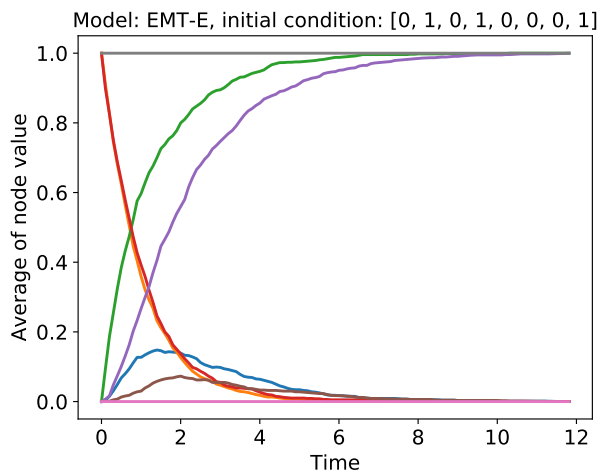

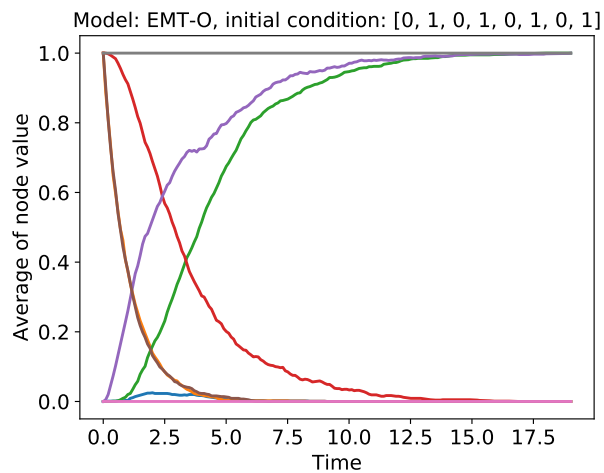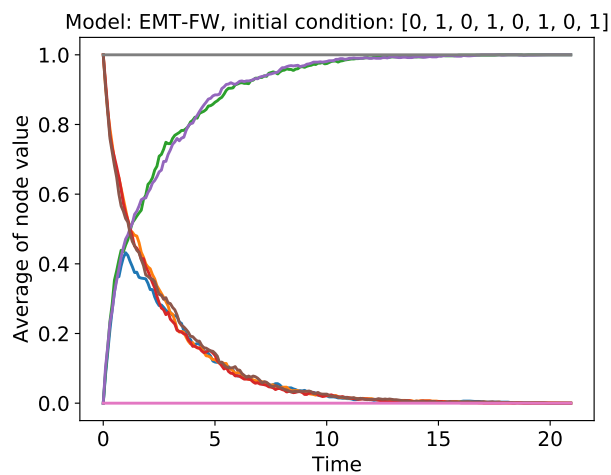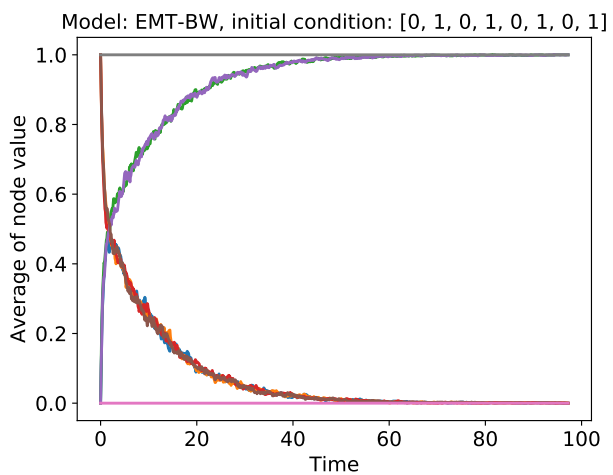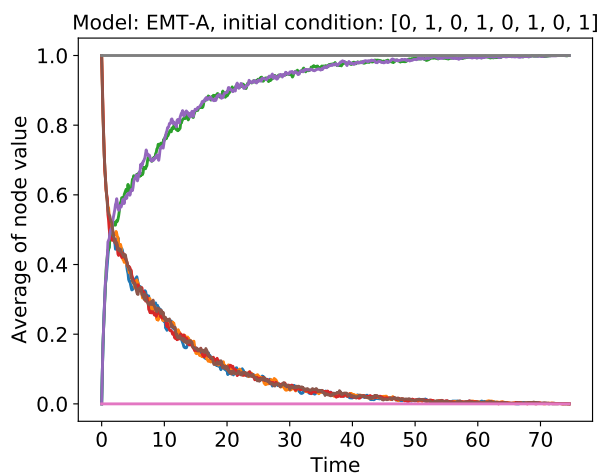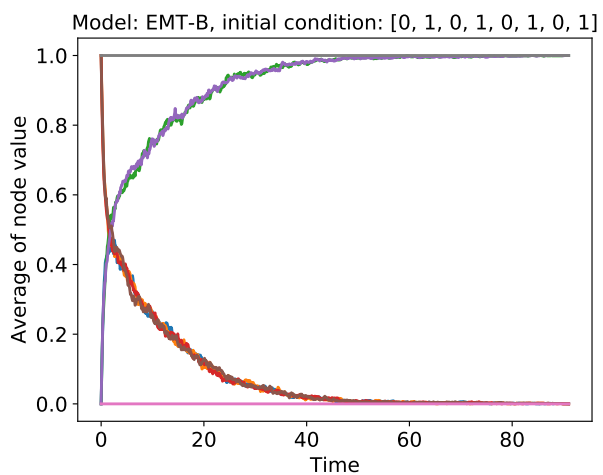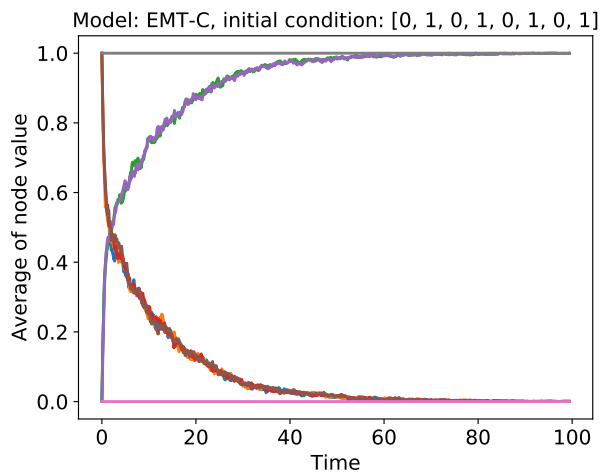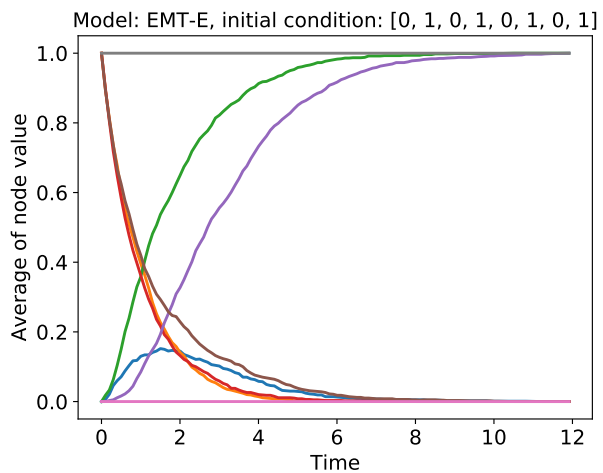

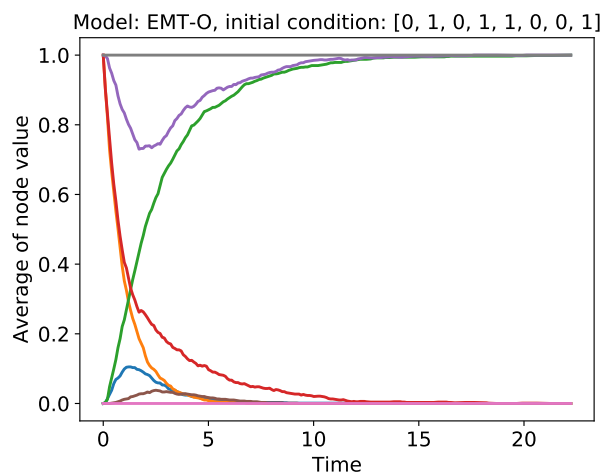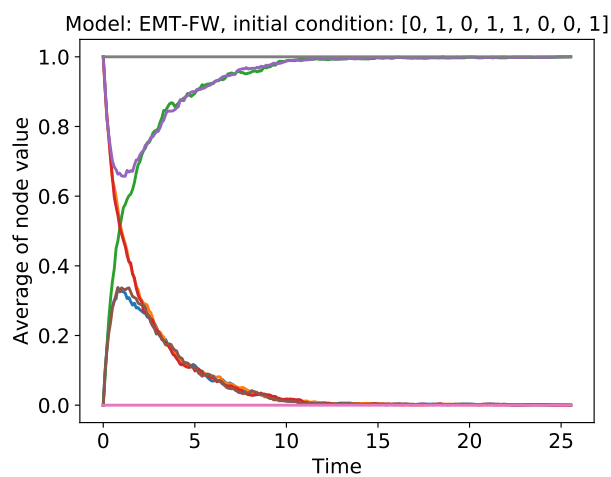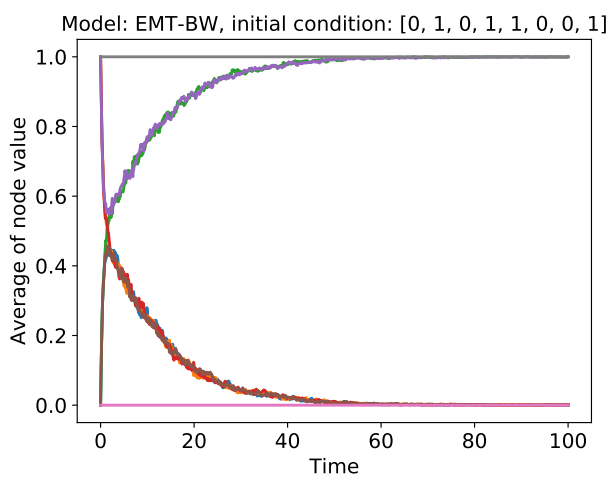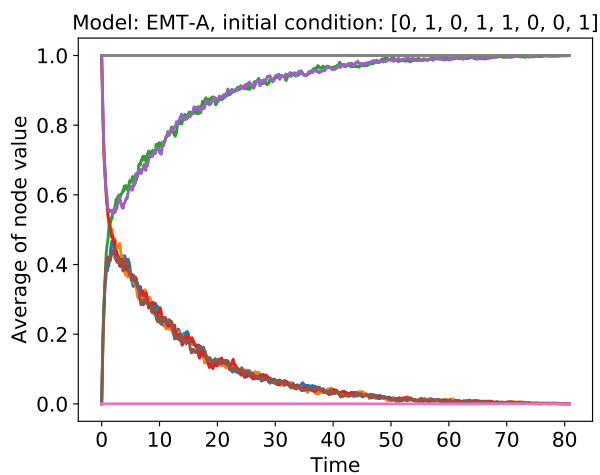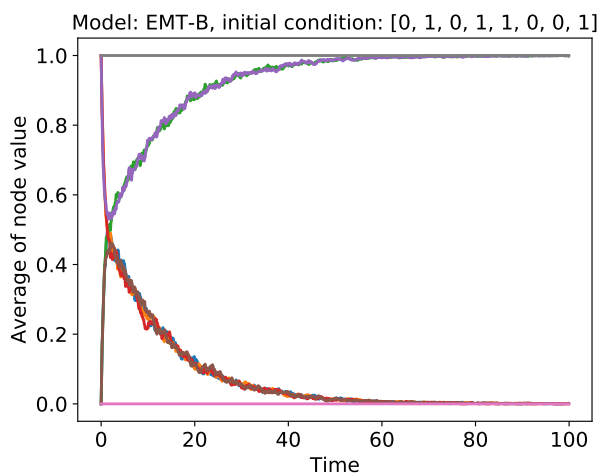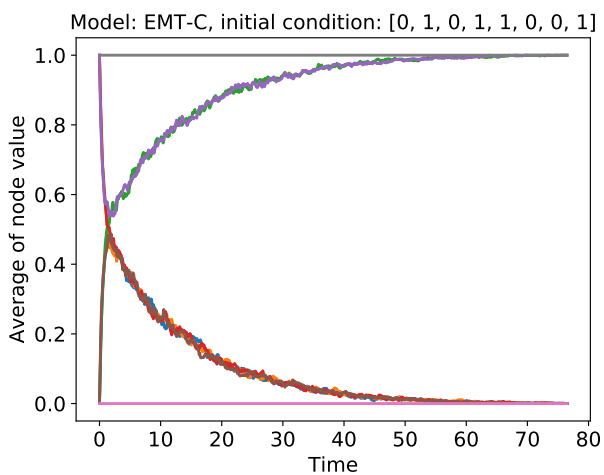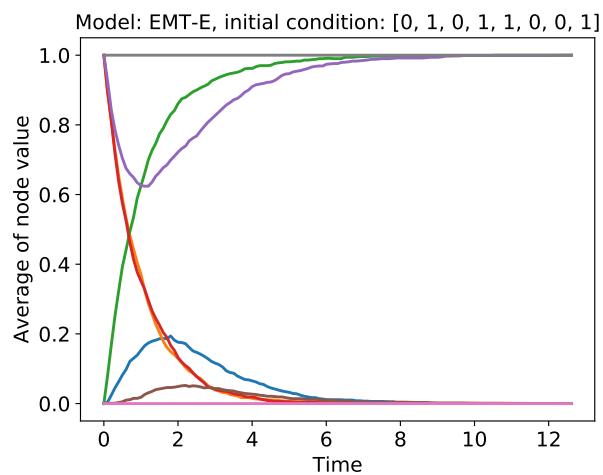

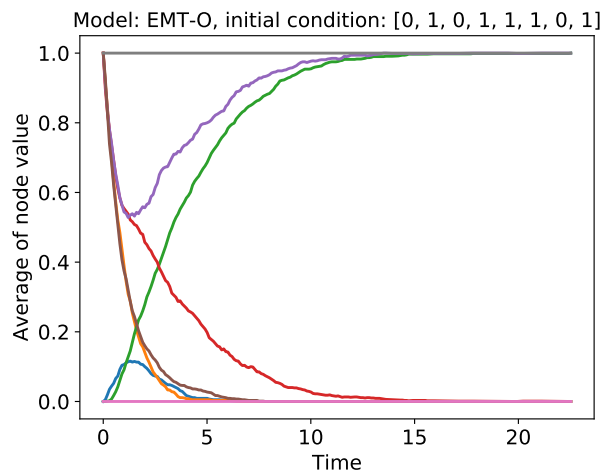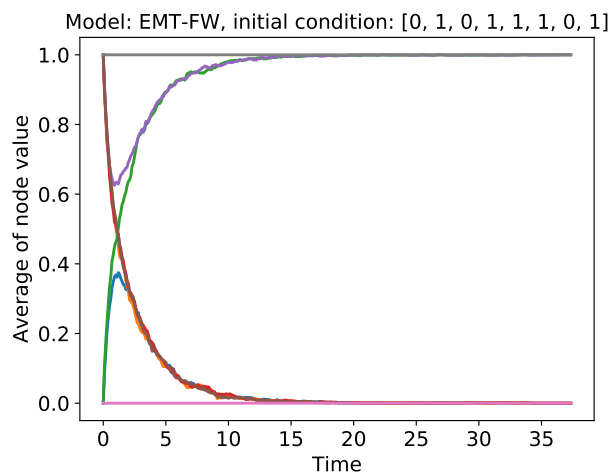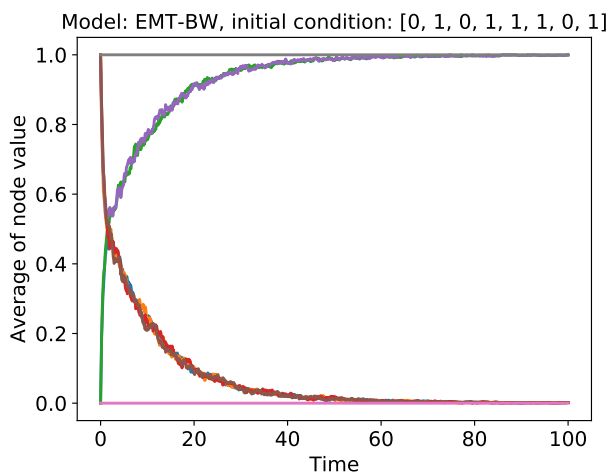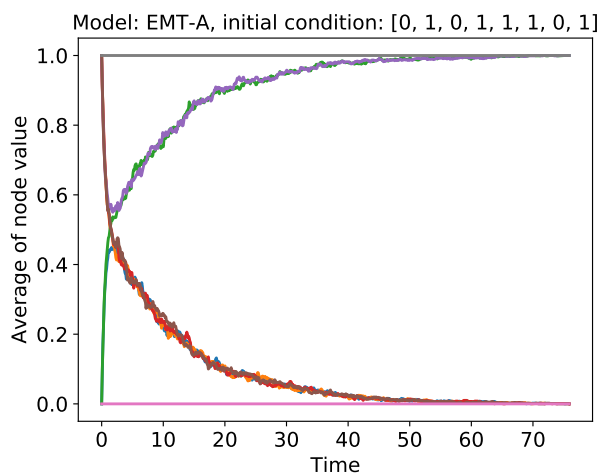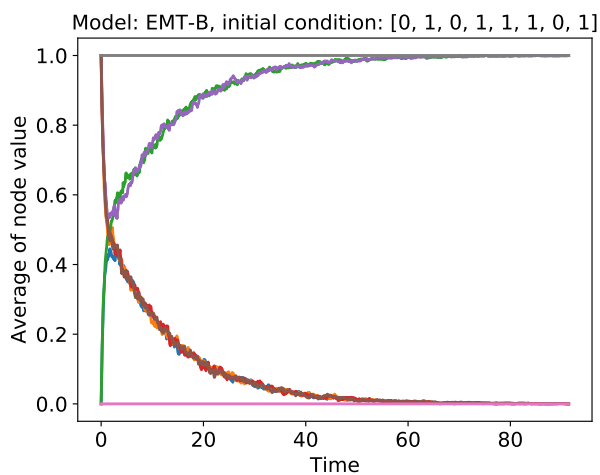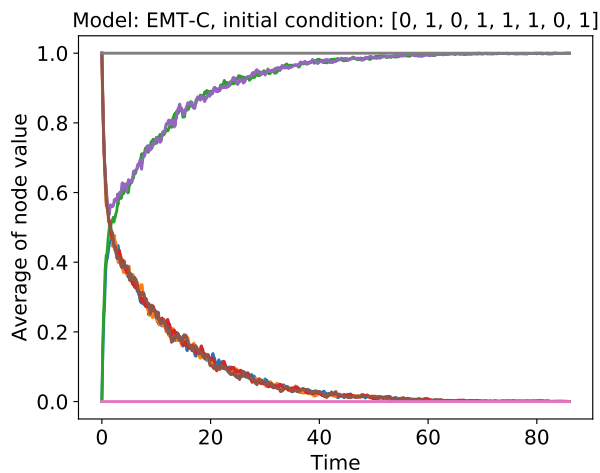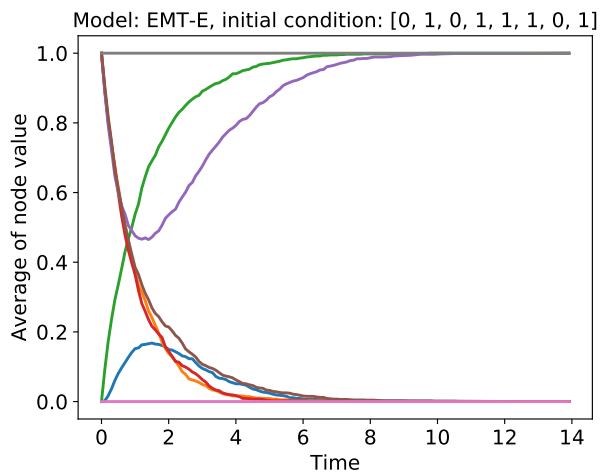

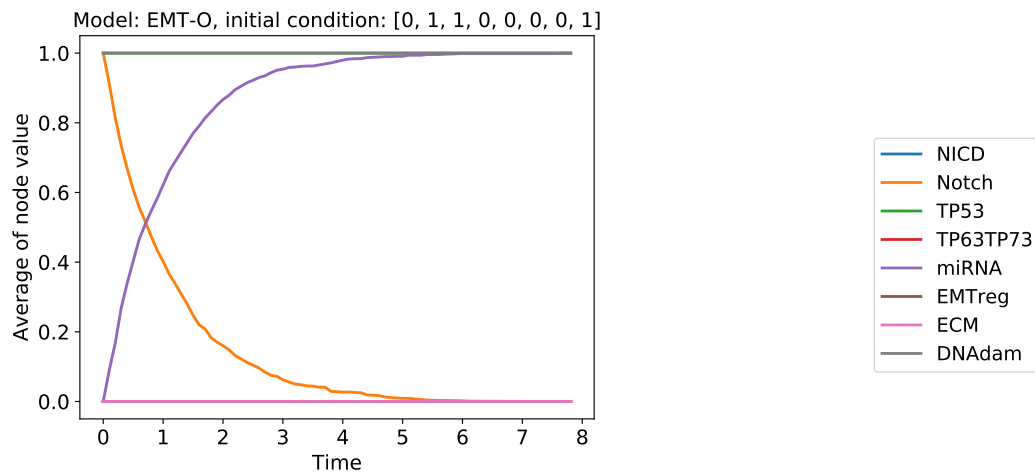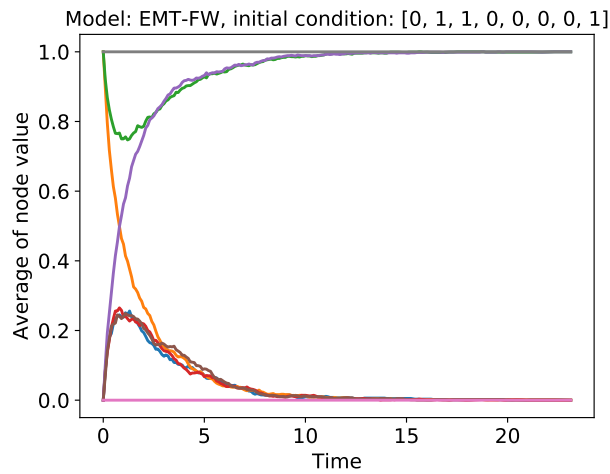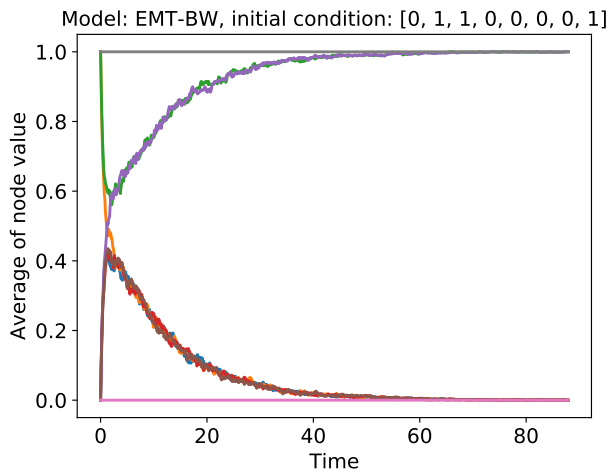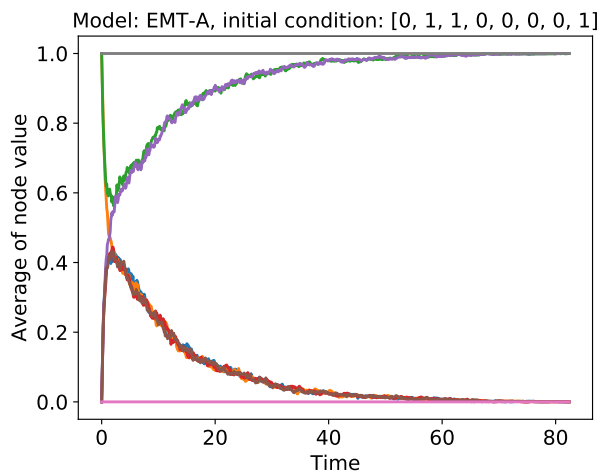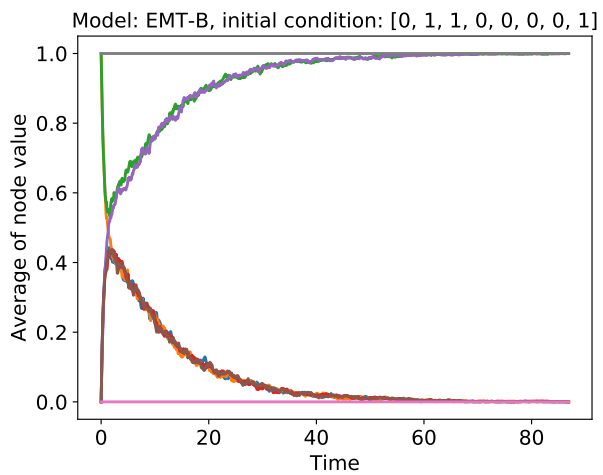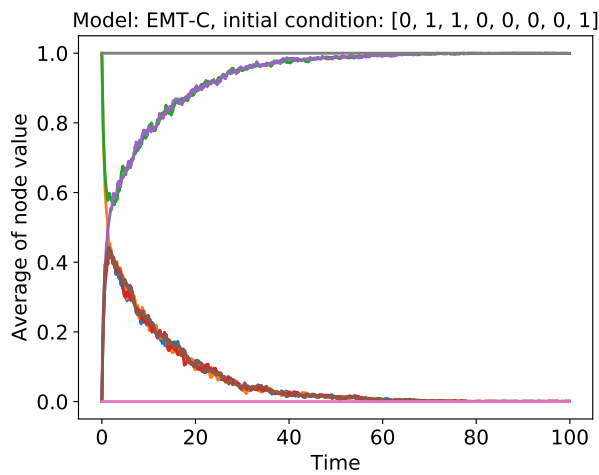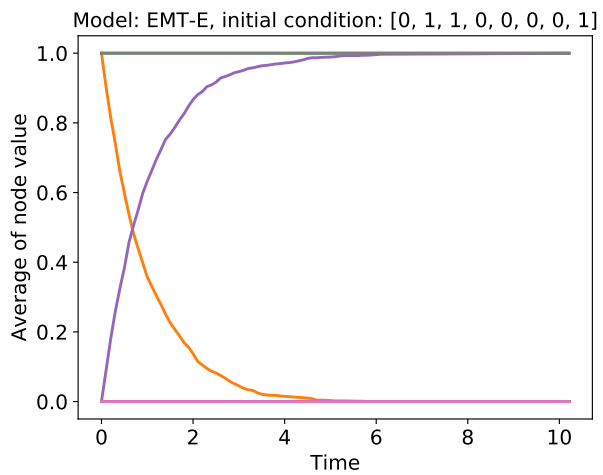

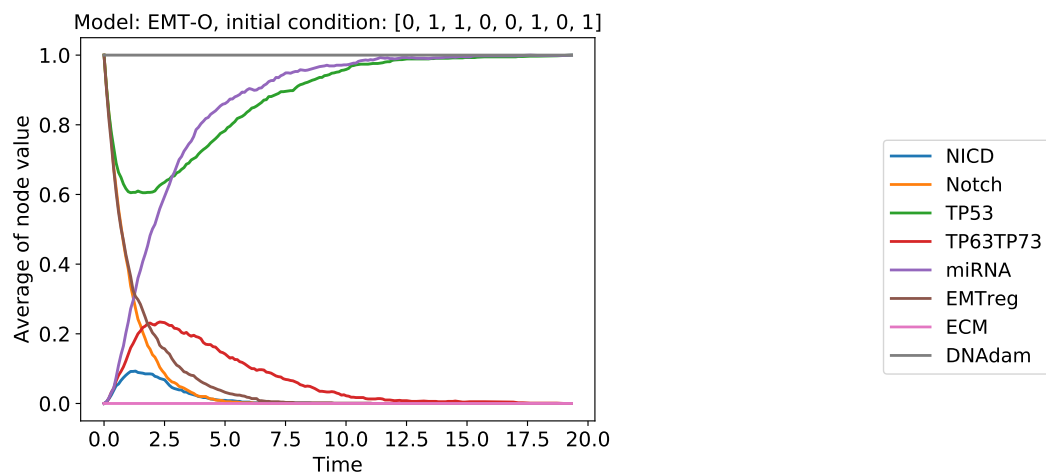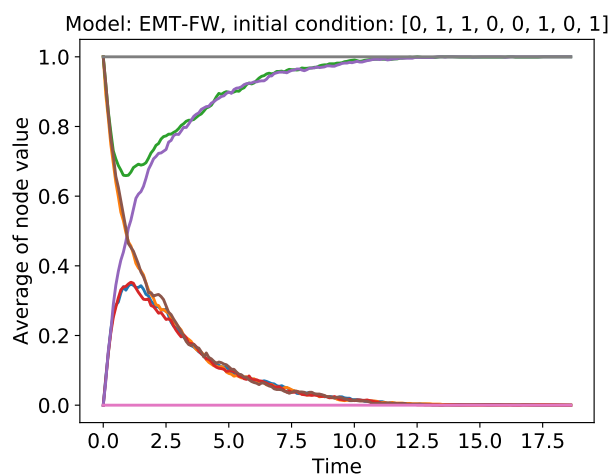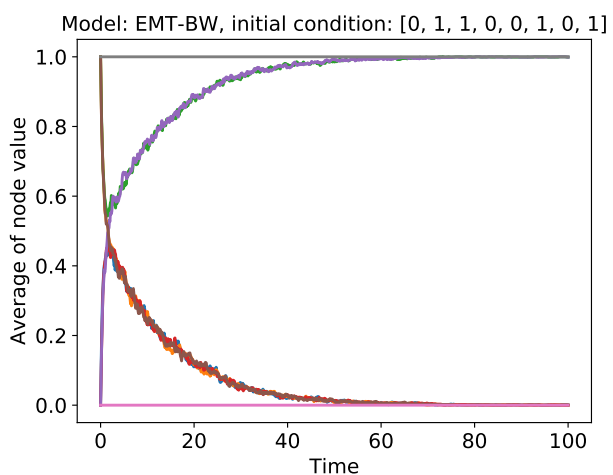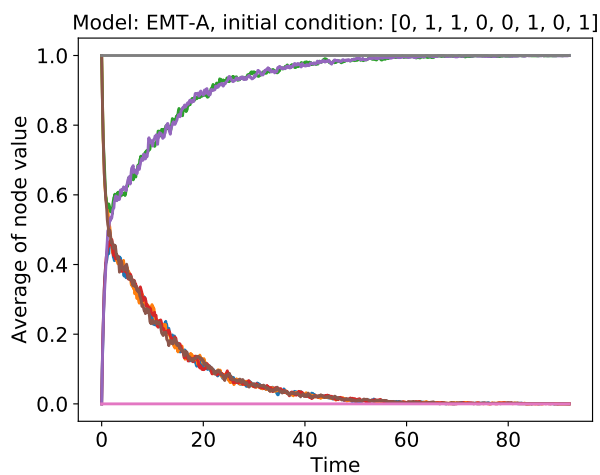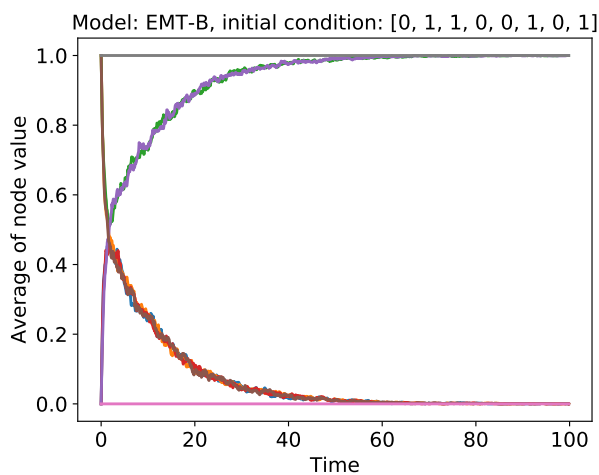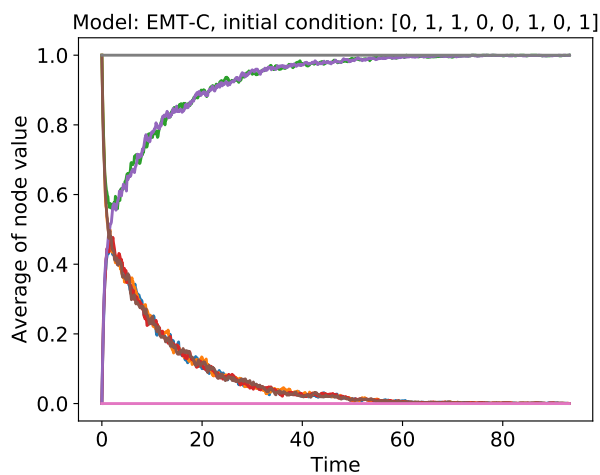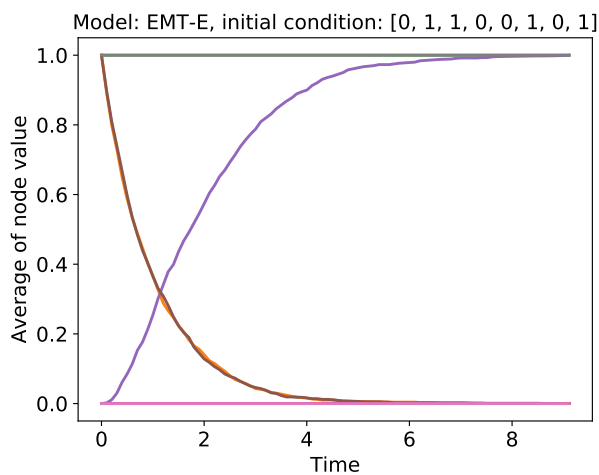

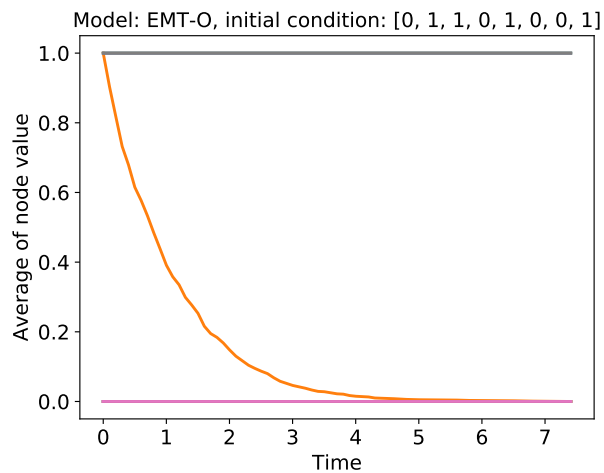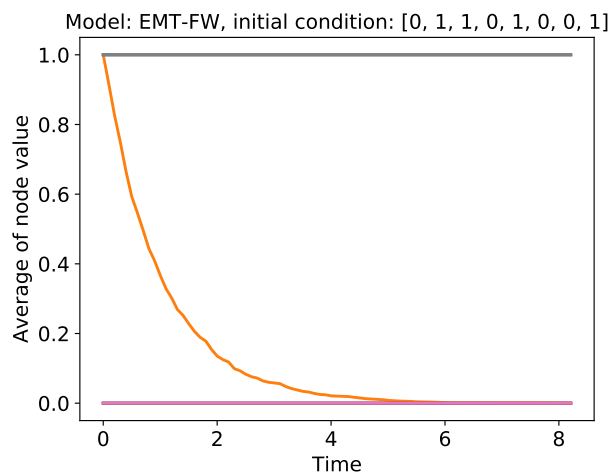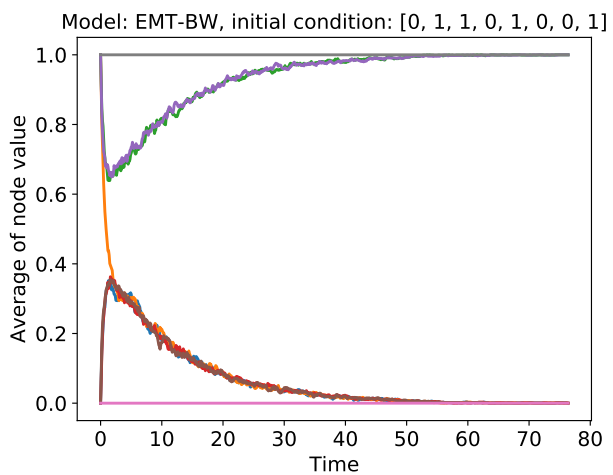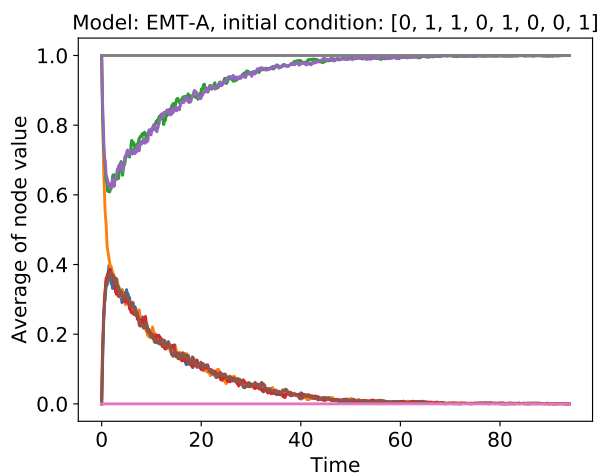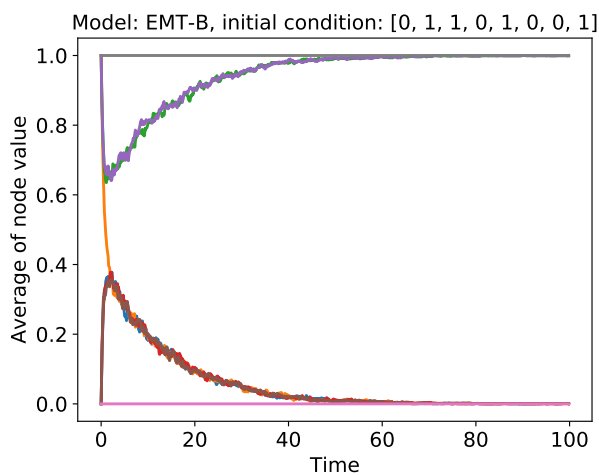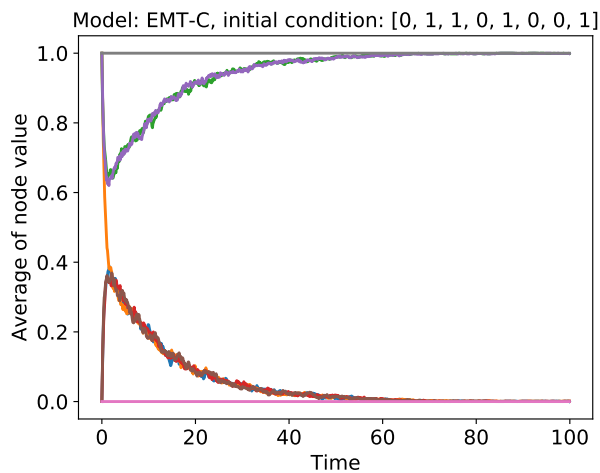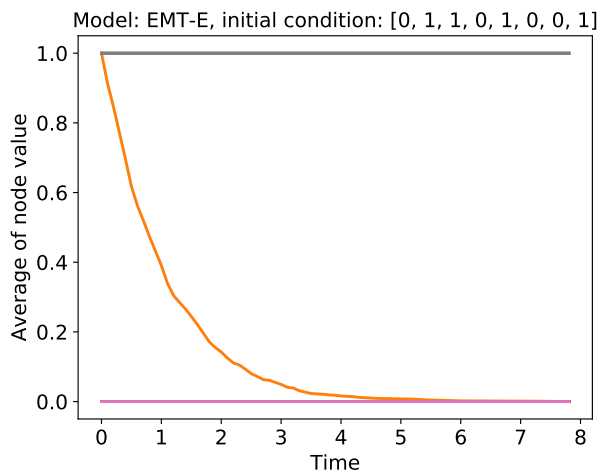

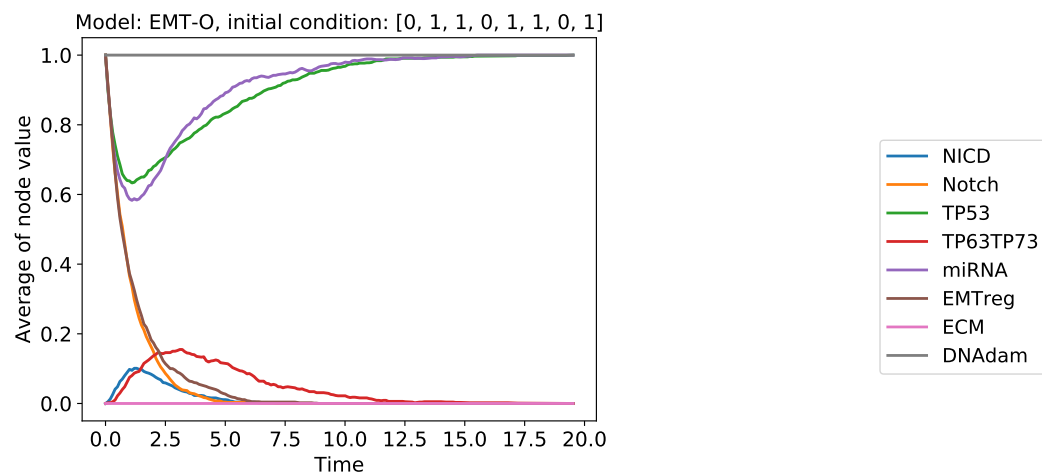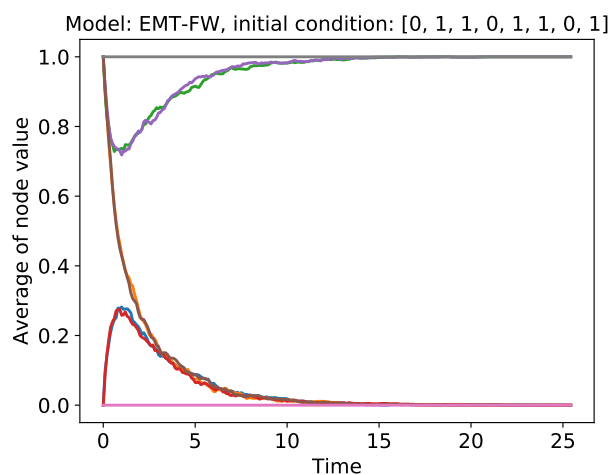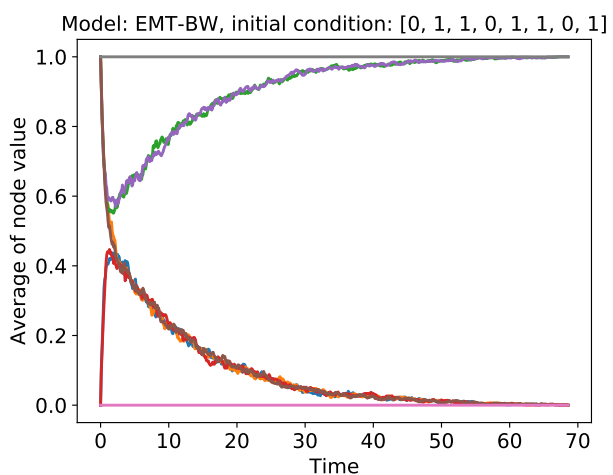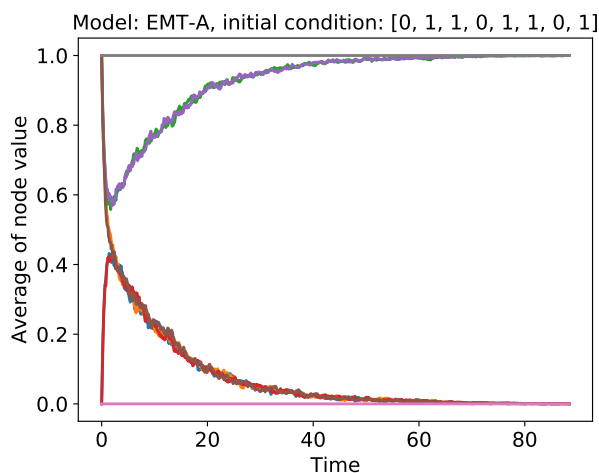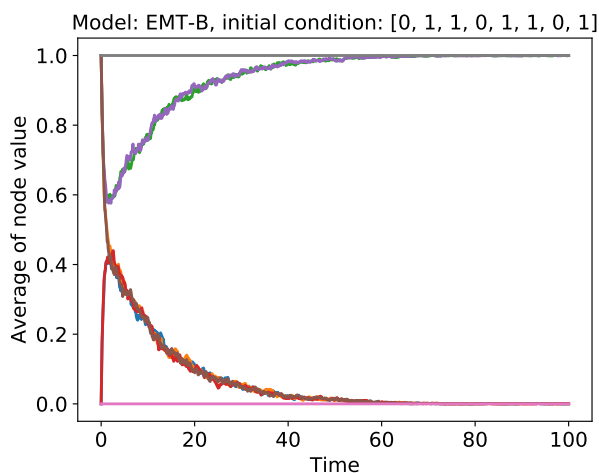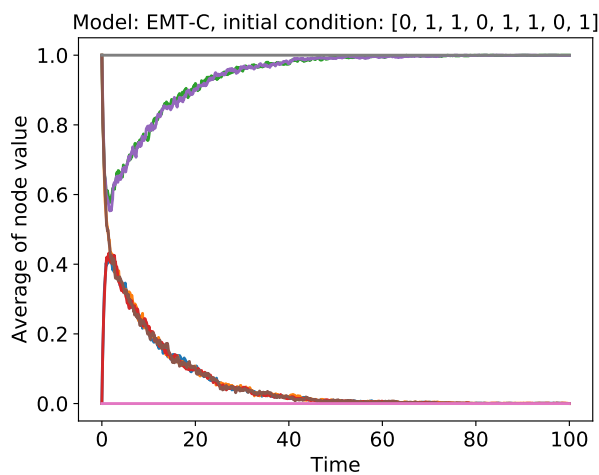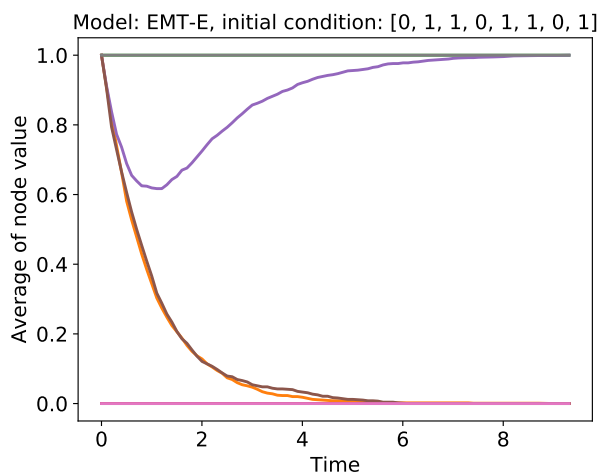

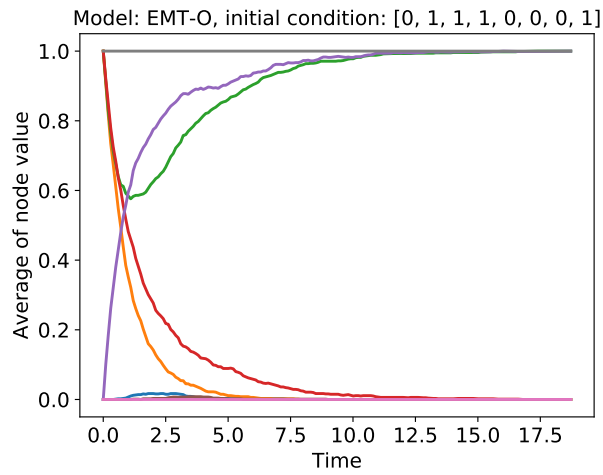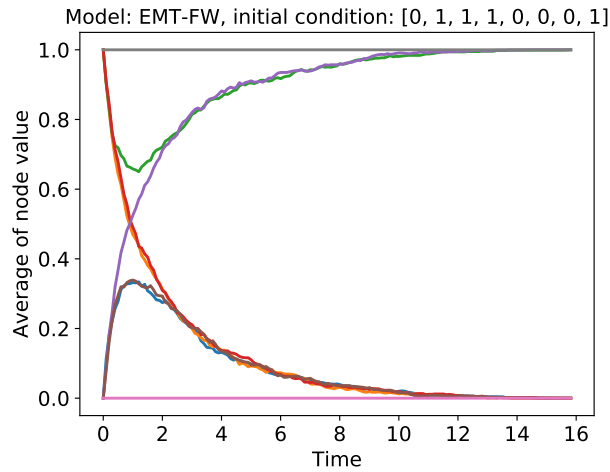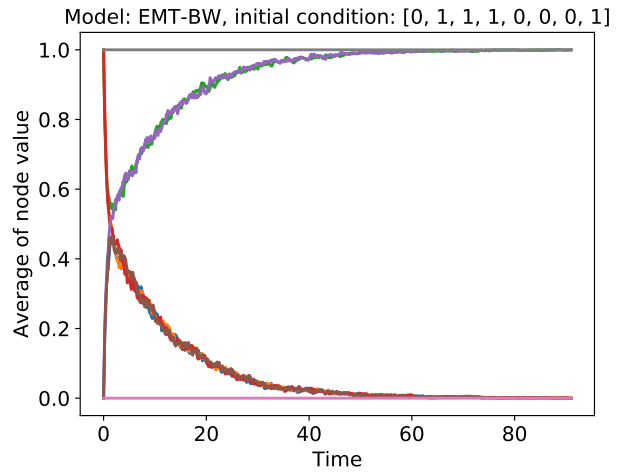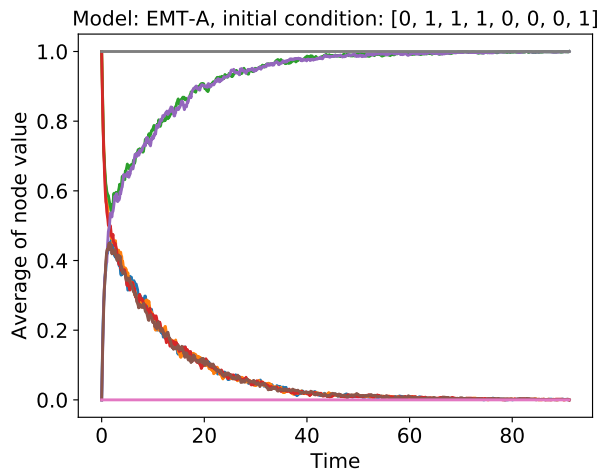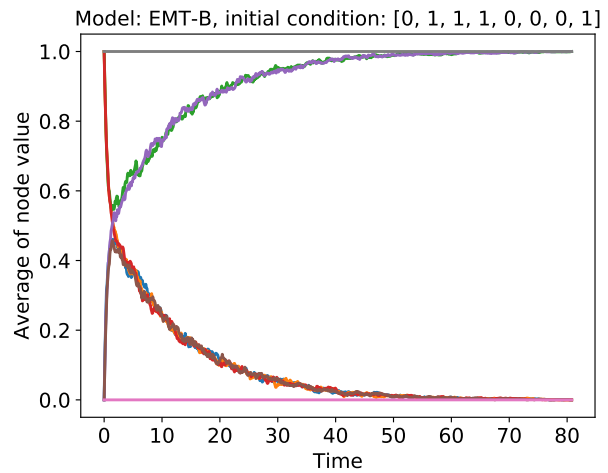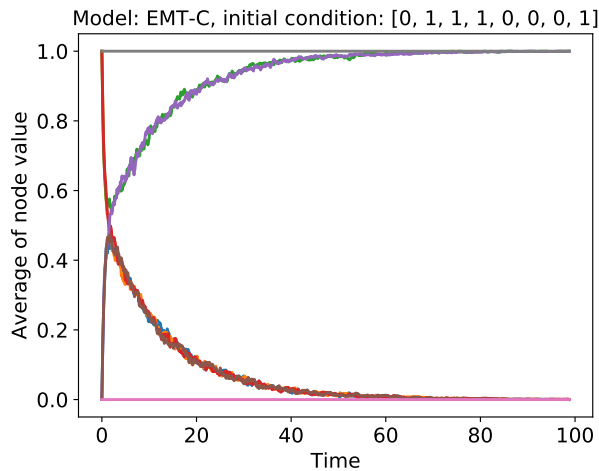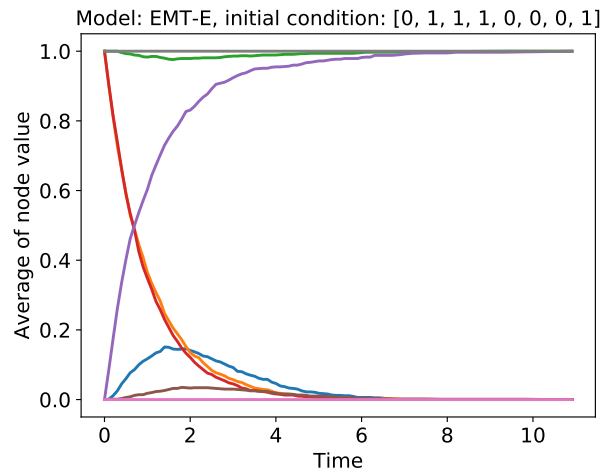

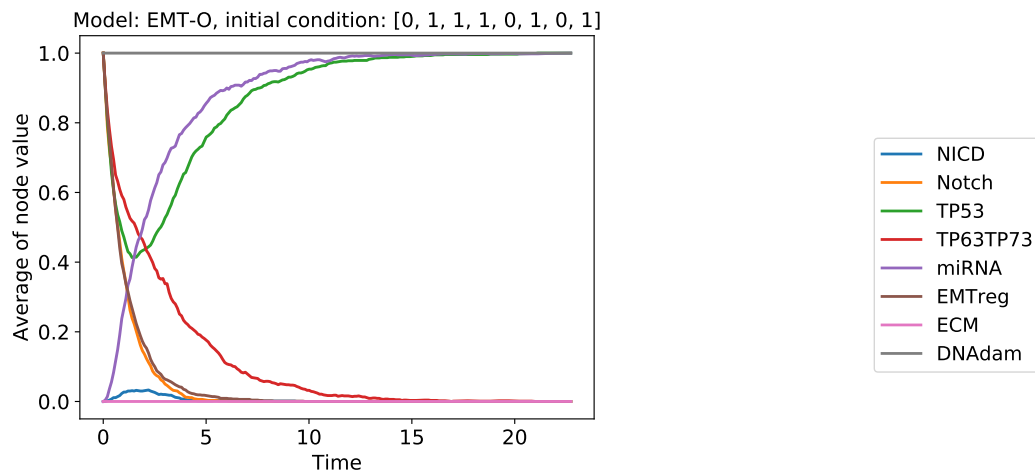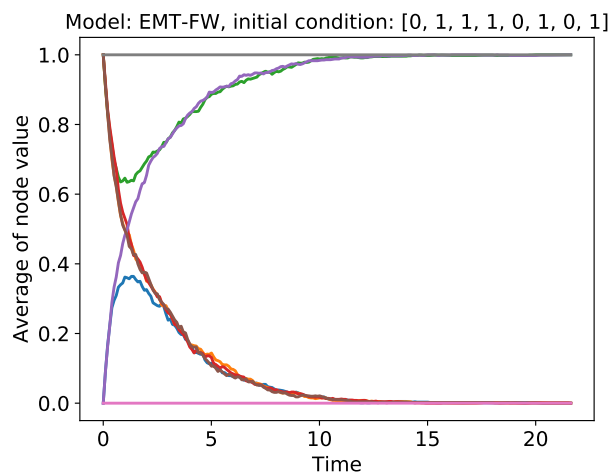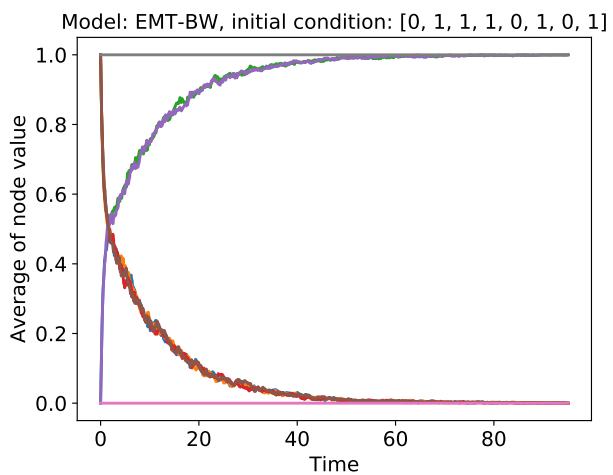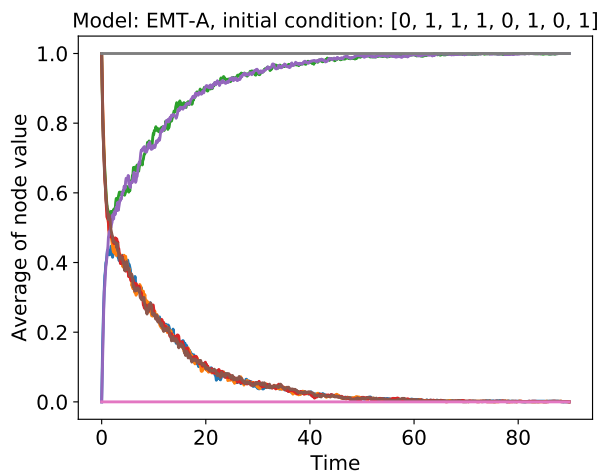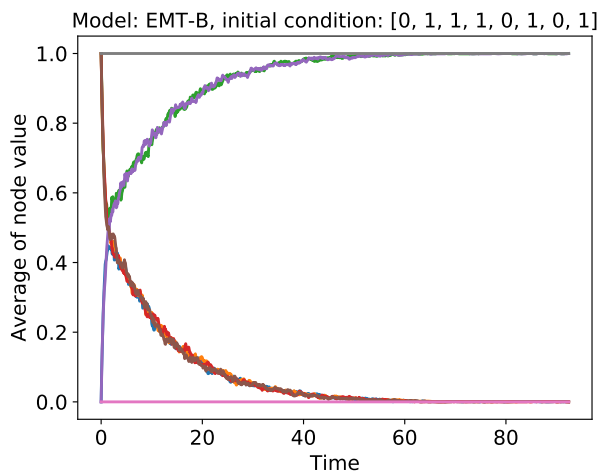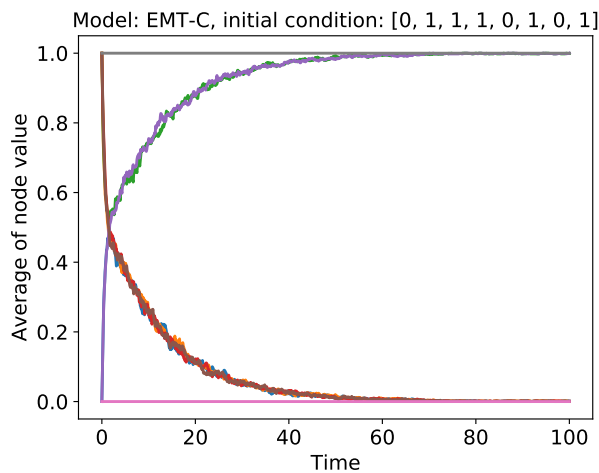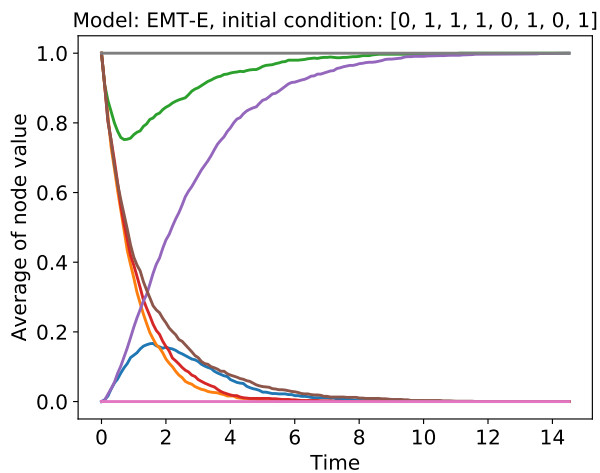

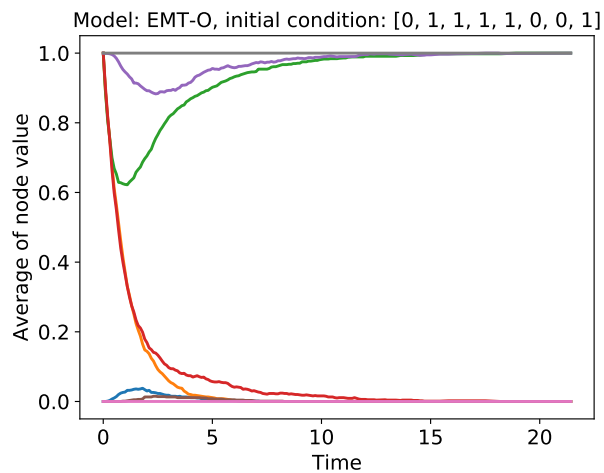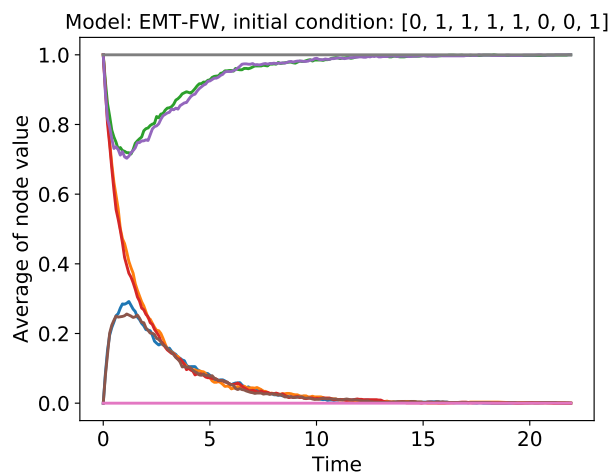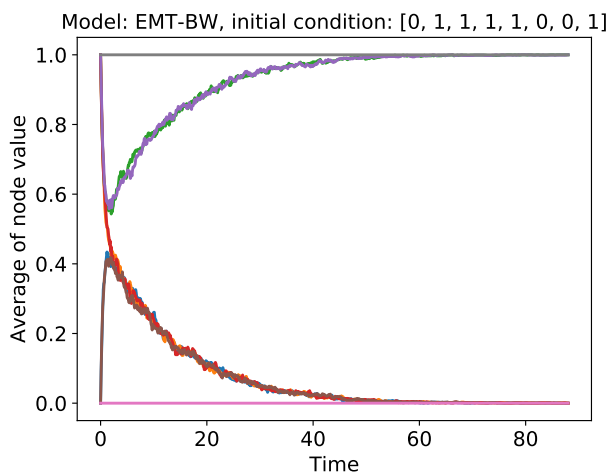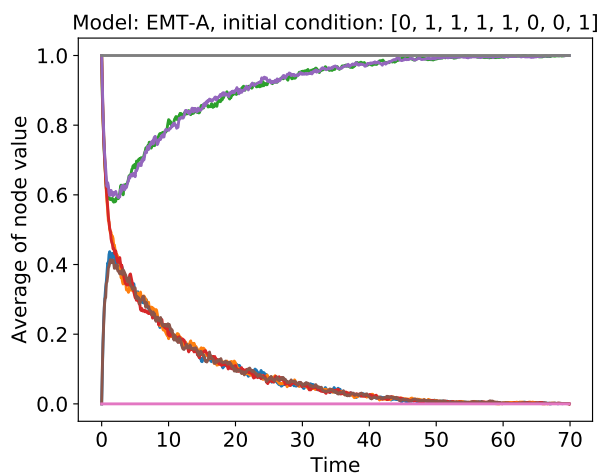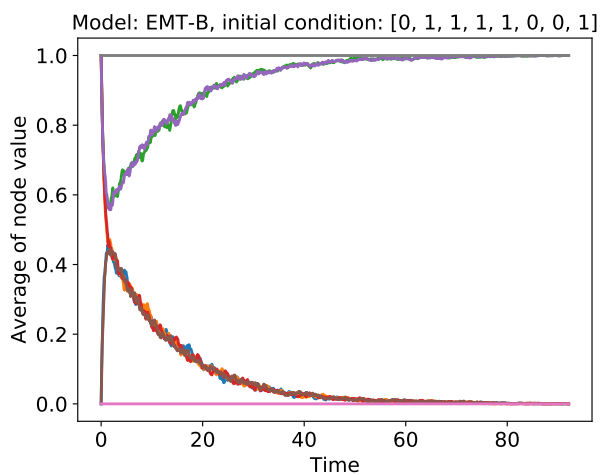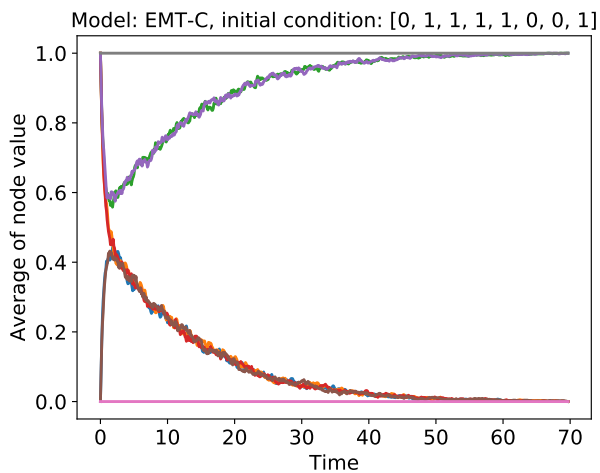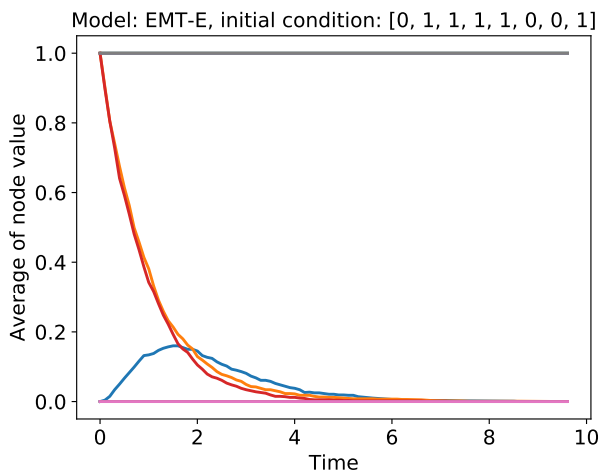

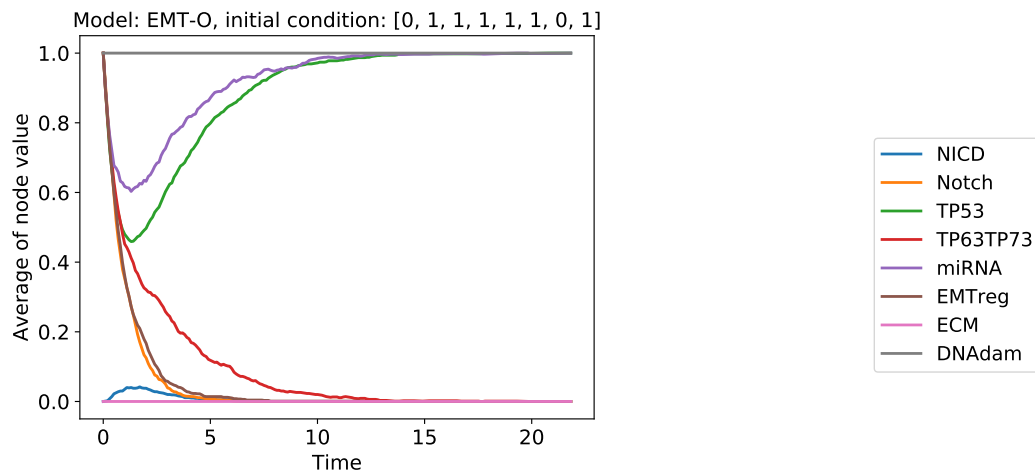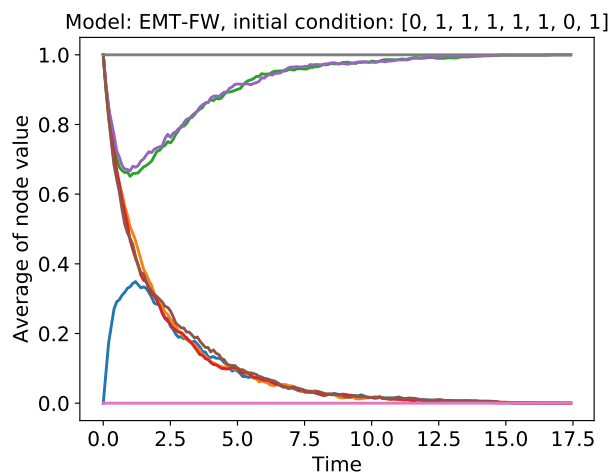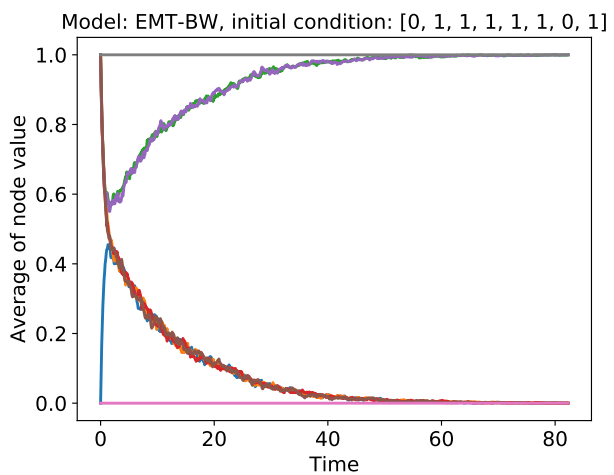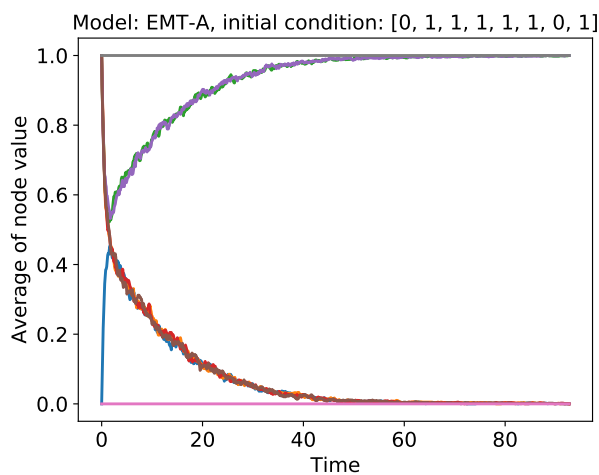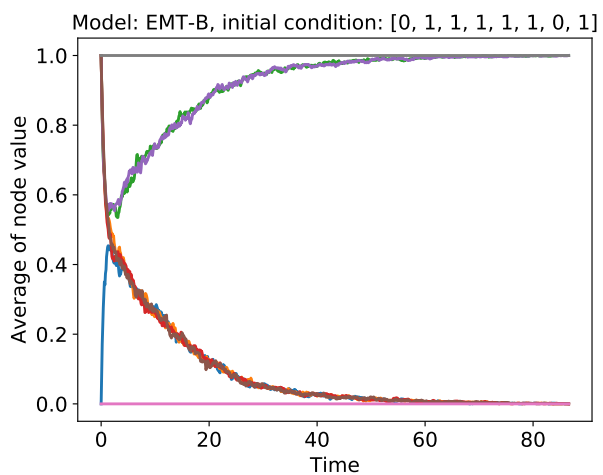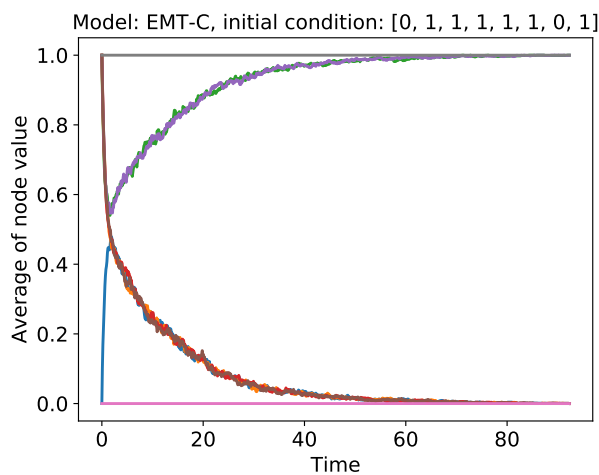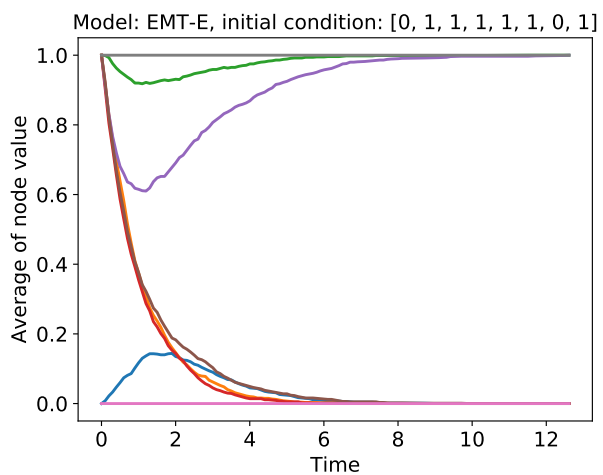

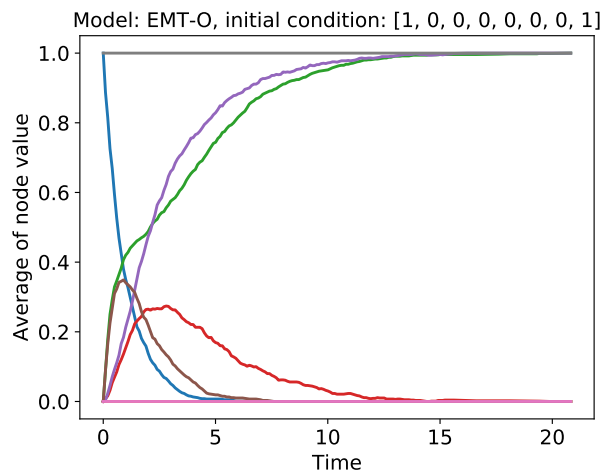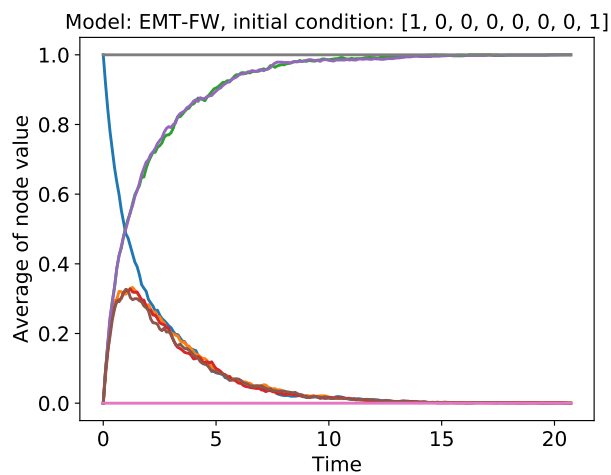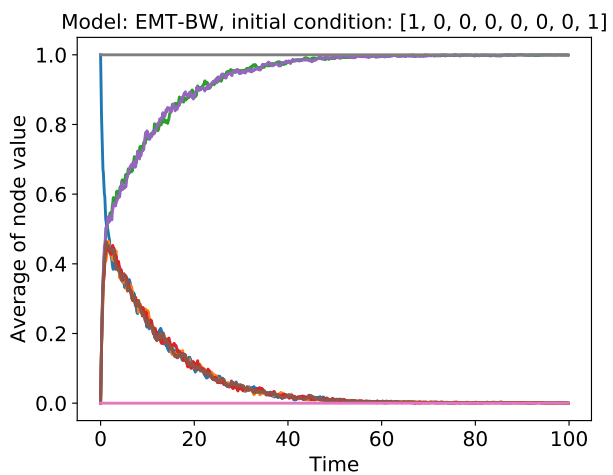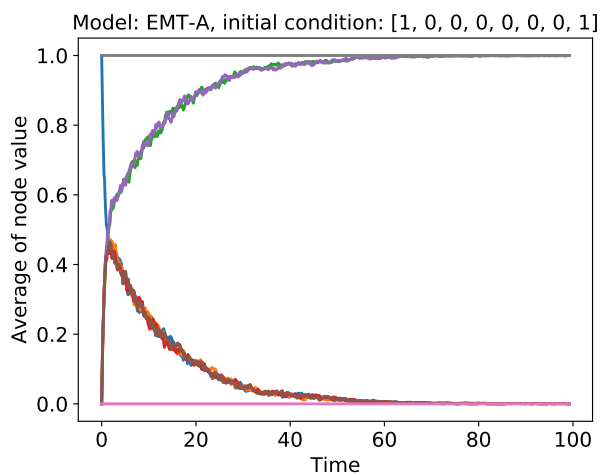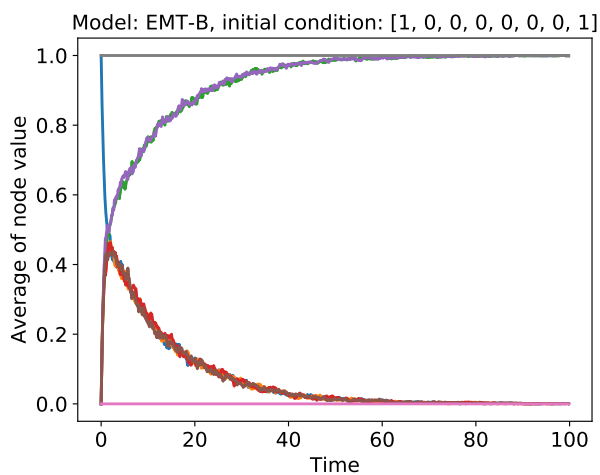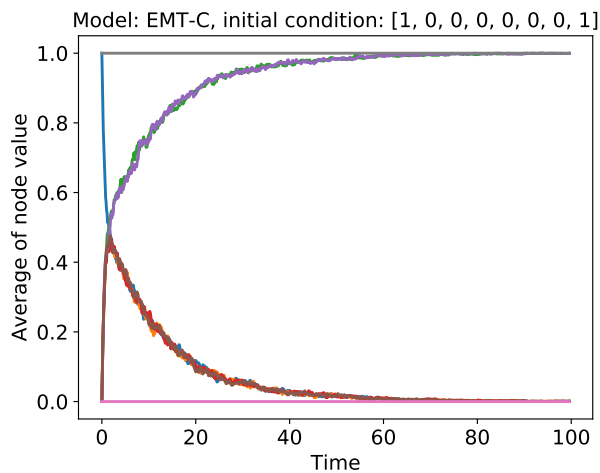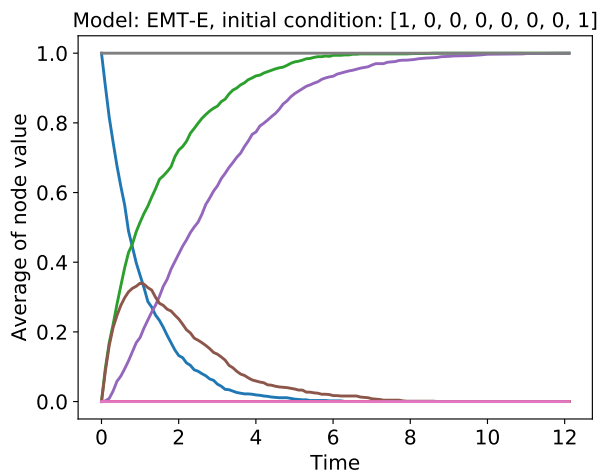

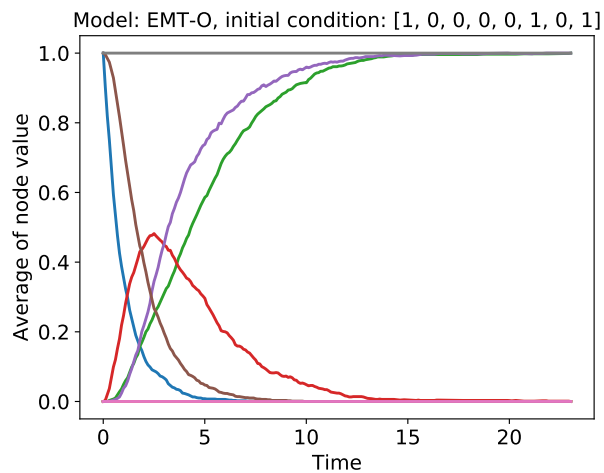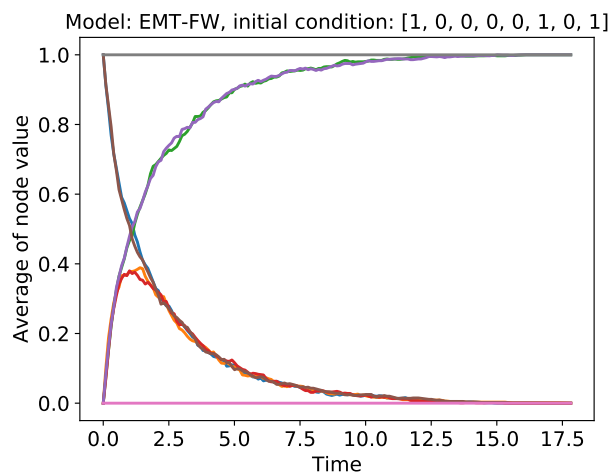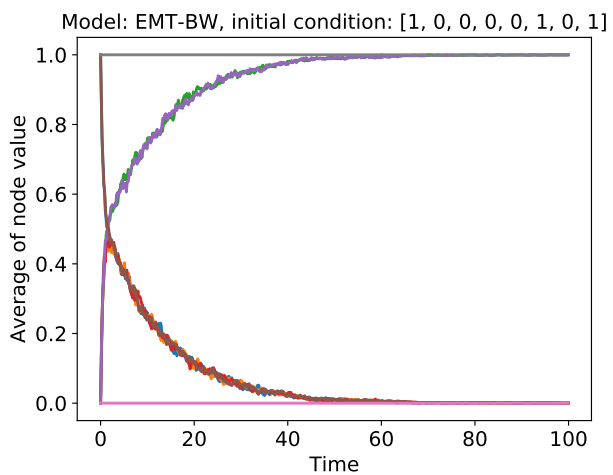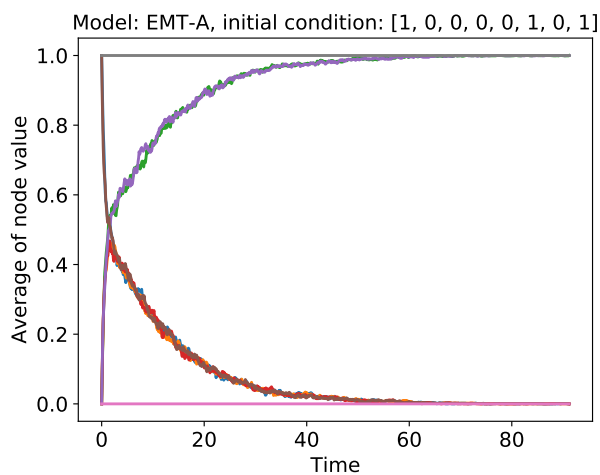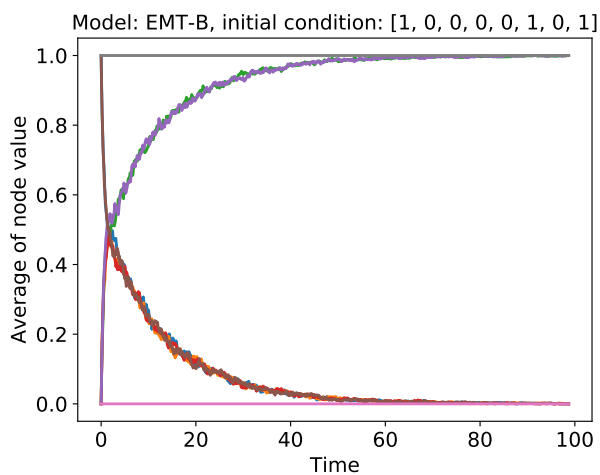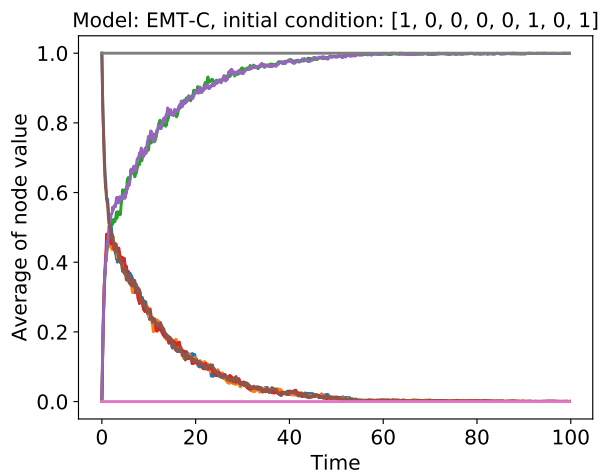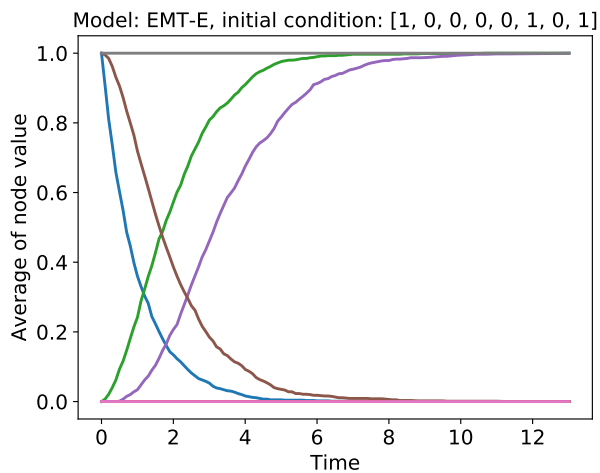

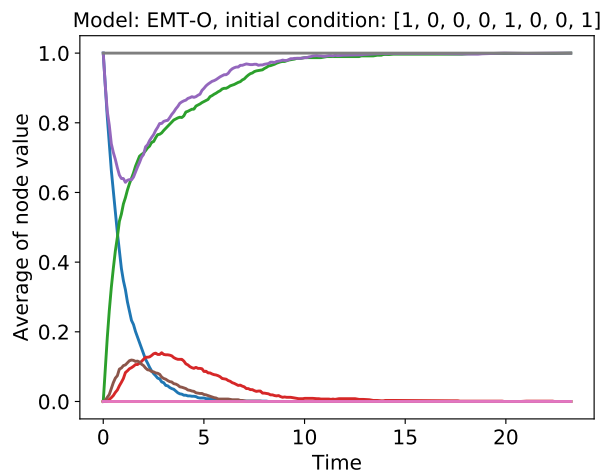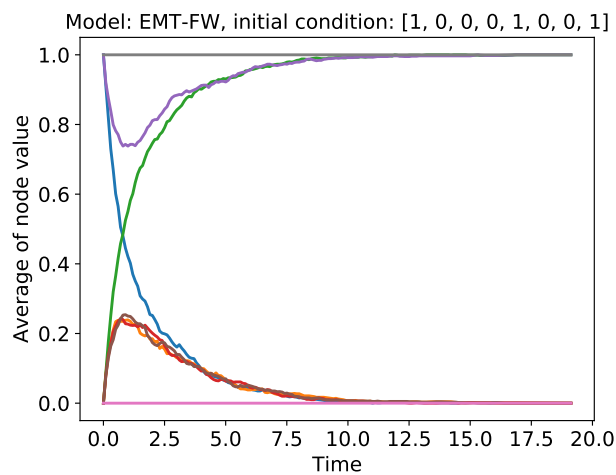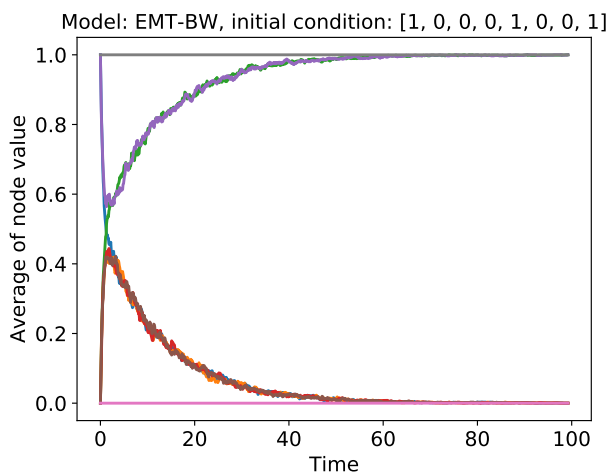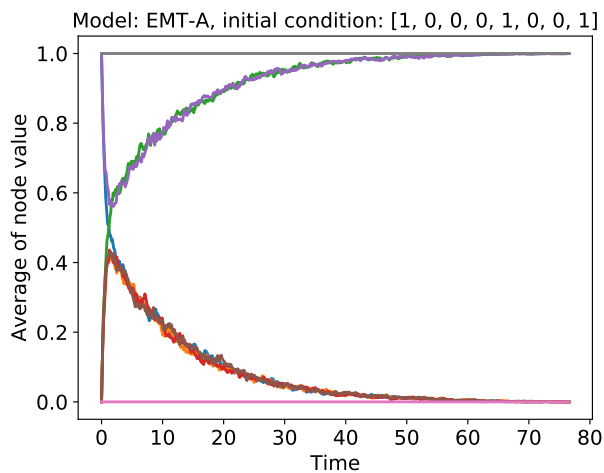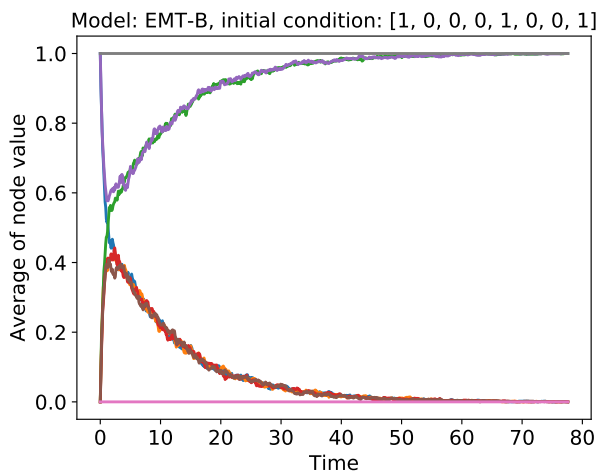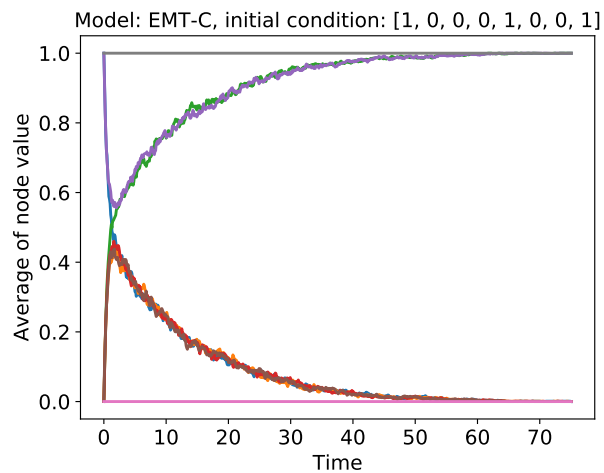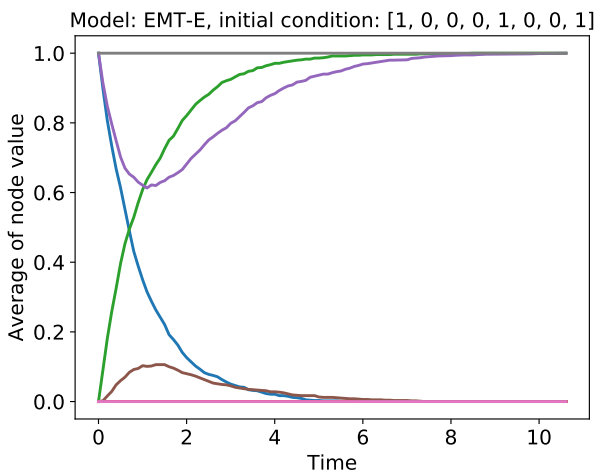

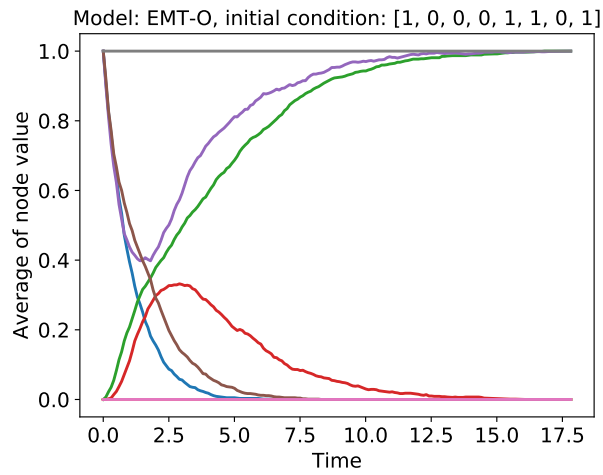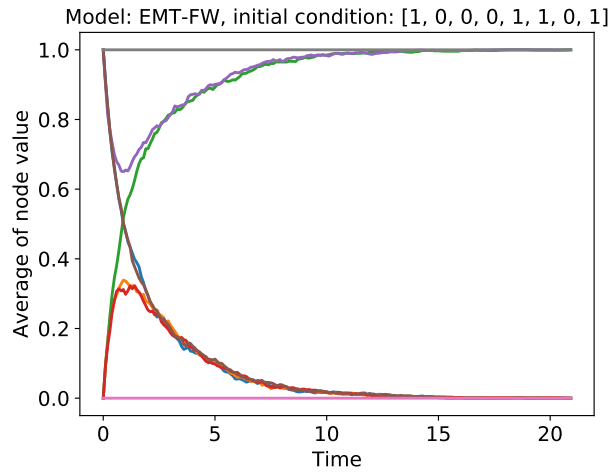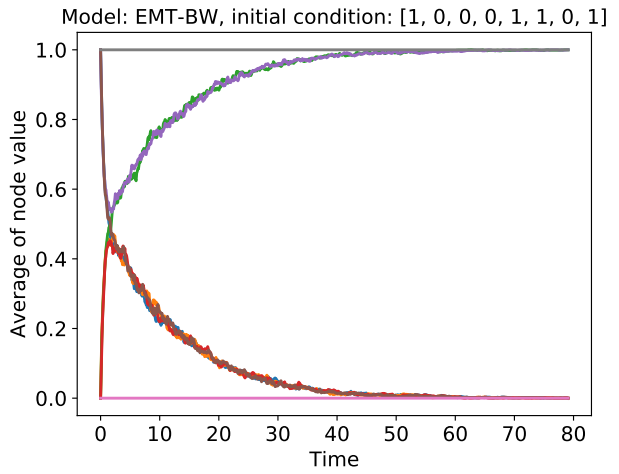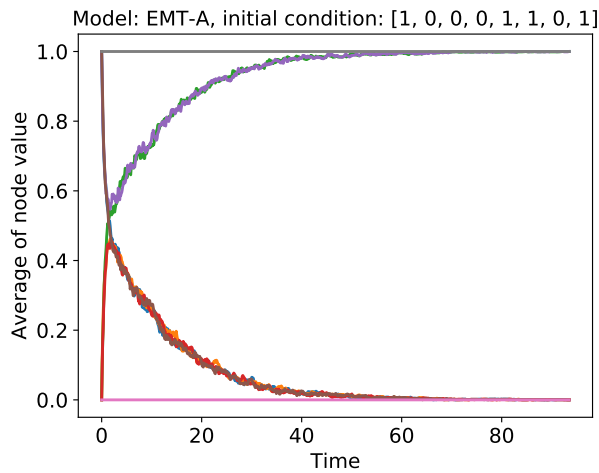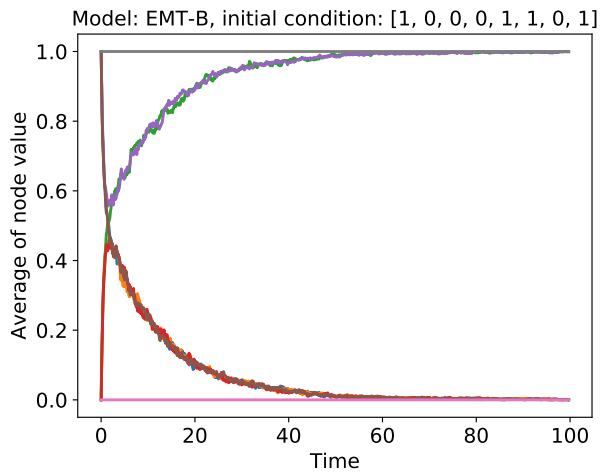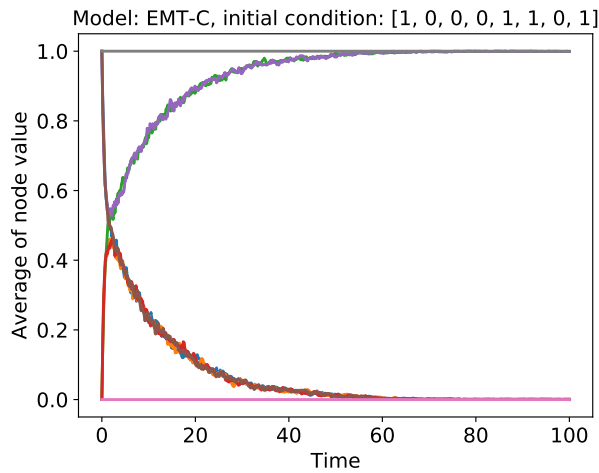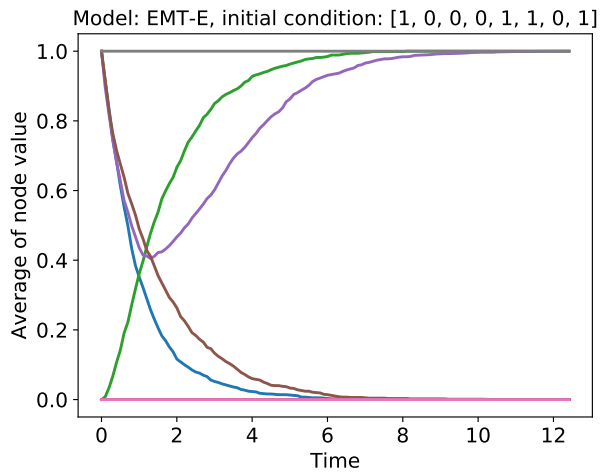

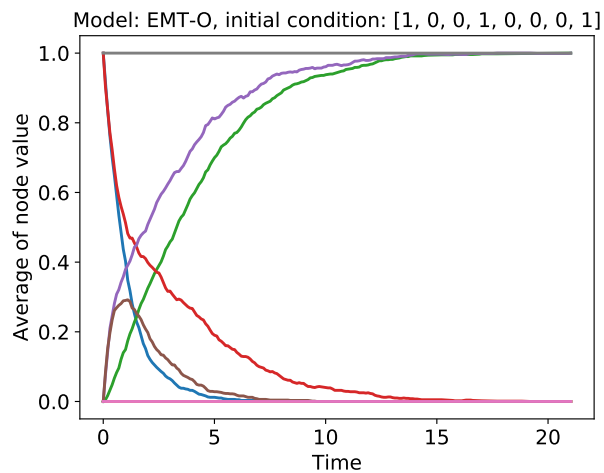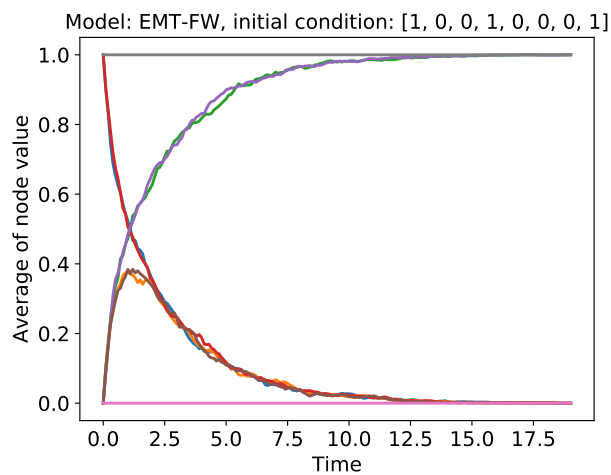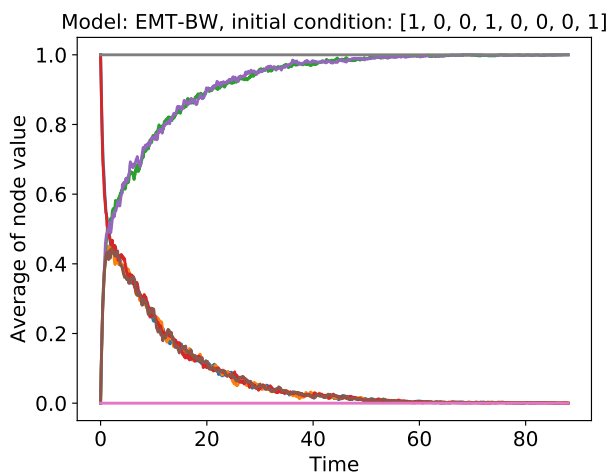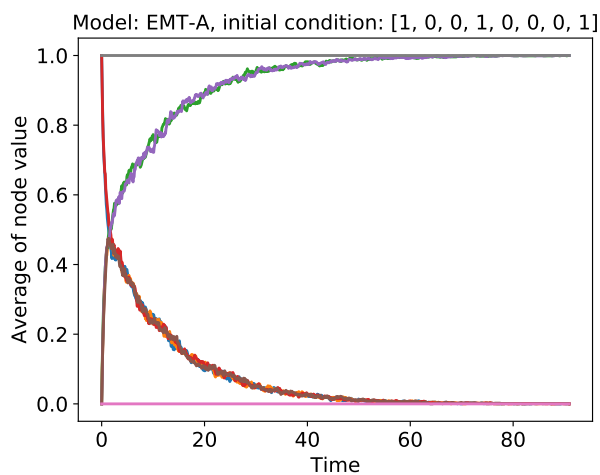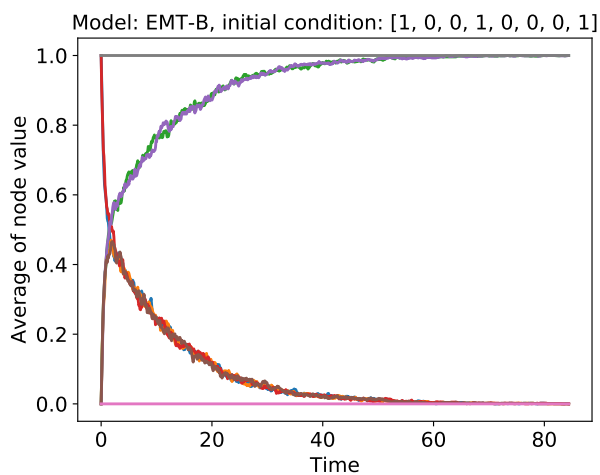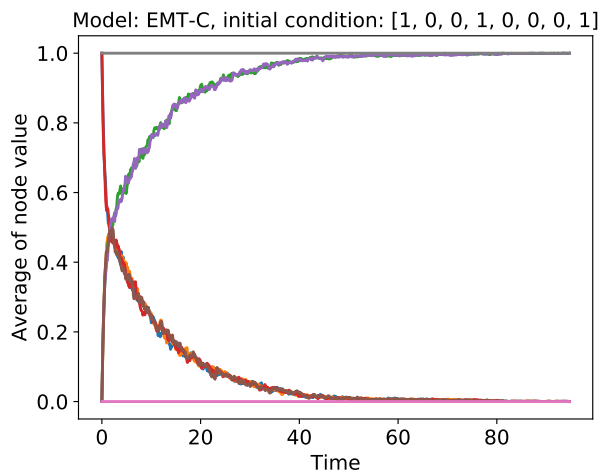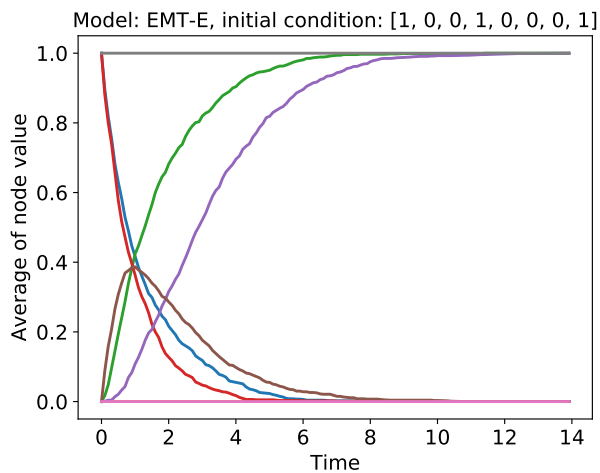

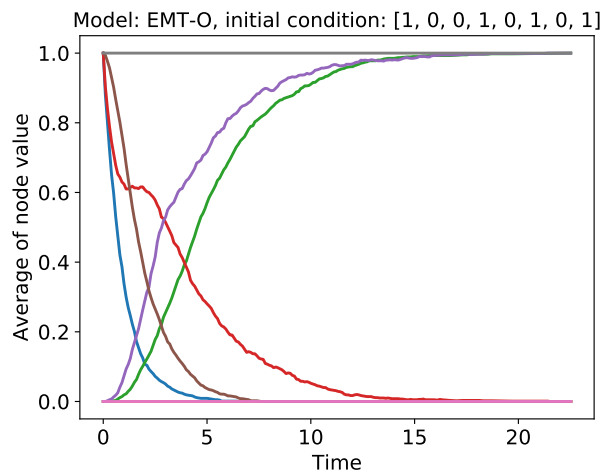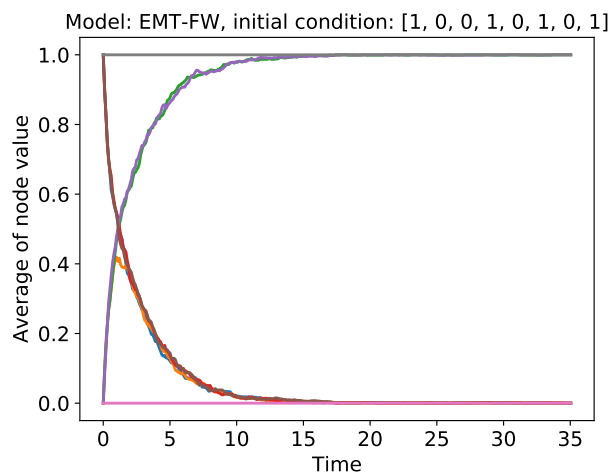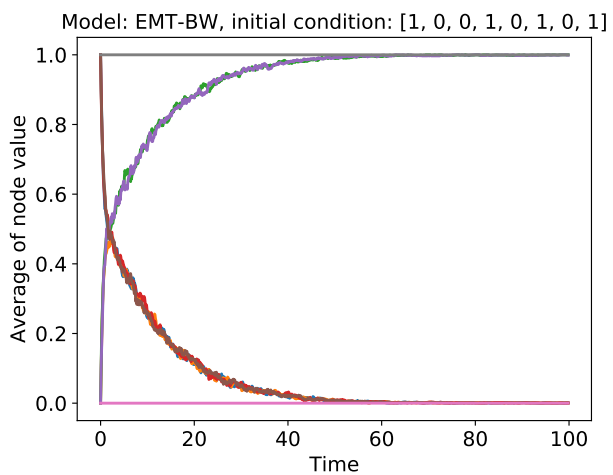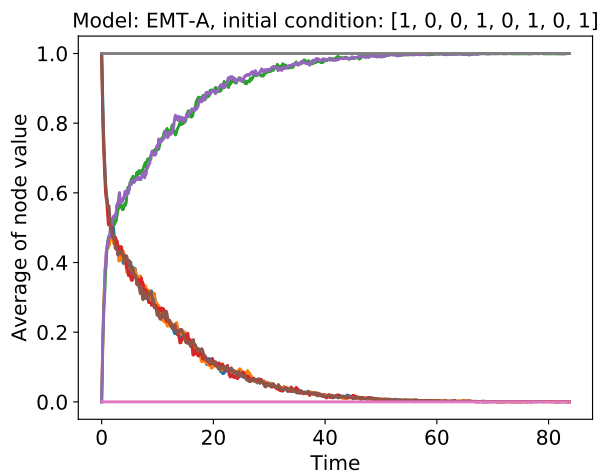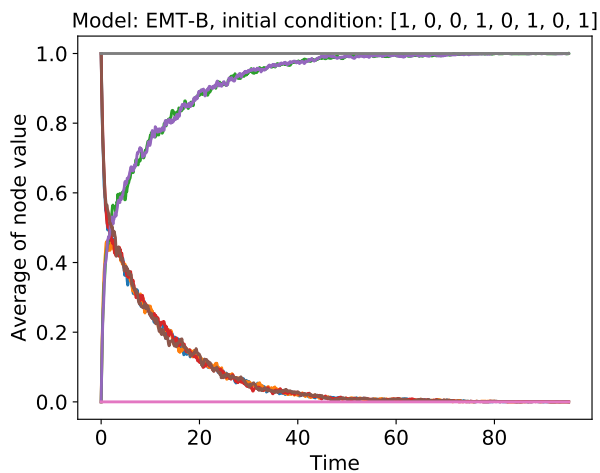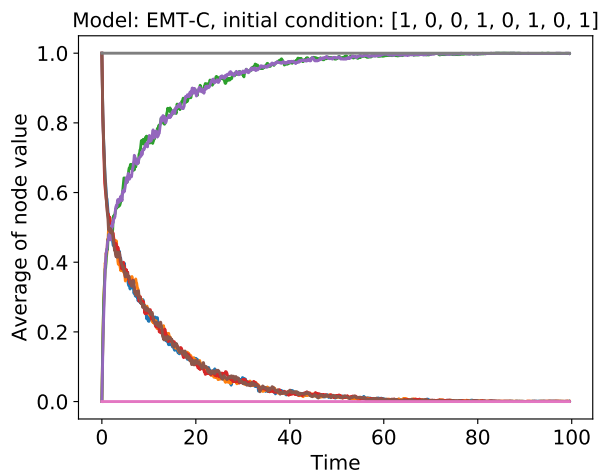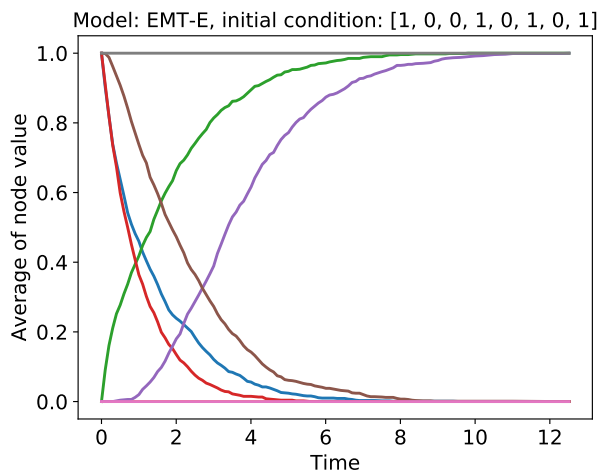

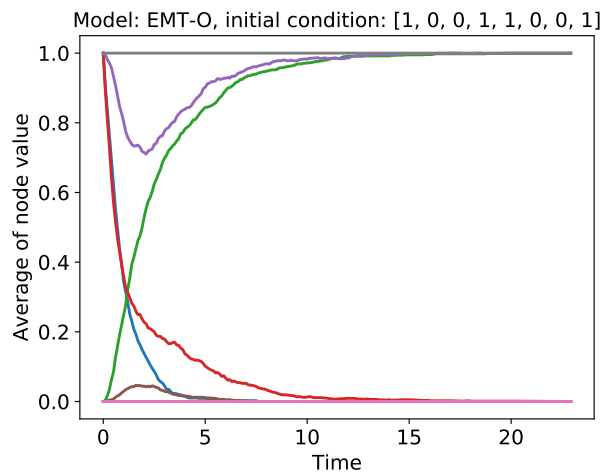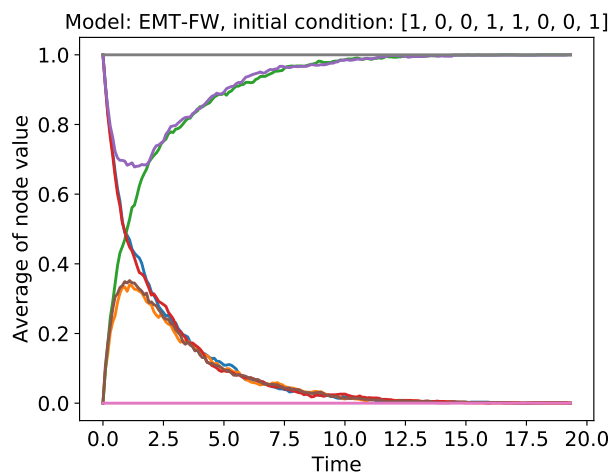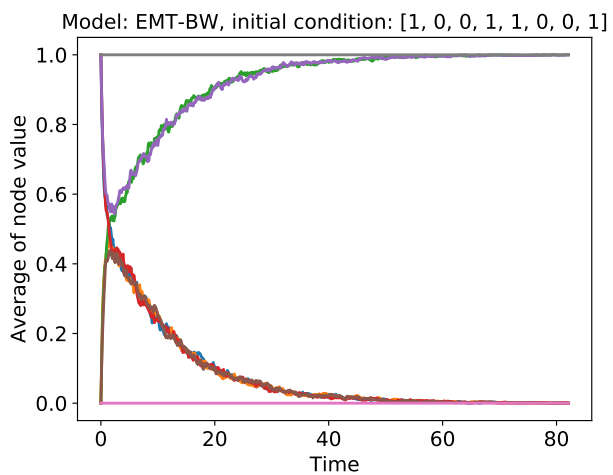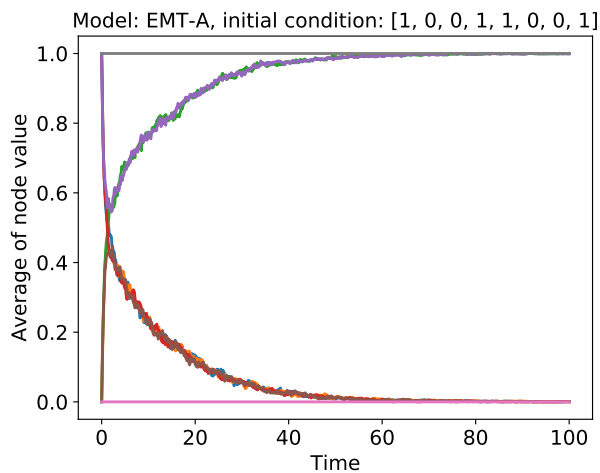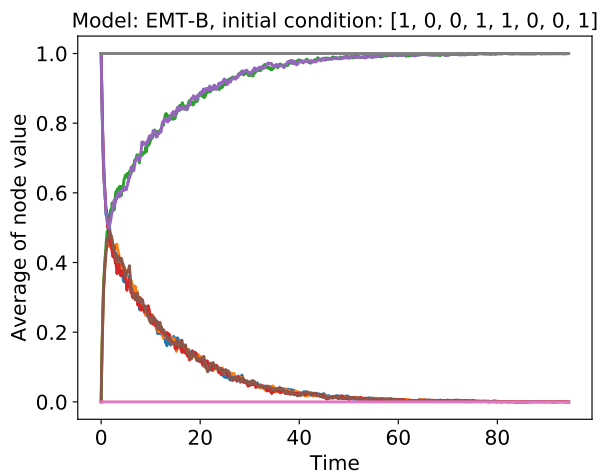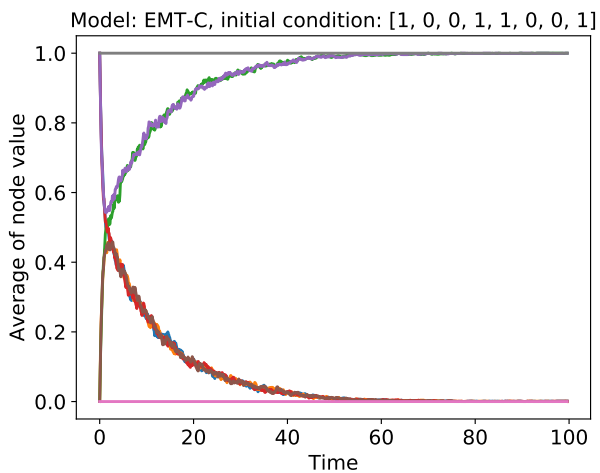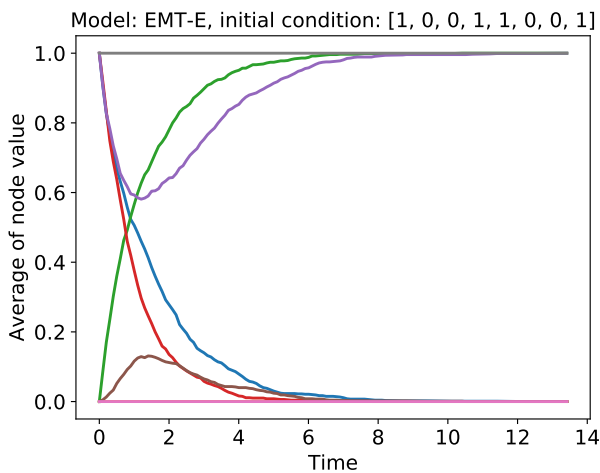

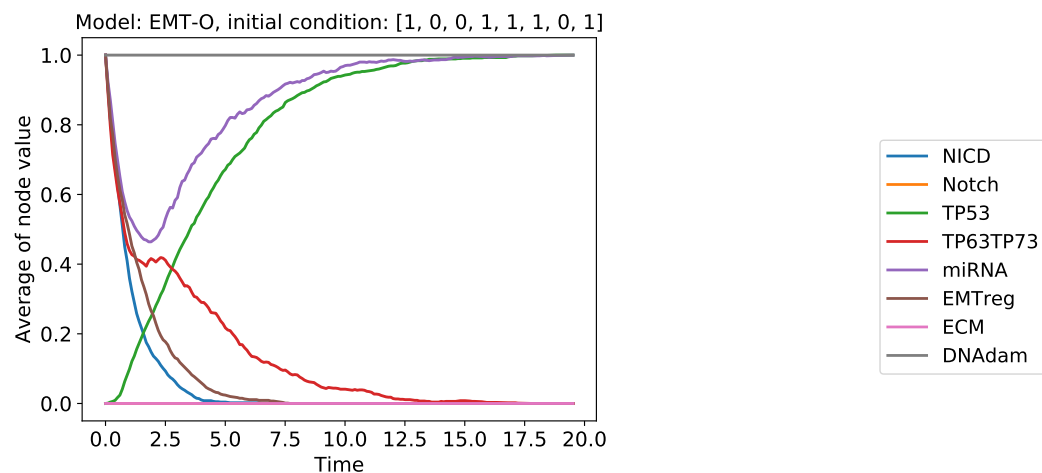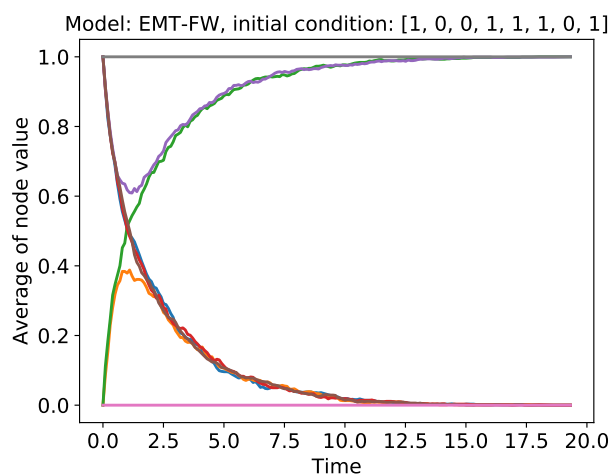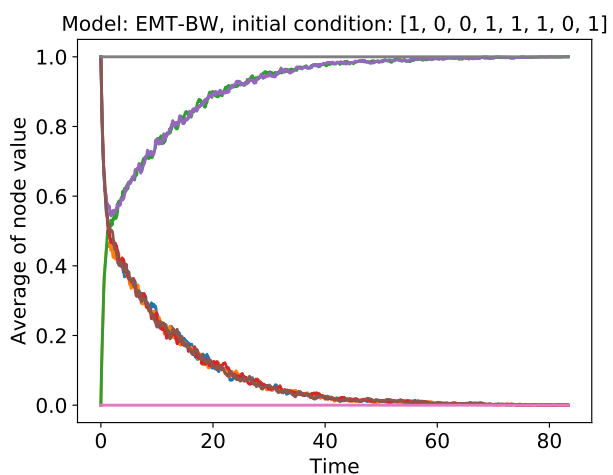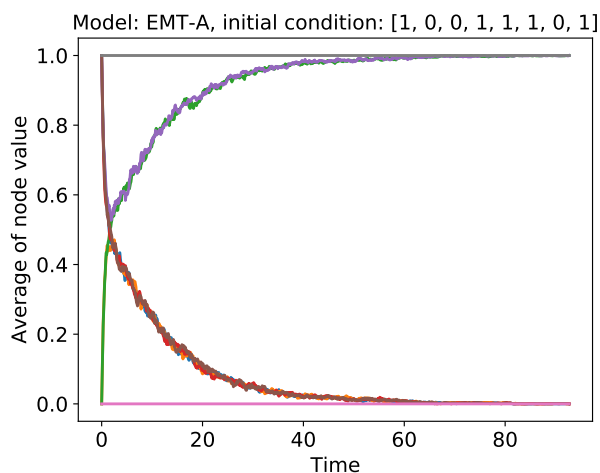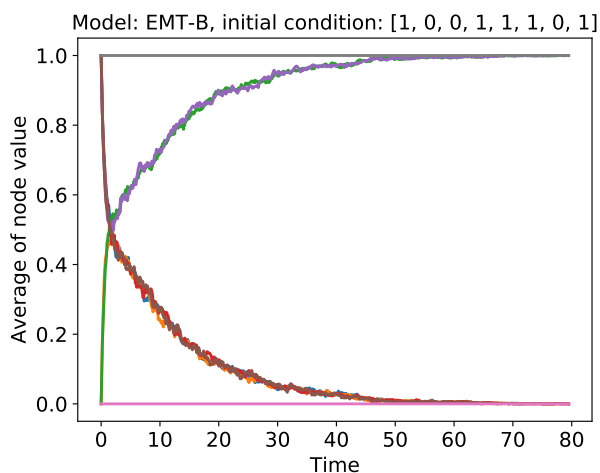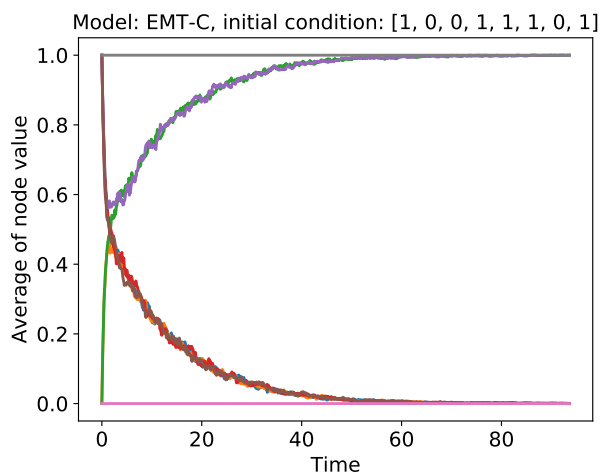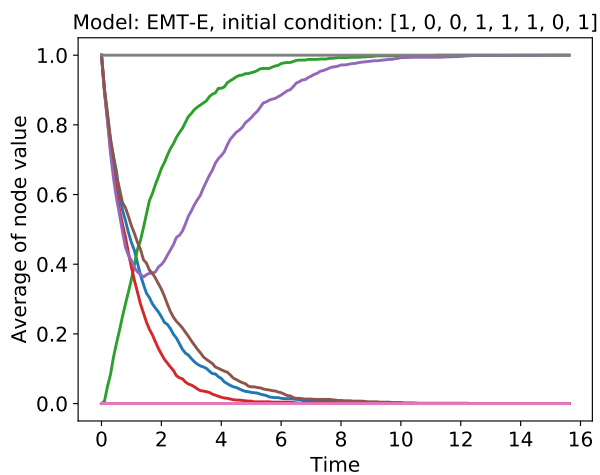

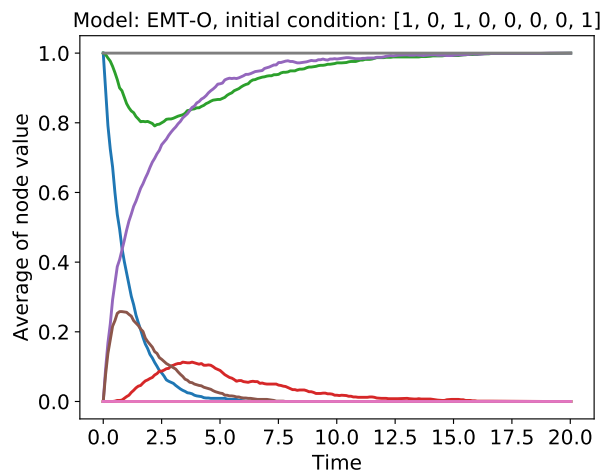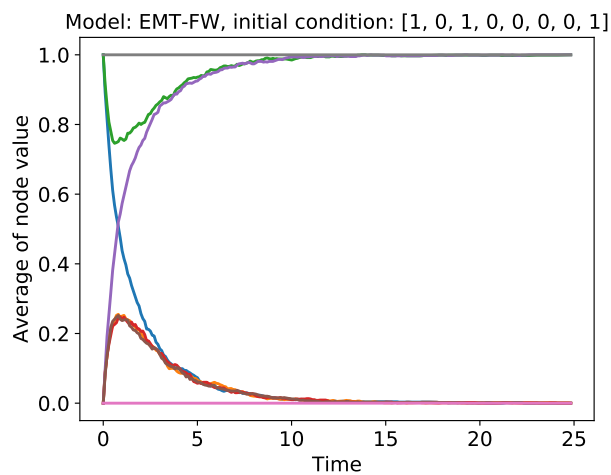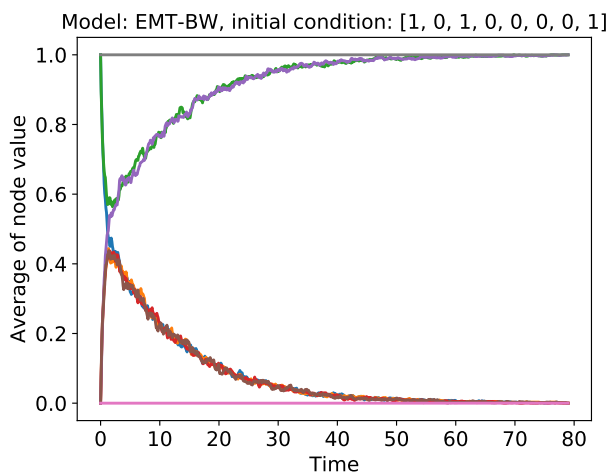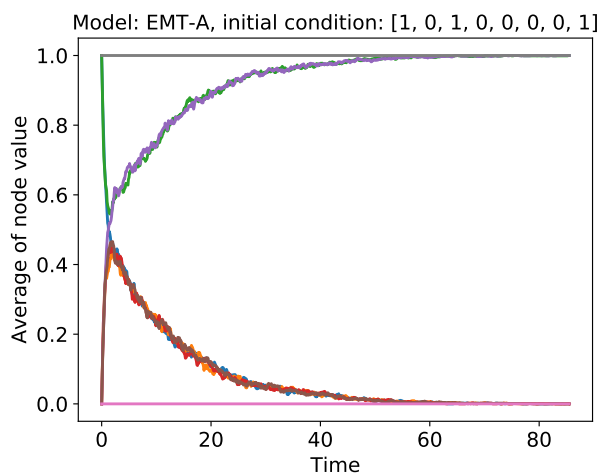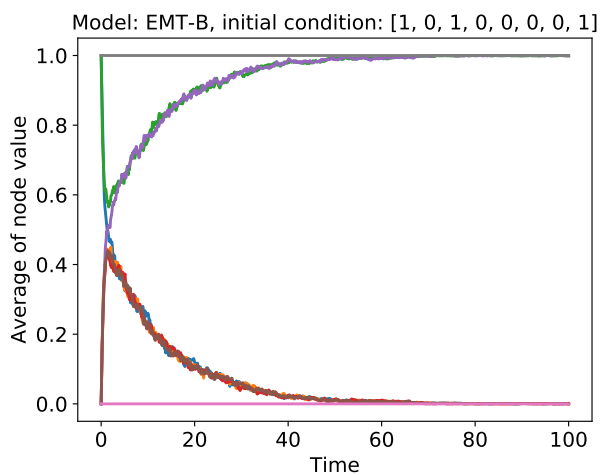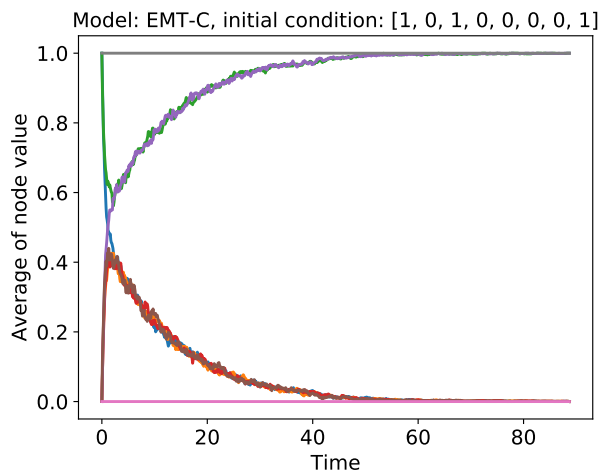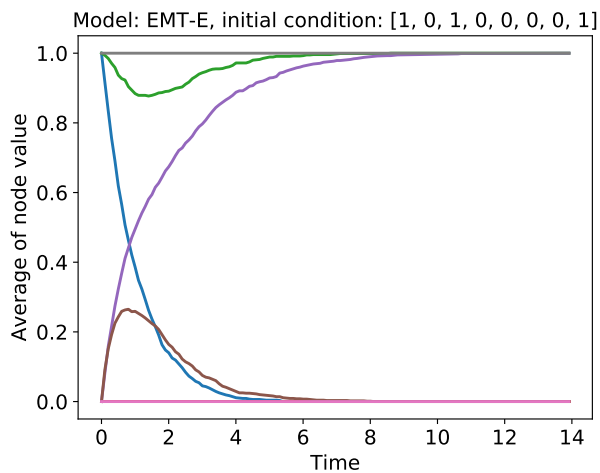

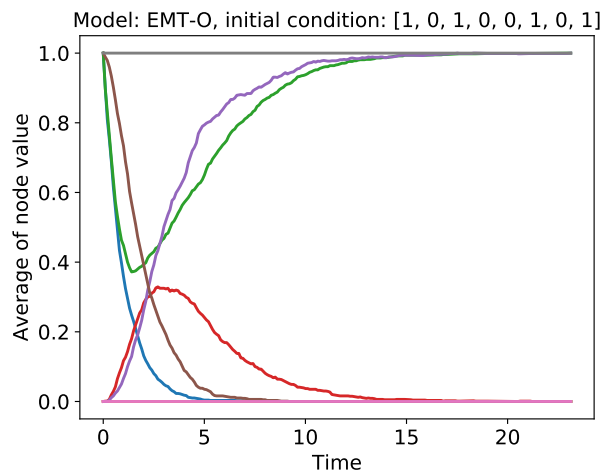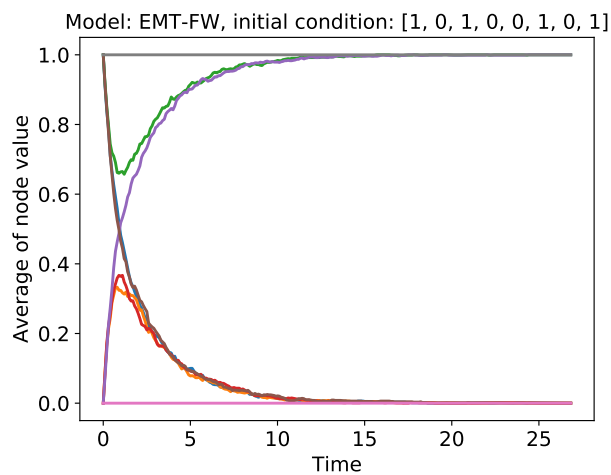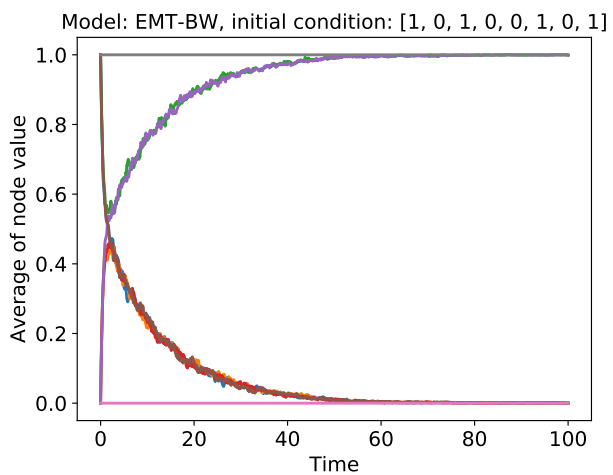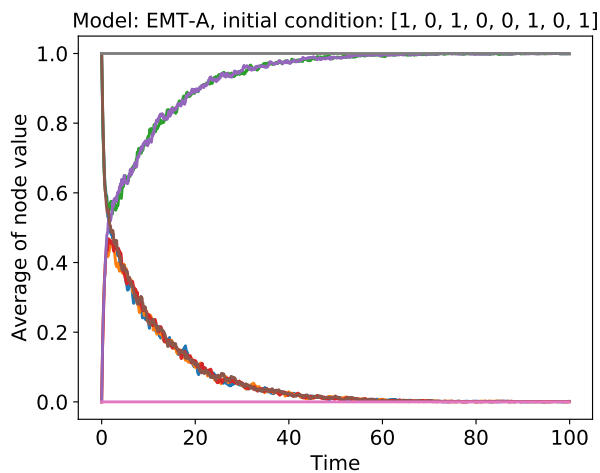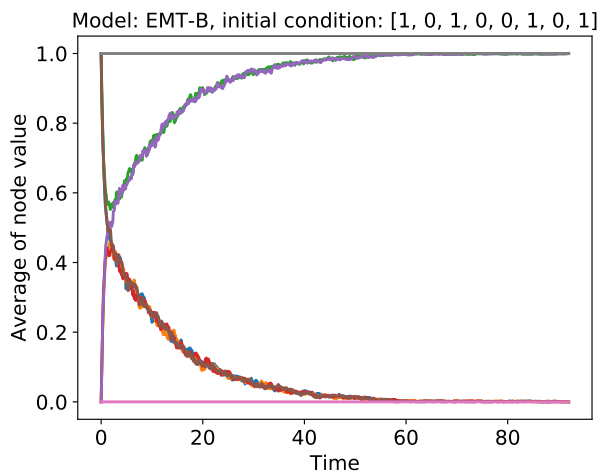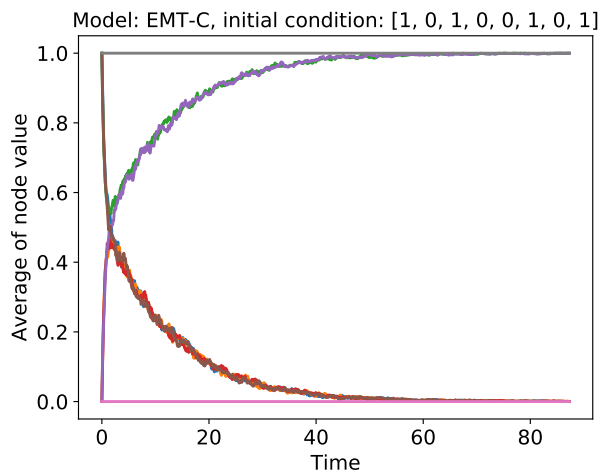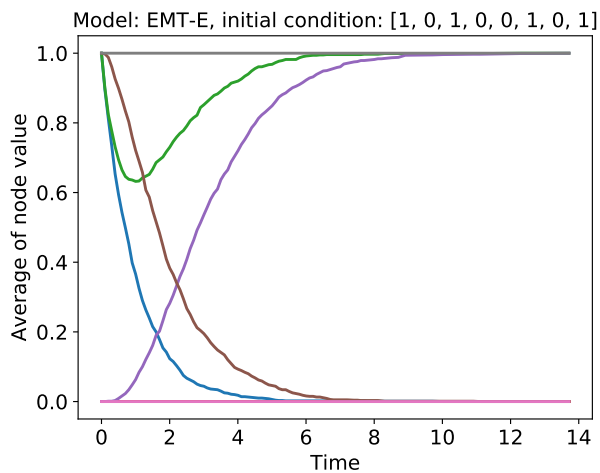

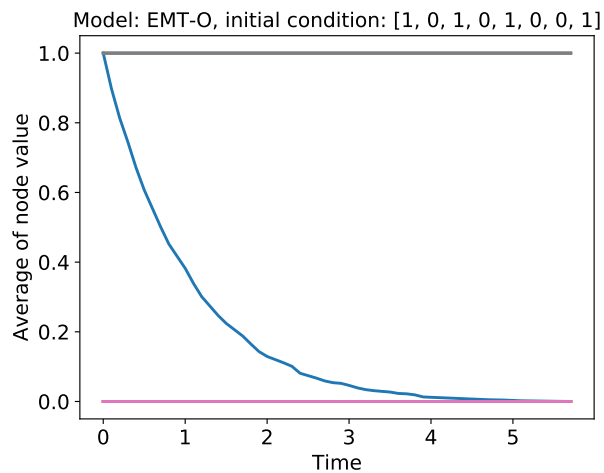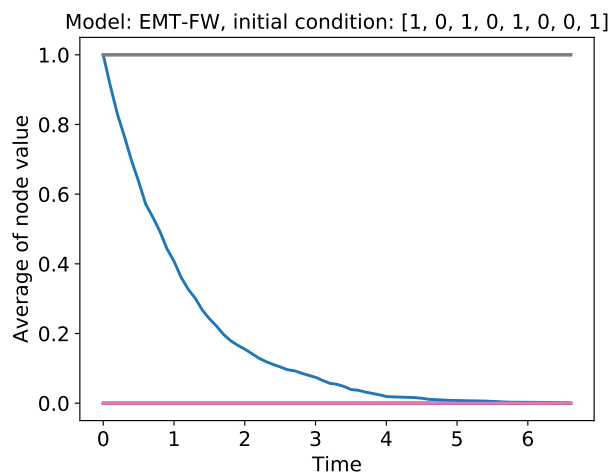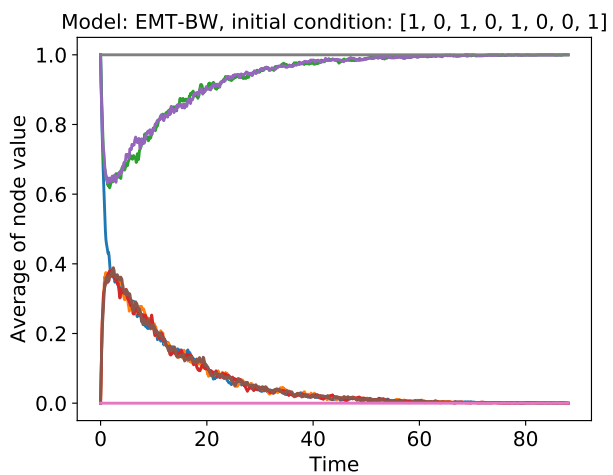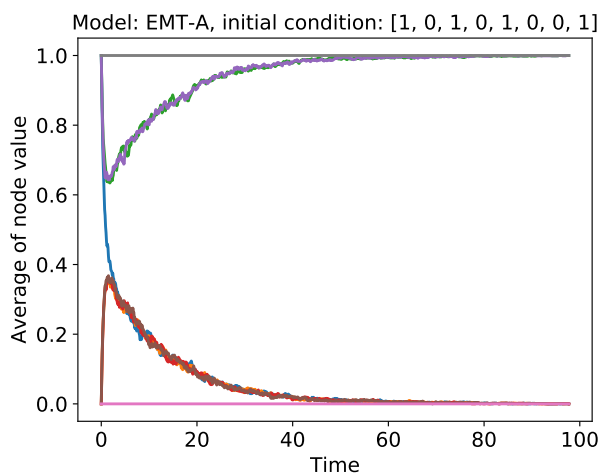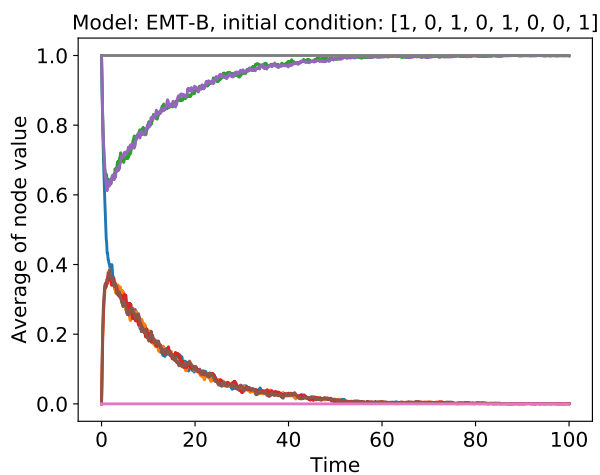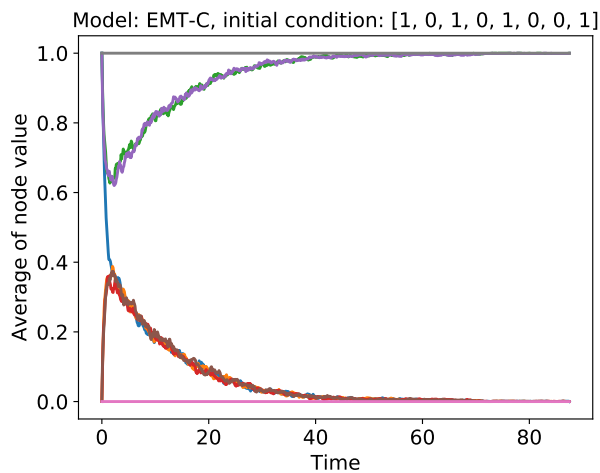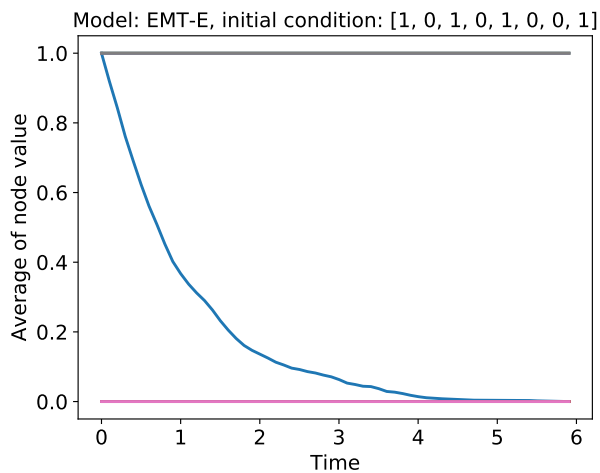

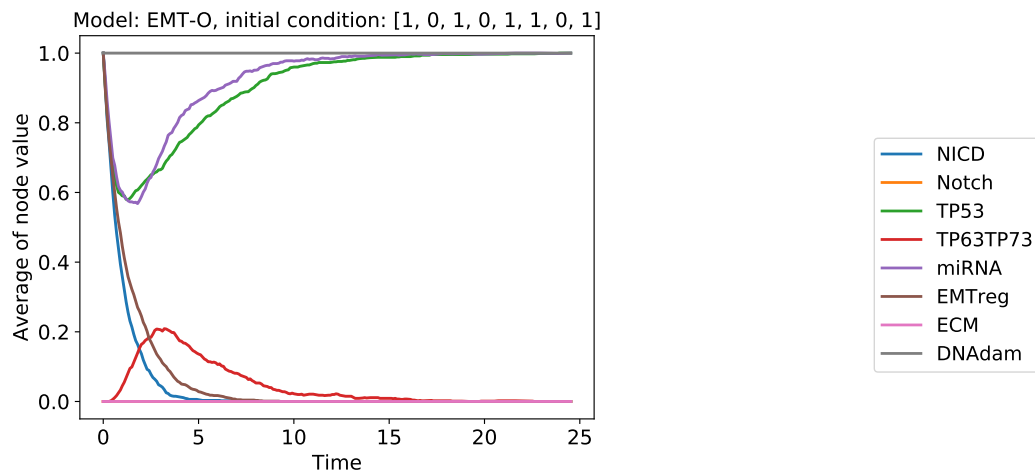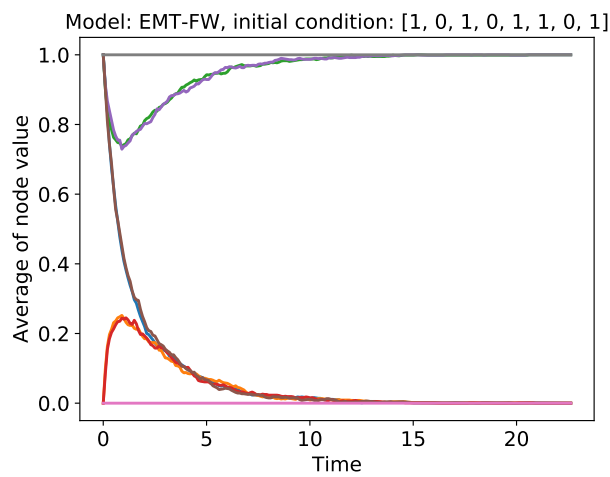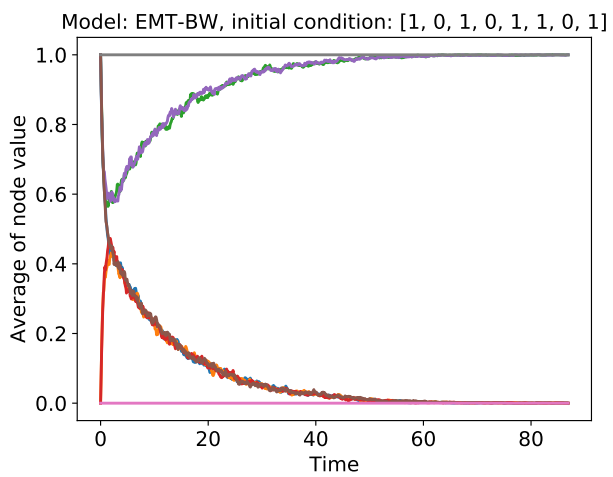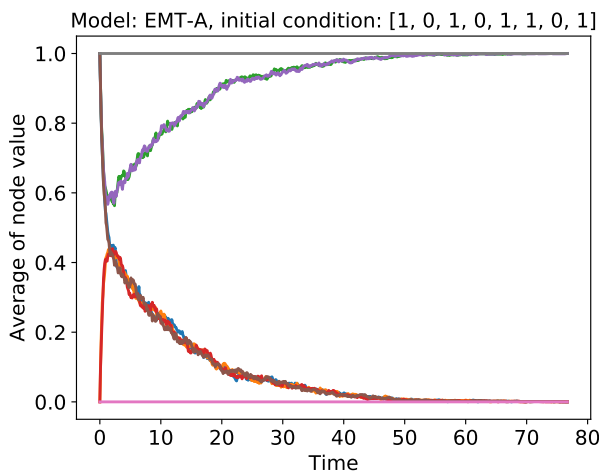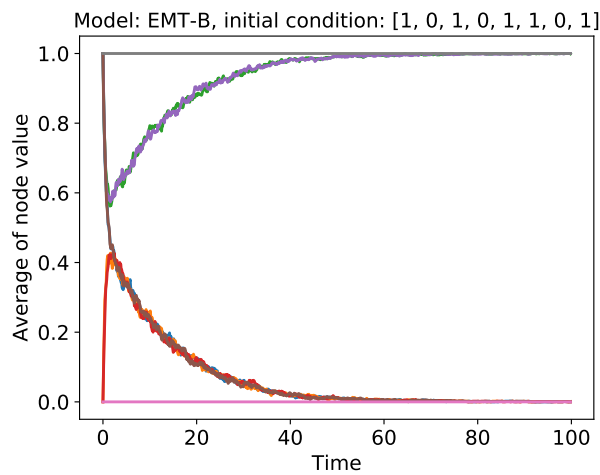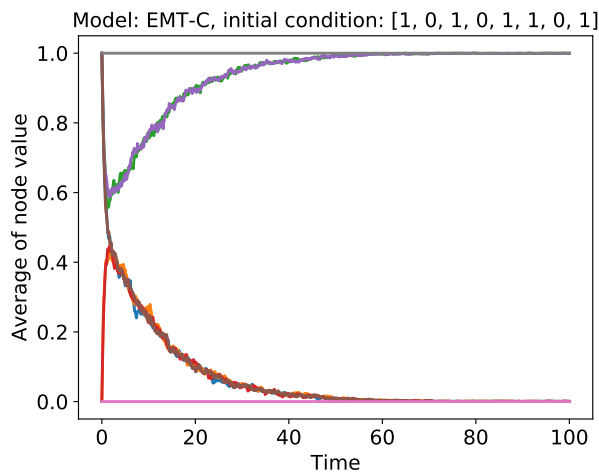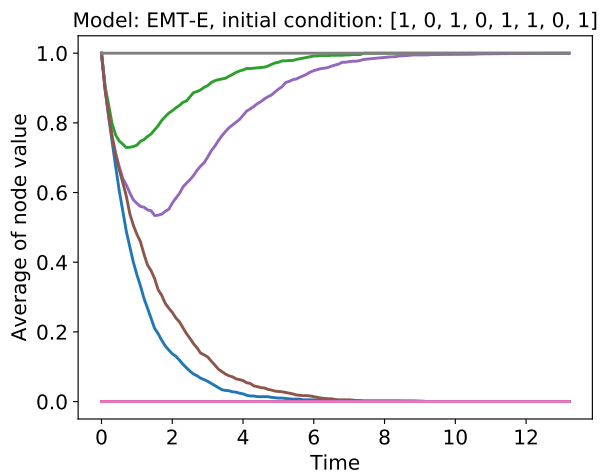



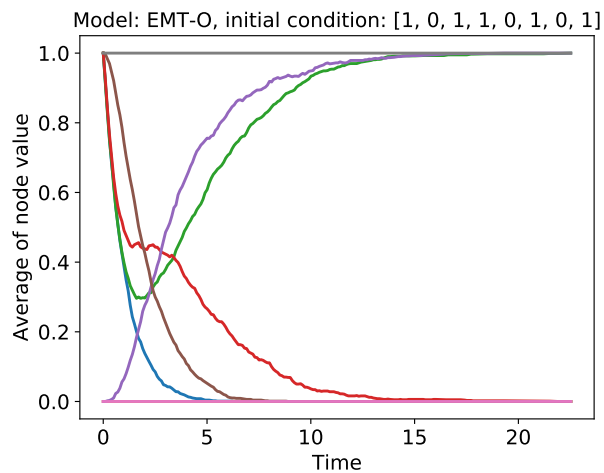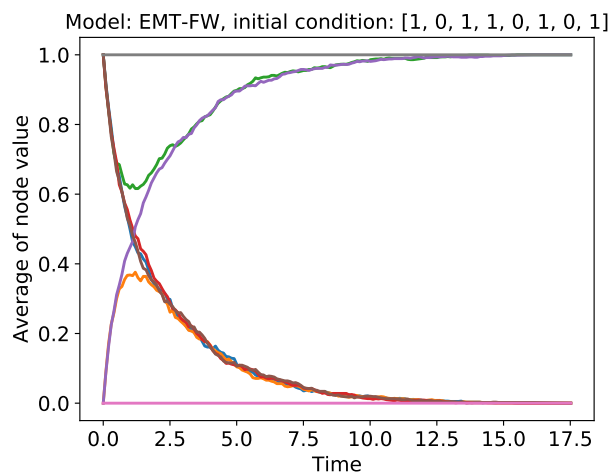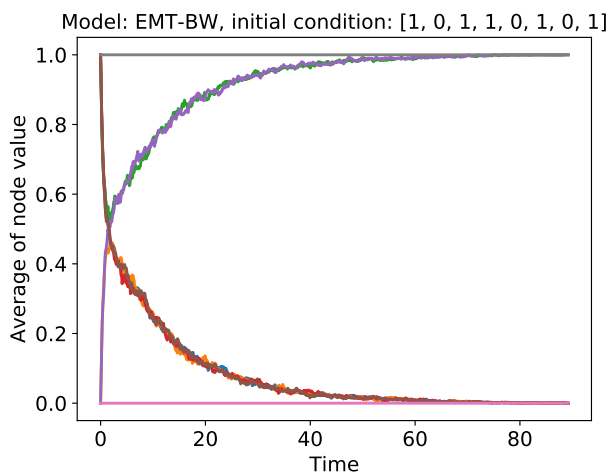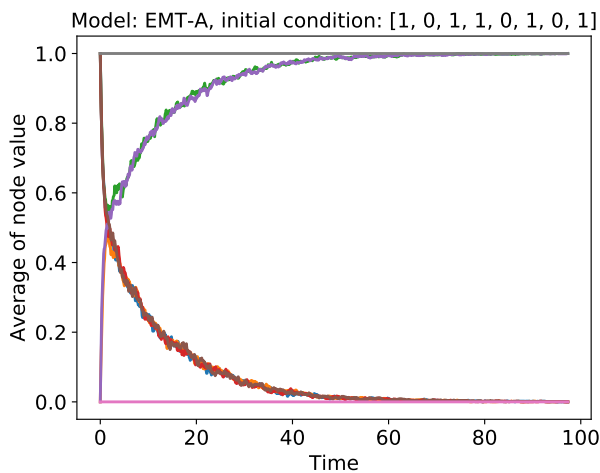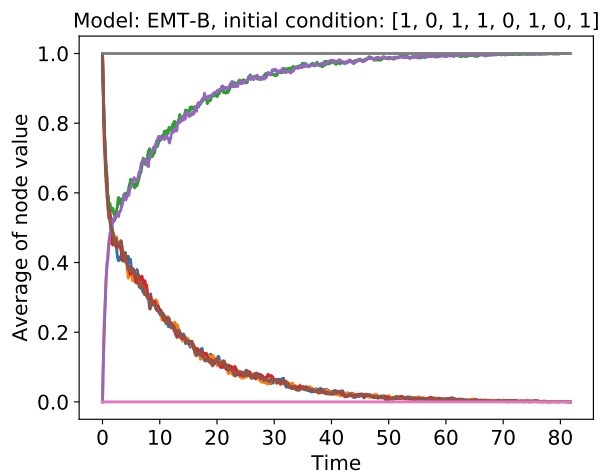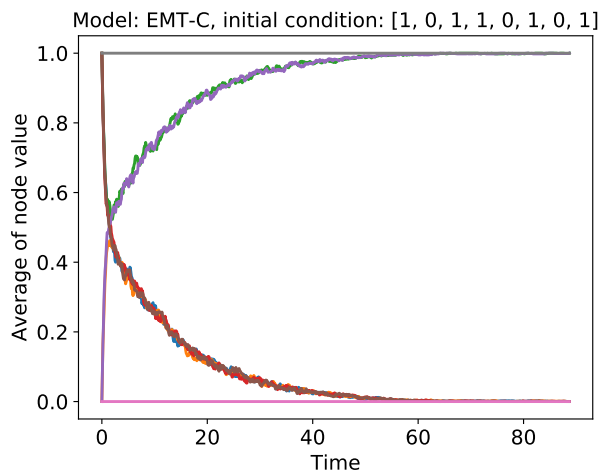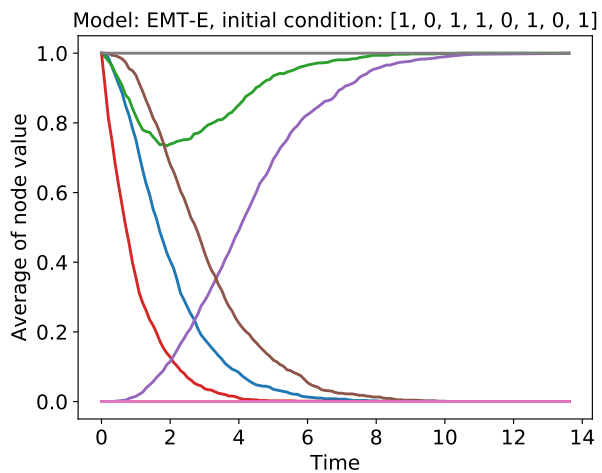

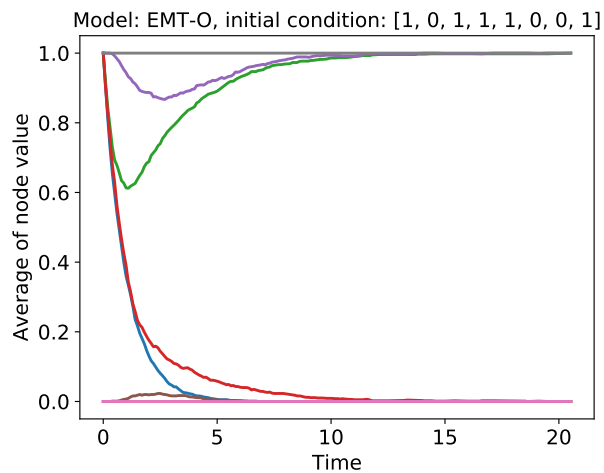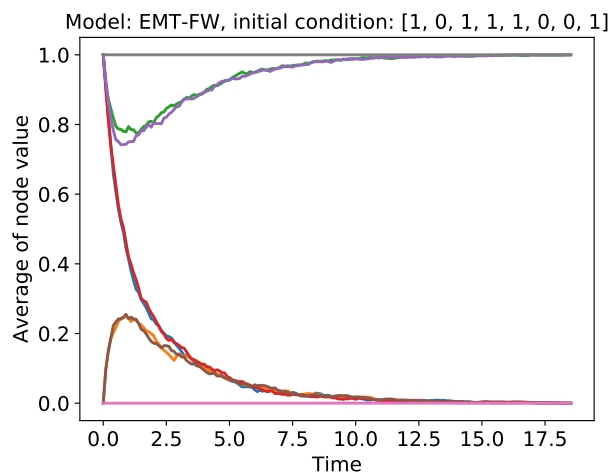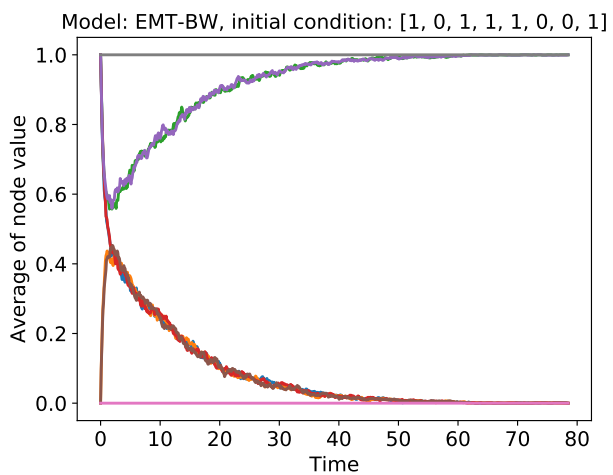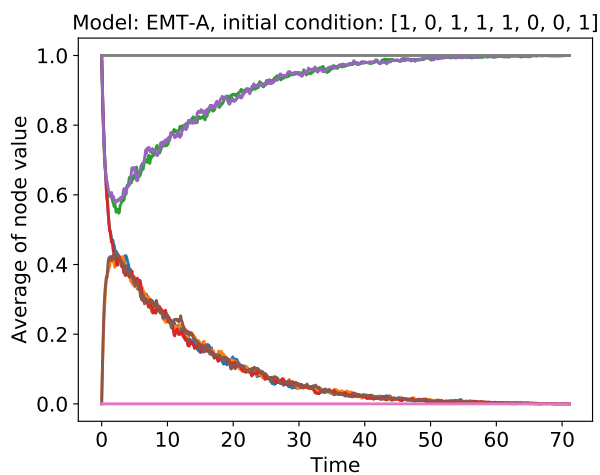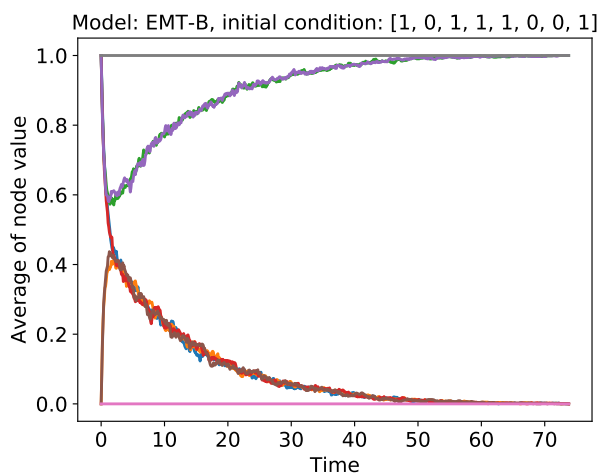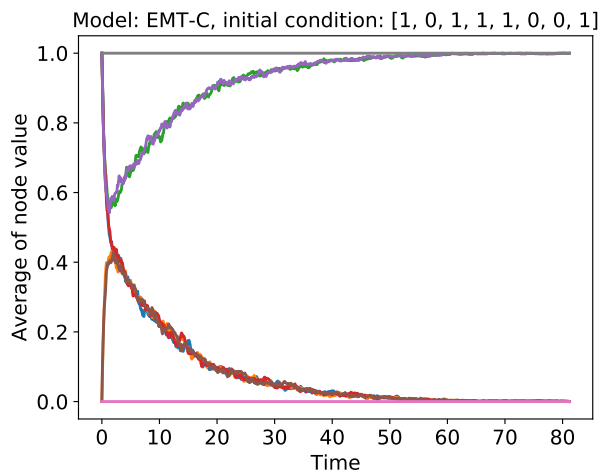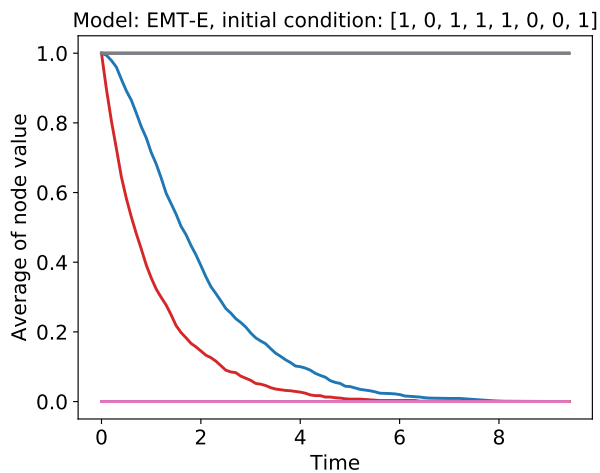

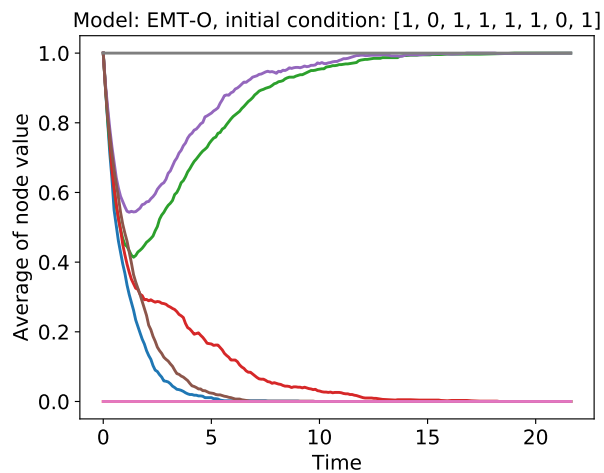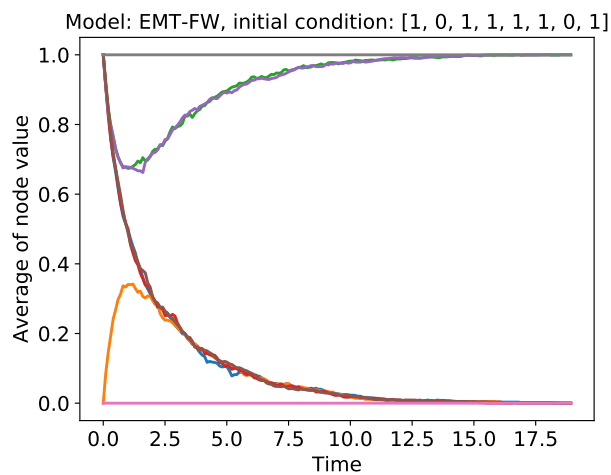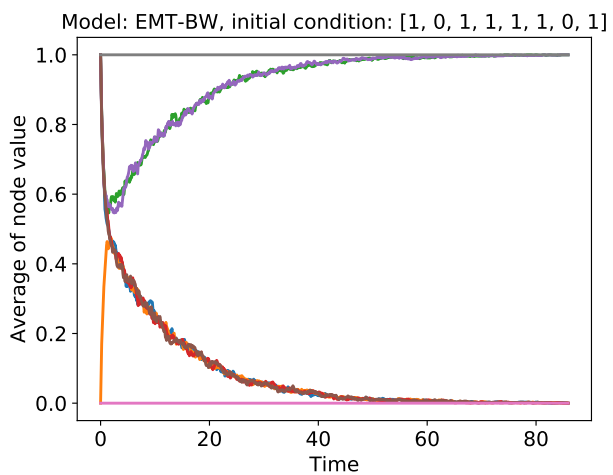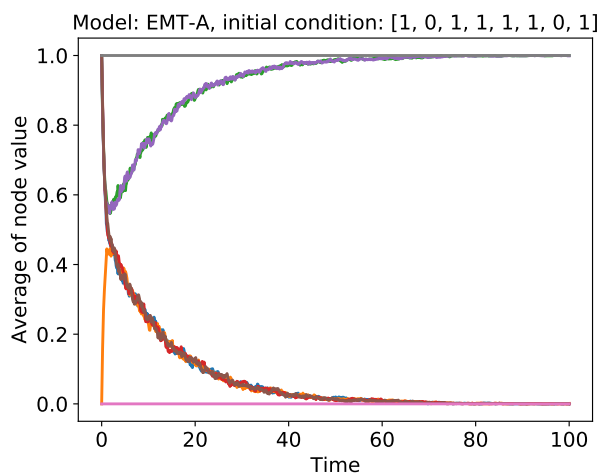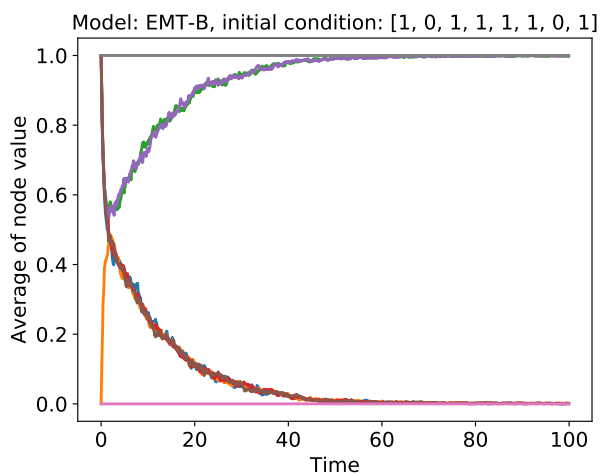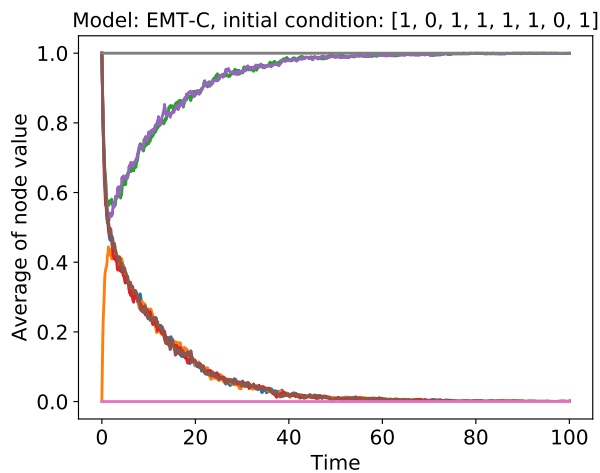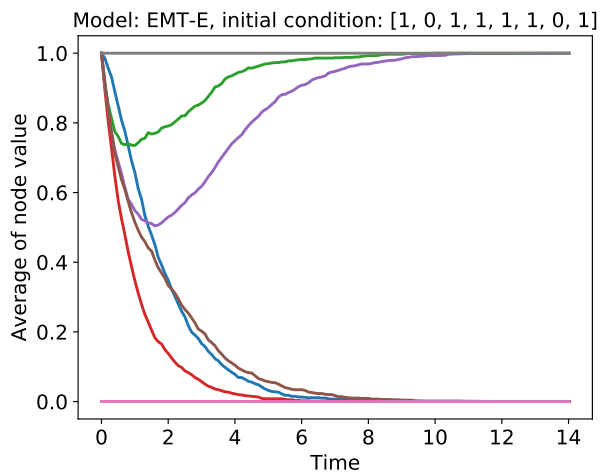

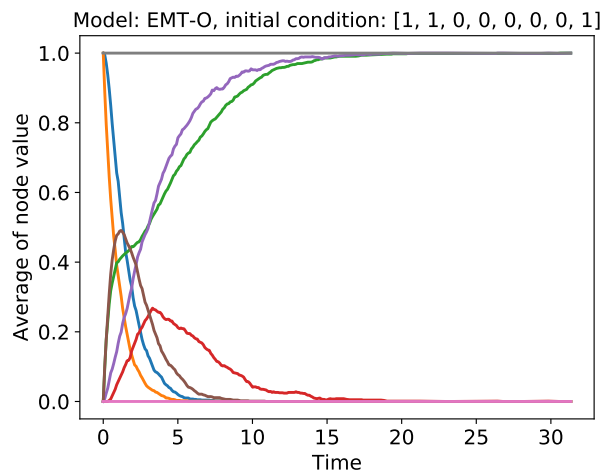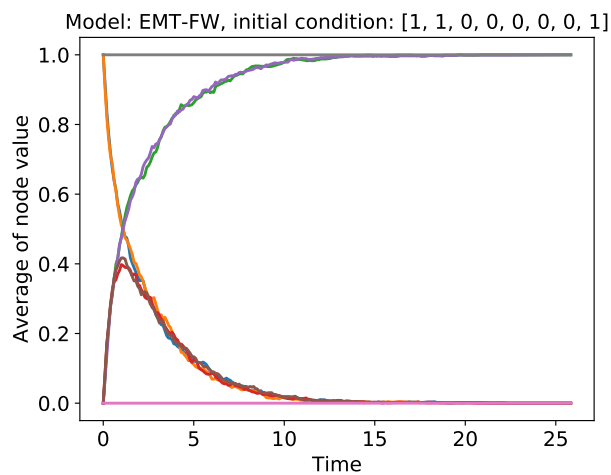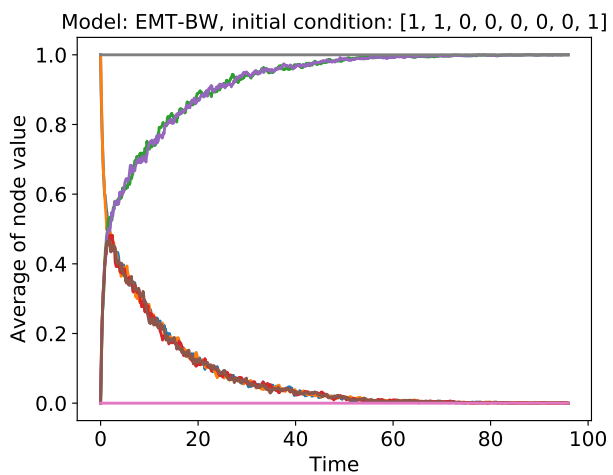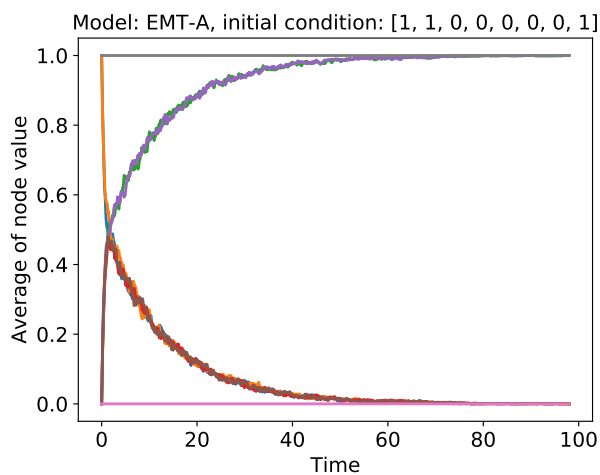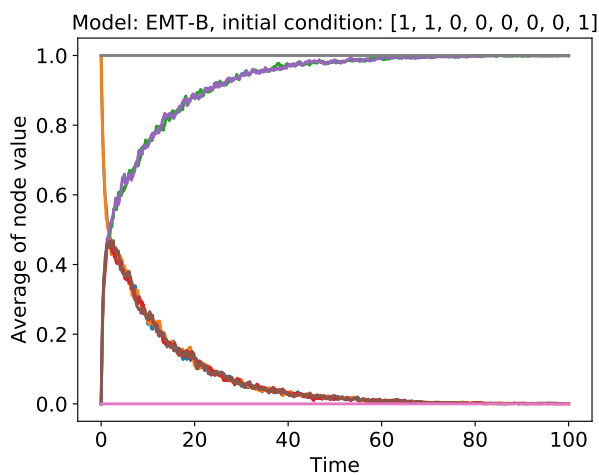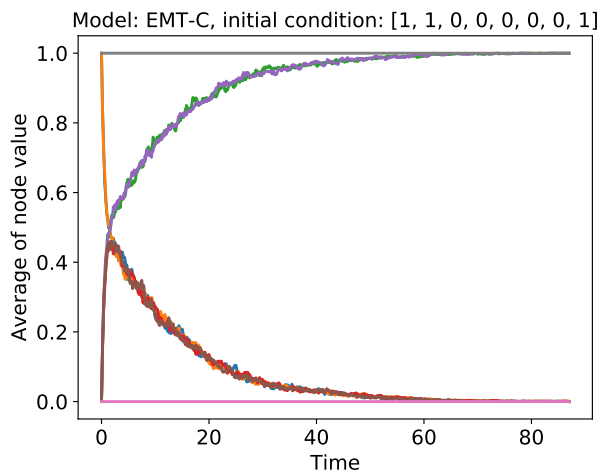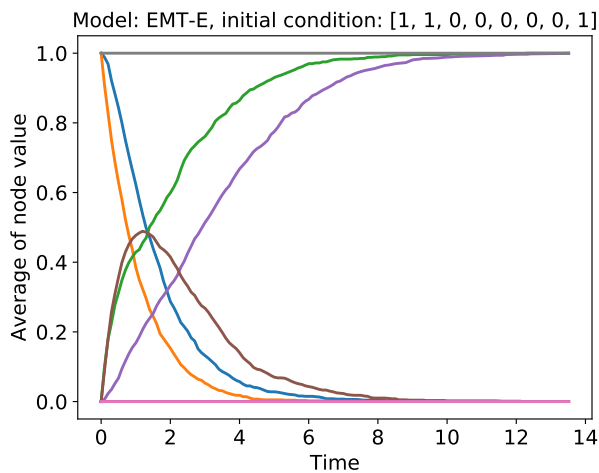

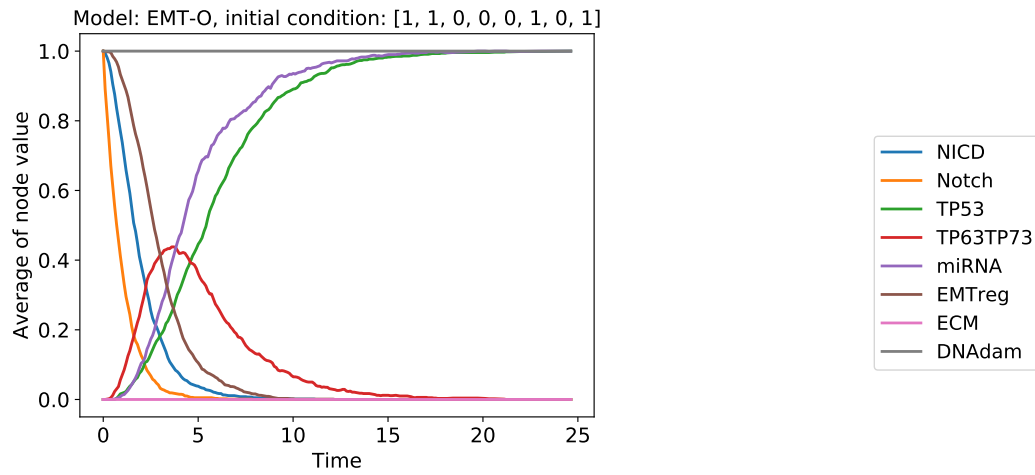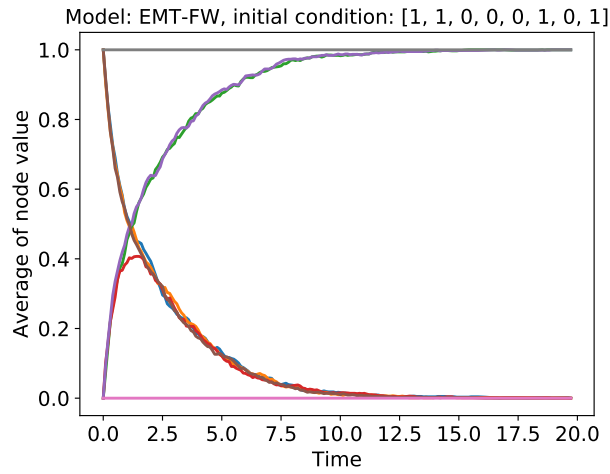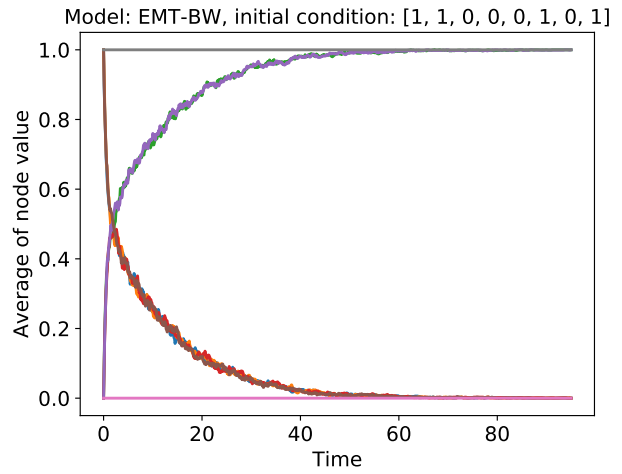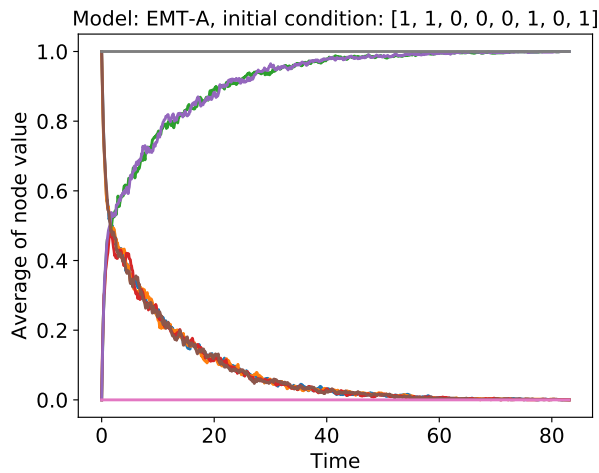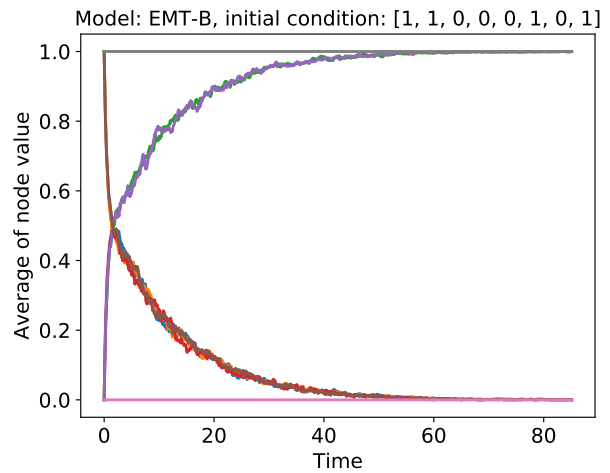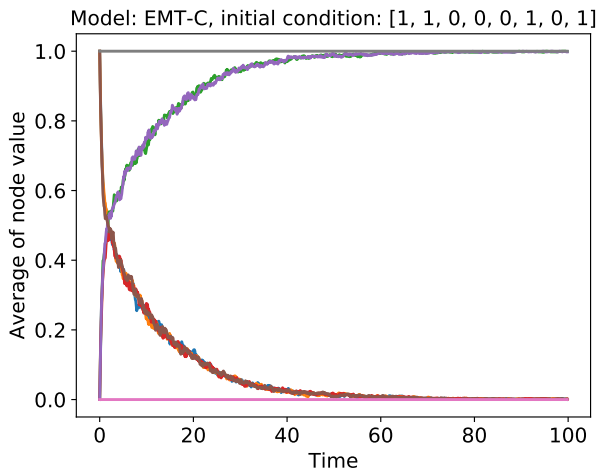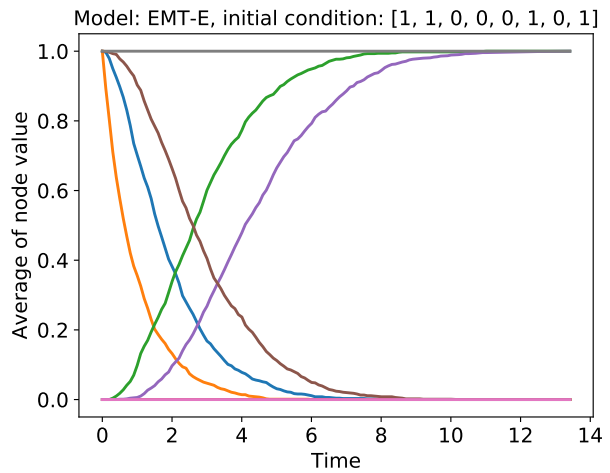

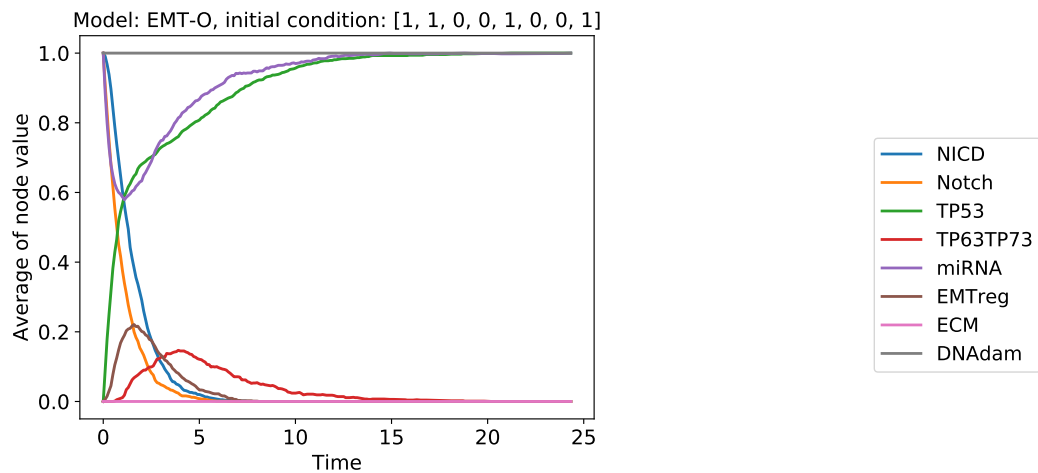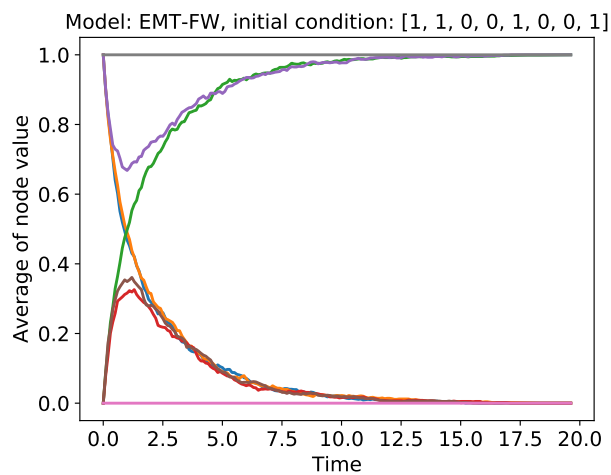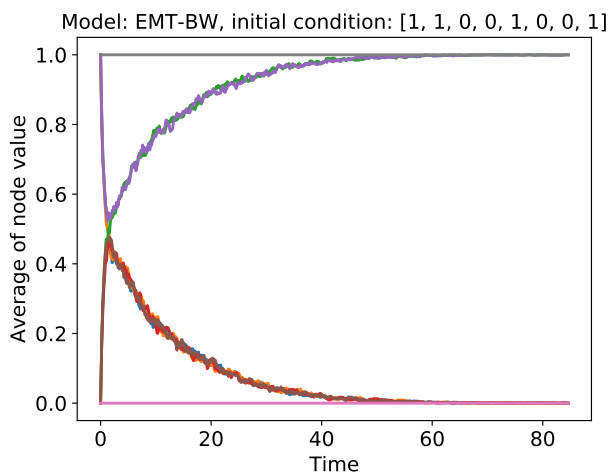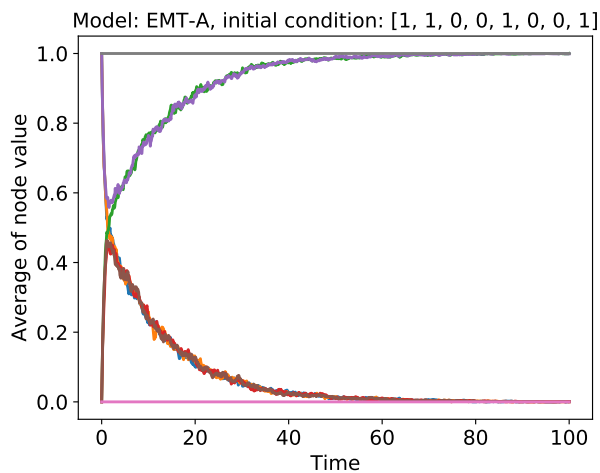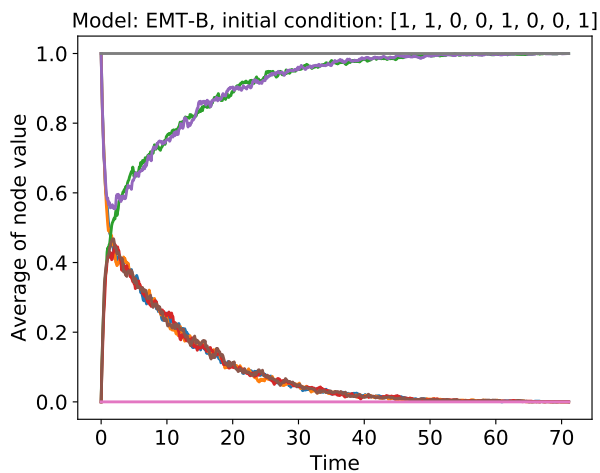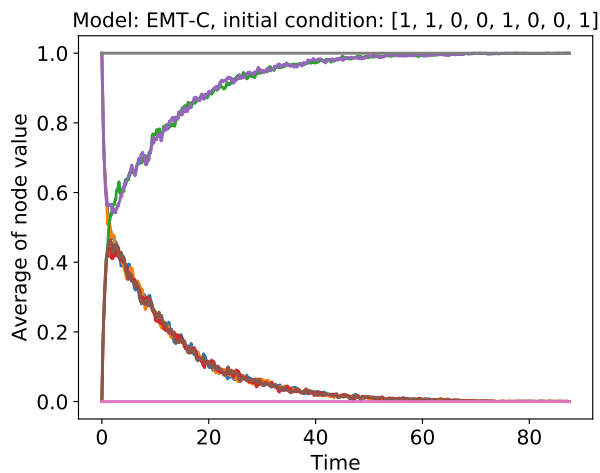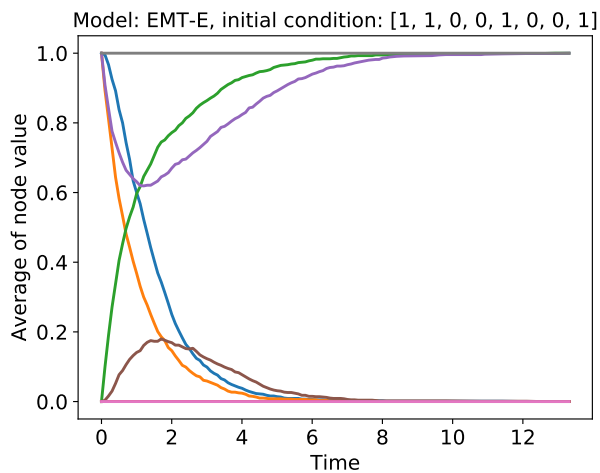

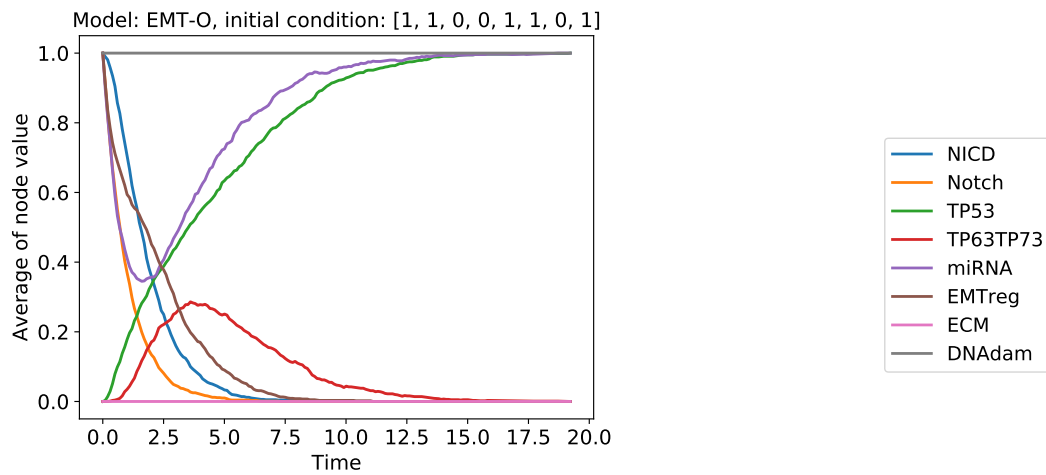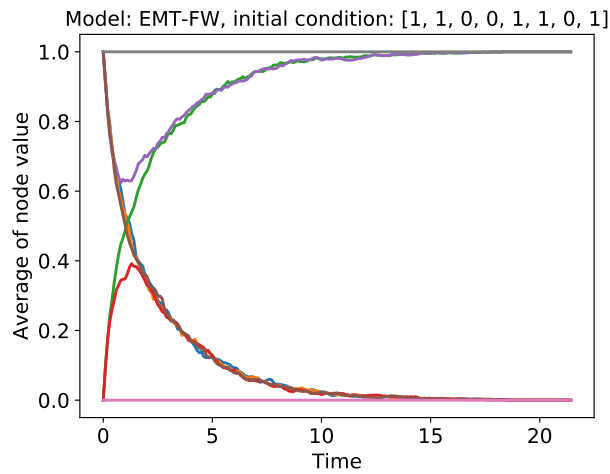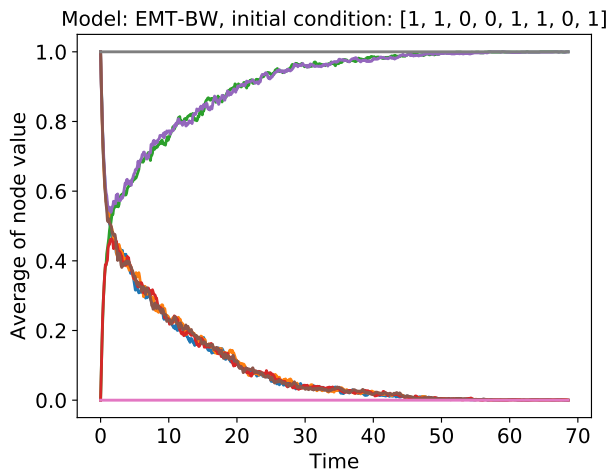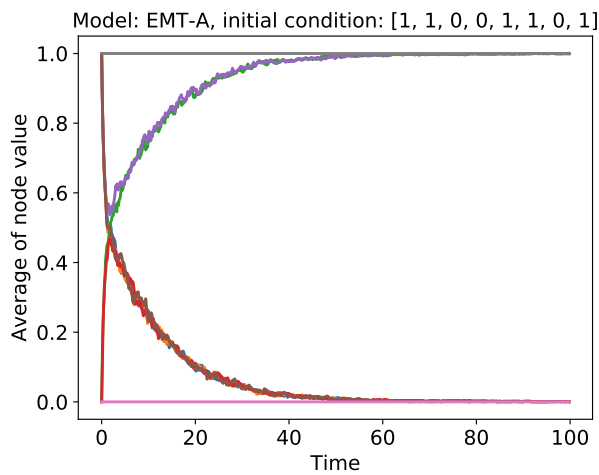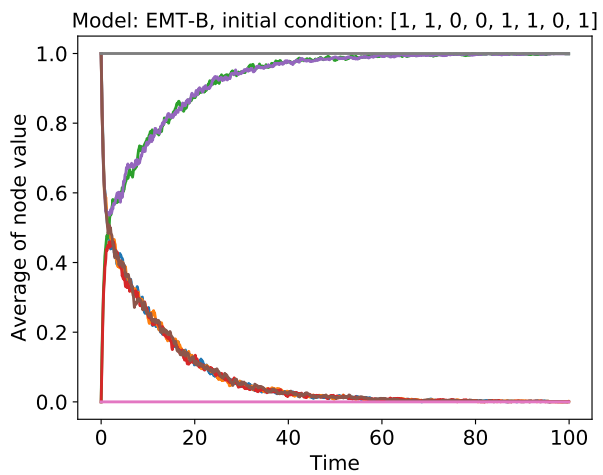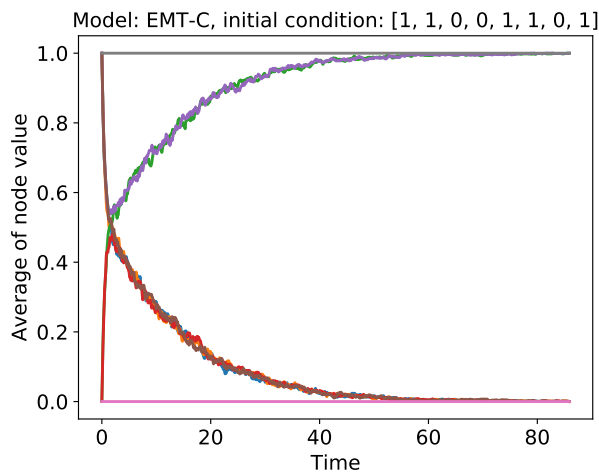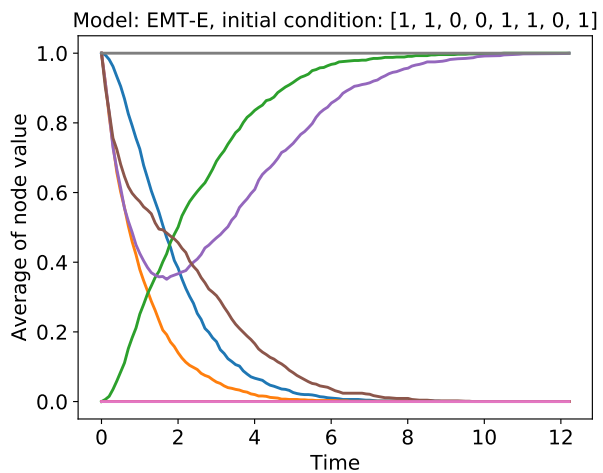

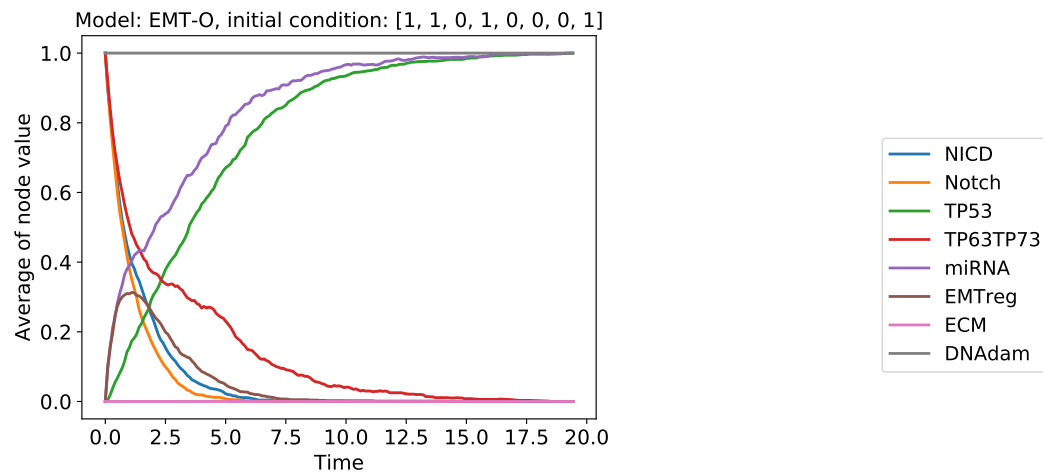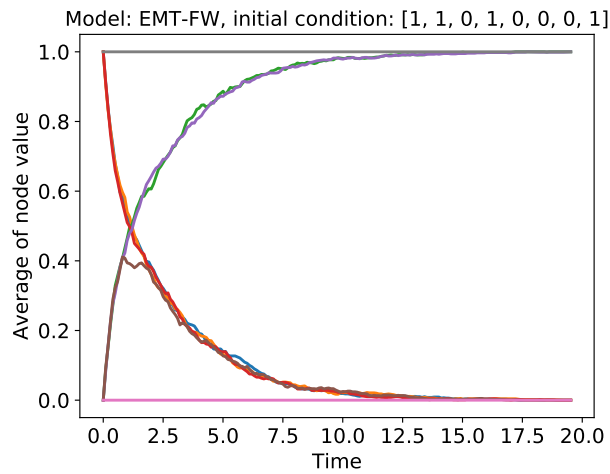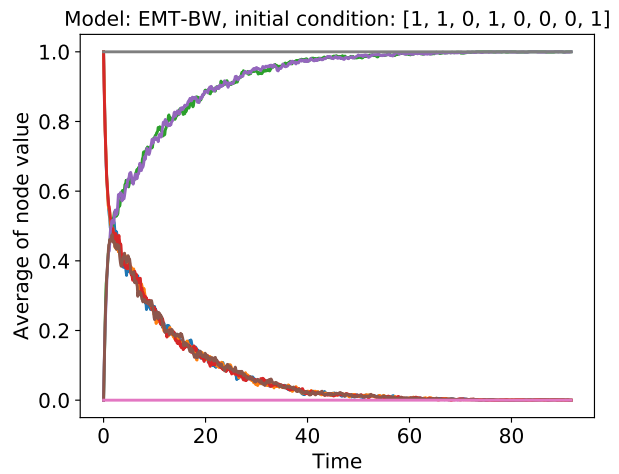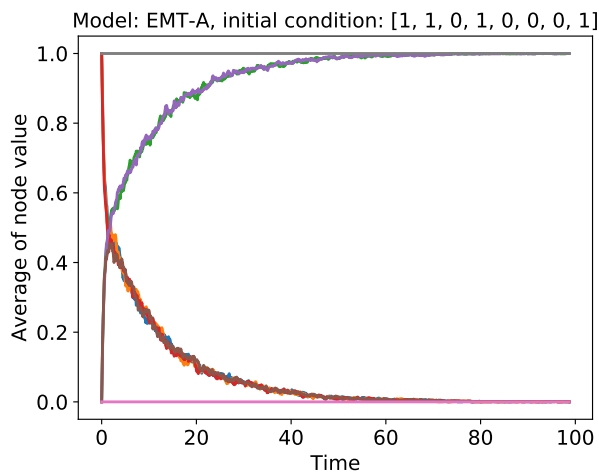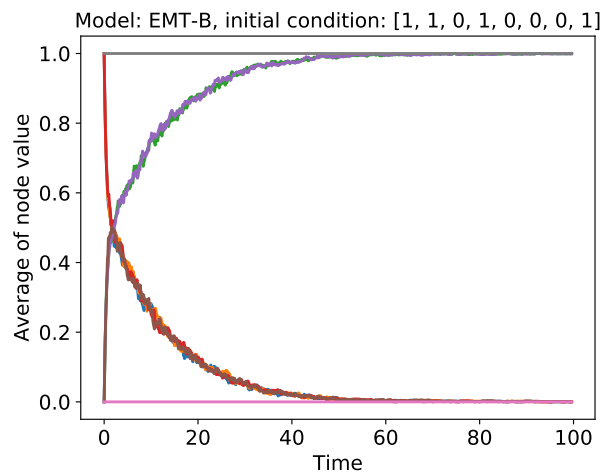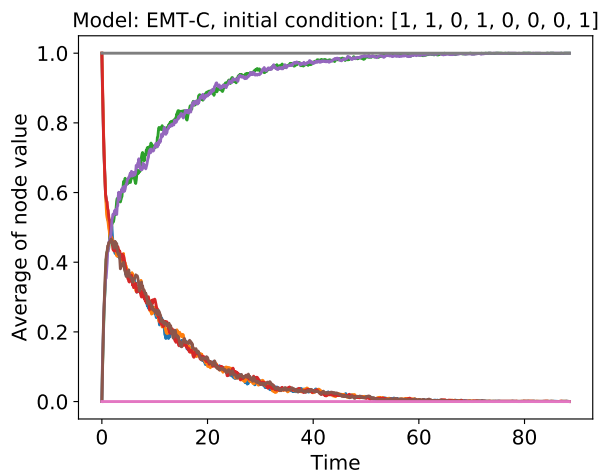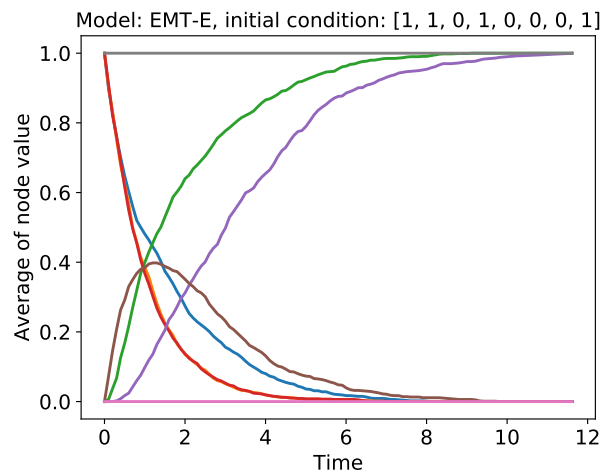

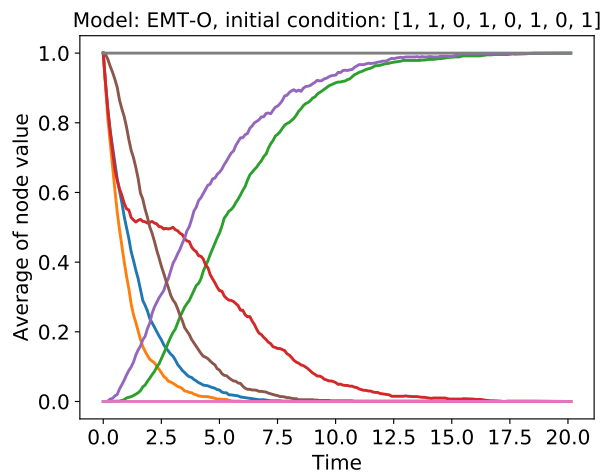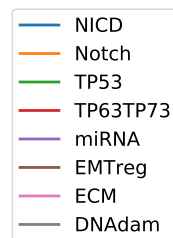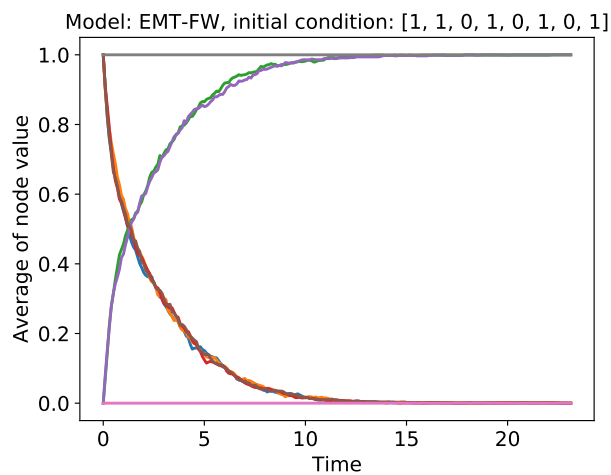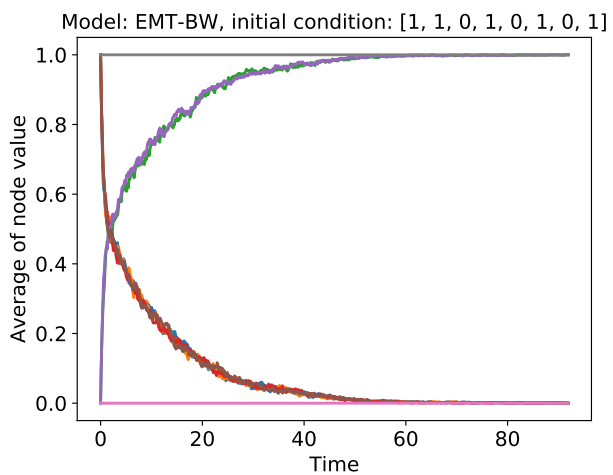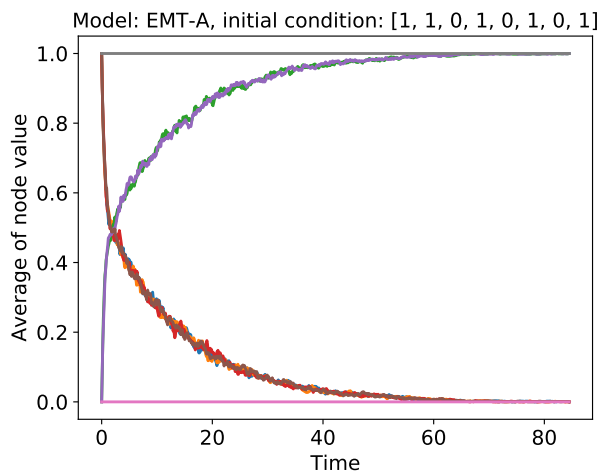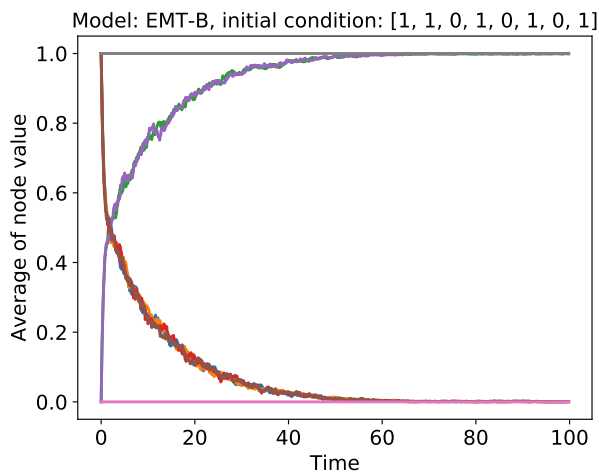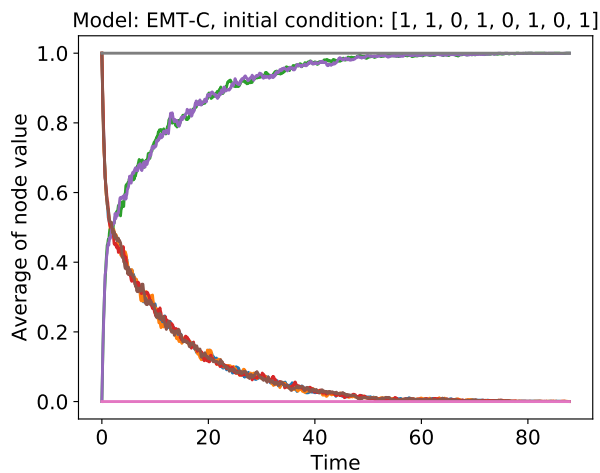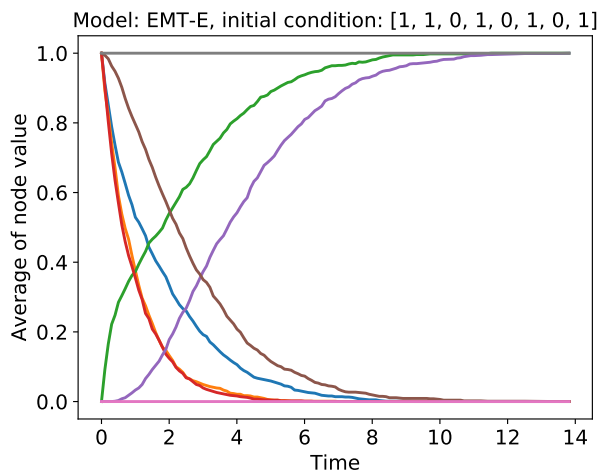

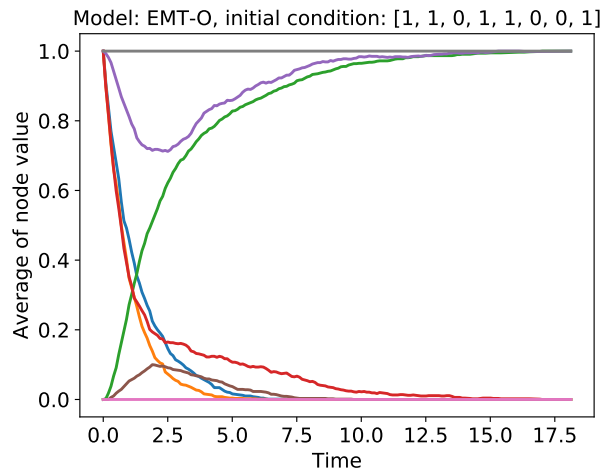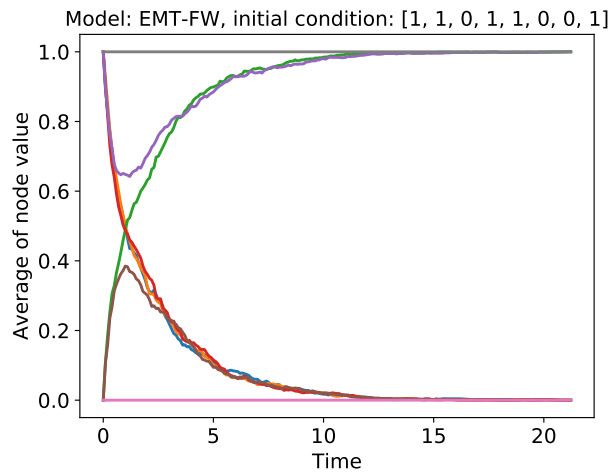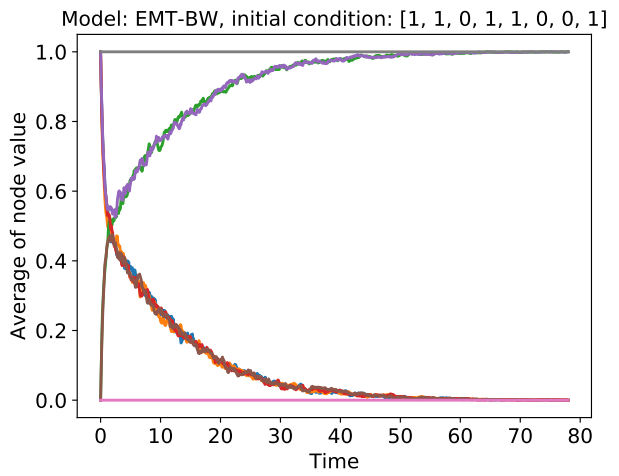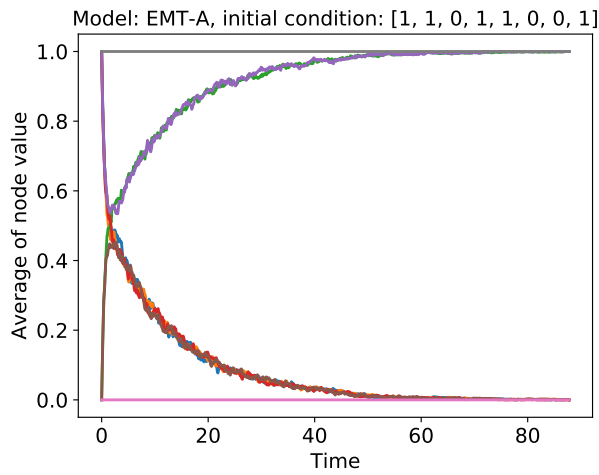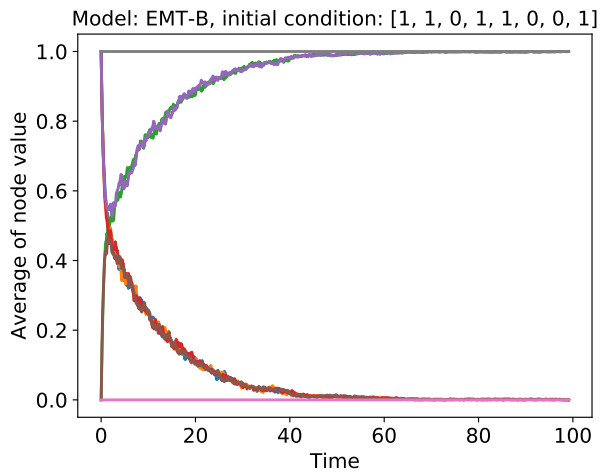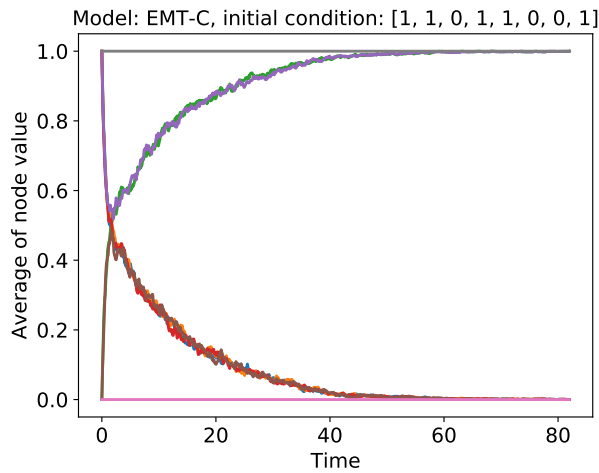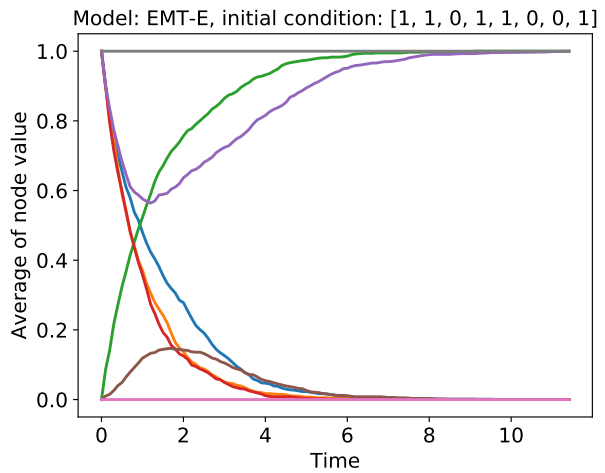

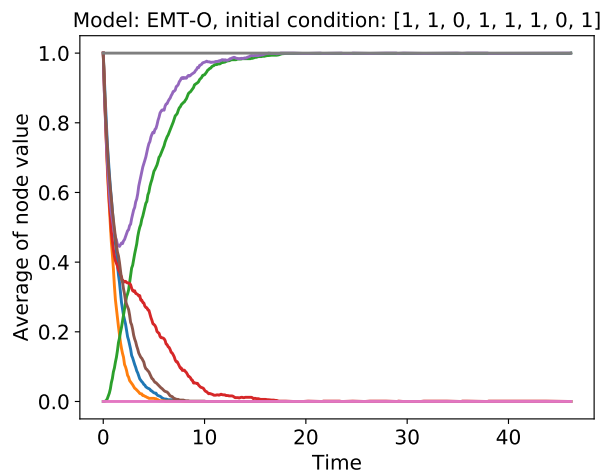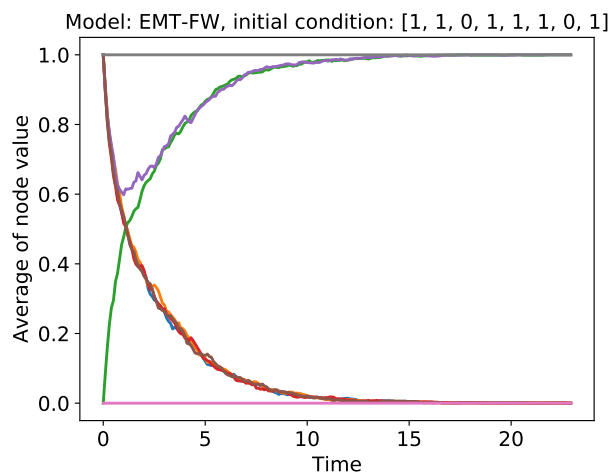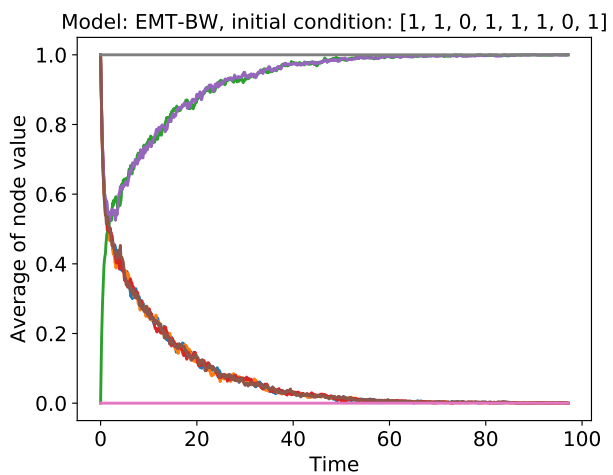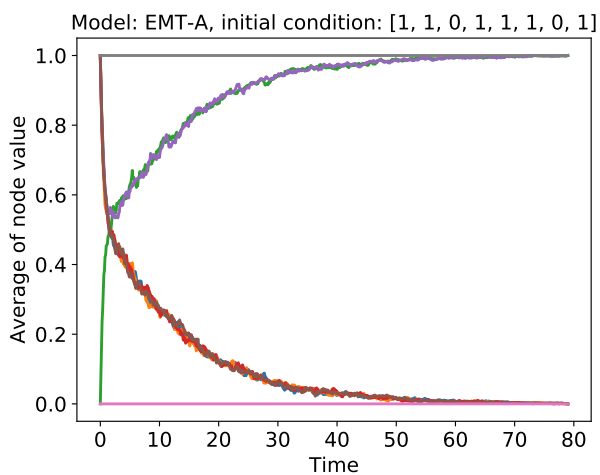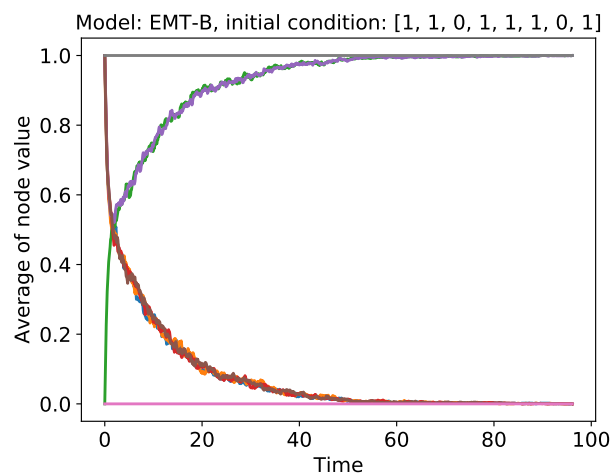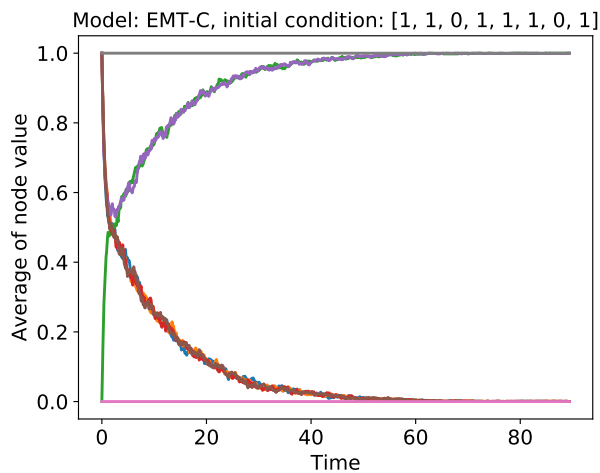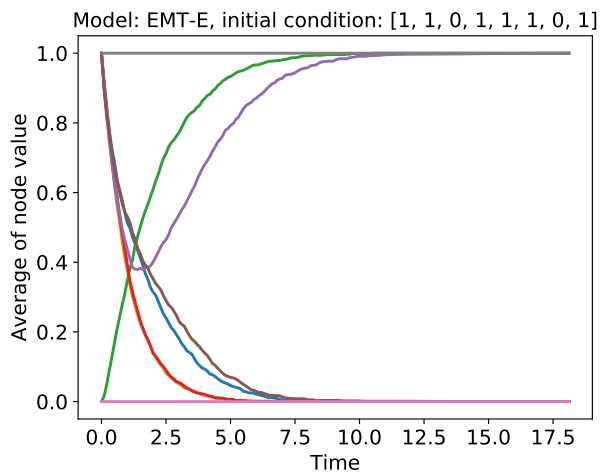

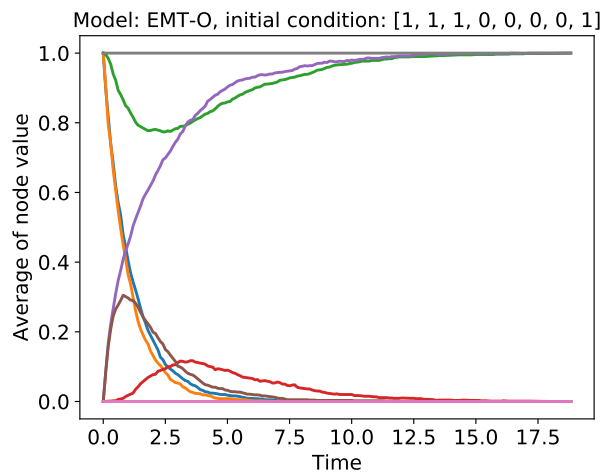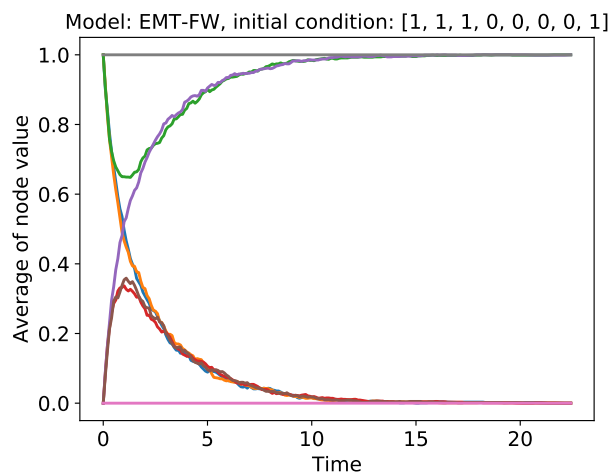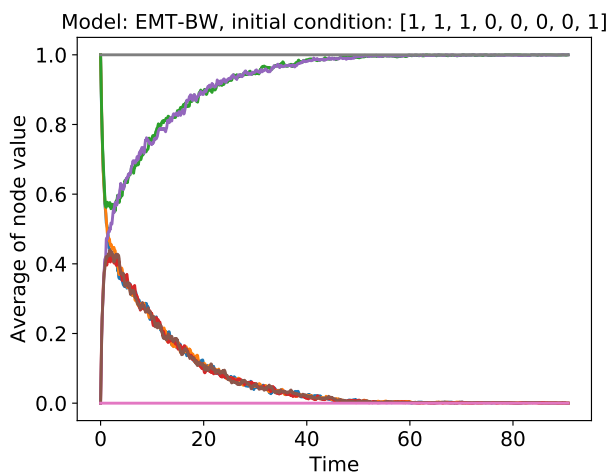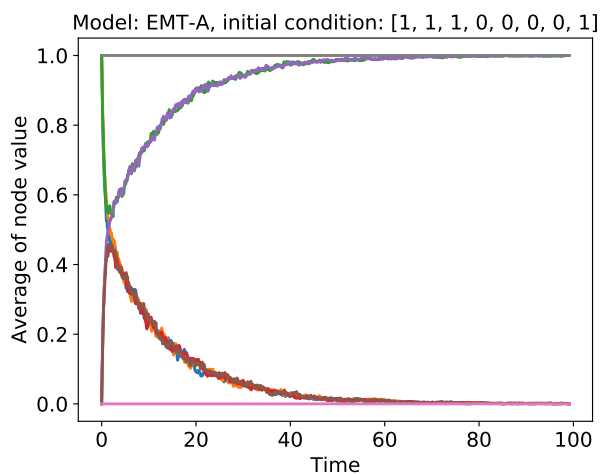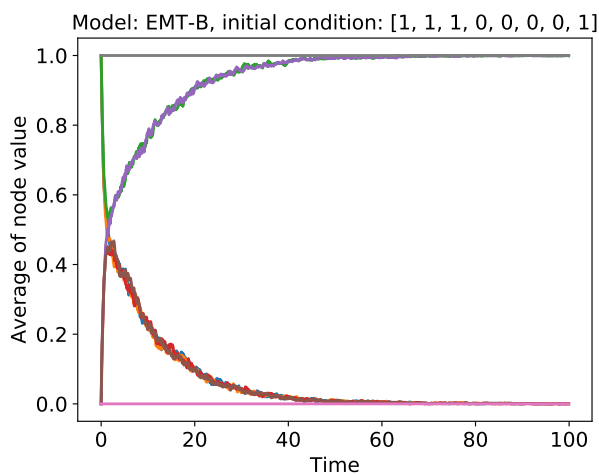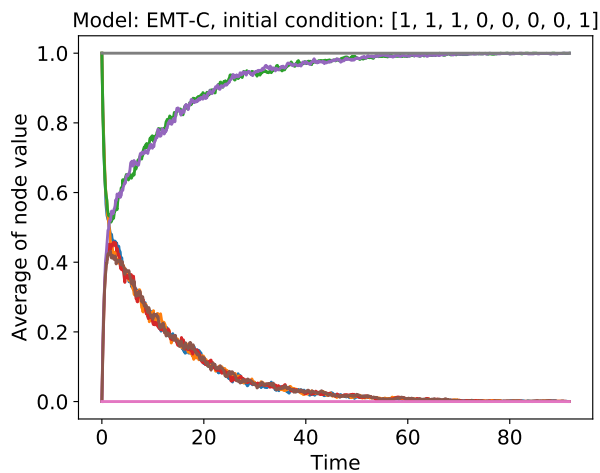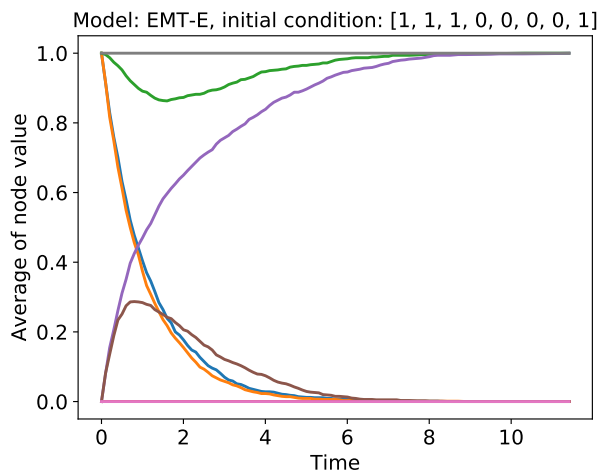

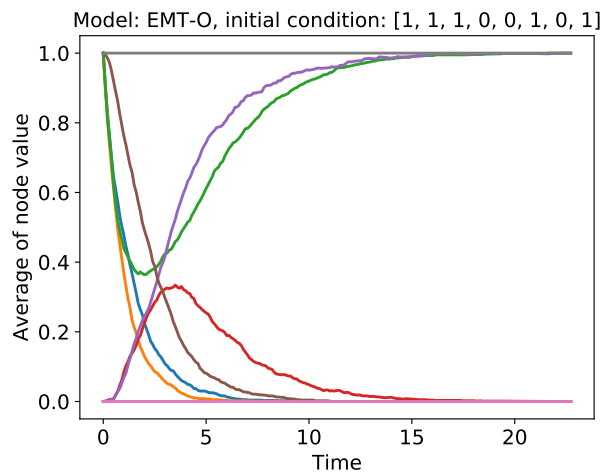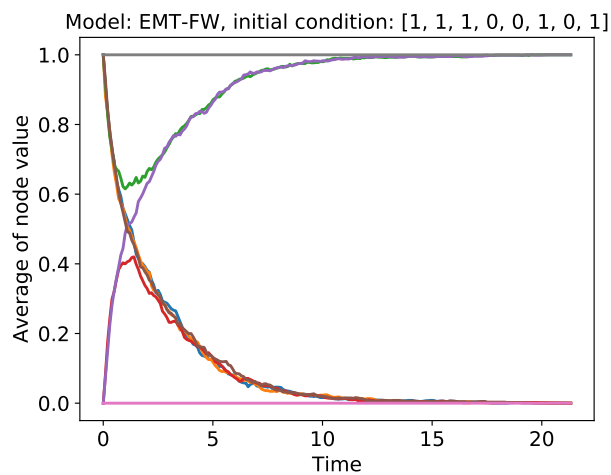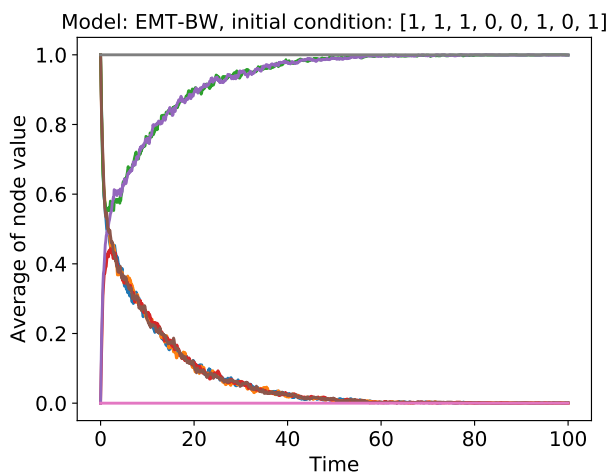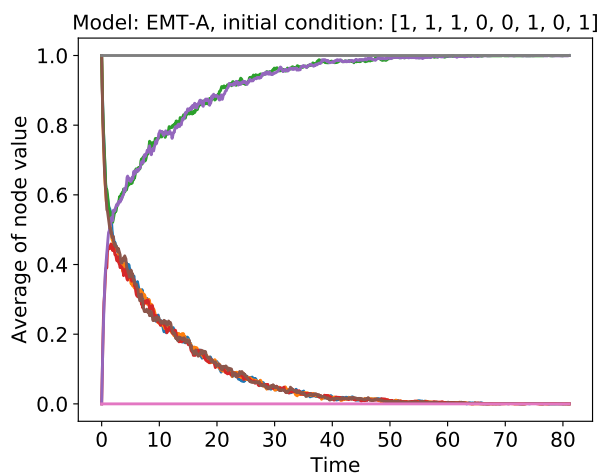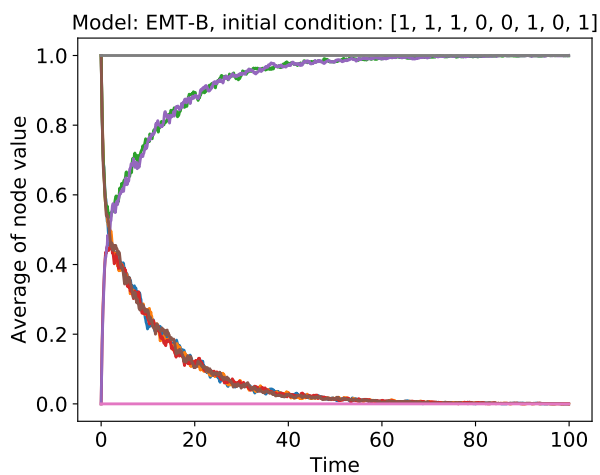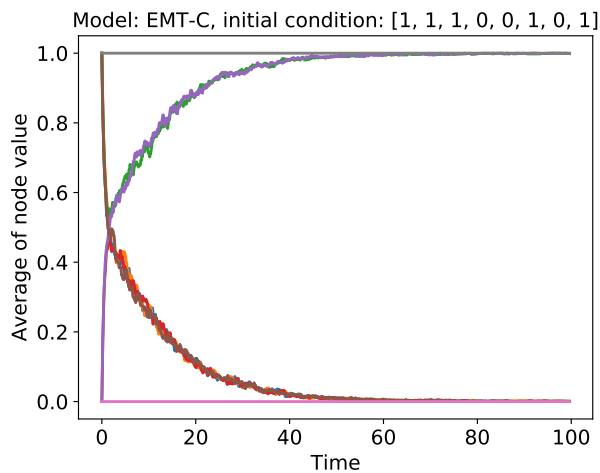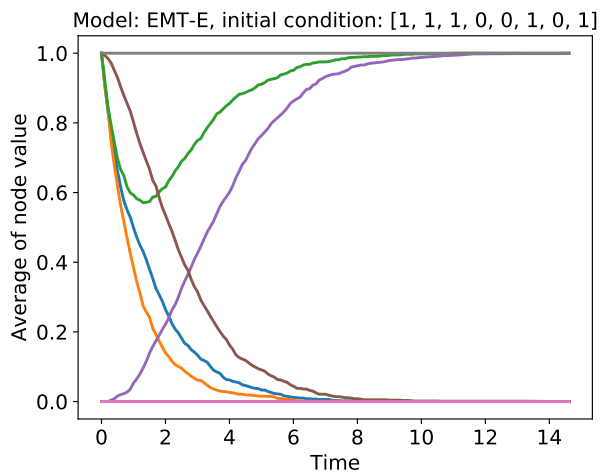

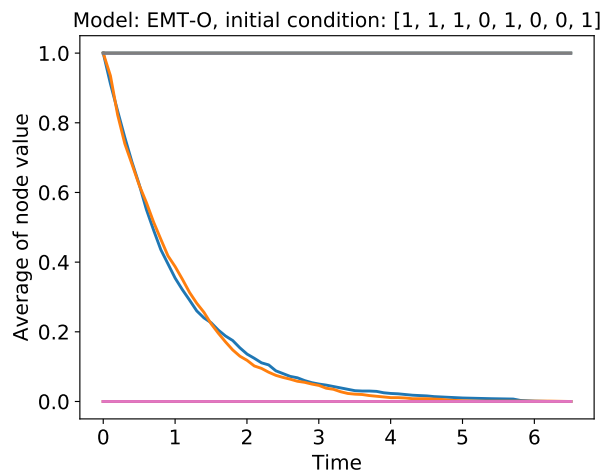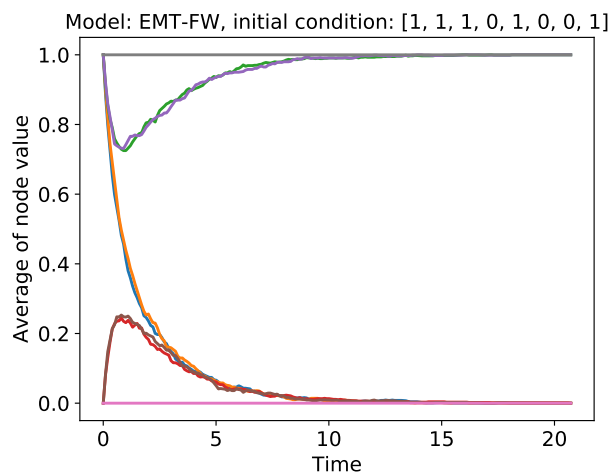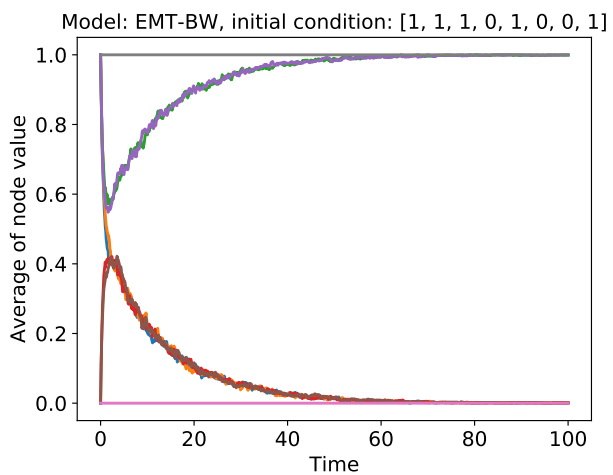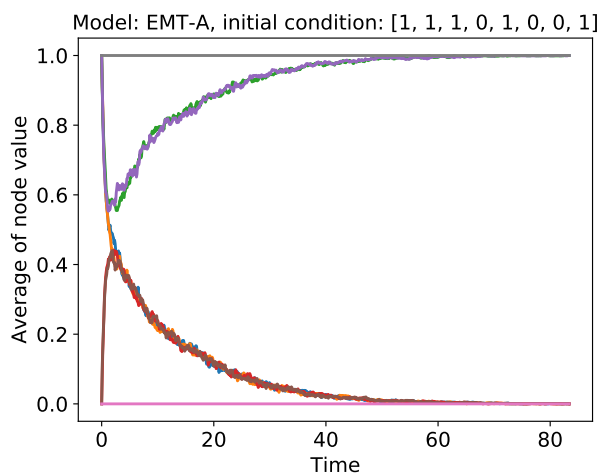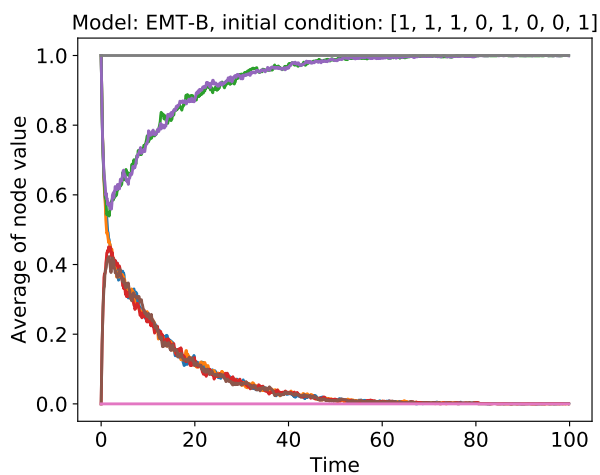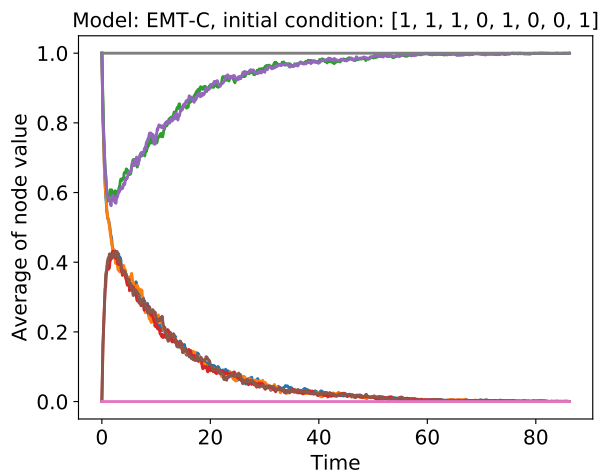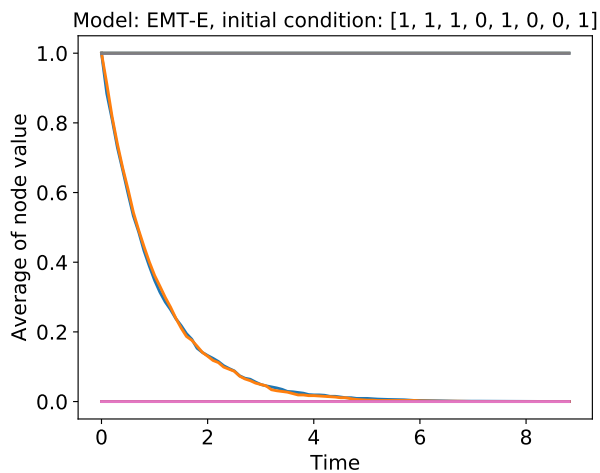

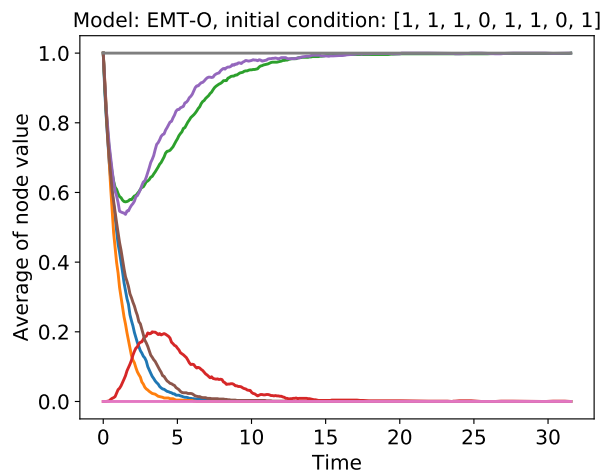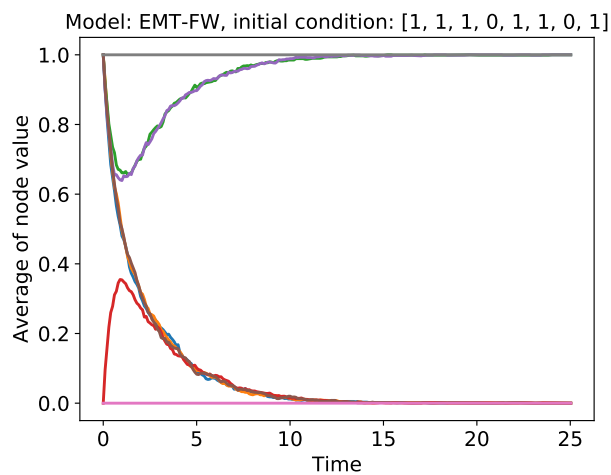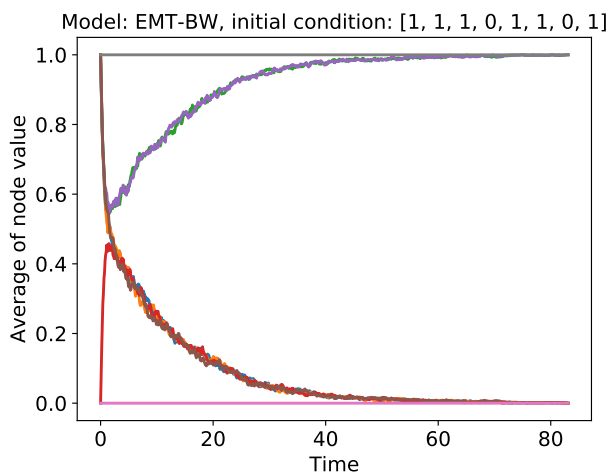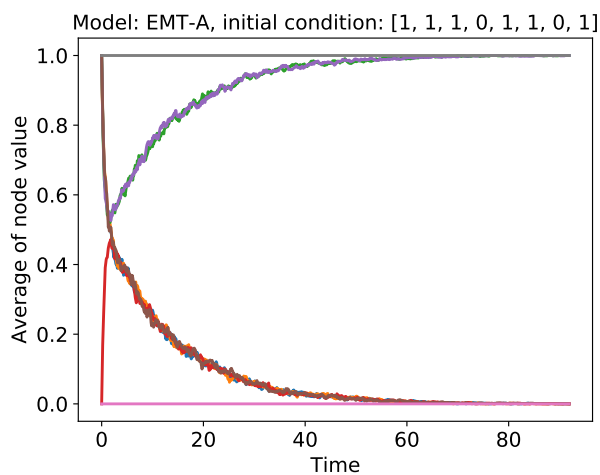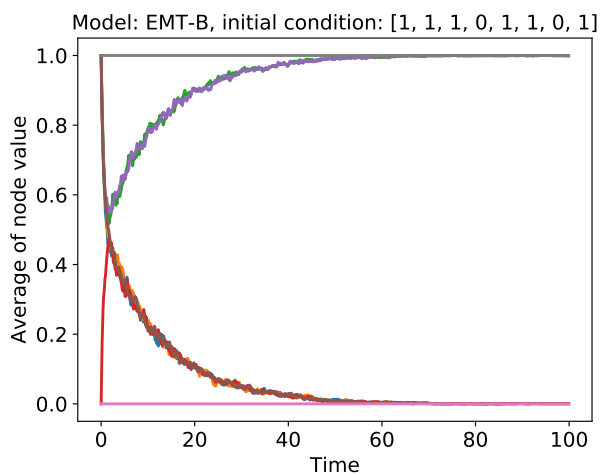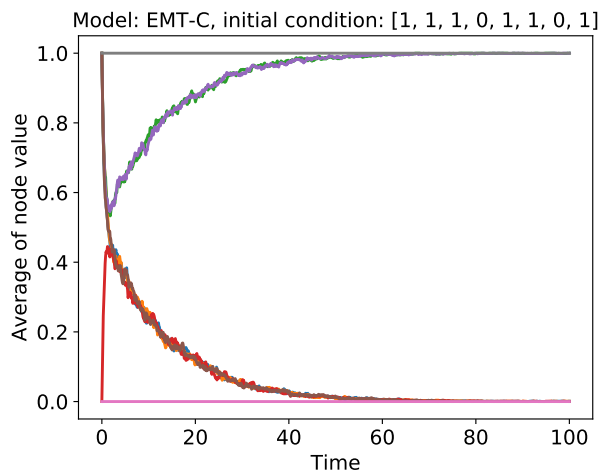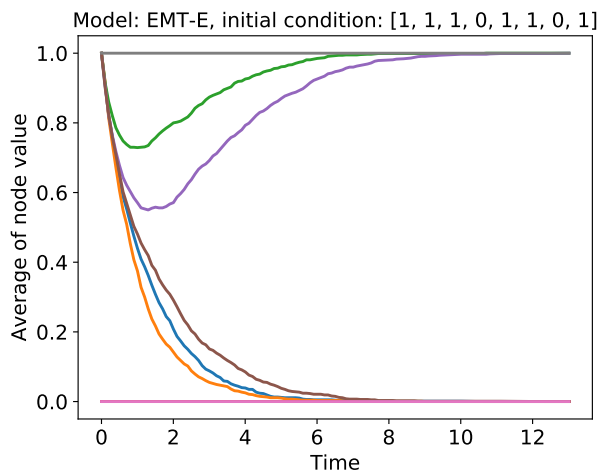

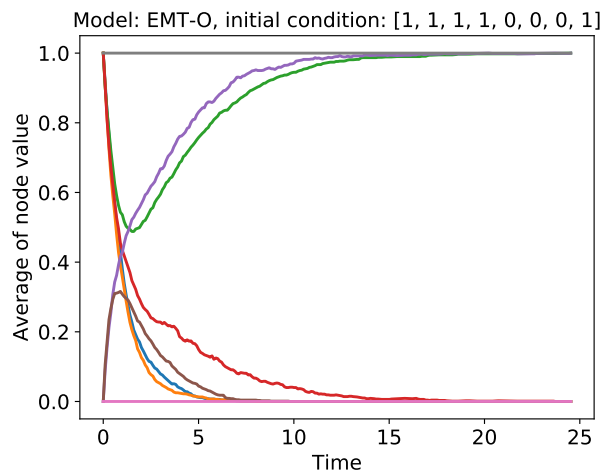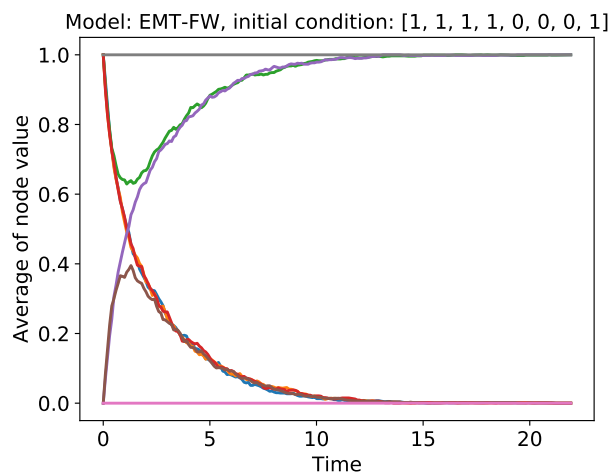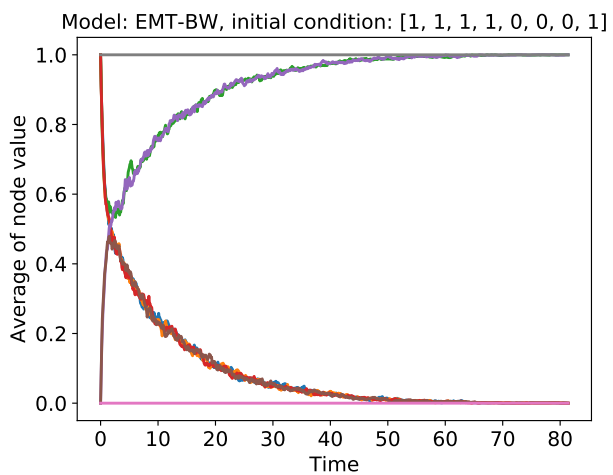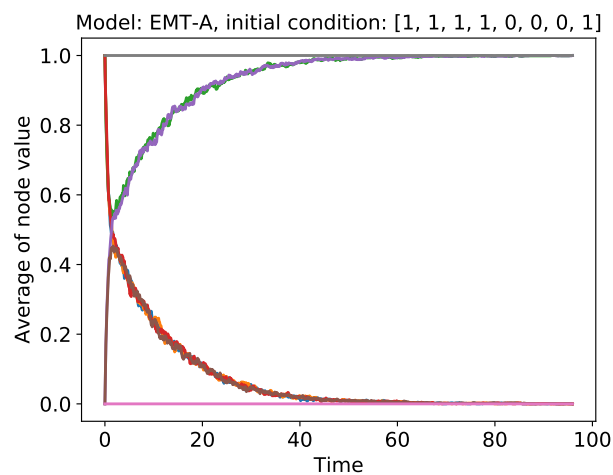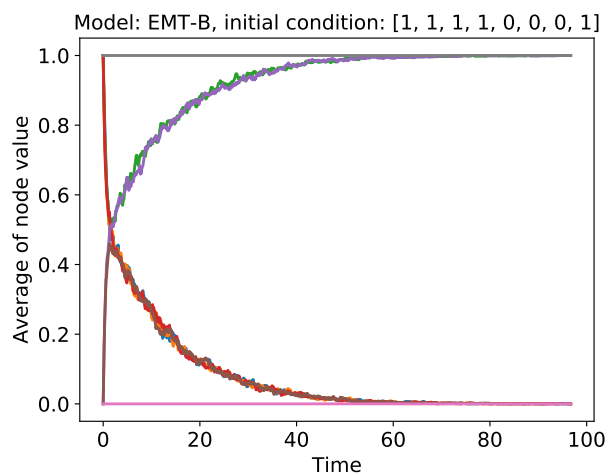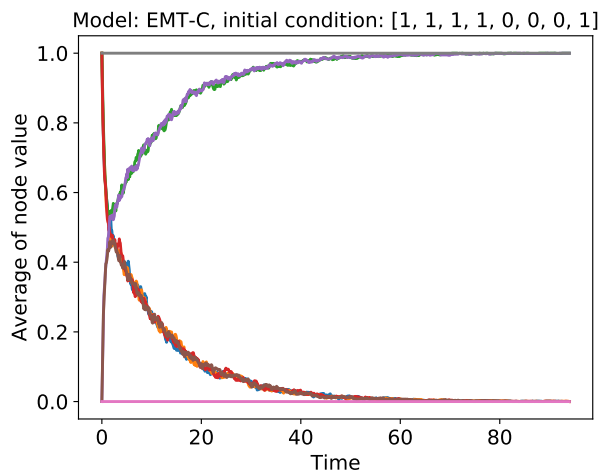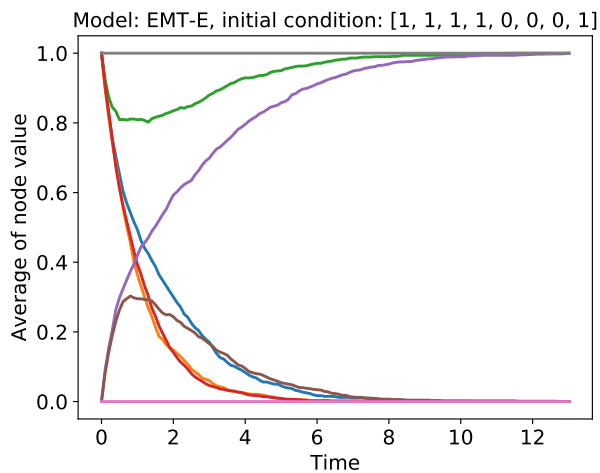

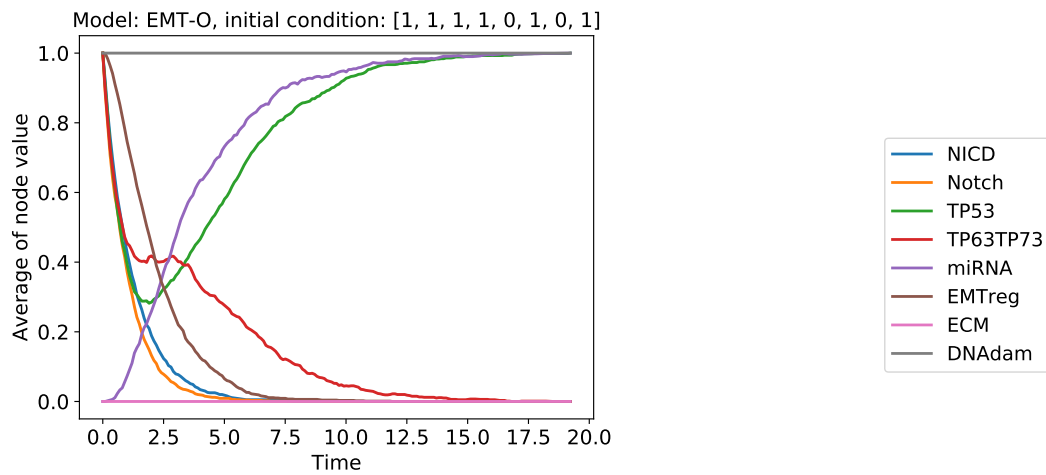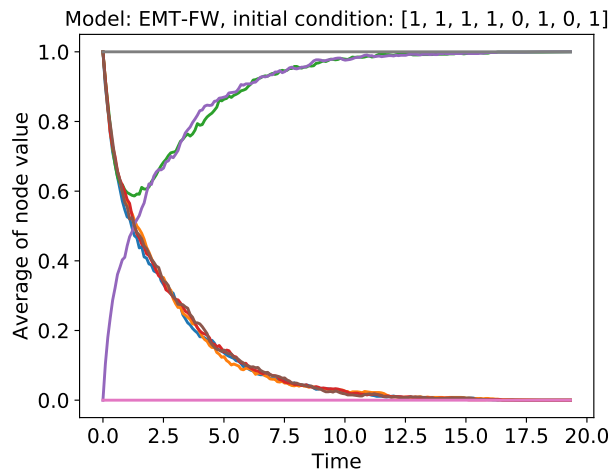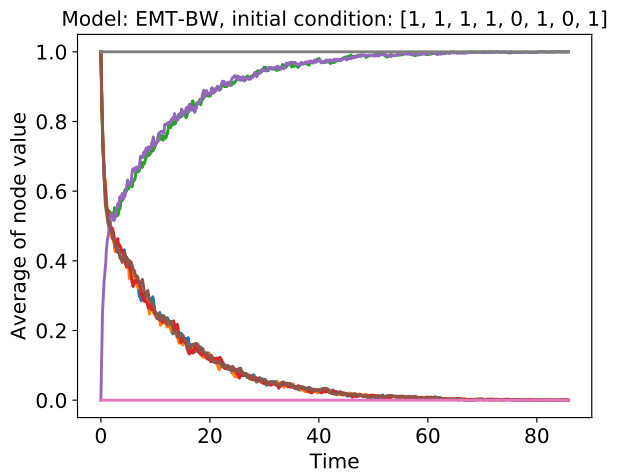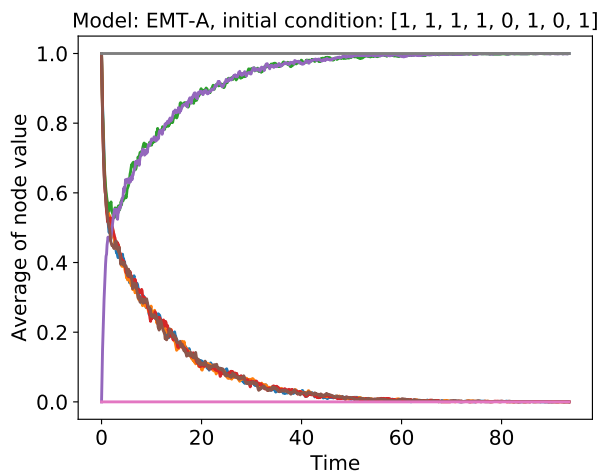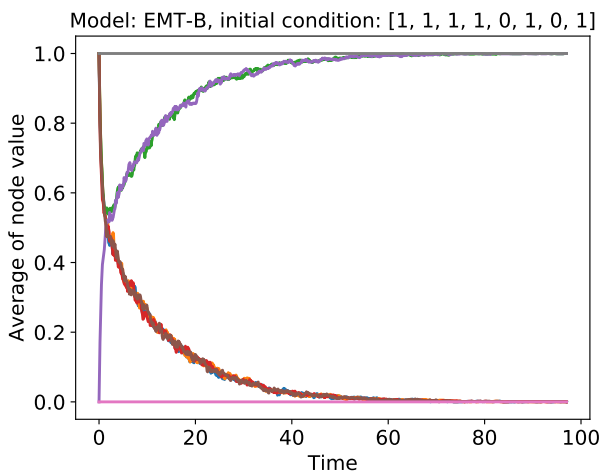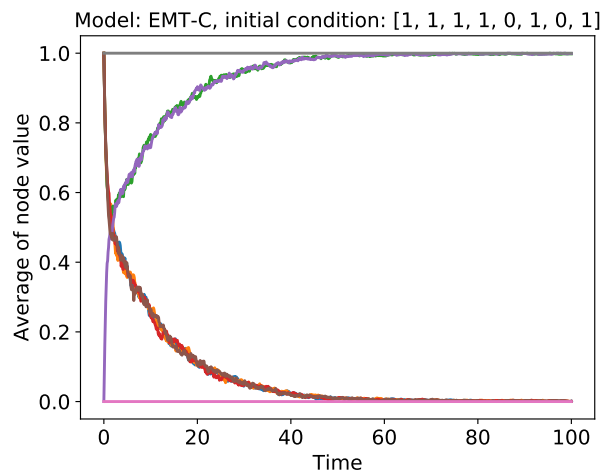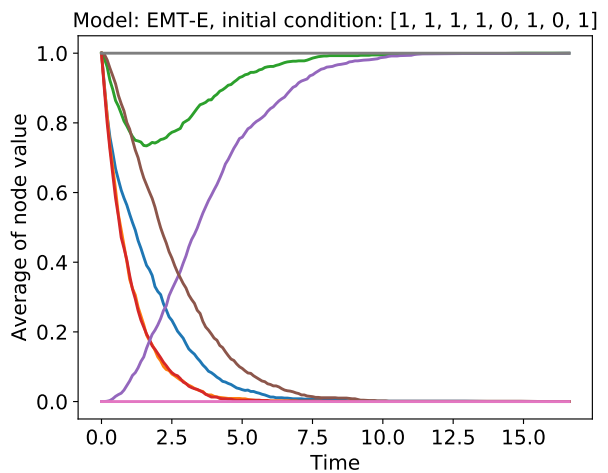

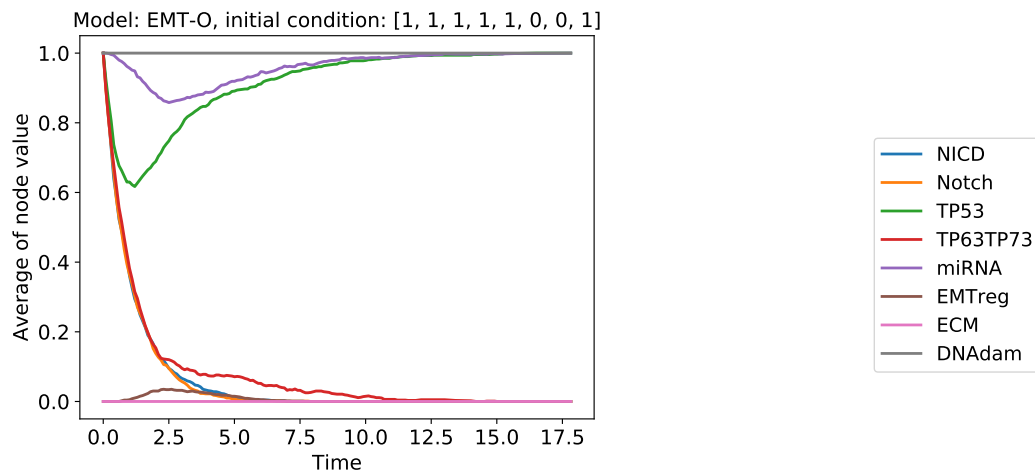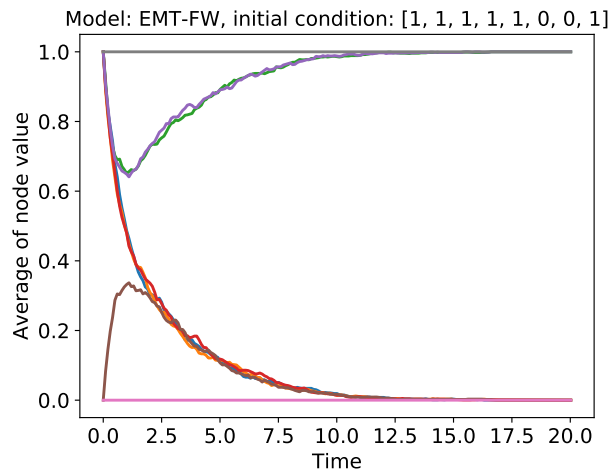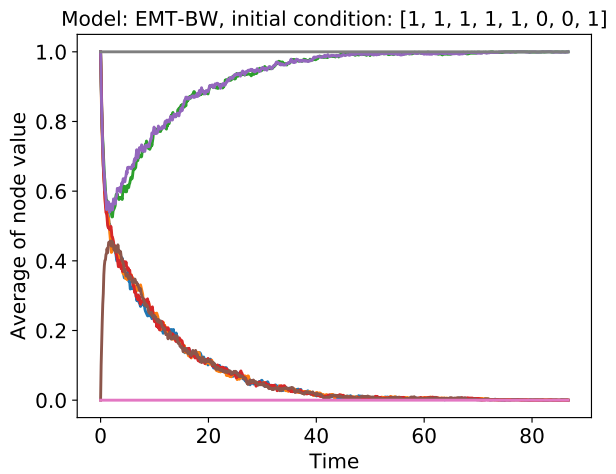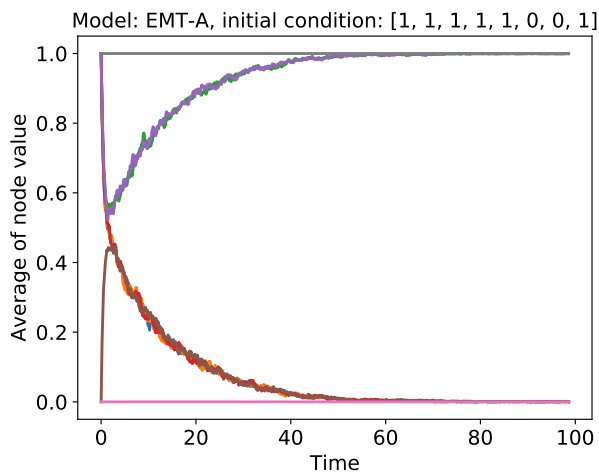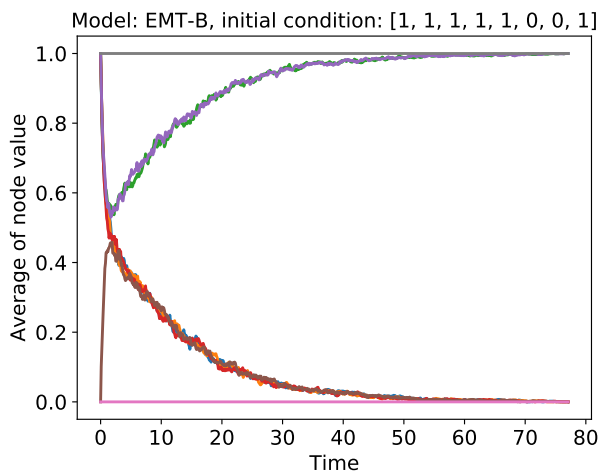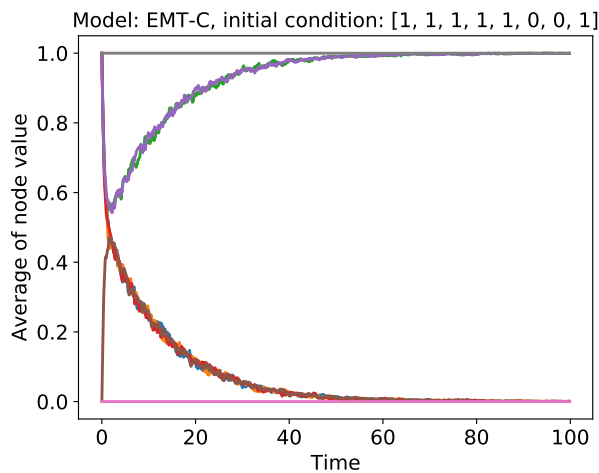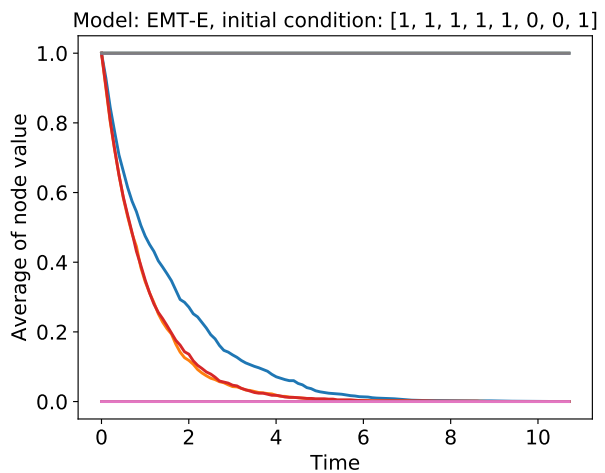

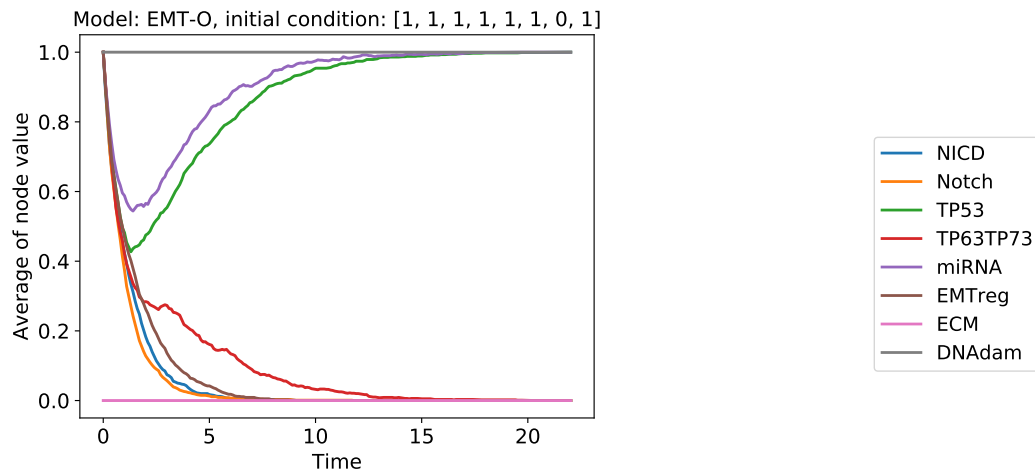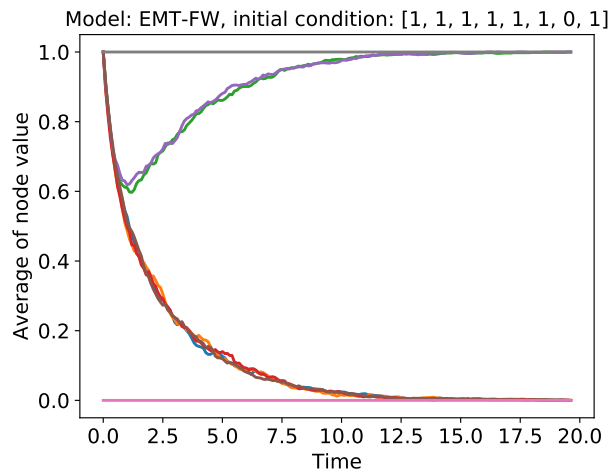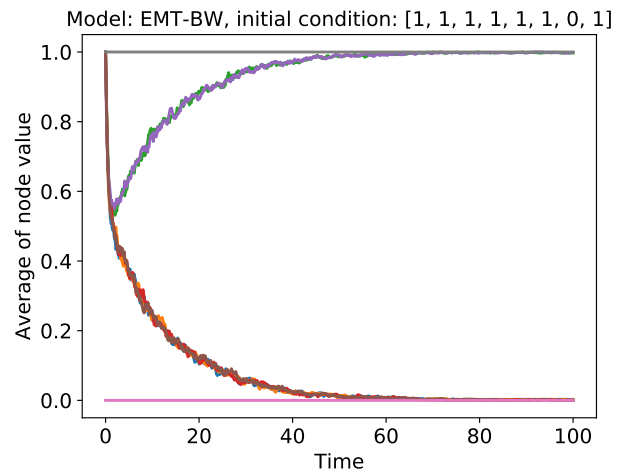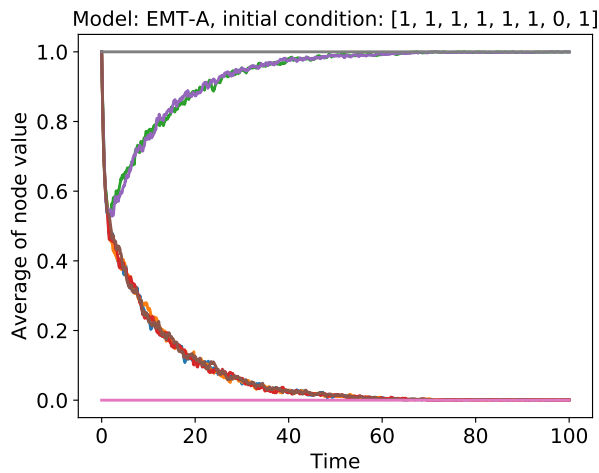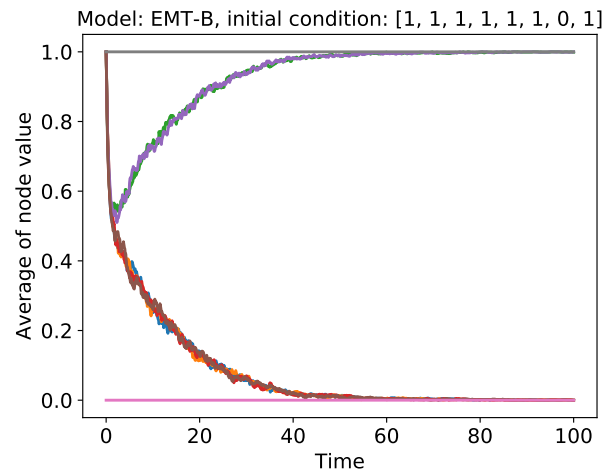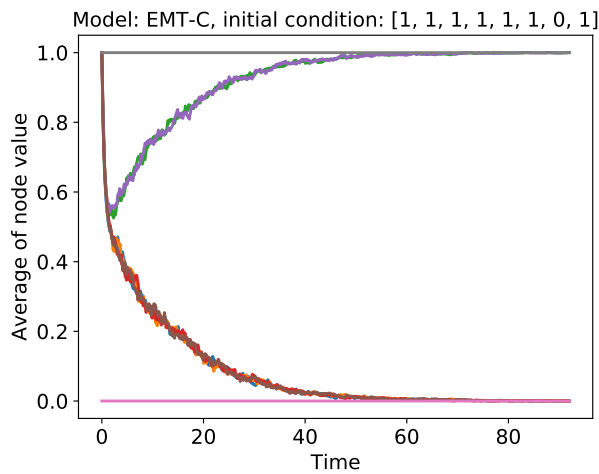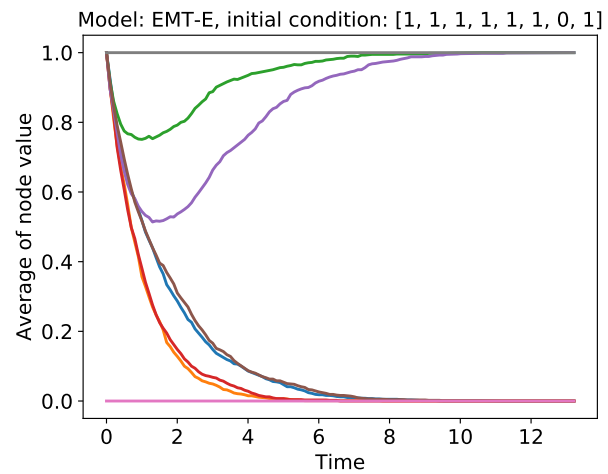

Supplement: S4 File — (PDF) [file pcbi.1009035.s004.pdf]

# Parameter set $(\text{ECM}, \text{DNAdam}) = (1, 0)$ :

Steady states:  $(0, 0, 1, 0, 1, 0, 1, 0)$ ,  $(1, 1, 0, 0, 0, 1, 1, 0)$

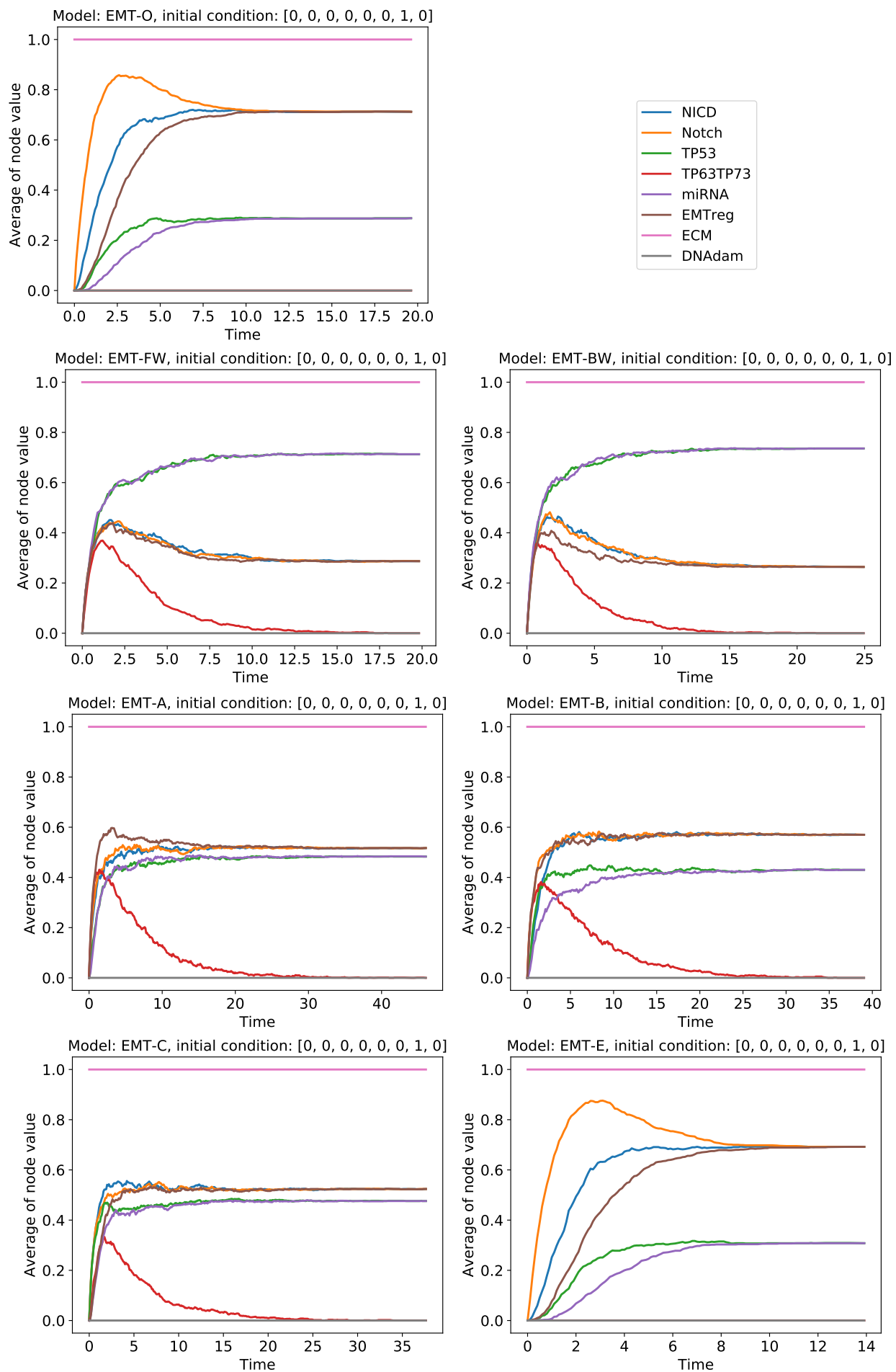

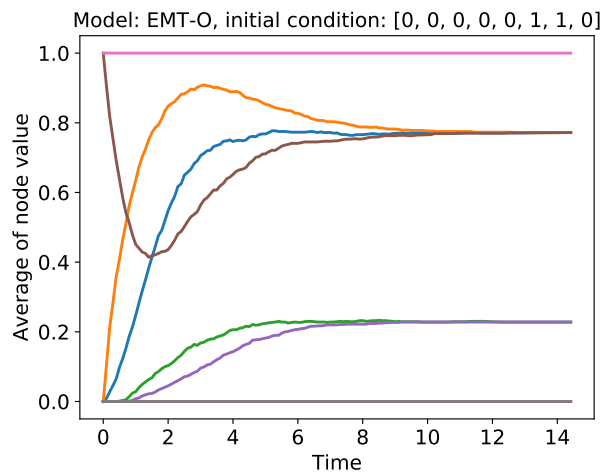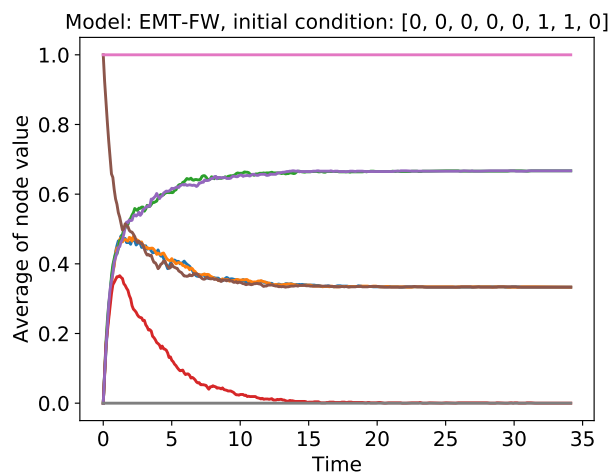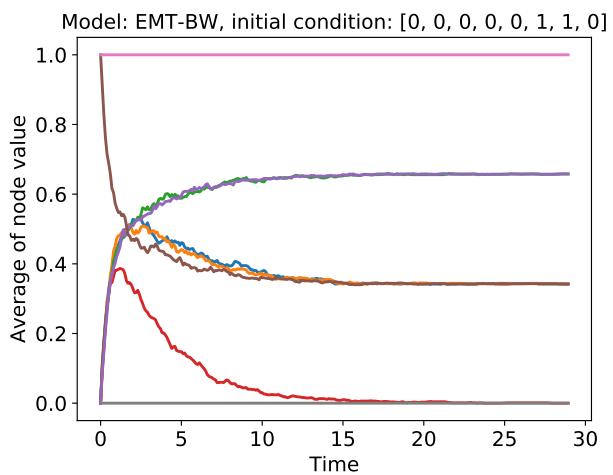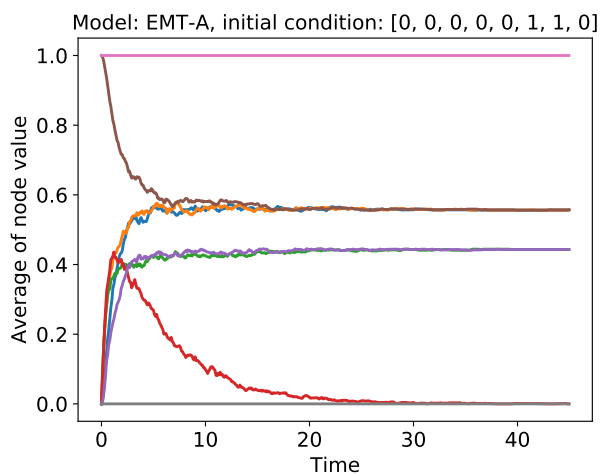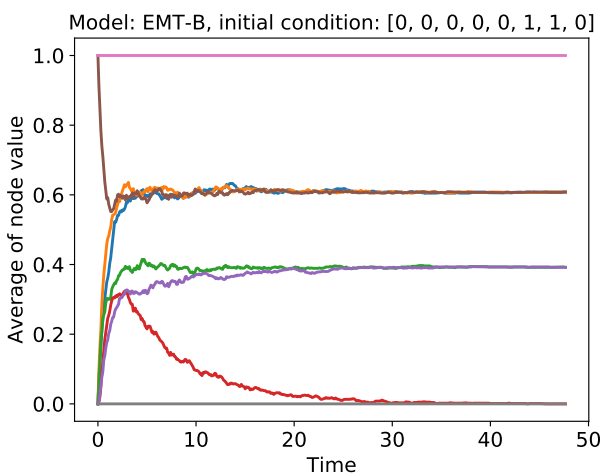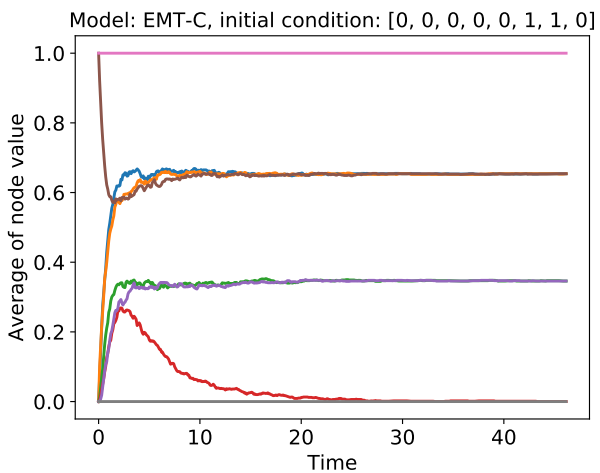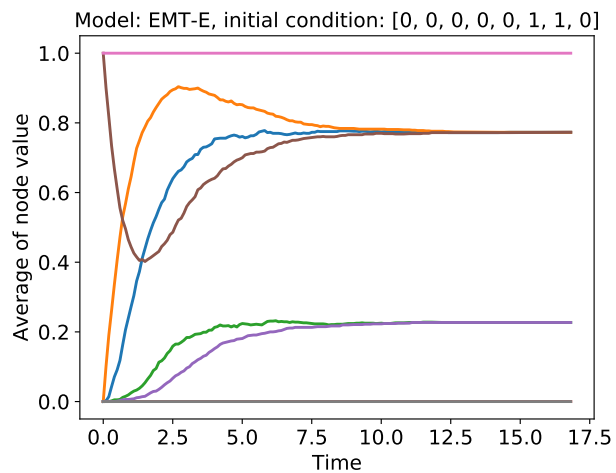

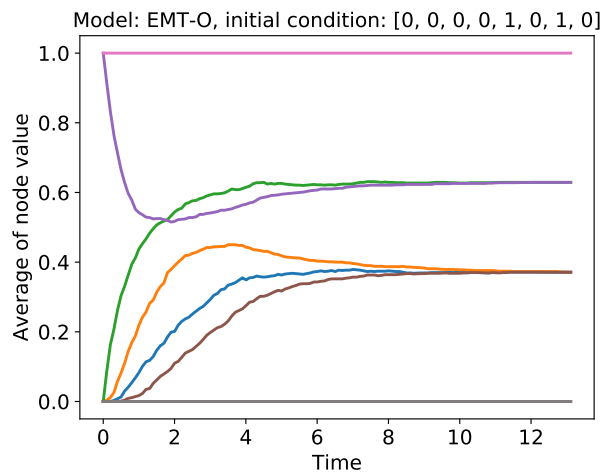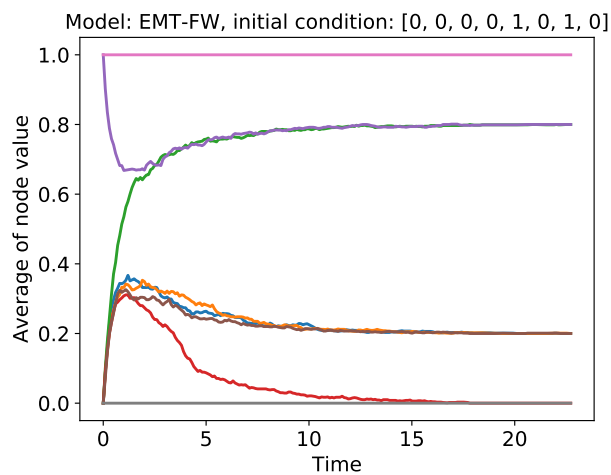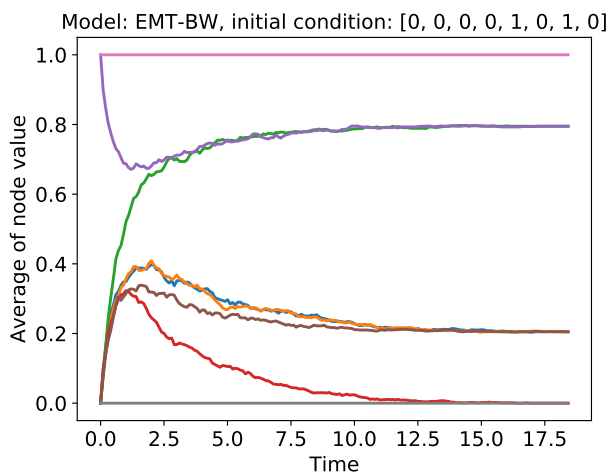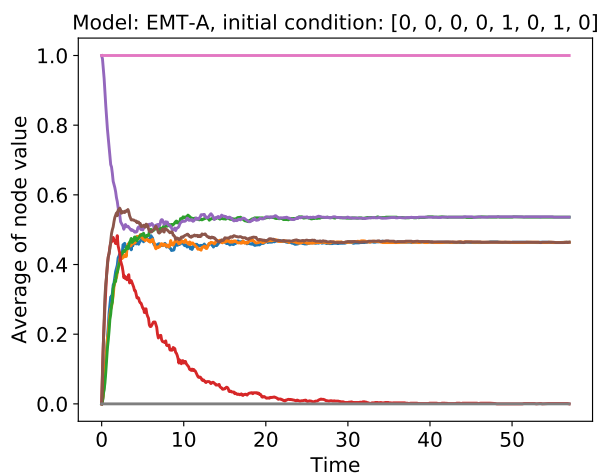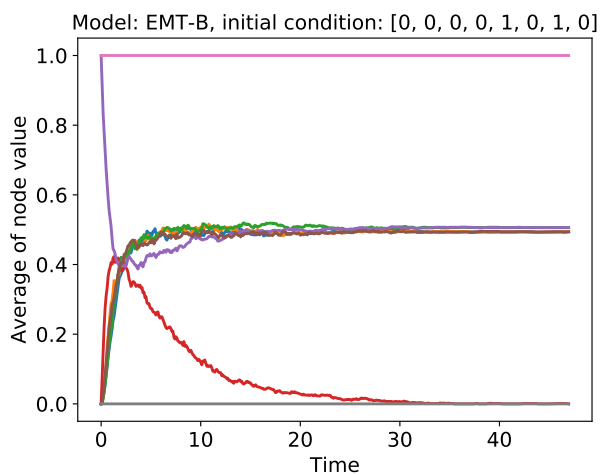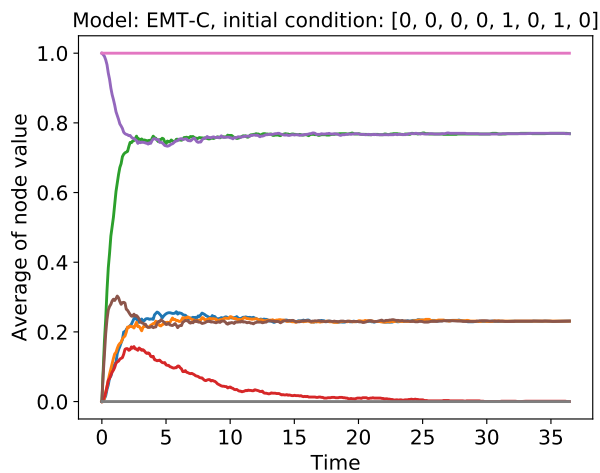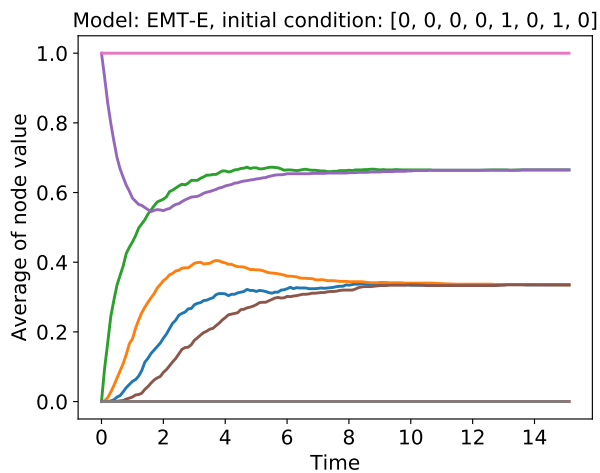

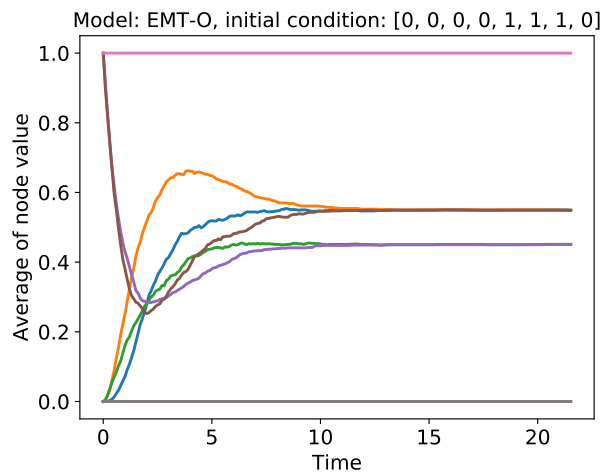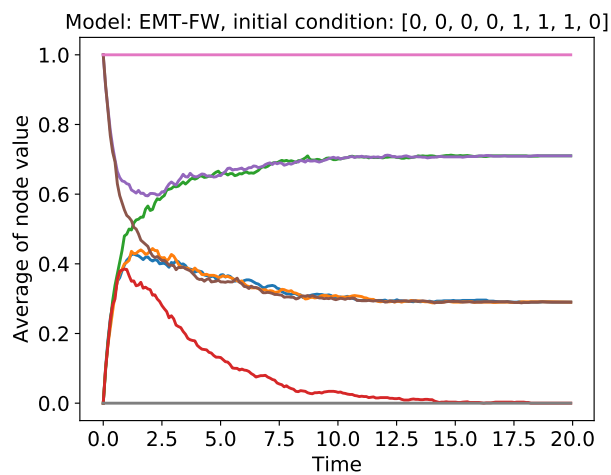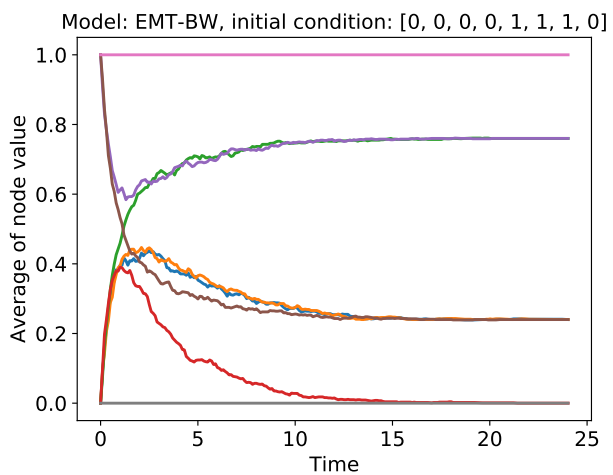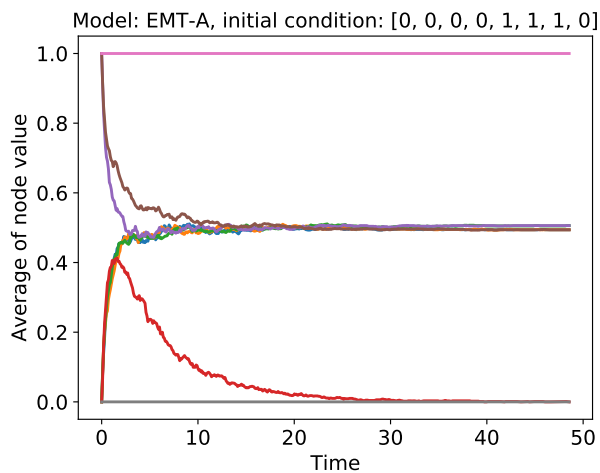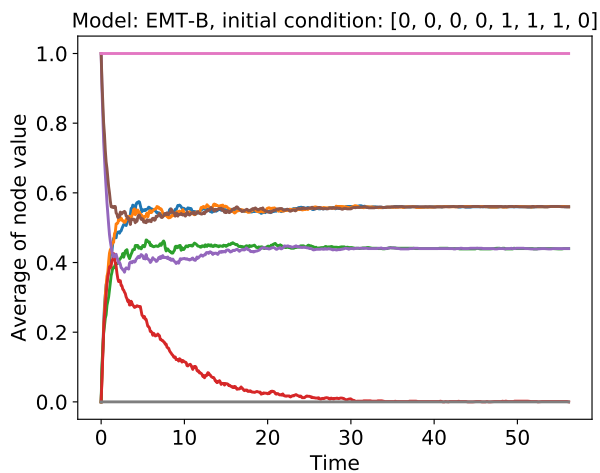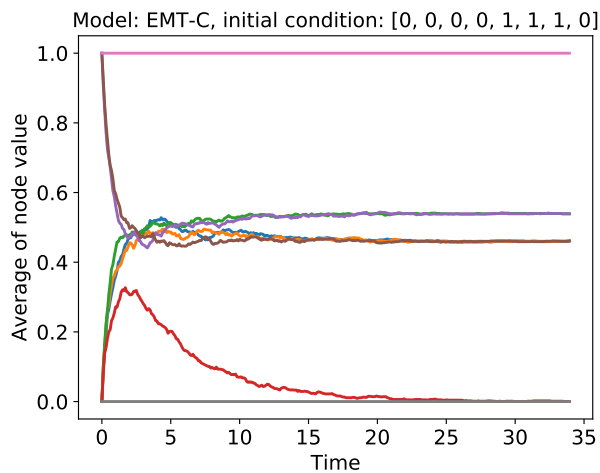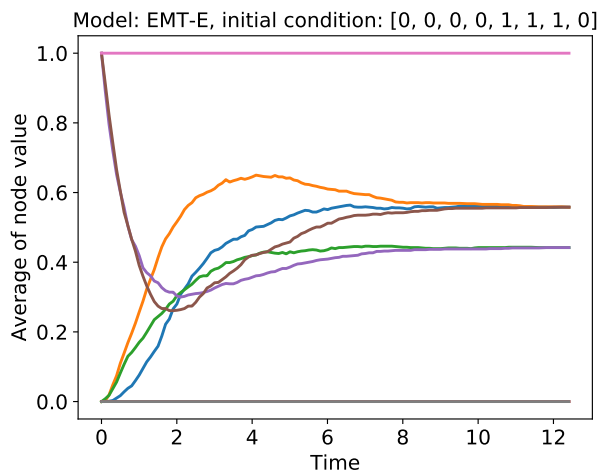

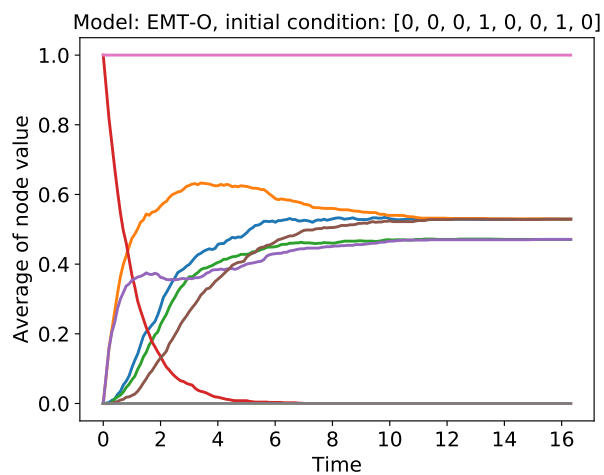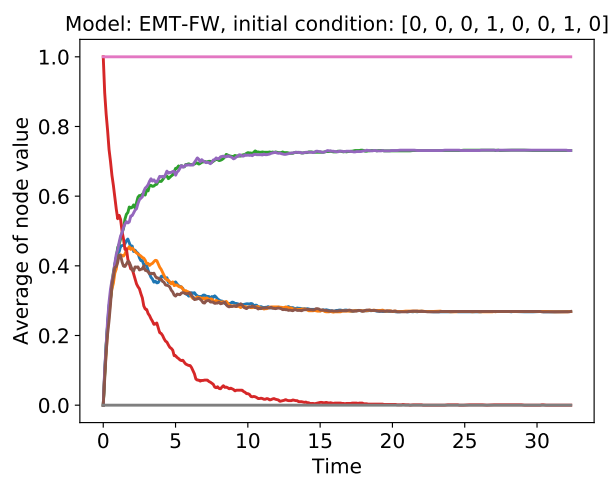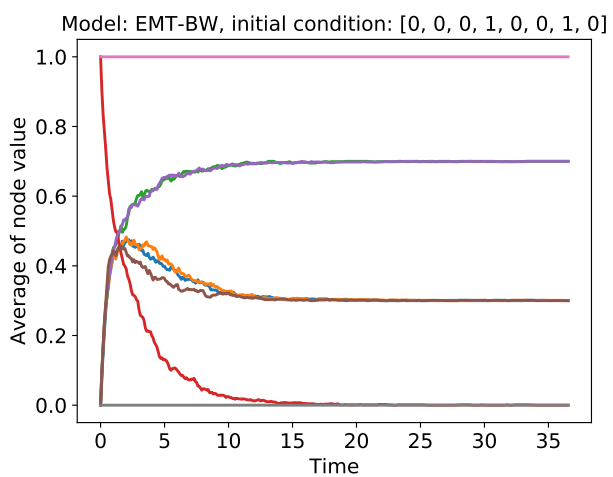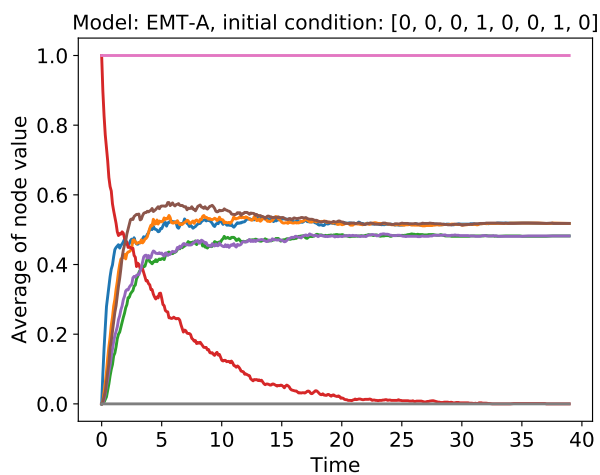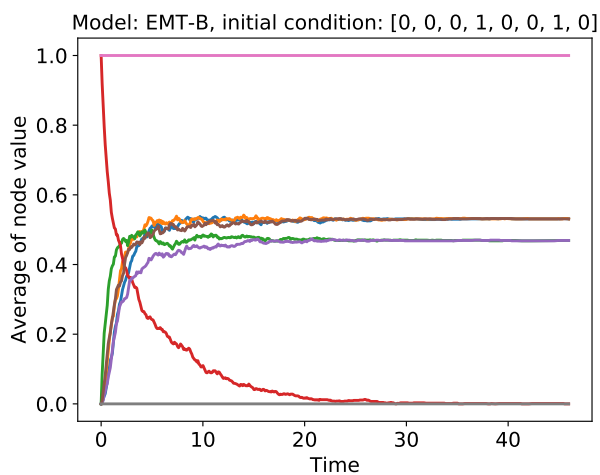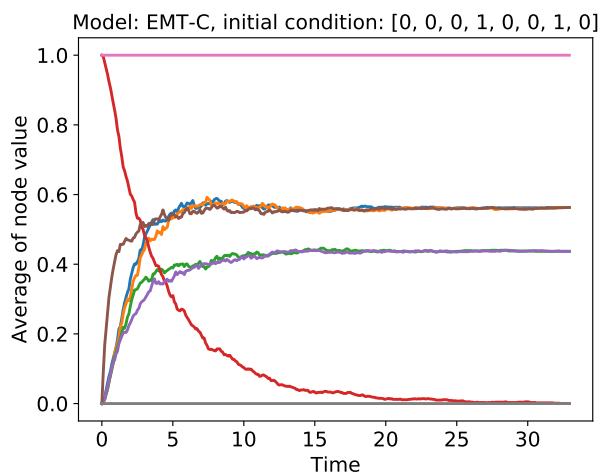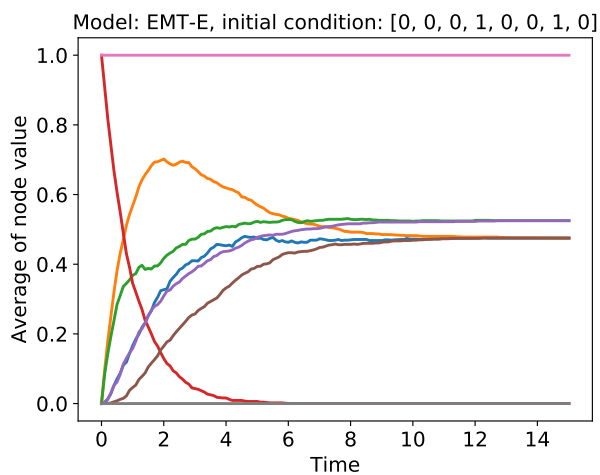

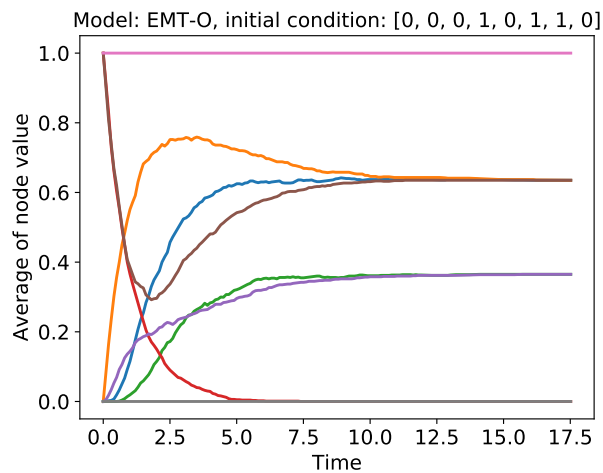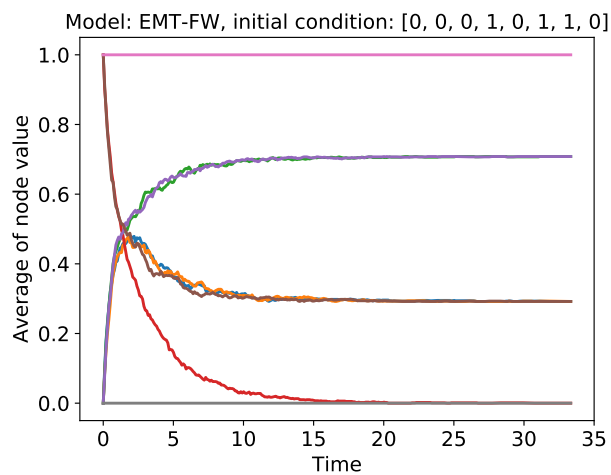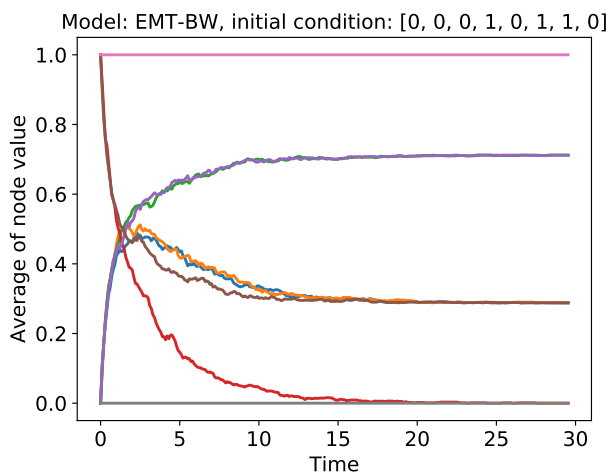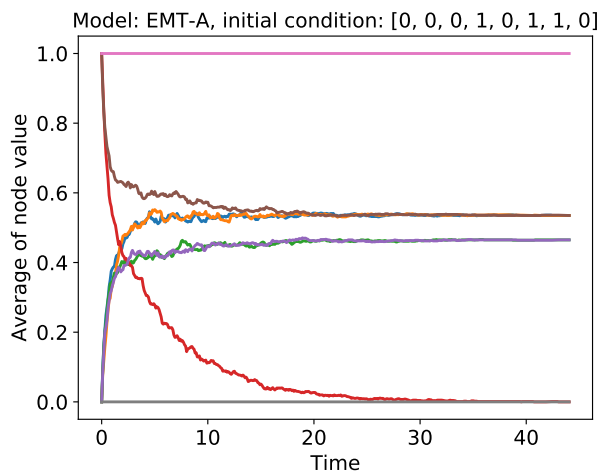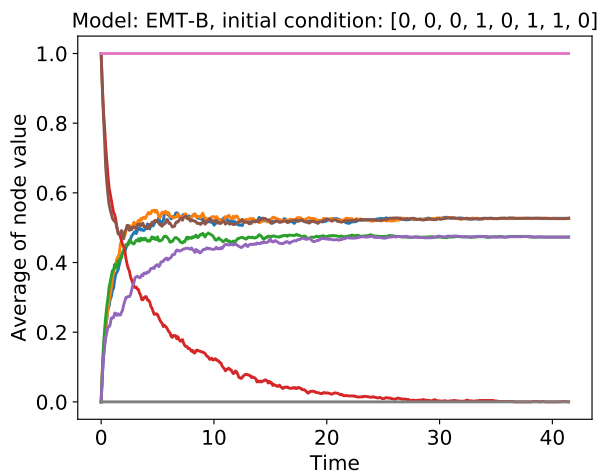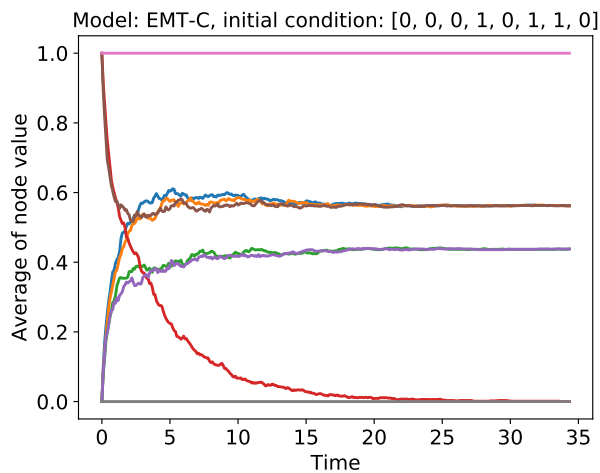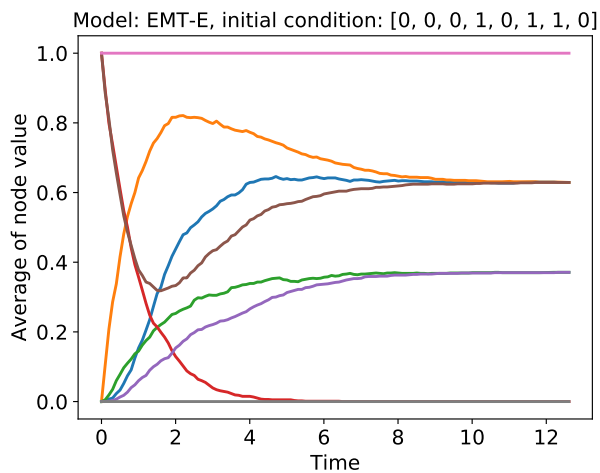

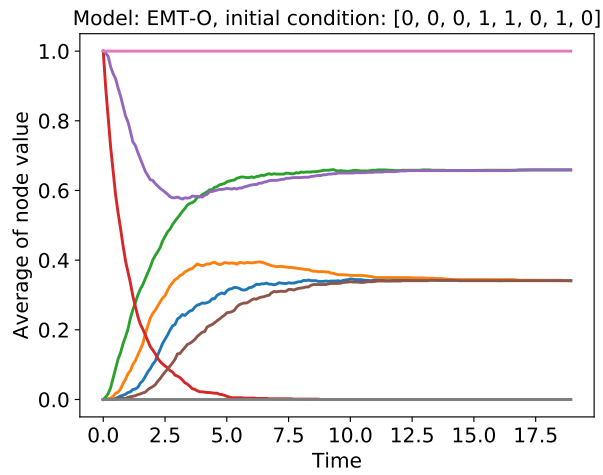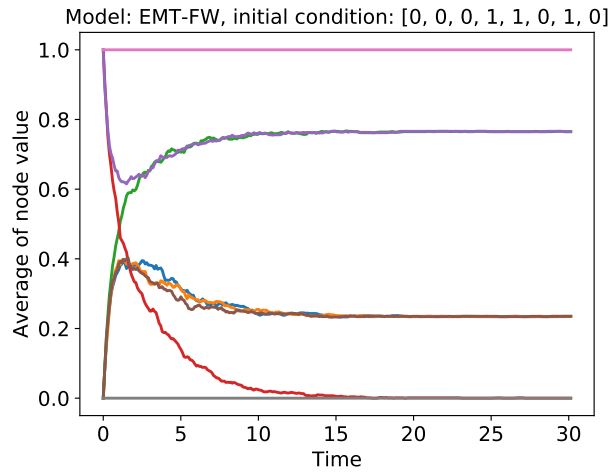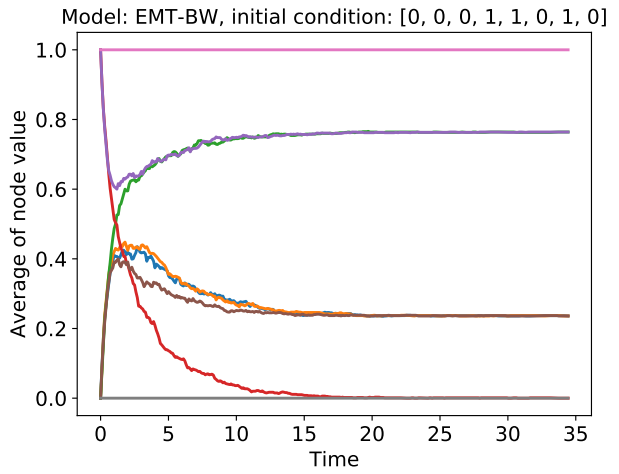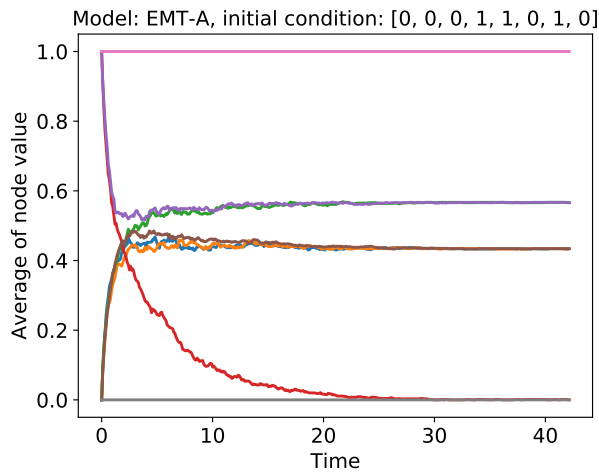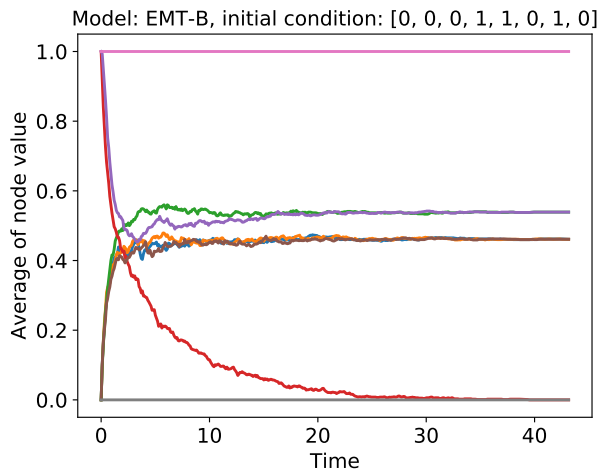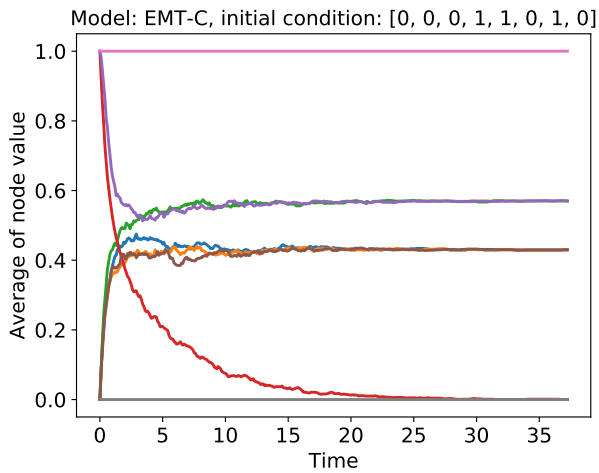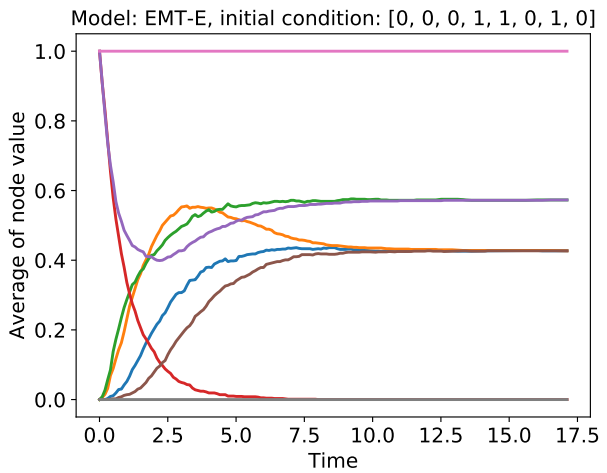

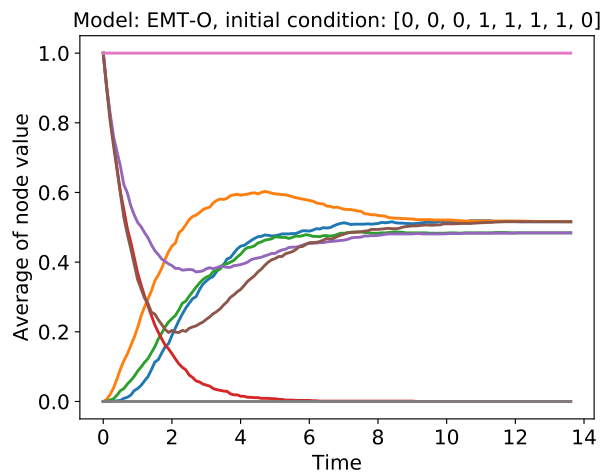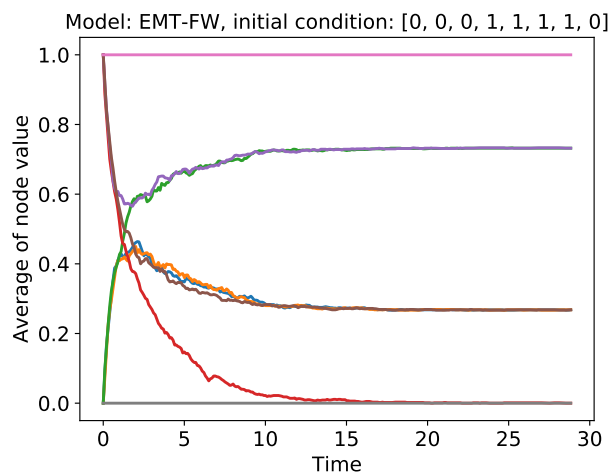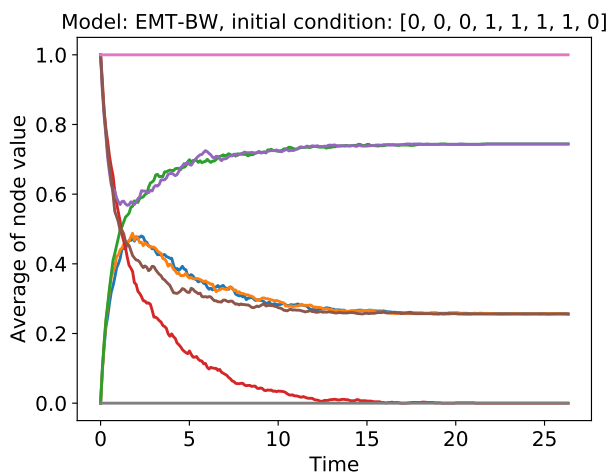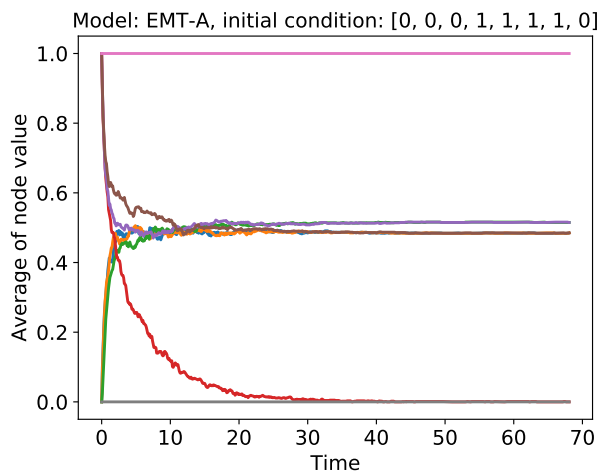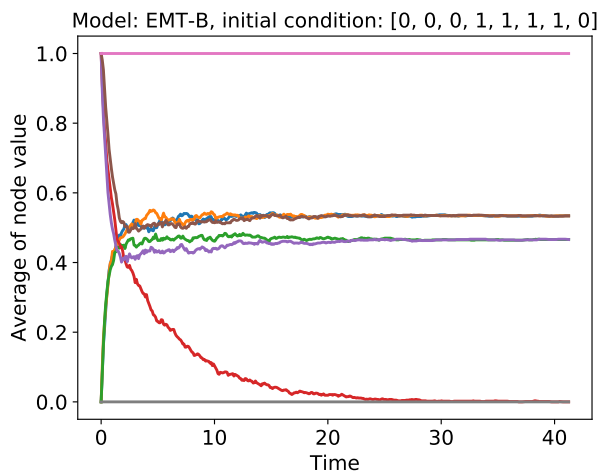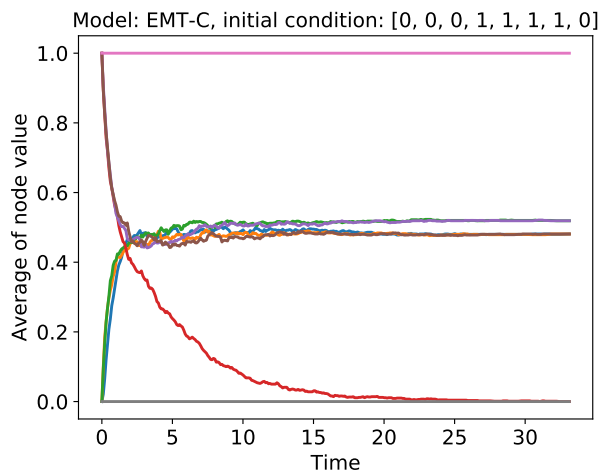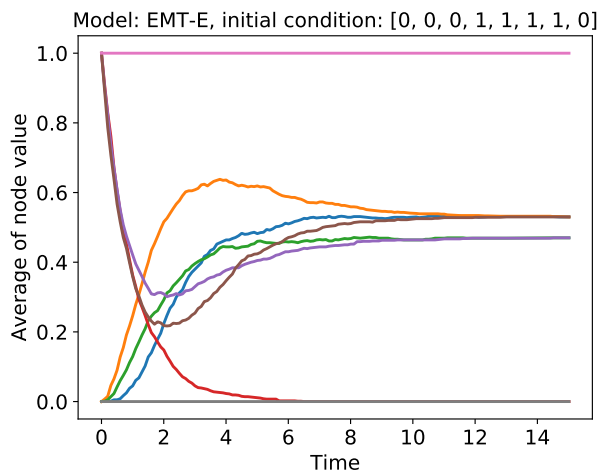

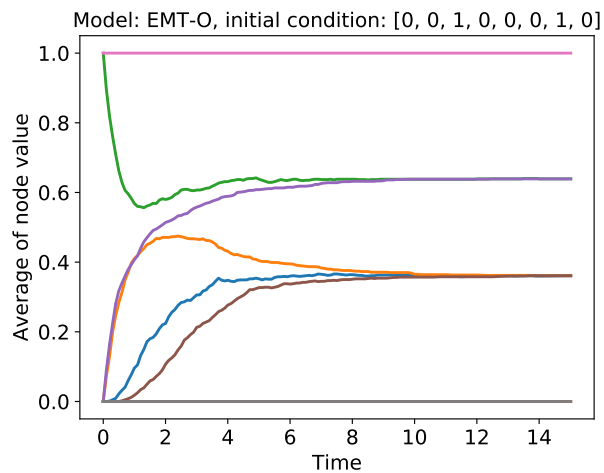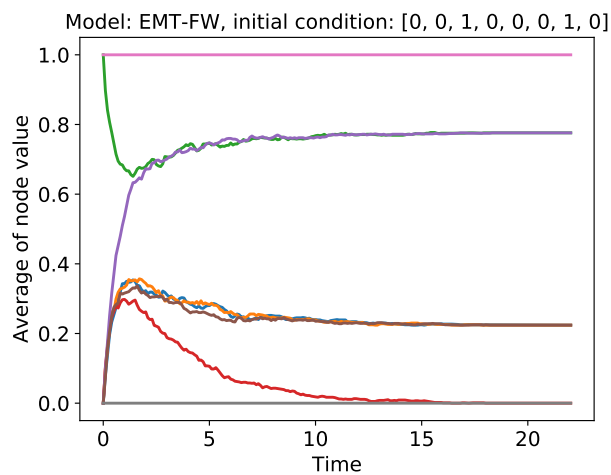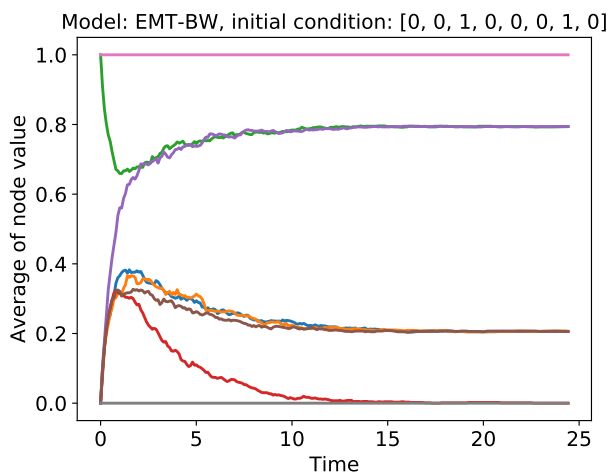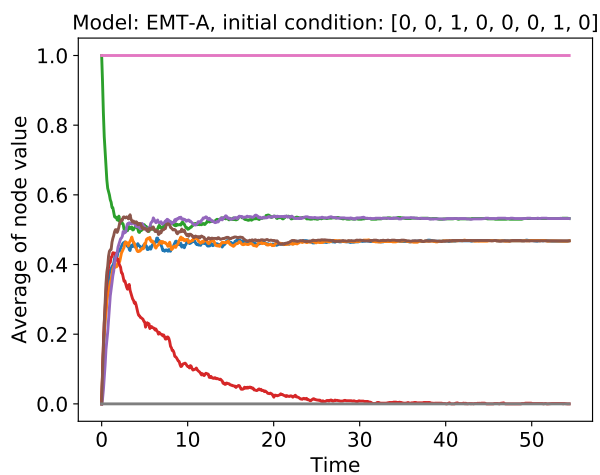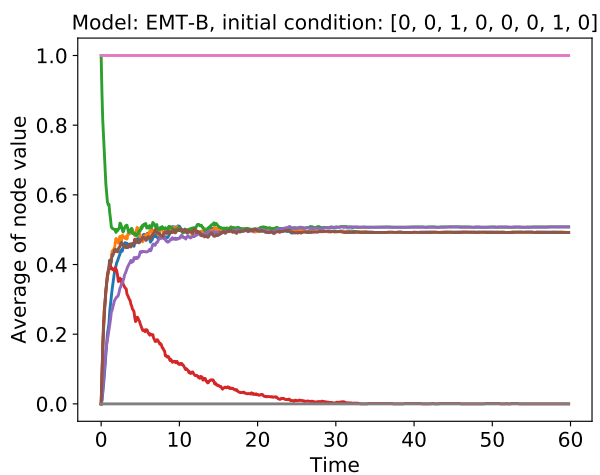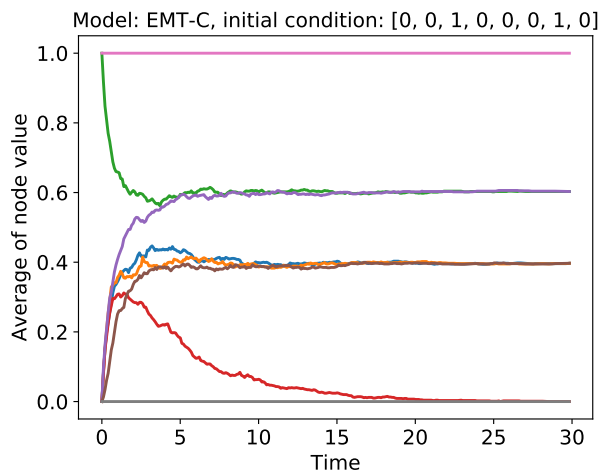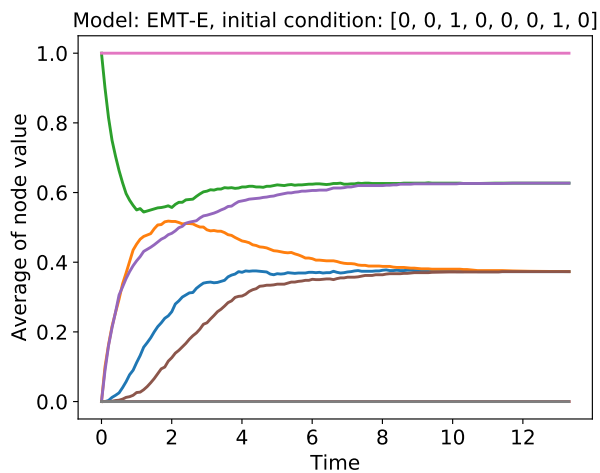

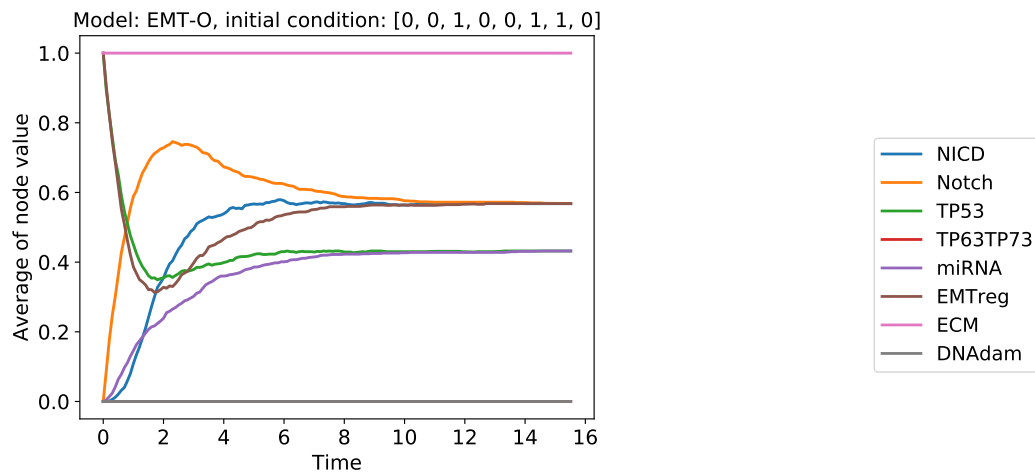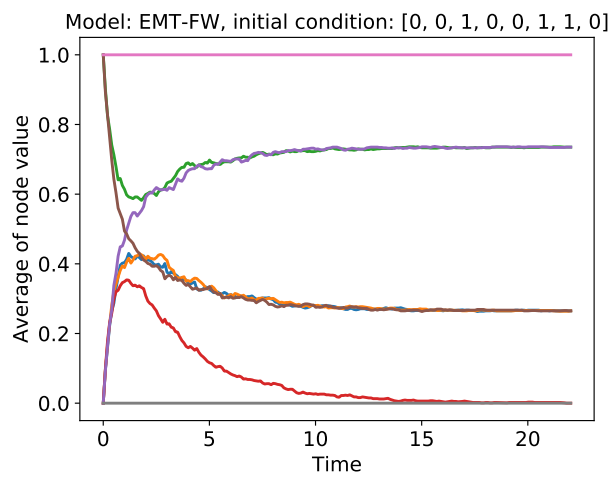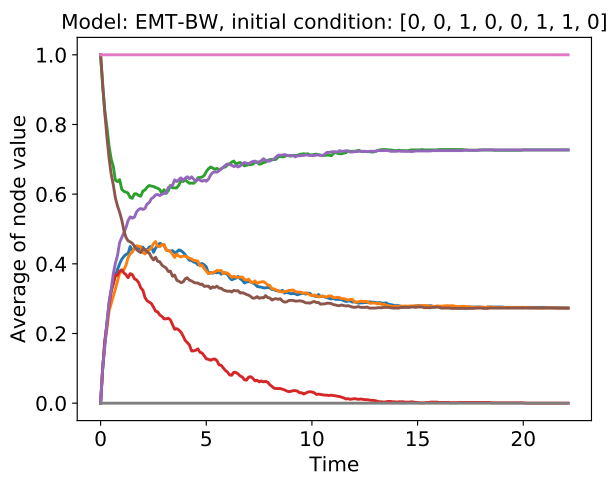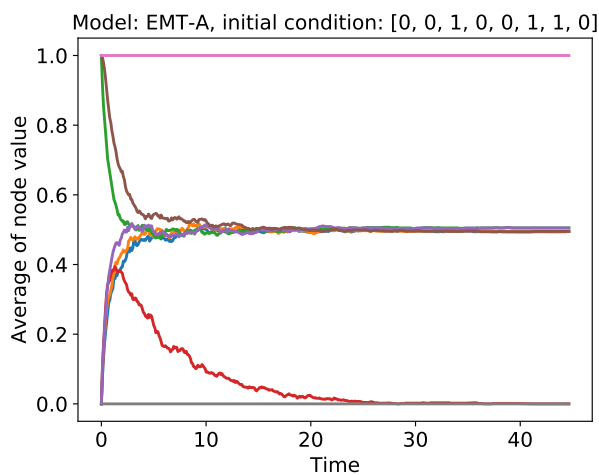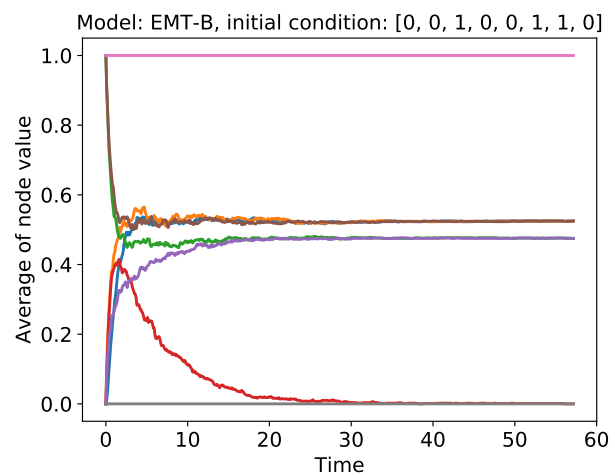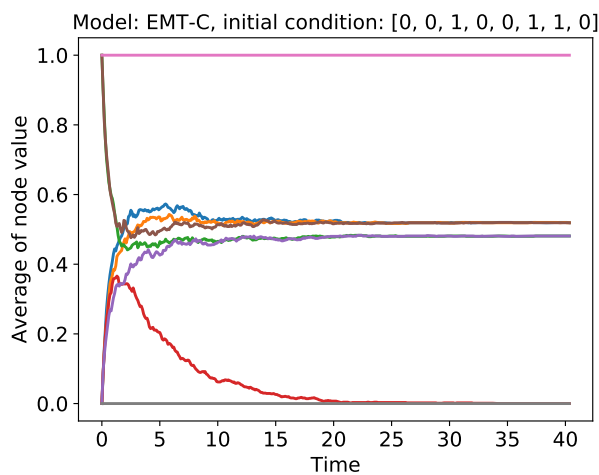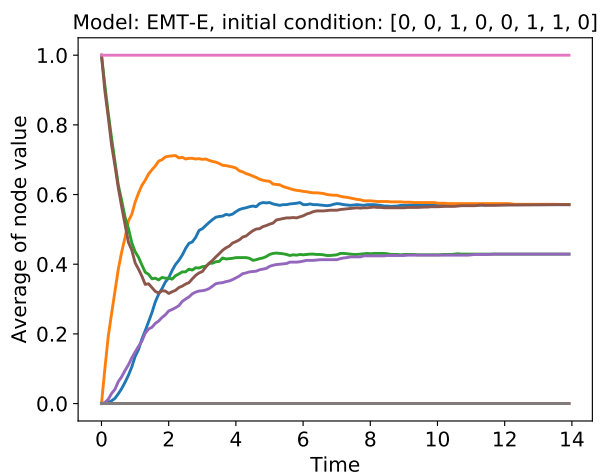

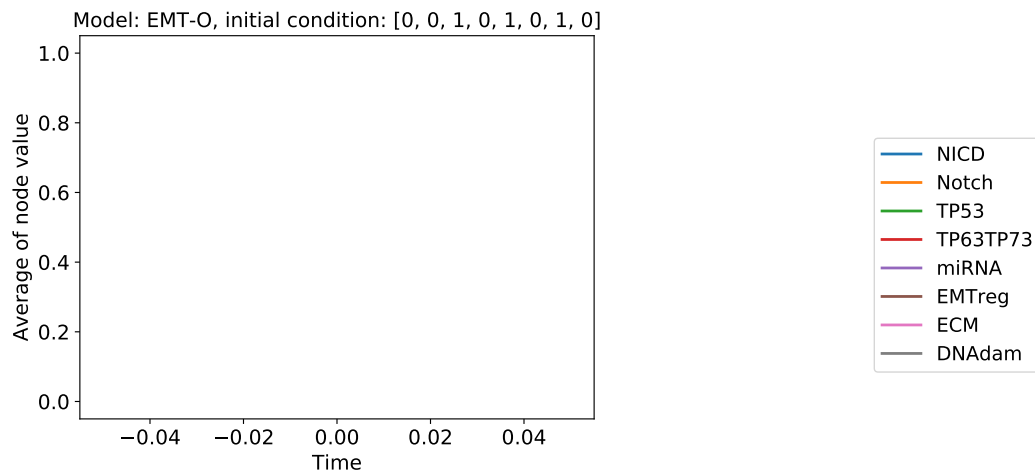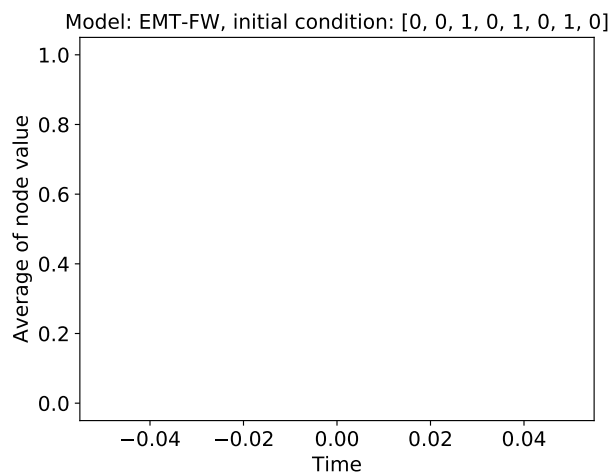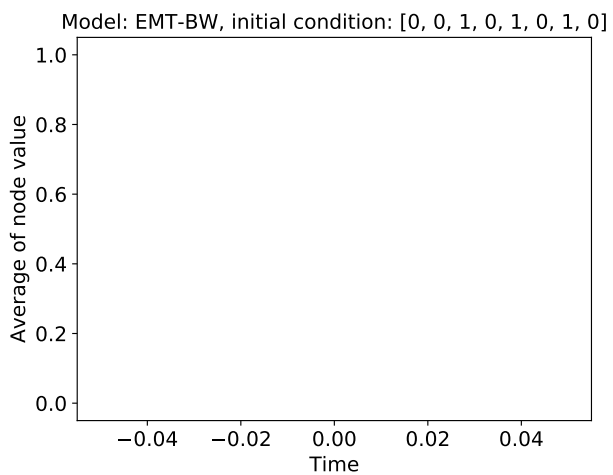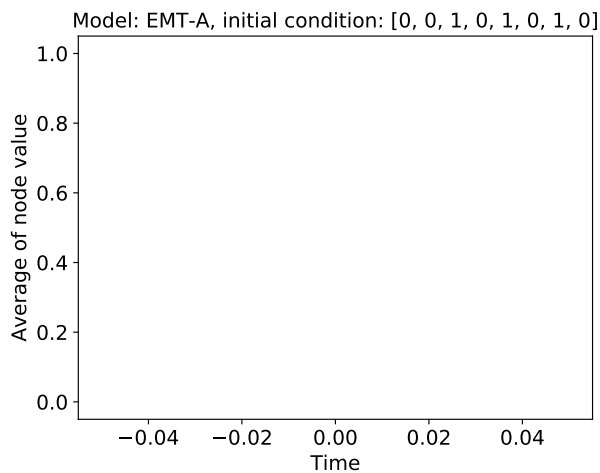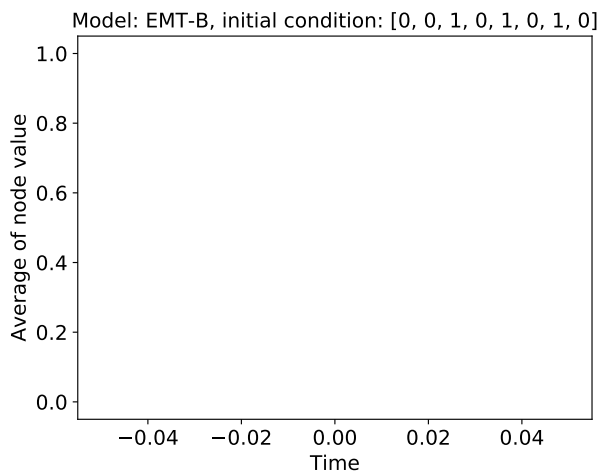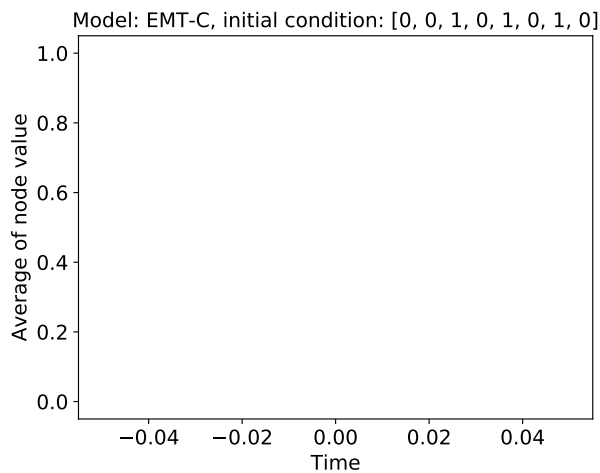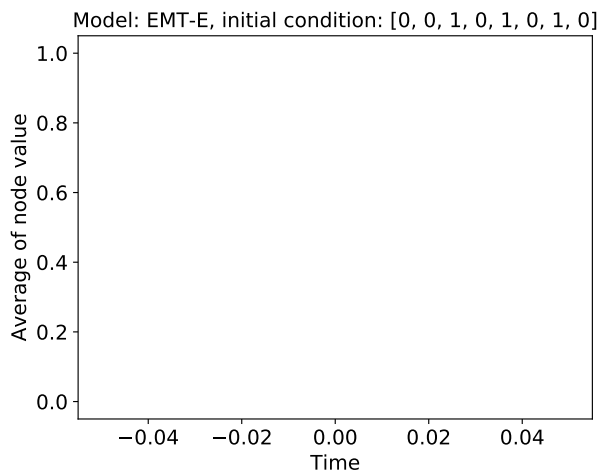

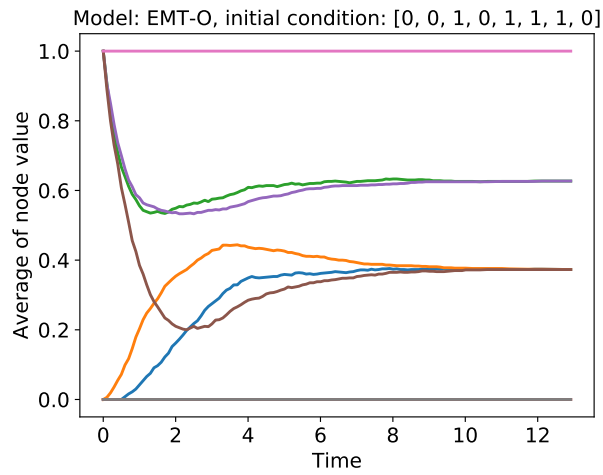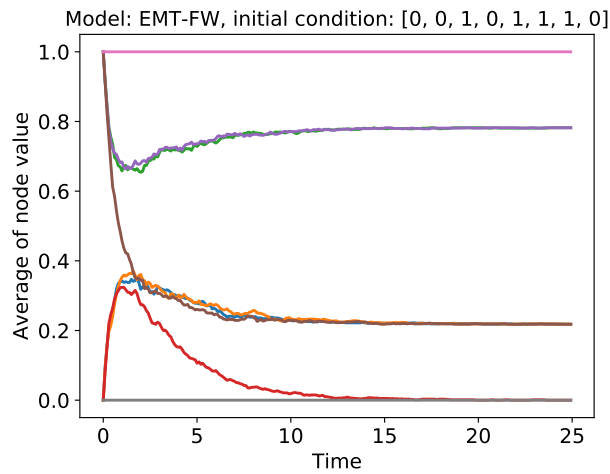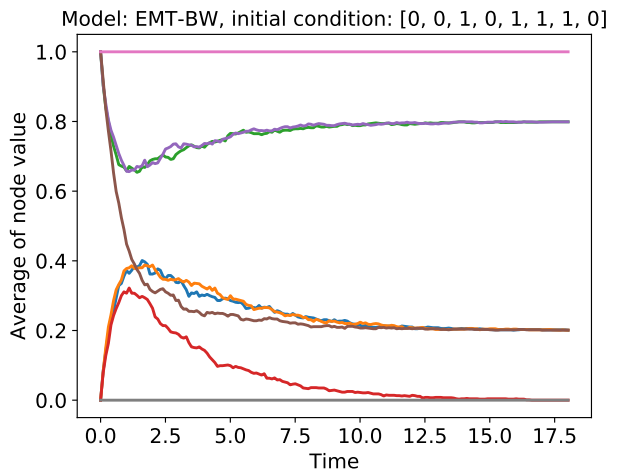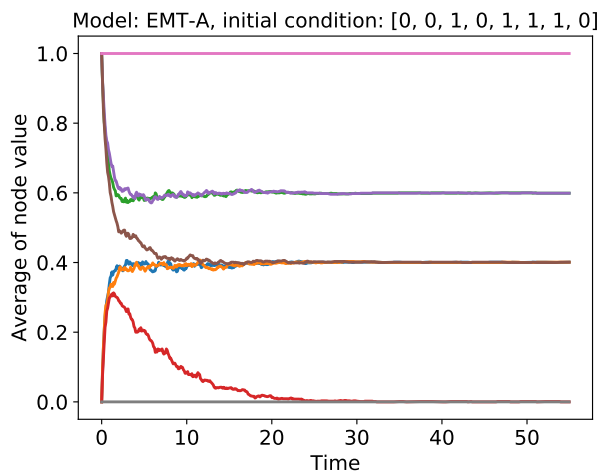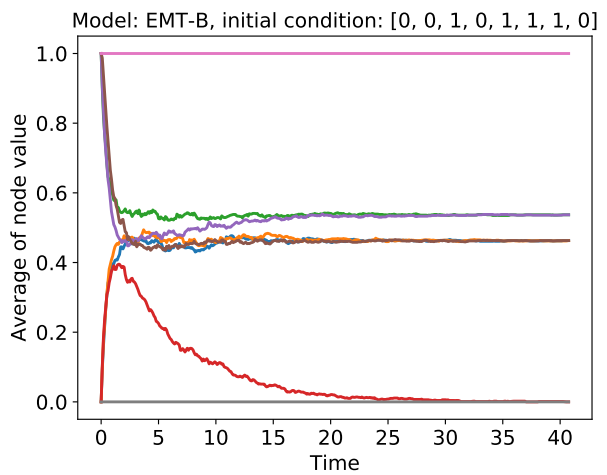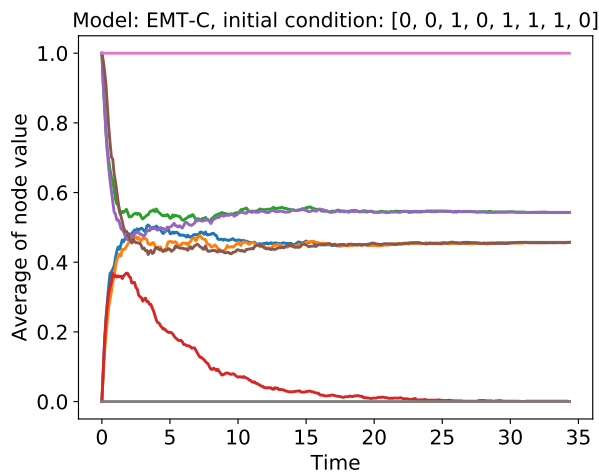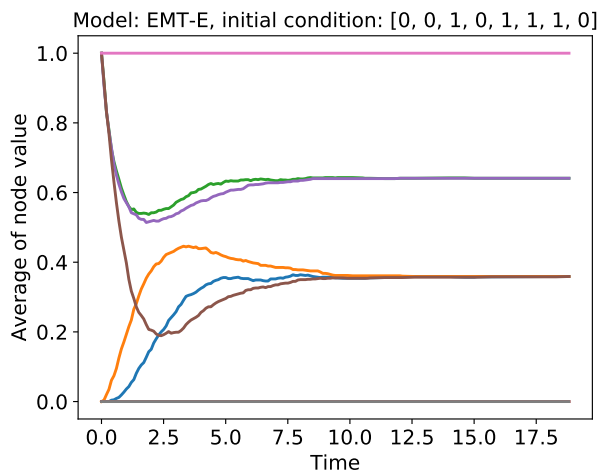

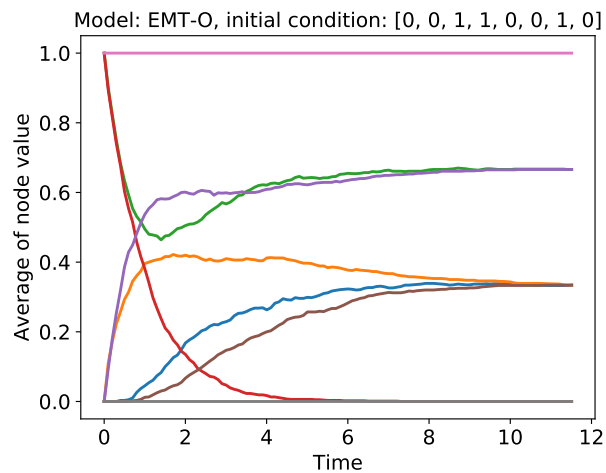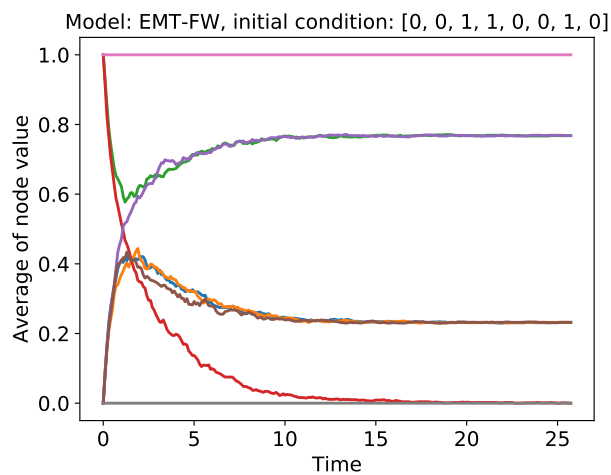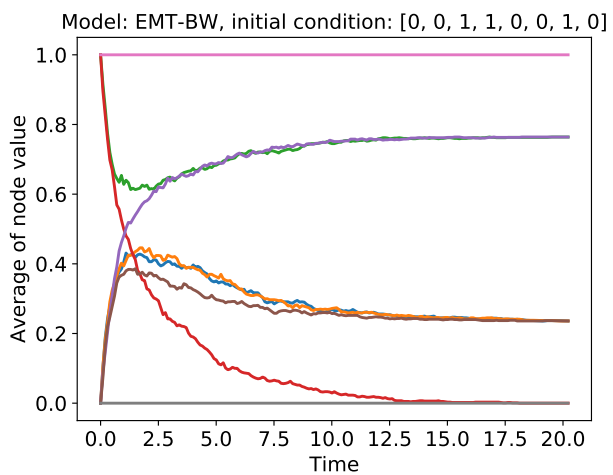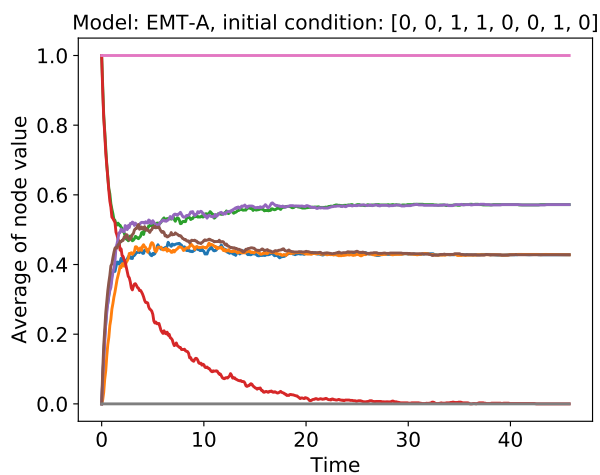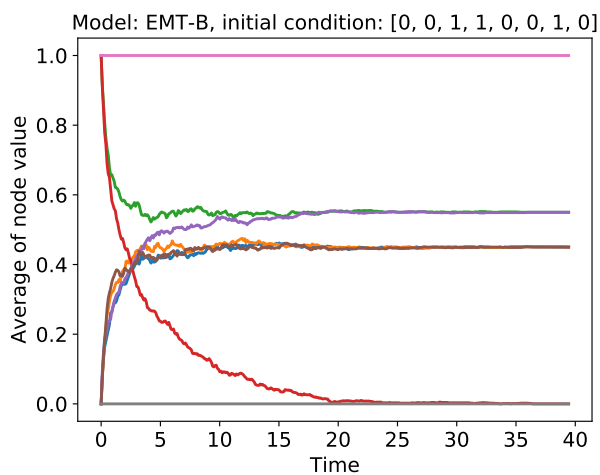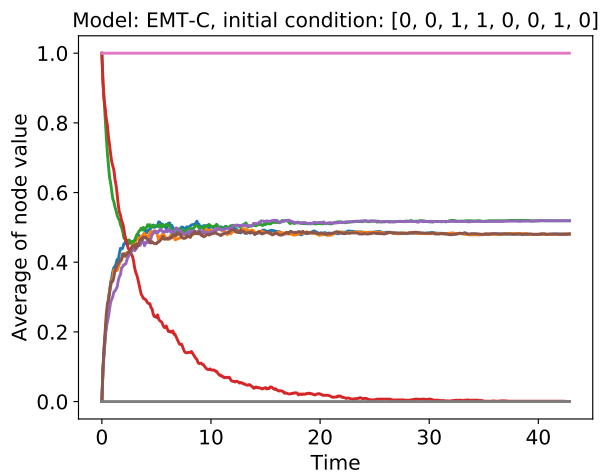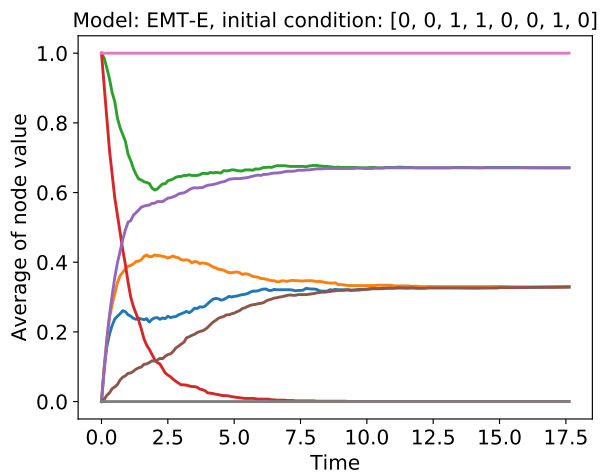

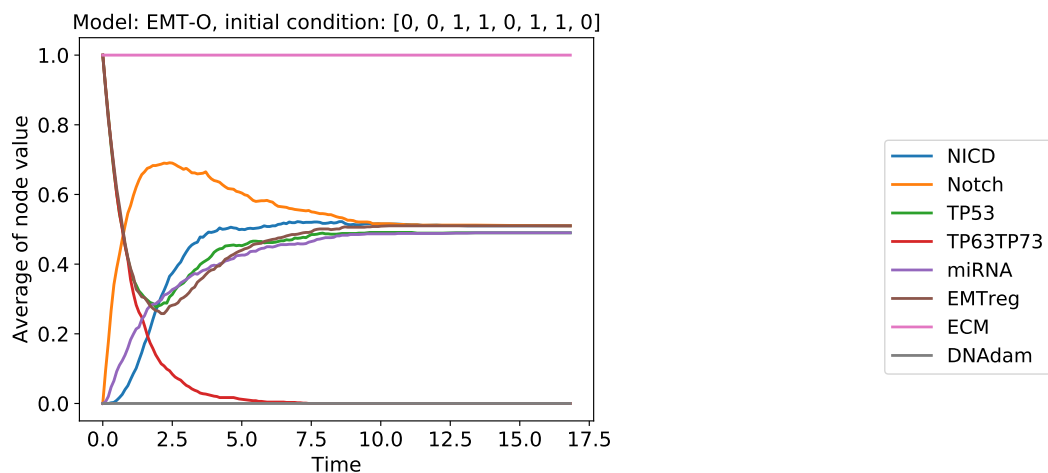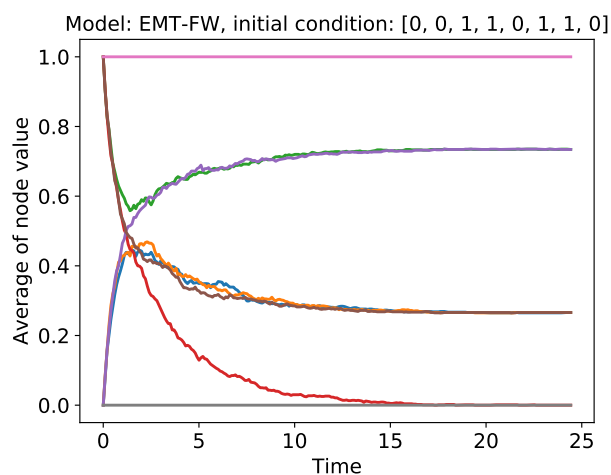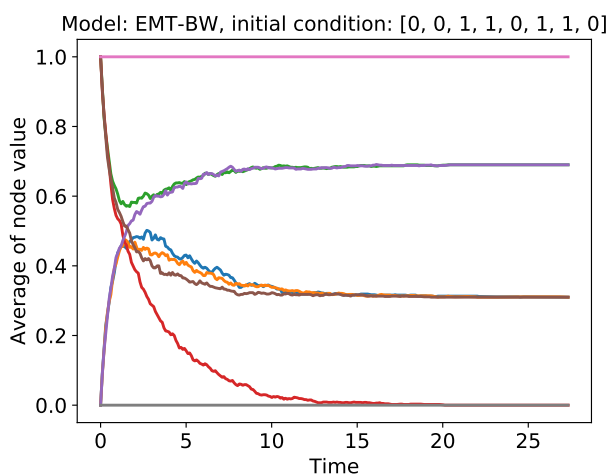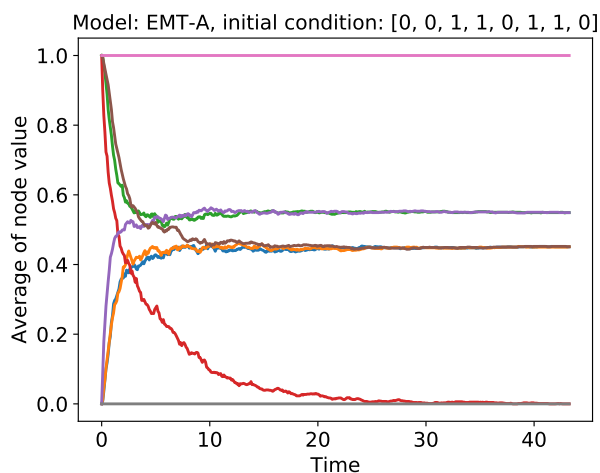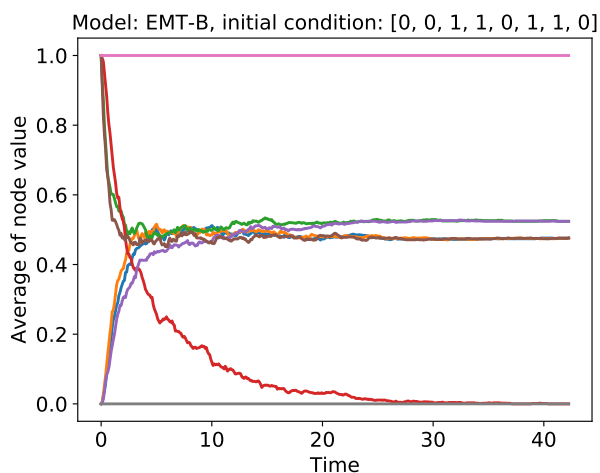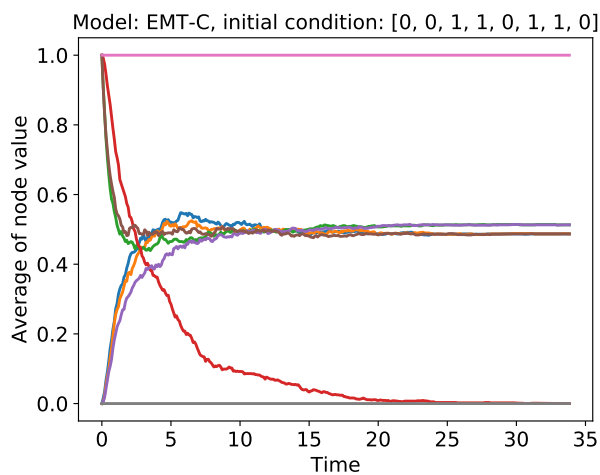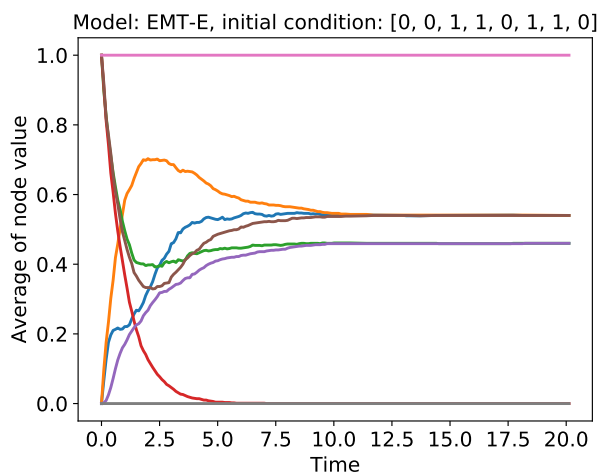

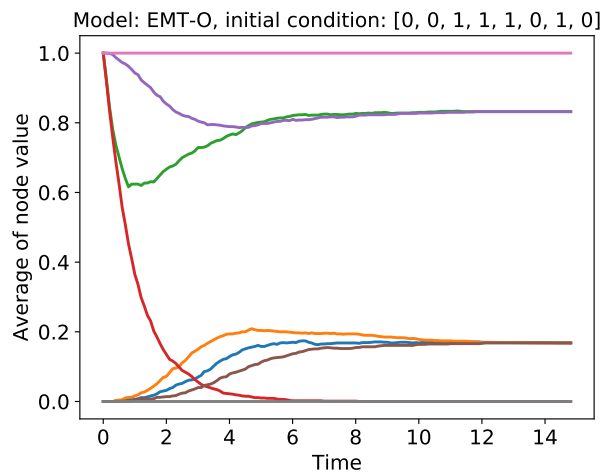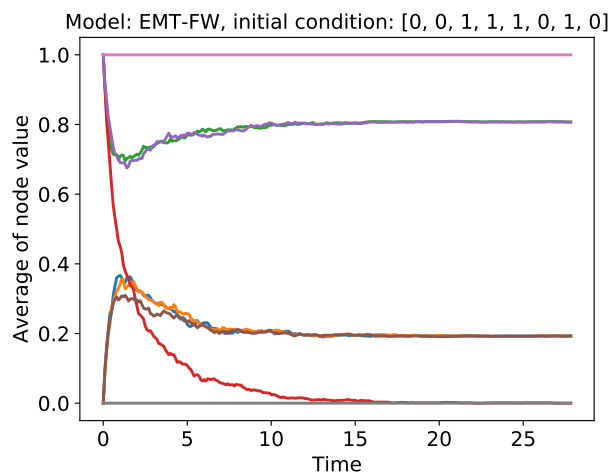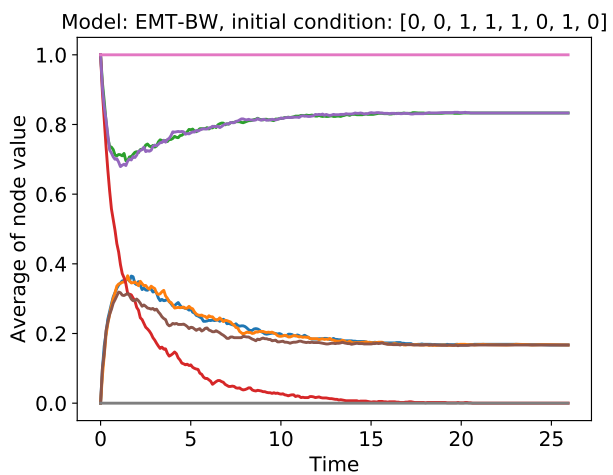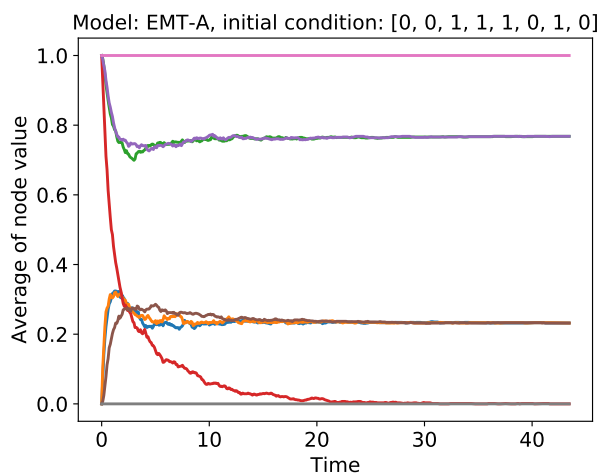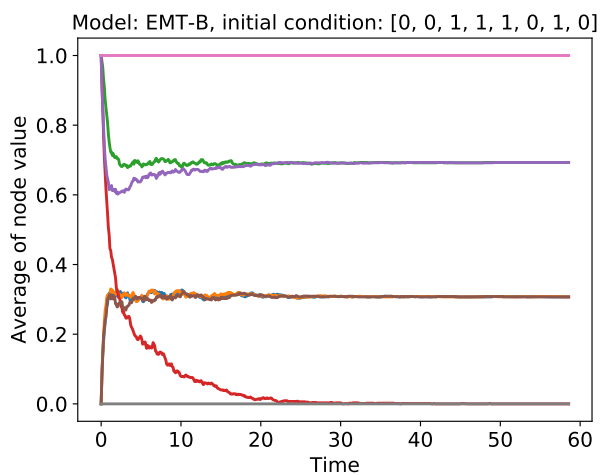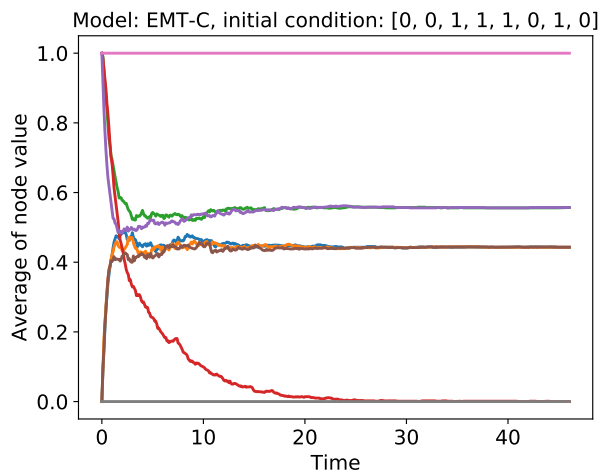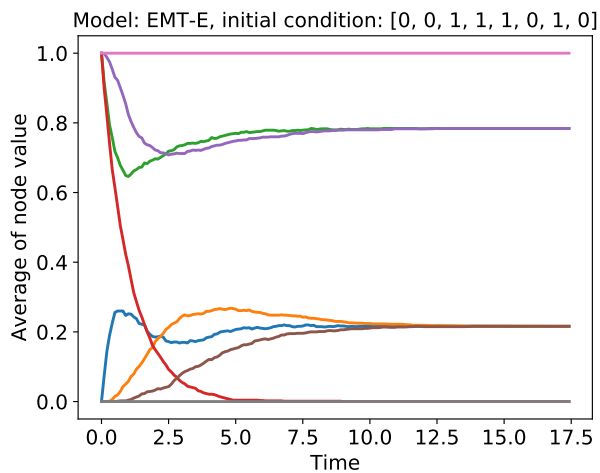

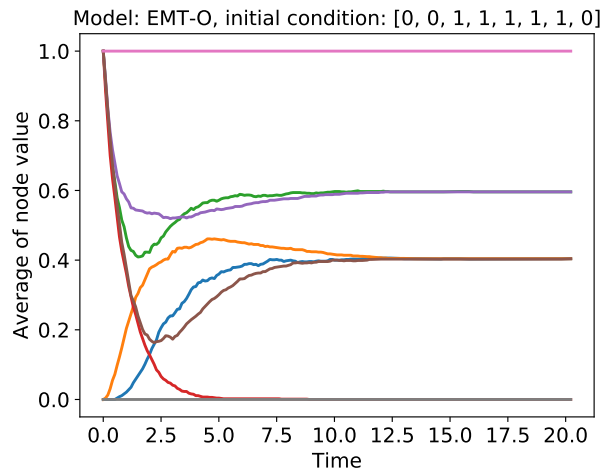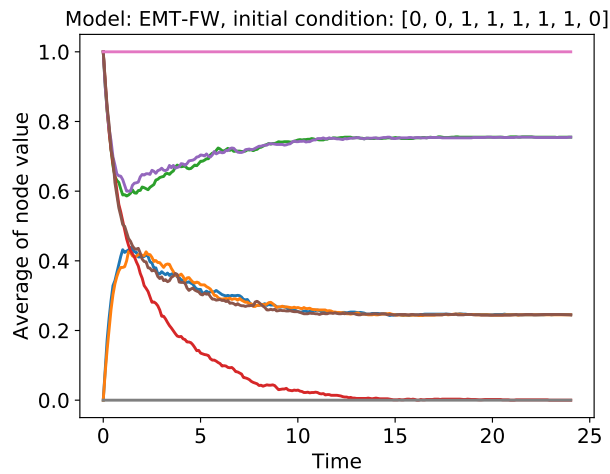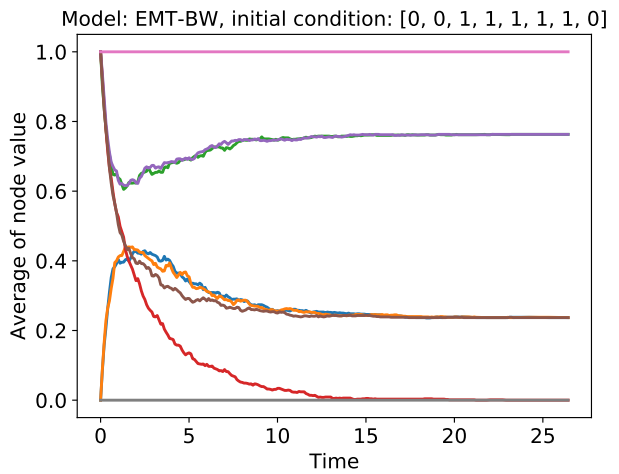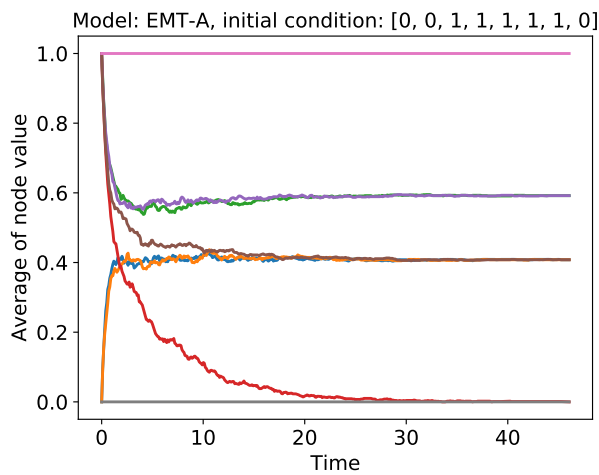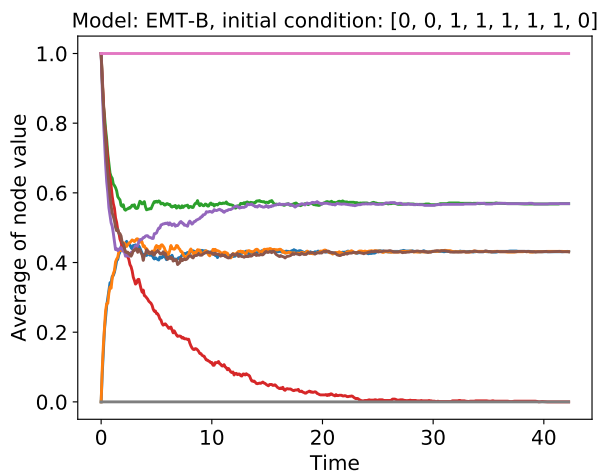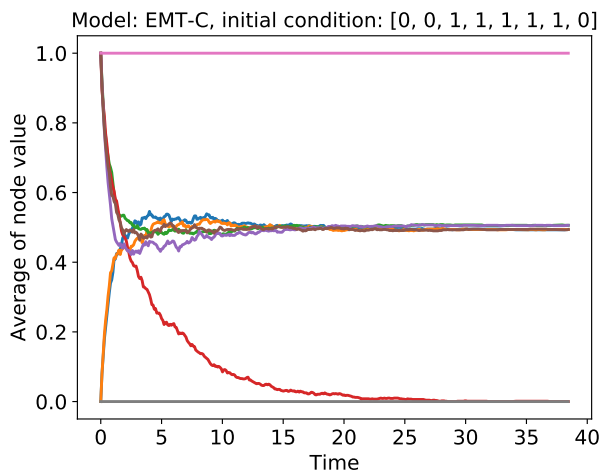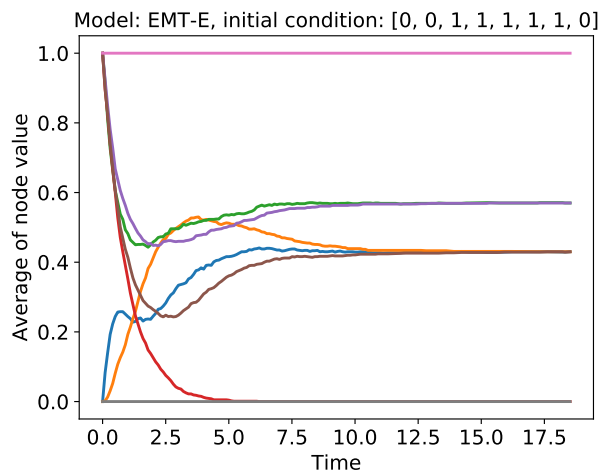

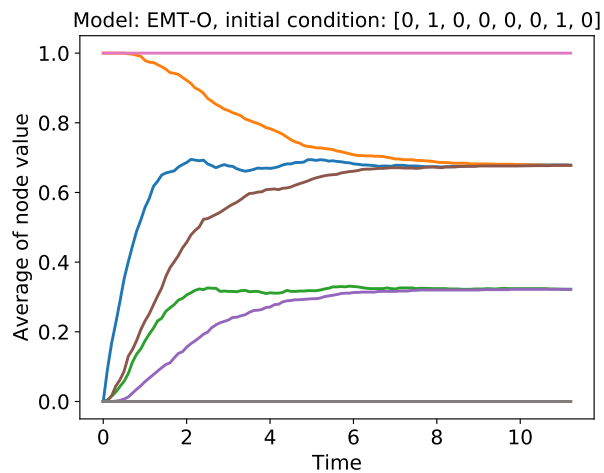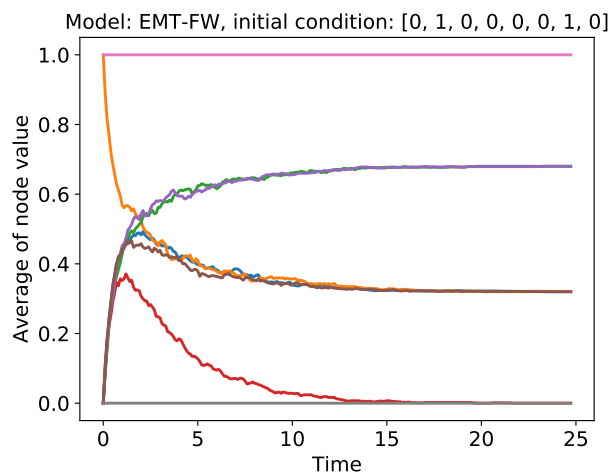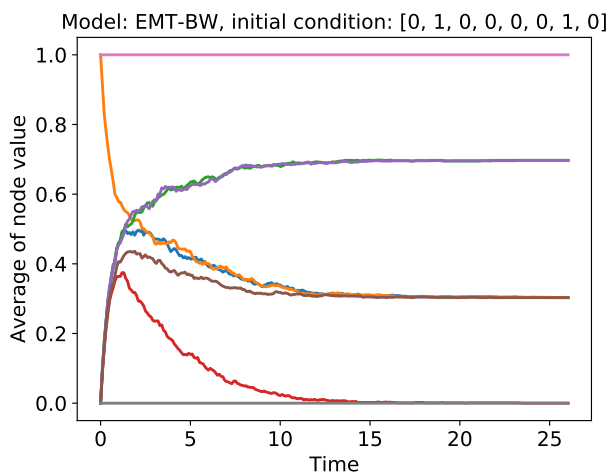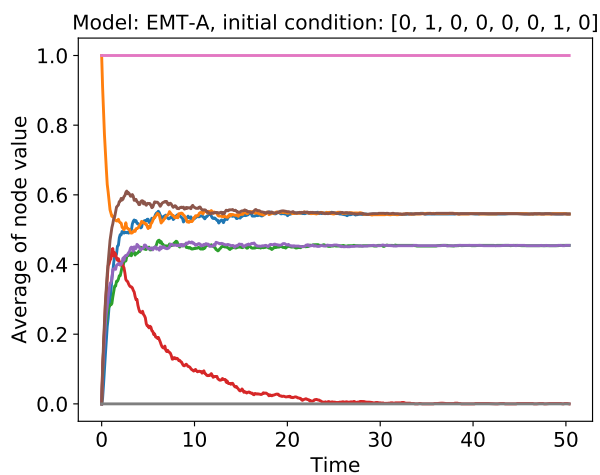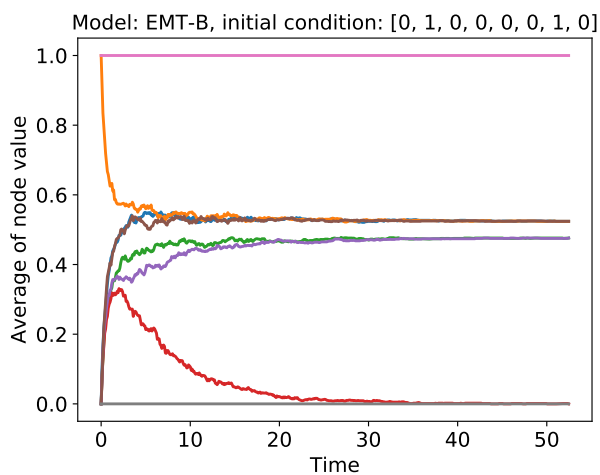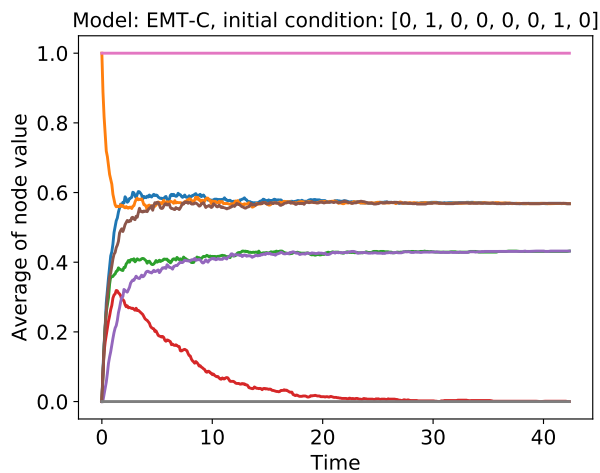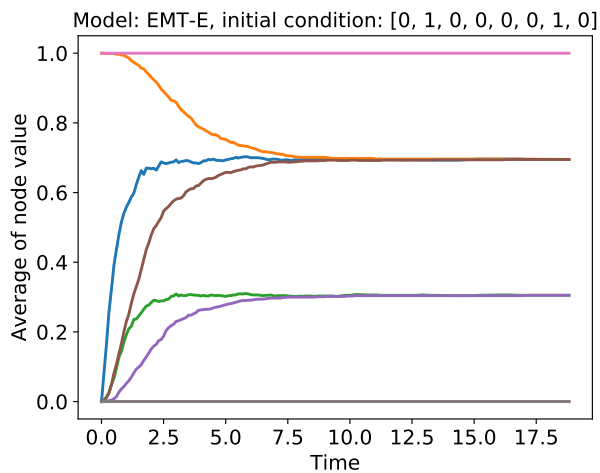

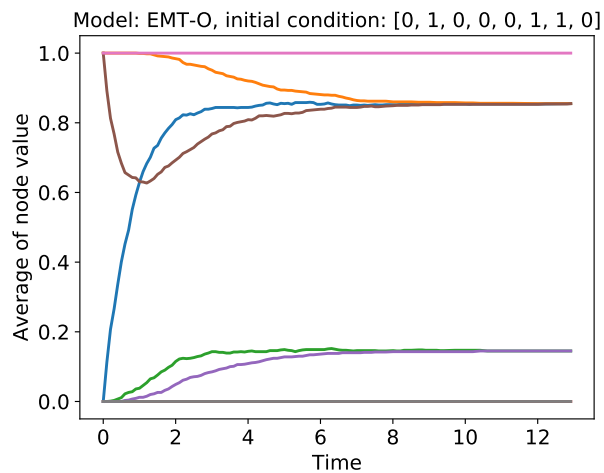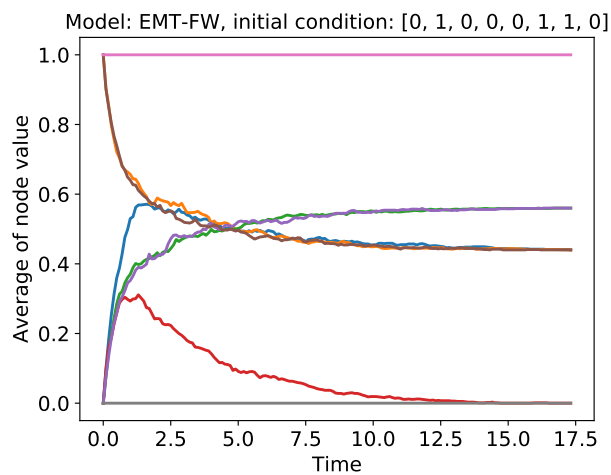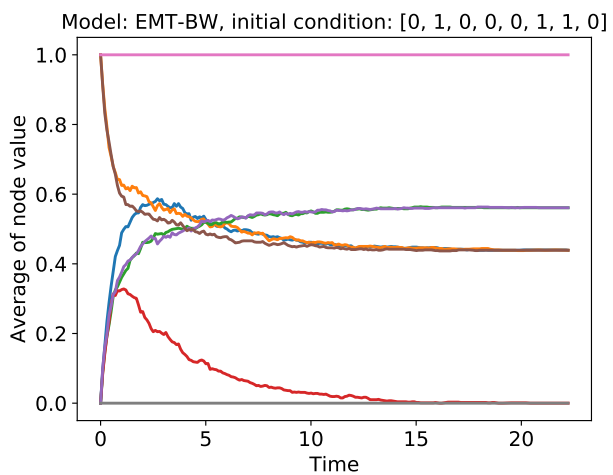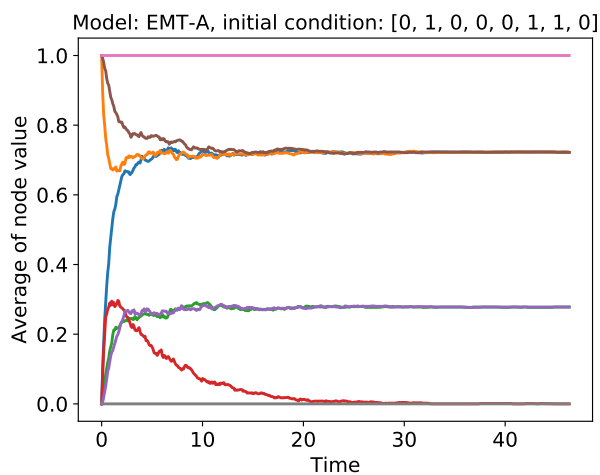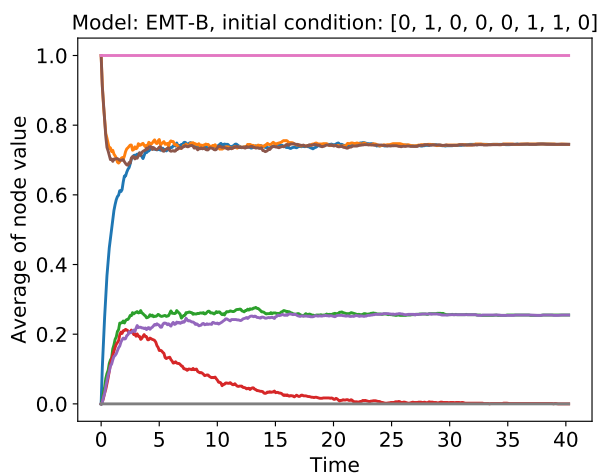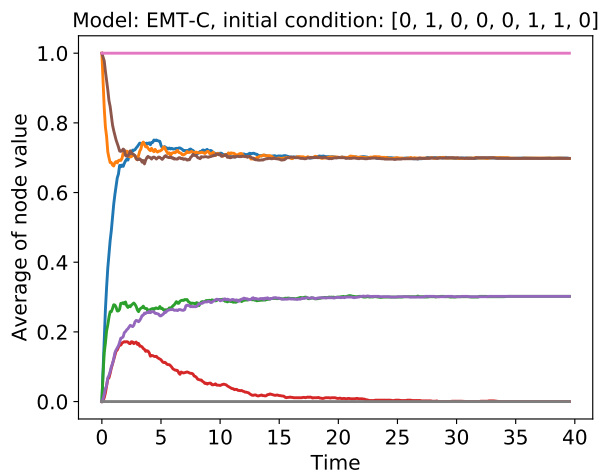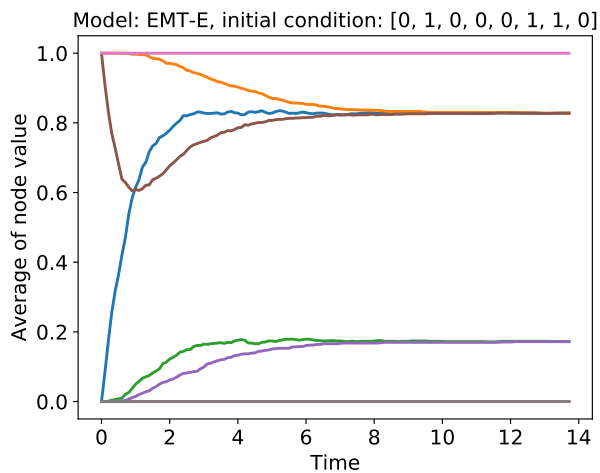

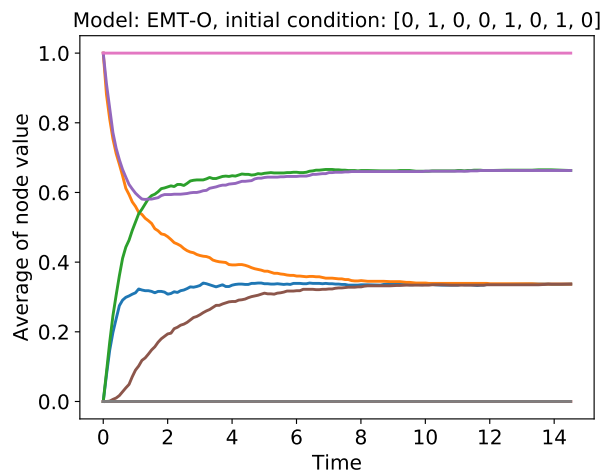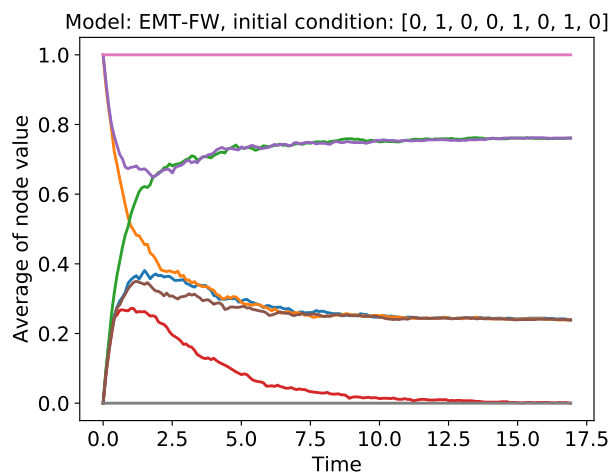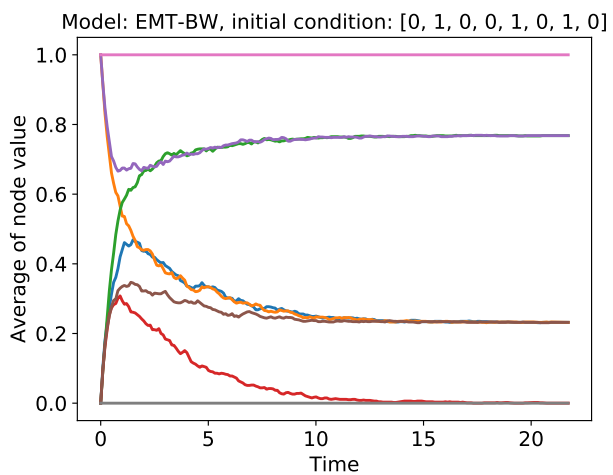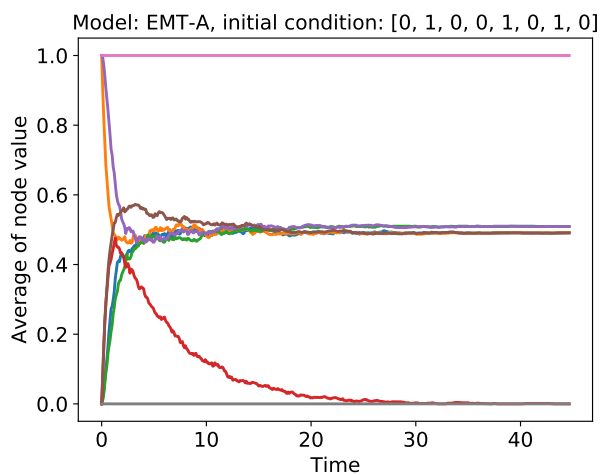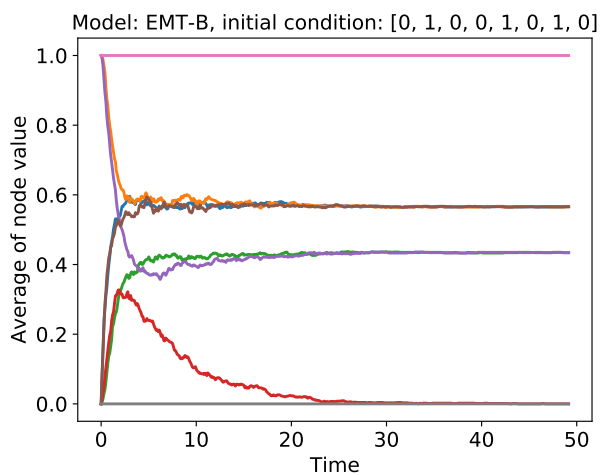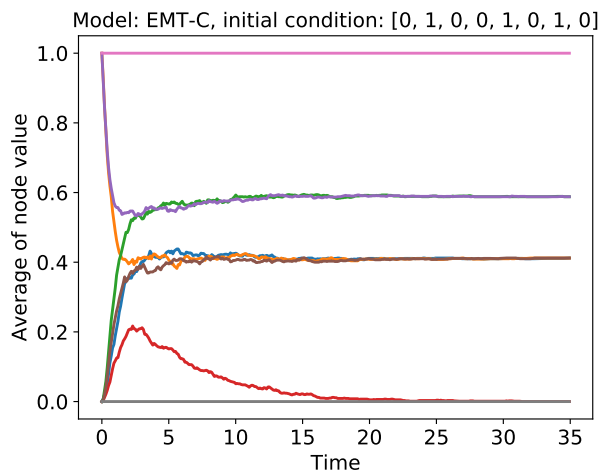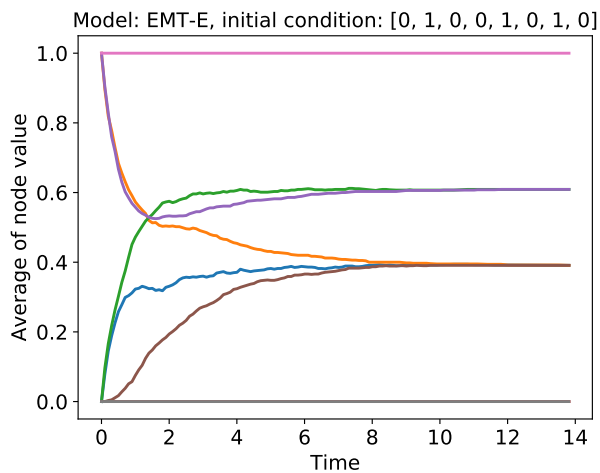

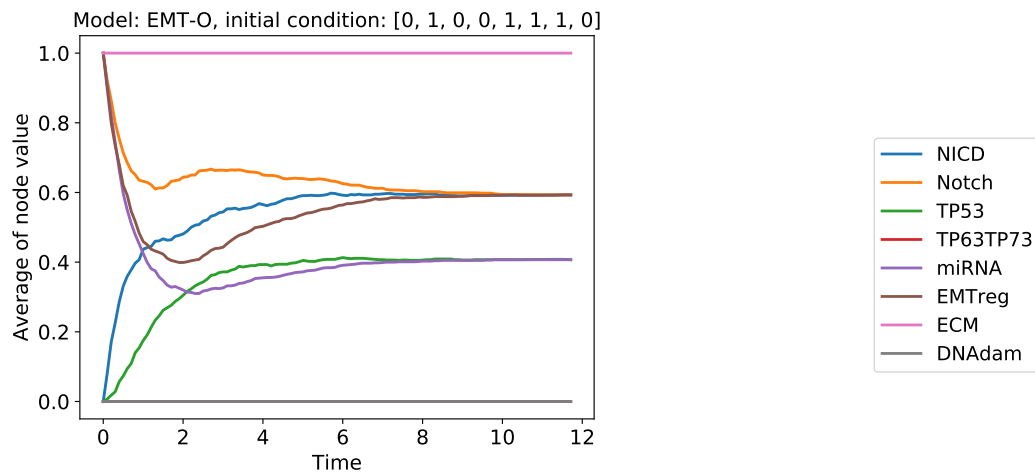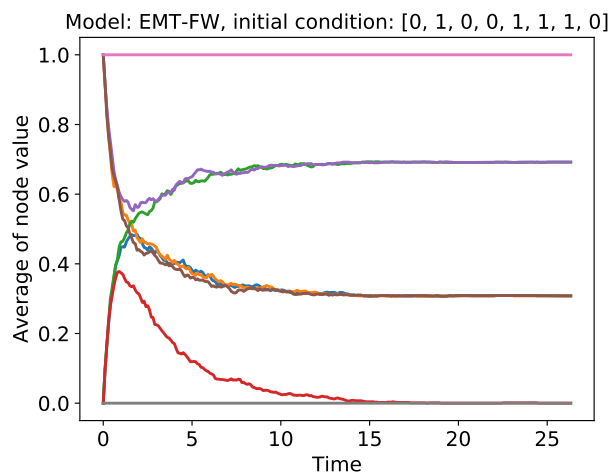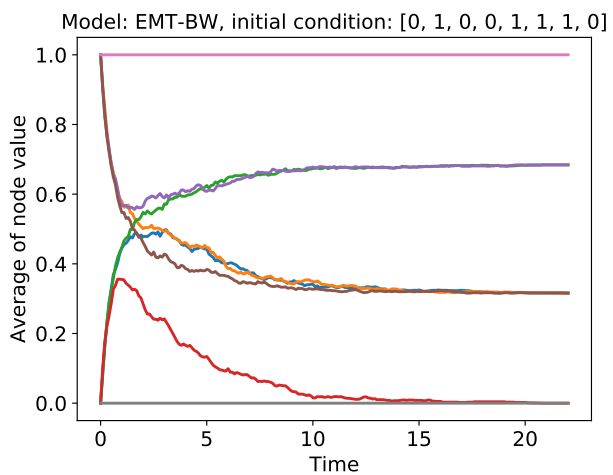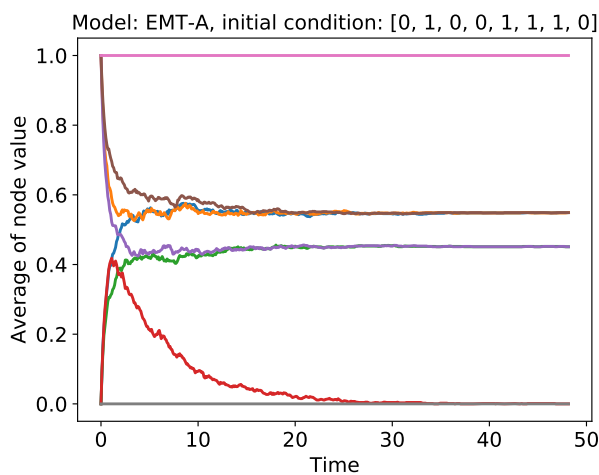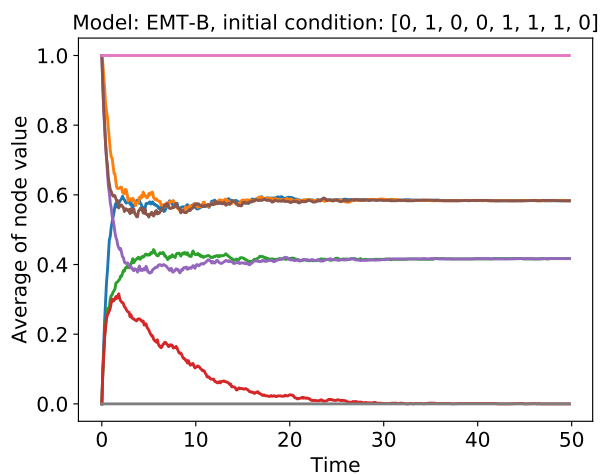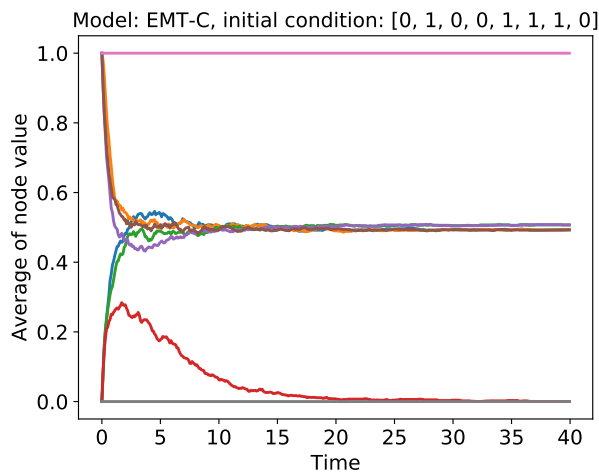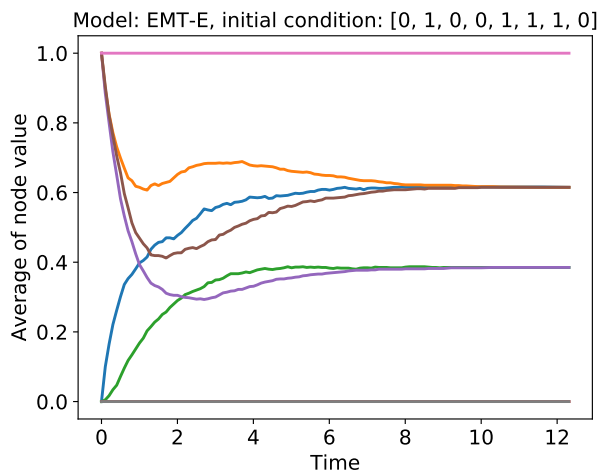

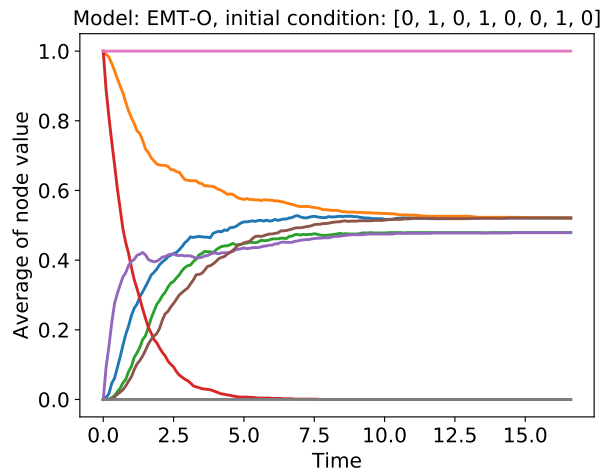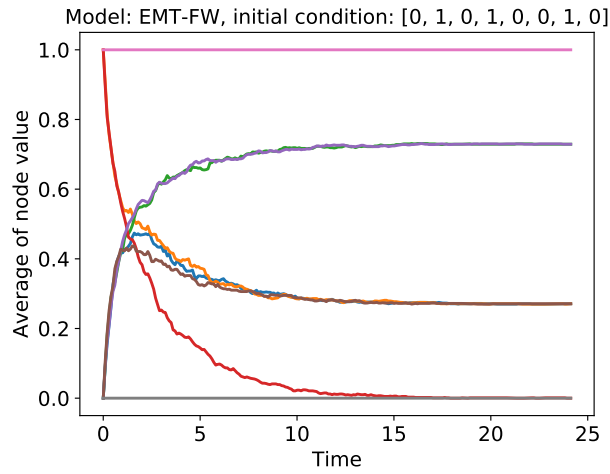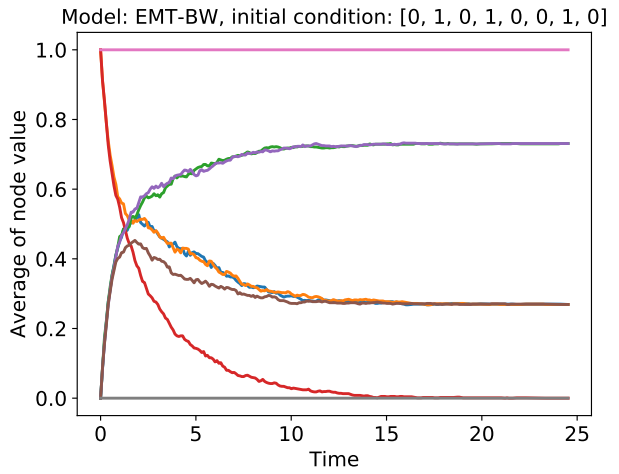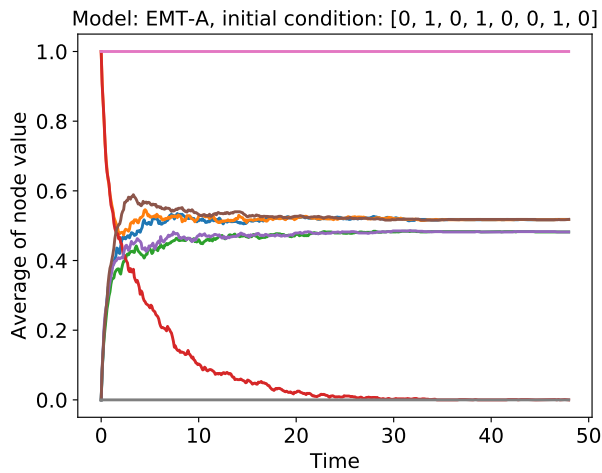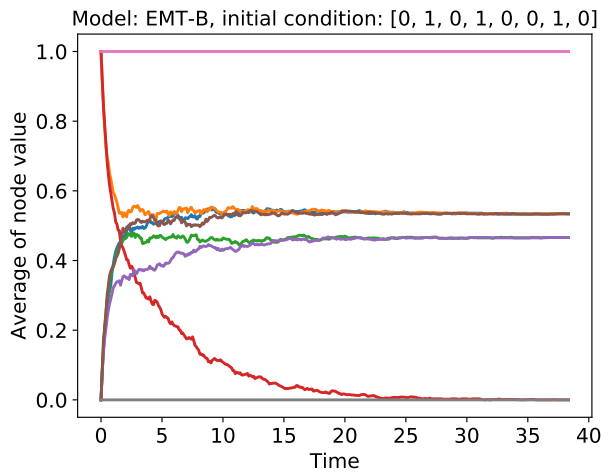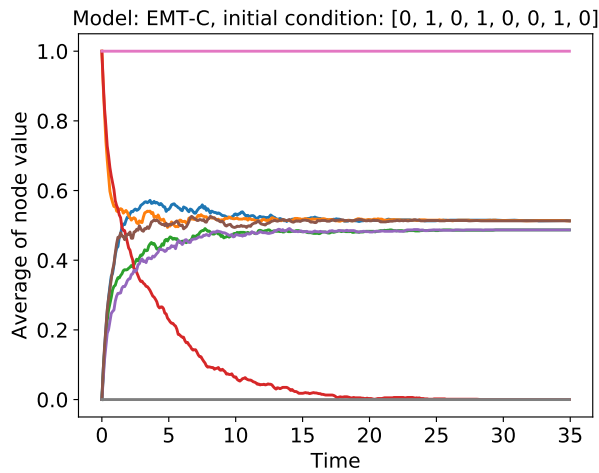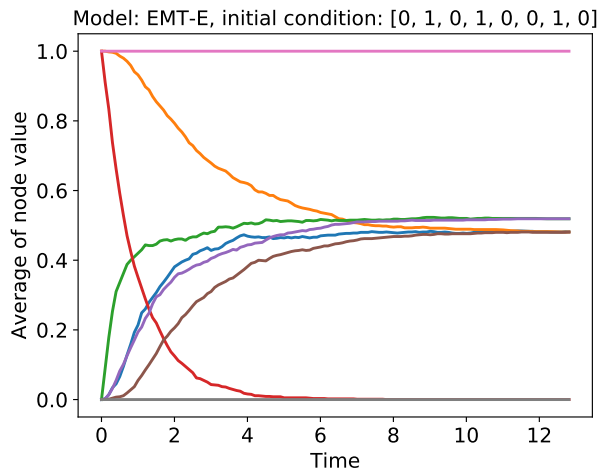

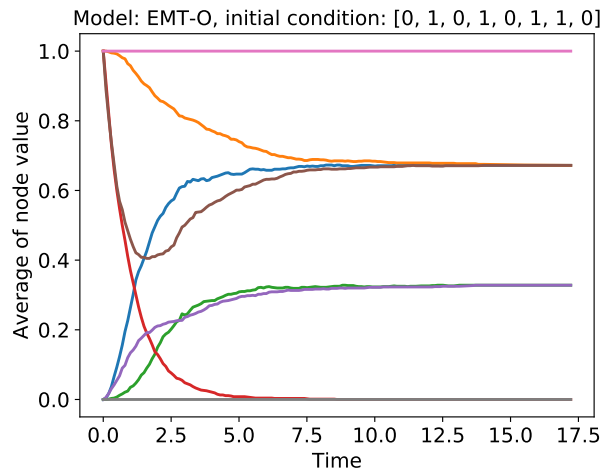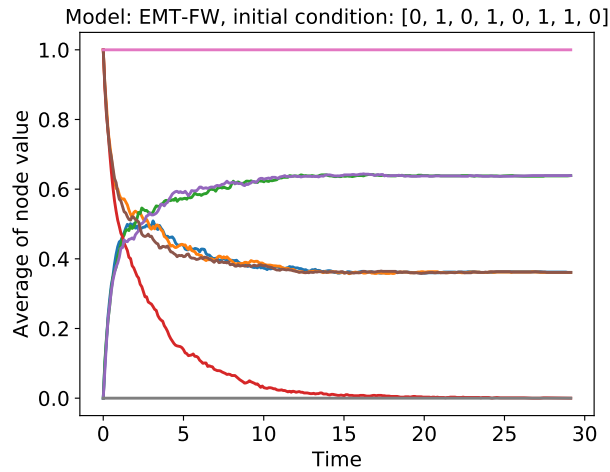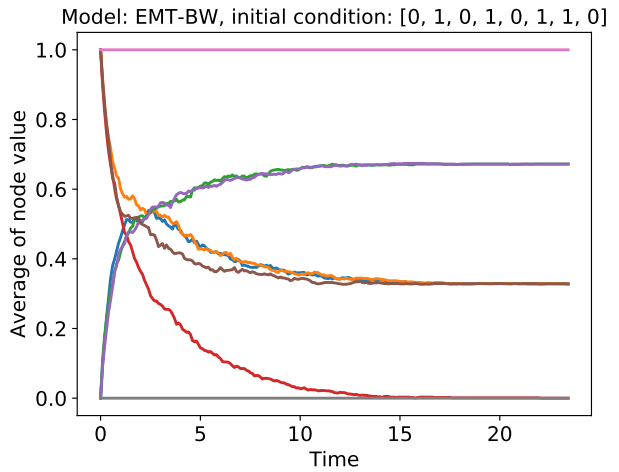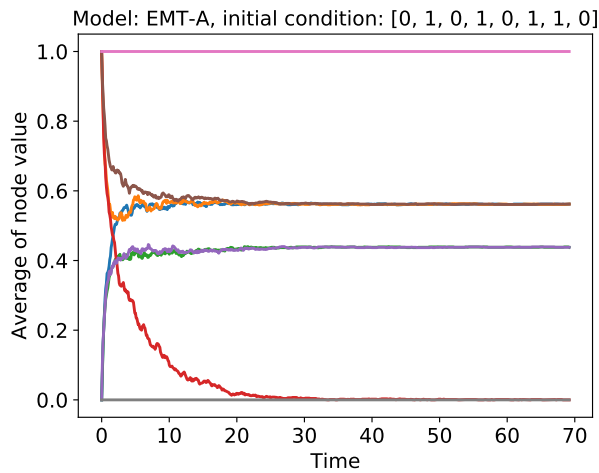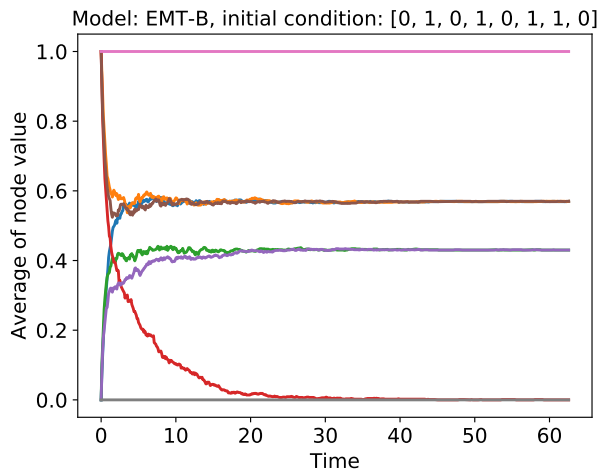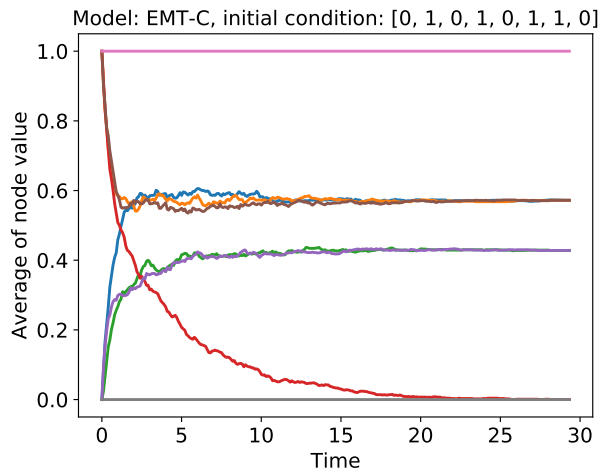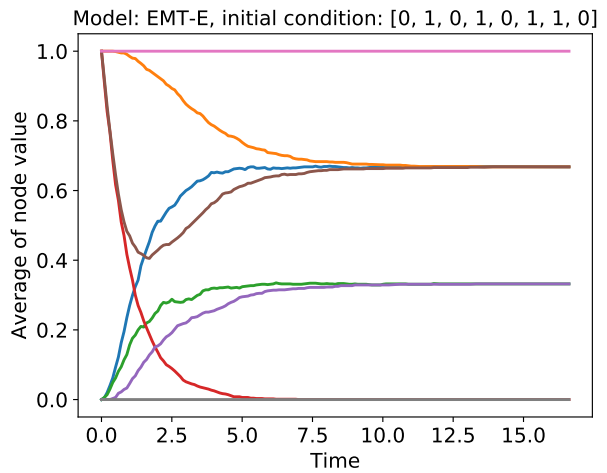

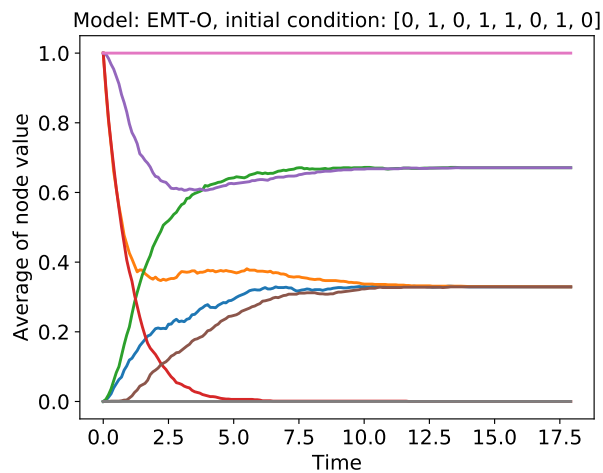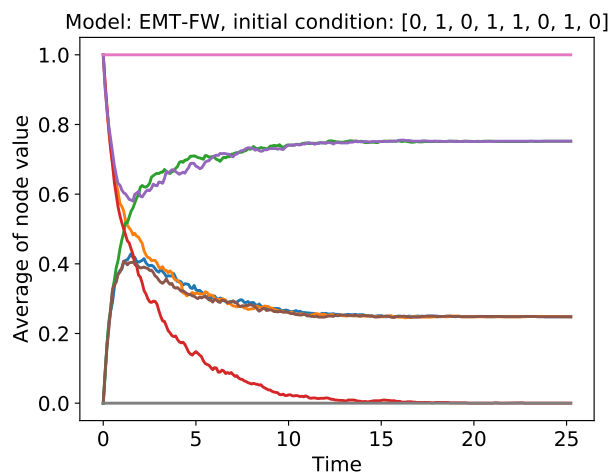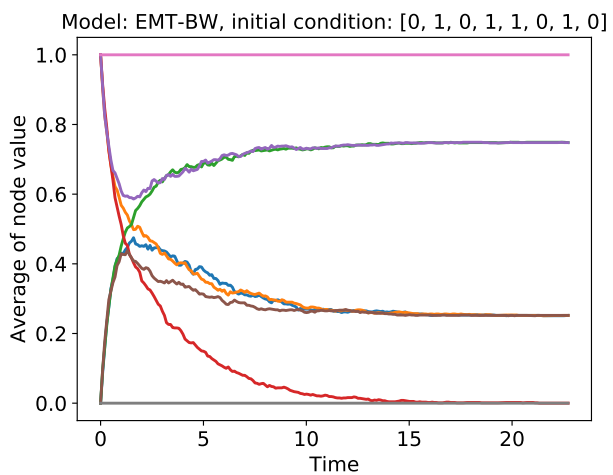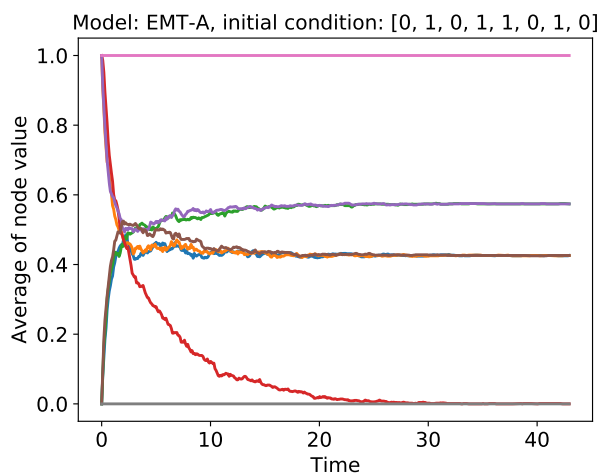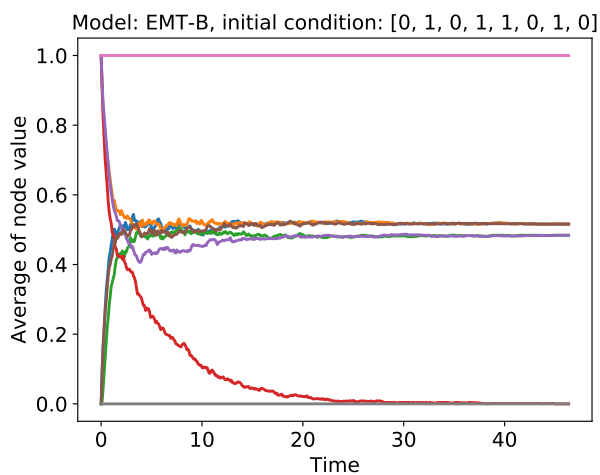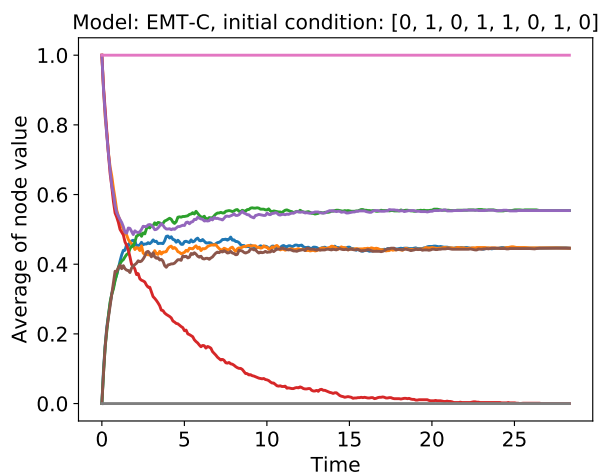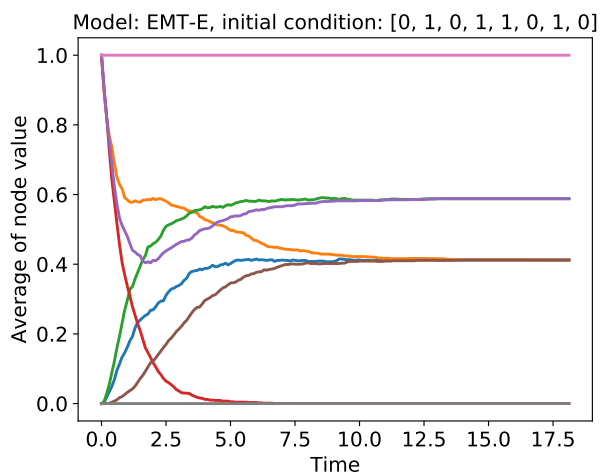

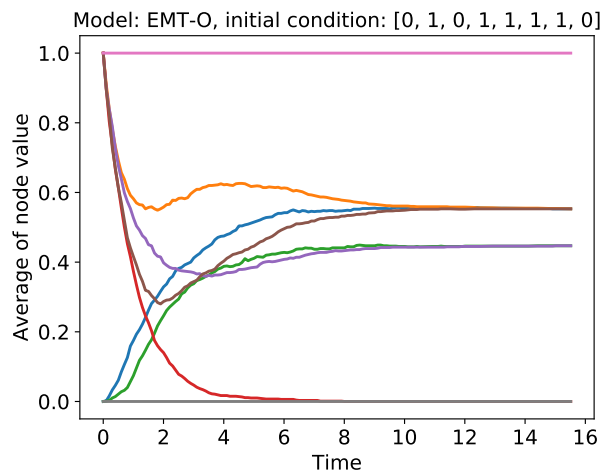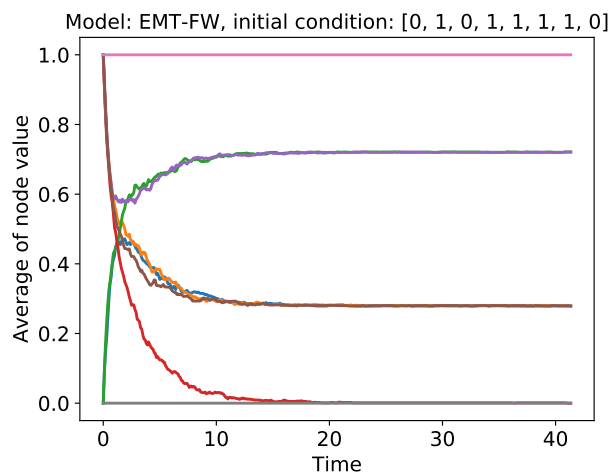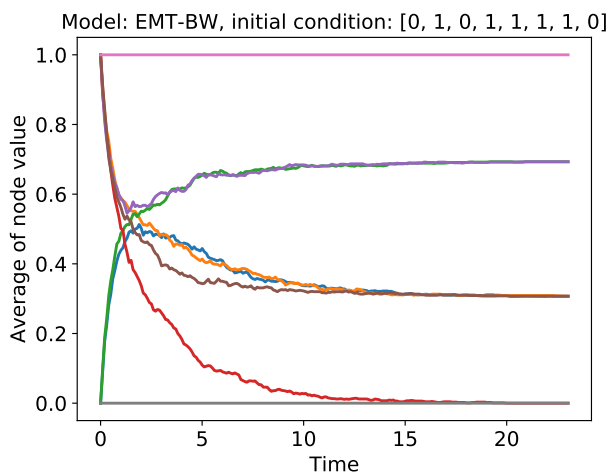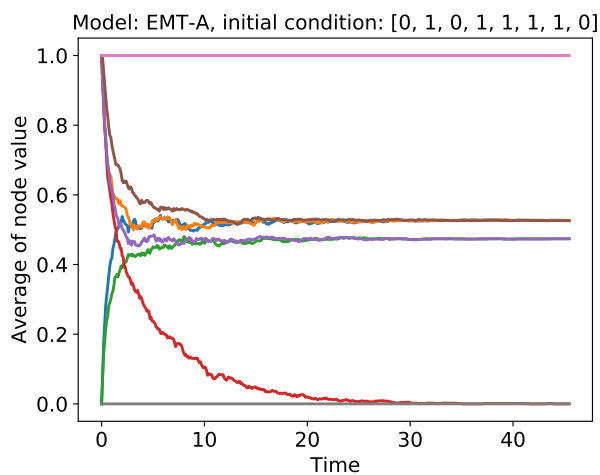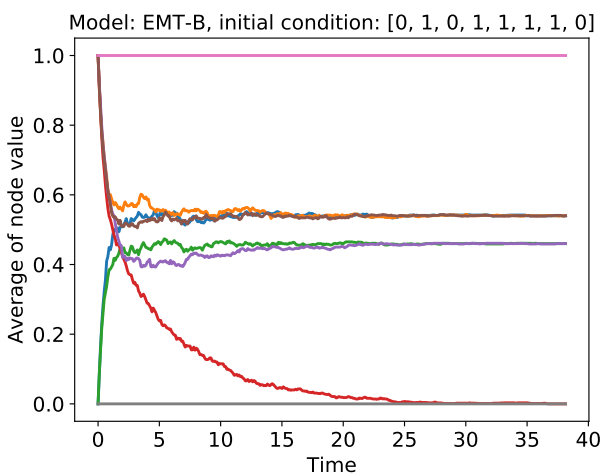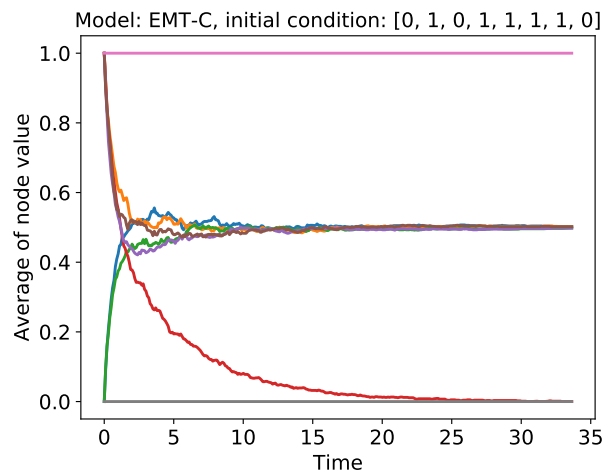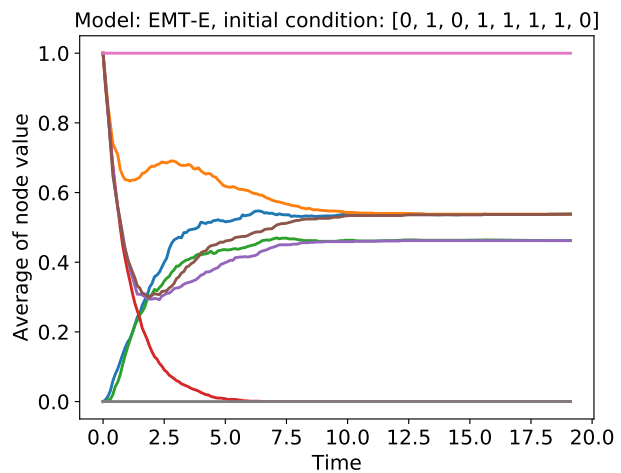

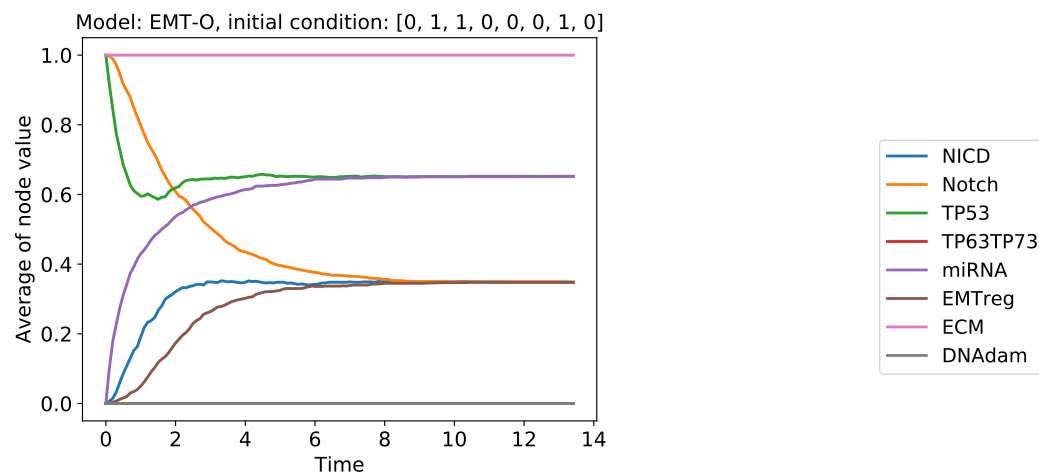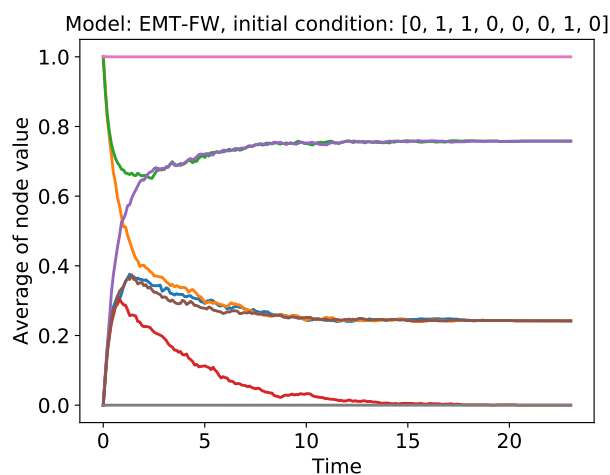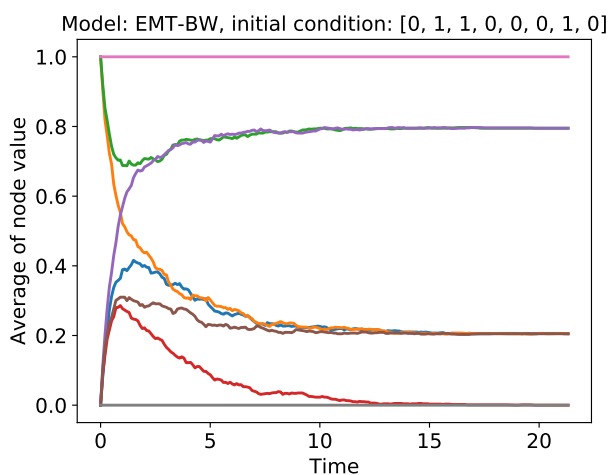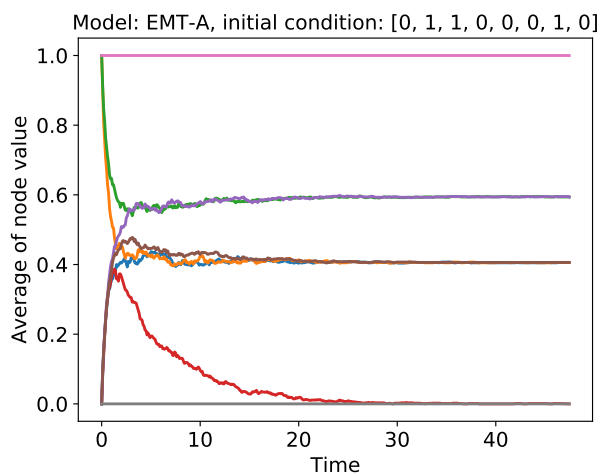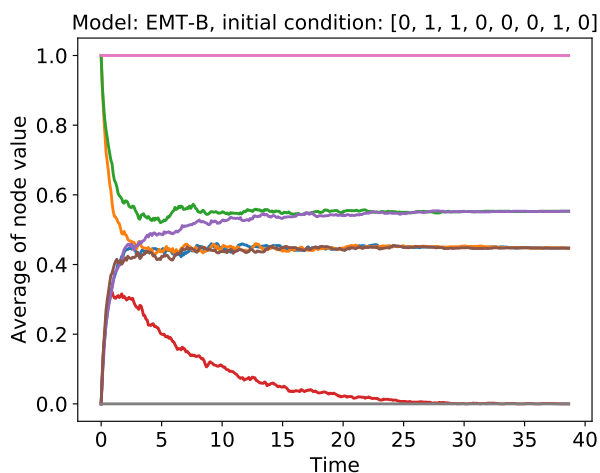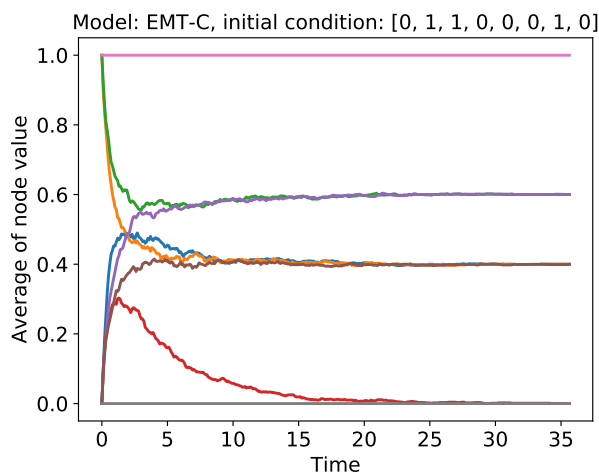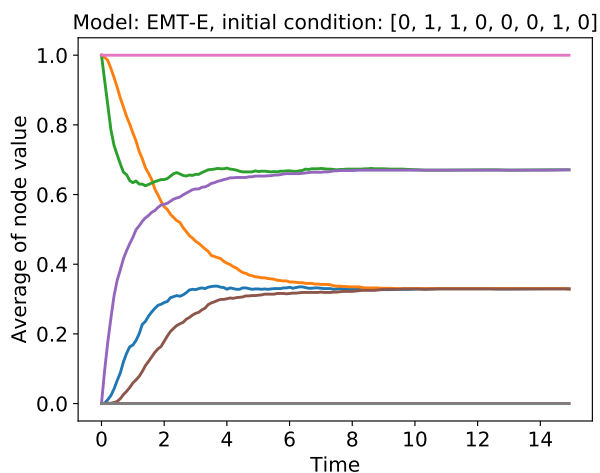

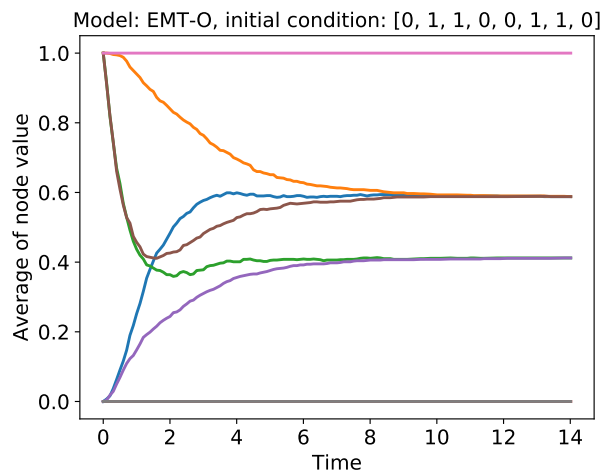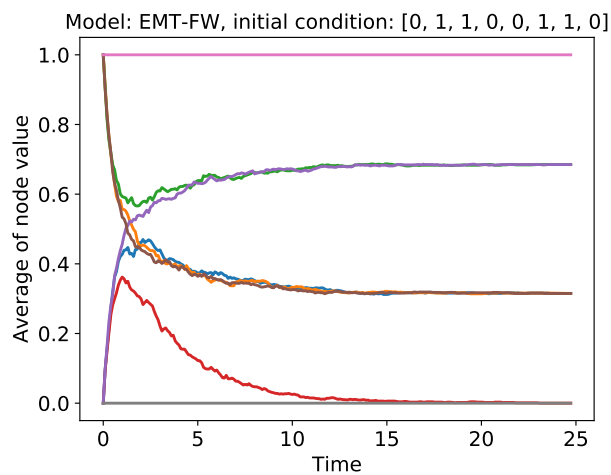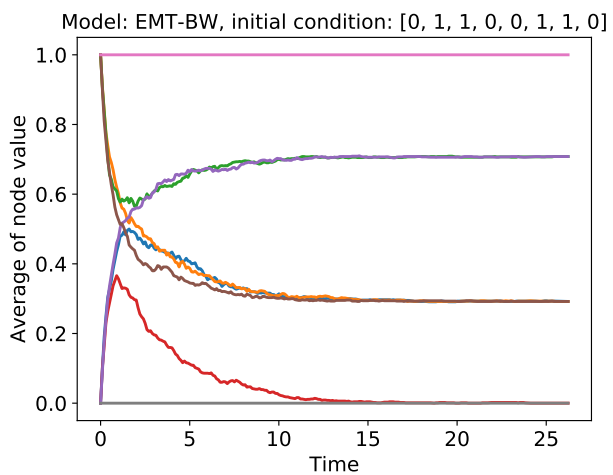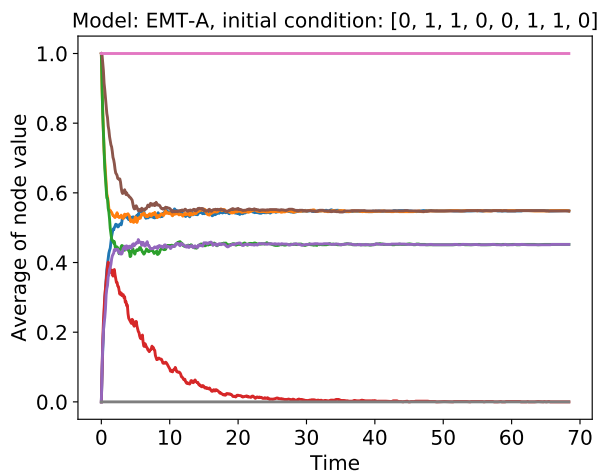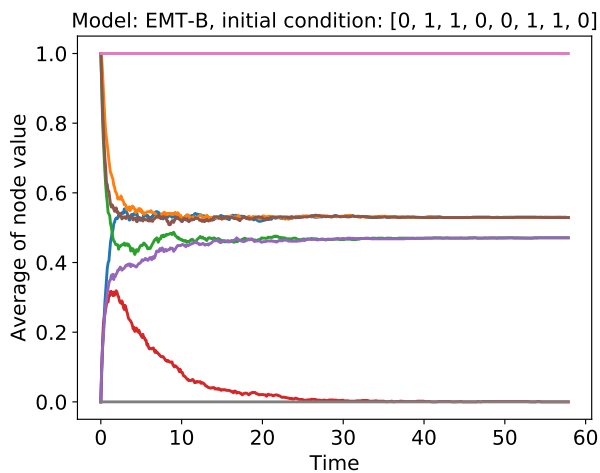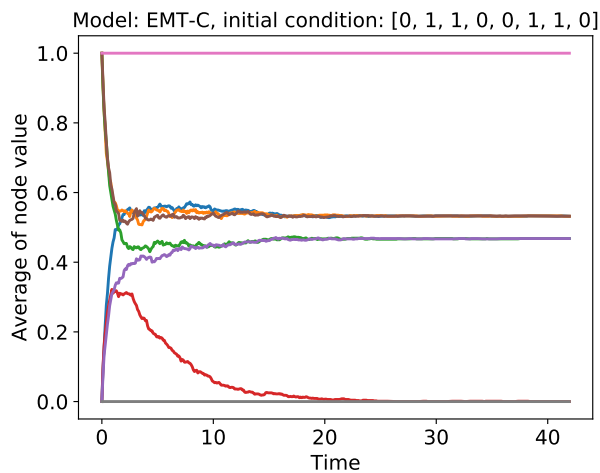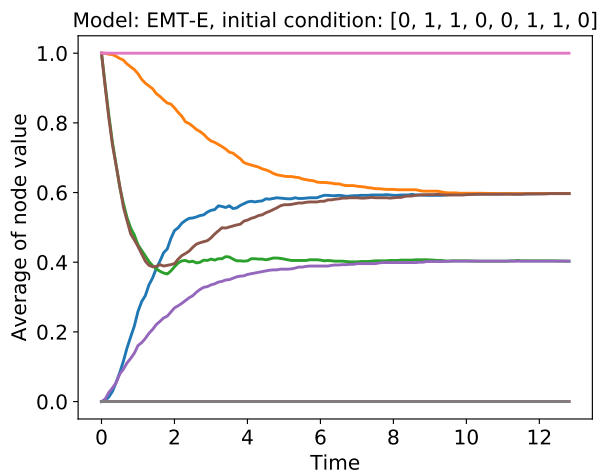

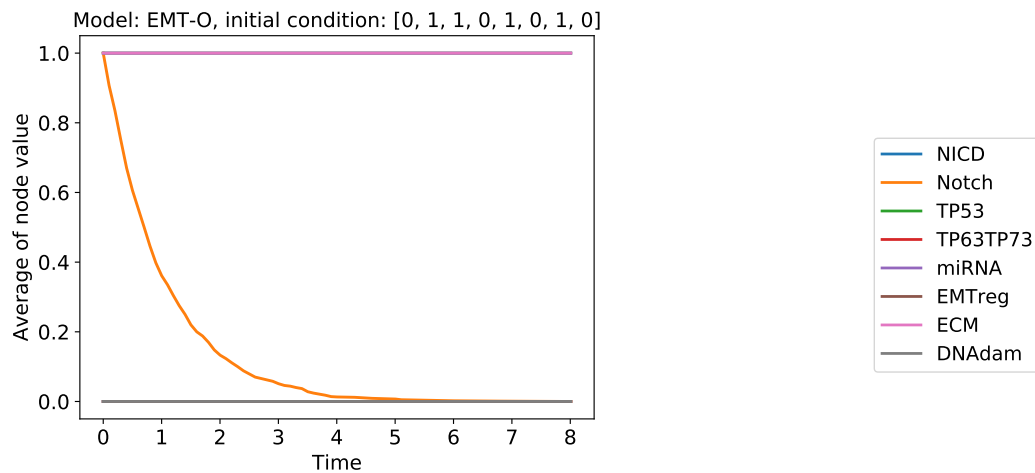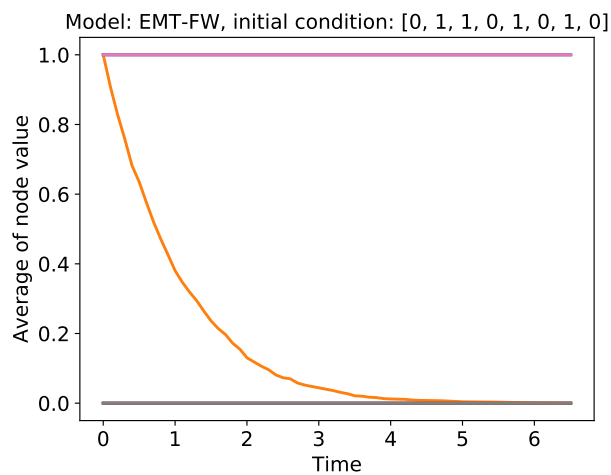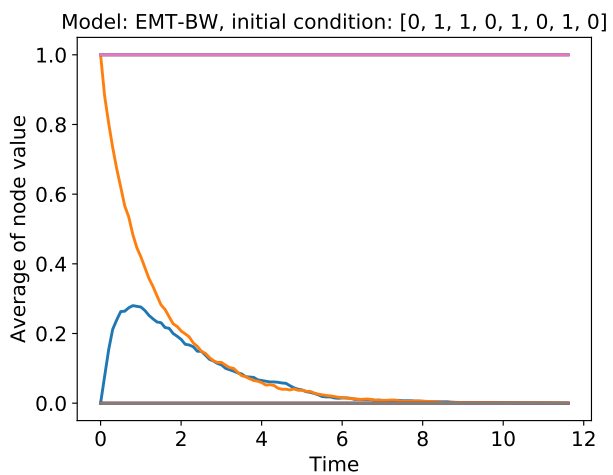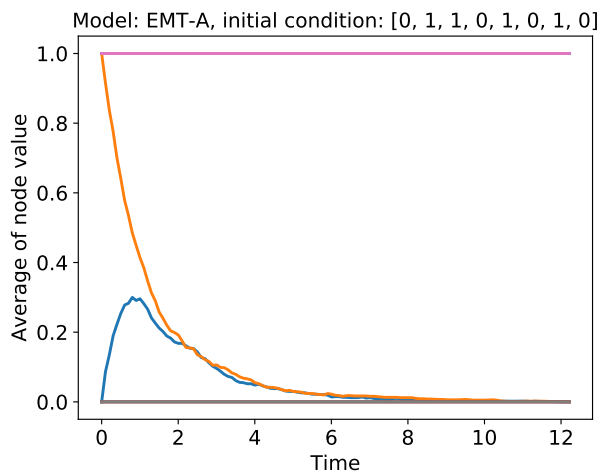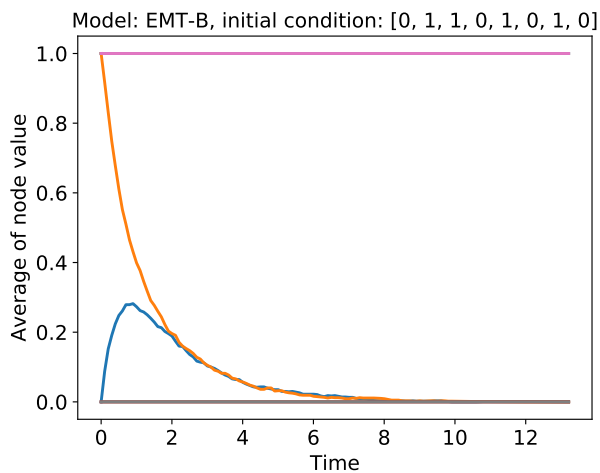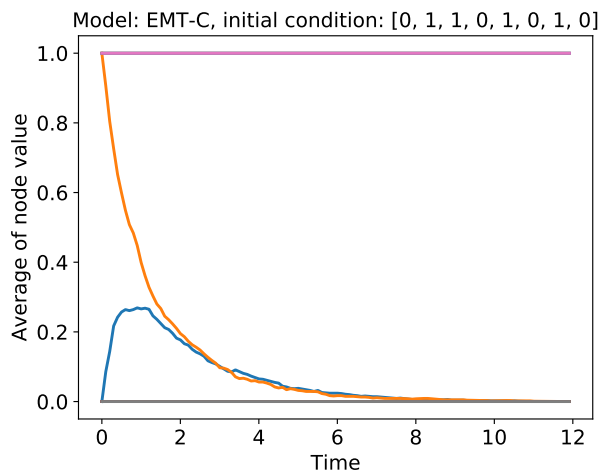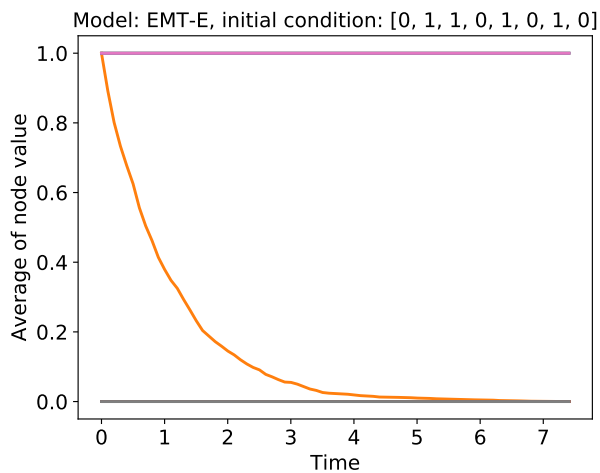

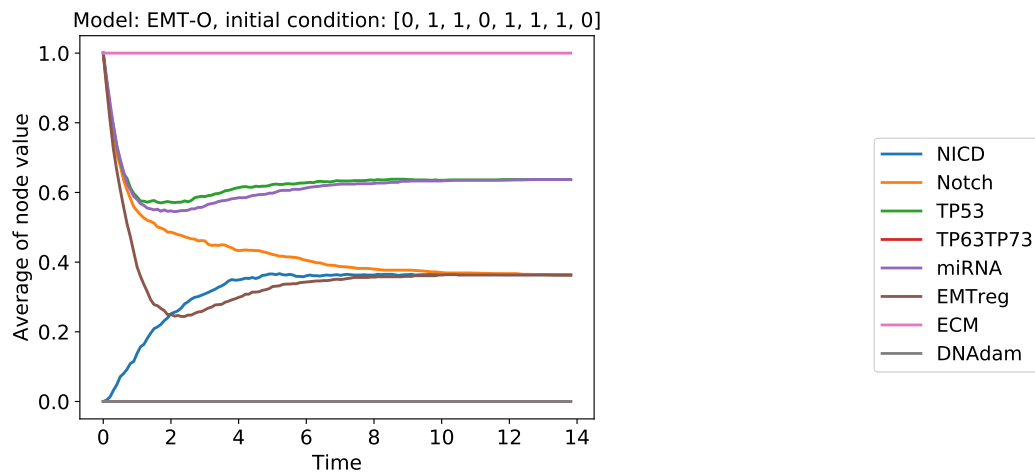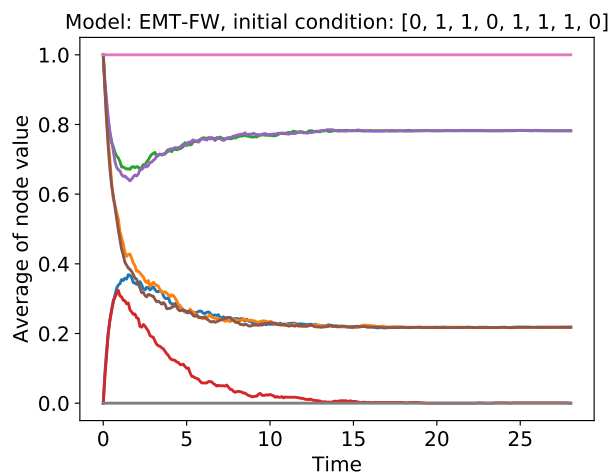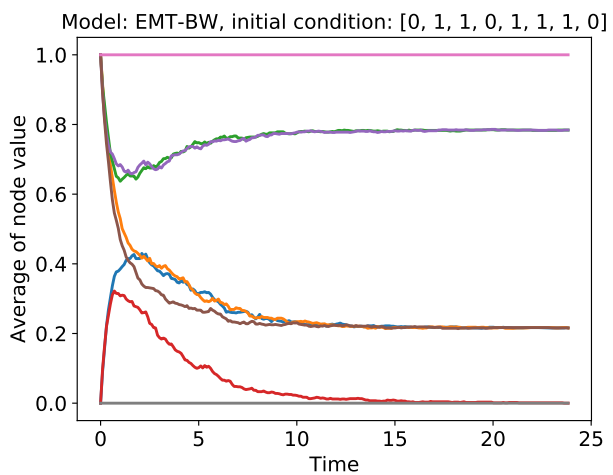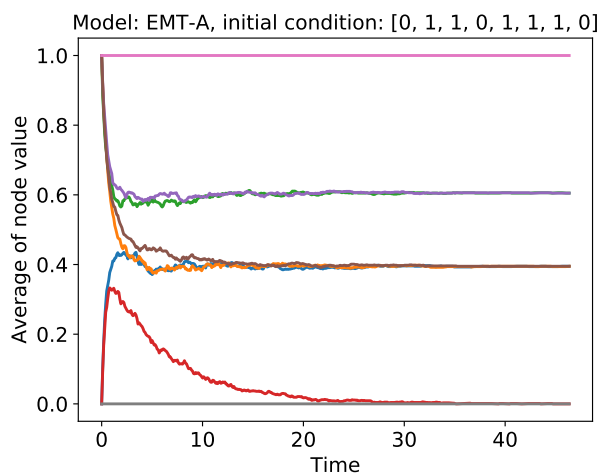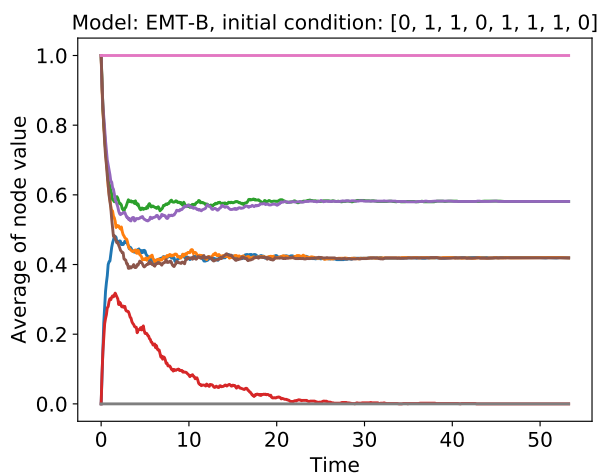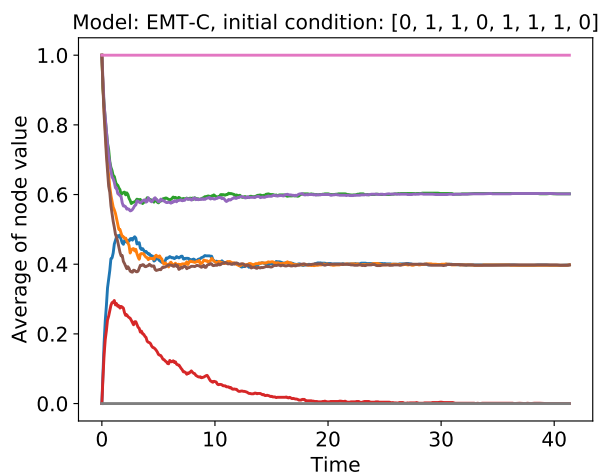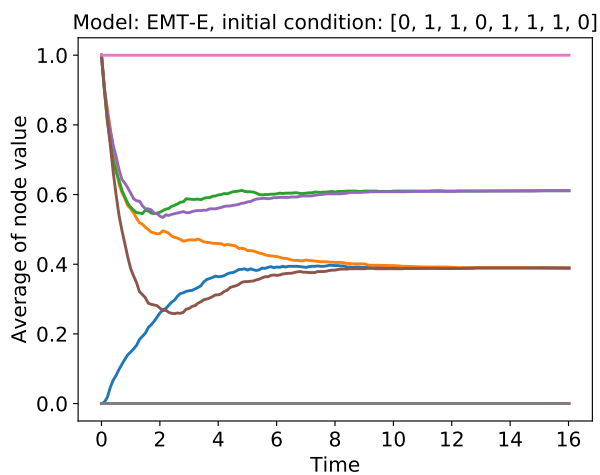

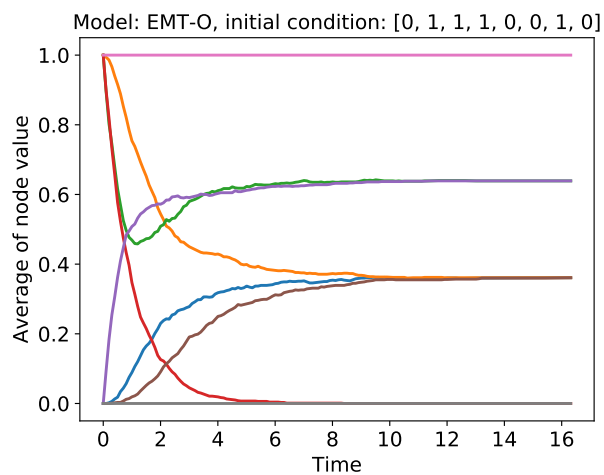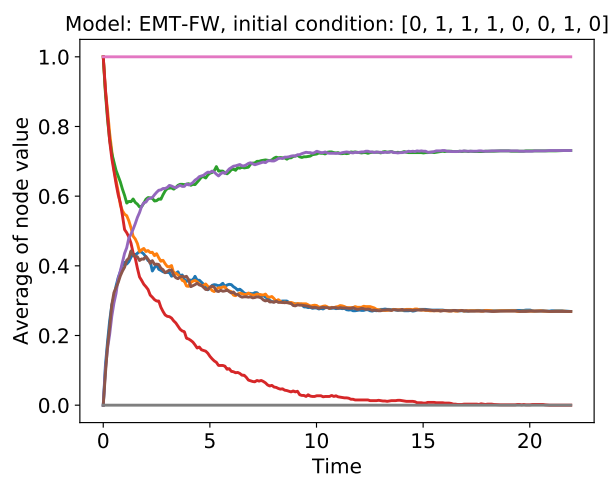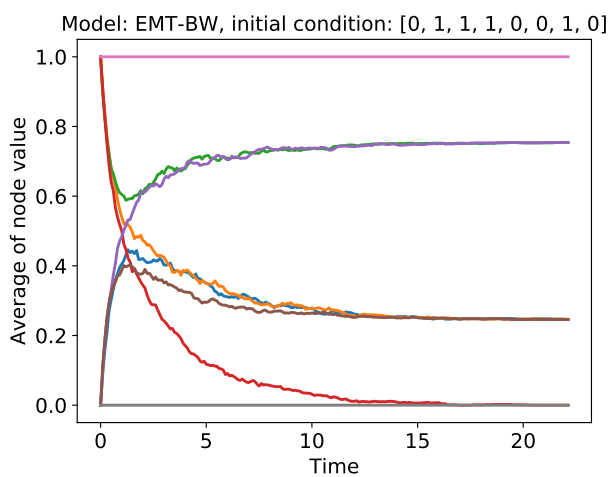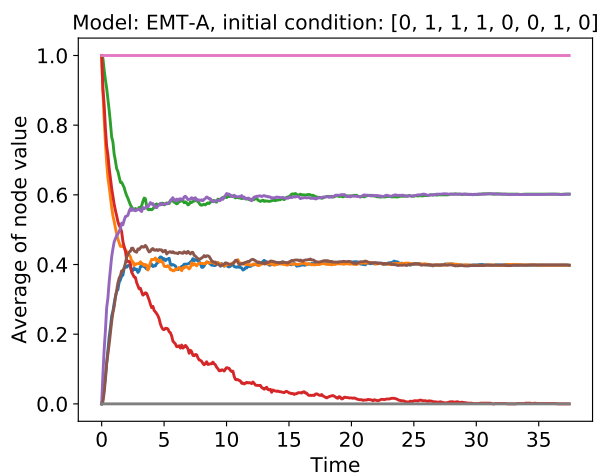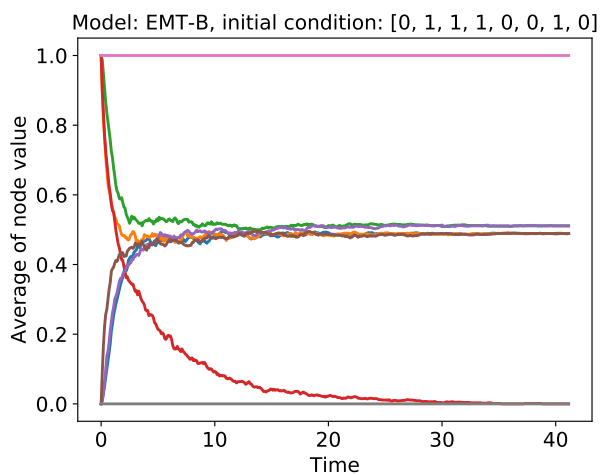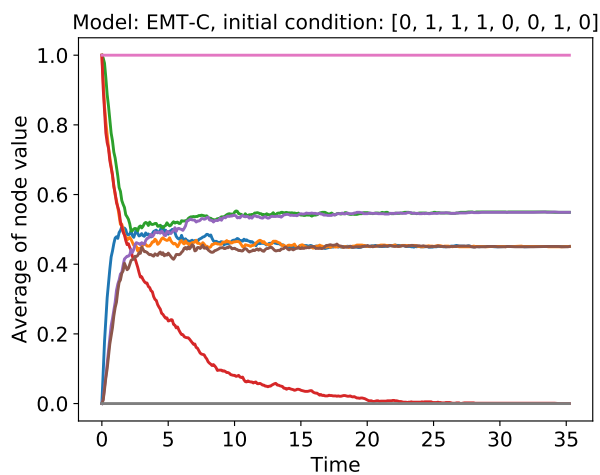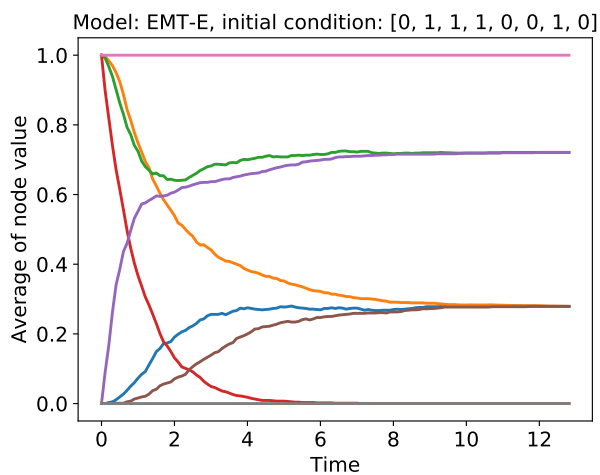

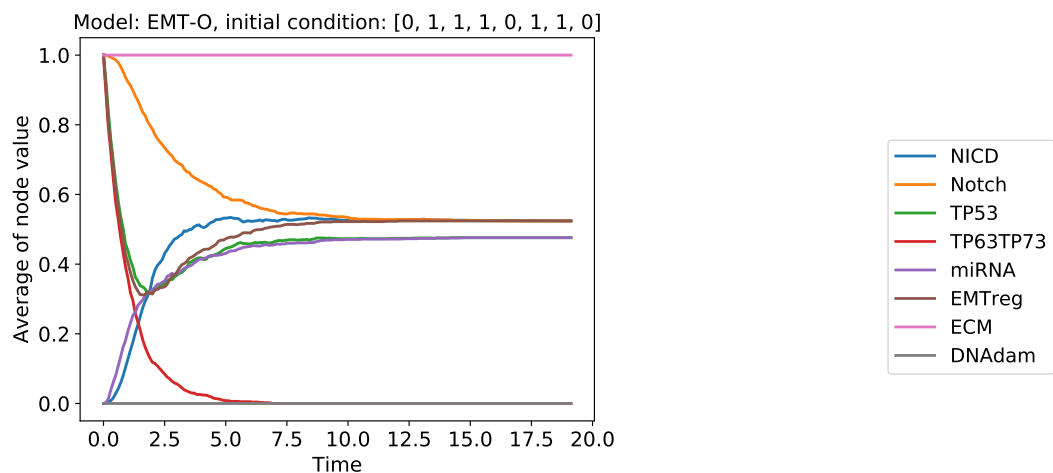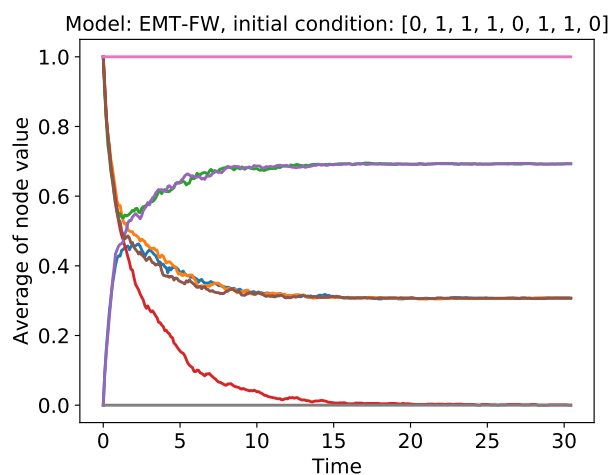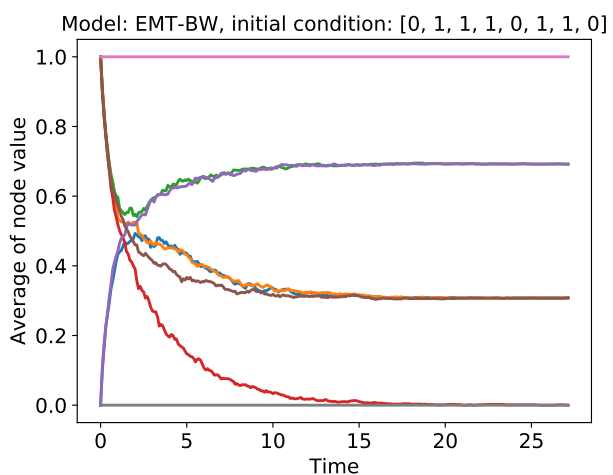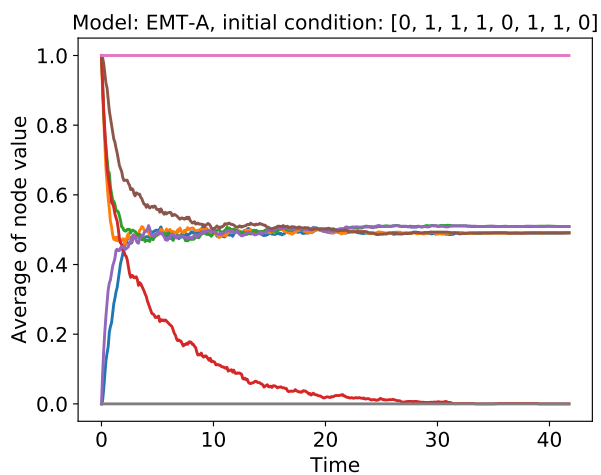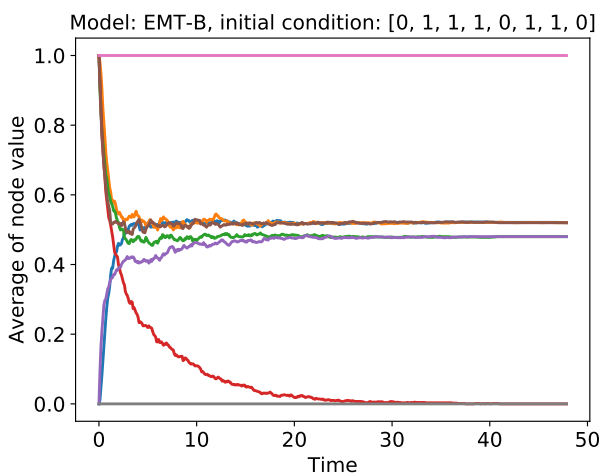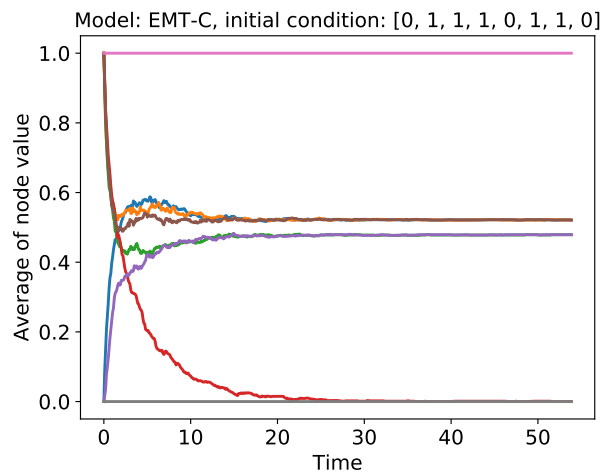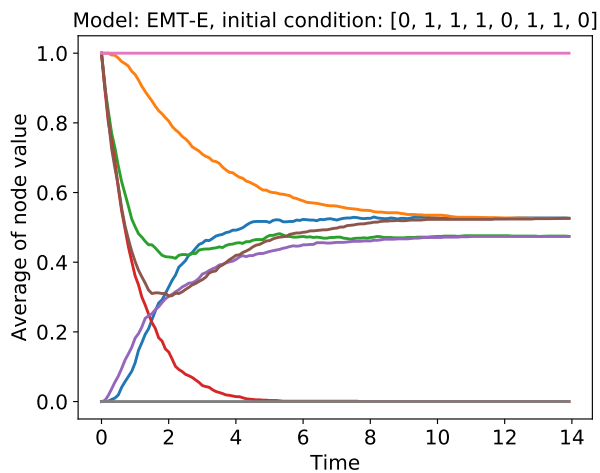

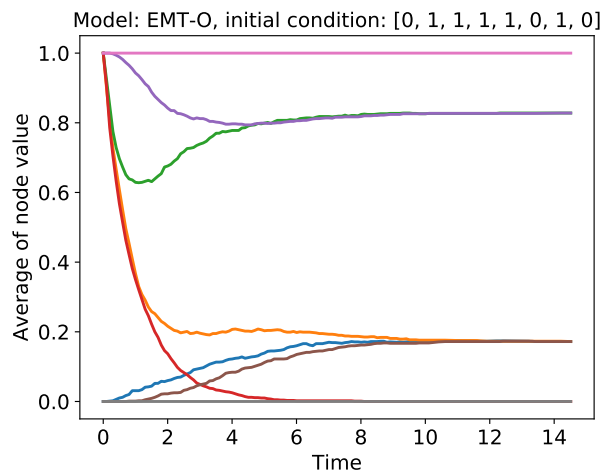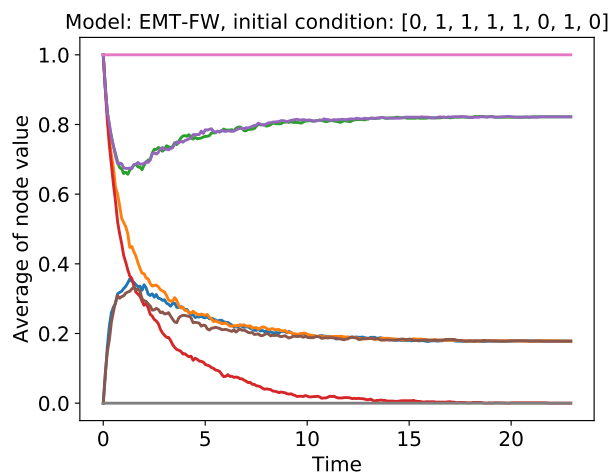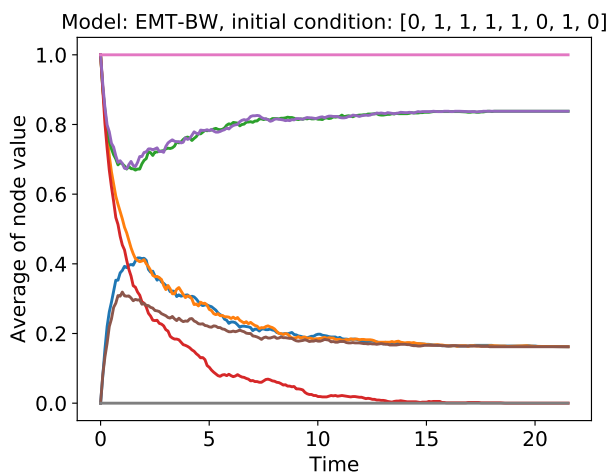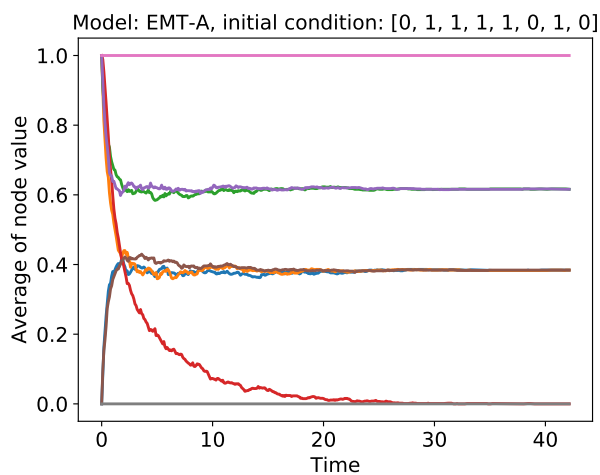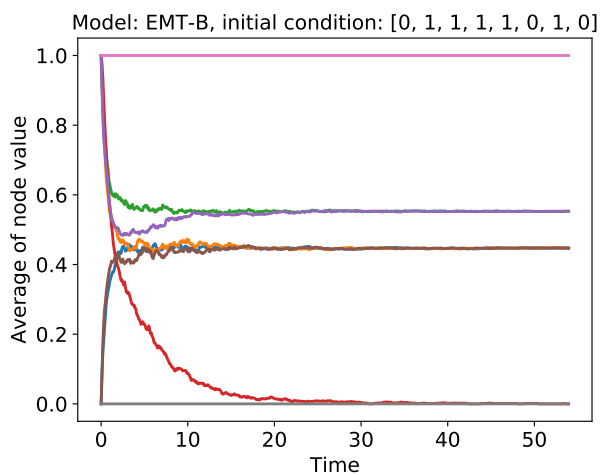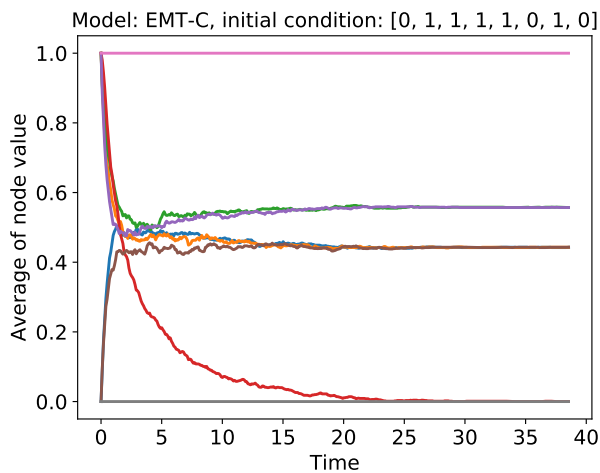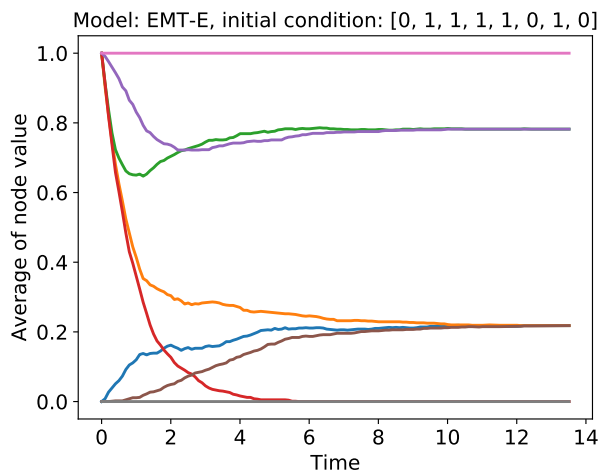

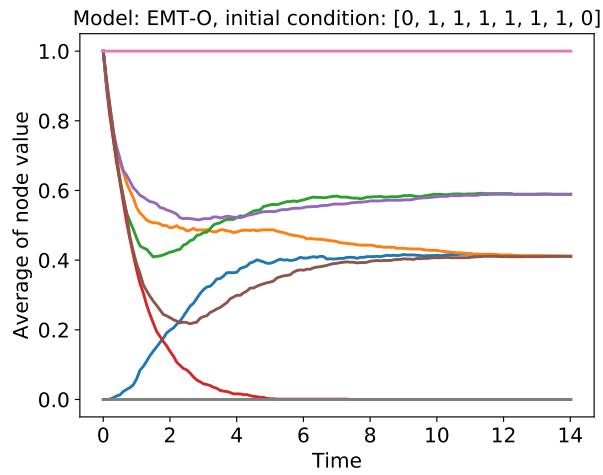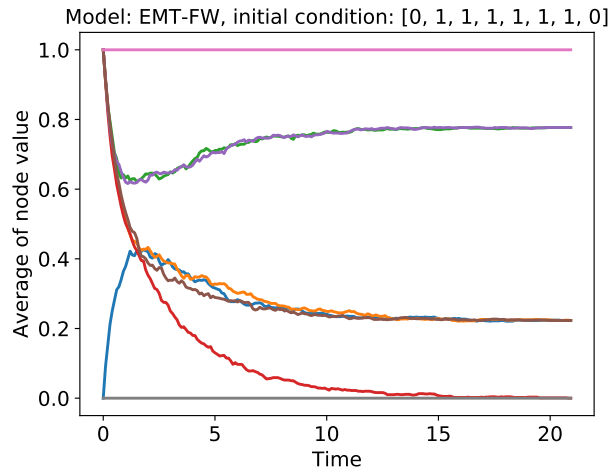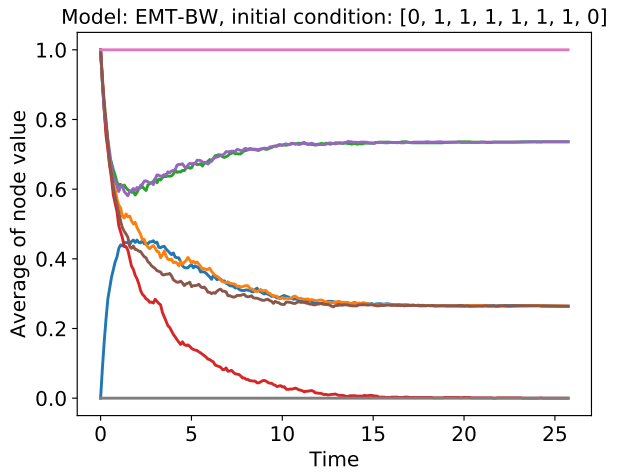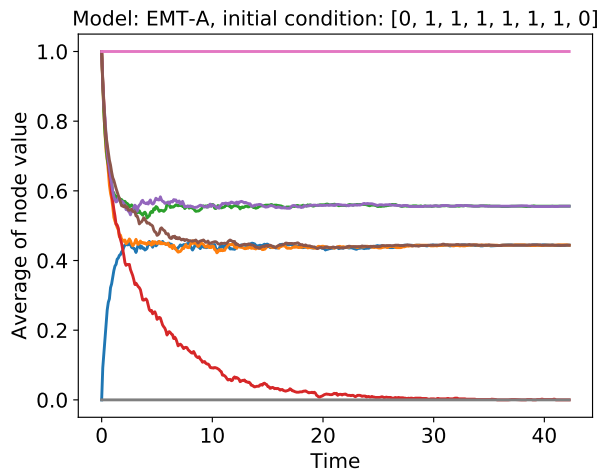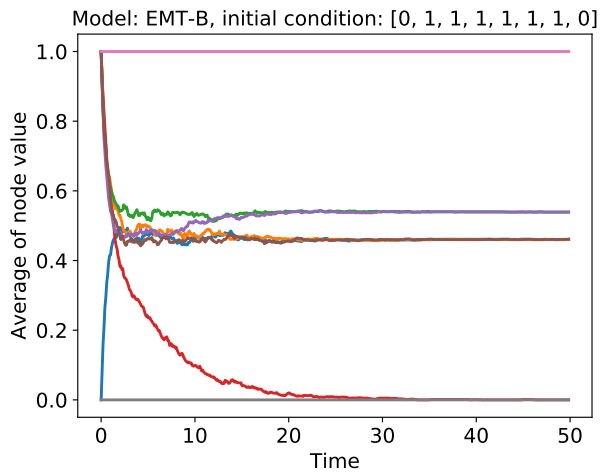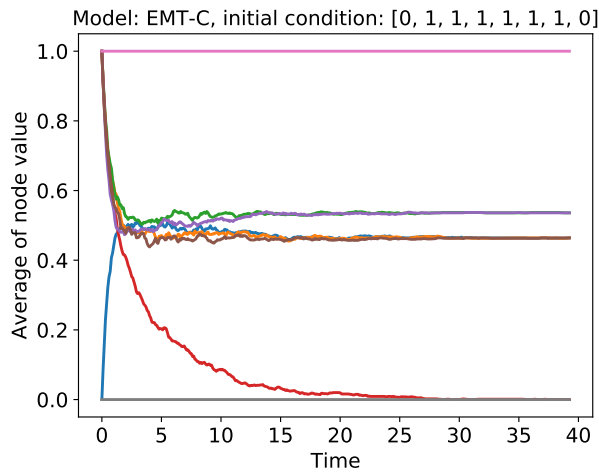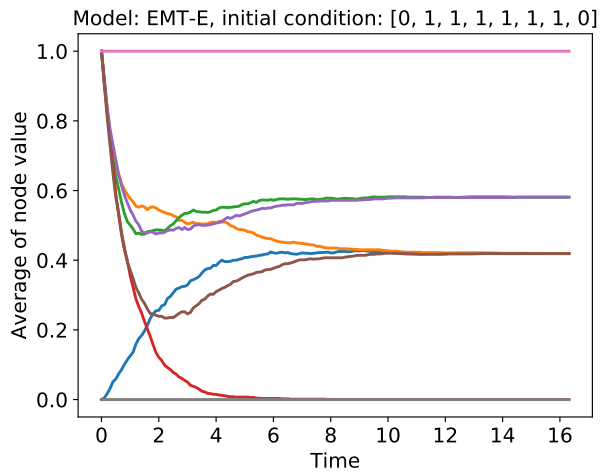

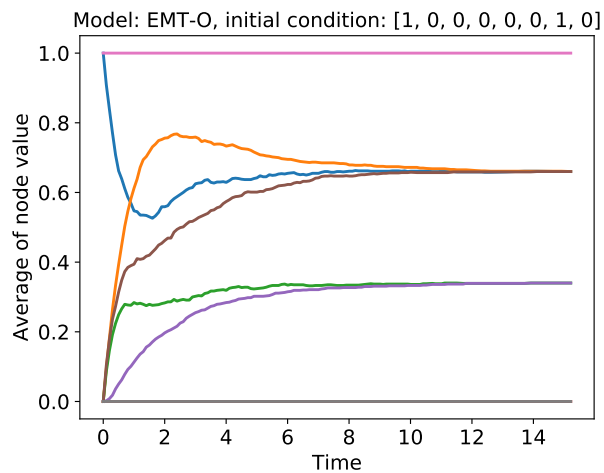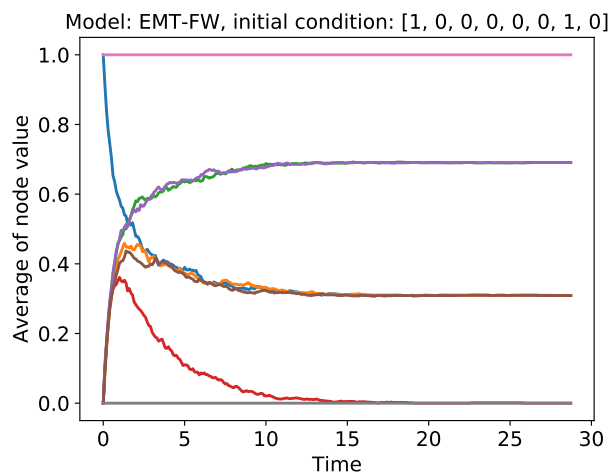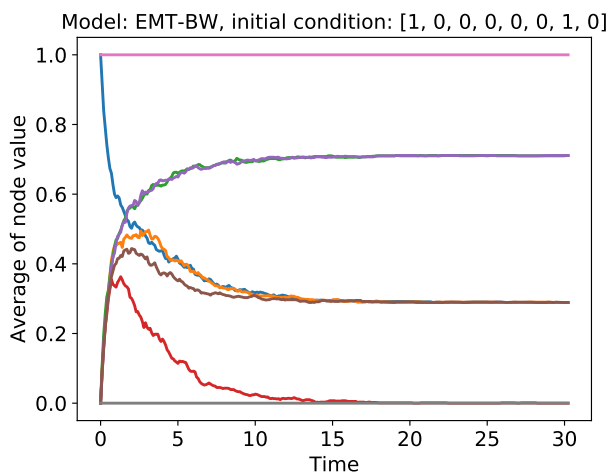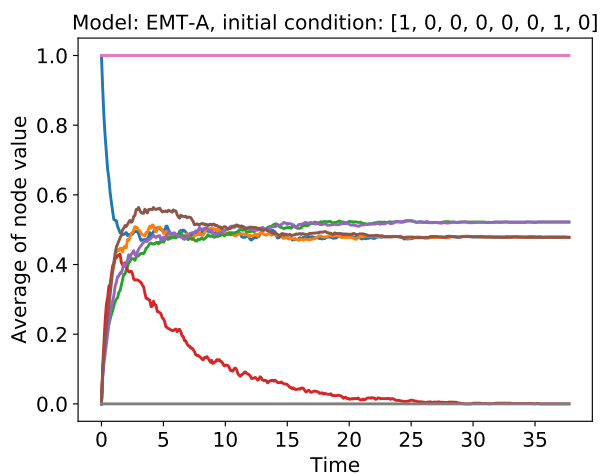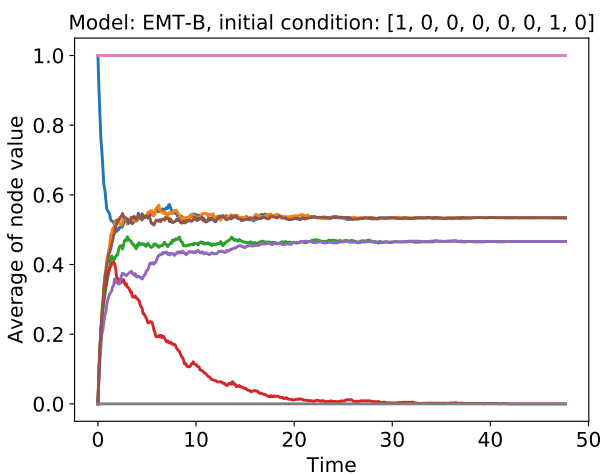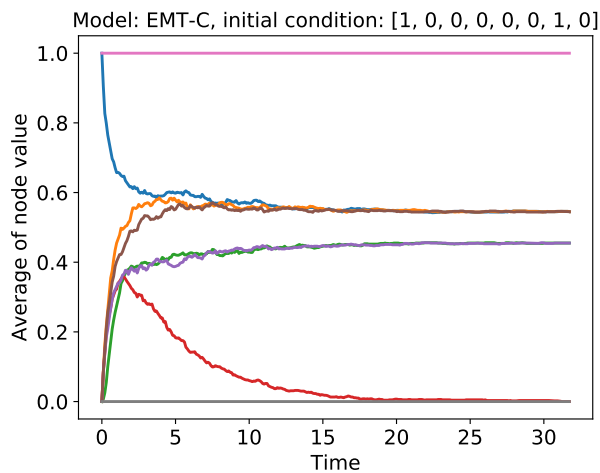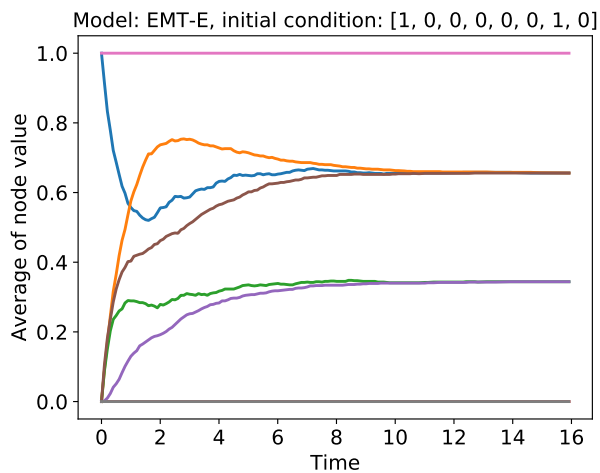

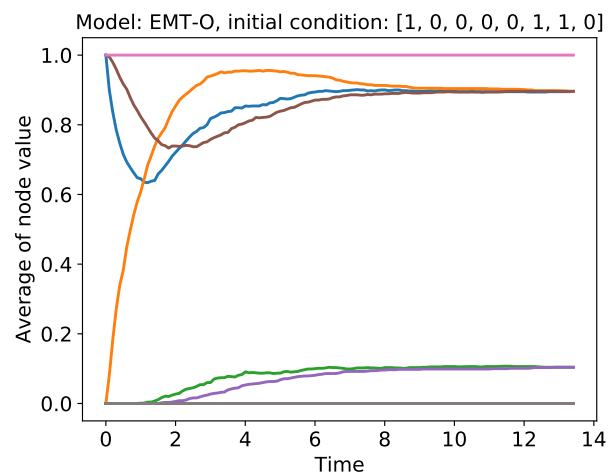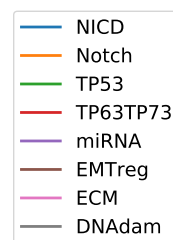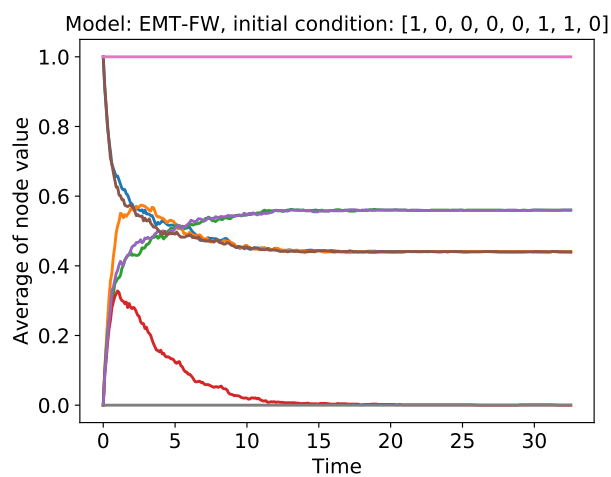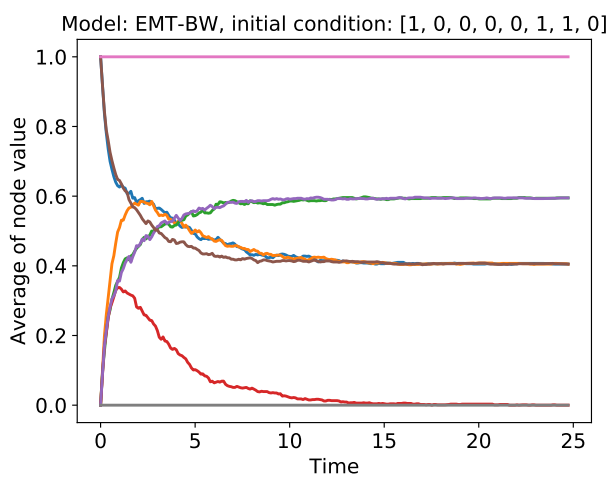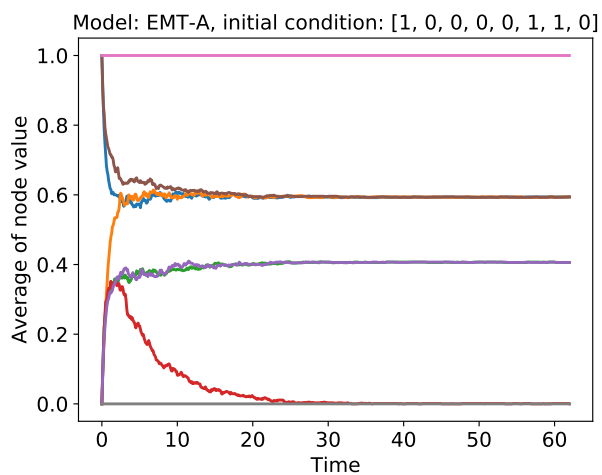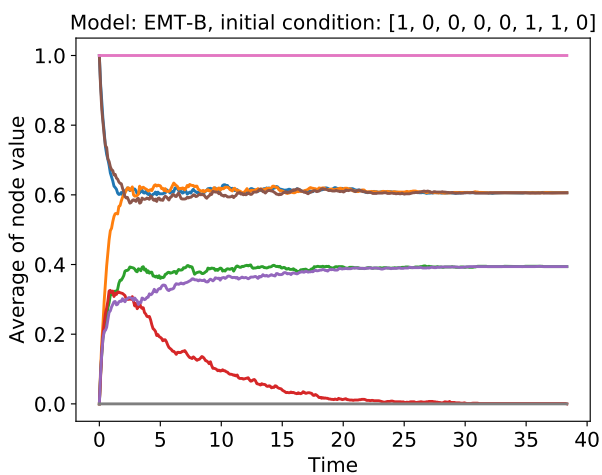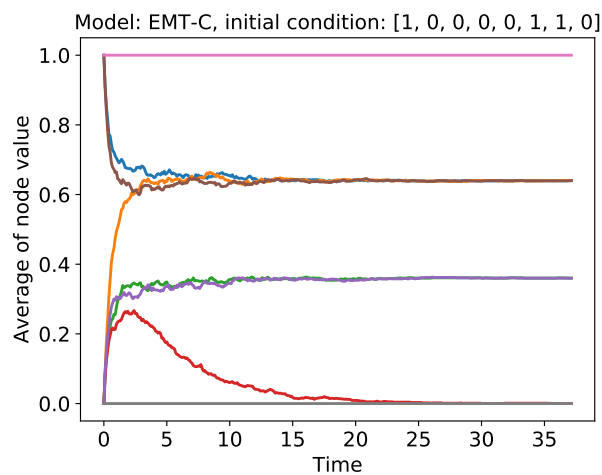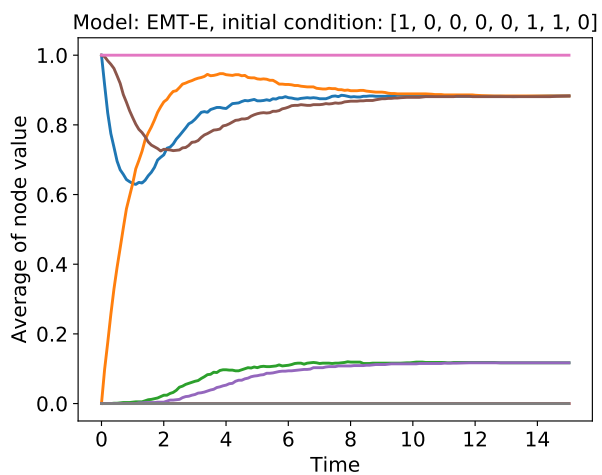

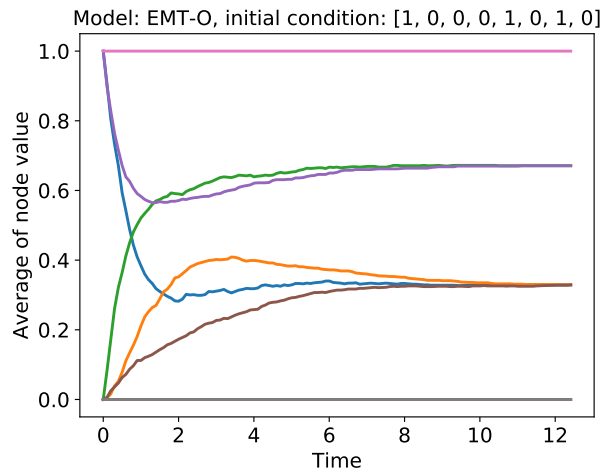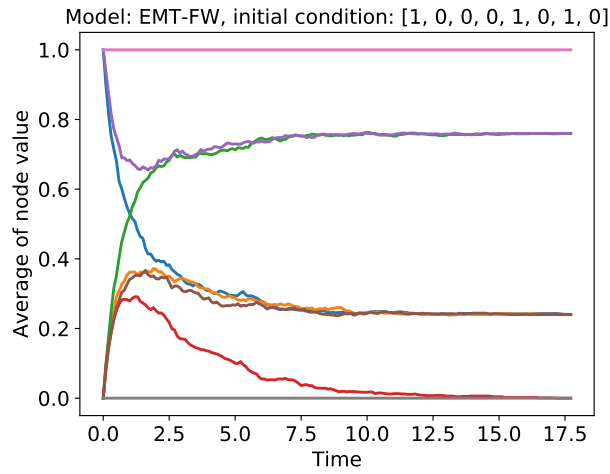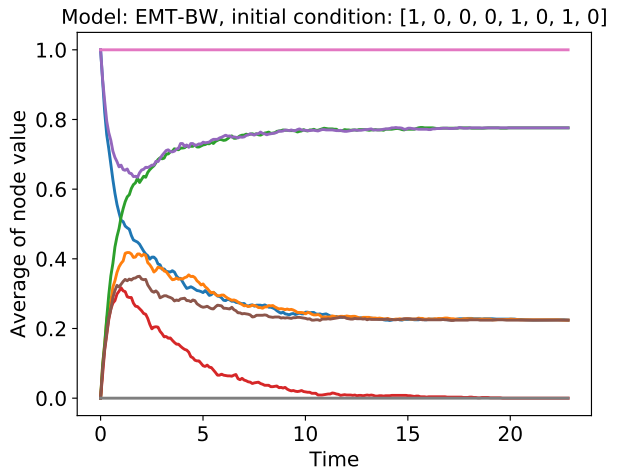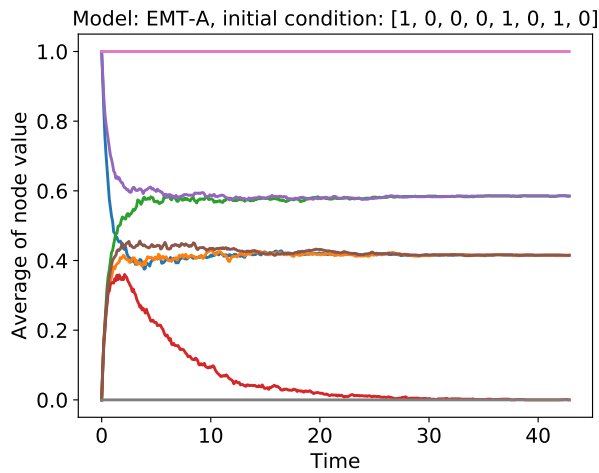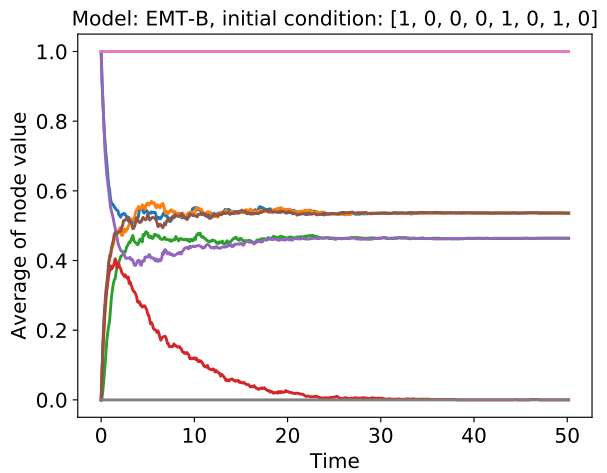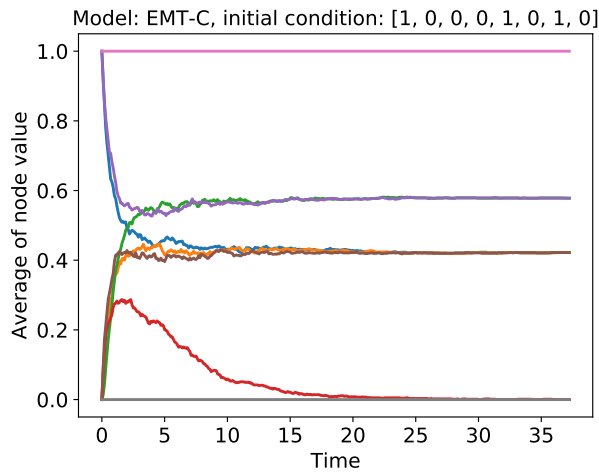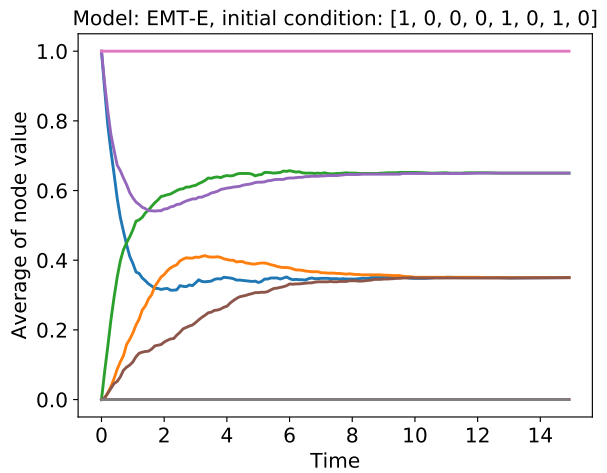

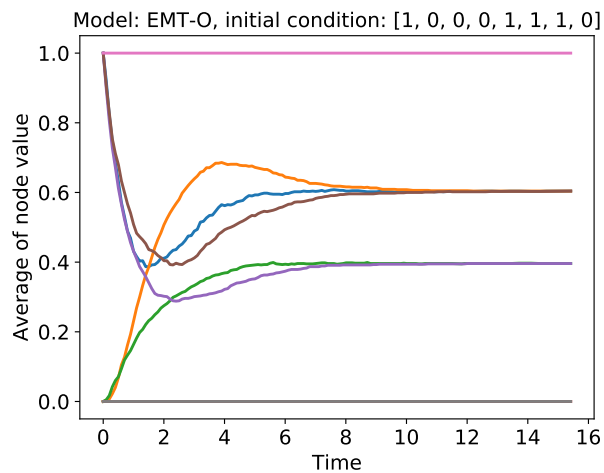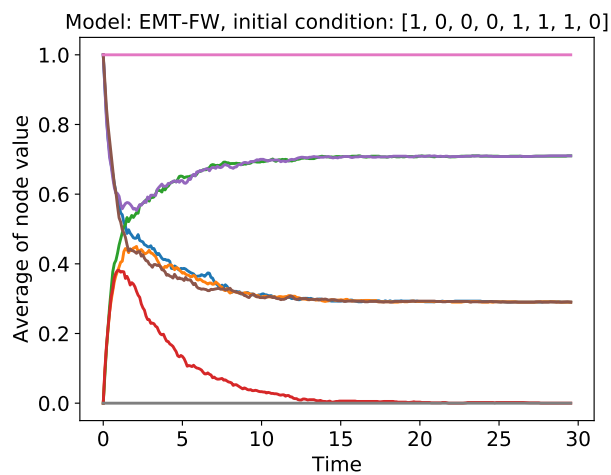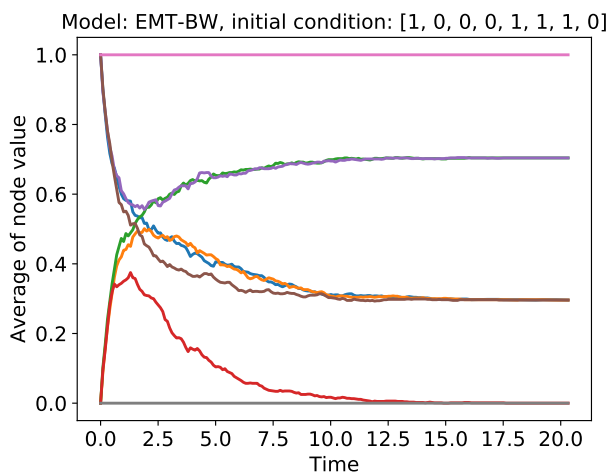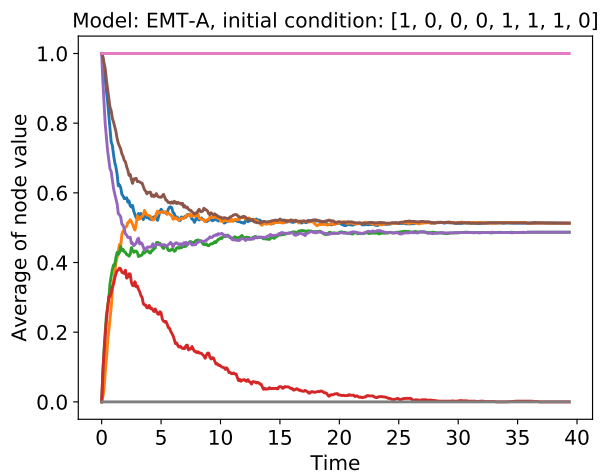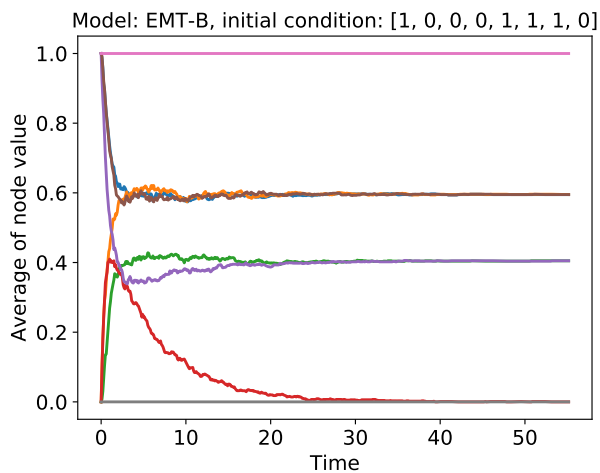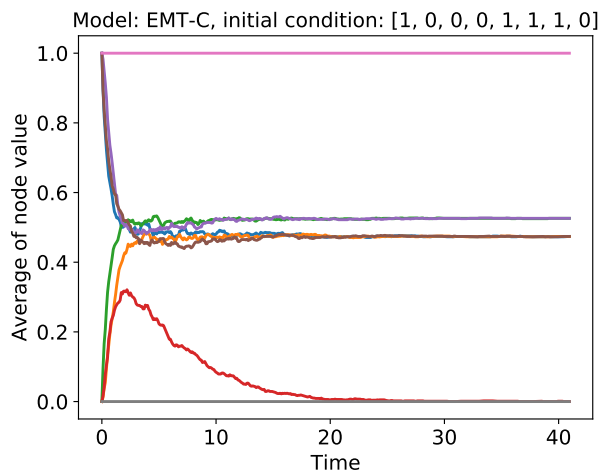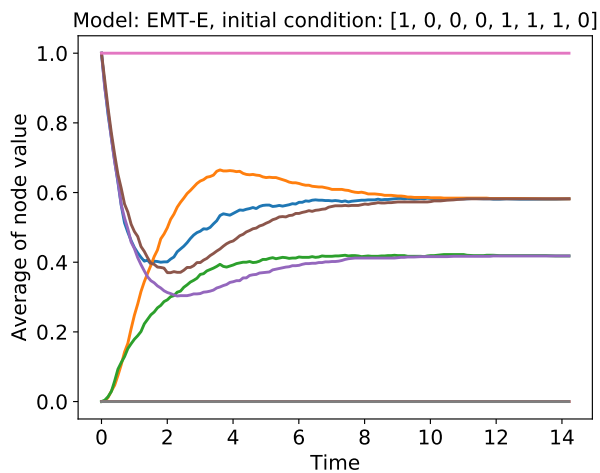

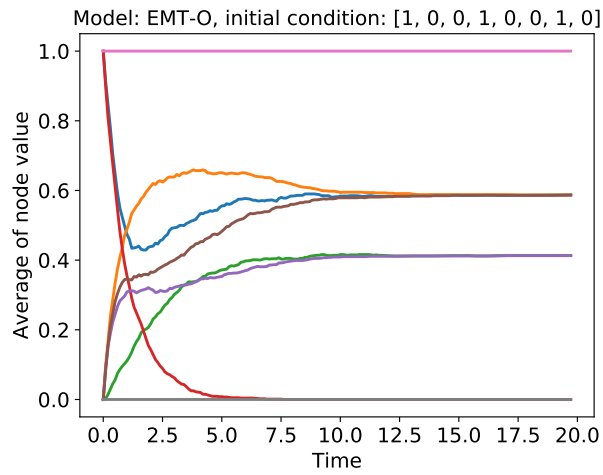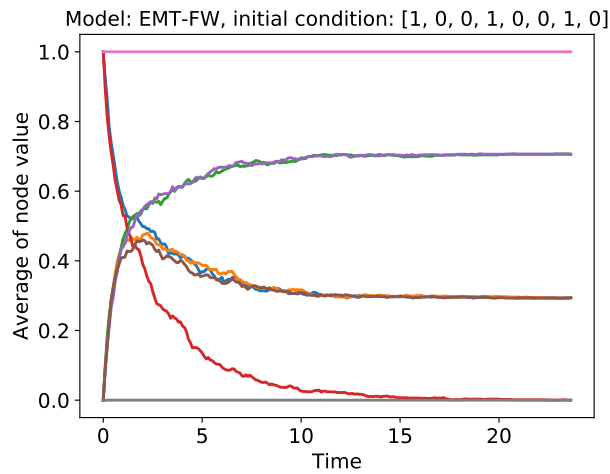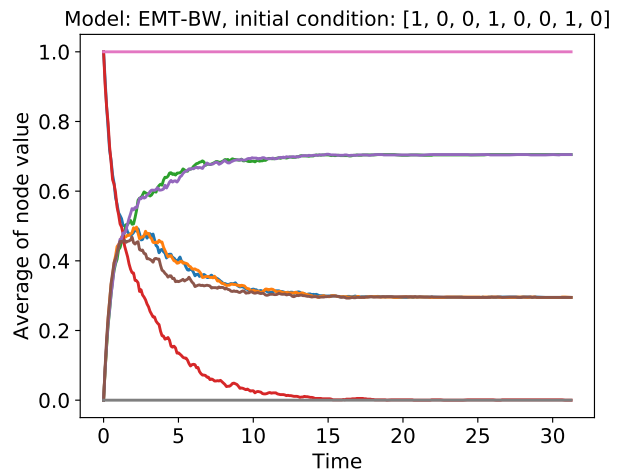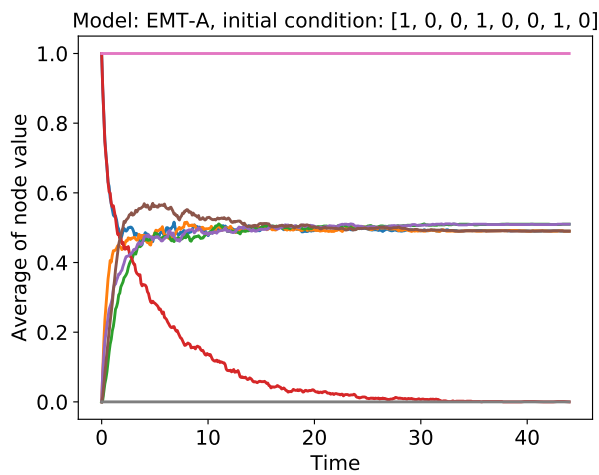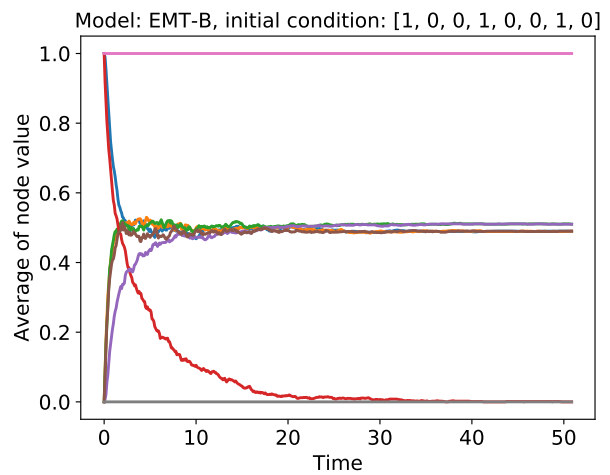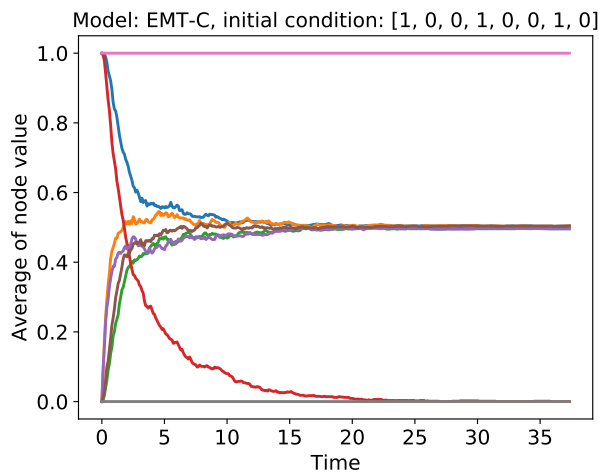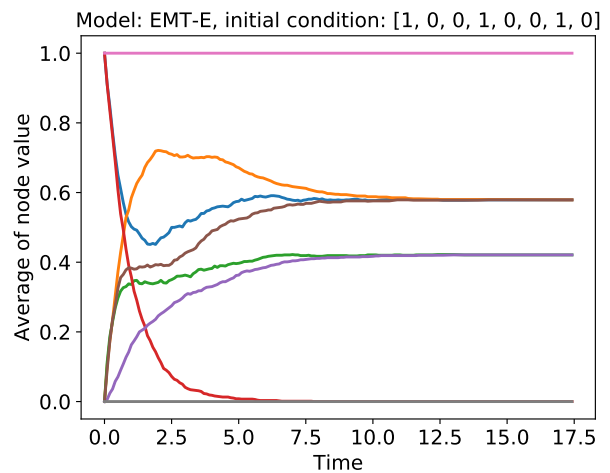

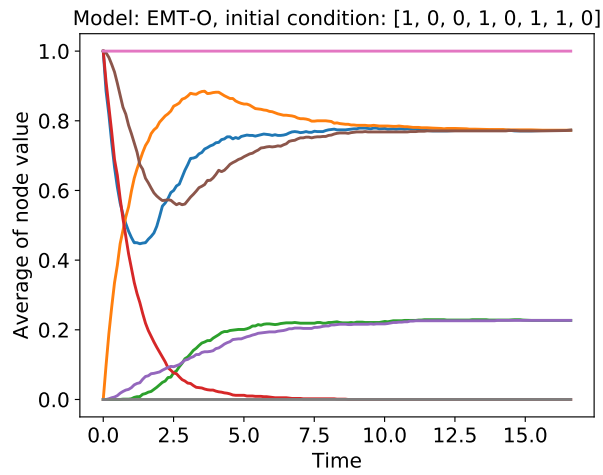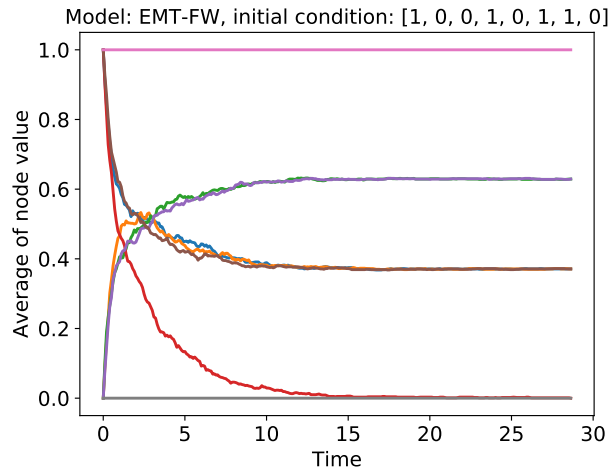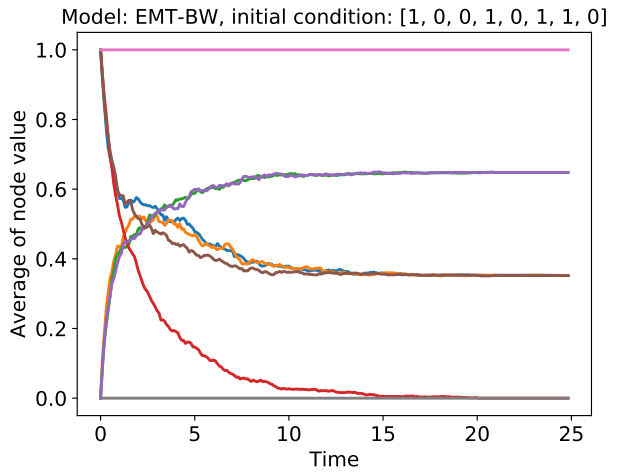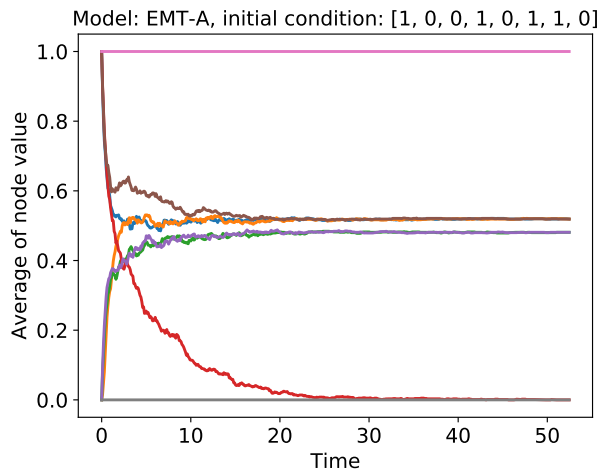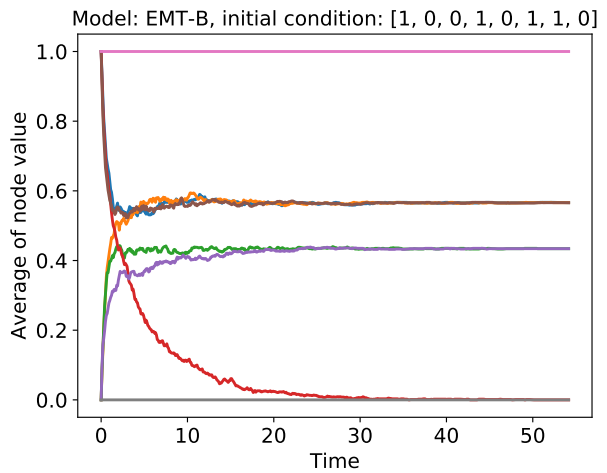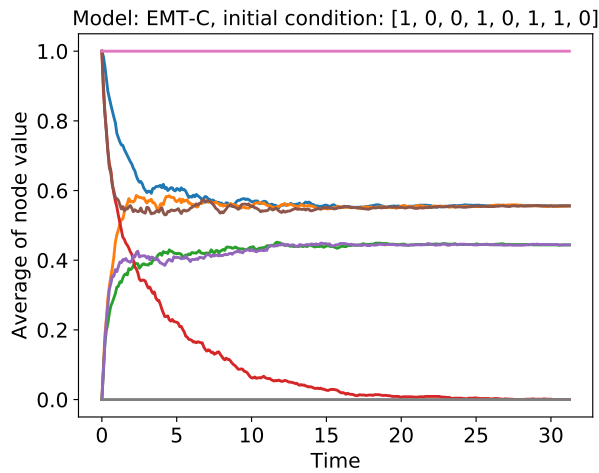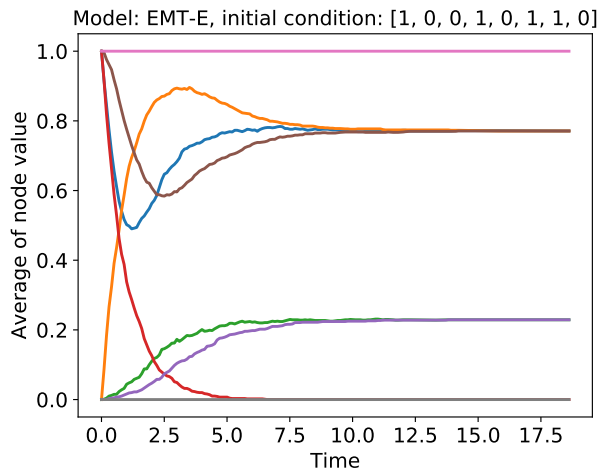

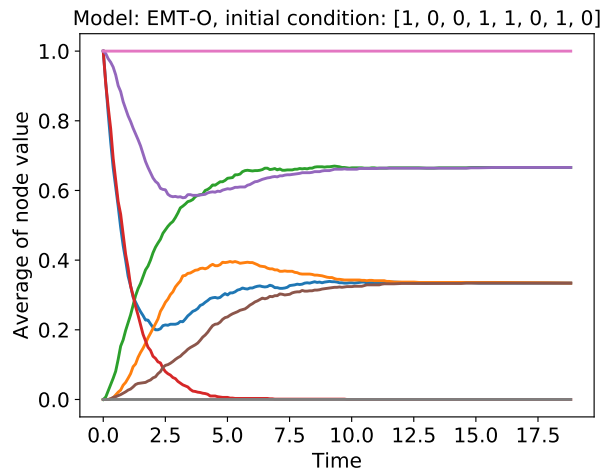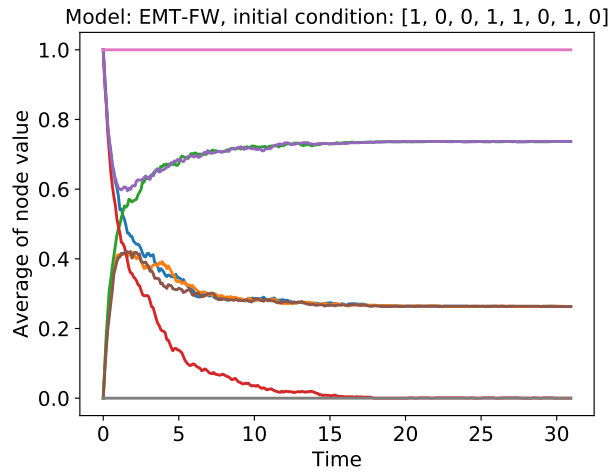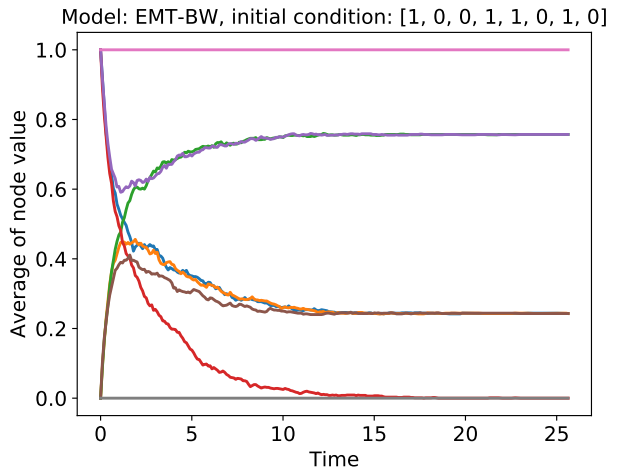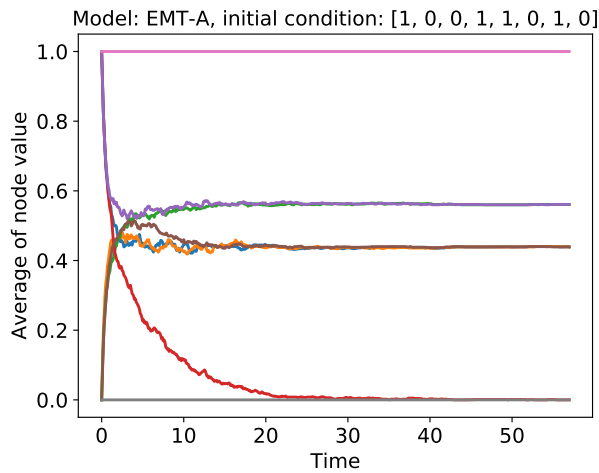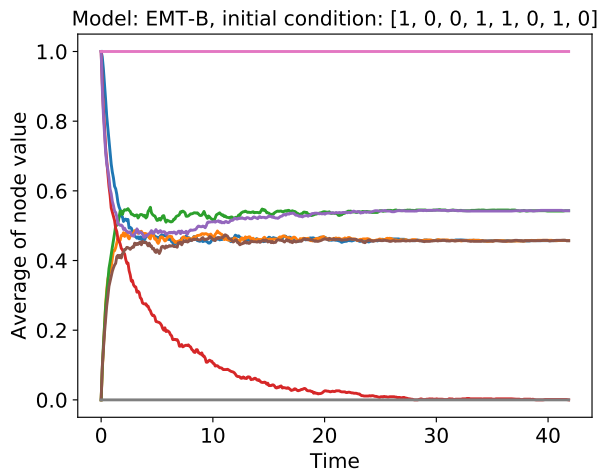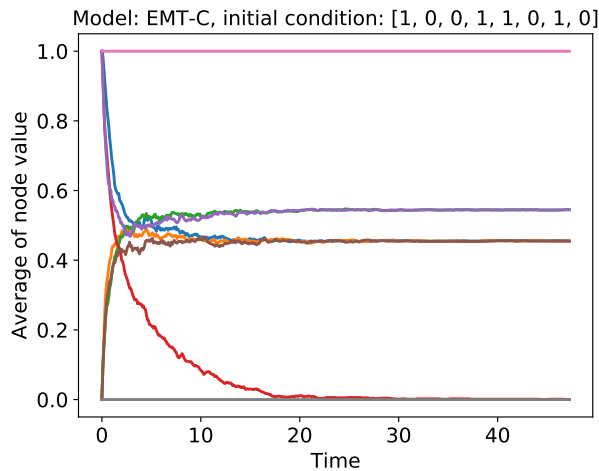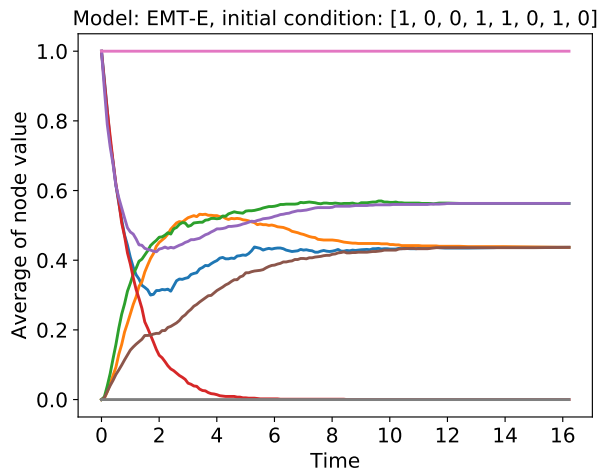

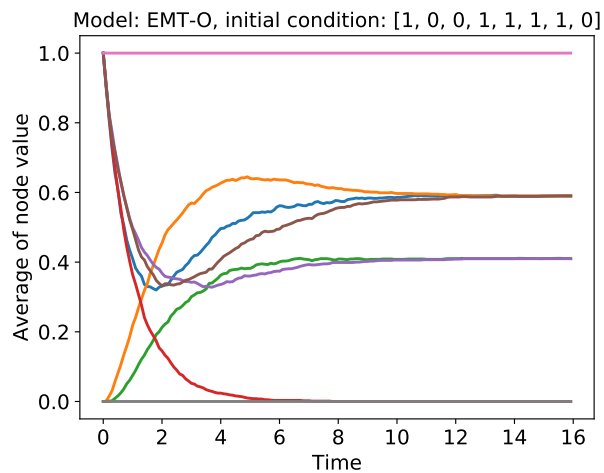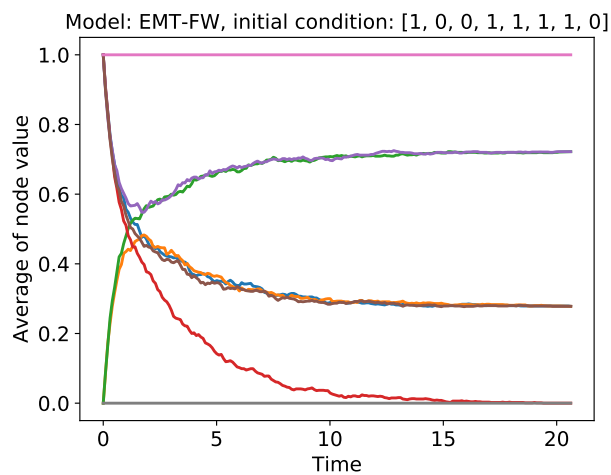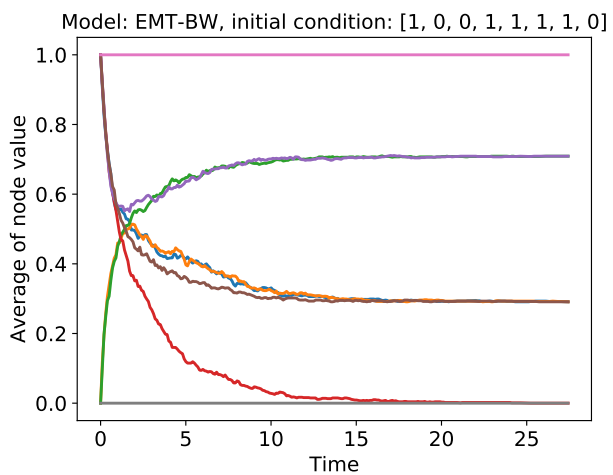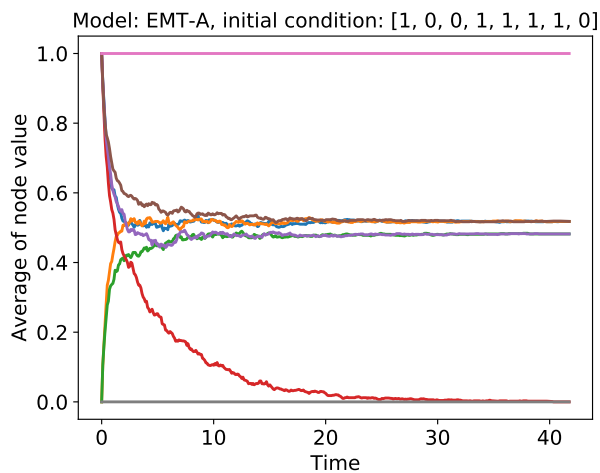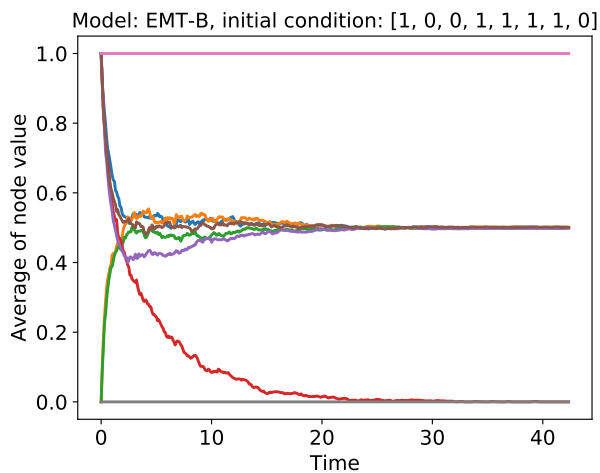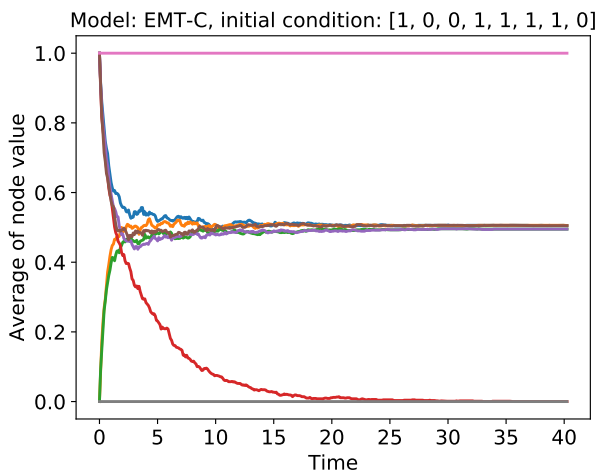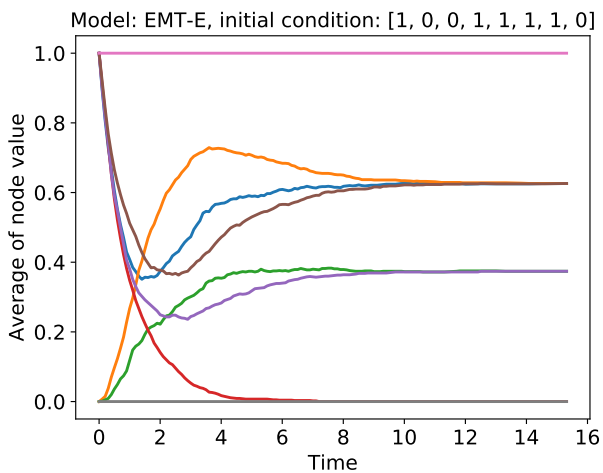

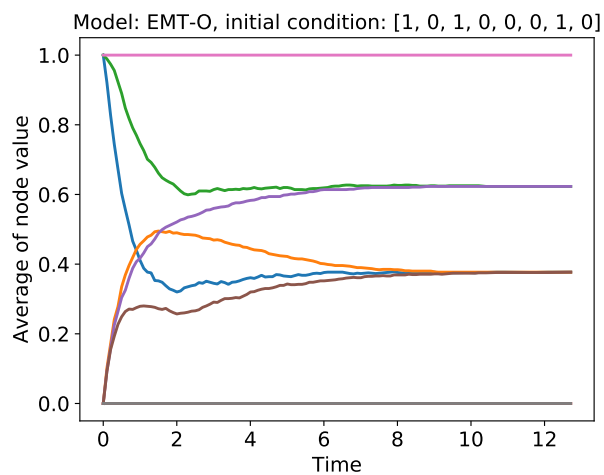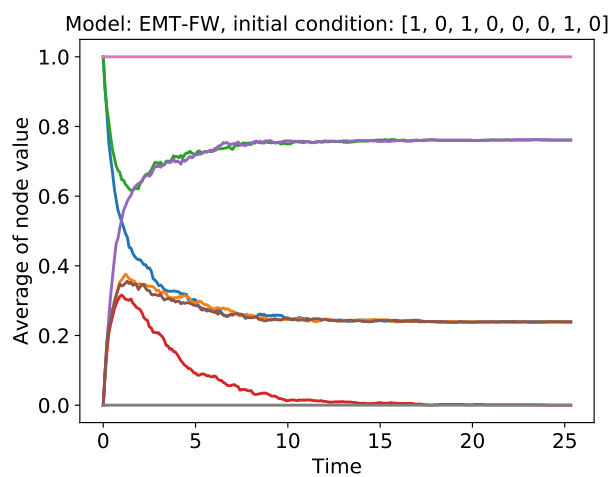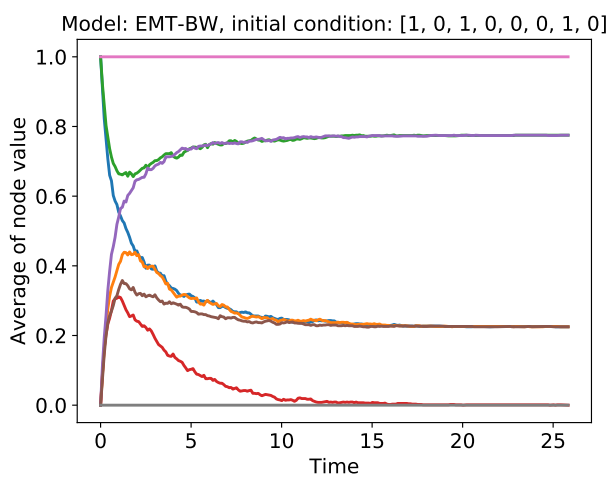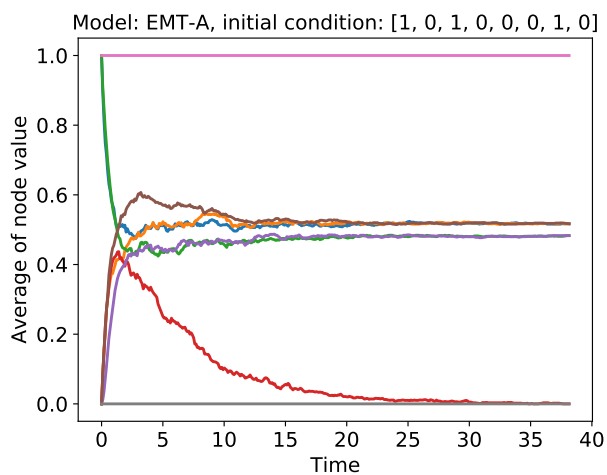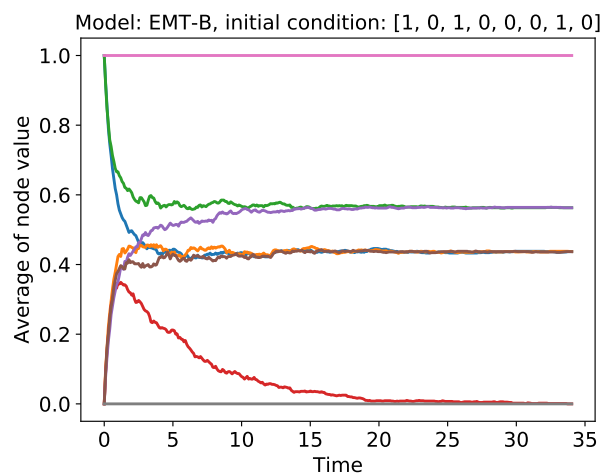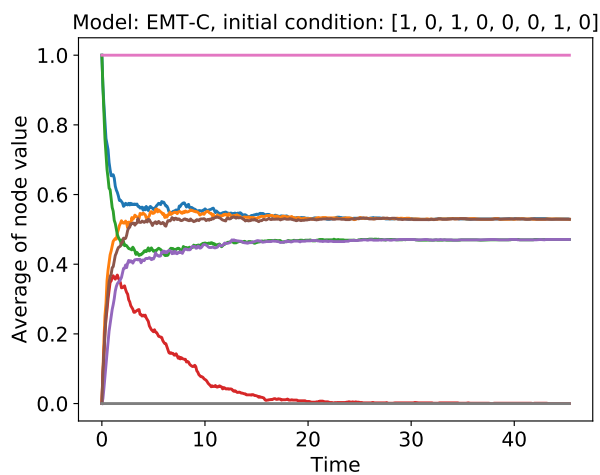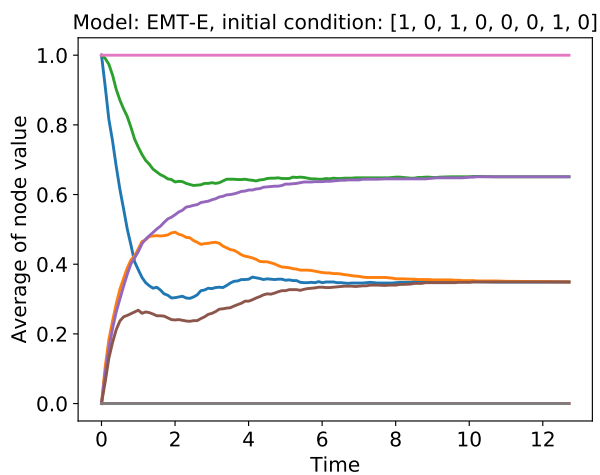

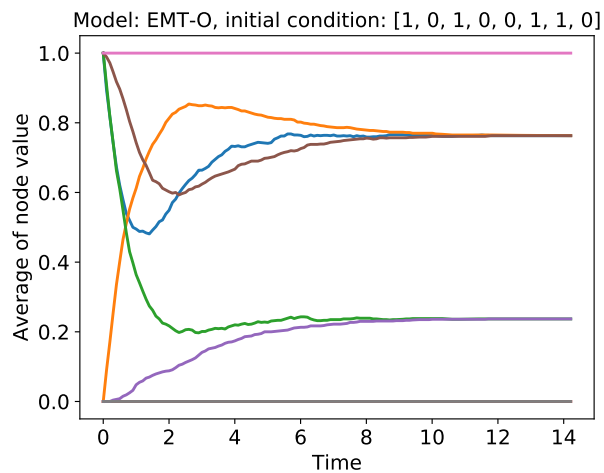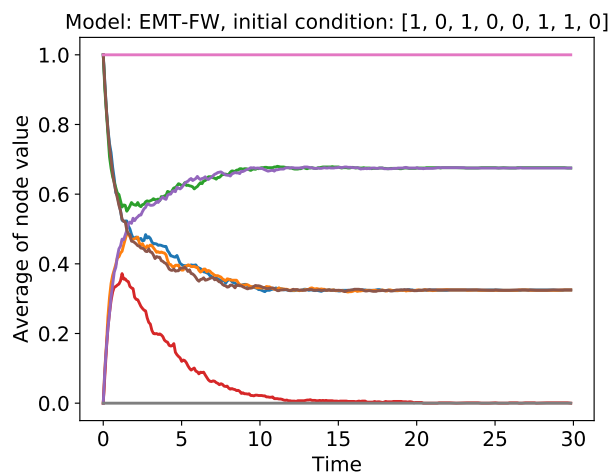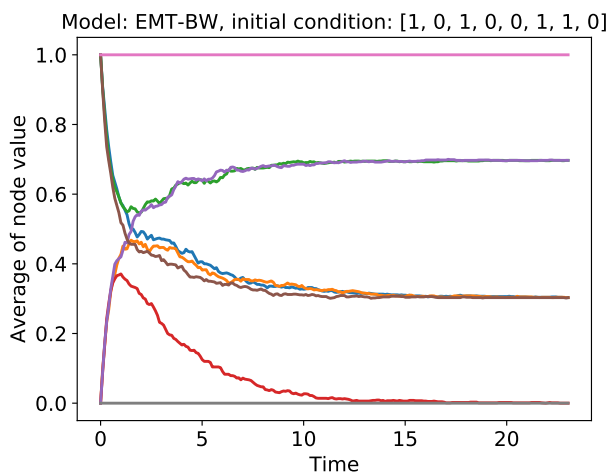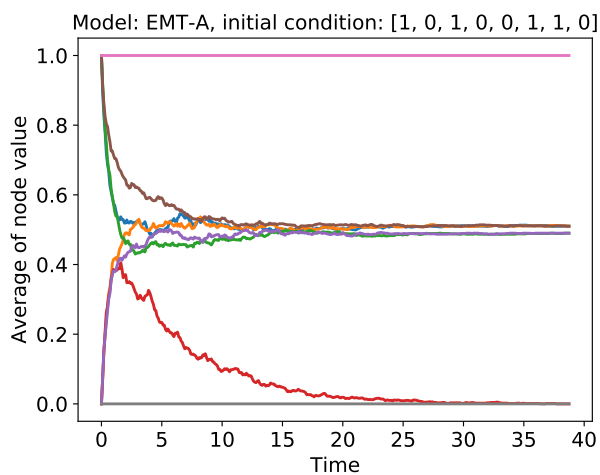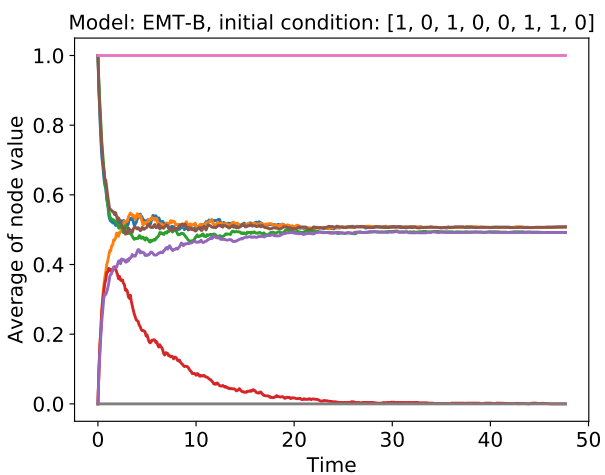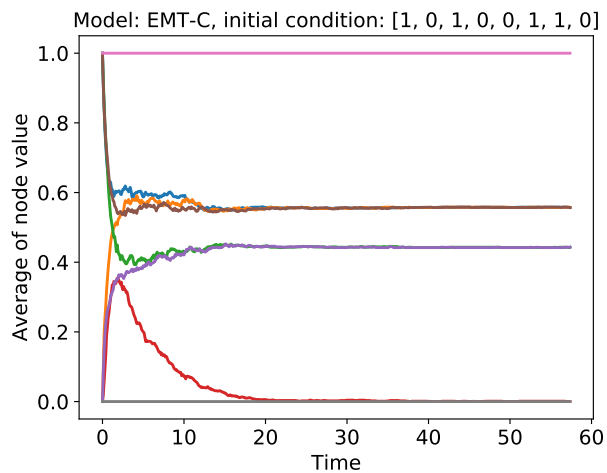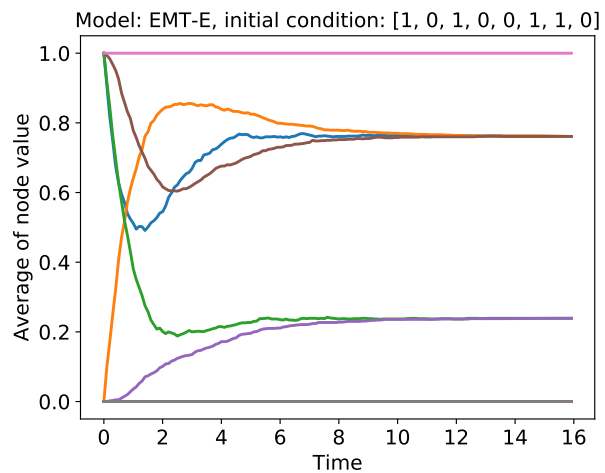

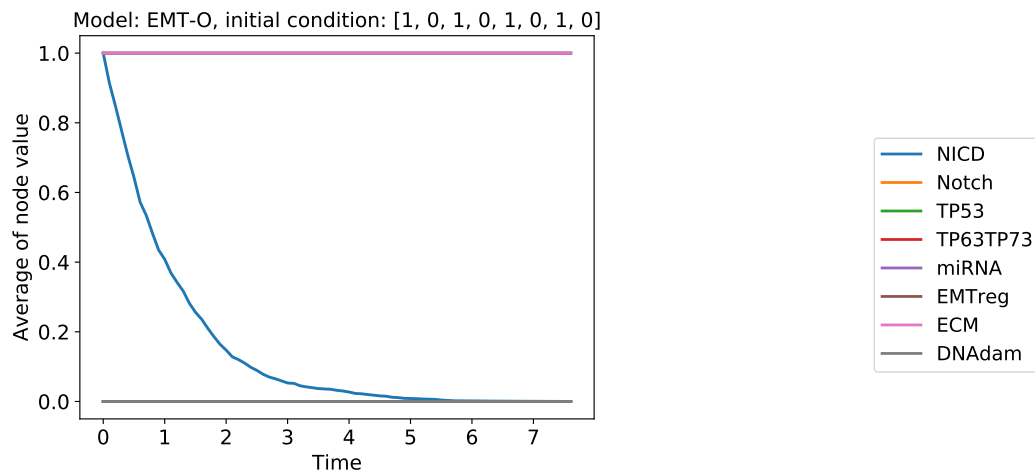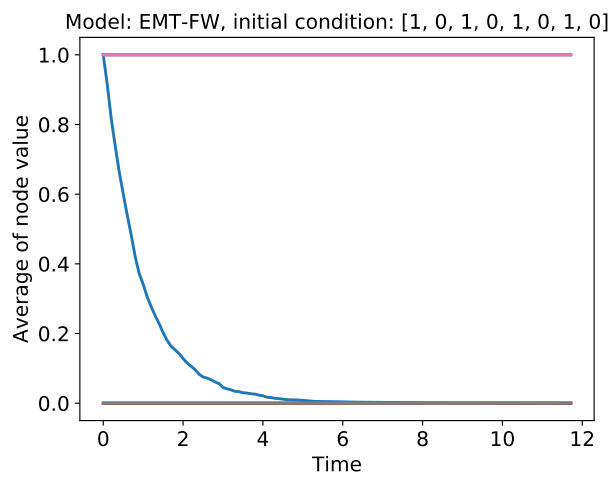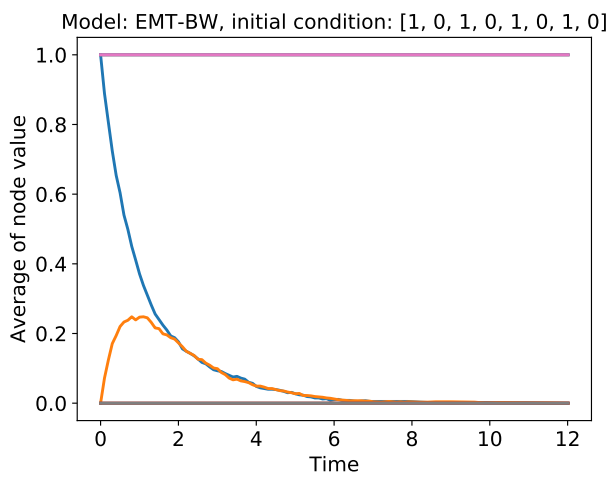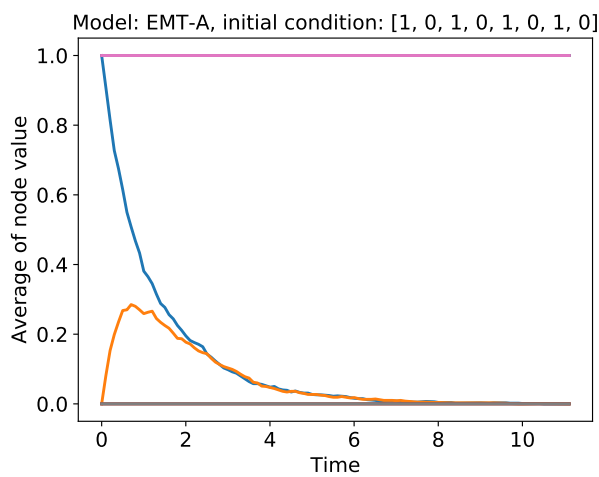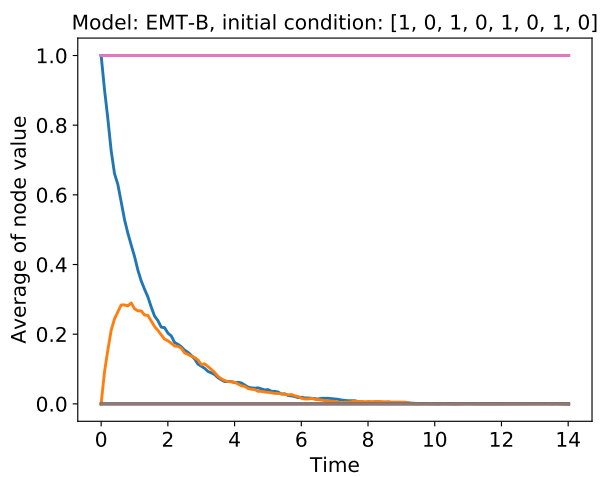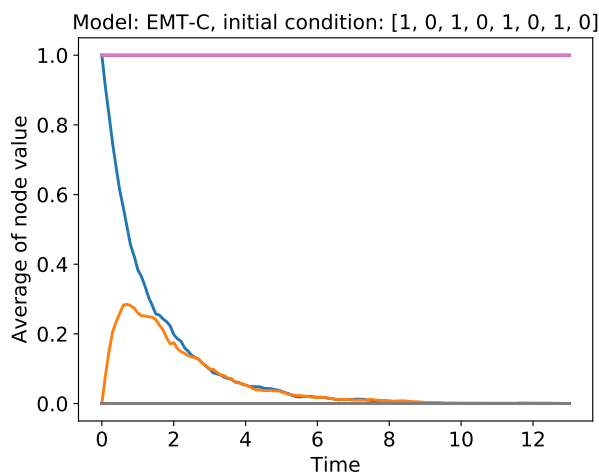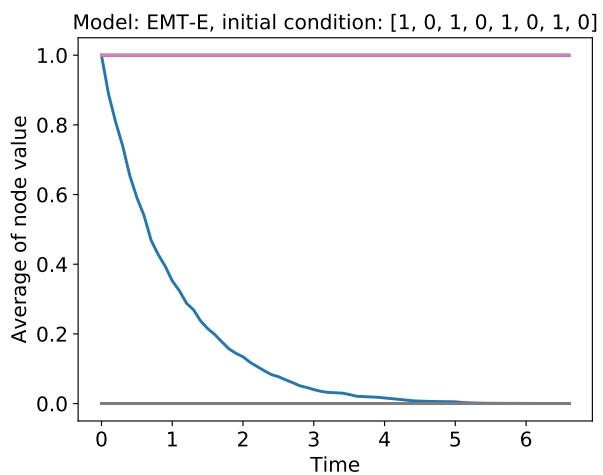

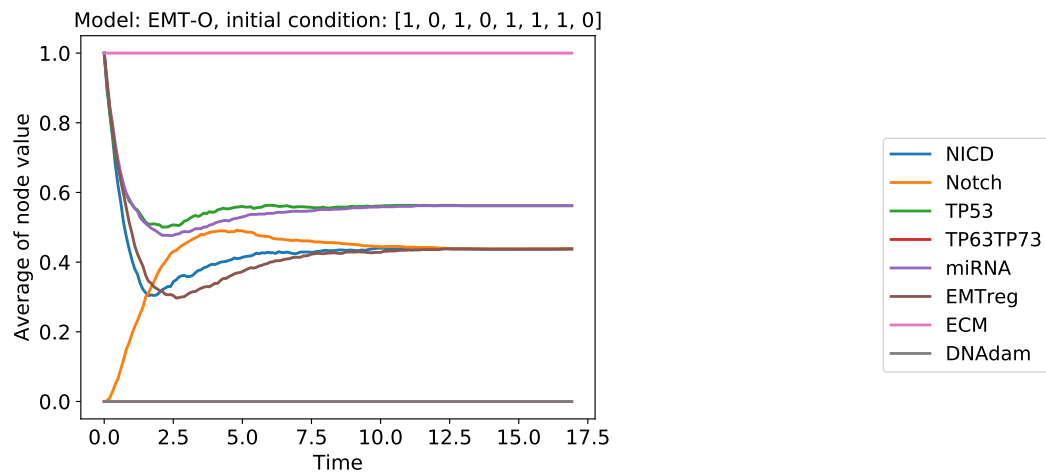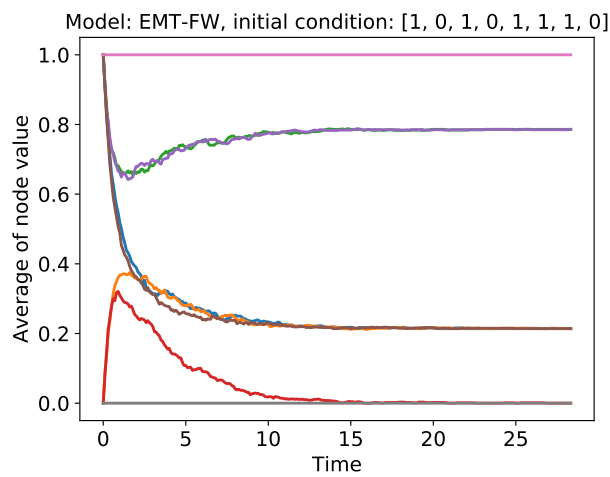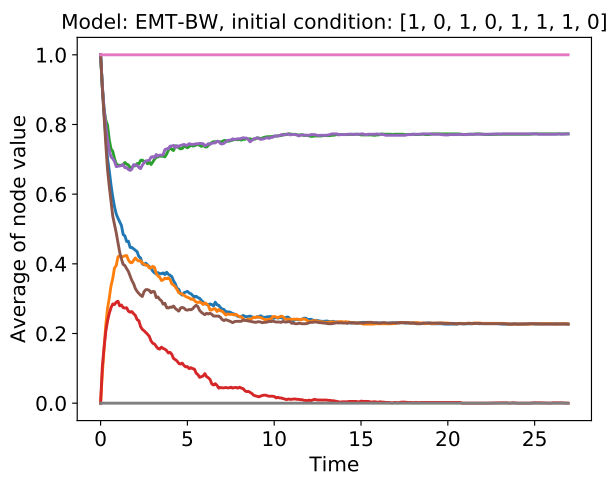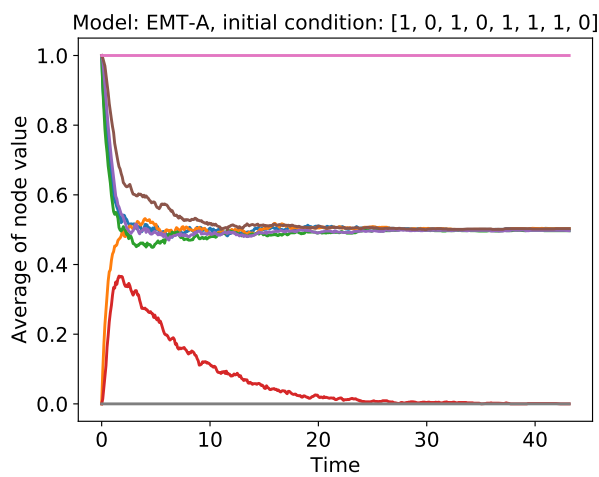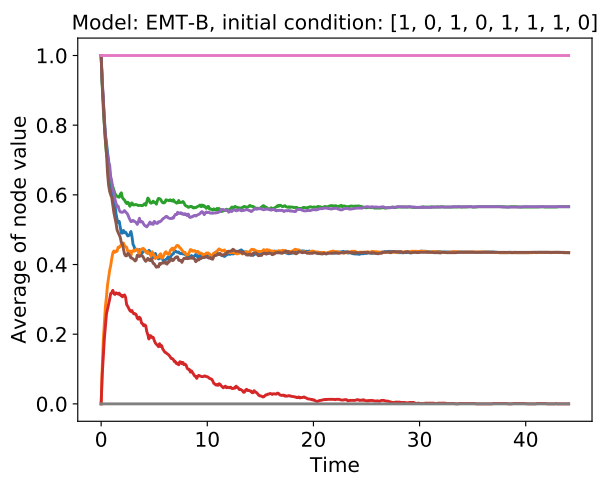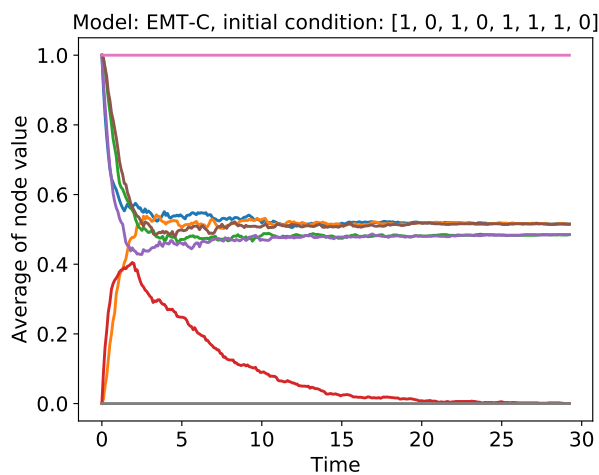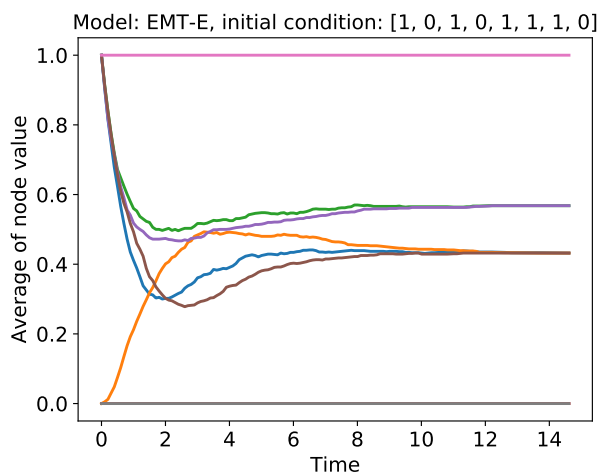

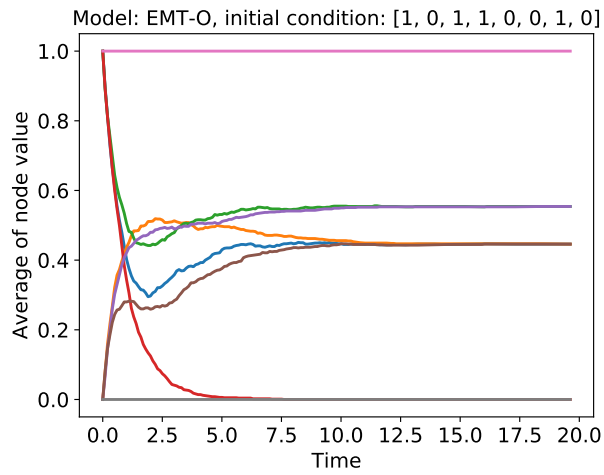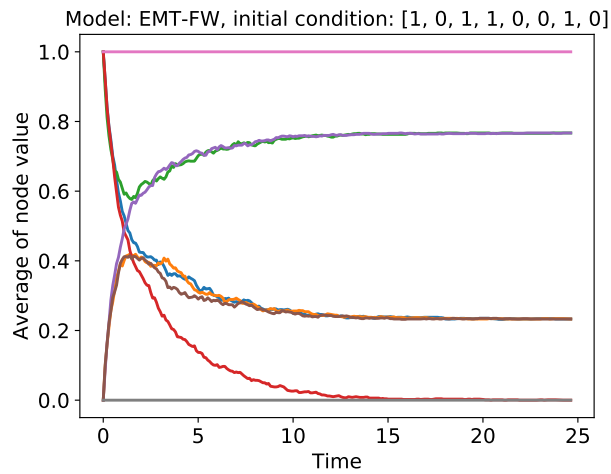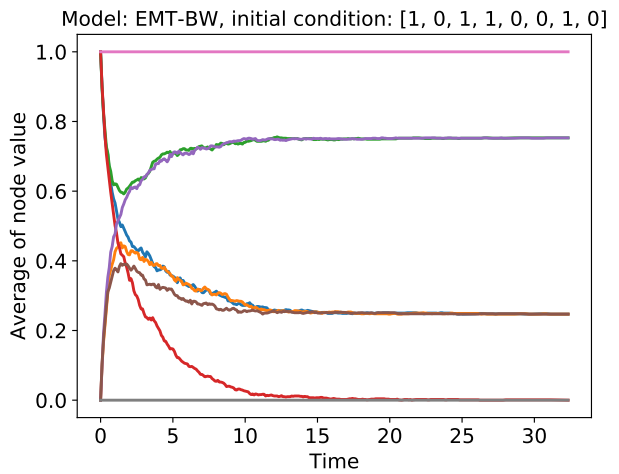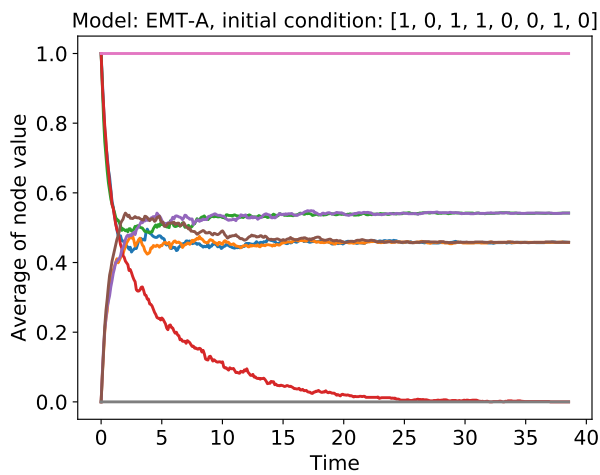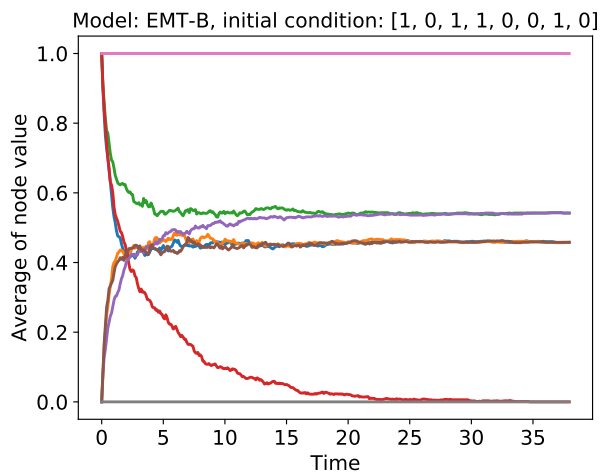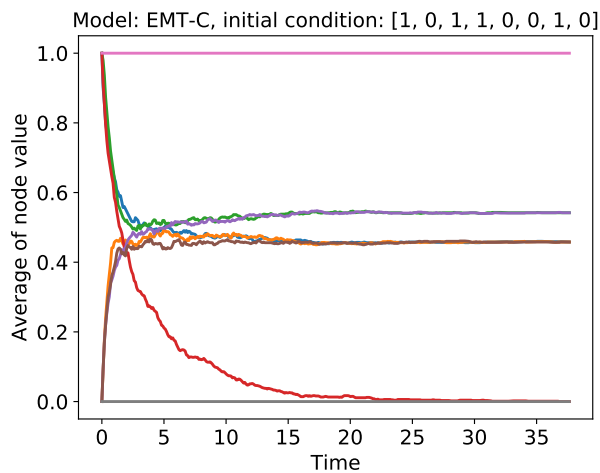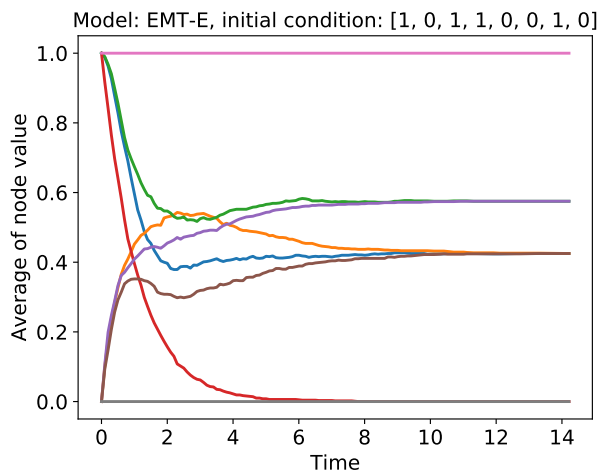

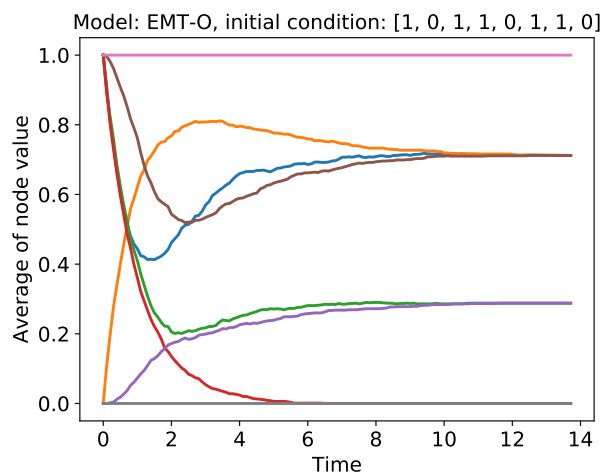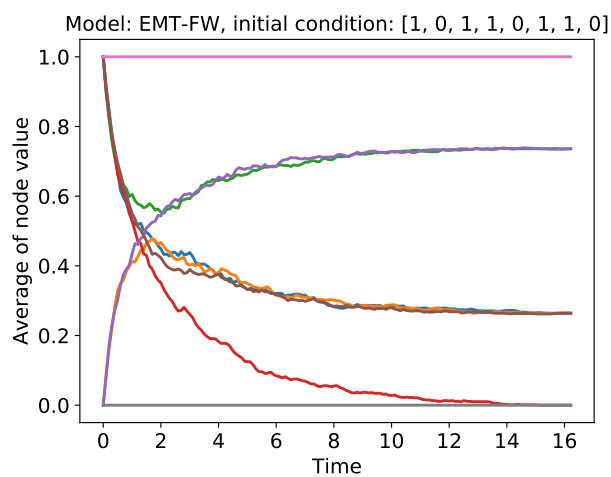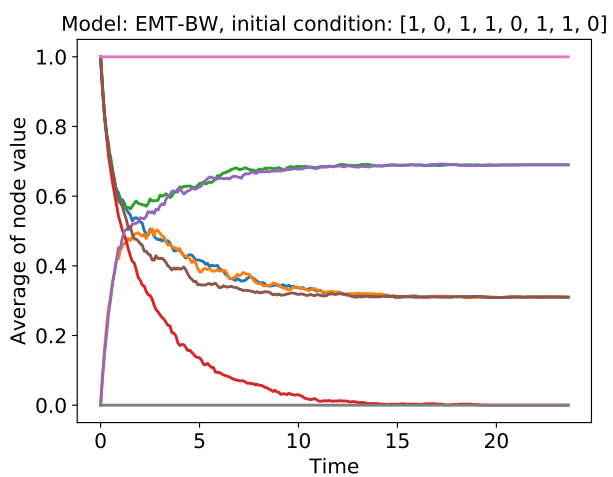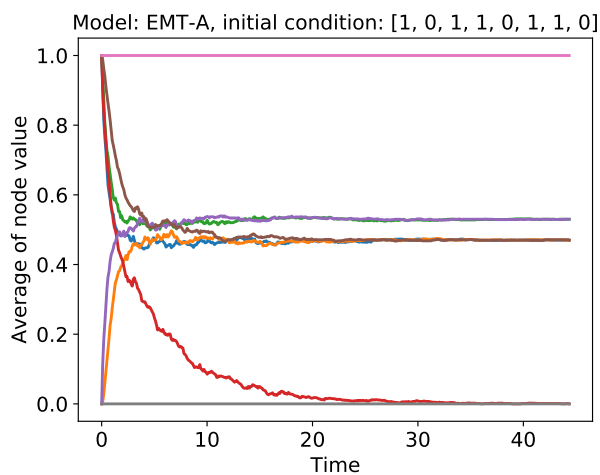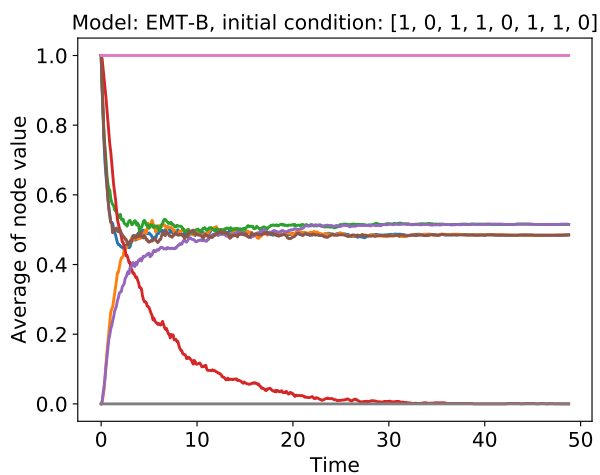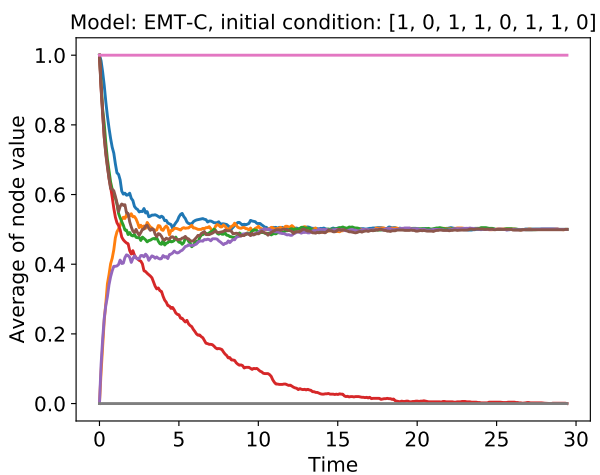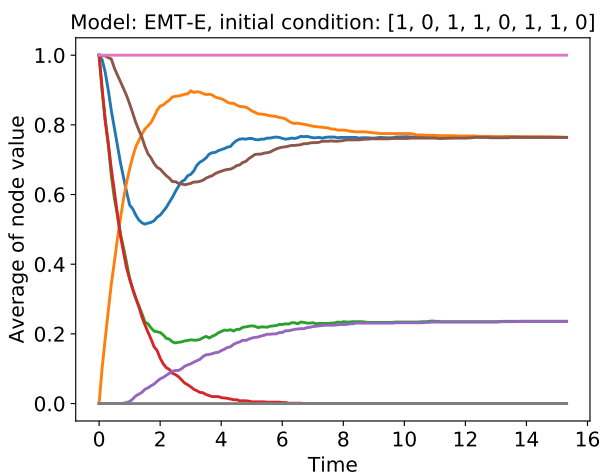

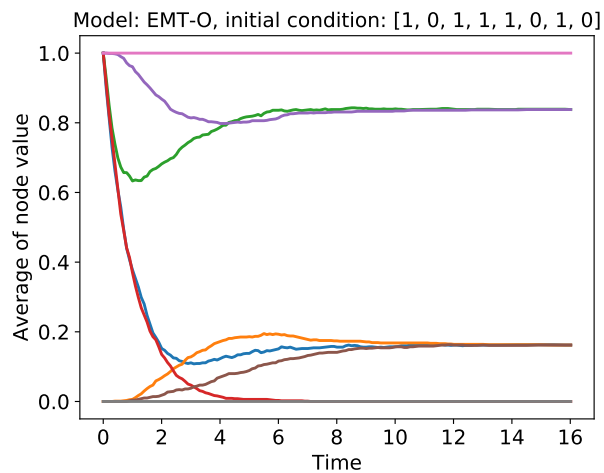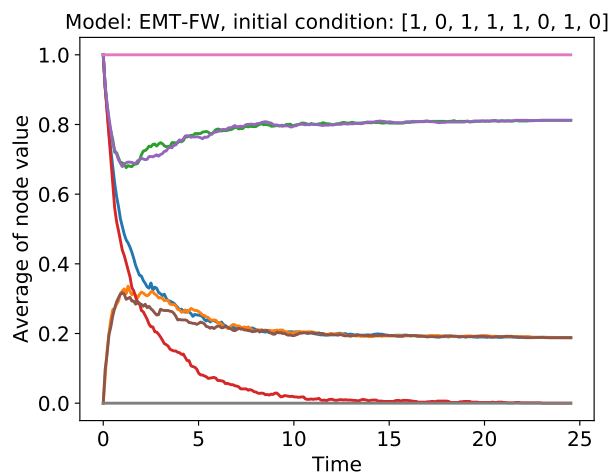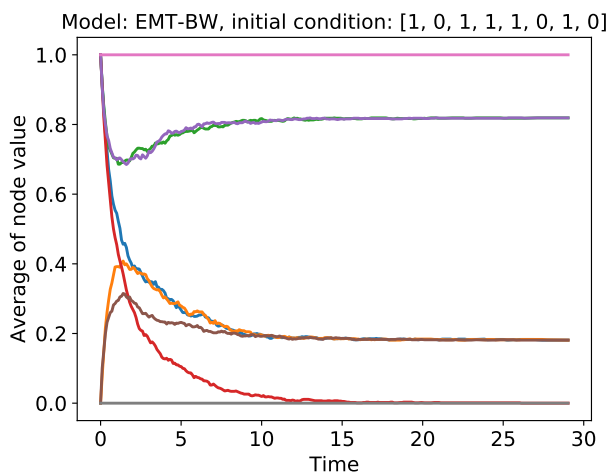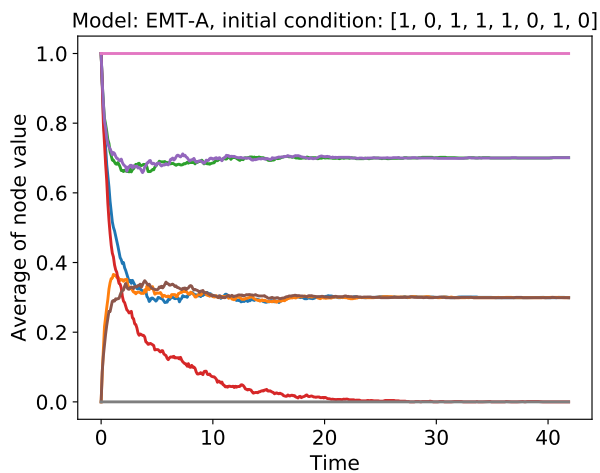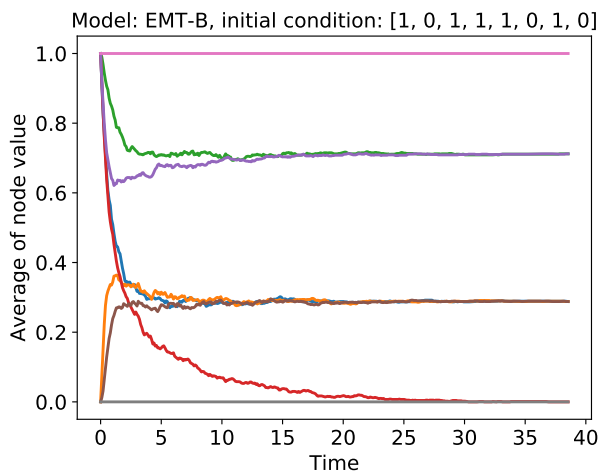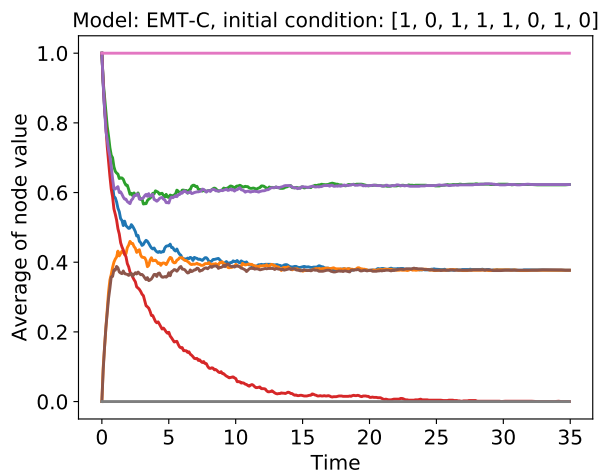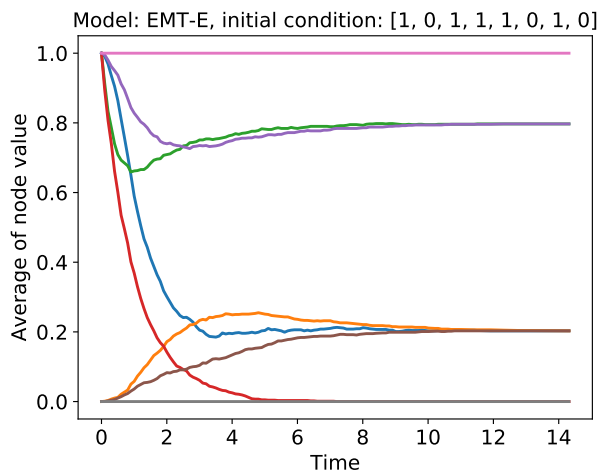

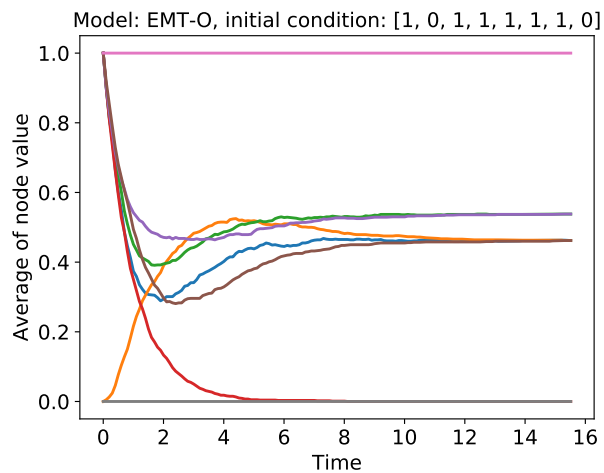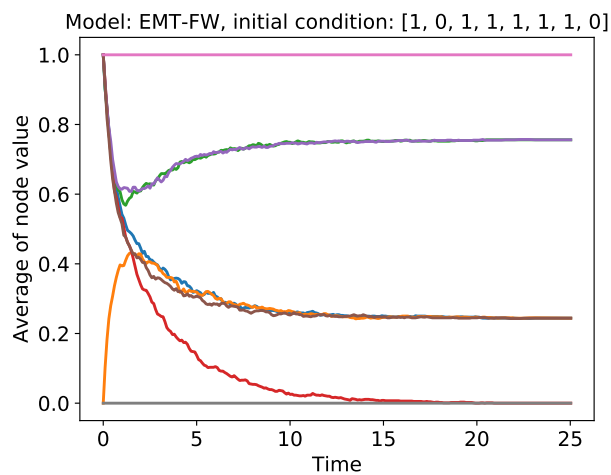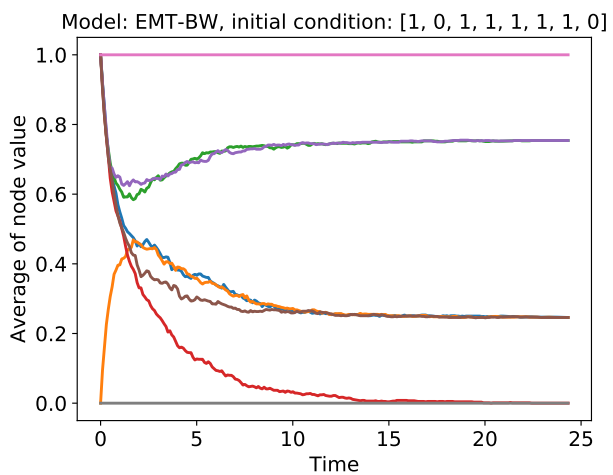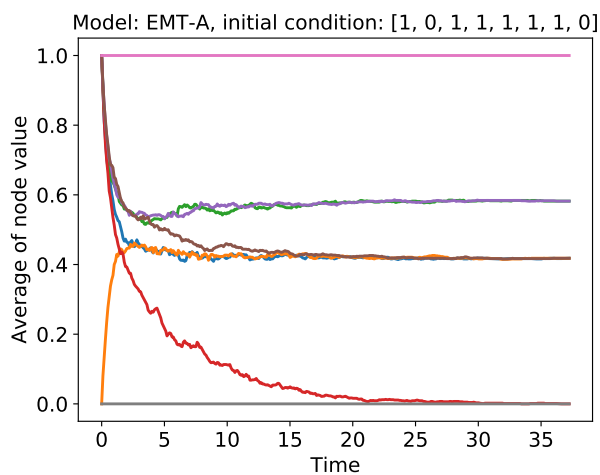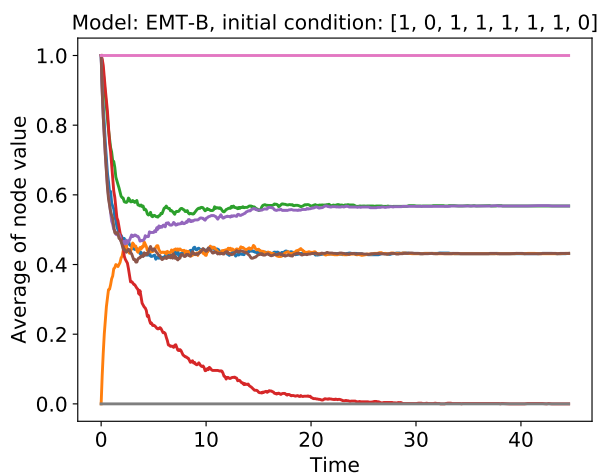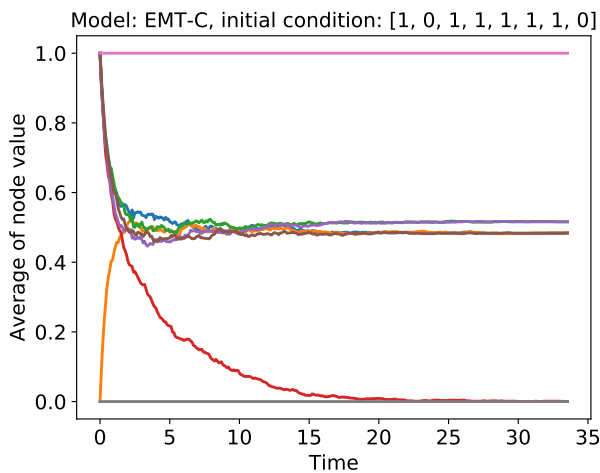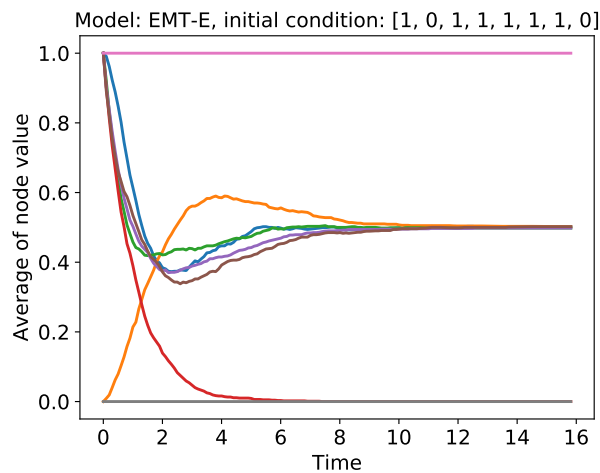

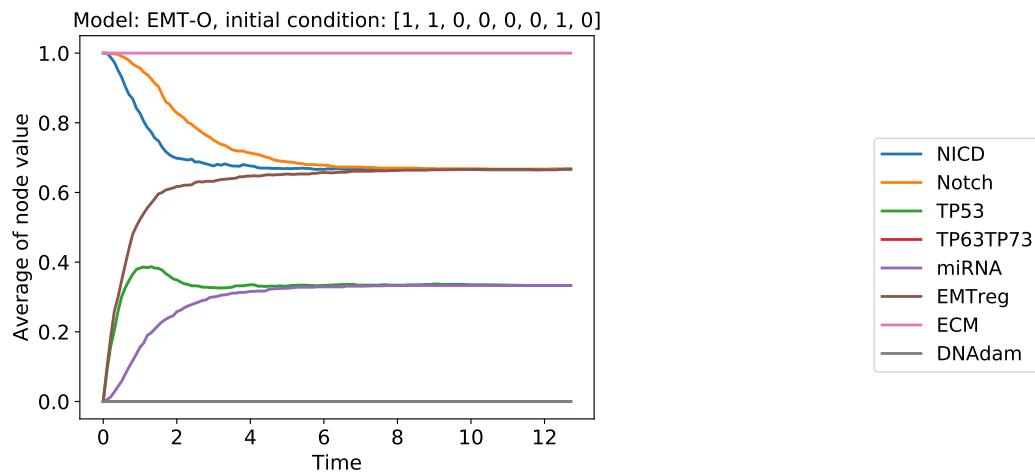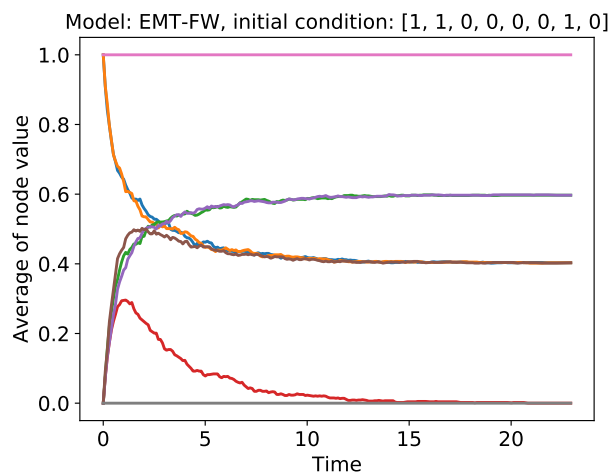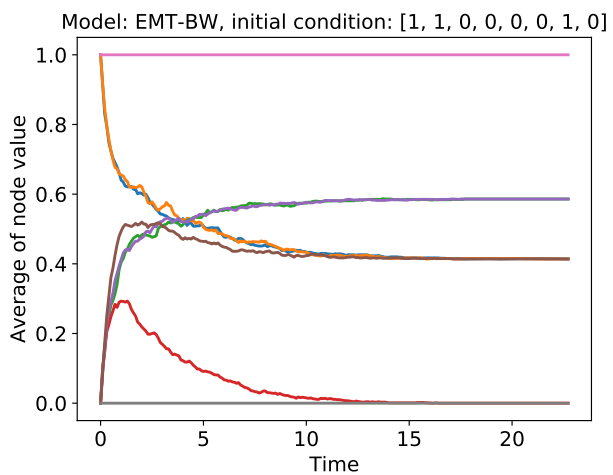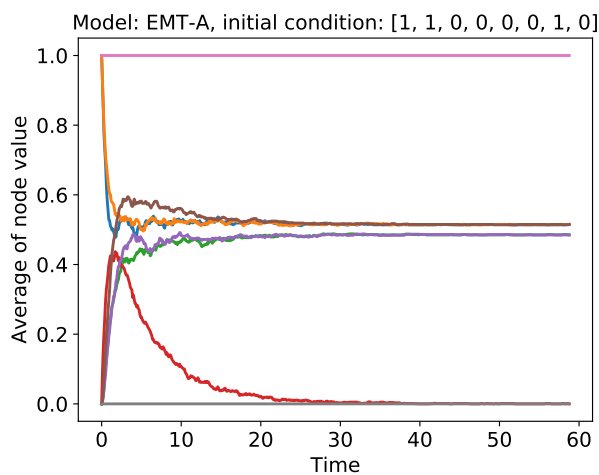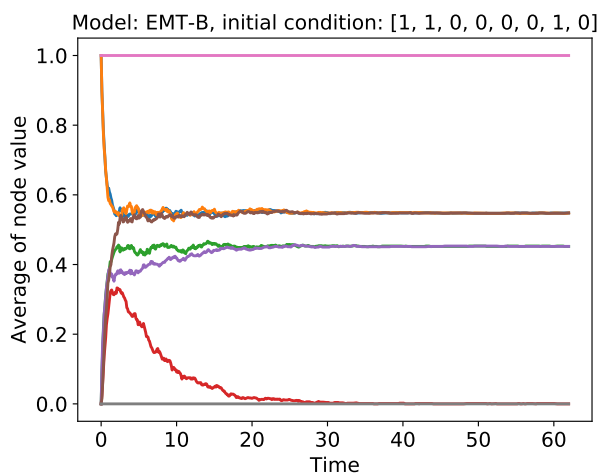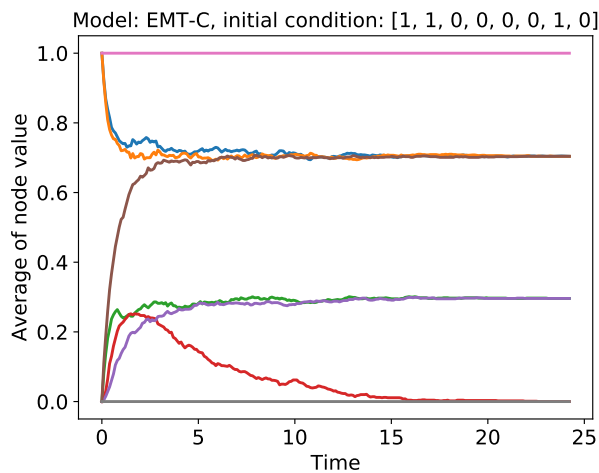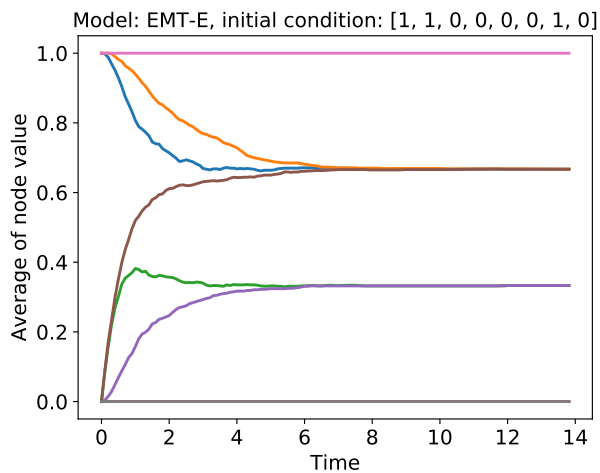

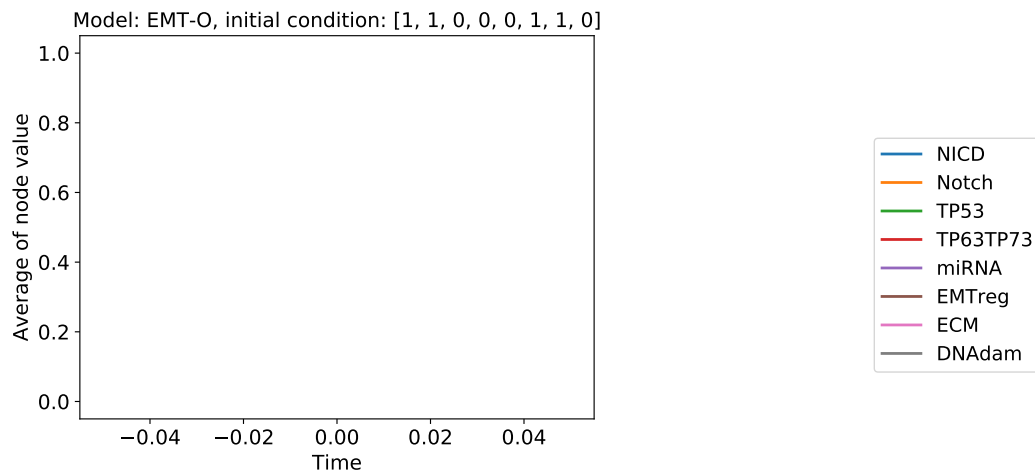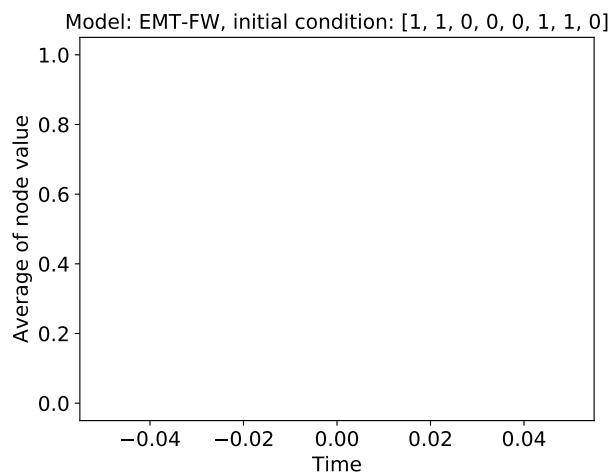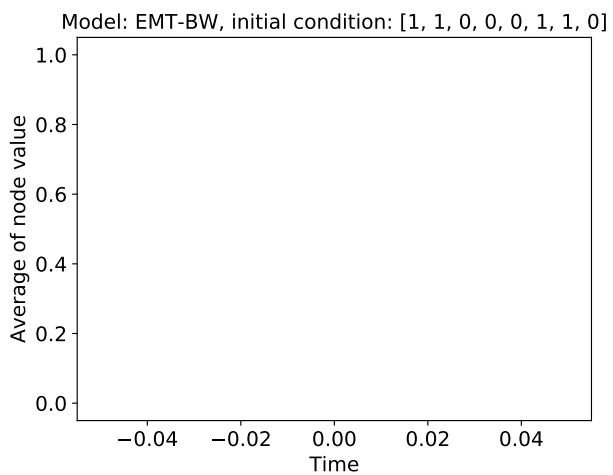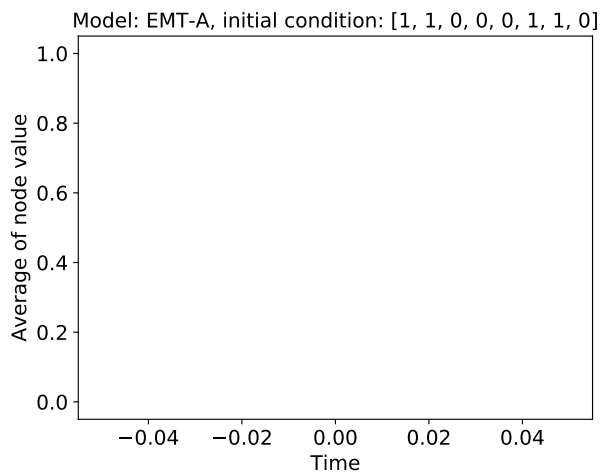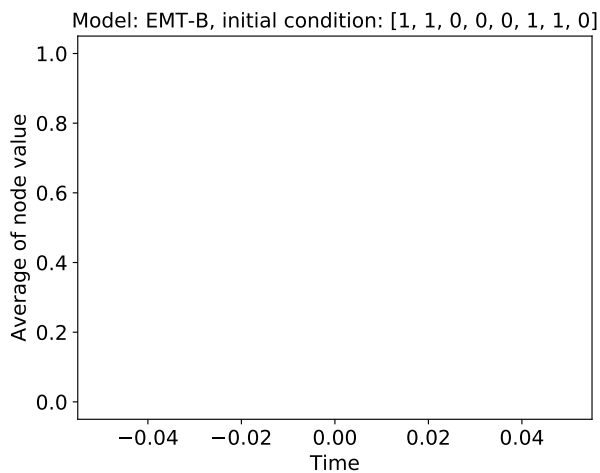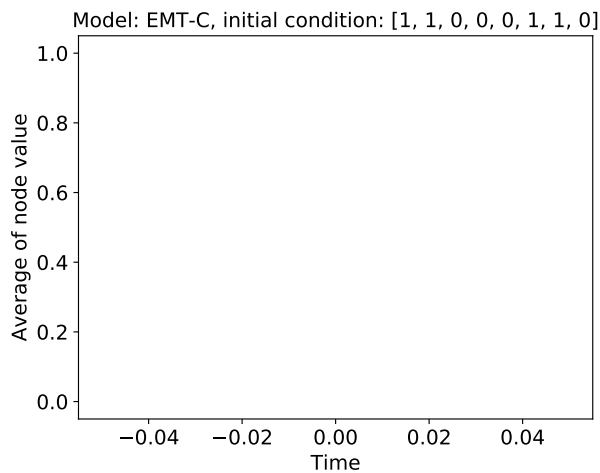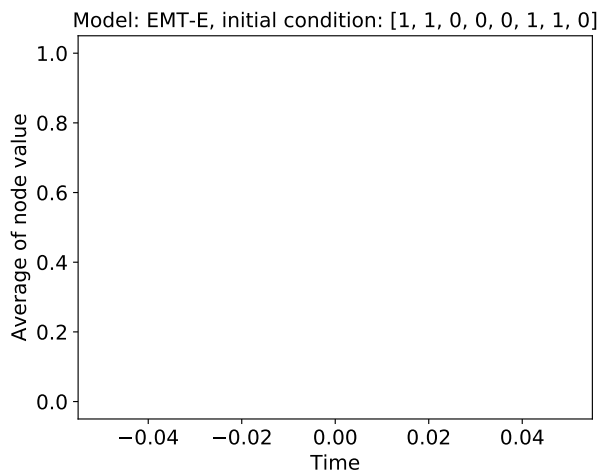

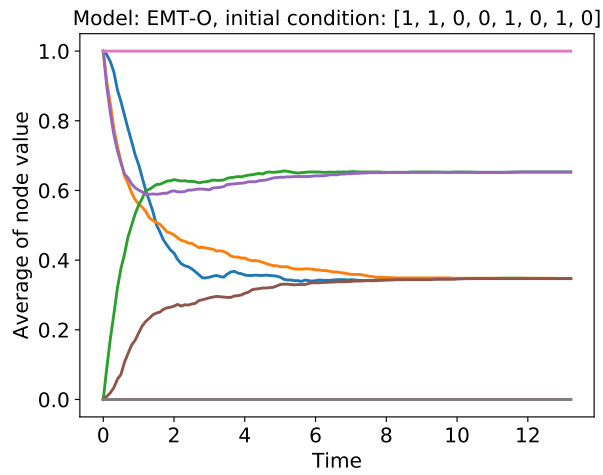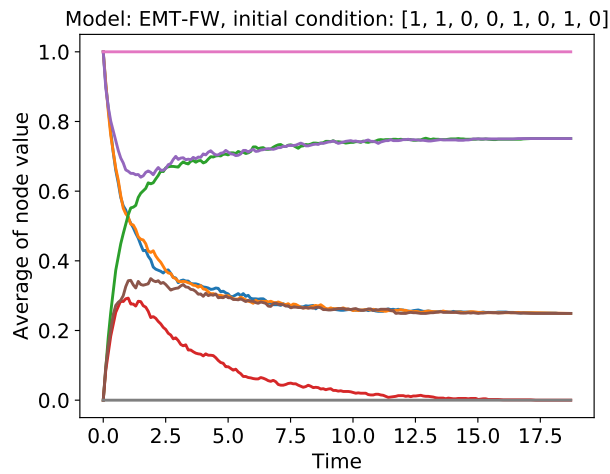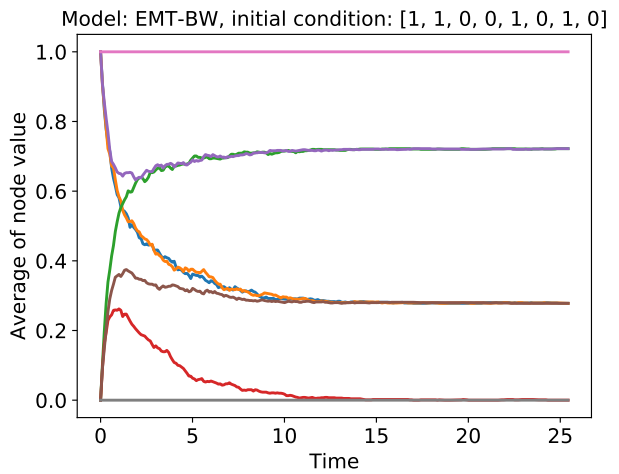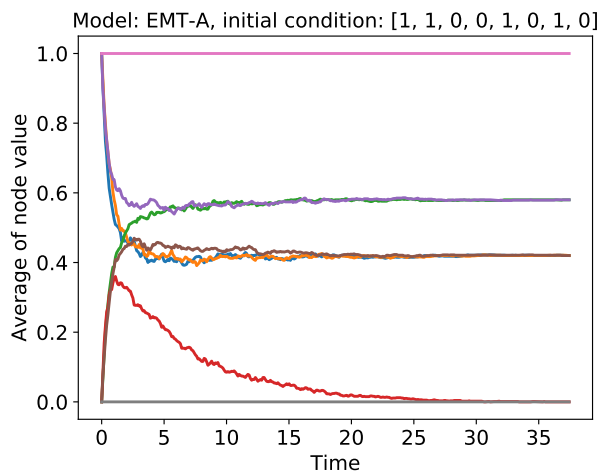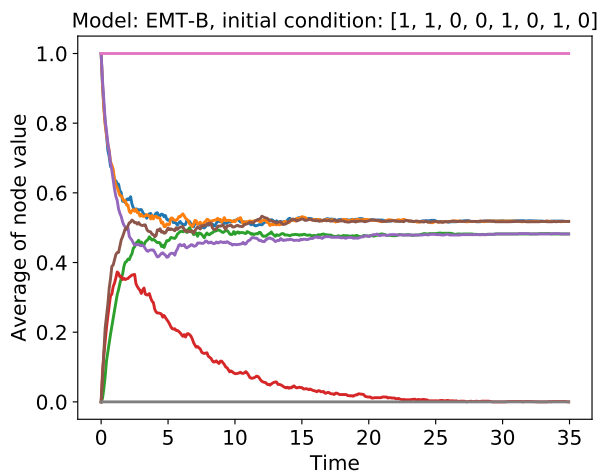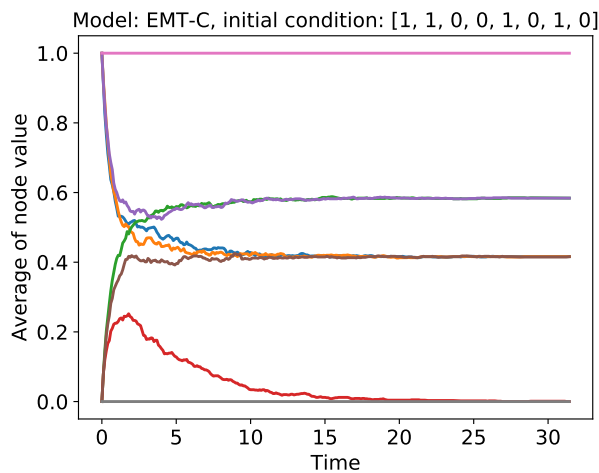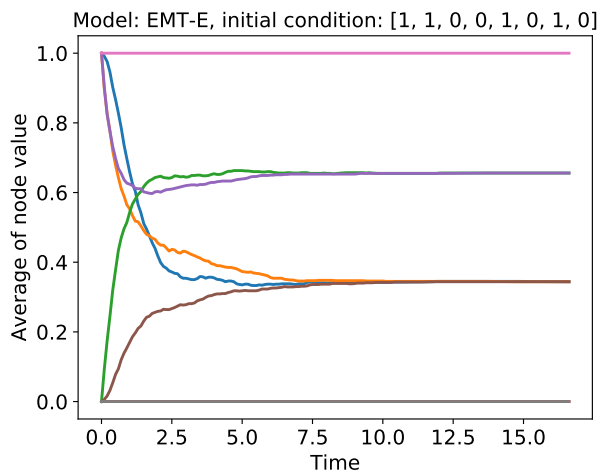

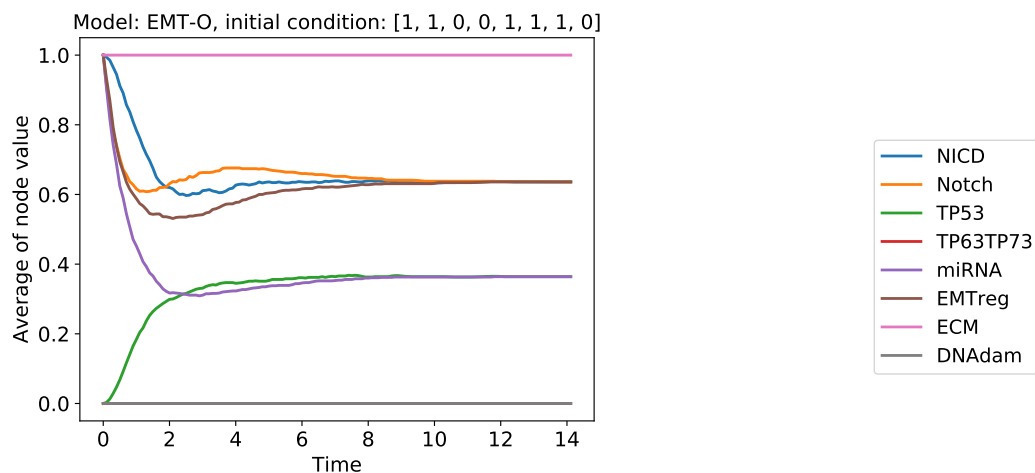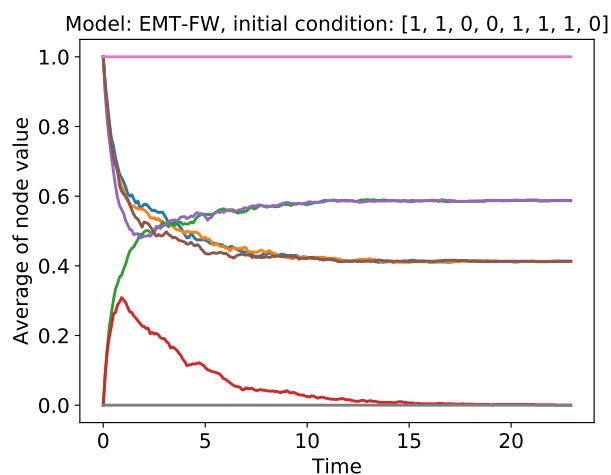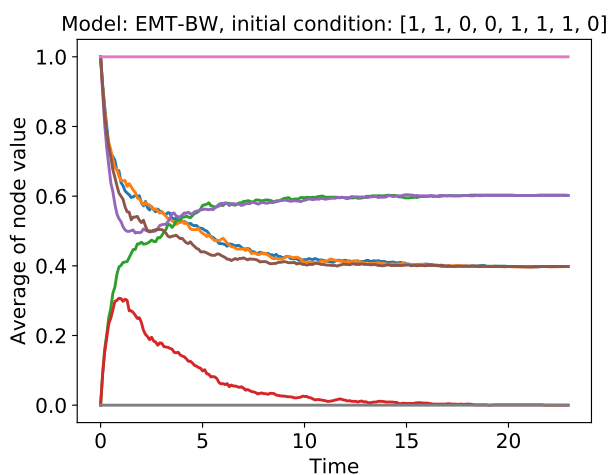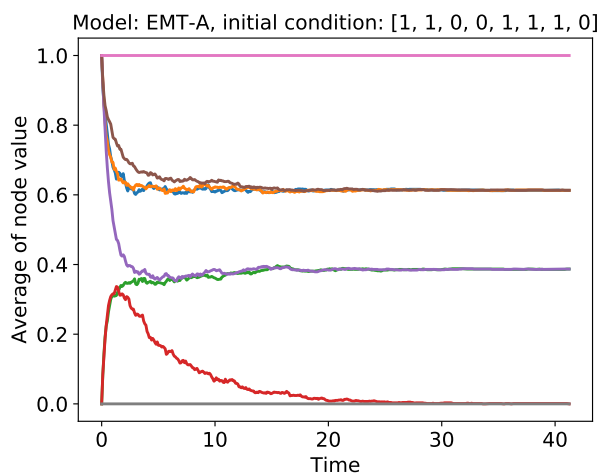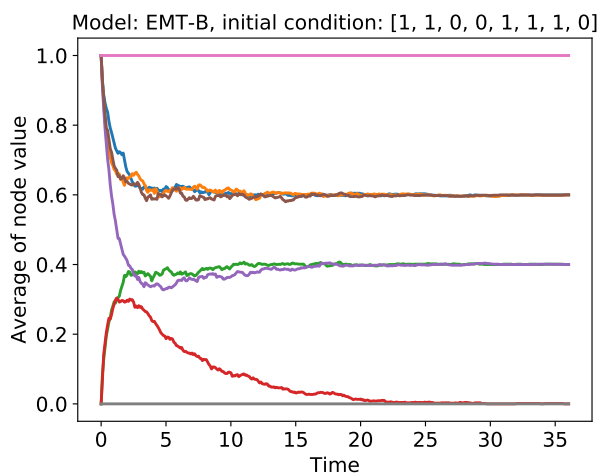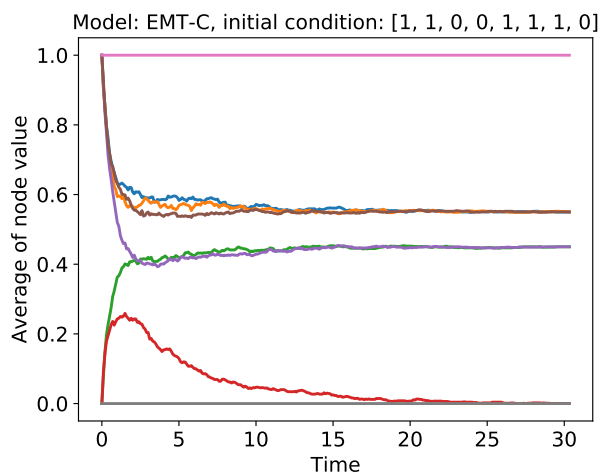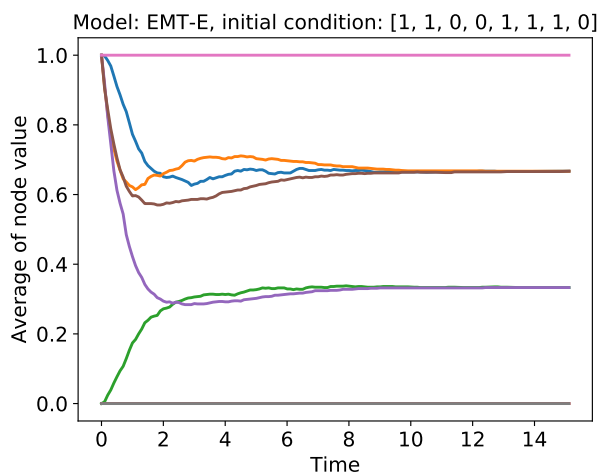

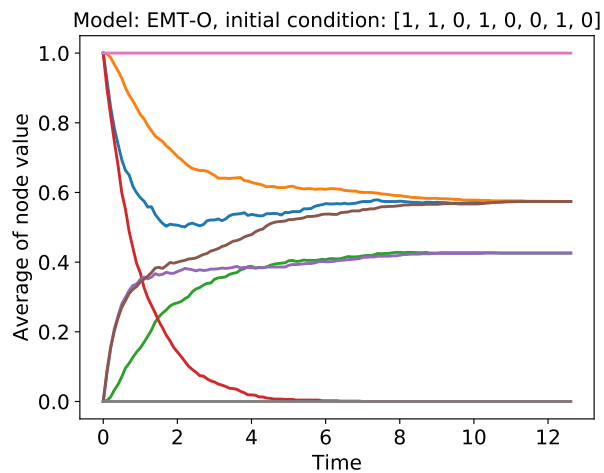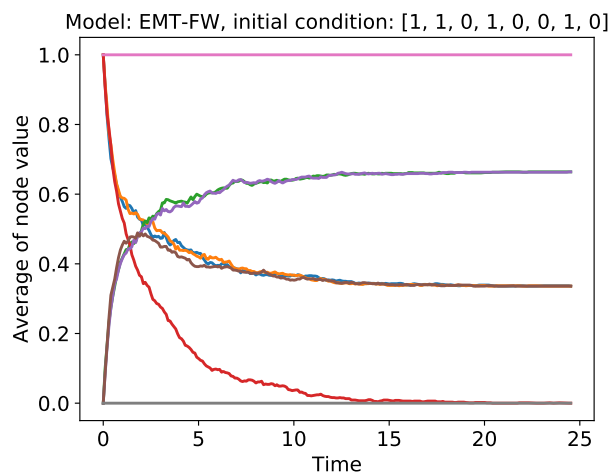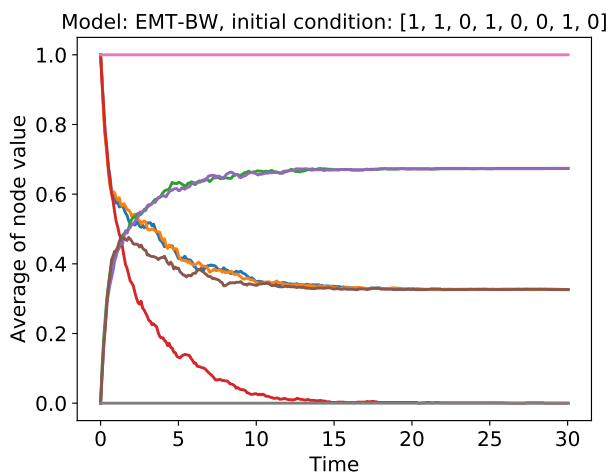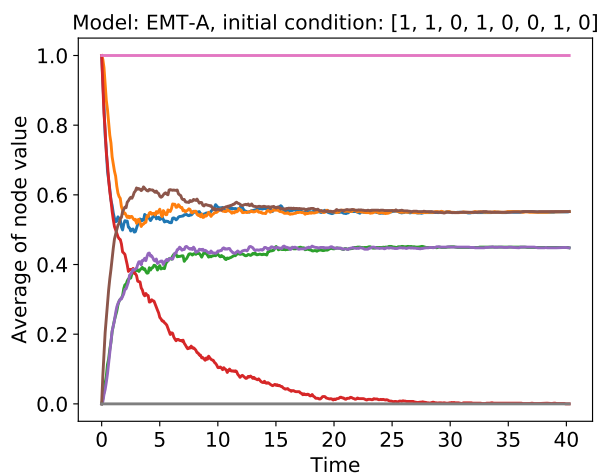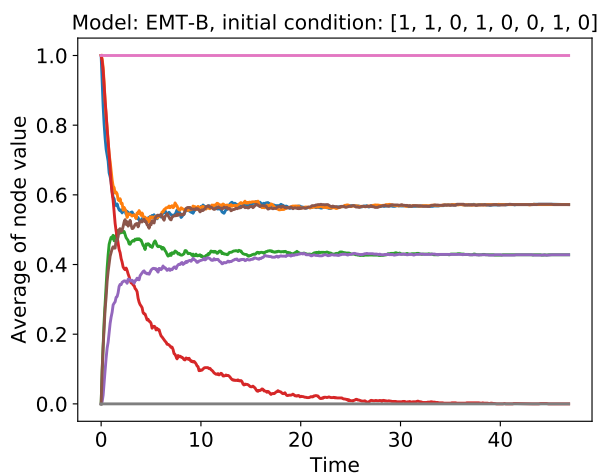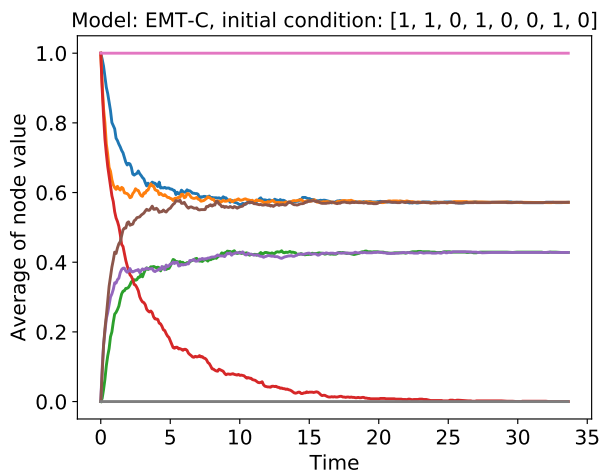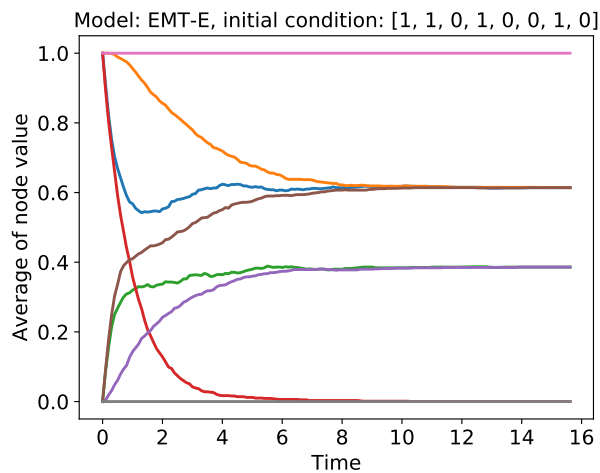

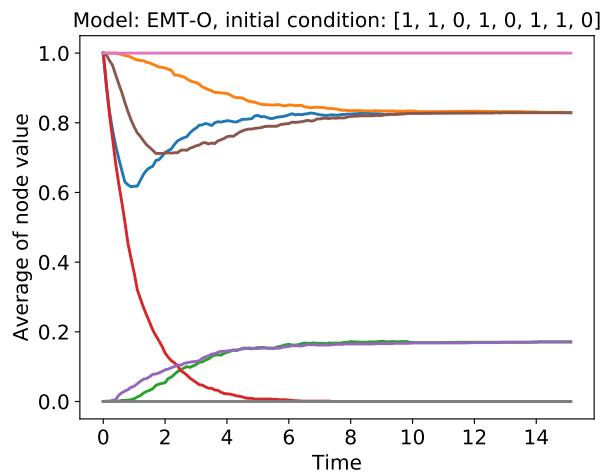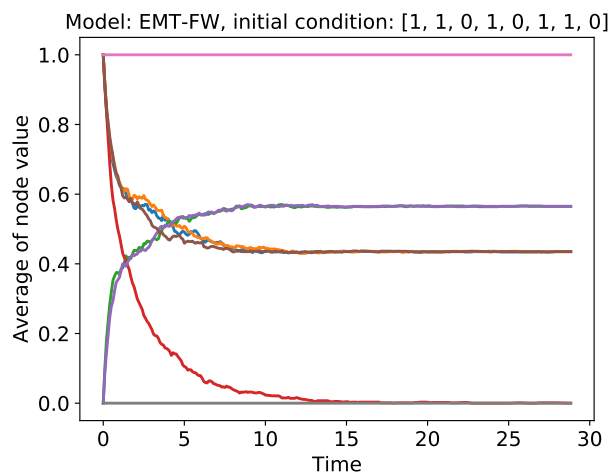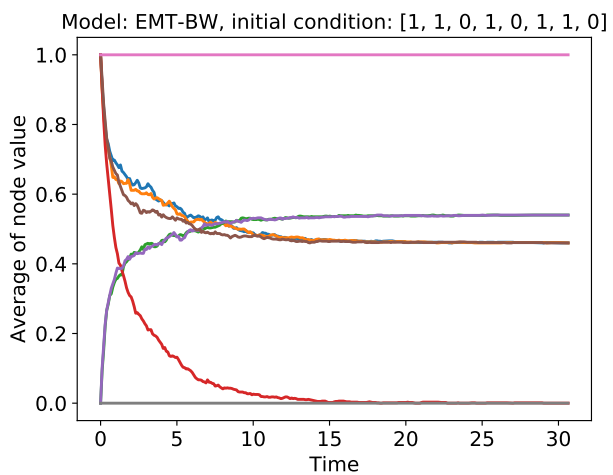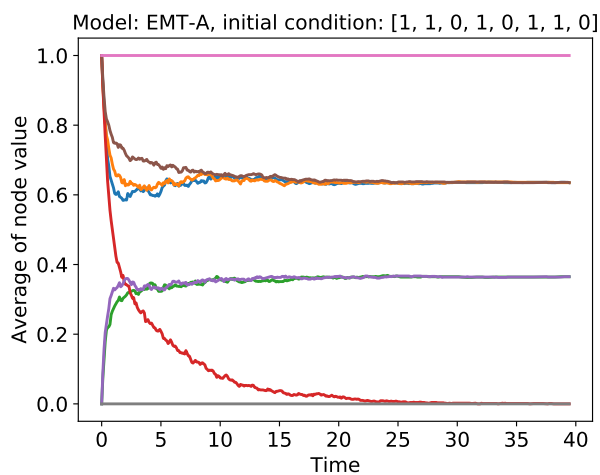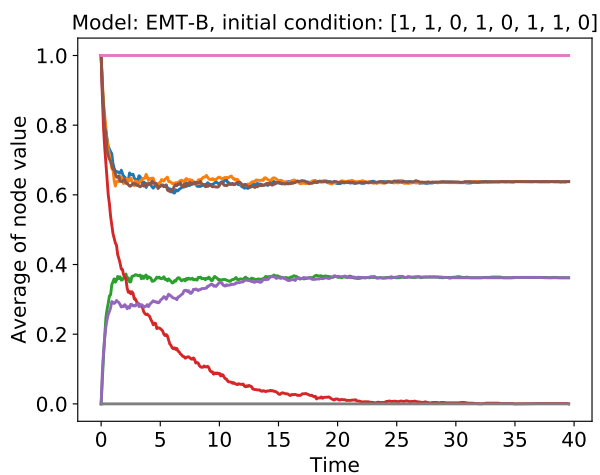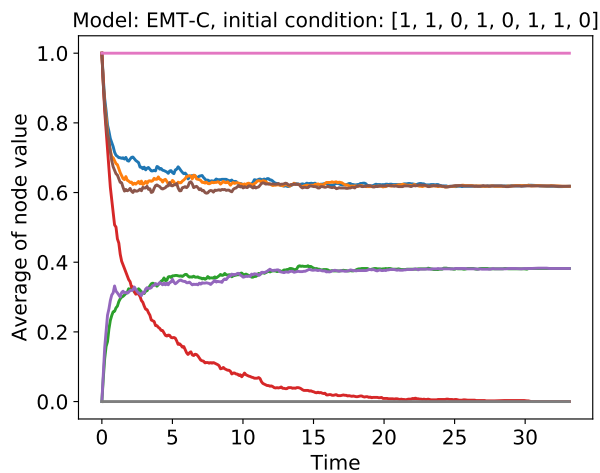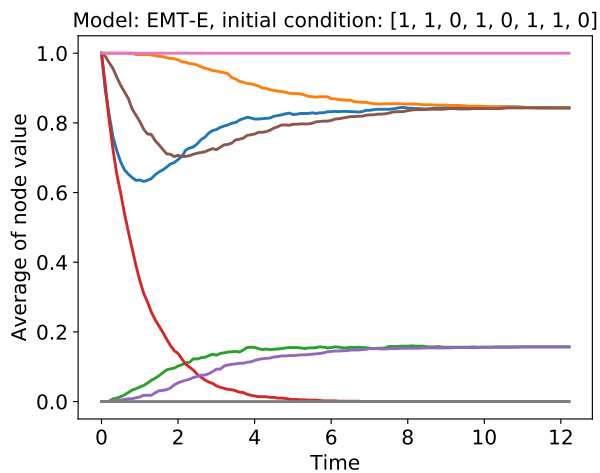

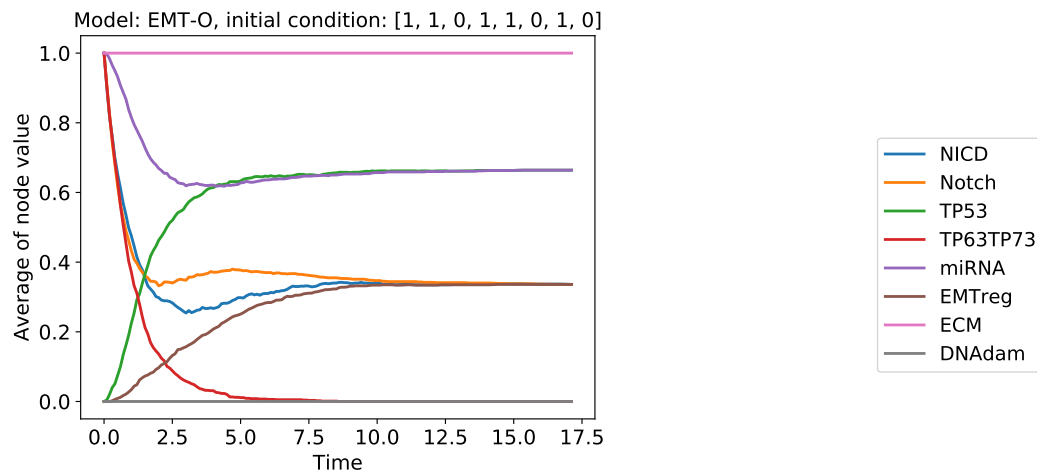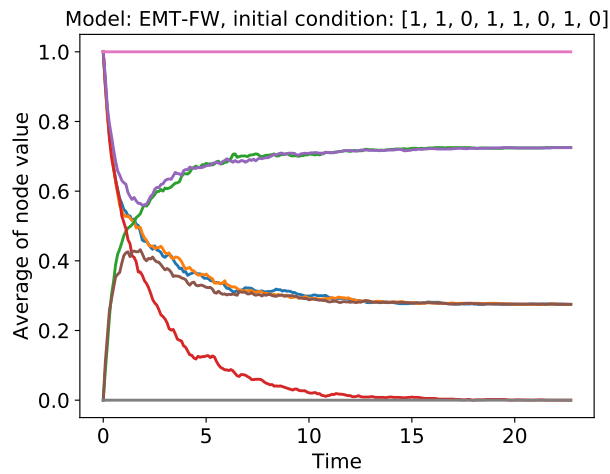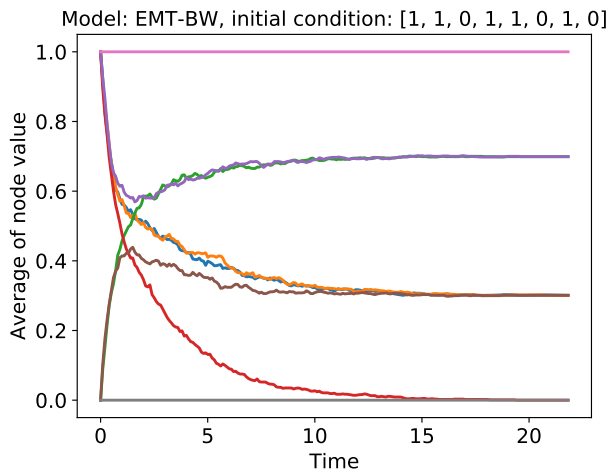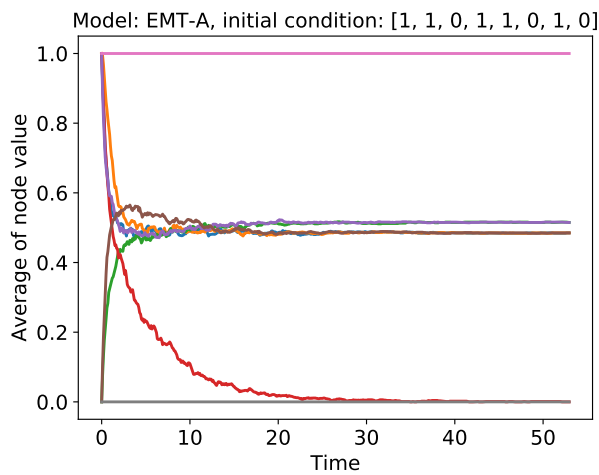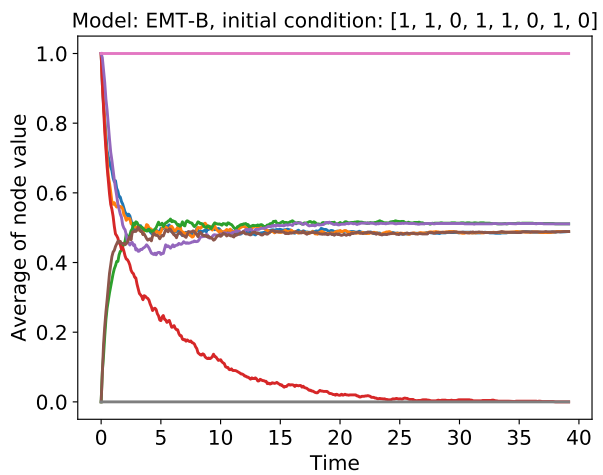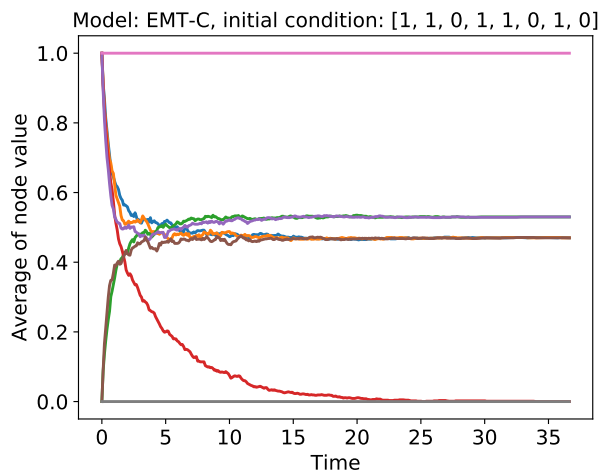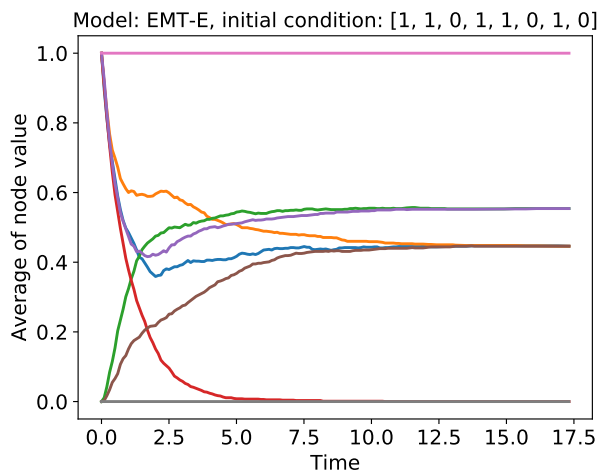

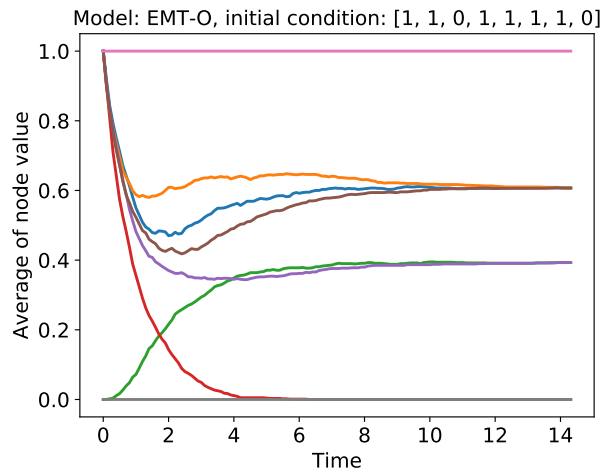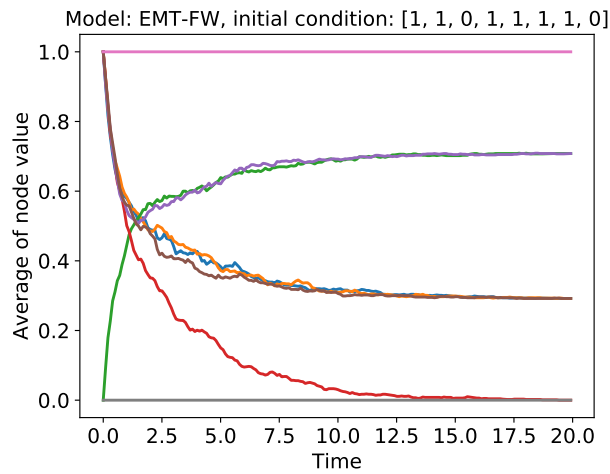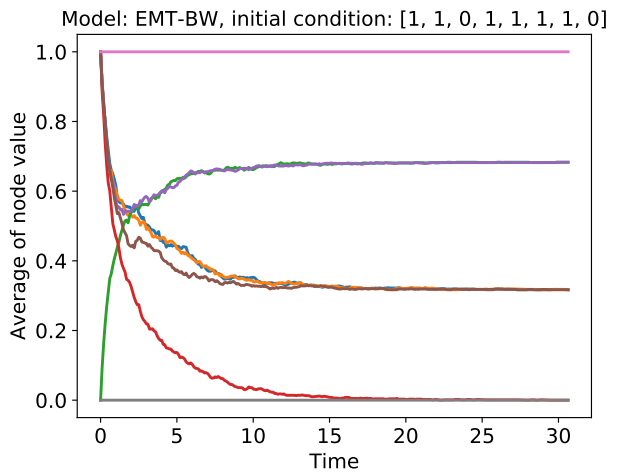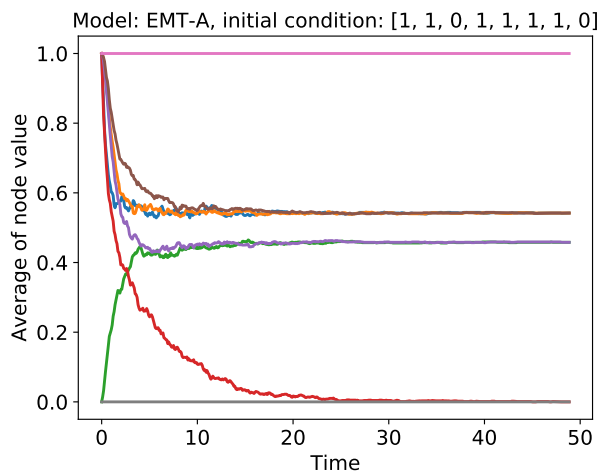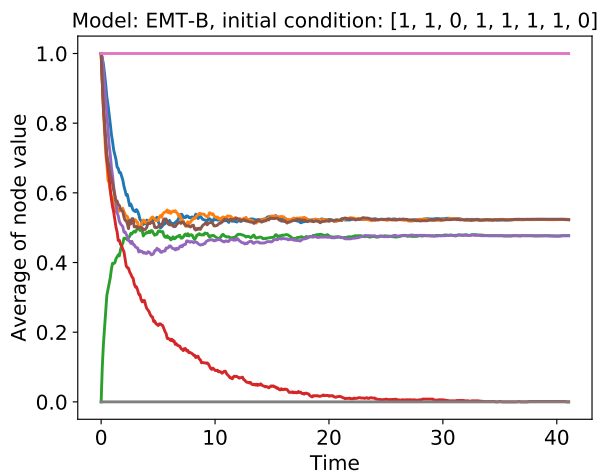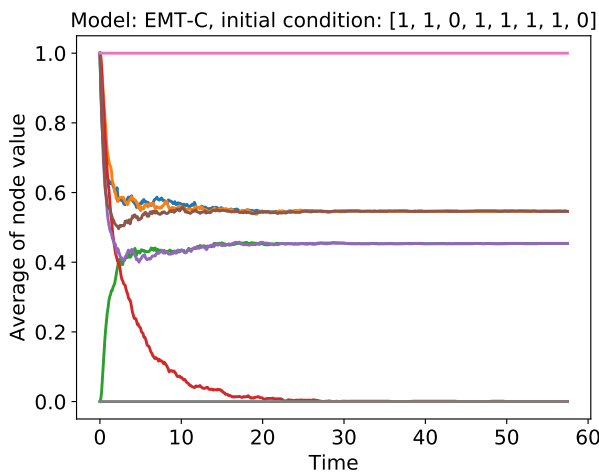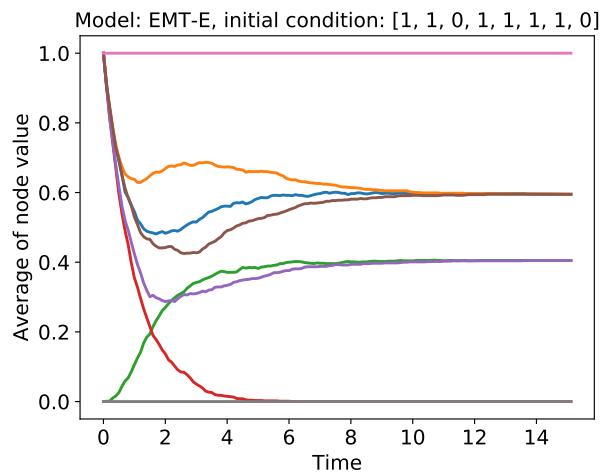

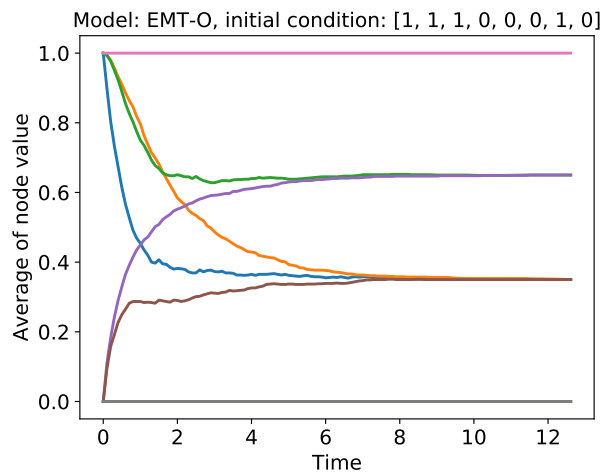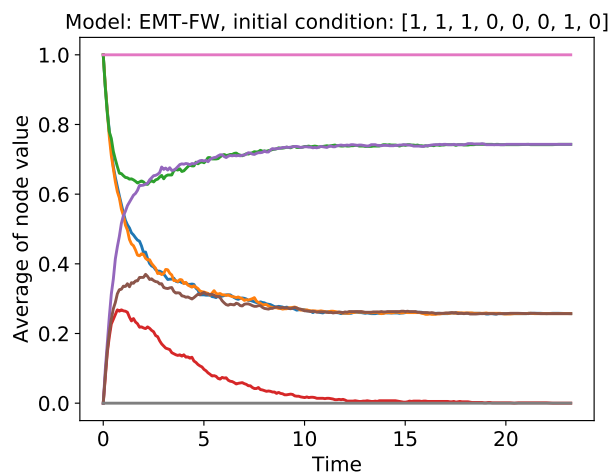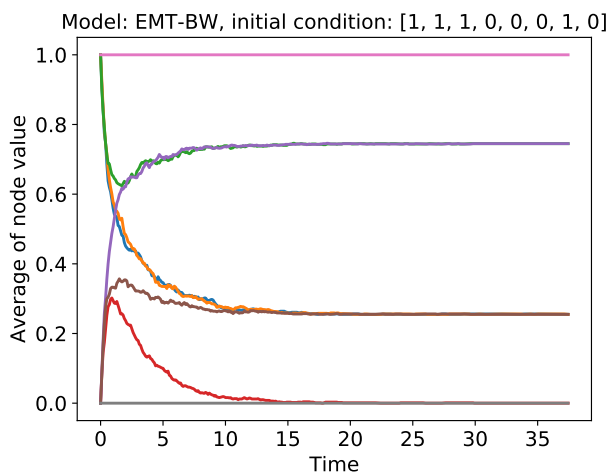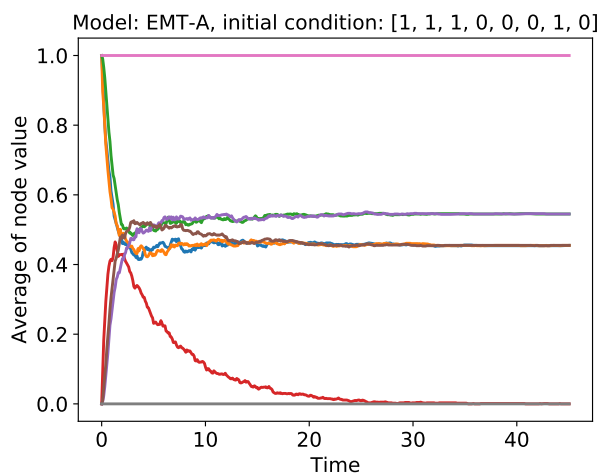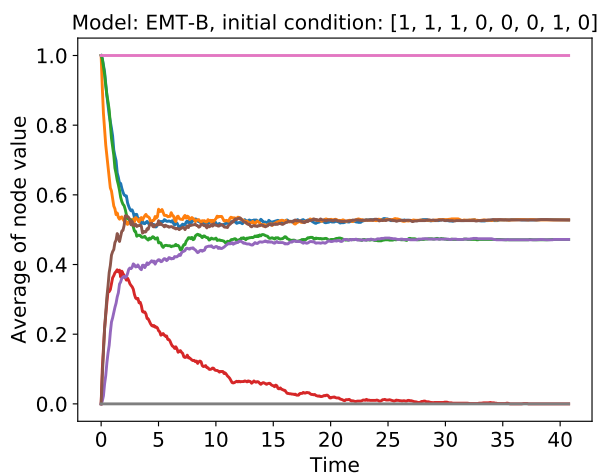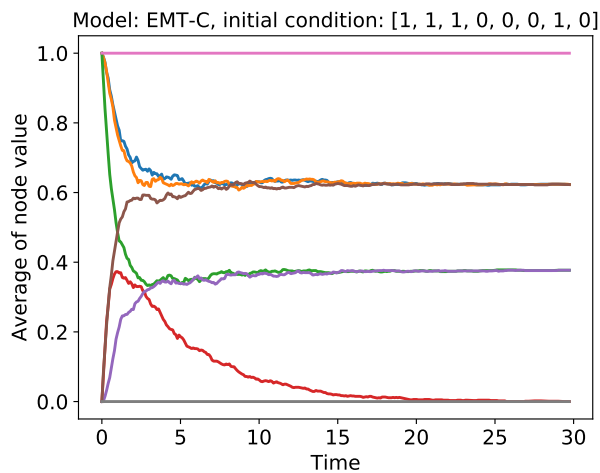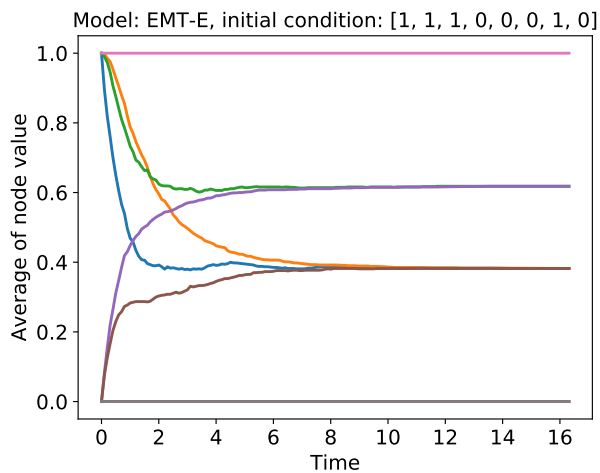

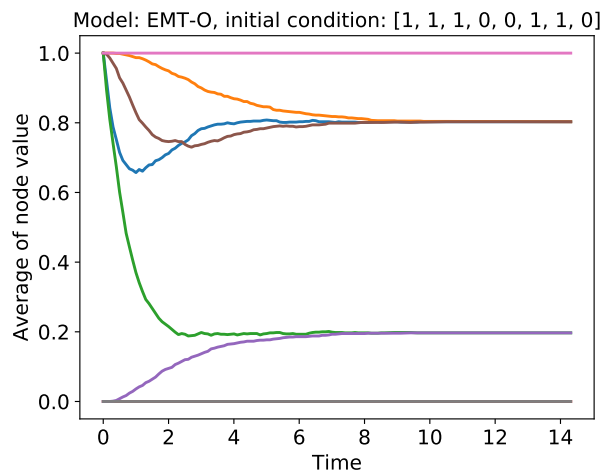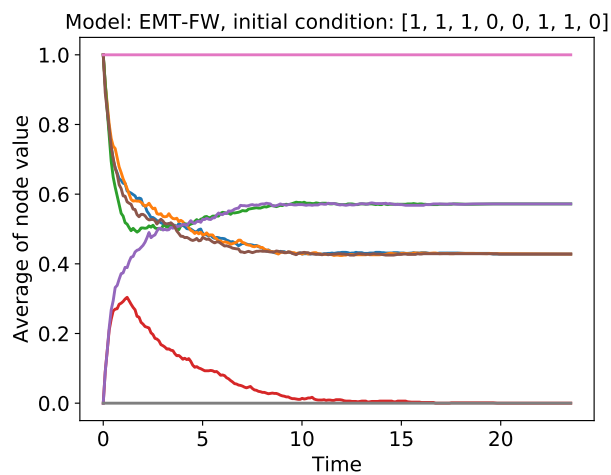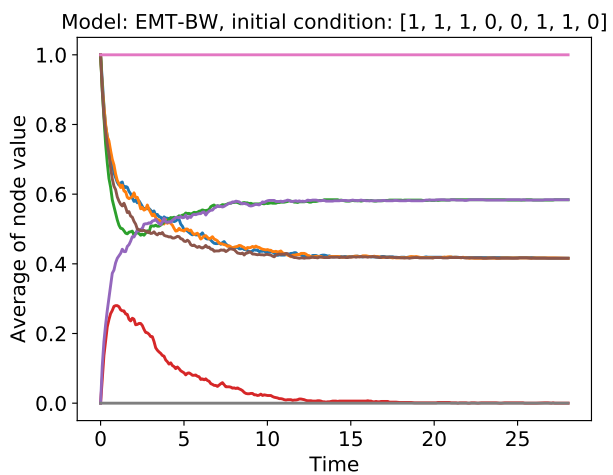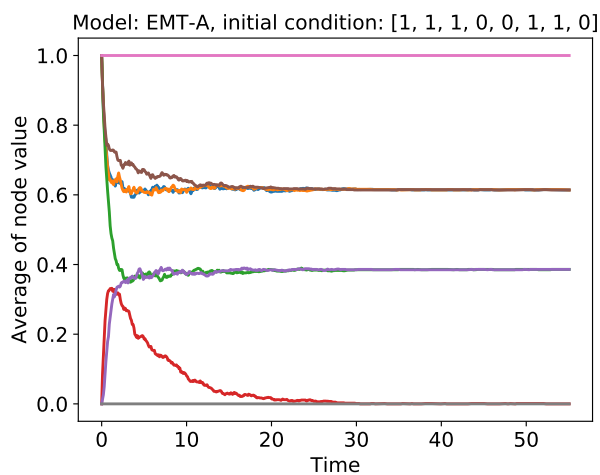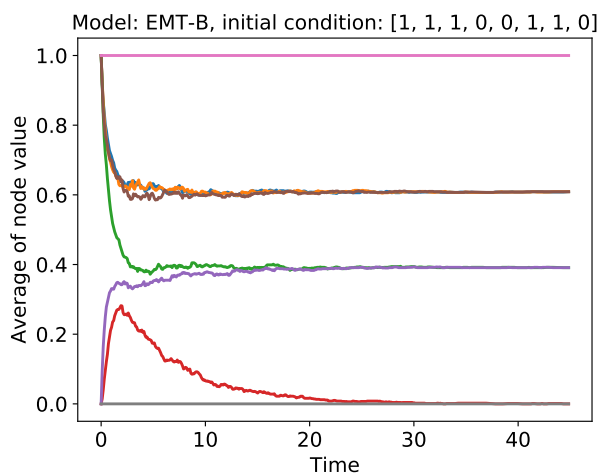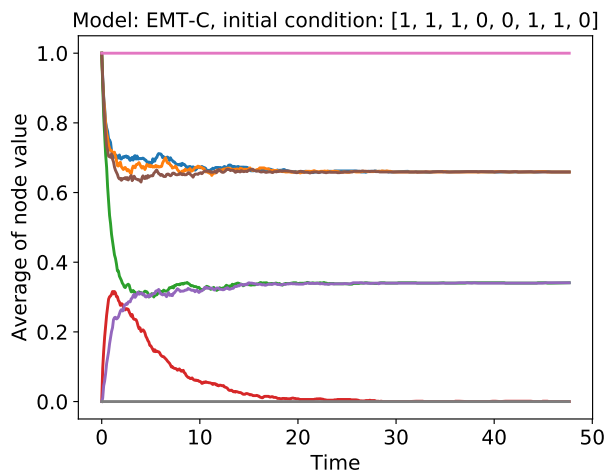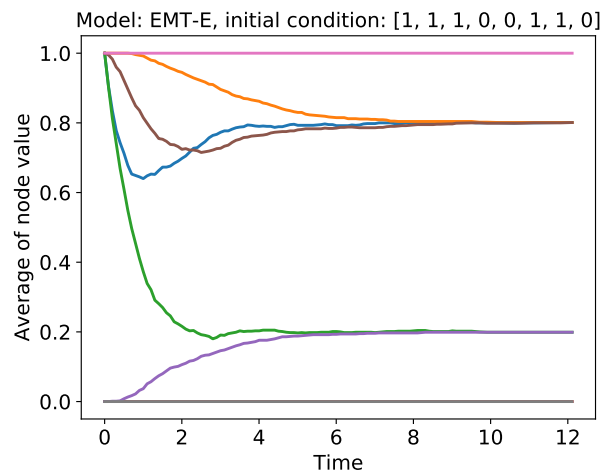

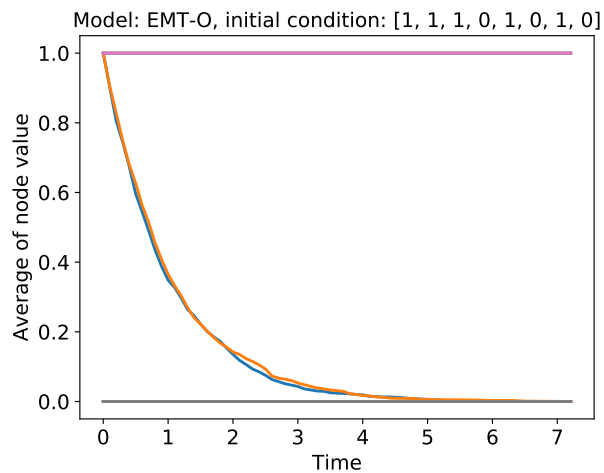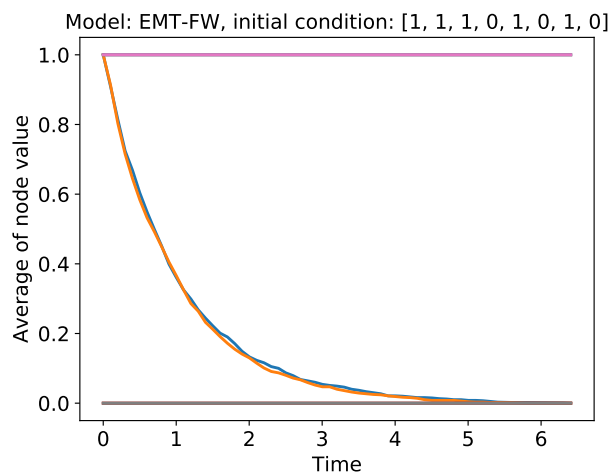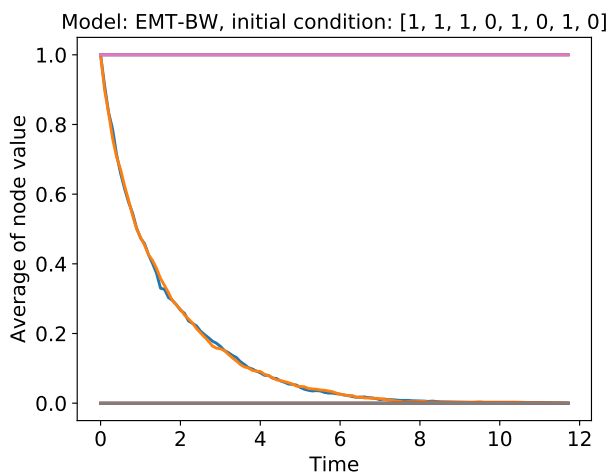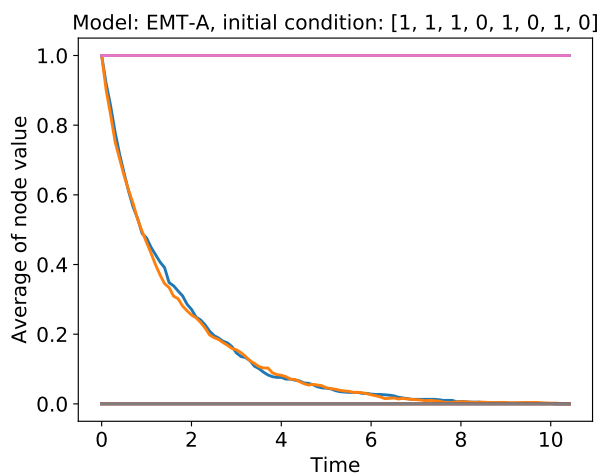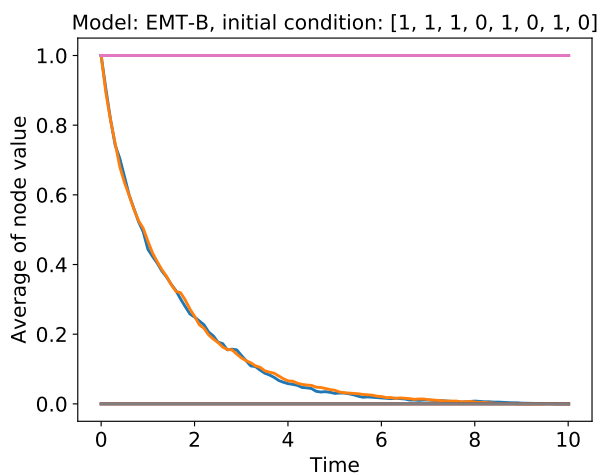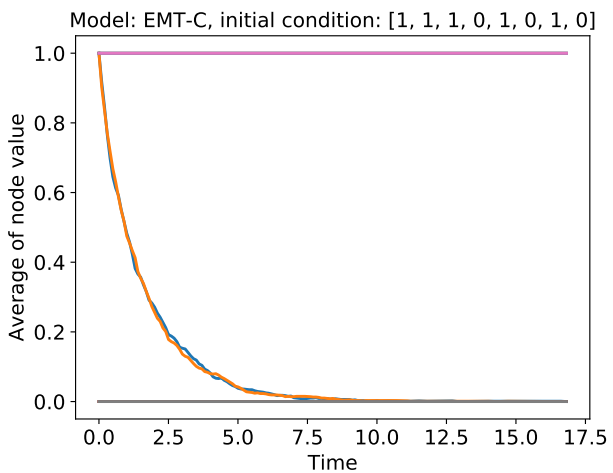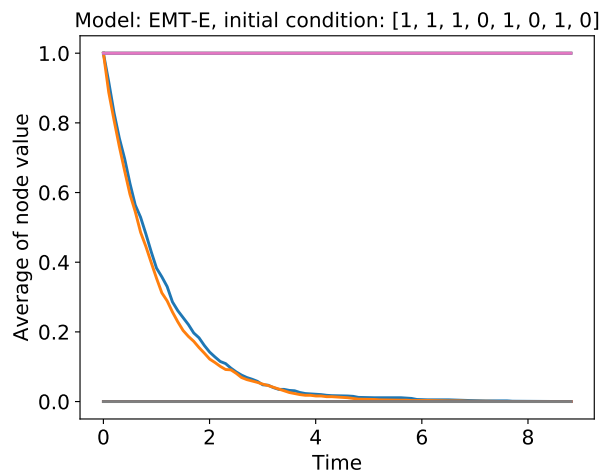

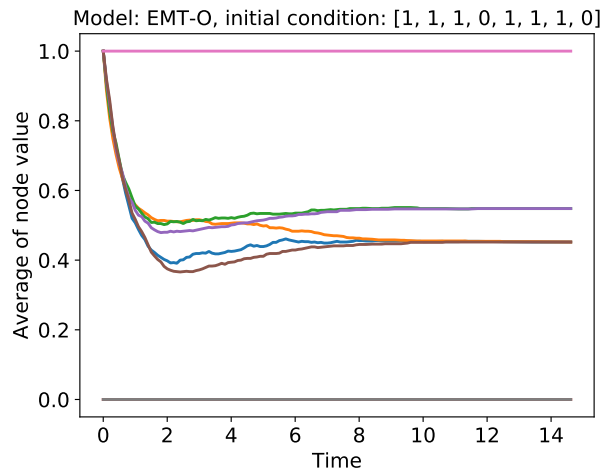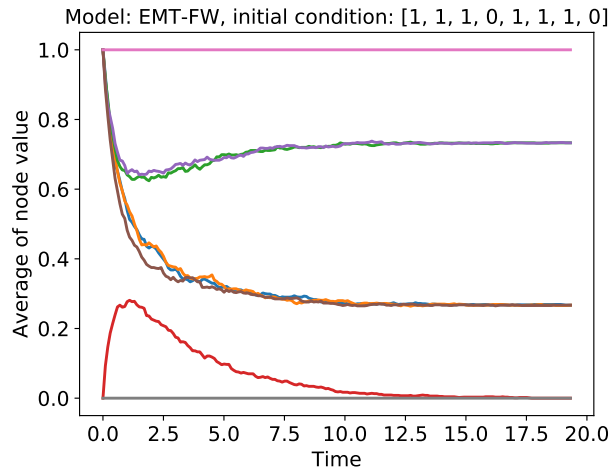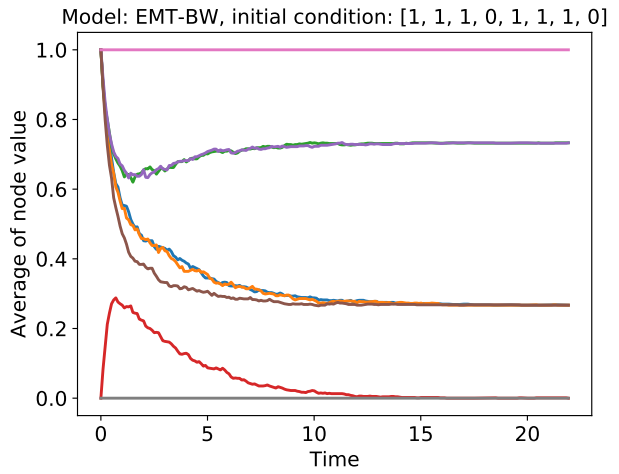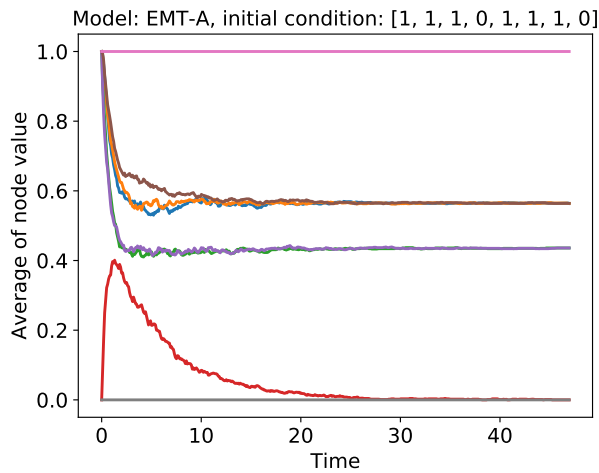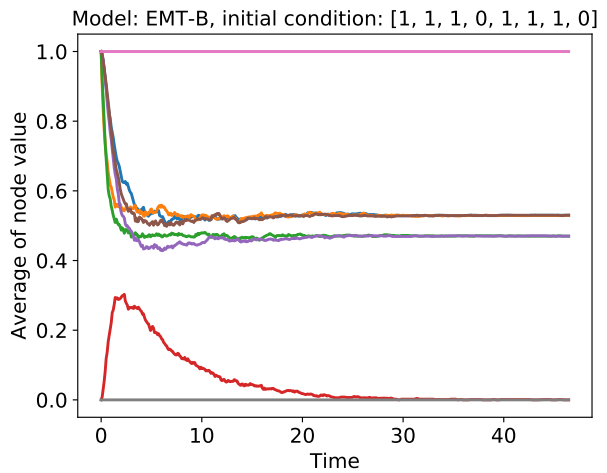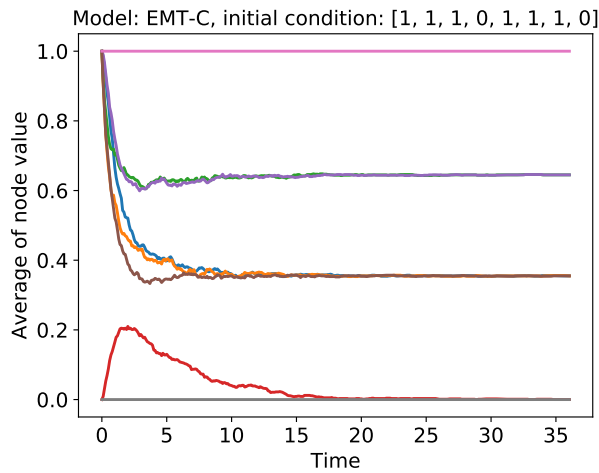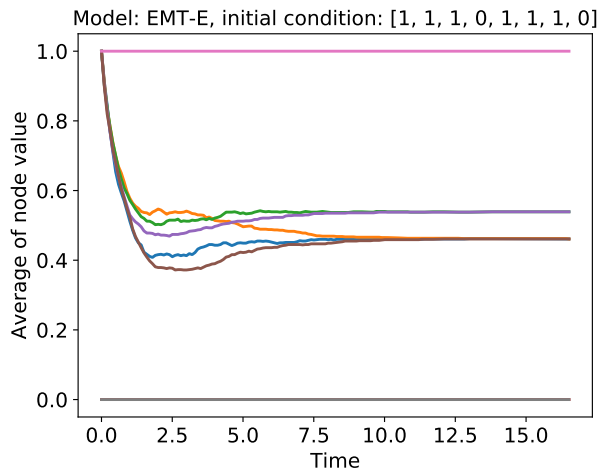

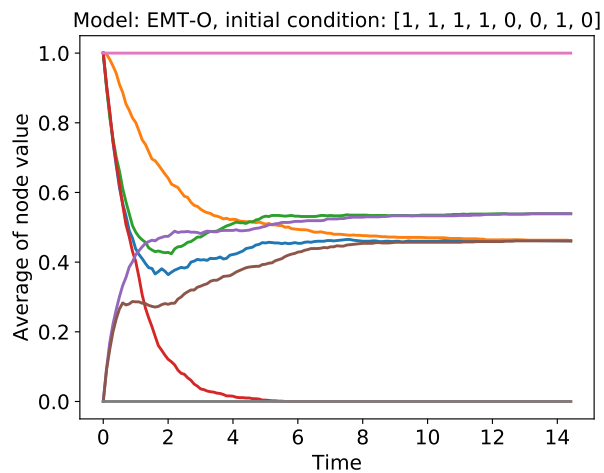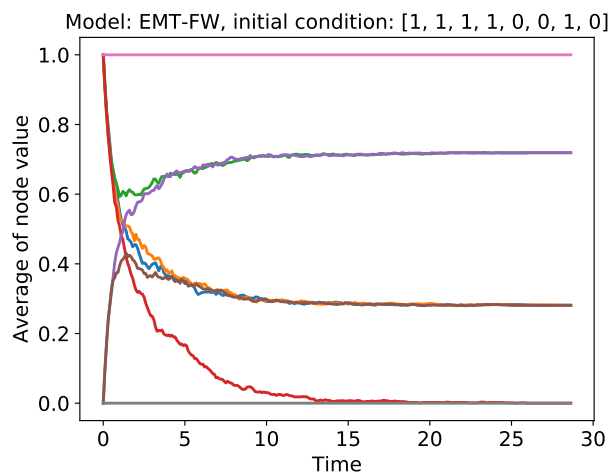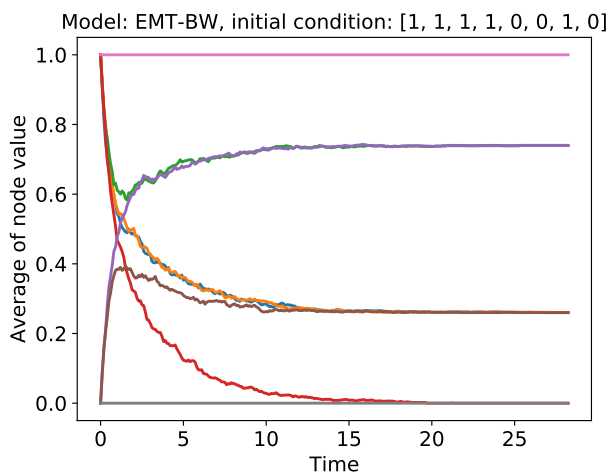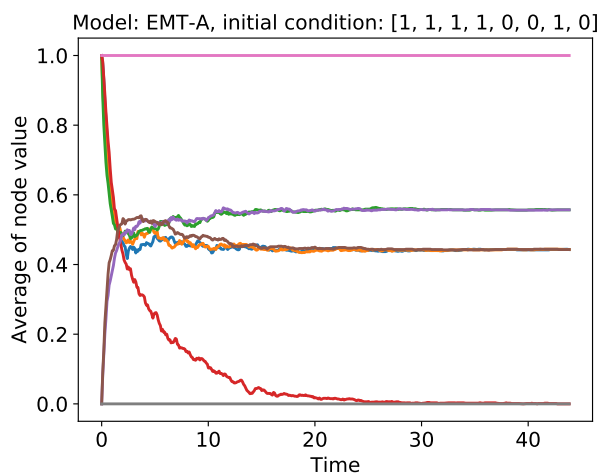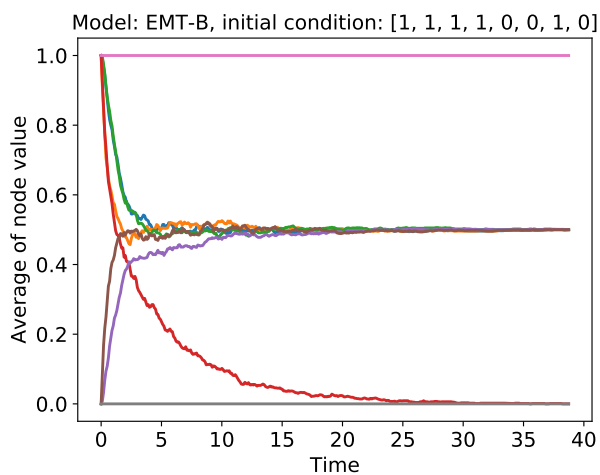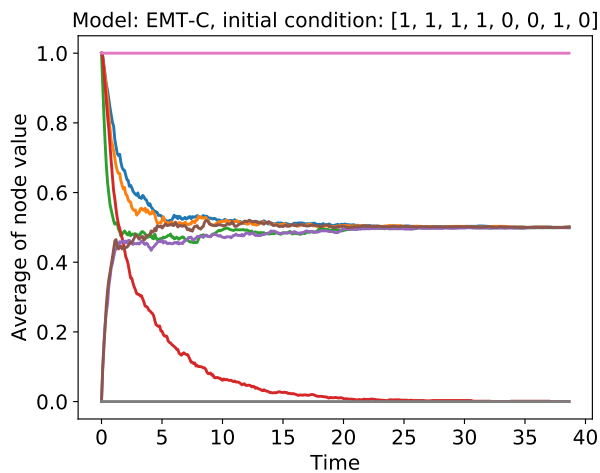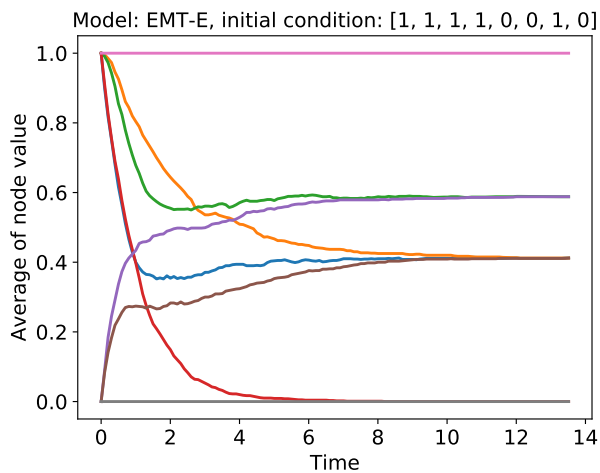

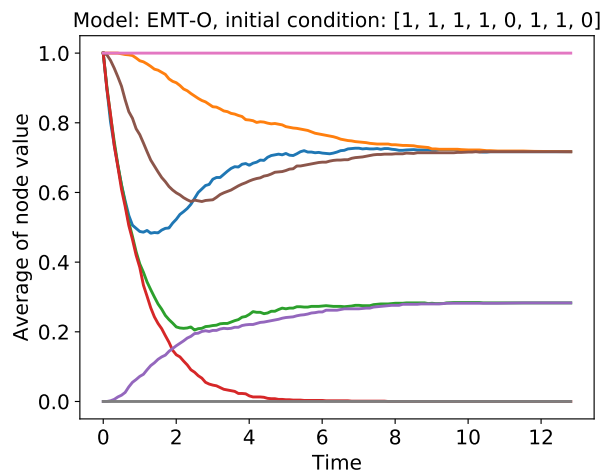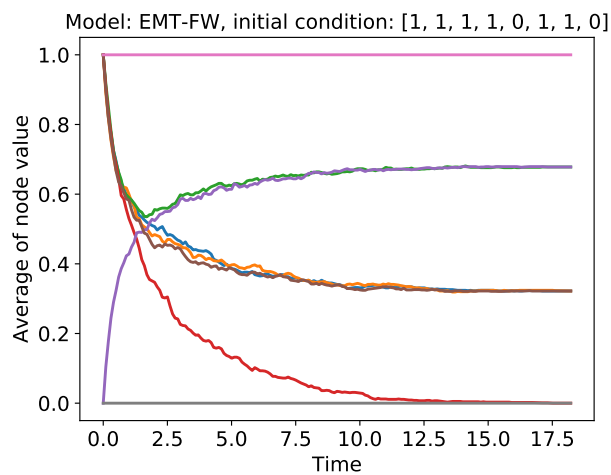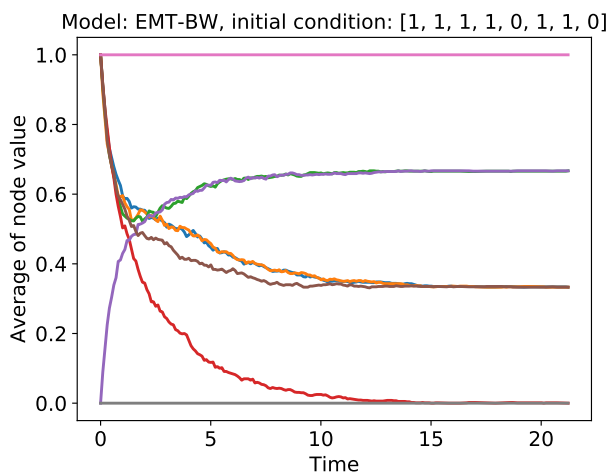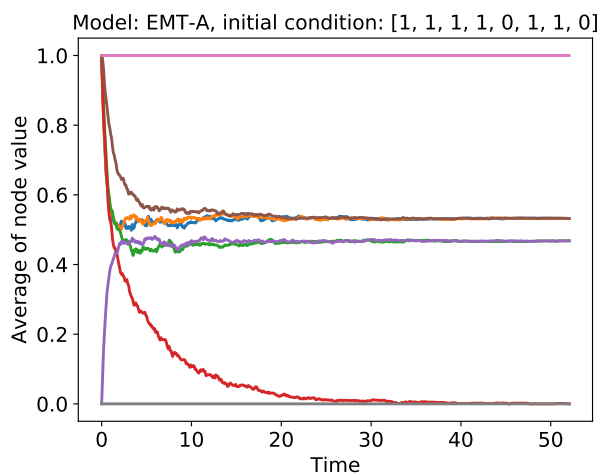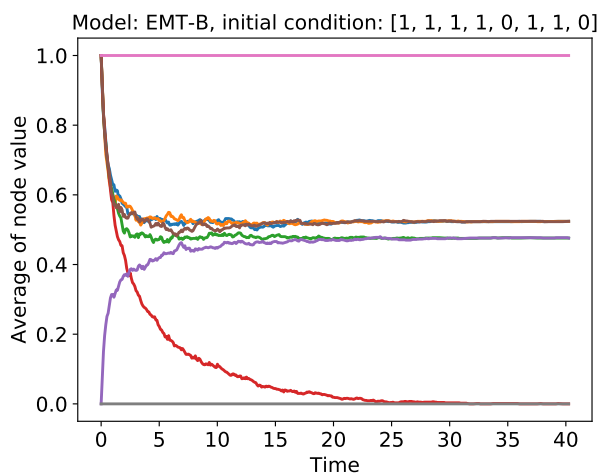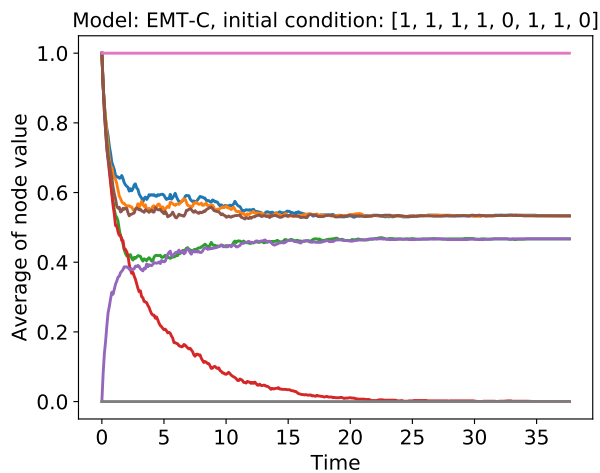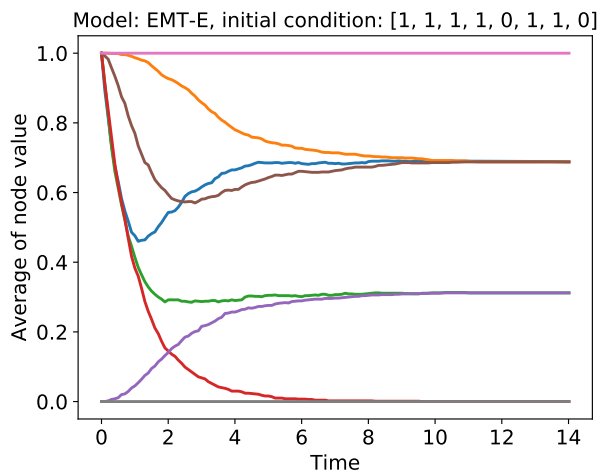

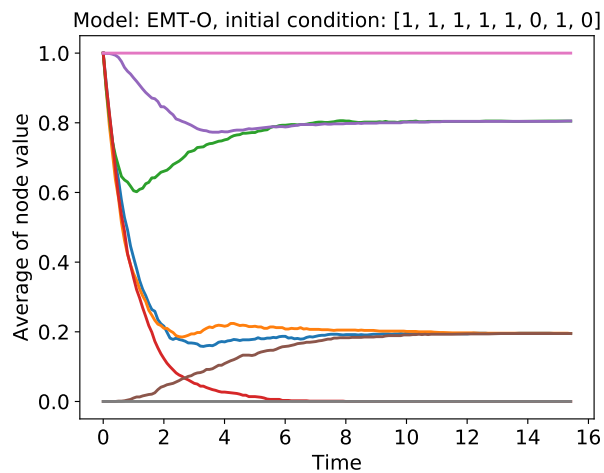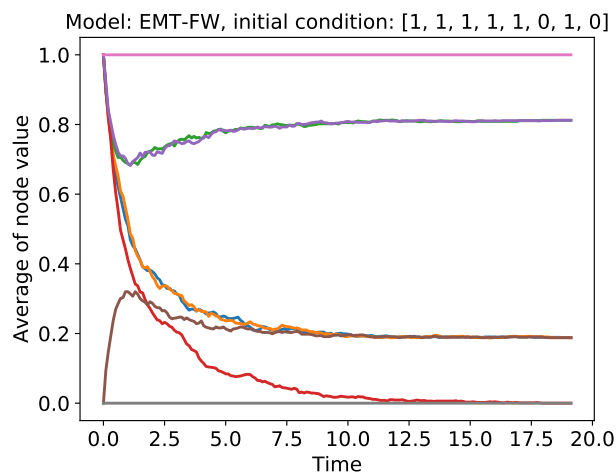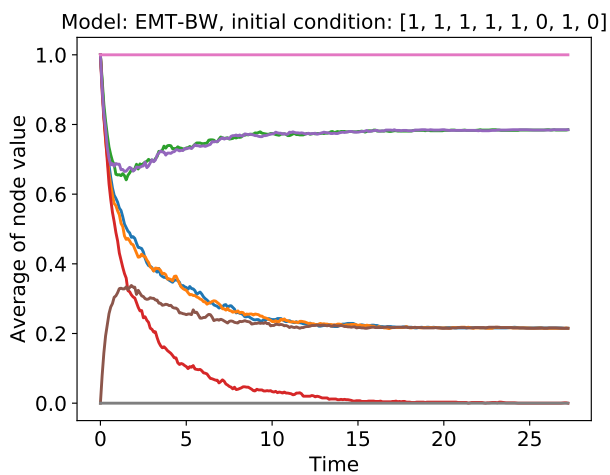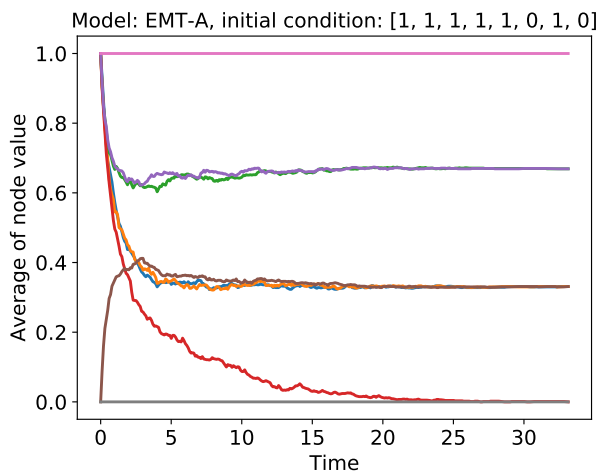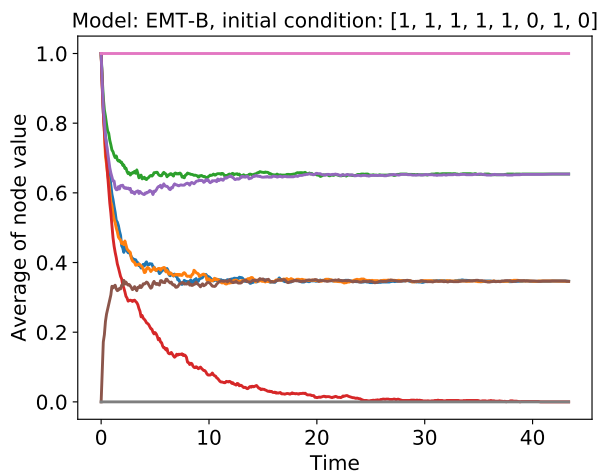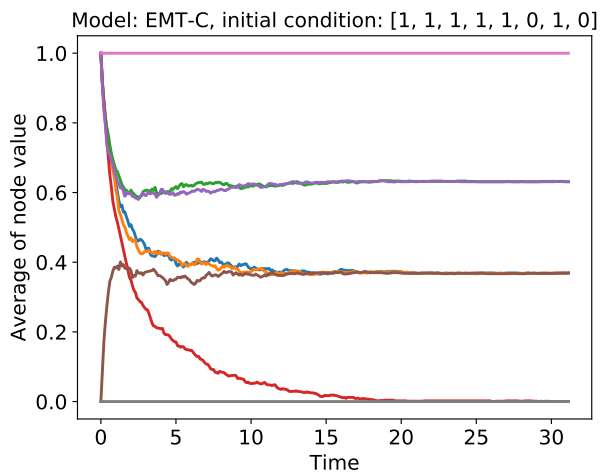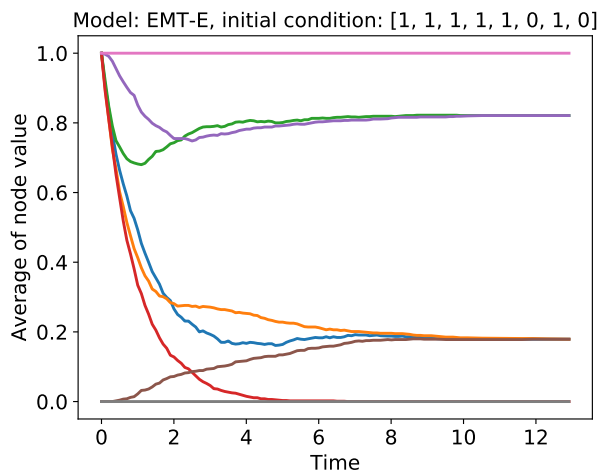

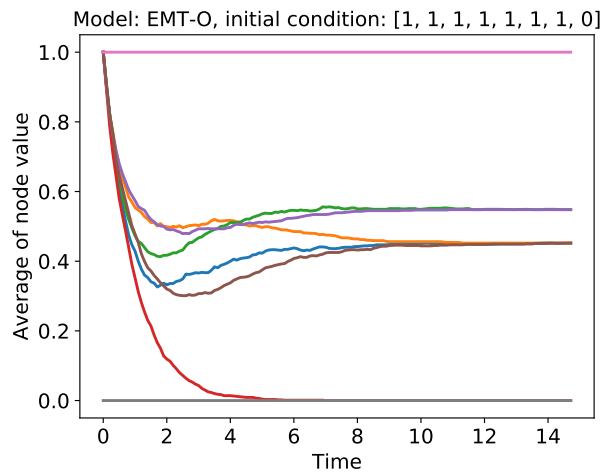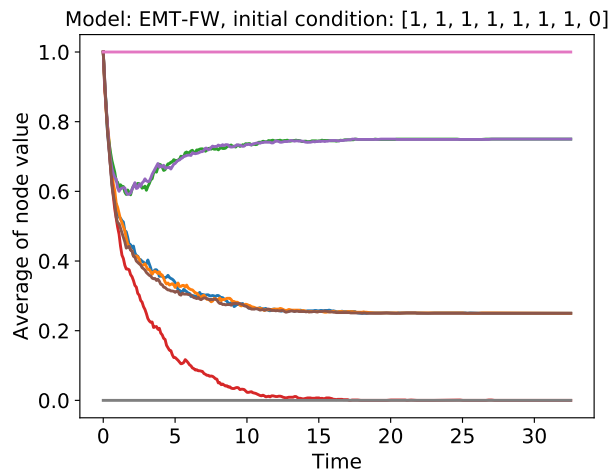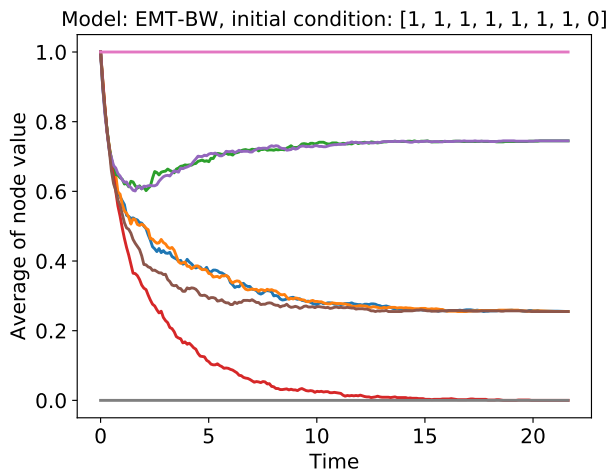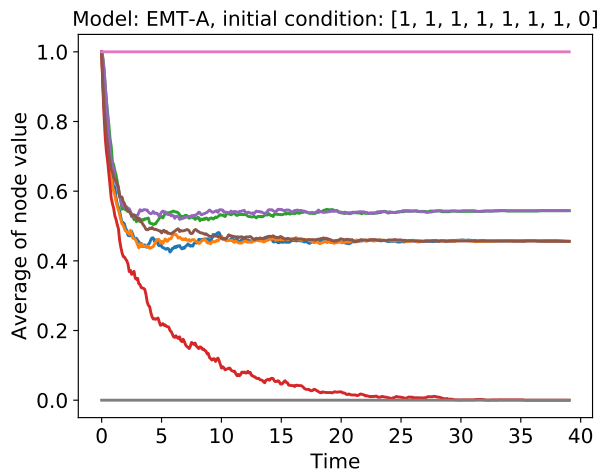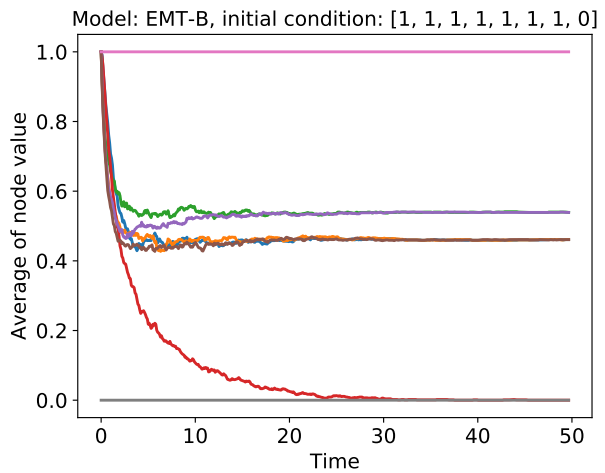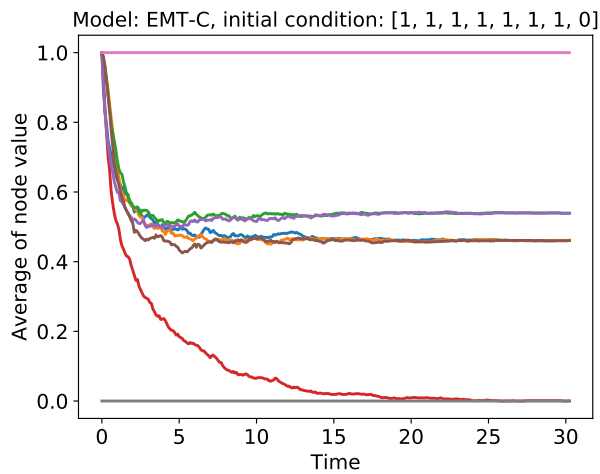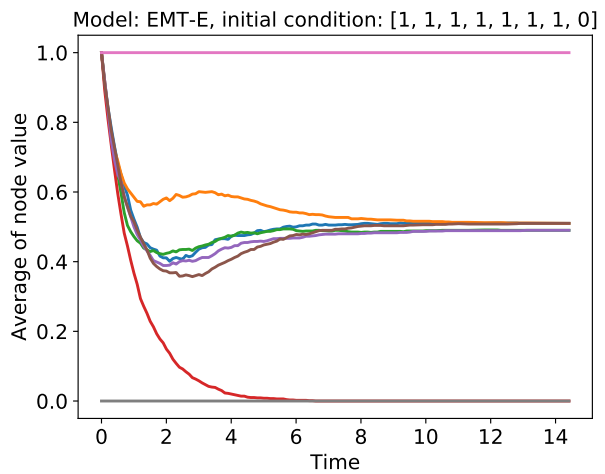

Supplement: S5 File — (PDF) [file pcbi.1009035.s005.pdf]

# Parameter set (ECM, DNAdam) = (1, 1):

Steady states: (0, 0, 1, 0, 1, 0, 1, 1), (1, 1, 0, 0, 0, 1, 1, 1)

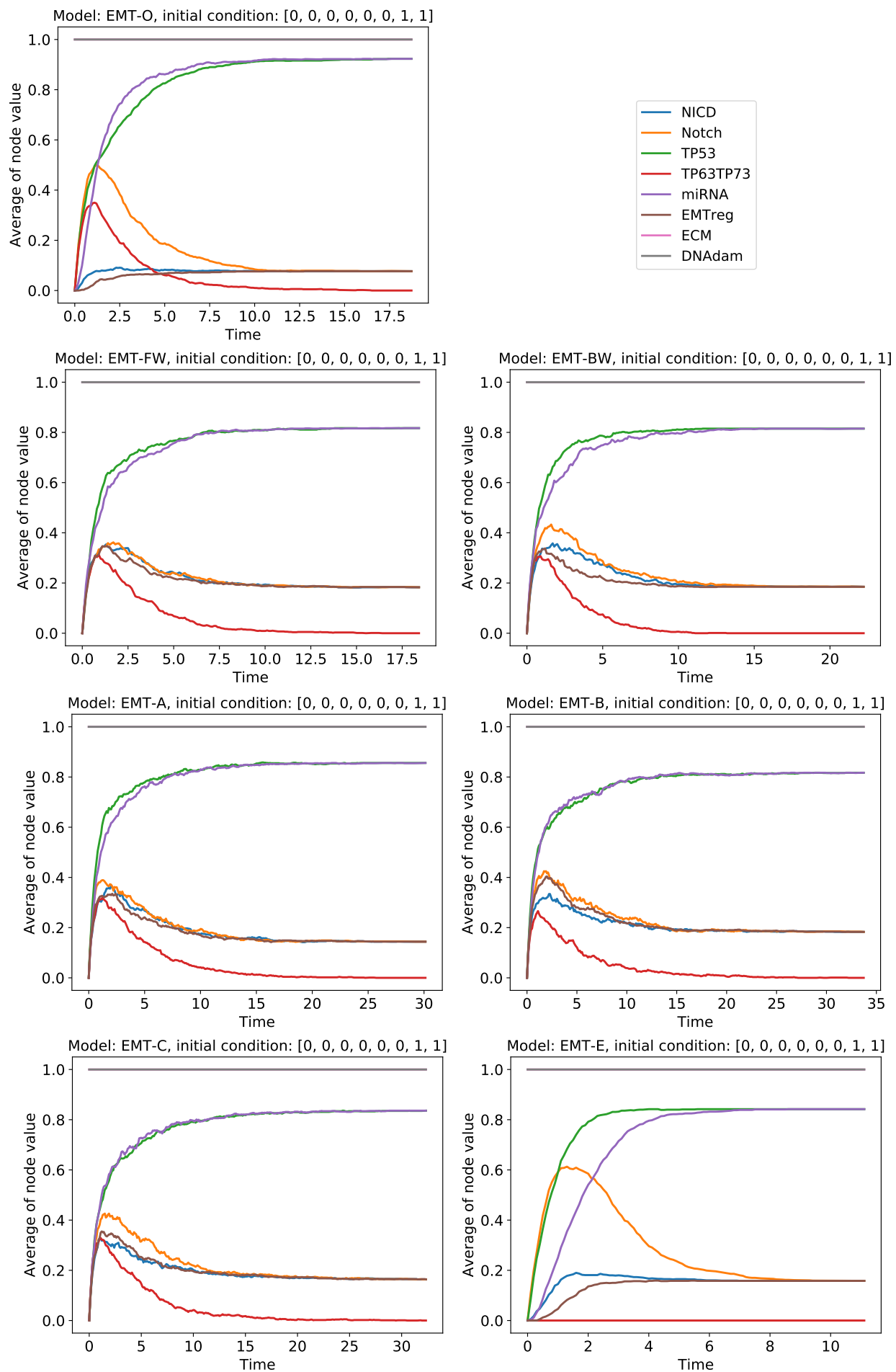

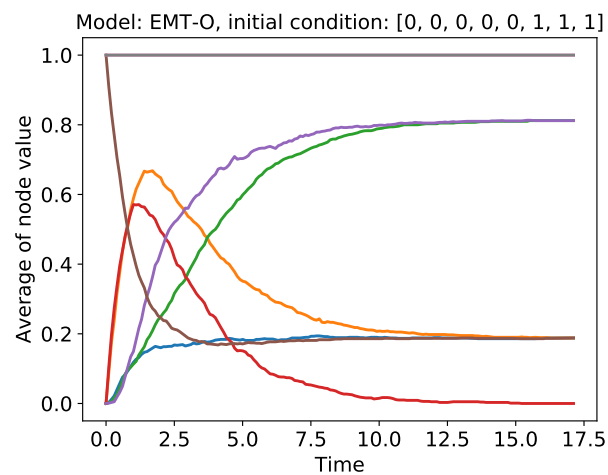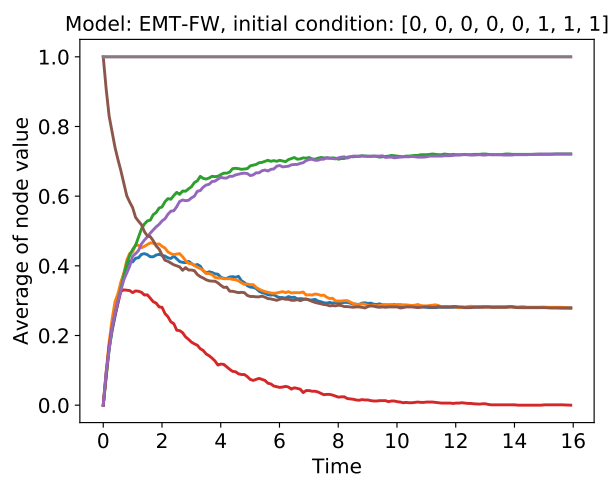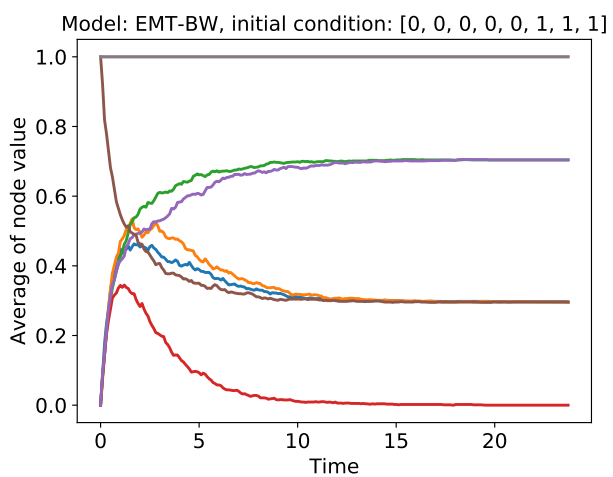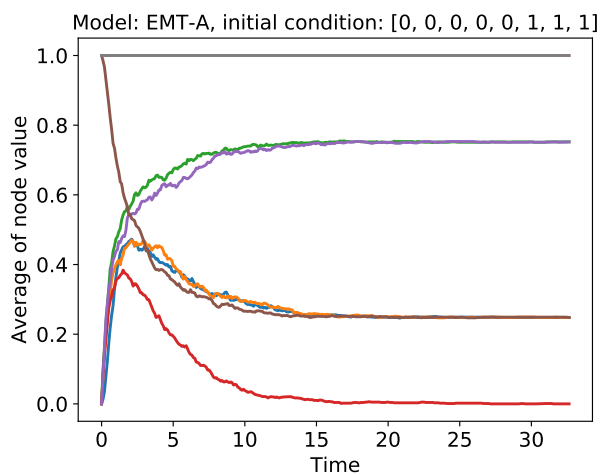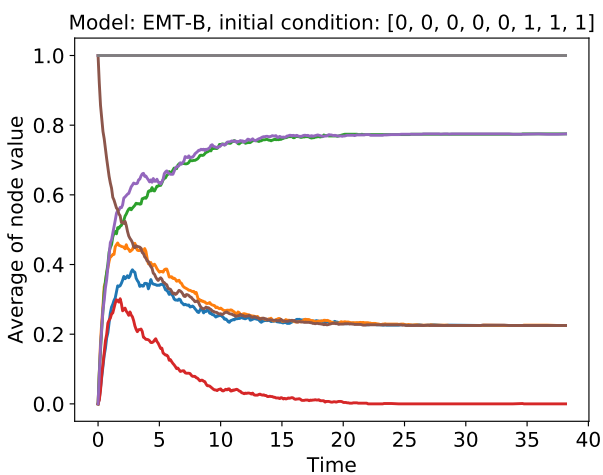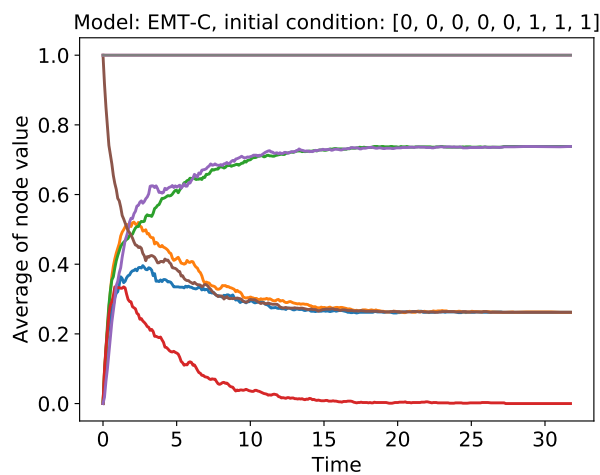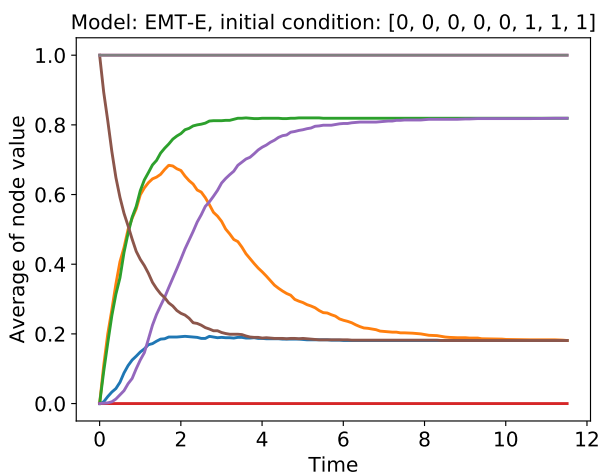

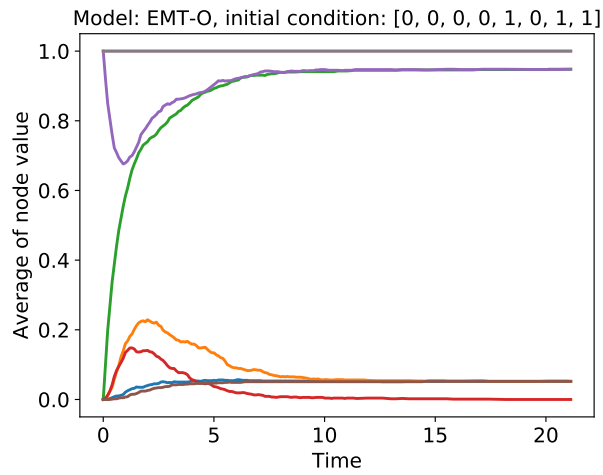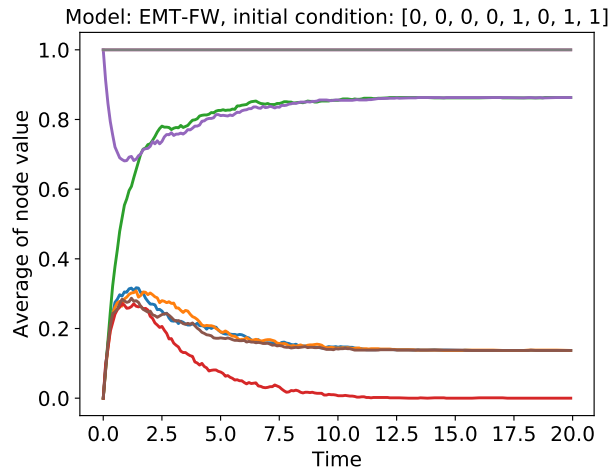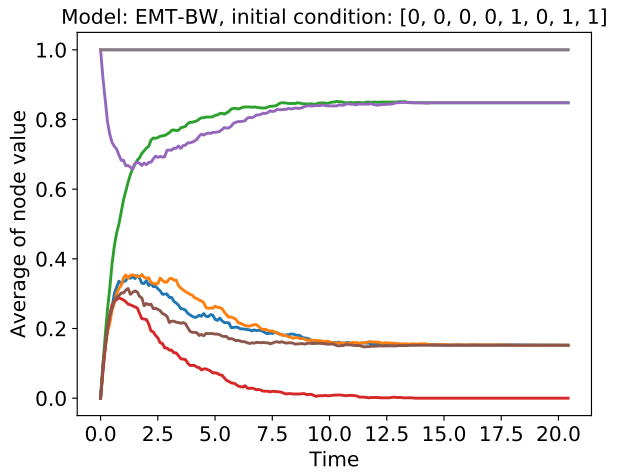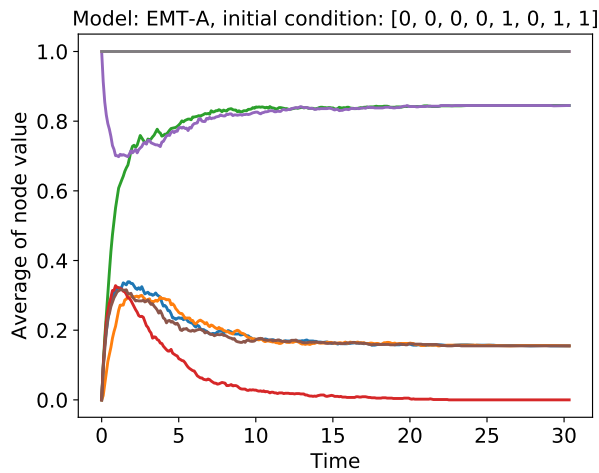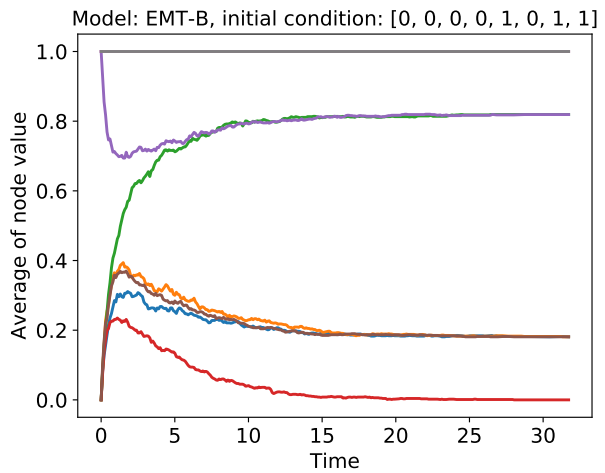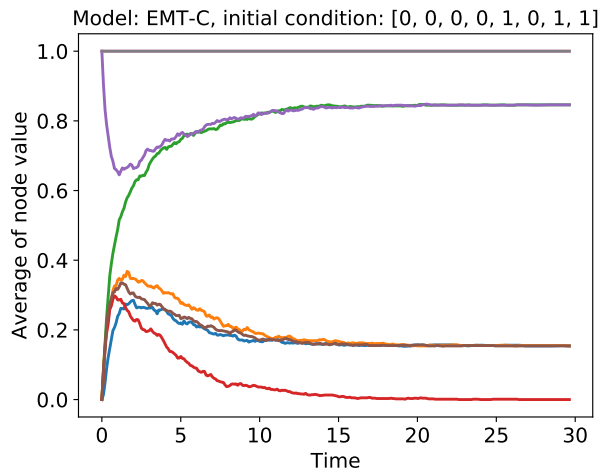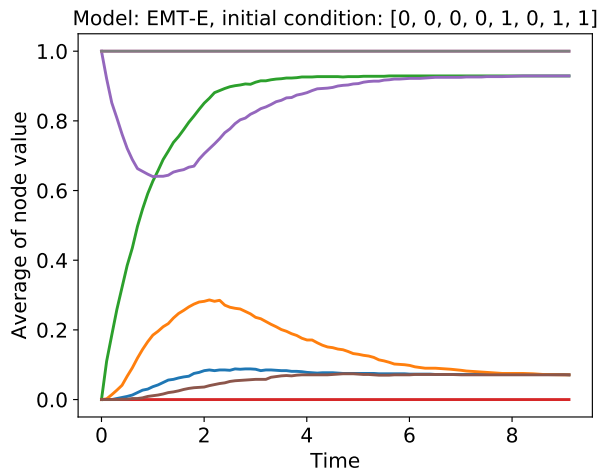

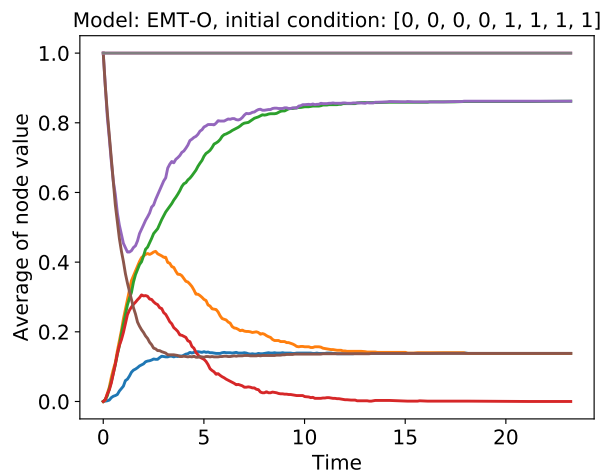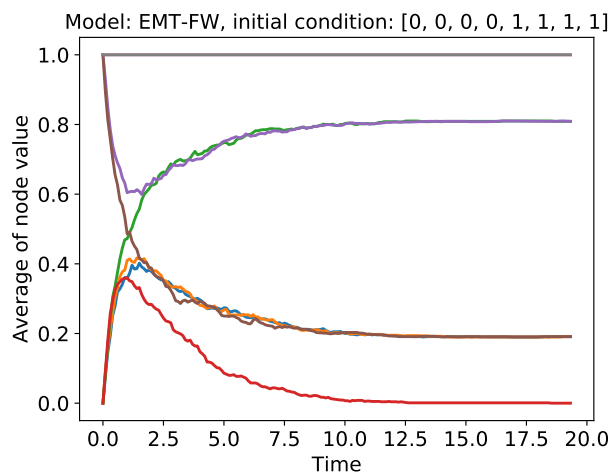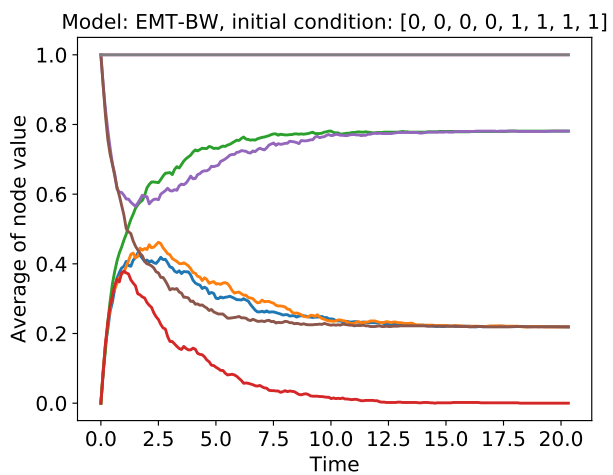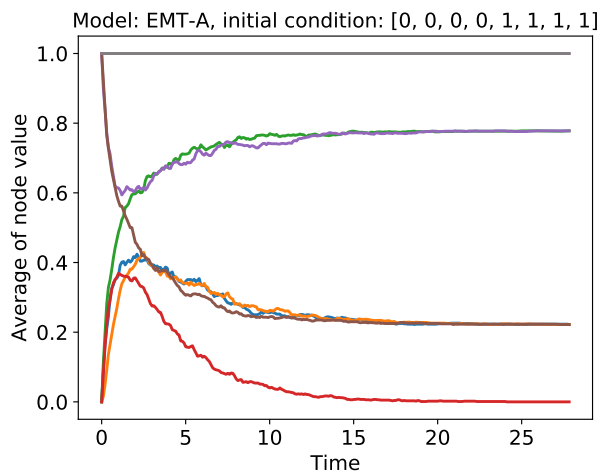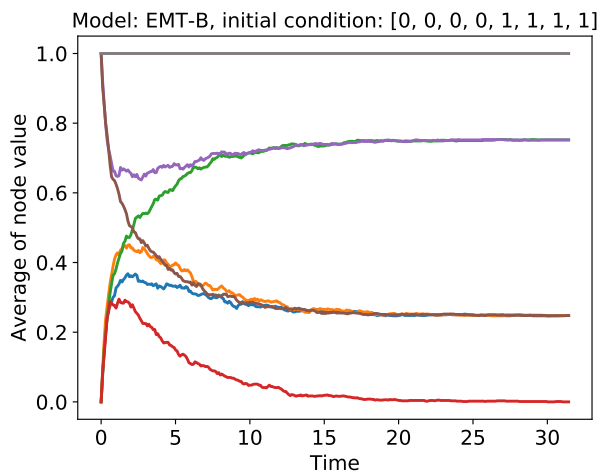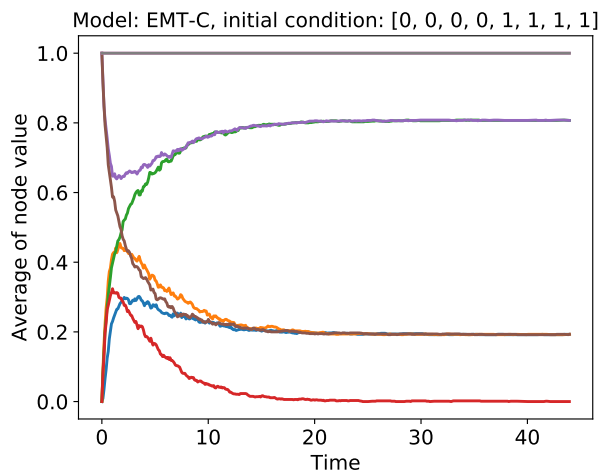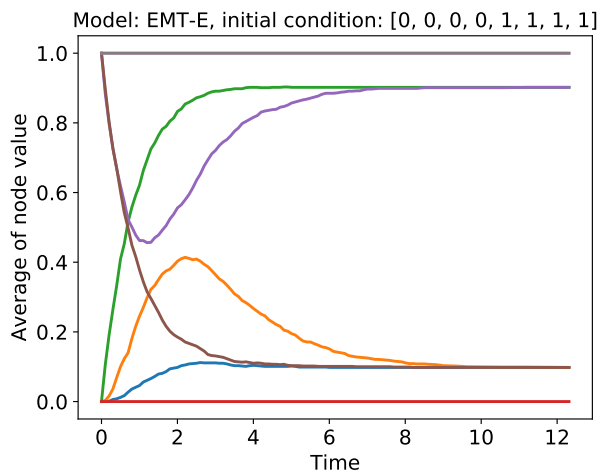

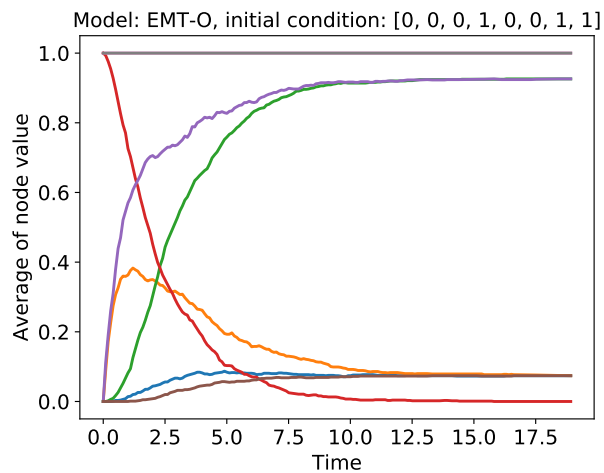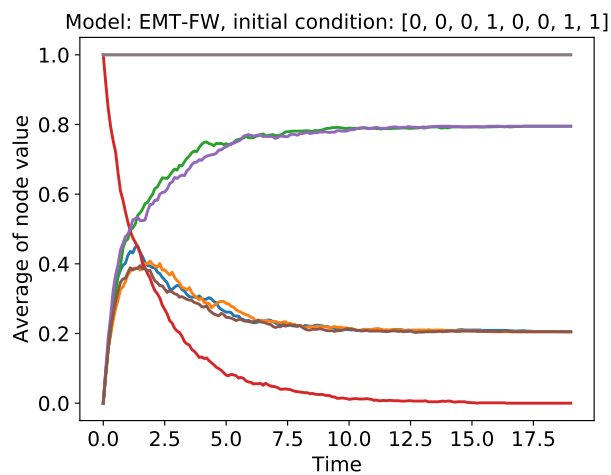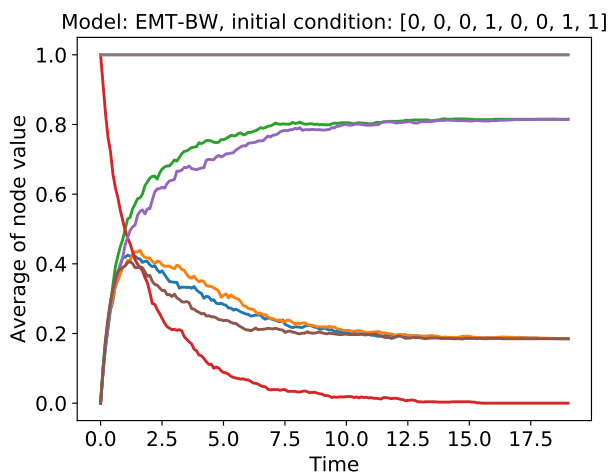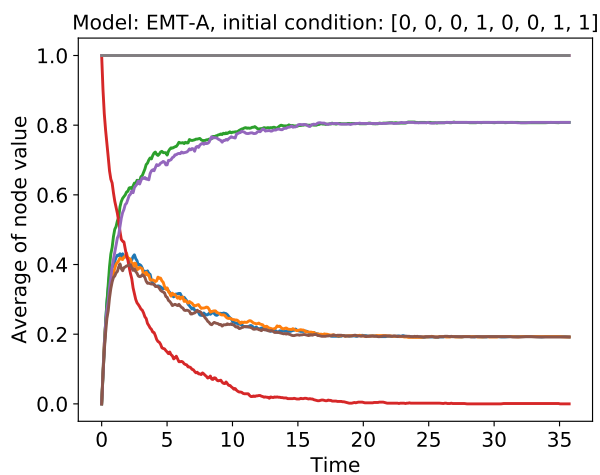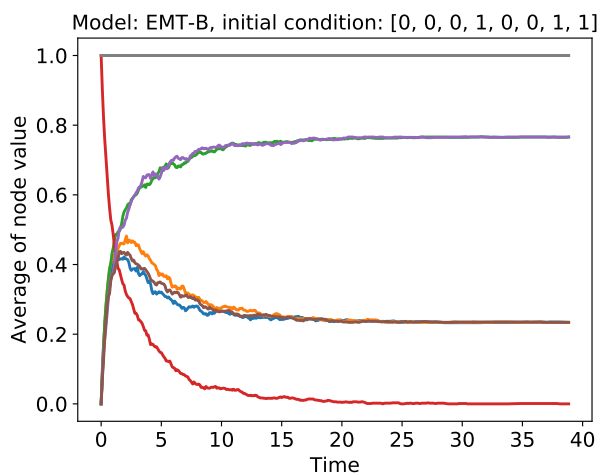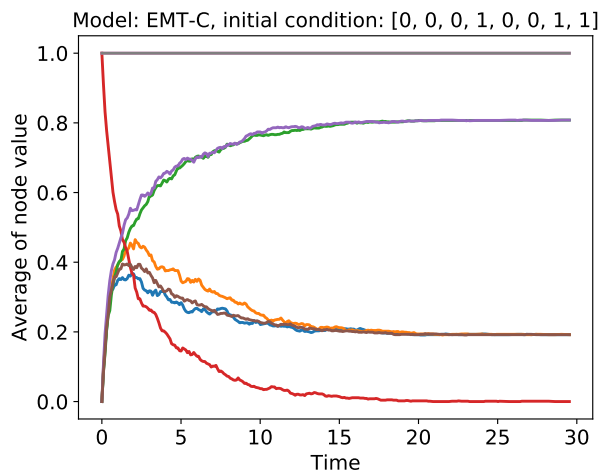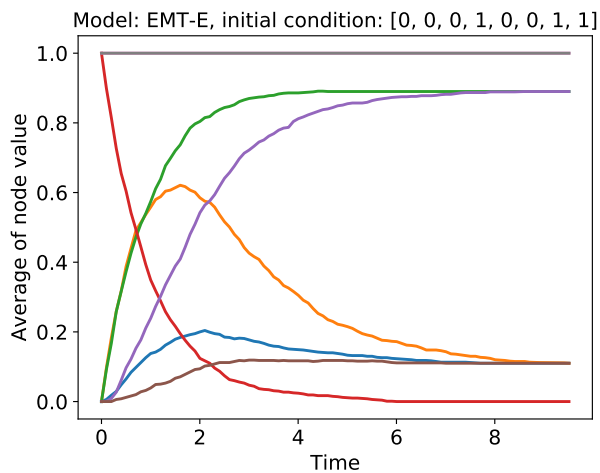

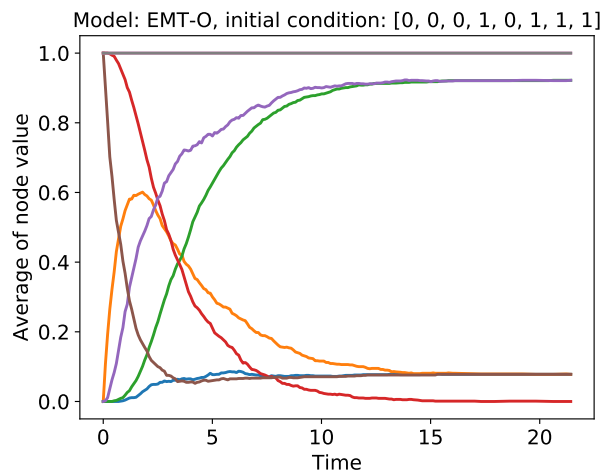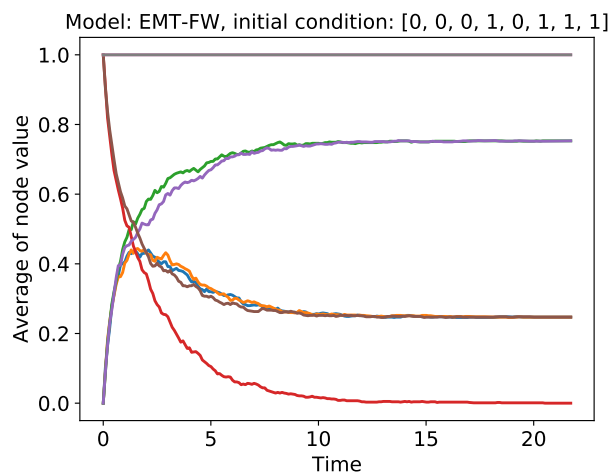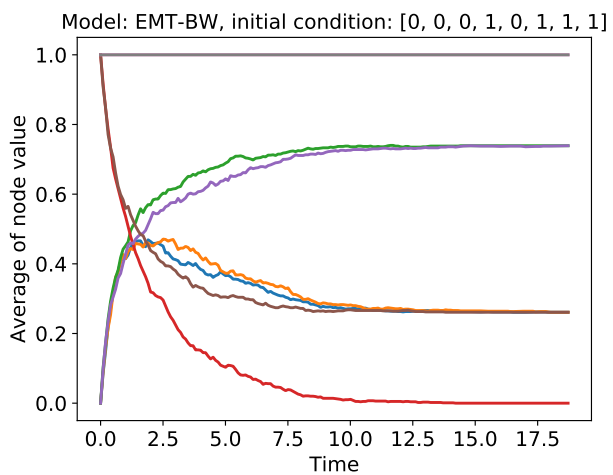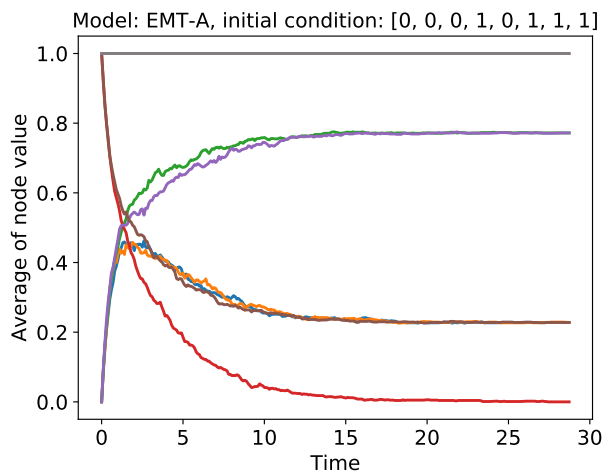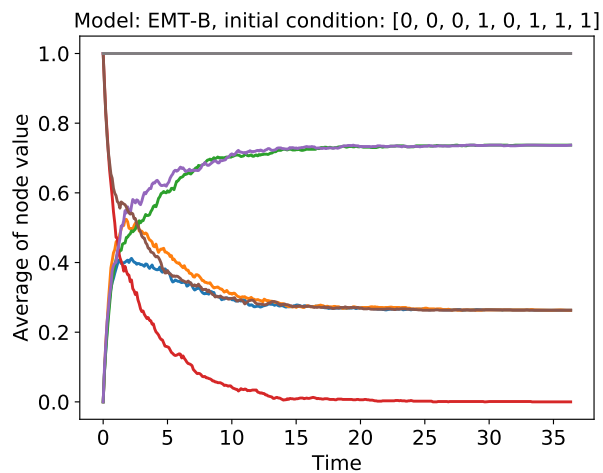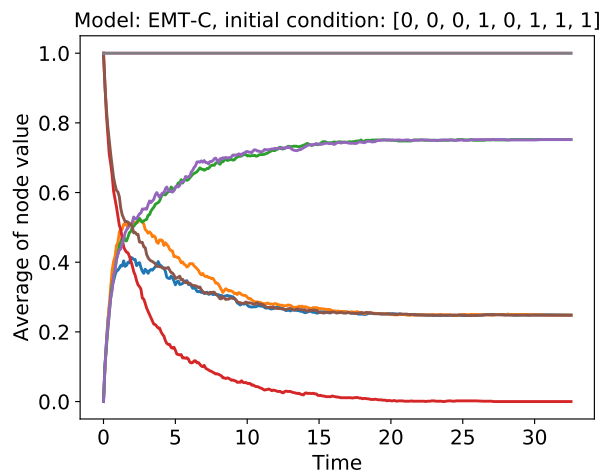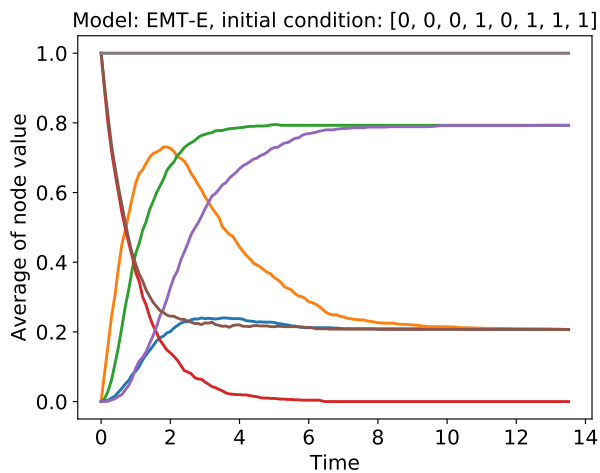

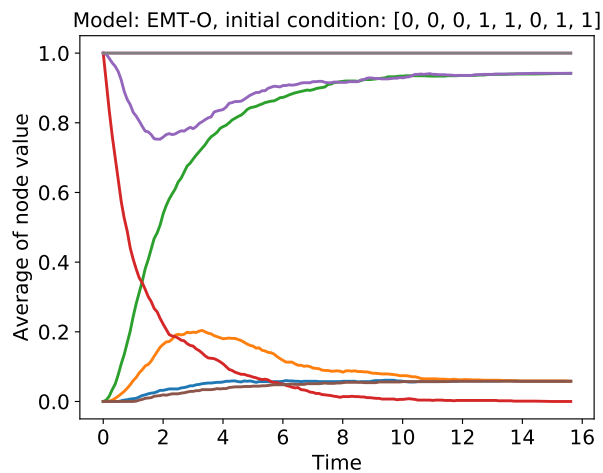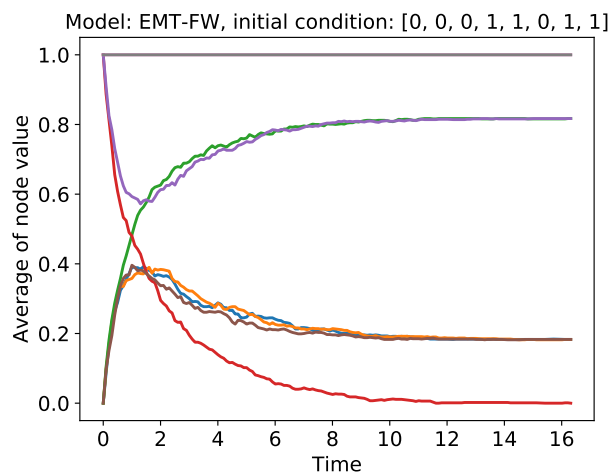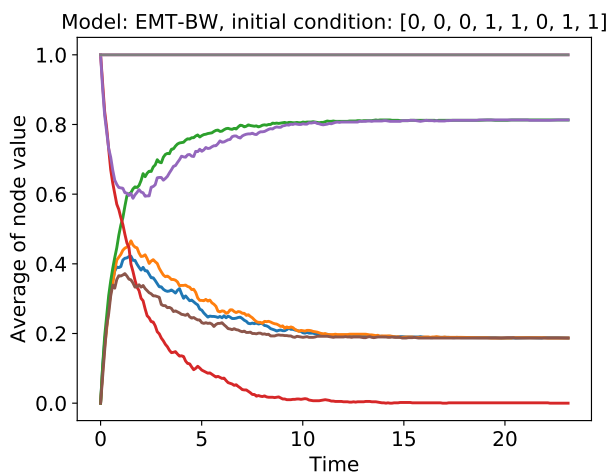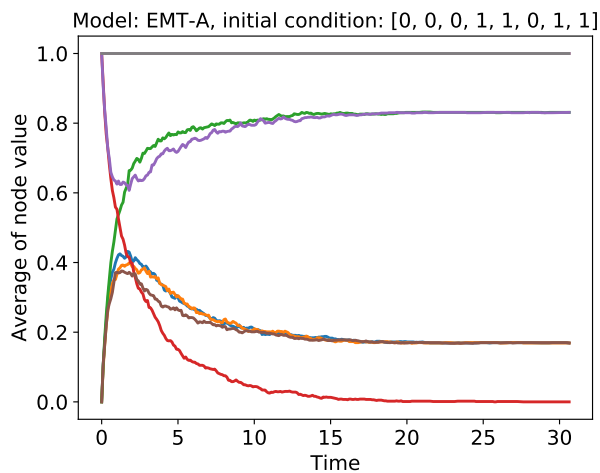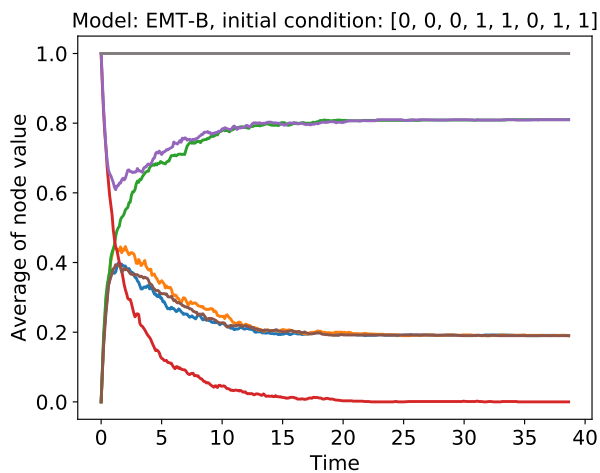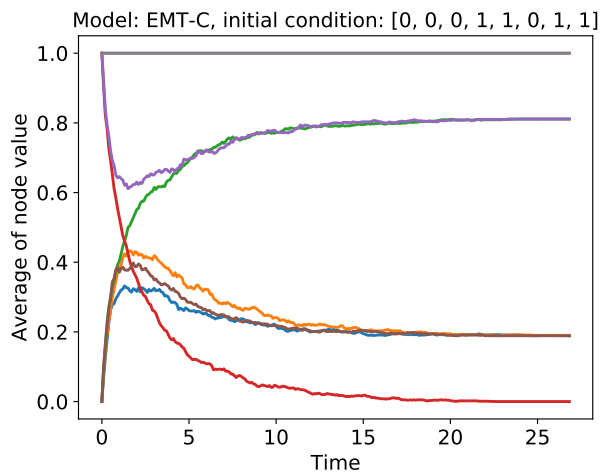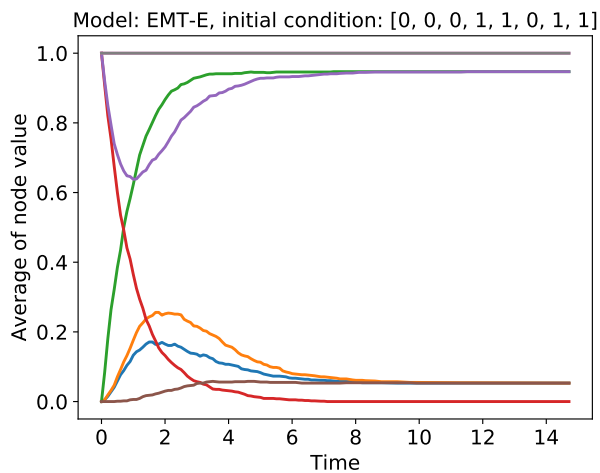

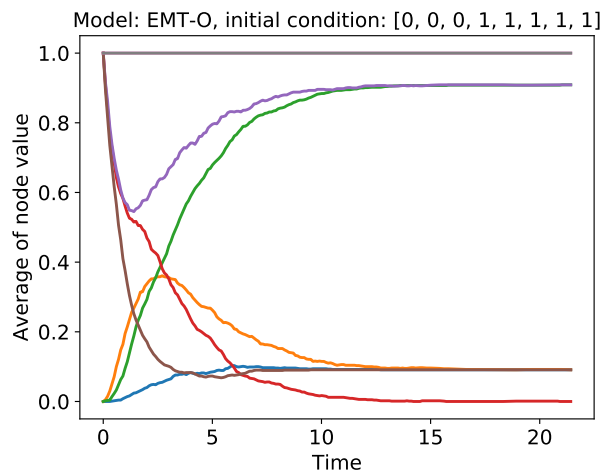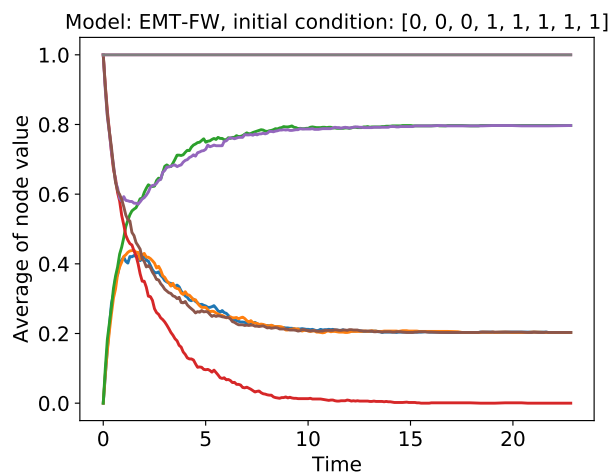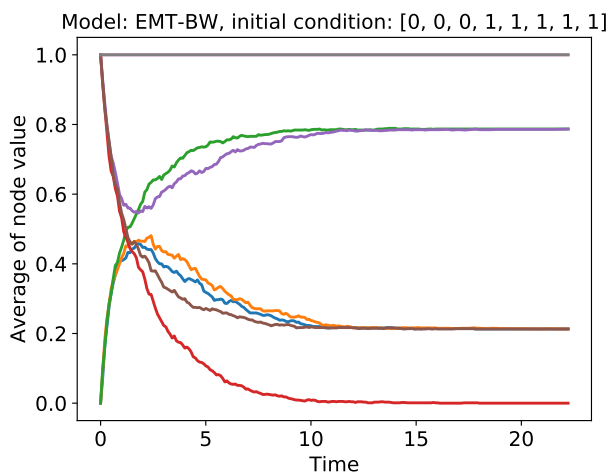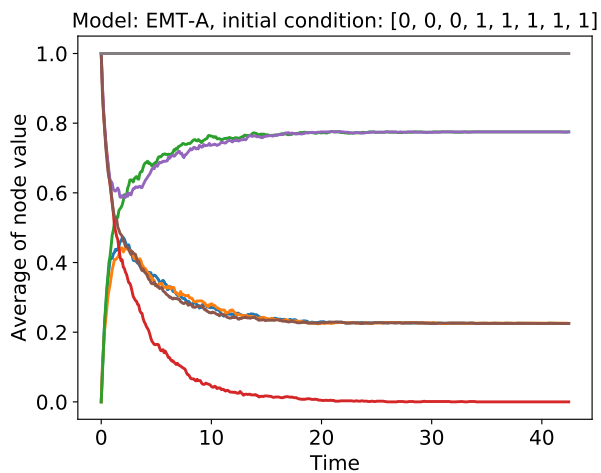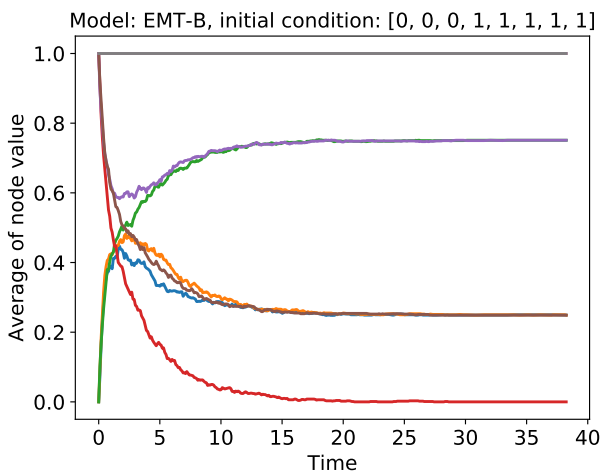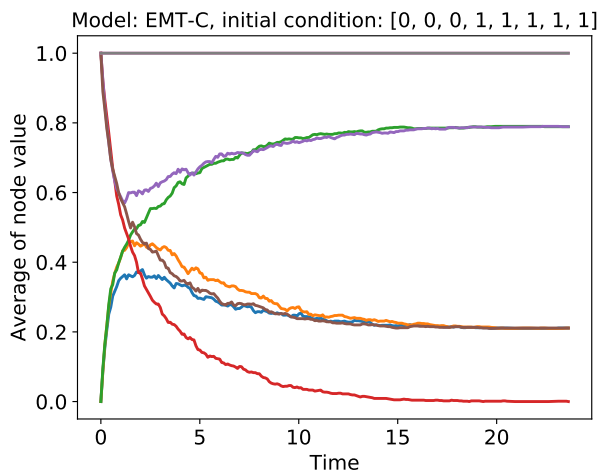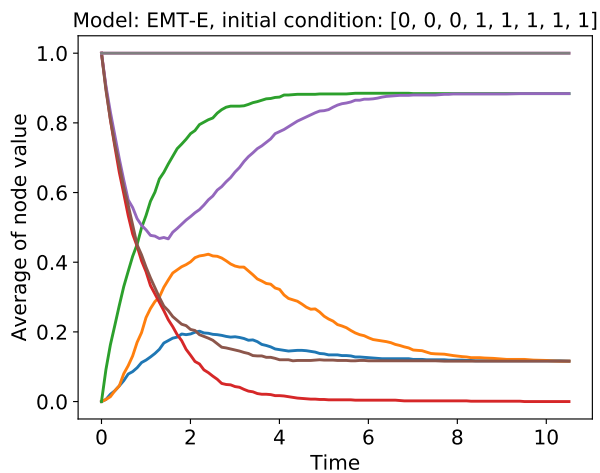

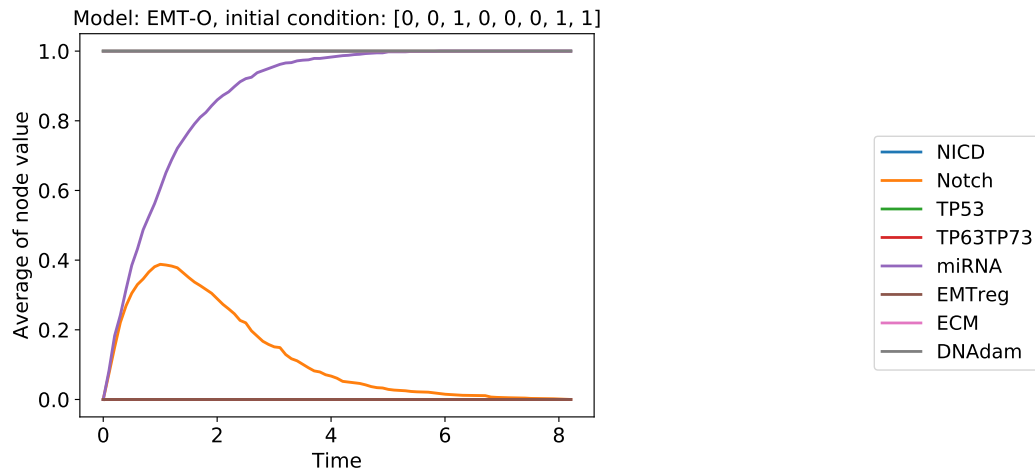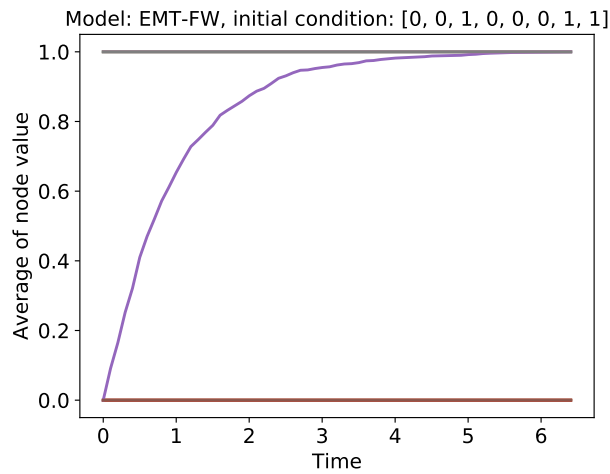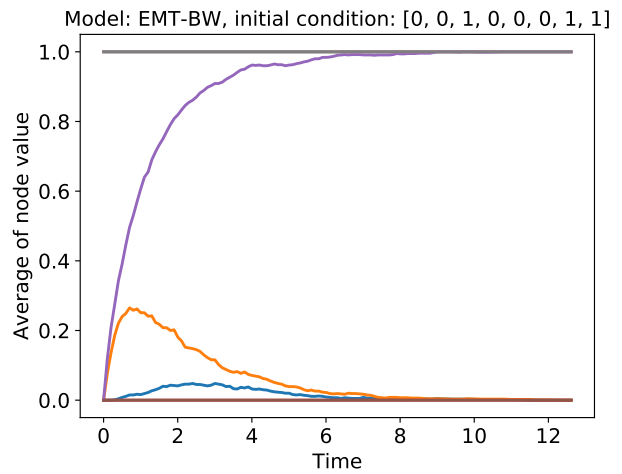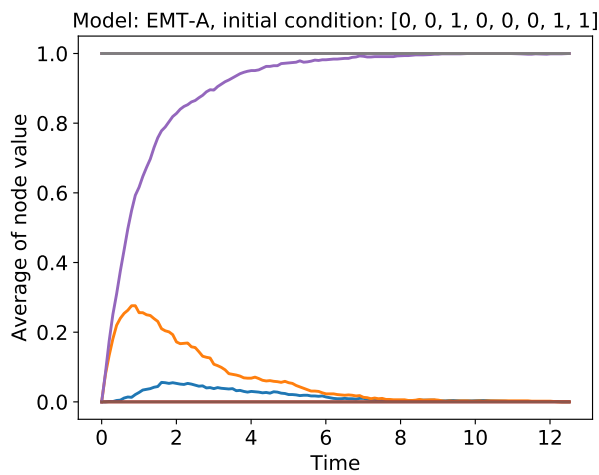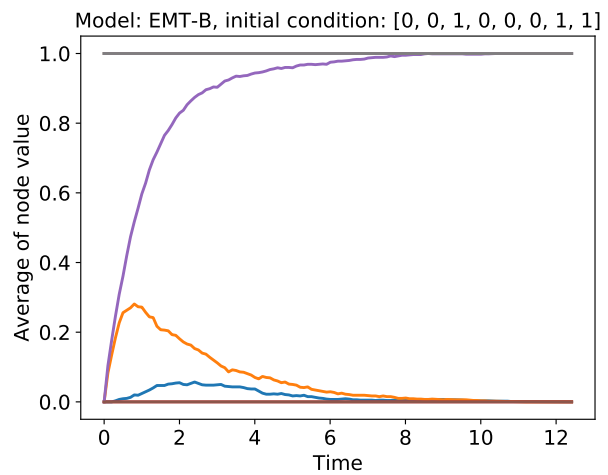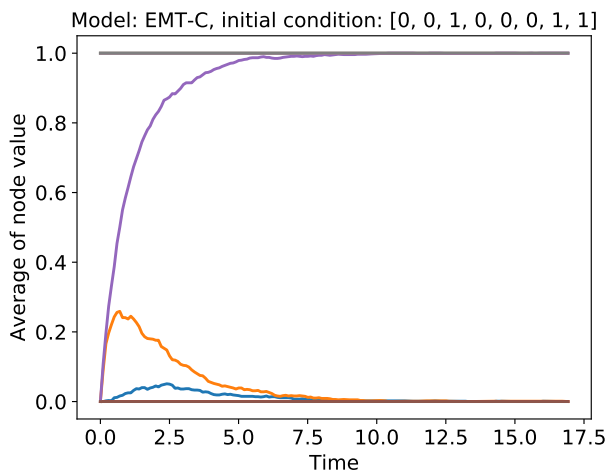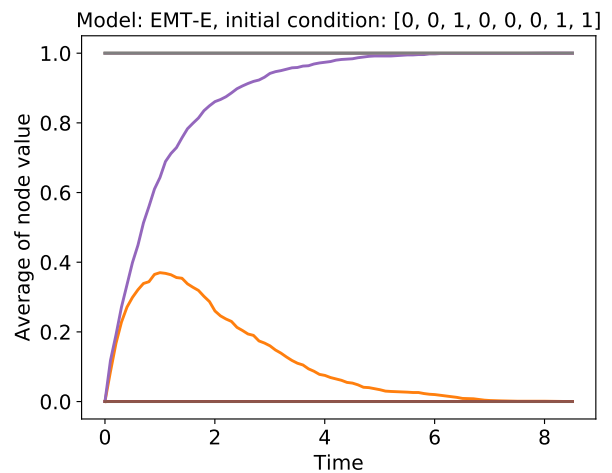

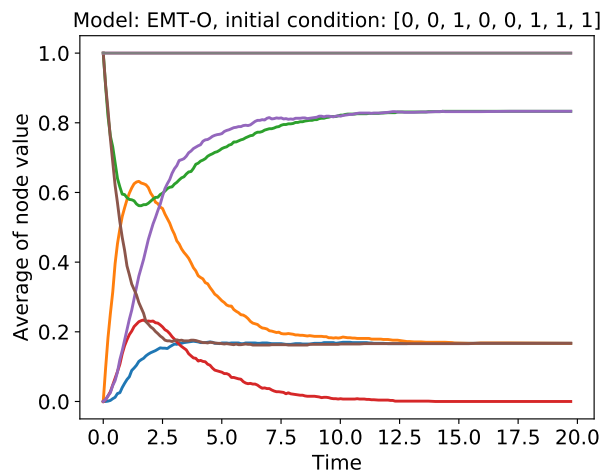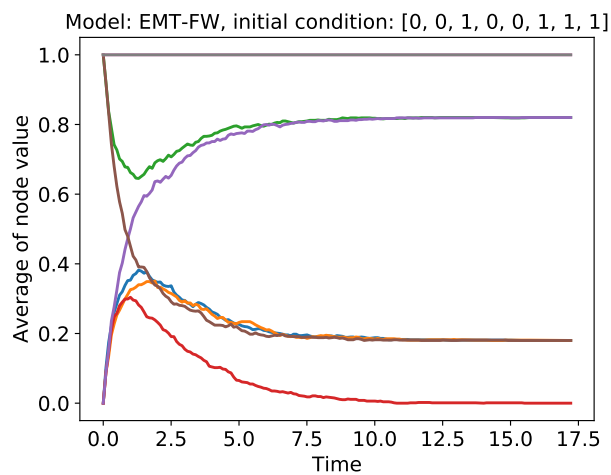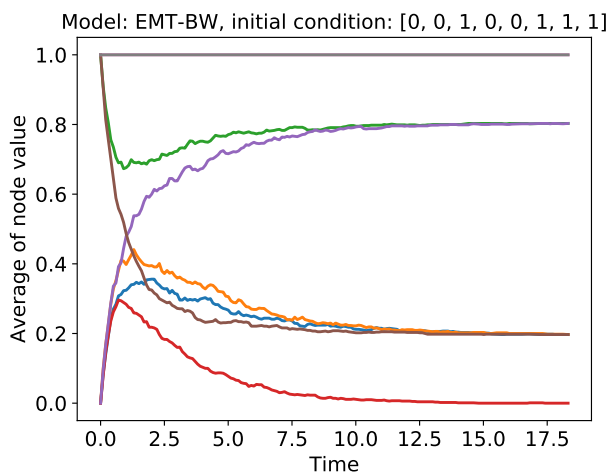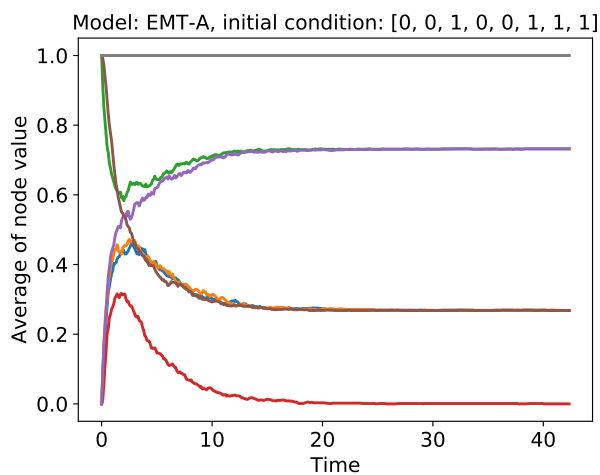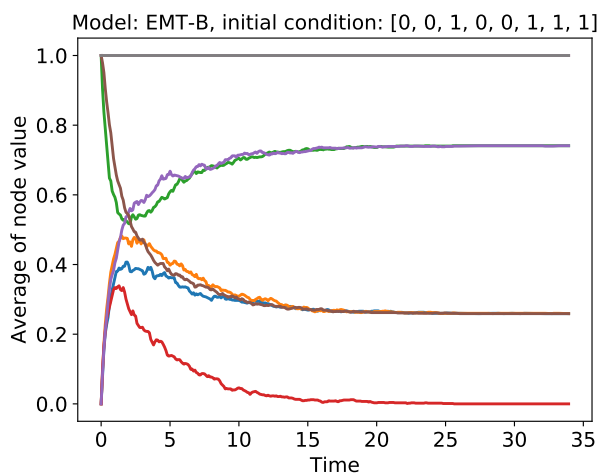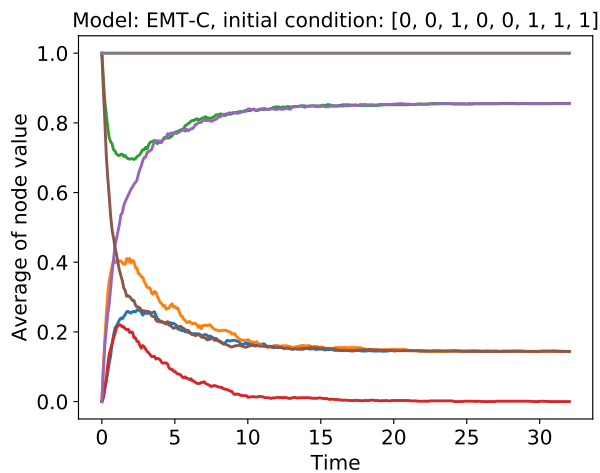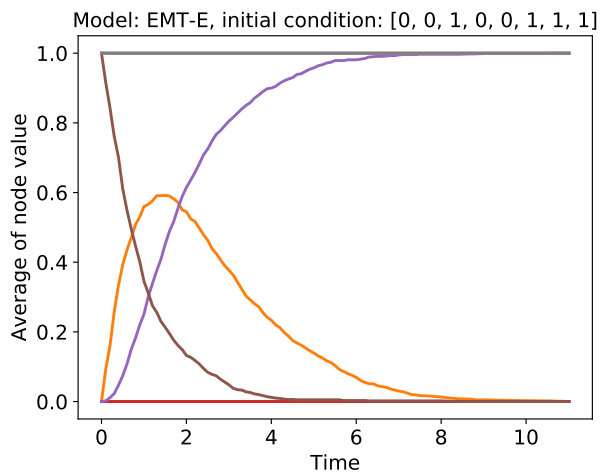

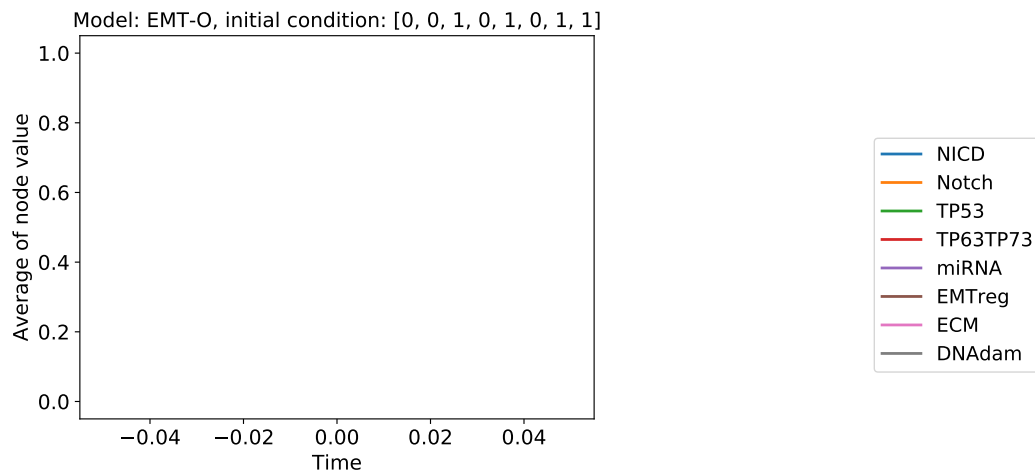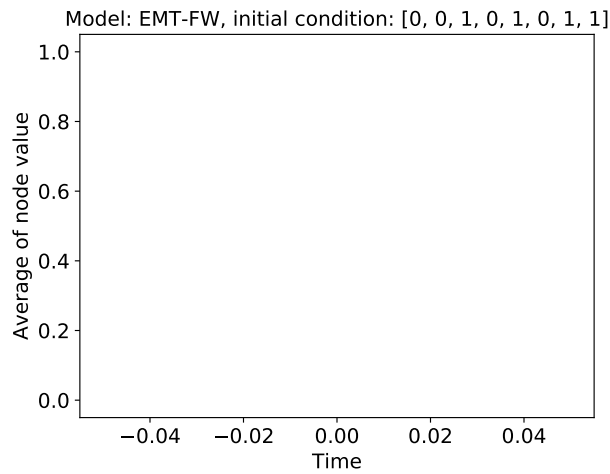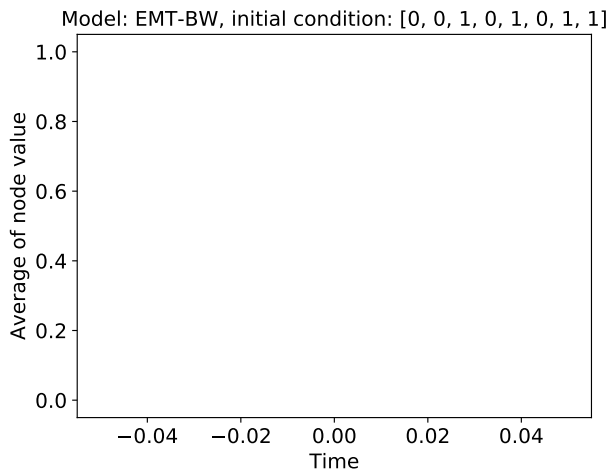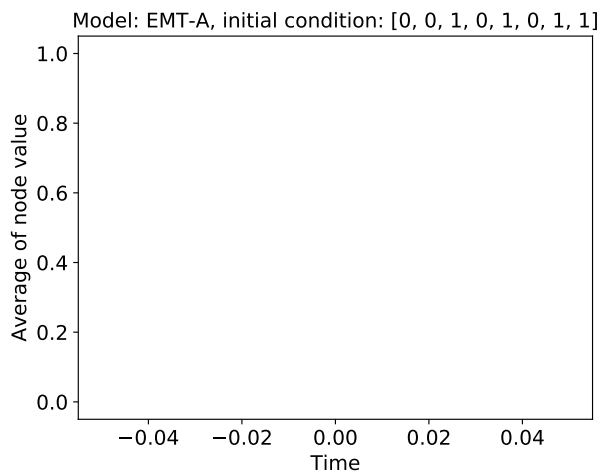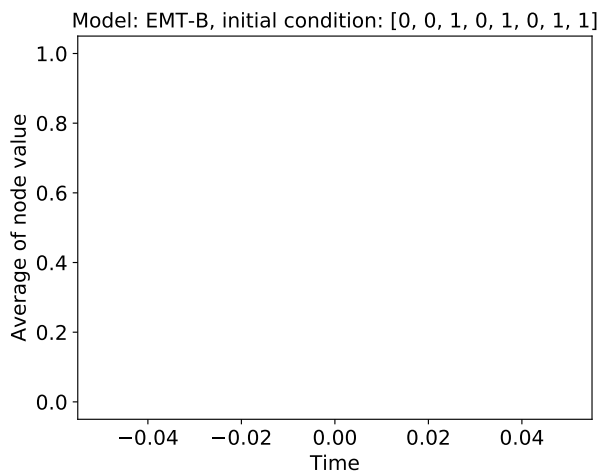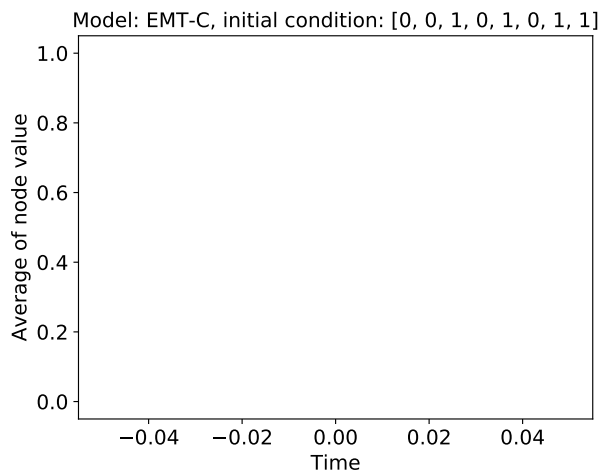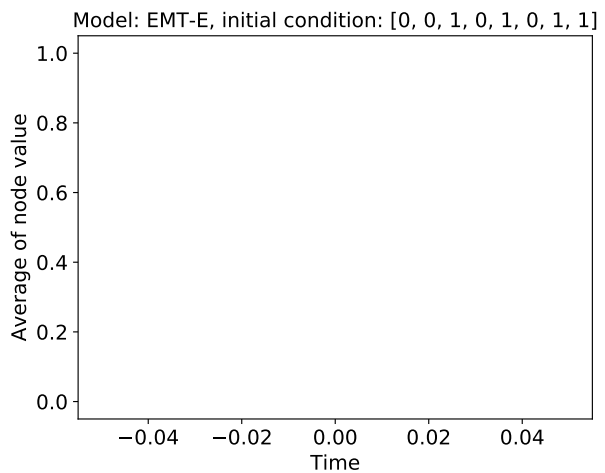

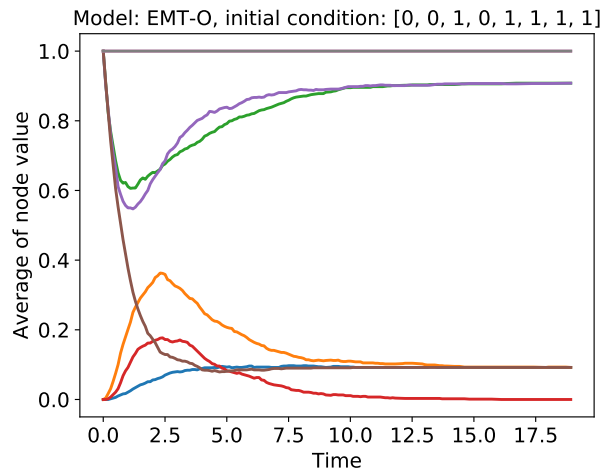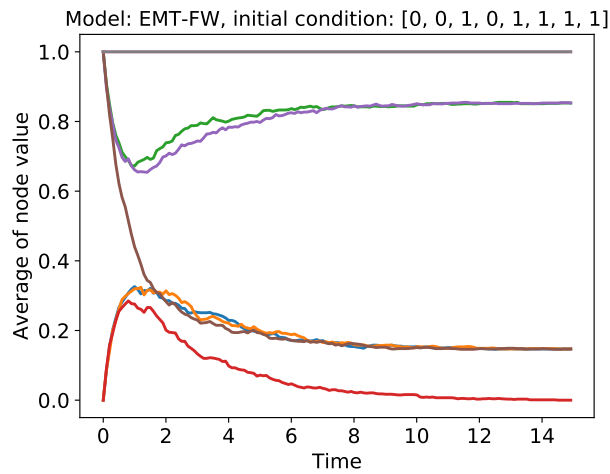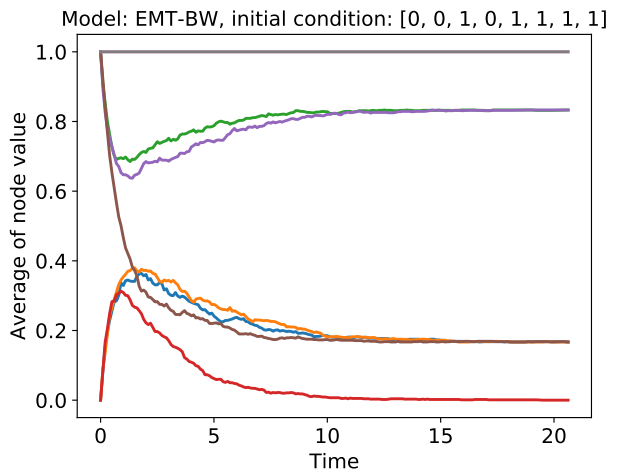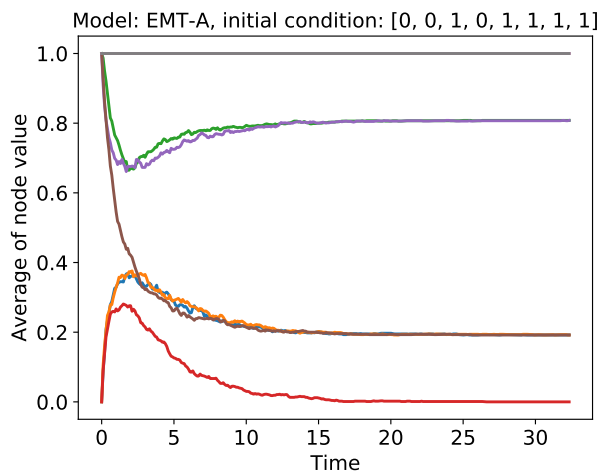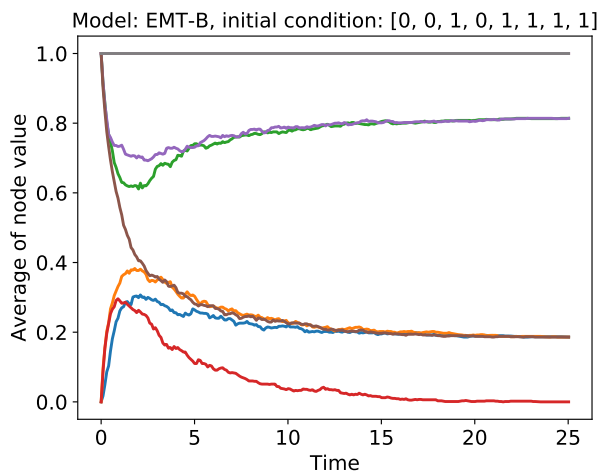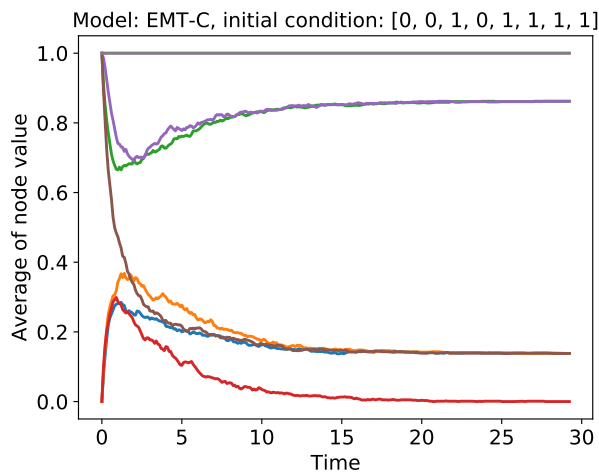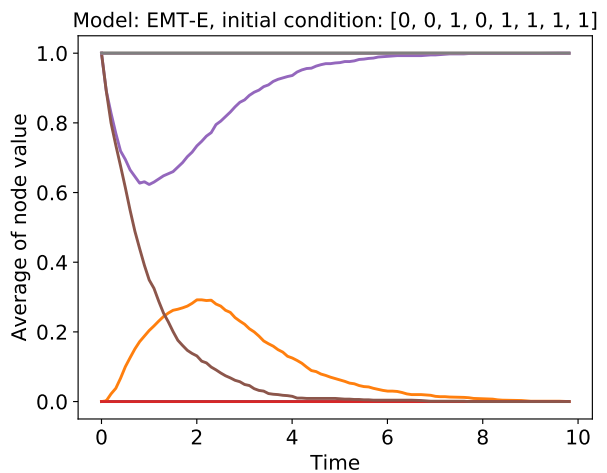

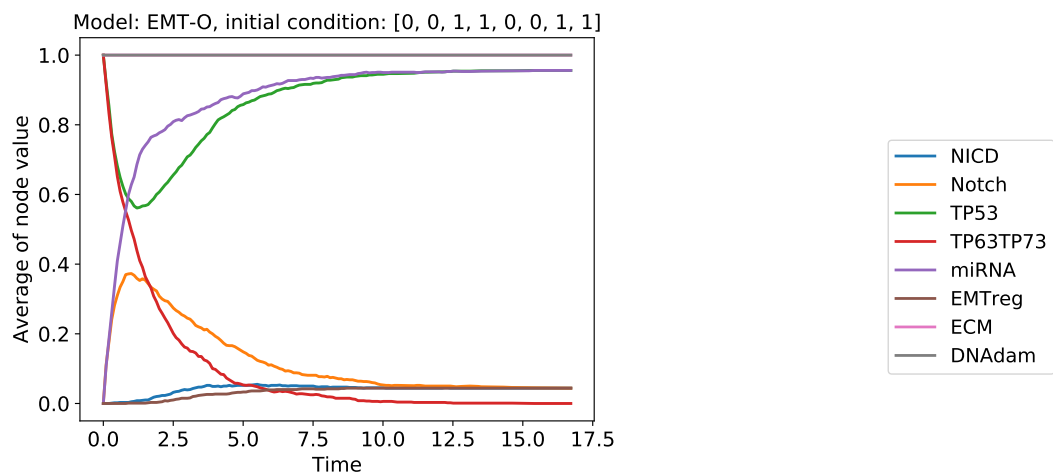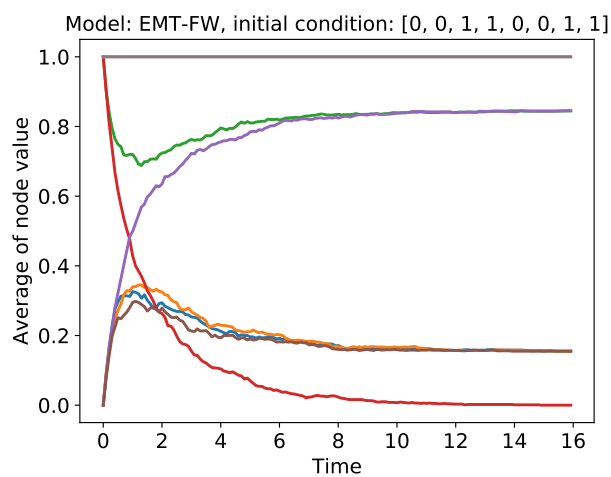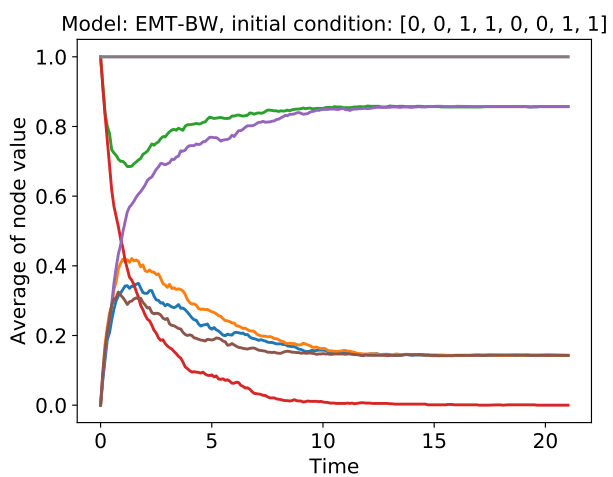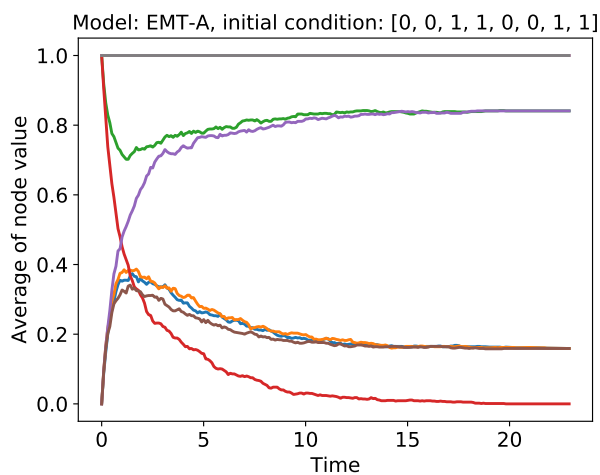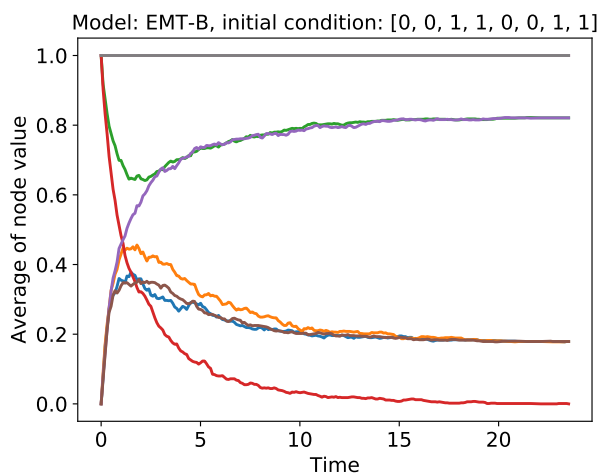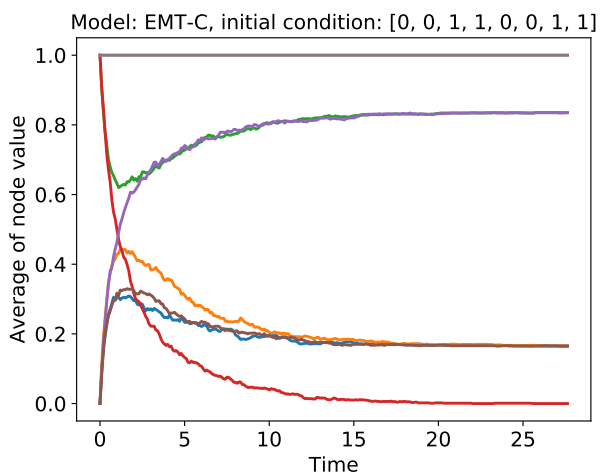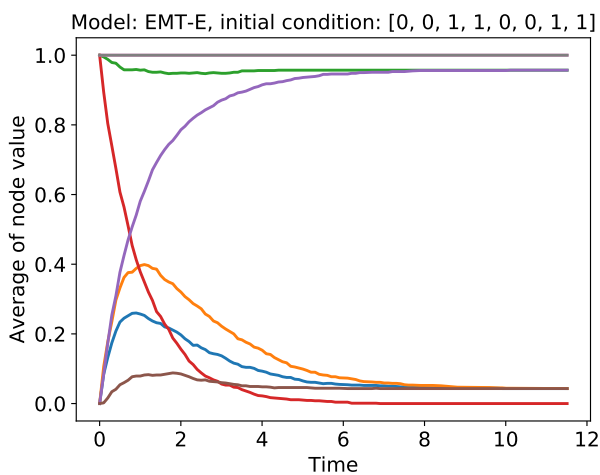

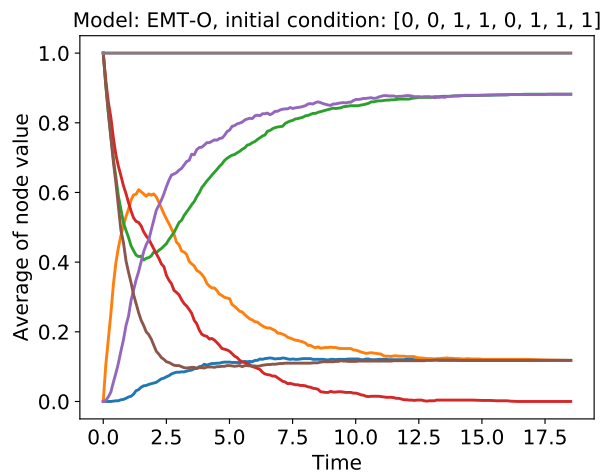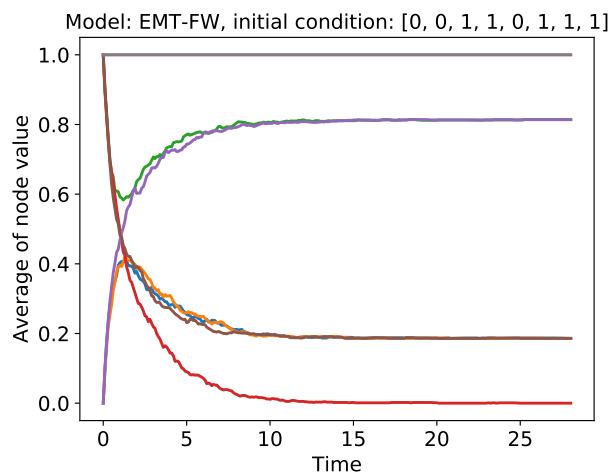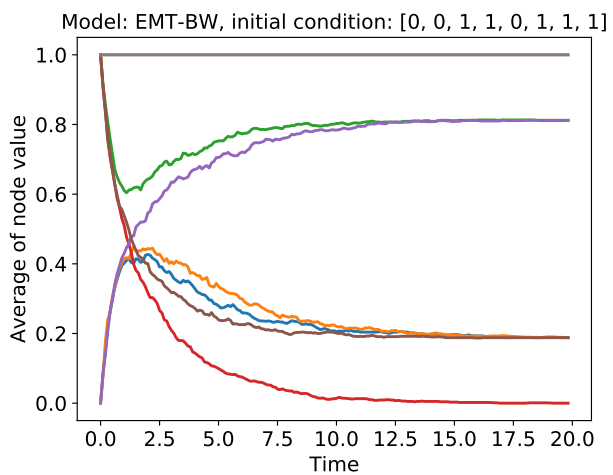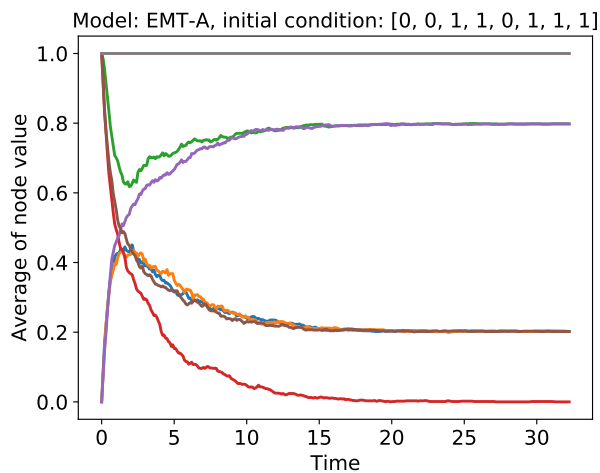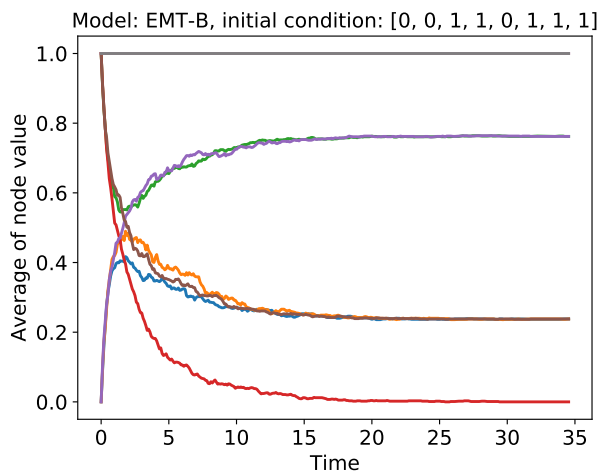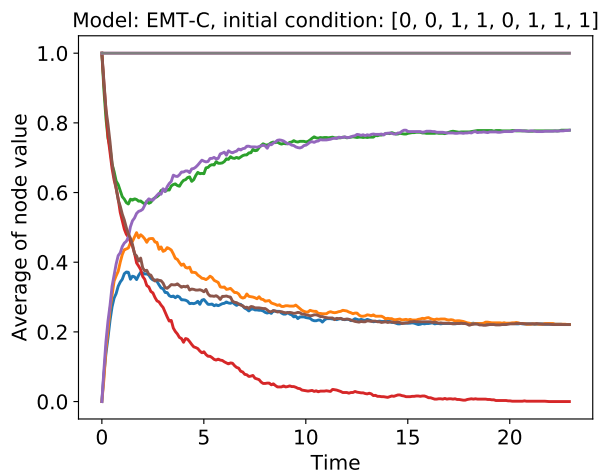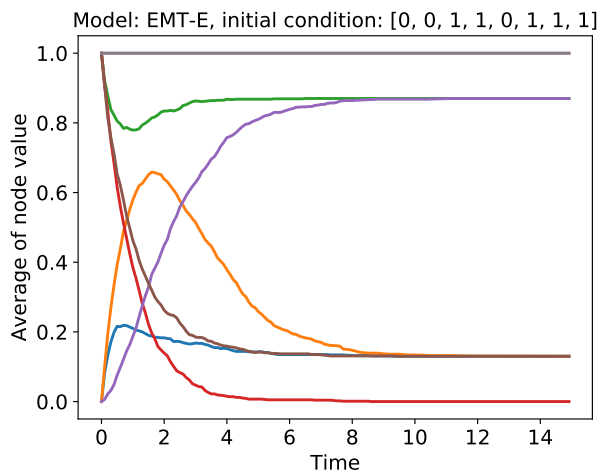

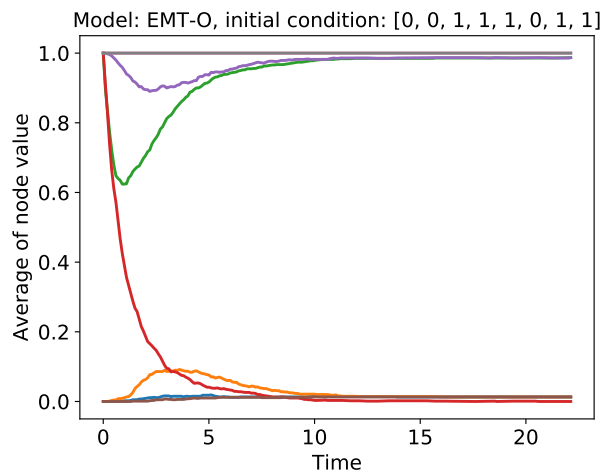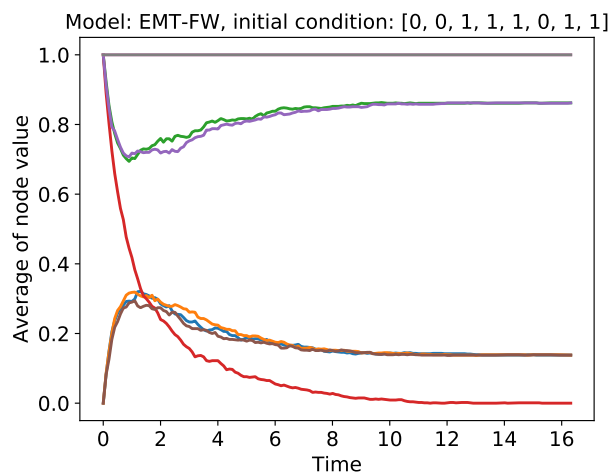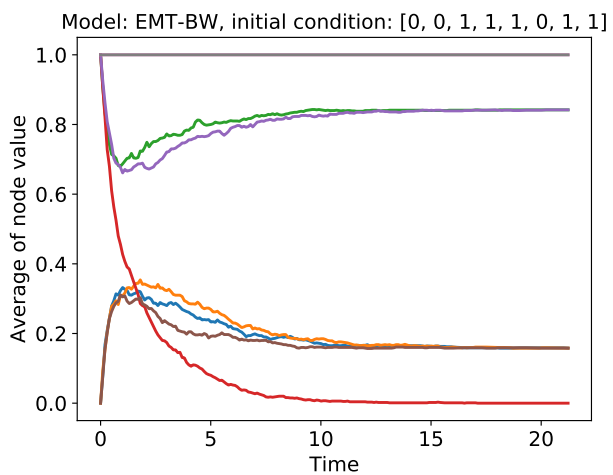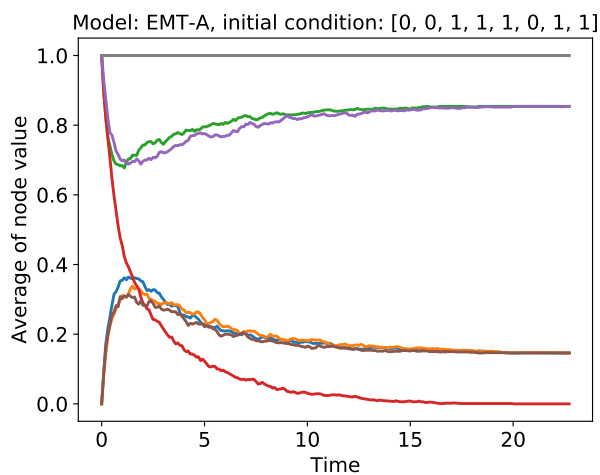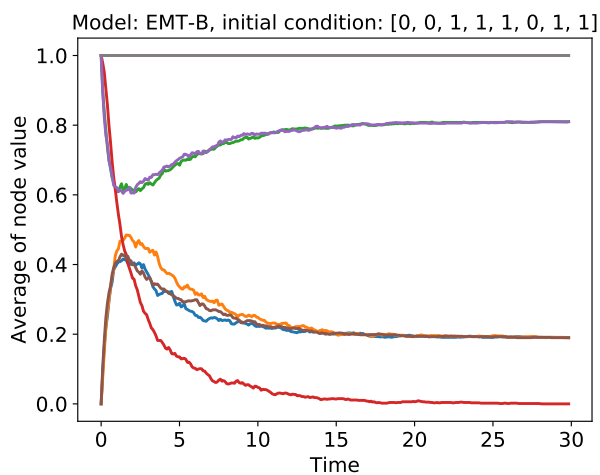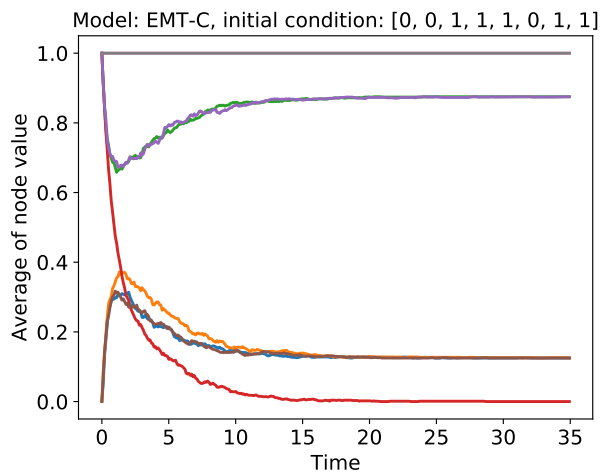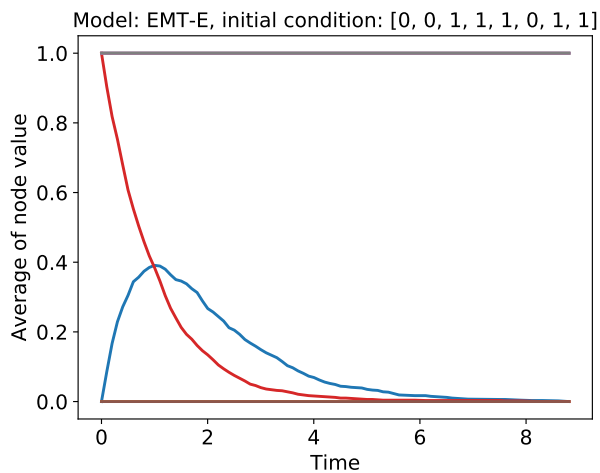

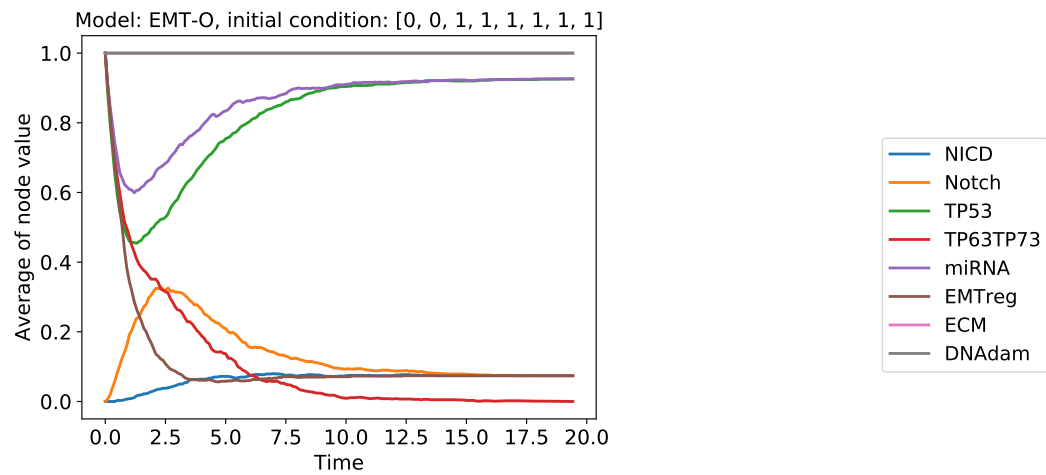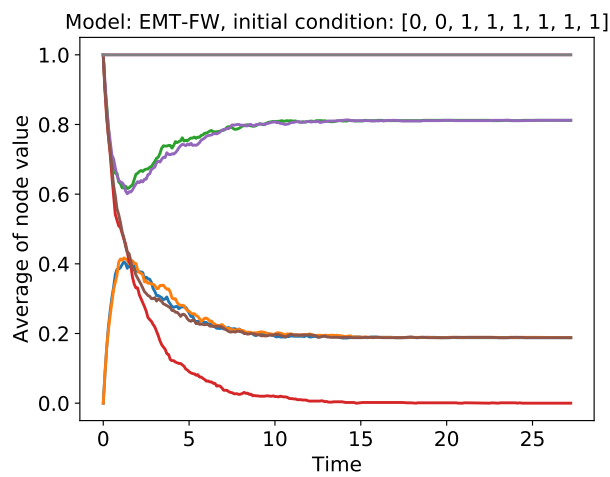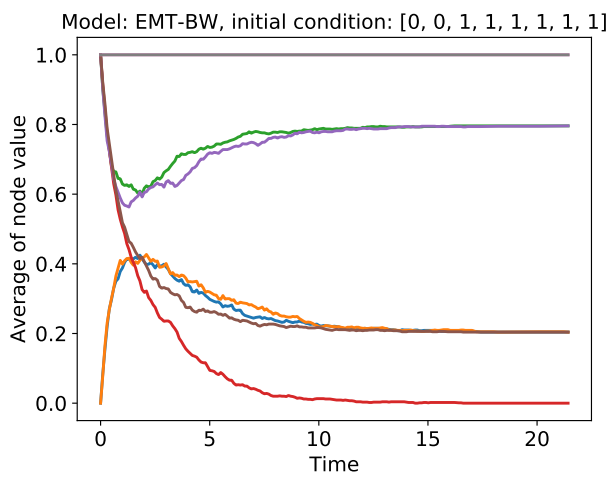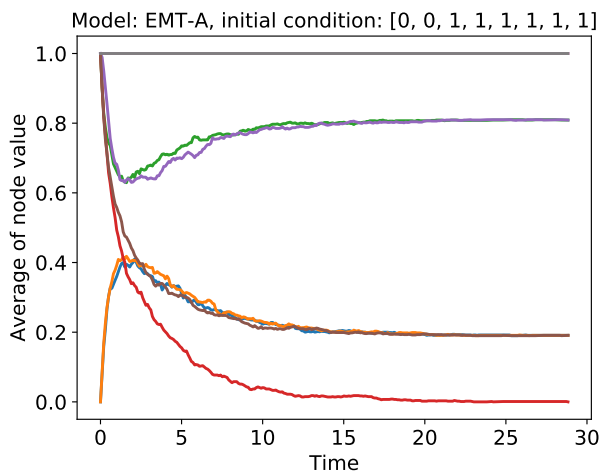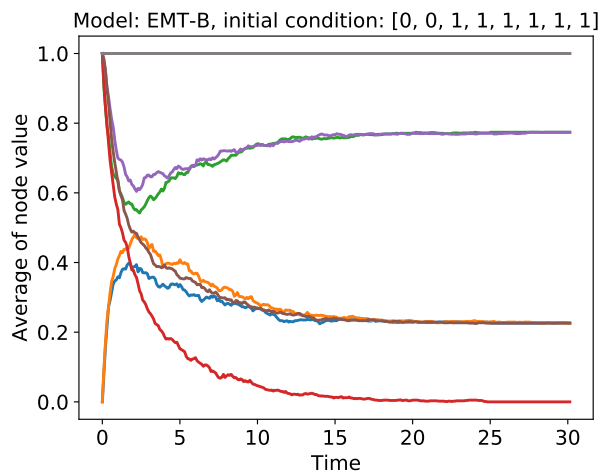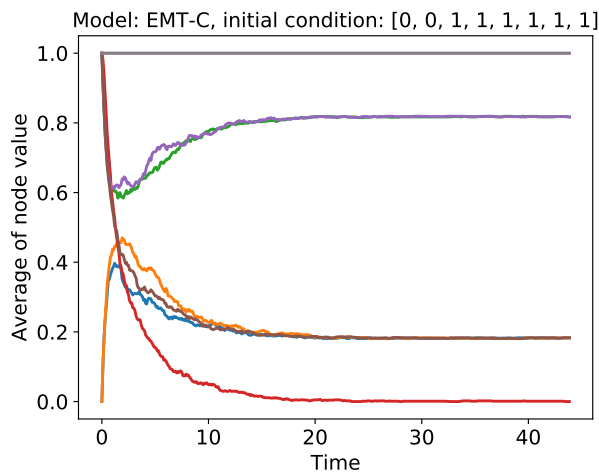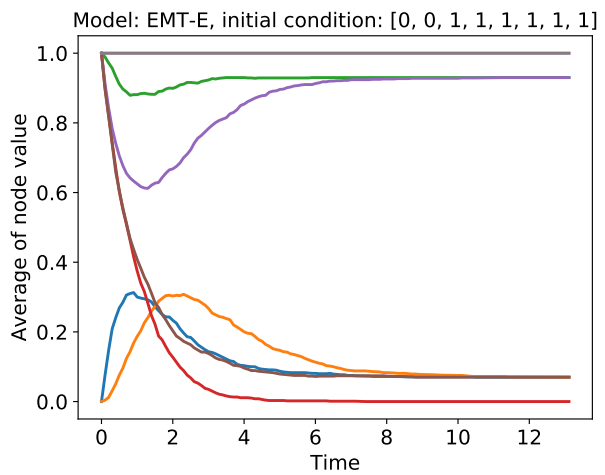

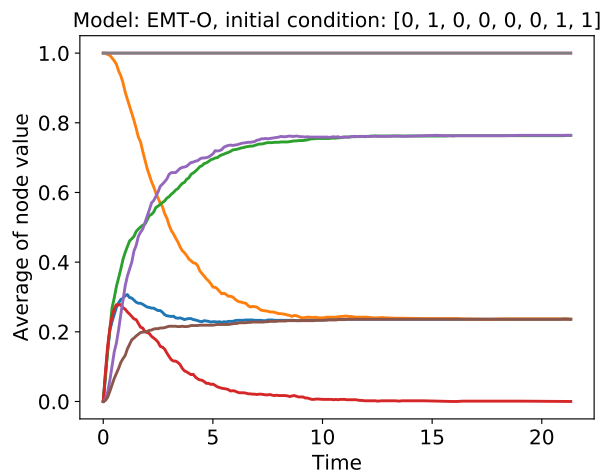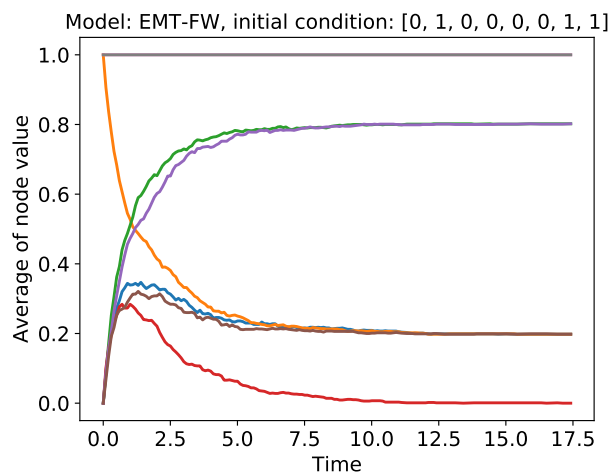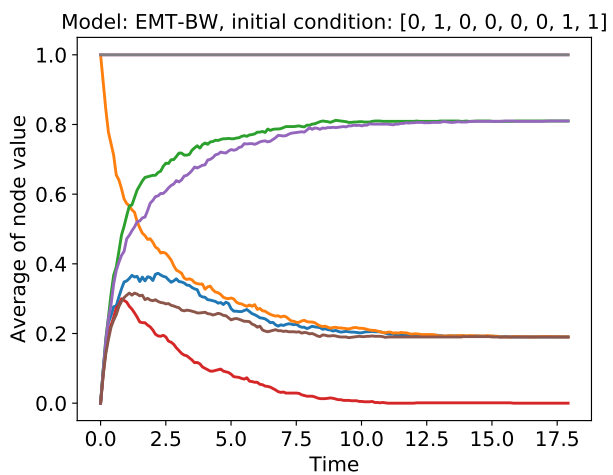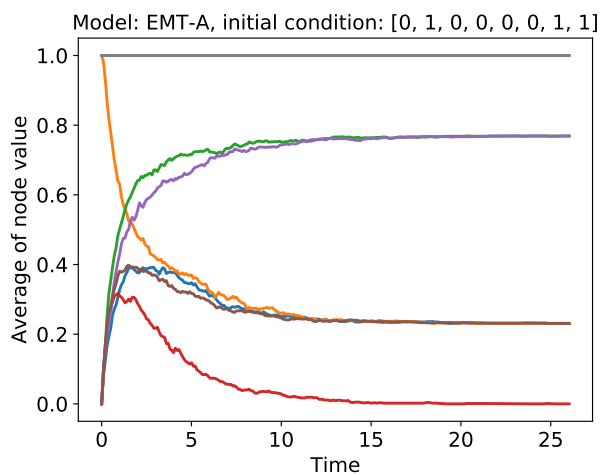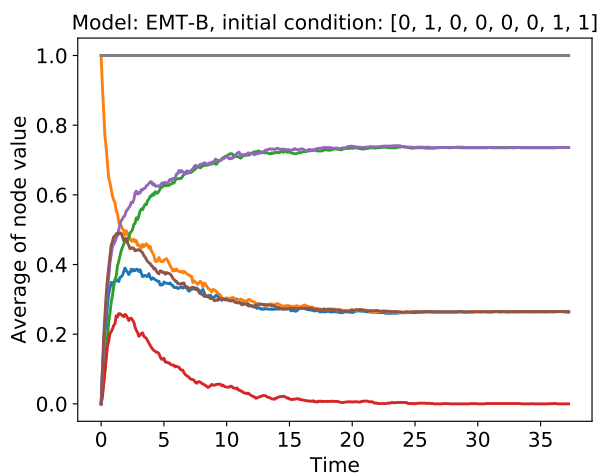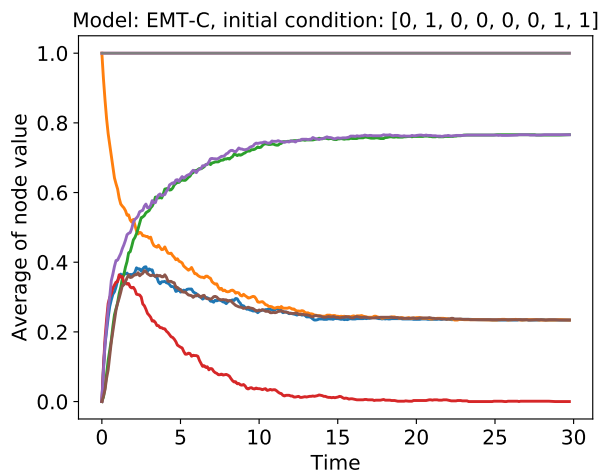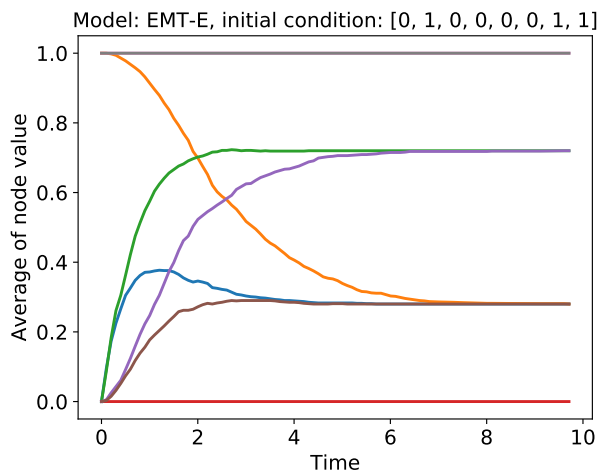

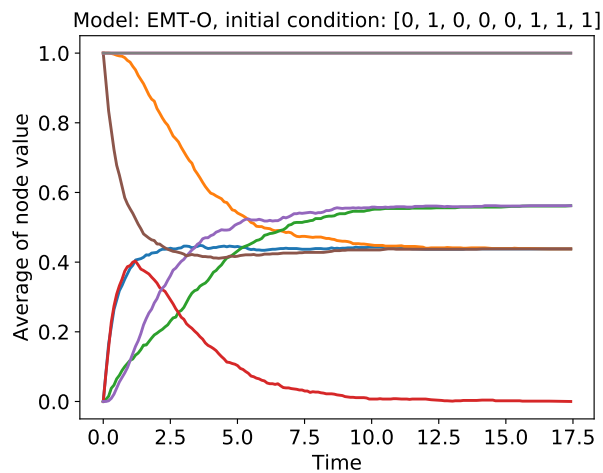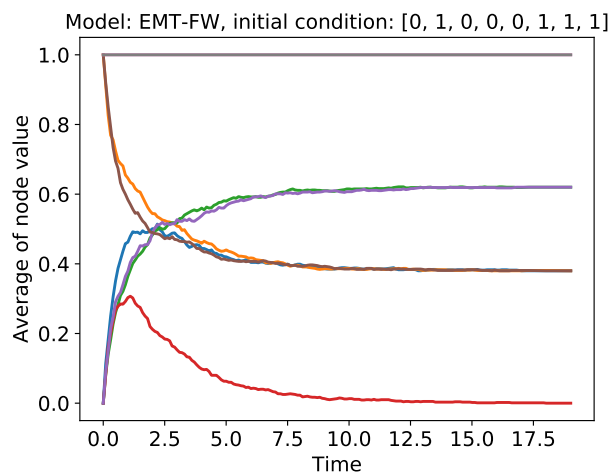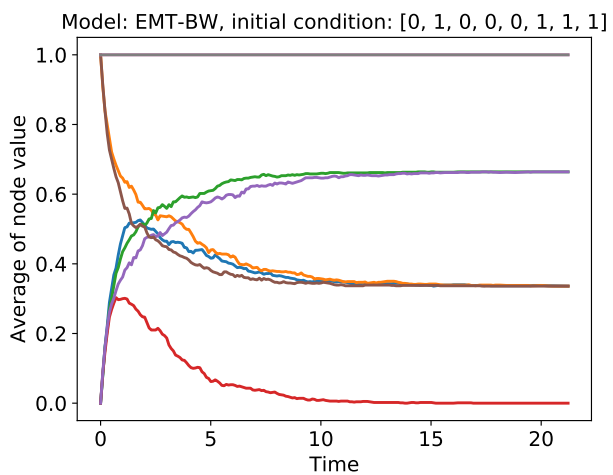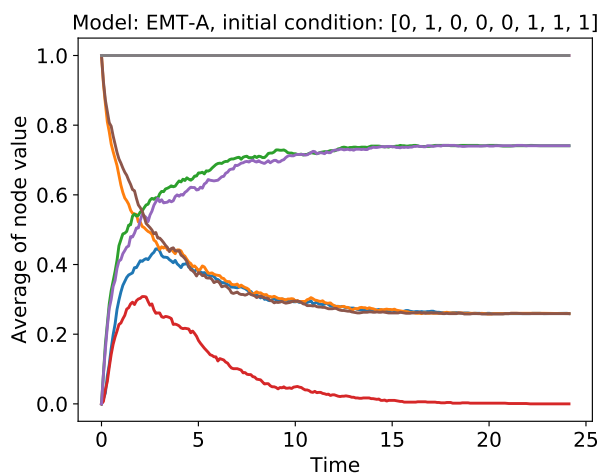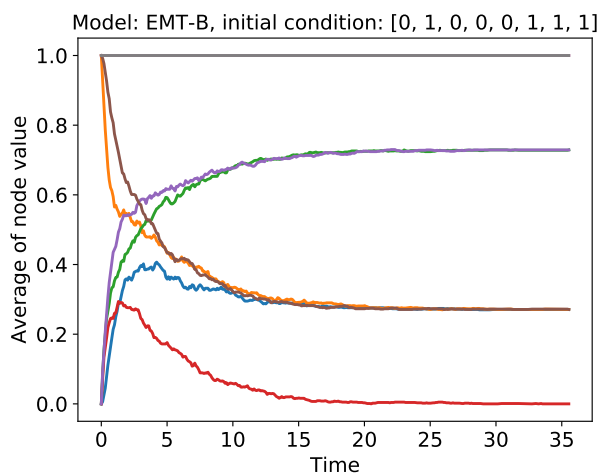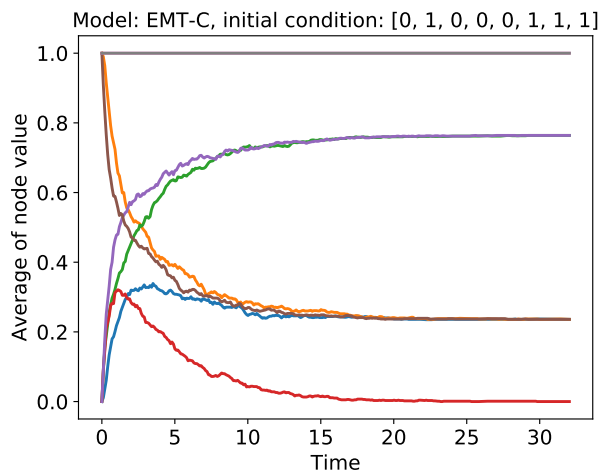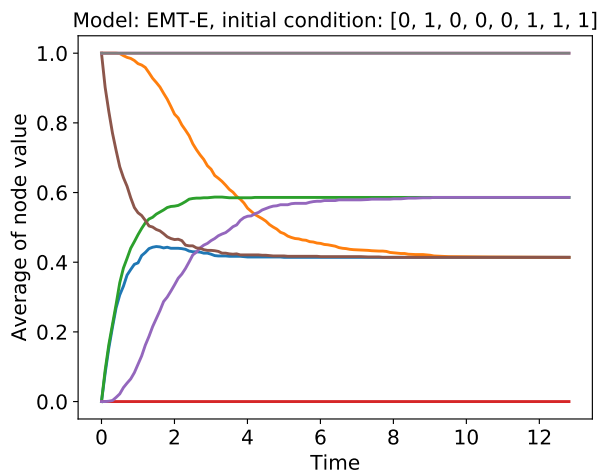

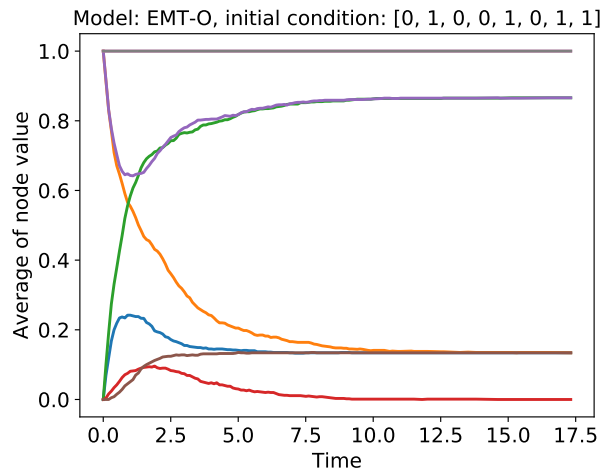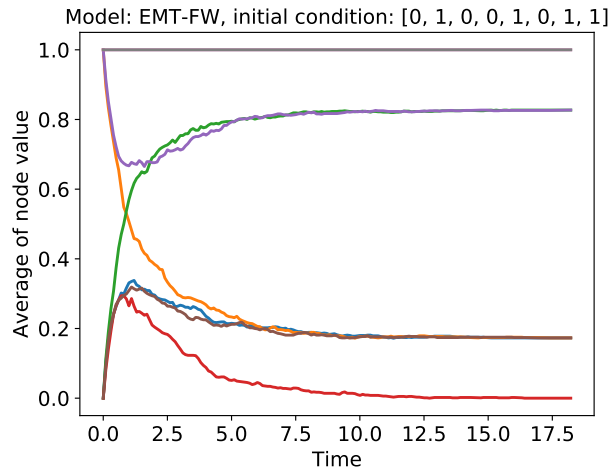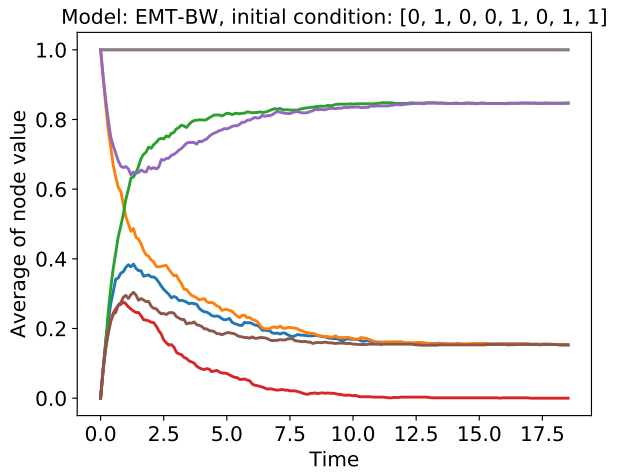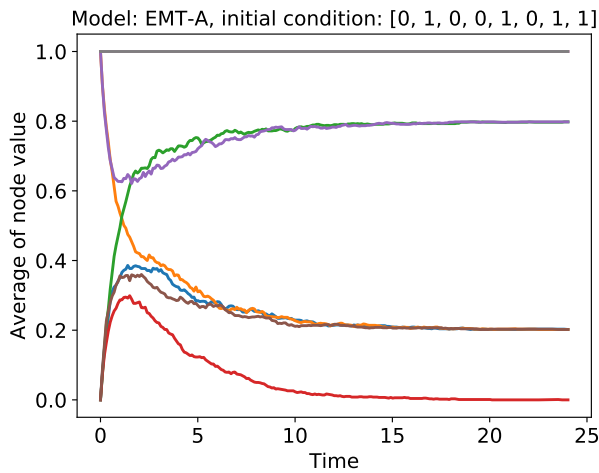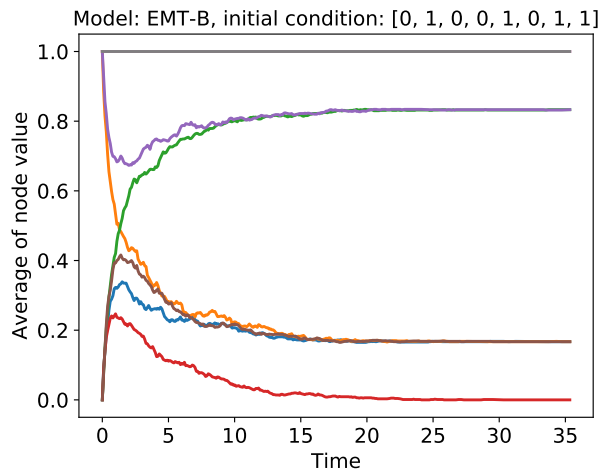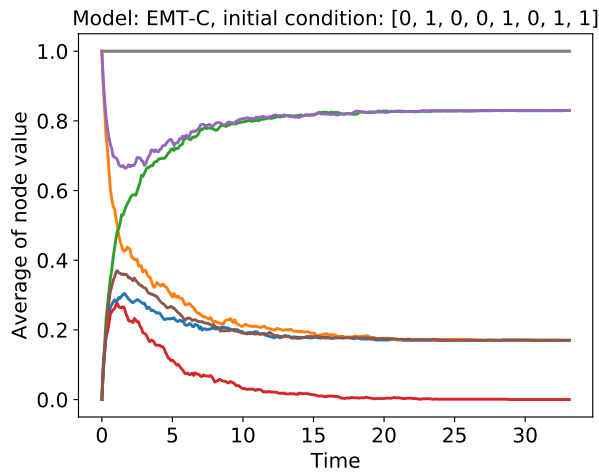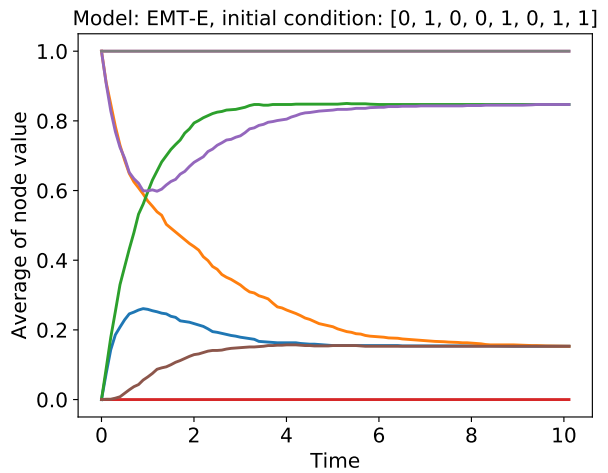

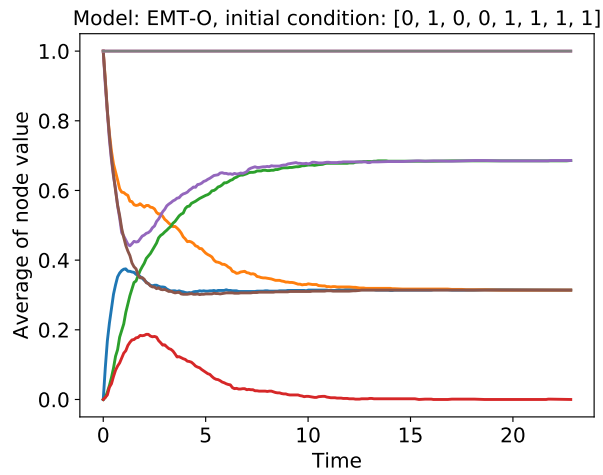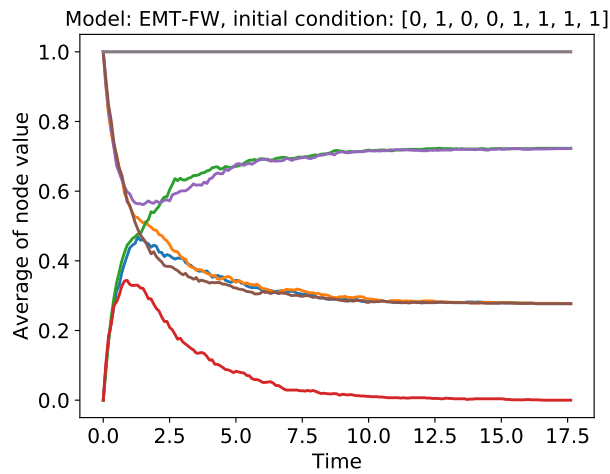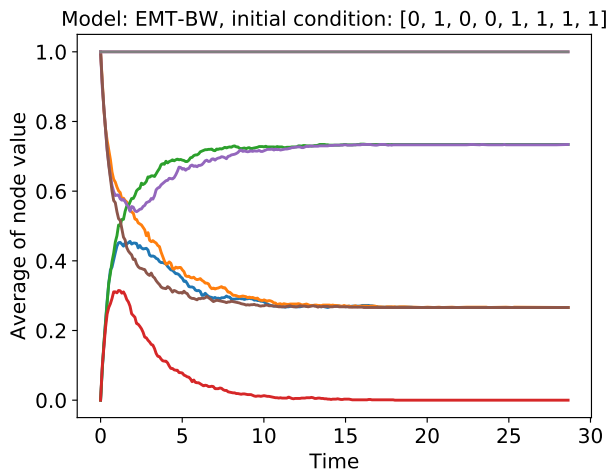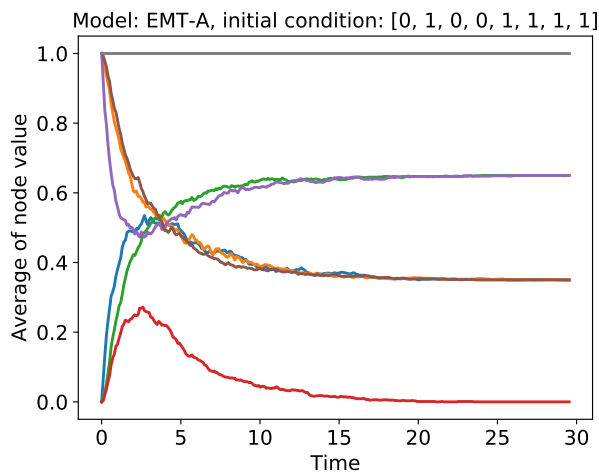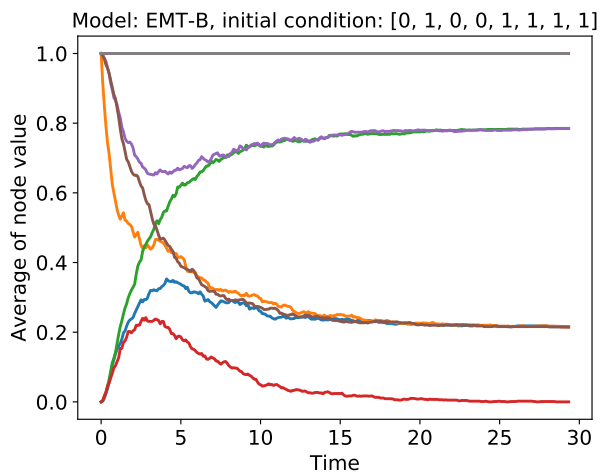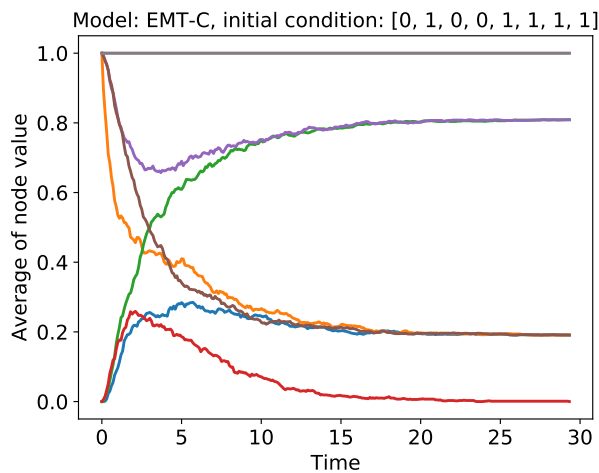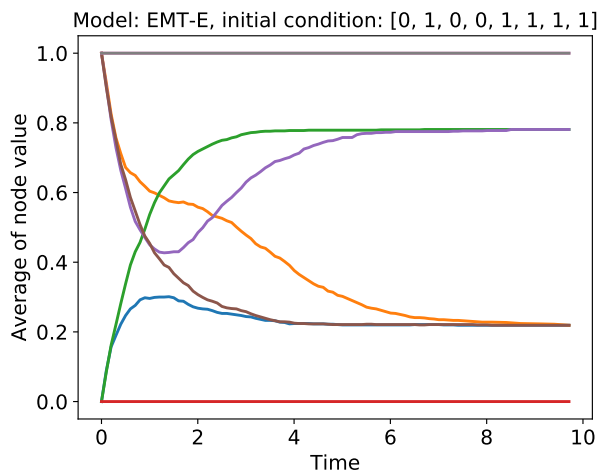

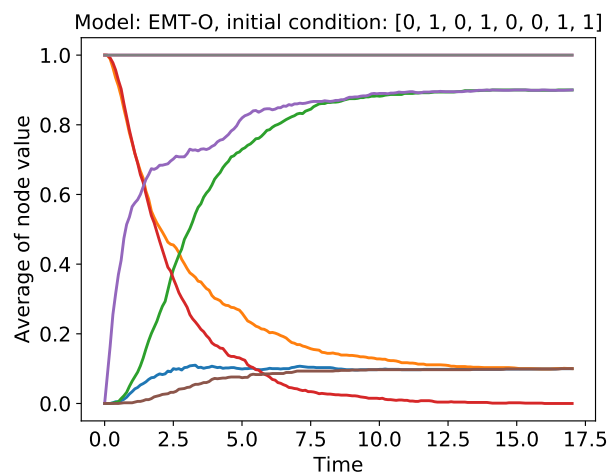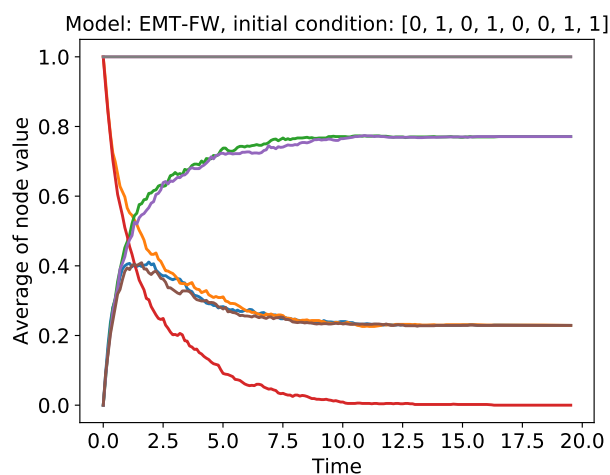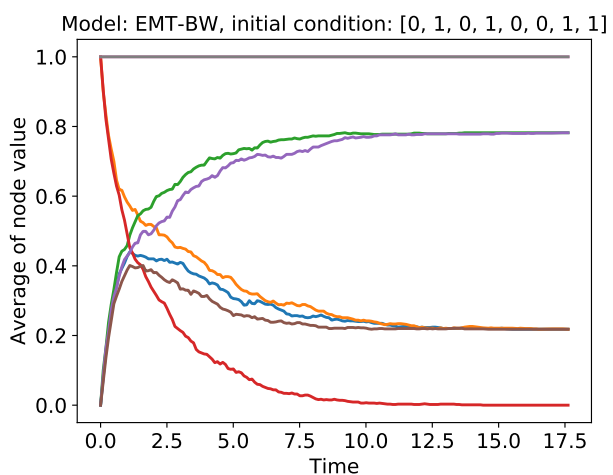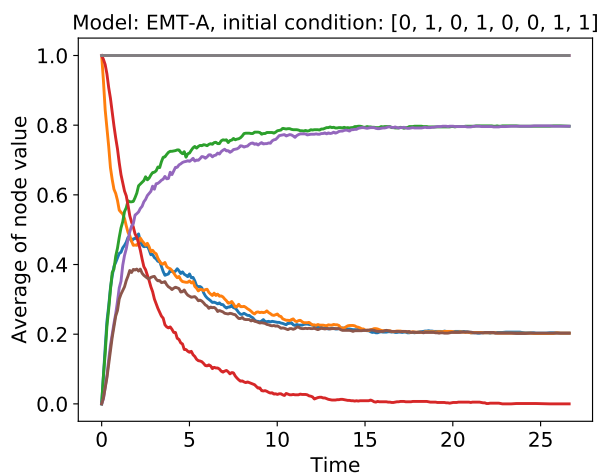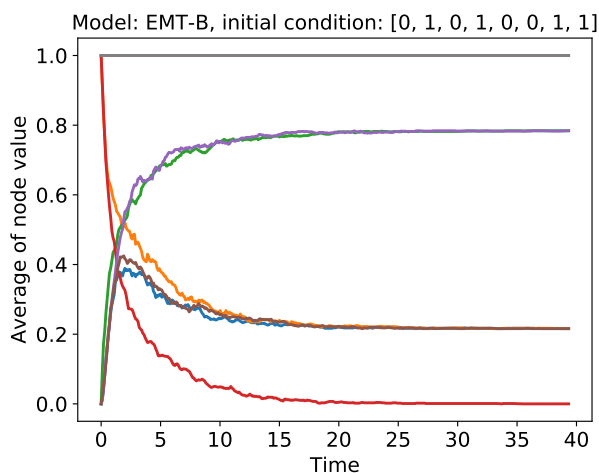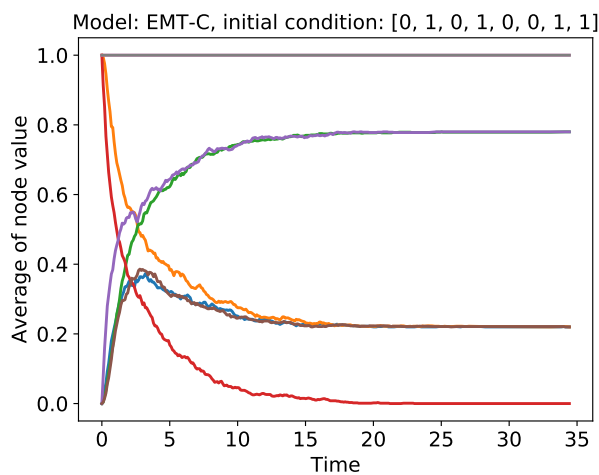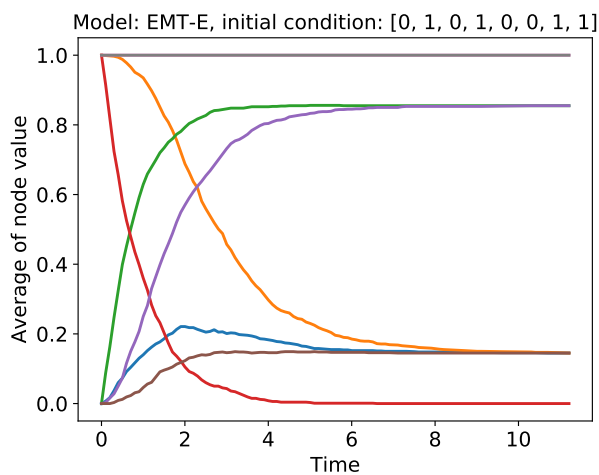

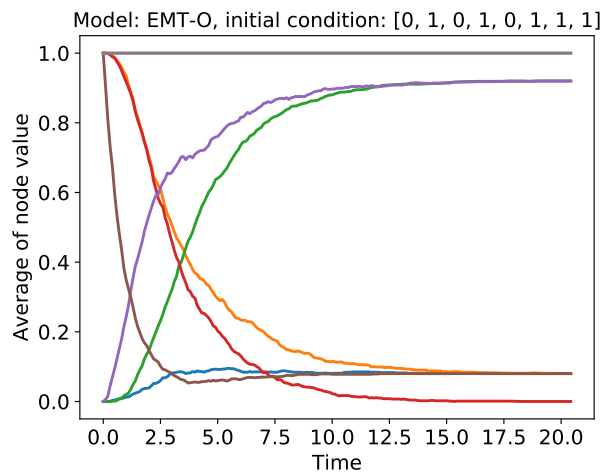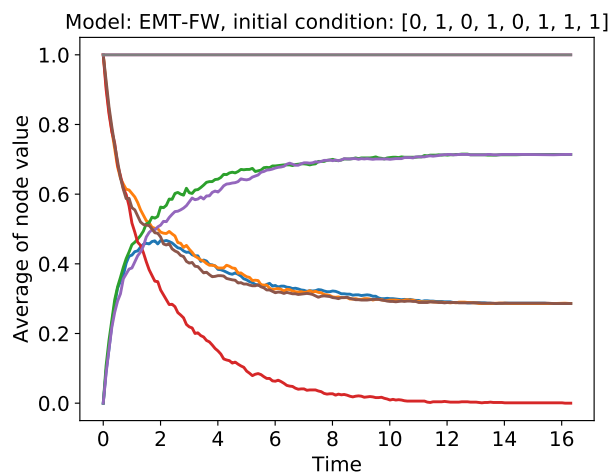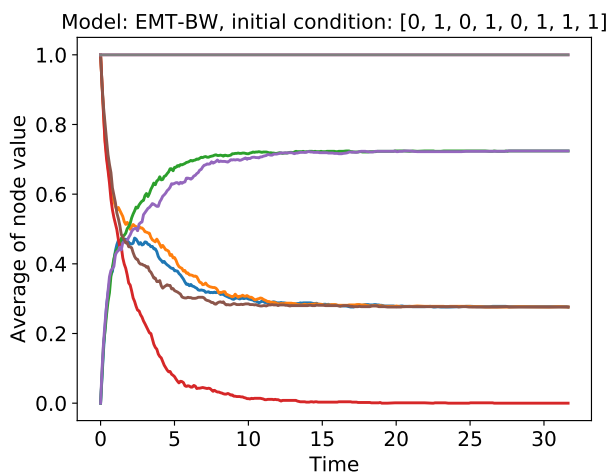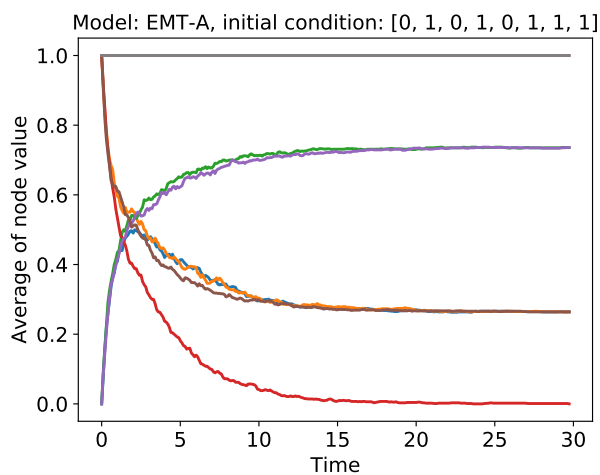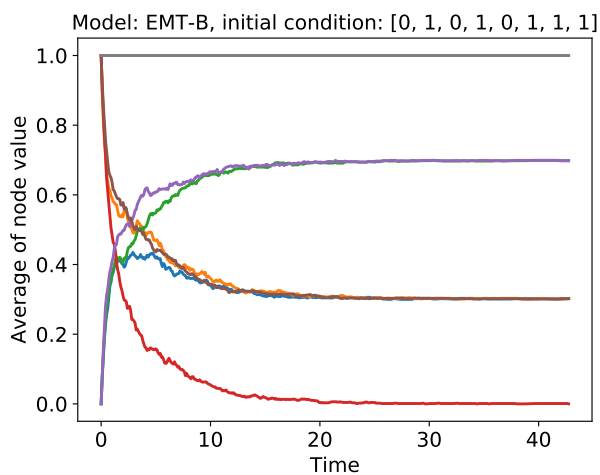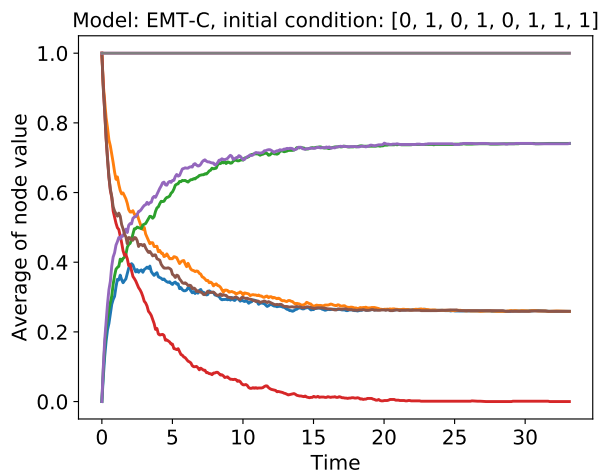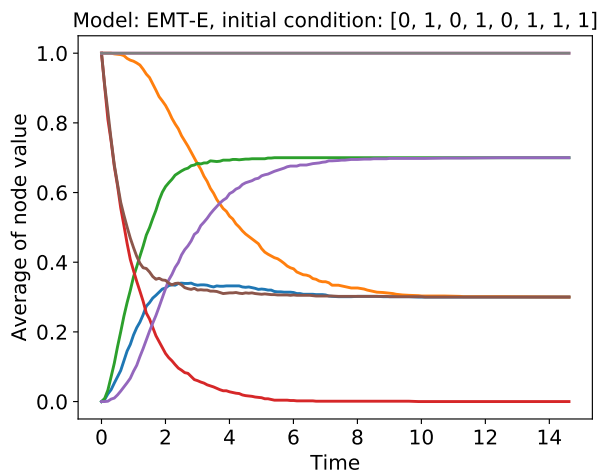

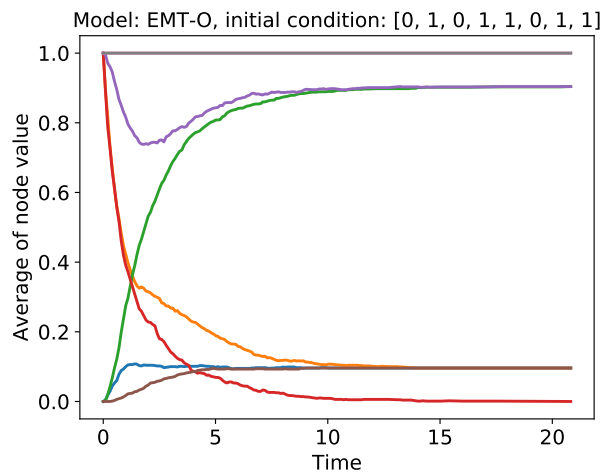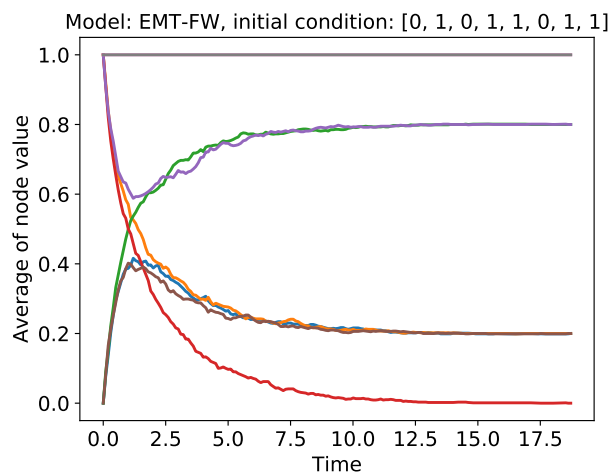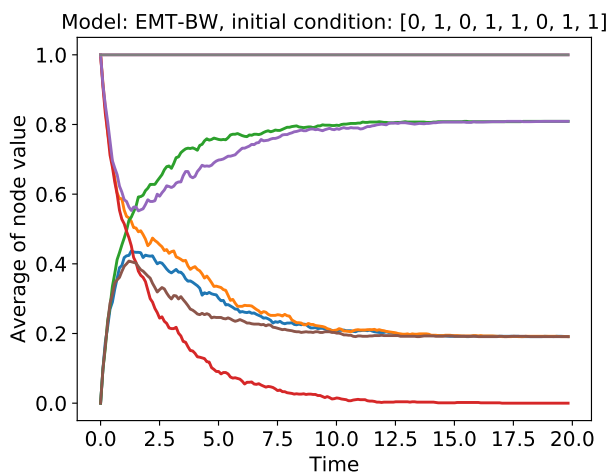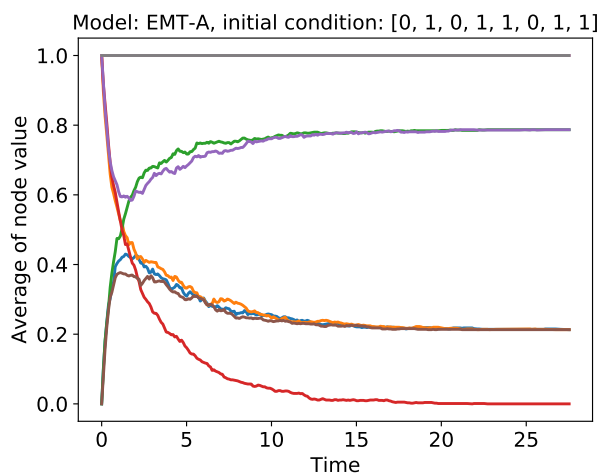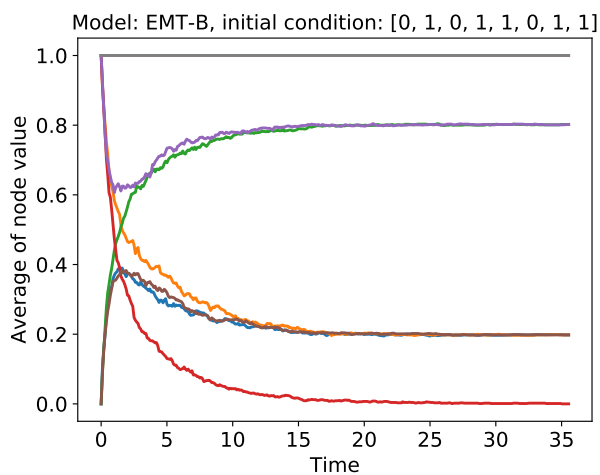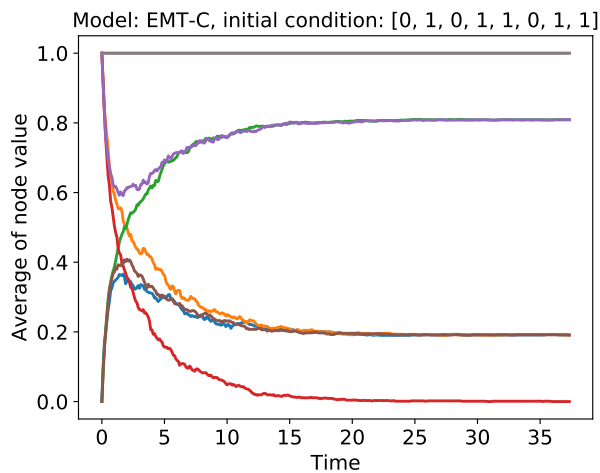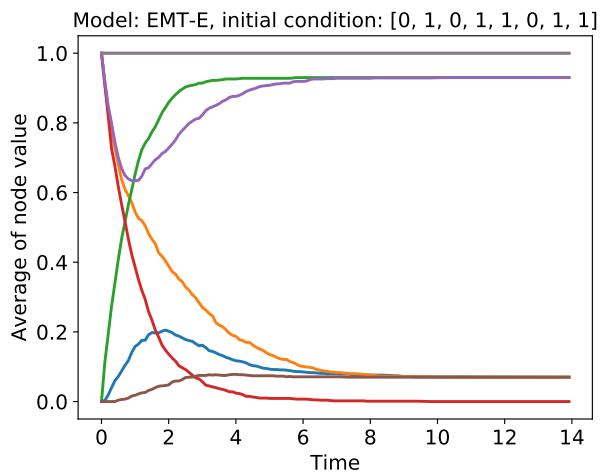

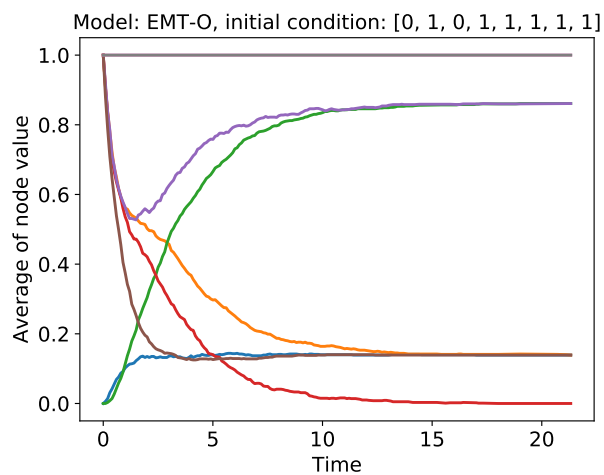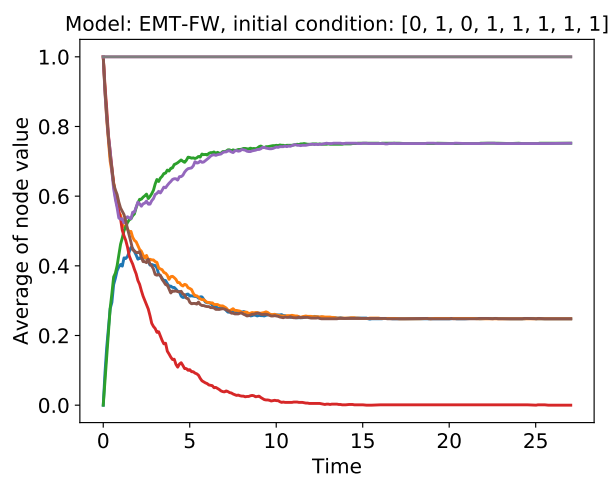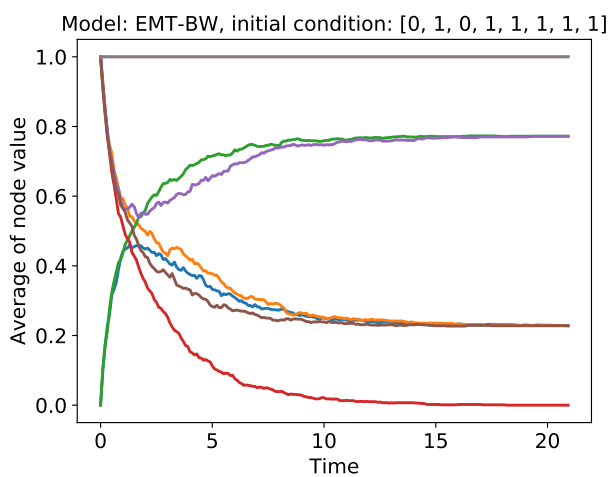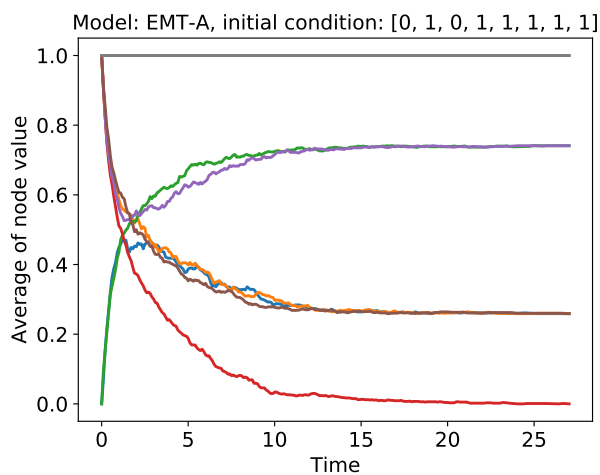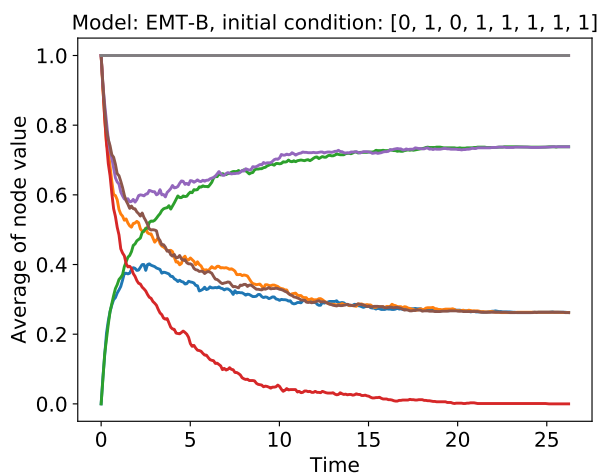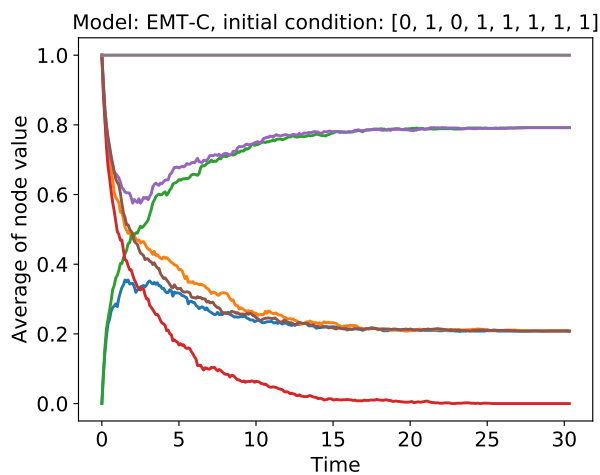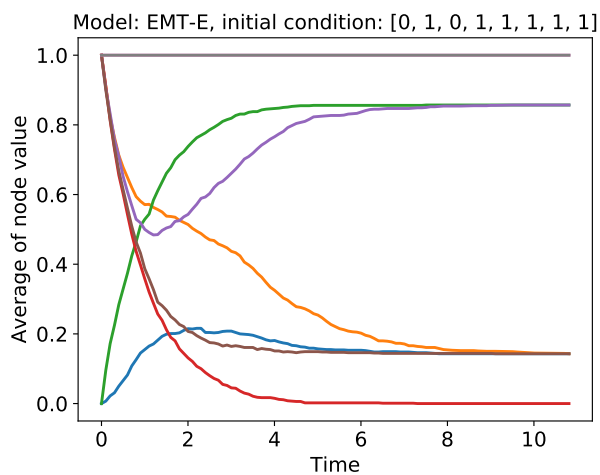

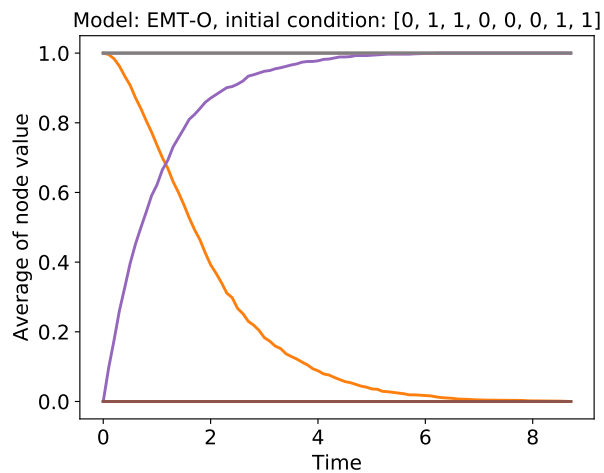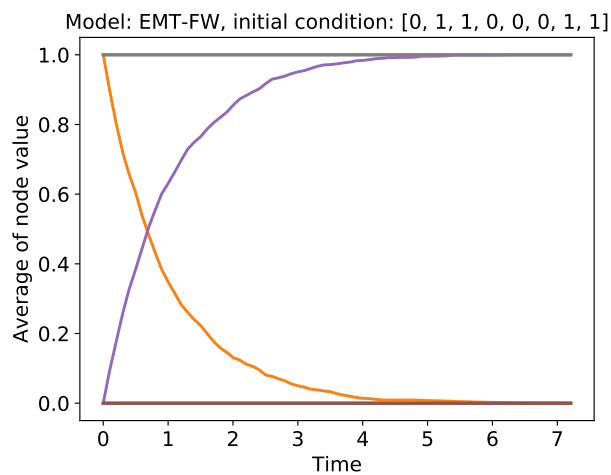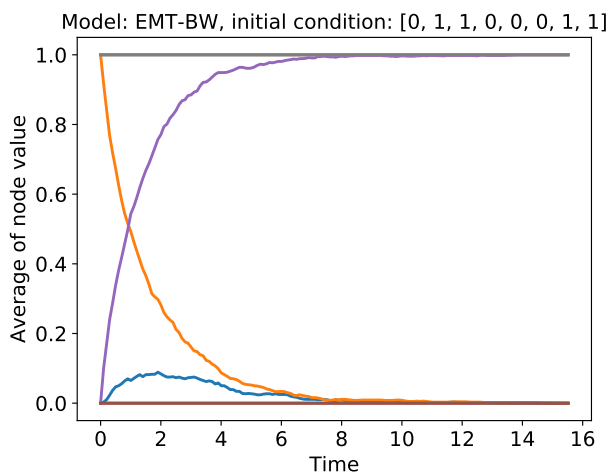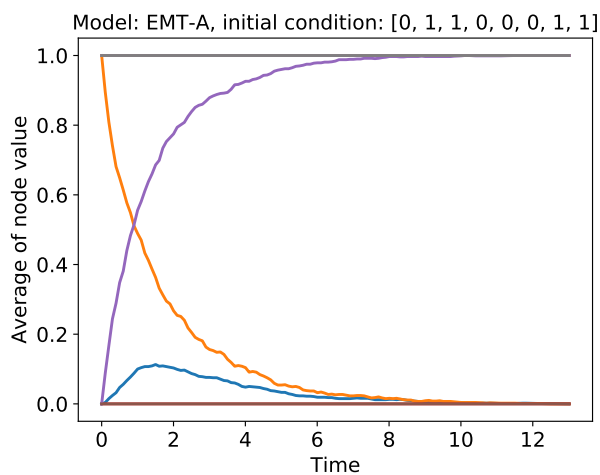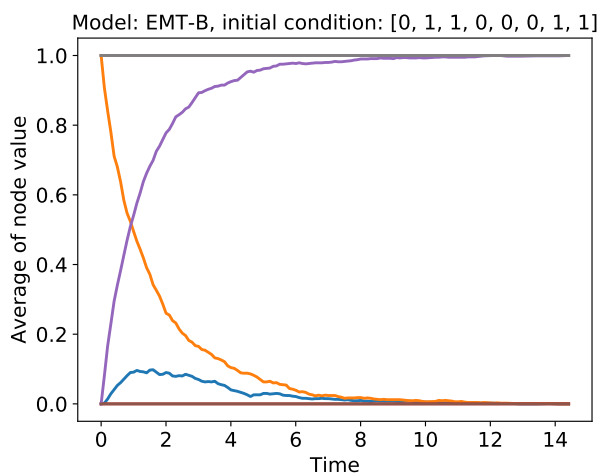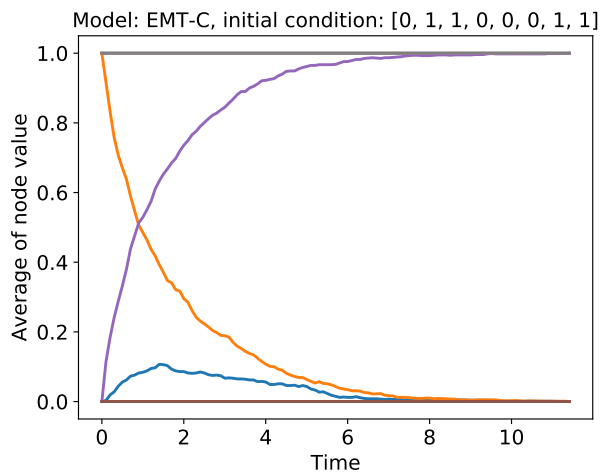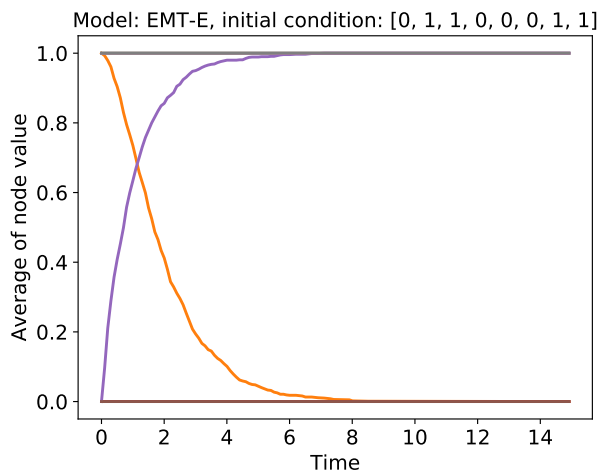

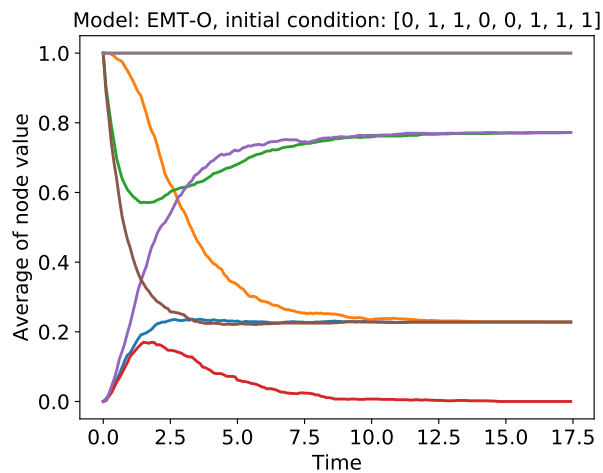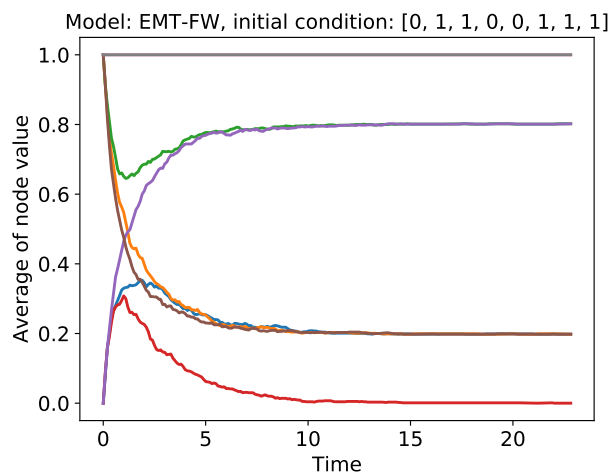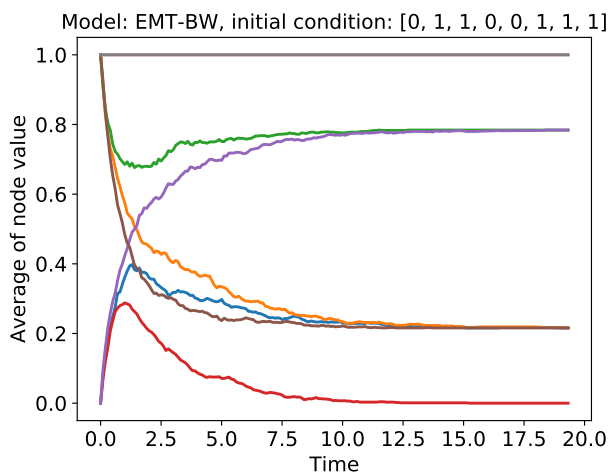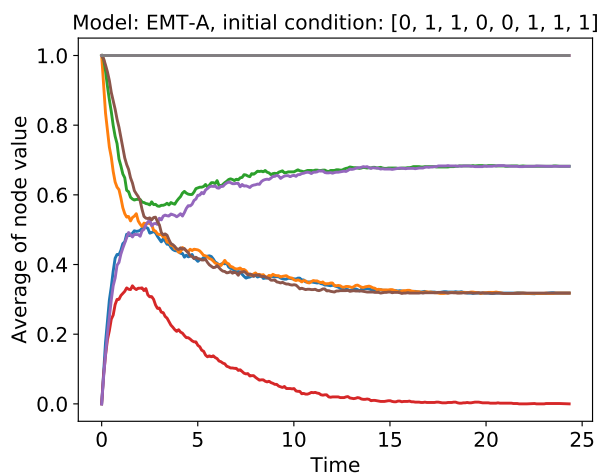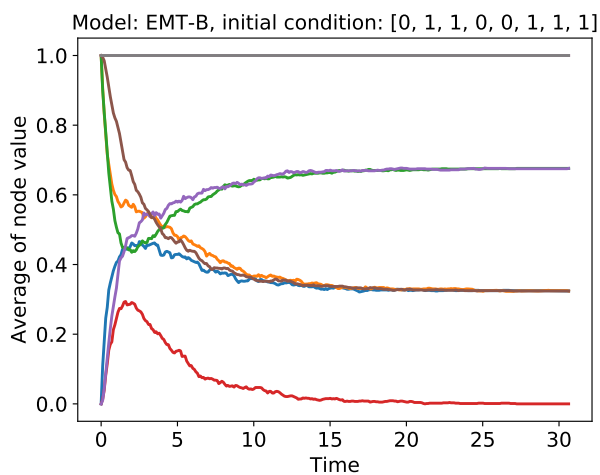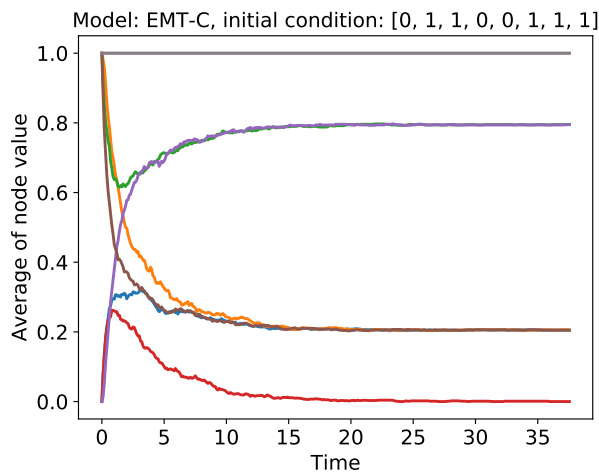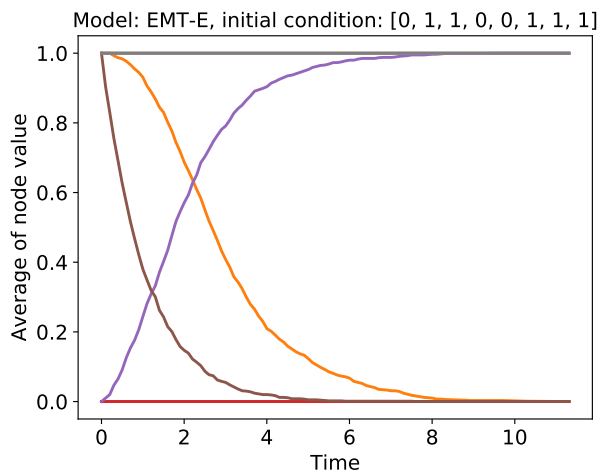

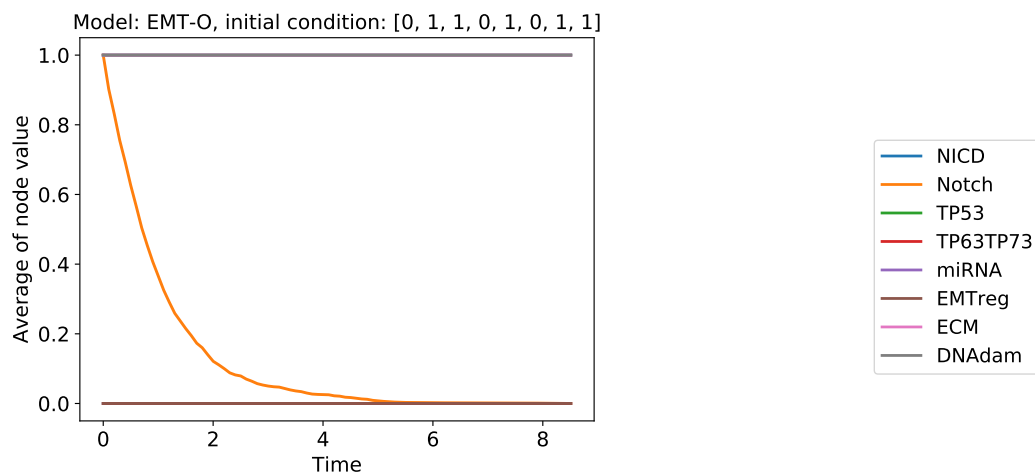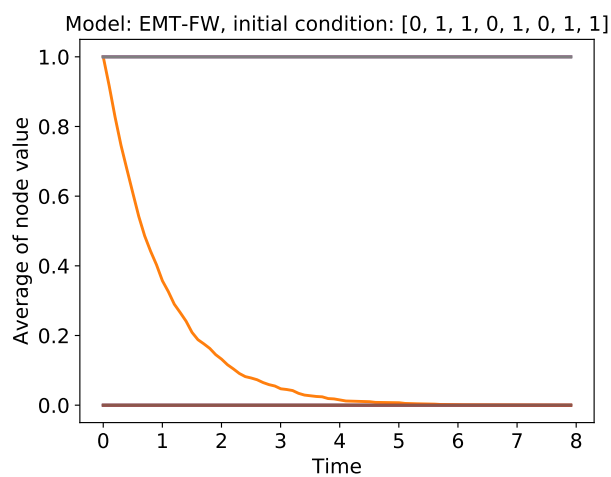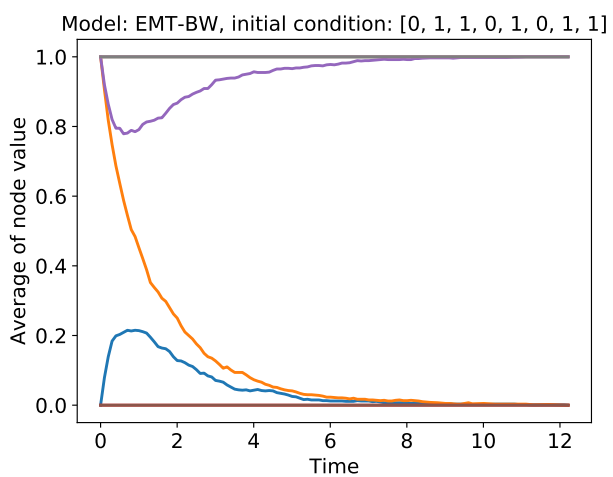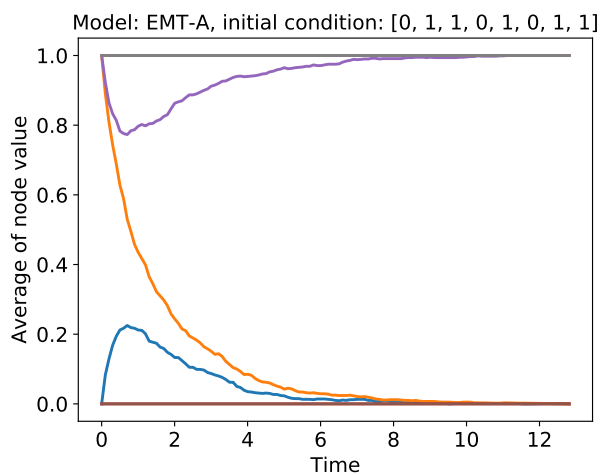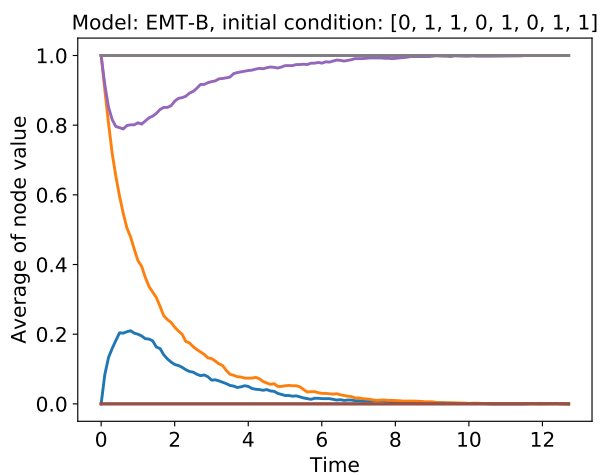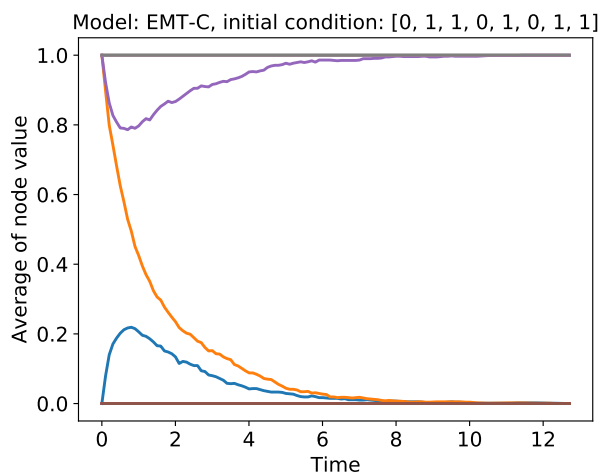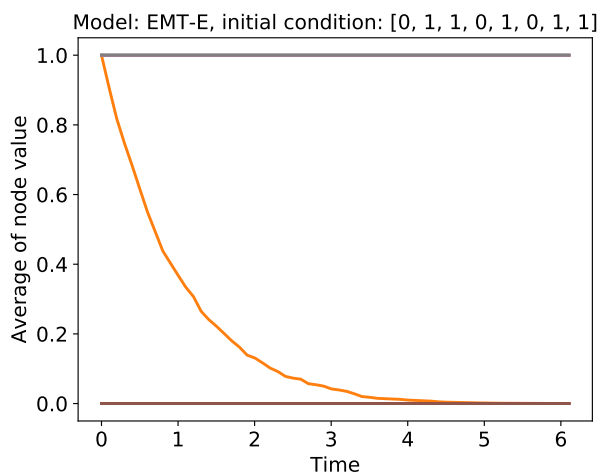

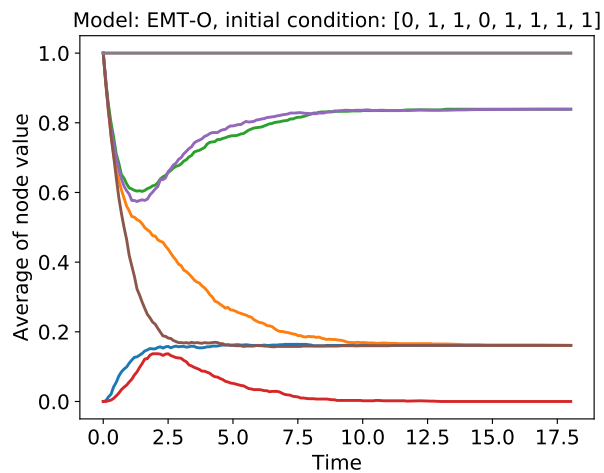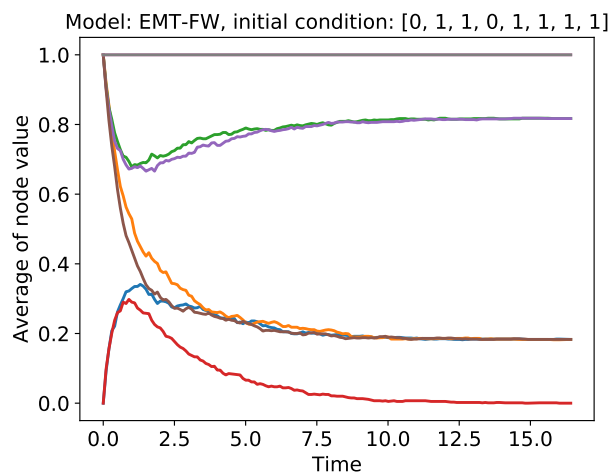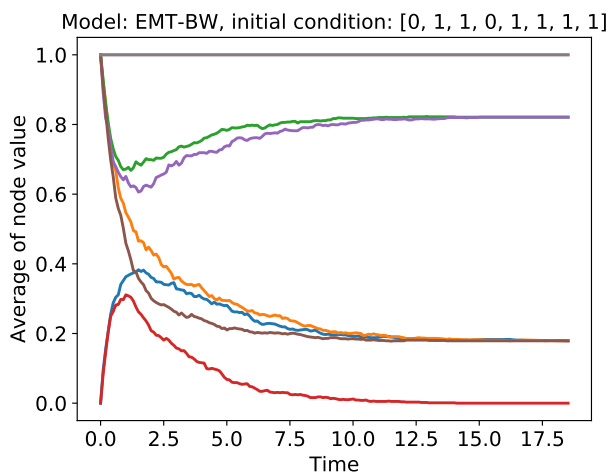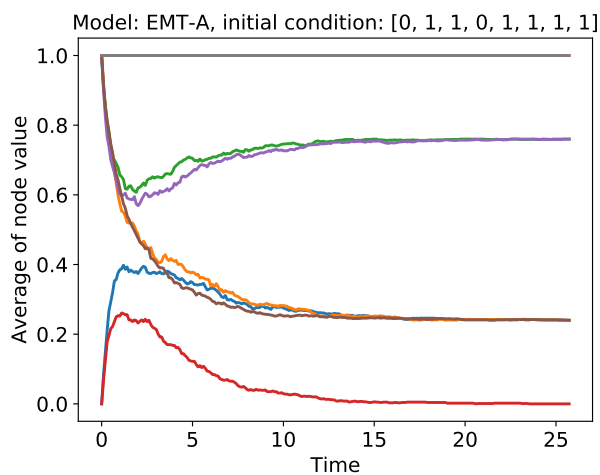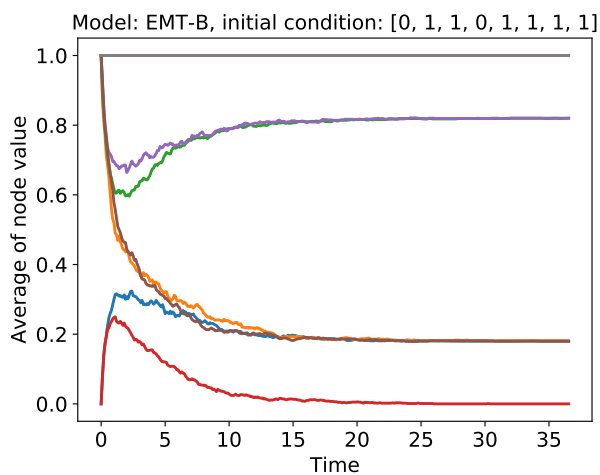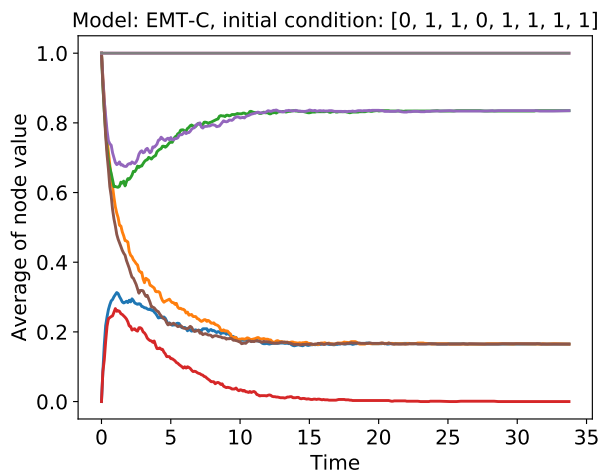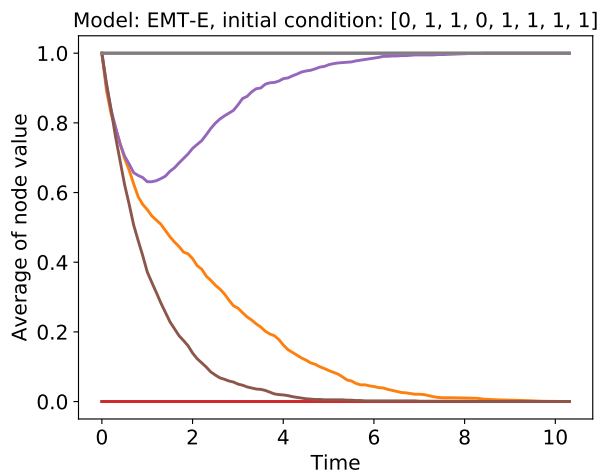

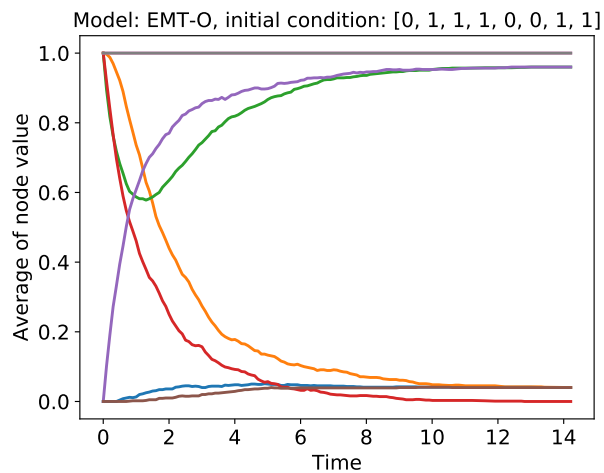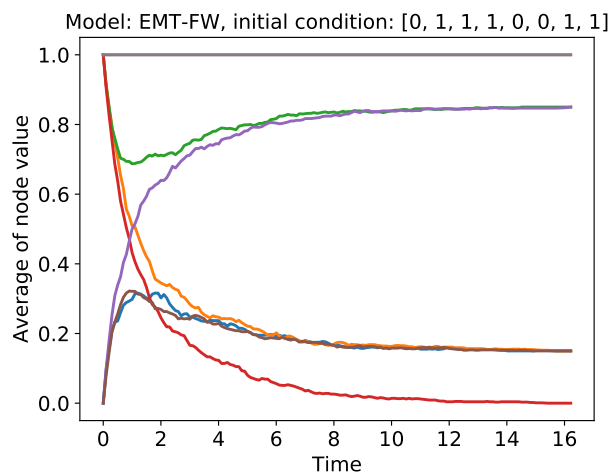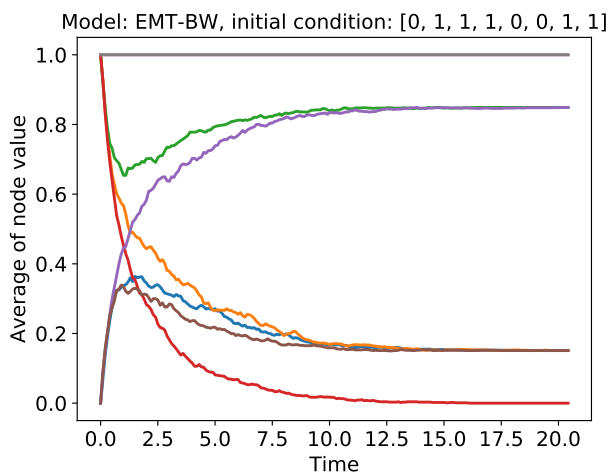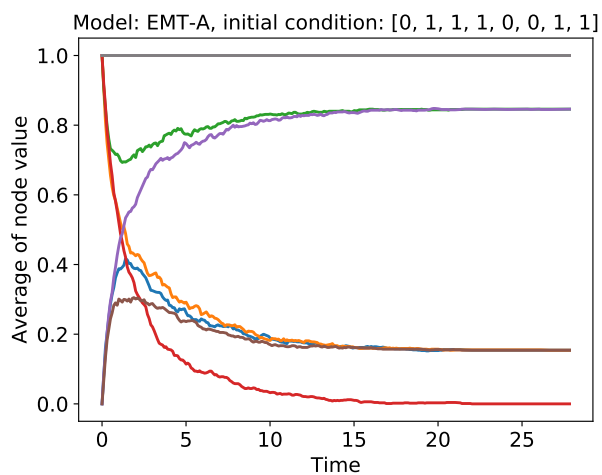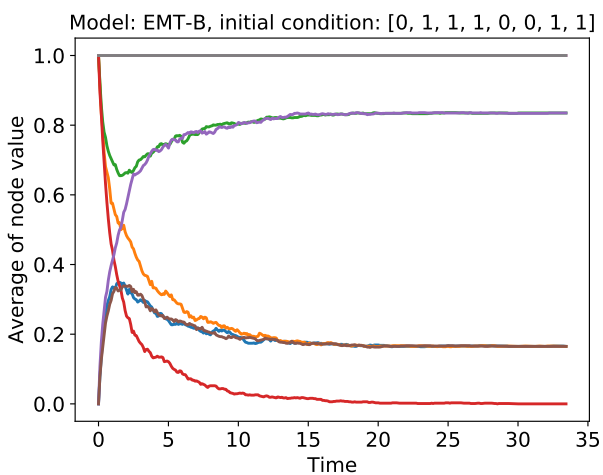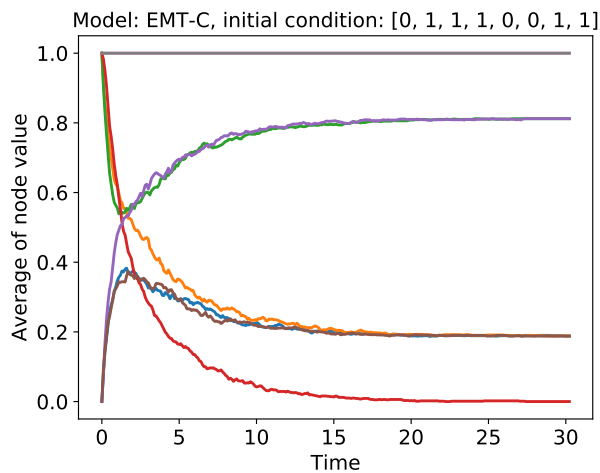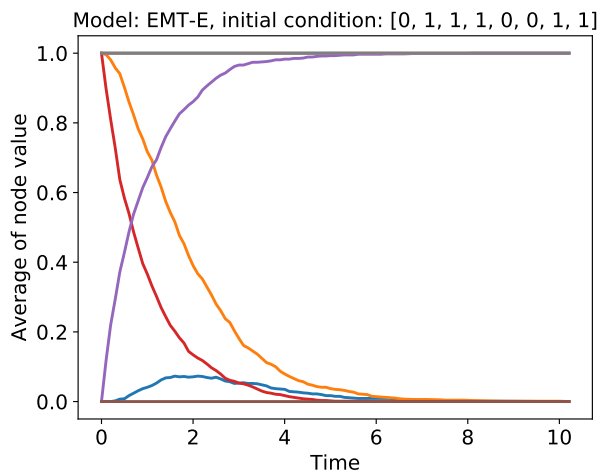

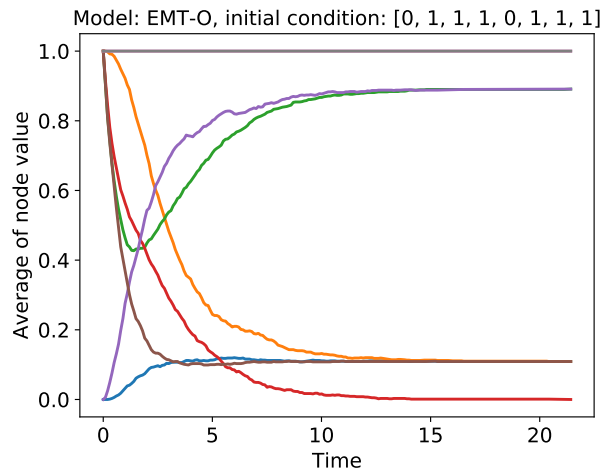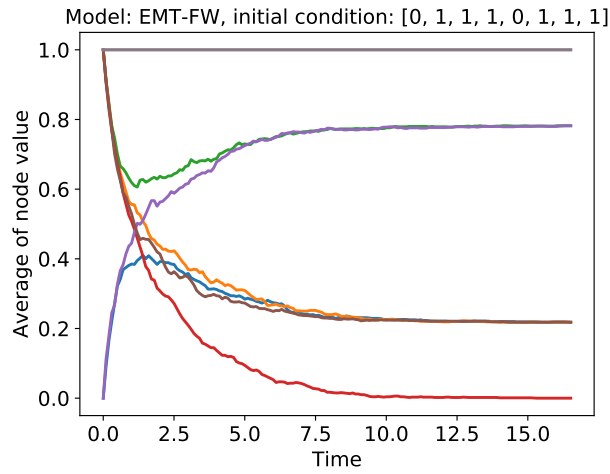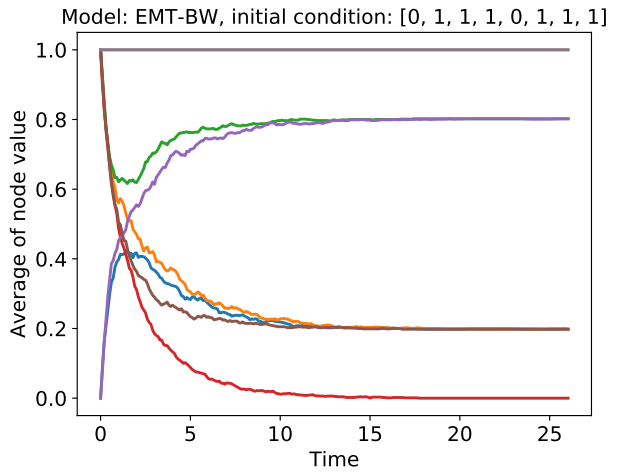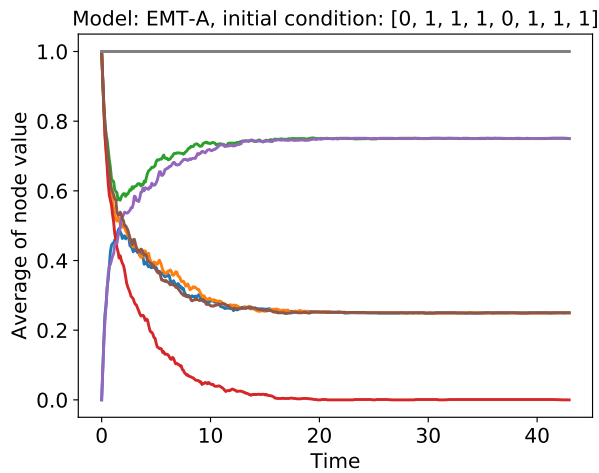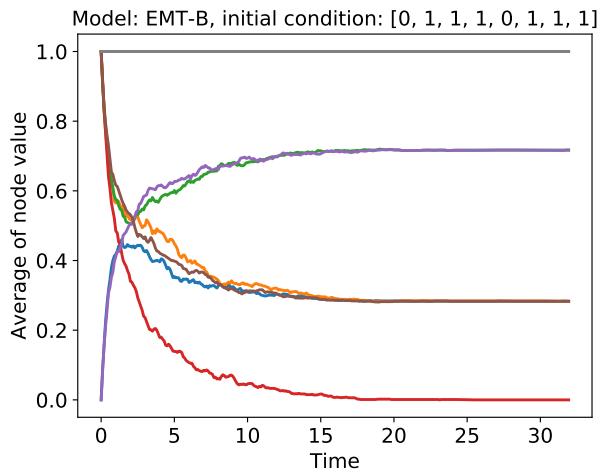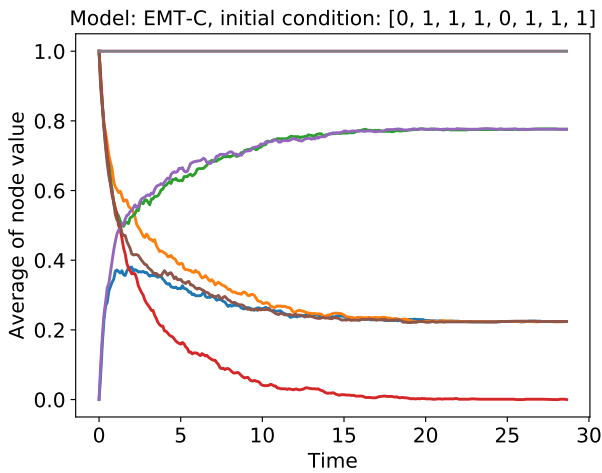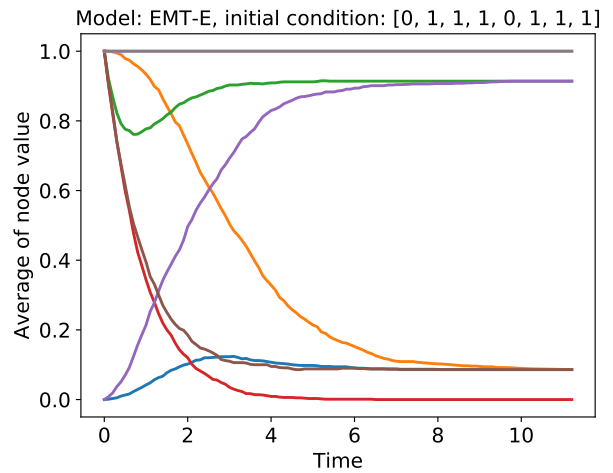

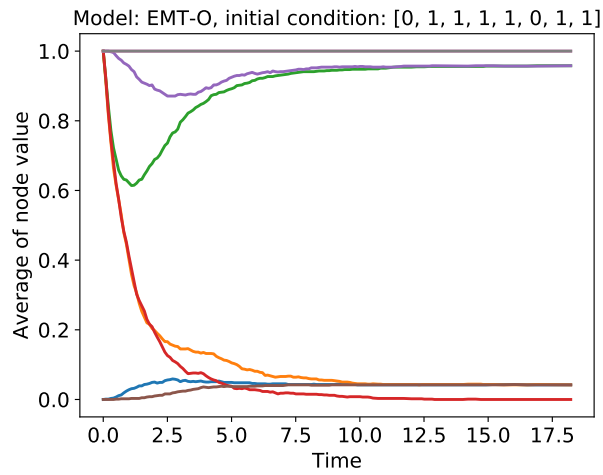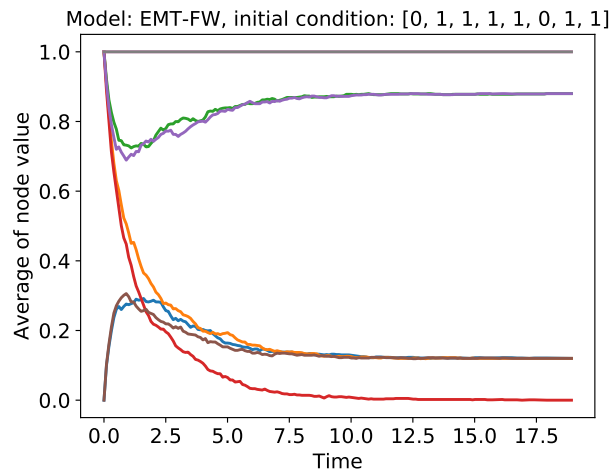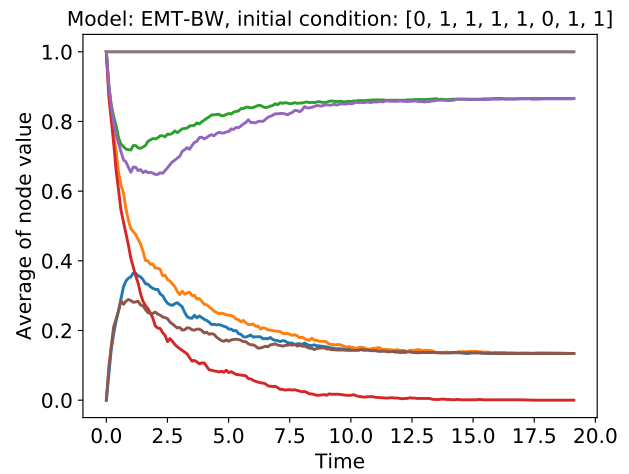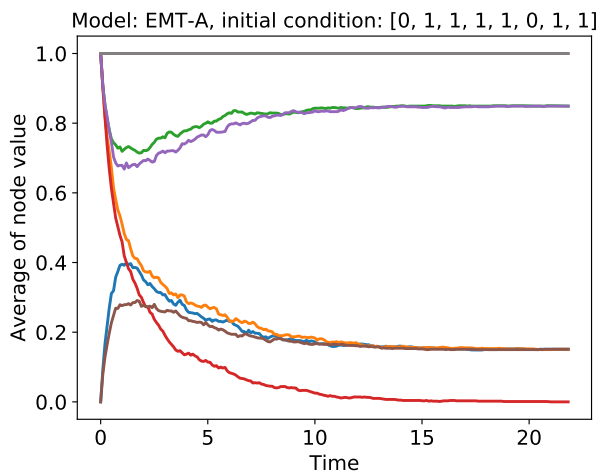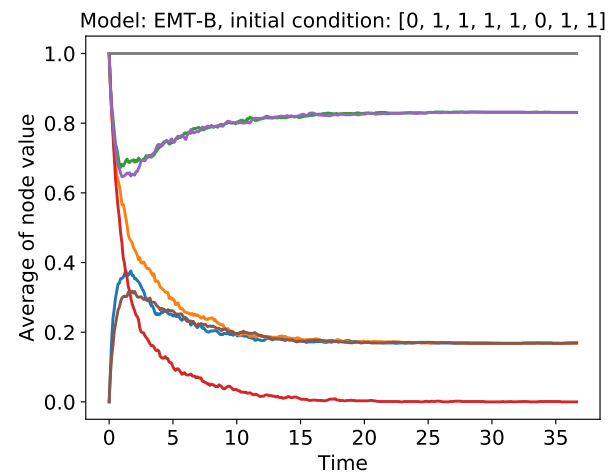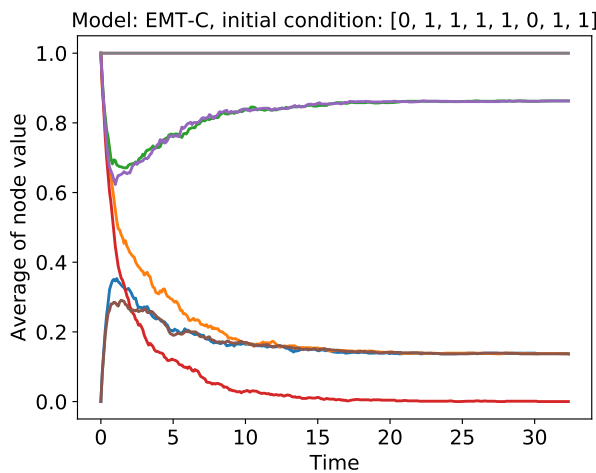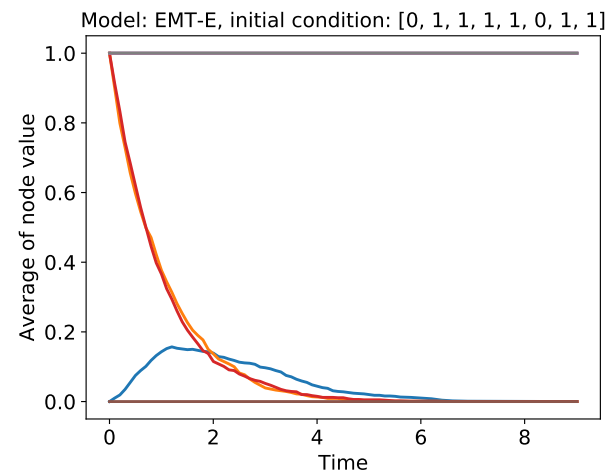

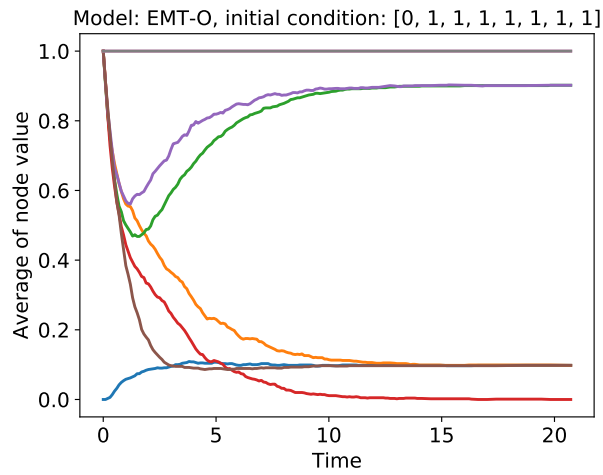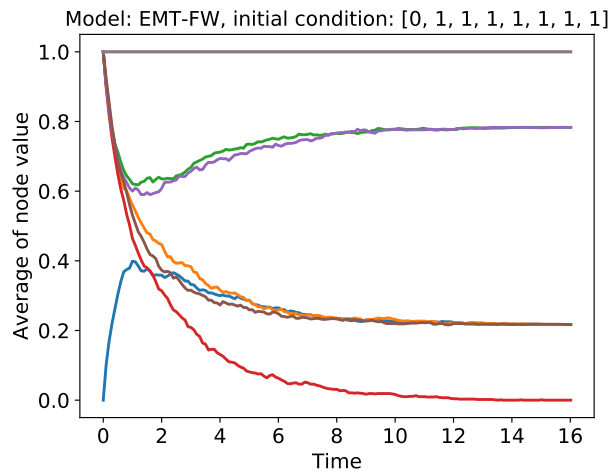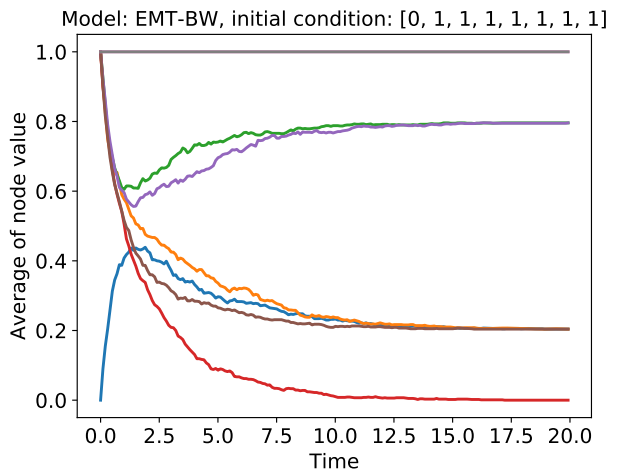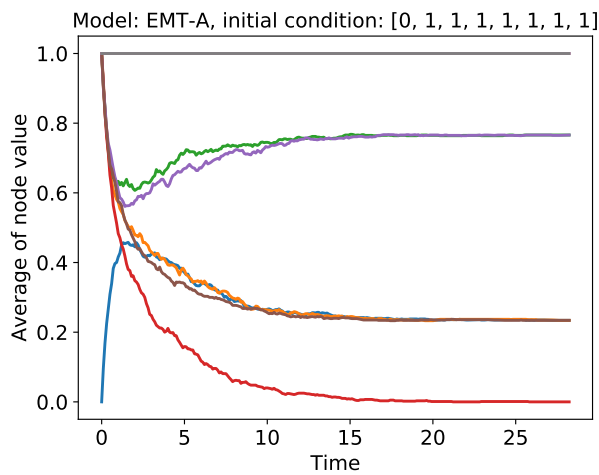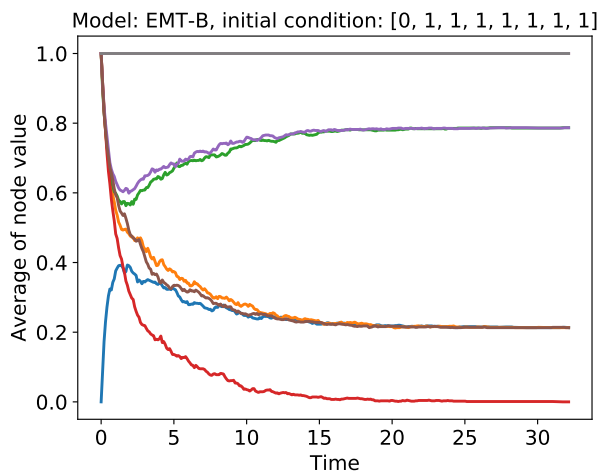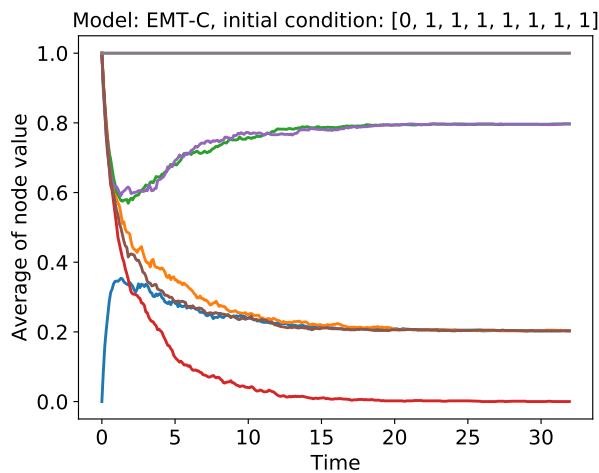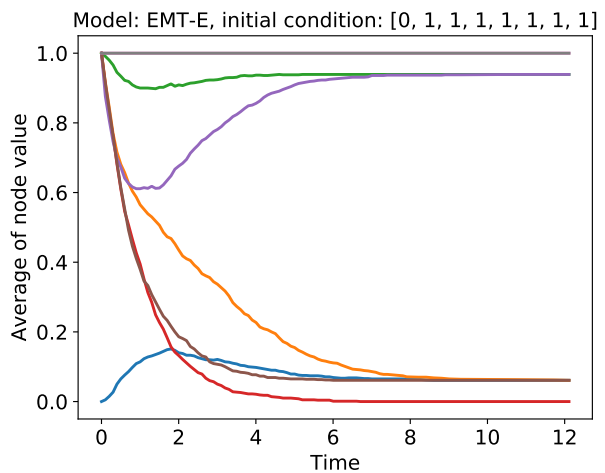

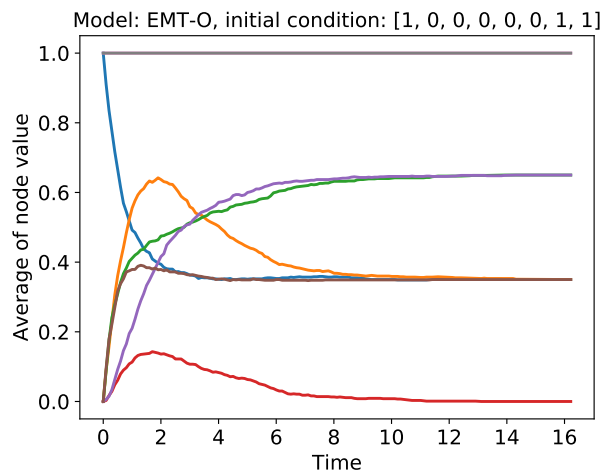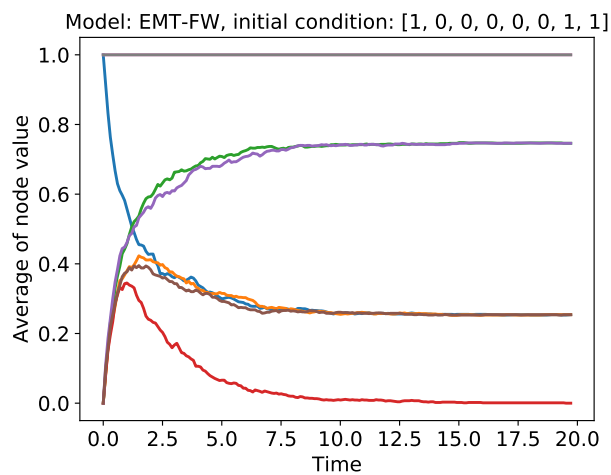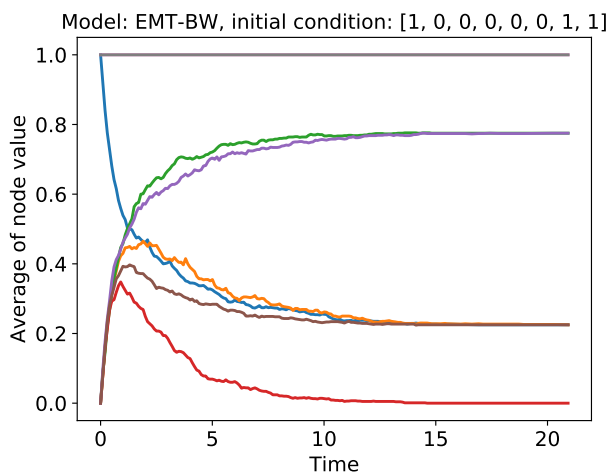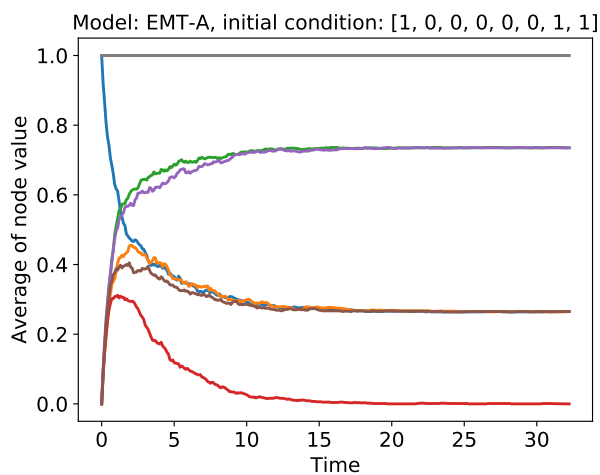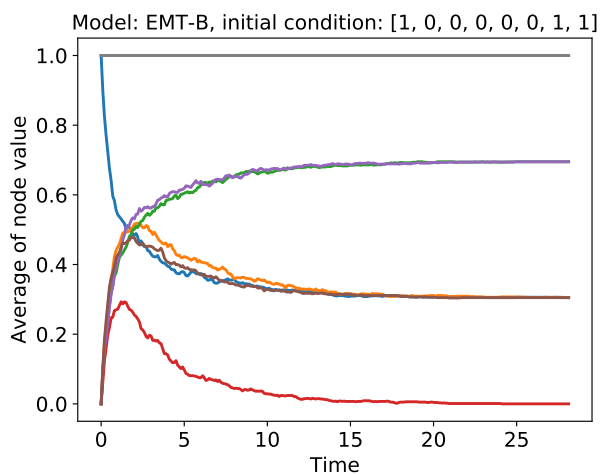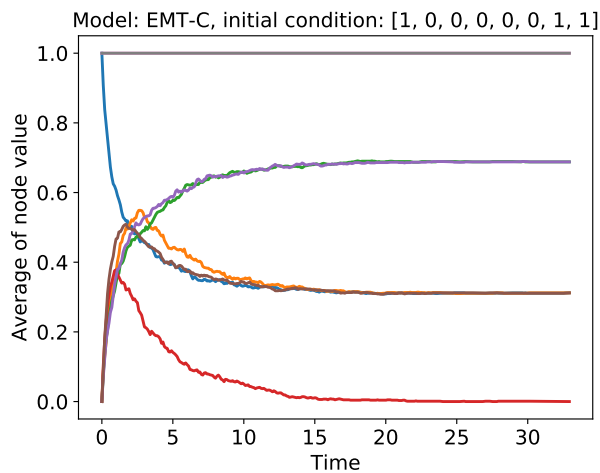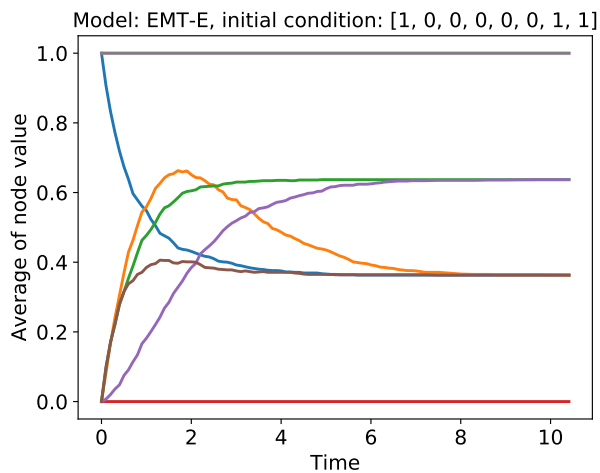

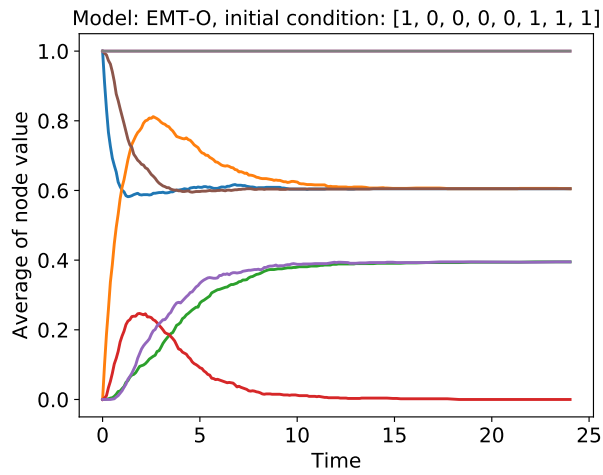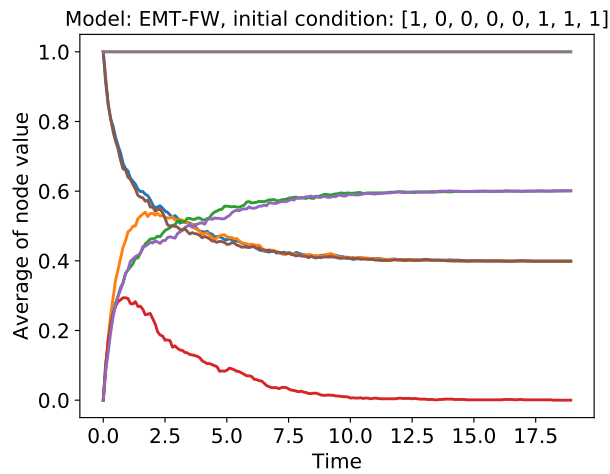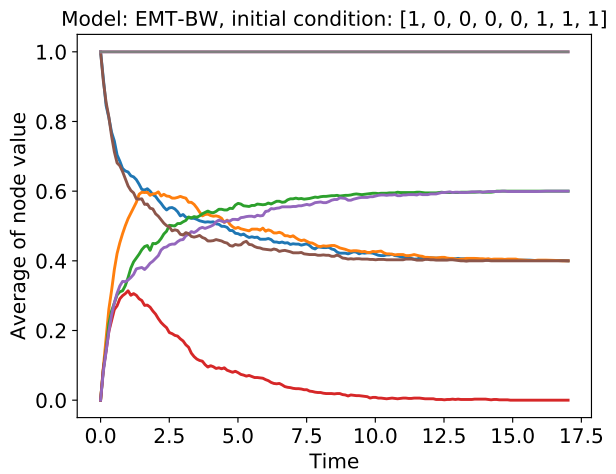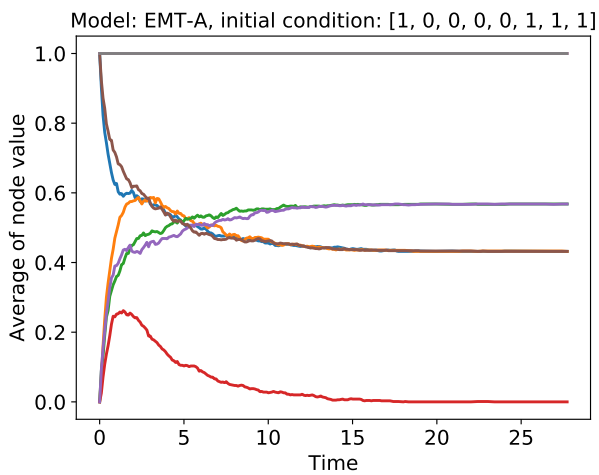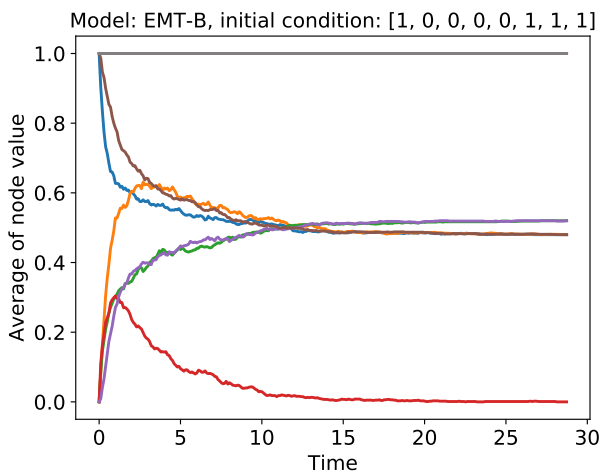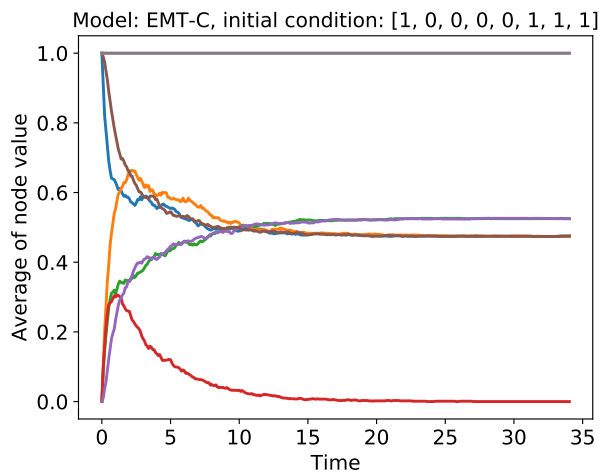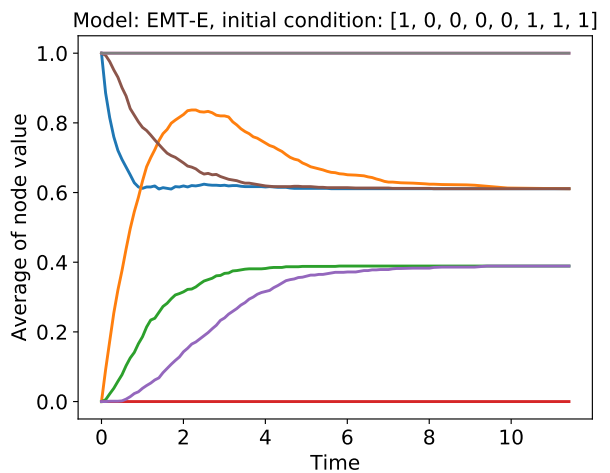

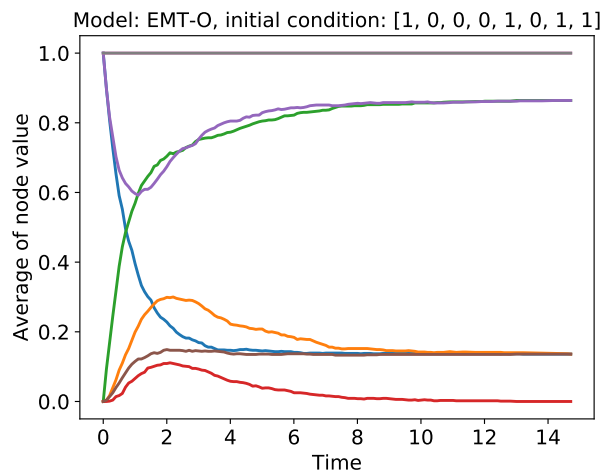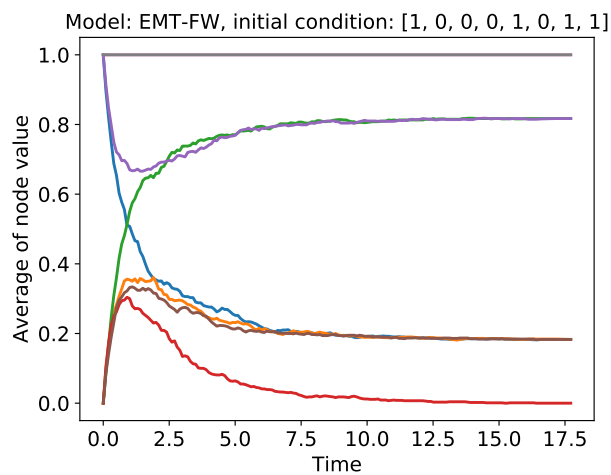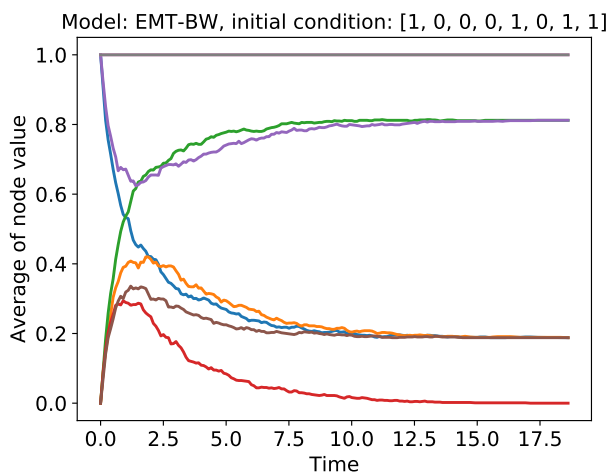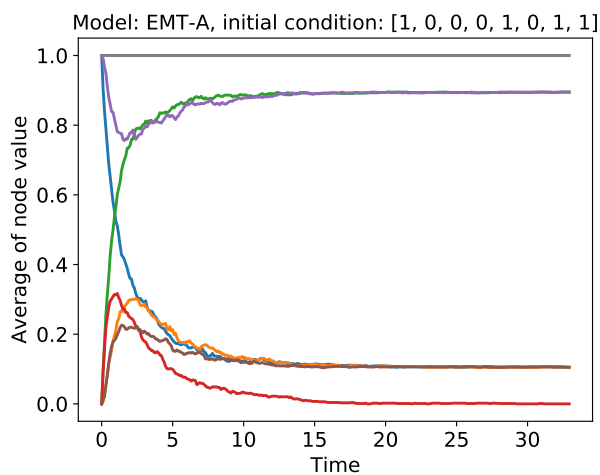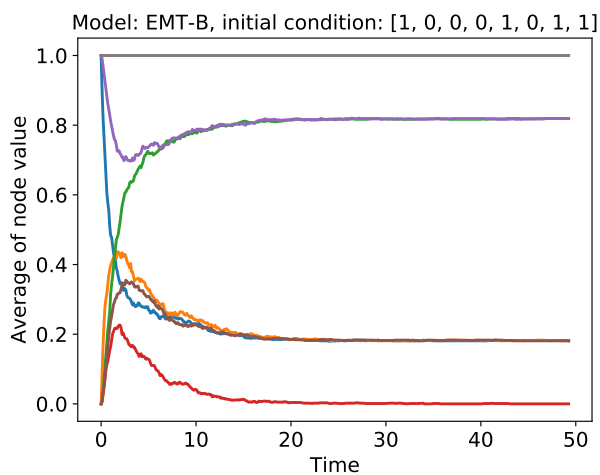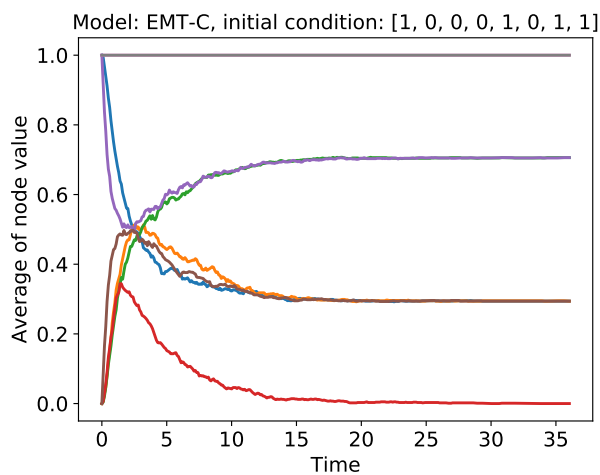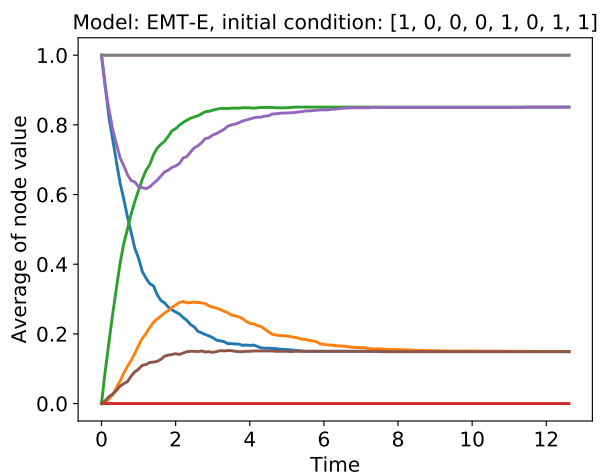

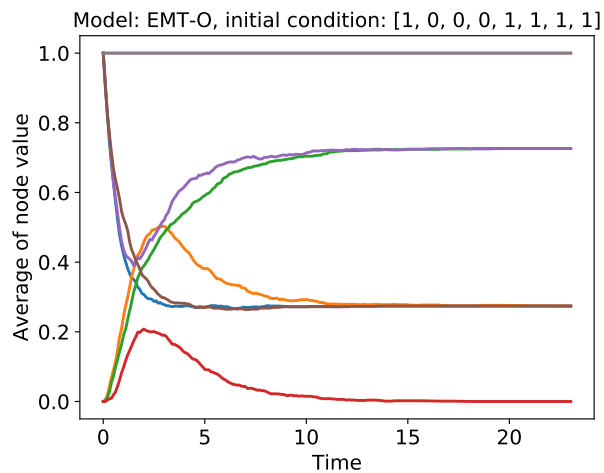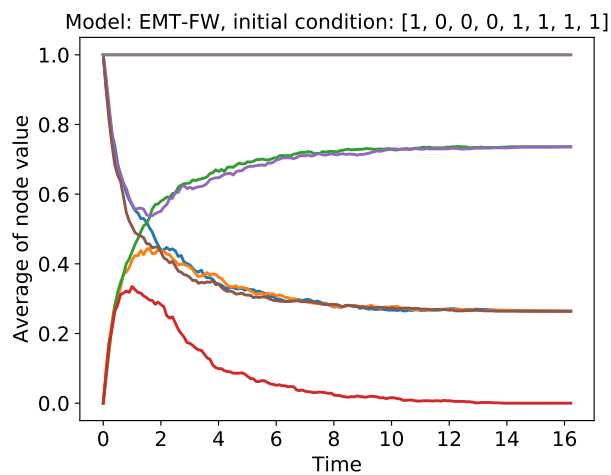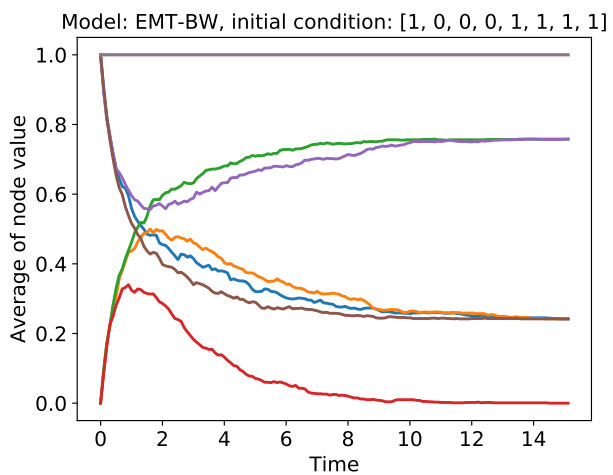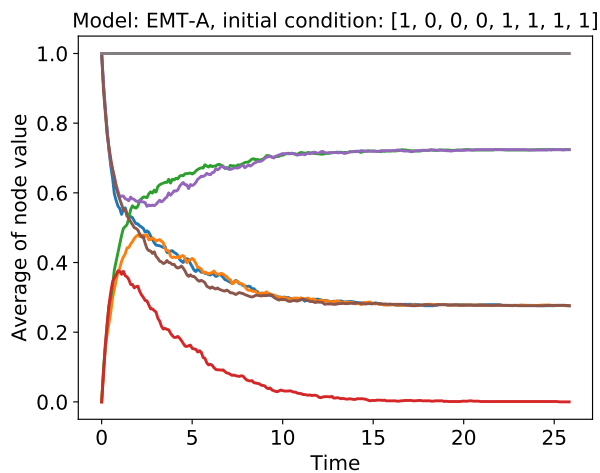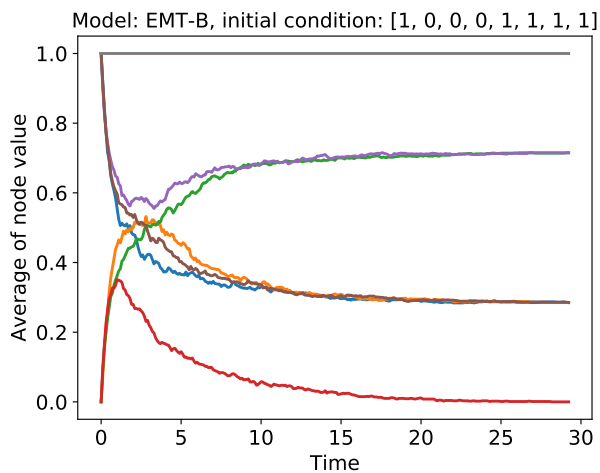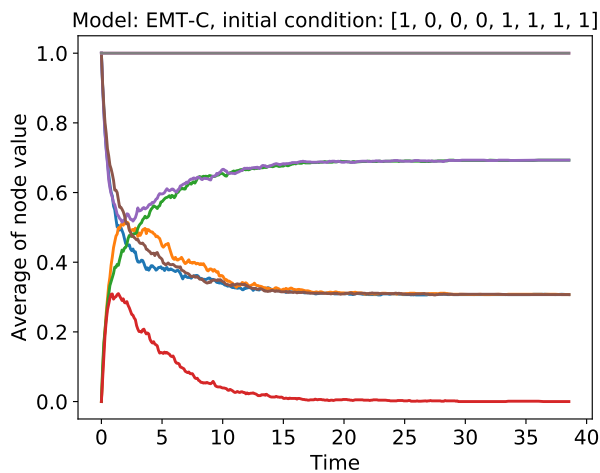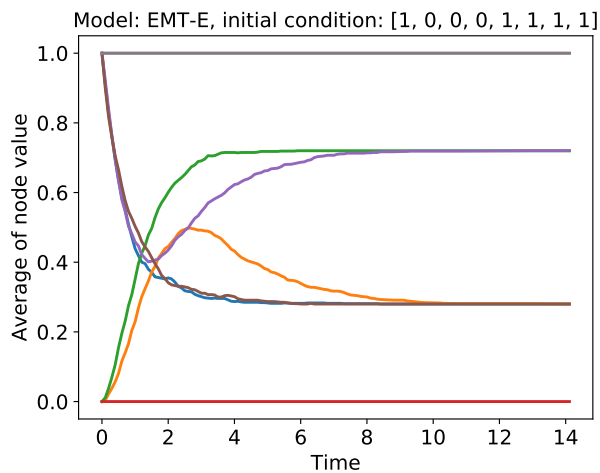

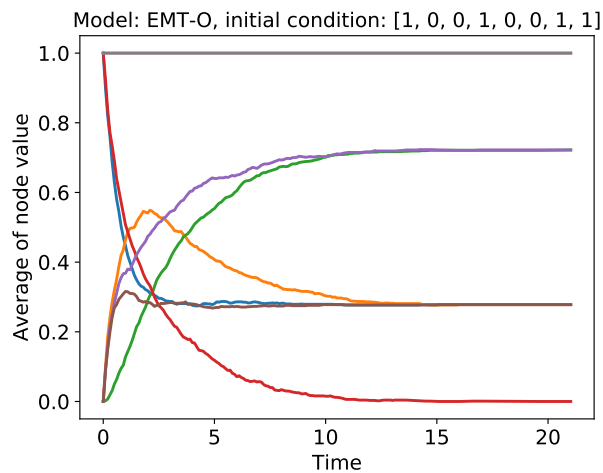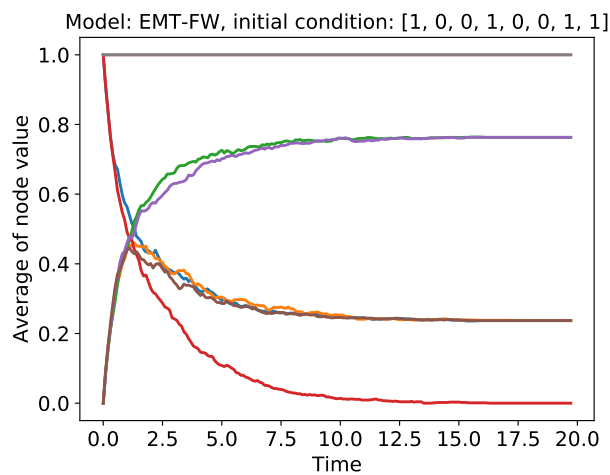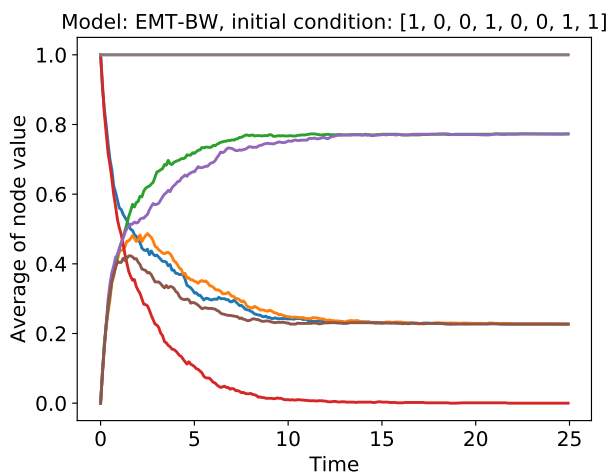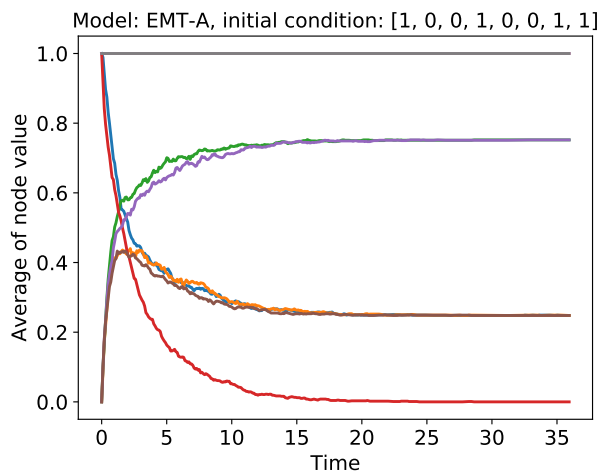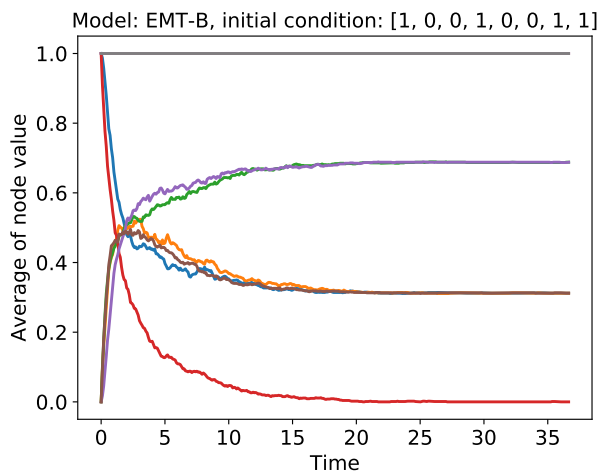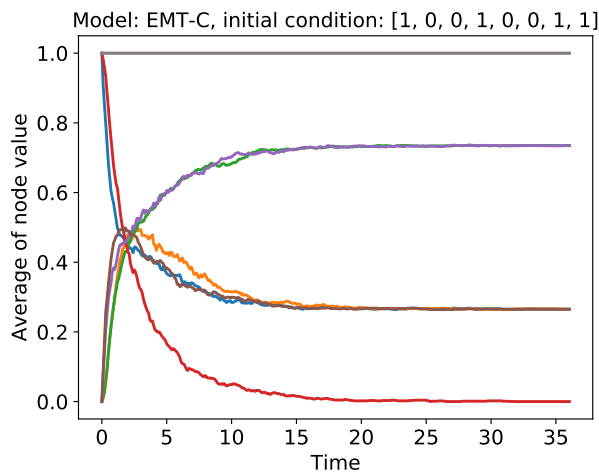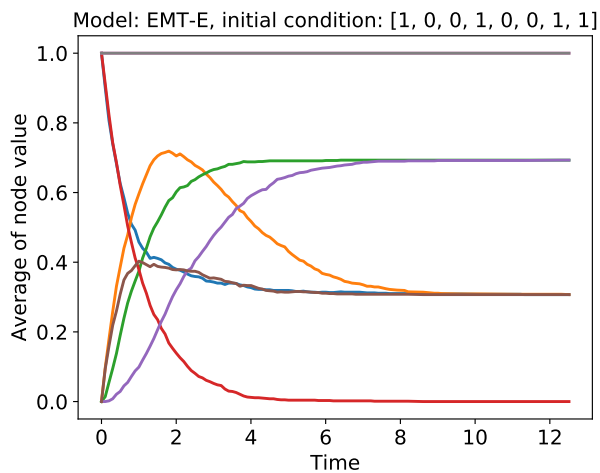

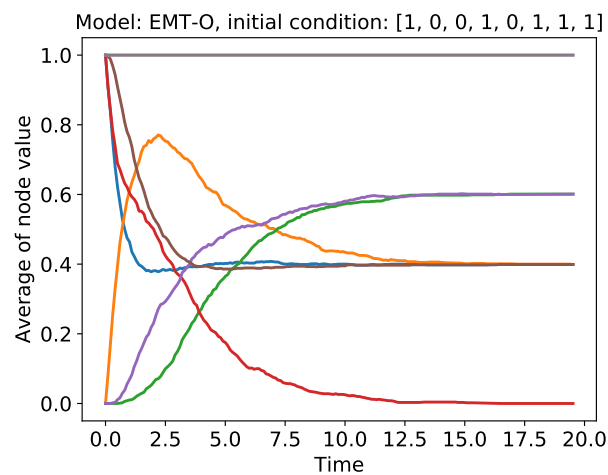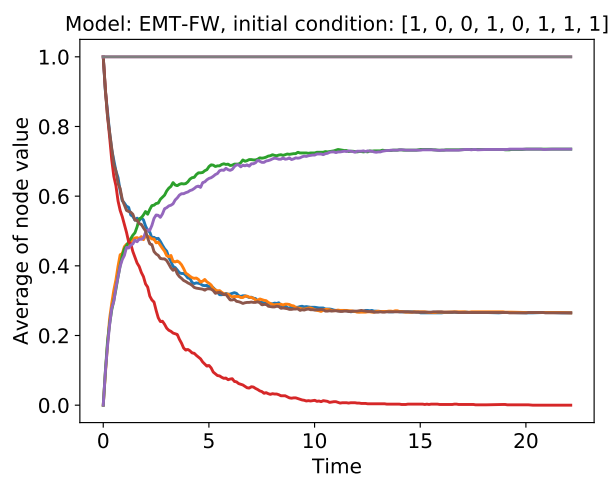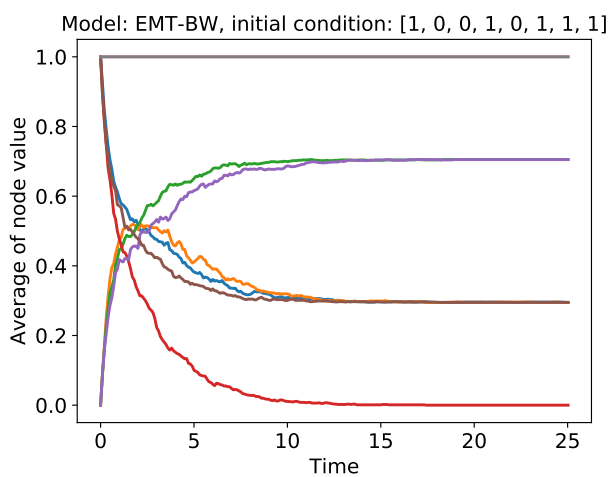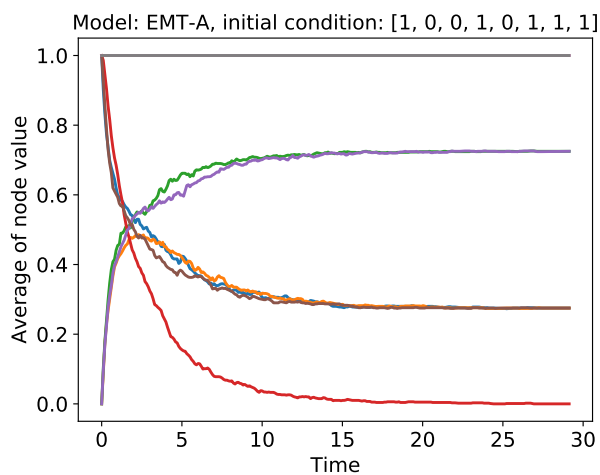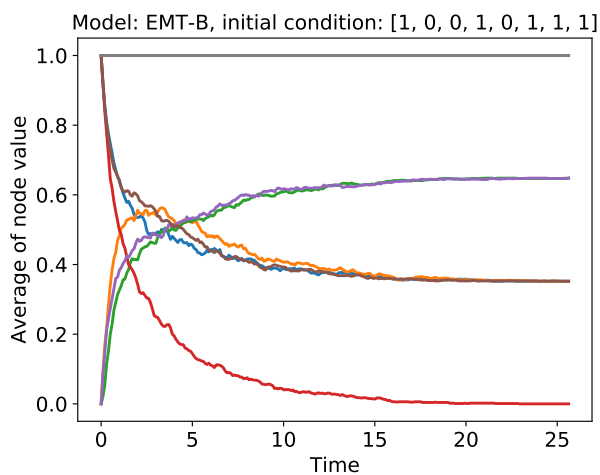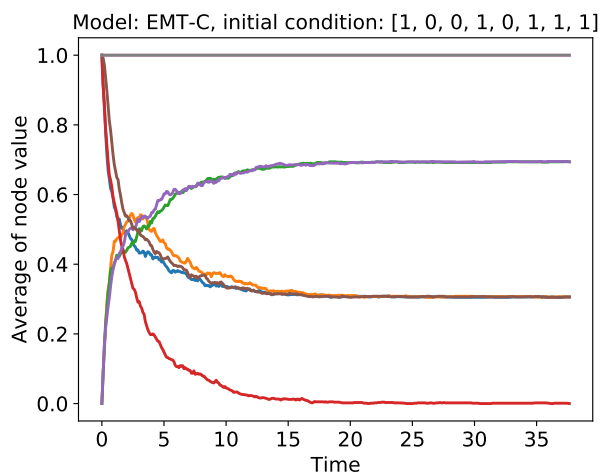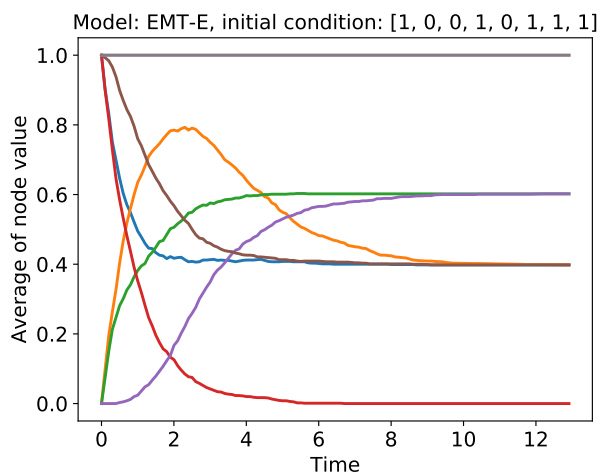

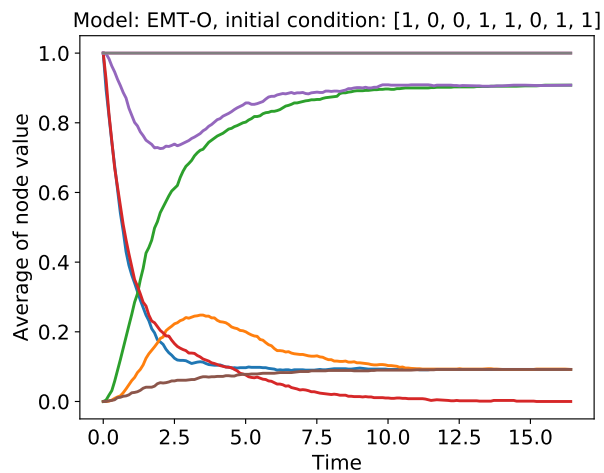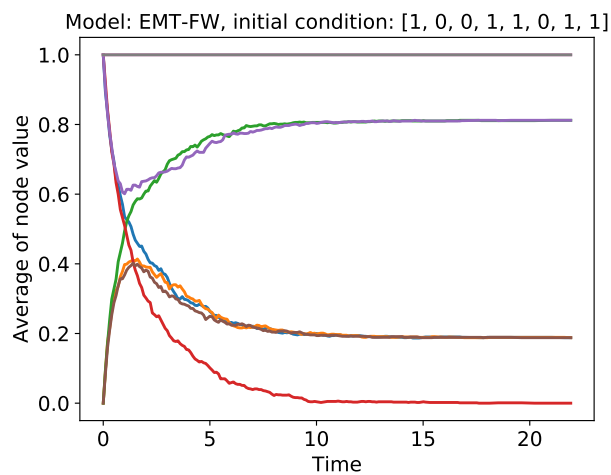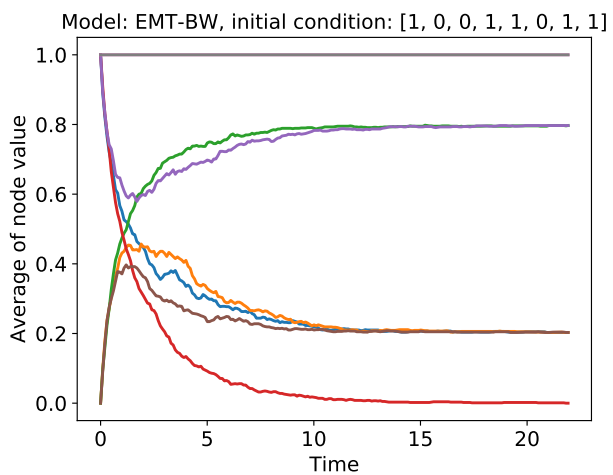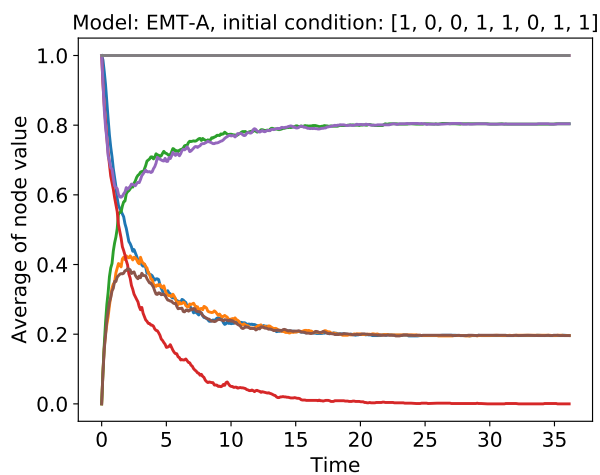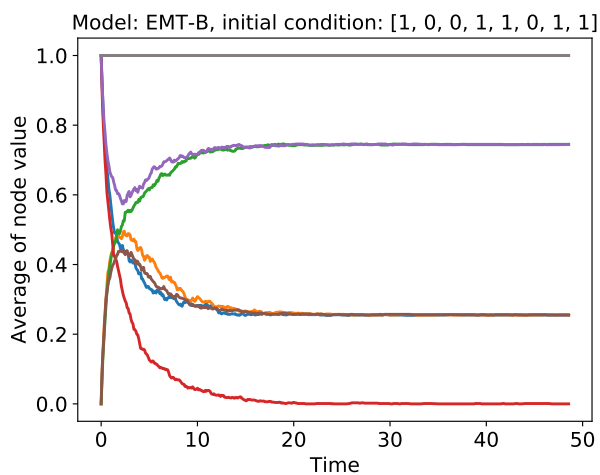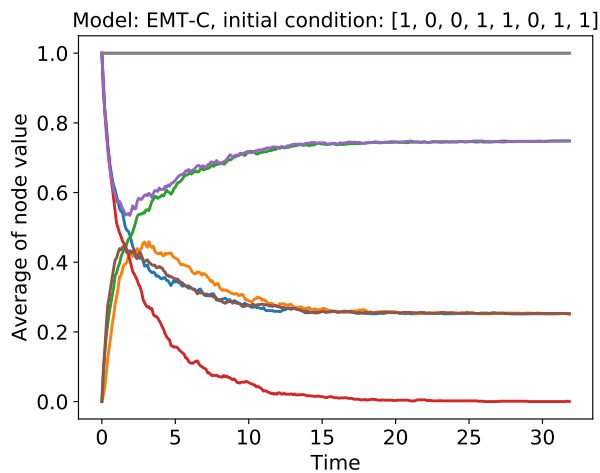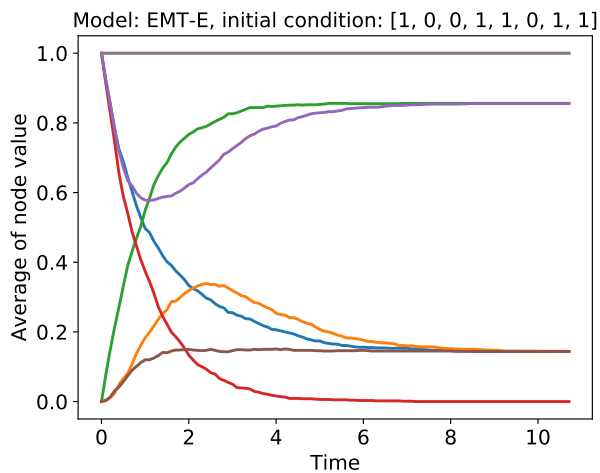

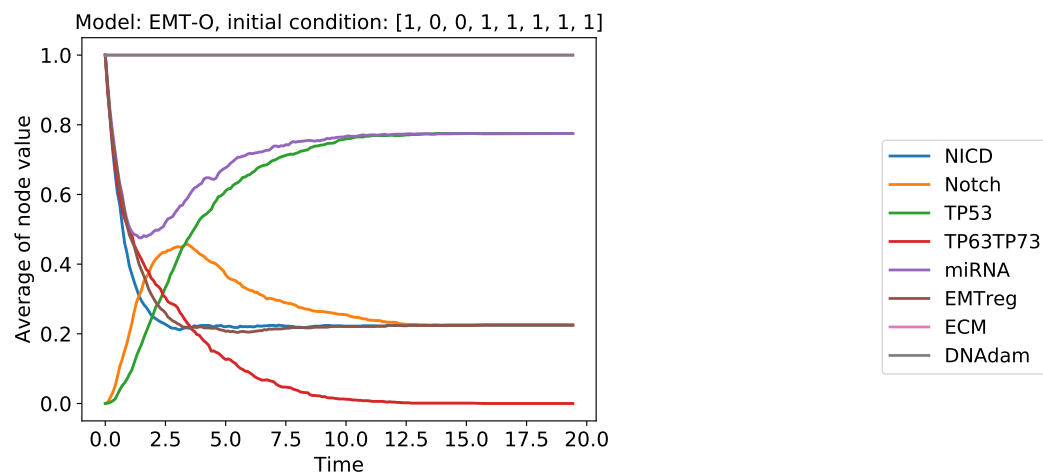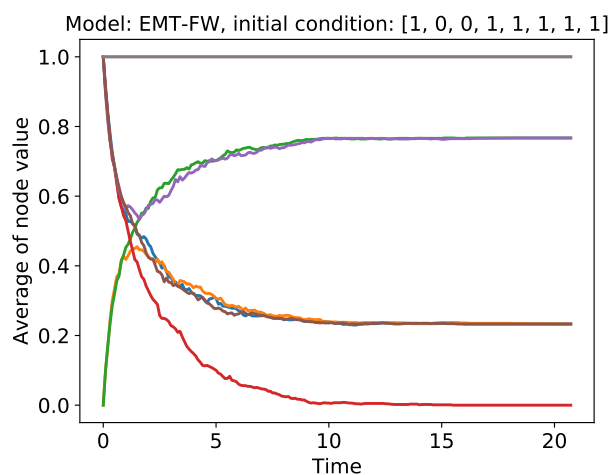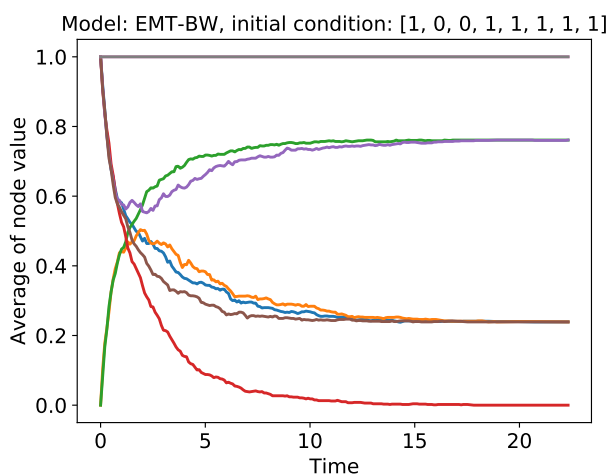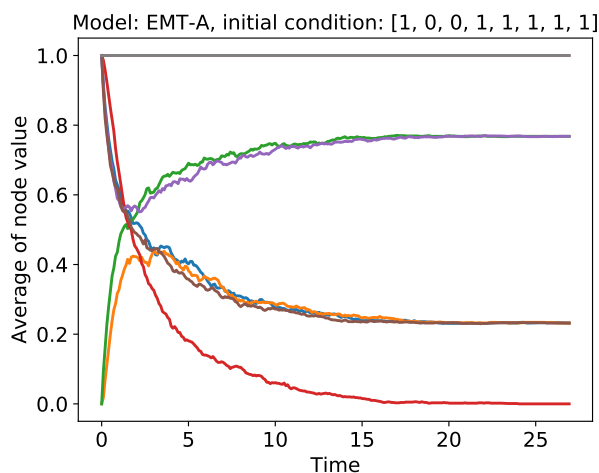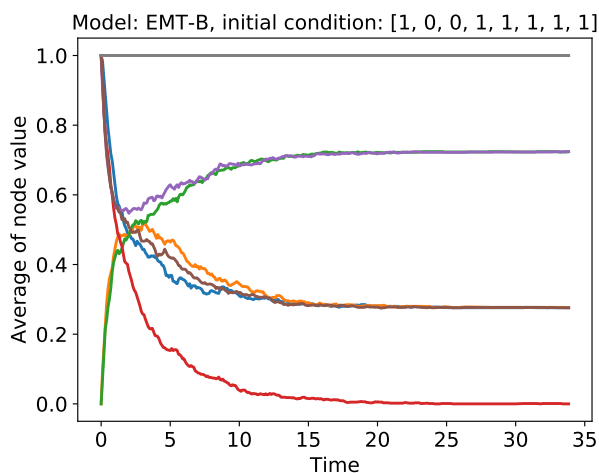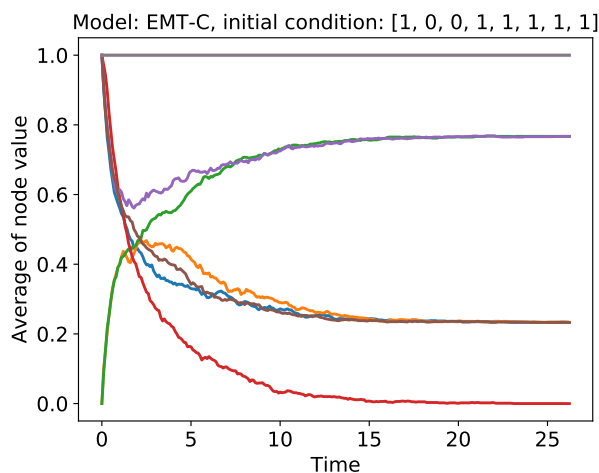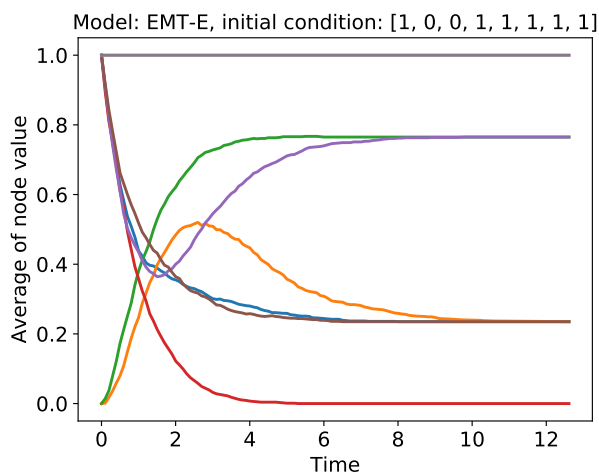

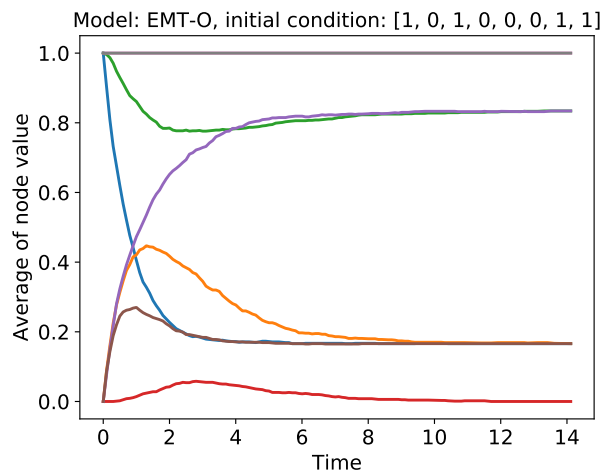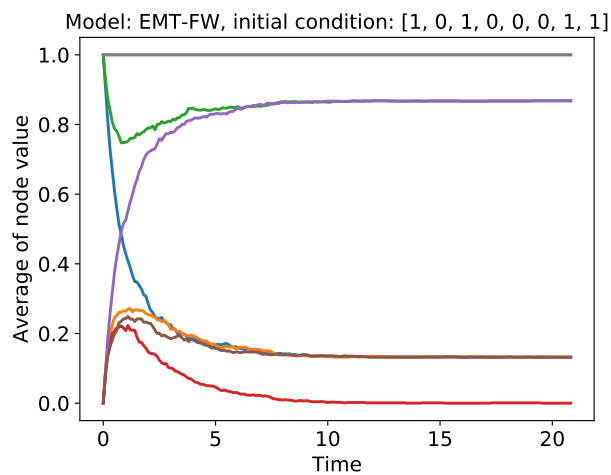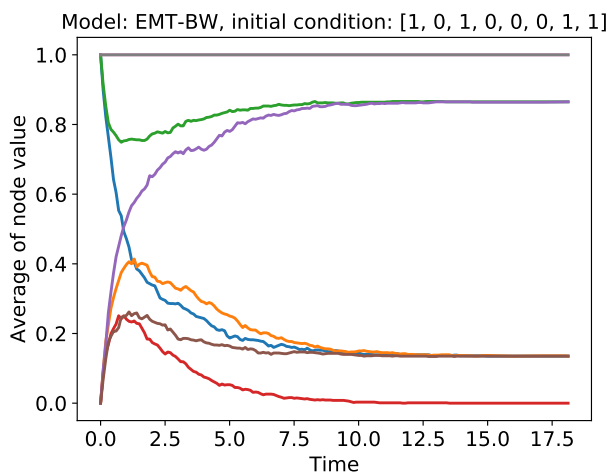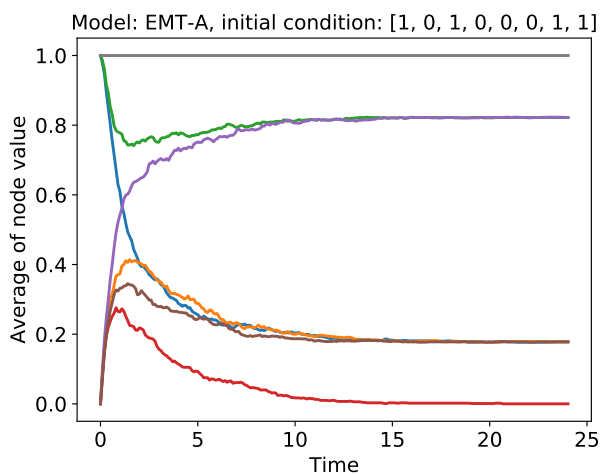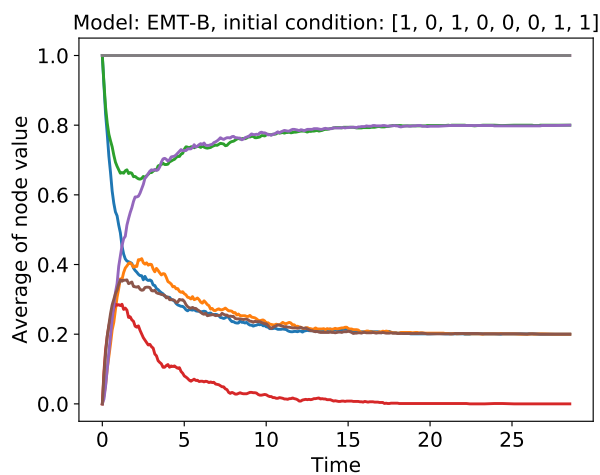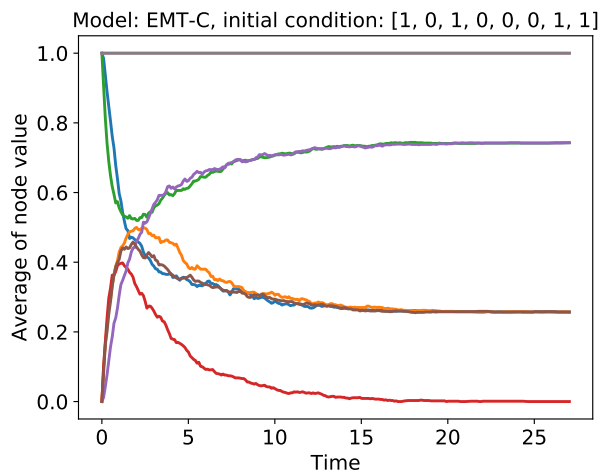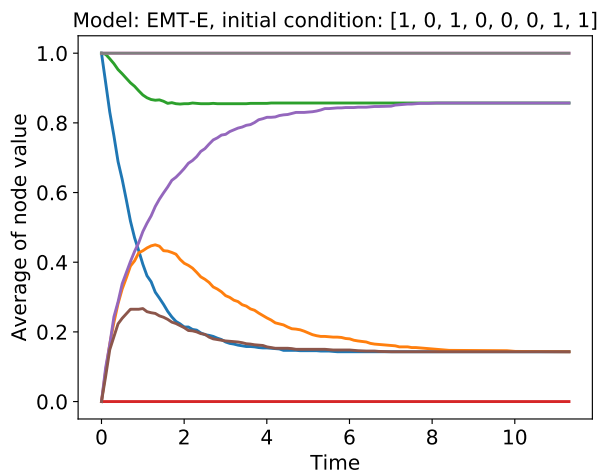

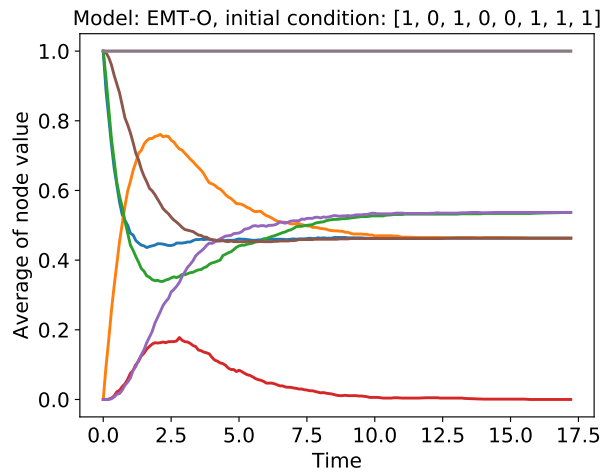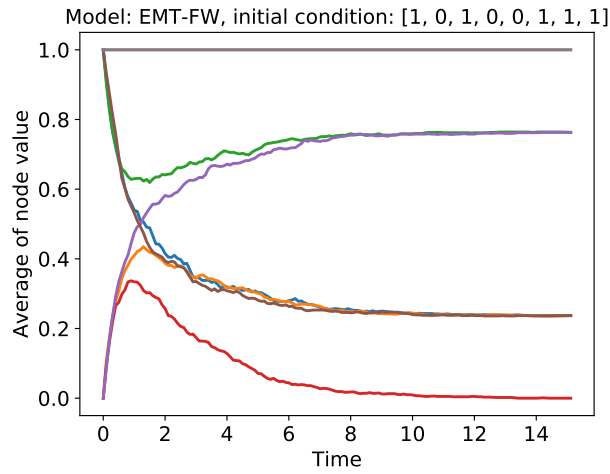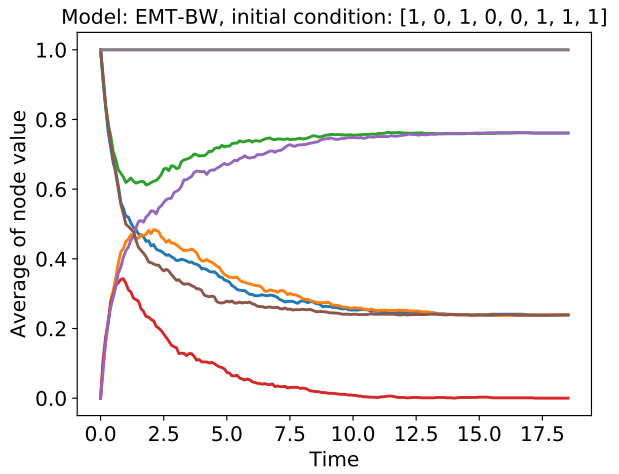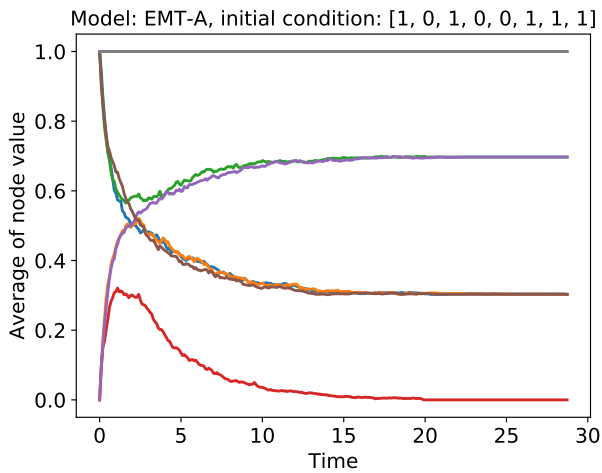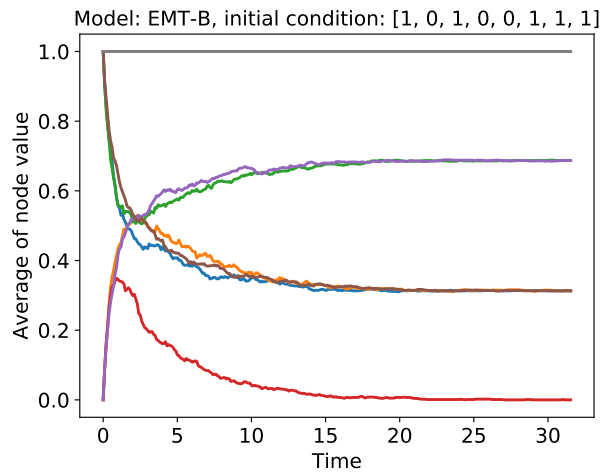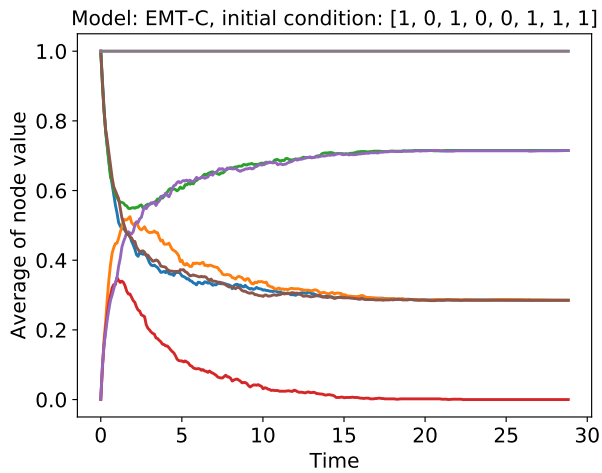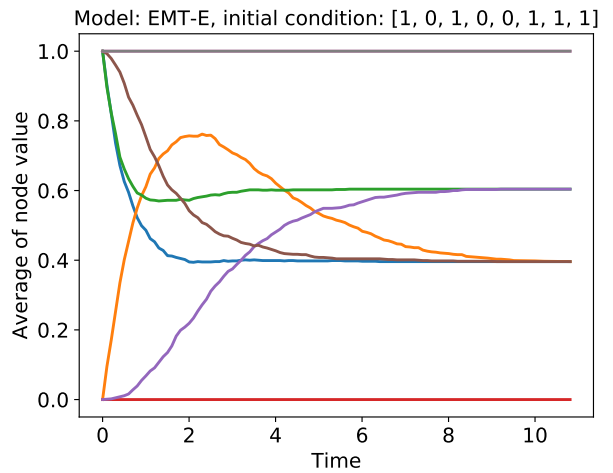

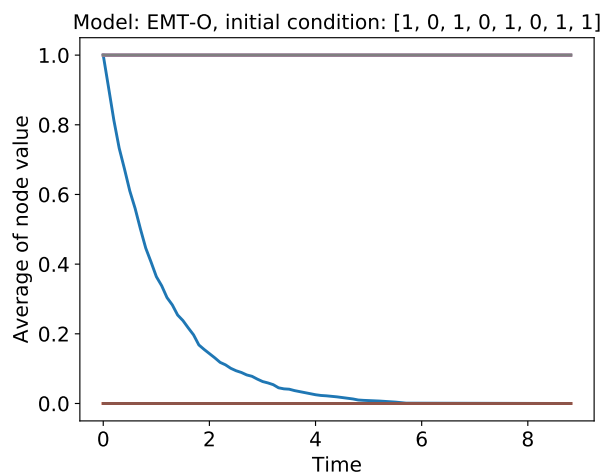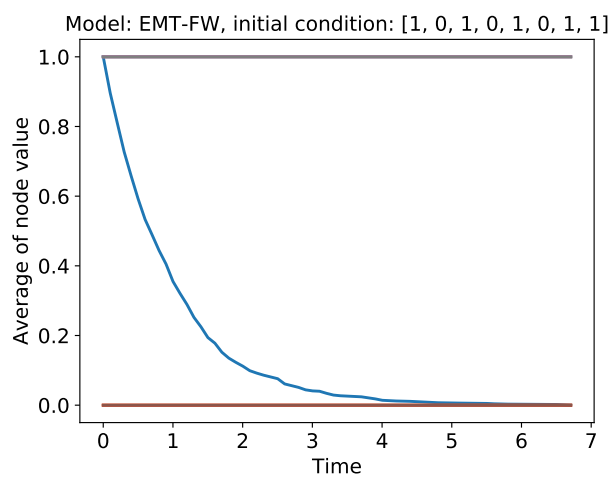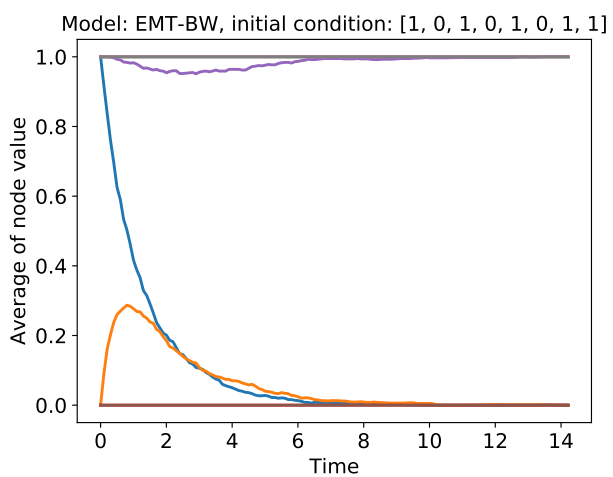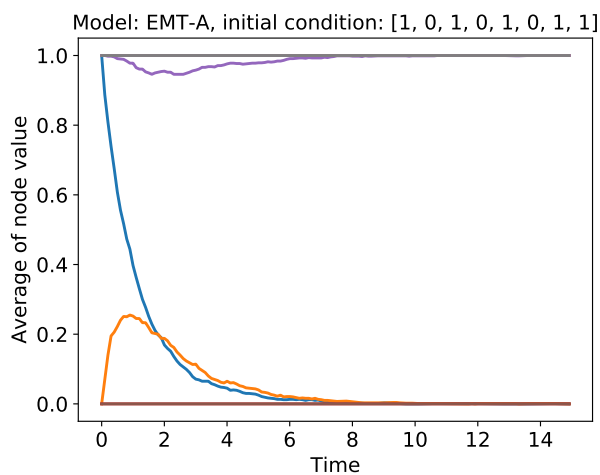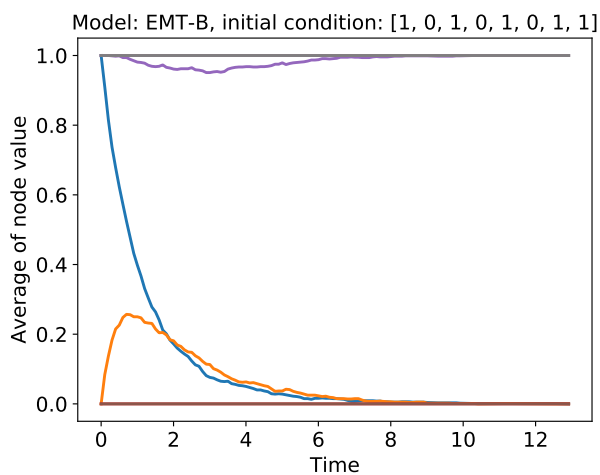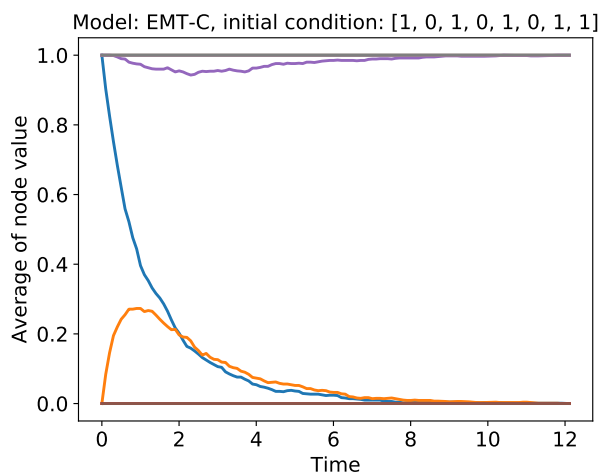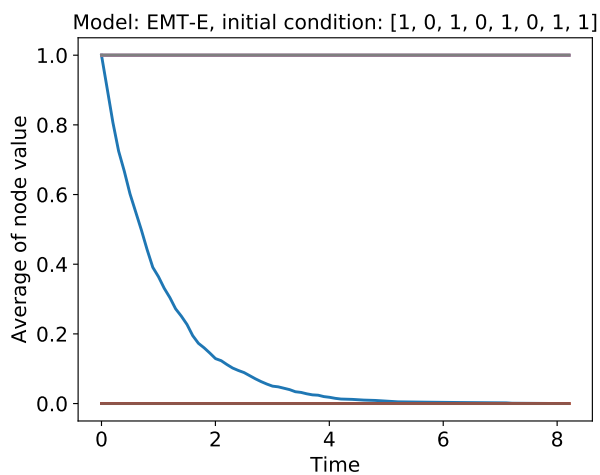

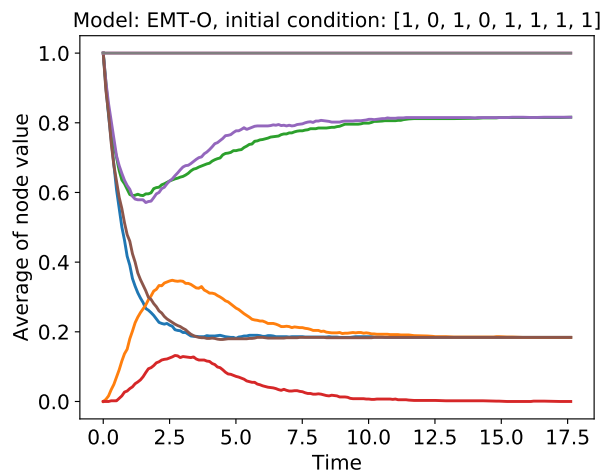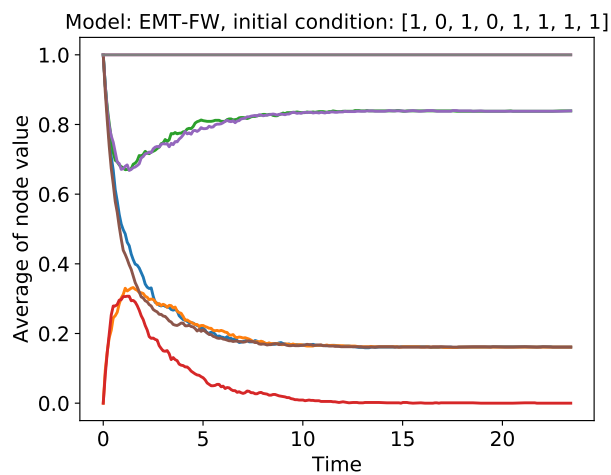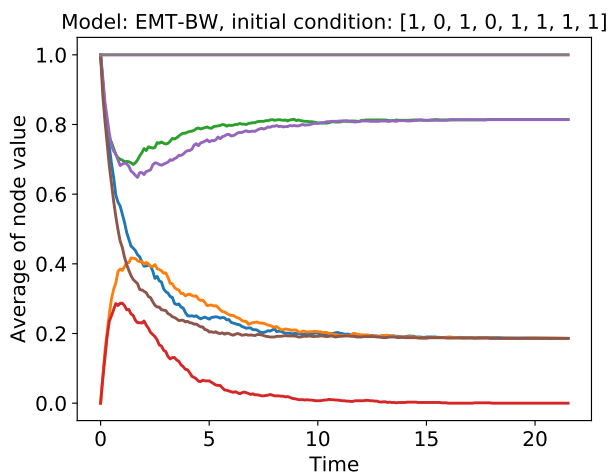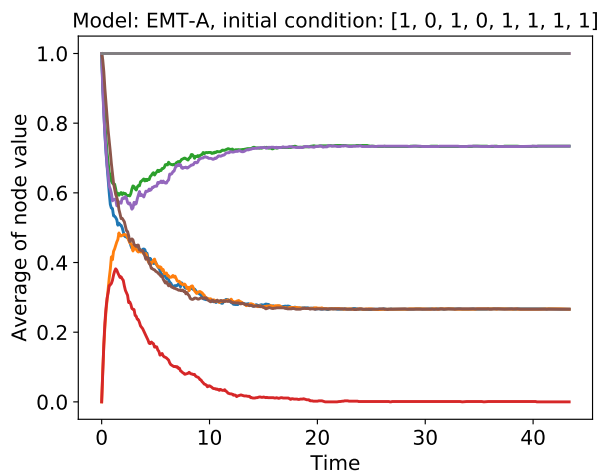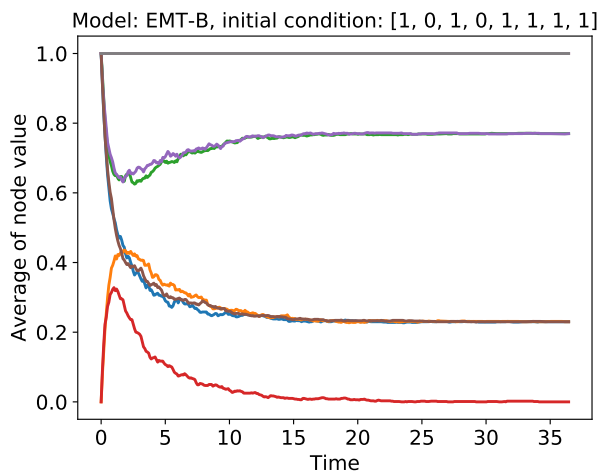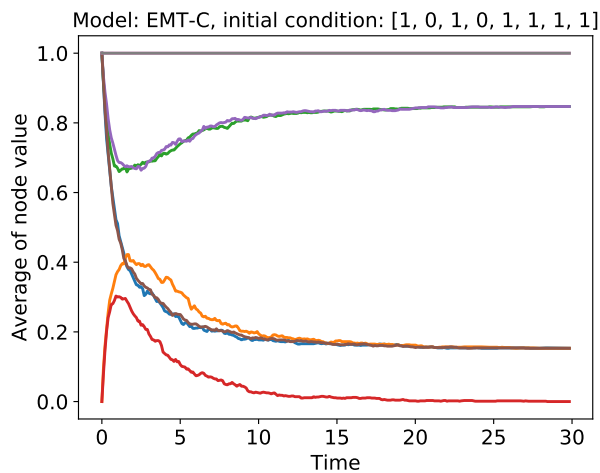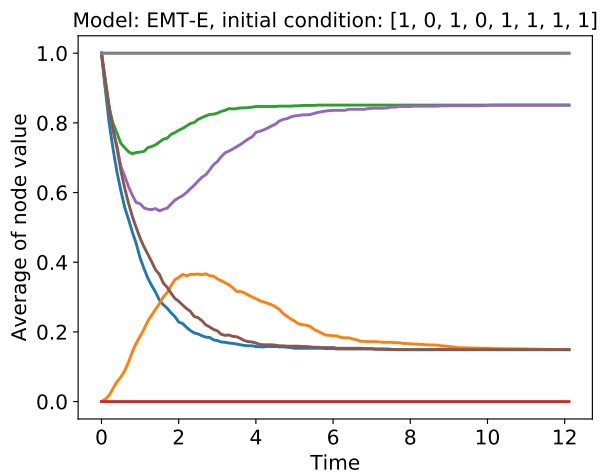

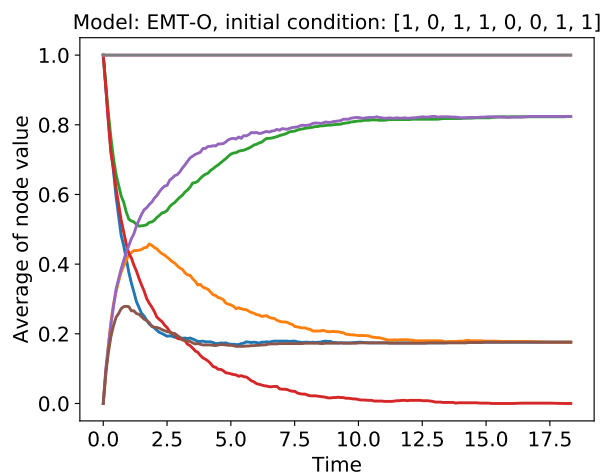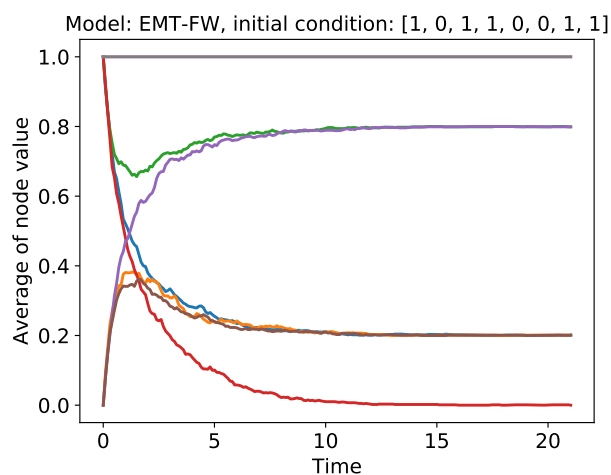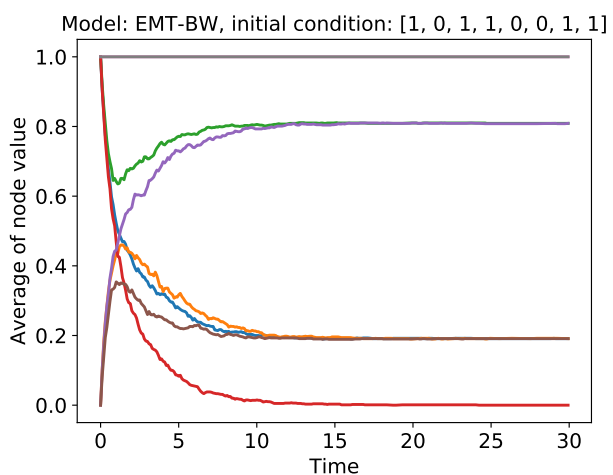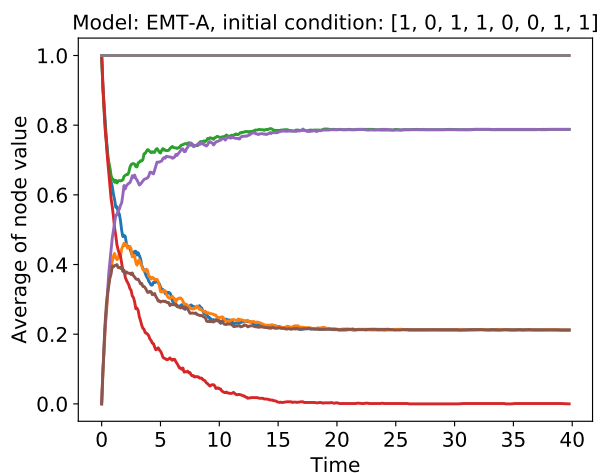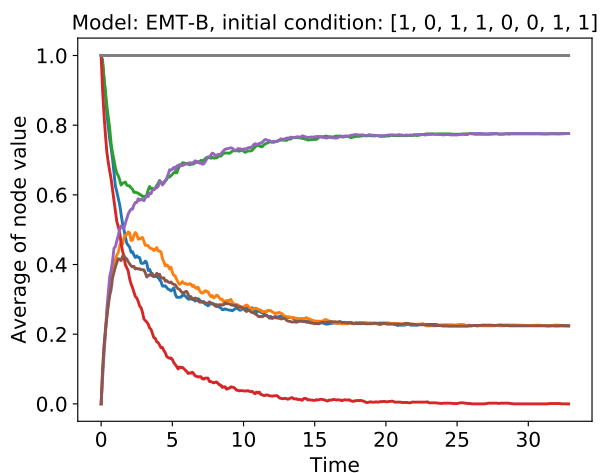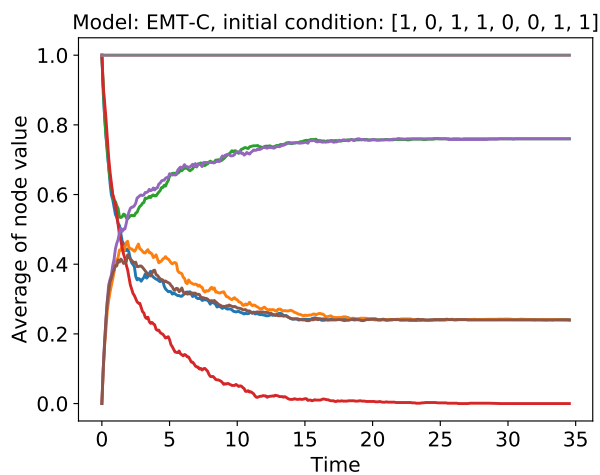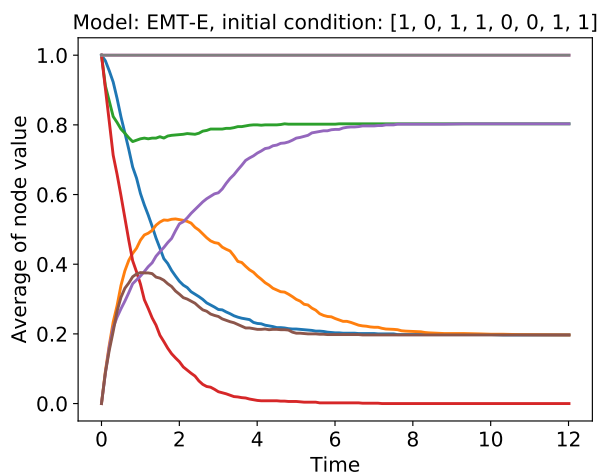

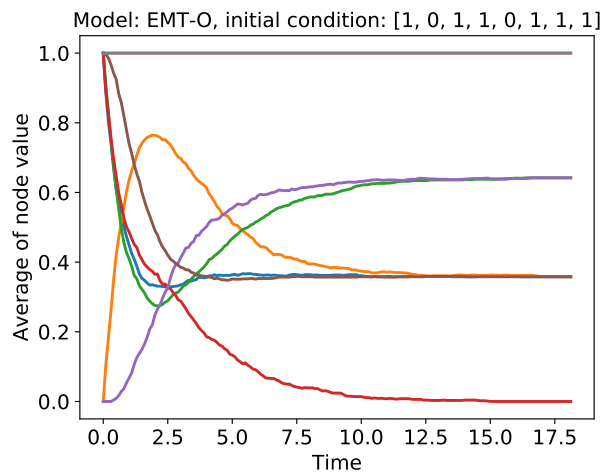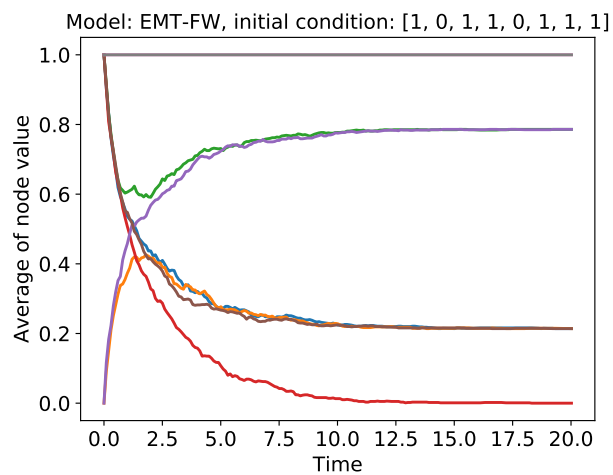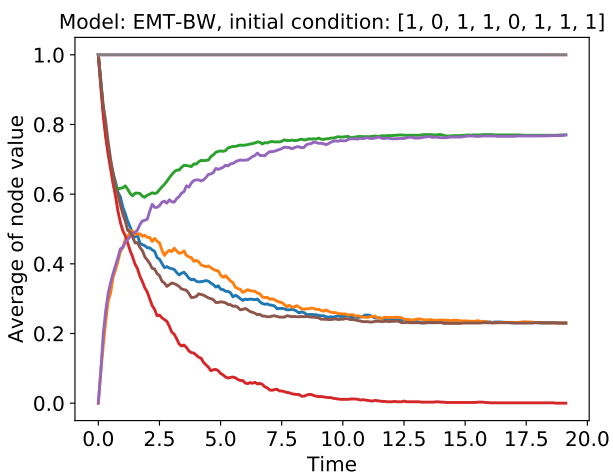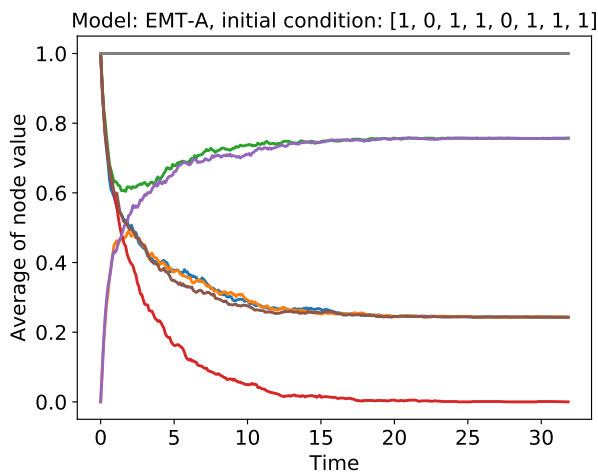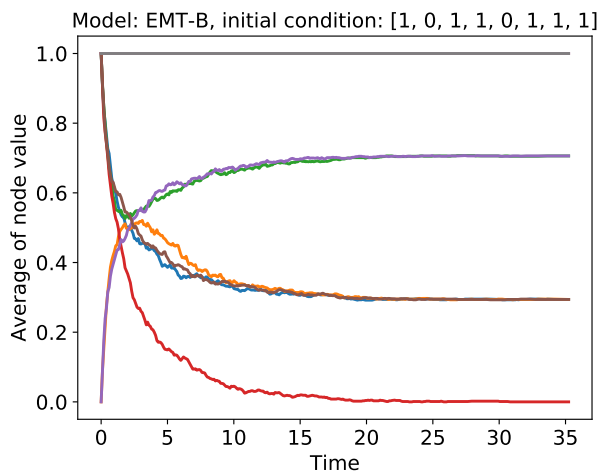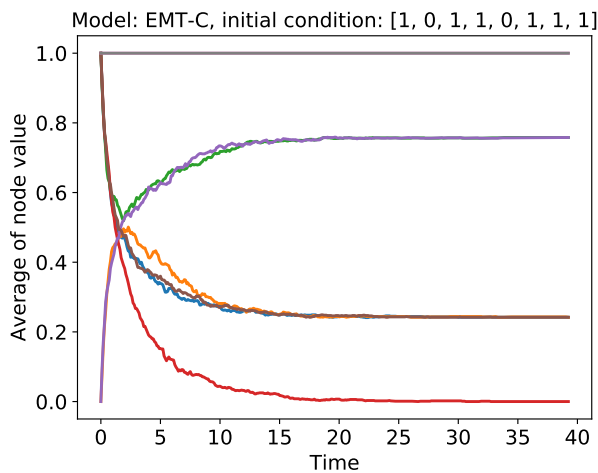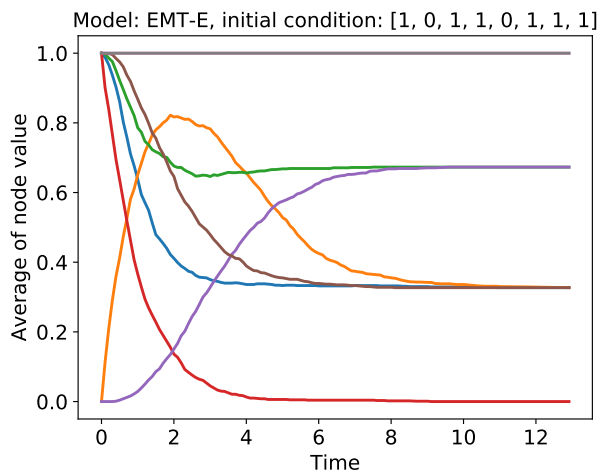

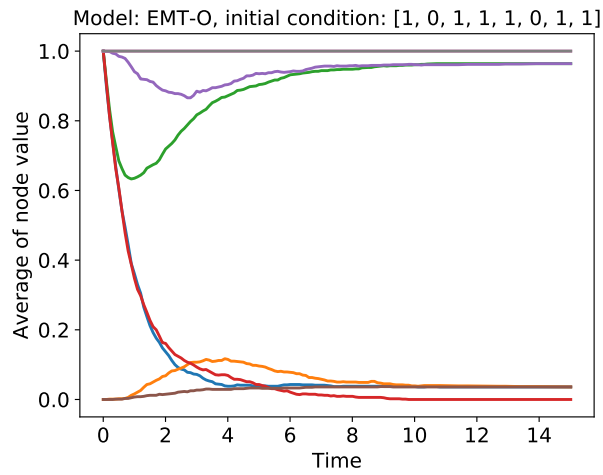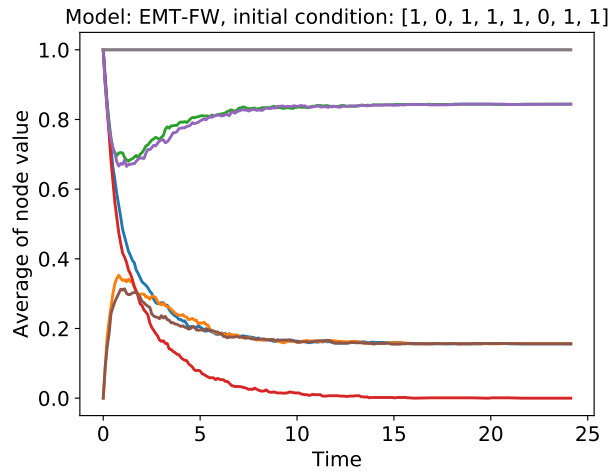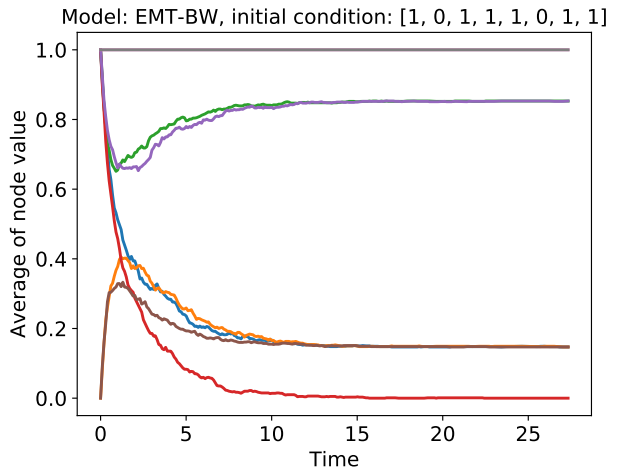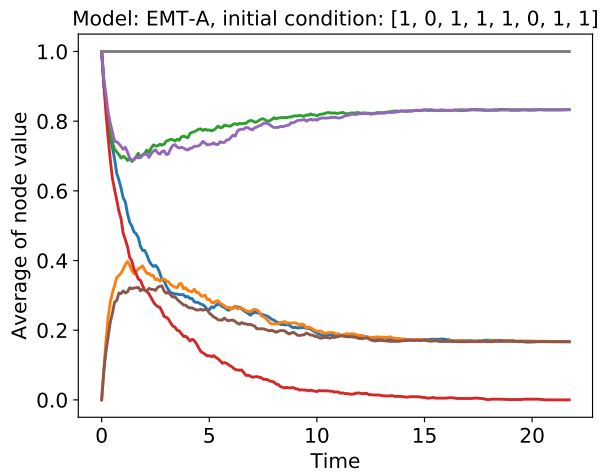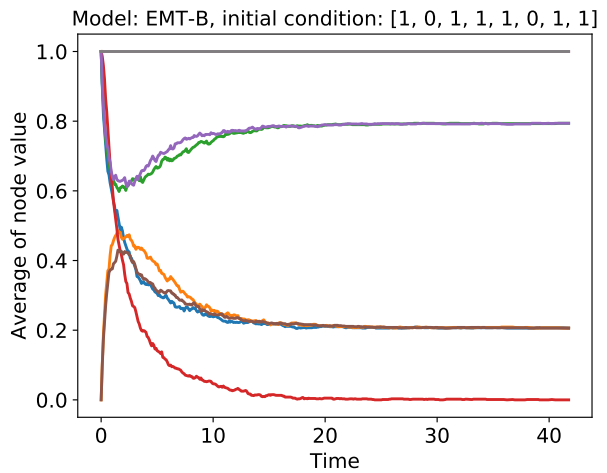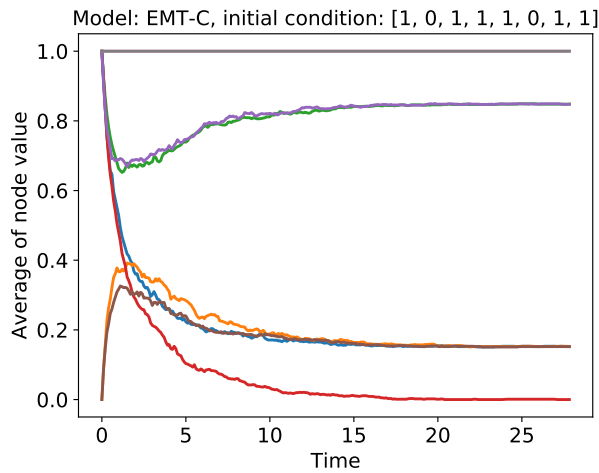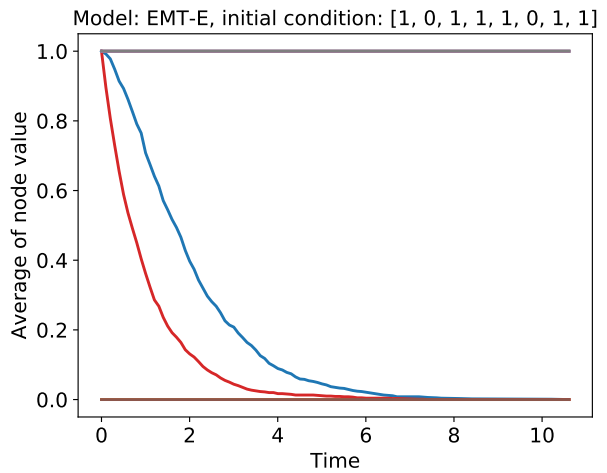

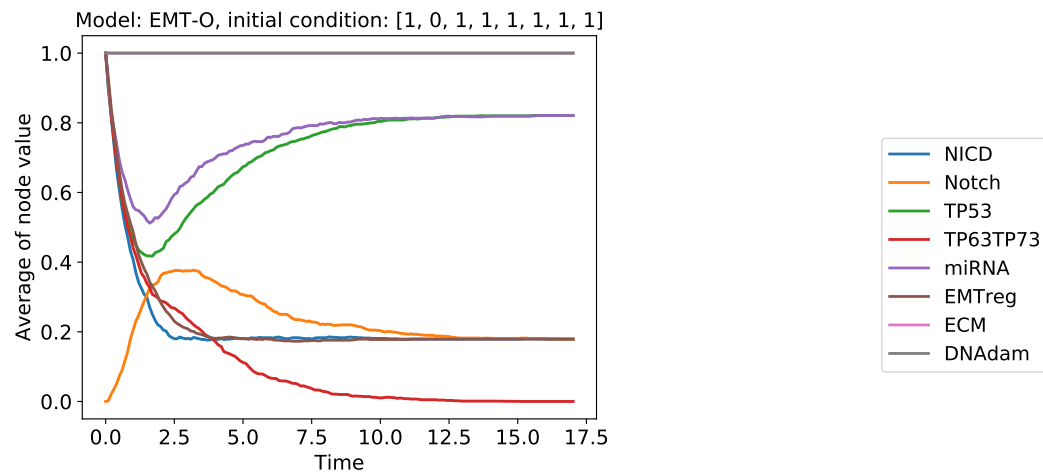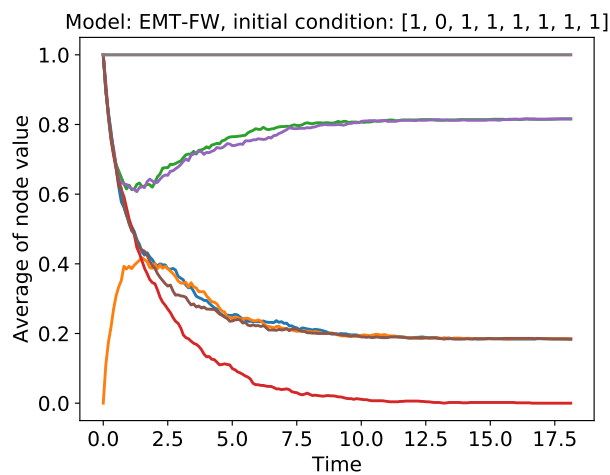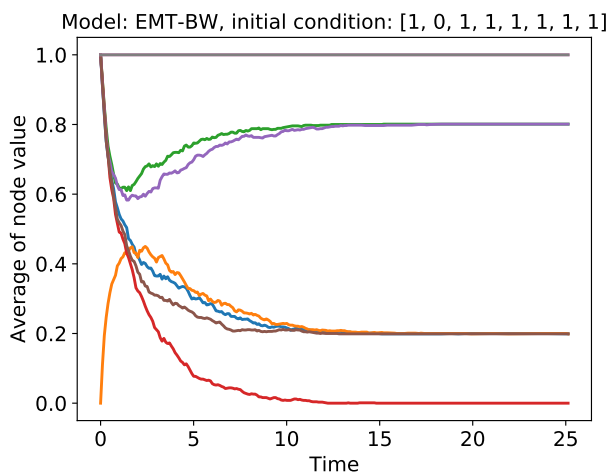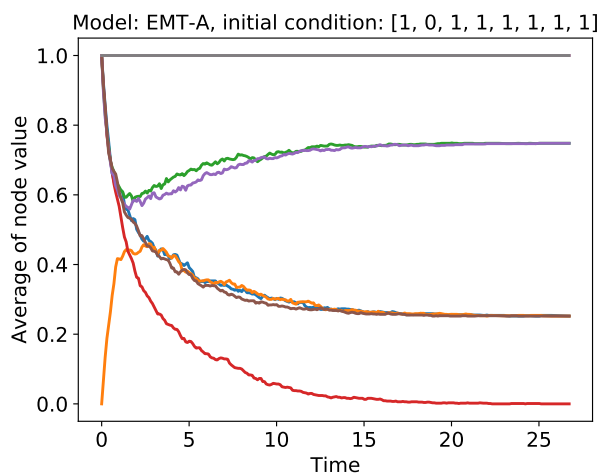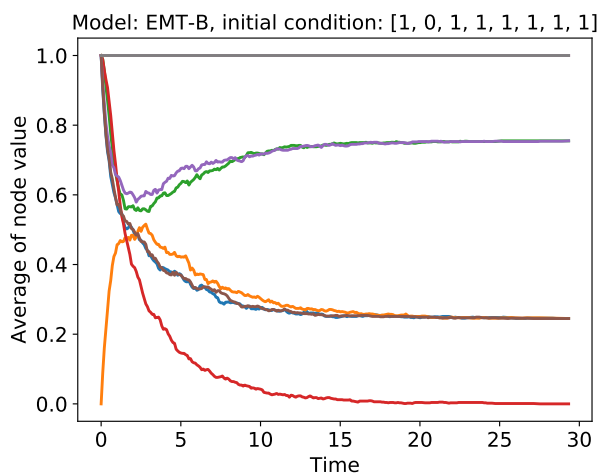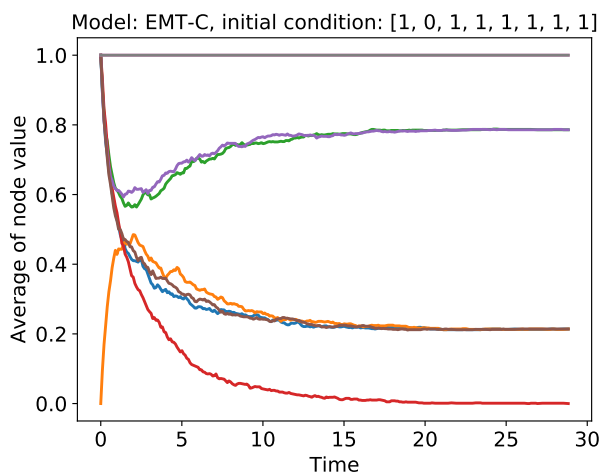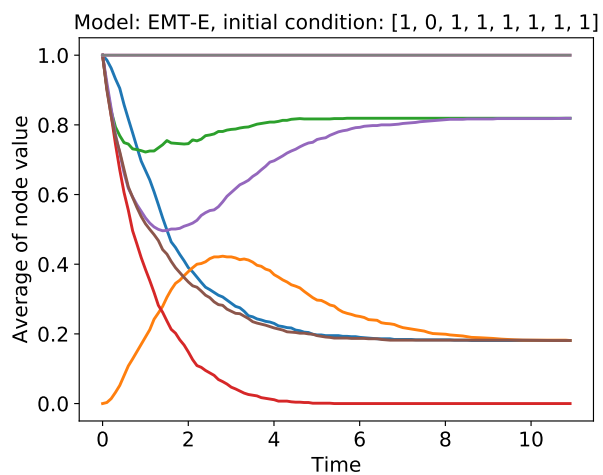

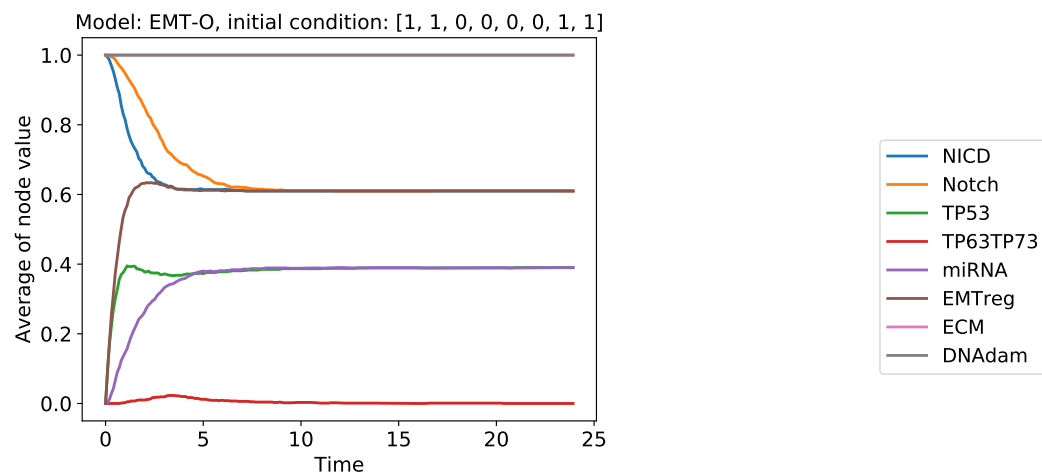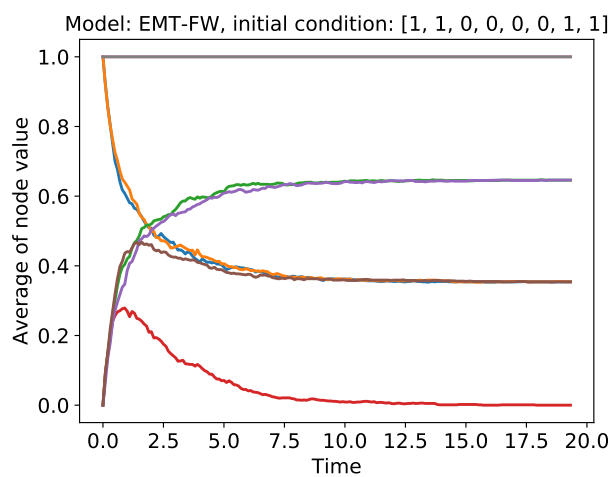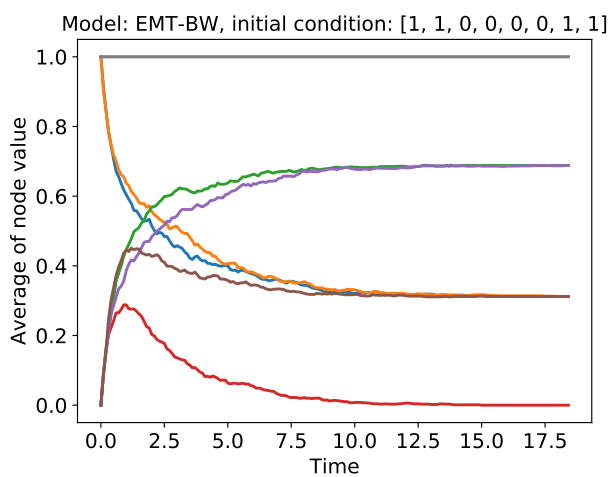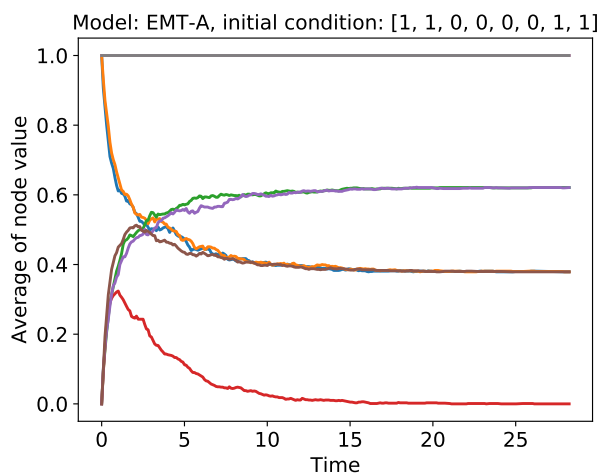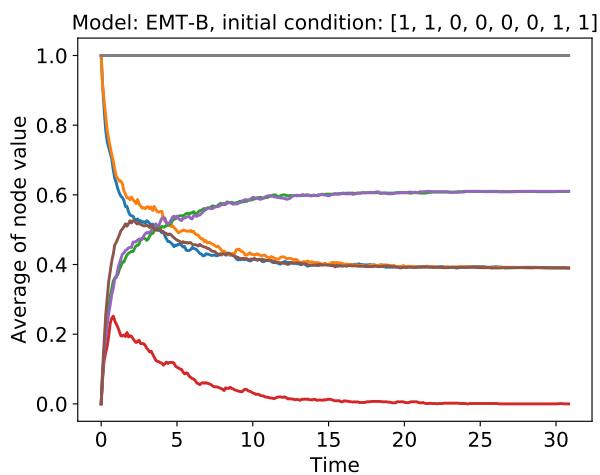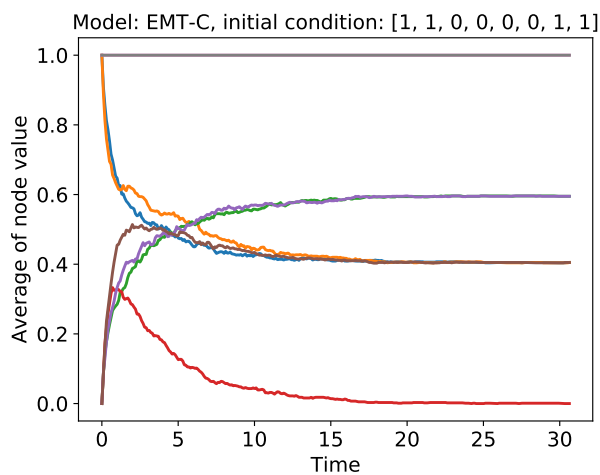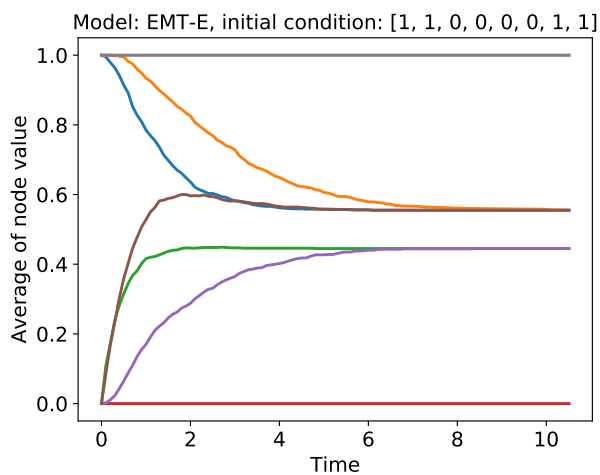

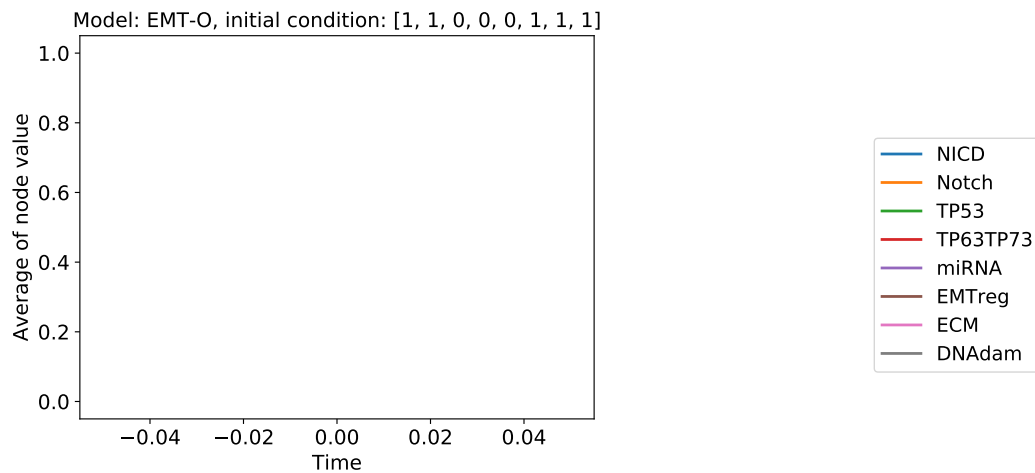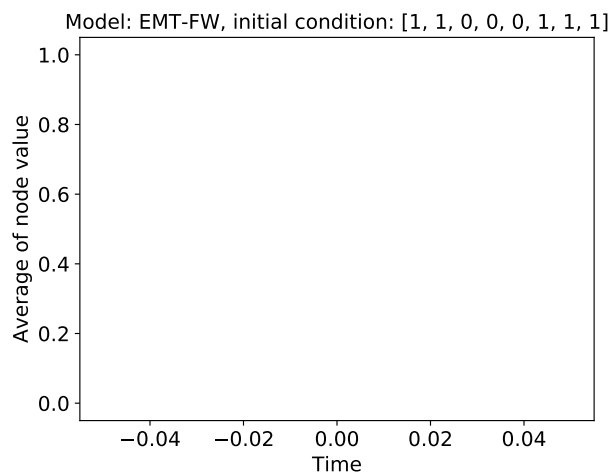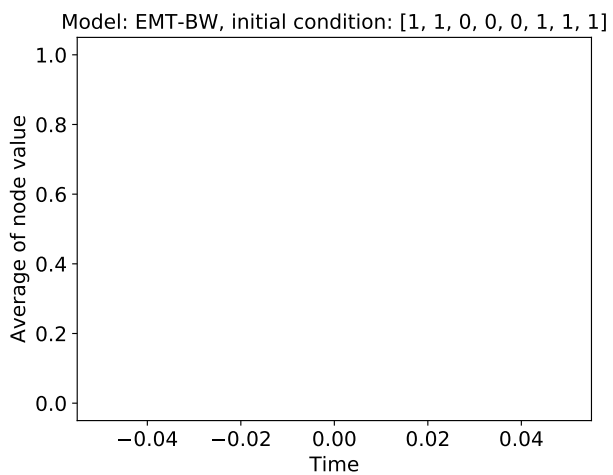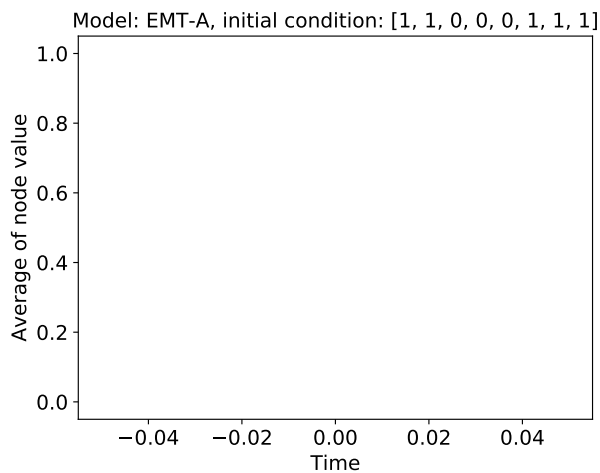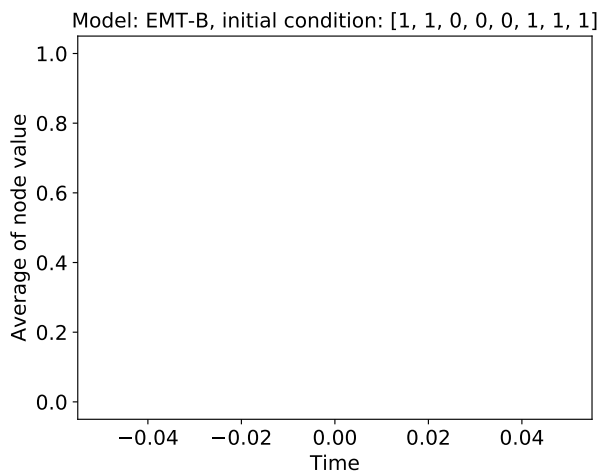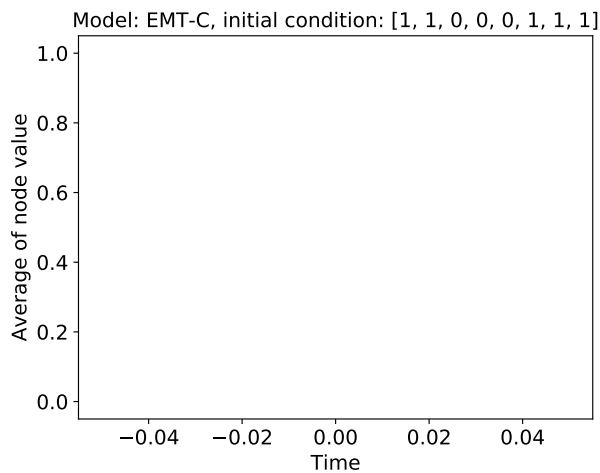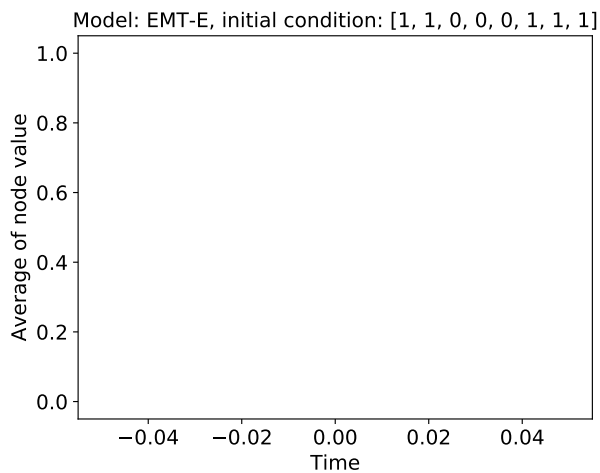

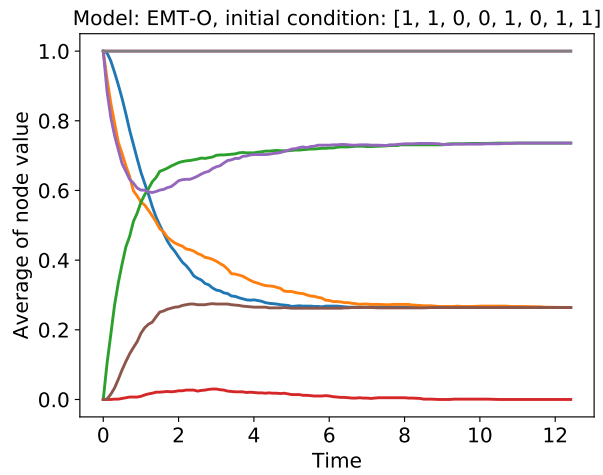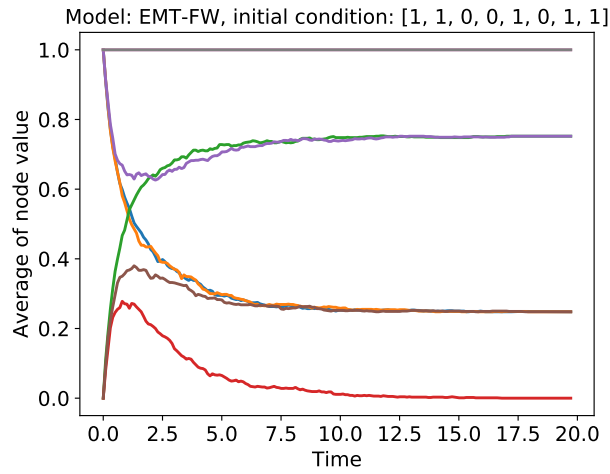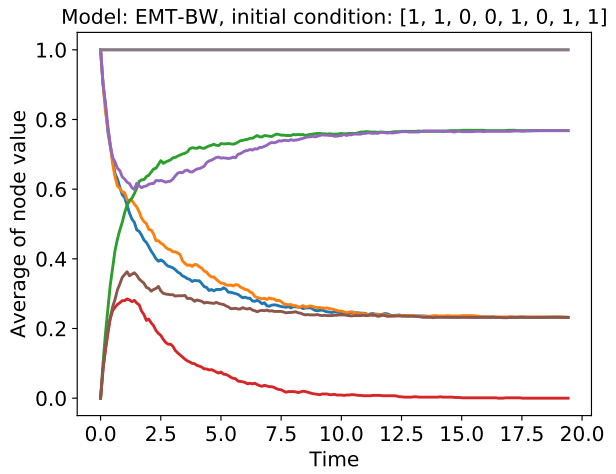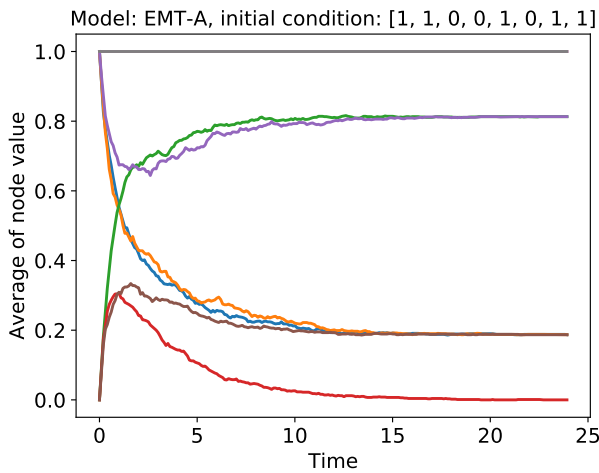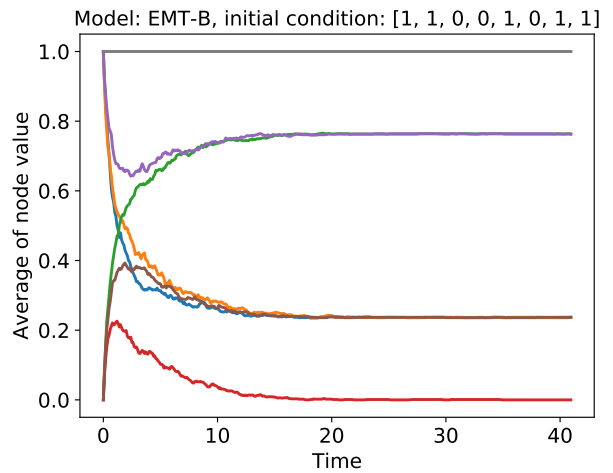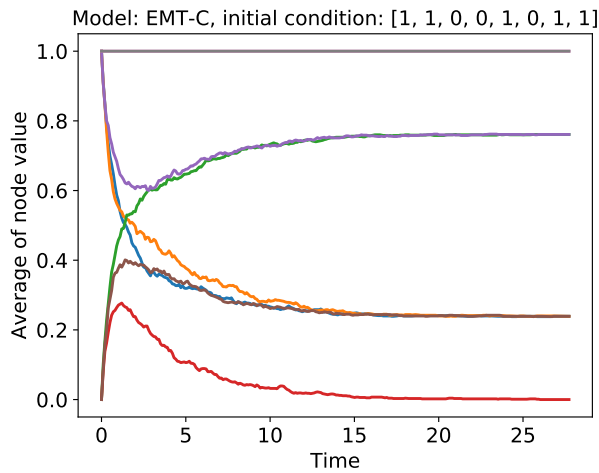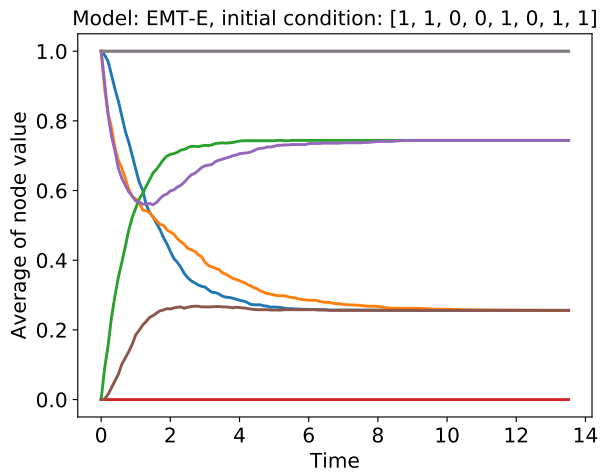

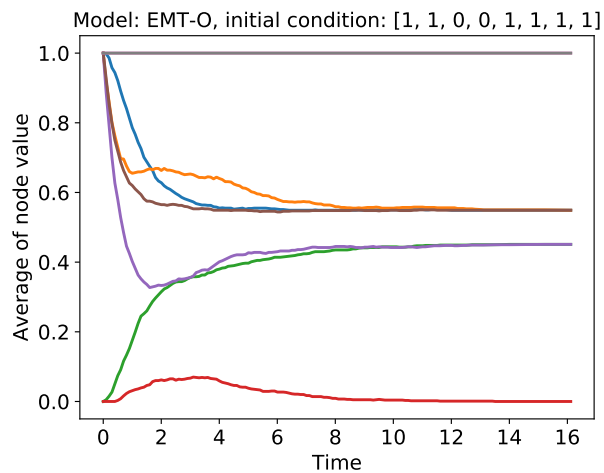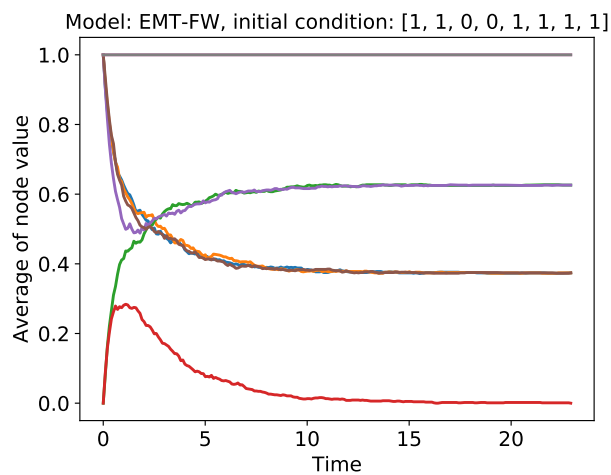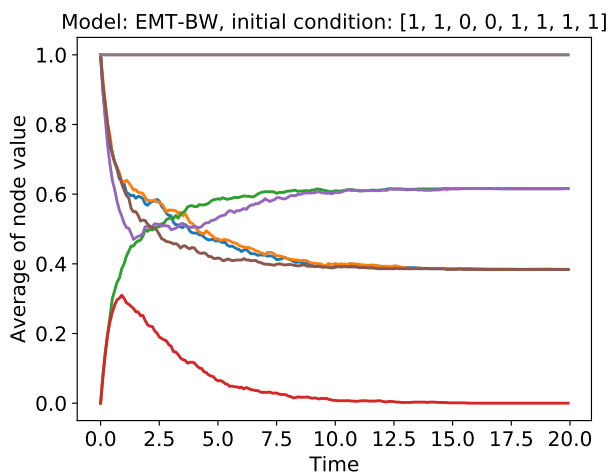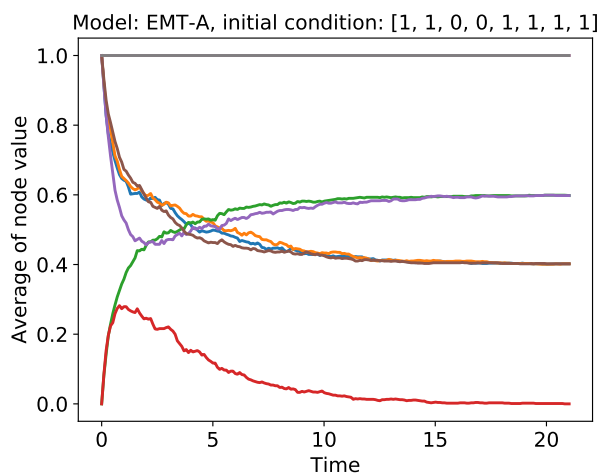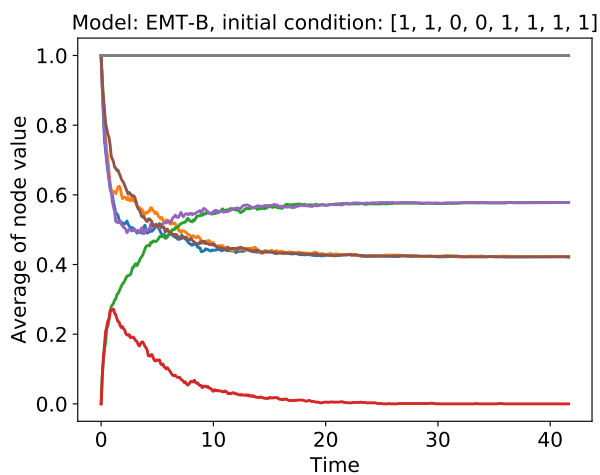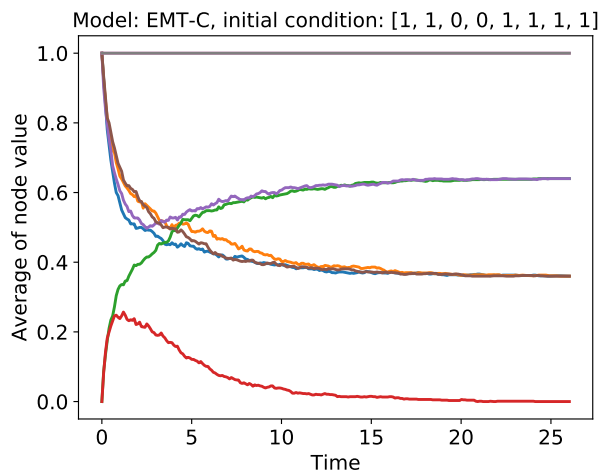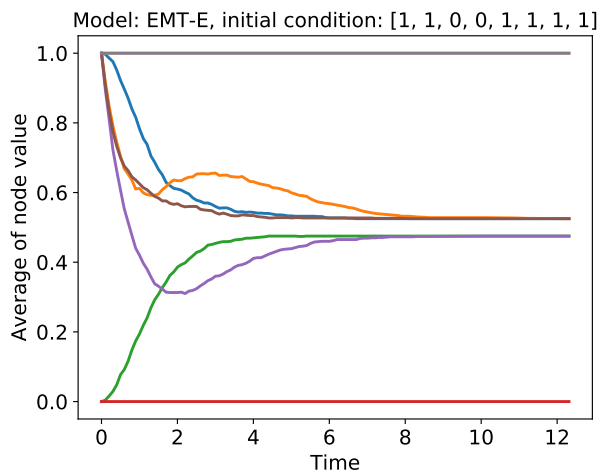

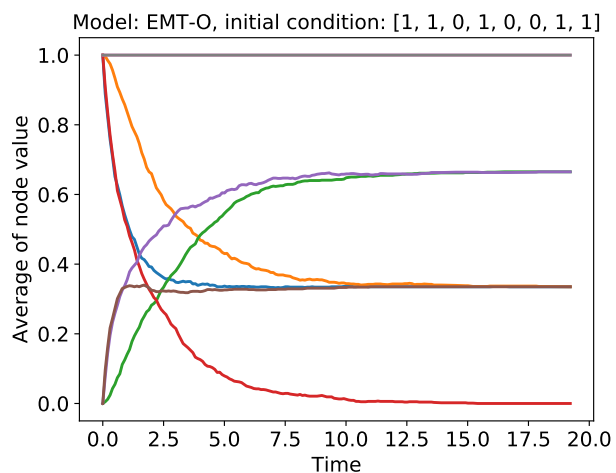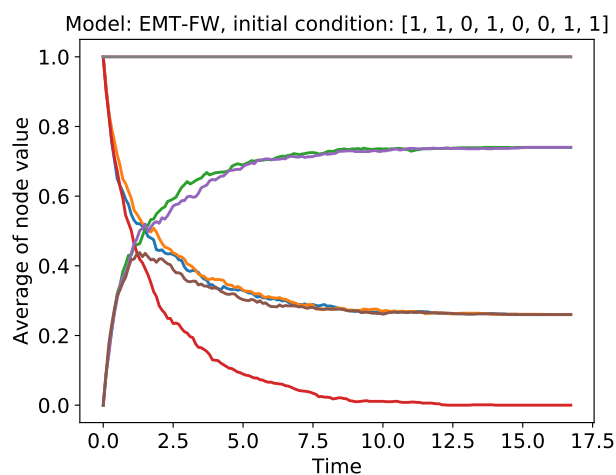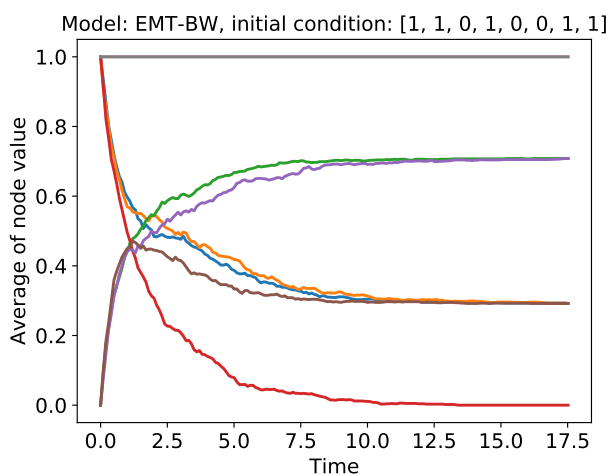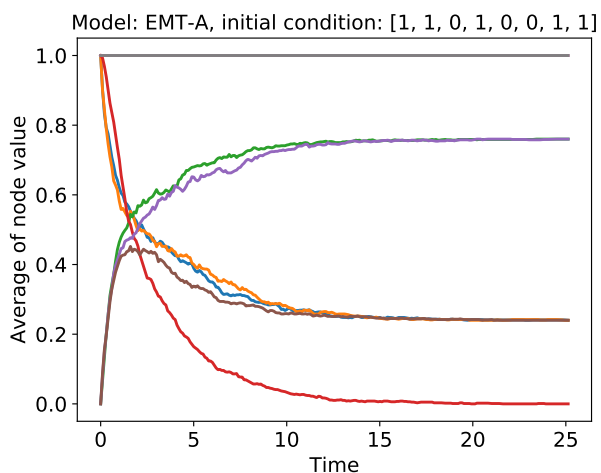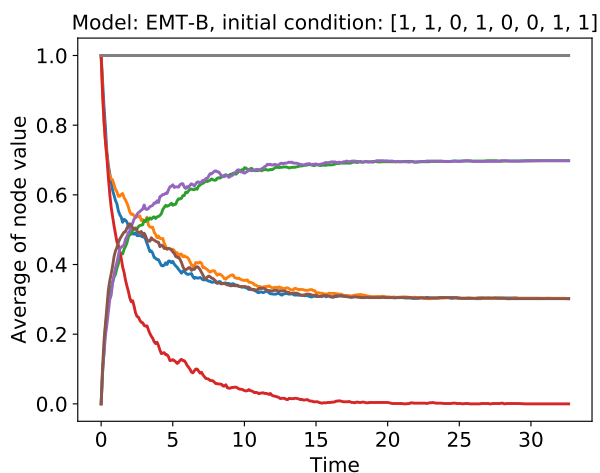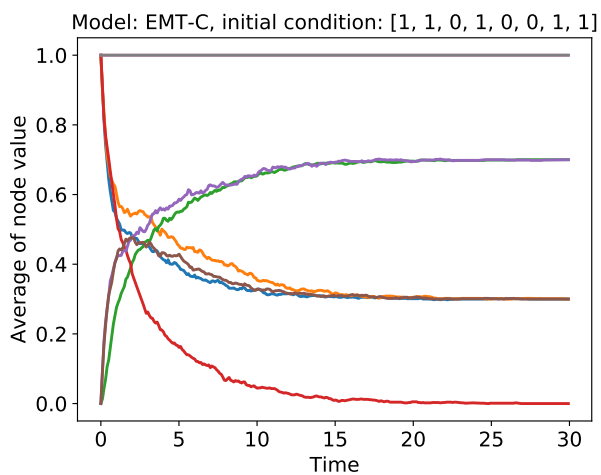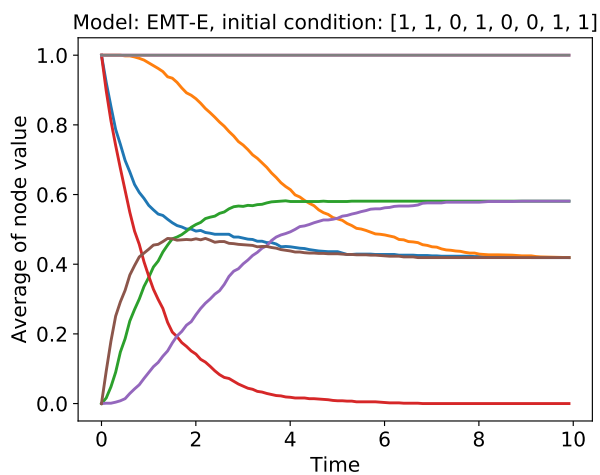

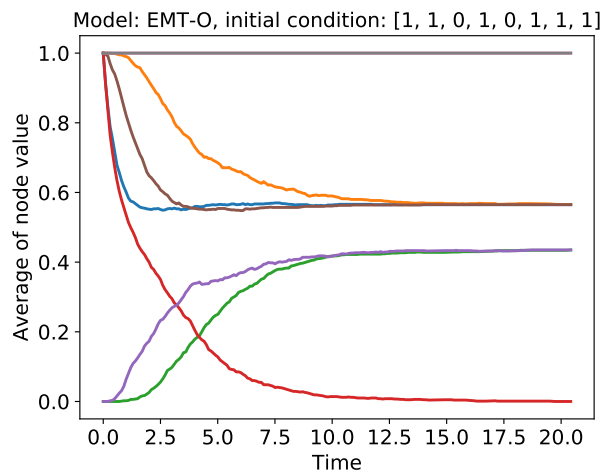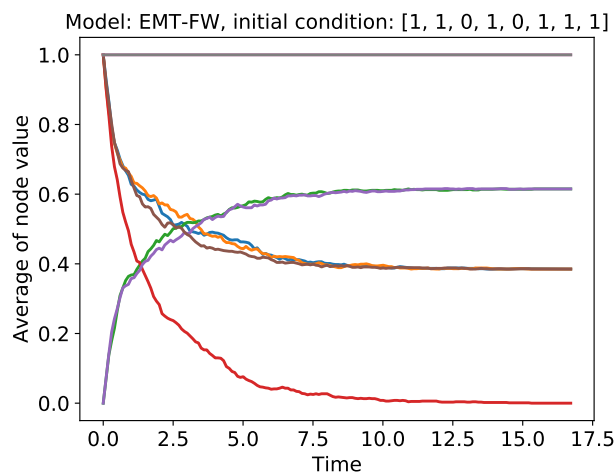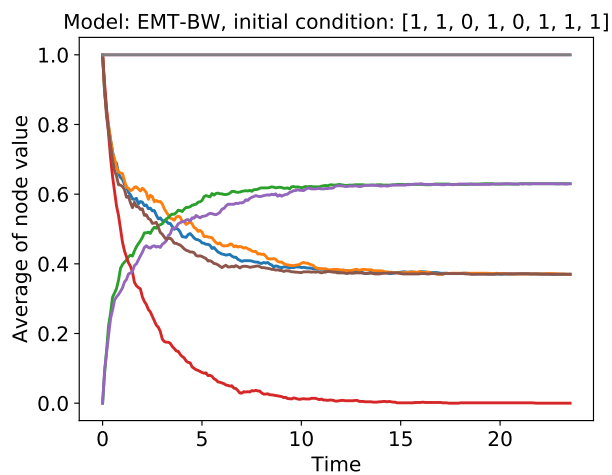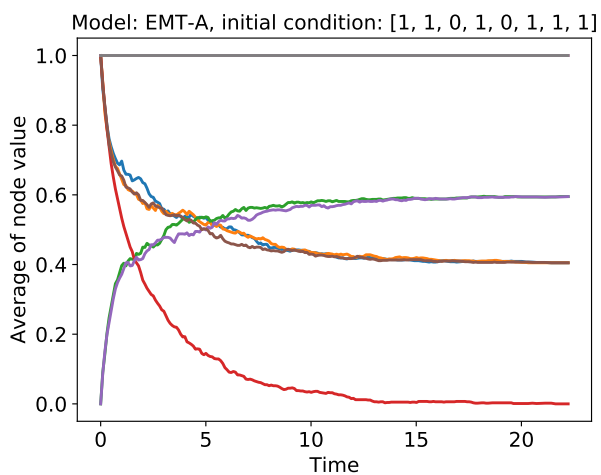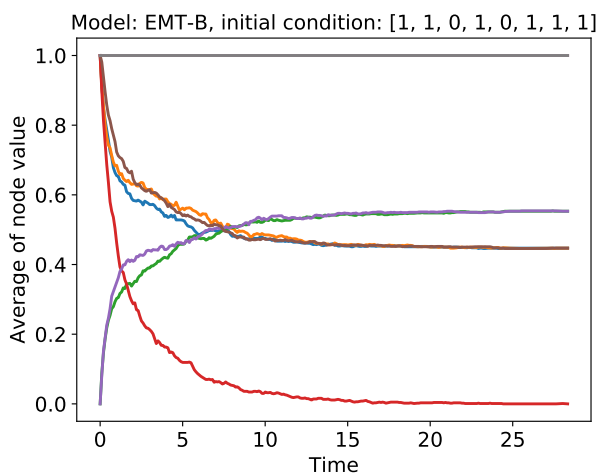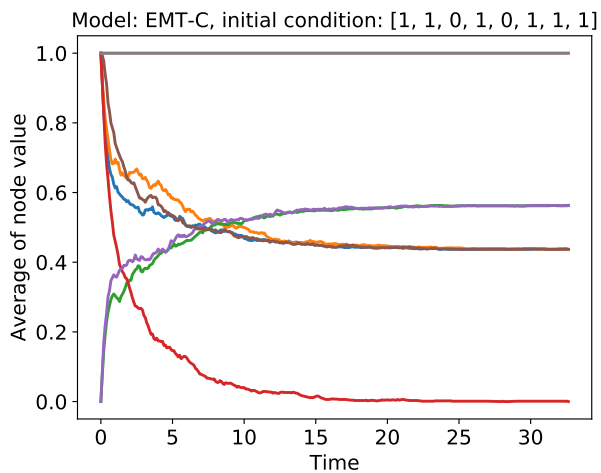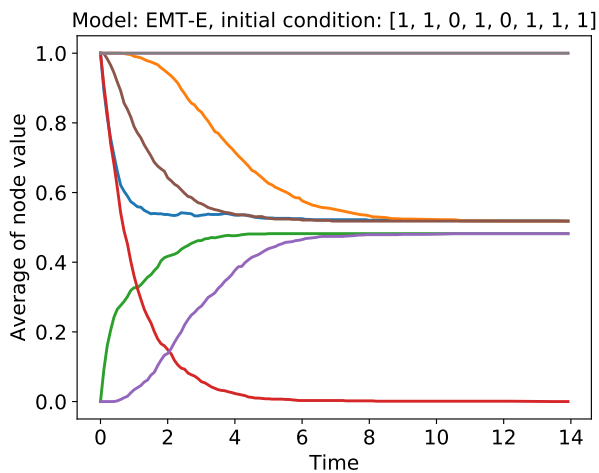

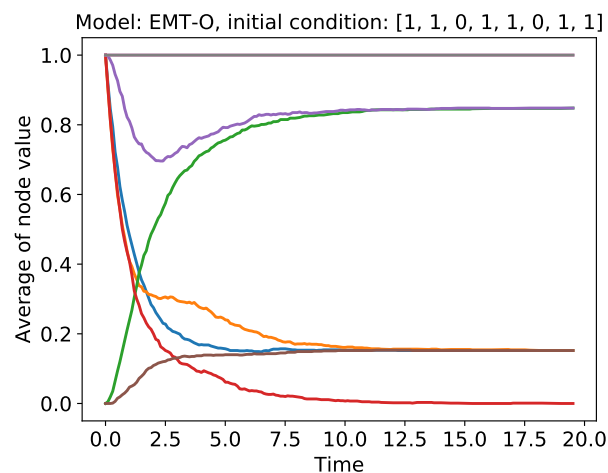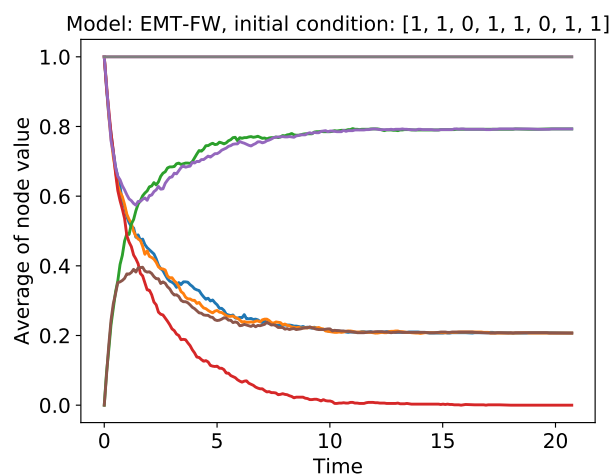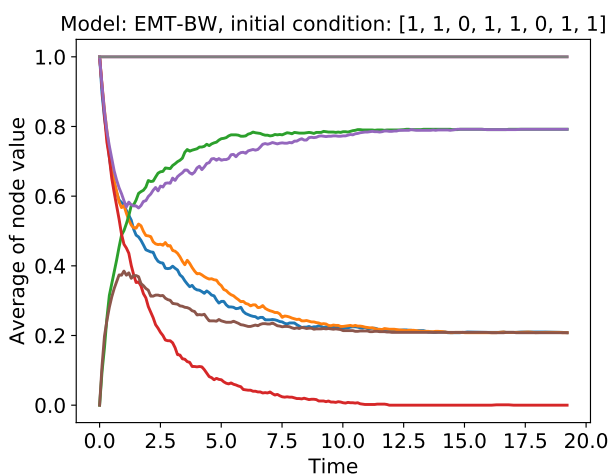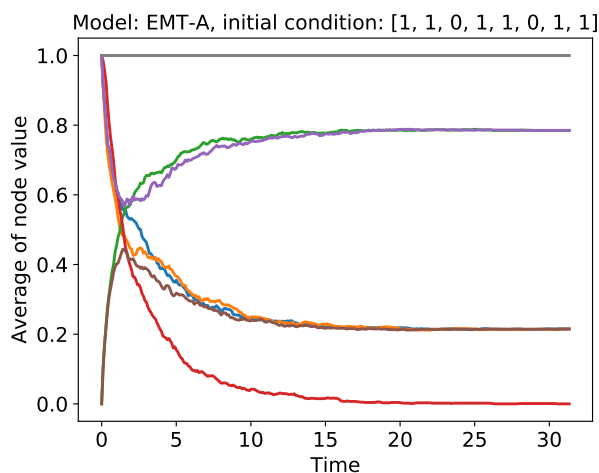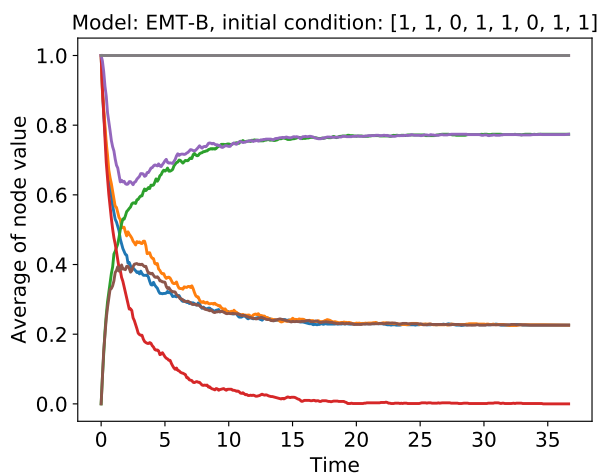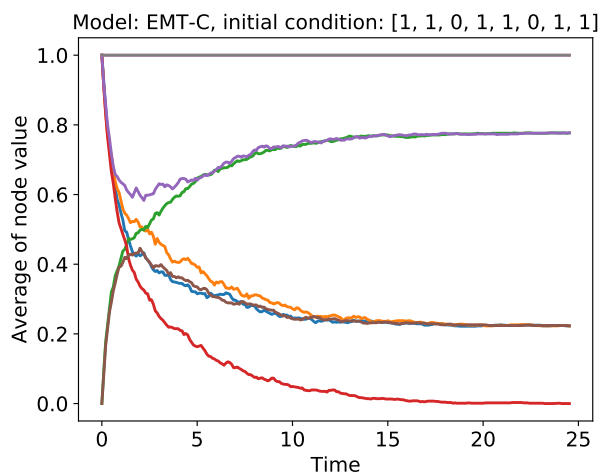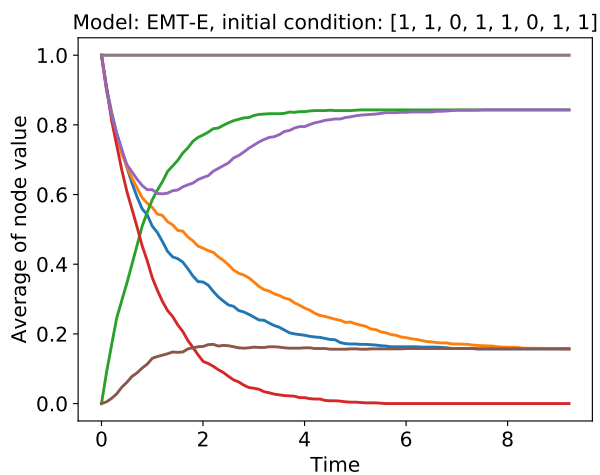

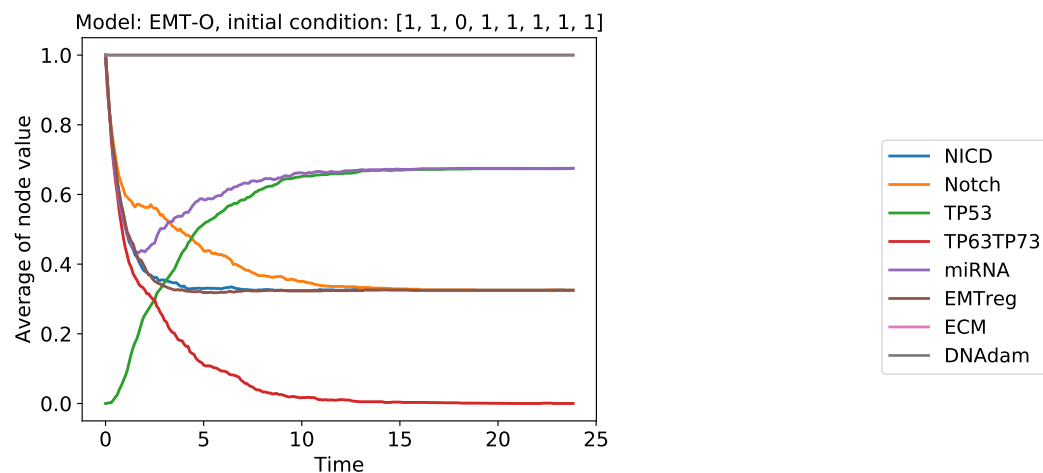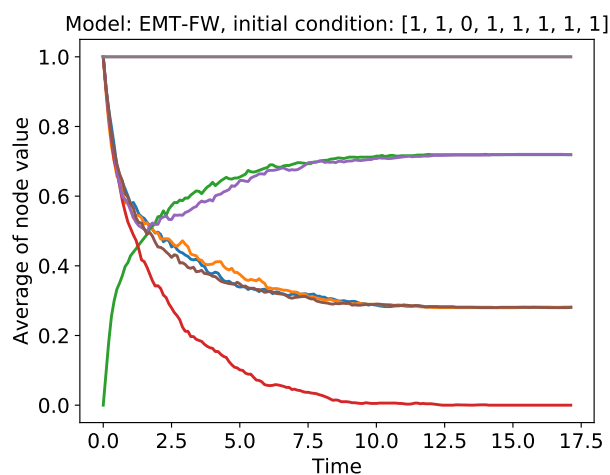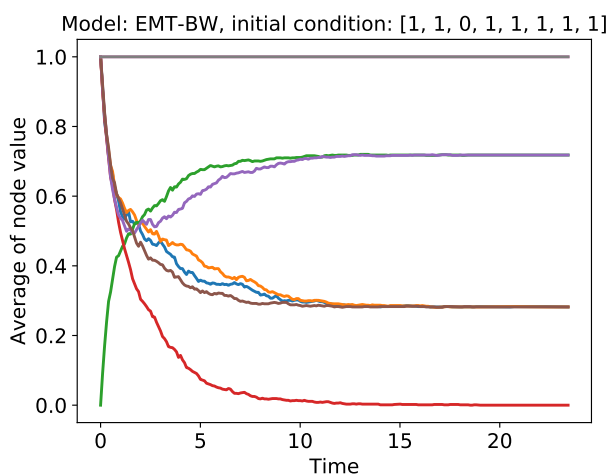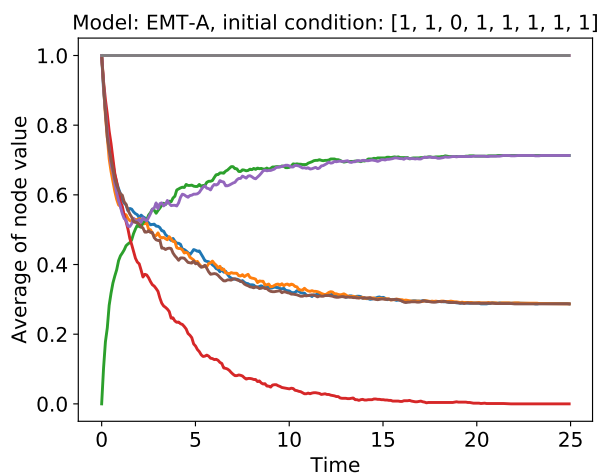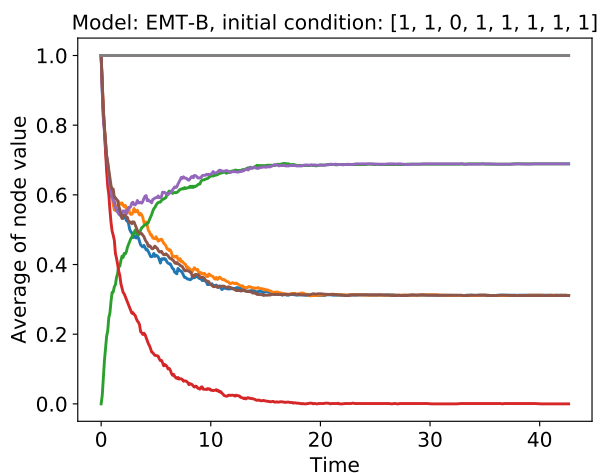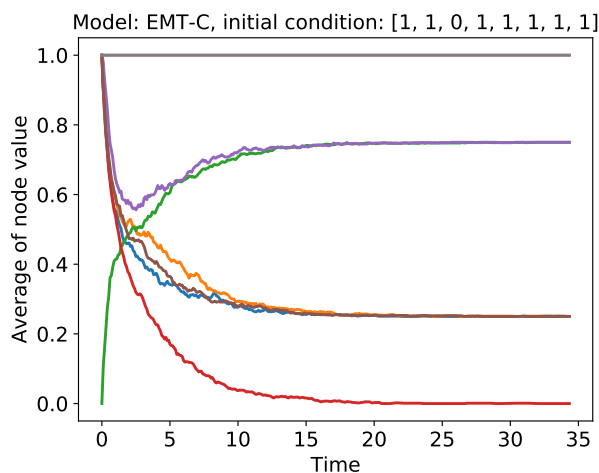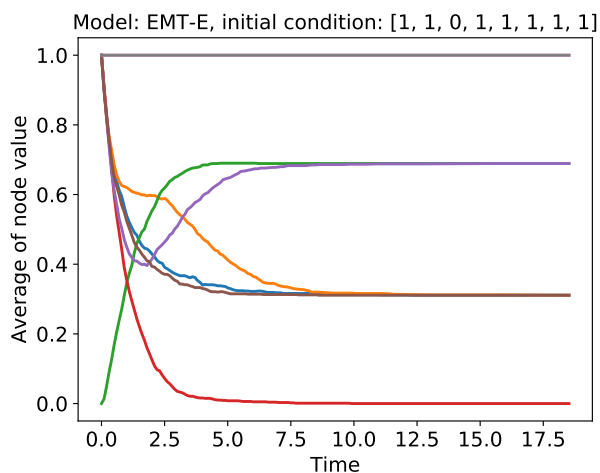

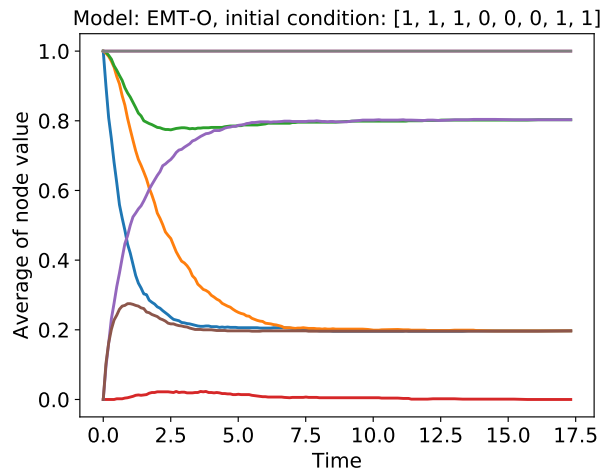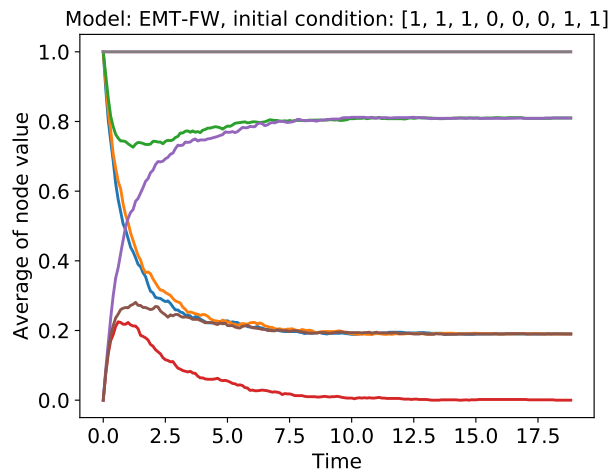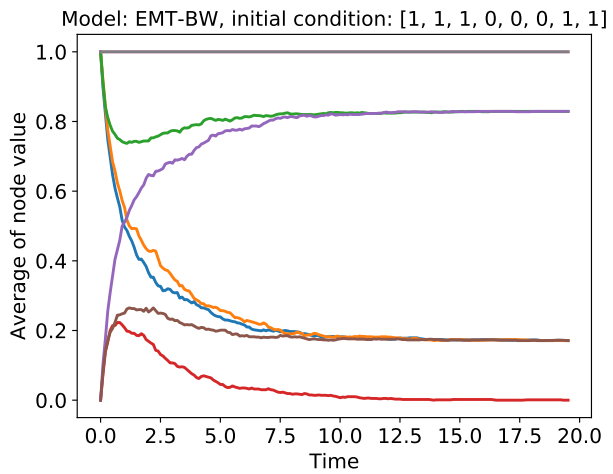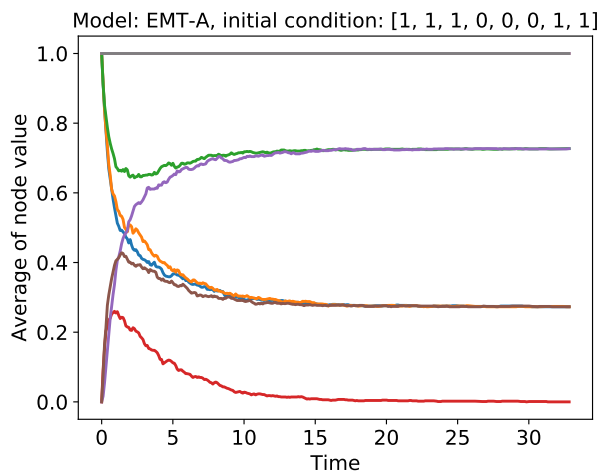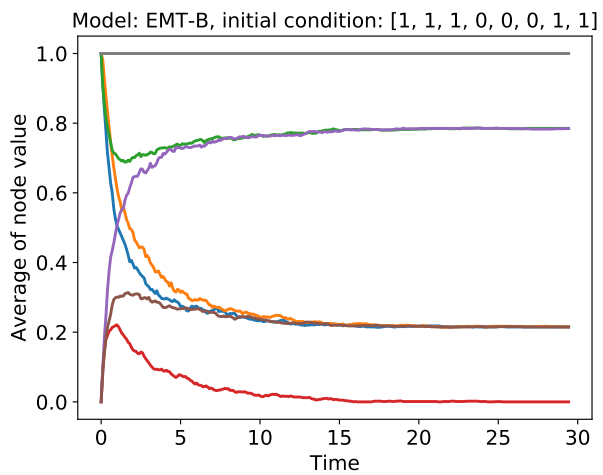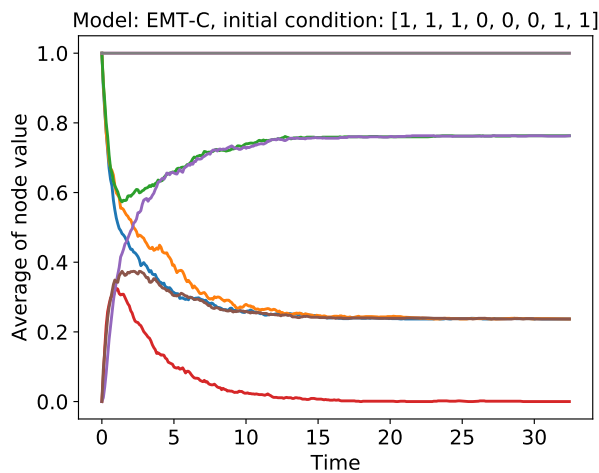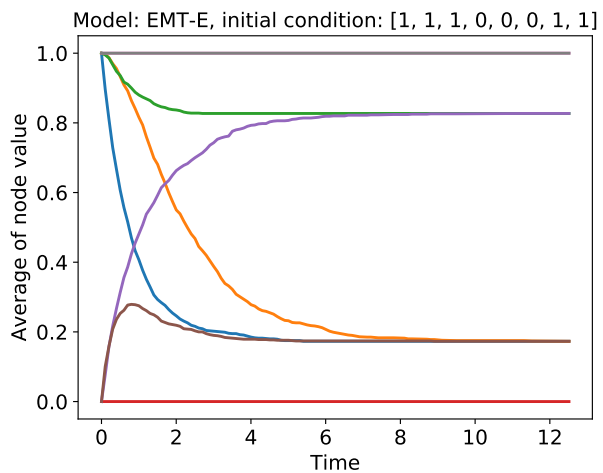

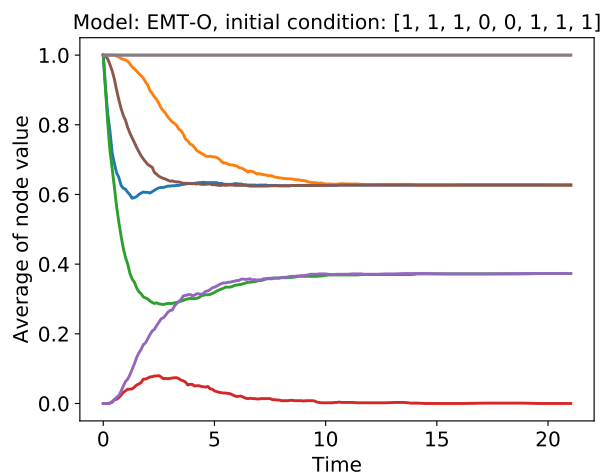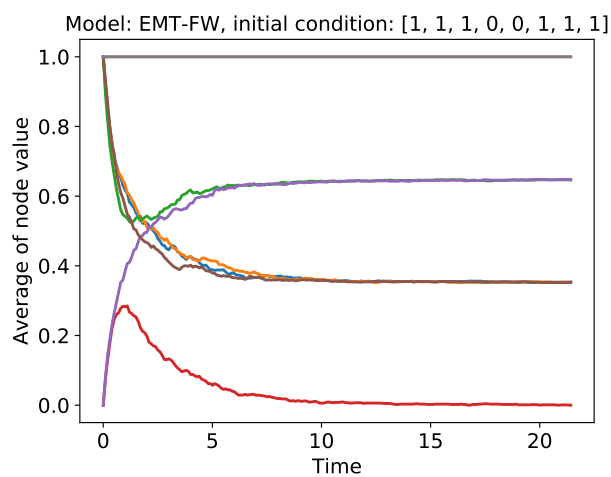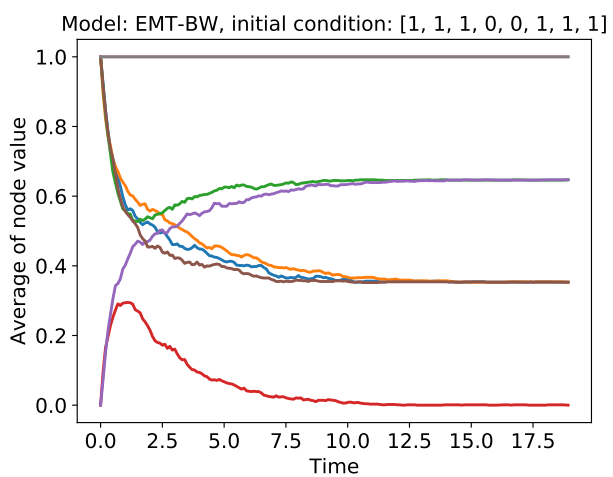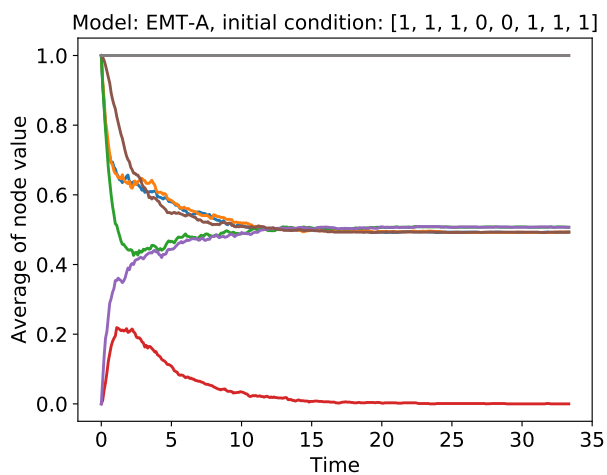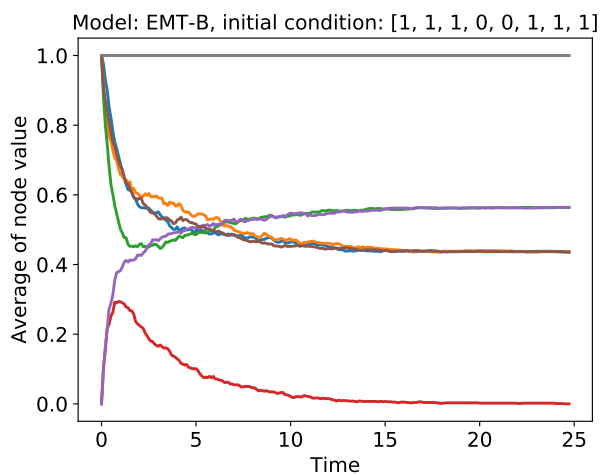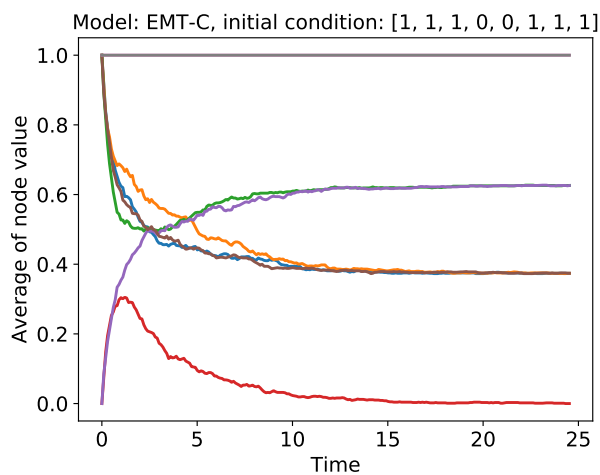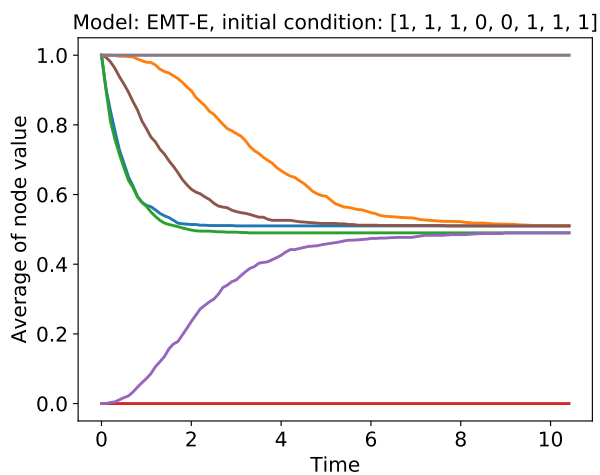

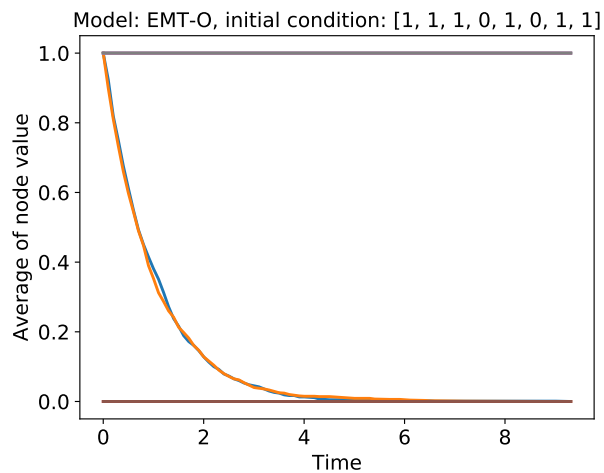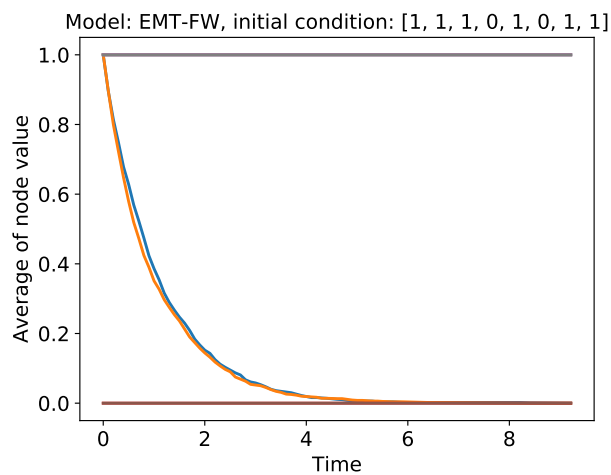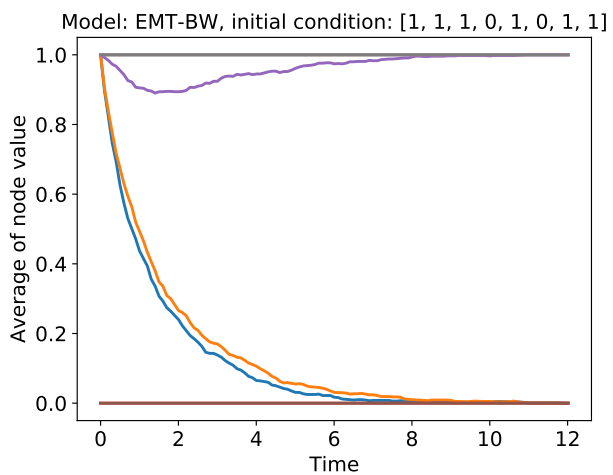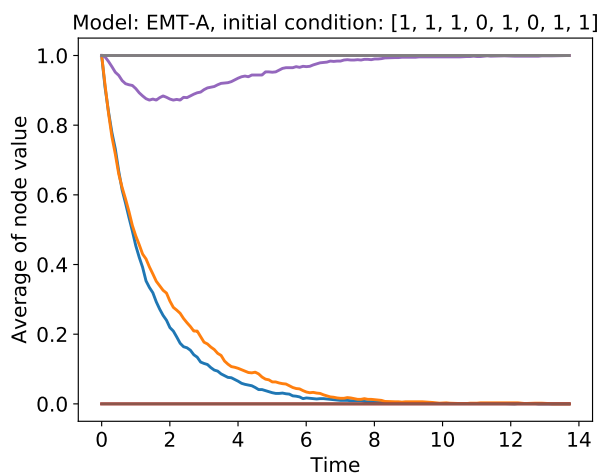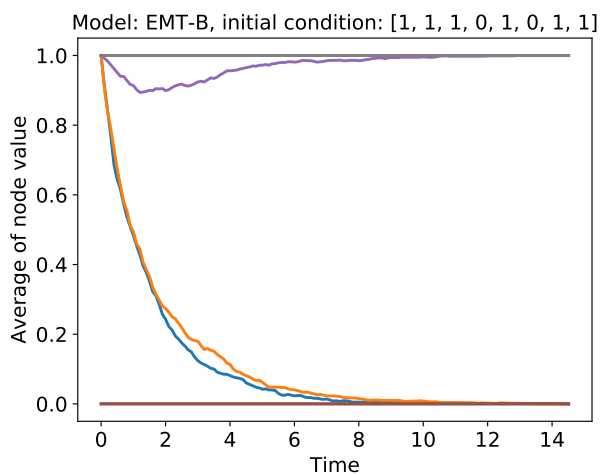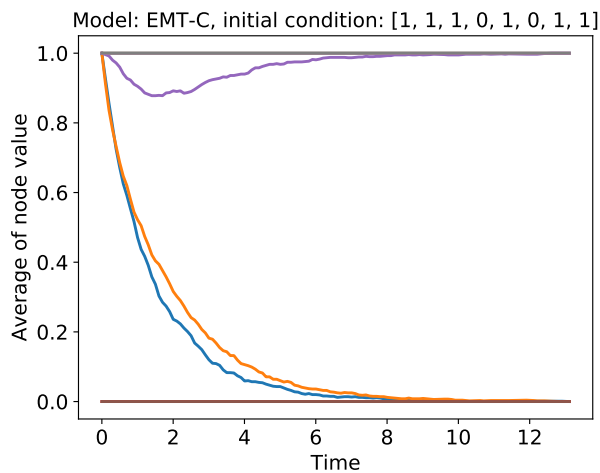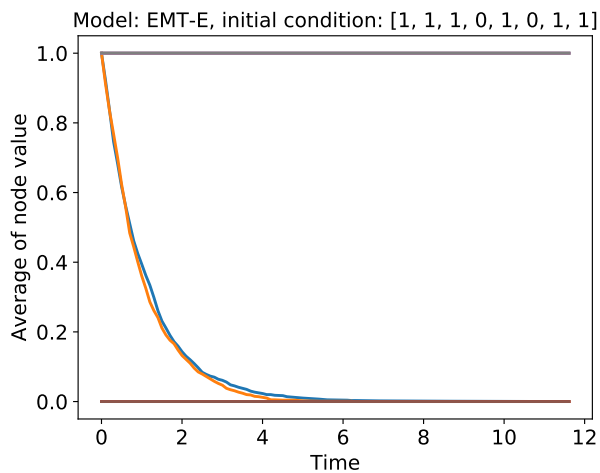

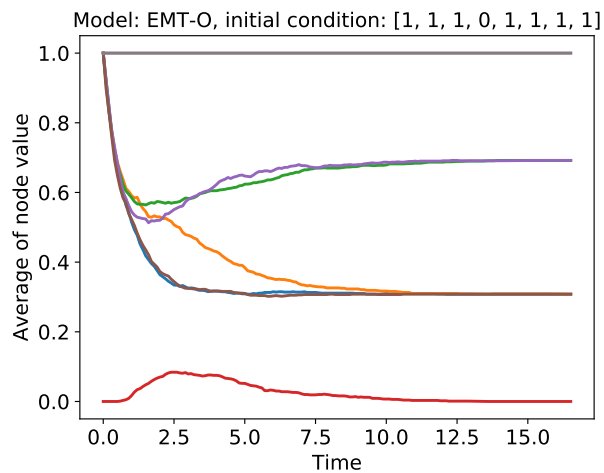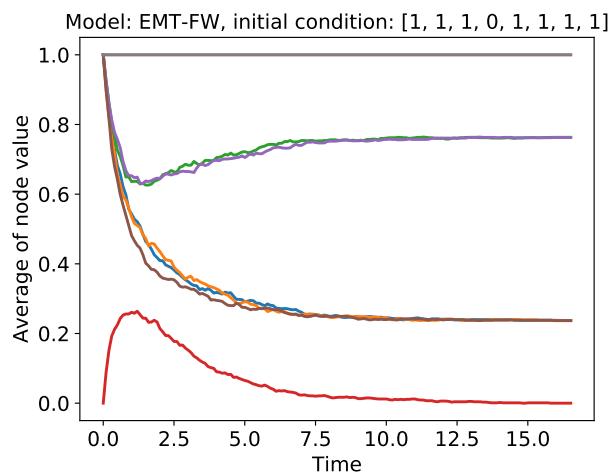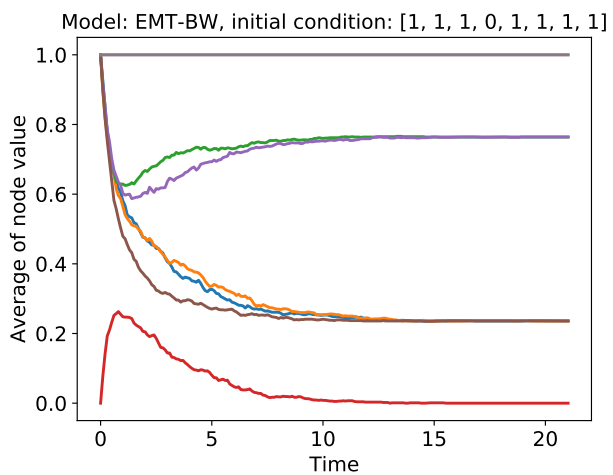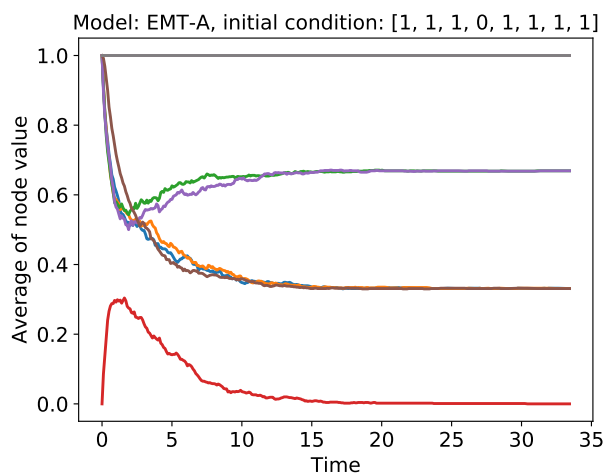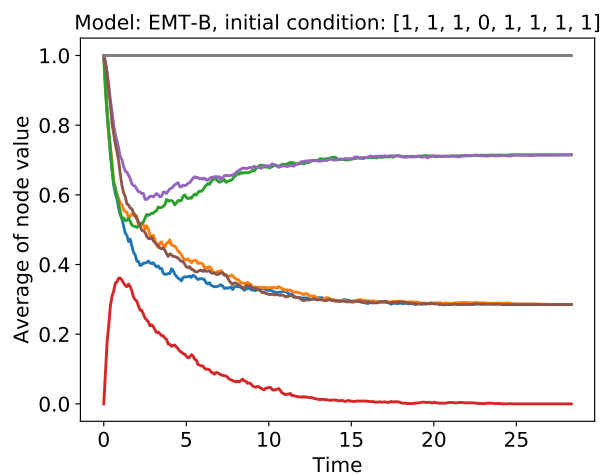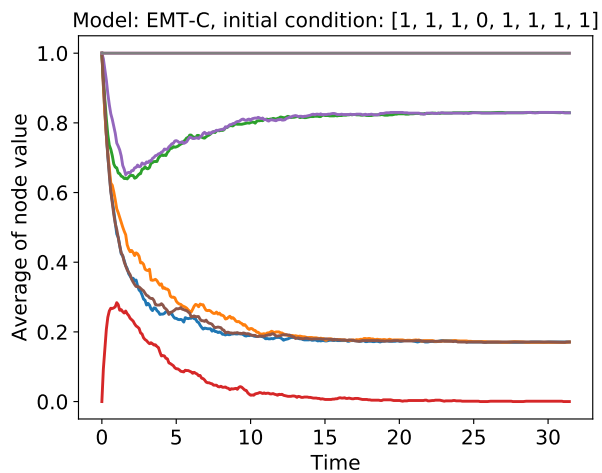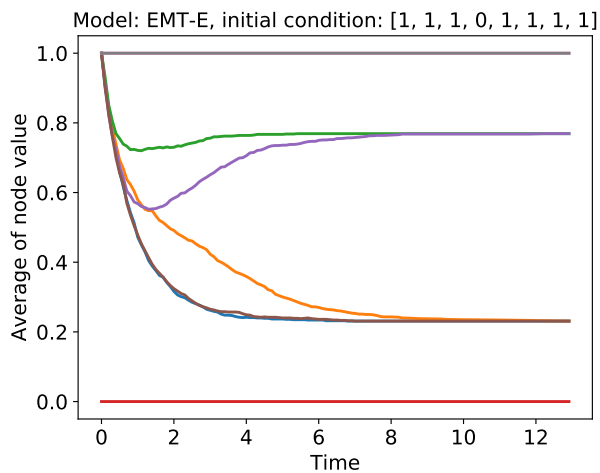

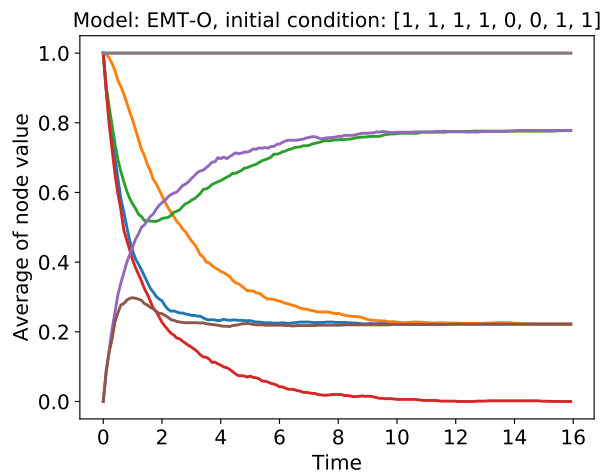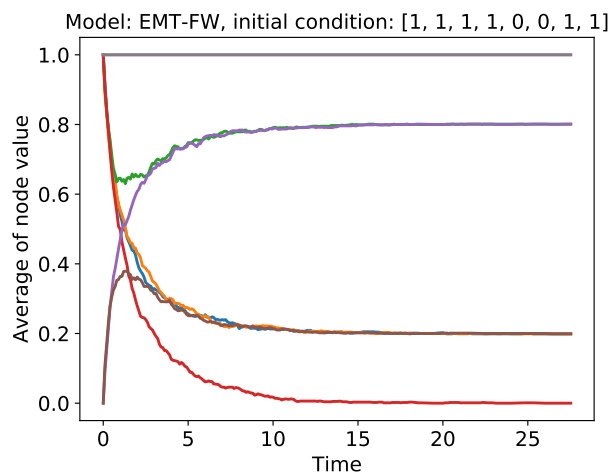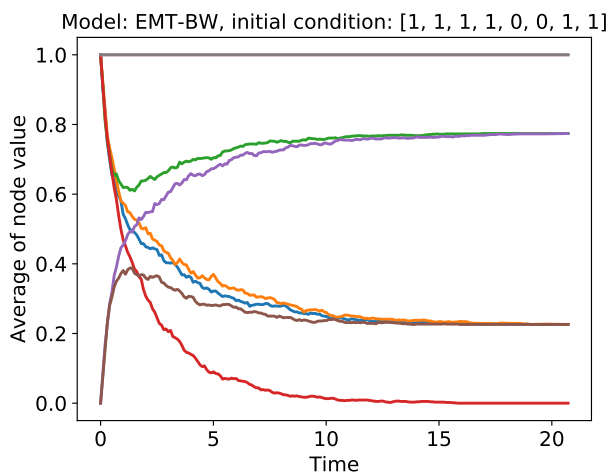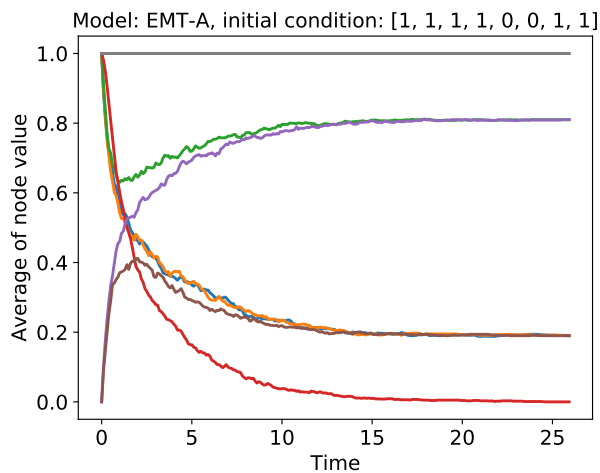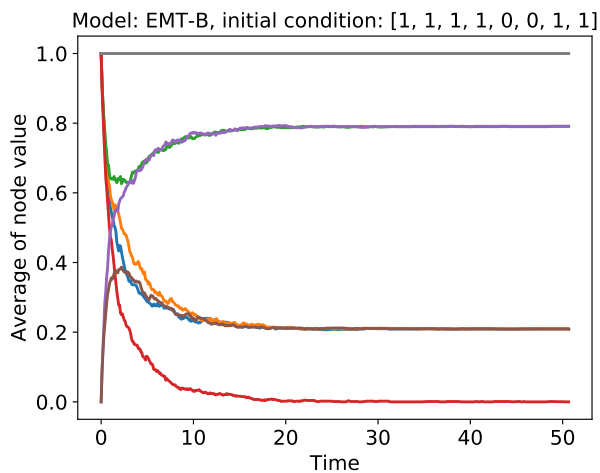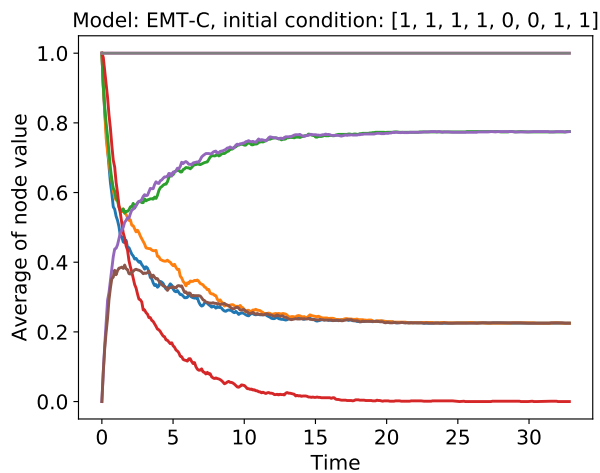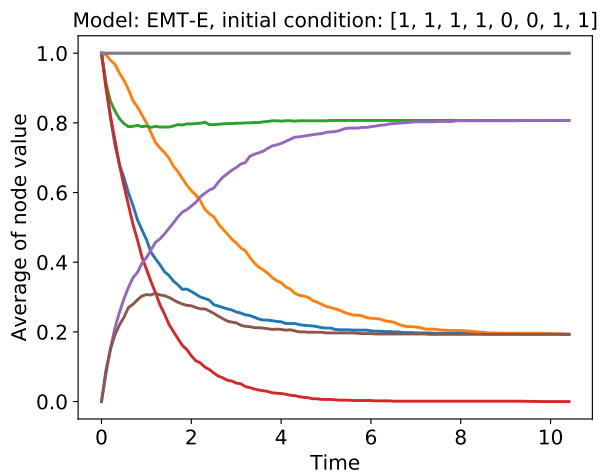

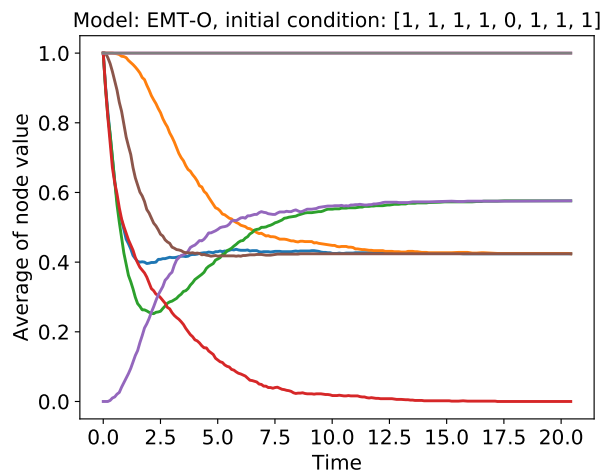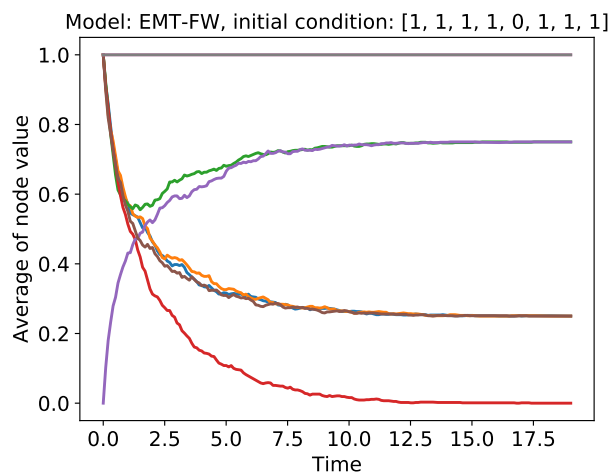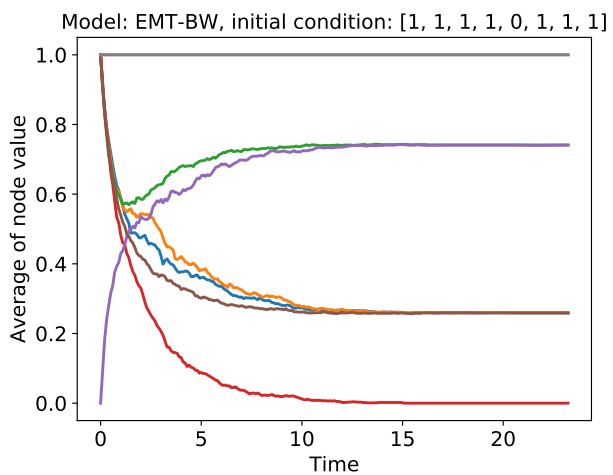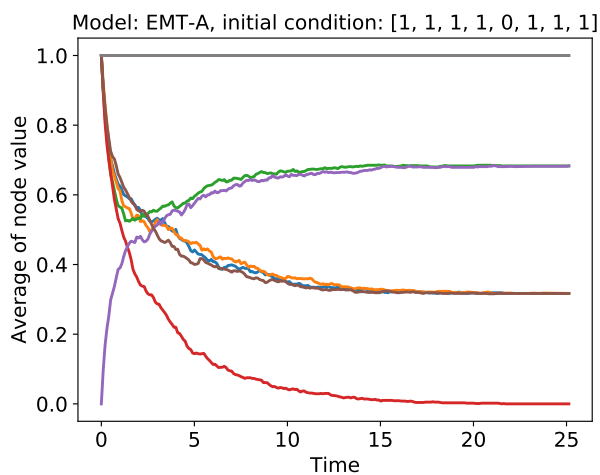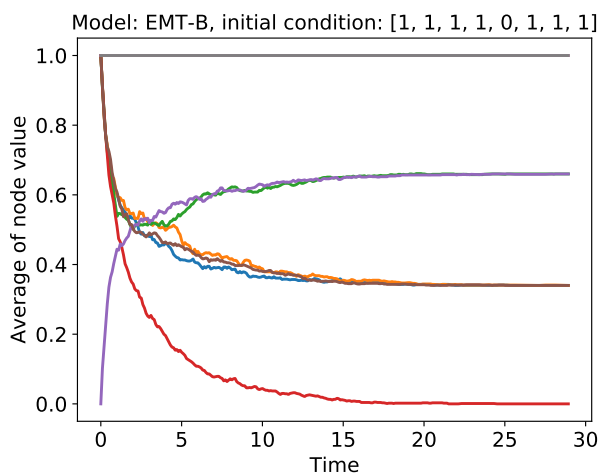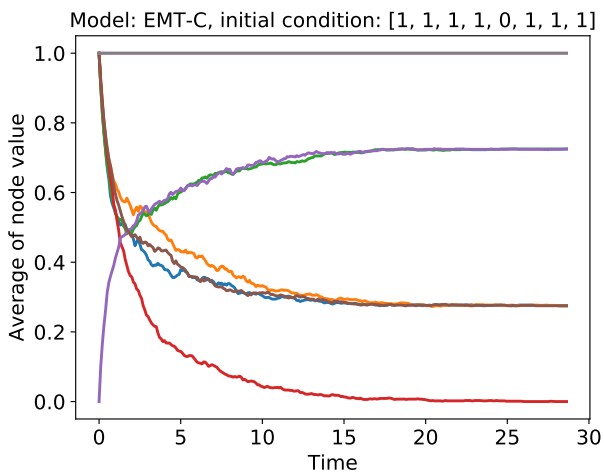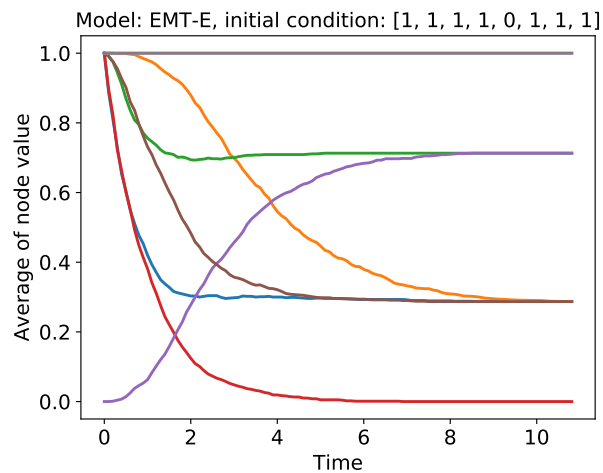

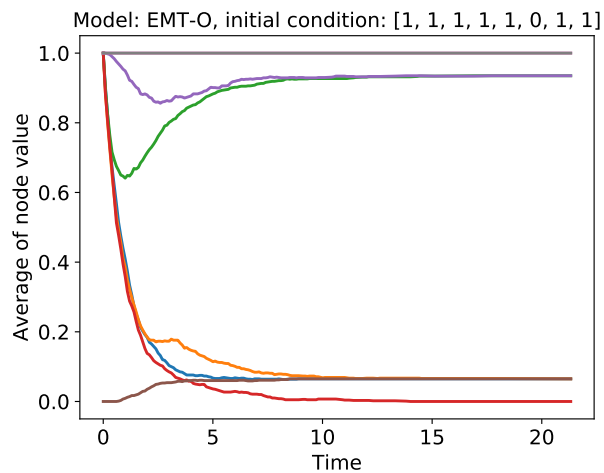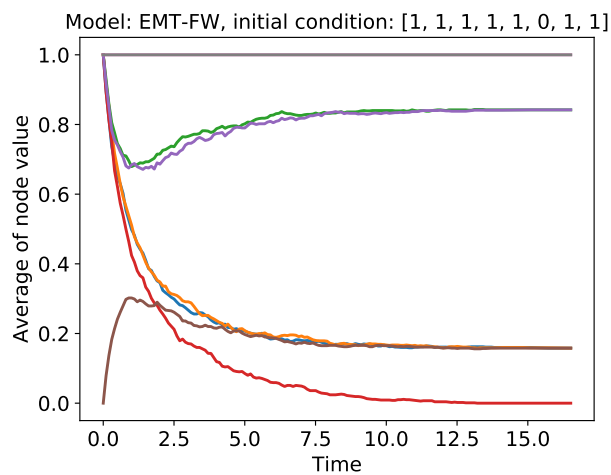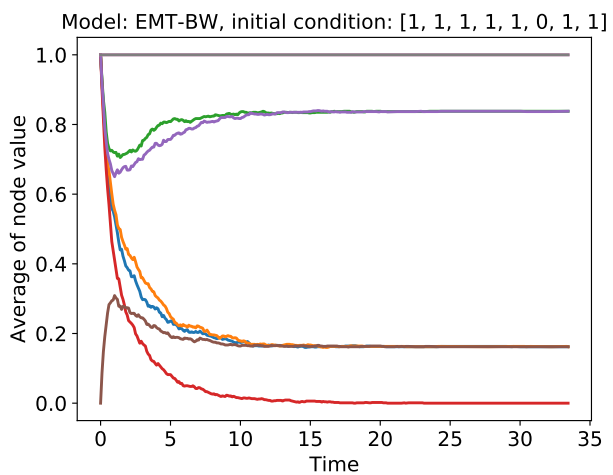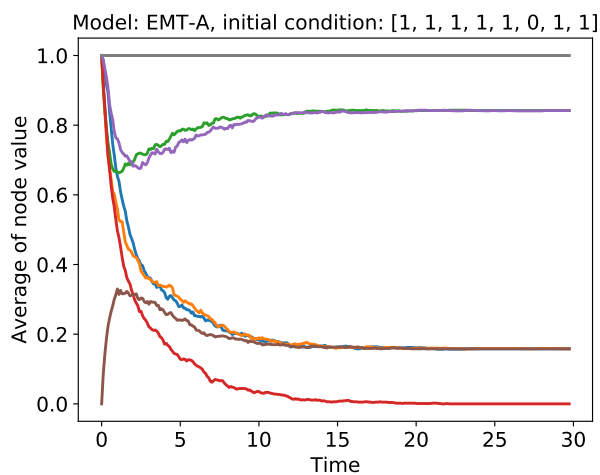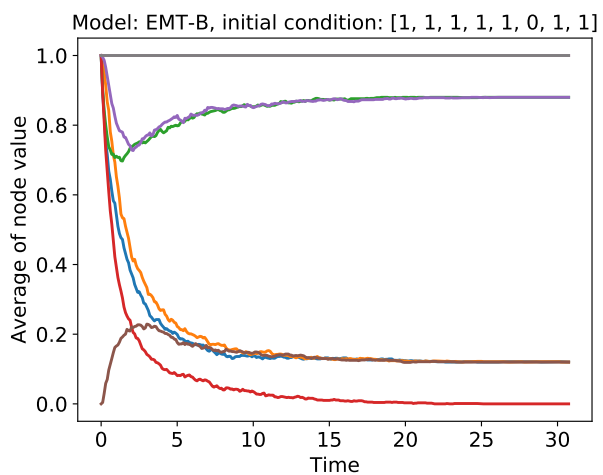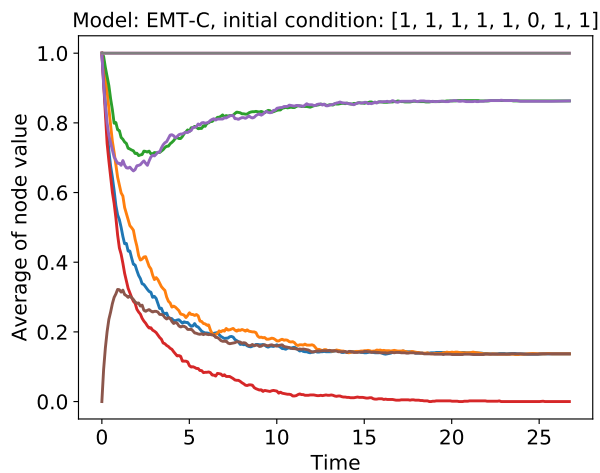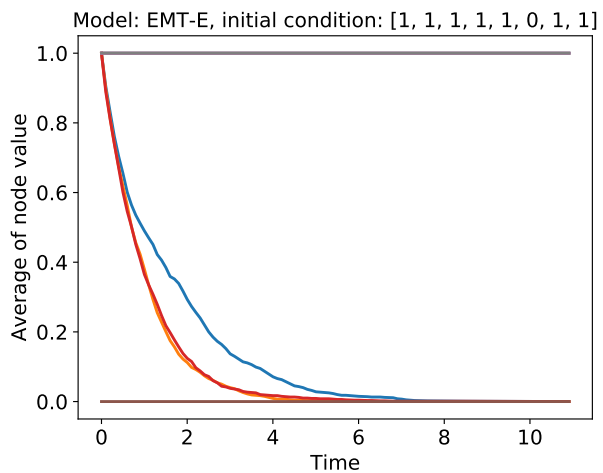

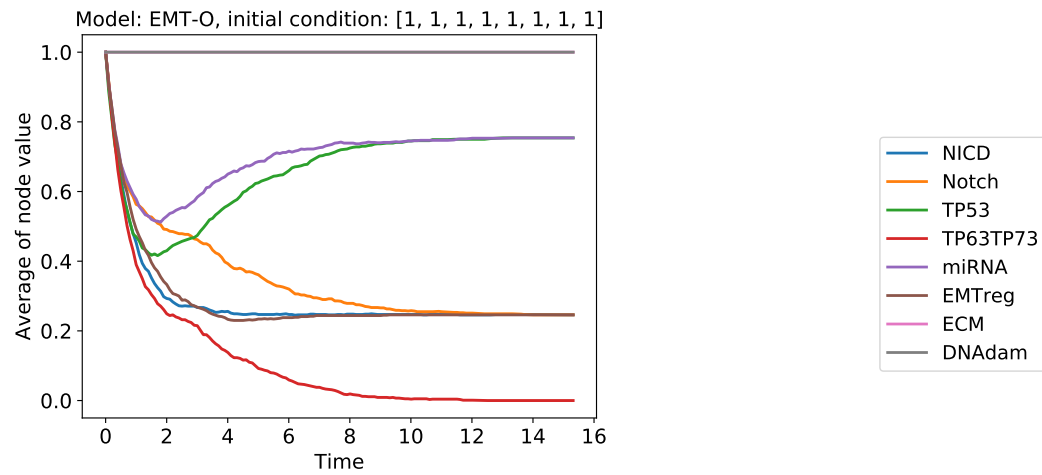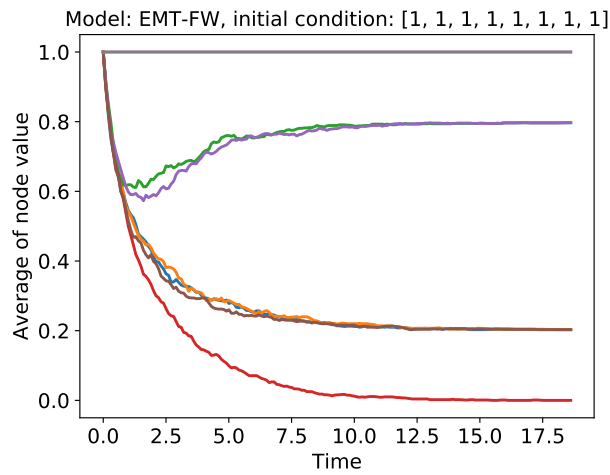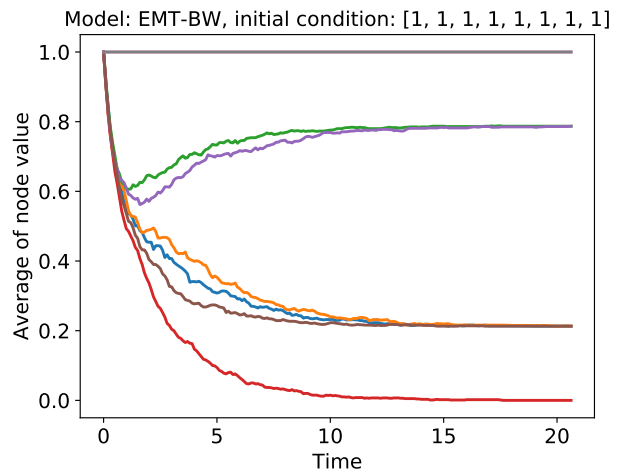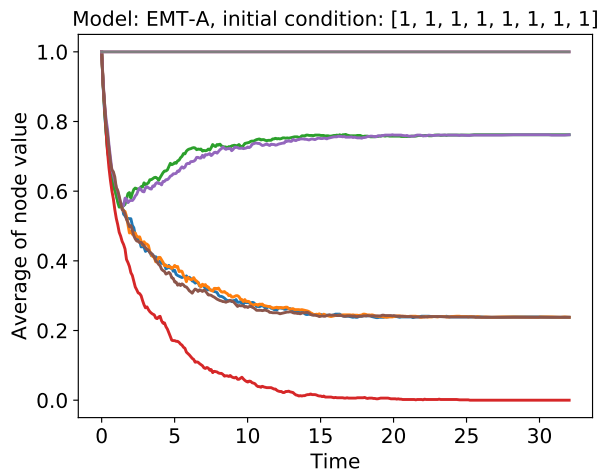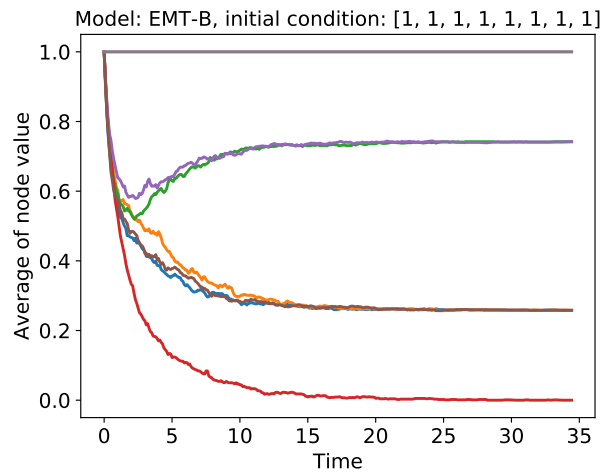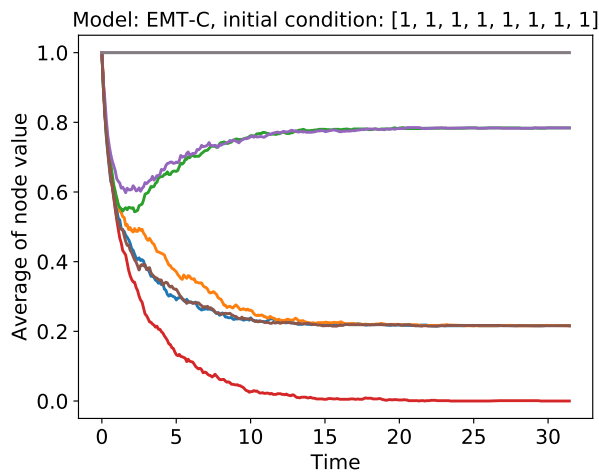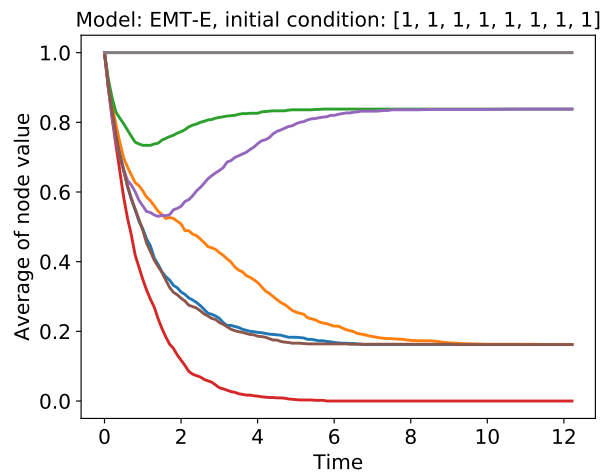

Supplement: S6 File — (PDF) [file pcbi.1009035.s006.pdf]

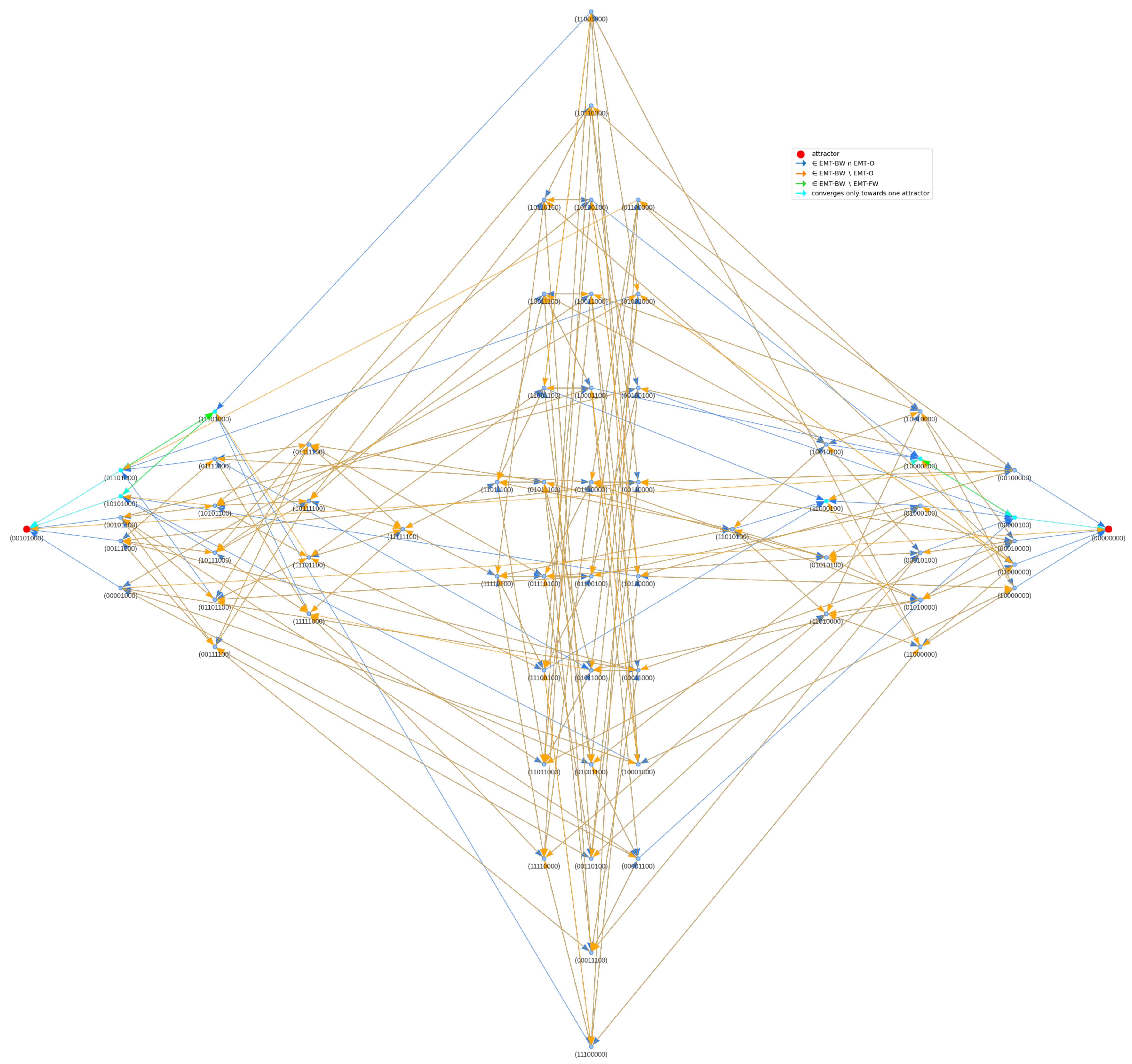

Supplement: S3 Fig — The bright red circles are the target attractors of the system. The blue lines are the lines that occur in EMT-O, as well as in EMT-BW. The orange lines are transitions that are part of EMT-BW but not part of EMT-O. The green lines are transitions that are part of EMT-BW but not EMT-FW. The light blue lines depict states that are part of the network, but only have access to one of the attractors and not both of them. (PDF) [file pcbi.1009035.s009.pdf]

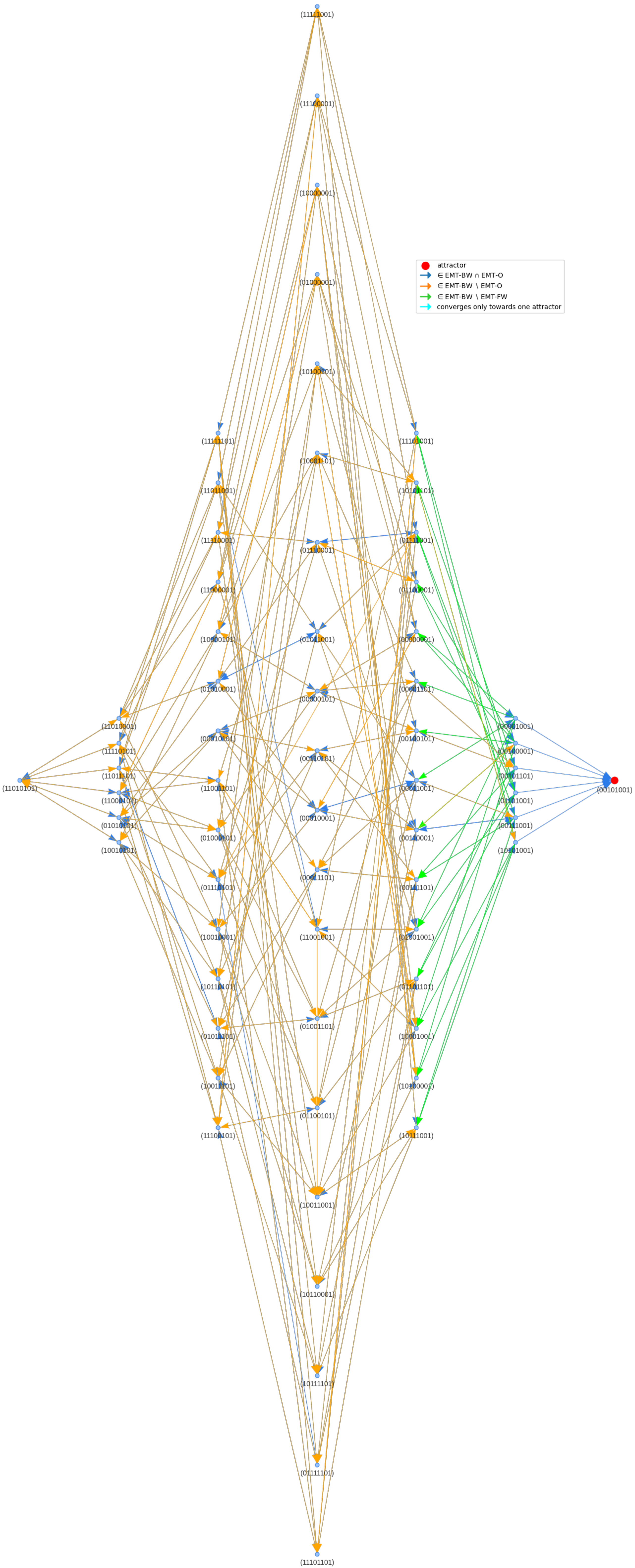

Supplement: S4 Fig — The bright red circle is the target attractors of the system. The blue lines are the lines that occur in EMT-O, as well as in EMT-BW. The orange lines are transitions that are part of EMT-BW but not part of EMT-O. The green lines are transitions that are part of EMT-BW but not EMT-FW. (PDF) [file pcbi.1009035.s010.pdf]

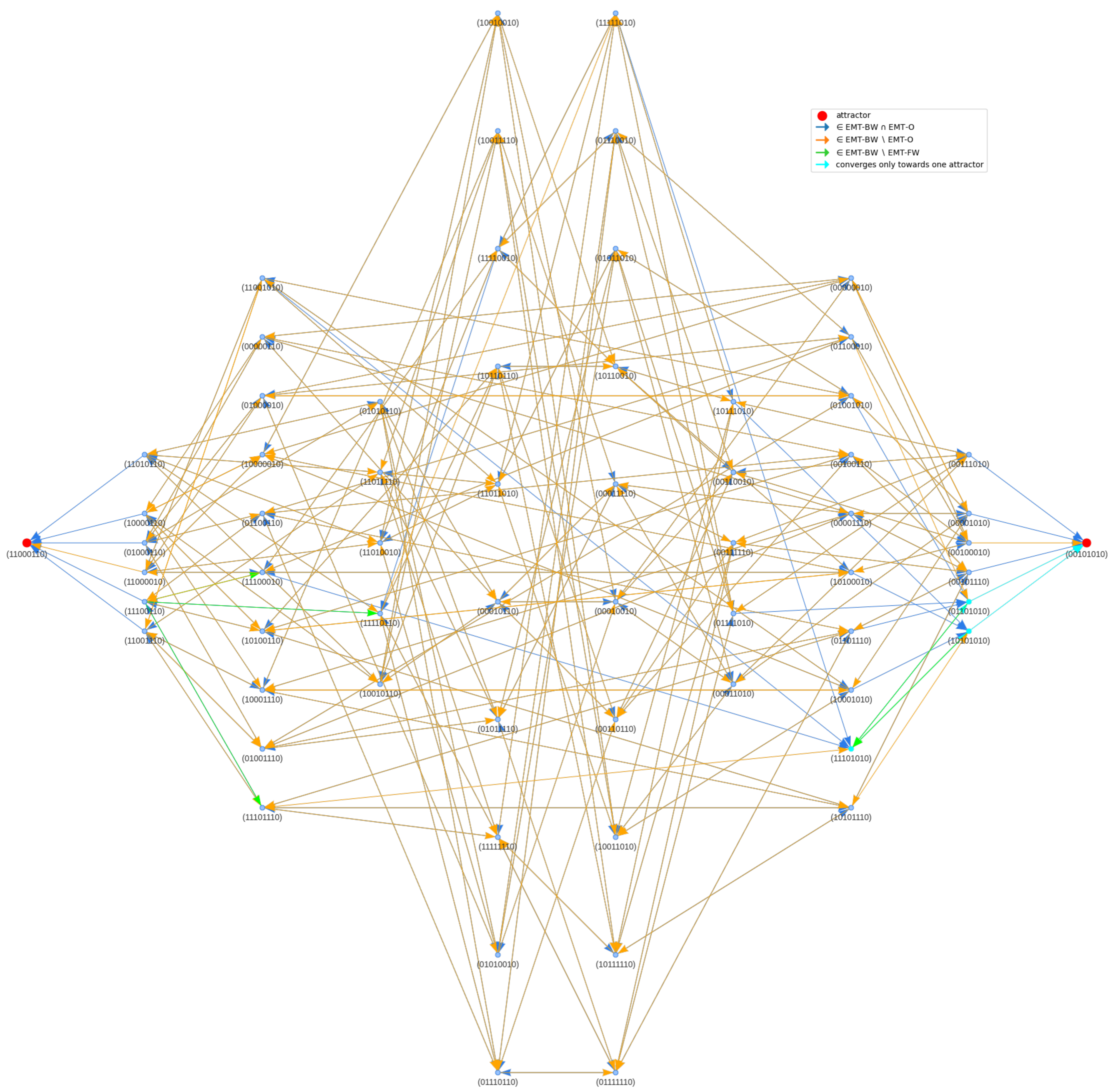

Supplement: S5 Fig — The bright red circles are the target attractors of the system. The blue lines are the lines that occur in EMT-O, as well as in EMT-BW. The orange lines are transitions that are part of EMT-BW but not part of EMT-O. The green lines are transitions that are part of EMT-BW but not EMT-FW. The light blue lines depict states that are part of the network, but only have access to one of the attractors and not both of them. (PDF) [file pcbi.1009035.s011.pdf]

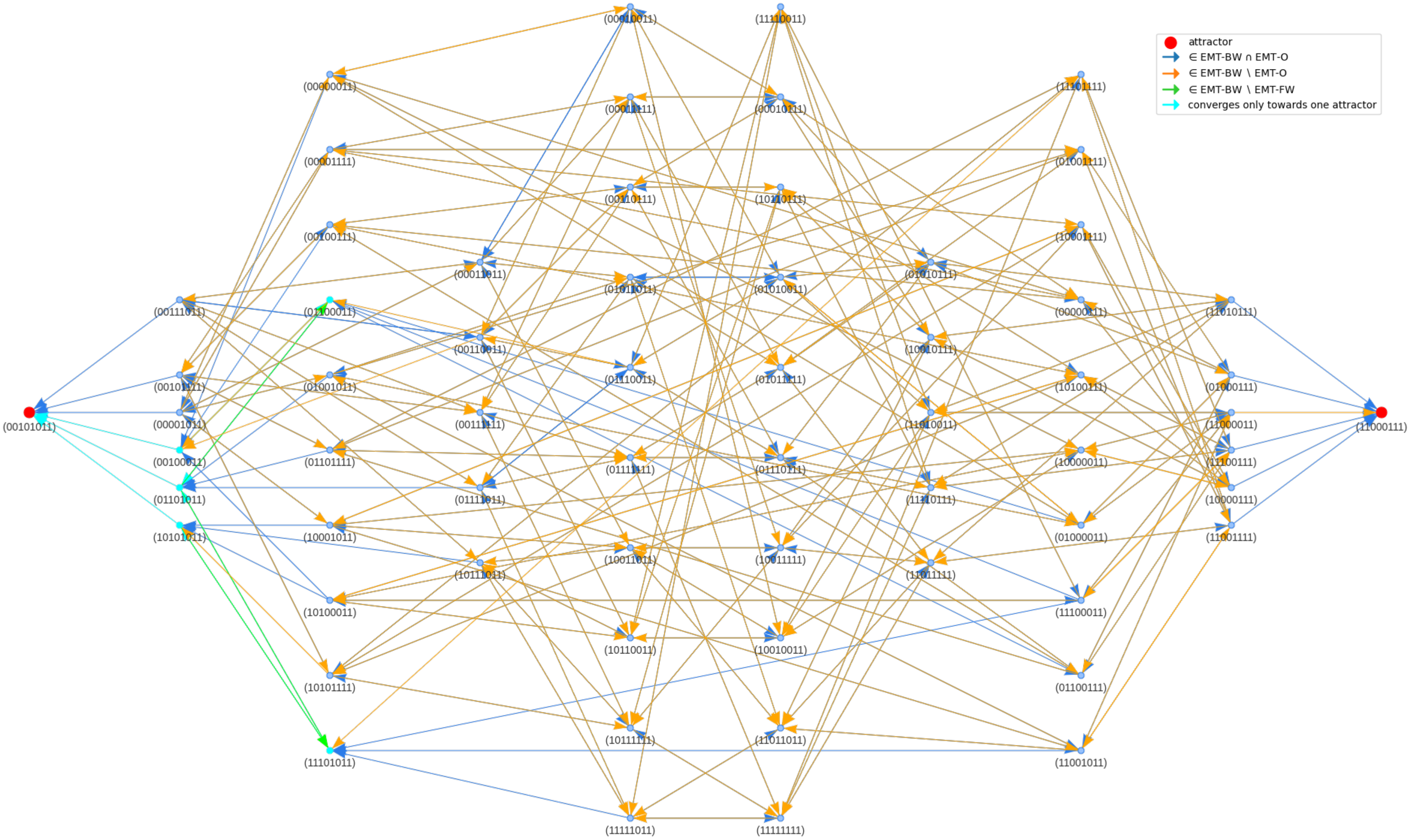

Supplement: S6 Fig — The bright red circles are the target attractors of the system. The blue lines are the lines that occur in EMT-O, as well as in EMT-BW. The orange lines are transitions that are part of EMT-BW but not part of EMT-O. The green lines are transitions that are part of EMT-BW but not EMT-FW. The light blue lines depict states that are part of the network, but only have access to one of the attractors and not both of them. (PDF) [file pcbi.1009035.s012.pdf]

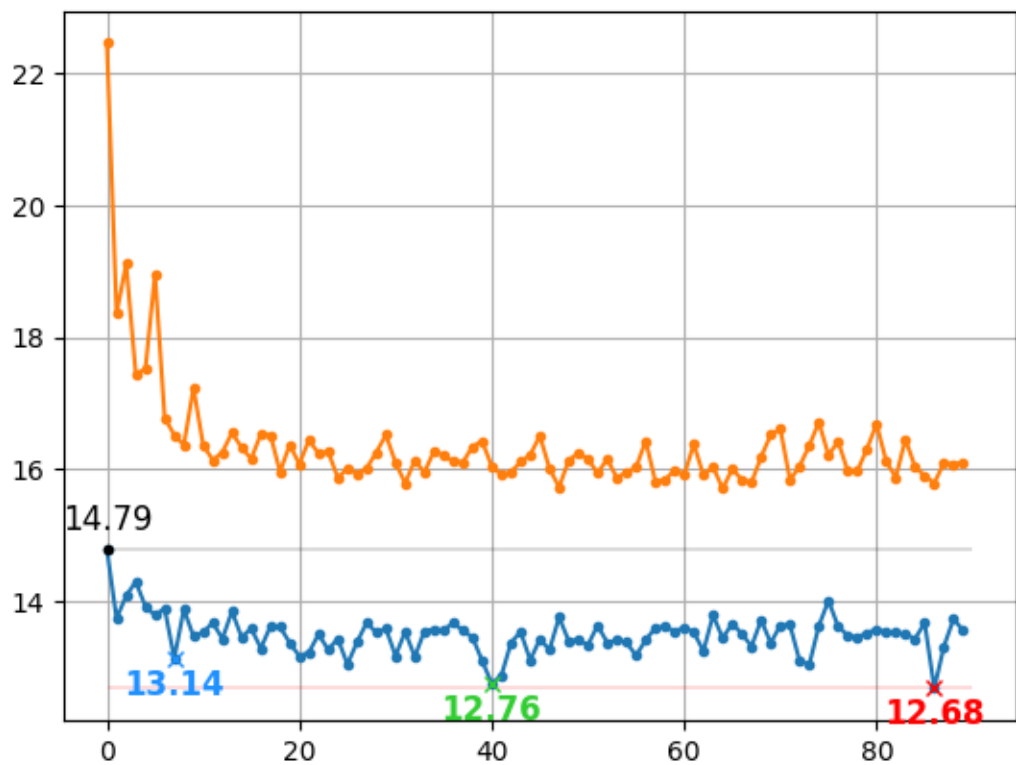

Supplement: S7 Fig — Each generation has 150 individuals and each individual chooses the minimum out of 50 independent runs for its RMS. Setup for the DEAP algorithms can be found in S1 Table. The orange line is largest RMS for each generation, and the blue line is the smallest RMS both chosen as the smallest number of 50 random simulations. The smallest RMS within 90 generations is 12.68 (EMT-C) at generation number 86. We reach a similar RMS at generation 40 with a value of 12.76 (EMT-B). An early fit after 7 generations already achieves an RMS of 13.14 (EMT-A). (PDF) [file pcbi.1009035.s013.pdf]

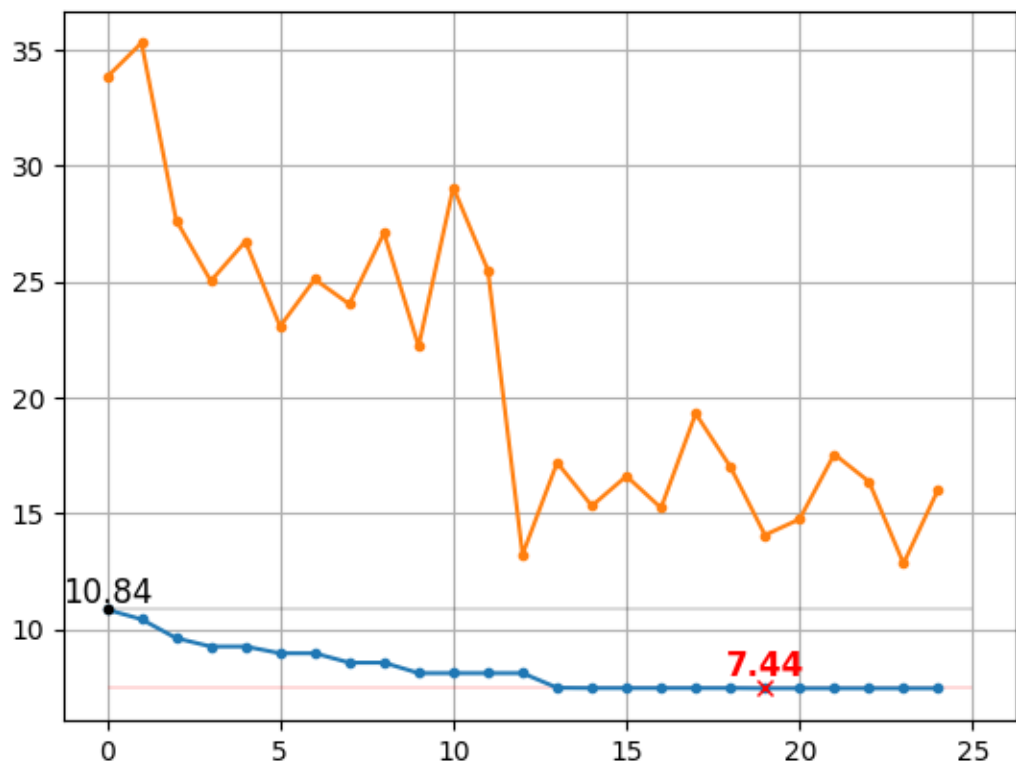

Supplement: S8 Fig — Each generation has 150 individuals. Setup for the DEAP algorithms can be found in S1 Table. The orange line is largest RMS for each generation, and the blue line is the smallest RMS. The smallest RMS within 25 generations is 7.44 at generation number 20 (EMT-E). The fit found before that is similarly high with a value of 7.45. (PDF) [file pcbi.1009035.s014.pdf]
