# Supplementary material for: Unsupervised logic-based mechanism inference for network-driven biological processes
Source: PLoS Comput Biol. 2021 Jun 2;17(6):e1009035. doi: 10.1371/journal.pcbi.1009035 (PMC8202945; doi:10.1371/journal.pcbi.1009035)
Supplement: S1 Fig — The states of Hamming distance 2 to the attractor are marked in pink. Each of these pink states has the possibility to switch one of the species E, S, ES, and P. If the bit flip results in a possible pathway, the new state is colored in green, otherwise it stays black. For the state (E, S, ES, P) = (0, 0, 1, 1), e.g., the flip of bit E results into the state (1, 0, 1, 1), which is a Hamming distance away from the attractor, and therefore a viable pathway. The state (1, 0, 1, 1) is therefore marked in green. Since state (0, 0, 1, 1) needs to swap the E-bit, this state will get added to the transition list for species E (see Table 3 of the main paper). For this pathway to reach the destination, the state (1, 0, 1, 1) needs to flip its third bit (denoted in green over the arrow) to reach the red attractor (therefore, (1, 0, 1, 1) is sorted in the transition list for ES in Table 3). The flip of S and P result in the states (0, 1, 1, 1) and (0, 0, 1, 0) respectively, which are a Hamming distance of 3 away from the attractor. This would mean that the pathway is going away from the attractor, and therefore these states are not considered in the forward only algorithm. Therefore, these states are denoted in black. Note, however, that these states would be sorted into the backward pathways algorithm. Flipping the bit for ES results in the state (0, 0, 0, 1). This state has been eliminated as initial condition since a system in which only P exists is not meaningful. This path therefore will also not be sorted in any of the transition lists and therefore stays black. (PDF) [file pcbi.1009035.s007.pdf]

$$
\begin{pmatrix}0\\0\\1\\1\end{pmatrix}
\begin{array}{lll}
\xrightarrow{E} \begin{pmatrix}1\\0\\1\\1\end{pmatrix} & \xrightarrow{3} & \begin{pmatrix}1\\0\\0\\1\end{pmatrix} \\
\xrightarrow{S} \begin{pmatrix}0\\1\\1\\1\end{pmatrix} & \mathrm{d} = 3 & \\
\xrightarrow{ES} \begin{pmatrix}0\\0\\0\\1\end{pmatrix} & \text{IC!} & \\
\xrightarrow{P} \begin{pmatrix}0\\0\\1\\0\end{pmatrix} & \mathrm{d} = 3 &
\end{array}
$$

$$
\begin{pmatrix}1\\1\\0\\0\end{pmatrix}
\begin{array}{lll}
\xrightarrow{E} \begin{pmatrix}0\\1\\0\\0\end{pmatrix} & \text{IC!} & \\
\xrightarrow{S} \begin{pmatrix}1\\0\\0\\0\end{pmatrix} & \text{IC!} & \\
\xrightarrow{ES} \begin{pmatrix}1\\1\\1\\0\end{pmatrix} & \mathrm{d} = 3 & \\
\xrightarrow{P} \begin{pmatrix}1\\1\\0\\1\end{pmatrix} & \xrightarrow{2} & \begin{pmatrix}1\\0\\0\\1\end{pmatrix}
\end{array}
$$

$$
\begin{pmatrix}1\\0\\1\\0\end{pmatrix}
\begin{array}{lll}
\xrightarrow{E} \begin{pmatrix}0\\0\\1\\0\end{pmatrix} & \mathrm{d} = 3 & \\
\xrightarrow{S} \begin{pmatrix}1\\1\\1\\0\end{pmatrix} & \mathrm{d} = 3 & \\
\xrightarrow{ES} \begin{pmatrix}1\\0\\0\\0\end{pmatrix} & \text{IC!} & \\
\xrightarrow{P} \begin{pmatrix}1\\0\\1\\1\end{pmatrix} & \xrightarrow{3} & \begin{pmatrix}1\\0\\0\\1\end{pmatrix}
\end{array}
$$

$$
\begin{pmatrix}1\\1\\1\\1\end{pmatrix}
\begin{array}{lll}
\xrightarrow{E} \begin{pmatrix}0\\1\\1\\1\end{pmatrix} & \mathrm{d} = 3 & \\
\xrightarrow{S} \begin{pmatrix}1\\0\\1\\1\end{pmatrix} & \xrightarrow{3} & \begin{pmatrix}1\\0\\0\\1\end{pmatrix} \\
\xrightarrow{ES} \begin{pmatrix}1\\1\\0\\1\end{pmatrix} & \xrightarrow{2} & \begin{pmatrix}1\\0\\0\\1\end{pmatrix} \\
\xrightarrow{P} \begin{pmatrix}1\\1\\1\\0\end{pmatrix} & \mathrm{d} = 3 &
\end{array}
$$
