# Supplementary material for: Unsupervised logic-based mechanism inference for network-driven biological processes
Source: PLoS Comput Biol. 2021 Jun 2;17(6):e1009035. doi: 10.1371/journal.pcbi.1009035 (PMC8202945; doi:10.1371/journal.pcbi.1009035)
Supplement: S2 Fig — On top, we see the possible evolution of states with a Hamming distance of 3 away from the attractor in blue. The state (E, S, ES, P) = (0, 0, 1, 0), e.g., can flip either the first or the fourth bit, to reach one of the pink states introduced in S1 Fig. The state (0, 0, 1, 0) gets therefore sorted into the transition list of E as well as P. It would be sorted into the S list, if we consider backward pathways as well, but flipping ES leads to an unfeasible state in the system, which is why this state is never sorted into the transition list for ES. On the bottom, we see the starting pathway for the state that is a Hamming distance of 4 away from the attractor and how to reach the Hamming distance states of 3 to create the desired pathway. (PDF) [file pcbi.1009035.s008.pdf]

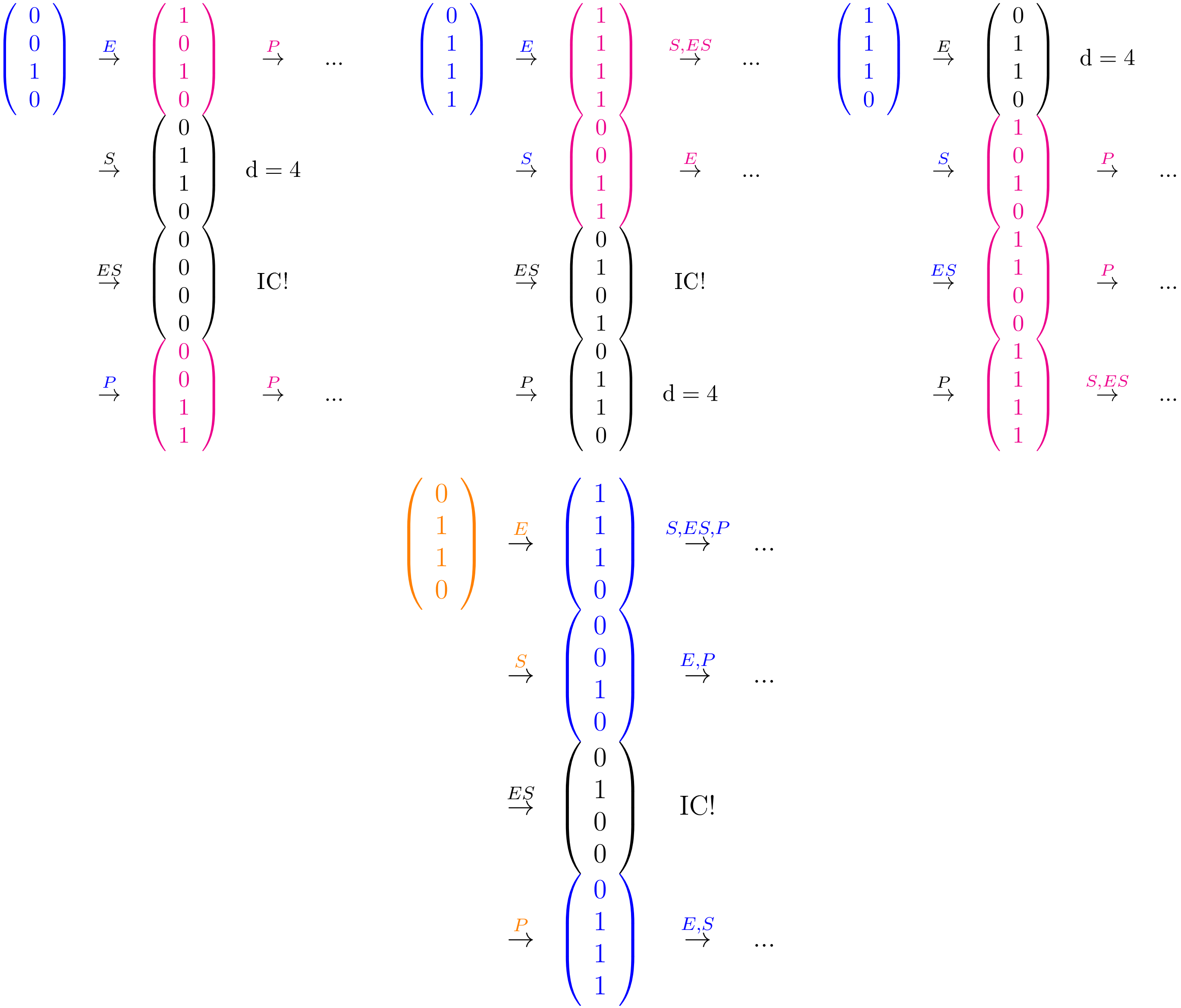
E
P
...
S
d = 4
ES
IC!
P
P
...
E
S,ES
...
S
E
...
ES
IC!
P
d = 4
E
d = 4
S
P
...
ES
P
...
P
S,ES
...
E
S,ES,P
...
S
E,P
...
ES
IC!
P
E,S
...
