# Supplementary material for: Unsupervised logic-based mechanism inference for network-driven biological processes
Source: PLoS Comput Biol. 2021 Jun 2;17(6):e1009035. doi: 10.1371/journal.pcbi.1009035 (PMC8202945; doi:10.1371/journal.pcbi.1009035)
Supplement: S1 Table — (PDF) [file pcbi.1009035.s015.pdf]

opt	unsupervised	expert guidance
pop size	150	150
selection	selTournament(tournsize = 15)	selTournament(tournsize = 3)
crossover	cxTwoPoint()	cxUniform(indp=0.05)
mutation	mutPolynomialBounded(eta=20, low=0, up=1, indpb=0.1)	mutPolynomialBounded(eta=20, low=0, up=1, indpb=0.1)
CXPB	0.9	0.9
MUTPB	0.2	0.2
