# Supplementary material for: Unsupervised logic-based mechanism inference for network-driven biological processes
Source: PLoS Comput Biol. 2021 Jun 2;17(6):e1009035. doi: 10.1371/journal.pcbi.1009035 (PMC8202945; doi:10.1371/journal.pcbi.1009035)
Supplement: Attachment — Submitted filename: Response_rev_v1.pdf [file pcbi.1009035.s016.pdf]

We thank the reviewers and greatly appreciate their constructive feedback, which greatly improved the quality of our manuscript. We have made changes to address all the reviewer comments including the addition of one more author who was responsible for code maintenance and upload to GitHub, to make the code more easily accessible.

The edits in the manuscript in response to the comments by Reviewer #1 have been written in blue text.

Reviewer #1: This manuscript proposes an unsupervised method to infer Boolean (logical) functions from the knowledge of all initial states that can converge to an attractor. The work could be a useful contribution, complementary to other existing network inference approaches. Unfortunately, several aspects of the work are not clearly presented or not sufficiently addressed. In addition, the abstract contains statement that are not borne out in the manuscript.

We thank the reviewer for their positive comments and recognizing the potential impact of the work. We have tried our best to address the reviewers' comments as described below.

Major comments:

1. The algorithm expects binary state information for every element (node) in the system. The two examples presented in the manuscript used all the 2^N states of the system. Some of these states were attractors and for all the remaining states the algorithm needed to know to which attractor they converge. There are at least two difficulties of generating this required input knowledge. How are the binary states decided from continuous experimental data? How realistic is to require that every state of the system was considered as an initial state in an experimental investigation? To my knowledge, it is generally assumed that there is a single, "natural" initial state. It is not general practice (and may be impractical) in experiments to start the system from every state it could have. How would the algorithm cope with having only a fraction of the information it needs?

We thank the reviewer for this insightful feedback. We addressed the general point of how to gain Boolean data from experimental knowledge, by including some references in the second paragraph of the Introduction section that deal with the problem of data discretization. Since the experimental setup usually does not always deliver the required input for the mathematical setup, we have clarified these steps throughout the text and added a paragraph in the Discussion section to note ways in which users could account for lack-of-knowledge in their modeling approach.

2. The algorithm generates Boolean rules (functions) for each node. This is not the same as "mechanistic hypotheses of cellular processes" (as stated in the abstract). Consider the sets of Boolean functions inferred for the enzyme-substrate system (line 142, or 178, or 199). What are the mechanistic hypotheses expressed by these functions? Is the implicit assumption that a Boolean function is already a mechanistic hypothesis? If so, this should be stated explicitly and supported by evidence.

We thank the reviewer for this point and acknowledge that the generation of a Boolean ruleset is not equivalent to chemical mechanisms. To address this point, we added a final paragraph to the Methods section that discusses this difference and stresses the difficulty of interpretation. We furthermore aimed to demonstrate such an example at the end of the section Network inference for Enzyme-Substrate dynamics, after we demonstrated our derivation of the method. In addition, we added a short reference to this problem at the last paragraph of the beginning of the Application to an established model section and included the full rulesets for all discussed models (EMT-FW, EMT-BW, EMT-A, EMT-B, EMT-C) for demonstration purpose in the supplement. We also added a sentence regarding the chemical interpretation of the model at the end of section Model selection using expert knowledge. Finally, we also modified the last sentence of the abstract to remove the “mechanistic hypothesis of cellular processes” phrase, which we agree could be misconstrued by readers.

3. The method of generating the Boolean functions from the ordered state transitions is not explained clearly. In line 135, what is a “transition state”? what does it mean to “ translate every transition state via the logical operator AND” ? What does this mean: “An exclusive XOR with the active species ensures that the species activates only with the states from the given list.”?

We appreciate the reviewer’s careful reading of our work to understand our method. We acknowledge that the way we presented this in the first draft of the work may not be sufficiently clear. To address the reviewer’s comment, we extended the section Network inference for Enzyme-Substrate dynamics with the Boolean notations and provide a step-by-step description of the method.

4. The last sentence of the abstract states “We then conclude by placing our results in the context of ongoing work in the field and discuss how our approach could be used to infer mechanisms of signaling, gene-regulatory, and any other input-output processes describable by logic-based mechanisms. “ In fact, very little of this is done in the manuscript; more depth is needed on both parts. There is a considerable literature of Boolean network inference. The manuscript cites three references in which state or time course information was used to infer a Boolean model, but the context does not make it clear that these references also had algorithms (and in fact software). A comparison with existing methods is needed. Also, the discussion merely restates the sentence “our approach can be used to infer used to infer the mechanisms of signaling, gene-regulatory, and any other input-output processes in an automatic (i.e. fully unsupervised) way”; more discussion of the connection of a Boolean function to mechanism is needed.

We thank the reviewer for pointing out this issue and apologize that we fell short in our effort to indeed place our work in the context of other efforts in the field. As the reviewer points out, there is considerable literature of Boolean network inference. Unfortunately, we do not understand the reviewer’s comment that “A comparison with existing methods is needed” since the reason to develop this approach is precisely that there are no other automated Boolean-logic rule inference methods, to our knowledge. To address the reviewer’s comments,

we have added references for Boolean network inference in the Introduction as well as references for popular Boolean software tools available in the field. We have also revised the abstract to make our claims more realistic and aligned with the manuscript. We hope these changes address the reviewer's concerns.

More minor comments:
5. The "network" that is generated by this algorithm (see for example top right of Fig. 1) is in fact the state transition graph of the Boolean system. The nodes are states of the system and the edges are allowed transitions between states. The language needs to be clarified to avoid confusion with the interaction (or regulatory) network.

We agree with the reviewer, that the wording "network" might become confusing in the context. We therefore changed the wording to "state transition graph" in the caption of figure 1 and figure 3, and discussed this in the last paragraph of the Introduction section.

6. In line 14, "each edge corresponds to a Boolean rule or set of rules that describes the interaction between nodes " is not sufficiently accurate and may be misunderstood. An edge between two gene products corresponds to an interaction. A Boolean rule describes the regulation of each node (gene product) by all of its regulators. It reflects all edges incident on a node. An edge can be equivalent to a Boolean rule if and only if there is a single regulator. An edge does not participate in multiple Boolean rules.

Similar to point 5, we agree with the reviewer, that our wording could be misinterpreted, but we added language to the last paragraph of the Introduction and extended the paragraph for response to point 1 in the Methods section to clarify this issue.

The edits in the manuscript in response to the comments by Reviewer 2 have been written in red text.

Reviewer #2: Let me start with my conclusions: the work presented by the authors is great, an interesting read, and a very good example of the much-needed work in formalizing Boolean model inference when applied to the dynamics of intracellular networks. I think this work would be a perfect addition to PLoS Computational Biology.

In this work, the authors describe a model inference framework for Boolean systems with stochastic asynchronous dynamics in which the required prior knowledge consists of mapping between initial conditions (IC) and attractors (A). They include several variants of their framework: one that includes only forward paths in state space from the IC to the attractor (F), one that includes backward transitions that still satisfy the IC->A conditions (B), one that includes expert knowledge in terms of prior-knowledge network (E), and others that constrain the model based on the frequency in which each attractor is reached using a genetic algorithm for model selection.

The authors introduce their method using the classic enzyme, substrate, product model of Michaelis-Menten kinetics, and then proceed to illustrate it to an 8-variable model of the Epithelial-to-Mesenchymal Transition. Some of their main findings are that (1) the forward-only variant often does not recapitulate many of the transitions observed in the reference model, (2) that backward-transition variant can often add too many more transitions than the ones in the reference model, (3) the expert knowledge approach can often help select the appropriate transitions and better match the reference model, and this is further improved when combined with the genetic algorithm approaches to match the frequency to reach each attractor, (4) some of the Boolean rules are not fully constrained by the IC->A + prior-knowledge network, but some are not.

I do have some comments and questions, which I think would greatly improve the manuscript if addressed:

We thank the reviewer for the enthusiastic comments regarding our work. Indeed, we agree that much work is still needed toward formalizing Boolean model inference and we are grateful that our contributions are appreciated.

Major:

1) “Boolean networks assume that the species are either present (1) or absent (0), i.e. E, S, ES, P \in {0,1}, and that all reactions are equally likely, i.e. all rate constants are equal to 1.”

I do not agree with the “all reactions are equally likely” of this sentence, and I think this needs a clarification. What the authors describe is true for the case when the update probability of each node in the stochastic asynchronous scheme is set to be the same for all nodes. It is known that this not a good assumption for many systems, and some previous work has implemented more realistic alternatives (e.g., by updating different kind of nodes with different frequencies, see PMID: 22673395, PMID: 25189528).

I do think this is an important point that should be discussed (perhaps in a “Current limitations and future work” type of subsection?), since the frequency of reaching an attractor would be very sensitive to deviations from the uniform update probability of a node.

We thank the reviewer for this very insightful point. In fact, we have considered this as an avenue for future work. In this paper, we only consider the stochastic asynchronous scheme where “all reactions are equally likely”. We therefore chose to keep this phrasing in the introduction of the Enzyme-Substrate dynamics. However, in response to this comment, we added a paragraph at the end of the Discussion section, where we discuss different updating schemes as an important addition to an inference pipeline, including the suggested references. We want to point out, however, that the way we set up both our

rule creator, as well as our optimizer, it would be straightforward to exchange the asynchronous updating scheme with a more sophisticated one.

2) It is strange to me that the expert knowledge example of the Michaelis-Menten kinetics did not include the one based on the prior-network informed approach. There might be an underlying reason for this, but I think the authors should include this, as it becomes important in the more complicated EMT example.

We thank the reviewer to make us aware of this point. Indeed, there is a reason why we chose different ways of incorporating expert knowledge into the system: while the Enzyme-Substrate model is based upon a kinetic model, we do not have the same kinetic information when we try to recapture the EMT example. In response to the reviewer's comment, we have clarified this point at the beginning of the Model Selection Using Expert Knowledge section.

3) Page 15. "I.e., if we want to eliminate a dependency of a variable, we need to eliminate each transition that does not include its symmetric counter part in the transition list."

It is not clear to me that this the only way, but I do agree this is a possible way to "eliminate dependency of a variable". E.g., if we have f(A,X,Y,Z,W), it is entirely possible that the real function is g(X,Y,A) = f(A=1,X,Y,Z=0,W=0), as long as it does not break the appropriate IC -> Attractor transitions. So, my general question is, (i) is it true that this way to eliminate dependency of a variable is able to obtain any function that can satisfy the IC->A priors, and (ii) is this true even if there is co-dependence between the functions of two variables (e.g. the IC->A priors can be satisfied if {f_variable1 = f1, f_variable2 = f2} or {f_variable1 = g1, f_variable2 = g2}, but not if {f_variable1 = f1, f_variable2 = g2} or {f_variable1 = g1, f_variable2 = f2}.

We are very thankful to the reviewer for the effort spent understanding our technique. We agree that the answer to both of points, in principle, is "not necessarily", since the removal of the states without any checks can result in the complete loss of the path towards a given attractor. However, our code does include a state reachability check, but we accidentally omitted this point for this context. As a response to this point, we have now added this discussion in the section Model Selection Using Expert Knowledge, at the point where we introduce Algorithm 1.

4) Page 16. "of 16 x 1 x 8388608 x 256 x 32 x 1 > 1e12 possible networks to choose from." This is related to my above question on co-dependence between the functions of two variables that satisfy the IC->A priors. Shouldn't there be correlation between the choice of rules in one variable vs the one in the other? The fact that the number of rules is multiplied suggests that there is no correlation between the function in each of the variables. Is the multiplication of rule numbers possible because there is a guarantee that the IC -> Attractor transitions are respected?

This is a very insightful observation and we greatly appreciate this being pointed out by the reviewer! The method is set up in a way that the reachability check is part of the optimization process. The numbers that we reported were obtained by estimating all possible ways of elimination using brute-force, but some of those networks included in the calculation could indeed violate the reachability check. The check, however, assures that we are not simulating these non-reachable networks. Also, as the reviewer inferred, the rules for each species are independent, which is why we can multiply the number of networks in this case. In response to this insightful comment, we added a clarification in the caption of Table 7 and a sentence in the section Model selection using expert knowledge after Algorithm 1 to emphasize that each of the networks can be combined, before we list these numbers. In addition, to further clarify this point, we included a network selection example in the supplement for one of the species as a demonstration.

5) On the inclusion of signs on the prior-knowledge network. Is there are a reason why the prior-knowledge network did not include the sign of the interactions? It seems to me that this might drastically reduce the number of possible Boolean functions in Table 7. Even if the algorithm has difficulty constraining Boolean functions to the sign-definite ones during the optimization processes, the authors should still be able to report how many of the ones in Table 7 are consistent with the known sign of the interactions (for the sign-definite functions of the reference model). If it is difficult to implement this, this should be discussed in a “Current limitations and future work” subsection.

We thank the reviewer for this thoughtful question. We did not include an explicit mention of the directionality of the rules because by definition, Boolean rules are inherently directional. We believe this confusion may come from the chemical-kinetics based description of chemical reactions, where sign-definite interactions can be defined. However, in Boolean formalisms we are merely describing the logical combinations that flip a bit for a given species.

6) Partial knowledge of the {IC->A} priors. I think it is important to have a discussion of what would happen if one only has partial knowledge of the {IC->A} priors, since this is one of the distinguishing features of this approach. Ideally, one would like a quantification for what happens with the two models described as one decreases from a full to partial knowledge of the {IC->A} priors. This is also important if one wants to apply this to a larger model, for which full knowledge of the {IC->A} priors would be impossible.

We agree with the reviewer, that this is an important part of the discussion. As a matter of fact, a similar issue has also been raised by Reviewer 1, and we therefore included a paragraph in the Discussion section (blue) to suggest some possible ways to account for the lack of knowledge as a modeling point.

7) GitHub implementation. Currently, the GitHub implementation seems bare-bones. I would say that, at minimum, one should be able to reproduce the results of the

manuscript, and it should be clear (through a detailed read me) how to do it. Ideally, one would like to be able to provide a set of {IC->A} priors, and possibly a network, and obtain the rules of the ensemble of models.

We thank the reviewer for this feedback. We have updated the readme in the repository and extended the content to include both the EMT model as well as the enzyme-substrate kinetics model. We included a Jupyter notebook for the latter in a way that we think makes it now accessible and possible to reproduce the results that we use in this paper. To address this point, we have added an author that helped with this part of the work, as well as the write up fo the pertinent sections.

Others:

8) It is not clear to me if the forward-only network includes all possible orders of forward paths that can be obtained from the given ICs to the given attractors

As a response to another reviewer, we separated the Methods and Results into separate sections. As a response to this comment, we added a paragraph to the beginning of the Methods section to clear up this confusion.

9) Page 7. “Backward dynamic paths to enable dynamic loops (ES-B)”. I did not notice at first that ALL backward transitions are added (except the ones that destroy attractors). I was also very confused about how the information on which ones to add would be added, but I see the authors discuss that later in the section. I would recommend including a note on adding all allowed backward transitions being and oversimplification, and that this will be discussed later.

The beginning of the Methods section should now address this point as well.

Minor:

- Figures S1-S2 are very hard to follow as they currently are. Additional explanation of the underlying “notation” of the figure and the colors would be helpful.
- Figure S3-S6. A legend for the color scheme would be extremely helpful, so that one doesn’t have to rely on the caption. Also a more detailed description of the figure would be helpful (e.g., red are the target attractors).
- The authors use the word “pathway” to describe paths in the state transition network. I have to say I was first confused by this terminology, since “pathway” has a particular biological meaning that is associated with the biological network, while the authors use it to describe the state transition network. I would recommend using “path” or “path towards X”, or some other alternative, instead of “pathway”.

- Fig 3. “The darker the arrow, the more simulation steps are necessary to reach this particular transition in principle.” I am not sure what the authors mean with “in principle”. I think that extra pair of words is not needed.
- Fig 3. “The x-axis represents the number of simulation steps it takes to reach the steady state.”. I am not sure the “it takes to reach the steady state” part is needed.
- Page 10. “Application to an established model: Epithelial to Mesenchymal Transitios”. There is a typo; this should say “Transition”.
- Page 12. “we extended Table 4 by the according frequencies for our automatically created”. There is a typo, “Table 4 by the according frequencies”.
- Page 10. “For the other three subnetworks, the frequencies to enter into a given steady state generally match well with EMT-O.”. I am not sure I agree. (1,1) does seem to match, (0,1) must match (by construction), but (0,0) seems slightly better than the (0,1) results but not by much.

To address these points, we

- Rewrote the captions of Figures S1 and S2 to discuss the interpretation of the figures in detail
- Added a legend to Figures S3-S6, and added the target attractors to the caption
- Discussed the usage of “pathway” at the end of the Methods section
- Updated the figure and caption for Figure 3
- Fixed the typo “Transitios” -> “Transitions”
- Changed the following words: “according frequencies” -> “corresponding frequencies”, “see” -> “interpret”

The edits in the manuscript in response to the comments by Reviewer 3 have been written in green text.

Reviewer 3: Prugger *et al.* introduce an algorithm which can automatically construct boolean rules and generate network structure hypotheses for biological mechanistic and boolean models based on given initial states and attractors(a form of different desired states of molecular species). Boolean modelling of biochemical reaction networks is important for modelling (and interpreting experimental data) of very large networks. This is possible due to the abstract and simplistic nature (non-parametric) of the models.

Their contribution lies in aiding modellers to experiment and design models by explorative workflows where the bulk of the work (constructing candidate models) can be done automatically without prior knowledge. The result shows that little prior knowledge will produce many candidate models, and therefore also be far away from the true dynamics. However, introducing more complicated transitions improves the predictions, and further adding domain knowledge yield results very close to the true desired dynamics.

While the method itself has the potential to be very useful for modellers, the presentation and structure of the paper for a more general audience needs major revisions.

We thank the reviewer for their feedback regarding our work and its potential. We are also grateful for the reviewer's comments, which we believe have greatly improved our manuscript.

Major:

1. The introduction to the problem lacks sufficient background. Certainly, a lot of work on constructing boolean rules and network inference has been done. Further, the authors should consider presenting the problem to a more general biological audience.

   We thank the reviewer for this insightful comment. In response to this comment, we added a paragraph to the Introduction to better explain the biological relevance of Boolean rules and their translation into networks. We also extended the Introduction by adding additional references for Boolean rules and network inference papers.

2. The proposed methodology is referred to as unsupervised by the authors. However, there is no clear explanation in the introduction to this statement. The notion of creating maps between inputs(initial states) and outputs (attractors) which are obtained from experimental data, surely sounds like a supervised or semi-supervised problem, therefore causing confusion. Further, "unsupervised" often refers to "unsupervised learning" in the context of machine learning algorithms. However, machine learning is never mentioned. Is the proposed method considered to be a machine learning algorithm? Distance based, genetic optimization etc.

   We thank the reviewer for this interesting point. In the optimization field, unsupervised simply means "automated, without human interaction", but as the reviewer points out, this term has become closely associated with Machine Learning methods. To make this point clearer, we have added clarifying text to this term throughout the text to define exactly what we mean by "unsupervised".

3. Methods & Results: The integration of Methods and Results makes the reading difficult. The introduction to Methods tries to summarize the core components of the proposed methodology, however it's not until after the ES example that one gets a clear understanding of how the algorithm works. Fig.1 is not very informative and does not add much to the interpretation. Further, I understand that ES can be seen as a contribution to results. However it's main purpose is to act as a demonstrating example to introduce the methodology. I would recommend the following structure to the authors: As a supplement to fig 1 I would include an algorithmic presentation of the proposed method, including the core components and the different versions/extensions (forward, backward and domain knowledge etc). Consider separating Methods & Results, where "Method" would consist of a clear summary of the proposed method followed by the ES example. The Result section would then demonstrate the proposed on a realistic and challenging example.

   We thank the reviewer for this comment. Following the reviewer's suggestions, we have separated the Methods and Result sections from each other to make the paper easier to read.

We also updated the structure of the paper to make it easier to read. We hope these changes have made the paper easier to read.

4. The quality of the figures are poor. Some of them have low resolution and a lot of white spaces. There are a lot of opportunities for improvements here. Further, the quality is even poorer in supplemental materials. Consider using a shared axis, increase font size and use high DPI and high quality format in accordance with the journal's instructions. Make figures more compact.

   We thank the reviewer for this comment. As a response, we have done the following:

   - Figure 1: boldface text; heavier lines; saved as pdf; increased size.
   - Figure 2: heavier lines; increased font size for title, legend, x-/y-labels, x-/y-ticks; saved as pdf.
   - Figure 3: heavier lines for the network graphs and the simulations; saved as pdf; increased label size in the network graphs; increased the size of the figure by using the full-page width
   - Figure 4: heavier lines; saved as pdf; increased the size of the figure by using the full-page width,
   - Figure 5: saved as pdf; increased the size of the figure by using the full-page width,
   - Figure 6: increased font size for x-/y-labels, x-/y-ticks, legend; saved as pdf

5. There is no indication of the complexity or the computational burden of the proposed method. It is only mentioned that it is done in parallel, how is it done in parallel, how does it scale and what roughly would be a realistic cost estimate in core hours?

   We thank the reviewer for pointing out this accidental omission on our part. We have added some information about the used architecture, runtime, and scaling to the Materials and Methods section

6. Conclusion does not include any mention of the results.

   Again, we thank the reviewer for this accidental omission. In response to this comment we have added remarks about our results to the Discussion and Conclusion sections.

Minor:

1. Using boolean state representations and boolean operations in text(especially in tables) gets easily overwhelming as a reader. Consider using other representations to simply the reading.

   We agree with the reviewer, that the chosen notation can be hard to read for non-experts. We tried multiple formats and different notations but none yielded an easier format. In response to this and other reviewer comments, we included a description of the mathematical notation to the Network Inference for Enzyme-Substrate Dynamics section, before we introduce the algorithm. We believe this greatly helps in following the notation in the text that follows.

2. The hamming distance needs to be defined.

   We thank the reviewer for pointing out this unintended omission. In response, we have added a short explanation in the Methods section, where the concept is first introduced.

3. Certain claims throughout the text are missing references. For example line 32-33 and 535-536.

   Thanks for noting these. We have added citations as requested.

4. Some few sentences need to be rephrased (language). For example line 34.

   We have carefully cleaned up the grammar and language of the paper to the best of our capabilities.

Other Changes:

- Corrected Figure 6 (EMT-F/EMT-B -> EMT-FW/EMT-BW)
- Corrected a typo in the translation of the states to the MM ruleset.
- Corrected a wrong arrow in the transition graphs of Figure 3.
- Updated Acknowledgements section.
- Added Mr. Perry T. Wasdin as an additional author.
- Updated Dr. Leonard A. Harris' affiliation.
